# China’s Legal Protection System for Pangolins: Past, Present, and Future

**DOI:** 10.3390/ani15162422

**Published:** 2025-08-18

**Authors:** Da Su, Kai Wu, Anzi Nie

**Affiliations:** 1 Postdoctoral Program in Law, School of Law, Xiamen University, Xiamen 361005, China; sdepl@xmu.edu.cn; 2 Sturm College of Law, University of Denver, Denver, CO 80210, USA; kai.wu@du.edu; 3 Daniel Felix Ritchie School of Engineering & Computer Science, University of Denver, Denver, CO 80210, USA; 4 School of Law, Zhongnan University of Economics and Law, Wuhan 430073, China

**Keywords:** pangolin conservation, wildlife protection in China, Chinese law, biodiversity

## Abstract

This article examines the historical evolution, contemporary dynamics, and future trajectory of China’s legal and judicial framework for pangolin protection. By reviewing over seventy years of regulatory changes, case law, and policy implementation, it outlines three distinct phases: the early emphasis on pangolins as medicinal and export resources (1949–1989); the phase of conflicted protection and utilization under regulatory expansion (1989–2020); and the post-2020 shift toward judicial activism and ecological civil litigation. We then highlight the long-standing contradiction between legislative protection and continued medicinal use, particularly the centuries-old use of pangolins and their derivatives in traditional Chinese medicine, a practice still acknowledged within certain state policies and regulatory frameworks, showing how these inconsistencies enabled persistent illegal exploitation despite regulatory controls. Through systematic analysis of public court records and case databases, the policy historical records reveal a marked increase in environmental public interest litigation since 2020. These lawsuits, often attached to criminal prosecutions, signal a transition from merely punitive approaches to restorative ones—anchored in ecological valuation of species and their services. Case studies illustrate how courts now impose not only wildlife resource loss fees, but also punitive damages and compensation for ecological service function loss. The article will elaborate in detail on the distinctions and interrelations among these types of compensation. The landmark Case No.17 also demonstrates this paradigm shift, wherein courts recognized pangolins’ role in balancing forest ecosystems. However, significant challenges persist. Valuation methodologies lack uniform standards; while the ecological value of pangolins has been recognized, their inherent value as individuals has not been emphasized within the legal system; judicial discretion varies across jurisdictions; and public interest organizations remain underutilized in litigation. Moreover, while the crackdown on organized crime succeeded in curbing mass trafficking, smaller-scale violations tied to cultural consumption for medicine use persist. The article concludes that judicial innovations, such as ecological judicial restoration bases and integration into China’s draft Ecological Environment Code, offer promising pathways forward. To enhance efficacy, it calls for standardization in ecological valuation, strengthened civil society participation, and nuanced differentiation in penal strategies between minor and serious offenses. This study ultimately positions judicial reform as the cornerstone of China’s evolving pangolin conservation strategy.

## Simple Summary

The historical development, current framework, and future trajectory of pangolin protection in Chinese law remains notably underexplored. Drawing on legislative texts, judicial decisions, and empirical case analysis, the study identifies three key stages in China’s pangolin protection regime: from solely utilization evolving into judicial protection. The historical retrospective analysis emphasizes how legislative inconsistencies historically hindered effective protection, particularly due to the medicinal use of pangolins in traditional Chinese medicine. Notably, the rise of environmental public interest litigation and the increased valuation of pangolins’ ecological function significantly reshaped judicial practice. We argue that judicial mechanisms—especially civil public interest lawsuits—are becoming the primary pathway for pangolin protection in China. While substantial progress has been made, challenges remain in standardizing valuations, curating a public interest litigation regime, and developing differentiated approaches to minor and organized crimes. The article contributes practically, suggesting targeted reforms to enhance pangolin protection within China’s broader ecological governance framework.

## 1. Introduction

Globally, the illegal wildlife trade poses a major threat to biodiversity [1]. In China, pangolins account for a significant proportion of this illicit trade [2]. Pangolin populations in China sharply declined since the last century due to illegal hunting and trade. Surveys conducted by the Chinese State Forestry Administration indicated approximately 64,000 pangolins in China in 1998, which fell to an estimated 25,100–49,450 by 2008 [2]. According to data released by the Convention on International Trade in Endangered Species of Wild Fauna and Flora (CITES) in 2018, the Chinese pangolin population decreased by 90% over 20 years [3].

The year 2020 was pivotal for pangolins in China [4]. In that year, the National People’s Congress issued the “Decision on Comprehensively Prohibiting the Illegal Trade of Wild Animals, Eliminating the Bad Habit of Excessive Consumption of Wild Animals, and Effectively Safeguarding the Life and Health of the People,” which banned the consumption of wild animals [5]. Subsequently, in June 2020, pangolins were reclassified from Class II to Class I (higher level critical) protected wildlife species under China’s national protection system [6]. Furthermore, recent environmental public interest litigations have seen new developments in the monetary valuation of pangolins’ ecological worth. In one case, the “wildlife resource compensation fee” for a single pangolin reached as high as RMB 450,000 [7], a significant increase from the previous official judicial valuation of RMB 8000 [8]. Pangolin scales constitute about one-fifth of their body weight [9] and the economic gain from them is a primary driver of pangolin-related crimes [10]. According to statements from arrested criminals, the smuggling cost per kilogram of pangolin in illegal trade ranges from RMB 80 to 150, but the selling price can be as high as around RMB 800 [11]. However, some also believe that the entire pangolin has medicinal value in traditional Chinese medicine (TCM) [12]. Some researchers distinguish between the use of pangolins for their “scales” and for their “meat.” The consumption of the former is influenced by factors such as traditional Chinese medical practice, patient demand, and economic means [10]. The latter, however, is primarily driven by curiosity, displays of social status, and commercial banquets [13]—practices more commonly observed in southern regions of China [14]. Addressing this issue requires acknowledging the evaluation of pangolin efficacy by traditional TCM and pharmacological research. Pangolin scales have been used in some TCM treatments for pain relief [15], anti-inflammatory, and blood-activating functions, with their medicinal use dating back to the Compendium of Materia Medica [16].

However, due to their endangered status and strict state control, the medicinal effects of pangolin scales cannot be verified through large-scale clinical trials in the modern medical sense; rather, their use is based on centuries of TCM experience [17]. Although the technical standards, clinical traditions, and national policies of the TCM industry have at times supported or implicitly recognized pangolin products as a type of “Chinese medicine [18],” this endorsement is rooted in the tradition of TCM, not in strict, reproducible scientific conclusions verifiable by modern medicine [19]. It is also worth noting that the vast majority of the Chinese public does not use pangolins or their products in their daily lives [20].

Given that illegal pangolin trade is cross-border and cross-regional, China signed mutual legal assistance treaties (MLATs) with countries such as Indonesia and Vietnam to cooperatively promote judicial matters concerning pangolins in a non-mandatory manner [21]. The treaty facilitates the collection of evidence related to transnational crimes, the arrest and prosecution of suspects, and the timely rescue of live pangolins. It also requires mutual recognition of each other’s civil and criminal judgments, excluding matters of sovereignty—which further helps prevent the repeated prosecution of the same offenders. Due to China’s vast territory and the rapid transformation of urban spaces, a real-time, effective monitoring system for pangolin distribution and population has yet to be established [22]. The illegal demand for pangolins and their products from China places immense pressure on relevant regulations [23]. Since current pangolin legislation cannot fully meet protection needs, and the medicinal use permits for pangolin scales have not been entirely abolished, judicial intervention has become an effective pathway for pangolin protection. Existing academic research rarely examines pangolin protection in China from the perspective of judicial operations. Huang et al. analyzed pangolin trade hotspots in China from 2011 to 2019 using judicial judgments [24]. Wu et al., on the other hand, argue that the “One Health” approach has become an important influencing factor in China’s wildlife-related judicial rulings [25].

Given that China is one of the largest markets for both legal and illegal pangolin trade globally, its legal stance on pangolins is not merely a matter of domestic wildlife protection. Owing to its geographic proximity to countries such as Vietnam and Malaysia, as well as its integration into global trade networks [26], the issue has taken on international legal relevance. Facing immense demand for pangolins and a corresponding large number of illegal crimes, what efforts has China made judicially in the past few decades? To what extent have these efforts protected pangolins? This paper traces the history of China’s legal and regulatory protection of pangolins and, through analysis of relevant judgments and cases, finds that unlike the legislative vacillation, China’s judicial attitude towards pangolin protection is continuously strengthening. Public interest litigation is emerging as a primary means of protecting endangered wildlife similar to pangolins. China’s public interest litigation has distinct characteristics and differs from the models in some other countries where individual citizens can serve as plaintiffs. Its primary purpose is to safeguard public interests and maintain public administrative order, with qualified public interest organizations or authorized procuratorates serving as plaintiffs, rather than private individuals [27].

## 2. Materials and Methods

This paper explores the historical evolution of legal protection for pangolins within China’s legal framework. The data for this study primarily consist of three main types: First, China’s legislative system regarding pangolins; China possesses a multi-tiered and diverse legal system. We conducted searches on “Peking University Law” [28], a highly authoritative legal and regulatory database in China. The search timeframe ranged from the founding of the People’s Republic of China to the time of this paper’s writing (1 July 2025). We employed a “full-text keyword fuzzy search” using “Pangolin” as the keyword. This legal and regulatory database encompasses official legislative documents from national to local levels. While some laws and regulations have since been invalidated or revised, they are not included in the tables within this paper. Furthermore, for the documents involved in this research, we prioritized official English versions where available; documents exclusively in Chinese were translated and proofread by the author. Second is Chinese judicial judgments involving pangolins; as noted in the article, Chinese judgments have been mandatory to upload to “China Judgments Online” [29] since July 2014. Our search on this database covered the period from its establishment until 22 May 2025. We set the keywords as “Pangolin” and “Public Interest Litigation” for a “full-text keyword fuzzy search”, which yielded cases numbered 3–50 in our tables. The selection scope includes all regions within mainland China. Cases that were nominally related to “pangolins” but substantively irrelevant—such as judgments involving companies named after pangolins—were excluded. Purely criminal cases were also excluded from the dataset. However, this database does not contain all Chinese cases; instead, it includes cases uploaded mandatorily or voluntarily by people’s courts at various levels and localities. In recent years, due to increasing pressure from case trial volumes, the overall proportion of uploaded cases has shown a downward trend. Nevertheless, this dataset remains the most comprehensive publicly available database of judicial judgments in China. Additionally, in 2024, the Supreme People’s Court established the “People’s Court Case Library” [30]. This database specifically uploads cases selected as “reference cases” and “guiding cases” by the judicial system. In 2024, the Supreme People’s Court issued guidance requiring “people’s courts at all levels, when trying cases, to search the People’s Courts Case Library and make judgments strictly in accordance with laws and judicial interpretations, normative documents, and with reference to similar cases in the library” [31]. We searched this database using “Pangolin “as the keyword, with the search period also extending from the database’s inception to May 22, 2025. This search yielded cases No. 1 and No. 2, as well as case No. 17, which was also found via “China Judgments Online.” All cases referenced in this paper have their original judgments publicly accessible through the aforementioned databases. Cases 1, 2, and 17 were sourced from the “People’s Courts Case Library “as “reference cases”. This indicates that cases selected as reference cases represent the authoritative recognition of the Supreme People’s Court, and judges can refer to the reasoning and core judicial opinions of these cases when adjudicating similar matters. Third includes other case reports, investigation details, or official legal documents, notices, and statements publicly available on credible media and platforms, not uploaded to the above databases. To ensure a comprehensive study of pangolin legal protection in China, this paper also involved searching the official websites of relevant national agencies, government departments, and judicial organs. The original texts of certain laws and regulations or other types of government policy documents, such as the “Opinions of the General Office of the State Council on Firmly Advancing the Ten-Year Fishing Ban in the Yangtze River,” [32] required access through these channels. This paper also included searches for articles in various authoritative mainstream Chinese media outlets, including “The Beijing News” [33], among others. Furthermore, Chinese government departments, judicial organs, and public welfare organizations now commonly operate “WeChat Official Accounts” to publish promotional articles. The nature of articles released via this platform signifies authoritative publication by the respective institutions or departments. We obtained valuable research materials from relevant official account articles, including detailed information on public interest litigation cases concerning pangolins initiated by the China Green Development Foundation [7,34,35]. Finally, some fragmented research data and content may have originated from other researchers’ academic papers. Overall, this study exhausted all publicly available research materials concerning pangolins obtainable through legal databases, case databases, public media, and other platforms in China.

Regarding research methodology, this study employs a research methodology that combines legal statute organization and analysis with case compilation and analysis. These methods are framed within a qualitative social science research approach, providing a holistic perspective on the position of pangolins within China’s legal landscape. The research generally follows a historically progressive logic, dividing existing data into three distinct periods: 1949–1989, 1989–2020, and 2020 onwards. For each period, we outline changes in the legal basis for pangolin protection, enforcement practices, and judicial processes. Various micro-level arguments and analyses are interwoven throughout this discussion. For instance, by examining shifts in the adjudicated monetary value of individual pangolins in judicial cases, we explore the underlying changes in the perception of their ecological worth. Given that China’s legal protection of pangolins progressed from being “legislative authority-centric” to demonstrating “cooperation between legislative and administrative powers”, and subsequently to the “active involvement of judicial power”, an analysis of judicial judgments, primarily focusing on China’s emerging environmental public interest litigation, appears in the latter half of the article. This also reflects the adjustments in China’s public authority regarding pangolin protection. Overall, this panoramic study of pangolins in China is currently unparalleled in both Chinese and English research papers. Furthermore, this paper adopts an integrated “macro and micro” research approach, aiming to comprehensively and objectively present the challenges and fates of pangolin species and individuals within China’s overall legal system and in specific legal cases.

## 3. Evolution of China’s Legislative Framework for Pangolin Protection

The legislative framework governing pangolin protection in China was systematically compiled using the Peking University Law Database. This comprehensive legal database provided access to all relevant statutory and regulatory documents. Within the Chinese legal system, these documents adhere to a hierarchy of legal authority: constitution > laws > administrative regulations > local regulations and rules. Furthermore, an important source of law in China is normative documents. Normative documents are official papers issued by administrative organs, possessing functions for managing public affairs and having general binding force [36]. However, they do not possess compulsory enforcement power over citizens; instead, they tend to provide guiding instructions with a lower degree of persuasiveness. Provided they do not conflict with superior laws, the legal force of normative documents can be higher than that of local regulations and rules [37]. The number of laws and regulations related to pangolins is substantial and varies in hierarchical level, as detailed in Table 1 below (more detailed information is available in Supplementary Material S1: Full-Text Links to Laws and Regulations Related to Pangolins in China, and Supplementary Material S2: Full Text of Laws and Regulations Related to Pangolins in China):

Some scholars broadly categorize China’s wildlife industry into three phases: the rise of wildlife domestication and breeding from the 1980s to the mid-1990s, its maturation from the 1990s to the early 21st century, and the period of industrial scale-up from the early 21st century to the present [38]. The legal protection of pangolins is closely linked to the development scale of China’s wildlife industry. Based on practical considerations, we divide the legal protection period for pangolins into three stages: 1949–1989, 1989–2020, and after 2020.

### 3.1. 1949–1989: The Single-Mode Stage of Economic Resource Utilization

#### 3.1.1. Early Policy Origins of Pangolin Protection in China

From the documents of 1962, it can be seen that China’s early wildlife policy was characterized by a “protection + breeding + hunting” approach, imbued with anthropocentric views that treated wildlife as economic resources. As pointed out in the “State Council’s Instructions on Actively Protecting and Rationally Using Wildlife Resources”: “Between 1950 and 1961, the fur of wild animals exported, including that of the weasel, was enough to exchange for 19,512 tractors of 25 horsepower.” During this period, whether pangolins could be hunted depended on the approval of local authorities. The document stated: “Hunting is prohibited or strictly controlled, and the amount of hunting each year must be approved by the provincial (regional, municipal) competent authorities.” Therefore, the hunting of pangolins was not strictly banned but was contingent on the approval of relevant local departments.

#### 3.1.2. The Influence of CITES on China’s Legislation

CITES came into force in 1975, with China joining as a party in 1981. A primary objective of CITES is to require its parties to fulfill their conservation obligations through appropriate policies, legislation, and procedures [39]. To this end, China adopted a legal transformation model, integrating CITES’s objectives and appendices into its domestic legal system.

This effort dates back to 1986, when the State Council, in a notice regarding a report on strengthening TCM, mentioned its decision to stop importing certain endangered animal products to uphold China’s international reputation as a CITES member [40]. Subsequently, the State Council also issued a specific notice on banning the trade of rhino horn and tiger bone [41].

CITES further spurred the development of extensive wildlife protection legislation in China, most notably the Regulations of the People’s Republic of China on the Import and Export of Endangered Wild Animals and Plants in 2006. Article 1 of this regulation explicitly states its purpose is to fulfill CITES obligations [42]. The most direct impact is that the CITES appendices form the fundamental basis for China’s tiered classification system for wildlife protection. This classification is also used as a reference for determining the value of illegal animal products in legal cases [43].

During the 1949–1989 period, pangolins, legally recognized as a “resource,” primarily served two purposes: First, as a crucial resource for the development of China’s indigenous TCM industry. The 1983 “Notice of the State Council’s Approval and Transmission of the State Administration of TCM’s Report on Traditional Chinese Medicine Work Issues” noted the significant demand for pangolins as TCM ingredients at the time and their extreme difficulty in artificial breeding. Legislation during this period aimed at pangolin protection was indirect; for instance, the “Measures for the Administration of Nature Reserves for Forests and Wild Animal Types” sought to protect wild animals, including pangolins, and their natural habitats. In the same year, the state also issued the “National Key Protected Wild Medicinal Animals List,” which designated pangolins as a “key protected wild medicinal animal.” Second, given China’s underdeveloped economy at the time, pangolin scales were also exported abroad as an important source of foreign exchange. During this stage, China’s conservation efforts for pangolins were primarily driven by their significance as a source of medicinal materials.

### 3.2. 1989–2020: The Stage of Parallel Utilization and Protection

#### 3.2.1. Strengthened Regulation of Illegal Activities Involving Pangolins

The promulgation of the “Wild Animal Conservation Law” and the “List of Key Protected Wild Animals” marked the true beginning of legal protection for pangolins in China. However, legislators in this period did not abandon the “utilization-oriented” concept towards wild animals. Although pangolins were classified as Class II national protected animals, and the “Wild Animal Conservation Law” stipulated that obtaining a permit from relevant administrative departments was mandatory for hunting or breeding such animals, the increasing demand for pangolins, changes in criminal methods, and advancements in science and technology during this stage led to frequent illegal pangolin-related crimes. During this phase, the Chinese government began to strengthen the deterrent effect of legal protection for pangolins. For instance, the 1990 “Circular of the General Office of the State Council on the Current Situation of Illegal Poaching, Acquisition, and Resale of Rare Wild Animals” publicized a case of illegal pangolin trafficking. Publishing cases of illegal poaching in such a manner for deterrence and warning was extremely rare in China’s legal regulatory history up to that point.

Concurrently, in response to severe pangolin poaching, the government repeatedly issued relevant documents to strengthen protection. For example, the “Circular of the General Office of the State Council on Transmitting the Ministry of Forestry’s Report on Strengthening Wild Animal Protection and Management” called for monitoring wild animal resources [44]. The “List of Animals, Animal Products, and Other Quarantine Objects Prohibited from Being Carried or Mailed into the People’s Republic of China” included pangolins among the animal species prohibited from being carried or mailed into the country. These restrictions do not apply if specifically approved by national animal and plant quarantine authorities and accompanied by official quarantine certificates from the exporting country or region. These measures aimed to disrupt the chain of illegal pangolin crime through regulation [45].

#### 3.2.2. Enhanced Oversight of Lawful Pangolin Trade and Use

Regulation of legal hunting and utilization of pangolins also continued to improve. The “Measures for Charging Management Fees for Terrestrial Wild Animal Resource Protection” established fees for wild animal resource protection and management, approval procedures, and special hunting permits; in some cases of legal pangolin hunting, the management fee was RMB 100 per animal. Concurrently, the “Criminal Law” also stipulated a series of crimes for protecting wild animals, including pangolins. A 2001 normative document set the case filing standard for illegal pangolin poaching at 4 animals, with 8 animals constituting a major case, and 16 animals a particularly major case. Although various legal documents were continuously formulated and implemented, Chinese legislators did not abandon the categorization of pangolins as “key protected wild medicinal animals.” For example, the 2004 “Guiding Opinions of the State Forestry Administration on Promoting Sustainable Development of Wild Animals and Plants” stated: “While strictly controlling the total consumption of (animal) resources, it is necessary to strengthen the orderly circulation of (animal) resources to prioritize the demand for resources in key areas and industries such as TCM and culture.” Clearly, pangolins fall within the scope of “TCM” utilization. Although China’s pangolin breeding is referred to as an “industry,” it lacks substantive technological feasibility [33]. In September 2007, the “National Plan for Biological Species Resource Protection and Utilization” officially mandated tackling pangolin breeding challenges and establishing pangolin breeding bases. In September 2007, the “Notice on Strengthening the Protection of Saiga Antelope, Pangolins, Rare Snakes, and Standardizing the Management of Their Products for Medicinal Use” officially halted the approval of legal pangolin hunting permits, thereby declaring the end of decades of private pangolin hunting.

In 2015, the State Forestry Administration, as the core regulatory body for wild animals, mandated that the use of pangolin products in prescriptions must carry a special identification label and designated specific hospitals for their use. In the same year, the “Pharmacopoeia of the People’s Republic of China” included pangolins in its list of medicinal sources. While the state’s action was intended to regulate the legal medicinal use of pangolins, it solidified the stability of the pangolin medicinal industry. However, in practice, it is difficult even for legal pharmaceutical companies to prove the legality of their pangolin product sources. The Pharmacopoeia serves as a statutory technical standard that relevant units involved in drug research, production, sales, use, and supervision must adhere to. The persistent poaching of pangolins is partly due to the multifaceted nature of pangolin-related crimes, which in China may involve the regulatory responsibilities of many different departments. In 2019, the Chinese government launched joint operations across various departments, primarily targeting ivory, rhino horn, tiger, pangolin, and their products through joint inspections, law enforcement, and punitive measures. Such “campaign-style enforcement” can temporarily reinforce local departments’ commitment to pangolin protection, but cannot fundamentally resolve the problem of illegal pangolin poaching.

### 3.3. 2020 to Present: Strengthening of Judicial Protection

#### 3.3.1. Legislation: The Beginning of a Comprehensive Ban on Consumption and Changes in the Legal Status of Pangolins

The year 2020 marked a watershed for wildlife protection in China. Under the framework of ecological civilization construction, protecting the ecological environment (including wild animals as a key component) has become a national policy objective and a guiding principle for the judiciary [46]. The uses for illegally hunted pangolins primarily fall into two categories: consumption and medicinal use. Unlike medicinal use, consumption historically remained in a legal policy gray area. The 2020 “Decision of the Standing Committee of the National People’s Congress on Comprehensively Prohibiting the Illegal Trade of Wild Animals, Eliminating the Bad Habit of Excessive Consumption of Wild Animals, and Effectively Safeguarding the Life and Health of the People” signified the commencement of China’s comprehensive ban on wildlife consumption [5]. Furthermore, Article 31 of the latest 2023 “Wild Animal Conservation Law of the People’s Republic of China” explicitly prohibits the consumption, hunting for consumption purposes, and purchasing for consumption of wild animals under state key protection, as well as other terrestrial wild animals with important ecological, scientific, or social value protected by the state [47].

The year 2020 was referred to as the “year of life and death” for pangolins by conservationists primarily because both consumption and medicinal uses were officially removed from key documents. The “Chinese Pharmacopoeia” removed pangolins in May 2020, meaning that, under statutory technical standards, pangolins were no longer deemed suitable for medicinal use and ceased to be recognized as a source of medicinal material. This measure was a timely response to the pandemic, aiming to signal, from the perspective of official technical standards, a lack of support for the medicinal use of pangolins amid ongoing uncertainty about zoonotic disease risks. At the same time, it also signaled that the state was likely to reassess the legal status of pangolins in the near future. As discussed earlier, since the founding of the People’s Republic of China, legislation consistently acknowledged the medicinal role of pangolins. This change would impact a series of regulations related to pangolin medicinal use. However, it does not imply a complete prohibition on pangolins as a TCM ingredient, as statutory technical standards serve merely as references and lack the compulsory enforcement power of law. A more significant change occurred on 3 June 2020, when pangolins were officially upgraded from Class II to Class I national protected animals. Unlike the Pharmacopoeia, which serves only as technical guidance, this reclassification carries binding legal force and is subject to mandatory enforcement. This legal elevation brought about the following changes: First, in terms of regulatory authority, supervision shifted from provincial, autonomous region, and municipal people’s government wildlife protection departments to the State Council’s wildlife protection department. Second, regarding the valuation of pangolins and their products, when pangolins were Class II protected animals, the value of a whole pangolin was five times the benchmark value stipulated in the “Benchmark Value Standards Catalog for Terrestrial Wild Animals.” After being upgraded to Class I protected animals, the overall value became ten times the benchmark value [8]. Third, the regulatory authority for the domestication and breeding of pangolins also changed from the provincial, autonomous region, and municipal forestry administrative departments to the State Forestry Administration, and the sale of these pangolins also required approval from the relevant departments [48]. (4) In terms of wild animal resource protection and management fees, the fee to be paid by suppliers for approved sale, acquisition, and utilization of pangolins increased from 6% to 8% of the transaction value [49]. The exclusion of pangolins and their products from statutory technical standards potentially opens the door for the legal prohibition of other wild animal products for medicinal use, such as bear paws and tiger bones, which are frequently illegally hunted and trafficked for medicinal purposes in China.

However, the cessation of legal medicinal use for pangolins cannot be achieved overnight. In November 2024, the National Forestry and Grassland Administration, the National Administration of Traditional Chinese Medicine, and the National Medical Products Administration issued a departmental normative document. Although this document also emphasized strengthening pangolin protection and strictly prohibiting the use of pangolin scales from unknown sources, it managed the medicinal use of pangolin scales from the perspective of “resource conservation” rather than outright prohibition or cessation of medicinal use [50]. This reflects a contradictory aspect in China’s legislative philosophy for pangolin protection: on the one hand, strictly protecting pangolin populations as wild animals and combating illegal activities such as hunting and illicit transport through the “Criminal Law” and “Wild Animal Conservation Law”, but simultaneously permitting the medicinal use of pangolin scales. Yet, artificial breeding of pangolins remains an immature technology globally, implying that legally used pangolin scales are either imported or still sourced from wild pangolins.

#### 3.3.2. Enforcement: Strengthened Measures and Remaining Challenges

It is unrealistic to expect the complete eradication of pangolin use in TCM within a short period, given its deep-rooted history spanning thousands of years in China. Nevertheless, the shift in the legal classification of pangolins since 2020 significantly strengthened enforcement efforts, which can be observed in several key areas:

First, there has been an intensified crackdown on organized and cross-regional pangolin-related crimes. In 2020, Chongqing Customs, in coordination with public security agencies in Nanning, Hefei, Chengdu, and other cities, dismantled a smuggling network and seized 441.03 kg of pangolin scales [51].

Second, regulatory oversight of medical institutions authorized to use pangolins has been reinforced. In May 2025, after six years of investigation, a pharmaceutical company was prosecuted for illegally selling pangolin scales. Between 2015 and May 2019, a company based in Chongqing unlawfully sold 7300 kg of pangolin products to 111 companies and hospitals across the country, profiting approximately 44 million RMB. Most of the pangolins had been illegally sourced across borders and processed, packaged, and distributed by the company. In China, the production of pangolin-based products requires administrative approval and the use of official labels, while purchasing entities must also hold appropriate permits. In this case, not only was the seller prosecuted, but many of the purchasing companies were also investigated and convicted [52].

This case highlights several key enforcement challenges in the protection of pangolins in China. First, the use of pangolins in TCM is deeply entrenched. Legally utilized pangolin materials are typically processed into scale-based decoctions, which are consumed by soaking in water or alcohol, or ground into medicinal powders.

Second, even authorized users may engage in illegal practices. Legally approved pangolin products are required to carry a “green wildlife protection label,” issued by government authorities under strict weight limits. However, violations often involve the counterfeiting or misuse of labels, falsified inventory reports, and manipulated audits. In practice, pangolin-based medicines sold in retail pharmacies rarely feature official labeling, making it difficult for consumers or inspectors to verify their legality. The complexity of the previously mentioned case—which took six years to investigate and prosecute—reveals the high degree of regulatory uncertainty in this area: even licensed pharmaceutical companies may struggle to ensure that all pangolin scales used are sourced legally. Moreover, most retailers pay little attention to whether the products they sell carry the required green labels [53].

Third, there is a serious lack of transparency in the disclosure of legally required information. Although the law mandates the public availability of data, such as licenses and special labels for the legal use of wildlife, a public interest organization that submitted information disclosure requests to all 31 provincial-level authorities in China received responses from only 17, with few providing substantive data [54].

Fourth—and perhaps most fundamentally—is the lack of sufficient funding, personnel, and technical resources within the relevant government agencies. These departments are already burdened with a wide range of duties, including inspections, planning, and document management. Additionally, geographic variation in pangolin-related crimes means that even where local governments made substantial enforcement efforts, such violations are difficult to eliminate entirely. Offenses related to pangolins are most commonly concentrated in regions such as Fang Chenggang in Guangxi Province, Guangzhou in Guangdong Province, and Kunming in Yunnan Province. One study analyzing 366 criminal cases involving wildlife smuggling from 2017 to 2021 found that 76 took place in Guangdong, 71 in Guangxi, and 56 in Yunnan [55]. The high case numbers in Guangxi and Yunnan are largely due to their proximity to China’s borders, as most pangolins involved were illegally smuggled in. Guangdong’s numbers reflect its longstanding local tradition of consuming wild animals, combined with its role as a major trade hub.

Fifth, and perhaps the most difficult to address, is the problem of corruption within the bureaucratic system. Illegal sales and processing of pangolin products by legally registered companies are often linked to corrupt practices among regulatory officials. In one case, the head of a wildlife protection station was found to have accepted bribes totaling over RMB 8 million over a 13-year period in exchange for facilitating the approval of pangolin product operations and inventory declarations for pharmaceutical companies. These approval processes are complex and document-heavy, and unless triggered by a special enforcement campaign or criminal investigation, signs of corruption are rarely detected during routine oversight. As one corporate employee reportedly stated, “As long as the money is in place, the approvals can be granted” [56].

#### 3.3.3. Ethics and Social Development in Pangolin Protection

Encouragingly, the 2020 ban on the consumption of wild animals, along with the establishment of China’s ecological civilization framework, provided strong momentum and triggered a top-down activation of societal regulatory interaction. First, public oversight has been significantly enhanced. The concept of ecological civilization reshaped public attitudes toward nature, and the COVID-19 pandemic prompted a reassessment of human–wildlife relationships. Many citizens began actively providing information to assist law enforcement and judicial authorities in combating crimes involving pangolins. In one Beijing case, a tip from the public led police to discover 1.49 tons of pangolin scales in a private villa, which eventually uncovered that a legally registered pharmaceutical company was illegally producing and selling pangolin products [57]. Some public figures also played a role in guiding public values—for example, Chinese actress Angelababy served for years as a public ambassador for pangolin protection, participating in WildAid campaigns and appealing to the public, from a mother’s perspective, to reject consumption of pangolins so that pangolin babies can grow up safely [58].

Second, NGOs found new opportunities to participate in enhancing the legal protection of pangolins. While lacking formal authority, these organizations supplemented regulatory capacity by contributing to the collection of evidence in illegal wildlife trade investigations and assisting forestry departments in rescuing live pangolins. They also played a role in monitoring law enforcement and judicial proceedings, especially in high-profile cases. For instance, after a case involving the illegal trade of over 5000 pangolins became public, a relevant NGO promptly contacted the authorities. NGO oversight also contributed to improving the wildlife rescue system, with cases of administrative and environmental public interest litigation brought against government-run wildlife rescue centers for improper handling of pangolins [59].

Third, there is growing interaction between public awareness and legal consciousness. The 2020 ban on wild animal consumption was not only a legal change, but also deeply impacted daily life. Today, restaurants across China prominently display signs prohibiting the consumption of wild animals, and government agencies regularly carry out legal education campaigns. In some cases, courts even held hearings in national parks—where the wildlife was harmed—to visually enhance public awareness [60]. This growing civic consciousness is also reflected in public responses to major legislative consultations. The 2022 draft amendment to the Wildlife Protection Law received 12,057 public comments, many of which—such as those concerning wildlife release, rescue, and information transparency—were eventually adopted [61]. Similarly, during the 40-day consultation period for the draft of the Ecological and Environmental Code conducted from April to June 2025, over 11,000 public comments were submitted, reflecting the rising ecological awareness among Chinese citizens [62].

Together, the contradictions in legislation, enforcement pressures, and the rise of public consciousness created fertile ground for the emergence and development of China’s unique public interest litigation system.

## 4. Environmental Public Interest Litigation: The Future of Pangolin Judicial Protection

### 4.1. Three Modes of Legal Protection for Pangolins in China

Legal protection for pangolins in China can be categorized into three modes: (a) Front-end protection: This involves providing more natural growth space for pangolins by protecting their habitats, safeguarding the overall natural environment, and controlling urban expansion. (b) Mid-stream prohibition: This targets indirect harmful illegal activities such as illegal consumption, sale, and transportation of pangolins. Examples include establishing a food ban system and issuing various permits to combat behaviors that could indirectly lead to a reduction in pangolin populations. The legal consequences primarily involve administrative penalties. In practice, regulation mainly focuses on pharmaceutical companies. When pangolin-based medicinal products lack the required wildlife management labels, TCM labels, or fail to prove the legal origin of pangolin ingredients, the market supervision authorities may order corrective action. Without involving criminal liability, administrative penalties, such as fines, confiscation of illegal gains, and business suspension, may be imposed. These decisions are also published online for public access [63]. For instance, the “Wild Animal Conservation Law” stipulates that those who trade wild animals or their products under the guise of rescue will be fined 2 to 20 times the value of the wild animals and their products, and their illegal information will be recorded in their social credit history and publicly disclosed [47]. (the method for calculating value will be explained in detail later). (c) End-stage deterrence: This targets direct harmful illegal activities against pangolins, primarily manifested in the design of pangolin-related offenses within the “Criminal Law.”

China made significant progress in ecological and environmental protection in recent years, greatly intensifying efforts to protect environments affecting wildlife survival, such as forests, land, and rivers [64]. Front-end protection policies for pangolins have been continuously strengthened, and pangolin habitats have seen some recovery. However, the immense economic profits behind pangolins still drive many individuals to illegally poach, transport, purchase, and sell them. There is substantial data to prove this; for instance, approximately 90,000 pangolins were illegally smuggled into China between 2007 and 2016 [65]. The channels, methods, and technologies for illegal pangolin-related crimes are also evolving. For example, in 2015, online criminal activities accounted for 20.09% of 443 illegal wildlife trade cases, which surged to 51.05% by 2019 [66]. Therefore, mid-stream prohibitions on pangolin consumption and illegal medicinal use are necessary. Since 2020, end-stage deterrence measures have also been continuously strengthened. Illegal hunting and killing of precious and endangered wild animals, as well as illegal acquisition, transportation, and sale of precious and endangered wild animals and their products, are now categorized as “crimes endangering precious and endangered wild animals.” The “Criminal Law” shifted its philosophy from “maintaining the management order of wild animal resources” (from the 1979 Criminal Law) to a “risk prevention-oriented” approach after 2020. This risk prevention is reflected in two aspects: first, preventing the extinction of wild animal populations and maintaining biodiversity; second, preventing the risk of zoonotic diseases potentially caused by illegal hunting, consumption, and transportation of wild animals. Consequently, the criminal law protection for pangolins, as an end-stage deterrence, has become stricter.

In China, crimes involving pangolins are considered “administrative offenses,” meaning actions such as hunting, transporting, or consuming pangolins first violate the “Wild Animal Protection Law” or “Environmental Protection Law”, meaning that the legislature purposely set up these punishments for managerial concerns other than purely remedying the victims of wrongful harmdoing. Once they meet the criteria for criminalization, they transition into criminal offenses. The boundary between administrative violations and criminal offenses involving wildlife is generally determined by the species involved, the extent of harm, and the means used. For pangolins—classified as precious and endangered wildlife, harming even a single individual constitutes a criminal offense. In contrast, harming wild boars or mountain goats without causing serious consequences may result only in administrative penalties [67].

Prosecutors will initiate criminal proceedings against suspects. Depending on the specific illegal and criminal acts related to pangolins, the potential charges and penalties are as follows: (1) Smuggling pangolins and their products constitutes the crime of smuggling precious animals, with a basic penalty of 5–10 years of fixed-term imprisonment and a fine; in particularly serious circumstances, the penalty is 10 years or more of fixed-term imprisonment or life imprisonment, along with confiscation of property. (2) Illegal hunting, killing, purchasing, selling, or transporting of pangolins constitutes the crime of endangering precious animals, with a basic penalty of fixed-term imprisonment of up to 5 years or criminal detention, along with a fine. Depending on the quantity and value of pangolins and their products, serious circumstances incur 5–10 years of fixed-term imprisonment and a fine, while particularly serious circumstances incur 10 years or more of fixed-term imprisonment and a fine or confiscation of property. (3) Additionally, criminals who conceal illicit gains from pangolins and their products may also be charged with the crime of concealing criminal proceeds [68]. Concurrently, China’s criminal justice system often imposes “ancillary sanctions”, such as professional prohibition, deprivation of benefits, and restriction of qualifications, in addition to basic criminal penalties, depending on the circumstances. These measures collectively constitute the deterrent actions of the “Criminal Law” against those who harm pangolins.

However, despite such a stringent criminal penalty system, why do pangolin crimes in China persist, and why are pangolin populations still sharply declining, with their habitats continuously being destroyed? The main reasons are as follows: (1) The economic profits associated with pangolin-related crimes are extremely high [69]. These crimes are typically characterized by organized syndicates, and criminals, despite knowing the criminal consequences, are willing to take risks due to economic incentives. In 2017, there was a repeat offender who was caught smuggling 1.5 tons of pangolin scales [70]. At the same time, widespread consumer misconceptions about the rarity of pangolins and the supposed medicinal or culinary value of their meat and products remain a deep-rooted reason why criminal law alone cannot eradicate pangolin-related crimes [66]. This issue is not unique to China; around the world, most illegal pangolin poaching is carried out by villagers living near pangolin habitats. Their motivations vary—from economic gain to traditional beliefs in the medicinal or dietary value of pangolins—making the drivers behind such crimes highly complex [71]. (2) Although the penalties are severe, the consequences primarily involve deprivation of personal liberty and economic sanctions against the offenders. Pangolin populations, as the victims of illegal activities, do not receive ecological remediation directly from the severity of these penalties. The last nationwide survey on the population and distribution of pangolins in China was conducted in 1998, with results released in 2003. At that time, the wild pangolin population was estimated at 60,000, with an ecological density ranging from 0.001 to 0.056 individuals per square kilometer. Years later, conducting such surveys has become nearly impossible due to the species’ extreme rarity, making it difficult to monitor individuals at fixed times and locations [72]. As a result, government agencies can only infer the distribution of pangolin populations based on sporadic sightings without access to accurate population data [73]. Thus, under China’s current three modes of pangolin legal protection, while offenders are sanctioned, this sanction is not directly linked to the ecological recovery of pangolin populations. This sanction system primarily serves as a societal deterrent rather than focusing on the specific circumstances of the pangolin species. Because both administrative and criminal sanctions target only the offender’s property, personal freedom, and social credibility, these measures cannot remedy the harm suffered by the pangolins [74].

### 4.2. Establishment of China’s Environmental Public Interest Litigation System

#### 4.2.1. Fundamental Legislation on Environmental Public Interest Litigation in China

After the amendment of the “Environmental Protection Law” in 2014, environmental public interest litigation was established [27]. The 2017 revision of the Administrative Procedure Law of the People’s Republic of China authorized the People’s Procuratorates to file public interest lawsuits against government agencies that fail to fulfill their environmental protection duties [75]. It is worth noting that China is currently the only country in the world where procuratorial organs have the authority to initiate public interest lawsuits against government departments. Furthermore, the 2019 “Several Provisions of the Supreme People’s Court on the Trial of Ecological Environment Damage Compensation Cases (for Trial Implementation)” stipulated that provincial and municipal people’s governments and their relevant departments/agencies, or departments entrusted by the State Council to exercise ownership over all citizen-owned natural resource assets, may file “ecological environment damage compensation lawsuits” against perpetrators of ecological damage [76].

After several years of development, commonly applied forms include the following: (1) environmental civil public interest litigation initiated by procuratorial organs; (2) environmental administrative public interest litigation initiated by procuratorial organs; (3) environmental civil public interest litigation initiated by social organizations; and (4) ecological environment damage compensation litigation initiated by government departments/agencies. It should be noted that these different forms of litigation may coexist. For suspects involved in smuggling or harming pangolins, they may simultaneously face criminal prosecution initiated by procuratorial organs, criminal proceedings with attached civil public interest litigation initiated by procuratorial organs, civil public interest litigation initiated by social organizations, and ecological environment damage compensation litigation initiated by the government. However, in practice, the proportion of public interest lawsuits initiated by social organizations is relatively low. Among all types of environmental public interest lawsuits, there were only 57 cases in 2017, 65 in 2018, 179 in 2019, and 103 in 2020. In contrast, environmental civil public interest lawsuits initiated by procuratorial organs were 791, 1737, 2309, and 3454 cases, respectively, for the same years [77]. Moreover, ecological environmental damage compensation lawsuits initiated by government departments are still developing and often conflict with criminal prosecutions and criminal proceedings with attached civil public interest litigation initiated by procuratorial organs in practice (e.g., the suspect may have already been subjected to compulsory measures restricting their freedom, and their property may have been frozen or confiscated). Therefore, pangolin-related ecological environmental damage compensation lawsuits are currently almost non-existent.

It should also be noted that China’s People’s Procuratorates perform three key functions: first, initiating public prosecutions in criminal cases and providing sentencing recommendations; second, filing public interest lawsuits in specific areas to safeguard public interests; and third, supervising the performance of duties by government departments and administrative agencies. In recent years, the procuratorates exercised considerable discretion and assumed a prominent role in the fields of ecological and wildlife protection.

#### 4.2.2. The Emergence of Public Interest Litigation on Wildlife Conservation

Wild animals were initially not included within the scope of environmental public interest litigation. On 27 January 2020, the Supreme People’s Procuratorate issued the “Notice on Conscientiously Implementing the Central Government’s Epidemic Prevention and Control Deployments and Resolutely Doing a Good Job in the Procuratorial Organs’ Epidemic Prevention and Control Work,” which officially encouraged procuratorial organs at all levels and localities to explore public interest litigation concerning wild animals [78].

### 4.3. Current Status of Pangolin Public Interest Litigation System

Based on the case retrieval from “China Judgements Online” and the “People’s Court Case Library” as mentioned in Section 2, Materials and Methods, the effective judicial precedents obtained for public interest litigation concerning pangolins are shown in the Table 2 (more detailed information is available in Supplementary Material S3: Full-Text Links to Judgments in Pangolin-Related Public Interest Litigation Cases in China, and Supplementary Material S4: Full Text of Judgments in Pangolin-Related Public Interest Litigation Cases in China):

**Table 2:** Pangolin-Related Public Interest Litigation Cases in China.

No.	Case No./Date	Involved Persons	Number of Pangolins	Quantity of Products (KG)	Value of Products (RMB)	Criminal Liability			Civil Liability
						Crime	Imprisonment/Probation	Fine/Confiscation	
1	(2020) Jing 04 Criminal First Instance No. 25	1	/	Pangolin scales (undisclosed quantity)	275,200	Smuggling of precious animal products	Imprisonment for 2 years and 6 months	Fine of RMB 25,000	/
2	(2021) Gui 14 Criminal First Instance No. 79	1	/	1443.16 kg pangolin scales	113 million	Smuggling of precious animal products	Life imprisonment	Confiscation of property	/
3	(2023) Yun 3123 Criminal First Instance No. 516	1	/	6.95 kg pangolin scales	1,233,984	Endangering precious and endangered species	Imprisonment for 3 years with 2 years’ probation	Fine of RMB 10,000	Compensation for wildlife resource losses of RMB 1,133,984.
4	(2023) Yue 1223 Criminal First Instance No. 80	3	/	3.03 kg Malayan pangolin scales, 1.29 kg tree pangolin scales, 0.39 kg mixed scales (Malayan, tree, giant pangolin)	244,940.61	Endangering precious and endangered species	Imprisonment for 2 years with 4 years’ probation	Fine of RMB 10,000	Compensation for wildlife resource losses of RMB 308,990.61.
5	(2022) Gui 0681 Criminal First Instance No. 3	1	/	61.35 kg Malayan pangolin scales	3,435,600	Endangering precious and endangered species (attempted)	Imprisonment for 6 years	Fine of RMB 50,000	Compensation for ecological resource damages of RMB 3,435,600 and a formal apology.
6	(2022) Liao 0112 Criminal First Instance No. 350	1	/	1.82 kg pangolin scales	116,544	Endangering precious and endangered species	Detention for 6 months with 1 year’s probation	Fine of RMB 10,000	Compensation for state economic loss of RMB 141,384.
7	(2021) Gui 0681 Criminal First Instance No. 248	1	/	0.82 kg South African pangolin scales	8640	Endangering precious and endangered species	Imprisonment for 6 months with 1 year’s probation	Fine of RMB 10,000	Compensation for ecological resource damage of RMB 8640.
8	(2021) Gan 0424 Criminal First Instance No. 397	6	2	/	160,000	Endangering precious and endangered species	Imprisonment ranging from 6 months to 1 year and 6 months	Fines ranging from RMB 4000 to RMB 8000	Compensation for wildlife resource losses of RMB 163,000.
9	(2021) Gui 0681 Criminal First Instance No. 159	1	/	0.03 kg of pangolin scales	64,000	Endangering precious and endangered species	Imprisonment for 5 years	Fine of RMB 50,000	Compensation for ecological resource damage of RMB 883,708.4 (including other wildlife species).
10	(2020) Yue 01 Civil First Instance No. 1868	1	11	/	Undisclosed	Illegal acquisition, sale of national protected wild animals	/	/	Compensation for ecological restoration costs of RMB 3,823,500 (including other animals) and a formal apology.
11	(2021) Yue 01 Civil First Instance No. 576	1	65	/	2.36 million	/	/	/	Compensation for ecological restoration costs of RMB 2.36 million (to be remitted to the state treasury for the restoration of damaged ecological environments) and a public apology.
12	(2021) Yue 01 Civil First Instance No. 577	1	80	/	1.52 million	/	/	/	Compensation for ecological restoration cost of RMB 1.52 million and a public apology.
13	(2021) Yue 1403 Criminal First Instance No. 165	1	3.27 pangolin scales	/	104,640	Endangering precious and endangered species	Imprisonment for 3 years	Fine of RMB 20,000	Compensation for wildlife resource loss of RMB 104,640 and a public apology.
14	(2021) Gui 0621 Criminal First Instance No. 53	2	1 kg of giant pangolin, South African pangolin scales	/	10,624	Endangering precious and endangered species	Imprisonment for 9 months	Fine of RMB 5000	Joint compensation for ecological resource damages of RMB 10,624.
15	(2021) Gui 0821 Criminal First Instance No. 78	1	1.95 kg pangolin scales	/	5581	Endangering precious and endangered species	Imprisonment for 2 years	Fine of RMB 100,000	Compensation for ecological damage of RMB 7753.
16	(2020) Gui 14 Criminal First Instance No. 48	7	768.56 kg pangolin scales	/	68,219,792	Smuggling of precious animal products	Imprisonment ranging from 5 to 14 years	Fines ranging from RMB 300,00 to RMB 1.5 million	Multiple offenders jointly compensated for wildlife resource losses totaling RMB 68,219,792; individual principal offenders separately compensated RMB 187,073, RMB 120,288, and RMB 3600, respectively, and issued formal apologies.
17	(2021) Lu 02 Civil First Instance No. 69 [50]	1	/	1	80,000	/	/	/	Compensation for wildlife resource losses of RMB 83,000 and ecological service function losses of RMB 907,500. Punitive compensation of RMB 99,050, of which RMB 24,924 was fulfilled through public service labor.
18	(2020) Zhe 07 Civil First Instance No. 299	1	/	8	320,000	Illegally purchasing and selling precious/rare and endangered wild animals and their products	Undisclosed	Undisclosed	Compensation for national wildlife resource loss of RMB 359,000 (including losses for other wildlife).
19	(2019) Yun 05 Criminal First Instance No. 219	7	Pangolin scales: 13.36 kg	3	3,103,680 (total value, including other wildlife species)	Smuggling precious animal products, illegally purchasing precious/rare and endangered wild animals	Imprisonment ranging from 1 to 11 years	Fines ranging from RMB 10,000 to RMB 110,000	Compensation for wildlife resource loss of RMB 3,103,680.
20	(2019) Yun 05 Criminal First Instance No. 234	1	14.1 kg of pangolin scales	/	Undisclosed	Smuggling of precious animal products	Undisclosed	Undisclosed	Compensation for wildlife resource loss of RMB 1,014,080 and a public apology.
21	(2020) Su 0102 Criminal First Instance No. 370	6	Five pangolin scale items (weight not disclosed)	/	60,000	Endangering precious and endangered species	Imprisonment ranging from 1 year and 3 months to 1 year and 6 months	Fines ranging from RMB 7000 to RMB 20,000	Compensation for pangolin-related damages of RMB 60,000 and a formal apology.
22	(2020) Su 0508 Criminal First Instance No. 1159	1	/	5	200,000	Endangering precious and endangered species	Imprisonment for 2 years	Fine of RMB 20,000	Compensation for wildlife resource loss of 200,000 and a public apology.
23	(2020) Min 0881 Criminal First Instance No. 278	1	/	7	280,000	Illegal Acquisition and sale of precious and endangered wild animals and their products	Imprisonment for 12 years and 8 months	Fine of RMB 400,000	Compensation for wildlife resource loss of RMB 285,000 and a public apology.
24	(2020) Min 0212 Criminal First Instance No. 246	1	/	1	40,000	Illegal acquisition and sale of precious and endangered wild animals and their products	Imprisonment for 1 years and 4 months with 2 years’ probation	Fine of RMB 5000	Compensation for ecological loss of RMB 40,000.
25	(2020) Yun 3103 Criminal First Instance No. 359	1	/	1	40,000	Illegal transportation of precious and endangered wild animal products	Imprisonment for 6 months	Fine of RMB 4000	Settlement reached; ecological restoration achieved through alternative forms of liability.
26	(2020) Yun 0422 Criminal First Instance No. 113	2	/	1	40,000	Smuggling of precious animal products	Imprisonment for 1 year with 1.5 years probation	Fine of RMB 2000.	Compensation for wildlife resource loss of RMB 40,000.
27	(2020) Yue 0204 Criminal First Instance No. 213	3	1.369 kg pangolin scales	1	105,554.67	Illegal acquisition and transportation of precious and endangered wild animals	Imprisonment ranging from 1 to 2 years	Fines ranging from RMB 10,000 to RMB 20,000	Compensation for ecological resource loss of RMB 80,000.
28	(2020) Min 0881 Criminal First Instance No. 197	1	/	1	40,000	Illegal acquisition of precious and endangered wild animal products	Imprisonment for 6 months with 1 year’s probation	Fine of RMB 40,000	Compensation for wildlife resource loss of RMB 40,000 and a public apology.
29	(2020) Min 0881 Criminal First Instance No. 180	1	/	1	40,000	Illegal acquisition of precious and endangered wild animal products	Imprisonment for 6 months with 1 year’s probation	Fine of RMB 40,000.	Compensation for wildlife resource loss of RMB 40,000 and a public apology.
30	(2020) Yue 1323 Criminal First Instance No. 473	4	/	2	80,000	Illegal hunting of precious and endangered wild Animals	Imprisonment ranging from 6 to 9 months	Fine of RMB 10,000.	Compensation for wildlife resource loss of RMB 80,000, RMB 6900 for appraisal and a public apology.
31	(2020) Min 0881 Criminal First Instance No. 159	1	/	1	40,000	Illegal acquisition of precious and endangered wild animal products	Imprisonment for 6 months with 1 year’s probation	Fine of RMB 40,000.	Compensation for wildlife resource loss of RMB 40,000 and a public apology.
32	(2020) Yue 0303 Criminal First Instance No. 265	5	/	18	Undisclosed	Illegal acquisition of precious and endangered wild animals	Imprisonment ranging from 1 year and 1 month to 10 years	Fines ranging from RMB 5000 to RMB 10,000	Voluntary payment of compensation and settlement reached (compensation amount undisclosed).
33	(2020) Gan 1002 Criminal First Instance No. 201-2	1	/	1	40,000	Illegal acquisition of precious wild animals	Imprisonment for 10 months	Fine of RMB 40,000	Compensation for wildlife resources loss of RMB 48,260 (including other animals).
34	(2019) Gan 0732 Criminal First Instance No. 265	1	/	43	1.565 million	Illegal acquisition and sale of precious and endangered wild animal products	Imprisonment for 3 years	Fine of RMB 70,000	Compensation for wildlife resources loss of RMB 364,000.
35	(2020) Chuan 0106 Criminal First Instance No. 335	4	0.04 kg of pangolin scales	/	2720	Illegal acquisition and sale of precious and endangered wild animal products	Imprisonment ranging from 10 months to 1 year and 6 months	Fines ranging from RMB 3000 to RMB 8000	Compensation for wildlife resource loss of RMB 22,440 and a formal apology.
36	(2020) Yun 0927 Criminal First Instance No. 52	1	0.86 kg of Malayan pangolin scales	/	76,352	Illegal acquisition, transportation, and sale of precious and endangered wild animal products	Imprisonment for 5 years and 6 months	Fine of RMB 20,000	Compensation for ecological loss of RMB 126,996 (including other animals) and a public apology.
37	(2020) Yun 2901 Criminal First Instance No. 116	1	0.637.kg of pangolins scales	/	43,520	Illegal acquisition of precious and endangered wild animal products	Imprisonment for 6 months	Fine of RMB 10,000	Claim dismissed due to no ecological/public harm.
38	(2020) Gan 0222 Criminal First Instance No. 24	4	Illegal excavation, claimed to be searching for pangolins Excluded as it was not a true pangolin-related offense	/	/	Crime of intentionally damaging cultural relics	/	/	/
39	(2019) Yue 0303 Criminal First Instance No. 855	1	/	10	undisclosed	Illegal acquisition of endangered wild animals	Imprisonment for 5 years	Fine of RMB 10,000	/
40	(2019) Yun 2627 Criminal First Instance No. 357	1	0.637 kg Pangolin scales	/	43,520	Illegal acquisition of precious and endangered wild animal products	Imprisonment for 9 months with one year’s probation	Fine of RMB 1000	The court found that the plaintiff’s mere act of purchasing did not harm ecological and environmental interests, and therefore reduced the compensation to RMB 1000 at its discretion.
41	(2019) Yun 2627 Criminal First Instance No. 358	1	2.312 kg pangolin scales	/	158,400	Illegal acquisition of precious and endangered wild animal products	Imprisonment for 3 years with 4 years’ probation	Fine of RMB 1000	The court found that the plaintiff’s mere act of purchasing did not harm ecological and environmental interests, and therefore reduced the compensation to RMB 2000 at its discretion.
42	(2019) Yun 2627 Criminal First Instance No. 361	1	0.574 kg pangolins scales	/	39,360	Illegal acquisition of precious and endangered wild animal products	Imprisonment for 8 months with 1 year’s probation	Fine of RMB 1000	The court found that the plaintiff’s mere act of purchasing did not harm ecological and environmental interests, and therefore reduced the compensation to RMB 1000 at its discretion.
43	(2019) Yun 2627 Criminal First Instance No. 362	1	0.67 kg pangolin scales	/	37,440	Illegal acquisition of precious and endangered wild animal products	Imprisonment for 8 months with 1 year’s probation	Fine of RMB 1000	The court found that the plaintiff’s mere act of purchasing did not harm ecological and environmental interests, and therefore reduced the compensation to RMB 1000 at its discretion.
44	(2019) Qian 2722 Criminal First Instance No. 232	1	/	2	80,000	Illegal acquisition of precious and endangered wild animal products	Imprisonment for 1 year	Fine of RMB 5000	Compensation for national resource loss of RMB 80,000.
45	(2019) Zhe 0781 Criminal First Instance No. 445	2	/	1	20,000	Illegal acquisition of precious and endangered wild animals; illegal sale of precious and endangered wild animals	Imprisonment ranging from 10 months to 1 year and 6 months	Fines ranging from RMB 15,000 to RMB 20,000	Voluntary payment of RMB 20,000 in public interest litigation compensation.
46	(2019) Yun 3102 Criminal First Instance No. 389	3	/	1	Undisclosed	Illegal hunting and killing of precious and endangered wild animals	Exempted from criminal punishment	/	Compensation for wildlife resource loss of RMB 40,000.
47	(2019) Gui 0902 Xing Chu No. 669	2	0.22 kg pangolins scales	10	Undisclosed	Illegal acquisition, transportation, and sale of precious and endangered wild animals and their products	Imprisonment ranging from 3 years to 5 years and 6 months	Fines ranging from RMB 20,000 to RMB 50,000	A formal apology.
48	(2020) Yun 0428 Criminal First Instance No. 126	2	1.905 kg of pangolin scales	/	106,240	Illegal sale of precious and endangered wild animal products	Imprisonment ranging from 3 years to 5 years	Fines ranging from RMB 10,000 to RMB 20,000	Compensation for wildlife resource loss of RMB 106,240.
49	(2020) Yue 1422 Criminal First Instance No. 85	2	/	1	40,000	Illegal acquisition of precious and endangered wild animals; illegal sale of precious and endangered wild animals	Imprisonment for 8 months with 1 year’s probation	Fine of RMB 2000	Compensation for ecological loss of RMB 40,000 and a public apology.
50	(2018) Yun 0428 Criminal First Instance No. 148	3	Suspected pangolin powder, porcupine spine powder, elephant skin powder(weight undisclosed)	/	Undisclosed	Illegal acquisition and transportation of precious and endangered wild animal products	Undisclosed	Undisclosed	Compensation for wildlife resource loss of RMB 14,747.

These data were derived from the following resources available in the public domain: China Judgments Online at http://wenshu.court.gov.cn (accessed on 16 August 2025); Case Library of the Supreme People’s Court at https://rmfyalk.court.gov.cn/ (accessed on 16 August 2025).

#### 4.3.1. The Increasing Prevalence of Public Interest Litigation in Pangolin-Related Cases

**Figure 1:**
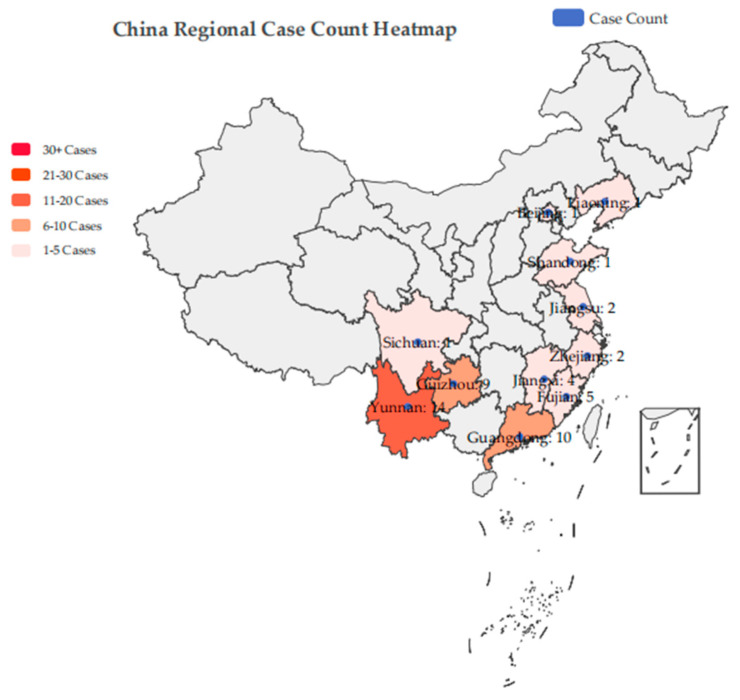
Heatmap of regional case counts for pangolin-related public interest litigation in China.

Public interest litigation cases concerning pangolin protection emerged in 2018 and since significantly increased due to a clear nation-wide judicial policy concentration after 2020, becoming a powerful tool for pangolin protection in a legal sense. The incidence patterns of public interest litigation often overlap with those of general criminal prosecutions, as most of the aforementioned cases are civil public interest lawsuits concurrently filed with criminal proceedings. The legal basis for this type of litigation is Article 97 of the “Procedural Rules for Public Interest Litigation by People’s Procuratorates,” implemented in 2021, which states the following: “When prosecuting criminal cases, if people’s procuratorates discover illegal acts that damage ecological environment and resource protection, infringe upon the legitimate rights and interests of numerous consumers in the food and drug safety sector, violate the legitimate rights and interests of minors, or infringe upon the names, portraits, reputations, or honors of heroes and martyrs, among other acts that harm public social interests, they may file criminal incidental civil public interest lawsuits with the people’s courts” [79]. Consequently, provinces such as Yunnan (cases with “Yun” in the case number), Guangxi (cases with “Gui”), and Guangdong (cases with “Yue”) have a higher number of public interest litigation cases, also because these areas are major habitats for wild pangolins in China. For clarity, Figure 1 presents case incidence overlaid on a map of China’s provincial structure:

Public interest litigation for pangolin protection is no longer a measure reserved only for particularly grave offenses; after 2020, it has been applied to nearly all illegal acts targeting pangolins. Criminal prosecution, as a traditional end-stage deterrent, imposes penalties of imprisonment and fines on offenders to deter and sanction them, simultaneously impacting their liberty and property. The specific crimes evolved from illegal transportation, acquisition, sale, and smuggling of precious and endangered wild animals to the “Crime of Endangering Precious and Endangered Wild Animals” after the 2021 amendment to the “Criminal Law.” Concurrently, incidental civil public interest litigation is broadly consistent with general civil public interest litigation, with liability consequences including paying ecological damage fees and offering public apologies or directly undertaking ecological restoration.

#### 4.3.2. Valuation of Involved Pangolins and Case Handling Procedures

Given that pangolin deaths are irreversible and artificial breeding cannot replenish pangolin populations, the public interest litigation liability for pangolin protection generally involves paying a certain fee for restoring pangolin habitats or other actions beneficial to pangolins and the overall ecological environment (the nomenclature for this fee may vary in judgments, such as “wildlife resource loss fee,” “ecological resource damage fee,” or “ecological compensation fee”). However, the current management of these ecological restoration fees by the court system is not yet standardized; some funds are directly deposited into the national treasury, others are managed by environmental protection departments, and some are transferred to ecological restoration fund accounts established by local governments. Funds typically deposited into the national treasury are converted into local government fiscal funds, which are not always exclusively allocated for ecological restoration [80]. Defendants are also sometimes required to issue public apologies (either during sentencing or through newspapers and media).

**Figure 2:**
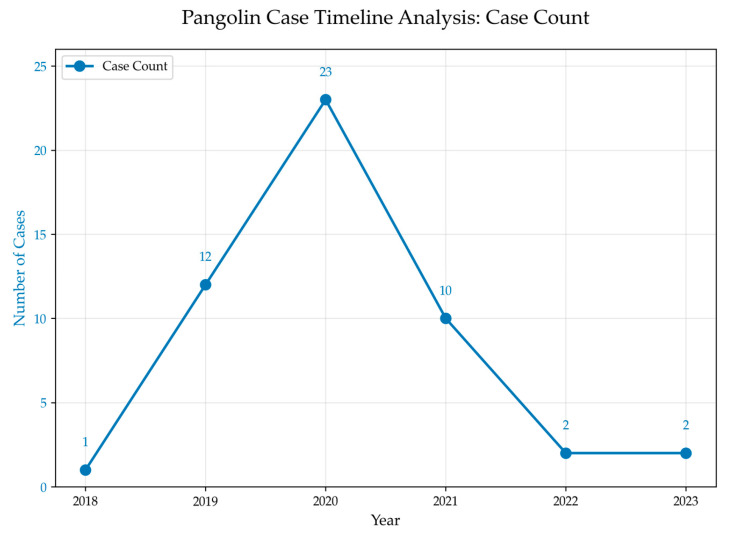
Timeline of case counts for pangolin-related public interest litigation in China.

Precisely because the destruction of individual wild animals and populations in public interest litigation for wildlife species protection cannot be fully reversed by the defendant’s remedial actions, the amount of ecological damage compensation in judicial rulings effectively reflects the judiciary’s valuation of pangolins. The courts determine the value of pangolins through the following steps: First, regardless of whether the illegal act targeting pangolins involves capture, acquisition, transportation, killing, or sale, as long as the act could directly or indirectly lead to the death of pangolins, criminal proceedings and incidental public interest litigation will be initiated against the suspect. Social organizations may also file separate civil public interest lawsuits. Second, calculating the value of pangolins is proven to be deceased based on evidence. If the seized pangolins are alive, no ecological environment resource damage is presumed; for example, in Case No. 11, the judge only recognized the calculation for 59 deceased pangolins, and 6 individuals whose status (dead or alive) could not be determined were not included in the ecological value loss. If it involves scales, flakes, or pangolin products, their value is converted; for instance, 495.38 g of pangolin scales in Case No. 26 were converted to represent the loss value of 1 pangolin. Third, by converting the calculated pangolin value into an ecological resource loss fee, demanding compensation from the defendant. The 50 identified cases have been organized by year, as shown in Figure 2:

As shown, 2018 marked the starting point of pangolin-related public interest litigation, with only one recorded case. This reflects the flexibility and innovation of local judicial practices at the time, as national judicial policies had not yet explicitly permitted public interest lawsuits for harm to wildlife. In 2020, following the national ban on the consumption of wild animals and supportive judicial policies, many procuratorates actively initiated civil public interest lawsuits in response to the government’s heightened attention to pangolin protection. The subsequent decline in case numbers does not indicate a decrease in public interest litigation activity, but is primarily due to the judicial system no longer requiring the strict uploading of all cases to the China Judgments Online database. Notably, many of these cases involved live pangolins, raising further questions about the appropriate handling and disposition of rescued animals.

### 4.4. The Valuation of Pangolins: From Individual Economic Value to Holistic Ecological Functional Value

#### 4.4.1. Changes in Judicial Valuation of Pangolins in Actual Cases

“Ecological civilization” reshaped China’s legal approach to nature, with a “harmonious coexistence between humanity and nature” becoming a guiding principle for China’s modernization drive [81]. The value of all natural beings, including pangolins, is gradually gaining recognition. The determination of pangolin value not only influences the amount of ecological restoration fees in public interest litigation, but also dictates the severity of criminal penalties in traditional criminal proceedings. For instance, in “crimes endangering precious and endangered wild animals,” if the value of illegally hunted animals exceeds RMB 2 million, it will be deemed “particularly serious,” leading to an increase in sentence from under 5 years to over 10 years of imprisonment [82].

The basis for determining the value of wild animals in cases is the 2017 “Methods for Value Assessment on Wild Animals and Their Products”. Article 4 of this document stipulates the following: “National Class I protected wild animals shall be valued at ten times the benchmark value listed for wild animals; National Class II protected wild animals shall be valued at five times the benchmark value listed for wild animals” [8]. The “Benchmark Value Standards Catalog for Terrestrial Wild Animals” sets the value of a pangolin at RMB 8000 per individual [83]. The catalog is formulated by the State Forestry Administration, with input from the departments of agriculture and rural affairs, natural resources, science and technology, ecology and environment, and health. China’s administrative system is characterized by specialized and finely divided responsibilities among government agencies, and the formulation of major documents or legislation typically involves interdepartmental coordination. After 2020, pangolins were upgraded from Class II to Class I protected wild animals; thus, the judicial valuation of a single wild pangolin in cases changed from the previous “8000 yuan/individual × 5 = 40,000 yuan” to “8000 yuan/individual × 10 = 80,000 yuan.” This change is clearly reflected in cases; for example, in Case No. 22 (2020), the value of a pangolin was assessed by the judge as RMB 40,000. In Case No. 8 (2021), the court ruled that the defendant’s actions of endangering two pangolins required them to pay a wildlife resource loss fee of RMB 80,000 per individual. As most seized items in cases are pangolin scales, the aforementioned document considers scales to be the most economically valuable part of a pangolin, and their removal usually leads to the pangolin’s death. Therefore, the value of pangolin scales is determined to be 80% of a single pangolin’s value. In individual cases, this valuation process is typically conducted by the State Forestry Administration’s Forest Public Security Identification Center, university teams, or technical institutions.

#### 4.4.2. The Application of Punitive Damages in Pangolin-Related Cases

Beyond the formal launch of environmental public interest litigation for pangolin protection and the adjustment of pangolin’s protection level after 2020, Case No. 17, as a reference case, warrants attention. The defendant, a shop owner, purchased rat snakes, bear paws, and pangolins in 2018 to make and sell food products. The court ruled that the defendant’s purchase “caused a reduction in the population of precious and endangered wild animals and damaged regional biodiversity and the balance of the natural ecological environment.” After 2020, in addition to hunting and killing pangolins, any indirect harmful acts, such as purchasing, selling, or consuming, will be considered direct destructive acts against pangolin populations. This differs from judicial interpretations prior to 2020. In Cases No. 40–43, courts in the Yunnan region held that the defendants’ mere acquisition of pangolins did not harm ecological interests. Therefore, while public interest litigation was supported, the compensation was not based on the full value loss of pangolins, but was discretionarily reduced to an ecological damage fee of RMB 1000–2000. Case No. 17 being selected as a “reference case” indicates an internal requirement within Chinese courts for judges to refer to its reasoning when adjudicating similar pangolin cases, including the determination of harmful acts and value calculation. A unique aspect of this case was the application of punitive damages in pangolin protection. Article 1232 of the “Civil Code of the People’s Republic of China” stipulates the following: “If an infringer intentionally pollutes the environment or damages the ecology in violation of legal provisions, causing severe consequences, the infringed party has the right to claim corresponding punitive damages” [84]. Punitive damages are an additional penalty imposed to further protect the ecological environment. In Case No. 17, the defendant was required to bear not only the wildlife loss fee RMB 99,050 in punitive damages. The case ultimately concluded through negotiation, with the defendant ordered to offset the punitive damages through public service labor. Another notable aspect is that, in addition to the aforementioned fees, the defendant in this case was also required to pay RMB 907,500 for “ecological environmental service function loss,” a sum significantly greater than the wildlife loss fee and punitive damages. The judge’s reasoning for this substantial payment was that “one pangolin can protect 250 mu of forest from termite infestation... the killing of pangolins led to a massive reproduction of termites, an increase in ant colony density, which intensified harm to local trees and forests, affecting the structure and function of the regional ecosystem.” Case No. 17 being chosen as a reference case demonstrates two judicial shifts in pangolin protection: (a) those who harm pangolins will be subject to punitive damages; and (b) in public interest litigation, in addition to the 10-times value loss fee for the pangolin’s demise, there may also be a requirement to bear ecological service function loss fees, which can be dozens of times higher than the individual pangolin’s RMB 8000 value.

#### 4.4.3. Emerging Expert Evaluation Models for Pangolin Valuation

The typical significance of this case represents the judiciary moving away from viewing pangolins merely as economic resources and instead adopting a holistic ecological protection perspective towards harmed pangolins. In a public interest litigation case initiated by a Chinese NGO against pangolin trading activities [7], the organization commissioned experts to conduct assessments and provided a detailed calculation method for ecological service functions: (a) Economic value of pangolins: 1. value of an adult individual pangolin V1 = RMB 10,000 (market price); and 2. reproductive value of an adult female pangolin V2 = RMB 100,000 (calculation basis: pangolins can live for over 20 years, conservatively estimated average female lifespan is 15 years, reproductive lifespan around 10 years, producing 1 litter per year, 1 offspring per litter, totaling 10 litters and 10 offspring in 10 years, calculated at RMB 10,000 per offspring, totaling RMB 100,000). (b) Ecological value of pangolins: 1. value of each individual protecting forests from termite infestation V3 = RMB 450,000 (calculation basis: 1 pangolin can protect 250 mu of forest from termite infestation annually; if this work was carried out manually, it would cost RMB 30,000 per person annually for labor and materials, assuming an average pangolin lifespan of 15 years); and 2. total resource value created by one pangolin in its lifetime V = V1 + 1/2 × V2 + V3 (assuming a sex ratio of 1:1) = 1 + 1/2 × 10 + 45 = RMB 510,000.

Although this calculation method was not fully accepted in the first instance of the court’s ruling, which only awarded RMB 1670 in compensation in 2020, the case saw a final judgment on appeal in December 2021, ultimately resulting in an award of RMB 80,000 in ecological damage compensation, calculated as 10 times the value of a pangolin as a Class I protected animal [85]. Despite this specific calculation method not being fully adopted in that case, Case No. 17’s substantial ecological service function loss fee and the judge’s reasoning suggest that the judge might have referenced the view that “one pangolin can protect 250 mu of forest from termite infestation.” Furthermore, China’s Supreme Judicial Organs also issue “guiding cases,” which have stronger guiding efficacy, and generally, local courts are not allowed to deviate from the content of guiding cases in adjudicating similar cases. On 28 February 2020, the Supreme People’s Procuratorate issued six guiding cases for public interest litigation in wildlife protection, the first of which concerned pangolins: In June 2018, Yuan Moumou, Du Moumou, and 21 others illegally acquired and sold 11 pangolins and their products, which are national key protected precious and endangered wild animals. After being subjected to criminal compulsory measures by the public security organs for suspected illegal acquisition and sale of precious and endangered wild animals and their products, the procuratorate deemed that they “had intent and actions of infringement, caused damage to wildlife resources and the ecological environment, and harmed public social interests.” Therefore, the procuratorate simultaneously filed a civil public interest lawsuit against them, demanding they pay an RMB 880,000 wildlife resource compensation fee [34].

### 4.5. Other Issues in the Empirical Analysis of Pangolin Judicial Protection

Beyond the shifts in judicial policy for pangolin protection, the collected cases also reveal other issues occurring in individual instances.

#### 4.5.1. Reduction in Large-Scale Organized Crime

This is linked to the establishment of official ecological accountability and environmental inspection systems. It can be observed that organized pangolin-related criminal activities are gradually decreasing. This is attributed to the intensified protection efforts by relevant departments. China now established an ecological and environmental inspection and accountability system, under which government officials must be held responsible for the environmental quality within their jurisdiction, including wild animal species. Environmental protection outcomes are also incorporated into their performance evaluations, and central and superior authorities conduct inspections of local officials’ ecological environmental protection efforts. In April 2025, the promulgation of the “Ecological Environment Protection Inspection Regulations” further strengthened the implementation of this system [86].

#### 4.5.2. Consumption and Medicinal Use Remain Mainstream Motives

In most of the non-gang-related cases we collected, the purpose of hunting or purchasing pangolins was still for consumption or medicinal use, and the cultural literacy of the offenders was generally not high. The high ecological compensation demands in public interest litigation will serve as a significant deterrent to them, and when the same pangolin is involved in different cases, each offender is required to bear this compensation (e.g., Cases No. 28 and No. 29 involved the same pangolin, with compensation demands applied to each offender).

#### 4.5.3. Issues in Pangolin Rescue and Management

During the case collection process, we noted that the management of wild pangolin rescue stations is not standardized. For example, in Case No. 44, after the police apprehended the criminals, two pangolins were sent to a local rescue station, where one later died and another went missing. Chinese public interest organizations also filed lawsuits against the Guangxi Terrestrial Animal Protection Center. In 2017, the Guangxi Maritime Police sent 32 seized pangolins to this rescue center, but all these quarantined and qualified pangolins died within two months. The public interest organization sued, arguing negligence on the part of the rescue center. However, the case was ultimately dismissed because the “subject of the lawsuit did not fall within the scope of public interest litigation” [35]. Furthermore, Case No. 30 is also worth mentioning. The criminal motive in this case differed from consumption or medicinal use. After meeting a renowned pangolin researcher, the offender actively sought out and hunted pangolins with the intent to profit from the researcher’s future establishment of a pangolin breeding base.

## 5. The Future of Legal Protection for Pangolins in China

Through the preceding sections, we thoroughly reviewed the past and present of pangolin legal protection within China’s legal system. Over the past few decades, China’s legal framework, in principle, defined pangolins as a wild animal resource with high economic value, deeply intertwined with the TCM industry. For instance, a 1983 document explicitly pointed out the high demand, both official and folk, for pangolins for medicinal use [87]. Subsequently, a collaborative model of species management and natural habitat protection was initiated, but the medicinal and consumptive use of individual pangolins was not legally prohibited, and national policy consistently acknowledged the existence of pangolin medicinal use. The “Criminal Law” and “Wild Animal Conservation Law” continuously strengthened the wildlife protection system, establishing methods for overall wildlife protection such as habitat monitoring, population monitoring, national parks, quarantine systems, and wild animal resource surveys. However, a contradictory aspect simultaneously exists: various legal measures concerning pangolins were aimed at standardizing their use as a medicinal resource, with protection serving merely as a means for their sustainable utilization. Hence, we observe a series of contradictory measures: the cessation of pangolin hunting permits in 2007, yet the inclusion of pangolins in the “Pharmacopoeia of the People’s Republic of China” in 2015; and the removal of pangolins from the “Chinese Pharmacopoeia” in 2020, yet their continued management as a clinical resource in 2024. This dualistic characteristic constitutes the fundamental nature of pangolin protection-related legislation. A complete legislative ban on the resource-based utilization of pangolins remains a distant prospect in the future. Currently, China has begun to encourage research into pangolin substitutes for medicinal use and manages existing pangolin scales under principles of “strict control and conservation.” With the further advancement of ecological civilization construction, we anticipate that pangolins, as objects of resource utilization, will legislatively undergo a complete transformation to be protected as animals with crucial functions for biodiversity.

Not only in China, but globally, the inherent lag of legislation and the lack of enforcement strength and capacity remain fundamental challenges faced by both pangolin conservationists and government authorities [39]. Globally, there are eight recognized pangolin species, with four occurring across 17 range states in Asia and four across 31 range states in Africa [88]. The conservation and regulatory frameworks for these species vary significantly between jurisdictions, shaped by divergent legal traditions, historical contexts, and enforcement capacities. In Asia, for example, jurisdictions influenced by both common law and civil law traditions—such as Nepal and China—adopt markedly different approaches when facing similar challenges. While China’s pangolin protection evolved through an increasingly active judicial framework, including the integration of ecological valuation into public interest litigation, Nepal’s system reflects a more recent statutory consolidation under the CITES Act of 2017, layered upon earlier wildlife laws. Field-based evidence from Nepal indicates persistent enforcement limitations despite progressive legal provisions, with localized socio-economic drivers and weak deterrence enabling continued illegal trade [26,71]. Comparative analysis demonstrates that understanding a country’s legal heritage and the operational realities of its enforcement institutions is a necessary first step toward building collaborative, cross-jurisdictional strategies for pangolin protection—particularly in the face of transboundary trafficking pressures (even before joining CITES, Nepal had collaborated with India—another neighbor country with strong common law tradition presence—to combat internal and transboundary wildlife crime) [39].

As vital actor in pangolin protection in Asia, China’s judiciary played an extremely effective role in ecological and environmental protection in recent years; it is certainly no longer a “castle in the desert” [64], but rather “the sharpest teeth” against environmental destroyers. Beyond the judiciary’s active involvement in pangolin protection and its continuous innovation of new litigation forms and liability types to strengthen individual and species protection for pangolins, a series of promising initiatives are currently underway.

### 5.1. Establishment of “Ecological Judicial Restoration Bases”

Recently, some courts innovated a new approach: after adjudicating an environmental case, they establish an “ecological judicial restoration base” in the damaged area. Courts and environmental protection departments delineate a specific region, requiring defendants to carry out various ecological restoration measures within this area, commonly including restocking (e.g., fish) or afforestation. Currently, for illegal hunting of birds, fish, and other wild animals, some courts, in conjunction with environmental protection departments, established specialized animal protection bases, requiring defendants to offset their ecological restoration liabilities by serving as animal guardians or patrollers at these sites. For instance, Shanghai established the “Chinese Sturgeon Nature Reserve Ecological Judicial Protection Base” specifically for the protection of Chinese sturgeons and their active river basins. Hunan established wild animal rescue bases, managed by ecological damage fees paid by defendants, equipped with professional rescue personnel, and so forth. In other cases, various monetary payments from defendants are directly transferred to wild animal rescue stations or zoos that require financial support, with these locations then designated as bases for standardized management [89]. This approach combines natural protected area governance with species protection, reflecting the current Chinese judiciary’s principle of “maximizing benefits for ecological environmental protection.”

### 5.2. Recent Promulgation of the “Ecological Environment Code (Draft)”

China’s “Ecological Environment Code (Draft)” has recently been content-available online for public discussion. This 1188-article code further strengthens the environmental protection obligations of China’s legislative, administrative, and judicial organs. Article 817 stipulates the government’s obligation for wild animal rescue and shelter and explicitly prohibits the buying and selling of wild animals and their products under the guise of wild animal shelter and rescue. Furthermore, it outlines a series of measures beneficial to pangolin protection, such as Article 828 concerning emergency protection for endangered wild animals and Article 822 on international cooperation for wildlife protection [90]. The eventual promulgation of the “Ecological Environment Code” will upgrade China’s environmental protection legal system through codification, and pangolins, as important wild animals, will be integrated into a more systematic and comprehensive protection framework.

### 5.3. Improvement of the Public Interest Litigation System

China’s public interest litigation system is undergoing rapid development. In addition to the criminal incidental civil public interest litigation collected above, it has now been established that civil public interest litigation can be separately initiated for actions that do not constitute a crime but may harm pangolin species. Concurrently, procuratorial organs can initiate administrative public interest lawsuits against government departments that are ineffective in protecting pangolins and other wild animal species to supervise their fulfillment of duties. China’s judicial system will become a powerful practical path for pangolin protection. It is important to note that the evolution of China’s environmental public interest litigations (EPIL) has, from its inception, been informed by sustained legal knowledge exchange and mutual learning with other jurisdictions long before recent international advice on this matter emerged [91]. Comparative insights from systems such as the U.S. public trust doctrine and Germany’s association standing model shaped China’s adoption of the “objective legality” approach, influencing both the scope of eligible plaintiffs and the balance between administrative authority and NGO participation [92,93].

### 5.4. Optimizing Judicial Protection of Pangolins in China

China’s judicial protection of pangolins still has areas requiring coordination and improvement. First, it can be observed that the application of compensation types in case judgments is not uniform. Some cases only require compensation for pangolin resource loss, while others additionally impose punitive damages and ecological service function loss fees. These latter two fees are substantially larger than the calculated value of pangolins themselves. To ensure legal uniformity and fairness, relevant documents should be formulated to standardize the use of these amounts. Second, there is a lack of unified authoritative opinions in determining the loss value of pangolins. In the cases above, some valuations were conducted by official government-affiliated appraisal agencies for the pangolins and their products involved, while others commissioned appraisal organizations or scientific research institutions, and some appraisers did not possess government-recognized qualifications. Additionally, the participation of public interest organizations remains weak. Among the cases collected in this paper, almost all cases were civil public interest lawsuits initiated incidentally by procuratorial organs, with very few cases initiated by public interest organizations, and most of those were dismissed. Public interest organizations can leverage public participation and also form “third-party oversight” to avoid issues of internal benefit ties within government departments; for example, by overseeing official wild animal shelter and rescue efforts. Lastly, when protecting pangolins through judicial means, attention should be paid to developing different governance approaches for minor and major offenses. For those with insufficient awareness of illegality and minor criminal circumstances, the focus can be on education, requiring offenders to offset their monetary liabilities by serving as wildlife guardians or patrollers. For serious organized crimes, however, the proportion of punishment needs to be further increased, and such penalties should not be offset by other means. This is because these offenders act with malicious intent, and their organized crimes are catastrophic to pangolin populations, as seen in Case No. 2 and Case No. 16, where the amounts involved reached tens of millions of yuan, and the quantity of pangolin products converted to pangolin numbers almost equals the total population of pangolins in a region.

China’s pangolin protection is gradually moving towards overcoming past legislative vacillation through the strengthening of judicial measures. It demonstrates a severe crackdown and punitive approach against those who harm pangolins, forming a multi-layered and superimposed protection system. The characteristics of this protection system include the following: (a) focusing on individual pangolins and their populations, protecting both their individual and population ecological values; (b) covering the entire chain of pangolin-related crimes, where both direct hunting/killing and indirect acts such as purchasing or consuming are considered “harming pangolins” in the eyes of judges; and (c) targeting not only criminals, but also the performance of governmental duties. This protection model will define the future of pangolin protection in China, ultimately aiming to achieve the ecological civilization goal of “harmonious coexistence between humanity and nature.” However, legal efforts primarily focused on shaping public attitudes toward banning the consumption and use of pangolins, as well as driving institutional changes to strengthen regulatory enforcement. It remains difficult to quantify the direct impact of these legal measures on pangolin populations. Nevertheless, there are signs of improvement. In 2023, the National Forestry and Grassland Administration reported significant improvements in the quality and connectivity of wild pangolin habitats, and monitoring in 2025 showed an expansion in the distribution range of wild pangolins in Guangdong Province [94].

## 6. Conclusions

China’s ecological civilization policy is progressively integrating nature into the nation’s legal system, which is fundamentally manifested through institutional design and legal enforcement rooted in an ecological holism. Pangolins consistently remained at the core of China’s wildlife crime chain, stemming from the dual influences of a traditional practice of consuming pangolin meat and their use as TCM ingredients. This study reveals that, initially, pangolins in China were merely regarded as wild animal resources awaiting exploitation. Legal frameworks were designed primarily to regulate hunting and utilization, largely neglecting the holistic protection of pangolin habitats and populations. Consequently, pangolin-related laws during this period should be characterized as ‘utilization management laws’ rather than ‘conservation laws’. From the late 20th century to 2020, accompanied by the establishment of a modern environmental legal system and the articulation of principles such as ecological priority and species protection, the legal framework governing pangolin protection in China gradually evolved. Although the legal medicinal use of pangolins and persistent illicit consumption remained, elements of a protective legal approach were progressively incorporated into the system. During this period, primary reliance was placed on the enforcement activities of administrative agencies, where police pursuit, prosecutorial actions, and judicial rulings by courts targeted illegal hunting and other criminal behaviors, imposing terminal sanctions through the deterrent power of criminal law. However, within this phase, the ecological value of pangolins remained unrecognized, and their worth in legal contexts could only be reflected through simplistic calculative standards. A value of RMB 8000 per individual clearly did not correspond to pangolins’ crucial role in China’s biodiversity and ecosystems. Consequently, criminal penalties alone have proven insufficient in deterring the hunting and consumption of pangolins. In many cases, pangolins ultimately enter the legal medicinal market through mechanisms of post hoc legitimization. With the deepening of ecological civilization system reforms and renewed legal attention to wildlife after 2020, pangolins, as a representative protected species, reflect the innovation within China’s wildlife protection legal framework. The ecological value of wild animals has become a key consideration. Furthermore, through the refinement of environmental public interest litigation, China’s judicial organs evolved from ‘castles in the desert’ into ‘claws and teeth,’ simultaneously punishing offenders and providing remedies for pangolins and their populations in the crackdown on illegal activities, thereby further protecting the overall ecological environment. While some deficiencies persist, this transformation reflects China’s ‘ecological shift’ in its overall approach to pangolins. Although the use of pangolins in medicine is still permitted, judiciary demonstrably adopted an extremely stringent stance. Under these circumstances, even the illegal hunting of a single pangolin will incur full legal liability. In sum, within China’s legal system, pangolins unequivocally transitioned from being mere resources to being recognized as ‘important ecological populations and individuals.’ Despite challenges such as difficulties in artificial breeding and the impact of further urbanization on wild populations, the law indeed created a place for pangolins. Nevertheless, current judicial protection for pangolins in China still faces issues such as inconsistencies in the application of compensation types in rulings and the weak participation of public interest organizations. From a long-term perspective, continuously advancing the improvement of legal systems and the innovation of judicial practices will provide robust support for pangolin conservation, fostering their population recovery and sustainable development within natural habitats, and further promoting the harmonious coexistence between humans and wild animals.

## Figures and Tables

**Table 1:** Laws and regulations related to pangolins in China.

Legal/Regulatory Document Title	Level of Legal Force	Effective Date	Key Points
Instruction of the State Council on Actively Protecting and Rationally Utilizing Wildlife Resources	Normative document of the State Council	14 Sept. 1962	Advocated a combined approach of “protection, breeding, and hunting”; hunting quotas required provincial approval.
Notice on Traditional Chinese Medicine Work Issues	Normative document of the State Council	13 Oct. 1983	Noted the high demand for pangolins in traditional Chinese medicine and the considerable challenges associated with captive breeding.
Administrative Measures for Forest and Wildlife Nature Reserves	Administrative regulation	1 June 1985	Regulated the management of forest and wildlife reserves.
National Key Protected Wild Medicinal Species List	Departmental normative document	30 Oct. 1987	Included pangolins as protected wild medicinal species.
List of Protected Wild Animals	Departmental regulation	14 Jan. 1989	Listed pangolins as second-class protected animals.
Wild Animal Conservation Law of the People’s Republic of China (amended five times in 2004, 2009, 2016, 2018, 2022)	Law	1 Mar. 1989	Specifies legislative objectives, departmental responsibilities, protection of wild animals and their habitats, wild animal management, legal liabilities, and other important aspects. The 2022 amendment strengthened habitat and ecosystem protection.
Circular on Illegal Hunting and Trade of Rare Wild Animals	Normative document of the State Council	12 May 1990	Reported a case involving illegal pangolin trade.
Letter from the Ministry of Forestry Requesting Assistance in Managing the Export of Proprietary Chinese Medicines Containing Wild Animal Medicinal Ingredients	Departmental normative document	15 June 1990	Stipulated that pangolins and other wild animals, if exported as traditional Chinese medicine, must undergo approval and consent before export certificates are issued.
Regulation of the People’s Republic of China on the Protection of Terrestrial Wild Animals (revised in 2011, 2016)	Administrative regulation	1 Mar. 1992	Provides specific regulations for the protection, hunting, domestication, breeding, and commercial utilization of wild animals.
Circular of the General Office of the State Council on Transmitting the Ministry of Forestry’s Report on Strengthening Wild Animal Protection and Management	Normative document of the State Council	23 Oct. 1991	Highlighted the severe situation of illegal hunting and trade of wild animals, urged stronger control over legal hunting, and suggested that provinces, autonomous regions, and municipalities conduct a national key protected wild animal resource survey every three to five years.
List of Animals, Animal Products, and Other Quarantine Objects Prohibited from Being Carried or Mailed into the People’s Republic of China	Departmental normative document	8 June 1992	Listed pangolins as prohibited for import/mail.
Measures for Charging Management Fees for Terrestrial Wild Animal Resource Protection	Departmental regulation	1 Jan. 1993	Established wild animal resource protection and management fees, approval procedures, and special hunting permits. In certain cases of legal pangolin hunting, the management fee was RMB 100 per animal.
Standards for Filing Cases of Illegal Poaching of National Key Protected Precious and Endangered Terrestrial Wild Animals	Departmental normative document	9 May 2001	Set the case filing standard for illegal pangolin poaching at 4 animals, with 8 animals considered a major case and 16 animals a particularly major case.
Notice of the General Office of the State Council on Strengthening the Protection and Management of Biological Species Resources	Normative document of the State Council	1 Mar. 2004	Aimed to strengthen the protection of biological species resources.
Guiding Opinions of the State Forestry Administration on Promoting Sustainable Development of Wild Animals and Plants	Departmental normative document	1 Sept. 2004	While strictly controlling the total consumption of resources, it emphasized the orderly circulation of resources to prioritize the demand for resources in key areas and industries such as traditional Chinese medicine and culture.
National Plan for Biological Species Resource Protection and Utilization	Departmental Work Document	24 Oct. 2007	Proposed establishing pangolin breeding bases to address technical challenges in pangolin reproduction.
Notice on Strengthening the Protection of Saiga Antelope, Pangolins, Rare Snakes, and Standardizing the Management of Their Products for Medicinal Use	Departmental normative document	12 Nov. 2007	Stopped issuing pangolin hunting permits; encouraged commercial-scale breeding of pangolins; controlled the total quantity of pangolin use in designated hospitals and pharmaceutical production; and implemented a special labeling system.
Announcement No. 8 of 2015 by the State Forestry Administration—Announcement on Matters Concerning Special Identification for the Management of Wild Animal Business and Utilization in China	Departmental normative document	28 Apr. 2015	Established special identification for wild animal business and utilization, mandating special labeling for pangolins used in prescriptions and designating specific hospitals.
Pharmacopoeia of the People’s Republic of China	Departmental Work Document	5 June 2015	Included pangolins in the Pharmacopoeia catalog.
Notice of the State Administration for Market Regulation and the National Forestry and Grassland Administration on Jointly Carrying Out Special Rectification Actions for Wild Animal Protection	Departmental working document	24 May 2019	Strengthened coordinated supervision, joint inspection, joint law enforcement, and joint punishment against illegal activities involving ivory, rhino horn, tiger, pangolin, and their products.
Decision of the Standing Committee of the National People’s Congress to Comprehensively Prohibit the Illegal Trade of Wild Animals, Break the Bad Habit of Excessive Consumption of Wild Animals, and Effectively Secure the Life and Health of the People	Legal decision	4 Feb. 2020	Comprehensively prohibited the consumption of “terrestrial wild animals with important ecological, scientific, or social value” and other terrestrial wild animals protected by the state, including those artificially bred or raised. Completely prohibited hunting, trading, and transporting terrestrial wild animals that naturally grow and reproduce in the wild for consumption purposes.
Chinese Pharmacopoeia	China’s National Statutory Drug Technical Standard	May 2020	Removed pangolins from the pharmacopoeia.
Announcement on Adjusting the Protection Level of Pangolins	Departmental work document	3 June 2020	Upgraded pangolins to Class I National Protected Animals.
Notice of the National Forestry and Grassland Administration, the National Administration of Traditional Chinese Medicine, and the National Medical Products Administration on Effectively Strengthening Pangolin Protection and Management	Departmental work document	13 Nov. 2024	The notice continued to define pangolin scales as a clinical resource, while also emphasizing the strengthening of artificial breeding, the development of resource banks, the encouragement of substitute research, and the strict control of their medicinal use based on resource conservation.

These data were derived from the following resources available in the public domain: PKULAW (Peking University Law Database) at http://pkulaw.com (accessed on 16 August 2025).

## Data Availability

All data created or analyzed in this study are available as Supplementary Material.

## Supplementary Materials

The following supporting information can be downloaded at: https://www.mdpi.com/article/10.3390/ani15162422/s1, Supplementary Material S1: Full Text Links to Laws and Regulations Related to Pangolins in China; Supplementary Material S2: Full Text of Laws and Regulations Related to Pangolins in China; Supplementary Material S3: Full Text Links to Judgments in Pangolin-Related Public Interest Litigation Cases in China; Supplementary Material S4: Full Text of Judgments in Pangolin-Related Public Interest Litigation Cases in China.
