# Supplementary material for: China’s Legal Protection System for Pangolins: Past, Present, and Future
Source: Animals (Basel). 2025 Aug 18;15(16):2422. doi: 10.3390/ani15162422 (PMC12383201; doi:10.3390/ani15162422)
Supplement: Supplementary file 1 [file animals-15-02422-s001.zip › Supplementary Material S4-Full Text of Judgments in Pangolin-Related Public Interest Litigation Cases in China/【10】刘厚够侵权责任纠纷民事一审民事判决书.pdf]

刘厚够侵权责任纠纷民事一审民事判决书

广东省广州市中级人民法院

民 事 判 决 书

（2020）粤 01 民初 1868 号

公益诉讼起诉人：广东省佛山市人民检察院，住所地广东省佛山市禅城区湖景路 28 号。

法定代表人：黄黎明，该院检察长。

出庭人员：杨楚君，该院检察官。

出庭人员：钟媛，该院检察官助理。

被告：刘厚够，男，1965 年 9 月 1 日出生，汉族，户籍所在地广东省英德市，现被羁押在广东省江门监狱。

委托诉讼代理人：刘嘉成，是刘厚够的儿子。

公益诉讼起诉人广东省佛山市人民检察院认为被告刘厚够破坏生态环境，损害社会公共利益，在履行诉前公告程序，公告期满，法律规定的机关和有关组织不提起诉讼后，依法向本院提起环境保护民事公益诉讼。本院于 2020 年 10 月 29 日立案后，依法适用普通程序，于 2021 年 9 月 17 日公开开庭进行了审理，公益诉讼起诉人广东省佛山市人民检察院指派杨楚君检察官、钟媛检察官助理出庭参加诉讼，被告刘厚够委托诉讼代理人刘嘉成到庭参加诉讼。本案现已审理终结。

广东省佛山市人民检察院向本院提出诉讼请求：1.判令刘厚够赔偿生态环境损失 3823500 元；2.判令刘厚够在国家级媒体上

就非法收购、出售国家保护野生动物损害社会公共利益的行为赔礼道歉。事实和理由：刘厚够从事穿山甲、大壁虎等野生动物买卖活动。于 2018 年 11 月 2 日，刘厚够在佛山市南海区大沥高速路口接货，购买得 6 只穿山甲活体。于 2019 年 2 月 18 日被告刘厚够通过微信与使用微信名“杰”的人联系后以 920 元每斤的价格向“杰”出售两只只穿山甲。上述共 8 只穿山甲，根据《国家重点保护野生动物名录》、《野生动物及其制品价值评估方法》及附件《陆生野生动物基准价值标准目录》等的规定，参考案涉动物价值鉴定意见书，可以认定该 8 只穿山甲的整体价值为人民币 32 万元。于 2019 年 3 月 24 日，民警在佛山市南海区桂城街道石啃村委会三村大桥坊一巷 16 号整栋 401 房抓获刘厚够，对其住处搜查时，民警搜出并扣押刘厚够待出售疑似穿山甲冰冻死体、疑似蜥蜴类动物冰冻死体一批，分别为马来穿山甲死体 3 只，属于《濒危野生动植物种国际贸易公约》CITES 附录 I 保护动物；孟加拉巨蜥死体 2 只，属于《濒危野生动植物种国际贸易公约》CITES 附录 I、国“三有”保护动物；圆鼻巨蜥死体 1 只，属于《濒危野生动植物种国际贸易公约》CITES 附录 II、国家一级保护动物；平胸龟死体 5 只，属于《濒危野生动植物种国际贸易公约》CITES 附录 I、国家“三有”保护动物；大壁虎死体 674 条，属于国家二级保护动物。上述动物的整体价值为人民币 3503500 元。于 2020 年 7 月 14 日，佛山市南海区人民法院作出(2019)粤 0605 刑初 2963 号刑事判决书，该判决书认为：刘厚够非法收

购、出售国家重点保护的珍贵、濒危野生动物，情节特别严重，已构成非法收购、出售珍贵、濒危野生动物罪，判处刘厚够有期徒刑 11 年，并处罚金 1 万元。刘厚够不服一审判决，提出上诉。佛山市中级人民法院于 2020 年 9 月 2 日作出(2020)粤 06 刑终 828 号刑事裁定书，维持原判。广东省佛山市人民检察院发现刘厚够非法收购、出售穿山甲、大壁虎等野生动物的行为后，于 2020 年 4 月 8 日在正义网上发布本案诉前公告，截至本案起诉前，未有法律规定的机关和有关组织向本院反馈拟就本案起诉，社会公共利益仍处于受侵害状态。刘厚够的行为违反了《中华人民共和国野生动物保护法》第二十七条第一款、第三十条第二款，《中华人民共和国环境保护法》第六条、第三十条的规定。根据《中华人民共和国环境保护法》第六十四条、《中华人民共和国侵权责任法》第十五条、《最高人民法院关于审理环境民事公益诉讼案件适用法律若干问题的解释》第十八条的规定，应承担生态环境损害赔偿责任及在国家级媒体上赔礼道歉的责任。根据《中华人民共和国民事诉讼法》第五十五条第二款、《最高人民法院、最高人民检察院关于检察公益诉讼案件适用法律若干问题的解释》第十三条第二款的规定，提起诉讼。

刘厚够辩称，刘厚够对广东省佛山市人民检察院提出的民事公益诉讼请求和事实没有异议，已经深刻认识到自己的行为对生态环境造成了损害，愿意根据法律、法规的规定并结合自己的经

济能力，承担民事赔偿责任，但希望能给予宽限期准备资金赔偿损失。

当事人围绕诉讼请求依法提交了证据，本院组织当事人进行了质证。刘厚够对于广东省佛山市人民检察院提交的证据均无异议并予以确认。对当事人无异议的证据，本院予以确认并在卷佐证。

根据当事人陈述和经审查确认的证据，本院认定事实如下：

2018年11月2日，刘厚够在佛山市南海区大沥高速路口接货，购买得6只穿山甲活体。

2019年2月18日被告刘厚够通过微信与使用微信名“杰”的人联系后以920元每厅的价格向“杰”出售两只只穿山甲。

2019年3月24日，民警在佛山市南海区桂城街道石啃村委会三村大桥坊一巷16号整栋401房抓获刘厚够，对其住处搜查时，民警搜出并扣押刘厚够待出售疑似穿山甲冰冻死体、疑似蜥蜴类动物冰冻死体一批，分别为马来穿山甲死体3只，属于《濒危野生动植物种国际贸易公约》CITES附录I保护动物；孟加拉巨蜥死体2只，属于《濒危野生动植物种国际贸易公约》CITES附录I、国“三有”保护动物；圆鼻巨蜥死体1只，属于《濒危野生动植物种国际贸易公约》CITES附录II、国家一级保护动物；平胸龟死体5只，属于《濒危野生动植物种国际贸易公约》CITES附录I、国家“三有”保护动物；大壁虎死体674条，属于国家二级保护动物。华南动物物种环境损害司法鉴定中心作出的华动

司鉴字[2019]第75号《司法鉴定意见书》载明，经鉴定上述动物的整体价值为人民币3503500元。以上事实均由已经生效的佛山市南海区人民法院作出(2019)粤0605刑初2963号刑事判决书和佛山市中级人民法院于2020年9月2日作出(2020)粤06刑终828号刑事裁定书予以确认。

诉讼中，刘厚够答辩称其承认其损害环境的事实，对广东省佛山市人民检察院的提起本案的全部诉讼请求和事实没有异议，且其同意履行全部诉讼请求。

另查明，《国家重点保护野生动物名录》中载明穿山甲为国家二级保护动物。《野生动物及其制品价值评估方法》第四条载明国家二级保护野生动物，按照所列野生动物基准价值的五倍核算。《陆生野生动物基准价值标准目录》载明穿山甲所有种的基准价值为8000元。

本院认为，广东省佛山市人民检察院提起本案生态破坏民事公益诉讼，符合《中华人民共和国民事诉讼法》第五十五条第二款、《最高人民法院、最高人民检察院关于检察公益诉讼案件适用法律若干问题的解释》第十三条规定的提起公益诉讼的条件和程序，诉讼主体适格，本院予以支持。

经审查，刘厚够非法收购、出售国家保护的野生动物，破坏了生态环境的事实清楚，刘厚够也不持异议，本院予以认定。《中华人民共和国民法典》第一千二百三十五条规定：“违反国家规定造成生态环境损害的，国家规定的机关或者法律规定的组织有

权请求侵权人赔偿下列损失和费用：……（四）清除污染、修复生态环境费用；……”《最高人民法院关于审理环境民事公益诉讼案件适用法律若干问题的解释》第十八条规定：“对污染环境、破坏生态，已经损害社会公共利益或者具有损害社会公共利益重大风险的行为，原告可以请求被告承担停止侵害、排除妨碍、消除危险、修复生态环境、赔偿损失、赔礼道歉等民事责任。”第二十条第二款规定：“人民法院可以在判决被告修复生态环境的同时，确定被告不履行修复义务时应承担的生态环境修复费用；也可以直接判决被告承担生态环境修复费用。”刘厚够非法收购、出售国家保护的野生动物，破坏了生物多样性及生态平衡，已经损害社会公共利益。经审查，刘厚够 2018 年 11 月 2 日和 2019 年 2 月 18 日非法收购、出售 8 只穿山甲活体，且 2019 年 3 月 24 日在刘厚够住处搜出的马来穿山甲死体 3 只、孟加拉巨蜥死体 2 只、平胸龟死体 5 只、大壁虎死体 674 条。对生态环境的损害已无法修复，故刘厚够依法应当承担赔偿修复生态环境费用损失的民事责任。

《最高人民法院关于审理环境侵权责任纠纷案件适用法律若干问题的解释》第八条规定：“对查明环境污染、生态破坏案件事实的专门性问题，可以委托具备相关资格的司法鉴定机构出具鉴定意见或者由负有环境资源保护监督管理职责的部门推荐的机构出具检验报告、检测报告、评估报告或者监测数据。”第十七条规定：“本解释适用于审理因污染环境、破坏生态造成损

害的民事案件，但法律和司法解释对环境民事公益诉讼案件另有规定的除外。”

广东省佛山市人民检察院主张依据《国家重点保护野生动物名录》、《野生动物及其制品价值评估方法》即其附件《陆生野生动物基准价值标准目录》等规定对刘厚够于2018年11月2日和2019年2月18日非法收购、出售8只穿山甲活体的行为，要求刘厚够赔偿国家野生动物资源损失即修复生态环境费用损失320000元有事实和法律依据，本院予以支持。

同时，对于刘厚够于2019年3月24日破坏生态环境事件发生后，广州海关佛山缉私分局就涉案生态环境损害事实的专门性问题，委托具备资格的华南动物物种环境损害司法鉴定中心进行鉴定评估，符合法律规定。华南动物物种环境损害司法鉴定中心受托出具的华动司鉴字[2019]第75号《鉴定意见书》，程序合法，依据充分，应予采纳。上述鉴定意见反映的马来穿山甲死体3只、孟加拉巨蜥死体2只、平胸龟死体5只、大壁虎死体674条在生态环境中的价值为3503500元，故可参照该价值确定上述事实的修复生态环境费用的数额。

综上，广东省佛山市人民检察院请求刘厚够赔偿野生动物资源损失即修复生态环境费用损失3823500元（即320000元＋350350元）及在国家级以上媒体就其非法收购、出售国家野生动物损害社会公共利益的行为进行赔礼道歉的诉讼请求，事实及法律依据充分，本院予以支持。《最高人民法院关于审理环境民

事公益诉讼案件适用法律若干问题的解释》第二十四条第一款规定：“人民法院判决被告承担的生态环境修复费用、生态环境受到损害至修复完成期间服务功能丧失导致的损失、生态环境功能永久性损害造成的损失等款项，应当用于修复被损害的生态环境。”据此，刘厚够承担的生态环境修复费用应当上缴国库用于修复被损害的生态环境。

综上所述，广东省佛山市人民检察院的诉讼请求成立，应予以支持。依照《中华人民共和国民法典》第一千二百三十五条第四项，《最高人民法院关于审理环境民事公益诉讼案件适用法律若干问题的解释》第十八条、第二十条第二款、第二十四条第一款规定，判决如下：

一、被告刘厚够自本判决发生法律效力之日起十日内赔偿生态环境修复费用 3823500 元（该费用上缴国库用于修复被损害的生态环境）；

二、被告刘厚够自本判决发生法律效力之日起十日内在国家级以上媒体就其非法收购、出售国家野生动物损害社会公共利益的行为进行赔礼道歉。

如果未按本判决指定的期间履行给付金钱义务，应当依照《中华人民共和国民事诉讼法》第二百五十三条规定，加倍支付迟延履行期间的债务利息。

本案受理费 37388 元，由被告刘厚够负担。

如不服本判决，可以在判决书送达之日起十五日内，向本院递交上诉状，并按对方当事人的人数提出副本，上诉于广东省高级人民法院。

审　判　长　　李　琦
审　判　员　　庞智雄
审　判　员　　刘　欢
人民陪审员　　严伟琦
人民陪审员　　刘权伟
人民陪审员　　谢兰英
人民陪审员　　严锡开
二〇二一年九月十八日
法官　助理　　蔡秀玲
书　记　员　　吴嘉茵
