# Supplementary material for: China’s Legal Protection System for Pangolins: Past, Present, and Future
Source: Animals (Basel). 2025 Aug 18;15(16):2422. doi: 10.3390/ani15162422 (PMC12383201; doi:10.3390/ani15162422)
Supplement: Supplementary file 1 [file animals-15-02422-s001.zip › Supplementary Material S4-Full Text of Judgments in Pangolin-Related Public Interest Litigation Cases in China/【11】中国生物多样性保护与绿色发展基金会;肖习文生态破坏民事公益诉讼民事一审民事判决书.pdf]

中国生物多样性保护与绿色发展基金会;肖习文生态破坏民事公益诉讼民事一审民事判决书

广东省广州市中级人民法院

民 事 判 决 书

（2021）粤 01 民初 576 号

原告：中国生物多样性保护与绿色发展基金会，住所地北京市海淀区板井路 69 号世纪金源国际公寓 1 单元 7B。

法定代表人：谢伯阳，该基金会理事长。

委托诉讼代理人：傅前明，北京市方正律师事务所律师。

被告：肖习文，男，汉族，1970 年 2 月 24 日出生，户籍所在地安徽省南陵县，原住广东省广州市白云区，现于湖南省邵阳监狱服刑。

原告中国生物多样性保护与绿色发展基金会与被告肖习文生态破坏民事公益诉讼一案，本院曾于 2020 年 6 月 11 日立案，于 2020 年 6 月 22 日作出（2020）粤 01 民初 763 号民事裁定，裁定本案移送北京市第四中级人民法院处理。后广东省高级人民法院于 2021 年 2 月 24 日作出（2021）粤民辖监 3 号民事裁定，裁定本案由本院审理。本院于 2021 年 3 月 16 日立案后，依法适用普通程序，于 2021 年 8 月 24 日公开开庭进行了审理，原告中国生物多样性保护与绿色发展基金会的委托诉讼代理人傅前明律师到庭参加诉讼，被告肖习文经传票传唤无正当理由拒不到庭参加诉讼。本案现已审理终结。

中国生物多样性保护与绿色发展基金会向本院提出诉讼请求：1、肖习文赔偿因生态环境侵权行为导致穿山甲灭失的生态服务功能损失（具体金额参照《野生动物及其制品价值评估方法》予以确定，每只8万元）；2、肖习文在国家级媒体上向社会公众公开赔礼道歉；3、肖习文承担中国生物多样性保护与绿色发展基金会的律师代理费（按法院判决肖习文承担损失金额的10%计算，最高不超过15万元）、差旅费3000元等为诉讼支出的费用；4、肖习文承担本案的全部诉讼费用，包括案件受理费、鉴定费等。事实和理由：中国生物多样性保护与绿色发展基金会是国务院批准设立、民政部登记注册、中国科学技术协会主管的非营利性社会组织，主要职责之一就是推动绿色发展事业，维护环境公共利益，在全国范围提起了多起环境公益诉讼，符合法律规定的环境民事公益诉讼原告资格。肖习文自2016年10月至2018年1月期间，在广东省广州市白云区租用房屋非法收购、出售国家二级保护野生动物穿山甲活体14只、穿山甲冻体27只。此外在2018年1月20日案发后，被从其租房内发现27只穿山甲冻体。上述事实，业经湖南省新邵县人民法院（2019）湘0522刑初39号刑事判决书予以认定。穿山甲属于《中华人民共和国野生动物保护法》规定的国家重点保护的珍贵、濒危野生动物，同时也被列入《濒危野生动植物种国际贸易公约》附录Ⅱ，被认为是全球受到非法贸易威胁最严重的哺乳类动物。穿山甲作为重要的生物资源，其既有保护森林、堤坝、维护生态平衡以及促进科

学研究等方面的巨大作用，又具有显著的生物多样性国际保护价值，联合国《生物多样性公约》第十五次缔约方大会今年将由我国承办。此外，全国人大常委会2020年2月24日通过《关于全面禁止非法野生动物交易、革除滥食野生动物陋习、切实保障人民群众生命健康安全的决定》，就全面禁止包括穿山甲在内的非法野生动物交易等问题作出了具体规定。肖习文组织、参与穿山甲非法贸易链条的行为，直接导致68只野生穿山甲的灭失，严重侵害了生态环境公共利益，应当承担相应的法律责任。为彰显生态文明理念、落实生物多样性保护、保护环境公共利益，恳请支持中国生物多样性保护与绿色发展基金会的全部诉讼请求。

被告肖习文未作答辩。

中国生物多样性保护与绿色发展基金会围绕诉讼请求依法提交了以下证据：1、中国生物多样性保护与绿色发展基金会的社会组织登记证书、法定代表人身份证明书、法定代表人身份证、章程、年度工作报告、五年内无违法记录证明，拟证明中国生物多样性保护与绿色发展基金会是合法的环境民事公益诉讼主体；2、湖南省新邵县人民法院（2019）湘0522刑初39号刑事判决书，拟证明肖习文导致68只野生穿山甲（活体与冻体）全部灭失；3、国家林业和草原局[2020]12号公告，拟证明从2020年6月3日起将穿山甲由国家二级保护野生动物调整为国家一级保护野生动物。肖习文对上述证据未提出质证意见。经审查，本院对上述证据1、2的真实性、合法性、关联性予以确认，对上述

证据 3 的真实性、合法性予以确认，但上述证据 3 系在中国生物多样性保护与绿色发展基金会所主张的生态破坏事实发生之后公布，与本案诉争事实无关，故对上述证据 3 的关联性不予确认。

根据当事人陈述和经审查确认的证据，本院认定事实如下：

中国生物多样性保护与绿色发展基金会系在中华人民共和国民政部登记的基金会法人，专门从事环境保护公益活动连续五年以上且无违法记录，具备提起生态破坏民事公益诉讼的主体资格。

2017 年 6 月至 12 月期间，肖习文分多次向案外人王某增收购国家二级保护野生动物穿山甲 11 只，向案外人全某收购穿山甲 2 只，向案外人尹某收购穿山甲 1 只，然后将上述 14 只穿山甲分别出售给案外人徐卫东、慈卫华、范某春、陈某炳、阮某洲、戴某、李某文、倪某、王某等人，其中 8 只穿山甲已被食用，6 只穿山甲被转卖后无法确定死活。

2017 年 12 月 9 日至 2018 年 1 月 1 日期间，肖习文向案外人徐卫东出售 4 只穿山甲冻体。2018 年 1 月期间，肖习文向案外人戴某甲出售 1 只穿山甲冻体。2018 年 1 月初，肖习文向案外人李某文出售 1 只穿山甲冻体。2017 年 12 月期间，肖习文向案外人刘某平出售 2 只穿山甲冻体。2017 年 12 月初，肖习文向案外人黄某江出售 2 只穿山甲冻体。2017 年 9 月至 10 月期间，肖习文向案外人周某林出售 2 只穿山甲冻体。2017 年 10 月至 11 月期间，肖习文向案外人王某出售 2 只穿山甲冻体。2017 年 8 月

至 9 月期间,肖习文向案外人陈某出售 10 只穿山甲冻体。以上肖习文共向案外人出售 24 只穿山甲冻体。

肖习文于 2018 年 1 月 20 日被抓获归案，公安民警从其租房内搜缴 27 只国家二级重点保护野生动物马来穿山甲冻体。

2019 年 9 月 9 日，湖南省新邵县人民法院对肖习文等被告人犯非法收购、出售珍贵、濒危野生动物、珍贵、濒危野生动物制品罪一案作出（2019）湘 0522 刑初 39 号刑事判决，认定了上述肖习文非法收购、出售穿山甲及穿山甲冻体的事实，判决肖习文犯非法收购、出售珍贵、濒危野生动物、珍贵、濒危野生动物制品罪，以及由侦查机关对扣押肖习文的上述 27 只穿山甲冻体予以没收，上缴国库。

上述肖习文非法收购、出售穿山甲整体共 65 只，其中确定已死亡（被食用及冻体）59 只，无法确定死活 6 只。穿山甲属所有种在 2020 年 6 月 3 日前为国家二级保护野生动物，自 2020 年 6 月 3 日国家林业和草原局公布 2020 年第 12 号公告之日起，调整为国家一级保护野生动物。根据国家林业局制定的《野生动物及其制品价值评估方法》的附件《陆生野生动物基准价值标准目录》，穿山甲科所有种的基准价值为 8000 元。根据上述《野生动物及其制品价值评估方法》第四条规定，野生动物整体的价值按照上述《陆生野生动物基准价值标准目录》所列该种野生动物的基准价值乘以相应的倍数核算，其中国家一级保护野生动物按照十倍核算，国家二级保护野生动物按照五倍核算。据此，上

述已死亡的59只穿山甲整体，按照国家二级保护野生动物核算其价值共236万元（8000元/只×5倍×59只）。

2020年4月20日，中国生物多样性保护与绿色发展基金会与北京市方正律师事务所就本案诉讼代理事宜签订《委托代理协议》，约定一审律师费按照判决书确定的环境损害赔偿金、环境修复（或者替代性修复）资金、环境整治资金或者被告因此而设立的环境保护类基金（或专项资金或环境慈善信托资金）总数额的10%计收。

本院认为，对肖习文破坏生态环境，损害社会公共利益的行为，中国生物多样性保护与绿色发展基金会依法可以提起生态破坏民事公益诉讼。

肖习文非法收购、出售国家保护的野生动物，破坏了生态环境的事实，已为人民法院发生法律效力的刑事判决所确认，肖习文对此也未提出异议，故本院予以认定。中国生物多样性保护与绿色发展基金会主张肖习文的行为导致68只穿山甲灭失的事实，与上述刑事判决认定肖习文非法收购、出售穿山甲整体共65只，其中确定已死亡59只，无法确定死活6只的事实不符，本院予以纠正。《中华人民共和国环境保护法》第六十四条规定：“因污染环境和破坏生态造成损害的，应当依照《中华人民共和国侵权责任法》的有关规定承担侵权责任。”《最高人民法院关于审理环境民事公益诉讼案件适用法律若干问题的解释》第十八条规定：“对污染环境、破坏生态，已经损害社会公共利益或者

具有损害社会公共利益重大风险的行为，原告可以请求被告承担停止侵害、排除妨碍、消除危险、修复生态环境、赔偿损失、赔礼道歉等民事责任。”第二十条第二款规定：“人民法院可以在判决被告修复生态环境的同时，确定被告不履行修复义务时应承担的生态环境修复费用；也可以直接判决被告承担生态环境修复费用。”肖习文非法收购、出售国家保护的野生动物，破坏了生物多样性及生态平衡，已经损害社会公共利益，且肖习文非法收购、出售的涉案穿山甲的部分系死体，对生态环境的损害已无法修复，故肖习文依法应当承担赔偿修复生态环境费用损失的民事责任。《野生动物及其制品价值评估方法》规定的野生动物整体的价值，属于野生动物在生态环境中的价值，故中国生物多样性保护与绿色发展基金会主张参照该价值确定本案修复生态环境费用的数额，并无不当，本院予以支持。但因本案破坏生态环境的行为发生在穿山甲属所有种由国家二级保护野生动物调整为国家一级保护野生动物之前，且肖习文非法收购、出售涉案穿山甲中有 6 只无法确定死活，不足以证明肖习文该部分行为破坏了生态环境，故本院按照已确定的涉案穿山甲死体 59 只为国家二级保护野生动物核算其价值，认定肖习文赔偿修复生态环境费用的数额为 236 万元。因此，中国生物多样性保护与绿色发展基金会要求肖习文赔偿穿山甲灭失的生态服务功能损失即修复生态环境费用损失，其中 236 万元部分的诉讼请求，事实及法律依据充分，本院予以支持；超过 236 万元部分的诉讼请求，依据不足，

本院不予支持，予以驳回。《最高人民法院关于审理环境民事公益诉讼案件适用法律若干问题的解释》第二十四条第一款规定："人民法院判决被告承担的生态环境修复费用、生态环境受到损害至修复完成期间服务功能丧失导致的损失、生态环境功能永久性损害造成的损失等款项，应当用于修复被损害的生态环境。"据此，肖习文承担的生态环境修复费用应当上缴国库用于修复被损害的生态环境。

《最高人民法院关于审理环境民事公益诉讼案件适用法律若干问题的解释》第二十二条规定："原告请求被告承担检验、鉴定费用、合理的律师费以及为诉讼支出的其他合理费用的，人民法院可以依法予以支持。"中国生物多样性保护与绿色发展基金会委托律师参加本案诉讼所支出的律师费及为本案诉讼而往来北京与广州之间所支出的差旅费，均属于中国生物多样性保护与绿色发展基金会为诉讼支出的合理费用，中国生物多样性保护与绿色发展基金会请求肖习文予以承担，符合上述司法解释规定，本院予以支持。但中国生物多样性保护与绿色发展基金会诉请的律师费数额不明确，诉请的差旅费数额没有举证证明，故本院综合考虑本案诉讼的公益性、本案诉讼的取证难度较低、律师相应的工作量、按照日常生活经验判断往来北京与广州之间的交通及住宿费用，以及律师同时代理本案和本院受理的中国生物多样性保护与绿色发展基金会提起的另一生态破坏民事公益诉讼案等情形及因素，酌情确定肖习文承担中国生物多样性保护与绿

色发展基金会为本案诉讼支出的律师费为1万元、差旅费为1500元。中国生物多样性保护与绿色发展基金会的该项诉讼请求超过前述金额部分，本院不予支持，予以驳回。

如上所述，肖习文破坏生态环境的行为已经损害了社会公共利益，故依照上述《最高人民法院关于审理环境民事公益诉讼案件适用法律若干问题的解释》第十八条规定，肖习文还应当通过公开赔礼道歉以示真诚悔过。因此，中国生物多样性保护与绿色发展基金会提出肖习文在国家级媒体上向社会公众公开赔礼道歉的诉讼请求，有事实及法律依据，本院予以支持。

综上所述，中国生物多样性保护与绿色发展基金会的诉讼请求部分成立，应予支持。肖习文经传票传唤无正当理由拒不到庭，本院依法缺席判决。依照《中华人民共和国环境保护法》第六十四条，《最高人民法院关于审理环境民事公益诉讼案件适用法律若干问题的解释》第十八条、第二十条第二款、第二十二条、第二十四条第一款，《中华人民共和国民事诉讼法》第一百四十四条规定，判决如下：

一、被告肖习文自本判决发生法律效力之日起十日内赔偿生态环境修复费用236万元（该费用上缴国库用于修复被损害的生态环境）；

二、被告肖习文自本判决发生法律效力之日起十日内向原告中国生物多样性保护与绿色发展基金会赔偿律师费1万元及差旅费1500元；

三、被告肖习文自本判决发生法律效力之日起十日内在国家级媒体上发表经本院认可的赔礼道歉声明。

如果未按本判决指定的期间履行给付金钱义务，应当依照《中华人民共和国民事诉讼法》第二百五十三条规定，加倍支付迟延履行期间的债务利息。

本案受理费25772元，由被告肖习文负担。

如不服本判决，可以在判决书送达之日起十五日内，向本院递交上诉状，并按对方当事人的人数提出副本，上诉于广东省高级人民法院。

审　判　长　　庞智雄

审　判　员　　李　琦

审　判　员　　林旭群

人民陪审员　　王肇霞

人民陪审员　　温志伟

人民陪审员　　李清萍

人民陪审员　　严伟琦

二〇二一年九月十三日

法官　助理　　江亭利

书　记　员　　林美欣
