# Supplementary material for: China’s Legal Protection System for Pangolins: Past, Present, and Future
Source: Animals (Basel). 2025 Aug 18;15(16):2422. doi: 10.3390/ani15162422 (PMC12383201; doi:10.3390/ani15162422)
Supplement: Supplementary file 1 [file animals-15-02422-s001.zip › Supplementary Material S4-Full Text of Judgments in Pangolin-Related Public Interest Litigation Cases in China/【13】佃锡煌刑事一审刑事判决书.pdf]

佃锡煌刑事一审刑事判决书

广东省梅州市梅县区人民法院

刑 事 附 带 民 事 判 决 书

（2021）粤 1403 刑初 165 号

公诉机关暨附带民事公益诉讼起诉人梅州市梅县区人民检察院。

被告人佃锡煌，男，1959 年 6 月 20 日出生，初中文化，户籍所在地潮州市潮安县，住原籍。因涉嫌犯走私珍贵动物罪于 2019 年 3 月 24 日被梅州海关缉私分局刑事拘留，同年 3 月 25 日被监视居住；因涉嫌犯危害珍贵、濒危野生动物罪于 2020 年 3 月 26 日被梅州市公安局梅县区分局取保候审。2021 年 8 月 31 日经本院决定被逮捕。现羁押于梅州市梅县区看守所。

辩护人黄思玲，广东义致律师事务所律师，由梅州市梅县区法律援助处指派。

梅州市梅县区人民检察院以梅县区检刑诉〔2021〕131 号起诉书指控被告人佃锡煌涉嫌犯危害珍贵、濒危野生动物罪，于 2021 年 7 月 8 日向本院提起公诉。同日，梅州市梅县区人民检察院以梅县区检刑附民公诉〔2021〕1 号刑事附带民事公益诉讼起诉书向本院提起附带民事公益诉讼。本院受理后，依法组成合议庭，公开开庭进行了合并审理。梅州市梅县区人民检察院指派检察员李培军、李冰出庭支持公诉，指派检察员李培军、巫翠玲

出庭参加附带民事公益诉讼，被告人佃锡煌及辩护人黄思玲到庭参加诉讼。现已审理终结。

梅州市梅县区人民检察院指控，2017 年，被告人佃锡煌为治疗自身疾病，未经野生动物保护部门批准，擅自在梅州市梅江区以每斤 200 元左右的价格向摆地摊的中老年男子收购穿山甲鳞片共计 3270 克，然后放置于梅州市梅县区其居住的地方以备食用。2019 年 3 月 14 日，梅州海关缉私分局依法在佃锡煌居住的梅县区住家搜查出一批疑似穿山甲鳞片、收据、两部手机，并予以扣押。

2019 年 4 月 8 日，经华南动物物种环境损害司法鉴定中心鉴定，从被告人佃锡煌家中扣押的疑似穿山甲鳞片分别系哺乳纲鳞甲目穿山甲科穿山甲属马来穿山甲的鳞片和树穿山甲的鳞片，该两类穿山甲均属于国家二级重点保护珍贵、濒危动物，本案涉案穿山甲鳞片的涉案价值为 104640 元。

2020 年 3 月 25 日，梅州市公安局梅县区分局电话传唤被告人佃锡煌，佃锡煌接电话后自行如期到案接受讯问，并如实供述其犯罪事实。

对指控的上述犯罪事实，公诉机关当庭宣读和出示了被告人供述、证人证言、辨认笔录、现场勘查笔录、现场图、现场照片、鉴定文书、归案经过、人口信息表等证据材料，据此，认为被告人佃锡煌的行为已触犯《中华人民共和国刑法》第三百四十一条之规定，应当以危害珍贵、濒危野生动物罪追究其刑事责任。被

告人佃锡煌有自首情节，根据《中华人民共和国刑法》第六十七条第一款之规定，可以从轻或者减轻处罚。被告人佃锡煌自愿如实供述自己的罪行，承认指控的犯罪事实，愿意接受处罚，根据《中华人民共和国刑事诉讼法》第十五条之规定，可以从宽处理。建议判处被告人佃锡煌有期徒刑三年至五年，并处罚金。诉请依法惩处。

梅州市梅县区人民检察院同时向本院提起附带民事公益诉讼，认为被告人佃锡煌的行为破坏了野生动物资源的多样性和生态环境的平衡，侵害了生态环境和资源保护公益，根据《中华人民共和国民法典》第一百七十九条、第一千二百二十九条、第一千二百三十五条之规定，请求判令被告人佃锡煌赔偿 104640 元用于野生动物保护和生态修复，并对其非法收购珍贵、濒危野生动物制品损害生态环境和资源保护的行为在市级以上的媒体上向公众公开赔礼道歉。

被告人佃锡煌对起诉书指控的犯罪事实无异议，表示认罪，请求法庭从轻处罚；对附带民事公益诉讼起诉人的诉讼请求无异议。辩护人对公诉机关指控被告人的行为构成危害珍贵、濒危野生动物罪无异议，对量刑提出：1.佃锡煌在接到办案机关的电话传唤后，如期到案主动接受讯问，并如实供述犯罪事实，具有自首情节，依法可以减轻处罚；2.佃锡煌法律意识淡薄，为治疗自身疾病使用民间偏方而购买了穿山甲鳞片自用，购入的价格不高，社会影响不大，且佃锡煌身患主动脉硬化、胆结石等多种疾

病，时常要到医院治疗，2020年先后两次到医院接受了手术治疗；3.佃锡煌是初犯、偶犯，在取保候审期间也遵守相关规定，没有再犯的危险。综上，请求法庭对被告人佃锡煌减轻处罚，并适用缓刑。

经审理查明，2017年，被告人佃锡煌为治疗自身疾病，未经野生动物保护部门批准，擅自在梅州市梅江区以每斤200元左右的价格向摆地摊的中年男子收购穿山甲鳞片约3.4公斤，放置于梅州市梅县区其居住的地方以备食用。2019年3月14日，梅州海关缉私分局依法在被告人佃锡煌位于梅县区的住家搜查出疑似穿山甲鳞片两包、手机两部及收据一批，并予以扣押。

2019年4月8日，经华南动物物种环境损害司法鉴定中心称重及鉴定，从被告人佃锡煌家中扣押的疑似穿山甲鳞片两包共净重3270克，分别系哺乳纲鳞甲目穿山甲科穿山甲属马来穿山甲的鳞片和树穿山甲的鳞片，该两类穿山甲均属于国家二级重点保护珍贵、濒危动物,穿山甲鳞片的涉案价值为104640元。查获的穿山甲鳞片已由梅州海关缉私分局移送广东省野生动物救护中心依法处置。

2020年3月25日，梅州市公安局梅县区分局电话传唤被告人佃锡煌，佃锡煌接电话后自行如期到案接受讯问，并如实供述其犯罪事实。

在诉讼过程中，被告人佃锡煌与公益诉讼起诉人达成了调解协议，被告人佃锡煌愿意按照公益诉讼起诉人的请求赔偿104640

元用于野生动物保护和生态修复，并已经在梅州市电视台对其非法收购珍贵、濒危野生动物制品损害生态环境和资源保护的行为向公众公开赔礼道歉。

上述事实，有经法庭出示、质证，并经庭审查证属实的梅州市公安局梅县区分局森林分局的受案登记表及梅州市公安局梅县区分局的立案决定书，梅州海关缉私分局受案登记表及立案决定书，搜查笔录，扣押决定书及扣押清单，梅州海关缉私分局出具的关于查获穿山甲鳞片重量的情况说明，被告人指认扣押物品照片，华南动物物种环境损害司法鉴定中心鉴定意见书、关于涉案动物制品价值的补充说明及关于送检材料的补充说明，现场勘查笔录、现场图、现场照片，被告人辨认现场笔录，证人李某、古某、杨某、王某1、王某2、温某、江某、梁某等人的证言，梅州海关缉私分局出具的查获穿山甲鳞片已移送广东省野生动物救护中心的情况说明，广东省野生动物救护中心出具的野生动物及其产品接收专用收据，梅州市梅县区人民检察院对佃锡煌提起民事公益诉讼的公告，南方医科大学口腔医院的诊断证明书、出院记录等书证，中山大学附属第一医院疾病证明书、出院记录等书证，梅江区看守所的暂不收押通知书，梅州市公安局梅县区分局森林分局出具的情况说明，被告人佃锡煌的供述，调解笔录，归案经过证明，户籍证明及无犯罪记录查询情况证明等证据证实。

本院认为，被告人佃锡煌违反野生动物保护法规定，为治疗自身疾病，非法向他人收购珍贵、濒危野生动物制品，数额达104640元，情节严重，其行为已构成危害珍贵、濒危野生动物罪，应依法惩处。公诉机关指控被告人的罪名成立。被告人佃锡煌的犯罪行为破坏了野生动物资源的多样性和生态环境的平衡，侵害了生态环境和资源保护公益，依法应承担相应的民事赔偿责任。公益诉讼起诉人提出的附带民事公益诉讼请求成立，予以支持。被告人佃锡煌与公益诉讼起诉人自愿达成调解协议，本院予以准许。案发后，被告人佃锡煌在接到公安机关电话传唤后，自行如期到案接受讯问，并如实供述其犯罪事实，视为自首，依法予以减轻处罚。被告人佃锡煌承认指控的犯罪事实，愿意接受处罚，且主动在市级电视台向公众公开赔礼道歉，愿意履行赔偿义务，依法予以从轻处罚。公诉机关的量刑建议理由充分，刑罚适当，依法予以采纳。辩护人请求从轻处罚意见可以酌情采纳。被告人收购珍贵、濒危野生动物制品的行为属情节严重，辩护人请求适用缓刑的辩护意见不予采纳。根据被告人的犯罪事实、性质、情节和对社会的危害程度，依照《中华人民共和国刑法》第三百四十一条、第六十七条第一款、第六十四条及《中华人民共和国民法典》第一百七十九条、第一千二百二十九条、第一千二百三十五条之规定，判决如下：

一、被告人佃锡煌犯危害珍贵、濒危野生动物罪，判处有期徒刑三年六个月，并处罚金人民币二万元。

（刑期从判决执行之日起计算，判决执行以前先行羁押二日，羁押一日折抵刑期一日，即自2021年8月31日起至2025年2月25日止；罚金自判决确定之日起一个月内缴纳）。

二、被告人佃锡煌应在本判决生效后十日内赔偿因非法收购珍贵、濒危野生动物制品造成的国家野生动物资源损失104640元，用于野生动物保护和生态修复。

三、被告人佃锡煌应在市级以上的媒体上对其非法收购珍贵、濒危野生动物制品损害生态环境和资源保护的行为向公众公开赔礼道歉（已履行完毕）。

四、扣押的穿山甲鳞片由广东省野生动物救护中心依法处置。

如不服本判决，可在接到判决书的第二日起十日内，通过本院或直接向广东省梅州市中级人民法院提出上诉。书面上诉的，应当提交上诉状正本一份、副本二份。

审　判　长　　魏伟辉

审　判　员　　魏东华

审　判　员　　张巧玲

人民陪审员　　冯才建

人民陪审员　　陈伟强

人民陪审员　　侯秀玲

人民陪审员　　唐　雯

二〇二一年八月三十一日

法官 助理 叶 舒

书 记 员 张旭辉
