# Supplementary material for: China’s Legal Protection System for Pangolins: Past, Present, and Future
Source: Animals (Basel). 2025 Aug 18;15(16):2422. doi: 10.3390/ani15162422 (PMC12383201; doi:10.3390/ani15162422)
Supplement: Supplementary file 1 [file animals-15-02422-s001.zip › Supplementary Material S4-Full Text of Judgments in Pangolin-Related Public Interest Litigation Cases in China/【14】黄某鹏;黄某师危害珍贵、濒危野生动物刑事一审刑事案件判决书.pdf]

黄某鹏;黄某师危害珍贵、濒危野生动物刑事一审刑事案件判决书

广西壮族自治区上思县人民法院

刑 事 附 带 民 事 判 决 书

（2021）桂0621刑初53号

公诉机关暨附带民事公益诉讼起诉人广西壮族自治区上思县人民检察院。

被告人黄某鹏，男，1990年2月23日出生于广西灵山县，汉族，本科肄业，务工人员，户籍所在地广西灵山县，住钦州市钦南区。因涉嫌犯非法出售珍贵、濒危野生动物制品罪于2020年11月26日被刑事拘留，同年12月24日被逮捕。现羁押于防城港市第一看守所。

辩护人陆党，广西桂信律师事务所律师。

律师助理杨开恩，广西桂信律师事务所实习律师。

被告人黄某师，男，1977年8月26日出生于广西上思县，壮族，中专文化，户籍所在地上思县，住上思县。因涉嫌犯非法收购珍贵、濒危野生动物制品罪于2020年11月30日被取保候审。

辩护人王粒冲，广西济顺律师事务所律师。

上思县人民检察院以上检刑诉〔2021〕Z19号起诉书指控被告人黄某鹏、黄某师犯危害珍贵、濒危野生动物罪，于2021年4月2日向本院提起公诉，本院于当日立案。在诉讼过程中，上

思县人民检察院以上检刑附民公诉〔2021〕1号刑事附带民事公益诉讼起诉书向本院提起附带民事公益诉讼。本院依法组成合议庭，于2021年7月21日公开开庭进行合并审理。上思县人民检察院指派检察官黄慧东、吴甘雨出庭支持公诉暨参加附带民事公益诉讼，被告人黄某鹏及其辩护人陆党、被告人黄某师及其辩护人王粒冲等到庭参加诉讼。现已审理终结。

上思县人民检察院指控，2020年10月20日，被告人黄某师联系被告人黄某鹏欲购买穿山甲鳞片。11月10日，黄某鹏联系“AAA文玩药材”帮黄某师购买穿山甲鳞片。11月12日，黄某鹏通过微信转发穿山甲鳞片的图片和视频给黄某师，黄某师决定购买穿山甲鳞片1千克，两人商定价格为每千克4200元。后黄某鹏转账3400元给“AAA文玩药材”用于购买穿山甲鳞片。11月18日，“AAA文玩药材”通过快递公司将疑似野生动物制品包裹寄给黄某鹏。11月25日18时许，黄某鹏到安州汽配城寄存点领取涉嫌野生动物制品包裹时被当场抓获。经检查，该包裹内装有疑似穿山甲鳞片，共重1千克。2020年11月26日，民警在上思县团结东路86号将被告人黄某师抓获。经云南濒科委司法鉴定中心对查获的疑似穿山甲鳞片1千克的名称、种属、保护级别及价值进行鉴定，鉴定的结论为：查获送检的疑似穿山甲鳞片来源于鳞甲目穿山甲科穿山甲属大穿山甲Manisgigantea，价值人民币10624元。对指控的事实，公诉机关提交了相应的证据予以证实。公诉机关认为，被告人黄某鹏、

黄某师的行为均构成危害珍贵、濒危野生动物罪。被告人黄某鹏、黄某师到案后，如实供述罪行，承认指控的犯罪事实，愿意接受处罚，可以从轻处罚。提请本院依法判处。

刑事附带民事公益诉讼起诉人上思县人民检察院提出附带民事公益诉讼请求：1.判令被告人黄某鹏、黄某师连带承担生态资源受损费用人民币10624元。2.判令被告人黄某鹏、黄某师当庭向社会公众赔礼道歉。

被告人黄某鹏对公诉机关指控的犯罪事实与罪名无异议，对附带民事公益诉讼起诉人提出的主张无异议，并表示愿意按照诉讼请求履行。其辩护人辩称，黄某鹏平时有工作，参与本案是因为帮助黄某师配药治病的需要，主观恶性不大。穿山甲鳞片是他人猎杀穿山甲后提取的动物制品，在取货时被公安机关当场抓获，社会危害性低。黄某鹏归案后如实供述犯罪事实及认罪认罚，又被关押了八个多月，受到了法律制裁。请求法庭适用缓刑。

被告人黄某师对公诉机关指控的犯罪事实与罪名无异议，对附带民事公益诉讼起诉人提出的主张无异议，并表示愿意按照诉讼请求履行。其辩护人辩称，黄某师没有收到穿山甲鳞片和支付货款，是犯罪未遂，可以判处更轻的刑罚。

经审理查明，2020年10月20日，被告人黄某师通过微信联系被告人黄某鹏欲购买穿山甲鳞片。11月10日，黄某鹏通过微信联系微信昵称为“ＡＡＡ文玩药材”购买穿山甲鳞片，经商量大甲片价格为每千克3400元，小甲片价格为每千克2500元。

11 月 12 日，黄某鹏通过微信转发穿山甲鳞片的图片和视频给黄某师，黄某师决定购买穿山甲鳞片 1 千克，两人商定价格为每千克 4200 元，两人约定黄某师收到甲片后付款给黄某鹏，并将收货地址和电话通过微信发给黄某鹏。后黄某鹏转 3400 元给“ＡＡＡ文玩药材”用于购买穿山甲鳞片，并将收货地址发给了“ＡＡＡ文玩药材”。11 月 18 日，“ＡＡＡ文玩药材”通过快递公司将 1 千克国家一级保护野生动物，经济价值为 10624 元的鳞甲目穿山甲科穿山甲属大穿山甲Ｍａｎｉｓｇｉｇａｎｔｅａ和南非穿山甲Ｍａｎｉｓｔｅｍｍｉｎｃｋｉｉ的鳞片寄给黄某鹏。11 月 25 日 18 时许，黄某鹏到钦州市钦南区安州汽配城Ｅ栋 008 号韵达快递安州汽配城寄存点领取涉嫌野生动物制品包裹时，被当场抓获。

在本案审理期间，黄某鹏、黄某师已经当庭向社会公众赔礼道歉。过后又交来 10624 元赔偿款。

上述事实有经庭审质证、确认的受案登记表，户籍证明，前科记录说明，抓获经过，搜查笔录及扣押清单，提取笔录、电子数据检查笔录，称重笔录，证人邓某、黎某证言，辨认笔录及照片，司法鉴定意见书，被告人黄某鹏、黄某师的供述等证据证实。

本院认为,被告人黄某鹏非法收购、出售国家一级重点保护的珍贵、濒危野生动物大穿山甲、南非穿山甲鳞片，被告人黄某师非法收购国家一级重点保护的珍贵、濒危野生动物大穿山甲、南非穿山甲鳞片，两被告人均构成危害珍贵、濒危野生动物罪。

公诉机关指控的罪名成立。被告人黄某鹏、黄某师到案后，如实供述罪行，愿意接受处罚，本院依法予以从轻处罚。被告人黄某鹏、黄某师危害珍贵、濒危野生动物，严重破坏野生动物资源，应承担相应的民事责任，附带民事公益诉讼起诉人的主张事实清楚，证据充分，本院予以支持。根据被告人的犯罪事实、性质、情节和对社会的危害程度，依照2015年《中华人民共和国刑法》第三百四十一条第一款、第十二条、第五十二条、第五十三条、第六十七条第一款、第七十二条第一、三款、第七十三条第二、三款，《中华人民共和国野生动物保护法》第三条第一款、第二十七条第一款，《中华人民共和国民法典》第九条、第一百七十九条第一款第（八）项和第（十一）项、第一百八十七条、第一千一百六十八条、第一千二百二十九条，《最高人民法院、最高人民检察院关于检察公益诉讼案件适用法律若干问题的解释》第二十条之规定，判决如下：

一、被告人黄某鹏犯危害珍贵、濒危野生动物罪，判处有期徒刑九个月，并处罚金人民币五千元；

（刑期从判决执行之日起计算，判决执行以前先行羁押的，羁押一日折抵刑期一日，即自2020年11月26日起至2021年8月25日止。罚金限于判决生效后30日内向本院缴纳，期满未缴纳的，强制缴纳。）

二、被告人黄某师犯危害珍贵、濒危野生动物罪，判处有期徒刑九个月，缓刑一年六个月，并处罚金人民币五千元。

（缓刑考验期限，从判决确定之日起计算；罚金限于判决生效后30日内向本院缴纳，期满未缴纳的，强制缴纳。）

三、判令被告人黄某鹏、黄某师连带承担生态资源受损费用人民币10624元。

四、判令被告人黄某鹏、黄某师当庭向社会公众赔礼道歉。

如不服本判决，可在接到判决书的第二日起十日内，通过本院或者直接向广西壮族自治区防城港市中级人民法院提出上诉。书面上诉的，应当提交上诉状正本一份，副本八份。

审　判　长　　吕德钦

审　判　员　　苏润青

审　判　员　　赵世强

人民陪审员　　陆　颖

人民陪审员　　何景鑫

人民陪审员　　陶文兴

人民陪审员　　唐庆烈

二〇二一年八月十六日

法官　助理　　唐　瑶

书　记　员　　陈炳妃

附相关法条：

《中华人民共和国刑法》

第三百四十一条非法猎捕、杀害国家重点保护的珍贵、濒危野生动物的，或者非法收购、运输、出售国家重点保护的珍贵、

濒危野生动物及其制品的，处五年以下有期徒刑或者拘役，并处罚金；情节严重的，处五年以上十年以下有期徒刑，并处罚金；情节特别严重的，处十年以上有期徒刑，并处罚金或者没收财产。违反狩猎法规，在禁猎区、禁猎期或者使用禁用的工具、方法进行狩猎，破坏野生动物资源，情节严重的，处三年以下有期徒刑、拘役、管制或者罚金。

第十二条中华人民共和国成立以后本法施行以前的行为，如果当时的法律不认为是犯罪的，适用当时的法律；如果当时的法律认为是犯罪的，依照本法总则第四章第八节的规定应当追诉的，按照当时的法律追究刑事责任，但是如果本法不认为是犯罪或者处刑较轻的，适用本法。

本法施行以前，依照当时的法律已经作出的生效判决，继续有效。第五十二条判处罚金，应当根据犯罪情节决定罚金数额。

第五十三条罚金在判决指定的期限内一次或者分期缴纳。期满不缴纳的，强制缴纳。对于不能全部缴纳罚金的，人民法院在任何时候发现被执行人有可以执行的财产，应当随时追缴。

由于遭遇不能抗拒的灾祸等原因缴纳确实有困难的，经人民法院裁定，可以延期缴纳、酌情减少或者免除。

第六十七条犯罪以后自动投案，如实供述自己的罪行的，是自首。对于自首的犯罪分子，可以从轻或者减轻处罚。其中，犯罪较轻的，可以免除处罚。

被采取强制措施的被告人、被告人和正在服刑的罪犯，如实供述司法机关还未掌握的本人其他罪行的，以自首论。

被告人虽不具有前两款规定的自首情节，但是如实供述自己罪行的，可以从轻处罚；因其如实供述自己罪行，避免特别严重后果发生的，可以减轻处罚。

第七十二条对于被判处拘役、三年以下有期徒刑的犯罪分子，同时符合下列条件的，可以宣告缓刑，对其中不满十八周岁的人、怀孕的妇女和已满七十五周岁的人，应当宣告缓刑：

（一）犯罪情节较轻；

（二）有悔罪表现；

（三）没有再犯罪的危险；

（四）宣告缓刑对所居住社区没有重大不良影响。

宣告缓刑，可以根据犯罪情况，同时禁止犯罪分子在缓刑考验期限内从事特定活动，进入特定区域、场所，接触特定的人。

被宣告缓刑的犯罪分子，如果被判处附加刑，附加刑仍须执行。

第七十三条拘役的缓刑考验期限为原判刑期以上一年以下，但是不能少于二个月。

有期徒刑的缓刑考验期限为原判刑期以上五年以下，但是不能少于一年。

缓刑考验期限，从判决确定之日起计算。

《中华人民共和国野生动物保护法》

第三条野生动物资源属于国家所有。

国家保障依法从事野生动物科学研究、人工繁育等保护及相关活动的组织和个人的合法权益。

第二十七条禁止出售、购买、利用国家重点保护野生动物及其制品。

因科学研究、人工繁育、公众展示展演、文物保护或者其他特殊情况，需要出售、购买、利用国家重点保护野生动物及其制品的，应当经省、自治区、直辖市人民政府野生动物保护主管部门批准，并按照规定取得和使用专用标识，保证可追溯，但国务院对批准机关另有规定的除外。

实行国家重点保护野生动物及其制品专用标识的范围和管理办法，由国务院野生动物保护主管部门规定。

出售、利用非国家重点保护野生动物的，应当提供狩猎、进出口等合法来源证明。

出售本条第二款、第四款规定的野生动物的，还应当依法附有检疫证明。

《中华人民共和国民法典》

第九条民事主体从事民事活动，应当有利于节约资源、保护生态环境。

第一百七十九条承担民事责任的方式主要有：

（一）停止侵害；

（二）排除妨碍；

（三）消除危险；

（四）返还财产；

（五）恢复原状；

（六）修理、重作、更换；

（七）继续履行；

（八）赔偿损失；

（九）支付违约金；

（十）消除影响、恢复名誉；

（十一）赔礼道歉。

法律规定惩罚性赔偿的，依照其规定。

本条规定的承担民事责任的方式，可以单独适用，也可以合并适用。

第一百八十七条民事主体因同一行为应当承担民事责任、行政责任和刑事责任的，承担行政责任或者刑事责任不影响承担民事责任；民事主体的财产不足以支付的，优先用于承担民事责任。

第一千一百六十八条二人以上共同实施侵权行为，造成他人损害的，应当承担连带责任。

第一千二百二十九条因污染环境、破坏生态造成他人损害的，侵权人应当承担侵权责任。

《最高人民法院、最高人民检察院关于检察公益诉讼案件适用法律若干问题的解释》

第二十条人民检察院对破坏生态环境和资源保护，食品药品安全领域侵害众多消费者合法权益，侵害英雄烈士等的姓名、肖像、名誉、荣誉等损害社会公共利益的犯罪行为提起刑事公诉时，可以向人民法院一并提起附带民事公益诉讼，由人民法院同一审判组织审理。

人民检察院提起的刑事附带民事公益诉讼案件由审理刑事案件的人民法院管辖。
