# Supplementary material for: China’s Legal Protection System for Pangolins: Past, Present, and Future
Source: Animals (Basel). 2025 Aug 18;15(16):2422. doi: 10.3390/ani15162422 (PMC12383201; doi:10.3390/ani15162422)
Supplement: Supplementary file 1 [file animals-15-02422-s001.zip › Supplementary Material S4-Full Text of Judgments in Pangolin-Related Public Interest Litigation Cases in China/【15】蒙某、广西壮族自治区平南县人民检察院非法收购、运输、出售珍贵、濒危野生动物、珍贵、濒危野生动物制品罪刑事一审刑事判决书.pdf]

蒙某、广西壮族自治区平南县人民检察院非法收购、运输、出售珍贵、濒危野生动物、珍贵、濒危野生动物制品罪刑事一审刑事判决书

广西壮族自治区平南县人民法院

刑 事 附 带 民 事 判 决 书

（2021）桂 0821 刑初 78 号

公诉机关暨刑事附带民事公益诉讼起诉人广西壮族自治区平南县人民检察院。

被告人蒙定，男，1976 年 10 月 31 日出生，汉族，初中文化，住广西平南县。因涉嫌犯危害珍贵、濒危野生动物罪于 2020 年 9 月 9 日被刑事拘留，同年 10 月 15 日被逮捕。现羁押于平南县看守所。

辩护人暨附带民事公益诉讼代理人蒙建花，广西正大五星（港南）律师事务所律师。

平南县人民检察院以平检刑诉［2021］18 号起诉书指控被告人蒙定犯危害珍贵、濒危野生动物罪，于 2021 年 1 月 15 日向本院提起公诉，并于 2021 年 1 月 19 日以平检民行刑附民公诉[2021]1 号刑事附带民事公益诉讼起诉书对被告人蒙定提起附带民事公益诉讼，本院于同年 1 月 21 日立案后，依法组成合议庭，于 2021 年 3 月 24 日公开开庭进行了合并审理。平南县人民检察院指派检察员谢喜昌出庭支持公诉，指派检察员卢坚出庭就刑事

附带民事公益诉讼部分发表意见，被告人蒙定及其辩护人暨诉讼代理人蒙建花到庭参加了诉讼。现已审理终结。

平南县人民检察院指控，在2018年下半年到2019年上半年，被告人蒙定在其位于平南县蒙定金银首饰店内先后销售穿山甲片900克、500克给朱某2（另案处理），朱某2将穿山甲片拿回其经营的坤大首饰店加工销售，后被平南县森林公安查获朱某2在蒙定处购买的尚未销售出去的穿山甲片及其制品净重302.2克。2020年6月25日，被告人蒙定在其经营首饰店内以7667元价钱分两次出售穿山甲片共2434克给朱某1（另案处理），朱某1将穿山甲片拿回其经营的坤大首饰店加工销售，后被平南县森林公安查获朱某1在蒙定处收购的尚未销售出去的穿山甲片及其制品净重833.6克。2020年6月28日平南县森林公安局在被告人蒙定经营的首饰店查获鳞甲目穿山甲属动物鳞片净重0.03千克，日常销售记录涉及穿山甲片加工出售记录为517笔，涉及销售金额共人民币133715元。

公诉机关对指控的事实提供了相应的证据予以证实，被告人蒙定明知是穿山甲片而非法收购、加工、出售穿山甲制品，其行为已经触犯了《中华人民共和国刑法》第三百四十一条的规定，犯罪事实清楚，证据确实充分，应当以危害珍贵、濒危野生动物罪追究其刑事责任。提请本院依法作出判处。

公益诉讼起诉人认为蒙定非法收购、出售珍贵、濒危野生动物制品，破坏野生动物资源，危害生态平衡和生态安全，损坏国

家利益和社会公共利益，根据《最高人民法院、最高人民检察院关于检察公益诉讼案件适用法律若干问题的解释》第二十条的规定，提请本院判令被告人蒙定赔偿涉案濒危野生动物穿山甲制品的价值损失共计人民币7753元，并在判决生效之日起一个月内在市级以上媒体公开道歉。

被告人蒙定提出其没有买卖野生动物制品的行为的辩解意见，公诉机关指控其犯危害珍贵、濒危野生动物罪不能成立，不应当承担公益诉讼赔偿责任。

辩护人蒙建花提出公诉机关指控被告人犯危害珍贵、濒危野生动物罪证据不足；且侦查机关存在程序违法，所收集的证据不能作为证据使用的辩护意见。蒙建花还提出生态环境造成的损失无法证实由被告人行为造成，被告人不应当承担赔偿、赔礼道歉义务的代理意见。

经审理查明，在2018年下半年到2019年上半年，被告人蒙定在其位于平南县蒙定金银首饰店内先后销售穿山甲片给朱某2（已判刑），朱某2将穿山甲片拿回其经营的坤大首饰店加工销售，后公安机关在朱某2处查获尚未销售的鲮鲤科穿山甲属动物甲片及其制品净重361克（其余鉴定不出属性）。2020年6月26日，被告人蒙定在其经营首饰店内以7667元价钱分两次出售穿山甲片给朱某1（已判刑），朱某1将穿山甲片拿回其经营的坤大首饰加工销售，公安机关在朱某1处查获鳞甲目鲮鲤科穿山甲属动物鳞片及其制品净重1545克（其余鉴定不出属性）。2020

年6月28日公安机关在被告人蒙定经营的首饰店查获鳞甲目穿山甲属动物鳞片净重30克，日常销售记录涉及穿山甲片加工出售记录为500余笔，销售金额共人民币13万余元。经鉴定，在蒙定处查获的30克鳞甲目穿山甲属动物鳞片价值86元。

另查明，被告人的行为造成生态环境受到损害，功能丧失导致的损失为7753元。

上述事实，有公诉机关提供，并经法庭举证、质证、查证属实的下列证据予以证实：

1.受案登记表、立案决定书证实，本案受案、立案情况。

2.户籍证明证实，蒙定于1976年10月31日出生，案发时已达到刑事责任年龄。

3.到案经过证实，2020年9月9日蒙定被公安机关抓获。

4.搜查证、搜查笔录、照片、扣押决定书及清单证实，2020年6月28日公安机关在蒙定处扣押疑似龟甲2个，疑似穿山甲片217.4克（珠子）、30.3克半加工甲片，紫色ＶＩＶＯ手机一台、销售记录22本等物品，搜查过程中朱某1全程在场见证。

5.销售记录清单、活页，蒙定指认销售记录本照片证实，蒙定加工出售穿山甲制品的销售记录，涉及穿山甲加工出售记录有500余次，涉及销售金额13万余元。

6.微信聊天记录、微信转账截图证实，2020年7月28日公安机关依法对朱某1手机信息进行恢复、固定和提取电子数据，朱某1与蒙定商议关于穿山甲片买卖的事情，后朱某1于2020

年6月26日17时许转账7667元给蒙定，聊天和转账记录经朱某1指认。

7.国家林业和草原局公告证实，2020年6月3日国家林业和草原局发布公告将穿山甲属所有种由国家二级保护野生动物调整为国家一级保护野生动物。

8.证人朱某1证言，其经朱某2介绍后得知蒙定处有穿山甲片出售。2020年5、6月份其通过微信联系蒙定购买甲片，在微信商谈好价格后，其亲自到蒙定的店里拿了497.9克甲片，另外1936.1克是蒙定通过平南至六陈镇的客车寄给其的，蒙定把甲片邮寄后专门拍了一张邮寄班车的照片给其。其在蒙定处购买的上述2434克穿山甲片部分甲片已用来加工，部分还是完整甲片，购买这些甲片其通过微信支付了7667元购买甲片款给蒙定。

9.证人朱某2证言，2018年下半年其到蒙定的首饰店里以2970元价钱购买了约900克甲片。2019年上半年，其到蒙定金银首饰店又购买了500克这样的穿山甲片，加上一些银首饰一共转账约4000元给蒙定。公安机关在2020年6月28日在其经营的坤大首饰店内搜出的疑是穿山甲制品和穿山甲片，以及同年6月30日其亲自上交公安机关的其中26片厚的穿山甲片，都是从蒙定那里购买的。买货回来后用店内机器进行加工成吊牌和珠子，卖给顾客。其余公安机关查获的159片穿山甲片薄片是平时顾客拿料到店里加工留下来的边料。

10.证人潘某证言，2020年6月份前其是蒙定金银首饰加工店营业员，老板是蒙定，其负责出货并在销售登记本上做好记录，卖了什么东西就在上面登记并签字，其签的是艳字，都是如实记录的。平时在店里摆有甲片和甲珠，出卖了也要登记，其不知道甲片和甲珠的来源。

11.被告人蒙定供述，2002年开始其在平南县开蒙定金银加工首饰店。经清点公安机关在其经营的店铺搜出的物品疑似穿山甲珠子217.4克，疑似穿山甲片30.3克，疑似龟甲2个。疑似龟甲是在2019年10月份在玉林花鸟市场买的。疑似穿山甲珠子217.4克，疑似穿山甲片30.3克是平时在店里帮顾客加工疑似穿山甲片时自己留下来的。其雇请3个女员工，平时店里的销售，销售员都会做好记录。经清点销售记录中涉及穿山甲的出售记录有500余次，销售金额13万余元。朱某1曾通过微信转了7667元的货款给其。

12.鉴定意见（1）广西壮族自治区森林公安局物证鉴定所桂林公（刑）鉴（法物）字［2020］201、222、224号鉴定书证实，在蒙定处扣押的动物产品中经鉴定为鳞甲目穿山甲属动物鳞片产品的重0.03千克；在朱某1处扣押到疑似穿山甲片和疑似穿山甲加工成的珠子、摆件以及疑似龟片经鉴定，属于鳞甲目鲮鲤科（穿山甲科）穿山甲属动物鳞片产品的重1.545千克（价值4423元）；在朱某2处扣押到的疑是甲片经鉴定属于鲮鲤科穿山甲属动物产品的共重0.361千克。

（2）平南县价格认定中心平价公定（2020）280、281、283号关于穿山甲甲片的价格认定结论书证实，0.03千克穿山甲甲片价值86元、0.3746千克穿山甲价值1072元，1.545千克穿山甲片价值4423元。

13.现场勘验笔录、现场图及照片证实，蒙定金银加工首饰店的位置，现场照片可见现场扣押的疑似穿山甲片制品。

14.辨认笔录及照片证实，朱某1、朱某2辨认出出卖穿山甲片的人就是蒙定。

15.指认照片证实，蒙定指认在其经营的店铺内扣押的疑似穿山甲及制品等物品；朱某1、朱某2指认出蒙定交易穿山甲片的地点即蒙定金银首饰店，指认与蒙定购买的穿山甲片；朱某1、朱某2指认蒙定微信；朱某1指认2020年6月26日与蒙定购买穿山甲片的微信交易记录照片。

16.电子证据检查工作记录、提取到的电子证据证实，2020年7月20日公安机关对朱某1使用的手机进行电子数据检查，提取到朱某1“朱二136××××****”与蒙定“蒙定137××××****”的聊天和转账记录，2020年6月26日朱某1通过微信转账了7667元作为甲片的货款，并且证实在交易之前蒙定和朱某1在商谈交易价格。

17.公益诉讼起诉人提供的主要证据材料亦和公诉人提供的基本一致。

本院认为，被告人蒙定明知是珍贵、濒危野生动物制品而非法收购、加工、出售，其行为已触犯刑律，应以危害珍贵、濒危野生动物罪追究其刑事责任，依法应处五年以下有期徒刑或者拘役，并处罚金。公诉机关指控被告人蒙定犯危害珍贵、濒危野生动物罪罪名成立。关于被告人蒙定及辩护人蒙建花提出公诉机关指控被告人蒙定出售珍贵、濒危野生动物制品的行为证据不足，指定的罪名不能成立的辩解及辩护意见，经查，蒙定向朱某 1、朱某 2 等人加工、销售珍贵、濒危野生动物制品的事实，有购买人朱某 1、朱某 2 的证言，销售人员潘某的证言以及销售记录本、朱某 1 与蒙定的聊天、转账记录、辨认笔录予以证实，并且公安民警还分别从朱某 1、朱某 2、蒙定处分别扣押到涉案的珍贵、濒危野生动物甲片及制品，亦佐证了蒙定向朱某 1、朱某 2 等人出售珍贵、濒危野生动物制品的事实，故对被告人及辩护人提出的上述辩解及辩护意见，本院不予采纳；关于辩护人提出的侦查机关侦查程序严重违法，提取到的证据不能作为定案依据的辩护意见，没有事实依据，本院不予采纳。被告人蒙定因其行为对生态环境造成的损失，应予以赔偿，并根据侵权责任应当承担赔礼道歉的义务。依照《中华人民共和国刑法》第三百四十一条第一款、第四十七条、第五十二条、第五十三条、《中华人民共和国民法典》第一百七十九条、第一千二百三十五条以及《最高人民法院关于审理破坏野生动物资源刑事案件具体应用法律若干问题的解释》第一条、第二条、《最高人民法院、最高人民检察院

关于检察公益诉讼案件适用法律若干问题的解释》第二十条的规定，判决如下：

一、被告人蒙定犯危害珍贵、濒危野生动物罪，判处有期徒刑二年，并处罚金人民币十万元；

（刑期从判决执行之日起计算。判决执行以前先行羁押的，羁押一日折抵刑期一日，即自2020年9月9日起至2022年9月8日止；罚金限在判决生效后十日内缴纳。期满不缴纳的，强制缴纳。）

二、被告人蒙定在本判决生效后十日内赔偿因其行为导致的生态环境造成的损失共计人民币7753元给附带民事公益诉讼起诉人平南县人民检察院，并在判决生效之日起一个月内在市级以上媒体公开道歉。

如不服本判决，可在接到判决书的第二日起十日内，通过本院或者直接向贵港市中级人民法院提出上诉。书面上诉的，应当提交上诉状正本一份，副本五份。

审　判　长　　杨华卿

人民陪审员　　胡琼连

人民陪审员　　廖日凤

二〇二一年四月十三日

法官　助理　　韦露丝

书　记　员　　郑敏慧
