# Supplementary material for: China’s Legal Protection System for Pangolins: Past, Present, and Future
Source: Animals (Basel). 2025 Aug 18;15(16):2422. doi: 10.3390/ani15162422 (PMC12383201; doi:10.3390/ani15162422)
Supplement: Supplementary file 1 [file animals-15-02422-s001.zip › Supplementary Material S4-Full Text of Judgments in Pangolin-Related Public Interest Litigation Cases in China/【16】崇左市人民检察院、吕明、蒙广辉等走私珍贵动物、珍贵动物制品罪一审刑事判决书.pdf]

崇左市人民检察院、吕明、蒙广辉等走私珍贵动物、珍贵动物制品罪一审刑事判决书

广西壮族自治区崇左市中级人民法院

刑 事 附 带 民 事 判 决 书

（2020）桂14刑初48号

公诉机关暨公益诉讼起诉人广西壮族自治区崇左市人民检察院。

被告人暨刑事附带民事公益诉讼被告王亚伟，男，1990年4月8日出生，汉族，初中文化，农民，住安徽省亳州市谯城区。因涉嫌犯走私珍贵动物制品罪，于2020年3月10日被指定监视居住，同年3月26日被刑事拘留，同年4月15日被逮捕，现羁押于凭祥市看守所。

辩护人暨委托诉讼代理人杨晓峰，广西程和程律师事务所律师。

辩护人暨委托诉讼代理人关海涛，安徽彭线旗律师事务所律师。

被告人暨刑事附带民事公益诉讼被告全高德，绰号“阿小”，男，1986年11月22日出生，壮族，小学文化，农民，住广西凭祥市。因涉嫌犯走私珍贵动物制品罪，于2020年3月9日刑事拘留，同年4月15日被逮捕，现羁押于凭祥市看守所。

辩护人暨委托诉讼代理人马清文，广西欣源律师事务所律师。

被告人暨刑事附带民事公益诉讼被告潘礼勇，男，1986 年 12 月 6 日出生，汉族，初中文化，农民，住广西凭祥市（户籍地：广西桂平市）。因涉嫌犯走私珍贵动物制品罪，于 2020 年 3 月 10 日被刑事拘留，同年 4 月 15 日被逮捕，现羁押于崇左市看守所。

辩护人暨委托诉讼代理人赵荣科，广西起航律师事务所律师。

辩护人暨委托诉讼代理人渠双平，北京市铭基律师事务所律师。

被告人暨刑事附带民事公益诉讼被告鄂东丰，绰号“老鄂”，男，1971 年 1 月 31 日出生，壮族，小学文化，农民，住广西凭祥市。因涉嫌犯走私珍贵动物制品罪，于 2020 年 3 月 9 日被刑事拘留，同年 4 月 15 日被逮捕，现羁押于凭祥市看守所。

辩护人暨委托诉讼代理人梁汉松，广西程和程律师事务所律师。

被告人暨刑事附带民事公益诉讼被告吕明，男，1973 年 9 月 22 日出生，壮族，初中文化，农民，住广西凭祥市。因涉嫌犯走私珍贵动物制品罪，于 2020 年 3 月 9 日被刑事拘留，同年 4 月 15 日被逮捕，现羁押于崇左市看守所。

辩护人暨委托诉讼代理人伦武，广西聚同律师事务所律师。

被告人暨刑事附带民事公益诉讼被告蒙广辉，男，1987 年 6 月 7 日出生，壮族，中专文化，农民，住广西凭祥市。因涉嫌犯

走私珍贵动物制品罪，于2020年3月10日被刑事拘留，同年4月15日被逮捕，现羁押于崇左市看守所。

辩护人暨委托诉讼代理人唐诗雅，广西东方意远（凭祥）律师事务所律师。

被告人暨刑事附带民事公益诉讼被告何云强，男，1979年1月26日出生，壮族，小学文化，农民，住广西宁明县。因涉嫌犯走私珍贵动物制品罪，于2020年3月24日被凭祥海关缉私分局取保候审，同年6月16日被崇左市人民检察院取保候审，2021年5月14日被逮捕，现羁押于凭祥市看守所。

崇左市人民检察院以崇检刑诉（2020）61号起诉书指控被告人王亚伟、全高德、潘礼勇、鄂东丰、蒙广辉、吕明、何云强等七人犯走私珍贵动物制品罪，于2020年7月20日向本院提起公诉。在诉讼过程中，公益诉讼起诉人崇左市人民检察院以崇检刑附民公诉（2020）1号刑事附带民事公益诉讼起诉书向本院提起附带民事公益诉讼。经查，崇左市人民检察院于2020年5月21日公告了案件相关情况，公告期内未有法律规定的机关和有关组织提起民事公益诉讼。本院依法组成合议庭，公开开庭进行了合并审理。崇左市人民检察院指派检察员蒙加信出庭支持公诉，指派副检察长、检察员潘小玉出庭对公益诉讼履行职务。被告人暨刑事附带民事公益诉讼被告王亚伟、全高德、潘礼勇、鄂东丰、蒙广辉、吕明、何云强及其各自辩护人暨委托诉讼代理人杨晓峰、关海涛，马清文，赵荣科、渠双平，梁汉松、伦武、唐

诗雅到庭参加诉讼。期间，因本案附带民事诉讼延期审理一次。本案现已审理终结。

崇左市人民检察院指控：2019 年 12 月开始，被告人王亚伟与王某（另案处理）合谋从越南走私穿山甲鳞片运至安徽亳州等地销售牟利，由王亚伟负责联系越南卖家、协商价格及安排接收、发运穿山甲鳞片等事宜，王某负责在亳州收货、销售、支付货款。为确保穿山甲鳞片顺利运达亳州，王亚伟雇请被告人全高德负责组织人员前往中越边境接收、送至指定地点；雇请被告人潘礼勇负责组织人员将接收到的穿山甲鳞片，进行重新包装，再通过百世快运物流发运至亳州让王某接货。

一、2020 年 3 月 4 日，王亚伟通知全高德去中越边境接收穿山甲鳞片。同月 5 日，全高德安排被告人鄂东丰前往中越边境 1099 号界碑附近，接收他人从越南偷运入境的穿山甲鳞片，并驳装至车牌号为桂Ｆ×××××的面包车上，由被告人吕明驾驶该车拉运至全高德家中存放。期间，全高德驾车在路段望风看路、指挥吕明行车。同月 6 日，全高德驾驶车牌号为桂Ｆ×××××的面包车将上述穿山甲鳞片运到凭祥市锦华酒店附近停车，将车钥匙留在车内后离开。随后被告人何云强根据潘礼勇的安排，前往停车点驾驶该桂Ｆ×××××面包车到位于那勤村水泥厂附近的一物流仓库内，将车上的穿山甲鳞片进行拆分和重新包装后，通过百世快运物流发往亳州。

同月7日，王亚伟再次通知全高德接收穿山甲鳞片，全高德再次安排鄂东丰到边境接货、吕明驾车拉运至全高德家卸货存放。同月8日，全高德驾驶车牌号为桂Ｆ×××××的面包车将该批穿山甲鳞片运至凭祥市停车，将车钥匙留在车内后离开。何云强按照潘礼勇安排，前往上述停车点将桂Ｆ×××××的面包车驾驶到物流仓库，将车上的穿山甲鳞片重新包装后，再次通过百世快运物流发往亳州。

同月9日凌晨，凭祥海关缉私分局在南宁百世快运中转仓库内查获运单号为11610145164、11610145186快递两单，缴获了穿山甲鳞片两批，总净重462.06千克。

二、2020年3月8日凌晨，全高德根据王亚伟的通知，安排被告人蒙广辉驾驶车牌号为桂Ｆ×××××的面包车，到中越边境1130至1132号界碑（隘口村）附近，接取从越南偷运入境的穿山甲鳞片一批，并拉运到全高德家存放。当日23时许，凭祥海关缉私分局在全高德位于广西凭祥市的家中查获穿山甲鳞片12袋，总净重300.2千克。另外，凭祥海关缉私分局在潘礼勇家中查获穿山甲鳞片4小袋，总净重4.72千克；在全高德的家中查获穿山甲鳞片5袋，总净重5.98千克；在鄂东丰的家中查获干海马24只，总净重68.7克。经鉴定，本案缴获的穿山甲鳞片为树穿山甲、南非穿山甲、黑腹长尾穿山甲、大穿山甲所有，均列入《濒危野生动植物种国际贸易公约》（ＣＩＴＥＳ）附录Ⅰ，经济价值为人民币6852.7153万元（以下币种均为人民币）；

缴获的疑似海马干为棘海马，列入国家ＩＩ级保护动物和《濒危野生动植物种国际贸易公约》（ＣＩＴＥＳ）附录ＩＩ，经济价值为0.36万元。

经查实，王亚伟走私入境的穿山甲鳞片共计768.56千克，经济价值为6821.9792万元；全高德走私入境的穿山甲鳞片共计774.54千克，经济价值为6840.6865万元；潘礼勇走私入境的穿山甲鳞片共计473.08千克，经济价值为4169.3385万元；鄂东丰走私入境的穿山甲鳞片共计468.36千克、干海马68.7克，经济价值为4157.6697万元；蒙广辉走私入境的穿山甲鳞片共计300.2千克，经济价值为2664.6695万元；吕明走私入境的穿山甲鳞片共计468.36千克，经济价值为4157.3097万元；何云强走私入境的穿山甲鳞片共计468.36千克,经济价值为4157.3097万元。

公诉机关为证实以上事实，向本院移送并当庭出示、宣读了相关物证，书证，证人证言，被告人供述和辩解，鉴定意见，勘验、检查、辨认笔录，视听资料，电子证据等。

公诉机关认为，被告人王亚伟、全高德、潘礼勇、鄂东丰、蒙广辉、吕明、何云强违反海关法规，逃避海关监管，走私珍贵动物制品入境，其行为触犯了《中华人民共和国刑法》第一百五十一条第二款，犯罪事实清楚，证据确实、充分，应当以走私珍贵动物制品罪追究其刑事责任。提请本院依法判处。

附带民事公益诉讼起诉人崇左市人民检察院向本院提出诉讼请求。1．判令王亚伟、全高德、鄂东丰、吕明、潘礼勇、何云强共同承担因侵权行为造成的野生动物资源损失费 41573097 元。2．判令王亚伟、全高德、蒙广辉共同承担因侵权行为造成的野生动物资源损失费 26646695 元。3．判令鄂东丰承担因侵权造成的野生动物资源损失费 3600 元。4．判令全高德承担因侵权造成的野生动物资源损失费 187073 元。5．判令潘礼勇承担因侵权造成的野生动物资源损失费 120288 元。6．判令王亚伟、全高德、潘礼勇、鄂东丰、蒙广辉、吕明、何云强对其侵权行为在国家级以上媒体向社会公众赔礼道歉。

事实和理由：刑事附带民事公益诉讼被告实施了刑事诉讼指控的破坏生态环境的行为，应当承担相应的赔偿责任。穿山甲属于国家二级保护动物，列入《濒危野生动植物种国际贸易公约》（CITES）附录 I。野生动物资源属国家所有，且我国是濒危野生动植物种国际贸易公约缔约国，依法负有保护职责。王亚伟、全高德、潘礼勇、鄂东丰、蒙广辉、吕明、何云强的行为造成野生动物资源受损、生物多样性遭到破坏，已对生态环境造成严重破坏，导致国家利益和社会公共利益受损。根据《中华人民共和国野生动物保护法》、《中华人民共和国环境保护法》、《最高人民法院关于审理环境民事公益诉讼案件适用法律若干问题的解释》、《最高人民法院最高人民检察院关于检察公益诉讼案件

适用法律若干问题的解释》及《中华人民共和国民事诉讼法》等有关规定，提起附带民事公益诉讼，请依法判处。

被告人暨刑事附带民事公益诉讼被告王亚伟辩称涉案穿山甲鳞片鉴定价值过高。愿意承担其行为造成的生态资源损坏赔偿费。其辩护人提出：1．涉案走私的鳞片鉴定价值过高，且应区分货物是野生还是人工饲养；2．对王亚伟量刑应考虑王亚伟在本案中的地位作用；3．王亚伟归案后认罪态度好，如实供述，积极配合，无犯罪前科；4．王亚伟有两个未成年的小孩及年迈的父母需要抚养；综上建议对王亚伟从轻判处。5．对于民事诉讼，因本案因果关系不明，不能依照鉴定价值作为要求被告人承担赔偿数额的依据，应当驳回公益诉讼的诉讼请求。

被告人暨刑事附带民事公益诉讼被告全高德辩称涉案穿山甲鳞片鉴定价值过高。愿意承担其行为造成的生态资源损坏赔偿费，但数额太高其赔偿不起。其辩护人提出：1．全高德受他人雇请，是从犯，应从轻、减轻处罚；2．指控全高德的数额有误，数额相差6.3千克，全高德从越南购买的鳞片是为了给其姐姐治病；3．涉案鳞片价值鉴定过高，希望能重新鉴定；4．全高德到案后如实供述，自愿认罪认罚，无前科。建议对全高德从宽处罚，建议量刑判处有期徒刑五到十年，罚金五到十万元。

5.民事诉讼请求直接套用刑事案件涉案价值没有法律依据，建议法庭驳回。

被告人暨刑事附带民事公益诉讼被告潘礼勇辩称涉案穿山甲鳞片鉴定价值过高。愿意在能力范围内承担生态资源损坏赔偿费。其辩护人提出：1．潘礼勇没有走私穿山甲鳞片的主观故意和逃避海关监管的行为，其行为构成掩饰隐瞒犯罪所得罪，不构成走私珍贵动物制品罪；2．潘礼勇没有直接参与走私，社会危害性小，其受人雇请仅起到辅助的作用，是从犯，其与王亚伟不属于刑法第一百五十六条规定的走私犯共犯；3．潘礼勇有坦白情节，认罪认罚，积极退赃，有悔罪表现，无前科；4．云南濒科委司法鉴定执行出具的鉴定意见，不能作为认定本案涉案穿山甲鳞片价值的认定。5．公益诉讼起诉人提起公益诉讼没有事实及法律依据，与目前司法案例相悖，依法应当驳回。

被告人暨刑事附带民事公益诉讼被告鄂东丰对指控的事实和罪名均没有异议，愿意承担其行为造成的生态资源损坏赔偿费，但数额太高其赔偿不起。其辩护人提出：1．鄂东丰是从犯中的从犯；2．鄂东丰认罪态度好，认罪认罚；3．涉案鳞片鉴定价格过高；4．鄂东丰家庭经济困难，其主观恶性小。建议对鄂东丰从轻判处。5．猎杀野生动物才能提起公益诉讼，本案是走私，不应提起公益诉讼。

被告人暨刑事附带民事公益诉讼被告蒙广辉对指控的事实和罪名均没有异议，且愿意承担其行为造成的生态资源损坏赔偿费。其辩护人提出：1．涉案鳞片鉴定价值过高；2．蒙广辉是从犯；3．蒙广辉没有前科是初犯、偶犯，主观恶性不大；4．蒙广

辉愿意退赃，且其家庭经济困难。建议对蒙广辉从轻处罚。5．公益诉讼起诉人提起公益诉讼没有事实及法律依据，依法应当驳回公益起诉人的诉讼请求。

被告人暨刑事附带民事公益诉讼被告吕明辩称其只是一名司机，受全高德的雇请拉货，没有参与分红，也不知道拉的货物为何物，其愿意认罪认罚，但不同意赔偿生态资源的费用。其辩护人提出：吕明是从犯，归案后认罪态度好，认罪认罚，且家庭困难，建议对吕明从轻、减轻处罚。公益诉讼起诉人提起公益诉讼依据不严谨，依法应当驳回。

被告人暨刑事附带民事公益诉讼被告何云强辩称其只是个打工的，其愿意承担其行为造成的生态资源损坏赔偿费，但数额太高其承担不起。希望法庭对其从轻处理，并判处缓刑。

潘礼勇辩护人向本院提交转账收据和赡养证明，拟证实潘礼勇家属已代潘礼勇退出其非法所得4万元及潘礼勇需抚养一个叫潘胜奇的老人。

鄂东丰的辩护人提交了《今日头条》截图及渠历村出具的证明，拟证实根据穿山甲鳞片的照片，大概二三十片就达到了360克，云南鉴定中心的鉴定意见不科学；鄂东丰需照顾其亲属的情况。

各被告人及其他辩护人均无证据向法庭提交。

经审理查明，2020年3月至案发时，被告人王亚伟与王某（另案处理）合谋从越南走私穿山甲鳞片运至国内安徽亳州等地

销售牟利。由王亚伟负责联系越南卖家、商议购买价格、并组织人员在凭祥市接收、发运穿山甲鳞片，王某在安徽亳州负责接收、销售。在实施过程中，王亚伟与被告人全高德商定，由全高德负责组织人员前往中越边境接运偷运入境的穿山甲鳞片送至凭祥市区指定地点，王亚伟按每千克130元的价格支付给全高德报酬。同时，王亚伟与被告人潘礼勇商定，由潘礼勇安排人员到指定地点接收穿山甲鳞片进行拆分、重新包装后，通过百世快运物流发送至安徽亳州，王亚伟以每千克80元的价格支付潘礼勇通过物流寄运的费用。

2020年3月4日，被告人王亚伟与越南穿山甲鳞片卖家联系后，通知被告人全高德到中越边境接收。次日，全高德安排被告人鄂东丰前往中越边境1099号界碑附近，接收他人从越南偷运入境的穿山甲鳞片，并安排被告人吕明负责驾车（车牌号桂Ｆ×××××）拉运涉案穿山甲鳞片，后吕明拉运至全高德家中存放。期间，全高德驾车在路段望风，指挥吕明行车路线。同月6日，全高德驾驶车牌号为桂Ｆ×××××的面包车将上述穿山甲鳞片运到凭祥市锦华酒店附近停车，将车钥匙留在车内后离开。随后被告人何云强根据潘礼勇的安排，前往停车点驾驶该桂Ｆ×××××面包车到位于那勤村水泥厂附近的一物流仓库内，将车上的穿山甲鳞片进行拆分和重新包装后，通过百世快运物流发往亳州。

同月 7 日，被告人王亚伟又通知全高德接收穿山甲鳞片，被告人全高德便又安排被告人鄂东丰到边境接货，安排被告人吕明驾车（车牌号桂Ｆ×××××）拉运至全高德家卸货存放。次日，全高德驾驶车牌号为桂Ｆ×××××的面包车将该批穿山甲鳞片运至凭祥市停车，将车钥匙留在车内后离开。被告人何云强按照潘礼勇安排，前往上述停车点将桂Ｆ×××××的面包车驾驶到百世快运物流仓库，将车上的穿山甲鳞片重新包装后，再次通过百世快运物流发往亳州。同月 9 日凌晨，凭祥海关缉私分局在南宁百世快运中转仓库内查获运单号为 11610145164、11610145186 快递两单，缴获了穿山甲鳞片两批，第一批净重 225.34 千克，第二批净重 236.72 千克，总净重 462.06 千克。

2020 年 3 月 8 日凌晨，被告人王亚伟再次通知被告人全高德到边境接取走私入境的穿山甲鳞片，全高德安排被告人蒙广辉驾驶车牌号为桂Ｆ×××××的面包车，到中越边境 1130 至 1132 号界碑（隘口村）附近，接取从越南偷运入境的穿山甲鳞片，并拉运到全高德家存放。当日 23 时许，凭祥海关缉私分局在全高德位于广西凭祥市的家中查获该穿山甲鳞片 12 袋，总净重 300.2 千克。

另外，凭祥海关缉私分局在潘礼勇家中查获穿山甲鳞片 4 小袋，总净重 4.72 千克；在全高德的家中查获穿山甲鳞片 5 袋，总净重 5.98 千克；在鄂东丰的家中查获棘海马 24 只，总净重 68.7 克。同月 24 日，凭祥海关缉私分局在潘礼勇租用的凭祥市

水泥厂附近一仓库里的一辆废弃小货车副驾驶室内查获何云强未发运的穿山甲鳞片 6.3 千克。

经鉴定，本案缴获的全部穿山甲鳞片为树穿山甲、南非穿山甲、黑腹长尾穿山甲、大穿山甲所有，为国家二级保护动物，均列入《濒危野生动植物种国际贸易公约》（ＣＩＴＥＳ）附录Ｉ，缴获的疑似海马干为棘海马，列入国家ＩＩ级保护动物和《濒危野生动植物种国际贸易公约（ＣＩＴＥＳ）附录ＩＩ。第一批穿山甲鳞片 225.34 千克，经济价值为 20008860 元。第二批穿山甲鳞片 236.72 千克，经济价值为 21012011 元。第三批穿山甲鳞片 300.20 千克，经济价值为 26646695 元。在凭祥市内查获穿山甲鳞片 6.3 千克，经济价值为 559200 元。在全高德的家中查获穿山甲鳞片净重 5.98 千克，经济价值为 187073 元。在潘礼勇家中查获的穿山甲鳞片 4.72 千克，经济价值为 120288 元。在鄂东丰的家中查获的棘海马净重 68.9 克，经济价值为 3600 元，总计经济价值为 68527153 元。

经查实，王亚伟走私入境的穿山甲鳞片共计 768.56 千克，经济价值为 68219792 元；全高德走私入境的穿山甲鳞片共计 774.54 千克，经济价值为 68406865 元；潘礼勇走私入境的穿山甲鳞片共计 473.08 千克，经济价值为 41693385 元；鄂东丰走私入境的穿山甲鳞片共计 468.36 千克、棘海马 68.7 克，经济价值为 41576697 元；蒙广辉走私入境的穿山甲鳞片共计 300.2 千克，经济价值为 26646695 元；吕明走私入境的穿山甲鳞片共计

468.36 千克，经济价值为 41573097 元；何云强走私入境的穿山甲鳞片共计 468.36 千克，经济价值为 41573097 元。

另查明，被告人王亚伟、潘礼勇、蒙广辉、何云强在审查起诉阶段自愿认罪认罚，并签署了认罪认罚具结书，同意公诉机关量刑建议：1．判处王亚伟有期徒刑十年至十四年，并处罚金人民币八十万至一百五十万元；2．判处潘礼勇、蒙广辉有期徒刑四年至六年，并处罚金人民币八万至二十万元；3．判处何云强有期徒刑二年至五年，并处罚金人民币一万至三万元。被告人全高德、鄂东丰、吕明在法庭审理阶段自愿认罪认罚。

上诉事实有公诉机关当庭举证、质证的下列证据证实：

1．受案登记表、立案决定书证实：凭祥海关缉私分局根据线索于 2020 年 3 月 8 日对本案立案侦查。

2．涉案物证照片证实：侦查机关查获的部分涉案疑似穿山甲鳞片、棘海马的特征、数量等。

3．抓获（到案）经过证实：2020 年 3 月 8 日，侦查人员将被告人王亚伟、全高德、潘礼勇、鄂东丰、蒙广辉、吕明等 6 人抓获归案，同月 24 日，被告人何云强主动到凭祥海关缉私分局投案。

4．搜查笔录、扣押决定书、扣押清单、过磅记录证实：（1）侦查机关在凭祥市前进村派桑屯二队 31-2 号全高德家中，扣押用黄色编织袋包装疑似穿山甲鳞片 12 袋，净重 300.2 千克；透明塑料袋包装疑似穿山甲鳞片 5 袋，净重 5.98 千克；在潘礼勇

住所查获疑似穿山甲鳞片４袋，净重４.72千克；在南宁市平安不动产Ａ1栋４号库查扣快递编号为“11610145164”疑似穿山甲鳞片共计20件，总共净重225.34千克；在南宁市平安不动产Ａ1栋４号库百世快运处查扣快递编号为“11610145186”的疑似穿山甲鳞片共净重236.72千克；在凭祥市水泥厂附近一仓库里内扣押疑似穿山甲鳞片２袋，净重6.3千克；在鄂东丰凭祥市友谊镇委渠历屯18号家中，查获涉案棘海马净重68.7克。（2）扣押王亚伟手机二部、工商银行卡一张（尾号0418）；扣押全高德手机一部、桂Ｆ×××××五菱牌面包车一辆、邮政储蓄卡一张（尾号6336）；扣押潘礼勇手机三部中国银行（尾号3515）、农业银行银行卡各一张（尾号5819）；扣押鄂东丰手机二部；扣押蒙广辉手机二部；扣押吕明手机一部、桂Ｆ×××××五菱牌面包车一辆等物品。

５．取样记录证实：侦查机关对涉案单位制品提取样本进行封存送检。

６．银行流水明细、协助冻结财产通知书证实：户名为ＶＵＴＲＡＧＩＡＮＧ的农业银行账户（尾号4376）流水与王某银行账户有交易往来，账户余额为20139.44元，并予以冻结；王亚伟邮政银行卡（尾号6336）账号与62284********账号有多次交易记录；

7．合肥海关缉私局“3.7走私珍贵动物制品案”案卷壹卷证实：涉及本案的王某证言、银行卡流水明细；涉嫌走私穿山甲鳞片案鉴定意见等。

8．证人王某证言证实：2019年5月，王亚伟找到其说在广西凭祥那边能找到穿山甲鳞片，搞回亳州可以赚钱。后两人商量好，王亚伟负责在广西凭祥跟“阿飞”（能从越南拿到穿山甲鳞片）对接，王亚伟在凭祥收到货后，通过百世快运邮寄到亳州，其在亳州负责接货和销售，再把货款打给王亚伟。其主要使用尾号为9431的农村信用社银行卡和微信名叫“阿莫西林”给王亚伟转账；用尾号为9672的农业银行卡给名叫“ＶＵＴＲＡＧＩＡＮＧ”的越南账户转货款，前后订了二笔货共800公斤，分2次转订货款共53.6万元。

9．证人白某证言证实：其系百世快运凭祥2部的员工，潘礼勇是老板。2020年3月8日，网店接收潘礼勇19件棕色纸箱包装的货物，从凭祥发往安徽，因潘礼勇是老板，其不验货。

10.证人贾某证言证实:其是与王亚伟到凭祥考察药材生意。

11．证人黄某1证言证实：其系百世快运凭祥2部的员工，潘礼勇是老板。2020年3月7日、8日潘礼勇自己拉过两批货到网点，是潘礼勇自己接的单。3月7日共发货20件，收货人是李志；3月8日，发货共19件，收件人记录是“陈”，两人均是安徽亳州市人。

12．证人蒙某1证言证实：2020年3月7日，蒙广辉跟其借车牌号为桂ＦＰ61**面包车，说去装点货，并透露是越南走私进来的。当天晚上，蒙广辉给其发微信，让其在渠光屯村口检查点当志愿者时帮忙看路。

13．证人蒙某2证言证实：2020年3月7日下午，蒙广辉打电话给其去给他看路，蒙广辉在中越1130-1134号界碑附近帮别人出货。蒙广辉花名“阿辉”。

14．证人蒙某3证言证实：2020年3月8日凌晨，蒙广辉通过微信电话让其开摩托车上渠广屯后山“32码头”（中越边境1132号界碑）把蒙广辉在越南的货拉到村里，其因车坏了没有去。

15．证人黄某2证言证实：2019年底，顺丰快递收到一批八角，收件人驾驶一部面包车收货；2020年3月8日，安徽亳州人王某通过顺丰快递往凭祥发来220公斤八角，顺丰公司准备派件的时候发现收件人电话已经关机，后该批货物退回。

16．王亚伟、王某出入境记录证实：被告人王亚伟2018年8月14日至2020年1月19日有多次出关到越南；王某2019年10月16日、17日出关到越南。

17．送所羁押的情况说明证实：被告人潘礼勇、蒙广辉、吕明、全高德、鄂东丰于2020年3月9日被拘留，因新型冠状肺炎疫情的原因，需进行核酸检测，同月11日完成收押。

18．户籍证明、无犯罪记录证明证实：本案被告人案发时均已达到完全刑事责任年龄，且无犯罪记录。

19．电子证据检验报告南缉刑鉴电字［2020］021、107-118号证实：侦查机关依法提取涉案手机内的电子数据，手机微信信息记录等涉及本案的走私情况。

20．云南濒科委司法鉴定中心司法鉴定意见书濒司鉴（动）字［2020］290号及鉴定意见通知书证实：涉案动物制品的种属、保护级别及价值分别为树穿山甲、南非穿山甲、大穿山甲、黑腹长尾穿山甲，所有穿山甲均属于《濒危野生动植物种国际贸易公约》（CITES）Ⅰ级保护物种；棘海马属于《濒危野生动植物种国际贸易公约》（CITES）II级保护物种、国家II级保护物种，总经济价值为68530753元。上述鉴定意见已告知七被告人。

21．辨认笔录证实：（1）被告人王亚伟辨认出被告人全高德、潘礼勇，“老鄂”即被告人鄂东丰、“阿飞”即古黄飞、王某、被告人何云强；（2）被告人全高德辨认出王亚伟、“鄂哥”、“老鄂”即鄂东丰、“阿辉”即蒙广辉、被告人吕明；（3）潘礼勇辨认出王亚伟（4）鄂东丰辨认出“阿小”即全高德；（5）蒙广辉辨认出“姐夫”即全高德；（6）吕明辨认出全高德；（7）何云强辨认出潘礼勇、王亚伟；（8）蒙某1辨认出蒙广辉。

22．现场勘查检查工作笔录、现场勘查照片证实：侦查机关对涉案货物走私进境、过驳及交货地点进行现场勘查，被告人对现场进行了指认。

23．视听及电子数据光盘证实：凭祥海关缉私分局依法讯问被告人及辩认过程等进行了同步录音录像。

24．被告人王亚伟供述：大约是 2019 年 12 月，其去越南跟供货人阿飞（音）联系，他跟其说他有 16 吨的穿山甲鳞片，但是货还在非洲，其回家之后把情况跟王某说，王某也有意向跟阿飞合作，让其帮忙联系。2020 年 3 月 1 日，阿飞报了穿山甲鳞片的价格给其，长片每公斤 800 元，圆片每公斤 950 元，阿飞负责把货运到凭祥。同月 3 日晚上其从安徽到凭祥与阿飞见面，他说现在货没办法送到凭祥，要其找人在边境接货。次日中午，其通过约全高德见面，其问他能不能帮其出穿山甲鳞片，他说可以帮出，商定的价格是从谅山运到凭祥每公斤要 130 元报酬，当时全高德给了其一个谅山接货人的电话，让其把货交给这个人，其它事不要其管，其就把这个谅山接货人的电话发给阿飞，阿飞说先做 800 公斤的穿山甲鳞片长片，最后分三批入境，其记得第一批是 250 公斤，3 月 6 日到货，第二批是 250 公斤，3 月 7 日到货，最后一批是 300 公斤，3 月 8 日到货。

这 800 公斤穿山甲鳞片订货后，其把阿飞提供的账户给王某，所有购买穿山甲鳞片的费用以及做工费用都是由王某出，其微信上也有转账记录。这 800 公斤穿山甲鳞片其纯粹是帮王某牵线，其没有任何报酬。全高德跟其要第一批出货的工钱时，其到他家里去，看到“老鄂”也在，全高德说是“老鄂”帮忙做出来的。第二批 250 公斤穿山甲鳞片也是通过这个接货电话做出来。

但是第三批货在出货之前，全高德打电话给其说找阿辉做，收押最后一次 300 公斤是由阿辉帮做入境的。每批穿山甲鳞片都是晚上交接货入境，但是具体在边境什么位置入境其不清楚。其不认识阿辉，其只是听全高德提过名字，“老鄂”其认识。

为了将穿山甲鳞片通过物流发运，其通过农文庆介绍知道百世快运可以帮运送穿山甲鳞片，2019 年 10 月，其找到凭祥的百世快运的老板阿勇，跟他商谈。2020 年 3 月这 800 公斤穿山甲鳞片其找阿勇帮其发运物流，运费每公斤 80 元，不包含打包费。当时阿勇说货不要直接送到百世快运，送到大富豪路口。每次需要发运物流，其都是等全高德通知，然后联系阿勇派人去与全高德交接货，阿勇在凭祥把穿山甲鳞片打包、装运之后，把快递单号通过微信发给其，其记得这 800 公斤鳞片已经办理了两批物流，共 500 公斤货，最后一批 300 公斤还没有办理就被抓了，这些穿山甲鳞片都是发运到安徽亳州给王某，收货人地址、电话也是王某提供给其的。其是微信昵称“！”，阿勇使用的微信昵称是“勇往直前”。

经查看其与阿勇合作穿山甲鳞片的微信聊天记录，其可以确认，2020 年 3 月 7 日阿勇发给其“11610145164”、2020 年 3 月 8 日阿勇发给其“11610145186”，这两则消息内容代表 2 批每批 250 公斤穿山甲鳞片的快递单号。全高德是用一辆灰色的五菱牌，车牌号带有“285”字样，当时其也通过微信把车牌号发给了阿勇。经查看其与潘礼勇的微信聊天记录，有一个“Ｖ”字（Ｖ

ＵＴＲＡＧＩＡＮ6）开头的越南人开户名，（账户 62×××76），是其将该账户发给了王某进行了货款转账。2020 年 3 月 4 日，王某用尾号“9672”的账户该账户转了一笔 170000 元和一笔 165000 的款，共计 335000 元，这是 500 公斤穿山甲鳞片的货款；同年3月7日王某用尾号“9672”的账户向该账户转了一笔100000 元和一笔 101000 元的款，共计 201000 元，这是 300 公斤穿山甲鳞片的货款。以上 4 笔款项共计 536000 元是向阿飞订购 800 公斤穿山甲鳞片的钱。2020 年 3 月 6 日王某用尾号 9672 的账户向其转账 20000 元，同月 8 日向其转账 20000 元，其中有 20000 元是王某帮阿飞垫付给全高德的做工费，还有 20000 元应该是借给全高德的钱。

25．被告人全高德供述：大概是今年的 3 月初，王亚伟联系其说他最近在越南有一批穿山甲鳞片大概是 500 公斤，想找其帮忙弄到国内来，为了能赚点钱，当时商量好，王亚伟负责联系越南货主，其在弄怀那边找码头把鳞片从越南偷运进来，王亚伟按照每公斤鳞片 130 元的工钱给其。从 3 月 4 日到被抓获当日，其一共帮王亚伟从越南运了三次穿山甲鳞片到中国，一共是 800 公斤。

第一次是今年的 3 月 4 日晚上，王亚伟发微信给其说今晚做 250 公斤，其就告诉王亚伟让越南货主把鳞片搬到渠历那里的码头，王亚伟把越南货主的电话发给其，然后其找了“鄂哥”，把越南货主的电话告诉“鄂哥”，由“鄂哥”找越南工人把 250 公

斤鳞片从越南搬运到国内来，其又找其老表吕明开他的面包车把鳞片装车拉到其家里来，其和吕明一起卸货，一共是10包，用黄色编织袋装的，和被抓获当晚在其家查扣的那12包一样的包装。第二天中午，王亚伟告诉其把鳞片装到面包车上，送到原来检验检疫局门口，让其把车停在那里，车钥匙留在车上，他派人过来接货，同日下午王亚伟给了其2万块工钱，然后还给了其一张银行卡，让其拿着这张银行卡再取2万块，第一次王亚伟一共给了其4万块的工钱。

渠历是在弄怀互市区里面，大概是109几到1100界碑那一带，“鄂哥”是从这一带的中越界碑的码头把穿山甲鳞片从越南运到中国的。“鄂哥”是渠历那里的人，其没有和他具体说是什么货，但是他肯定知道是走私货。吕明肯定也知道是走私货，因为是在界碑那里搬过来的，都是越南工人搬。

第二次和第一次的情况差不多，3月6日晚上，王亚伟说今晚再做250公斤，其还是让他告诉越南货主把鳞片运到渠历那里，其再告诉“鄂哥”，“鄂哥”再联系越南工人把鳞片从越南那边搬过来装到吕明的面包车上，然后吕明再开车拉到其家里。因为当晚“鄂哥”说越南那边不给上货，所以这次鳞片一直到第二天中午才从渠历出来拉到其家里。3月8日中午，王亚伟让其把鳞片装好，开车到原来检验检疫局门口他派人过来接车，后他让其从那张银行卡里再取2万块钱工钱。其面包车车号是桂F××××。吕明的面包车的车号是桂F车牌，尾数是2218。

第三次是 3 月 7 日中午，王亚伟告诉其还有 300 公斤的鳞片要过来，王亚伟还说做这些鳞片不赚什么钱，想按照每公斤 120 块钱的工钱给其，其想了想也答应了。因为“鄂哥”说渠历那里越南那边暂时上不了货，所以其就找了“阿辉”，“阿辉”说越南工人的工钱要 40 块钱每公斤，“阿辉”他自己要收 50 块每公斤，其自己得 30 块钱每公斤，其就答应了“阿辉”。其把王亚伟给其的越南货主电话给了“阿辉”，由他联系越南货主和工人，3 月 8 日中午，“阿辉”安排面包车把 300 公斤的鳞片拉到其家，还没来得及交给王亚伟，就被查获了。“鄂哥”的工钱还没有给，“阿辉”的工钱其先给了 2 万元，其老婆也在场。其在地图上确认将第二批穿山甲鳞片交给王亚伟的位置、锦华酒店旁边的交货现场照片及桂 F×××××五菱面包车行车记录。

另外在其住的房间里，在一楼上二楼的楼梯上有个铁皮柜，在铁皮柜里放有用塑料袋装的 5 小袋穿山甲鳞片，大概有 5-6 斤，这 5 小袋鳞片是其从越南买回来给其姐姐做药用的。

26．被告人潘礼勇供述：2019 年 6 月，其认识的“阿伟”（王亚伟）。同年 7 月左右，其开始帮“阿伟”发运穿山甲鳞片到南宁再到安徽，其开始不知道那些“鳞片”是什么，后“阿伟”说是穿山甲鳞片但是是用来做药材，其见有钱赚也装作不知道了，其还分四次偷了“阿伟”4 小袋的穿山甲麟片放到其家里阳台柜子里。当时“阿伟”告诉其他会找人开一台面包车拉着穿山甲鳞片到大富豪 KTV 附近的路口停在路边，司机把车钥匙放在车

上然后离开，这时候其联系“老何”去找到那台面包车把货拉回百世快运公司。2020年3月6日，“阿伟”通知其有250公斤穿山甲鳞片到了大富豪KTV附近的路边面包车里，其又让“老何”去开那台面包车把穿山甲鳞片拉到其在那勤村水泥砖厂附近的一个仓库里，并且让“老何”把这些蛇皮袋包装的穿山甲鳞片在仓库内的一个小房间里面重新拆开换纸箱包装。但是当天因为纸箱不够，3月7日打包好了才发往南宁。次日，其让“老何”又按照同样的流程去接了“阿伟”的250公斤穿山甲鳞片并且重新打包更换包装，“老何”打包好后，其把打包好的穿山甲鳞片拉回百世快运公司贴好面单再给“老何”和其他货物一起发运到南宁分拣中心去。“老何”打包时在穿山甲鳞片上面铺上一些八角，然后封好箱子。因为“老何”很老实，其开始以为自己犯的错误是小错误，觉得“老何”是无辜的，对不起他，所以其把“老何”去做的事都说成是其自己做了。2019年其帮“阿伟”运输的穿山甲麟片量不是很多，其2019年收到报酬2万元钱左右，2020年3月7日发运完第一批穿山甲鳞片以后其收到了“阿伟”2万元报酬，第二批穿山甲鳞片报酬还没结算。八角是“阿伟”给其的。其确认《王亚伟与潘礼勇微信聊天记录》表的内容，是其和“阿伟”的微信聊天记录，里面讲到的“等下其叫他开机”是指等下其叫“老何”开机。“你叫他放车在路边”是其叫“阿伟”让送货人像以前一样放车在大富豪KTV路边，钥匙放车上。“开了，但是他没拿，他在街上过去”是指“老何”没拿那台手机，

他现在在街上送货，直接去大富豪KTV附近接货了。“17156512757”、“安徽省亳州市谯城区天运物流园”就是“阿伟”告诉其发运3月6日那批穿山甲鳞片的收货地址和电话号码。“今天来不及了”、“明天打包先”是指其所供述的3月6日接到的那批穿山甲鳞片因为纸箱不够没有打包完，3月7日打包完再发运到南宁再到安徽。“公斤数”、“250”就是指这批穿山甲鳞片有250公斤。“11610145164”是3月6日接到但是在3月7日才发运到南宁再到安徽的那批250公斤穿山甲鳞片的快运单号。“安徽省亳州市谯城区建设南湖春城”、“17105571072”是指3月8日那250公斤穿山甲鳞片的收货地址和电话号码。“11610145186”是指3月8日那批穿山甲鳞片的快运单号。其让黄某1将其填写的面单录入系统。2020年3月24日，民警在其位于凭祥市水泥厂附近的仓库中搜查并扣押了约6公斤的疑似穿山甲鳞片，其不知情。其确认全高德涉案走私数量及价值统计表，共涉嫌走私473.08千克，价值41693385元及在其家查获净重4.72千克的鳞片。

27．被告人鄂东丰供述：上个月“阿小”（全高德）跟其说有个安徽老板找他帮忙出点鳞片，帮接一次货给其1万块钱。第一次2020年3月4日晚上，“阿小”打电话给其，要其到渠历1094界碑附近等越南人送货过来，送货的两个越南人分别叫“阿忠”、“阿道”。其开着摩托车过去，从越南人手里接了10袋鳞片，鳞片使用黄色编织袋包装，其接到后在路边等并打电话给

“阿小”，“阿小”安排了一台面包车来接货，其帮忙把鳞片搬到车上后就回家了。第二次是3月6日，也是晚上10点钟左右，跟第一次一样，其又接了10袋鳞片，也是黄色编织袋包装，也是面包车把鳞片拉走。其搬货的时候感觉每袋有20斤左右，其总共帮他接了20袋，加起来大概400斤。“阿小”给了其1万元报酬。

28. 被告人蒙广辉供述：2020年3月6日，姐夫（全高德）跟其联系，说这两天有鳞片出来，让其帮接一下，工钱每公斤90块钱，货到了之后越南送货人会打电话联系其。3月7日“姐夫”发微信告诉其有12包的鳞片，其跟朋友蒙某1借了一辆面包车（车牌是桂F×××××），晚上越南人打电话给其说货到了，大概凌晨1点，其开面包车上到隘口街后面小路跟越南人对接，具体入境点在1130界碑至1132界碑这一段边境。货都搬上面包车上后其发微信给姐夫说货已经接到，第二天下午，其把货拉到派桑屯姐夫家里，共12袋，姐夫说每袋25公斤，总共300公斤，晚上姐夫拿了2万块钱给其。

29. 被告人吕明供述：2020年3月5日晚，其老表全高德叫其去渠历帮拉货，到了之后，几个越南工人把货搬到其车上，卸货的时候其才知道一共有10包；第二次是3月7日晚上，全高德叫其去渠历村边界拉货，装货地点在渠历村路口进去，下坡之后拐两个弯就到了，在山脚下有一条小路通到越南，货是越南工人从小路搬过来装到其车上，然后其拉到全高德家里卸货，也

是 10 包。具体是什么货全高德没有跟其说。货都是用蛇皮袋包装。这些货都是从越南那边搬山搬过来的走私货，搬货的时候感觉很轻，松松的，里面东西是一片一片。全高德主要是负责看路面情况。

30．被告人何云强供述：2019 年 7、8 月，其老板潘礼勇跟其说，客户有一些药材要其去装一下拉回公司发物流，客户已经把货装在面包车上了，车型是短板款五菱面包车，到了之后会有人跟其电话联系，并且给其一部用来联系的按键手机。其按照潘礼勇的指示，来到凭祥市旁，当时其看到他所说的面包车，但是没有看到司机，潘礼勇告诉其车钥匙放在车座上，让其开车回百世快运公司就可以了。其驾驶那部面包车到位于凭祥市内的百世快运公司后，潘礼勇让其把车上的货搬下来，那些货都是用纸箱包装的，其没有打开看，但摸起来是片状物，潘礼勇让其把这些货搬到公司里的员工宿舍小屋后，让其把面包车停回大富豪路口。2019 年 10 月份左右，潘礼勇又让其去大富豪路口接了一车货到百世快运公司的小屋，具体经过与第一次情况一样，那批货同样是片状物品。

今年（2020 年）3 月 7 日，潘礼勇又要其去凭祥大富豪路口接手一辆装有货的面包车，并且给了其一部按键手机。之后，其骑着电车去了大富豪路口，当时有一名男子走过来跟其说，车在香格里拉-陌陌酒吧路段，那名男子也跟着其一起过去跟其说，车就在前面第一个车位，说完就离开了，其看到锦华停车场门口

路边停了一辆面包车，车牌尾数是285，这部车与2019年其去大富豪路口接货的车是同一辆车，其驾驶这部面包车把货拉回百世快运公司，潘礼勇让其把货搬到公司小屋里，这批货是黄色编织袋包装的，总共有10袋，潘礼勇让其把这些货拆开重新打包，并告诉其公司阁楼上有八角，打包的时候每一包放一些八角，其打开黄色编织袋之后，发现这些货是一些鳞片状物，其把编织袋里的货用黄色纸箱进行打包，并且在每包货里都放进去一些八角，其打包途中用手机搜索了一下这些货物，百度上显示这些货是穿山甲鳞片。这批穿山甲鳞片总共打了20个纸箱的包装件，当天下午，和其它物流快递货物一起装到物流车上，其开车运到南宁百世快运物流总部。第二次是2020年3月8日下午，潘礼勇又通知其去接装有货的面包车，方式方法同第一次一样，一个男子打那部按键手机告诉其面包车停放的位置，车辆同样是那部尾号为285的面包车，货物同样是黄色编织袋包装的穿山甲鳞片，数量是10袋，其按照潘礼勇的要求将车开回到公司，不过拉到公司的另外一个新仓库去打包，当时潘礼勇驾车给其带路，到了之后，潘礼勇从新仓库拿出八角和纸箱包装给其，让其打包，这一次打包了19件纸箱件，当天下午7点钟左右，这19个纸箱的穿山甲鳞片和其它物流货物一起装到物流车发往南宁百世快运总部。

另查明，刑事附带民事公益诉讼起诉人除了认可本案公诉人所举的刑事部分证据亦作为其提起民事公益诉讼证据之外，还提

交了崇左市人民检察院线索移送函、公告、立案决定书和孟涛博士关于王亚伟等人走私珍贵野生动物制品对生态环境损害的说明，拟证实崇左市人民检察院作为公益诉讼起诉人程序合法，主体适格。孟涛博士的意见认为本案被告王亚伟等人走私珍贵野生动物制品，间接造成大量穿山甲死亡，影响森林生态结构的完整，破坏当地生态平衡和生态系统的稳定，进而导致全球生态环境受到损害。

以上证据来源合法，真实有效。且证据相互印证，已形成证据链条，足以认定。

针对各被告人及其辩护人提出的辩解和辩护意见，根据本案的事实和证据，依照法律规定，本院综合评判意见如下：

1．关于刑事附带民事公益诉讼。民事公益诉讼被告王亚伟、全高德、潘礼勇、鄂东丰、蒙广辉、吕明、何云强共同走私穿山甲鳞片，对于造成生态资源损害具有直接因果关系，起诉人起诉要求其承担公益侵害责任，符合民事公益诉讼的起诉条件。穿山甲属于国家二级保护动物，列入《濒危野生动植物种国际贸易公约》（CITES）附录Ⅰ，我国也是濒危野生动植物种国际贸易公约缔约国，依法负有保护职责。穿山甲有主要的生长区域，其的生长、生存已与当地生态环境形成了相对固定的生态关系，这种动态平衡关系维系着当地的生态平衡。人为造成穿山甲种群数量的减少，直接影响着生态系统的稳定性。七被告的行为造成野生动物资源受损、生物多样性遭到破坏，已对生态环境造成严重损

害，导致国家利益和社会公共利益受损。各被告虽不是直接猎杀者，但其走私行为推动了野生动物资源损失的进度，加快了物种灭绝的速度。鄂东丰还走私棘海马，亦应承担赔偿责任。故七被告行为造成野生动物资源损失，依法应当承担民事赔偿责任，本院确定各被告行为造成损失数额如下：王亚伟对走私入境共计 768.56 千克（225.34+236.72+300.2+6.3），价值共 68219792 元的穿山甲鳞片承担责任，全高德除与王亚伟承担上述责任外，还单独承担其购买放在家中的穿山甲鳞片 5.98 克，价值 187073 元的责任，即 68406865（68219792+187073）元；潘礼勇对其中 473.08 千克（225.34+236.72+4.72+6.3），价值 41693385 元的穿山甲鳞片共同承担责任；鄂东丰除对其中 468.36 千克（225.34+236.72+6.3），价值 41573097 元穿山甲鳞片共同承担责任外，还对 68.7 克，价值 3600 元的棘海马价值承担责任，即共 41576697 元；蒙广辉对其中 300.2 千克，价值 26646695 元的穿山甲鳞片共同承担责任；吕明、何云强对其中 468.36 千克（225.34+236.72+6.3），价值 41573097 元穿山甲鳞片共同承担责任。故在总的野生动物资源损失费 68527153 元中，被告王亚伟、全高德、潘礼勇、鄂东丰、吕明、何云强共同承担其中 41573097 元，王亚伟、全高德、蒙广辉共同承担其中 26646695 元，全高德、潘礼勇、鄂东丰分别单独承担其中 187073 元、120288 元和 3600 元。被告及其代理人提出被告不承担赔偿责任的答辩意见不予支持。

2．关于云南濒科委司法鉴定中心出具的涉案穿山甲鳞片的价值鉴定意见是否可以采信的问题。经查，该鉴定系侦查机关委托具有鉴定质证的人员和部门依法作出，鉴定依据、程序及鉴定结果合法、准确，应予采信，对于辩护人提出鉴定意见系根据专家的意见作出，没有法律依据，鉴定的价值偏高与实际不符，不应采信的意见。根据《最高人民法院最高人民检察院公安部司法部关于依法惩治非法野生动物交易犯罪的指导意见》第七条的规定，对于专门性问题可参照具有专门知识的人出具的认定意见作出，故云南濒科委司法鉴定中心根据专家意见及掌握的单只穿山甲鳞片重量等事实，折算涉案穿山甲数量，再根据现行有效的原国家林业局的有关计算涉案野生动物的价值，且涉案的穿山甲及棘海马均不是驯养的动物，故该鉴定意见符合本案事实，应予采信。被告人及辩护人对此提出的异议意见不能成立，不予采纳。

3．关于各被告人的犯罪地位与作用。经查，被告人王亚伟为获取不法利益，与境外走私分子共谋走私，且积极联系全高德、潘礼勇等将涉案物品走私入境，并运往外地，起主要作用，是主犯；被告人全高德接受王亚伟请托后积极组织鄂东丰、蒙广辉、吕明等到边境接收涉案穿山甲鳞片，并负责交接涉案物品，其行为系走私行为得以实现的关键环节，亦起主要作用，是主犯，全高德的辩护人提出全高德系从犯的辩护意见不成立，不予采纳；被告人潘礼勇、鄂东丰、蒙广辉、吕明、何云强接受委托，协助

托运或运输涉案物品，起辅助作用，是从犯，但潘礼勇相对其他从犯作用较大。

4．关于辩护人提出被告人归案后如实供述犯罪事实，认罪态度好，有悔罪表现，经查证属实，予以采纳，依法可从轻处罚，但提出被告人家庭困难或家人需要照顾等原因建议对被告人从轻处罚，如属实，虽令人同情，但没有法律依据。辩护人的该辩护意见不成立，不予采纳。

5．关于指控被告人全高德犯罪数量的问题。经查，全高德参与走私的穿山甲鳞片的数量为：侦查机关在南宁百世快运中转仓库内查获的两批净重462.06千克，在其家中查获的300.2千克、在潘礼勇租用仓库里的一辆废弃小货车副驾驶室内查获何云强未发运的6.3千克及全高德违法向越南人购买存放于家中的5.98千克，共计774.54千克，公诉机关指控的数量准确，辩护人提出全高德存放于家中的鳞片是为了给其姐姐治病，不应计入走私数量的辩护意见没有法律依据，不予采纳。

6．关于被告人潘礼勇的行为定性。经查，潘礼勇明知他人实施走私行为仍提供运输等方便，根据刑法第一百五十六条规定，其行为系走私的共犯，辩护人提出潘礼勇的行为系掩饰隐瞒犯罪所得没有事实及法律依据，不予采纳。

本院认为，被告人王亚伟、全高德、潘礼勇、鄂东丰、蒙广辉、吕明、何云强违反海关法规，逃避海关监管，伙同他人走私国家禁止进出口的珍贵动物制品。其行为均已构成走私珍贵动物

制品罪，公诉机关指控的罪名成立。七被告人走私数额均属于“情节特别严重”，应当依法惩处。在共同犯罪中，被告人王亚伟、全高德是主犯，应按照其所参与的全部犯罪进行处罚。被告人潘礼勇、鄂东丰、蒙广辉、吕明、何云强起次要作用，是从犯，依法应当从轻、减轻处罚。王亚伟、全高德、潘礼勇、鄂东丰、蒙广辉、吕明归案后，如实供述犯罪事实，是坦白，依法可以从轻处罚；何云强自动投案，如实供述自己的罪行，是自首，依法可以从轻、减轻处罚；潘礼勇积极退出犯罪所得赃款，有悔罪表现，可酌情从轻处罚；七被告人主动认罪认罚，依法可从宽处罚，王亚伟、潘礼勇、蒙广辉、何云强认罪认罚早于全高德、鄂东丰、吕明，对其从宽处罚幅度应相对较大。综合全案事实，决定予以被告人王亚伟、全高德从轻处罚，予以被告人潘礼勇、鄂东丰、蒙广辉、吕明、何云强减轻处罚。

被告人王亚伟、全高德、潘礼勇、鄂东丰、蒙广辉、吕明、何云强的行为破坏生态环境，应当承担相应的民事赔偿责任。附带民事公益诉讼起诉人的诉讼请求，符合法律规定及合理部分予以支持。

根据各被告人犯罪的事实、情节和对社会的危害程度。依照《中华人民共和国刑法》第一百五十一条第二款、第一百五十六条、第二十五条第一款、第二十六条第一、四款、第二十七条、第六十七条第一、三款、第五十二条、第五十三条、第六十四条、第六十一条，《最高人民法院、最高人民检察院关于办理走私刑

事案件适用法律若干问题的解释》第九条第三款第（二）项，《中华人民共和国刑事诉讼法》第一百零一条第二款、第十五条、第二百零一条，《中华人民共和国野生动物保护法》第三条第一款、第六条第一款、第二十七条第一款、第三十五条第一、二、四款，《中华人民共和国民法典》第二百四十二条、第一千一百六十五条第一款、第一千一百六十八条、第一千一百六十七条，《最高人民法院关于审理环境民事公益诉讼案件适用法律若干问题的解释》第十八条，《最高人民法院最高人民检察院关于检察公益诉讼案件适用法律若干问题的解释》第二十条的规定，判决如下：

一、被告人王亚伟犯走私珍贵动物制品罪，判处有期徒刑十四年，并处没收个人财产人民币一百五十万元；

（刑期自 2020 年 3 月 26 日起至 2034 年 3 月 17 日止。判决执行以前指定监视居住二日折抵刑期一日，即指定监视居住自 2020 年 3 月 10 日至 2020 年 3 月 26 日，折抵刑期 8 天。）

二、被告人全高德犯走私珍贵动物制品罪，判处有期徒刑十三年，并处没收个人财产人民币一百二十万元；

（刑期自 2020 年 3 月 9 日起至 2033 年 3 月 8 日止。）

三、被告人潘礼勇犯走私珍贵动物制品罪，判处有期徒刑六年，并处罚金人民币十五万元；

（刑期自 2020 年 3 月 10 日起至 2026 年 3 月 9 日止。）

四、被告人鄂东丰犯走私珍贵动物制品罪，判处有期徒刑五年六个月，并处罚金人民币十万元；

（刑期自 2020 年 3 月 9 日起至 2025 年 9 月 8 日止。）

五、被告人吕明犯走私珍贵动物制品罪，判处有期徒刑五年三个月，并处罚金人民币八万元；

（刑期自 2020 年 3 月 9 日起至 2025 年 6 月 8 日止。）

六、被告人蒙广辉犯走私珍贵动物制品罪，判处有期徒刑五年两个月，并处罚金人民币四万元；

（刑期自 2020 年 3 月 10 日起至 2025 年 5 月 9 日止。）

七、被告人何云强犯走私珍贵动物制品罪，判处有期徒刑五年，并处罚金人民币三万元；

（刑期自 2021 年 5 月 14 日起至 2026 年 5 月 13 日止。）

八、附带民事公益诉讼被告王亚伟、全高德、潘礼勇、鄂东丰、吕明、何云强连带赔偿动物资源损失费人民币 41573097 元；

九、附带民事公益诉讼被告王亚伟、全高德、蒙广辉连带赔偿动物资源损失费人民币 26646695 元；

十、附带民事公益诉讼被告全高德、潘礼勇、鄂东丰还分别单独承担赔偿动物资源损失费人民币 187073 元、120288 元和 3600 元。

十一、附带民事公益诉讼王亚伟、全高德、潘礼勇、鄂东丰、蒙广辉、吕明、何云强共同在广西壮族自治区省级以上媒体向社会公众公开赔礼道歉；

十二、扣押在案的涉案动物制品穿山甲鳞片、棘海马等予以没收；扣押在案的被告人王亚伟手机二部，全高德手机一部，桂

Ｆ×××××五菱牌面包车，潘礼勇手机三部，鄂东丰手机二部、蒙广辉手机二部，吕明手机一部，桂Ｆ×××××五菱牌面包车及被冻结的户名为ＶＵＴＲＡＧＩＡＮＧ的农业银行账户（尾号4376）余额20139.44元及被告人潘礼勇退出的违法所得人民币4万元予以没收，上缴国库；

十三、继续追缴被告人全高德、鄂东丰、蒙广辉违法所得人民币4万元、1万元和2万元，上缴国库。

十四、驳回附带民事公益起诉人请求判令附带民事公益诉讼被告王亚伟、全高德、潘礼勇、鄂东丰、蒙广辉、吕明、何云强对其侵权行为在国家级以上媒体向社会公众赔礼道歉的诉讼请求。

上列被告人刑期从判决执行之日起计算。判决执行以前先行羁押的，羁押一日折抵刑期一日。罚金及动物资源损失费限于判决生效后三十日内缴纳，逾期则强制缴纳。

如不服本判决，可在接到判决书的第二日起十日内，通过本院或者直接向广西壮族自治区高级人民法院提出上诉，书面上诉的，应当提交上诉状正本一份，副本十份。

审判长　　农　伟

审判员　　黄秋艳

审判员　　韦连任

二〇二一年五月三十一日

法官助理　　卢素云

书 记 员　　农玉琪

附本案相关法条：

《中华人民共和国刑法》

第一百五十一条第二款走私国家禁止出口的文物、黄金、白银和其他贵重金属或者国家禁止进出口的珍贵动物及其制品的，处五年以上十年以下有期徒刑，并处罚金；情节特别严重的，处十年以上有期徒刑或者无期徒刑，并处没收财产；情节较轻的，处五年以下有期徒刑，并处罚金。

第一百五十六条与走私罪犯通谋，为其提供贷款、资金、帐号、发票、证明，或者为其提供运输、保管、邮寄或者其他方便的，以走私罪的共犯论处。第二十五条共同犯罪是指二人以上共同故意犯罪。二人以上共同过失犯罪，不以共同犯罪论处；应当负刑事责任的，按照他们所犯的罪分别处罚。

第二十六条组织、领导犯罪集团进行犯罪活动的或者在共同犯罪中起主要作用的，是主犯。

……

对于第三款规定以外的主犯，应当按照其所参与的或者组织、指挥的全部犯罪处罚。

第二十七条在共同犯罪中起次要或者辅助作用的，是从犯。对于从犯，应当从轻、减轻处罚或者免除处罚。

第六十七条第三款犯罪嫌疑人虽不具有前两款规定的自首情节，但是如实供述自己罪行的，可以从轻处罚；因其如实供述自己罪行，避免特别严重后果发生的，可以减轻处罚。

第五十二条判处罚金，应当根据犯罪情节决定罚金数

第五十三条罚金在判决指定的期限内一次或者分期缴纳。期满不缴纳的，强制缴纳。对于不能全部缴纳罚金的，人民法院在任何时候发现被执行人有可以执行的财产，应当随时追缴。如果由于遭遇不能抗拒的灾祸缴纳确实有困难的，可以酌情减少或者免除。

第六十四条犯罪分子违法所得的一切财物，应当予以追缴或者责令退赔；对被害人的合法财产，应当及时返还；违禁品和供犯罪所用的本人财物，应当予以没收。没收的财物和罚金，一律上缴国库，不得挪用和自行处理。

第六十一条对于犯罪分子决定刑罚的时候，应当根据犯罪的事实、犯罪的性质、情节和对于社会的危害程度，依照本法的有关规定判处。

《最高人民法院、最高人民检察院关于办理走私刑事案件适用法律若干问题的解释》

第九条第三款第（二）项具有下列情形之一的，应当认定为刑法第一百五十一条第二款规定的“情节特别严重”：

（二）走私珍贵动物制品数额在一百万元以上的；

《中华人民共和国刑事诉讼法》

第一百零一条第二款如果是国家财产、集体财产遭受损失的，人民检察院在提起公诉的时候，可以提起附带民事诉讼。

第十五条犯罪嫌疑人、被告人自愿如实供述自己的罪行，承认指控的犯罪事实，愿意接受处罚的，可以依法从宽处理。

第二百零一条对于认罪认罚案件，人民法院依法作出判决时，一般应当采纳人民检察院指控的罪名和量刑建议，但有下列情形的除外：

（一）被告人的行为不构成犯罪或者不应当追究其刑事责任的；

（二）被告人违背意愿认罪认罚的；

（三）被告人否认指控的犯罪事实的；

（四）起诉指控的罪名与审理认定的罪名不一致的；

（五）其他可能影响公正审判的情形。

人民法院经审理认为量刑建议明显不当，或者被告人、辩护人对量刑建议提出异议的，人民检察院可以调整量刑建议。人民检察院不调整量刑建议或者调整量刑建议后仍然明显不当的，人民法院应当依法作出判决。

《中华人民共和国野生动物保护法》

第三条第一款野生动物资源属于国家所有。

第六条第一款任何组织和个人都有保护野生动物及其栖息地的义务。禁止违法猎捕野生动物、破坏野生动物栖息地。

第二十七条禁止出售、购买、利用国家重点保护野生动物及其制品。

第三十五条中华人民共和国缔结或者参加的国际公约禁止或者限制贸易的野生动物或者其制品名录，由国家濒危物种进出口管理机构制定、调整并公布。

进出口列入前款名录的野生动物或者其制品的，出口国家重点保护野生动物或者其制品的，应当经国务院野生动物保护主管部门或者国务院批准，并取得国家濒危物种进出口管理机构核发的允许进出口证明书。海关依法实施进出境检疫，凭允许进出口证明书、检疫证明按照规定办理通关手续。

……

列入本条第一款名录的野生动物，经国务院野生动物保护主管部门核准，在本法适用范围内可以按照国家重点保护的野生动物管理。

《中华人民共和国民法典》

第二百四十二条法律规定专属于国家所有的不动产和动产，任何组织或者个人不能取得所有权。

第一千一百六十五条行为人因过错侵害他人民事权益造成损害的，应当承担侵权责任。

第一千一百六十八条二人以上共同实施侵权行为，造成他人损害的，应当承担连带责任。

第一千一百六十七条侵权行为危及他人人身、财产安全的，被侵权人有权请求侵权人承担停止侵害、排除妨碍、消除危险等侵权责任。

《最高人民法院关于审理环境民事公益诉讼案件适用法律若干问题的解释》

第十八条对污染环境、破坏生态，已经损害社会公共利益或者具有损害社会公共利益重大风险的行为，原告可以请求被告承担停止侵害、排除妨碍、消除危险、修复生态环境、赔偿损失、赔礼道歉等民事责任。

《最高人民法院、最高人民检察院关于检察公益诉讼案件适用法律若干问题的解释》

第二十条人民检察院对破坏生态环境和资源保护，食品药品安全领域侵害众多消费者合法权益，侵害英雄烈士等的姓名、肖像、名誉、荣誉等损害社会公共利益的犯罪行为提起刑事公诉时，可以向人民法院一并提起附带民事公益诉讼，由人民法院同一审判组织审理。

人民检察院提起的刑事附带民事公益诉讼案件由审理刑事案件的人民法院管辖。
