# Supplementary material for: China’s Legal Protection System for Pangolins: Past, Present, and Future
Source: Animals (Basel). 2025 Aug 18;15(16):2422. doi: 10.3390/ani15162422 (PMC12383201; doi:10.3390/ani15162422)
Supplement: Supplementary file 1 [file animals-15-02422-s001.zip › Supplementary Material S4-Full Text of Judgments in Pangolin-Related Public Interest Litigation Cases in China/【17】青岛市人民检察院诉某艺术中心生态破坏民事公益诉讼案.pdf]

2023-11-2-466-021

青岛市人民检察院诉某艺术中心生态破坏民事公益诉讼案

——生态环境侵权领域惩罚性赔偿及惩罚性赔偿中的劳务代偿适用

关键词 民事 生态破坏民事公益诉讼 野生动物 生态环境服务功能损失 惩罚性赔偿 劳务代偿

基本案情

公益诉讼起诉人山东省青岛市人民检察院起诉称：被告某艺术中心非法收购大王蛇3条、穿山甲1只、熊掌4只，将部分野生动物做成菜品销售，请求法院判令某艺术中心赔偿野生动物价值损失8.3万元和生态环境功能损失，承担惩罚性赔偿并公开赔礼道歉。

被告某艺术中心辩称：最高人民检察院相关文件要求切实保护民营企业合法权益，且被告营业执照经营者未参与实际经营，对被告承担的民事责任应从轻考量。

法院经审理查明：某艺术中心系经营餐饮服务的个体工商户，其在未依法取得收购、出售野生动物行政许可的情况下，先后购入大王蛇3条、穿山甲1只、熊掌4只，将部分野生动物做成菜品销售。案发后，公安机关从某艺术中心查获大王蛇1条、熊掌1只。经国家林业局森林公安司法鉴定中心鉴定，大王蛇为孟加拉眼镜蛇，被列入《国家保护的有益的或者有重要经济、科学研究价值的陆生野生动物名录》；熊掌为棕熊熊掌，棕熊被列入《国家重点保护野生动物名录》二级；穿山甲于2020年6月被确定为国家一级保护野生动物。按照《野生动物及其产品（制品）价格认定规则》《野生动物及其制品价值评估方法》《陆生野生动物基准价值标准目录》的规定，某艺术中心破坏生态行为造成的野生动物损失为83000元。专家意见认定某艺术中心非法收购、出售涉案珍贵、濒

危野生动物的行为造成生态环境服务功能损失907500元。

案件审理过程中，经公益诉讼起诉人提出劳务代偿方案建议，被告某艺术中心签署了劳务代偿同意书,同意本案部分惩罚性赔偿以劳务代偿方式履行。

山东省青岛市中级人民法院于2021年1月29日作出（2021）鲁02民初69号民事判决：（一）被告某艺术中心于判决生效之日起十五日内赔偿破坏生态行为造成的野生动物损失83000元、生态环境服务功能损失907500元；（二）被告某艺术中心承担惩罚性赔偿99050元。其中74126元于判决生效之日起十五日内缴纳；24924元以被告指定二人每人提供六十日生态环境公益劳动的方式承担，由法院指定协执单位青岛市崂山区司法局管理和指导，最迟于2022年1月28日前完成。如某艺术中心未提供生态环境公益劳动或提供的生态环境公益劳动未能经法院审核通过，则应在法院审核之日起十五日内承担惩罚性赔偿24924元；（三）被告某艺术中心于判决生效之日起十五日内在全国性媒体上公开赔礼道歉（媒体和内容由法院审定，若被告不履行，法院将公开刊登判决，费用由被告负担）；（四）专家意见费15150元，由被告某艺术中心负担。宣判后，某艺术中心未提起上诉。判决已经发生效力。

裁判理由

法院生效裁判认为：人类、野生动物及其他物种相互依存，共同维护着自然界的稳定、和谐和发展，保护野生动物、维护生物多样性就是保护人类自己。穿山甲、棕熊及孟加拉眼镜蛇均为珍贵、濒危野生动物，除其自身价值外，对保护生物多样性、维护生态系统平衡具有重要作用。被告某艺术中心虽然不是穿山甲、棕熊、孟加拉眼镜蛇的直接的猎杀者，但其实施收购、出售的行为为猎杀珍贵、濒危野生动物提供了动机和市场，其违法行为对于生态环境损害具有直接的因果关系，依照《

人民法院案例库

最高人民法院关于审理环境民事公益诉讼案件适用法律若干问题的解释》第十八条“对污染环境、破坏生态，已经损害社会公共利益或者具有损害社会公共利益重大风险的行为，原告可以请求被告承担停止侵害、排除妨碍、消除危险、修复生态环境、赔偿损失、赔礼道歉等民事责任”的规定，应承担相应民事责任。被告某艺术中心收购、出售珍贵、濒危野生动物的主观故意明显，其行为导致了珍贵、濒危野生动物的减少，加深了濒危程度，破坏了生态资源和环境平衡，造成了严重后果，且对生态环境的损害后果在未修复前具有持续性的特点，依照《中华人民共和国民法典》第一千二百三十二条规定“侵权人违反法律规定故意污染环境、破坏生态造成严重后果的，被侵权人有权请求相应的惩罚性赔偿”，可判令其承担惩罚性赔偿。综合其主观故意、危害后果以及在本案中悔改态度较好，愿意提供生态环境公益劳动，以自己实际行动保护生态环境等情节，酌情判令其承担惩罚性赔偿99050元。惩罚性赔偿具有惩罚和遏制不法行为的多重功能，对预防同类型损害发生具有十分重要意义。在与被告协商一致的情况下，由被告以提供环境公益劳动的方式承担全部或者部分惩罚性赔偿，在法院主持下，被告某艺术中心与公益诉讼起诉人协商一致，愿意以提供环境公益劳务方式承担全部或者部分惩罚性赔偿。环境资源公益劳动的工作量应相当于其折抵的惩罚性赔偿，可以参照上一年度青岛市职工年平均工资作为折算标准，即（职工年平均工资75806元÷365天）×劳务代偿拟定时限60天×2人=24924元。劳务代偿工作由法院确定的协执单位管理和指导，公益诉讼起诉人可以对劳务代偿进行监督，如被告未能提供环境资源公益劳动，或者提供的环境资源公益劳动未能经法院审核通过，则仍应承担判决确定的相应惩罚性赔偿责任。综上，公益诉讼起诉人的诉讼请求有事实和法律依据，提供的劳务代偿方案合理，予以支持。

人民法院案例库

裁判要旨

1.侵权人非法收购、出售珍贵、濒危野生动物，造成野生动物损失、生态环境服务功能损失等严重后果的，法院可依据公益诉讼起诉人申请，判令侵权人承担相应的惩罚性赔偿。

2.人民法院判令侵权人承担惩罚性赔偿的案件，可以判决侵权人以提供有益于生态环境公共利益保护的劳动方式承担部分惩罚性赔偿责任。

关联索引

《中华人民共和国民法典》第1232条

《最高人民法院关于审理环境民事公益诉讼案件适用法律若干问题的解释》第20条

《最高人民法院关于审理环境公益诉讼案件的工作规范（试行）》第33条

一审：山东省青岛市中级人民法院（2021）鲁02民初69号民事判决（2021年1月29日）

人民法院案例库
