# Supplementary material for: China’s Legal Protection System for Pangolins: Past, Present, and Future
Source: Animals (Basel). 2025 Aug 18;15(16):2422. doi: 10.3390/ani15162422 (PMC12383201; doi:10.3390/ani15162422)
Supplement: Supplementary file 1 [file animals-15-02422-s001.zip › Supplementary Material S4-Full Text of Judgments in Pangolin-Related Public Interest Litigation Cases in China/【18】朱卓敏侵权责任纠纷一审民事判决书.pdf]

朱卓敏侵权责任纠纷一审民事判决书

浙江省金华市中级人民法院

民 事 判 决 书

（2020）浙 07 民初 299 号

公益诉讼起诉人：义乌市人民检察院。

委托诉讼代理人：何晓玉，该院检察员。

被告：朱卓敏，男，1971 年 10 月 9 日出生，汉族，住浙江省金华市义乌市，现羁押于乔司监狱。

公益诉讼起诉人义乌市人民检察院与被告朱卓敏侵权责任公益诉讼一案，本院于 2020 年 9 月 15 日立案受理后，依法组成合议庭，于 2020 年 11 月 19 日公开开庭进行了审理。义乌市人民检察院指派检察员何晓玉出庭履行职务，被告朱卓敏参加诉讼。本案现已审理终结。

义乌市人民检察院向本院提出诉讼请求：1、判令被告朱卓敏赔偿野生动物资源损失人民币 359000 元；2、判令被告朱卓敏在金华市级媒体上公开赔礼道歉。事实和理由:该院在履行法律监督职责中发现，朱卓敏非法收购、出售珍贵、濒危野生动物及其制品，破坏生物多样性，损害社会公共利益。金华市人民检察院于 2020 年 3 月 26 日立案，经浙江省人民检察院批准，于 2020 年 4 月 23 日指定该院管辖，该院于 2020 年 6 月 4 日履行公告程序。经依法审查查明:2018 年 12 月至 2019 年 5 月，朱卓敏为牟取非法利益，从上家吴某处分多次收购 8 只穿山甲和 3 只熊掌，

并加价卖给下家。另朱卓敏为自己食用从安徽一上家处收购冰冻猫头鹰1只。根据《野生动物及其制品价值评估方法》相关规定，穿山甲价值人民币320000元，褐林鸮价值人民币15000元。经鉴定，熊掌价值人民币24000元。该院认为，朱卓敏非法收购、出售珍贵、濒危野生动物及其制品，其行为对野生动物资源和生态环境造成破坏，损害了社会公共利益，其行为违反了《中华人民共和国野生动物保护法》第三条第一款、第六条第一款第十条第二十七条、第三十条第二款的规定。根据《中华人民共和国侵权责任法》第四条第一款、第十五条的规定，应承担相应的侵权责任。检察机关发现被告违法行为后于2020年6月4日在正义网进行公告，公告期满后相关组织没有提起民事公益诉讼，社会公共利益仍处于受损害状态。根据《中华人民共和国民事诉讼法》第五十五条第二款、《最高人民法院、最高人民检察院关于检察公益诉讼案件适用法律若干问题的解释》第十三条第二款的规定，提起本案诉讼。

义乌市人民检察院为支持其诉讼请求，提交了如下证据：

1、当事人朱卓敏的陈述;

2、正义网公告、实物照片、微信截图、转账记录、搜查笔录、扣押物品清单、身份辨认笔录、现场辨认笔录、全国常住人信息;

3、证人吴某、王某的证言;

4、国家林业局森林公安司法鉴定中心物证鉴定书、价格认定结论书;

5、现场检查笔录及照片。

共同证明起诉书列明的事实，被告朱卓敏确实有加价出售穿山甲、熊掌，为了自己食用购买褐林鸮的事实，义乌市人检察院提起公益诉讼有法律依据。

被告朱卓敏辩称，其已经被判刑，家里经济条件也不好。上家已经转了好几手，其获利并不多。其出售的动物及制品不是中国的，而是来自越南。其上家也没有被提起民事公益诉讼。

被告朱卓敏未提交证据。

经庭审质证，被告对证据证明的事实没有异议，但认为穿山甲到其手中时已经经过好几手，且不是中国的而是来自越南，被告已经被判刑并被判处罚金，不能仅对被告提起公益诉讼。另外，穿山甲也不是被告抓来的，要求其进行赔偿没有依据。

本院经审查认为，公益诉讼起诉人提交的证据真实、合法，且能够达到公益诉讼起诉人的证明目的，本院予以采纳。

本院经审理查明：2017 年，被告朱卓敏向他人收购冰冻猫头鹰 1 只。

2018 年 12 月至 2019 年 5 月期间，被告朱卓敏共向吴某收购穿山甲 8 只及熊掌 3 只，并将其中 2 只穿山甲予以出售。

根据国家林业局《野生动物及其制品价值评估办法》，野生动物整体的价值，按照《陆生野生动物基准价值标准目录》所列

该种野生动物的基准价值乘以相应的倍数核算，其中国家二级保护野生动物，按照所列野生动物基准价值的五倍核算。其中穿山甲基准价值 8000 元、鸮形目基准价值 3000 元。根据江西百信司法鉴定中心出具的司法鉴定书，案涉疑似活体、冰冻穿山甲属于哺乳纲鳞甲目鲮鲤科，疑似冰冻熊掌属于哺乳纲食肉目熊科，均属于国家二级濒危野生保护动物。根据国家林业局森林公安司法鉴定中心出具的物证鉴定书，案涉被查获的冰冻猫头鹰系褐林鸮，褐林鸮被列入《国家重点保护野生动物名录》二级。即穿山甲的整体价值为 8000*5=40000 元，猫头鹰的整体价值为 3000*5=15000 元。另，根据义乌市涉案物品价格认证中心出具的《价格认定结论书》，案涉熊掌的价格为 8000 元/只。

综上，被告收购、出售的珍贵、濒危野生动物价值共计 359000 元。

另查明，检察机关已按《最高人民法院、最高人民检察院关于检察公益诉讼案件适用法律若干问题的解释》第十三条之规定依法进行公告，公告期间为三十日。公告期满,法律规定的机关和有关组织未提起诉讼。

又经核查，朱卓敏因犯非法收购、出售珍贵、濒危野生动物、珍贵、濒危野生动物制品罪，于 2020 年 7 月 9 日被浙江省义乌市人民法院（2020）浙 0782 刑初 561 号刑事判决判处有期徒刑五年六个月，并处罚金人民币二万元。

本院认为，人与自然和谐相处是人类社会追求的重要目标，依法保护野生动物对于保障生物多样性，保护生态系统完整性具有重要意义。根据《中华人民共和国野生动物保护法》第三条之规定，野生动物资源属于国家所有。本案中，被告朱卓敏收购、出售的穿山甲、猫头鹰、熊掌均属国家二级保护动物或其制品，其进行非法收购、出售，破坏了野生动物资源，侵害了公共利益，已经构成侵权，依法应按《中华人民共和国侵权责任法》第十五条之规定承担赔偿损失、赔礼道歉的侵权责任。在法定公告期满，法律规定的机关和有关组织未提起诉讼的情况下，人民检察院依法可以提起本案公益诉讼。被告提出案涉穿山甲的来源是国外而非国内，其不应承担赔偿责任的理由，缺乏事实和法律依据，本院不予采纳。关于被告提出其已经被追究刑事责任，无需再承担民事赔偿责任的意见，《中华人民共和国侵权责任法》第四条规定，侵权人因同一行为应当承担行政责任或者刑事责任的，不影响依法承担侵权责任。关于被告提出不应仅对其一个人主张赔偿责任的意见，《中华人民共和国侵权责任法》第十三条规定，法律规定承担连带责任的，被侵权人有权请求部分或者全部连带责任人承担责任，故检察机关先行向部分侵权人主张权利，并无不当。综上，公益诉讼起诉人义乌市人民检察院提出的诉请于法有据，本院予以支持。依据《中华人民共和国侵权责任法》第六条第一款、第十五条、《中华人民共和国野生动物保护法》第三条

第一款、《最高人民法院、最高人民检察院关于检察公益诉讼案件适用法律若干问题的解释》第十三条之规定，判决如下：

一、被告朱卓敏于本判决生效之日起十五日内赔偿国家野生动物资源损失359000元；

二、被告朱卓敏于本判决生效之日起十五日内在金华市级媒体上公开赔礼道歉（赔礼道歉内容应先报本院审查）。如被告朱卓敏拒不履行，本院将在金华市级媒体上公布判决的主要内容，相应费用由被告朱卓敏负担。

如未按本判决指定的期间履行给付金钱义务，应当按照《中华人民共和国民事诉讼法》第二百五十三条之规定，加倍支付迟延履行期间的债务利息。

案件受理费6685元，由被告朱卓敏负担。

如不服本判决，可在判决书送达之日起十五日内，向本院递交上诉状，并按对方当事人的人数提出副本，上诉于浙江省高级人民法院。

审 判 长　单晓剑

审 判 员　徐青雅

审 判 员　虞 行

人民陪审员　杨 翠

人民陪审员　方 群

人民陪审员　许华山

人民陪审员　徐 景

二〇二〇年十一月二十六日

代书　记员　　余美子
