# Supplementary material for: China’s Legal Protection System for Pangolins: Past, Present, and Future
Source: Animals (Basel). 2025 Aug 18;15(16):2422. doi: 10.3390/ani15162422 (PMC12383201; doi:10.3390/ani15162422)
Supplement: Supplementary file 1 [file animals-15-02422-s001.zip › Supplementary Material S4-Full Text of Judgments in Pangolin-Related Public Interest Litigation Cases in China/【19】邵南、段美英一审刑事判决书.pdf]

邵南、段美英一审刑事判决书

中华人民共和国
云南省保山市中级人民法院
刑 事 附 带 民 事 判 决 书

（2019）云05刑初219号

公诉机关暨公益诉讼起诉人中华人民共和国云南省保山市人民检察院。

被告人邵南（自报身份），女，1985年1月16日出生，缅甸身份证编号1/KPN(N)004175，景颇族，缅甸学籍三年级，农民，原籍缅甸，现住腾冲市。因本案于2019年6月19日被刑事拘留，7月24日被逮捕，现羁押于云南省腾冲市看守所。

指定辩护人古嘉帆，云南正旭律师事务所律师。

缅语翻译杨加凤，云南省腾冲市人民法院工作人员。

被告人段美英，女，1963年8月21日出生于云南省腾冲市，汉族，小学文化，农民，住腾冲市。因本案于2019年6月19日被刑事拘留，7月25日被逮捕，现羁押于云南省腾冲市看守所。

辩护人王维正、闫潮芳，云南正旭律师事务所律师。

被告人李长雄，男，1966年9月2日出生于云南省腾冲市，汉族，小学文化，农民,住腾冲市。因本案于2019年6月5日被刑事拘留，6月19日被逮捕，现羁押于云南省腾冲市看守所。

辩护人杨荣钦，云南正旭律师事务所律师。

被告人宋德万，男，1965年1月9日出生于云南省腾冲市，汉族，小学文化，农民,住腾冲市。因本案于2019年7月15日被腾冲市森林公安局取保候审，9月19日被保山市人民检察院取保候审，同年11月7日被本院取保候审。现在家。

辩护人宋怀周，云南正旭律师事务所律师。

被告人密加旺，男，1974年9月14日出生于云南省腾冲市，汉族，初中文化，农民,住腾冲市。因本案于2019年7月15日被腾冲市森林公安局取保候审，9月19日被保山市人民检察院取保候审，同年11月7日被本院取保候审。现在家。

指定辩护人金芋廷，云南腾大律师事务所律师。

云南省保山市人民检察院于2019年11月1日以保检四部刑诉[2019]3号起诉书向本院提起公诉，于2019年11月29日以保检民公（2019）53050000005号、（2019）53050000006号、53050000007号、（2019）53050000008号刑事附带民事公益诉讼起诉书分别对被告邵南、段美英、李长雄、宋德万、密加旺提起刑事附带民事公益诉讼。本院审查后，认为应合并审理，依法组成合议庭，于2019年12月17日在云南省腾冲市人民法院公开开庭审理了本案。云南省保山市人民检察院指派检察员李茜、代理检察员李垚出庭支持公诉，指派检察员王银珍、书记员余亚斌出庭支持公益诉讼。被告人邵南及其辩护人古嘉帆、被告人段美英及其辩护人王维正、闫潮芳、被告人李长雄及其辩护人杨荣

钦、被告人宋德万及其辩护人宋怀周、被告人密加旺及其辩护人金芋廷、翻译人员杨某2均到庭参加诉讼。现已审理终结。

公诉机关指控，2019年2月至6月期间，被告人邵南多次从缅甸收购白眉长臂猿头颅、活体穿山甲及穿山甲甲片后绕关避卡走私入境，为牟利先后出售给被告人段美英白眉长臂猿头颅四个、穿山甲甲片2千克；出售给被告人李长雄活体穿山甲2只，出售给被告人宋德旺、密加旺活体穿山甲1只；出售给陈某（另案）白眉长臂猿头颅1个，穿山甲甲片12.36千克。经鉴定，上述由邵南走私入境的珍贵动物及珍贵动物制品经济价值共计3103680元，其中：

2019年5月至6月期间，被告人段美英明知邵南出售的白眉长臂猿头颅、穿山甲甲片系从缅甸走私入境，先后两次分别于腾冲市滇滩镇西营村路边、固东镇“四川林茂饭店”内，以共计7600元的价格向邵南购买白眉长臂猿头颅4个、穿山甲甲片共2千克，经鉴定经济价值为1736960元。

2019年6月5日凌晨2时许，被告人李长雄明知邵南出售的活体穿山甲系走私入境，于腾冲市滇滩镇吉盛招待所9号房间内向邵南购买活体穿山甲，后驾驶其车牌云M×××××白色五菱宏光面包车运输，至被腾冲市森林公安局抓获，当场从李长雄驾驶车辆第二排和第三排座椅中间查获活体穿山甲2只，经鉴定经济价值为80000元。

2019 年 6 月 18 日 10 时许，被告人宋德万、密加旺合伙向邵南购买活体穿山甲，三人于腾冲市环岛路边正在交易时，被腾冲市森林公安局民警抓获，当场查获活体穿山甲 1 只，经鉴定经济价值为 40000 元。

公诉机关认定上述事实的证据有物证，书证，证人证言，鉴定意见，勘验检查、辨认笔录，被告人的供述与辩解，视听资料等。公诉机关认为被告人邵南多次从境外走私白眉长臂猿头颅、活体穿山甲、穿山甲甲片入境的行为，已触犯《中华人民共和国刑法》第一百五十一条第二款之规定，应当以走私珍贵动物、珍贵动物制品罪追究其刑事责任。被告人段美英、李长雄明知邵南出售的白眉长臂猿头颅、活体穿山甲、穿山甲甲片系从缅甸走私入境，仍向其收购的行为，已触犯《中华人民共和国刑法》第一百五十一条第二款、第一百五十五条之规定，应当分别以走私珍贵动物制品罪、走私珍贵动物罪追究二被告人刑事责任。被告人宋德万、密加旺向邵南收购活体穿山甲的行为，已触犯《中华人民共和国刑法》第三百四十一条第一款之规定，应当以非法收购珍贵、濒危野生动物罪追究二被告人刑事责任。被告人宋德万、密加旺系共同犯罪，同时适用《中华人民共和国刑法》第二十五条之规定。建议对被告人李长雄、宋德万、密加旺从轻判处，并适用缓刑。

公益诉讼起诉人基于上述犯罪事实，认为国家重点保护野生动物及其生存环境，禁止任何单位和个人非法捕猎、杀害或者非

法收购、运输、出售。本案涉及的白眉长臂猿、穿山甲被列入《濒危野生动植物种国际贸易公约》附录Ⅰ，列入附录Ⅰ的物种是指所有受到和可能受到贸易影响而有灭绝危险的物种。我国是《濒危野生动植物种国际贸易公约》缔约国，被列入名录的野生动物，入境我国后按照国家重点保护的野生动物管理。野生动物是全人类的宝贵资源和共同财富，是地球自然系统中不可替代的重要组成部分。野生动物一旦减少，生物多样性就要遭受破坏，人类的生存环境也要受到影响。综上，被告邵南、段美英、李长雄、宋德万、密加旺的行为违反了国家有关野生动物保护法律法规的规定，严重破坏了生物多样性和自然生态平衡，造成野生动物资源受损，社会公共利益受到侵害。根据《中华人民共和国野生动物保护法》第二十七条第一款、第三十五条第一、二、四款、《中华人民共和国侵权责任法》第八条、第十四条和最高人民法院《关于审理环境民事公益诉讼案件适用法律若干问题的解释》第十八条的规定，应当承担赔礼道歉、赔偿损失的侵权民事责任。并分别提出了如下诉讼请求：

一、请求判令被告邵南、段美英、李长雄、宋德万、密加旺对其侵权行为在市级以上媒体公开赔礼道歉；

二、请求判令被告邵南与陈某共同承担因侵权行为造成的野生动物资源损失费人民币 1246720 元。

三、请求判令被告邵南与段美英共同承担因侵权行为造成的野生动物资源损失费人民币 1736960 元。

四、请求判令被告邵南与李长雄共同承担因侵权行为造成的野生动物资源损失费人民币 80000 元。

五、请求判令被告邵南与宋德万、密加旺共同承担因侵权行为造成的野生动物资源损失费人民币 40000 元。

被告人邵南对起诉书指控事实无异议，辩解鉴定价值过高。请求从宽判处。对公益诉讼表态认错，但对赔偿表示无能力支付。辩护人意见，对指控罪名不持异议，认为涉案的鉴定价值过高，超出市场价值过大。被告人邵南系缅甸籍人，对中国法律不熟知，主观犯意较小，归案后如实供述，系坦白，家庭贫困，请求从宽判处。对公益诉讼答辩称赔偿主体应是缅甸国，对诉请的赔偿金额不认可。

被告人段美英对起诉书指控事实无异议，辩解购买涉案珍贵动物制品主要是药用，没有破坏生态的犯意。已意识到自己的错误，请求从宽判处。对公益诉讼表示认错，但对赔偿部分表示无能力支付。辩护人意见，对指控罪名不持异议，认为：①涉案的鉴定价值过高，以国家林业局 2017 第 46 号令作为鉴定依据不合法，根据从旧兼从轻原则及法律的效力属性，本案段美英涉案物品应根据林某 2 通字[1996]8 号文进行价值评判，段美英的涉案价值只应计算为 325718.08 元。②辩护人曾向法庭提交申请，要求鉴定人出庭作证，但鉴定人并未出庭，该鉴定意见不能作为定案的依据。③被告人段美英涉案的物品已被公安机关扣缴，段美英的行为没有给国家造成实质性损害。④段美英系初犯、偶犯，

认罪态度较好，请求给予减轻处罚。对公益诉讼答辩称赔偿主体不适格，段美英收购制品的行为并未直接对生态环境造成损害，不排除该珍贵动物系自然死亡的情形，不能认定段美英构成侵权造成了生态破坏，且未造成实际损失，请求判决驳回附带民事公益诉讼人的诉讼请求。

被告人李长雄对起诉书指控事实不持异议。对公益诉讼表态承认错误，但对赔偿部分表示无能力承担。辩护人意见，对起诉书指控罪名无异议，认为被告人李长雄走私珍贵动物穿山甲仅2只，且被抓获时为活体，并未造成死亡后果，犯罪情节非常轻微，且犯罪也并非为了牟利，归案后认罪、悔罪。综上，对照量刑标准，应在二年以下判处有期徒刑，并适用缓刑。对公益诉讼答辩称，涉案穿山甲是在养护过程中死亡，被告李长雄愿意承担养护期间的费用，请求法庭予以单独计算赔偿金额。

被告人宋德万对起诉书指控事实不持异议。对公益诉讼表示认错，但对民事赔偿表示无力承担。辩护人意见，对指控罪名不持异议，被告人认罪认罚，系初犯、有坦白情节，请求判处一年以下有期徒刑并适用缓刑。对公益诉讼答辩称被告人宋德万购买的是穿山甲活体，造成死亡的损失不应由被告人宋德万承担，请求判决驳回附带民事公益诉讼起诉人的诉讼请求。

被告人密加旺对起诉书指控事实不持异议。对公益诉讼的诉讼请求表示无意见。辩护人意见，对起诉书指控罪名不持异议，

认为密加旺系初犯、从犯，且认罪认罚，请求法庭从轻判处。对公益诉讼尊重被告密加旺的意见。

经审理查明，2019年2月至6月期间，被告人邵南为牟取利益，多次从缅甸收购白眉长臂猿头颅、活体穿山甲及穿山甲甲片后绕关避卡走私入境，先后出售给被告人段美英白眉长臂猿头颅四个鉴定价值1600000元、穿山甲甲片2千克鉴定价值136960元；出售给被告人李长雄活体穿山甲2只鉴定价值80000元；出售给被告人宋德旺、密加旺活体穿山甲1只鉴定价值40000元；出售给陈某（另案）白眉长臂猿头颅1个鉴定价值400000元、穿山甲甲片12.36千克鉴定价值846720元。综上，上述由邵南走私入境的珍贵动物及珍贵动物制品经济价值共计3103680元，其中：

2019年5月至6月期间，被告人段美英明知邵南出售的白眉长臂猿头颅、穿山甲甲片系从缅甸走私入境，先后两次分别于腾冲市滇滩镇西营村路边、固东镇“四川林茂饭店”内，以共计7600元的价格向邵南购买白眉长臂猿头颅4个、穿山甲甲片共2千克，鉴定价值共计为1736960元。

2019年6月5日凌晨2时许，被告人李长雄明知邵南出售的活体穿山甲系走私入境，于腾冲市滇滩镇吉盛招待所9号房间内向邵南购买活体穿山甲，后驾驶其车牌云M×××××白色五菱宏光面包车运输至腾冲市时，被腾冲市森林公安局抓获，当场

从其驾驶车辆第二排和第三排座椅中间查获活体穿山甲 2 只，鉴定价值共计为 80000 元。

2019 年 6 月 18 日 10 时许，被告人宋德万、密加旺合伙向邵南购买活体穿山甲，三人于腾冲市环岛路边正在交易时，被腾冲市森林公安局民警抓获，当场查获活体穿山甲 1 只，鉴定价值为 40000 元。

另查明，涉案被查获的 3 只活体穿山甲在养护期间已经死亡。

上述犯罪事实，有开庭审理时经举证、质证的下列证据予以证实：

1.被告人的身份信息材料、邵南持有的中缅边界通行证复印件、李长雄前科判决。证实各被告人均具备刑事责任能力，基本情况与起诉书指控一致。其中被告人邵南持有的身份证虽然显示系缅甸联邦共和国国民，但经云南省公安厅出入境管理局照会缅甸驻昆明领事馆，未收到回复，仍以自报身份论。同时证实邵南持有中缅边境通行证出入境情况。被告人李长雄曾因犯非法收购珍贵动物制品罪于 2009 年 12 月 23 日被云南省腾冲市人民法院判处有期徒刑六个月，缓刑一年，具有犯罪前科。

2.抓获经过。腾冲市森林公安分局侦查员证实：在打击破坏野生动物资源违法犯罪专项行动中，根据情报线索，于 2019 年 6 月 5 日凌晨 3 时 10 分许，在腾冲市响水沟路段抓获了购买穿山甲后正在运输途中的被告人李长雄；于 2019 年 6 月 18 日 10

时 20 分许，在腾冲市子环岛旁现场抓获了正在进行穿山甲交易的邵南、宋德万、密加旺；于 2019 年 6 月 18 日 18 时 06 分许，在腾冲市腾越镇玉宸翠景小区门口查获携带穿山甲甲片的段美英，根据其供述，又到其小区住宅内查获 3 个疑似白眉长臂猿头颅。

3.现场勘验、搜查笔录、扣押清单及照片。

（1）证实抓获被告人邵南后，公安人员于 2019 年 6 月 19 日对邵南位于明光镇自治村喂猪坝住宅进行了搜查，从其家中正房右侧邵南卧室内外侧板壁旁的一个红色冰箱内查获疑似熊油制品 1 袋，在冰箱旁一个绿色铁皮箱子内查获矿泉水瓶装的疑似熊油 30 瓶，在绿色铁皮箱旁简易衣柜顶查获被烤干的疑似野生动物胆 1 个。在邵南的一辆银色缅牌三菱越野车内查获疑似野牛胆制品 2 个。腾冲市森林公安局民警对查获的上述物品进行了勘验检查、编号、称量、扣押。对邵南上交其贩卖野生动物及制品的部分赃款共计 17719.5 元进行扣押。

（2）证实抓获被告人段美英后，公安人员于 2019 年 6 月 19 日对从段美英处查获的疑似野生动物制品进行勘验并编号 1-8 号，其中 1-4 号为动物甲片制品 4 袋，甲片颜色黑褐色，形如瓦状，呈菱形、盾状、折合状，甲片有纵纹，边缘光滑，甲片之间有硬毛，特征与穿山甲甲片特征相符，为疑似穿山甲甲片。1 号为黑色塑料袋包装 1 袋重 0.5kg，2 号为黑色塑料袋包装 1 袋重 0.5kg，3 号为黑色塑料袋包装 1 袋重 0.5kg，4 号为黑色塑

料袋包装 1 袋重 0.5kg。5-8 号为疑似猴子头颅 4 个，4 个头颅小，面部短而扁，除面部留有体毛外，其余部分体毛被剥离，裸露的头骨上留有烟熏、火烧痕迹，头颅面部特征均表现为额部附近有一道明显白纹，如同白色眉毛，具白色眼眉，此形态特征与白眉长臂猿形态特征相似，为疑似白眉长臂猿头颅。其中 5 号头颅长 15cm，宽 8cm，重 0.12kg；6 号头颅长 14cm，宽 8cm，重 0.12kg；7 号头颅长 14cm，宽 8cm，重 0.12kg；8 号头颅长 12cm，宽 7cm，重 0.10kg。民警对上述 1 至 8 号疑似野生动物制品进行了提取及扣押。

（3）证实抓获被告人宋德万、密加旺时，对现场进行了勘验：白色塑料编织袋上写有“紫霞-白砂糖-密朋东亚糖业-广西海棠东亚糖业有限公司”字样，编织袋内装有一只彩色尼龙袋，彩色尼龙袋有强烈酸臭气味，内装有一只活体动物，该动物特征表现为头小呈圆锥状，吻长无齿，眼小而圆，四肢粗短，五趾具强爪，全身有鳞甲，为疑似穿山甲活体，长 88cm，重 4.1kg。型号 SPORT 黑色旧双肩背包内装有一把杆秤，该杆秤无型号，最大称重量 15kg，长 40cm。民警对上述物品进行了提取及扣押。

（4）证实现场查获李长雄时，李长雄用于运输疑似穿山甲活体的车辆为一辆白色五菱牌微型车，型号为五菱宏光 S，车牌云M×××××，该车行车证上所有人为李长雄。车内有 3 排座位，中排和后排座位中间摆放有 2 只绿色编织袋，2 只白色尼龙头透气口袋，一个纸盒，一个纸袋，一个红色编织袋。2 只白色

尼龙透气口袋内分别装有1只疑似野生动物活体，勘验人员对该2只疑似野生动物活体编号为1号、2号，1号、2号疑似野生动物活体特征为体型狭长，全身有鳞甲，呈褐色，背面略隆起，头呈圆锥状，眼小，四肢粗短，足具五趾，有足爪，尾扁平而长，为疑似穿山甲活体。1号右后脚残缺、残缺尾部伤口已愈合，长94厘米，重5.36千克；2号长83厘米，重4.48千克。民警对疑似穿山甲活体和车牌云M×××××白色五菱微型车进行了扣押。

4.各被告人的辨认笔录及照片。

（1）被告人邵南的辨认笔录及照片。①被告人邵南对出售给宋德万、密加旺的1只穿山甲、出售给李长雄的2只穿山甲、出售给段美英的白眉长臂猿头颅4个、穿山甲甲片2千克、出售给陈某的白眉长臂猿头颅1个及穿山甲甲片12.36千克进行了辨认指证。②被告人邵南对其本人用于运输野生动物及制品的缅甸牌号9J-7507银灰色三菱越野车进行了指认；③被告人邵南对驾驶车辆帮其走私运输穿山甲入境的“滇滩的老师傅”即徐某（另处）进行了指认；④被告人邵南对与其购买野生动物及制品的李长雄、陈某、段美英的照片进行了辨认。⑤被告人邵南对走私入境地点、交易地点进行了指认。

（2）被告人李长雄的辨认笔录及照片。被告人李长雄对现场查获的2只活体穿山甲进行了指认、对用于运输的云M×××

××车辆及穿山甲装放位置进行了指认、对其购买穿山甲的“吉盛招待所”的房间进行了指认。

（3）被告人段美英的辨认笔录及照片。被告人段美英对其购买被查获的4只猴头头颅、四袋穿山甲甲片进行了指认，对住宅内存放该野生动物制品的地点、固东镇“四川林茂饭店”交易地点进行了指认；对出售给其野生动物制品的缅甸妇女（邵南）照片进行了辨认指证。

（4）被告人宋德万、密加旺的辨认笔录及照片。被告人宋德万、密加旺均对现场查获的活体穿山甲1只、杆秤及包装袋各1个进行了辨认指证。

5.《国家林业局令》第46号。证实涉案野生动物及其制品经济价值认定依据。

6.鉴定意见及通知书、价值情况说明。

（1）云南濒科委司法鉴定中心出具的濒司鉴（动）字[2019]641号司法鉴定意见书及鉴定意见通知书证实：经对从邵南家查获的疑似熊油制品和疑似野牛胆制品鉴定，鉴定意见为由于检材为动物残体或提炼物，无可识别的动物鉴别特征，无法践行种属、保护级别和价值鉴定。

（2）云南濒科委司法鉴定中心出具的濒司鉴（动）字[2019]639号司法鉴定意见书及鉴定意见通知书证实：经对从段美英处查获疑似穿山甲甲片4袋、疑似白眉长臂猿头颅4个鉴定，鉴定意见为1-4号检材源自鳞甲目穿山甲科穿山甲属穿山甲

Manissp，为国家Ⅱ级保护动物，列入《濒危野生动植物种国际贸易公约》（CITES）附录Ⅰ；5-8号检材源自灵长目长臂猿科白眉长臂猿属白眉长臂猿Hylobateshoolock，为国家Ⅰ级保护动物，列入《濒危野生动植物种国际贸易公约》（CITES）附录Ⅰ。1-8号检材经济价值为1736960元。

（3）云南濒科委司法鉴定中心出具的濒司鉴（动）字[2019]640号司法鉴定意见书及鉴定意见通知书证实：经对在邵南、宋德万、密加旺交易现场查获疑似穿山甲活体1只进行鉴定，鉴定意见为送检照片中疑似穿山甲活体来源于鳞甲目穿山甲科穿山甲属印度穿山甲Maniscrassicaudata，列入《濒危野生动植物种国际贸易公约》（CITES）附录Ⅰ。腾冲市森林公安局出具关于该穿山甲价值的情况说明一份：该穿山甲经济价值为40000元。

（4）云南濒科委司法鉴定中心出具的濒司鉴（动）字[2019]562号司法鉴定意见书证实：经对现场查获李长雄涉嫌运输的2只疑似穿山甲活体鉴定，鉴定意见为送检照片中2只疑似穿山甲活体来源于鳞甲目穿山甲科穿山甲属穿山甲Manispentadactyla，穿山甲为国家Ⅱ级保护野生动物，列入《濒危野生动植物种国际贸易公约》（CITES）附录Ⅰ。腾冲市森林公安局出具关于该2只穿山甲价值的情况说明一份：该2只穿山甲经济价值为80000元。

（5）云南濒科委司法鉴定中心出具的濒司鉴（动）字[2019]254号司法鉴定意见书证实：陈某家中查获的疑似野生动物及野生动物制品经鉴定后的物种来源及经济价值。其中其指证跟邵南购买的穿山甲甲片部分即26号、32号、33号检材（计12.36千克）源自鳞甲目穿山甲科穿山甲属穿山甲Manissp，为国家Ⅱ级保护动物，列入《濒危野生动植物种国际贸易公约》（CITES）附录Ⅰ；疑似猴头一个即21号检材具有白眉长臂猿的典型特征，为国家Ⅰ级保护动物，列入《濒危野生动植物种国际贸易公约》(CITES)附录Ⅰ。该鉴定意见评定涉案猴头价值400000元。腾冲市森林公安局出具涉案的12.36千克穿山甲甲片价值的情况说明一份：该穿山甲甲片经济价值为846720元。

7.被告人供述和辩解。

（1）被告人邵南的供述和辩解。

被告人邵南在侦查阶段先后有六次供述，证实其本人曾出售给陈某20多市斤穿山甲甲片、1个猴头；出售给李长雄3只穿山甲活体共重约25市斤；出售给段美英4个后头、4市斤穿山甲甲片；出售给宋德万、密加旺1只穿山甲活体。这些野生动物及其制品都是向缅甸“阿撒”和“莫萨”购买后走私入境的。

（2）被告人李长雄的供述和辩解。被告人李长雄在侦查阶段先后有四次供述，证实其以6600元的价格在滇滩镇吉盛招待所9号房间向一个缅甸女人购买了2只穿山甲活体，共重19市斤。一只是寸文辉让帮买的，一只其自用。知道穿山甲是这个缅

甸女人从缅甸石灰卡拿来的，也知道穿山甲是国家重点保护的野生动物。

（3）被告人段美英的供述和辩解。被告人段美英在侦查阶段先后有五次供述，证实其分别于2019年5月底和6月12日先后两次共向一名缅甸妇女购买穿山甲甲片共4市斤，黑猴头共4个。买这些东西主要是自己做药吃，如果有人需要也会出售获利。以前在农村做草药生意，清楚国家禁止非法收购、出售野生动物及野生动物制品的。但自认为其是购买来自己食用，顺便出售一点获利，认为数量少不会有事，晓不得有那么严重。

（4）被告人宋德万的供述和辩解。被告人宋德万在侦查阶段先后有三次供述，证实其不知道邵南的穿山甲的来源。但知道穿山甲属于国家重点保护动物，是禁止非法收购、出售的。其和密加旺合伙向邵南购买1只穿山甲活体，交易过程中被当场抓获。

（5）被告人密加旺的供述和辩解。被告人密加旺在侦查阶段先后有三次供述，自己和宋德万合伙向邵南购买1只穿山甲活体，交易过程中被当场。不知道邵南的穿山甲的来源，但知道穿山甲属于国家重点保护动物，是禁止非法收购、出售的。

8.证人证言。

（1）证人余某（系邵南的丈夫）证实：2019年6月5日凌晨邵南在滇滩镇吉盛招待所9号客房出售给一名男子2只活体穿

山甲；6 月 14 日宋德万联系余某向购买穿山甲甲片，余某告知要问邵南。

（2）证人木某证实：2019 年 6 月 18 日邵南卖给两个男子穿山甲后被抓获期间其与邵南在一起，但是没有参与邵南买卖穿山甲，事前不知道邵南是卖穿山甲。

（3）证人段某（系被告人段美英妹妹）证实：2019 年 6 月初，段美英拿过一个黑猴头给自己吃了治头疼，但自己没敢吃。6 月 18 日将该黑猴头交给侦查机关。并对自己摆放黑猴头的房间进行了辨认指证。

（5）证人寸文辉证实，其没有让李长雄为自己购买穿山甲。

（6）证人张某（滇滩镇吉盛招待所经营者）证实：2019 年 6 月 5 日有一对夫妇（女的是缅甸人，男的是中国人）到吉盛招待所休息了一下，夜里就走了，不知道对方在自己的招待所里卖穿山甲。

（7）证人杨某 1 证实：2019 年 6 月 17 日问过宋德万是否有穿山甲甲片，后宋德万说有一只活的穿山甲问要不要，然后自己就答应了。6 月 18 日早上 9 点多宋德万打电话说去马站拿穿山甲了，之后就没有再联系。

（8）证人彭某证实：2019 年 6 月 18 日早上宋德万向自己借过一把秤，宋德旺告知是去买白鱼，和宋德万一起的还有一个不知道叫什么名字的人。

（9）证人陈某证实：自己向余某的妻子（邵南）以 1500 元的价格购买过穿山甲甲片，以 200 元的价格购买过 1 个黑猴头，还购买过 2 只死的穿山甲，交易地点是滇滩镇吉盛招待所。

（10）证人林某 1 证实：其曾向李长雄购买过 2 只穿山甲死体。

（11）证人徐某证实：2019 年 6 月初帮一名缅甸女子接送过 1 只活体穿山甲，2019 年春节前该女子乘坐其车时候看到该女子背包内有 3 个猴子头。

9.视听资料。腾冲市滇滩镇吉盛招待所 2019 年 6 月 5 日 0 时 39 分至 2 时 39 分监控视频，证实邵南与李长雄在该招待所有过穿山甲交易情形。

10.情况说明。证实涉案查获的 3 只穿山甲活体经委托云南鲮鲤科技有限公司饲养，因穿山甲不进食，发生了先后死亡的情形。

附带民事公益诉讼起诉人同时提交了以下证据：

1.公益诉讼起诉人的机构证明，证实诉讼主体适格。

2.提起公益诉讼的公告、请示、批复，证实提起刑事附带民事公益诉讼程序合法。

3.对各被告提起公益诉讼的询问笔录，证实进一步核实了各被告的涉案事实。

4.证人胡某证言，证实其系云南鲮鲤科技开发有限公司职工，腾冲市森林公安局委托送养的李长雄案的 2 只活体穿山甲，

宋德万、密加旺案的 1 只活体穿山甲因有被注水或灌沙嫌疑，未能饲养成活，先后已经死亡的事实。

5.现场检查照片，证实已对腾冲市森林公安局赃物保管室进行检查，发现涉案的 3 只穿山甲已死亡被冷藏于冰柜。

6.刑事部分已经收集在案证据复印件，证实公诉机关指控各被告的犯罪事实客观存在。

上述证据，收集程序合法，内容客观真实，与本案有关联性，能够印证本案认定事实，本院予以确认。

被告人段美英的辩护人向法庭提交了林某 2 通[1996]8 号文、林护字[1993]72 号文，欲证实涉案物品的鉴定应以此为据，段美英涉案的猴头、穿山甲甲片价值只应计算得 325718.08 元；并提供了三份网上裁判文书，以证实类似案件判处情况。对该组证据，经当庭举证、质证，合议庭认为上述两份文件内容已被 2017 年 12 月 15 日公布实施的《国家林业局令》第 46 号取代，相关案例均是在该令实施前判处的，不具有指导意义，故对该组证据不予采纳。

本院认为，被告人邵南为牟利，先后走私、出售给他人活体穿山甲 3 只、白眉长臂猿头颅 5 个、穿山甲甲片 14.36 千克，鉴定价值人民币 3103680 元，其行为已触犯刑律，构成走私珍贵动物、珍贵动物制品罪。被告人段美英明知邵南出售的珍贵动物制品系走私入境，仍向其购买白眉长臂猿头颅 4 个、穿山甲甲片 2 千克，鉴定价值达人民币 1736960 元，其行为已触犯刑律，构成

走私珍贵动物制品罪。被告人李长雄明知邵南出售的珍贵动物系走私入境，仍向其购买活体穿山甲 2 只，鉴定价值达人民币 80000 元，其行为已触犯刑律，构成走私珍贵动物罪。被告人宋德万、密加旺合伙向被告人邵南购买活体穿山甲 1 只，鉴定价值达 40000 元，二人的行为已触犯刑律，均构成非法收购珍贵、濒危野生动物罪。公诉机关指控各被告人的犯罪事实清楚、证据确实、充分，罪名成立，本院予以确认。各辩护人均提出涉案鉴定价值过高的辩护意见，经审查，本案鉴定意见系具有鉴定资质的机构、人员依程序作出，合法有效，对该项辩护意见本院不予采纳。被告人邵南走私珍贵动物、珍贵动物制品情节特别严重，鉴于其能坦白认罪，本院依法予以从轻处罚。被告人段美英走私珍贵动物制品情节特别严重，鉴于其能坦白认罪，本院依法予以从轻处罚，其辩护人关于鉴定人拒不出庭作证的辩护意见与客观事实不符，本院不予采纳。被告人李长雄走私珍贵动物情节严重，虽认罪认罚，鉴于其曾因非法收购珍贵动物制品罪已被判处刑罚，仍不思悔改，主观犯意较深，对公诉机关及其辩护人关于从轻判处的量刑意见予以支持，但不予适用缓刑。被告人宋德万、密加旺共同非法收购珍贵、濒危野生动物，系共同犯罪，因二人作用地位相当，本院不予区分主从，鉴于二人能坦白认罪，本院依法予以从轻处罚并采纳公诉机关及辩护人的量刑意见，予以适用缓刑。

刑事附带民事公益诉讼被告邵南、段美英、李长雄、宋德万、密加旺明知穿山甲、白眉长臂猿等物种系国家保护的野生动物仍

然予以走私、贩卖、收购的行为已对生态环境造成严重破坏，造成野生动物资源受损、生物多样性遭到破坏，社会公共利益受损。各被告人虽不是直接猎杀者，但其买卖行为推动了野生动物资源损失的进度，加快了物种灭绝的速度，各被告人应分别承担相应的侵权责任。公益诉讼起诉人针对被告邵南、段美英、李长雄、宋德万、密加旺提出的诉讼请求与法有据，本院予以支持。野生动物资源属国家所有，且我国是《濒危野生动植物种国际贸易公约缔约国》，依法负有保护职责，而保山市人民检察院作为国家法律监督机关，有权提起刑事附带民事公益诉讼，故被告邵南、段美英的辩护人对公益诉讼起诉人主体不适格的答辩意见不能成立，对该答辩意见本院不予支持。各答辩人对公益诉讼赔偿金额过高的异议不能成立，本院不予支持。本案中关于诉请邵南与另案被告陈某共同承担赔偿责任部分，因陈某并非本案当事人，故不在本案中判决陈某承担侵权责任。

根据《中华人民共和国刑法》第一百五十一条第二款、第一百五十五条、第三百四十一条第一款、第二十五条、第六十七条第三款、第六十四条，《中华人民共和国刑事诉讼法》第一百零一条第二款，最高人民法院、最高人民检察院《关于办理走私刑事案件适用法律若干问题的解释》第九条第三款（二）项，《中华人民共和国野生动物保护法》第三条第一款、第二十七条第一款、第三十五条第一、二、四款，《中华人民共和国侵权责任法》第四条第一款、第六条第一款、第八条、第十五条第一款第(六)、

(七)项、第二款，最高人民法院《关于审理环境民事公益诉讼案件适用法律若干问题的解释》第十八条的规定，判决如下：

一、被告人邵南犯走私珍贵动物、珍贵动物制品罪，判处有期徒刑十一年，并处没收个人财产人民币 110000 元。

（刑期从判决执行之日起计算。判决执行前先行羁押的，羁押一日折抵刑期一日，即自 2019 年 6 月 19 日起至 2030 年 6 月 18 日止）

二、被告人段美英犯走私珍贵动物制品罪，判处有期徒刑十年，并处没收个人财产人民币 100000 元。

（刑期从判决执行之日起计算。判决执行前先行羁押的，羁押一日折抵刑期一日，即自 2019 年 6 月 19 日起至 2029 年 6 月 18 日止）

三、被告人李长雄犯走私珍贵动物罪，判处有期徒刑二年，并处罚金人民币 20000 元。

（刑期从判决执行之日起计算。判决执行前先行羁押的，羁押一日折抵刑期一日，即自 2019 年 6 月 5 日起至 2021 年 6 月 4 日止）

四、被告人宋德万犯非法收购珍贵、濒危野生动物罪，判处有期徒刑一年，缓刑二年，并处罚金人民币 10000 元。

（缓刑考验期从判决确定之日起计算）

五、被告人密加旺犯非法收购珍贵、濒危野生动物罪，判处有期徒刑一年，缓刑二年，并处罚金人民币 10000 元。

（缓刑考验期从判决确定之日起计算）

六、附带民事公益诉讼被告邵南、段美英、李长雄、宋德万、密加旺分别对其侵权行为在保山市市级以上媒体公开赔礼道歉。限本判决生效次日起30日内完成。

七、附带民事公益诉讼被告邵南赔偿因其侵权行为造成的野生动物资源损失费人民币1246720元，上缴国库。限本判决生效次日起30日内赔偿。

八、附带民事公益诉讼被告邵南与被告段美英连带赔偿因其侵权行为造成的野生动物资源损失费人民币1736960元，上缴国库。限本判决生效次日起30日内赔偿。

九、附带民事公益诉讼被告邵南与被告李长雄连带赔偿因其侵权行为造成的野生动物资源损失费人民币80000元，上缴国库。限本判决生效次日起30日内赔偿。

十、附带民事公益诉讼被告邵南与被告宋德万、密加旺连带赔偿因其侵权行为造成的野生动物资源损失费人民币40000元，上缴国库。限本判决生效次日起30日内赔偿。

十一、扣押在案的穿山甲甲片2千克、穿山甲死体3只、白眉长臂猿头颅4个、疑似熊油制品30瓶及1袋、三菱车1辆（缅甸车牌号9J-7507）、微型车1辆（MEA699）、人民币17719.5元依法予以没收；白色塑料编织袋1只、彩色尼龙袋1只、黑色双肩包1个作为物证随案保存。

如不服本判决，可在接到判决书的第二日起十日内，通过本院或直接向中华人民共和国云南省高级人民法院提出上诉。书面上诉的，应当提交上诉状正本一份，副本二份。

审　判　长　张　艳　昌

审　判　员　丁　　　烈

审　判　员　赵　爱　超

人民陪审员　寸　待　勇

人民陪审员　郭　兆　锦

人民陪审员　夏　治　伟

人民陪审员　段　体　要

二〇一九年十二月二十四日

书　记　员　张杨文婧
