# Supplementary material for: China’s Legal Protection System for Pangolins: Past, Present, and Future
Source: Animals (Basel). 2025 Aug 18;15(16):2422. doi: 10.3390/ani15162422 (PMC12383201; doi:10.3390/ani15162422)
Supplement: Supplementary file 1 [file animals-15-02422-s001.zip › Supplementary Material S4-Full Text of Judgments in Pangolin-Related Public Interest Litigation Cases in China/【1】李某走私珍贵动物制品案.pdf]

2024-06-1-082-001

李某走私珍贵动物制品案

——非原产于我国物种的保护级别的认定

关键词 刑事 走私珍贵动物制品罪 保护级别 非原产物种

基本案情

被告人李某从几内亚出发，经迪拜转机后，于2019年1月19日抵达北京首都国际机场T3航站楼，入境时选走无申报通道，未向海关申报任何货物、物品，海关关员在其携带的行李箱中查获疑似穿山甲鳞片4包。经鉴定，上述疑似穿山甲鳞片来源于鳞甲目穿山甲科长尾穿山甲属黑腹长尾穿山甲和树穿山甲，共计价值人民币275 200元。

北京市第四中级人民法院于2020年11月26日作出（2020）京04刑初25号刑事判决：被告人李某犯走私珍贵动物制品罪，判处有期徒刑二年六个月，并处罚金人民币二万五千元。宣判后，在法定期限内没有上诉、抗诉，判决已发生法律效力。

裁判理由

法院生效裁判认为：对于李某的辩护人所提不认可云南某某司法鉴定中心《司法鉴定意见书》按照国家一级保护动物基准价值的10倍核算涉案金额，应当按照国家二级保护动物基准价值的5倍核算案涉金额的相关辩护意见，经查，《司法鉴定意见书》“基本情况”部分表明北京海关缉私局的鉴定事项为穿山甲鳞片的保护级别和价值，“分析说明”部分详细解释了该鉴定机构认定黑腹长尾穿山甲和树穿山甲是《濒危野生动植物种国际贸易公约》（CITES）附录I物种、二者均是（等同于）中国国家一级保护野生动物及按照所列野生动物基准价值的十倍核算的理由及法律依据，云南某某司法鉴定中心的鉴定程序规范、合法，鉴定意

见客观真实，可以作为定案的依据，故李某的辩护人的辩护意见缺乏事实和法律依据，法院不予采纳。故法院依法作出如上裁判。

裁判要旨

在无特别规定时，《濒危野生动植物种国际贸易公约》（CITES）附录Ⅰ与附录Ⅱ中非原产于我国的物种分别对应国家一级保护野生动物和国家二级保护野生动物。

关联索引

《中华人民共和国刑法》第151条第2款

《最高人民法院、最高人民检察院关于办理走私刑事案件适用法律若干问题的解释》（法释〔2014〕10号）第9条

一审：北京市第四中级人民法院（2020）京04刑初25号刑事判决（2020年11月26日）

人民法院案例库
