# Supplementary material for: China’s Legal Protection System for Pangolins: Past, Present, and Future
Source: Animals (Basel). 2025 Aug 18;15(16):2422. doi: 10.3390/ani15162422 (PMC12383201; doi:10.3390/ani15162422)
Supplement: Supplementary file 1 [file animals-15-02422-s001.zip › Supplementary Material S4-Full Text of Judgments in Pangolin-Related Public Interest Litigation Cases in China/【20】常小华走私珍贵动物、珍贵动物制品一审刑事判决书(FBM-CLI.C.103532608).pdf]

常小华走私珍贵动物、珍贵动物制品一审刑事判决书

常小华走私珍贵动物、珍贵动物制品一审刑事判决书

云南省保山市中级人民法院

刑事附带民事判决书

(2019)云05刑初234号

公诉机关暨附带民事公益诉讼起诉人云南省保山市人民检察院。

被告人暨附带民事公益诉讼被告常小华。因本案于2019年5月1日被刑事拘留，同年5月10日被逮捕，现羁押于云南省腾冲市看守所。

傈某。

指定辩护人赵圭，云南天之泰律师事务所律师。

云南省保山市人民检察院以保检四部刑诉[2019]5号起诉书指控被告人常小华犯走私珍贵动物制品罪，于2019年11月27日向本院提起公诉，并作为公益诉讼起诉人以保检民公[2019]53050000001号刑事附带民事公益诉讼起诉书向本院提起附带民事公益诉讼。本院受理后，依法组成合议庭，于2019年12月17日在腾冲市人民法院第一法庭公开开庭进行了合并审理。云南省保山市人民检察院指派检察员刘双道、书记员余亚斌出庭支持公诉并参加附带民事公益诉讼。被告人暨附带民事公益诉讼被告常小华及其辩护人赵圭、翻译余某到庭参加诉讼。本案现已审理终结。

云南省保山市人民检察院指控：2019年4月30日，被告人常小华安排他人将穿山甲甲片从缅甸携带运输至中国腾冲市极源酒店。当日，腾冲市森林公安局民警在滇滩镇极源酒店抓获常小华，当场查获常小华放置在极源酒店的穿山甲甲片一袋，净重14.1千克。经鉴定，查获的穿山甲甲片来源于鳞甲目穿山甲科,穿山甲为国家ＩＩ级保护野生动物，被列为《濒危野生动植物种国际贸易公约》（ＣＩＴＥＳ）附录Ｉ,经济价值为966080元。

2019年4月被告人常小华携带6只熊掌从缅甸入境至中国腾冲市，在滇滩仁和食馆以人民币600元出售给陈昌友（另处）。2019年4月26日腾冲市森林公安局民警在陈昌友（另处）家查获该6只熊

掌。经鉴定，6只熊掌来源于熊科熊属黑熊，黑熊是国家ＩＩ级保护野生动物，黑熊列入《濒危野生动植物种国际贸易公约》（ＣＩＴＥＳ）附录Ｉ，6只黑熊熊掌至少来源于2只黑熊，经济价值为48000元。

公诉机关认定上述事实的证据有：物证；书证；证人证言；被告人的供述和辩解；鉴定意见；勘验、检查、辨认等笔录；视听资料等。公诉机关认为，被告人常小华非法走私穿山甲甲片14.1千克、熊掌6只，经济价值合计101.408万元，其行为触犯了《中华人民共和国刑法》第一百五十一条第二款的规定，应当以走私珍贵动物制品罪追究其刑事责任。

公益诉讼起诉人保山市人民检察院诉称：经依法审查查明的犯罪事实，被告人常小华走私珍贵动物制品的犯罪行为违反了国家有关野生动物保护法律、法规的规定，严重破坏了生物多样性和自然生态平衡，造成野生动物资源受损，社会公共利益受到侵害，根据《中华人民共和国野生动物保护法》第二十七条第一款、第三十五条第一、二、四款、《中华人民共和国侵权责任法》第八条、第十四条和最高人民法院《关于审理环境民事公益诉讼案件适用法律若干问题的解释》第十八条的规定，1、请求判令被告常小华对其走私珍贵动物制品的行为在市级以上媒体公开赔礼道歉；2、请求判令被告常小华与陈昌友共同承担因走私珍贵动物制品造成的野生动物资源损失费48000元；3、请求判令被告常小华承担因走私珍贵动物制品造成的野生动物资源损失费966080元。

被告人常小华对指控的行为过程和罪名不持异议，辩称其不知道是穿山甲甲片，但在最后陈述中承认自己知道是穿山甲甲片而予运输。对附民公益诉讼请求，其表示愿意承担赔礼道歉和赔偿的责任，但是没有钱赔偿。其辩护人提出的辩护意见是：被告人常小华有坦白情节，鉴定意见的鉴定价值过高。

经审理查明:2019年4月30日，被告人常小华安排他人将穿山甲甲片从缅甸携带运输至中国腾冲市极源酒店。当日，腾冲市森林公安局民警在滇滩镇极源酒店抓获常小华，当场查获常小华放置在极源酒店的穿山甲甲片一袋，净重14.1千克。经鉴定，查获的穿山甲甲片来源于鳞甲目穿山甲科,穿山甲为国家ＩＩ级保护野生动物，被列为《濒危野生动植物种国际贸易公约》（ＣＩＴＥＳ）附录Ｉ,经济价值为966080元。

2019年4月被告人常小华携带6只熊掌从缅甸入境至中国腾冲市，在滇滩镇仁和食馆以人民币600元出售给陈昌友（另处）。2019年4月26日腾冲市森林公安局民警在陈昌友（另处）家查获该6只

熊掌。经鉴定，6只熊掌来源于熊科熊属黑熊，黑熊是国家ＩＩ级保护野生动物，黑熊列入《濒危野生动植物种国际贸易公约》（ＣＩＴＥＳ）附录Ｉ，6只黑熊熊掌至少来源于2只黑熊，经济价值为48000元。

被告人常小华走私珍贵动物制品经济价值共计人民币1014080元。

上述事实，有下列经当庭举证、质证的证据证实：

一、刑事部分；

1.物证：查获的穿山甲甲片14.1千克。证实被告人常小华走私珍贵动物制品的部分犯罪对象。

2.书证：（1）户口证明、HIV抗体确证检测报告单，证实：被告人常小华的身份信息，辖区内无违法犯罪记录。2015年2月10日经腾冲疾控中心检测，被告人常小华HIV-1抗体阳性。

（2）腾冲市森林公安局抓获经过，证实：2019年4月30日，腾冲市森林公安局民警在打击野生动物资源专项行动中根据案件线索到腾冲市极源酒店内抓获被告人常小华，民警在常小华的带领下到极源酒店一楼总台右边的一间房屋内查获穿山甲甲片1编织袋。

（3）出入境记录查询结果单、被告人常小华提交的缅甸与中国边界通行证及车辆进境申报单，证实：被告人常小华于2019年4月30日19时18分持有缅甸与中国边界通行证（证件号：036977）驾驶KSR8350北汽汽车从猴桥口岸入境；被告人常小华多次持缅甸与中国边界通行证出入中国境内。

（4）腾冲市滇滩镇极源酒店胡云若出具的情况说明，说明：腾冲市滇滩镇极源酒店内安装的监控视频只能保存7日，2019年4月30曰19时0分至20时40分极源酒店监控视频已被覆盖，现已无相关监控视频。

（5）腾森公（刑）立字（2019）6号立案决定书，证实：犯罪嫌疑人陈昌友涉嫌非法收购、出售珍贵、濒危野生动物、珍贵濒危野生动物制品案腾冲市森林公安局于2019年4月26日立案侦查。

（6）腾森公（刑）立字（2019）6号立案决定书，证实：犯罪嫌疑人徐志富涉嫌非法运输珍贵、濒危野生动物，珍贵、濒危野生动物制品案于2019年7月5日被立案侦查。

（7）常小华与徐志富的通话记录材料，证实：常小华与徐志富2019年4月份之间多次电话联系，其中2019年4月30日通话15次。

（8）常小华与陈昌友（武学奇）的通话记录材料，证实：2019年4月1日至4月26日，陈昌友与

常小华通话12次。

（9）腾冲市森林公安局出具的情况说明，证实①131××××0112登记注册人名为“哄大力"，该号码实际使用人为常小华，其在缅甸板瓦手机店购买时店主帮忙登记注册。②158××××3481登记注册人为武学奇，该号码为陈昌友之子武学奇登记注册后给陈昌友使用，实际使用人为陈昌友。③本案线索来源是办理陈昌友案中，陈昌友供述常小华是自己在缅甸的上家，且手机联系，民警对二人微信聊天记录进行勘验检查发现常小华近期有作案的可能后，在滇滩境内布控后抓获常小华。抓获当时查获常小华衣服口袋中的一部ＶＩＶＯ手机，对手机检查时未发现有价值信息，于2019年5月2日由常小华母亲三路波领回。④对常小华的第2-5次讯问，因腾冲市看守所录音录像设备存在故障未能进行刻录。侦查人员在抓获常小华时使用汉语与常小华交流，常小华能使用汉语回答侦查人员提问，整个侦查过程中常小华都是使用汉语与侦查人员交流，没有提出需要聘请翻译人员的要求。

3.证人证言：证人谷某证言，证实：我在腾冲市子边经营着极源酒店，昨天下午8点30分左右，腾冲市森林公安局民警在我经营酒店一楼总台右手边闲置的餐厅大厅内查获一袋穿山甲壳，货主也抓着了。我看到货主后我才知道，你们抓获他前他给我打过一个电话问我有没有房间了，我告诉他房间卖完了没有了，他给我打电话后不久就被抓获了。他的穿山甲甲片放在我酒店一楼总台右手边闲置的餐厅大厅内，那个大厅是闲着的，门随时开着，他什么时候去放的我没有看到。

4.被告人的供述与辩解：（1）被告人常小华的供述与辩解，证实：我叫常小华，十年前我到缅甸取了一个媳妇，两年前我在缅甸办了马帮丁（缅甸身份证），缅甸名字叫吴勒么撒。我现在的中国身份证没有注销，我放在缅甸住处，没有带过来，身份证号码53xxx83××××××××，户籍所在地：云南省怒江傈僳族自治州泸水县。我使用的微信名叫“神爱世人"，微信号是“ｗｘｉｄ-ｉＤ5ｒ894ｘｗ0ｖｂｉ22"。电话号码是131××××0112。

2019年4月的一天，“哇大"（与运输穿山甲的“哇大"不是同一人）问我中国是否有人要熊掌，他那里有6只，叫我帮联系，但是熊掌有点变质（有臭味），卖得多少钱都可以，到时候他分点钱给我，我就打电话给腾冲市的陈昌友，他说要的，由于熊掌变质了就讲好80元人民币每市斤，我在缅甸板瓦和陈昌友联系好后，我在板瓦同“哇大"拿了熊掌，拿了之后我在板瓦找了一个叫“依得"的当地村民，说好给他50元钱人民币，叫“依得"帮我从缅甸板瓦背着熊掌从小路绕开边防检查站到中国的小路边等我，到时候我会来和他接货，之后我就从板瓦通过边检站正常入境到中国，在口岸我找了一辆客运

车，上车后过了600米左右，我在小路边向“依得"接到熊掌，并给了他50元钱，他原路返回板瓦，我坐着车带着熊掌到滇滩，当我到滇滩的时候陈昌友已经在滇滩仁和食馆边等着我了，我俩在仁和食馆后门板栗树下交货，当时我们讲好熊掌80元每市斤，我们俩交易完后我就离开了，我自己由于感冒，我在滇滩输液打针，到第二天我才回板瓦，回到缅甸板瓦后“哇大"给了我300元钱。

2019年4月30日早上，“哇大"开车来我家找我，说叫我帮他从板瓦带点穿山甲甲片到中国，给我人民币1500元钱，他告诉我货在板瓦菜市场的一家卖百货的店铺，之后我就打电话给“老谢"（徐志富）叫他帮我拉些“片片"（指穿山甲甲片），他说他正好要拉菜来板瓦。我和“哇大"在菜市场等着“老谢"，到中午些的时候“老谢"到板瓦菜市场，当时哇大、“老谢"我们3人都在场，我们讲找一个人将货从小路背到中国检查站公路边，到时候就叫老谢帮拉运到滇滩，老谢到路边打着转弯灯等着，背货的人会来找他，老谢说可以的。我拿到甲片后找了板瓦当地的村民“阿的"，我叫他帮我把穿山甲甲片拿到中国滇滩边防检查站滇滩一侧，我开给他100元钱，到时候会有一张白色的轿车在路边打着转弯灯接货，我叫“阿的"将货交给开车的人就可以，具体入境路线叫他自己想办法，我先从缅甸板瓦驾车到滇滩等着。随后我就驾车先从缅甸板瓦通过滇滩口岸到了滇滩镇街子极源酒店等着，期间我还联系了“老谢"好几次，问他到什么地方了，是否接到货之类的话，过了一段时间，“老谢"帮我拉着货到极源酒店交给我，我给了“老谢"100元钱后他就走了，我接到甲片后我就将甲片拿到极源酒店一楼总台右手边房屋一进门的地上摆着，我在酒店边的餐馆准备吃饭时被你们查获了。穿山甲甲片带来到滇滩后准备联系出售给中国腾冲市的陈昌友。

（2）另案犯罪嫌疑人陈昌友的供述与辩解，证实：我收购、出售过的野生动物有活体穿山甲、穿山甲甲片、熊掌、熊胆、眼镜蛇、猴头、豪猪刺等，我跟郭茂昌、余学纲的妻子、张绍伟以及常小华购买的。2019年4月26日公安在我家查获的6只熊掌、3个熊胆（大的两个是常小华卖给我的，小的1个是马国武拿给我的这个应该是假的）以及熊油都是常小华卖给我的。我跟常小华买的6只熊掌他卖给我的时候有点变质（臭了），是以80元每市斤的价格购买的，总的是一千五还是一千六百块钱我记不清了。跟常小华购买熊掌的地点是在滇滩仁和食馆的后大门边一棵板栗树脚，我已经带公安去进行过辨认了。我使用的电话号码是158×××3481，登记着武学奇的名字。

（3）另案犯罪嫌疑人徐志富的供述与辩解，证实：2019年4月月底的一天上午，我用我的云M×××××小汽车帮别人拉运蔬菜到缅甸板瓦，还没到板瓦就接到怒江男子的电话说让我帮他带点东西

，到下午的时候我又帮别人拉着菜去到板瓦时，他在板瓦见到我的车就叫停，我就问他打电话给我让我帮拉的东西是什么，他说是“片片"（我自己清楚是穿山甲甲片），他说让我帮他拉着从滇滩口岸入境到滇滩，我说我不敢拉，之后我在板瓦拉到客人又返回了滇滩，又从滇滩拉运客人又到了缅甸板瓦，我准备返回滇滩时他又打电话给我，问我在哪里，我说我到口岸缅甸一侧的花杆了，然后他让我等着他，我就到了口岸中国一侧的边检站停车场下来一点的地方等他，他来到后跟我说他已经找人将甲片从缅甸走小路背到中国境内了，在下面的那个大弯子处，让我拉着他去大弯子处拿，拿了后再拉运他和甲片去滇滩镇街子极源酒店，我就拉着他到了大弯子小路口，他下车和一个男子拿了一袋甲片，之后他将甲片装上我的车子后备箱，我就将他送到了极源酒店，送到后他将甲片从我车子的后备箱拿下来，然后付给我了100元钱，我就开车走了。穿山甲甲片是用一个白色的编织袋包装着。

5.鉴定意见

（1）云南濒科委司法鉴定中心出具的濒司鉴（动）字（2019）250号及濒司鉴（动）字（2019）254号《司法鉴定意见书》2份、鉴定意见告知书，证实：①经鉴定，送检的14.1kg检材来源于鳞甲目穿山甲科穿山甲属Manissp。穿山甲为国家Ⅱ级保护野生动物，列入《濒危野生动植物种国际贸易公约》（CITES）附录I。经济价值为966080元。

②经鉴定，送检的（2、22-25）号检材来源于熊科熊属黑熊Ursusthibetanus，黑熊是国家II级保护野生动物，列入《濒危野生动植物种国际贸易公约》（CITES）附录I。经济价值为48000元。

上述鉴定意见2019年5月10日、7月3日已告知被告人常小华。

6.勘验、检查、辨认、侦查实验等笔录

（1）现场辨认笔录3份及辨认照片，证实：①2019年4月30日20:43-21:05，被告人常小华对其藏匿穿山甲甲片的地点（极源酒店一楼、一楼房间）、穿山甲甲片以及驾驶的车辆（ＫＲＳ-8530）进行了辨认。附指认照片6张。（见证人：段某1）

②2019年5月1日11:39-11:55，被告人常小华对其涉嫌走私的穿山甲的外包装（白色口袋）、内包装（绿色口袋）及口袋内装的穿山甲甲片进行辨认。附照片4张。（见证人：杨某）

③2019年5月1日14:10-15:05，被告人常小华对雇请“阿的"运输甲片绕开滇滩边防检查站通过的小道、其驾车进入中国境内通过边检站的地点及在滇滩镇与“阿的"交接货的小路路口分别进行辨认。附照片4张。（见证人：段某1）

（2）被告人常小华辨认笔录3份及辨认照片，证实：①2019年7月2日13:02-13:20，被告人常小华对出售给陈昌友熊掌的交易地点（滇滩仁和食管、食管后大门、食管后大门板栗树下）进行了指认。附照片3张。（见证人：段某1）

②2019年7月2日15:28-15:49，被告人常小华对出售给陈昌友的熊掌进行了指认。附照片2张。（见证人：杨某）

③2019年6月27日15:35-15:42，被告人常小华对10张不同免冠照片中辨认出7号照片男子（徐志富）就是2019年4月30日帮运输穿山甲甲片的男子“老谢"。附照片2张。（见证人：董某）

（3）现场勘验笔录及现场照片，证实：腾冲市森林公安局于2019年5月1日对查获被告人常小华的穿山甲甲片进行了勘验检查。经勘验，疑似穿山甲甲片用一个绿色编织袋装，编织袋口用白色包装绳捆扎，绿色编织袋外套有一个印有“鳄鱼"图案及“ＣＲＯＣＯＤＩＬＥ"字样的白色编织袋，打开两个编织袋，里面放有大量疑似穿山甲甲片，甲片呈黑褐色，边缘光滑，甲片基有纵纹，纵纹条数不一，随甲片大小而定，一部分甲片中央有龙骨状突起，一部分甲片为儿片粘在一起，甲片之间有硬毛，甲片分别呈菱形状、盾状、折合状三种形状，其特征与穿山甲甲片特征相符。勘查人员对查获的甲片进行称重，经称重，该疑似穿山甲甲片重14.1ｋｇ（除去口袋净重）。民警对上述疑似穿山甲甲片进行了提取、登记。（见证人：杨某）

（4）手机勘查笔录及微信聊天截图，证实：陈昌友与常小华自2019年3月15日至4月23日微信聊天记录情况，主要系陈昌友与常小华之间联系购买动物制品、走私运输的内容。附聊天截图24张。（见证人：杨某）

（5）徐志富辨认笔录，证实：2019年7月27日，徐志富从10张不同的免冠照片中辨认出7号照片男子（常小华），就是2019年4月月底雇请他从滇滩境内公路边与一个缅甸人接货后运输穿山甲甲片到滇滩镇极源酒店的人。附辨认照片2张。（见证人：董某）

（6）陈昌友辨认笔录，证实：2019年7月2日，陈昌友对跟常小华收购熊掌的地点（滇滩仁和食馆、板栗树下面）进行辨认。（见证人：段某2）

（7）扣押笔录、扣押决定书、扣押清单、发还清单，证实：2019年4月30日23:10-23:40，民警依法扣押查获的疑似穿山甲甲片14.4千克，及常小华驾驶的白色北汽汽车（ＫＲＳ-8530，后发还给常小华）。（见证人：杨某）

二、附带民事公益诉讼部分；

附带民事公益诉讼起诉人除了举出常小华犯罪的证据（已包含在刑事证据部分）之外，还举证：

1.组织机构证明，证实附带民事公益诉讼起诉人的主体身份及诉讼资格。

2.人民检察院公告、请示、批复，证实附带民事公益诉讼起诉人履行、完成了诉前公告程序及相关程序手续。

3.询问笔录，证实常小华走私穿山甲甲片14.1千克及熊掌6只的行为，与刑事证据一致。

附带民事公益诉讼被告常小华未向本院提交证据。

上述证据的收集程序合法，内容相互印证的部分客观真实，能够充分关联证实本案犯罪事实，及附带民事公益诉讼起诉人的诉讼请求，本院予以确认。

本院认为，被告人常小华走私价值1014080元的穿山甲甲片14.1千克、熊掌6只，其行为已触犯刑律，构成走私珍贵动物制品罪。公诉机关指控被告人常小华的犯罪事实清楚、证据确实、充分，罪名成立，本院予以确认。被告人常小华走私珍贵动物制品情节特别严重，鉴于其能坦白认罪，本院依法予以从轻处罚。其辩护人提出坦白从轻的辩护意见本院予以采纳；提出鉴定价值过高的辩护意见，经审查，本案鉴定意见系具有鉴定资质的机构、人员依程序作出，合法有效，辩护人此项辩护意见本院不予采纳。被告人常小华的犯罪行为还造成野生动物资源受损，社会公共利益受到侵害，公益诉讼起诉人的诉讼请求有充分证据证实，于法有据，但因为陈昌友并非本案当事人且未经审判，故不应在本案判决陈昌友承担赔偿责任。公益诉讼起诉人针对被告常小华的诉讼请求予以支持。依据《中华人民共和国刑法》第一百五十一条第二款、第六十七条第三款、第六十四条，最高人民法院、最高人民检察院《关于办理走私刑事案件适用法律若干问题的解释》第九条第三款（二）项，《中华人民共和国野生动物保护法》第三条第一款、第二十七条第一款、第三十五条第一、二、四款，《中华人民共和国侵权责任法》第六条第一款,第十五条第一款第(六)、(七)项、第二款，最高人民法院《关于审理环境民事公益诉讼案件适用法律若干问题的解释》第十八条的规定，判决如下：

一、被告人常小华犯走私珍贵动物制品罪，判处有期徒刑十年，并处没收个人财产人民币100000元。

（刑期从判决执行之日起计算。判决执行前先行羁押的，羁押一日折抵刑期一日，即自2019年5月1日起至2029年4月30日止）

二、附带民事公益诉讼被告常小华对其走私珍贵动物制品的行为在保山市市级以上媒体公开赔礼道歉。限本判决生效次日起30日内履行完成。

三、附带民事公益诉讼被告常小华赔偿因走私珍贵动物制品造成的野生动物资源损失费1014080元，上缴国库。限本判决生效次日起30日内赔偿。

四、驳回刑事附带民事公益诉讼起诉人云南省保山市人民检察院的其他诉讼请求。

如不服本判决，可在接到判决书的第二日起十日内，通过本院或者直接向云南省高级人民法院提出上诉。书面上诉的，应当提交上诉状正本一份，副本二份。

审　判　长　　丁　烈

审　判　员　　张艳昌

审　判　员　　赵爱超

人民陪审员　　寸待勇

人民陪审员　　郭兆锦

人民陪审员　　夏治伟

人民陪审员　　段体要

二〇一九年十二月十九日

书　记　员　　杨立丽

©北大法宝：（www.pkulaw.com）专业提供法律信息、法学知识和法律软件领域各类解决方案。北大法宝为您提供丰富的参考资料，正式引用法规条文时请与标准文本核对。 欢迎查看所有产品和服务。

法宝快讯： 如何快速找到您需要的检索结果？ 法宝 V6 有何新特色？

扫描二维码阅读原文

原文链接： https://www.pkulaw.com/pfnl/a6bdb3332ec0adc46f56a9d4351ac0fc4f953a3d558dfbdebdfb.html
