# Supplementary material for: China’s Legal Protection System for Pangolins: Past, Present, and Future
Source: Animals (Basel). 2025 Aug 18;15(16):2422. doi: 10.3390/ani15162422 (PMC12383201; doi:10.3390/ani15162422)
Supplement: Supplementary file 1 [file animals-15-02422-s001.zip › Supplementary Material S4-Full Text of Judgments in Pangolin-Related Public Interest Litigation Cases in China/【21】葛峰、章浩等非法出售珍贵、濒危野生动物制品一审刑事判决书.pdf]

葛峰、章浩等非法出售珍贵、濒危野生动物制品一审刑事判决书

江苏省南京市玄武区人民法院

刑 事 判 决 书

（2020）苏 0102 刑初 370 号

公诉机关南京市栖霞区人民检察院。

被告人葛峰，男，1992 年 6 月 8 日出生于江苏省徐州市，汉族，初中文化，个体户，户籍地江苏省徐州市鼓楼区，住江苏省徐州市。因涉嫌非法收购、出售珍贵、濒危野生动物制品罪，于 2018 年 12 月 8 日被刑事拘留，2019 年 1 月 14 日被取保候审。

指定辩护人庄宇，北京市隆安（南京）律师事务所律师。

被告人章浩，男，1990 年 12 月 18 日出生于江苏省徐州市，汉族，初中文化，个体户，户籍地江苏省徐州市铜山区，住江苏省徐州市鼓楼区。因涉嫌非法收购、出售珍贵、濒危野生动物制品罪，于 2018 年 12 月 8 日被刑事拘留，2019 年 1 月 14 日被取保候审。

指定辩护人朱芳雄，江苏永衡昭辉律师事务所律师。

被告人常晓霞，女，1989 年 4 月 10 日出生于江苏省盐城市，汉族，专科毕业，个体户，住江苏省盐城市经济技术开发区。因涉嫌非法收购、出售珍贵、濒危野生动物制品罪，于 2018 年 12 月 8 日被刑事拘留，2019 年 1 月 14 日被取保候审。

指定辩护人刘凯，江苏苏商律师事务所律师。

被告人周洋，男，1997年7月24日出生于山东省菏泽市，汉族，中专文化，无业，户籍地山东省菏泽市鄄城县，住北京市海淀区。因涉嫌非法收购、出售珍贵、濒危野生动物制品罪，于2018年12月10日被刑事拘留，2019年1月14日被取保候审。

指定辩护人王青松，江苏苏商律师事务所律师。

被告人苗禹，男，1981年2月18日出生于天津市红桥区，汉族，专科毕业，住天津市红桥区。因涉嫌非法收购、运输、出售珍贵、濒危野生动物制品罪，于2019年1月24日被刑事拘留，同年2月1日被取保候审。

指定辩护人宋璐，江苏泽栖律师事务所律师。

被告人沈才伟，男，1985年8月29日出生于重庆市合川区，汉族，初中文化，无业，住重庆市合川区。因涉嫌非法收购、运输、出售珍贵、濒危野生动物制品罪，于2019年4月25日被刑事拘留，同年5月25日被取保候审。

指定辩护人季一刚，江苏苏商律师事务所律师。

南京市栖霞区人民检察院以宁栖检诉刑诉〔2020〕314号起诉书指控被告人葛峰、章浩、常晓霞、周洋、苗禹、沈才伟犯非法出售珍贵、濒危野生动物制品罪，向本院提起公诉。本院受理后，依法组成合议庭，公开开庭审理了本案。南京市栖霞区人民检察院指派检察官鞠鹏出庭支持公诉，被告人葛峰、章浩、常晓霞、周洋、苗禹、沈才伟及各自辩护人到庭参加诉讼。本案现已审理终结。

南京市栖霞区人民检察院指控，2016年以来，被告人葛峰同被告人章浩共同出资在江苏省徐州市八里村一民房里加工野生动物制品并出售，被告人葛峰主要负责购买原料、出售野生动物制品，被告人章浩具体负责加工。2018年12月7日,公安机关在被告人葛峰、章浩的加工点及住处等地，查获一批野生动物制品、骨骼。其中，熊科动物犬齿3件，穿山甲属动物甲片5件，玳瑁背甲盾片212片，鸮形目鸟类脚3只，河马牙、河马牙制品；另查获被告人葛峰所有的544.45克象牙、象牙制品。前述物品均系国家重点保护的珍贵、濒危野生动物制品。

2018年8月29日，被告人苗禹通过微信向被告人葛峰出售一批抹香鲸牙齿碎料，获利3500元。2019年1月24日，公安机关在被告人苗禹住处查获抹香鲸牙23个，重5114.4克。

2018年7月25日、9月17日，被告人沈才伟通过微信先后两次以1800元每公斤的价格向被告人章浩销售河马牙两公斤，获利3600元。

2018年11月15日，被告人葛峰通过微信向吴某出售带有一颗河马牙珠子的木质手串一条，得款299元。同年11月29日，被告人葛峰通过微信向被告人常晓霞出售河马牙圈子一个，圈子上带有一个抹香鲸牙的珠子，得款100元。同年11月22日，被告人葛峰通过微信向被告人周洋出售云豹槽牙一个，得款80元。

2018年4月9日，被告人常晓霞通过微信向齐某（另案处理）出售象牙手串一个，重65.44克，价值2732.77元，得款

1800 元；同年 9 月 1 日，被告人常晓霞通过微信向齐某出售象牙佛牌一个，重 23.47 克，价值 980.11 元，得款 500 元。公安机关另在被告人常晓霞住处查获抹香鲸牙制品 2 个。

2018 年 5 月 19 日，被告人周洋通过微信以 6400 元的价格向高某出售非洲狮爪。同年 10 月 2 日，被告人周洋通过微信向高某出售一个象牙观音头制品，重 25.77 克，价值 1076.16 元，得款 688 元。同年 11 月 18 日，被告人周洋通过微信向高某出售一串象牙手串，重 50.35 克，价值 2102.62 元，得款 1400 元。

公诉机关认为，应当以非法出售珍贵、濒危野生动物制品罪追究被告人葛峰、章浩、常晓霞、周洋、苗禹、沈才伟的刑事责任。现提请法院依法惩处。

被告人葛峰对公诉机关指控的犯罪事实及罪名不持异议，表示认罪认罚。其辩护人提出如下意见：被告人葛峰没有前科劣迹，具有坦白情节，认罪悔罪态度良好，主观恶性较小，部分野生动物制品尚未销售系犯罪未遂，社会危害性相对不大，请求从宽处理并适应缓刑。

被告人章浩对公诉机关指控的犯罪事实及罪名不持异议，表示认罪认罚。其辩护人提出如下意见：被告人章浩没有前科劣迹，具有坦白情节，认罪认罚，愿意退赃退赔，悔罪态度良好，获利和社会危害性较小，请求从轻处理并适用缓刑。

被告人常晓霞对公诉机关指控的犯罪事实及罪名不持异议，表示认罪认罚。其辩护人提出如下意见：被告人常晓霞没有前科，

归案后如实供述犯罪事实，自愿认罪认罚，愿意修复生态环境，主观恶性较小，社会危害性不大，请求从宽处理并适用缓刑。

被告人周洋对公诉机关指控的犯罪事实及罪名不持异议，表示认罪认罚。其辩护人提出如下意见：被告人周洋没有前科，具有坦白情节，认罪悔罪态度良好，犯罪情节相对较轻，自愿缴纳生态修复费用，请求从轻处罚并适用缓刑。

被告人苗禹对公诉机关指控的犯罪事实及罪名不持异议，表示认罪认罚。其辩护人提出如下意见：被告人苗禹没有前科劣迹，主动退缴违法所得，认罪认罚，具有坦白情节，主观恶性不深，社会危害性不大，请求从轻处罚并适用缓刑。

被告人沈才伟对公诉机关指控的犯罪事实及罪名不持异议，表示认罪认罚。其辩护人提出如下意见：被告人沈才伟系初次犯罪，具有坦白情节，认罪认罚，主动退出违法所得，悔罪态度较好，社会危害性较小，请求从轻、减轻处罚并适用缓刑。

经审理查明,2016 年以来，被告人葛峰、章浩共同出资在江苏省徐州市八里村一民房里加工野生动物制品并对外出售，被告人葛峰主要负责购买原料、出售野生动物制品，被告人章浩主要负责加工事宜。有关具体事实如下：

2018 年 7 月 25 日、9 月 17 日，被告人沈才伟先后两次以 1800 元每公斤的价格向被告人章浩出售河马牙两公斤，获利 3600 元。

2018年8月29日，被告人苗禹向被告人葛峰出售一批抹香鲸牙齿碎料，获利3500元。

2018年11月15日，被告人葛峰以299元的价格向南京市栖霞区的吴某出售一条带有一颗河马牙珠子的木质手串。同年11月29日，被告人葛峰以100元的价格向被告人常晓霞出售一个河马牙圈子，圈子含有一个抹香鲸牙珠子。同年11月22日，被告人葛峰以80元的价格向被告人周洋出售一个云豹槽牙。

另查明：2018年4月9日，被告人常晓霞以1800元的价格向齐某（另案处理）出售一串手串，其中7颗珠子为象牙制品，经称量重65.44克（价值2726.69元）。同年9月1日，被告人常晓霞以500元的价格向齐某出售一个象牙佛牌，经称量重23.47克（价值977.93元）。

2018年5月19日，被告人周洋以6400元的价格向高某出售一个非洲狮爪。同年10月2日，被告人周洋以688元的价格向高某出售一个象牙观音头制品，经称量重25.77克（价值1073.76元）。同年11月18日，被告人周洋以1400元的价格向高某出售一串手串，其中12颗珠子为象牙制品，经称量重50.35克（价值2097.93元）。

2018年12月7日，被告人葛峰、章浩、常晓霞被公安机关抓获归案；同年12月10日，被告人周洋被公安机关抓获归案；2019年1月24日、4月25日，被告人苗禹、沈才伟分别被公安机关抓获归案。归案后，各被告人均如实供述了犯罪事实，被告

人葛峰、章浩退出违法所得 479 元，被告人常晓霞、周洋、苗禹、沈才伟分别退出违法所得 2300 元、8500 元、3500 元、3600 元。

此外，公安机关在被告人葛峰、章浩的加工点及住处等地，查获熊科动物犬齿 3 件，穿山甲属动物甲片 5 件，玳瑁背甲盾片 212 片，鸮形目鸟类脚 3 只，河马牙、河马牙制品以及被告人葛峰所有的 544.45 克象牙、象牙制品；在被告人苗禹的住处查获抹香鲸牙 23 个，合计重 5114.4 克；在被告人常晓霞住处查获抹香鲸牙制品 2 个。

经鉴定，上述案涉野生动物制品均系国家重点保护的珍贵、濒危野生动物制品。其中，熊科动物被列入 1989 年颁布的《国家重点保护野生动物名录》一级或二级，同时被列入《濒危野生动植物种国际贸易公约》（CITES 公约，2017 版）附录 I 或附录 II；穿山甲属动物被列入 1989 年颁布的《国家重点保护野生动物名录》一级，同时被列入 CITES 公约（2017 版）附录 I 或附录 II；玳瑁属于海龟科动物，被列入 1989 年颁布的《国家重点保护野生动物名录》二级，同时该科所有种被列入 CITES 公约（2017 版）附录 I；鸮形目所有种被列入 1989 年颁布的《国家重点保护野生动物名录》二级，同时该目所有种被列入 CITES 公约（2017 版）附录 I 或附录 II；河马被列入 CITES 公约（2017 版）附录 II；亚洲象被列入 1989 年颁布的《国家重点保护野生动物名录》一级，同时被列入 CITES 公约（2017 版）附录 I;非洲象被核准为国家一级重点保护动物，同时被列入 CITES 公约

（2017 版）附录 I 或附录 II；云豹、豹被列入 1989 年颁布的《国家重点保护野生动物名录》一级；抹香鲸被列入 CITES 公约（2017 版）附录 I；非洲狮被列入 CITES 公约（2017 版）附录 II。

本案审理中，南京市栖霞区人民检察院向本院提起附带民事公益诉讼，请求判令：1.葛峰承担大象损害赔偿费用 31741 元；2.葛峰、章浩连带承担穿山甲损害赔偿费用 60000 元、鸮形目鸟损害赔偿费用 2250 元、熊科动物损害赔偿费用 1000 元；3.葛峰、章浩、沈才伟连带承担河马损害赔偿费用 2500 元；4.葛峰、章浩、周洋连带承担云豹损害赔偿费用 300 元；5.周洋承担大象损害赔偿费用 4437 元、非洲狮损害赔偿费用 625 元；6.苗禹承担香鲸损害赔偿费用 3450 元；7.常晓霞承担大象损害赔偿费用 5183 元；8.各被告共同承担专家鉴定费 7000 元；9.各被告在国家媒体上公开赔礼道歉。前述费用现已赔偿在案。

上述事实，被告人葛峰、章浩、常晓霞、周洋、苗禹、沈才伟供认不讳。本案事实，另有常住人口信息表、接处警登记表、受案登记表、立案决定书、抓获经过、接受证据清单、扣押决定书、扣押清单、微信聊天截图等书证，证人吴某、张某 1、张某 2、高某、文某、齐某、于某的证言，勘验、检查、辨认笔录，国家林业局森林公安司法鉴定中心出具的物证鉴定书，视听资料、电子数据等证据证实。上述证据均经庭审质证，合法有效，具有证明效力。

本院认为,珍贵、濒危野生动物制品的价值，依照国家野生动物保护主管部门的规定核定；核定价值低于实际交易价格的，以实际交易价格认定。依据《国家林业局关于发布破坏野生动物资源刑事案件中涉及走私的象牙及其制品价值标准的通知》（林濒发[2001]234 号）规定，一根象牙的价值为 25 万元；对于无法确定是否属一根象牙切割或者雕刻成的象牙块或象牙制品，应根据其重量来核定，单价为 41667 元/千克。

根据《中华人民共和国刑法》第三百四十一条第一款、《最高人民法院、最高人民检察院关于执行<中华人民共和国刑法>确定罪名的补充规定（七）》有关规定，被告人葛峰、章浩、常晓霞、周洋非法收购、出售国家重点保护的珍贵、濒危野生动物制品，被告人苗禹、沈才伟非法出售国家重点保护的珍贵、濒危野生动物制品，均构成危害珍贵、濒危野生动物罪。被告人葛峰、章浩共同故意实施危害珍贵、濒危野生动物犯罪行为，系共同犯罪。被告人葛峰、章浩、苗禹的部分犯罪行为已经着手实行，由于意志以外的原因而未得逞，系犯罪未遂，可以比照既遂犯从轻或者减轻处罚。被告人葛峰、章浩、常晓霞、周洋、苗禹、沈才伟具有坦白情节，依法均可以从轻处罚，有关辩护意见予以采纳。各被告人主动退赃退赔，本院酌情从轻处罚。据此，根据本案犯罪事实、性质、情节和对于社会的危害程度，结合各被告人的认罪悔罪表现，依照《中华人民共和国刑法》第三百四十一条第一款，第二十五条第一款，第二十三条，第六十七条第三款，第七

十二条第一款、第三款，第七十三条第二款、第三款，第五十二条，第六十四条之规定，判决如下：

一、被告人葛峰犯危害珍贵、濒危野生动物罪，判处有期徒刑二年，缓刑三年，并处罚金人民币二万元。

（缓刑考验期限，从判决确定之日起计算。罚金于判决生效之日起一个月内缴纳。）

被告人章浩犯危害珍贵、濒危野生动物罪，判处有期徒刑二年，缓刑三年，并处罚金人民币二万元。

（缓刑考验期限，从判决确定之日起计算。罚金于判决生效之日起一个月内缴纳。）

被告人常晓霞犯危害珍贵、濒危野生动物罪，判处有期徒刑一年三个月，缓刑一年六个月，并处罚金人民币七千元。

（缓刑考验期限，从判决确定之日起计算。罚金于判决生效之日起一个月内缴纳。）

被告人周洋犯危害珍贵、濒危野生动物罪，判处有期徒刑一年六个月，缓刑二年，并处罚金人民币一万元。

（缓刑考验期限，从判决确定之日起计算。罚金于判决生效之日起一个月内缴纳。）

被告人苗禹犯危害珍贵、濒危野生动物罪，判处有期徒刑一年三个月，缓刑一年六个月，并处罚金人民币七千元。

（缓刑考验期限，从判决确定之日起计算。罚金于判决生效之日起一个月内缴纳。）

被告人沈才伟犯危害珍贵、濒危野生动物罪，判处有期徒刑一年三个月，缓刑一年六个月，并处罚金人民币七千元。

（缓刑考验期限，从判决确定之日起计算。罚金于判决生效之日起一个月内缴纳。）

二、被告人葛峰、章浩、常晓霞、周洋、苗禹、沈才伟所退违法所得予以没收，上缴国库。扣押在案的珍贵、濒危野生动物制品予以没收。

如不服本判决,可在接到判决书的第二日起十日内，通过本院或者直接向江苏省南京市中级人民法院提出上诉。书面上诉的，应当提交上诉状正本一份，副本二份。

审　判　长　　高　伟

审　判　员　　安洪强

审　判　员　　赵　佳

人民陪审员　　张苏莉

人民陪审员　　杨杏年

人民陪审员　　王　勇

人民陪审员　　陈　刚

二〇二一年三月二十二日

书　记　员　　吴双香
