# Supplementary material for: China’s Legal Protection System for Pangolins: Past, Present, and Future
Source: Animals (Basel). 2025 Aug 18;15(16):2422. doi: 10.3390/ani15162422 (PMC12383201; doi:10.3390/ani15162422)
Supplement: Supplementary file 1 [file animals-15-02422-s001.zip › Supplementary Material S4-Full Text of Judgments in Pangolin-Related Public Interest Litigation Cases in China/【22】吴克银非法收购、运输、出售珍贵、濒危野生动物、珍贵、濒危野生动物制品罪一审刑事判决书.pdf]

吴克银非法收购、运输、出售珍贵、濒危野生动物、珍贵、濒危野生动物制品罪一审刑事判决书

苏州市姑苏区人民法院

刑 事 附 带 民 事 判 决 书

(2020)苏0508刑初1159号

公诉机关暨刑事附带民事公益诉讼起诉人：江苏省宜兴市人民检察院。

被告人暨刑事附带民事公益诉讼被告：吴克银，1965年1月22日生，汉族，无业，住安徽省芜湖市南陵县。曾因犯非法收购、出售珍贵、濒危野生动物罪，于2016年10月26日被江苏省宜兴市人民法院判处有期徒刑一年，缓刑二年，并处罚金人民币三万元。因涉嫌非法收购、出售珍贵、濒危野生动物、珍贵、濒危野生动物制品犯罪，于2020年5月28日被刑事拘留，同年6月27日被监视居住，同年8月11日被逮捕。现羁押于宜兴市看守所。

指定辩护人：林国征、宜兴市法律援助中心律师。

江苏省宜兴市人民检察院以宜检刑诉〔2020〕1532号起诉书指控被告人吴克银犯非法收购、出售珍贵、濒危野生动物、珍贵、濒危野生动物制品罪，向本院提起公诉，另以宜检刑附民公诉〔2020〕9号刑事附带民事公益诉讼起诉书向本院提起刑事附带民事公益诉讼。本院受理后依法适用普通程序组成合议庭，于同年3月12日公开开庭审理了本案。江苏省宜兴市人民检察院

指派检察员莫修龙、检察官助理舒畅出庭支持公诉并代表该院就刑事附带民事公益诉讼出庭履行职责，被告人暨刑事附带民事公益诉讼被告吴克银及其指定辩护人林国征到庭参加诉讼。本案现已审理终结。

江苏省宜兴市人民检察院指控：1.2019年5月至2019年9月期间，被告人吴克银在明知穿山甲属于国家重点保护野生动物的情况下，仍从徐某(另案处理)处收购穿山甲活体5只，后全部出售给程某(另案处理)等人。2.2019年8月，被告人吴克银在明知黑熊属于国家重点护野生动物的情况下，向黄某(另案处理)出售熊掌1只。2020年5月20日，被告人吴克银被公安机关抓获归案。被告人吴克银自愿认罪认罚。

为证实上述指控，公诉机关提供了相关证据，认为被告人吴克银违反野生动物保护法规，非法收购、出售国家重点保护的珍贵、濒危野生动物、珍贵、濒危野生动物制品，其行为触犯了《中华人民共和国刑法》第三百四十一条第一款的规定应当以非法收购、出售珍贵、濒危野生动物、珍贵、濒危野生动物制品罪追究其刑事责任。被告人吴克银自愿认罪认罚，可以依法从宽处理。

刑事附带民事公益诉讼起诉人江苏省宜兴市人民检察院向本院提出诉讼请求：1.请求判令吴克银支付国家野生动物资源损失费共计人民币200000元；2.请求判令吴克银在《宜兴日报》上，就其非法收购、出售珍贵、濒危野生动物的行为赔礼道歉。事实和理由：同刑事指控的事实外，经宜兴市森林警察大队出具

价值评估意见认定，在 2020 年 6 月 3 日之前，穿山甲属所有物种的每只基准价值为人民币 40000 元。本案涉案珍贵、濒危野生动物价值人民币 200000 元。被告吴克银违反国家野生动物保护法规，非法收购、出售国家重点保护动物穿山甲，造成了国家野生动物资源损失和生态环境破坏，损害了社会公共利益。根据《中华人民人民共和国侵权责任法》第二条、第十五条，《中华人民共和国环境保护法》第六十四条，《最高人民法院关于审理环境民事公益诉讼案件适用法律若干问题的解释》第十八条的规定，应承担相应的法律责任。

被告人暨刑事附带民事公益诉讼被告吴克银对公诉机关指控的罪名与事实无异议，表示自愿认罪，请求从轻处罚。对于刑事附带民事公益诉讼赔偿部分，其表示没有能力赔偿。

指定辩护人对公诉机关指控的罪名不持异议，提出以下从轻处罚的意见：一、认罪认罚，庭审中如实供述，应当从轻处罚。二、不是犯意提起者，因他人要购买其才收购、出售。三、法律意识淡薄，获利较少。请求对被告人从轻处罚。

经审理查明，2019 年 5 月至同年 9 月期间，被告人吴克银在明知穿山甲属于国家重点保护野生动物的情况下，仍从徐某(另案处理)处收购穿山甲活体 5 只，并全部出售给开设饭店的程某(另案处理)等人供食客食用，共计非法获利人民币 60000 元左右。被告人吴克银还在出售过程中为程某等人宰杀上述穿山甲。2019 年 8 月，被告人吴克银在明知黑熊属于国家重点护野生动

物的情况下，向黄某(另案处理)出售熊掌 1 只供人食用，非法获利人民币 3000 元。

被告人吴克银收购、出售上述穿山甲时，穿山甲属动物均被列入《国家重点保护野生动物名录》二级，同时均被列入《濒危野生动植物种国际贸易公约》(CITES)附录Ⅰ或Ⅱ；熊科动物在我国分布的均被列入《国家重点保护野生动物名录》，同时均被列入《濒危野生动植物种国际贸易公约》(CITES)附录Ⅰ或Ⅱ。

2020 年 5 月 20 日，被告人吴克银被公安机关抓获归案，并进行新冠病毒医学隔离观察，5 月 28 日被刑事拘留，6 月 27 日被监视居住，8 月 11 日被逮捕。被告人吴克银在审查起诉阶段自愿签署认罪认罚具结书，表示认罪认罚。

另查明，被告人吴克银长期从事动物经营活动，为饭店提供大傀、蛇、穿山甲等动物或动物制品，曾因犯非法收购、出售珍贵、濒危野生动物罪，于 2016 年 10 月 26 日被江苏省宜兴市人民法院判处有期徒刑一年，缓刑二年，并处罚金人民币三万元。

江苏省宜兴市人民检察院发现涉案违法行为后，经诉前公告程序，公告期满后没有适格主体提起民事公益诉讼。因社会公共利益仍处于受损害状态，故向本院提出刑事附带民事公益诉讼，提出前述诉讼请求。

就刑事部分的案件事实，被告人吴克银供认不讳，且有公诉机关提供的被告人吴克银的供述与辩解及辨认动物的照片，证人徐某、程某、黄某、殷某、朱某、傅某等人的证言及辨认动物的

照片、微信交易记录、农业银行卡交易明细、扣押决定书、扣押清单、提取笔录、通过图片辨别野生动物种类的专家意见、宜兴市公安局高塍派出所出具的情况说明、人口信息、刑事判决书、认罪认罚具结书等证据予以证实。上述证据均经法庭举证、质证，证据来源合法、内容客观，具有证明效力，本院予以确认。

就刑事附带民事部分的案件事实，公益诉讼起诉人除与刑事证据相同外，另提交宜兴市森林警察大队出具的价值评估意见及正义网的公告截图，证明每只穿山甲基准价是 4 万元及本案民事公益诉讼起诉前履行了公告程序。对上述民事部分补充的证据，被告吴克银无异议，本院予以确认。

本院认为，被告人吴克银违反国家野生动物保护法规，非法收购、出售国家重点保护的珍贵、濒危野生动物穿山甲五只、非法出售国家重点保护的珍贵、濒危野生动物制品熊掌一只，其行为已构成危害珍贵、濒危野生动物罪，依法处五年以下有期徒刑或者拘役，并处罚金。公诉机关的指控成立。被告人吴克银认罪认罚，依法从宽处理。对辩护人认为被告人有认罪认罚情节予以从轻处罚的辩护意见予以采纳。被告人吴克银长期从事动物经营活动，为饭店提供动物或动物制品，并曾因非法收购、出售珍贵、濒危野生动物罪受过刑事处罚，本案中其为多家饭店提供活体穿山甲、熊掌并为饭店经营者宰杀穿山甲供人食用，不仅破坏了野生动物资源，还因滥食野生动物的行为会对公众健康构成不可测

的威胁，其犯罪行为的社会危害性大，对辩护人认为被告人不是犯意提起者的意见不予采纳。

就刑事附带民事公益诉讼起诉人请求判令吴克银支付国家野生动物资源损失费共计人民币200000元，按每只被被告吴克银收购、出售并宰杀的穿山甲基准价4万元计算，本院予以支持。因被告人的违法所得属于破坏野生动物资源所获得的非法利益，与本案野生动物资源损害赔偿责任款项具有同质属性。故对被告人违法所得的追缴应优先用于野生动物资源损失赔偿。对于要求被告吴克银承担公开赔礼道歉的民事责任，本院也予以支持。

据此，本院依照《中华人民共和国刑法》第三百四十一条第一款、第五十二条、第五十三条、第六十四条、第六十五条、《中华人民共和国刑事诉讼法》第十五条、第二百零一条、《最高人民法院最高人民检察院关于执行〈中华人民共和国刑法〉确定罪名的补充规定（七）》、《中华人民共和国民事诉讼法》第五十五条第二款、《最高人民法院关于审理环境民事公益诉讼案件适用法律若干问题的解释》第一条、第十八条之规定，判决如下：

一、被告人吴克银犯危害珍贵、濒危野生动物罪，判处有期徒刑二年，并处罚金人民币二万元(刑期从判决执行之日起计算，判决执行以前先行羁押的，羁押一日折抵刑期一日，2020年5月20日至6月26日被隔离观察及刑事拘留的38日折抵刑期38日，2020年6月27日至8月10日被监视居住的45日折抵刑期

23日，刑期自2020年8月11日起至2022年6月10日止；罚金自判决生效次日起三十日内缴纳，并上缴国库）。

二、被告人吴克银的违法所得人民币六万三千元，予以追缴，并用于赔偿国家野生动物资源损失。

三、被告人吴克银在本判决生效之日起十五日内，就其危害国家重点保护的珍贵、濒危野生动物的行为在《宜兴日报》或其他当地市级以上媒体公开赔礼道歉。

四、被告人吴克银本判决生效之日起十五日内支付第二项抵扣余下的国家野生动物资源损失费人民币十三万七千元。

如未按本判决指定的期限履行给付义务，应当依照《中华人民共和国民事诉讼法》第二百五十三条的规定，加倍支付迟延履行期间的债务利息。

如不服本判决，可在接到判决书的第二日起十日内，通过本院或者直接向江苏省南京市中级人民法院提出上诉。书面上诉的，应提交上诉状正本一份，副本二份。

审　判　长　　陈　勇

人民陪审员　　俞静玲

人民陪审员　　倪　青

二〇二一年三月二十二日

书　记　员　　袁小英
