# Supplementary material for: China’s Legal Protection System for Pangolins: Past, Present, and Future
Source: Animals (Basel). 2025 Aug 18;15(16):2422. doi: 10.3390/ani15162422 (PMC12383201; doi:10.3390/ani15162422)
Supplement: Supplementary file 1 [file animals-15-02422-s001.zip › Supplementary Material S4-Full Text of Judgments in Pangolin-Related Public Interest Litigation Cases in China/【23】余龙辉非法收购、运输、出售珍贵、濒危野生动物、珍贵、濒危野生动物制品罪一审刑事判决书.pdf]

余龙辉非法收购、运输、出售珍贵、濒危野生动物、珍贵、濒危野生动物制品罪一审刑事判决书

福建省漳平市人民法院

刑 事 附 带 民 事 判 决 书

（2020）闽 0881 刑初 278 号

公诉机关暨附带民事公益诉讼起诉人：福建省漳平市人民检察院。

被告人暨附带民事公益诉讼被告:余龙辉，男，1974 年 4 月 12 日出生，汉族，小学文化程度，经商，出生地和户籍地福建省漳平市，住福建省漳平市。因涉嫌非法收购、出售珍贵、濒危野生动物、珍贵、濒危野生动物制品罪，于 2020 年 2 月 23 日被漳平市公安局刑事拘留，同年 3 月 30 日经漳平市人民检察院批准逮捕，次日由漳平市公安局执行逮捕。现羁押于漳平市看守所。

辩护人陈海汀，福建博平律师事务所律师。

漳平市人民检察院以漳检检一刑诉[2020]240 号起诉书指控被告人余龙辉犯非法收购、出售珍贵、濒危野生动物、珍贵、濒危野生动物制品罪，于 2020 年 11 月 19 日向本院提起公诉。同时，公益诉讼起诉人漳平市人民检察院以漳检检四刑附民公诉[2020]7 号刑事附带民事公益诉讼起诉书向本院提起附带民事公益诉讼。经查，漳平市人民检察院于 2020 年 4 月 3 日公告了准备提起刑事附带民事公益诉讼的相关情况，公告期内没有法律规定的机关和组织提起诉讼。本院依法组成合议庭，于 2020 年 12

月24日公开开庭合并审理了本案。漳平市人民检察院指派检察员郭佳旺出庭支持公诉，刑事附带民事公益诉讼起诉人指派检察员游修林出庭履行职务。被告人暨附带民事公益诉讼被告余龙辉、辩护人陈海汀到庭参加诉讼。本案现已审理终结。

公诉机关漳平市人民检察院指控：2018年至2019年期间，被告人余龙辉违反野生动物保护法规，在未经野生动物管理部门批准的情况下，多次向他人非法收购、出售国家保护野生动物及制品穿山甲7只、平胸龟23只、鬣羚制品，价值405880元，其中：

1、2018年1月19日，被告人余龙辉在未经野生动物管理部门批准的情况下，以每斤1200元的价格向李某1（另案处理）出售1只重6.57斤的穿山甲冻体以及其它野生动物制品，得款7884元。经福建省鼎力司法鉴定中心鉴定：该穿山甲为国家二级野生保护动物—穿山甲（国家林业和草原局2020年第12号公告穿山甲为国家一级野生保护动物），价值40000元。

2、2018年农历3、4月份期间，被告人余龙辉在未经野生动物管理部门批准的情况下，以每斤700元的价格交付陈某1（另案处理）寄卖1只重2.8斤的穿山甲冻体，后陈某1将该只穿山甲冻体归还余龙辉。经福建省鼎力司法鉴定中心鉴定：该穿山甲为国家二级野生保护动物—穿山甲，价值40000元。

3、2018年10月11日，被告人余龙辉在未经野生动物管理部门批准的情况下，以每斤1200元的价格向詹某（另案处理）

收购 3 只重总重 4.2 斤的鹰嘴龟，付款 5040 元。2019 年 6 月 14 日，以每斤 1500 元的价格向詹某出售 2 只总重 2.03 斤的鹰嘴龟，得款 3045 元，经福建省鼎力司法鉴定中心鉴定：该 5 只鹰嘴龟为国家一级野生保护动物—平胸龟（大头龟、鹰嘴龟），价值 25000 元。2019 年初，被告人余龙辉以每斤 1350 元的价格向詹某出售 1 只重 5.6 斤的穿山甲冻体，得款 7560 元，经福建省鼎力司法鉴定中心鉴定：该穿山甲为国家二级野生保护动物—穿山甲，价值 40000 元。2018 年 10 月至 2019 年 11 月期间，被告人余龙辉在未经野生动物管理部门批准的情况下，先后 6 次向詹某出售山羊制品，共得款 10880 元，经国家林业局森林公安司法鉴定中心鉴定，该山羊制品为鬣羚，属国家二级保护野生动物。

4、2019 年 3 月 29 日，被告人余龙辉在未经野生动物管理部门批准的情况下，以每斤 1250 元的价格向陈某 2（另案处理）收购 1 只重 9.8 斤的穿山甲，付款 12250 元。经福建省鼎力司法鉴定中心鉴定：该穿山甲为国家二级野生保护动物—穿山甲，价值 40000 元。

5、2019 年 4 月 11 日至同年 10 月 31 日期间，被告人余龙辉在未经野生动物管理部门批准的情况下，先后 5 次以每斤 1400-1500 元的价格向陈某 3（已判刑）出售 14 只鹰嘴龟，得款 29634 元。经福建省鼎力司法鉴定中心鉴定：该 14 只鹰嘴龟为国家一级野生保护动物—平胸龟（大头龟、鹰嘴龟），价值 70000 元。

6、2019年5月17日，被告人余龙辉在未经野生动物管理部门批准的情况下，以每斤800元的价格向黄某（另案处理）出售1只重12.8斤的穿山甲冻体，得款11550元。经福建省鼎力司法鉴定中心鉴定：该穿山甲为国家二级野生保护动物—穿山甲，价值40000元。

7、2019年7月18日，被告人余龙辉在未经野生动物管理部门批准的情况下，以每斤650元的价格向叶某1（已判刑）出售1只重5.2斤的穿山甲冻体，得款3380元。经福建省鼎力司法鉴定中心鉴定：该穿山甲为国家二级野生保护动物—穿山甲，价值40000元。

8、2019年7月18日，被告人余龙辉在未经野生动物管理部门批准的情况下，以每斤1600元的价格向高某1（另案处理）出售2只总重1.28斤的鹰嘴龟，得款2048元。经福建省鼎力司法鉴定中心鉴定：该2只鹰嘴龟为国家一级野生保护动物—平胸龟（大头龟、鹰嘴龟），价值10000元。

9、2019年9月份，被告人余龙辉在未经野生动物管理部门批准的情况下，以每斤1650元的价格向汪某（已判刑）出售2只重分别为0.6斤、0.98斤的鹰嘴龟，得款2607元。经福建省鼎力司法鉴定中心鉴定：该2只鹰嘴龟为国家一级野生保护动物—平胸龟（大头龟、鹰嘴龟），价值10000元。

10、2019年10月25日，被告人余龙辉在未经野生动物管理部门批准的情况下，以每斤1500元的价格向叶某2（已判刑）

出售1只重12斤的穿山甲冻体以及其它野生动物制品，叶某2在其与李某3合股经营的漳平“湘满楼”饭店加工给李某2（已判刑）宴请他人食用，之后，李某2付款给叶某220000元。经福建省鼎力司法鉴定中心鉴定：该穿山甲为国家二级野生保护动物—穿山甲，价值40000元。

公诉机关当庭提供了有关证据予以证实其所指控的犯罪事实，认为被告人余龙辉违反野生动物保护法规，在未经野生动物管理部门批准的情况下，非法收购、出售国家保护野生动物及其制品穿山甲7只、平胸龟23只及鬣羚制品，价值405880元，情节特别严重，其行为触犯了《中华人民共和国刑法》第三百四十一条第一款，犯罪事实清楚，证据确实、充分，应当以非法收购、出售珍贵、濒危野生动物、珍贵、濒危野生动物制品罪追究其刑事责任。被告人余龙辉在公诉期间自愿认罪认罚，依照《中华人民共和国刑事诉讼法》第十五条的规定，可从宽处理。建议判处被告人余龙辉有期徒刑十二年六个月至十三年六个月，并处罚金400000元。

附带民事公益诉讼起诉人漳平市人民检察院向本院提出诉讼请求：1、判令被告余龙辉赔偿国家野生动物资源损失折合245880元；2、判令被告余龙辉与叶某1、叶某2、汪某、陈某3连带赔偿野生动物资源损失折合160000元；3、判令被告余龙辉在漳平市（县级以上新闻媒体赔礼道歉。案件审理过程中，附带民事公益诉讼起诉人变更第1、第2项诉讼请求为：1、判令被

告余龙辉赔偿野生动物资源损失折合 285000 元；2、判令被告余龙辉与陈某 3 连带赔偿野生动物资源损失折合 70000 元。事实和理由：2018 年 1 月至 2019 年 10 月，被告余龙辉，未经野生动物管理部门批准，非法收购、出售国家保护野生动物及制品穿山甲 7 只、鹰嘴龟 23 只及鬣羚制品。具体：1、2018 年 1 月 19 日，被告余龙辉未经批准许可，以每斤 1200 元的价格向李某 1（另案处理）出售 1 只重 6.57 斤的穿山甲冻体，得款 7884 元。2、2018 年农历 3、4 月份期间，被告余龙辉在未经批准许可，以每斤 700 元的价格向陈某 1（另案处理）寄卖 1 只重 2.8 斤的穿山甲冻体，后陈某 1 将该只穿山甲冻体归还余龙辉。3、2018 年 10 月 11 日，被告余龙辉未经批准许可，以每斤 1200 元的价格向詹某（另案处理）收购 3 只总重 4.2 斤的鹰嘴龟，付款 5040 元；2019 年 6 月 14 日，以每斤 1500 元的价格向詹某出售 2 只总重 2.03 斤的鹰嘴龟，得款 3045 元；2019 年初，被告余龙辉以每斤 1350 元的价格向詹某出售 1 只重 5.6 斤的穿山甲冻体，得款 7560 元；2018 年 10 月至 2019 年 11 月期间，被告余龙辉未经批准许可，先后 6 次向詹某出售山羊制品，经国家林业局森林公安司法鉴定中心鉴定，该山羊制品ＤＮＡ为鬣羚所有，属国家二级保护野生动物，可以确定最起码存在至少一只鬣羚被非法收购、出售。4、2019 年 3 月 29 日，被告余龙辉未经批准许可，以每斤 1250 元的价格向陈某 2（另案处理）收购 1 只重 9.8 斤的穿山甲，付款 12250 元。5、2019 年 4 月 11 日至同年 10 月 31 日期间，被

告余龙辉未经批准许可，先后5次以每斤1400-1500元的价格向陈某3（已另案处理，已判决）出售14只鹰嘴龟，得款29634元。6、2019年5月17日，被告余龙辉未经野生动物管理部门批准的情况下，以每斤800元的价格向黄某（另案处理）出售1只重12.8斤的穿山甲冻体，得款11550元。7、2019年7月18日，被告余龙辉未经批准许可，以每斤650元的价格向叶某1（已另案处理，已判决）出售1只重5.2斤的穿山甲冻体，得款3380元。8、2019年7月18日，被告余龙辉未经批准许可，以每斤1600元的价格向高某1（另案处理）出售2只总重1.28斤的鹰嘴龟，得款2080元。9、2019年9月份，被告余龙辉未经批准许可，以每斤1650元的价格向汪某（已另案处理，已判决）出售2只重分别为0.6斤、0.98斤的鹰嘴龟，得款2607元。10、2019年10月25日，被告余龙辉未经批准许可，以每斤1500元的价格向叶某2（已另案处理，已判决）出售1只重12斤的穿山甲冻体，得款18000元。上述穿山甲、鹰嘴龟，经福建鼎力司法鉴定中心鉴定：穿山甲为国家二级野生保护动物，每只价值40000元；鹰嘴龟为国家一级野生保护动物，每只价值5000元。上述7只穿山甲、23只鹰嘴龟和1只鬣羚，总价值计445000元。认为，被告余龙辉未经批准许可，非法出售、收购国家保护的珍贵、濒危野生动物穿山甲、鹰嘴龟及鬣羚，其行为破坏了野生动物资源，影响野生动物生态平衡，损害了国家和社会公共利益，造成国家野生动物资源损失，价值达445000元，违反了《中华

人民共和国野生动物保护法》第三条第一款“野生动物资源属于国家所有”、第二十七条“禁止出售、购买、利用国家重点保护野生动物及其制品”的规定。根据《中华人民共和国侵权责任法》第四条第一款“侵权人因同一行为应当承担行政责任或者刑事责任的，不影响依法承担侵权责任”的规定，被告余龙辉在承担刑事责任的同时，仍应承担民事赔偿责任，赔偿国家野生动物资源损失。此前，本院已分别对叶某1、叶某2、汪某及陈某3、李某2非法收购、出售珍贵、濒危野生动物的行为另案提起刑事附带民事公益诉讼，诉请赔偿野生动物资源损失，漳平市人民法院分别以（2020）闽0881刑初159、180、197、209、210号刑事附带民事判决书分别判令叶某1赔偿40000元、叶某2赔偿40000元、李某2与叶某2共同赔偿40000元、汪某赔偿10000元、陈某3赔偿70000元，叶某1、叶某2及汪某已履行了赔偿义务，剩余陈某3尚未履行赔偿义务。根据《中华人民共和国侵权责任法》第八条第一款“二人以上共同实施侵权，造成他人损害的，应承担连带责任”，第十五条“承担侵权责任的方式主要有……（六）赔偿损失；（七）赔礼道歉；……”、第二款“以上承担侵权责任的方式，可以单独适用，也可以合并适用”的规定，被告余龙辉除应单独赔偿野生动物资源损失285000元外，还应与陈某3连带赔偿野生动物资源损失70000元及承担赔礼道歉责任。综上，被告余龙辉非法收购、出售珍贵、濒危野生动物及制品，其行为破坏了生态环境和生态资源，损害了国家和社会公共

利益，触犯了《中华人民共和国刑法》第三百四十一条第一款的规定，依法应当追究刑事责任，本院已向你院提起公诉。本案经正义网公告，在公告期间没有法律规定的机关和有关组织提起诉讼，现根据《中华人民共和国刑事诉讼法》第一百零一条第二款、《中华人民共和国民事诉讼法》第五十五条、《最高人民法院、最高人民检察院＜关于检察公益诉讼案件适用法律若干问题的解释＞》第二十条的规定，特提起附带民事公益诉讼，请依法裁判。

针对诉求，公益诉讼起诉人提供了立案决定书、公告；被告余龙辉的户籍证明、漳平市市场监督管理局出具的被告余龙辉个体工商户基本信息、营业执照；漳平市公安局提取的微信聊天记录截图、交易账单截图等书证、被告余龙辉的供述与辩解、辨认笔录；证人李某 1、陈某 1、詹某、陈某 2、陈某 3、黄某、叶某 1、叶某 2、高某 1、汪某及曾某等人的证言、漳平市公安局制作的现场勘验笔录、现场指认笔录、辨认笔录、现场照片；福建鼎力司法鉴定中心出具的司法鉴定意见书、国家林业局森林公安司法鉴定中心出具的物证鉴定书；漳平市人民法院（2020）闽 0881 刑初 159、180、197、209、210 号刑事附带民事判决书、漳平市人民检察院起诉书等证据材料。

2020 年 2 月 22 日，被告人余龙辉被漳平市公安局传唤到案。

被告人余龙辉的辩护人及其本人对公诉机关指控的犯罪事实和罪名均无异议。同时，辩护人认为，被告人余龙辉的行为虽

属犯罪情节特别严重，但属情节特别严重中相对较轻的情形；被告人余龙辉签署了认罪认罚具结书，自愿认罪认罚，确有悔罪行为；被告人余龙辉愿意缴交罚金和违法所得；被告人余龙辉愿意赔偿国家野生动物资源损失；被告人余龙辉是初犯。综上所述，虽然被告人余龙辉的行为构成非法收购、出售珍贵、濒危野生动物、珍贵、濒危野生动物制品罪，但其犯罪情节有相对较轻的情形，且自愿认罪认罚，愿意缴交罚金和违法所得及赔偿国家野生动物资源损失，是初犯，有多个从轻、从宽处罚的情节，恳请对被告人余龙辉判处较轻的刑罚，建议尽可能对其判处公诉机关量刑建议确定的最低刑罚甚至更轻的刑罚。针对公益诉讼起诉人的诉请及所列举的事实与依据，附带民事公益诉讼被告余龙辉亦无异议。

经审理查明，2018 年至 2019 年期间，被告人余龙辉未经野生动物管理部门的批准，多次向他人非法收购、出售国家保护野生动物及制品穿山甲 7 只、平胸龟 23 只、鬣羚制品，价值 405880 元，其中：1、2018 年 1 月 19 日，被告人余龙辉在未经野生动物管理部门批准的情况下，以每斤 1200 元的价格向李某 1（另案处理）出售 1 只重 6.57 斤的穿山甲冻体以及其它野生动物制品，得款 7884 元。经福建省鼎力司法鉴定中心鉴定：在实施犯罪行为时，该穿山甲为国家二级野生保护动物—穿山甲（国家林业和草原局 2020 年第 12 号公告穿山甲为国家一级野生保护动物），价值 40000 元。2、2018 年农历 3、4 月份期间，被告人

余龙辉在未经野生动物管理部门批准的情况下，以每斤 700 元的价格向陈某 1（另案处理）寄卖 1 只重 2.8 斤的穿山甲冻体，后陈某 1 将该只穿山甲冻体归还余龙辉。经福建省鼎力司法鉴定中心鉴定：在实施犯罪行为时，该穿山甲为国家二级野生保护动物—穿山甲，价值 40000 元。3、①2018 年 10 月 11 日，被告人余龙辉在未经野生动物管理部门批准的情况下，以每斤 1200 元的价格向詹某（另案处理）收购 3 只重总重 4.2 斤的鹰嘴龟，付款 5040 元。2019 年 6 月 14 日，以每斤 1500 元的价格向詹某出售 2 只总重 2.03 斤的鹰嘴龟，得款 3045 元，经福建省鼎力司法鉴定中心鉴定：该 5 只鹰嘴龟为国家一级野生保护动物—平胸龟（大头龟、鹰嘴龟），与国家一级野生保护动物四爪陆龟属同个纲和目，价值 25000 元。②2019 年初，被告人余龙辉以每斤 1350 元的价格向詹某出售 1 只重 5.6 斤的穿山甲冻体，得款 7560 元，经福建省鼎力司法鉴定中心鉴定：在实施犯罪行为时，该穿山甲为国家二级野生保护动物—穿山甲，价值 40000 元。③2018 年 10 月至 2019 年 11 月期间，被告人余龙辉在未经野生动物管理部门批准的情况下，先后 6 次向詹某出售山羊制品，共得款 10880 元，经国家林业局森林公安司法鉴定中心鉴定，该山羊制品为鬣羚，属国家二级保护野生动物。4、2019 年 3 月 29 日，被告人余龙辉在未经野生动物管理部门批准的情况下，以每斤 1250 元的价格向陈某 2（另案处理）收购 1 只重 9.8 斤的穿山甲，付款 12250 元。经福建省鼎力司法鉴定中心鉴定：在实施犯罪行为时，

该穿山甲为国家二级野生保护动物—穿山甲，价值 40000 元。5、2019 年 4 月 11 日至同年 10 月 31 日期间，被告人余龙辉在未经野生动物管理部门批准的情况下，先后 5 次以每斤 1400-1500 元的价格向陈某 3（已判刑）出售 14 只鹰嘴龟，得款 29634 元。经福建省鼎力司法鉴定中心鉴定：该 14 只鹰嘴龟为国家一级野生保护动物—平胸龟（大头龟、鹰嘴龟），与国家一级野生保护动物四爪陆龟属同个纲和目，价值 70000 元。6、2019 年 5 月 17 日，被告人余龙辉在未经野生动物管理部门批准的情况下，以每斤 800 元的价格向黄某（另案处理）出售 1 只重 12.8 斤的穿山甲冻体，得款 11550 元。经福建省鼎力司法鉴定中心鉴定：在实施犯罪行为时，该穿山甲为国家二级野生保护动物—穿山甲，价值 40000 元。7、2019 年 7 月 18 日，被告人余龙辉在未经野生动物管理部门批准的情况下，以每斤 650 元的价格向叶某 1（已判刑）出售 1 只重 5.2 斤的穿山甲冻体，得款 3380 元。经福建省鼎力司法鉴定中心鉴定：在实施犯罪行为时，该穿山甲为国家二级野生保护动物—穿山甲，价值 40000 元。8、2019 年 7 月 18 日，被告人余龙辉在未经野生动物管理部门批准的情况下，以每斤 1600 元的价格向高某 1（另案处理）出售 2 只总重 1.28 斤的鹰嘴龟，得款 2048 元。经福建省鼎力司法鉴定中心鉴定：该 2 只鹰嘴龟为国家一级野生保护动物—平胸龟（大头龟、鹰嘴龟），与国家一级野生保护动物四爪陆龟属同个纲和目，价值 10000 元。9、2019 年 9 月份，被告人余龙辉在未经野生动物管理部门

批准的情况下，以每斤 1650 元的价格向汪某（已判刑）出售 2 只重分别为 0.6 斤、0.98 斤的鹰嘴龟，得款 2607 元。经福建省鼎力司法鉴定中心鉴定：该 2 只鹰嘴龟为国家一级野生保护动物—平胸龟（大头龟、鹰嘴龟），与国家一级野生保护动物四爪陆龟属同个纲和目，价值 10000 元。10、2019 年 10 月 25 日，被告人余龙辉在未经野生动物管理部门批准的情况下，以每斤 1500 元的价格向叶某 2（已判刑）出售 1 只重 12 斤的穿山甲冻体以及其它野生动物制品，叶某 2 在其与李某 3 合股经营的漳平“湘满楼”饭店加工给李某 2（已判刑）宴请他人食用，之后，李某 2 付款给叶某 220000 元（其中穿山甲收取 18000 元）。经福建省鼎力司法鉴定中心鉴定：在实施犯罪行为时，该穿山甲为国家二级野生保护动物—穿山甲，价值 40000 元。

上述事实，被告人余龙辉在开庭审理过程亦无异议，且有现场查扣的羚羊制品物证；被告人余龙辉与李某 1、陈某 1、詹某、陈某 2、陈某 3 等人的微信转账记录、漳平市林业局的证明等书证；证人叶某 2、李某 2、李某 3、陈某 4、高某 2 等 29 人证人证言；被告人余龙辉的供述与辩解；福建鼎力司法鉴定中心出具的司法鉴定意见书、国家林业局森林公安鉴定中心出具的物证鉴定书；现场勘验笔录、指认笔录、辨认笔录、现场照片及视听资料光盘 2 张等证据证实，足以认定。

本院认为，被告人余龙辉违反野生动物保护法规，未经野生动物管理部门的批准，非法收购、出售国家保护野生动物及其制

品鹰嘴龟 23 只、穿山甲 7 只及鬣羚制品，价值达 405880 元，情节特别严重，其行为已构成非法收购、出售珍贵、濒危野生动物、珍贵、濒危野生动物制品罪，公诉机关指控的罪名成立，本院予以确认。被告人余龙辉在审判阶段自愿签署《认罪认罚具结书》，自愿认罪认罚，主动预缴了部分罚金，依法可以从轻处罚。公诉机关对被告人余龙辉适用刑罚种类、量刑幅度及罚金金额的建议适当，本院予以采纳。

附带民事公益诉讼被告余龙辉未经权力部门批准许可，非法收购、出售珍贵、濒危野生动物、珍贵、濒危野生动物制品，其行为破坏了野生动物生态资源，损害了国家和社会公共利益，违反了《中华人民共和国野生动物保护法》第三条第一款、第二十七条的规定，根据《中华人民共和国侵权责任法》第四条第一款的规定，被告余龙辉因其犯罪行为在承担刑事责任的同时，还需承担相应的民事责任。案涉中，被告余龙辉除个人实施了非法收购、出售珍贵、濒危野生动物、珍贵、濒危野生动物制品外，还与叶某 1、叶某 2、汪某、陈某 3、李某 2 等人共同实施了非法收购、出售珍贵、濒危野生动物及制品侵权行为，依照《中华人民共和国侵权责任法》第八条规定，应共同承担连带清偿责任。最高人民法院及最高人民检察院《关于检察公益诉讼案件适用法律若干问题的解释》［法释（2018）6 号］第十三条第一款“人民检察院在履行职责中发现破坏生态环境和资源保护、食品药品安全领域侵害众多消费者合法权益等损害社会公共利益的行为，

拟提起公益诉讼的，应当依法公告，公告期间为三十日”、第二款“公告期满，法律规定的机关和有关组织不提起诉讼的，人民检察院可以向人民法院提起诉讼”的规定，漳平市人民检察院在提起本案刑事附带民事公益诉讼前已依法履行相应的公告程序，在公告期间内，无其他法律规定的适格机关或者组织提起诉讼，漳平市人民检察院以公益诉讼起诉人身份就本案被告人余龙辉因犯罪行为侵权提起附带民事公益诉讼，主体适格。依照《野生动物及其制品价值评估办法》（国家林业局令 46 号）第四条第一款“野生动物整体的价值，按照《陆生野生动物基准价值标准目录》所列该种野生动物的基准价值乘以相应的倍数核算”、第二款第（一）项“国家一级保护野生动物，按照所列野生动物基准价值的十倍核算；国家二级保护野生动物，按照所列野生动物基准价值的五倍核算”的规定，被告余龙辉实施非法收购、出售野生动物鹰嘴龟、穿山甲及鬣羚制品犯罪行为时，鹰嘴龟属国家一级保护野生动物，穿山甲属国家二级保护野生动物，依照《野生动物及其制品价值评估办法》（国家林业局令 46 号）附件《陆生野生动物基准价值标准目录》规定，鹰嘴龟每只基准价值标准为 500 元，穿山甲每只基准价值标准为 8000 元。因此涉案野生动物 23 只鹰嘴龟的整体价值合计为 115000 元，7 只穿山甲的整体价值合计为 280000 元；根据犯罪现场被查扣的鬣羚制品，公益诉讼起诉人认定被告余龙辉至少存在一只鬣羚被非法收购、出售的事实，理由成立，本院予以支持，即依照《野生动物及其制

品价值评估办法》（国家林业局令46号）附件《陆生野生动物基准价值标准目录》等规定，鬣羚属国家二级保护野生动物，每只基准价为10000元，整体价值应为50000元。综上，被告余龙辉因其犯罪行为，给国家和社会造成野生动物资源损失合计445000元。与被告余龙辉共同实施的非法收购、出售珍贵、濒危野生动物行为的叶某1、叶某2、汪某、陈某3、李某2，已另案提起刑事附带民事公益诉讼，诉请赔偿野生动物资源损失，本院以（2020）闽0881刑初159、180、197、209、210号刑事附带民事判决书分别判令叶某1赔偿40000元、叶某2赔偿40000元、李某2与叶某2共同赔偿40000元、汪某赔偿10000元、陈某3赔偿70000元，其中叶某1、叶某2及汪某均已履行了赔偿义务，陈某3尚未履行赔偿义务。综上所述，漳平市人民检察院提起的刑事附带民事公益诉讼的诉讼请求，于法有据，本院予以支持。依照《中华人民共和国刑法》第三百四十一条第一款、第四十五条、第四十七条、第五十二条、五十三条第一款、第六十一条、第六十二条、第六十四条，《中华人民共和国刑事诉讼法》第十五条、第一百零一条第二款，《中华人民共和国侵权责任法》第四条第一款、第八条、第十五条第一款第（六）、第（七）项，最高人民法院及最高人民检察院《关于检察公益诉讼案件适用法律若干问题的解释》第二十条之规定，判决如下：

一、被告人余龙辉犯非法收购、出售珍贵、濒危野生动物、珍贵、濒危野生动物制品罪，判处有期徒刑十二年八个月，并处

罚金 400000 元（其中的罚金 200000 元已缴纳，余款限于本判决生效后十日内缴纳）；

（刑期从判决执行之日起计算。判决执行前先行羁押的，羁押一日折抵刑期一日。即自 2020 年 2 月 23 日起至 2032 年 10 月 22 日止）

二、被告人余龙辉的违法所得款共计 96588 元，予以没收。其中：侦查机关已冻结的被告人余龙辉名下在中国建设银行储蓄卡（账户：43××××06）违法所得款 75000 元，由漳平市公安局森林分局依法上缴国库；继续追缴违法所得余款 21588 元，并予以上缴国库；

三、附带民事公益诉讼被告余龙辉应自本判决生效之日起十日内赔偿国家野生动物资源损失折合 285000 元，并上缴国家指定账户；

四、附带民事公益诉讼被告余龙辉应自本判决生效之日起十日内与陈某 3 连带赔偿国家野生动物资源损失折合 70000 元，并上缴国家指定账户；

五、附带民事公益诉讼被告余龙辉应自本判决生效之日起十日内对其非法收购、出售珍贵、濒危野生动物、珍贵、濒危野生动物制品，破坏野生动物生态资源，损害国家和社会公共利益的行为，在漳平市（县级以上新闻媒体向社会公开赔礼道歉。

如果未按本判决指定的期间履行本判决第三、第四项给付金钱义务，应当依照《中华人民共和国民事诉讼法》第二百五十三

条规定，加倍支付迟延履行期间的债务利息。在漳平市（县级以上新闻媒体向社会公开赔礼道歉内容应先报本院审查，如附带民事公益诉讼被告余龙辉不履行赔礼道歉义务，本院将依法代履行，依此所产生的相应费用由附带民事公益诉讼被告余龙辉负担。

如不服本判决，可在接到判决书的第二日起十日内，通过本院或者直接向福建省龙岩市中级人民法院提出上诉。书面上诉的，应当提交上诉状正本一份，副本一份。

审　判　长　　朱隆武

审　判　员　　温英明

审　判　员　　洪亚香

人民陪审员　　赖家金

人民陪审员　　王精忠

人民陪审员　　郑淑琼

人民陪审员　　林燕红

二〇二一年一月五日

书　记　员　　王晓琳

附相关法律条文：

《中华人民共和国刑法》

第四十五条有期徒刑的期限，除本法第五十条、第六十九条规定外，为六个月以上十五年以下。

第四十七条有期徒刑的刑期，从判决执行之日起计算；判决执行以前先行羁押的，羁押一日折抵刑期一日。

第五十二条判处罚金，应当根据犯罪情节决定罚金数额。

第五十三条罚金在判决指定的期限内一次或者分期缴纳。期满不缴纳的，强制缴纳。对于不能全部缴纳罚金的，人民法院在任何时候发现被执行人有可以执行的财产，应当随时追缴。

由于遭遇不能抗拒的灾祸等原因缴纳确实有困难的，经人民法院裁定，可以延期缴纳、酌情减少或者免除。

第六十一条对于犯罪分子决定刑罚的时候，应当根据犯罪的事实、犯罪的性质、情节和对于社会的危害程度，依照本法的有关规定判处。

第六十二条犯罪分子具有本法规定的从重处罚、从轻处罚情节的，应当在法定刑的限度以内判处刑罚。

第六十四条犯罪分子违法所得的一切财物，应当予以追缴或者责令退赔；对被害人的合法财产，应当及时返还；违禁品和供犯罪所用的本人财物，应当予以没收。没收的财物和罚金，一律上缴国库，不得挪用和自行处理。

第三百四十一条【非法猎捕、杀害珍贵、濒危野生动物罪】【非法收购、运输、出售珍贵、濒危野生动物、珍贵、濒危野生动物制品罪】非法猎捕、杀害国家重点保护的珍贵、濒危野生动物的，或者非法收购、运输、出售国家重点保护的珍贵、濒危野生动物及其制品的，处五年以下有期徒刑或者拘役，并处罚金；

情节严重的，处五年以上十年以下有期徒刑，并处罚金；情节特别严重的，处十年以上有期徒刑，并处罚金或者没收财产。

【非法狩猎罪】违反狩猎法规，在禁猎区、禁猎期或者使用禁用的工具、方法进行狩猎，破坏野生动物资源，情节严重的，处三年以下有期徒刑、拘役、管制或者罚金。

《中华人民共和国刑事诉讼法》

第十五条犯罪嫌疑人、被告人自愿如实供述自己的罪行，承认指控的犯罪事实，愿意接受处罚的，可以依法从宽处理。

第一百零一条被害人由于被告人的犯罪行为而遭受物质损失的，在刑事诉讼过程中，有权提起附带民事诉讼。被害人死亡或者丧失行为能力的，被害人的法定代理人、近亲属有权提起附带民事诉讼。

如果是国家财产、集体财产遭受损失的，人民检察院在提起公诉的时候，可以提起附带民事诉讼。

《中华人民共和国侵权责任法》

第四条侵权人因同一行为应当承担行政责任或者刑事责任的，不影响依法承担侵权责任。

因同一行为应当承担侵权责任和行政责任、刑事责任，侵权人的财产不足以支付的，先承担侵权责任。

第八条二人以上共同实施侵权行为，造成他人损害的，应当承担连带责任。

第十五条承担侵权责任的方式主要有：

（一）停止侵害；

（二）排除妨碍；

（三）消除危险；

（四）返还财产；

（五）恢复原状；

（六）赔偿损失；

（七）赔礼道歉；

（八）消除影响、恢复名誉。

以上承担侵权责任的方式，可以单独适用，也可以合并适用。

最高人民法院最高人民检察院《关于检察公益诉讼案件适用法律若干问题的解释》

第二十条、人民检察院对破坏生态环境和资源保护、食品药品安全领域侵害众多消费者合法权益的犯罪行为提起刑事公诉的，可以向人民法院一并提起附带民事公益诉讼，由人民法院同一审判组织审理。

人民检察院提起的刑事附带民事公益诉讼案件由审理刑事案件的人民法院管辖。
