# Supplementary material for: China’s Legal Protection System for Pangolins: Past, Present, and Future
Source: Animals (Basel). 2025 Aug 18;15(16):2422. doi: 10.3390/ani15162422 (PMC12383201; doi:10.3390/ani15162422)
Supplement: Supplementary file 1 [file animals-15-02422-s001.zip › Supplementary Material S4-Full Text of Judgments in Pangolin-Related Public Interest Litigation Cases in China/【24】叶兴元非法收购、运输、出售珍贵、濒危野生动物、珍贵、濒危野生动物制品罪一审刑事判决书.pdf]

叶兴元非法收购、运输、出售珍贵、濒危野生动物、珍贵、濒危野生动物制品罪一审刑事判决书

福建省厦门市同安区人民法院

刑 事 附 带 民 事 判 决 书

（2020）闽 0212 刑初 246 号

公诉机关暨附带民事公益诉讼起诉人厦门市同安区人民检察院。

被告人暨附带民事公益诉讼被告叶兴元，男，1986 年 9 月 7 日出生于福建省诏安县，汉族，小学文化，个体户，户籍地福建省诏安县。因涉嫌犯非法收购、出售珍贵、濒危野生动物罪，于 2020 年 3 月 26 日被公安机关取保候审，同年 8 月 12 日被公诉机关取保候审，同年 9 月 2 日被本院取保候审。

辩护人暨附带民事公益诉讼委托代理人沈惠生、沈珍琴，福建上达律师事务所律师。

厦门市同安区人民检察院以同检刑诉〔2020〕230 号起诉书指控被告人叶兴元犯非法收购、出售珍贵、濒危野生动物、珍贵、濒危野生动物制品罪，于 2020 年 8 月 28 日向本院提起公诉，诉讼过程中以同检刑附民公诉〔2020〕3 号刑事附带民事公益诉讼起诉书向本院提起附带民事公益诉讼。本院依法适用普通程序并组成合议庭，于 2020 年 11 月 5 日公开开庭合并审理了本案。厦门市同安区人民检察院指派检察员林静娟出庭支持公诉、参加附带民事公益诉讼。被告人叶兴元及其辩护人沈惠生到庭参加诉

讼。期间，公诉机关建议延期审理，并于期满后建议恢复审理。现已审理终结。

公诉机关指控，2019 年 3 月，被告人叶兴元以人民币 100 余元价格向他人购买雀鹰死体一只。2019 年 12 月中旬，被告人叶兴元以每斤人民币 180 元的价格共计人民币 3000 多元向他人购买穿山甲冻死体（包含内脏、鳞片）一只。后被告人叶兴元将上述雀鹰、穿山甲死体存放在其租住的文屏路××号之 111 室冰箱内准备用于出售牟利，直至 2020 年 3 月 26 日被公安机关查获。经鉴定：穿山甲、雀鹰均被列入《国家重点保护野生动物名录》，属国家Ⅱ级重点保护野生动物。穿山甲 1 只价值为人民币 40000 元。2020 年 3 月 27 日，被告人叶兴元经电话通知到公安机关接受调查，如实供述上述犯罪事实。现上述雀鹰、穿山甲冰冻死体暂扣在公安机关。

为支持指控，公诉人当庭讯问了被告人叶兴元，宣读、出示了相关物证、书证、证人证言、鉴定意见、辨认笔录及照片、被告人叶兴元的供述与辩解等证据。

公诉机关认为，被告人叶兴元的行为应以非法收购、出售珍贵、濒危野生动物、珍贵、濒危野生动物制品罪追究刑事责任。被告人叶兴元犯罪以后自动投案，如实供述自己的罪行，系自首，可以从轻或减轻处罚。提请依法判处。

附带民事公益诉讼起诉人诉请判令：被告人叶兴元赔偿国家二级重点保护野生动物穿山甲 1 只、雀鹰 1 只的生态价值损失共

计人民币 65000 元。事实和理由：同刑事部分事实和理由，另经鉴定，穿山甲 1 只，生态价值为人民币 40000 元；雀鹰 1 只，生态价值为人民币 25000 元。

为支持诉请，附带民事公益诉讼起诉人举示了微信聊天记录、情况说明、证人证言、司法鉴定意见书等证据。

被告人叶兴元对公诉机关指控的事实和罪名均无异议。

辩护人提出被告人叶兴元系自首、自愿认罪认罚、初犯、偶犯、主动赔偿民事部分损失，具有法定从轻减轻和酌情从轻处罚情节，请求对被告人处以三年以下有期徒刑并适用缓刑。

对附带民事公益诉讼起诉人的诉讼请求，被告人叶兴元及其委托代理人均表示没有意见。

经审理查明：公安机关查获非法出售野生动物线索后，于 2020 年 3 月 26 日对思明区将军祠 30 号房进行突击检查，查获叶兴元在该处所存放蛇类等野生动物，经办案民警现场突审，叶兴元主动交代在其租住的文屏路××号之 111 室还存放一只穿山甲及雀鹰，民警立即赶赴该处所并在该处所冰箱内查获一只雀鹰死体及一只穿山甲冻死体（包含内脏、鳞片）。雀鹰死体一只系由被告人叶兴元于 2019 年 3 月以人民币 100 余元价格向他人购买。穿山甲冻死体（包含内脏、鳞片）一只系由被告人叶兴元于 2019 年 12 月中旬以每斤人民币 180 元的价格共计人民币 3000 多元向他人购买。后被告人叶兴元将上述雀鹰、穿山甲死体存放在文屏路××号之 111 室冰箱内准备用于出售牟利，并通过微信

向他人进行报价。现上述雀鹰、穿山甲冰冻死体暂扣在公安机关。经鉴定：穿山甲、雀鹰均被列入《国家重点保护野生动物名录》，属国家Ⅱ级重点保护野生动物；穿山甲1只，价值及生态价值均为人民币40000元；雀鹰1只，生态价值为人民币25000元。

2020年3月27日，被告人叶兴元经电话通知到公安机关接受调查，如实供述上述犯罪事实。

同时查明，2020年8月5日，厦门市同安区人民检察院就关于叶兴元非法收购、出售珍贵、濒危野生动物行为损害社会公共利益拟提起民事公益诉讼在《检察日报》上发布公告，公告期间为三十日。公告期间无任何机关或组织向其反馈拟提起公益诉讼。被告人叶兴元已向本院缴纳人民币5000元用于执行本案的罚金刑，并缴纳附带民事公益诉讼赔偿款人民币65000元。

上述事实，被告人叶兴元在开庭审理过程中亦无异议，并有经庭审质证的证人叶某、蔡某、邓某的证言；人员基本信息、到案经过、证明、身份证、居住证、微信聊天记录、情况说明、诉讼文书；江西亚林司法鉴定中心司法鉴定意见书；辨认、提取笔录及称重照片；被告人叶兴元的供述与辩解等证据证实，足以认定。

认定上述民事部分的事实，除上述证据外，有附带民事公益诉讼起诉人另行提供且经庭审质证的福建闽林司法鉴定中心司法鉴定意见书等证据证实，本院予以确认。

案件审理期间，本院委托厦门市思明区司法局对被告人叶兴元进行审前社会调查，厦门市思明区司法局出具评估意见为：被告人叶兴元基本具备实施社区矫正的条件。

本院认为，被告人叶兴元违反国家有关野生动物保护法规，非法收购、出售国家二级重点保护野生动物雀鹰1只以及穿山甲制品，制品价值人民币40000元，其行为已构成非法收购、出售珍贵、濒危野生动物、珍贵、濒危野生动物制品罪。公诉机关指控的罪名成立。被告人叶兴元已着手实施出售行为，但案涉雀鹰及穿山甲冻死体尚未实际售出，系犯罪未遂，依法可以比照既遂犯从轻或者减轻处罚。被告人叶兴元犯罪后自动投案并如实供述自己的罪行，系自首，依法可以从轻或者减轻处罚。被告人叶兴元主动缴纳生态价值损失赔偿款，可以酌情从轻处罚。结合厦门市思明区司法局出具的调查评估意见，本院认为对被告人叶兴元适用缓刑对所居住社区没有重大不良影响。综合本案的犯罪性质、情节、危害后果及被告人叶兴元的具体量刑情节，本院决定对被告人叶兴元从轻处罚并适用缓刑。辩护人相关辩解意见成立，本院予以采纳。

被告人叶兴元非法收购、出售珍贵、濒危野生动物、珍贵、濒危野生动物制品，造成国家野生动物资源受损，侵害社会公共利益，依法应承担相应的民事侵权责任。附带民事公益诉讼起诉人主张被告人叶兴元赔偿国家二级重点保护野生动物穿山甲1只、雀鹰1只的生态价值损失共计人民币65000元，有相应的事

实和法律依据，本院依法予以支持。综上，依照《中华人民共和国刑法》第三百四十一条第一款、第二十三条、第六十七条第一款、第七十二条、第七十三条、《最高人民法院关于审理破坏野生动物资源刑事案件具体应用法律若干问题的解释》第一条、《最高人民法院关于适用财产刑若干问题的规定》第二条、《中华人民共和国侵权责任法》第六条、第十五条、《最高人民法院、最高人民检察院关于检察公益诉讼案件适用法律若干问题的解释》第二十条、《最高人民法院关于审理环境民事公益诉讼案件适用法律若干问题的解释》第十八条之规定，判决如下：

一、被告人叶兴元犯非法收购、出售珍贵、濒危野生动物、珍贵、濒危野生动物制品罪，判处有期徒刑一年四个月，缓刑二年，并处罚金人民币五千元。

（缓刑考验期限，从判决确定之日起计算。罚金已缴纳。）

二、扣押在公安机关的雀鹰冻死体1只、穿山甲冻死体1只，予以没收。

三、被告人叶兴元应于本判决生效之日起三十日内支付生态价值损失赔偿款人民币65000元（赔偿款已缴纳）。

如不服本判决，可在接到判决书的第二日起十日内，通过本院或者直接向福建省厦门市中级人民法院提出上诉。书面上诉的，应当提交上诉状正本一份，副本二份。

审　判　长　洪　秀　娟

审　判　员　叶　采　惠

审　判　员　杨　　　　　　　　　郁

人民陪审员　叶棋发人民陪审员何祝琴

人民陪审员　蔡　　　　龙　　　　伟

人民陪审员　蔡　　　　秀　　　　端

二〇二〇年十二月三十日

法官　助理　颜　　　　振　　　　华

代书　记员　叶　　　　婉　　　　瑜

附：本判决所适用的法律依据

《中华人民共和国刑法》

第三百四十一条非法猎捕、杀害国家重点保护的珍贵、濒危野生动物的，或者非法收购、运输、出售国家重点保护的珍贵、濒危野生动物及其制品的，处五年以下有期徒刑或者拘役，并处罚金；情节严重的，处五年以上十年以下有期徒刑，并处罚金；情节特别严重的，处十年以上有期徒刑，并处罚金或者没收财产。

违反狩猎法规，在禁猎区、禁猎期或者使用禁用的工具、方法进行狩猎，破坏野生动物资源，情节严重的，处三年以下有期徒刑、拘役、管制或者罚金。

第二十三条已经着手实行犯罪，由于犯罪分子意志以外的原因而未得逞的，是犯罪未遂。

对于未遂犯，可以比照既遂犯从轻或者减轻处罚。

第六十七条犯罪以后自动投案，如实供述自己的罪行的，是自首。对于自首的犯罪分子，可以从轻或者减轻处罚。其中，犯罪较轻的，可以免除处罚。

被采取强制措施的犯罪嫌疑人、被告人和正在服刑的罪犯，如实供述司法机关还未掌握的本人其他罪行的，以自首论。

犯罪嫌疑人虽不具有前两款规定的自首情节，但是如实供述自己罪行的，可以从轻处罚；因其如实供述自己罪行，避免特别严重后果发生的，可以减轻处罚。

第七十二条对于被判处拘役、三年以下有期徒刑的犯罪分子，同时符合下列条件的，可以宣告缓刑，对其中不满十八周岁的人、怀孕的妇女和已满七十五周岁的人，应当宣告缓刑：

（一）犯罪情节较轻；

（二）有悔罪表现；

（三）没有再犯罪的危险；

（四）宣告缓刑对所居住社区没有重大不良影响。

宣告缓刑，可以根据犯罪情况，同时禁止犯罪分子在缓刑考验期限内从事特定活动，进入特定区域、场所，接触特定的人。

被宣告缓刑的犯罪分子，如果被判处附加刑，附加刑仍须执行。

第七十三条拘役的缓刑考验期限为原判刑期以上一年以下，但是不能少于二个月。

有期徒刑的缓刑考验期限为原判刑期以上五年以下，但是不能少于一年。

缓刑考验期限，从判决确定之日起计算。

《最高人民法院关于审理破坏野生动物资源刑事案件具体应用法律若干问题的解释》

第一条刑法第三百四十一条第一款规定的“珍贵、濒危野生动物”，包括列入国家重点保护野生动物名录的国家一、二级保护野生动物、列入《濒危野生动植物种国际贸易公约》附录一、附录二的野生动物以及驯养繁殖的上述物种。

《最高人民法院关于适用财产刑若干问题的规定》

第二条人民法院应当根据犯罪情节，如违法所得数额、造成损失的大小等，并综合考虑犯罪分子缴纳罚金的能力，依法判处罚金。刑法没有明确规定罚金数额标准的，罚金的最低数额不能少于一千元。

对未成年人犯罪应当从轻或者减轻判处罚金，但罚金的最低数额不能少于五百元。

《中华人民共和国侵权责任法》

第六条行为人因过错侵害他人民事权益，应当承担侵权责任。

根据法律规定推定行为人有过错，行为人不能证明自己没有过错的，应当承担侵权责任。

第十五条承担侵权责任的方式主要有：

（二）排除妨碍；

（三）消除危险；

（四）返还财产；

（五）恢复原状；

（六）赔偿损失；

（七）赔礼道歉；

（八）消除影响、恢复名誉。

以上承担侵权责任的方式，可以单独适用，也可以合并适用。

《最高人民法院、最高人民检察院关于检察公益诉讼案件适用法律若干问题的解释》

第二十条人民检察院对破坏生态环境和资源保护、食品药品安全领域侵害众多消费者合法权益等损害社会公共利益的犯罪行为提起刑事公诉时，可以向人民法院一并提起附带民事公益诉讼，由人民法院同一审判组织审理。

人民检察院提起的刑事附带民事公益诉讼案件由审理刑事案件的人民法院管辖。

《最高人民法院关于审理环境民事公益诉讼案件适用法律若干问题的解释》

第十八条对污染环境、破坏生态，已经损害社会公共利益或者具有损害社会公共利益重大风险的行为，原告可以请求被告承担停止侵害、排除妨碍、消除危险、恢复原状、赔偿损失、赔礼道歉等民事责任。
