# Supplementary material for: China’s Legal Protection System for Pangolins: Past, Present, and Future
Source: Animals (Basel). 2025 Aug 18;15(16):2422. doi: 10.3390/ani15162422 (PMC12383201; doi:10.3390/ani15162422)
Supplement: Supplementary file 1 [file animals-15-02422-s001.zip › Supplementary Material S4-Full Text of Judgments in Pangolin-Related Public Interest Litigation Cases in China/【25】郑凯非法收购、运输、出售珍贵、濒危野生动物、珍贵、濒危野生动物制品一审刑事判决书.pdf]

郑凯非法收购、运输、出售珍贵、濒危野生动物、珍贵、濒危野生动物制品一审刑事判决书

云南省芒市人民法院

刑 事 判 决 书

（2020）云 3103 刑初 359 号

公诉机关芒市人民检察院。

被告人郑凯，男，1974 年 8 月 12 日出生，汉族,初中文化，务农，户籍所在地四川省成都市邛崃市，现住云南省德宏傣族景颇族自治州芒市。曾因犯抢劫罪、盗窃罪被四川省成都市中级人民法院数罪并罚判处有期徒刑十四年，并处罚金人民币 15000 元，于 2013 年 7 月 4 日刑满释放。现因涉嫌犯非法运输珍贵、濒危野生动物制品罪于 2019 年 12 月 9 日被芒市公安局取保候审。2020 年 8 月 12 日被芒市人民检察院取保候审。同年 10 月 30 日被本院取保候审。

芒市人民检察院以芒检三部刑诉[2020]34 号起诉书，指控被告人郑凯犯非法运输珍贵、濒危野生动物制品罪，于 2020 年 10 月 22 日向本院提起公诉。本院依法组成七人合议庭，公开开庭审理了本案。芒市人民检察院指派检察官杨连文、检察官助理郝镜丹出庭支持公诉，被告人郑凯到庭参加诉讼。现已审理终结。

芒市人民检察院指控：被告人郑凯于 2019 年 11 月 10 日，驾驶车牌为川Ａ×××**的福克斯轿车从瑞丽市畹町前往芒市，途径芒市遮放镇戛中检查站时被执勤民警查获，当场从其驾驶的

车牌为川Ａ×××**的福克斯轿车后备箱内查获疑似穿山甲死体一只。经鉴定，查获的疑似穿山甲死体来源于鳞甲目科穿山甲属中华穿山甲Ｍａｎｉｓｐｅｎｔａｃｔｙｌａ,为国家Ⅱ级保护动物，被列入《濒危野生动植物种国际贸易公约》（ＣＩＴＥＳ）附录Ｉ，经济价值为人民币40000元。

针对上述指控，公诉机关向本院提交如下证据:物证，书证，证人证言，被告人供述和辩解，鉴定意见，勘验、检查、辨认等笔录，视听资料。公诉机关认为，被告人郑凯违反野生动物保护法律法规，非法运输国家重点保护野生动物，其行为已触犯《中华人民共和国刑法》第三百四十一条第一款，犯罪事实清楚，证据确实、充分，应当以非法运输珍贵、濒危野生动物制品罪追究其刑事责任。被告人自愿认罪认罚，可以依法从宽处理。据此，根据《中华人民共和国刑事诉讼法》第一百七十六条第一款之规定，提起公诉，提请本院依法判处。并向本院提交被告人认罪认罚具结书及量刑建议，建议以非法运输珍贵、濒危野生动物制品罪判处被告人郑凯有期徒刑六个月，并处罚金。

被告人郑凯对公诉机关指控的事实和罪名均无异议。自愿签字具结，在庭审过程中亦无异议。

经审理查明：被告人郑凯于2019年11月10日，驾驶车牌为川Ａ×××**的福克斯轿车从瑞丽市畹町前往芒市，途径芒市遮放镇戛中检查站时被执勤民警查获，当场从其驾驶的车牌为川Ａ×××**的福克斯轿车后备箱内查获疑似穿山甲死体一只。经

鉴定，查获的疑似穿山甲死体来源于鳞甲目科穿山甲属中华穿山甲Ｍａｎｉｓｐｅｎｔａｃｔｙｌａ,为国家Ⅱ级保护动物，被列入《濒危野生动植物种国际贸易公约》（ＣＩＴＥＳ）附录Ｉ，经济价值为人民币 40000 元。

上述事实，有当庭举证、质证的下列证据予以证实：受案登记表，受案回执，立案决定书，移交案件登记表，随案物品移交清单，被告人身份证复印件，抓获经过，现场清点、称量笔录附照片，扣押决定书，扣押清单，扣押笔录，接受证据清单，机动车行驶证复印件，发还清单，刑事判决书，协查函，释放证明，鉴定聘请书，司法鉴定委托书，司法鉴定意见书附相关鉴定资质证明，鉴定意见通知书，证人孙某俊、孙某誉的证言，被告人供述与辩解，现场辨认笔录附照片，现场勘验笔录附照片，和解协议，同步录音录像光盘等证据。

上述证据内容客观真实，来源合法，各证据之间能相互印证，具有法律证明效力，本院予以确认，并作为定案依据。

本院认为，被告人郑凯违反野生动物保护法律法规，非法运输国家重点保护的珍贵、濒危野生动物制品，其行为已触犯刑律，构成非法运输珍贵、濒危野生动物制品罪。公诉机关指控事实清楚，证据确实、充分，罪名成立，本院予以确认。被告人郑凯非法运输价值 40000 元动物制品，根据《中华人民共和国刑法》第三百四十一条第一款之规定，应处五年以下有期徒刑或者拘役，并处罚金。被告人自愿认罪认罚，可以依法从宽处理。在附带民

事公益诉讼中，被告人自愿和公益诉讼起诉人达成和解协议并确定履行时间，可酌情从轻处罚。公诉机关提出的被告人认罪认罚具结书及量刑建议，符合法律规定，本院予以采纳。综上，结合被告人犯罪的事实，情节，社会危害程度及悔罪表现。依照《中华人民共和国刑法》第三百四十一条第一款，第六十七条第三款，第六十一条，第五十二条，第五十三条，第六十四条及《中华人民共和国刑事诉讼法》第十五条之规定，判决如下：

一、被告人郑凯犯非法运输珍贵、濒危野生动物制品罪，判处有期徒刑六个月，并处罚金人民币四千元。

（刑期从判决实际执行之日起计算，判决执行以前先行羁押的，羁押一日折抵刑期一日。罚金限判决生效之日一次缴纳。）

二、在案扣押的穿山甲死体一只，依法予以没收。

如不服本判决，可在接到判决书的第二日起十日内，通过本院或者直接向德宏傣族景颇族自治州中级人民法院提出上诉，书面上诉的，应提交上诉状正本一份，副本二份。

审　判　长　　段学忠

审　判　员　　段丽红

审　判　员　　申　珺

人民陪审员　　赵仕芳

人民陪审员　　陈　玲

人民陪审员　　杨国琼

人民陪审员　　杨　利

二〇二〇年十二月三日

书　记　员　　董　娜
