# Supplementary material for: China’s Legal Protection System for Pangolins: Past, Present, and Future
Source: Animals (Basel). 2025 Aug 18;15(16):2422. doi: 10.3390/ani15162422 (PMC12383201; doi:10.3390/ani15162422)
Supplement: Supplementary file 1 [file animals-15-02422-s001.zip › Supplementary Material S4-Full Text of Judgments in Pangolin-Related Public Interest Litigation Cases in China/【26】张玉权、刘周艳走私珍贵动物、珍贵动物制品一审刑事判决书.pdf]

张玉权、刘周艳走私珍贵动物、珍贵动物制品一审刑事判决书

云南省澄江市人民法院

刑 事 附 带 民 事 判 决 书

（2020）云 0422 刑初 113 号

公诉机关暨附带民事公益诉讼起诉人：云南省澄江市人民检察院。

被告人暨附带民事公益诉讼被告：张玉权，男，1980 年 12 月 18 日生，汉族，云南省澄江市人，初中文化，户籍所在地云南省澄江市。因本案于 2020 年 1 月 18 日被澄江市森林公安局取保候审。

指定辩护人:符利果，云南澄海律师事务所律师。

被告人暨附带民事公益诉讼被告：刘周艳，男，1973 年 9 月 16 日生，汉族，云南省罗平县人，高中文化，户籍所在地云南省澄江市。因本案于 2020 年 1 月 18 日被澄江市森林公安局取保候审。

指定辩护人:刘宗琼，云南澄海律师事务所律师。

澄江市人民检察院以澄检一部刑诉〔2020〕38 号起诉书指控被告人张玉权、刘周艳犯走私珍贵动物制品罪，于 2020 年 5 月 26 日向本院提起公诉。公益诉讼起诉人澄江市人民检察院于 2020 年 5 月 26 日向本院提起附带环境民事公益诉讼。经查，澄江市人民检察院于 2020 年 3 月 9 日公告了案件相关情况，公告

期内未有法律规定的机关和组织提起民事公益诉讼。本院依法组成合议庭，于2020年7月7日公开开庭审理了本案。澄江市人民检察院指派检察员洪和兴、代理检察员洪晟尧出庭履行职务，上述人员均到庭参加诉讼。经报请玉溪市中级人民法院批准，本案延长审理期限三个月。现已审理终结。

澄江市人民检察院指控：被告人刘周艳及其家人听信穿山甲甲片能治疗其妻子的病，遂委托长期进出中缅边境的朋友张玉权帮忙从境外购买。2019年10月11日，张玉权与刘周艳用微信电话商定后，在缅甸0元的价格购买了一副疑似穿山甲甲片（495.38克），藏匿于其驾驶的云F×××**东风天龙牌大货车的工具箱内，从境外偷运回到澄江市鑫光车辆施救中心报废汽车回收厂，并将该副甲片转移到其云F×××**摩托车上。2019年10月14日17时，张玉权骑行至澄江市行政中心南大门处被查获。经鉴定，该副甲片为一只鳞甲目穿山甲科穿山甲的甲片，穿山甲属于国家Ⅱ级重点保护野生动物，1只穿山甲价值为40000元。

针对指控事实，公诉机关当庭列举了物证、书证、证人证言、被告人的供述和辩解、鉴定意见、现场勘验、指认笔录等证据予以证实。据此认为，被告人张玉权、刘周艳违反我国海关法规，逃避海关监管，明知是国家禁止进出口的珍贵动物制品，仍非法携带、运输珍贵动物制品入境，其行为依法构成走私珍贵动物制品罪；被告人张玉权、刘周艳在司法机关未确定犯罪嫌疑人，尚

在一般性排查询问时主动交代自己的罪行，到案后如实供述犯罪事实，具有自首情节，认罪认罚且签字具结，应依照《中华人民共和国刑法》第一百五十一条、第六十七条第一款、《中华人民共和国刑事诉讼法》第十五条之规定定罪处罚。诉请依法判处，并书面建议本院分别判处被告人张玉权、刘周艳一年以上二年以下有期徒刑，可适用缓刑，并处罚金人民币2000元以上5000元以下。

附带民事公益诉讼起诉人澄江市人民检察院向本院提出诉讼请求：1、判令二被告人共同连带赔偿自然资源损失补偿费人民币40000元；2、判令二被告人对其破坏野生动物资源的行为当庭进行公开道歉。事实及理由：

人与自然和谐相处是人类社会追求的重要目标，依法保护野生动物对于保障生物多样性，保护生态系统完整性具有重要意义。根据《中华人民共和国野生动物保护法》第三条第一款之规定，野生动物资源属于国家所有。本案中，穿山甲属于国家Ⅱ级重点保护野生动物，并列入《濒危野生动植物种国际贸易公约》附录Ⅰ，被告人张玉权、刘周艳非法走私珍贵动物制品的行为，破坏了野生动物资源，侵害了国家利益和社会公共利益，已经构成侵权。故依照《中华人民共和国侵权责任法》第六条第一款、第十五条、《中华人民共和国野生动物保护法》第三条第一款、《最高人民法院关于审理环境民事公益诉讼案件适用法律若干

问题的解释》第十八条的规定，提起附带民事公益诉讼，请依法裁判。

庭审中，被告人张玉权对起诉书指控的事实、罪名、量刑情节、量刑建议无异议，自愿认罪认罚且签字具结，但提出：其从境外带穿山甲甲片并不是为了获利，是因其与刘周艳的妻子罗艳兰是同学，罗艳兰患病多年，其是为了治病救人，穿山甲甲片在药店也能买到，其现在认识到自己的错误，请求对其从轻处罚；对于公益诉讼赔偿部分，其愿意赔偿，但希望数额能少一点。

被告人刘周艳对起诉书指控的事实、罪名、量刑情节、量刑建议无异议，自愿认罪认罚且签字具结，但提出：其购买穿山甲甲片是来治病，并不是用来买卖，希望法庭看在其妻子生病多年，对其从轻处罚；对于公益诉讼赔偿部分，其愿意赔偿，但请求少赔一点。

被告人张玉权的指定辩护人符利果对被告人张玉权的行为构成走私珍贵动物制品罪没有异议，但提出：被告人张玉权帮刘周艳从缅甸购买穿山甲并未获利；本案涉案的仅是穿山甲甲片，而鉴定结论是对整只穿山甲所作出的价值认定，现仅以涉案穿山甲是整只穿山甲甲片便认定属于整个穿山甲制品，从而认定价值为40000元不合理；被告人具有自首情节，系初犯、偶犯，认罪认罚且签字具结。综上，建议判处被告人张玉权一年有期徒刑，并适用缓刑，并处罚金2000元。

被告人刘周艳的指定辩护人刘宗琼对被告人刘周艳的行为构成走私珍贵动物制品罪没有异议，但提出：被告人刘周艳系为治疗妻子疾病而购买穿山甲甲片，而非为谋取不正当利益；被告人刘周艳并没有见到穿山甲甲片，也未直接购买穿山甲甲片，量刑时应考虑；被告人刘周艳具有自首情节，认罪认罚且签字具结，系初犯、偶犯。综上，建议判处被告人刘周艳一年有期徒刑，并适用缓刑，并处罚金2000元。

经审理查明：被告人刘周艳及其家人听信穿山甲甲片能治疗其妻子的病，遂委托长期进出中缅边境的朋友张玉权帮忙从境外购买。2019年10月11日，张玉权与刘周艳用微信电话商定后，由被告人张玉权在缅甸小勐拉的集市上以人民币2100元的价格购买了一副疑似穿山甲甲片（495.38克）后藏匿于张玉权驾驶的云F×××**东风天龙牌大货车的工具箱内，从境外偷运回到澄江市鑫光车辆施救中心报废汽车回收厂。2019年10月14日17时，被告人张玉权驾驶云F×××**号摩托车携带着该穿山甲甲片行至澄江市行政中心南大门处时被查获。经鉴定，该副甲片为一只鳞甲目穿山甲科穿山甲的甲片，穿山甲属于国家Ⅱ级重点保护野生动物，涉案的1只穿山甲价值为人民币40000元。

另查明：1、被告人张玉权、刘周艳尚在一般性排查询问时主动交代自己的罪行，到案后如实供述犯罪事实。

2、庭审中，被告人张玉权、刘周艳当庭进行了赔礼道歉，并得到附带民事公益诉讼起诉人的认可。

上述事实，有经庭审质证查实的内容真实、来源合法，且能相互印证，形成锁链的：受案登记表，立案决定书，查获经过，到案经过，被告人张玉权、刘周艳的供述和辩解，证人郝某、罗某、叶某的证言，鉴定聘请书、委托书，玉溪市玉林司法鉴定中心野生动物鉴定意见书，鉴定意见通知书，现场勘验笔录、示意图、照片，现场指认笔录、照片，检查笔录、照片，物证（穿山甲甲片）照片，称重照片，扣押决定书、笔录、清单，调取证据通知书、清单、进出收费站记录、鑫恒晨货运部运输合同、出入境通行证、海关公路车辆出境（港）申报单、出入境记录查询结果单，行驶证、驾驶证，云卡通，昆明市儿童医院门诊病历、门诊收费收据，罗艳兰的残疾人证、出院记录、病情诊断证明书，张玉权微信朋友圈截图，随案移送清单，电子数据检查笔录，户口证明等证据予以证实，足以认定。

本院认为，被告人张玉权、刘周艳违反我国海关法规，逃避海关监管，明知是国家禁止进出口的珍贵动物制品，仍非法携带、运输珍贵动物制品入境，二被告人的行为已构成走私珍贵动物制品罪，依律应处五年以上十年以下有期徒刑，并处罚金。公诉机关对二被告人的指控，事实清楚，证据确实充分，定性准确，本院予以支持。本案系共同犯罪。

关于二被告人及指定辩护人所提“被告人张玉权、刘周艳购买穿山甲甲片的目的是为了给刘周艳的妻子治病，不是为了获得不正当利益”的主要辩护意见，经查，本案被告人刘周艳的妻子

确患有疾病，但应采用合法的方式购买所需药材，二被告人违反国家法律走私珍贵动物制品的行为已触犯我国法律构成犯罪，其走私的目的不影响本案的定性，故对该辩护意见，本院不予采纳。被告人刘周艳的指定辩护人所提“刘周艳没有见到穿山甲甲片，也未直接购买穿山甲甲片”的辩护意见，本案是因被告人刘周艳在得知穿山甲甲片能治疗其妻子的病后，与被告人张玉权商议，委托被告人张玉权从境外购买穿山甲甲片后带回境内，应认定为本案走私珍贵动物制品罪的共犯，故对该辩护意见，本院不予采纳。关于本案涉案的穿山甲甲片的价值认定问题，经查，公安机关依法委托有资质的鉴定机构对涉案的穿山甲甲片进行鉴定，经鉴定，本案涉案的穿山甲甲片是一整只穿山甲的甲片，涉案的1只穿山甲价值为人民币40000元，该鉴定结论系有鉴定资质的机构依法作出，应予采纳，故对被告人张玉权的指定辩护人所提“本案涉案的穿山甲价值认定为40000元不合理”的辩护意见，本院不予采纳。二位指定辩护人所提“二被告人具有自首情节，认罪认罚且签字具结，系初犯、偶犯”的辩护意见与本案查明事实相符，本院予以采纳，依法决定对被告人张玉权、刘周艳减轻处罚，并适用缓刑。公诉机关及指定辩护人所提量刑意见适当，本院予以采纳。

被告人张玉权、刘周艳的犯罪行为损害了社会公共利益，依法应当承担相应的民事责任。附带民事公益诉讼起诉人所提请求判令被告人张玉权、刘周艳赔偿自然资源损失补偿费人民币

40000 元、当庭进行赔礼道歉的诉请符合法律规定，且有有效证据予以证实，本院予以支持；被告人张玉权、刘周艳所提少赔补偿费的请求，本院不予采纳。

据此，根据本案事实、情节、社会危害性，依照《中华人民共和国刑法》第一百五十一条第二款、第二十五条第一款、第六十七条第一款、第七十二条第一款、第三款、第七十三条第二款、第三款、第六十四条、《中华人民共和国民事诉讼法》第五十五条第二款、《最高人民法院、最高人民检察院关于检察公益诉讼案件适用法律若干问题的解释》第二十条、《最高人民法院关于审理环境民事公益诉讼案件适用法律若干问题的解释》第十八条、《中华人民共和国刑事诉讼法》第十五条之规定，判决如下：

一、被告人张玉权犯走私珍贵动物制品罪，判处有期徒刑一年，缓刑一年零六个月，并处罚金人民币 2000 元。（缓刑考验期从判决确定之日起计算；罚金限于判决生效后五日内缴纳。）

二、被告人刘周艳犯走私珍贵动物制品罪，判处有期徒刑一年，缓刑一年零六个月，并处罚金人民币 2000 元。（缓刑考验期从判决确定之日起计算；罚金限于判决生效后五日内缴纳。）

三、随案移送的穿山甲甲片 495.38 克，予以没收。

四、由附带民事公益诉讼被告张玉权、刘周艳连带赔偿国家野生动物资源损失费人民币 40000 元。缴至澄江市财政局指定的账户，用作保护当地野生动物。（于本判决生效后五日内支付。）

五、由附带民事公益诉讼被告张玉权、刘周艳当庭向社会公众赔礼道歉。（本案系庭审直播，已经当庭履行。）

如不服本判决，可在接到判决书的第二日起十日内，通过本院或直接向云南省玉溪市中级人民法院提出上诉。书面上诉的，应当提交上诉状正本一份，副本四份。

审　判　长　　包广良

审　判　员　　溥喜燕

审　判　员　　刘美菊

人民陪审员　　吴金花

人民陪审员　　保玉莲

人民陪审员　　赵仕彬

人民陪审员　　王建寿

二〇二〇年十一月二十日

书　记　员　　余梓燕
