# Supplementary material for: China’s Legal Protection System for Pangolins: Past, Present, and Future
Source: Animals (Basel). 2025 Aug 18;15(16):2422. doi: 10.3390/ani15162422 (PMC12383201; doi:10.3390/ani15162422)
Supplement: Supplementary file 1 [file animals-15-02422-s001.zip › Supplementary Material S4-Full Text of Judgments in Pangolin-Related Public Interest Litigation Cases in China/【27】邹九月、王龙昌、吴位源非法收购、运输、出售珍贵、濒危野生动物、珍贵、濒危野生动物制品罪一案刑事一审判决书.pdf]

邹九月、王龙昌、吴位源非法收购、运输、出售珍贵、濒危野生动物、珍贵、濒危野生动物制品罪一案刑事一审判决书

广东省韶关市浈江区人民法院
刑 事 附 带 民 事 判 决 书

（2020）粤 0204 刑初 213 号

公诉机关暨附带民事公益诉讼起诉人：韶关市浈江区人民检察院。

被告人暨附带民事公益诉讼被告邹九月，男，汉族，1971 年 10 月 18 日出生，公民身份号码 362************819，小学文化，个体户，户籍地及住址广东省韶关市武江区*******************。2020 年 6 月 18 日因涉嫌犯非法收购、运输、出售珍贵、濒危野生动物罪被刑事拘留，同年 7 月 15 日被逮捕。现羁押于韶关市看守所。

辩护人暨委托诉讼代理人陈小雄、陈晓琴，广东众同信律师事务所律师。

被告人暨附带民事公益诉讼被告王龙昌，男，汉族，1962 年 8 月 22 日出生，公民身份号码 330************318，小学文化，个体户，户籍地及住址浙江省江山市***********。2020 年 6 月 9 日因涉嫌犯非法收购、运输珍贵、濒危野生动物罪被刑事拘留，同年*月 15 日被逮捕。现羁押于韶关市看守所。

辩护人暨委托诉讼代理人张海星，广东明沃律师事务所律师。

被告人暨附带民事公益诉讼被告吴位源，男，汉族，1970年2月24日出生，公民身份号码330************110，初中文化，个体户，户籍地浙江省江山市市区市后淤109幢302室，住浙江省江山市**********。2020年6月9日因涉嫌犯非法收购、运输、珍贵、濒危野生动物罪被刑事拘留，同年7月15日被逮捕。现羁押于韶关市看守所。

辩护人暨委托诉讼代理人黄飙，广东众同信律师事务所律师。

韶关市浈江区人民检察院以浈检刑诉〔2020〕43号起诉书，指控被告人邹九月犯非法收购、运输、出售珍贵、濒危野生动物、珍贵、濒危野生动物制品罪、诈骗罪、被告人王龙昌、吴位源犯非法收购、运输珍贵、濒危野生动物罪，于2020年9月29日向本院提起公诉，本院于2020年10月9日立案。在诉讼过程中韶关市浈江区人民检察院以浈检刑附民公诉〔2020〕1号刑事附带民事公益诉讼起诉书，向本院提起公益诉讼。经查，韶关市浈江区人民检察院于2020年8月6日公告了案件相关情况，公告期内未有法律规定的机关和有关组织提起民事公益诉讼。本院依法组成合议庭公开开庭进行了合并审理，韶关市浈江区人民检察院指派检察员李连东出庭履行职务，被告人邹九月、王龙昌、吴位

源及辩护人陈晓琴、张海星、黄飙到庭参加诉讼。本案现已审理终结。

公诉机关指控：（一）非法收购、运输、出售珍贵、濒危野生动物、珍贵、濒危野生动物制品

2013 年，被告人邹九月非法收购领角鸮活体一只用于泡酒，酒瓶存放在浈江区一市场漂布塘综合楼 B202 出租屋内。2020 年 6 月 9 日被公安机关查获。

2015 年，被告人邹九月非法收购赛加羚羊（又名高鼻羚羊）角两片及穿山甲鳞片若干，并将穿山甲鳞片藏匿于浈江区一市场漂布塘综合楼的 B202 出租屋内，将赛加羚羊角藏匿于武江区碧桂园太阳城天麓山 20 街 97 号住处。2020 年 6 月 9 日均被公安机关查获。

2020 年年初，被告人邹九月在五里亭市场非法收购穿山甲一只，6 月 8 日，被告人吴位源经被告人王龙昌介绍，从浙江来韶关，以 15000 元的价格向邹九月收购该穿山甲死体，6 月 9 日完成交易后，王龙昌、吴位源驾乘浙 H*****汽车将所购穿山甲运回浙江，途径韶关东收费站时被公安机关查获。

经鉴定，涉案穿山甲属国家一级重点保护野生动物，领角鸮（鸱鸮科）属国家二级重点保护野生动物，赛加羚羊属国家一级保护野生动物。根据《野生动物及其制品价值评估办法》（国家林业局令第 46 号）及《陆生野生动物基准价值标准目录》等相关规定，涉案穿山甲整体的价值为 80000 元，领角鸮整体的价值

为 15000 元。涉案穿山甲鳞片（1.369 千克）价值 25554.67 元，赛加羚羊角价值 5000 元。

（二）诈骗

2020 年 4 月 22 日，被告人邹九月从朱某文处购买两个猪胆，将该两个猪胆冒充为熊胆，以共 4500 元的价格出售给谢某营。

2020 年 6 月 8 日，被告人邹九月在向吴位源出售穿山甲时，以价格低廉的水牛角冒充价格昂贵的犀牛角，诱骗吴位源以 23000 元的价格购买。

韶关市浈江区人民检察院提起附带民事公益诉讼称：邹九月非法收购、运输、出售珍贵、濒危野生动物以及非法收购珍贵、濒危野生动物制品，王龙昌介绍他人非法收购珍贵、濒危野生动物以及运输珍贵、濒危野生动物，吴位源非法收购、运输珍贵、濒危野生动物，其三人的行为违反了《中华人民共和国野生动物保护法》的有关规定，其三人虽不是直接的猎捕者，但为猎捕野生动物提供了动机和市场，对生态环境损害具有直接的因果关系，导致野生动物数量减少，破坏了生态环境，损害了社会公共利益，应承担相应的民事责任。根据《中华人民共和国侵权责任法》第七条、第八条、第十三条、第十五条和《最高人民法院关于审理环境民事公益诉讼案件适用法律若干问题的解释》第十八条的规定，邹九月、王龙昌、吴位源应承担赔偿损失的民事责任。请求判令：1.判决邹九月、王龙昌、吴位源对因其三人共同侵权行为造成的生态资源损失（穿山甲一只）80000 元承担连带赔偿

责任；2.判决邹九月对因其非法收购珍贵、濒危野生动物（领角鸮一只）以及非法收购珍贵、濒危野生动物制品（穿山甲鳞片1.369千克、赛加羚羊角两片）造成的生态资源损失合计45554.67元承担赔偿损失的民事责任。

经审理另查明，2020年6月9日王龙昌、吴位源被抓获归案，同年6月18日，邹九月主动到公安机关投案。

被告人邹九月对公诉机关指控的犯罪事实无异议并自愿认罪认罚。对附带民事公益诉讼起诉人提出的诉讼请求无异议，表示愿意赔偿。

辩护人暨委托诉讼代理人提出如下意见：1.对公诉机关指控邹九月犯非法收购、运输、出售珍贵、濒危野生动物、珍贵、濒危野生动物制品罪无异议；2.邹九月的出售行为发生在疫情期间，但非法收购、运输珍贵野生动物及其制品的行为均非发生在疫情期间，应予以区分；3.邹九月系自首，认罪态度好；4.邹九月在收购穿山甲鳞片时，穿山甲仍属国家二级保护动物，不能按国家一级保护动物的标准对其进行处罚和赔偿；5.邹九月没有主观诈骗吴位源的故意，王龙昌与邹九月也不清楚牛角是否属于犀牛角，认定其诈骗故意的证据不足。附带民事公益诉讼部分：1.邹九月愿意赔偿生态资源损失，但主要赔偿责任主体是猎杀者而不是邹九月；2.邹九月收购穿山甲及其鳞片发生在穿山甲是国家二级保护动物期间，邹九月出售穿山甲的前几天才升为一级保护动物，应按二级保护动物计算生态资源损失更适当。

被告人王龙昌对公诉机关指控的犯罪事实无异议并自愿认罪认罚。对附带民事公益诉讼起诉人提出的诉讼请求无异议，表示愿意赔偿。

辩护人暨委托诉讼代理人提出如下意见：1.王龙昌只是进行居间介绍，只能认定其非法运输珍贵、濒危野生动物罪；2.王龙昌归案后如实供述，自愿认罪；3.被告人无前科；4.王龙昌是居间介绍的角色，仅起辅助性作用，应认定为从犯；5.王龙昌经营野生动物养殖，政府的工作需要其配合，应从保护民营企业的角度对其从轻处罚，并考虑对其适用缓刑。

被告人吴位源对公诉机关指控的犯罪事实无异议并自愿认罪认罚。对附带民事公益诉讼起诉人提出的诉讼请求无异议，表示愿意赔偿。

辩护人暨委托诉讼代理人提出如下意见：1.吴位源的主观恶性小，其是因妻子身体需要，故铤而走险购买野生动物；2.吴位源自愿认罪认罚，希望合议庭对其作出公正处罚。

上述指控及另查明的事实，有公诉机关提交并经法庭举证、质证、认证的受案登记表、立案决定书、到案经过、户籍证明、扣押决定书、扣押清单、扣押笔录、微信转账记录、称量笔录、称量照片、公告、证人黄某方、曾某、邹某华、邹某龙、邹某文、郑某菊、杨某婵、邹某长、邹某月、邹某坤、姜某清、朱某文、谢某营、陈某敏、毛某的、毛某慧证言、被告人邹九月、王龙昌、吴位源供述与辩解、韶关市林业局韶关市野生动物种类鉴定证

书、华南动物物种环境损害司法鉴定中心司法鉴定意见书、关于涉案野生动物组织价值的说明、价格认定结论书、现场勘验、检查笔录、指认照片、现场照片、辨认笔录等证据证实，足以认定。

对辩护人暨委托诉讼代理人提出的意见，本院综合评析如下：

1.被告人邹九月非法出售穿山甲的行为发生在穿山甲由国家二级重点保护动物调整为国家一级重点保护动物之后，以非法收购、运输、出售珍贵、濒危野生动物罪对其定罪量刑并无不当，对该辩护意见不予采纳，但对其非法收购穿山甲鳞片的行为在量刑时予以酌情考量。

2.辩护人关于邹九月诈骗吴位源主观故意证据不足的意见，经查，邹九月自述以几百元从地摊购买动物角，并称一眼就看出是水牛角，在与吴位源喝茶时主动拿出来试喝，在王龙昌称这是犀牛角时又并未否认，致使吴位源误以为是犀牛角，最终以每克230元进行交易，邹九月主观上具有非法占有的目的，对该辩护意见不予采纳。

3.被告人王龙昌居间介绍邹九月、吴位源非法买卖穿山甲，作用积极、主动，不应定为从犯，对辩护人相关辩护意见不予采纳；被告人邹九月在案发后主动投案，归案后如实供述自己的罪行，是自首，被告人王龙昌、吴位源归案后如实供述自己的罪行，三名被告人均自愿认罪认罚，依法可以分别从轻、从宽处罚，对

辩护人的相关辩护意见予以采纳。三名被告人在疫情期间实施妨害疫情防控的犯罪行为，应从重处罚。

4.关于赔偿穿山甲及其制品的生态资源损失应以基准价值五倍计算的意见，经查，穿山甲已被调整为国家一级重点保护动物，理应按照国家一级重点保护动物进行生态环境修复，附带民事诉讼被告也应赔偿相应的生态资源损失，对该辩护意见不予采纳。

本院认为，被告人邹九月、王龙昌、吴位源分别非法收购、运输、出售国家重点保护的珍贵、濒危野生动物，或者珍贵、濒危野生动物制品；被告人邹九月以非法占有为目的，虚构事实，隐瞒真相，骗取他人钱财，数额较大；被告人邹九月的行为构成非法收购、运输、出售珍贵、濒危野生动物、珍贵、濒危野生动物制品罪、诈骗罪，依法应实行数罪并罚；被告人王龙昌、吴位源的行为构成非法收购、运输珍贵、濒危野生动物罪。公诉机关对被告人邹九月、王龙昌、吴位源的指控，犯罪事实清楚，证据确实、充分，罪名成立，应予以认定。被告人邹九月、王龙昌、吴位源的行为，破坏了生态环境，损害了社会公共利益，刑事附带民事公益诉讼起诉人的诉讼请求，有相关部门出具的鉴定意见书、情况说明、价格认定结论书等证据予以证实，于法有据，应予支持。根据被告人的犯罪事实、犯罪性质、量刑情节及造成的社会危害后果，依照《中华人民共和国刑法》第三百四十一条第一款、第二百六十六条、第二十五条、第六十四条、第六十七条

第一、三款、最高人民法院《关于审理破坏野生动物资源刑事案件具体应用法律若干问题的解释》第一条及《中华人民共和国侵权责任法》第六条、第八条、第十五条及最高人民法院、最高人民检察院《关于检察公益诉讼案件适用法律若干问题的解释》第二十条之规定，判决如下：

一、被告人邹九月犯非法收购、运输、出售珍贵、濒危野生动物、珍贵、濒危野生动物制品罪，判处有期徒刑二年，并处罚金人民币二万元，犯诈骗罪，判处有期徒刑一年，并处罚金人民币二万元，数罪并罚，决定执行有期徒刑二年六个月，并处罚金人民币四万元。（刑期从判决执行之日起计算，判决执行以前先行羁押的，羁押一日折抵刑期一日，即从2020年6月18日起至2022年12月17日止。罚金限于本判决发生法律效力之日起十日内向本院缴交，上缴国库。）

二、被告人王龙昌犯非法收购、运输珍贵、濒危野生动物罪，判处有期徒刑一年，并处罚金人民币一万元。（刑期从判决执行之日起计算，判决执行以前先行羁押的，羁押一日折抵刑期一日，即从2020年6月9日起至2021年6月8日止。罚金限于本判决发生法律效力之日起十日内向本院缴交，上缴国库。）

三、被告人吴位源犯非法收购、运输珍贵、濒危野生动物罪，判处有期徒刑一年，并处罚金人民币一万元。（刑期从判决执行之日起计算，判决执行以前先行羁押的，羁押一日折抵刑期一日，

即从2020年6月9日起至2021年6月8日止。罚金限于本判决发生法律效力之日起十日内向本院缴交，上缴国库。）

四、附带民事公益诉讼被告邹九月、王龙昌、吴位源连带赔偿生态资源损失费用80000元、邹九月赔偿生态资源损失费用45554.67元，上述款项于本判决生效后十日内支付至韶关市浈江区财政局的公益诉讼案件赔偿金财政专账。

五、追缴被告人邹九月的违法所得人民币30000元、被告人王龙昌的违法所得人民币8000元，予以没收，上缴国库。

六、扣押的1只穿山甲（死体）、1只领角鸮（死体）、2片赛加羚羊角、1.369千克穿山甲鳞片，予以没收，由公安机关依法处理。

如不服本判决，可在接到判决书的第二日起十日内，通过本院或直接向广东省韶关市中级人民法院提出上诉，书面上诉的，应当提交上诉状正本一份，副本二份。

审　判　长　　杨　新

审　判　员　　王　伟

人民陪审员　　刘永德

二〇二〇年十月二十九日

书　记　员　　吴　迪
