# Supplementary material for: China’s Legal Protection System for Pangolins: Past, Present, and Future
Source: Animals (Basel). 2025 Aug 18;15(16):2422. doi: 10.3390/ani15162422 (PMC12383201; doi:10.3390/ani15162422)
Supplement: Supplementary file 1 [file animals-15-02422-s001.zip › Supplementary Material S4-Full Text of Judgments in Pangolin-Related Public Interest Litigation Cases in China/【28】李学哲非法收购、运输、出售珍贵、濒危野生动物、珍贵、濒危野生动物制品罪一审刑事判决书.pdf]

李学哲非法收购、运输、出售珍贵、濒危野生动物、珍贵、濒危野生动物制品罪一审刑事判决书

福建省漳平市人民法院

刑 事 附 带 民 事 判 决 书

（2020）闽 0881 刑初 197 号

公诉机关暨附带民事公益诉讼起诉人：福建省漳平市人民检察院。

被告人暨附带民事公益诉讼被告:李学哲，男，1976 年 11 月 5 日出生，朝鲜族，高中文化程度，务工，出生地黑龙江省宁安市，户籍地山东省威海市，住福建省漳平市。因涉嫌非法收购珍贵、濒危野生动物制品罪，于 2020 年 7 月 15 日被漳平市公安局取保候审，同年 8 月 3 日由漳平市人民检察院决定取保候审，2020 年 9 月 1 日由本院决定取保候审。现居家候审。

漳平市人民检察院以漳检检四刑诉[2020]12 号起诉书指控被告人李学哲犯非法收购珍贵、濒危野生动物制品罪，于 2020 年 8 月 31 日向本院提起公诉。公益诉讼起诉人漳平市人民检察院于 2020 年 9 月 24 日以漳检检四刑附民公诉[2020]5 号刑事附带民事公益诉讼起诉书向本院提起附带民事公益诉讼。经查，漳平市人民检察院于 2020 年 7 月 17 日公告了准备提起刑事附带民事公益诉讼的相关情况，公告期内未有法律规定的机关和组织提起诉讼。本院依法组成合议庭，于 2020 年 10 月 22 日公开开庭合并审理了本案。漳平市人民检察院指派检察员黄记超出庭支持

公诉，公益诉讼起诉人指派检察员游修林出庭履行职务。被告人暨附带民事公益诉讼被告李学哲到庭参加诉讼。本案现已审理终结。

公诉机关漳平市人民检察院指控：2019年10月25日，被告人李学哲在未经野生动物管理部门批准的情况下，向叶某（另案处理）购买一只穿山甲冻体等野生动物制品，并在叶某与李某合股经营的漳平“湘满楼”饭店加工，用于李学哲宴请他人食用，吃完后，李学哲付给叶某人民币20000元。经福建省鼎力司法鉴定中心鉴定：该野生动物冻体为国家二级保护野生动物——穿山甲（国家林业和草原局2020年第12号公告穿山甲为国家一级保护野生动物），价值人民币40000元。

公诉机关当庭提供了有关证据予以证实其所指控的犯罪事实，认为被告人李学哲违反野生动物保护法规，非法收购穿山甲冻体一只，其行为触犯了《中华人民共和国刑法》第三百四十一条第一款，犯罪事实清楚，证据确实、充分，应当以非法收购珍贵、濒危野生动物制品罪追究其刑事责任。被告人李学哲犯罪后自动投案，如实供述自己的罪行，是自首，可从轻处罚。被告人李学哲自愿认罪认罚，依照《中华人民共和国刑事诉讼法》第十五条的规定，可从宽处理。建议判处被告人李学哲有期徒刑六个月，缓刑一年，并处罚金人民币40000元。

附带民事公益诉讼起诉人漳平市人民检察院向本院提出诉讼请求：1、判令被告李学哲对叶某赔偿野生动物资源损失折合

人民币 40000 元承担连带清偿责任；2、判令被告李学哲在漳平市县级以上新闻媒体赔礼道歉。事实和理由：2019 年 10 月 25 日，被告人李学哲在未经野生动物管理部门批准的情况下，向叶某（另案处理）购买一只穿山甲冻体等野生动物制品，并在叶某与李某合股经营的漳平“湘满楼”饭店加工，用于李学哲宴请他人食用，吃完后，李学哲付给叶某人民币 20000 元。经福建省鼎力司法鉴定中心鉴定：该野生动物冻体为国家二级保护野生动物——穿山甲（国家林业和草原局 2020 年第 12 号公告穿山甲为国家一级保护野生动物），价值人民币 40000 元。认为，被告李学哲非法收购珍贵、濒危野生动物制品，其行为违反了《中华人民共和国野生动物保护法》第二十七条“禁止出售、购买、利用国家重点保护野生动物及其制品”的规定，根据《中华人民共和国野生动物保护法》第三条第一款的规定：“野生动物资源属于国家所有”，被告李学哲非法收购珍贵、濒危野生动物制品并经叶某（另案处理）与他人合股经营的饭店加工供他人食用的行为，破坏了野生动物资源，影响野生动物生态平衡，损害了国家和社会公共利益，造成国家野生动物资源损失，价值人民币 40000 元，根据《中华人民共和国侵权责任法》第四条第一款“侵权人因同一行为应当承担行政责任或者刑事责任的额，不影响依法承担侵权责任”、第十五条“承担侵权责任的方式主要有……（六）赔偿损失；（七）赔礼道歉；……”的规定，被告李学哲在承担刑事责任的同时，仍应承担民事赔偿责任，赔偿国家野生动物资源

损失，以保护国家野生动物资源，拯救珍贵、濒危野生动物，维护生物多样性和生态平衡，推进生态文件建设，维护国家和社会公共利益。同时认为，被告李学哲非法收购与叶某（另案处理）非法出售珍贵、濒危野生动物穿山甲冻体系二人共同实施了侵权行为，破坏了野生动物生态资源，造成国家和社会公共利益的损害。叶某（另案处理）非法出售珍贵、濒危野生动物穿山甲的冻体的行为，本院已以漳检检四刑附民公诉［2020］3号起诉书另案对叶某（另案处理）提起刑事附带民事诉讼。根据《中华人民共和国侵权责任法》第八条“二人以上共同实施侵权，造成他人损害的，应承担连带责任”的规定，被告李学哲应对叶某的侵权行为承担连带赔偿责任。综上，被告李学哲非法收购穿山甲制品，其行为触犯了《中华人民共和国刑法》第三百四十一条第一款的规定，以涉嫌非法收购珍贵、濒危野生动物制品罪，依法应当追究刑事责任，已向漳平市人民法院提起公诉，现根据《中华人民共和国刑事诉讼法》第一百零一条第二款、《中华人民共和国民事诉讼法》第五十五条、《最高人民法院、最高人民检察院＜关于检察公益诉讼案件适用法律若干问题的解释＞》第二十条的规定，特提起附带民事公益诉讼，请依法裁判。

针对诉求，公益诉讼起诉人提供了立案决定书、公告、请示报告批复；被告李学哲的户籍证明、被告李学哲的供述与辩解、辨认笔录；证人叶某、李某等人的证言和福建鼎力司法鉴定中心出具的司法鉴定意见书；漳平市公安局提取的银行转账记录、微

信聊天记录、本院漳检检四刑诉［2020］12起诉书等刑事案件证据材料。

被告人李学哲对公诉机关指控的犯罪事实和罪名均无异议，并签订了认罪认罚具结书，表示自愿认罪认罚。针对公益诉讼起诉人的诉请及所列举的事实与依据，附带民事公益诉讼被告李学哲亦无异议。

经审理查明，2019年10月25日，被告人李学哲在未经野生动物管理部门批准的情况下，向叶某（另案处理）购买一只穿山甲冻体等野生动物制品，并在叶某（另案处理）与李某合股经营的漳平“湘满楼”饭店加工，用于被告人李学哲宴请他人食用，之后，被告人李学哲付给叶某（另案处理）人民币20000元。经福建省鼎力司法鉴定中心鉴定：该野生动物冻体为国家二级保护野生动物—穿山甲（2020年6月3日，国家林业和草原局2020年第12号公告，穿山甲属所有种由国家二级保护野生动物调整为国家一级保护野生动物），价值人民币40000元。

另查明，2020年7月15日，被告人李学哲主动到漳平市公安局森林分局治安股投案。

再查明，叶某因违反野生动物保护法规，非法收购、出售国家二级保护野生动物-穿山甲冻体一只，2020年9月25日被漳平市人民法院以非法收购、出售珍贵、濒危野生动物制品罪，以（2020）闽0881刑初180号刑事附带民事判决书，判决：1、被告人叶某犯非法收购、出售珍贵、濒危野生动物制品罪，判处有

期徒刑六个月，缓刑一年，并处罚金人民币四万元；2、没收被告人叶某上缴漳平市公安局森林分局的违法所得款人民币一万九千零六十二元。该款由漳平市公安局森林分局负责上缴国库；3、附带民事公益诉讼被告叶某应自本判决生效之日起十五日内赔偿国家野生动物资源损失折合人民币四万元，并上缴国库；4、附带民事公益诉讼被告叶某应自本判决生效之日起十日内对其非法收购、出售珍贵、濒危野生动物制品，破坏野生动物生态资源，损害了国家和社会公共利益行为，在漳平市（县级以上新闻媒体向社会公开赔礼道歉。

上述事实，被告人暨附带民事公益诉讼被告李学哲在开庭审理过程亦无异议，且有漳平市公安局森林分局的情况说明等书证；证人叶某、李某、高某等人的证人证言；被告人李学哲的供述与辩解；福建鼎力司法鉴定中心出具的司法鉴定意见书；现场指认笔录、辨认笔录及现场照片等证据证实，足以认定。

本院认为，被告人李学哲违反野生动物保护法规，非法收购在实施犯罪行为时属国家二级保护野生动物-穿山甲冻体一只，其行为已构成非法收购珍贵、濒危野生动物制品罪，公诉机关指控的罪名成立，本院予以确认。被告人李学哲在审查起诉阶段自愿签署《认罪认罚具结书》，在提起公诉期间主动预缴了罚金，依法可以从宽处罚；被告人李学哲犯罪后自动投案，如实供述自己的罪行，是自首，可以从轻处罚。综上，公诉机关对被告人李

学哲建议适用行罚种类、量刑幅度及罚金金额建议适当，本院予以采纳。

附带民事公益诉讼被告李学哲非法收购珍贵、濒危野生动物制品的行为，破坏了野生动物生态资源，损害了国家和社会公共利益，违反了《中华人民共和国野生动物保护法》第三条第一款、第二十七条的规定，根据《中华人民共和国侵权责任法》第四条第一款、第十五条第一款第（六）项、第（七）项的规定，附带民事公益诉讼被告李学哲应按照法律规定承担民事赔偿等责任。最高人民法院及最高人民检察院《关于检察公益诉讼案件适用法律若干问题的解释》［法释（2018）6 号］第十三条第一款“人民检察院在履行职责中发现破坏生态环境和资源保护、食品药品安全领域侵害众多消费者合法权益等损害社会公共利益的行为，拟提起公益诉讼的，应当依法公告，公告期间为三十日。”、第二款“公告期满，法律规定的机关和有关组织不提起诉讼的，人民检察院可以向人民法院提起诉讼。”的规定，漳平市人民检察院在提起本案刑事附带民事公益诉讼前已依法履行了相应的公告程序，在公告期间内，无其他法律规定的适格机关或者组织提起诉讼，漳平市人民检察院作为公益诉讼起诉人提起附带民事公益诉讼，主体适格。依照《野生动物及其制品价值评估办法》（国家林业局令 46 号）第四条第一款“野生动物整体的价值，按照《陆生野生动物基准价值标准目录》所列该种野生动物的基准价值乘以相应的倍数核算”、第二款第（一）项“国家一级保护野

生动物，按照所列野生动物基准价值的十倍核算；国家二级保护野生动物，按照所列野生动物基准价值的五倍核算”的规定，被告李学哲实施非法收购穿山甲制品犯罪行为时，穿山甲属国家二级保护动物，《野生动物及其制品价值评估办法》（国家林业局令 46 号）附件《陆生野生动物基准价值标准目录》规定穿山甲科所有种基准价值为 8000 元，涉案野生动物穿山甲的整体价值为人民币 40000 元。同时，被告李学哲非法收购与叶某（另案处理）非法出售珍贵、濒危野生动物穿山甲冻体系二人共同实施了侵权行为，破坏了野生动物生态资源，造成国家和社会公共利益的损害。叶某非法出售珍贵、濒危野生动物穿山甲的冻体的行为，漳平市人民检察院以附带民事公益诉讼起诉人已另案提起刑事附带民事诉讼，漳平市人民法院另案已依法作出责令叶某赔偿的判决，目前叶某尚未履行赔偿义务。根据《中华人民共和国侵权责任法》第八条“二人以上共同实施侵权，造成他人损害的，应承担连带责任”的规定，被告李学哲应对叶某的侵权行为承担连带赔偿责任。综上，漳平市人民检察院提起本案刑事附带民事公益诉讼的诉讼请求，于法有据，本院予以支持。依照《中华人民共和国刑法》第三百四十一条第一款、第四十五条、第四十七条、第五十二条、第六十一条、第六十二条、第六十七条第一款、第七十二条、第七十三条第二款、第三款，《中华人民共和国刑事诉讼法》第十五条、第一百零一条第二款，《中华人民共和国侵权责任法》第四条第一款、第八条、第十五条第一款第（六）、

第（七）项，最高人民法院、最高人民检察院《关于检察公益诉讼案件适用法律若干问题的解释》第二十条之规定，判决如下：

一、被告人李学哲犯非法收购珍贵、濒危野生动物制品罪，判处有期徒刑六个月，缓刑一年，并处罚金四万元（罚金已缴纳）；

（缓刑考验期限，从判决确定之日起计算）

二、附带民事公益诉讼被告李学哲应自本判决生效之日起十五日内与叶某（另案处理）连带赔偿国家野生动物资源损失折合四万元，并上缴国库；

三、附带民事公益诉讼被告李学哲应自本判决生效之日起十五日内对其非法收购珍贵、濒危野生动物制品，破坏野生动物生态资源，损害国家和社会公共利益行为，在漳平市（县级以上新闻媒体向社会公开赔礼道歉。

如果未按本判决指定的期间履行给付金钱义务，应当依照《中华人民共和国民事诉讼法》第二百五十三条规定，加倍支付迟延履行期间的债务利息。赔礼道歉内容应先报本院审查，如附带民事公益诉讼被告李学哲不履行赔礼道歉义务，本院将依法代履行，依此所产生的相应费用由附带民事公益诉讼被告李学哲负担。

如不服本判决，可在接到判决书的第二日起十日内，通过本院或者直接向福建省龙岩市中级人民法院提出上诉。书面上诉的，应当提交上诉状正本一份，副本一份。

审　判　长　　朱隆武

审　判　员　　温英明

审　判　员　　洪亚香

人民陪审员　　朱桂静

人民陪审员　　赖家金

人民陪审员　　叶冬梅

人民陪审员　　林卫平

二〇二〇年十月二十二日

书　记　员　　林紫荔

附相关法律条文：

《中华人民共和国刑法》

第四十五条有期徒刑的期限，除本法第五十条、第六十九条规定外，为六个月以上十五年以下。

第四十七条有期徒刑的刑期，从判决执行之日起计算；判决执行以前先行羁押的，羁押一日折抵刑期一日。

第五十二条判处罚金，应当根据犯罪情节决定罚金数额。

第六十一条对于犯罪分子决定刑罚的时候，应当根据犯罪的事实、犯罪的性质、情节和对于社会的危害程度，依照本法的有关规定判处。

第六十二条犯罪分子具有本法规定的从重处罚、从轻处罚情节的，应当在法定刑的限度以内判处刑罚。

第六十七条犯罪以后自动投案，如实供述自己的罪行的，是自首。对于自首的犯罪分子，可以从轻或者减轻处罚。其中，犯罪较轻的，可以免除处罚。

被采取强制措施的犯罪嫌疑人、被告人和正在服刑的罪犯，如实供述司法机关还未掌握的本人其他罪行的，以自首论。

犯罪嫌疑人虽不具有前两款规定的自首情节，但是如实供述自己罪行的，可以从轻处罚；因其如实供述自己罪行，避免特别严重后果发生的，可以减轻处罚。

第七十二条对于被判处拘役、三年以下有期徒刑的犯罪分子，同时符合下列条件的，可以宣告缓刑，对其中不满十八周岁的人、怀孕的妇女和已满七十五周岁的人，应当宣告缓刑：

（一）犯罪情节较轻；

（二）有悔罪表现；

（三）没有再犯罪的危险；

（四）宣告缓刑对所居住社区没有重大不良影响。

宣告缓刑，可以根据犯罪情况，同时禁止犯罪分子在缓刑考验期限内从事特定活动，进入特定区域、场所，接触特定的人。

被宣告缓刑的犯罪分子，如果被判处附加刑，附加刑仍须执行。

第七十三条拘役的缓刑考验期限为原判刑期以上一年以下，但是不能少于二个月。

有期徒刑的缓刑考验期限为原判刑期以上五年以下，但是不能少于一年。

缓刑考验期限，从判决确定之日起计算。

第三百四十一条【非法猎捕、杀害珍贵、濒危野生动物罪】【非法收购、运输、出售珍贵、濒危野生动物、珍贵、濒危野生动物制品罪】非法猎捕、杀害国家重点保护的珍贵、濒危野生动物的，或者非法收购、运输、出售国家重点保护的珍贵、濒危野生动物及其制品的，处五年以下有期徒刑或者拘役，并处罚金；情节严重的，处五年以上十年以下有期徒刑，并处罚金；情节特别严重的，处十年以上有期徒刑，并处罚金或者没收财产。

【非法狩猎罪】违反狩猎法规，在禁猎区、禁猎期或者使用禁用的工具、方法进行狩猎，破坏野生动物资源，情节严重的，处三年以下有期徒刑、拘役、管制或者罚金。

《中华人民共和国刑事诉讼法》

第十五条犯罪嫌疑人、被告人自愿如实供述自己的罪行，承认指控的犯罪事实，愿意接受处罚的，可以依法从宽处理。

第一百零一条被害人由于被告人的犯罪行为而遭受物质损失的，在刑事诉讼过程中，有权提起附带民事诉讼。被害人死亡或者丧失行为能力的，被害人的法定代理人、近亲属有权提起附带民事诉讼。

如果是国家财产、集体财产遭受损失的，人民检察院在提起公诉的时候，可以提起附带民事诉讼。

《中华人民共和国侵权责任法》

第四条侵权人因同一行为应当承担行政责任或者刑事责任的，不影响依法承担侵权责任。

因同一行为应当承担侵权责任和行政责任、刑事责任，侵权人的财产不足以支付的，先承担侵权责任。

第八条二人以上共同实施侵权行为，造成他人损害的，应当承担连带责任。

第十五条承担侵权责任的方式主要有：

（一）停止侵害；

（二）排除妨碍；

（三）消除危险；

（四）返还财产；

（五）恢复原状；

（六）赔偿损失；

（七）赔礼道歉；

（八）消除影响、恢复名誉。

以上承担侵权责任的方式，可以单独适用，也可以合并适用。

最高人民法院最高人民检察院《关于检察公益诉讼案件适用法律若干问题的解释》

第二十条、人民检察院对破坏生态环境和资源保护、食品药品安全领域侵害众多消费者合法权益的犯罪行为提起刑事公诉

的，可以向人民法院一并提起附带民事公益诉讼，由人民法院同一审判组织审理。

人民检察院提起的刑事附带民事公益诉讼案件由审理刑事案件的人民法院管辖。
