# Supplementary material for: China’s Legal Protection System for Pangolins: Past, Present, and Future
Source: Animals (Basel). 2025 Aug 18;15(16):2422. doi: 10.3390/ani15162422 (PMC12383201; doi:10.3390/ani15162422)
Supplement: Supplementary file 1 [file animals-15-02422-s001.zip › Supplementary Material S4-Full Text of Judgments in Pangolin-Related Public Interest Litigation Cases in China/【2】古某飞走私珍贵动物制品案.pdf]

2023-06-1-082-001

古某飞走私珍贵动物制品案

——境外投案认定自首的实质把握和证据审查

关键词 刑事 走私珍贵动物制品罪 自首 如实供述 境外证据 证明资格

基本案情

公诉机关指控：被告人古某飞参与多起走私犯罪：（1）2019年12月，被告人古某飞与王某伟（已判决）、王某昆（已判决）合谋从越南走私穿山甲鳞片运至国内安徽等地销售牟利。古某飞负责提供货源。（2）2020年3月初，被告人古某飞与宋某昊、黄某、李某泰（均另案处理）合谋从越南走私穿山甲鳞片运至国内重庆后转运安徽等地销售牟利，古某飞负责提供货源，并雇请苏某海（已判决）负责组织人员在中越边境看路、接货，雇请刘某伦（已判决）组织人员运送及通过物流发运涉案穿山甲鳞片。（3）2020年6月7日，古某飞及其越南妻子“阮某”与农某龙（已判决）共谋将象牙制品从越南走私入境发运至天津，古某飞负责在越南提供货源，农某龙负责在广西南宁市后由接收并通过物流发运至古某飞指定的国内地址。公诉机关认为，被告人古某飞违反海关法规，逃避海关监管，伙同他人走私国家禁止进出口的珍贵动物制品入境，其行为已触犯了《中华人民共和国刑法》第151条第2款，犯罪事实清楚，证据确实、充分，应当以走私珍贵动物制品罪追究其刑事责任。

古某飞及其辩护人向法庭提交了称系越南公安机关出具《越南社会主义共和国报告（收到罪犯自我供认/认罪）》附翻译文书，拟证实古某飞系自动投案，具有自首情节。

经审理查明：被告人古某飞身居越南，但通过电话、微信等方式组

织、参与走私珍贵动物制品的犯罪活动，违反海关法规，逃避海关监管，伙同他人从越南走私穿山甲鳞片1443.16千克和象牙54.0871千克入境，经鉴定经济价值分别为11299.6048万元和225.3646万元。具体犯罪事实与公诉机关指控的相一致，已构成走私珍贵动物制品罪。

2021年5月7日，凭祥海关缉私分局在掌握古某飞涉嫌走私穿山甲片和象牙制品线索后，对其立案侦查，同时开具拘留证对古某飞上网追逃。2021年7月2日，越南警方将古某飞遣返回国。同日，中华人民共和国友谊关出入境边防检查站接收后将古某飞移交给凭祥海关缉私分局。古某飞以遣返方式到案，不属于自动投案，且到案后没有如实供述自己的主要犯罪事实，依法不构成自首。

广西壮族自治区崇左市中级人民法院于2022年3月9日作出（2021）桂14刑初79号刑事判决：被告人古某飞犯走私珍贵动物制品罪，判处无期徒刑，剥夺政治权利终身，并处没收个人全部财产。宣判后，被告人古某飞提起上诉。广西壮族自治区高级人民法院于2022年7月11日作出（2022）桂刑终123号刑事裁定：驳回上诉，维持原判。

裁判理由

被告人古某飞伙同他人走私国家禁止进口的珍贵动物制品，情节特别严重，其行为已构成走私珍贵动物制品罪。被告人古某飞不成立自首。经查：1.古某飞提供的《越南社会主义共和国报告（收到罪犯自我供认/认罪）》及越文版的来源不明，既未经所在国公证机关的证明或所在国中央外交主管机关或者其授权机关认证，也未经我国驻该国使领馆认证，不符合《最高人民法院关于适用〈中华人民共和国刑事诉讼法〉的解释》第77条第2款规定的证据形式要求，不能作为证据使用。2.凭祥海关缉私分局出具的《到案经过》《情况说明》《古某飞到案前电话记录说明》等证明，凭祥海关缉私分局在已经掌握古某飞涉嫌走私穿山甲鳞

片和象牙制品线索后，决定对古某飞立案侦查并网上追逃，古某飞被越南警方控制后遣返回国。古某飞归案后，在第一次讯问及《自述材料》中均供认，其回国接受凭祥海关缉私分局讯问后方得知其涉嫌走私珍贵动物制品，且辩称没有参与任何违法行为，只是从事翻译、接待工作，回国是为了解释清楚问题，直至一审庭审，古某飞仍不如实供述主要犯罪事实。综上，古某飞没有自动投案，到案后没有如实供述自己的主要犯罪事实，依法不构成自首，也不具有坦白情节。

裁判要旨

1.对境外在逃人员的自首认定，应结合自首制度的立法目的考虑在逃人员所处地域特殊性，对接受其投案的机关主体结合实际把握；“如实供述”是自首的构成要件，境外出逃人员到案后应如实供述主要犯罪事实，不能如实供述其主要犯罪事实，或者推翻其在境外的有罪供述的，不能认定为自首。

2.对于境外在逃人员及其辩护人、诉讼代理人提交的来自境外的证据材料，法院应根据《最高人民法院关于适用〈中华人民共和国刑事诉讼法〉的解释》第77条第2款之规定进行先审查形式之证据资格后审查内容之证明力的递进式审查。

关联索引

《中华人民共和国刑法》第67条、第151条第2款

《最高人民法院关于适用〈中华人民共和国刑事诉讼法〉的解释》第77条第2款

一审：崇左市中级人民法院（2021）桂14刑初79号刑事判决（2022年3月9日）

二审：广西壮族自治区高级人民法院（2022）桂刑终123号刑事裁定（2022年7月11日）

人民法院案例库

本案例文本已于2024年3月6日作出调整

人民法院案例库
