# Supplementary material for: China’s Legal Protection System for Pangolins: Past, Present, and Future
Source: Animals (Basel). 2025 Aug 18;15(16):2422. doi: 10.3390/ani15162422 (PMC12383201; doi:10.3390/ani15162422)
Supplement: Supplementary file 1 [file animals-15-02422-s001.zip › Supplementary Material S4-Full Text of Judgments in Pangolin-Related Public Interest Litigation Cases in China/【30】赖春城、徐北强、谢佛松等非法猎捕、杀害珍贵、濒危野生动物罪一案刑事一审判决书.pdf]

赖春城、徐北强、谢佛松等非法猎捕、杀害珍贵、濒危野生动物罪一案刑事一审判决书

广东省惠东县人民法院

刑 事 附 带 民 事 判 决 书

（2020）粤 1323 刑初 473 号

公诉机关暨附带民事公益诉讼起诉人惠东县人民检察院。

被告人暨附带民事公益诉讼被告：赖某城，男，1959 年 10 月 29 日出生，居民身份证号码 442************814，汉族，文盲，群众，户籍所在地广东省惠东县***********，因涉嫌非法猎捕珍贵、濒危野生动物罪，于 2020 年 4 月 8 日被抓获，次日被惠东县公安局刑事拘留；经惠东县人民检察院批准，于 2020 年 5 月 16 日被惠东县公安局执行逮捕。

辩护人暨委托诉讼代理人刁惠虹、何亮，北京市盈科（惠州）律师事务所律师。

被告人袁某良暨附带民事公益诉讼被告：男，1970 年 6 月 14 日出生，居民身份证号码 442************817，汉族，小学文化，群众，户籍所在地广东省惠东县***********，因涉嫌非法猎捕珍贵、濒危野生动物罪，于 2020 年 4 月 17 日被惠东县公安局刑事拘留；经惠东县人民检察院批准，于 2020 年 5 月 16 日被惠东县公安局执行逮捕。

辩护人叶朝标，惠东县司法和信访局公职律师。

辩护人朱宝莹，惠东县法律援助处律师。

被告人徐某强暨附带民事公益诉讼被告：男，1976 年 8 月 26 日出生，居民身份证号码 441***********514，汉族，小学文化，群众，户籍所在地广东省惠东县**********，因涉嫌非法猎捕珍贵、濒危野生动物罪，于 2020 年 4 月 9 日被抓获，次日被惠东县公安局刑事拘留；经惠东县人民检察院批准，于 2020 年 5 月 16 日被惠东县公安局执行逮捕。

辩护人陈惠良，广东商达（惠州）律师事务所律师。

被告人谢某松暨附带民事公益诉讼被告：男，1962 年 10 月 19 日出生，居民身份证号码 442***********61X，汉族，小学文化，群众，户籍所在地广东省惠东县**********，因涉嫌非法猎捕珍贵、濒危野生动物罪，于 2020 年 4 月 23 日被抓获，次日被惠东县公安局刑事拘留；经惠东县人民检察院批准，于 2020 年 5 月 16 日被惠东县公安局执行逮捕。

辩护人黄泽楚，广东伟伦律师事务所律师。

惠东县人民检察院以惠东检诉刑诉〔2020〕Z457 号起诉书指控被告人赖某城、袁某良、徐某强、谢某松犯非法猎捕珍贵、濒危野生动物罪，于 2020 年 8 月 14 日向本院提起公诉。公益诉讼起诉人惠东县人民检察院以惠东检民行刑附民公诉〔2020〕Z2 号起诉书向本院提起刑事附带民事公益诉讼。经查，惠东县人民检察院于 2020 年 5 月 18 日公告了案件相关情况，公告期内未有法律规定的机关和有关组织提起民事公益诉讼。本院适用普通程序，公开开庭审理了本案。惠东县人民检察院指派检察员王春光

出庭支持公诉，指派检察员肖致春、熊晓珊到庭参加公益诉讼。被告人赖某城、袁某良、徐某强、谢某松、辩护人刁惠虹、叶朝标、朱宝莹、陈惠良、黄泽楚到庭参加了诉讼，现已审理终结。

惠东县人民检察院指控：2019 年 4 月，被告人赖某城通过穿山甲保护活动认识了从事穿山甲物种种群及栖息地研究的华南师范大学教授吴某宝，并开始陆续发送其日常发现的穿山甲洞穴照片给吴以协助吴进行科学研究。2019 年 7 月份开始，赖某城组建“野生动物保护团队”，并相继邀请被告人袁某良、徐某强、谢某松及胡某新、蓝某洪、赖某生、陈某明、刘某红加入。该团队平时主要进行寻找穿山甲踪迹工作，协助吴某宝对该地的穿山甲种群及栖息地进行研究。

2019 年 11 月，赖某城、袁某良产生了通过猎捕穿山甲供吴某宝进行科学研究以获取奖金以及获取吴的信任以便日后当地若筹建穿山甲保护基地能够优先获得一份工作的想法。赖某城和袁某良向团队成员表达了该想法，要求团队成员加紧寻找穿山甲踪迹，拍摄穿山甲洞穴照片。不久，谢某松、徐某强向赖某城、袁某良报告称其二人在惠东县************凤彩坑山附近发现了几个新鲜的穿山甲洞穴。随后，四人一起上山查看发现的新鲜穿山甲洞穴，认为该洞穴可能有穿山甲出没，决定猎捕穿山甲。经商量决定由熟悉当地情况的谢某松和徐某强负责具体实施猎捕穿山甲，同时猎捕穿山甲的事情对团队其余五名成员保密。两日后，谢某松、徐某强将用烂布及塑料胶片改良过的“山猪剪”

及报警器等猎捕穿山甲的工具安装完毕后，将该情况通过电话报告了赖某城、袁某良。

2020 年 1 月上旬的一天，谢某松、徐某强通过“山猪剪”捕获一只幼年穿山甲，但发现时该幼年穿山甲已死亡并发臭。谢某松和徐某强将该情况报告赖某城和袁某良，赖某城让谢某松先将该幼年穿山甲尸体晾在现场通风去除臭味后取回。2020 年春节前后，赖某城将该穿山甲尸体埋藏在其家中菜园。

1 月 7 日凌晨 5 时许，谢某松、徐某强再一次通过“山猪剪”捕获一只成年穿山甲，向赖某城、袁某良汇报后将穿山甲带回徐某强家，赖某城、袁某良随即也赶到徐某强家。接着，赖某城、袁某良打电话给吴某宝，谎称其捡到穿山甲。四人商量统一口径对外宣称该穿山甲系在白盆珠镇坑屯村委增坑山捡到的，并决定将穿山甲带到坑屯村委袁某良家饲养。随后，该四人通知了团队其他五名成员。1 月 10 日，吴某宝指派其助理张某华及三名学生到白盆珠镇，对该穿山甲进行了为期三日的科学实验。实验过后，赖某城等人继续将穿山甲带回袁某良家中饲养，因照料不当该穿山甲于 1 月 23 日死亡。袁某良于是将该穿山甲尸体冷藏。4 月 5 日，袁某良听从赖某城的安排将冷藏的穿山甲尸体宰杀取出内脏及鳞片，并把穿山甲尸体带到白盆珠水库边埋藏，而取出的内脏则埋藏在其家中菜地。

4 月 8 日，公安机关于白盆珠镇横坑村委万潭村将被告人赖某城传唤至公安局接受调查。4 月 9 日，被告人徐某强到公安机

关投案。4月17日，公安机关于白盆珠镇坑屯村委将被告人袁某良传唤至公安局接受调查。4月23日，公安机关于宝口镇五一村委将被告人谢某松传唤至公安局接受调查。

经华南动物物种环境损害司法鉴定中心鉴定，两只涉案穿山甲确定为：哺乳纲（MAMMALIA）鳞甲目(PHOLIDOTA)穿山甲科(Manidae)穿山甲属(Manis)中华穿山甲(Manispentadactyla)的死体。根据国务院关于《国家重点保护野生动物名录》的批复(国函[1988]144号)附件所列《国家重点保护野生动物名录》规定中华穿山甲（Manispentadactyla)属于国家二级重点保护珍贵、濒危野生动物。

惠东县人民检察院向法庭递交了相关证据，认为被告人赖某城、袁某良、徐某强、谢某松未办理特许猎捕证，非法猎捕国家重点保护的珍贵、濒危野生动物，其行为触犯了《中华人民共和国刑法》第三百四十一条第一款之规定，犯罪事实清楚，证据确实、充分，应当以非法猎捕珍贵、濒危野生动物罪追究其刑事责任。建议判处被告人赖某城、袁某良有期徒刑七至十个月，并处罚金，建议判处被告人徐某强、谢某松有期徒刑六至九个月，并处罚金。提请本院依法判处。

公益诉讼起诉人向本院提起诉讼请求：

1.判令被告赖某城、袁某良、徐某强、谢某松共同承担因侵权行为造成的国家野生动物资源损失费80000元（支付至国家金库惠州市**支库，银行账号：191************001)；

2.判令被告赖某城、袁某良、徐某强、谢某松共同承担鉴定费用6900元（支付至国家金库惠州市**支库，银行账号：191***********001)；

3.判令被告赖某城、袁某良、徐某强、谢某松在市级以上电视台及全市范围内发行的报纸上公开道歉。

事实和理由：

2019年11月，赖某城、袁某良、谢某松、徐某强等人在惠东县宝口镇附近山坑处发现了新的穿山甲洞穴，为了非法获取利益，赖某城、袁某良、谢某松、徐某强共同商量决定猎捕穿山甲，并由熟悉当地情况的谢某松和徐某强负责猎捕。随后，四人在未取得合法的特许猎捕证的情况下，由谢某松、徐某强在山林里安装改良过的“山猪剪”及报警器等猎捕工具，安装完毕后，谢某松、徐某强将该情况报告给赖某城、袁某良。2020年1月上旬，谢某松、徐某强等人通过“山猪剪”捕获一只幼年穿山甲，但发现时该幼年穿山甲已死亡，后赖某城将该穿山甲尸体埋葬在其家中菜园。2020年1月7日凌晨5时许，谢某松、徐某强等人再次通过“山猪剪”捕获一只成年穿山甲，并将穿山甲带到袁某良家，后因照料不当该穿山甲于1月23日死亡。同年4月5日，袁某良听从赖某城的安排将穿山甲尸体宰杀取出内脏和鳞片，并把穿山甲的尸体带到白盆珠水库边埋葬，而取出的内脏则埋葬在自家菜地。

2020年4月28日、5月26日，经华南动物物种环境损害司法鉴定中心鉴定，两只涉案穿山甲确定为：哺乳纲(MAMMALIA)鳞甲目（PHOLIDOTA)穿山甲科（Manidae)穿山甲属(Manis)中华穿山甲（Manispentadactyla)的死体。中华穿山甲（Manispentadactyla)属于国家二级重点保护珍贵、濒危野生动物。两只穿山甲的整体价值为80000元，鉴定费用6900元。

附带民事公益诉讼起诉人惠东县人民检察院认为，赖某城、袁某良、徐某强、谢某松的行为违反了国家有关野生动物保护法律法规的规定，导致两只中华穿山甲死亡的结果，破坏了生物多样性和生态系统平衡，造成野生动物资源损失，国家利益和社会公共利益受到侵害。根据《中华人民共和国野生动物保护法》第三条、第六条、第二十一条、第二十四条、《中华人民共和国侵权责任法》第八条、第十五条以及最高人民法院《关于审理环境民事公益诉讼案件适用法律若干问题的解释》第十八条的规定，应当承担赔偿损失、赔礼道歉的民事侵权责任。因被告赖某城、袁某良、徐某强、谢某松的行为构成非法猎捕珍贵、濒危野生动物罪，依法应当追究刑事责任，本院已于2020年8月12日以惠东检诉刑诉[2020]Z457号起诉书向你院提起公诉。现根据《中华人民共和国民事诉讼法》第五十五条第二款、《最高人民法院、最高人民检察院关于检察公益诉讼案件适用法律若干问题的解释》第二十条的规定，向你院提起附带民事公益诉讼，请依法裁判。

被告人暨附带民事公益诉讼被告赖某城、袁某良、徐某强、谢某松对公诉机关指控的犯罪事实不持异议，对附带民事公益诉讼表示愿意赔偿经济损失。

辩护人暨委托诉讼代理人刁惠虹辩称，被告人赖某城积极赔偿损失，并表示愿意赔礼道歉，家属表示愿意缴纳罚金，归案后如实交代了自己的犯罪事实，认罪认罚，其猎捕穿山甲不是出于获利或者杀戳的目的，被告人赖某城是初犯、偶犯，依法可从轻处罚宣告缓刑。

辩护人叶朝标、朱宝莹辩称，被告人袁某良在共同犯罪中起次要作用，是从犯，其没有具体实施猎捕穿山甲，也是被赖某城纠集犯罪，袁某良主观恶性较小，归案后如实交代了自己的犯罪事实，认罪认罚，请求从轻处罚宣告缓刑。

辩护人陈惠良辩称，被告人徐某强有自首情节，自愿认罪认罚，在共同犯罪中被人纠集实施犯罪，请求从轻处罚。

辩护人黄泽楚辩称，被告人谢某松主观恶性小，不是出于食用猎捕穿山甲，谢某松在共同犯罪中起次要作用，是从犯，归案后如实交代了自己的犯罪事实，认罪认罚，请求从轻处罚适用缓刑。

经审理查明：2019 年 4 月，被告人赖某城通过穿山甲保护活动认识了从事穿山甲物种种群及栖息地研究的华南师范大学教授吴某宝，并开始陆续发送其日常发现的穿山甲洞穴照片给吴以协助吴进行科学研究。2019 年 7 月份开始，赖某城组建“野

生动物保护团队”，并相继邀请被告人袁某良、徐某强、谢某松及胡某新、蓝某洪、赖某生、陈某明、刘某红加入。该团队平时主要进行寻找穿山甲踪迹工作，协助吴某宝对该地的穿山甲种群及栖息地进行研究。

2019 年 11 月，赖某城、袁某良产生了通过猎捕穿山甲供吴某宝进行科学研究以获取奖金以及获取吴的信任以便日后当地若筹建穿山甲保护基地能够优先获得一份工作的想法。赖某城和袁某良向团队成员表达了该想法，要求团队成员加紧寻找穿山甲踪迹，拍摄穿山甲洞穴照片。不久，谢某松、徐某强向赖某城、袁某良报告称其二人在惠东县************凤彩坑山附近发现了几个新鲜的穿山甲洞穴。随后，四人一起上山查看发现的新鲜穿山甲洞穴，认为该洞穴可能有穿山甲出没，决定猎捕穿山甲。经商量决定由熟悉当地情况的谢某松和徐某强负责具体实施猎捕穿山甲，同时将猎捕穿山甲的事情对团队其余五名成员保密。两日后，谢某松、徐某强将用烂布及塑料胶片改良过的“山猪剪”及报警器等猎捕穿山甲的工具安装完毕后，将该情况通过电话报告了赖某城、袁某良。

2020 年 1 月上旬的一天，谢某松、徐某强通过“山猪剪”捕获一只幼年穿山甲，但发现时该幼年穿山甲已死亡并发臭。谢某松和徐某强将该情况报告赖某城和袁某良，赖某城让谢某松先将该幼年穿山甲尸体晾在现场通风处去除臭味后取回。2020 年春节前后，赖某城将该穿山甲尸体埋藏在其家中菜园。

1 月 7 日凌晨 5 时许，谢某松、徐某强再一次通过“山猪剪”捕获一只成年穿山甲，向赖某城、袁某良汇报后将穿山甲带回徐某强家，赖某城、袁某良随即也赶到徐某强家。接着，赖某城、袁某良打电话给吴某宝，谎称其捡到穿山甲。四人商量统一口径对外宣称该穿山甲系在白盆珠镇坑屯村委增坑山捡到的，并决定将穿山甲带到坑屯村委袁某良家饲养。随后，该四人通知了团队其他五名成员。1 月 10 日，吴某宝指派其助理张某华及三名学生到白盆珠镇，对该穿山甲进行了为期三日的科学实验。实验过后，赖某城等人继续将穿山甲带回袁某良家中饲养，因照料不当该穿山甲于 1 月 23 日死亡。袁某良于是将该穿山甲尸体冷藏。4 月 5 日，袁某良听从赖某城的安排将冷藏的穿山甲尸体宰杀取出内脏及鳞片，并把穿山甲尸体带到白盆珠水库边埋藏，而取出的内脏则埋藏在其家中菜地。

4 月 8 日，公安机关于白盆珠镇横坑村委万潭村将被告人赖某城传唤至公安局接受调查。4 月 9 日，被告人徐某强到公安机关投案。4 月 17 日，公安机关于白盆珠镇坑屯村委将被告人袁某良传唤至公安局接受调查。4 月 23 日，公安机关于宝口镇五一村委将被告人谢某松传唤至公安局接受调查。

经华南动物物种环境损害司法鉴定中心鉴定，两只涉案穿山甲确定为：哺乳纲（MAMMALIA）鳞甲目(PHOLIDOTA)穿山甲科(Manidae)穿山甲属(Manis)中华穿山甲(Manispentadactyla)的死体。根据国务院关于《国家重点保护野生动物名录》的批复(国

函[1988]144号)附件所列《国家重点保护野生动物名录》规定中华穿山甲（Manispentadactyla)属于国家二级重点保护珍贵、濒危野生动物。中华穿山甲基准价值每只8000元，整体价值，按照所列野生动物基准价值的五倍核算。2只中华穿山甲整体价值80000元。惠东县公安局森林分局支付鉴定费用6900元。

上述事实，有公诉机关和公益诉讼起诉人向法庭递交的下列证据证实：

1、受案登记表及立案决定书，证实案件来源及公安机关依法启动本案的侦查程序。

2、到案经过，证实4月8日，公安机关于白盆珠镇横坑村委万潭村将被告人赖某城传唤至公安局接受调查。4月9日，被告人徐某强到公安机关投案。4月17日，公安机关于白盆珠镇坑屯村委将被告人袁某良传唤至公安局接受调查。4月23日，公安机关于宝口镇五一村委将被告人谢某松传唤至公安局接受调查。

3、现场勘查笔录、方位图、现场照片及情况说明，证实案发地点及现场概况。

4、被告人供述

（1）被告人赖某城的供述和辨认，证实2019年农历11月下旬至12月初（具体日期记不清楚）某一天凌晨约5时许接到袁某良的电话，袁某良同我说，徐某强抓到穿山甲，叫我和他一起上宝口，然后我就起床等袁某良过来载我，没有多久，袁某良

就驾驶汽车到我家载我到宝口镇左坑村徐某强家老屋，到了徐某强家老屋时看见谢某松也在场，然后谢某松从徐某强老屋厨房对门的那间小房拿出用白色蛇皮袋装着的穿山甲出来，然后谢某松把穿山甲放出来，让穿山甲自行爬行，看是否有受伤，当时我和袁某良看见穿山甲并没有受伤，穿山甲大约有 10 斤重，是雄性穿山甲，之后袁某良就打电话给华南师范大学的吴教授没接通，我就再次拨打吴教授的电话，接通后，我就跟吴教授说抓到有穿山甲，因我不会普通话，我就把电话交给袁某良沟通，后打完电话袁某良就载上我和穿山甲，我就用衣服遮住穿山甲放在大腿上带到袁某良白盆珠镇坑屯村委的家，当时接近天亮，到了袁某良家之后，我就把穿山甲放在袁某良家二楼的卫生间，然后我就叫刘某红以及蓝姓朋友等人过来袁某良家看穿山甲，之后我们分工找白蚁喂食穿山甲，在饲养穿山甲过程中，我及胡某新、袁某良、徐某强，刘某红、蓝姓朋友、谢某松等人商量好一致说穿山甲是在白盆珠镇坑屯村委抓到的，是因为袁某良说要欺骗吴教授他们说是在坑屯村委抓到的穿山甲。约几天后张某华教授来到袁某良的家中，后我们带上穿山甲一起陪同张某华及其学生一起到坑屯村委做科研，做科研做了三天后学生准备回去并让我们把穿山甲放生，后我们把穿山甲带至袁某良家中饲养，之后我也没有见过穿山甲，约春节前后，袁某良打电话给我说，穿山甲死了并把死了的穿山甲冰冻起来，在我被公安机关抓获之前，我也去袁某良家中几次但是没有见过穿山甲。

这条穿山甲是徐某强及谢某松猎捕的，徐某强和我说是在宝口镇五一村委一座山梁上猎捕的，说是我们一起寻找穿山甲洞穴时的位置猎捕的，那个山名我不知道叫什么是谢某松带我及徐某强、袁某良三个人到五一村委山寻找过，在一座不知山名的山梁上，我们发现有几个有穿山甲轨迹的洞穴，我就提出用捕兽夹猎捕穿山甲，袁某良就说不要伤到穿山甲，徐某强就说用布包裹着捕兽夹就不会伤到穿山甲。我们回来后，徐某强及谢某松两人找到捕兽夹后再上山就将捕兽夹放置在穿山甲洞穴口。过了好多天，我就接到袁某良电话说抓到有穿山甲，事情经过就如上述所说。

约 2019 年 10 月份左右（具体日期我记不起）某一天中午，徐某强打电话给我说，有一条死了的穿山甲，问我怎么处理，我让他送到我家来，第二天早上徐某强和谢某松两人就用纸箱装着那只死了的穿山甲送到我家来，我让他们放在我家的炒茶房，徐某强就把包裹好的死了的穿山甲悬挂在炒茶房里，当时徐某强说那条死了的穿山甲有 2 斤至 3 斤重，雌雄我不清楚。约在我家悬挂有 10 多天，徐某强及谢某松又抓到上述所说的那条 10 多斤的穿山甲。春节前因为那条死了的穿山甲发臭，我就把它埋在我家的菜园里，现在去挖掘可能还能挖掘出来。因为我没有问徐某强及谢某松死了的那条穿山甲从何处来，他们也没有说从何处来，我不清楚死了的那条穿山甲从何处来。那条死了的穿山甲是如何

死亡的我不清楚，徐某强和谢某松只说捡到来的。我能指认我埋藏那条死了的穿山甲的位置。

我们共有 9 个人，他们是赖某生、徐某强、谢某松、袁某良、刘某红、陈某斗、蓝姓朋友、胡某新和我，是在 2019 年新历约 8 月份期间组成的，当时我认识吴教授及张教授等人，吴教授也叫我们去寻找穿山甲做研究，所以我们就组织在一起，当时我们也想从吴教授手里弄点钱，至今没有收取任何钱。猎捕穿山甲的工具在何处我不清楚，要徐某强和谢某松才知道。之前袁某良提出要猎捕穿山甲来给吴教授做科研，我们从中搞这个项目来做，赚点钱。因为我同袁某良等人商量好说，这条穿山甲是从坑屯村委捡到的，所以我一直坚持这个说法，是我的错，现在我如实交代所有事情。今日我所交代的是属实，之前交代的大部分都是假的，这次交代是真实的。

去年曾在袁某良家中我们 9 个人商量过想办法抓到穿山甲来弄钱，然后大家分钱，抓到穿山甲的人分多一点。我们是有一个团队，是大约 2019 年农历九月份左右成立的，与华南师范大学教授吴某宝教授合作的研究穿山甲的，我们团队的成员有我、袁某良、谢某松、徐某强、胡某新、赖某生、陈某明、蓝某洪、刘某红，我们团队平时主要是找穿山甲洞、拍摄穿山甲照片，提供穿山甲的踪迹给吴某宝教授等一系列保护穿山甲等活动。

辨认笔录，赖某城辨认出唐北强（徐某强）；蓝某洪；放置树弓索套猎捕穿山甲的袁某良；赖某生；谢某松；胡某新、刘某红、陈某明。

（2）被告人袁某良的供述，证实 2019 年 7 月 8 日，我和刘某红、胡某新三人驾车前往徐某强家玩，因徐某强听说赖某城在宝口镇佐坑村委野生动物保护基地工作，希望认识赖某城通过他的关系搞到保护基地做工，我知道同学刘某红认识赖某城，所以找同学刘某红、胡某新一起到徐某强家玩还约了赖某城。到徐某强家后，我和胡某新、徐某强认识了赖某城，徐某强就提出要加入赖某城的保护基地工作，赖某城没有作表态，说了基地的工作是寻找穿山甲踪迹，拍摄相关照片交给上级部门。我看赖某城的态度应该能安排我们到基地工作，于是就提出我们三同学也要此工作，赖某城表态你们看能否找到穿山甲踪迹再说。第二日，我找到了同村的蓝某洪，咨询了他关于穿山甲踪迹的信息，蓝某洪说坑屯增坑山就看过有穿山甲洞穴，我说你带我去找穿山甲洞穴，我支付你两百元人民币，蓝某洪同意并约定第二日一起到增坑山。第二日上午 7 时度，我和蓝某洪来到增坑山找到穿山甲洞穴，将洞穴拍摄照片给赖某城看，结果赖某城很满意，还说要我带他到现场看洞穴。几日后，蓝某洪和我带赖某城到增坑山看穿山甲洞穴，蓝某洪也是那时才认识赖某城的。看完洞穴后的一段时间，我和胡某新、刘某红为争取基地的工作就陪同赖某城到宝口镇国和村委了解穿山甲的踪迹信息，因为我与当地的村民有交

情。了解到国和村委的山林有穿山甲的踪迹后我们也不定时上山寻找，找到的穿山甲洞穴赖某城会发照片给一个吴某宝教授（当时是不认识）。断续工作至 9 月份期间，通过赖某城的关系，把吴某宝教授及专家带来了我家，然后我召集了刘某红、胡某新、蓝某洪，陪同吴某宝等人到增坑山对穿山甲洞穴做考察，吴某宝等人离开后，我和赖某城、刘某红、胡某新仍继续到宝口镇国和村找新的穿山甲洞穴，其中一日，赖某城在国和村的山林找到了穿山甲的粪便，第二日便叫我陪他一起到华南师范大学找吴某宝教授汇报，我们将穿山甲粪便交给了吴某宝教授做研究，吴某宝也安排学生接待我们，第二日我们就离开广州，离开时吴某宝转账人民币 2000 元给我作为我们的辛苦费．回到家后我除去乘车费用支付了约人民币 600 元给赖某城。回到家后我和胡某新、刘某红说了到过吴某宝的单位，觉得工作的事情有着落，于是更加积极协助赖某城找穿山甲踪迹，约一个月后，吴某宝再次带学生来我家，然后我和赖某城、胡某新、刘某红陪同吴某宝到国和村委查看检到粪便的穿山甲洞穴，吴某宝因身体不适没有上山，由与他一起的张某华上山考察，第二日吴某宝等人就离开了，我看吴某宝对找寻穿山甲踪迹很重视就对工作的事情更加有信心了。又过了一个月的时间，赖某城告诉我过几日中央及各省的专家要来增坑山对穿山甲洞穴进行考察，说安排在我家接待，专家们来到的当日，赖某城召集我、胡某新、刘某红、徐某强、谢某松、赖某生、蓝某洪等人到场陪同考察，当日离开后，我们八人就在

我家谈寻找穿山甲踪迹的事情，然后由赖某城安排分工，赖某城和我、刘某红、胡某新一组负责到国和村委的山林，徐某强、赖某生、谢某松负责宝口镇五一村的山林，蓝远红则被我安排负责坑屯村委的山林。考察团离开后因跟踪器断电的原因我还邀请了陈某明加入，但陈某明没有一起巡山林。直到 2020 年 1 月份上旬，徐某强打电话告诉我在宝口镇五一村的山林找到新的穿山甲洞穴，还将发现的情况报告给赖某城知道，第二日我驾自己的吉普车（车牌号记不清楚）载赖某城前往五一村与徐某强、谢某松会合，然后谢某松带我们志新发现的穿山甲洞穴，停车后步行约 30 分钟到达，到后通过徐某强的介绍，我就拍摄洞穴视频将情之兄介绍给吴某宝，然后四人坐在一旁喝水，赖某城开始对洞穴进行分析，认为这几日穿山甲会返回，还说要是能抓到活体穿山甲就最好，可以交给吴某宝及相关部门，然后折市场价格换取奖金，奖金大份安排给抓到的人，其余安排给队员（指我们寻穿山甲的九人），四人同意了赖某城的提议。接着大家又商量用什么方法抓穿山甲，赖某城就强调不能弄伤穿山甲，否则吴教授会有很大意见，于是赖某城就提出用捕兽夹来猎捕，并在夹子内缠布抓以免夹伤穿山甲，他还说了要考虑到防止破坏洞穴周边的草地导致穿山甲不会回来这个洞穴，猎捕不需要多人参与，因我和赖某城都不是宝口本地人，就决定由徐某强、谢某松两人上山猎捕穿山甲，商量完后我就和赖某城一起回家了。1 月 7 日凌晨 5 时左右，赖某城打电话给我，说徐某强和谢某松在五一村的山林里

猎捕到了穿山甲，让我接他前往徐某强家，我就立刻驾车前往赖某城家接他，接到他后就和他一起前往徐某强家，在开车前往徐某强家的路上徐某强通过微信发送了猎捕穿山甲的照片和视频给我，但照片和视频是我后来才看到的。我和赖某城到了徐某强家后，徐某强和谢某松已经回到徐某强家中了，然后徐某强就把穿山甲从蛇皮袋中取出，我拿出手机对穿山甲拍摄照片，同时察看穿山甲身上有无伤痕，穿山甲在地上行走时较为缓慢，紧接着徐某强又从房间内拿出电子秤对穿山甲进行称重，显示的重量是11.5斤。查看完穿山甲的情况后，我们就把穿山甲装回蛇皮袋内，赖某城让我打电话给吴某宝，但吴某宝没有接听，然后赖某城又用他的手机打给吴某宝，吴某宝就接了电话，接着他就把手机交给我由我同吴某宝交谈，我就向吴某宝汇报说捡到了活体穿山甲，并询问吴某宝诗宝如何处理，吴某宝回复我说他准备要做手术，说晚些再回复我具体如何处理。和吴某宝通完电话后，赖某城便和我们三人商量，说吴某宝问起穿山甲的来源，就统一回答说是我和赖某城在坑屯村的山上检到的，因为吴某宝和专家们都到过坑屯考察，说在坑屯捡到穿山甲影响力比较大，他还说我比较年轻，对外说是我看见后徒手检到的比较合适。这时候吴某宝就回电赖某城，说他会安排张某华教授带学生三日后到达惠东对穿山甲进行实验，结束通话后赖某城就说把穿山甲饲养在我家中，等待张某华和学生到来，我也同意将穿山甲饲养在我家中。说完之后我们四人就分别离开，赖某城和我共车回我家，他在副

驾驶座抱着穿山甲，经过马山省道的视频监控时，赖某城让我放慢车速并用外套挡住穿山甲。回到我家后，我把门前放置的铁笼拿回一楼卫生间处，并将穿山甲关在铁笼内，但又担心被其他人员看见，就把穿山甲转移至二楼的卫生间内饲养。当晚，我和赖某城就召集了徐某强、谢某松、刘某红、胡某新、蓝某洪、陈爱民到我家中，赖某城告诉他们，穿山甲是徐某强和谢某松在五一村的山林猎捕到的，为了应对日后可能遇到的调查，赖某城告诉他们要统一口径说是我和他在坑屯村增坑山检到穿山甲，有什么法律责任都由赖某城承担，猎捕穿山甲得到的奖金大部分都分给猎捕到穿山甲的人，其余的钱就由剩下的成员平分，但当时没有谈到具体的金额数字。

到了 1 月 10 日上午，张某华教授就带了三名学生（一男二女）来到我家，我和赖某城就召集所有成员在我家吃午饭，吃午饭后我们就陪同张某华教授和三名学生一起上到增坑山，搭起棚子准备开始做科学实验，实验开始前我们和三名学生及穿山甲一起拍了照片。张某华教授因为有事，上山查看了现场后就下山离开回广州了，留下三名学生在山上进行科学实验。为了进行实验，学生把穿山甲放回洞内，并放置探测仪器在洞口，科学实验进行了整整 72 小时，期间我们的成员有轮流上山陪同学生并给学生送水送饭，科学实验结束后，我们就协助学生把穿山甲从洞穴中挖出来，然后就带着穿山甲和学生一起下山回到我家中。回到我家后，三名学生在我家休息了一晚，就回去广州了，穿山甲则继

续饲养在我家中。穿山甲饲养在我家中，我有咨询张某华如何喂食穿山甲，也按照张某华的方法喂食了穿山甲，但穿山甲不怎么进食。到了 1 月 22 日晚，胡某新在我家帮忙喂食穿山甲，我拍摄了视频发到团队的微信群。1 月 23 日蓝某洪来我家帮忙喂食穿山甲时发现穿山甲死亡了，我就立刻打电话告诉赖某城，赖某城让我打电话询问吴某宝如何处理，我就打电话把事情告诉吴某宝，吴某宝让我与张某华联系，联系到张某华后，张某华告诉我先保存穿山甲的尸体，后续他们会过来取，于是我就把穿山甲的尸体放在家里的雪拒中急冻。一直存放到 4 月 2 日，张某华告诉我要带记者到坑屯穿山甲洞穴调查，就由我就陪同记者到坑屯山林进行调查，由赖某城陪同张某华，当晚张某华和记者就离开了。张某华和记者离开后赖某城告诉我说，张某华让我把穿山甲的内脏及小部分鳞片取出保存，他会抽空过来取走。在 4 月 5 日我就将穿山甲的内脏及部分鳞片取出，并分别保存在雪拒及床底，剩余的穿山甲尸体我就用两层白色保鲜袋装好，再套上一层白色蛇皮袋，然后我独自把尸体带到白盆珠水库边掩李里。到了 4 月 8 日我知道赖某城被公安机关抓获后，我就打电话给蓝某洪，想让他和胡某新把尸体转移到增坑山的穿山甲洞穴掩埋，以制造穿山甲是放生后才死亡的假象，但又考虑到日后如果被公安机关调查，交代不出尸体的位置会更不利于自己而作罢。于是我就打电话给我妻子蓝某珍，让她把穿山甲的内脏和鳞片埋到我家菜园里。到了 4 月 17 日，公安机关就到我家中把我传唤至惠东县公

安局森林分局。事情的经过就是这样。赖某城当时有提出用缠布的捕兽夹来猎捕穿山甲，但因为猎捕时我没有一起去，我不知道他们两人是否用赖某城所提出的方法来猎捕穿山甲。猎捕穿山甲是徐某强和谢某松去实施的，我和赖某城没有参与。胡某新、蓝某洪没有参与猎捕穿山甲。我与赖某城、徐某强、蓝某洪、胡某新、谢某松没有在增坑山猎捕穿山甲。我和赖某城等人没有用铁笼猎捕穿山甲赖某城是横坑人，蓝某洪是坑屯人，赖某城和蓝某洪是经过我介绍才认识的，他们两个应该互相不熟悉。我们团队的成员没有分到奖金。2020 年 4 月 22 日，公安机关带我到白盆珠水库边掩土里穿山甲处指认现场，并在现场查获被我掩埋的穿山甲尸体，另外当天公安机关也在我妻子协助下在我家菜园处查获被掩埋的穿山甲的内脏及鳞片。现穿山甲的尸体、内脏及鳞片都已被公安机关扣才甲。公安机关在白盆珠水库边查获的穿山甲尸体与在我家菜园处查获的内脏及鳞片是同属于一只穿山甲。

（3）被告人徐某强的供述，证实 2019 年农历 9 月份期间，我和谢某松、赖某生通过袁某良的介绍加入了赖某城织的寻找穿山甲踪迹的团队，工作是找穿山甲洞穴和安装跟踪器，有成效有可能得到保护野生动物基地的保护员工作，加入团队后知道队员有袁某良、陈某明、蓝某洪、刘某红、胡某新。期间我按照赖某城和袁某良的安排在惠东县辖区的山林了解穿山甲踪迹的信息和寻找穿山甲洞穴。至 11 月份，赖某城电话告知我，吴某宝及各省的专家近期要来坑屯村委增坑山做穿山甲洞穴考察，让我一

定要到场陪同。专家们来考察后的当日晚上，赖某城召集了我、袁某良、赖某生、蓝某洪、刘某红、胡某新、陈某明、谢某松共九人在袁某良家中商量事情。赖某城首先提出，说吴某宝教授需要活体穿山甲装跟踪芯片做研究。要求我们要抓到活体穿山甲，在抓的过程不可以搞伤穿山甲，袁某良附和说要抓到活体穿山甲。我听说不能搞伤穿山甲就提意见用山猪剪抓，在山猎剪两边用烂布包上再加一层塑料胶片就不会搞伤穿山甲，赖某城同意我的提议，抓到活体穿山甲的奖金大约有 40 至 50 万人民币，到时后抓到的人分多点，其他队员平分奖金，接着赖某城就安排我、谢某松、赖某生负责到宝口镇五一村的山林寻找，其他队员均机动作业，商量完后我们就各自回家。为了抓到穿山甲我从网上购买了五个报警器预备安装。在农历 11 月份期间（具体的时间记不清楚），我和谢某松在五一村的山林寻到了几个有穿山甲活动痕迹的洞穴，将发现的情况告诉赖某城和袁某良，第二日，赖某城和袁某良就赶来宝口镇五一村与我、谢某松会合，然后一起步行上山查看穿山甲洞穴，看完新的洞穴后我们四人还在山上商量，大家都认为穿山甲会返回洞穴，赖某城就说按照上次商量的抓方法抓穿山甲，还安排由我和谢某松尽快安装山猎剪抓捕穿山甲，我们三人均同意。两日后，我到谢某松家里与他一起准备山猎剪，将烂布及塑料胶片绑定到山猎剪，然后带上五个山猪剪步行上五一村的山林穿山甲洞穴，在山上我们将五个山猪剪分别装在五个不同的穿山甲洞穴，还配了我买的报警器（捕到猎物就会

向我的手机报警），其中捕到的两只穿山甲山猪剪均是我安装的，装完后我在山上就电话先后告知了赖某城和袁某良，然后就离开了。过了两日的中午，谢某松打电话告诉我，他在五一的山林2号报警器位置发现捕到了死体穿山甲，穿山甲已经发出臭味，问我要怎么处理。我打电话给袁某良，赖某城问他们的意见，赖某城告诉我让穿山甲留在山林晒，除去臭味后取回，我按他的要求告诉了谢某松。谢某松回复我是按要求作业，还把2号报警及山猪剪留在洞口。又过了五日后的凌晨，2020年1月7日（当日我发了微信视频给袁某良）凌晨4时30分度，我手机接到1号报警器的信号，我立即起床没有洗刷就骑摩托车赶去谢某松家，接上他后来到五一村的山脚步行上穿山甲洞穴位置，到洞穴后已经是凌晨5时度。看见1号报警器的山猪剪捕到了一只穿山甲，穿山甲左后脚被夹住，我上前将穿山甲取出，山猪剪和报警器扔在现场，然后就电话告知袁某良，并告诉他赖某城的电话打不通，袁某良回复我会联系赖某城一起赶上我家中会合，另外在山林的死体穿山甲也要带回．电话挂后我就将穿山甲放在草地上拍摄照片视频，并将照片视频微信发送给袁某良。接着，我用谢某松带来的蛇皮袋（白色）装好活体穿山甲，谢某松取回死体穿山甲一起下山，我把谢某松送到家后就回家了，谢某松将死体穿山甲（猎捕至死）处理好后才自己骑摩托车过来我家，据他说将死体穿山甲挂在家里的猪棚。谢某松来到我家后就一起等赖某城他们过来接收穿山甲。约7时度，袁某良驾一辆小轿车载了赖某城赶到我

家（什么牌子、车牌号我不清楚），我就对活体穿山甲进行称重，显示重量是 11.5 斤，然后赖某城就问我是不是查看的洞穴捕到的，我说是，赖某城就用电话打给吴某宝教授，说捡到了穿山甲要怎么处理，电话挂后，赖某城说吴某宝教授交代我们先饲养穿山甲，过几日他安排的学生过来对穿山甲进行研究，还说对来研究的学生专家们说是从坑屯增坑山捡的，因为原有专家们到增坑山做穿山甲洞穴研究，这样说影响力比较好，说完，赖某城就用原来的蛇皮袋将穿山甲装好与袁某良共车回坑屯村委袁某良家，我骑摩托车载谢某松走另一条路去袁某良家。到袁某良家后，穿山甲已经被关押在铁笼内放置在二楼的卫生间，队员们就陆续来到袁某良家，具体谁人通知他们过来我不清楚。九名队员到齐后，赖某城和袁某良均表态说穿穿山甲要说是从增坑山捡的，还要强调是赖某城和袁某良捡的，赖某城还表态，到位的关于活体穿山甲的奖金分配，我和谢某松抓的较辛苦，奖金相对分得较多，没有说具体金额，剩下的奖金就由其他七名队员平分，大家也同意了统一口径说是从增坑山捡来的穿山甲及奖金安排。商量完后我和谢某松就先行离开。三日后，我和谢某松接到袁某良的电话通知，要我们到增坑山陪同华南师范大学的学生做研究吞我们到场后看见学生在准备仪器，活体穿山甲也被带到现场，连续做研究约三日后我们九名队员按学生要求将穿山甲从洞内取回，然后由袁某良带回家继续饲养，在饲养期间，我没再看见穿山甲，只是在微信看过穿山甲的喂食视频（具体什么时间我记不清楚），后

来我问过袁某良穿山甲的去向，他说被吴教授他们带走了。一直到 2020 年农历 2 份期间，赖某城说吴某宝教授要来接收死体穿山甲，让我带去他家由他转交给吴某宝，我和谢某松按赖某城的要求将死体穿山甲用蛇皮袋装好送至他家，赖某城让我们将穿山甲死体挂在炒茶房，之后就没再看见死体穿山甲也没问赖某城穿山甲的去向。猎捕穿山甲的情况就是这样。我可以指认猎捕穿山甲的山猪剪。原有的供述是不真实的，当时想逃避法律责任所说才会这样供述，经过教育我认清了自己的行为是无法逃避，所以这次接受审查就如实的交代自己的行为，这次的供述没有虚假，均是事实，希望政府部门能对我从轻处理。猎捕穿山甲没有得到奖金。

死体穿山甲重约有 3 斤，身上没有明显伤痕，没发现其他特征。我们在袁某良商量时，赖某城提出我和谢某松在宝口五一村抓到活体穿山甲较辛苦，应分得较多的奖金。胡某新、赖某生、刘某红、蓝某洪、陈某明是知道由我和谢某松在五一村抓到的穿山甲，但具体怎么抓到他们应该不清楚。猎捕的死体穿山甲赖某城、袁某良、谢某松是知情的，其他队员知不知道我不肯定，我没有向其他队员说抓到死体穿山甲的事情。

（4）被告人谢某松的供述，证实农历 2019 年 10 月份在袁某良家开会时，赖某城说过猎捕到活体穿山甲会有钱，猎捕到活体穿山甲的人能分到大头，其余的钱就由剩余的团队成员平分，但当时没有说具体能拿到多少钱。后来在抓到穿山甲的第二天晚

上在袁某良家时，赖某城和袁某良就对我和徐某强说，是我们2人猎捕到了穿山甲，会在猎捕穿山甲得到的钱里抽出2万元人民币由我们2个人分，我和徐某强就说猎捕到穿山甲得到钱也有他和袁某良的功劳，应该由4个人平分。但直到现在我和徐某强也没有拿到钱。农历2019年10月我们团队9名成员集中在袁某良家开会时，当时赖某城有说过按照地域来分配找新穿山甲洞的工作，我和徐某强是负责在佐坑村委及五一村委周边寻找新穿山甲洞，赖某生是负责在兴家村委附近寻找新穿山甲洞，其他坑屯村委的成员如何分配我就记不清楚了。在开会以后，我大概找了4次才找到猎捕穿山甲的那个新穿山甲洞，当时是徐某强和我一起寻找到的。找到新的穿山甲洞后，徐某强就打电话通知了袁某良，第二天袁某良和赖某城在我、徐某强的带领下去到新穿山甲洞处查看，查看了洞穴后赖某城就说这个新穿山甲洞较有可能猎捕到穿山甲，袁某良和赖某城就让我尽快带捕兽夹来猎捕穿山甲。于是在查看完新穿山甲洞的第二天，我和徐某强就带了5个捕兽夹回到新穿山甲洞处放置捕兽夹。我和徐某强带了5个捕兽夹和3个报警器上山，在新穿山甲洞洞口回到新穿山甲洞处放置捕兽夹。周围穿山甲洞洞口放置并打开捕兽夹，并把捕兽夹连接上报警器（其中新穿山甲洞洞口放置的捕兽夹单独连接1个报警器，另外4个捕兽夹则是2个较靠近的捕兽夹共用1个报警器），布置好后我们就下山离开了。我在之前供述中说是我一个人上山且只放置了1个捕兽夹当时我记错了，这次供述的才是真实的情

况。猎捕到穿山甲当晚，我就把 3 个报警器都拆下带下山了，回到家后我担心直接扔掉报警器会被人发现，就在家把报警器烧掉了。捕兽夹比较重，就留在山上没有带走。公安机关带我到穿山甲洞处指认现场时，5 个捕兽夹都是打开的状态是因为我担心有人路过会发现这个猎捕的现场，而打开的捕兽夹比较低不容易被人发现，所以猎捕到穿山甲后，我就把猎捕到穿山甲的那个捕兽夹重新打开了，另外 4 个捕兽夹我也没有去合上。我和徐某强还在山上另外放置了 3 个捕兽夹，但那 3 个捕兽夹没有猎捕到穿山甲。在猎捕到活体穿山甲的前几天（具体日期记不清楚），我重新回到穿山甲洞处查看捕兽夹有无被其他动物或者树枝阻挡，发现有一个捕兽夹已经猎捕到一只小的穿山甲，重量大约有 3 斤左右，已经死亡并且发臭了，我就打电话把这个情况告诉徐某强，说死体穿山甲发臭不好带下山，打算放在山上风干一段时间等味道散去再带走，徐某强就转告给了赖某城和袁某良，然后他们两人都表示同意。我就用一个蛇皮袋（是当时上山布置捕牌夹时用来装捕兽夹的，布置完捕兽夹后就留在了现场）把死体穿山甲装起来，绑在树上风干。过了大约两天（还没有抓到那只较大的活体穿山甲），我就独自土山把死体穿山甲装进塑料袋里带下山，然后把死体穿山甲放在我家鸡棚内，这个情况我也告诉了徐某强、赖某城和袁某良。直到春节过后（大约是年初四左右，具体日期记不清楚），赖某城让我把死体穿山甲交给他，我就把死体穿山甲用塑料袋和蛇皮袋装好，和徐某强一起乘摩托车把死体穿

山甲送到他家。死体被赖某城带走后我就没有过问了，要赖某城才比较清楚。

5、证人吴某宝证言，证实我是在 2020 年 1 月 8 日左右听袁某良说在惠东白盆珠镇坑屯发现了一只活体穿山甲，于是在 1 月 10 日我就派张某华带领王某华、徐某娜、于某爽三名学生前往惠东县白盆珠镇坑屯*进行科学考察的。因为我个人身体不适，没有和张某华及三名学生一起前往惠东。王某华，男，年龄大约 25 岁，四川德阳人，联系电话 130*****580;徐某娜，女，年龄大约 24 岁，安徽人，联系电话 132*****261；于某爽，女，年龄大约 25 岁，辽宁大连人，联系电话 156*****869。

2020 年 1 月 8 日左右（具体时间记不清楚）袁某良打电话告诉我说，他在惠东白盆珠镇坑屯获得了一只活体穿山甲。当时袁某良说穿山甲是被倒下的按树砸到的，然后被他捡到带回家。他当时有告诉我在哪获得的穿山甲，但因为当时我在住院，且他的电话信号不好，我没听清他说的地方是哪里。我没有看到过袁某良获得的穿山甲活体。我在 1 月 8 日听到袁某良说惠东发现有活体穿山甲，于是在 1 月 10 日就派张某华教授和王某华、徐某娜、于某爽三名学生到惠东进行资料收集和科学考察。张某华教授送三名学生到惠东后当晚就回来了，三名学生就留在当地进行科学观察实验。2019 年 4 月份我们在一个保护穿山甲的活动上认识的赖某城和袁某良的。认识了他们两人后，他们偶尔会把在做农活中发现的穿山甲洞拍照发给我，以协助我们进行科学研

究，但是我和赖某城、袁某良之间没有签订协议或合同。我主要是与袁某良沟通比较多，袁某良普通话水平较好，方便和我们沟通，但赖某城偶尔也会和我进行交流。我让赖某城、袁某良把发现的穿山甲洞拍照后发给我们，并把位置记好，以便以后带我们去进行科学考察。我没有要求赖某城、袁某良等村民捕猎穿山甲用于科学实验。我没有提供科研经费或者报酬给赖某城和袁某良，但我们去到惠东进行科学考察时，会在袁某良家食宿，并且袁某良会开车送我们去科学考察，每次我都会给袁某良一些食宿费和车费，每次给的食宿费和车费大约为两千人民币。华南师范大学有同国家林业局野生动植物保护与自然保护区管理司签订中国鳞甲目动物（穿山甲）物种种群及栖息地现状专项调查的业务委托合同。我没有向广东省林业局办理穿山甲的特许捕猎证，因为我们没有打算捕猎穿山甲。王某华、徐某娜、于某爽三名学生在惠东坑屯山上完成科学观察实验后，穿山甲的去向我不清楚，因为三名学生回到学校后我还在住院，不久之后就放寒假了，所以没来得及向学生了解相关情况。袁某良、赖某城事后也没有告诉我穿山甲的去向。在袁某良告诉我发现穿山甲时，他就提出要放生穿山甲，我也有提醒他要放生穿山甲。王某华、徐某娜、于某爽三名学生没有把穿山甲带回华南师范大学。

6、鉴定意见

（1）华南动物物种环境损害司法鉴定中心《动物物种初步鉴定意见》，证实疑似穿山甲死体 1 只、疑似穿山甲内脏 1 副、疑似穿山甲鳞厅 370 克。是穿山甲属物种死体 1 只。

（2）华南动物物种环境损害司法鉴定中心《关于华动司鉴字[2020]第 541 号司法鉴定意见书中涉案动物价值的说明》，证实惠东县公安局森林分局于 2020 年 4 月 23 日委托鉴定疑似穿山甲死体 1 只、疑似穿山甲内脏 1 副、疑似穿山甲鳞片 1 批,我中心出具了相应的司法鉴定意见书(华动司鉴字[2020]第 541 号),关于司法鉴定意见书中涉案动物的价值说明如下。

根据国务院关于《国家重点保护野生动物名录》的批复(国函[1988]144 号)规定中华穿山甲(Manispentadacyla)属于国家二级重点保护的珍贵、濒危野生动物。

根据《野生动物及其制品价值评估方法》(国家林业局令第 46 号，自 2017 年 12 月 15 日起施行)第四条规定:国家二级保护野生动物的整体价值,按照所列野生动物基准价值的五倍核算。

根据《野生动物及其制品价值评估方法》(国家林业局令第 46 号，自 2017 年 12 月 15 日起施行)中规定中华穿山甲(Manispentadactyla)的基准价值均为 8000 元/只。

（3）华南动物物种环境损害司法鉴定中心《司法鉴定意见书》和说明，证实 1.送检的疑似穿山甲死体，编为 1 号，经鉴定确定为:哺乳纲（MAMMALIA）鳞甲目(PHOLIDOTA)穿山甲科

(Manidae）穿山甲属(Manis)中华穿山甲(Manispentadactyla)的死体。

2.送检的疑似穿山甲内脏1副,编为2号,经鉴定确定为:哺乳纲（MAMMALIA）鳞甲目(PHOLIDOTA)穿山甲科(Manidae）穿山甲属(Manis)中华穿山甲(Manispentadactyla)的组织。

3.送检的疑似穿山甲鳞片1批,编为3号,净重370克,经鉴定确定均为:哺乳纲（MAMMALIA）鳞甲目(PHOLIDOTA)穿山甲科(Manidae）穿山甲属(Manis)中华穿山甲(Manispentadactyla)的鳞片。

根据国务院关于《国家重点保护野生动物名录》的批复(国函[1988]144号)附件所列《国家重点保护野生动物名录》规定中华穿山甲（Manispentadactyla)属于国家二级重点保护珍贵、濒危野生动物。

根据《濒危野生动植物种国际贸易公约》《CITES)附录（2019年版)规定中华穿山甲(Manispentadactyla)被列入《濒危野生动植物种国际贸易公约》(CITES)附录I。

4.华南动物物种环境损害司法鉴定中心《司法鉴定意见书》,证实送检的疑似穿山甲死体1只,已腐臭,经鉴定确定为:哺乳纲MAMMALIA)鳞甲目(PHOLIDOTA)穿山甲科(Manidae)穿山甲属(Manis)中华穿山甲(Manispentadactyla)的死体。

根据国务院关于《国家重点保护野生动物名录》的批复(国函[1988]144号)附件所列《国家重点保护野生动物名录》规定

中华穿山甲（Manispentadactyla)属于国家二级重点保护珍贵、濒危野生动物。

根据《濒危野生动植物种国际贸易公约》《CITES)附录(2019年版)规定中华穿山甲(Manispentadactyla)被列入《濒危野生动植物种国际贸易公约》

7、惠东县人民检察院在正义网的《公告》（刊登时间：2020年5月18日），证实惠东县人民检察院履行诉前程序，于2020年5月18日在正义网刊登公告，督促法律规定的职能机关和社会组织提起诉讼，自公告之日起三十日内相关的职能机关和社会组织均未提起诉讼，社会公共利益仍然处于受侵害状态，惠东县人民检察院提起公益诉讼的主体适格。

8、发票，证实惠东县公安局森林分局为鉴定支付鉴定费6900元。

上述证据，均经庭审举证、质证，来源合法，内容真实，本院予以认定。

对辩护人辩解的意见，本院评判如下：

1、关于本案被告人袁某良、徐某强、谢某松是否是从犯问题，经查，本案赖某城、袁某良、徐某强、谢某松先商量猎捕穿山甲，具体由徐某强、谢某松负责猎捕，猎捕后向赖某城、袁某良报告，并将其中一只放到袁某良家中饲养。四被告人在共同犯罪中分工不同，作用相当，不是本案从犯。

2、关于辩护人提到对被告人适用缓刑问题。经查，四被告人非法猎捕野生动物并造成两只穿山甲死亡，给生态环境造成损害，不属于情节较轻，不宜适用缓刑。

3、被告人徐某强是否构成自首问题。经查，被告人徐某强归案后前五次讯问笔录未如实交代犯罪事实，不符合自首需如实交代犯罪事实的条件，但其主动投案可作为量刑情节考虑。

综上，对辩护人的相关辩护意见，本院不予采纳。

本院认为，被告人赖某城、袁某良、徐某强、谢某松未办理特许猎捕证，非法猎捕国家重点保护的珍贵、濒危野生动物，其行为构成非法猎捕珍贵、濒危野生动物罪，公诉机关指控的被告人犯罪事实清楚，证据确实充分，罪名成立，本院予以支持。鉴于被告人不是以捕食为目的猎捕野生动物，归案后如实交代了自己的犯罪事实，认罪认罚，依法可从宽处罚，公诉机关的量刑建议与其罪行相符，本院予以采纳。

被告人赖某城、袁某良、徐某强、谢某松非法猎捕野生动物导致野生动物死亡，应承担赔偿责任，按照鉴定机构的说明，中华穿山甲属于国家二级重点保护的珍贵、濒危野生动物，整体价值按照所列野生动物基准价值的五倍核算。公益诉讼起诉人要求四被告人赔偿国家野生动物资源损失费 80000 元和鉴定费 6900 元的诉讼请求，本院予以支持。四被告人的行为破坏生态，公益诉讼起诉人要求四被告人公开赔礼道歉的诉讼请求，本院予以支持。综上，结合各被告人在共同犯罪中的作用、地位、归案后的

认罪态度等情节，依照《中华人民共和国刑法》第三百四十一条第一款、第六十七条第三款、第六十四条、第五十二条、第五十三条及《最高人民法院关于适用财产刑若干问题的规定》第五条以及《中华人民共和国野生动物保护法》第三条、第六条、第二十一条、第二十四条、《中华人民共和国侵权责任法》第八条、第十五条以及最高人民法院《关于审理环境民事公益诉讼案件适用法律若干问题的解释》第十八条之规定，判决如下：

一、被告人赖某城犯非法猎捕珍贵、濒危野生动物罪，判处有期徒刑九个月，并处罚金人民币一万元。

（刑期从判决执行之日起计算，判决执行以前先行羁押的，羁押一日折抵刑期一日，即自2020年4月8日起至2021年1月7日止；罚金已缴清。）

二、被告人袁某良犯非法猎捕珍贵、濒危野生动物罪，判处有期徒刑七个月，并处罚金人民币一万元。

（刑期从判决执行之日起计算，判决执行以前先行羁押的，羁押一日折抵刑期一日，即自2020年4月17日起至2020年11月16日止；罚金已缴清。）

三、被告人徐某强犯非法猎捕珍贵、濒危野生动物罪，判处有期徒刑六个月，并处罚金人民币一万元。

（刑期从判决执行之日起计算，判决执行以前先行羁押的，羁押一日折抵刑期一日，即自2020年4月9日起至2020年10月8日止；罚金已缴清。）

四、被告人谢某松犯非法猎捕珍贵、濒危野生动物罪，判处有期徒刑六个月，并处罚金人民币一万元。

（刑期从判决执行之日起计算，判决执行以前先行羁押的，羁押一日折抵刑期一日，即自2020年4月23日起至2020年10月22日止；罚金已缴清。）

五、被告人赖某城、袁某良、徐某强、谢某松共同赔偿国家野生动物资源损失费80000元和鉴定费6900元（支付至国家金库惠州市**支库，银行账号：191************001）。（该款已预交至本院执行款账户）

六、被告人赖某城、袁某良、徐某强、谢某松于本判决生效后十日内，在惠州日报或惠州电视台发表经本院认可的赔礼道歉声明。

七、随案移送的作案工具手机八部，予以没收上缴国库，扣押的其他作案工具由惠东县公安局没收销毁。

如不服本判决，可在接到判决书的第二日起十日内，通过本院或者直接向广东省惠州市中级人民法院提出上诉，书面上诉的，应当提交上诉状正本一份，副本二份。

审判长　　许小龙

审判员　　刘惠霞

审判员　　周凤珠

二〇二〇年九月二十三日

书记员　　林志敏

附法律条文：

《中华人民共和国刑法》

第三百四十一条非法猎捕、杀害国家重点保护的珍贵、濒危野生动物的，或者非法收购、运输、出售国家重点保护的珍贵、濒危野生动物及其制品的，处五年以下有期徒刑或者拘役，并处罚金；情节严重的，处五年以上十年以下有期徒刑，并处罚金；情节特别严重的，处十年以上有期徒刑，并处罚金或者没收财产。

违反狩猎法规，在禁猎区、禁猎期或者使用禁用的工具、方法进行狩猎，破坏野生动物资源，情节严重的，处三年以下有期徒刑、拘役、管制或者罚金。

第六十七条犯罪以后自动投案，如实供述自己的罪行的，是自首。对于自首的犯罪分子，可以从轻或者减轻处罚。其中，犯罪较轻的，可以免除处罚。

被采取强制措施的犯罪嫌疑人、被告人和正在服刑的罪犯，如实供述司法机关还未掌握的本人其他罪行的，以自首论。

犯罪嫌疑人虽不具有前两款规定的自首情节，但是如实供述自己罪行的，可以从轻处罚；因其如实供述自己罪行，避免特别严重后果发生的，可以减轻处罚。

第六十四条犯罪分子违法所得的一切财物，应当予以追缴或者责令退赔；对被害人的合法财产，应当及时返还；违禁品和供犯罪所用的本人财物，应当予以没收。没收的财物和罚金，一律上缴国库，不得挪用和自行处理。

第五十二条判处罚金，应当根据犯罪情节决定罚金数额。

第五十三条罚金在判决指定的期限内一次或者分期缴纳。期满不缴纳的，强制缴纳。对于不能全部缴纳罚金的，人民法院在任何时候发现被执行人有可以执行的财产，应当随时追缴。

由于遭遇不能抗拒的灾祸等原因缴纳确实有困难的，经人民法院裁定，可以延期缴纳、酌情减少或者免除。

《中华人民共和国野生动物保护法》

第三条野生动物资源属于国家所有。

国家保障依法从事野生动物科学研究、人工繁育等保护及相关活动的组织和个人的合法权益。

第六条任何组织和个人都有保护野生动物及其栖息地的义务。禁止违法猎捕野生动物、破坏野生动物栖息地。

任何组织和个人都有权向有关部门和机关举报或者控告违反本法的行为。野生动物保护主管部门和其他有关部门、机关对举报或者控告，应当及时依法处理。

第二十一条禁止猎捕、杀害国家重点保护野生动物。

因科学研究、种群调控、疫源疫病监测或者其他特殊情况，需要猎捕国家一级保护野生动物的，应当向国务院野生动物保护主管部门申请特许猎捕证；需要猎捕国家二级保护野生动物的，应当向省、自治区、直辖市人民政府野生动物保护主管部门申请特许猎捕证。

第二十四条禁止使用毒药、爆炸物、电击或者电子诱捕装置以及猎套、猎夹、地枪、排铳等工具进行猎捕，禁止使用夜间照明行猎、歼灭性围猎、捣毁巢穴、火攻、烟熏、网捕等方法进行猎捕，但因科学研究确需网捕、电子诱捕的除外。

前款规定以外的禁止使用的猎捕工具和方法，由县级以上地方人民政府规定并公布。

《中华人民共和国侵权责任法》

第八条二人以上共同实施侵权行为，造成他人损害的，应当承担连带责任。

第十五条承担侵权责任的方式主要有：

（一）停止侵害；

（二）排除妨碍；

（三）消除危险；

（四）返还财产；

（五）恢复原状；

（六）赔偿损失；

（七）赔礼道歉；

（八）消除影响、恢复名誉。

以上承担侵权责任的方式，可以单独适用，也可以合并适用。

《最高人民法院关于适用财产刑若干问题的规定》第五条刑法规定第五十三条规定的“判决指定的期限”应当在判决书中予

以确定；“判决指定的期限”应为从判决发生法律效力第二日起最长不超过三个月。

最高人民法院《关于审理环境民事公益诉讼案件适用法律若干问题的解释》第十八条对污染环境、破坏生态，已经损害社会公共利益或者具有损害社会公共利益重大风险的行为，原告可以请求被告承担停止侵害、排除妨碍、消除危险、恢复原状、赔偿损失、赔礼道歉等民事责任。
