# Supplementary material for: China’s Legal Protection System for Pangolins: Past, Present, and Future
Source: Animals (Basel). 2025 Aug 18;15(16):2422. doi: 10.3390/ani15162422 (PMC12383201; doi:10.3390/ani15162422)
Supplement: Supplementary file 1 [file animals-15-02422-s001.zip › Supplementary Material S4-Full Text of Judgments in Pangolin-Related Public Interest Litigation Cases in China/【32】吕东明、陈小佳非法收购、运输、出售珍贵、濒危野生动物、珍贵、濒危野生动物制品一审刑事判决书.pdf]

吕东明、陈小佳非法收购、运输、出售珍贵、濒危野生动物、珍贵、濒危野生动物制品一审刑事判决书

广东省深圳市罗湖区人民法院

刑 事 判 决 书

（2020）粤0303刑初265号

公诉机关深圳市罗湖区人民检察院。

被告人吕东明，男，1978年4月9日出生，汉族，高中文化，户籍所在地广东省，捕前住本市罗湖区。因本案，于2019年8月8日被传唤，次日被刑事拘留，同年9月11日被逮捕。现羁押于深圳市罗湖区看守所。

辩护人季文梅，深圳市罗湖区法律援助处指派广东诚公律师事务所律师。

被告人陈小佳，曾用名陈小加，男，1983年8月5日出生，汉族，小学文化，户籍所在地广东省陆丰市，捕前住本市罗湖区。因本案，于2019年8月8日被传唤，次日被刑事拘留，同年9月11日被逮捕。现羁押于深圳市罗湖区看守所。

辩护人罗广军，广东宝源律师事务所律师。

被告人胡永水，男，1965年11月29日出生，汉族，小学文化，户籍所在地安徽省芜湖市南陵县，捕前住本市宝安区。因本案，于2019年8月8日被传唤，次日被刑事拘留，同年9月11日被逮捕。现羁押于深圳市罗湖区看守所。

辩护人杨军福，广东鹏浩律师事务所律师。

被告人叶再坤，男，1968年10月16日出生，汉族，小学文化，户籍所在地福建省漳州市诏安县，捕前住本市龙华新区。因犯贩卖淫秽物品牟利罪，于2004年11月8日被福建省诏安县人民法院判处有期徒刑三年，缓刑五年。因本案，于2019年8月12日被传唤，次日被刑事拘留，同年9月11日被逮捕。现羁押于深圳市罗湖区看守所。

辩护人杨**晶，广东赛维律师事务所律师。

被告人沈少君，男，1967年10月25日出生，汉族，中专文化，户籍所在地广东省深圳市龙岗区，现住地本市罗湖区。因本案，于2019年8月8日被传唤，次日被刑事拘留，同年9月11日被取保候审。

辩护人周杨，深圳市罗湖区法律援助处指派广东华商律师事务所律师。

深圳市罗湖区人民检察院以深罗检刑诉〔2020〕316号起诉书指控被告人吕东明犯非法收购、运输、出售珍贵、濒危野生动物罪，被告人陈小佳犯非法运输、出售珍贵、濒危野生动物罪，被告人胡永水、叶再坤犯非法收购、出售珍贵、濒危野生动物罪，被告人沈少君犯非法收购珍贵、濒危野生动物罪，于2020年3月30日向本院提起公诉。本院依法组成合议庭，公开开庭进行审理。深圳市罗湖区人民检察院指派检察员张月出庭支持公诉，被告人吕东明、陈小佳、胡永水、叶再坤、沈少君及各自辩护人到庭参加诉讼。现已审理终结。

深圳市罗湖区人民检察院指控：被告人吕东明长期非法买卖穿山甲。经查明，2019 年 6 月 26 日，被告人吕东明以人民币 7400 元的价格向深圳市龙华街道豪客源农家菜店主即被告人叶再坤非法出售活体穿山甲 1 只，被告人叶再坤于当日将该只穿山甲非法出售给证人马某(已作不起诉)，并在自己店内为其宰杀加工用于公司聚餐食用，后证人马某将该只穿山甲的鳞片带走。民警于 2019 年 8 月 12 日在深圳市龙华新区望成路 81 号豪客源农家菜抓获被告人叶再坤，证人马某于当日被电话传唤到深圳市龙华区华侨山庄 10 栋 6 层，民警在马某位于深圳市龙华区的住处冰箱中查获上述穿山甲鳞片 1 袋。

2019 年 7 月 8 日，被告人胡永水将从被告人陈小佳处购得的活体穿山甲 1 只以人民币 16250 元的价格非法出售给证人李某 1（已作不起诉），并送至其深圳深茂能工程建设有限公司五楼厨房，为其宰杀加工以供食用。2019 年 8 月 12 日，民警在上述厨房抓获证人李某 1，查获穿山甲血拌米 1 盆。

2019 年 8 月初，被告人吕东明向广西籍被告人(身份不明，在逃）购买穿山甲一批，并雇佣被告人陈小佳于 2019 年 8 月 7 日晚在深圳机荷高速平湖接货，后运到深圳市罗湖区仓库存放。2019 年 8 月 8 日，民警根据线索在该仓库蹲守时，发现被告人陈小佳从仓库取走穿山甲 1 只，遂追踪深圳市宝安区灶下村，将欲进行交易的被告人胡永水、陈小佳抓获，当场查获穿山甲 1 只。另查明，被告人陈小佳在给被告人胡永水送货途中与被告人沈少

君谈妥交易穿山甲 1 只，被告人沈少君于当日微信转账人民币 1000 元作为定金，民警于当日在深州市罗湖区档抓获被告人沈少君。同时，民警在深圳市罗湖区仓库抓获被告人吕东明，现场查获剩余的穿山甲 17 只(其中，活体 15 只、冻体 2 只），并在其位于深圳市罗湖区的家中查获穿山甲鳞片泡酒 1 瓶。

经广东国检司法鉴定所鉴定，涉案 16 只穿山甲活体、2 只穿山甲冻体、穿山甲泡酒、穿山甲血拌米、穿山甲鳞片均为哺乳纲（MAMMALIA）鳞甲目（PHOLIDOTA）穿山甲科（Manidae)穿山甲属（Manis)物种，属于《濒危野生动植物种国际贸易公约》（CITES)(2017 版）附录 I 和国家二级重点保护野生动物。

为证明上述事实，公诉机关在法庭上出示或宣读了下列证据：1.物证：缴获的穿山甲 18 只、穿山甲鳞片 1 袋、穿山甲泡酒 1 瓶、穿山甲血拌米 1 盆、涉案车辆；2.书证：受案登记表、立案决定书、深圳公安局森林分局龙岗派出所线索来源、被告人身份信息、指认现场照片、微信聊天记录及交易记录截图、手机通话记录、搜查证、搜查笔录、扣押决定书、扣押清单、调取证据通知书、鉴定聘请书、鉴定意见通知书、广东省野生动物及其产品接收专用收据、公安机关出具的情况说明、广东鹏海司法鉴定所出具的情况说明、深圳市司法局准予变更决定书等；3.证人证言：被不起诉人马某、李某 1 的供述、证人黄某、周某、李某 2、邓某等 10 名证人的证言、民警出具的抓获经过；4.被告人的供述与辩解：被告人吕东明、陈小佳、胡永水、叶再坤、沈少君

的供述与辩解。5.鉴定意见：广东国检司法鉴定所、广东安证计算机司法鉴定所出具的司法鉴定意见书；6.勘验、检查、辨认、侦查实验等笔录：现场勘验检查笔录、被告人、证人的辨认笔录；7.视听资料、电子数据：光盘3张。

公诉机关认为，被告人吕东明非法收购、运输、出售珍贵、濒危野生动物，情节特别严重，犯罪事实清楚，证据确实、充分，其行为触犯了《中华人民共和国刑法》第三百四十一条第一款，应当以非法收购、运输、出售珍贵、濒危野生动物罪追究其刑事责任。被告人陈小佳非法运输、出售珍贵、濒危野生动物，情节特别严重，犯罪事实清楚，证据确实、充分，其行为触犯了《中华人民共和国刑法》第三百四十一条第一款，应当以非法运输、出售珍贵、濒危野生动物罪追究其刑事责任。被告人胡永水、叶再坤非法收购、出售珍贵、濒危野生动物，犯罪事实清楚，证据确实、充分，其行为触犯了《中华人民共和国刑法》第三百四十一条第一款，应当以非法收购、出售珍贵、濒危野生动物罪追究其刑事责任。被告人沈少君非法收购珍贵、濒危野生动物，犯罪事实清楚，证据确实、充分，其行为触犯了《中华人民共和国刑法》第三百四十一条第一款，应当以非法收购珍贵、濒危野生动物罪追究其刑事责任。被告人沈少君非法收购珍贵、濒危野生动物，由于意志以外的原因而未得逞，是犯罪未遂，根据《中华人民共和国刑法》第二十三条之规定，可以比照既遂犯从轻或者减轻发罚。被告人陈小佳、胡永水、叶再坤、沈少君归案后，如实

供述自己的罪行，是坦白，根据《中华人民共和国刑法》第六十七条第三款之规定，可以从轻处罚；被告人陈小佳、胡永水、叶再坤、沈少君自愿如实供述自己的罪行，承认指控的犯罪事实，愿意接受处罚，根据《中华人民共和国刑事诉讼法》第十五条之规定，可以依法从宽处理。根据《中华人民共和国刑事诉讼法》第一百七十六条第一款之规定，提起公诉，请依法判处。

被告人吕东明在庭审中承认控罪，但对公诉机关指控其收购穿山甲有异议，辩称穿山甲是其亲戚的，其只是帮亲戚养穿山甲。

被告人吕东明的辩护人为其作罪轻辩护：1.被告人自愿认罪认罚；2.被告人系初犯、偶犯，没有前科；3.被告人具有坦白情节，依法可以从轻处罚；4.被告人获利金额小，对珍稀动物资源造成的损害后果较小；5.被告人经济压力大，有3个未成年子女，2名老人需要抚养，妻子没有工作，希望罚金方面能够减免。

被告人陈小佳对起诉书指控的犯罪事实无异议，认罪认罚，恳请法院从轻处理。

被告人陈小佳的辩护人为其作罪轻辩护：1.被告人陈小佳是在同案犯吕东明的安排下去接货，被告人陈小佳是接受同案犯吕东明的雇佣，属于从犯，对于从犯应当从轻或者减轻处罚；2.涉案的大部分穿山甲都是活体，并没有造成严重的后果；且被告人陈小佳的运输行为具有偶然性，主观恶意不大，可以从轻处罚；3.被告人陈小佳归案后如实供述自己的罪行并当庭认罪认罚；4.

被告人陈小佳未受过行政及刑事处罚，本次是初犯、偶犯，请求法院酌情从轻处罚，判处5年以下有期徒刑。

被告人胡永水对起诉书指控的犯罪事实无异议，认罪认罚，恳请法院从轻处理。

被告人胡永水的辩护人为其作罪轻辩护：1.被告人胡永水归案后如实供述所犯罪行，属于坦白，可以从轻处罚；2.被告人胡永水系初犯、偶犯，可酌情从轻处罚。被告人人胡永水无犯罪前科，且文化程度低，法律知识和法律意识欠缺；3.被告人的行为虽构成犯罪，但犯罪情节相对较轻。被告人非法收购、出售穿山甲两只，获利不多，且其中一只尚存活，未完成交易，属于未遂，可以从轻、减轻处罚。4.被告人胡永水认罪认罚、真诚悔罪，请求从轻、减轻处罚。综上建议判处被告人胡永水有期徒刑一年。

被告人叶再坤对起诉书指控的犯罪事实无异议，认罪认罚，恳请法院从轻处理。

被告人叶再坤的辩护人为其作罪轻辩护：被告人到案后如实供述犯罪事实，自愿认罪认罚，确有悔罪表现，依法可以从轻处罚；被告人犯罪情节轻微，主观恶性较轻，社会危害性不大，且自愿认罪积极悔改，同时也表示对公诉机关提起的公益诉讼积极予以赔偿。综上，请求对被告人叶再坤从轻处罚。

被告人沈少君对起诉书指控的犯罪事实无异议，认罪认罚，恳请法院从轻处理。

被告人沈少君的辩护人为其作罪轻辩护：1.被告人沈少君系初犯，归案后认罪态度极好，具有认罪、坦白情节，有悔罪表现，依法可以从轻处罚；2.被告人沈少君系替朋友购买穿山甲，法律意识淡薄，没有主观犯罪故意；3.本案属于犯罪未遂，可以比照既遂犯从轻或者减轻处罚。

经审理查明：公诉机关指控被告人吕东明犯非法收购、运输、出售珍贵、濒危野生动物罪，被告人陈小佳犯非法运输、出售珍贵、濒危野生动物罪，被告人胡永水、叶再坤犯非法收购、出售珍贵、濒危野生动物罪，被告人沈少君犯非法收购珍贵、濒危野生动物罪的事实客观、真实，证明上述事实的证据来源合法，且经当庭质证，本院予以采信。

另查明，深圳市罗湖区人民检察院作为公益诉讼人与被告人吕东明、胡永水、叶再坤、陈小佳以及证人马某、李某1就本案刑事附带公益诉讼达成调解。

本院认为，被告人吕东明无视国家法律，非法收购、运输、出售珍贵、濒危野生动物，情节特别严重，已构成非法收购、运输、出售珍贵、濒危野生动物罪。被告人陈小佳无视国家法律，非法运输、出售珍贵、濒危野生动物，已构成非法运输、出售珍贵、濒危野生动物罪。被告人胡永水、叶再坤无视国家法律，非法收购、出售珍贵、濒危野生动物，均已构成非法收购、出售珍贵、濒危野生动物罪。被告人沈少君无视国家法律，非法收购珍贵、濒危野生动物，其行为已构成非法收购珍贵、濒危野生动物

罪。被告人沈少君非法收购珍贵、濒危野生动物，由于意志以外的原因而未得逞，是犯罪未遂，可以比照既遂犯从轻或者减轻发罚。被告人吕东明在共同犯罪中起主要作用，是主犯。被告人陈小佳在共同犯罪中起次要作用，是从犯，应当减轻处罚。被告人陈小佳、胡永水、叶再坤、沈少君归案后，如实供述自己的罪行，是坦白，可以从轻处罚；被告人陈小佳、胡永水、叶再坤、沈少君自愿如实供述自己的罪行，承认指控的犯罪事实，愿意接受处罚，可以依法从宽处理。辩护人与事实相符的相关辩护意见，本院予以采纳。被告人吕东明辩称涉案 17 只穿山甲是帮亲戚代养，经查，涉案 17 只穿山甲是在被告人吕东明租赁的罗湖区翠荫路 30 号仓库查获，被告人吕东明在公安机关供认涉案 17 只穿山甲系由其非法收购，并安排被告人陈小佳接货运输，该供述与被告人陈小佳的供述相吻合，在案证据足以认定上述涉案 17 只穿山甲系由被告人吕东明非法收购。被告人吕东明当庭翻供称系帮亲戚代养与查明的事实不符，本院不予采信。被告人吕东明、陈小佳、胡永水、叶再坤与公益诉讼人达成了调解，可以酌情从轻处罚。根据《中华人民共和国刑法》第三百四十一条、第二十三条、第二十五条、第二十六条、第二十七条、第六十七条第三款、第七十二条、第七十三条、第六十四条之规定，判决如下：

一、被告人吕东明犯非法收购、运输、出售珍贵、濒危野生动物罪，判处有期徒刑十年，并处罚金人民币 1 万元（刑期自判决执行之日起计算。判决执行以前先行羁押的，羁押一日折抵刑

期一日。即自2019年8月8日起至2029年8月7日止；罚金自本判决生效之日起十日内向本院缴纳）；

二、被告人陈小佳犯非法运输、出售珍贵、濒危野生动物罪，判处有期徒刑五年，并处罚金人民币5000元（刑期自判决执行之日起计算。判决执行以前先行羁押的，羁押一日折抵刑期一日。即自2019年8月8日起至2024年8月7日止；罚金自本判决生效之日起十日内向本院缴纳）；

三、被告人胡永水犯非法收购、出售珍贵、濒危野生动物罪，判处有期徒刑一年一个月，并处罚金人民币5000元（刑期自判决执行之日起计算。判决执行以前先行羁押的，羁押一日折抵刑期一日。即自2019年8月8日起至2020年9月7日止；罚金自本判决生效之日起十日内向本院缴纳）；

四、被告人叶再坤犯非法收购、出售珍贵、濒危野生动物罪，判处有期徒刑一年一个月，并处罚金人民币2000元（刑期自判决执行之日起计算。判决执行以前先行羁押的，羁押一日折抵刑期一日。即自2019年8月12日起至2020年9月11日止；罚金自本判决生效之日起十日内向本院缴纳）；

五、被告人沈少君犯非法收购珍贵、濒危野生动物罪，判处有期徒刑一年，缓刑一年六个月，并处罚金人民币5000元（缓刑考验期自判决确定之日起计算；罚金自本判决生效之日起十日内向本院缴纳）；

六、扣押在案的作案工具粤B×××**小汽车一辆、黑色vivo手机1部、苹果手机1部、华为手机1部、红米7手机1部，上缴国库，予以没收；查获在案的穿山甲活体已移交深圳市野生动物救护中心接收；扣押在案的穿山甲鳞片、穿山甲血拌米、穿山甲鳞片泡酒，由公安机关予以销毁。

如不服本判决，可于接到判决书的第二日起十日内，通过本院或者直接向广东省深圳市中级人民法院提起上诉。书面上诉的，应当提交上诉状正本一份、副本二份。

审　判　长　　连　亮

人民陪审员　　李彦凭

人民陪审员　　张美美

二〇二〇年八月二十八日

书　记　员　　王昕怡

法官助理卢欣璇

附相关法条：

《中华人民共和国刑法》

第三百四十一条非法猎捕、杀害国家重点保护的珍贵、濒危野生动物的或者非法收购、运输、出售国家重点保护的珍贵、濒危野生动物及其制品的处五年以下有期徒刑或者拘役并处罚金；情节严重的处五年以上十年以下有期徒刑并处罚金；情节特别严重的处十年以上有期徒刑并处罚金或者没收财产。

违反狩猎法规在禁猎区、禁猎期或者使用禁用的工具、方法进行狩猎破坏野生动物资源情节严重的处三年以下有期徒刑、拘役、管制或者罚金。

第二十三条已经着手实行犯罪，由于犯罪分子意志以外的原因而未得逞的，是犯罪未遂。

对于未遂犯，可以比照既遂犯从轻或者减轻处罚。

第二十五条共同犯罪是指二人以上共同故意犯罪。

二人以上共同过失犯罪，不以共同犯罪论处；应当负刑事责任的，按照他们所犯的罪分别处罚。

第二十六条组织、领导犯罪集团进行犯罪活动的或者在共同犯罪中起主要作用的，是主犯。

三人以上为共同实施犯罪而组成的较为固定的犯罪组织，是犯罪集团。

对组织、领导犯罪集团的首要分子，按照集团所犯的全部罪行处罚。

对于第三款规定以外的主犯，应当按照其所参与的或者组织、指挥的全部犯罪处罚。

第二十七条在共同犯罪中起次要或者辅助作用的，是从犯。

对于从犯，应当从轻、减轻处罚或者免除处罚。

第六十七条犯罪以后自动投案，如实供述自己的罪行的，是自首。对于自首的犯罪分子，可以从轻或者减轻处罚。其中，犯罪较轻的，可以免除处罚。

被采取强制措施的犯罪嫌疑人、被告人和正在服刑的罪犯，如实供述司法机关还未掌握的本人其他罪行的，以自首论。

犯罪嫌疑人虽不具有前两款规定的自首情节，但是如实供述自己罪行的，可以从轻处罚；因其如实供述自己罪行，避免特别严重后果发生的，可以减轻处罚。

第七十二条对于被判处拘役、三年以下有期徒刑的犯罪分子，同时符合下列条件的，可以宣告缓刑，对其中不满十八周岁的人、怀孕的妇女和已满七十五周岁的人，应当宣告缓刑：

（一）犯罪情节较轻；

（二）有悔罪表现；

（三）没有再犯罪的危险；

（四）宣告缓刑对所居住社区没有重大不良影响。

宣告缓刑，可以根据犯罪情况，同时禁止犯罪分子在缓刑考验期限内从事特定活动，进入特定区域、场所，接触特定的人。

被宣告缓刑的犯罪分子，如果被判处附加刑，附加刑仍须执行。

第七十三条拘役的缓刑考验期限为原判刑期以上一年以下，但是不能少于二个月。

有期徒刑的缓刑考验期限为原判刑期以上五年以下，但是不能少于一年。

缓刑考验期限，从判决确定之日起计算。

第六十四条犯罪分子违法所得的一切财物，应当予以追缴或者责令退赔；对被害人的合法财产，应当及时返还；违禁品和供犯罪所用的本人财物，应当予以没收。没收的财物和罚金，一律上缴国库，不得挪用和自行处理。
