# Supplementary material for: China’s Legal Protection System for Pangolins: Past, Present, and Future
Source: Animals (Basel). 2025 Aug 18;15(16):2422. doi: 10.3390/ani15162422 (PMC12383201; doi:10.3390/ani15162422)
Supplement: Supplementary file 1 [file animals-15-02422-s001.zip › Supplementary Material S4-Full Text of Judgments in Pangolin-Related Public Interest Litigation Cases in China/【33】付某非法收购、运输、出售珍贵、濒危野生动物、珍贵、濒危野生动物制品一审刑事判决书.pdf]

付某非法收购、运输、出售珍贵、濒危野生动物、珍贵、濒危野生动物制品一审刑事判决书

江西省抚州市临川区人民法院

刑 事 附 带 民 事 判 决 书

（2020）赣1002刑初201号之二

公诉机关暨附带民事公益诉讼起诉人：抚州市临川区人民检察院。

被告人暨附带民事公益诉讼被告付某，女，1994年8月1日出生于云南省宣威市，居民身份证号码，汉族，大学本科，户籍所在地江西省抚州市临川区，住抚州市。因涉嫌非法收购珍贵野生动物罪，于2020年1月30日被抚州市临川区森林公安局刑事拘留，同年2月26日经抚州市临川区人民检察院批准逮捕，次日由抚州市临川区森林公安局依法执行逮捕。现羁押在抚州市看守所。

抚州市临川区人民检察院以临检公刑诉（2020）188号起诉书指控被告人付某犯非法收购珍贵野生动物罪，于2020年5月21日向本院提起公诉。公益诉讼起诉人抚州市临川区人民检察院于2020年5月21日向本院提起附带非法收购珍贵野生动物民事公益诉讼。经查，抚州市临川区人民检察院于2020年4月3日公告了案件相关情况，公告期内未有法律规定的机关和有关组织提起民事公益诉讼。2020年6月9日本院因疫情原因中止审理，2020年7月27日恢复审理。本院依法组成七人合议庭，于

2020 年 8 月 3 日公开开庭审理了本案。抚州市临川区人民检察院指派检察官邓丽玲、丁燕、检察官助理吴赟娜子、陈逸君出庭履行职务，被告人暨附带民事公益诉讼被告付某到庭参加诉讼，本案现已审理终结。

公诉机关抚州市临川区人民检察院指控，2017 年 11 月份以来被告人付某系抚州矮子佬鱼馆的经营者。2019 年 7 月被告人付某在抚州市同淑路农贸市场马路边购买了一只穿山甲（死体），冰冻在其经营的餐馆冰柜中，准备用于制作菜肴招待亲朋与顾客。2020 年 1 月 30 日公安民警在其店内冰柜中查获该只穿山甲死体。经江西野生动植物司法鉴定中心鉴定，查获的穿山甲死体系国家二级重点保护动物。同日公安民警在矮子佬鱼馆内查获活体王锦蛇 4 条、死体王锦蛇 1 条、死体竹鼠 25 只、死体华南兔 22 只、浸泡药酒（内有疑似眼镜蛇）3 罐等物。

认定上述事实的证据如下：1、书证；2、现场勘验笔录；3、鉴定意见；4、证人余某 1 等人的证言；5、辨认笔录；6、被告人付某的供述与辩解。上述证据收集程序合法，内容客观真实，足以认定指控事实。被告人付某对指控的犯罪事实和证据没有异议，并自愿认罪认罚。公诉机关认为，被告人付某非法收购国家重点保护的珍贵野生动物，其行为触犯《中华人民共和国刑法》第三百四十一条第一款之规定，犯罪事实清楚、证据确实充分，应当以非法收购珍贵野生动物罪追究其刑事责任。被告人付某能如实供述自己的罪行，根据《中华人民共和国刑事诉讼法》第十

五条的规定，可以对被告人付某从宽处理。根据《中华人民共和国刑事诉讼法》第一百七十六条的规定，建议适用普通程序审理，请依法判处。

附带民事公益诉讼起诉人抚州市临川区人民检察院向本院提出诉讼请求：判令附带民事公益诉讼被告付某赔偿生态资源损失费人民币 48260 元。事实和理由：附带民事公益诉讼被告付某系抚州矮子佬鱼馆的经营者。2019 年 7 月被告人付某在抚州市同淑路农贸市场马路边购买了一只穿山甲（死体），冰冻在其经营的餐馆冰柜中，准备用于制作菜肴招待亲朋与顾客。2020 年 1 月 30 日公安民警在其店内冰柜中查获该只穿山甲死体。经江西野生动植物司法鉴定中心鉴定，查获的穿山甲死体系国家二级重点保护动物。同日公安民警在矮子佬鱼馆内查获活体王锦蛇 4 条、死体王锦蛇 1 条、死体竹鼠 25 只、死体华南兔 22 只、浸泡药酒（内有疑似眼镜蛇）3 罐等物。经江西野生动植物司法鉴定中心鉴定，付某非法收购的野生动物总价值为人民币 48260 元。附带民事公益诉讼起诉人认为，被告付某违反国家规定，非法收购国家重点保护动物穿山甲及“三有保护动物”王锦蛇、华南兔、竹鼠等，用于制作菜肴出售。被告的行为破坏了生态环境，损害了社会公共利益，违反了《中华人民共和国野生动物保护法》第二十七条第一款的规定，根据《中华人民共和国侵权责任法》第六条、第十五条、第十九条的规定，被告付某应承担赔偿损失的民事侵权责任。

被告人暨附带民事公益诉讼被告付某自愿认罪认罚，对起诉书指控的事实和罪名没有异议。并愿意承担附带民事公益诉讼的赔偿责任。

经审理查明，2017年11月份，被告人暨附带民事公益诉讼被告付某经工商部门登记为“抚州临川区矮子佬鱼馆”餐馆的经营者。2020年1月30日，公安民警对“抚州临川区矮子佬鱼馆”进行检查，在其店内冰柜中查获穿山甲（死体）1只，同时查获活体王锦蛇4条、死体王锦蛇1条、死体竹鼠25只、死体华南兔22只、浸泡药酒（内有疑似眼镜蛇）3罐等物。

经江西野生动植物司法鉴定中心鉴定，查获的穿山甲列入《国家重点保护野生动物名录》，并认定为国家二级重点保护动物，王锦蛇、竹鼠、华南兔列入《国家保护的有益的或者有重要经济、科学研究价值的陆生野生动物名录》。其中：穿山甲价值40000元/只、王锦蛇价值300元/只、竹鼠200元/只、华南兔80元/只，被告人暨附带民事公益诉讼被告付某非法收购的野生动物总价值为人民币48260元。

认定上述事实，有经庭审质证、认证的下列证据证实，本院予以确认。

1、物证书证。

（1）受案登记表、立案决定书。证实：本案案发经过。

（2）常住人口信息。证实：证明被告人付某具有完全刑事责任能力。

（3）被告人付某归案情况的说明。证实：被告人付某系经被查获后当场传唤到案。

（4）对在付某经营的临川区矮子佬鱼馆内查获的野生动物进行扣押的扣押决定书、扣押清单、扣押笔录及扣押物品照片。

（5）对付某持有的蓝色 vivo 手机进行扣押的扣押决定书、扣押清单、扣押笔录及扣押物品照片。

（6）抚州市野生动植物保护管理局出具的情况说明。证实：扣押野生动物物品种类、数量。

（7）在工商部门调取证据材料。证明案发时临川区矮子佬鱼馆经营者为付某。

（8）发还清单。证实：扣押的手机返还给余某 1。

2、现场勘验笔录。

证实：2020 年 1 月 30 日临川区森林公安局依法对矮子佬鱼馆内进行了勘验检查。

3、证人证言。

（1）证人余某 1 的证言。

证实：矮子佬餐馆的经营者的是付某。

（2）证人余某 2 的证言。

证实：2019 年 7 月份至 2020 年 1 月 28 日，余某 2 应聘为矮子佬鱼馆的厨师。矮子佬鱼馆由付某一人管理,她是餐馆唯一的老板。平时，余某 2、付某、吴敏（又叫余某 1）和占某在矮

子佬鱼馆工作。一般早上付某会去买菜,买好都放在餐馆。工资按人民币 6000 元 / 月结算。

（3）证人占某的证言。

证实：2019 年 9 月到 2020 年 1 月 18 日，占某在矮子佬鱼馆做事。付某是矮子佬鱼馆的老板，所有事情都由付某管理。矮子佬鱼馆还有三名员工,分别是占某、余某 2、吴敏(又叫余某 1)，付某负责管理餐馆，写菜单、收银等;我负责打杂,洗碗、洗菜、端菜、拖地都是我做的;余某 2 是店里的厨师,饭店的菜都是他做;吴敏负责切菜打下手。

（4）证人余某 3 的证言。

证实：余某 1 是余某 3 的次子，付某是余某 3 的二儿媳。矮子佬鱼馆是付某独自经营。

4、辨认笔录（附现场辨认照片）。

证实:被告人付某于 2020 年 1 月 30 日对在其临川区矮子佬鱼馆查获的蛇、兔、竹鼠、穿山甲、浸泡药酒三罐（内有蛇等）等野生动物的数量和物种依法进行了现场辩认。

5、被告人付某的供述。

证实：付某于 2017 年 8 月嫁给余某 1,余某 1 是厨师,付某就和余某 1 商量,打算在临川开家餐饮店。2017 年 11 月付某从其余某 1 小舅妈吴利红处接下临川区矮子佬鱼馆，并在工商局重新注册成立临川区矮子佬鱼馆，经营者为付某。经营鱼馆期间，付某负责买菜、点单、收银，在鱼馆内查获的野生动物系其在同

淑路及剪子口农贸市场分批次购买，其中穿山甲死体从同淑路农贸市场马路边购买，所购买的野生动物均打算招待亲朋与客人时烹饪。2017 年 11 月至今鱼馆有以下人员:付某，平时会根据客流量去菜市场买蔬菜、鱼类、肉类等家常菜备用,接待来客,订餐,结账,开发票等都是我负责。余某 1,担任厨师负责炒菜、切菜、配菜,打下手。吴连生,2017 年 11 月至 2019 年 11 月在鱼馆担任厨师,负责炒菜和打杂。余某 3,鱼馆在没有请占某等人之前余某 3 会到鱼馆帮忙洗菜、打扫卫生等。余某 2（余某 1 二舅余平昌的儿子）,2019 年 7 月担任鱼馆厨师后，菜基本是他做的。占某,她和余某 2 是夫妻,2019 年 9 月至今在鱼馆做事，负责洗碗、上菜、打扫卫生等。2020 年 1 月底接到有关部门通知,因新型肺炎疫情爆发,不允许 10 人以上聚餐。付某就让余某 2、占某等人回家了。店里留下付某和余某 1。2020 年 1 月 30 日上午 10 点钟左右,公安和林业局的人因新型肺炎疫情到鱼馆例行检查，在一楼小楼梯左手边房间里发现了三罐蛇类浸泡的酒,三楼小楼梯旁的一房间里发现了活体蛇和动物死体。查获的野生动物是付某分批次从同淑路菜市场周边或洋洲立交桥路边购买的。鱼馆收购、经营动物没有在林业部门办理手续，目前鱼馆只办理营业执照,食品经营许可证等。

6、附带民事公益诉讼起诉人提供的江西野生动植物司法鉴定中心鉴定书，证实付某非法收购的野生动物总价值为人民币48260 元。

本院认为，被告人付某违反法律规定，以营利为目的，非法收购野生动物穿山甲 1 只，王锦蛇 5 条、竹鼠 25 只、华南兔 22 只，其中穿山甲为二级国家重点保护的珍贵野生动物,根据《最高人民法院关于审理破坏野生动物资源刑事案件具体应用法律若干问题的解释》相关规定，购买此类穿山甲八只以下，处五年以下有期徒刑或者拘役，并处罚金。抚州市临川区人民检察院指控被告人付某犯非法收购珍贵野生动物罪的事实清楚，定罪准确，应予以确认。被告人付某违反《中华人民共和国野生动物法》规定擅自收购国家重点保护野生动物王锦蛇 5 条、竹鼠 25 只、华南兔 22 只的行为应酌情从重处罚。被告人付某红当庭认罪悔罪，并在检察院审查起诉期间自愿签订认罪认罚具结书，可依法从宽处理。抚州市临川区人民检察院提出被告人付某犯非法收购珍贵野生动物罪，判处有期徒刑十个月，并处罚金的量刑建议，符合被告人的犯罪事实和情节，贯彻了宽严相济的政策，本院予以采纳。

野生动物资源属于国家所有，不容任何人侵害。附带民事公益被告付某违反《中华人民共和国野生动物法》规定，非法收购国家重点保护动物穿山甲及有益的、有重要经济的、有科学研究价值的野生动物王锦蛇、华南兔、竹鼠等，用于制作菜肴出售。附带民事公益诉讼被告付某的违法行为破坏了生态环境，侵害国家利益和社会公共利益，抚州市临川区人民检察院提起附带民事公益诉讼，要求附带民事公益诉讼被告付某承担侵权民事赔偿责

任，符合法律规定，对其诉讼请求，本院依法予以支持。经江西野生动植物司法鉴定中心鉴定，附带民事公益诉讼被告付某收购的野生动物价值总价值为人民币48260元，附带民事公益诉讼被告付某应予全额赔偿。

综上，依照《中华人民共和国刑法》第三百四十一条第一款、第五十二条、第五十三条，《中华人民共和国刑事诉讼法》第十五条、《最高人民法院关于审理破坏野生动物资源刑事案件具体应用法律若干问题的解释》第一条、第二条以及《中华人民共和国环境保护法》第六十四条、《中华人民共和国野生动物保护法》第二十七条第一款、《中华人民共和国侵权责任法》第六条、第十五条第一款第（六）项、第十九条之规定，判决如下：

一、被告人付某犯非法收购珍贵野生动物罪，判处有期徒刑十个月，并处罚金40000元（已缴纳）。

（刑期从判决执行之日起计算。判决执行以前先行羁押的，羁押一日折抵刑期一日，即自2020年1月30日起至2020年11月29日止。）

二、判处附带民事公益诉讼被告付某于本判决生效三日内支付赔偿人民币48260元（已缴纳）。

如不服本判决，可在接到判决书的第二日起十日内，通过本院或者直接向抚州市中级人民法院提出上诉。书面上诉的，应当提交上诉状正本一份，副本二份。

审　判　长　　罗　晔

审　判　员　　黄国平

审　判　员　　李丽雯

人民陪审员　　万园秀

人民陪审员　　丁筱玲

人民陪审员　　张水兰

人民陪审员　　武贞娥

二〇二〇年八月四日

书　记　员　　黎蕾菲
