# Supplementary material for: China’s Legal Protection System for Pangolins: Past, Present, and Future
Source: Animals (Basel). 2025 Aug 18;15(16):2422. doi: 10.3390/ani15162422 (PMC12383201; doi:10.3390/ani15162422)
Supplement: Supplementary file 1 [file animals-15-02422-s001.zip › Supplementary Material S4-Full Text of Judgments in Pangolin-Related Public Interest Litigation Cases in China/【34】邱家平非法收购、运输、出售珍贵、濒危野生动物、珍贵、濒危野生动物制品一审刑事判决书.pdf]

邱家平非法收购、运输、出售珍贵、濒危野生动物、珍贵、濒危野生动物制品一审刑事判决书

江西省兴国县人民法院

刑 事 附 带 民 事 判 决 书

（2019）赣 0732 刑初 265 号

公诉机关暨附带民事公益诉讼起诉人江西省兴国县人民检察院。

被告人暨附带民事公益诉讼被告邱家平，男，1980 年 3 月 11 日出生于江西省万安县，汉族，初中文化，经商，户籍地址江西省万安县，案发前住广东省佛山市南海区。因涉嫌犯非法收购、出售珍贵、濒危野生动物制品罪，于 2018 年 12 月 31 日被兴国县森林公安局刑事拘留，2019 年 1 月 28 日被逮捕。现羁押于兴国县看守所。

辩护人暨委托诉讼代理人李华斌、蔡金英，江西宋城律师事务所律师。

江西省兴国县人民检察院以兴检刑检部刑诉〔2019〕209 号起诉书指控被告人邱家平犯非法收购、出售珍贵、濒危野生动物制品罪，于 2019 年 8 月 19 日向本院提起公诉，同时向本院提起附带环境民事公益诉讼。经查，兴国县人民检察院公告了案件相关情况，公告期内未有法律规定的机关和有关组织提起民事公益诉讼。本院于 2019 年 8 月 19 日立案受理，并依法组成合议庭，分别于 2019 年 10 月 15 日、2020 年 5 月 21 日公开开庭审理了

本案。兴国县人民检察院指派检察员李玉青、杨彩茂出庭履行职务，被告人暨附带民事公益诉讼被告邱家平及其辩护人暨委托诉讼代理人李华斌、蔡金英到庭参加诉讼。期间，经兴国县人民检察院建议，本院决定延期审理两次。现已审理终结。

公诉机关兴国县人民检察院指控：2017年11月至2018年1月，被告人邱家平先后三次出售了40只去鳞片冻体穿山甲给肖某（另案处理）。2018年1月20日，湖南省新邵县森林公安局民警在肖某租住地查获27只冻体穿山甲。经鉴定，查获的27只冻体穿山甲均为国家二级重点保护野生动物——马来穿山甲。

2018年4月，邱家平应刘某（已判刑）的要求出售了3只去鳞片冻体穿山甲给刘某。同年9月17日，兴国县森林公安局民警在刘某租用车库内查获1只冻体穿山甲。经鉴定，查获的该只冻体穿山甲为国家二级重点保护野生动物——中华穿山甲。

兴国县林业局根据国家林业局《野生动物及其制品价值评估办法》的规定，核定43只冻体穿山甲价值人民币156.52万元。

2018年12月31日，邱家平被抓获归案。2019年1月18日，邱家平的亲属代缴非法所得6300元。

公诉机关认为，被告人邱家平非法收购、出售国家二级保护动物去鳞死体穿山甲，情节特别严重，其行为触犯了《中华人民共和国刑法》第三百四十一条的规定，应当以非法收购、出售珍贵、濒危野生动物制品罪追究其刑事责任，同时建议判处其十四

年至十五年有期徒刑，并处罚金。公诉机关为支持指控向法庭提供了相关证据。

附带民事公益诉讼起诉人兴国县人民检察院向本院提出诉讼请求：判令邱家平赔偿非法收购、出售43只穿山甲制品造成生态资源受损的资源补偿费用156.52万元。事实和理由：邱家平非法收购、出售珍贵、濒危野生动物制品的行为侵犯了野生动物资源，破坏了生物多样性和生态平衡，损害了社会公共利益，承担刑事责任的同时还应当承担相应的民事责任。根据《野生动物及其制品价值评估办法》及其附件《陆生野生动物基准价值标准目录》的规定，每只穿山甲整体的价值按照基准价值的五倍核算，而每只穿山甲的基准价值为8000元，故每只穿山甲整体的价值为4万元。由于涉案的穿山甲为冻体且已去除鳞片，兴国县林业局野生动植物保护管理站核算每只去鳞片冻体穿山甲的价值为3.64万元。因此，邱家平应赔偿非法收购、出售43只去鳞冻体穿山甲造成生态资源受损的资源补偿费用156.52万元。

被告人暨附带民事公益诉讼被告邱家平辩称，我是卖了3只冻体穿山甲给刘某，但只卖了1只冻体穿山甲给肖某，冻体穿山甲的价值评估过高。

辩护人暨委托诉讼代理人提出的意见是：一、对公诉机关指控邱家平犯非法收购、出售珍贵、濒危野生动物制品罪无异议；二、公诉机关指控邱家平先后三次出售40只冻体穿山甲给肖某的事实不清，证据不足，无法认定。1、因肖某的供述在交易时

间、付款金额、付款方式上存在严重的出入与矛盾，极不稳定且反复多变，故不应采信，不能作为定案根据。2、公诉机关指控邱家平出售40只冻体穿山甲给肖某，没有形成完整的证据链。该部分指控只有肖某一人的供述，而邱家平对此予以否认。在邱家平否认指控的情况下，公诉机关不能进一步举证证明，以致事实不清。此外，肖某案的生效判决查明肖某从2016年10月就已开始穿山甲交易，其涉案的冻体穿山甲已远远超过了其供述的40只，而肖某与邱家平是从2017年下半年才开始交易穿山甲，故无法排除肖某的冻体穿山甲还来源于他人的可能性；三、在无法形成证据链，事实不清、真相存疑的情况下，应根据疑罪从无的原则剔除40只冻体穿山甲的认定。鉴于邱家平自认出售了1只冻体穿山甲给肖某，从有利于被告人的原则，本案邱家平收购、出售冻体穿山甲的数量应认定为4只；四、在刑事诉讼中，对被告人处罚的诉讼程序不同于行政处罚，关于涉案金额的认定依据必须以全国人大及其常委会的立法或最高检、最高院的司法解释来认定，而前述法律渊源并无按基准价核算的规定。《野生动物及其制品价值评估办法》来源于《野生动物保护法》的授权，而《野生动物保护法》为行政法，仅适用于行政处罚，属于行政规章，不能作为刑事处罚的依据用于刑事诉讼中。即使在《野生动物及其制品价值评估办法》中，对野生动物制品的估价也未明确规定必须按基准价五倍核算。实际上，作为冻体其价值远远低于活体，更不值五倍。按照活体价格减去鳞片价格的计算方法，既

不科学，也不符合常识，出具评估意见的机构也不具备法定资质。为体现法律的公平公正，邱家平的涉案金额应按照实际交易价格来认定。

综上所述，邱家平出售穿山甲的数量宜认定为4只。根据实际交易的价格核算，其涉案价值远未达到情节严重的标准。参照肖某案、刘某案及其他穿山甲公益诉讼案的判决结果，建议判处邱家平三年以下有期徒刑，并按照交易4只穿山甲的事实来确定邱家平的赔偿金额。

辩护人暨委托诉讼代理人提供的证据有：1、湖南省新邵县人民法院（2019）湘0522刑初39号刑事判决书，用于证明肖某案认定的事实及处理结果；2、病案资料，用于证明邱家平的女儿患有疾病。

经审理查明，被告人邱家平从他人处购买了10只去除鳞片的冻体穿山甲，而后加价出售给肖某（已另案处理）7只、刘某（已另案处理）3只。具体事实如下：

（一）2018年1月5日下午，经事先电话联系，邱家平将其从他人处购买的7只去鳞冻体穿山甲加价以23760元的价格出售给肖某。邱家平驾车将7只冻体穿山甲送至肖某在广州市白云区的住处附近交给肖某。肖某当天将货款给付邱家平，其中微信付款6255元，余款现金支付。

认定上述事实的证据有：

1、证人肖某的证言，证明2018年1月5日下午，他打电话叫邱家平送货（指去鳞冻体穿山甲）给他。邱家平通过微信发了条信息“88＊270，30.6＊245”给他，意思是“小的”88斤，每斤270元，“大的”30.6斤，每斤245元。邱家平开车来到他在广州市白云区的住处附近后叫他去拿货。他只要了“小的”88斤，一共7只。他当天把货款付给了邱家平，其中微信支付6255元，现金支付17505元。这7只冻体穿山甲被他卖掉了。

2、微信聊天、交易记录，证明2018年1月5日下午，邱家平通过微信发了条内容为“88＊270，30.6＊245”的信息给肖某，肖某当天下午微信支付6255元给邱家平。

3、辨认笔录，证明肖某、邱家平分别对对方进行了指认。

4、新邵县人民法院（2019）湘0522刑初39号刑事判决书、法律文书生效证明书，证明新邵法院认定肖某的涉案冻体穿山甲均来源于邱家平，该判决已发生法律效力。

5、被告人邱家平在侦查阶段的供述与辩解。邱家平归案后供述他和肖某之间存在冻体穿山甲交易，但辩解他只出售了1只给肖某。

关于邱家平出售肖某冻体穿山甲数量的认定问题。经查，2018年1月20日，湖南省新邵县森林公安局民警抓获肖某，并在其租住地（广东省广州市白云区同德围德康路180号紫薇苑501室）查获27只冻体穿山甲。经鉴定，该27只冻体穿山甲均为马来穿山甲，属国家二级重点保护野生动物。肖某归案后交代

其冻体穿山甲均来源于邱家平。2019 年 3 月 6 日，新邵县人民检察院指控肖某犯非法收购、出售珍贵、濒危野生动物、珍贵、濒危野生动物制品罪，向新邵县人民法院提起公诉。同年 9 月 9 日，新邵县人民法院作出（2019）湘 0522 刑初 39 号刑事判决。该判决认定：1、2017 年 6 月至 12 月，肖某分多次向王成增、全某、尹某收购穿山甲 14 只，然后加价出售给徐卫东、慈卫华、范长春、陈柏炳、阮洪洲、戴超、李成文、倪某和王某；2、2016 年 10 月至 2018 年 1 月，肖某分批从邱家平处收购冻体穿山甲，然后加价出售给他人。肖某已出售给徐卫东 4 只、刘富平 2 只、黄长江 2 只、周先林 2 只、李成文 1 只、戴超 1 只、王某 2 只、廖某 121.1 斤、陈某 10 只＋60.8 斤、饶某 49 斤，以及卢某 253.4 斤（冻体穿山甲和野生甲鱼混合重量）。至案发时止，尚有 27 只冻体穿山甲未出售。新邵法院认定前述事实的证据有：扣押清单，鉴定意见，证人陆某、程某 1、何某、梁某、叶某、黄某、倪某、王某、卢某、程某 2、陈某、饶某、尹某、全某、廖某 1 的证言，同案人徐卫东、慈卫华、王成增、杨春河、汤建军、刘富平、范长春、黄长江、阮宏洲、戴超、陈柏炳、李成文、周先林的供述和辩解，以及肖某的供述和辩解等证据。

公诉机关指控邱家平出售了 40 只冻体穿山甲给肖某，其依据是证人肖某的证言，微信聊天、交易记录和新邵法院（2019）湘 0522 刑初 39 号刑事判决书，以及新邵县森林公安局的搜查笔录、扣押清单。综合分析公诉机关据以指控的证据。本院审查认

为，肖某归案后虽供述其冻体穿山甲均来源于邱家平，但对交易时间、交易次数、交易数量（肖某先后供述的数量分别为30多只、30只、50只左右、40只）、付款金额、付款方式等细节问题的供述存在出入，反复多变，前后矛盾，故不能根据其供述来确定其与邱家平之间的交易数量；新邵法院认定肖某2016年10月至2018年1月分批从邱家平处收购冻体穿山甲，然后加价出售给他人，其中已出售的冻体穿山甲为24只＋130.9斤，尚未出售的冻体穿山甲为27只，此外还出售了253.4斤冻体穿山甲和野生甲鱼。但该院认定肖某涉案的冻体穿山甲来源于邱家平的依据仅仅只有肖某的供述，没有邱家平的供述及其他相关证据予以佐证，属孤证，且认定的交易时间（2016年10月至2018年1月）、交易数量（肖某涉案的冻体穿山甲为51只＋130.9斤，以及253.4斤冻体穿山甲和野生甲鱼），又与本案公诉机关的指控“2017年11月至2018年1月，邱家平先后三次出售了40只去鳞片冻体穿山甲给肖某”存在矛盾，亦不能根据新邵法院对肖某案作出的刑事判决来确定其与邱家平之间的交易数量；微信聊天、交易记录，只能证实肖某和邱家平之间有联系，以及肖某通过微信付款给邱家平的事实，且肖某同时也证实他和邱家平之间也存在甲鱼交易，微信支付给邱家平的钱，既有穿山甲交易的货款，也有甲鱼交易的货款，故不能据此确定肖某通过微信付给邱家平的钱全部是穿山甲的交易货款；新邵县森林公安局的搜查笔录、扣押清单，只能证实公安机关从肖某的租房内查获27只冻

体穿山甲这一客观事实，不能就此确定被查获的冻体穿山甲来源于邱家平。因此，公诉机关指控邱家平先后三次出售了40只冻体穿山甲给肖某，虽已体现了有利于被告人的原则，但由于公诉机关据以指控的证据不能形成完整的证据链，故本院对公诉机关的这一指控不予采纳。本院根据现有证据，并遵循有利于被告人原则，认定邱家平出售了7只冻体穿山甲给肖某。邱家平及其辩护人所提其只卖了1只冻体穿山甲给肖某的辩解、辩护意见，不能成立。

（二）2018年4月，邱家平从广州市从化区太平镇“兴富”山鸟集贸市场“老杨”处购买了3只去鳞冻体穿山甲，而后加价出售给刘某。邱家平通过物流将3只冻体穿山甲寄给刘某。同年9月17日，兴国县森林公安局民警在刘某租用的兴国县潋江镇“太鑫花园”小区车库内查获1只冻体穿山甲。经鉴定，该只穿山甲为中华穿山甲，属国家二级重点保护野生动物。

2018年12月31日，邱家平被佛山市公安局南海分局里水派出所民警抓获归案。2019年1月18日，邱家平的亲属代其将违法所得6300元退至兴国县森林公安局。

上述事实，有公诉机关提供的证人刘某、范某、徐某、廖某2的证言，辨认笔录，搜查笔录，现场勘验笔录，刑事摄影照片，提取笔录，扣押清单，兴广物流货物托运单，天网监控视频截图照片，车辆信息，住宿登记表，司法鉴定意见书，销毁笔录，办

案情况说明、现金缴款单、非税收入票据，本院刑事判决书，以及被告人邱家平在侦查阶段的供述等证据所证实。

另查明：

1、根据《野生动物及其制品价值评估办法》（国家林业局令第46号）和《陆生野生动物基准价值标准目录》的规定，野生动物整体的价值，按照该种野生动物的基准价值乘以相应的倍数核算，穿山甲科所有种的基准价值为每只8000元，作为国家二级保护野生动物，每只整体的价值应按照基准价值的五倍核算。兴国县林业局野生动植物保护管理站核算每只穿山甲的鳞片价值为3600元。因此，本案中10只去鳞片冻体穿山甲价值损失共计人民币36.4万元。前述事实，有公诉机关提供的兴国县林业局野生动植物保护管理站出具的《情况说明》予以证实。

2、被告人邱家平因于2012年11月中旬的一天和2013年2月6日分别收购、出售1只穿山甲给他人，茶陵县林业局于2014年4月17日决定对其处以行政处罚：没收违法所得12050元，并处罚款27950元。前述事实，有公诉机关提供的茶陵县林业局《林业行政处罚决定书》等书证所证实。

此外，公诉机关提供的以下综合证据。本院予以确认。

1、常住人口信息，证明被告人邱家平在作案时已达到刑事责任年龄。

2、抓获经过、归案情况说明，证明被告人邱家平系被公安机关抓获归案。

3、扣押清单，证明公安机关依法扣押了被告人邱家平的Sancup手机1部、华为荣耀手机1部。

本院认为，被告人邱家平违反国家有关野生动物保护法规，非法收购、出售国家二级保护野生动物穿山甲冻体，其行为构成非法收购、出售珍贵、濒危野生动物制品罪。公诉机关指控邱家平的罪名成立，本院予以确认。邱家平非法收购、出售国家二级保护野生动物穿山甲冻体，依法应处五年以下有期徒刑或者拘役，并处罚金。邱家平曾因贩卖穿山甲受到行政处罚，却继续贩卖穿山甲冻体，应对其酌情从重处罚。邱家平已退缴部分违法所得，可以对其酌情从轻处罚。

邱家平收购、出售国家二级保护野生动物穿山甲冻体的行为，为猎杀穿山甲提供了动机和市场，其行为既违反了国家有关野生动物保护法规，又对生态环境损害具有直接的因果关系，导致野生动物穿山甲数量减少，加深穿山甲濒危程度，破坏了生态资源和环境平衡，损害了社会公共利益，除应受到刑事处罚外，还应当承担相应的民事责任。附带民事公益诉讼起诉人要求邱家平赔偿其造成生态资源受损的资源补偿费用，符合相关法律规定，本院予以支持。

综上所述，依照《中华人民共和国刑法》第三百四十一条第一款、第六十四条、第三十六条第一款，《最高人民法院关于审理破坏野生动物资源刑事案件具体应用法律若干问题的解释》第一条、第二条，《最高人民法院、最高人民检察院关于检察公益

诉讼案件适用法律若干问题的解释》第二十条，《中华人民共和国民法总则》第一百八十七条、第一百七十九条，《中华人民共和国侵权责任法》第四条第一款、第十五条，《中华人民共和国野生动物保护法》第三条、第二十七条，《最高人民法院关于审理环境民事公益诉讼案件适用法律若干问题的解释》第十八条之规定，判决如下：

一、被告人邱家平犯非法收购、出售珍贵、濒危野生动物制品罪，判处有期徒刑三年，并处罚金人民币七万元。

（刑期从判决执行之日起计算。判决执行以前先行羁押的，羁押一日折抵刑期一日，即自2018年12月31日起至2021年12月30日止。）

（罚金限被告人邱家平在判决发生法律效力第二日起一个月内缴纳）。

二、被告人邱家平退缴的违法所得6300元，予以没收，上缴国库（由兴国县森林公安局负责上缴）。

三、继续追缴被告人邱家平的违法所得23760元，上缴国库。

四、随案移送的作案工具Sancup手机1部、华为荣耀手机1部，予以没收，上缴国库。

五、被告人邱家平于判决生效后三十日内向附带民事公益诉讼起诉人江西省兴国县人民检察院支付资源补偿费用364000元。

如不服本判决，可在接到判决书的第二日起十日内，通过本院或者直接向江西省赣州市中级人民法院提出上诉。书面上诉的，应当提交上诉状正本一份，副本二份。

审　判　长　　陈胜海

审　判　员　　李　斌

审　判　员　　肖　鹏

人民陪审员　　张祖平

人民陪审员　　温邦通

人民陪审员　　毛苏华

人民陪审员　　钟　焱

二〇二〇年六月二十二日

书　记　员　　刘　璐
