# Supplementary material for: China’s Legal Protection System for Pangolins: Past, Present, and Future
Source: Animals (Basel). 2025 Aug 18;15(16):2422. doi: 10.3390/ani15162422 (PMC12383201; doi:10.3390/ani15162422)
Supplement: Supplementary file 1 [file animals-15-02422-s001.zip › Supplementary Material S4-Full Text of Judgments in Pangolin-Related Public Interest Litigation Cases in China/【35】杨桥、张敏非法收购、运输、出售珍贵、濒危野生动物、珍贵、濒危野生动物制品一审刑事判决书.pdf]

杨桥、张敏非法收购、运输、出售珍贵、濒危野生动物、珍贵、濒危野生动物制品一审刑事判决书

成都市金牛区人民法院

刑 事 附 带 民 事 判 决 书

（2020）川0106刑初335号

公诉机关暨附带民事公益诉讼起诉人成都市金牛区人民检察院。

被告人杨桥，男，1971年1月5日出生，汉族，初中文化，经商，户籍地：四川省遂宁市安居区，住址：成都市新都区。2018年12月25日因涉嫌非法收购、出售珍贵、濒危野生动物制品罪被成都市公安局决定取保候审。2020年1月6日因涉嫌非法收购、运输、出售珍贵、濒危野生动物、珍贵、濒危野生动物制品罪被成都市金牛区人民检察院决定取保候审。

指定辩护人程渝，四川及第律师事务所律师。

被告人张敏，男，1982年12月24日出生，汉族，小学文化，户籍地：四川省成都市成华区，住址：同上。2019年8月28日因因涉嫌非法收购、出售珍贵、濒危野生动物制品罪被成都市公安局决定取保候审。

指定辩护人陈玲，四川文典律师事务所律师。

被告人苏兵，男，1984年10月8日出生，汉族，大学本科文化，经商，户籍地：四川省成都市金牛区，住址：四川省成都

市。2019 年 11 月 25 日因涉嫌非法收购、出售珍贵、濒危野生动物制品罪被成都市公安局决定取保候审。

指定辩护人陶沁，四川新开元律师事务所律师。

被告人叶明德，男，1957 年 6 月 16 日出生，汉族，高中文化，个体工商户，户籍地：四川省高县，住址：四川省成都市新都区。2019 年 8 月 28 日因涉嫌非法收购、出售珍贵、濒危野生动物制品罪被成都市公安局决定取保候审。

指定辩护人车忠河，四川文典律师事务所律师。

成都市金牛区人民检察院以成金检一部刑诉[2020]221 号起诉书指控被告人杨桥、张敏、苏兵、叶明德犯非法收购、出售珍贵、濒危野生动物制品罪，于 2020 年 5 月 22 日向本院提起公诉，在诉讼过程中，附带民事公益诉讼起诉人成都市金牛区人民检察院以成金检四部刑附民公诉[2020]1 号刑事附带民事公益诉讼起诉书向本院提起附带民事公益诉讼。本院依法组成合议庭，公开开庭进行了合并审理。成都市金牛区人民检察院指派检察员任立君、王军、史丽花，书记员欧静文出庭支持公诉及公益诉讼，被告人杨桥及其指定辩护人程渝，被告人张敏及其指定辩护人陈玲，被告人苏兵及其指定辩护人陶沁，被告人叶明德及其指定辩护人车忠河到庭参加诉讼。现已审理终结。

成都市金牛区人民检察院指控，2018 年 9 月 17 日，被告人叶明德将从他人处收购的 40 克猴骨以人民币 17.5 元价格向被告人杨桥出售。同日，被告人苏兵将从他人处收购的穿山甲片 40

克以人民币90元的价格向被告人杨桥出售。后被告人杨桥将上述猴骨、穿山甲片以人民币225元的价格出售给汪某海。经鉴定：上述疑似猴骨来源于灵长目猴科猕猴属藏酋猴Macacathibetana，为国家二级保护野生动物，其经济价值为人民币2，000元；上述疑似穿山甲片来源于鳞甲目穿山甲科穿山甲属穿山甲Manissp，为国家二级保护野生动物，其经济价值为人民币2,720元。

2018年10月9日，被告人苏兵将从他人处收购而来的穿山甲片40克以人民币90元的价格向被告人杨桥出售。同日，被告人张敏将从他人处收购的疑似虎骨20克以人民币280元的价格向被告人杨桥出售。后被告人杨桥将上述疑似虎骨、穿山甲片以人民币860元的价格出售给汪某海。经鉴定：上述疑似虎骨来源于食肉目猫科豹属狮Pantheraleo，狮列入CITES附录II（等同国家二级保护野生动物），其经济价值为人民币15，000元；上述疑似穿山甲片来源于鳞甲目穿山甲科穿山甲属穿山甲Manissp，为国家二级保护野生动物，其经济价值为人民币2，720元。

被告人杨桥、张敏分别于2018年10月22日、2019年8月27日被公安机关挡获，被告人叶明德、苏兵分别于2019年8月27日、2019年11月22日到公安机关投案，并如实供述了罪行。

公诉机关就上述指控事实向法庭出示的证据有：书证、物证、证人证言、被告人供述和辩解、鉴定意见、辨认笔录等，公诉机

关认为，被告人杨桥、张敏、苏兵、叶明德的行为均已构成非法收购、出售珍贵、濒危野生动物制品罪。被告人苏兵、叶明德犯罪后自动投案，如实供述自己的罪行，是自首，可以从轻处罚。被告人杨桥、张敏如实供述其罪行，系坦白，可以从轻处罚。被告人杨桥、张敏、苏兵、叶明德自愿认罪认罚，可以从宽处罚。公诉机关依照《中华人民共和国刑法》第三百四十一条第一款、第六十七条第一款、第三款，《中华人民共和国刑事诉讼法》第十五条，第一百七十六条第一款、第二款之规定提起公诉，建议判处被告人杨桥有期徒刑一年六个月，缓刑二年，并处罚金八千元；判处被告人张敏有期徒刑一年，缓刑二年，并处罚金五千元；判处被告人苏兵有期徒刑十个月，缓刑一年六个月，并处罚金四千元；判处被告人叶明德有期徒刑十个月，缓刑一年六个月，并处罚金三千元。

附带民事公益诉讼起诉人诉称，被告人杨桥、叶明德、张敏、苏兵以非法收购、出售国家二级重点保护野生动物制品，其行为违反了《中华人民共和国野生动物保护法》第三条“野生动物资源属于国家所有”、第二十七条“禁止出售、购买国家重点保护野生动物及其制品”之规定，破坏生物多样性、危害生态系统平衡，造成国家资源损失，损害了社会公共利益，根据《中华人民共和国民法总则》第一百七十九条、《中华人民共和国侵权责任法》第八条、第十五条之规定，请求判令：1、杨桥、叶明德、张敏、苏兵在成都市级以上媒体公开赔礼道歉；2、杨桥、叶明

德共同赔偿 2,000 元；3、杨桥、张敏共同赔偿 15,000 元；4、杨桥、苏兵共同赔偿 5,440 元。

被告人杨桥对公诉机关指控的罪名、犯罪事实无异议，自愿认罪认罚。对附民公益诉讼请求表示愿意赔偿。

被告人杨桥的指定辩护人对公诉机关指控的犯罪事实、罪名无异议，并提出：1、案涉的野生动物制品来源不合法；2、野生动物制品不易分辨；3、对部分证据的真实性存疑；4、被告人杨桥主观上具有买卖故意，但客观上存在疑问；5、被告人杨桥系坦白，认罪态度好；6、被告人系初犯、偶犯。综上，请求法庭从轻处罚。

被告人张敏对公诉机关指控的罪名、犯罪事实无异议，自愿认罪认罚。对附民公益诉讼请求表示愿意赔偿。

被告人张敏的指定辩护人对公诉机关指控的犯罪事实、罪名无异议，并提出：1、被告人张敏到案后如实供述自己的罪行，系坦白，且自愿认罪认罚，可以从轻处罚；2、被告人张敏主观恶性小，涉案金额小，社会影响有限，加之被告人系初犯、偶犯，请求从轻处罚。综上，请求得到从轻处罚。

被告人苏兵对公诉机关指控的罪名、犯罪事实无异议，自愿认罪认罚。对附民公益诉讼请求表示愿意赔偿。

被告人苏兵的指定辩护人对公诉机关指控的犯罪事实、罪名无异议，并提出：1、被告人苏兵的情节轻微，涉案价值较小；2、被告人苏兵犯罪后自动投案，系自首，且自愿认罪认罚，可以从

轻处罚；3、被告人苏兵积极进行了公益诉讼赔偿；4、建议罚金从轻处罚。综上，请求得到从轻处罚。

被告人叶明德对公诉机关指控的罪名、犯罪事实无异议，自愿认罪认罚。对附民公益诉讼请求表示愿意赔偿。

被告人叶明德的指定辩护人对公诉机关指控的犯罪事实、罪名无异议，并提出：1、被告人叶明德系初犯、偶犯，犯罪情节轻微，涉案价值较小；2、被告人叶明德犯罪后自动投案，系自首，且自愿认罪认罚，可以从轻处罚；综上，请求法庭从轻处罚。

经审理查明的事实与公诉机关指控的犯罪事实一致。另查明，涉案猴骨、狮骨、穿山甲片扣押在成都市。

在案件审理过程中，被告人杨桥、张敏、苏兵、叶明德将公益诉讼赔偿款 22,440 元已缴纳。

上述事实经庭审核实的证据有：受案登记表，立案决定书，到案经过，指认照片，称重照片，情况说明，营业执照，信誉卡，司法鉴定书，证人汪某某、邹某某、叶某的证言，被告人杨桥、张敏、苏兵、叶明德的户籍信息，被告人杨桥、张敏、苏兵、叶明德的供述和辩解，认罪认罚具结书等证据。

本院认为，被告人杨桥、张敏、苏兵、叶明德违反野生动物保护管理法律法规，未经许可非法收购、出售国家重点保护的珍贵、濒危野生动物制品，其行为均已构成非法收购、出售珍贵、濒危野生动物制品罪，公诉机关指控成立，量刑建议适当，本院予以支持。

对被告人的指定杨桥辩护人提出的辩护意见，本院综合评析如下：

1、案涉野生动物制品取证合法，其理由：

（1）根据我国法律规定，任何单位和个人发现违法犯罪线索时有权举报。案涉的汪某某发现其购买的野生动物制品可能涉嫌犯罪，立即向公安机关进行举报并提交相关证据，符合法律规定。

（2）在案件的侦查过程中，四名被告人对案涉野生动物制品均进行了辨认，并签字确认。

（3）被告人杨桥、张敏、苏兵、叶明德明知收购、出售案涉野生动物制品是违法行为。

（4）从“信誉卡”载明的物品及数量，汪某某提供的物品及证言，被告人杨桥、张敏、苏兵、叶明德的供述和辩解等证据，可以形成完整的证据锁链证明杨桥从张敏、苏兵、叶明德处购买的是野生动物制品。对辩护人提出的上述辩护意见，本院不予采纳。

2、涉案出售的野生动物制品不易分辨的问题。穿山甲制品具有较高的辨识度，且被告人杨桥在指认过程中准确的指认处案涉的野生动物制品，并签字确认。因此，被告人杨桥的辨认是客观真实的。对辩护人提出的上述辩护意见，本院不予采纳。

3、公诉机关在举证阶段并未出示“药方”，故对辩护人对“药方”系传来证据，其真实性存疑的辩护意见不予采纳；

4、被告人杨桥明知收购、出售珍贵、濒危野生动物制品系违法犯罪行为，与汪某某达成购买的合意、且多次向他人收购珍贵濒危野生动物制品的行为，被告人杨桥收购、出售珍贵濒危野生动物制品犯罪行为已经完成，系犯罪既遂。对辩护人提出的上述辩护意见，本院不予采纳。

5、辩护人怀疑汪某某调换物证的辩护意见，无事实依据和法律依据，本院不予采纳。

6、辩护人认为张敏、叶明德所出售的是牦牛骨、藏獒骨的辩护意见，与被告人张敏、叶明德的当庭供述明显不符，对辩护人提出的上述辩护意见，本院不予采纳。

7、对辩护人提出坦白的辩护意见，与本院查明事实一致，本院予以采纳。对辩护人提出的其他辩护意见，与本院查明事实不一致，本院不予采纳。

对被告人张敏的指定辩护人提出的坦白且自愿认罪认罚的辩护意见，与本院查明事实一致，本院予以采纳。对辩护人提出的其他辩护意见，与本院查明事实不一致，本院不予采纳。

被告人苏兵的指定辩护人提出的自首且自愿认罪认罚、积极赔偿公益诉讼赔偿可以减轻处罚的辩护意见，与本院查明事实一致，本院予以采纳。对辩护人提出的其他辩护意见，与本院查明事实不一致，本院不予采纳。

对被告人叶明德的指定辩护人提出的自首且自愿认罪认罚的辩护意见，与本院查明事实一致，本院予以采纳。对辩护人提出的其他辩护意见，与本院查明事实不一致，本院不予采纳。

被告人杨桥、张敏归案后如实供述自己的罪行，且自愿认罪认罚，予以从轻处罚。

被告人苏兵、叶明德犯罪后自动投案，如实供述自己的罪行，系自首，且自愿认罪认罚，予以从轻处罚。

被告人杨桥、张敏、苏兵、叶明德对公益诉讼进行赔偿，可以从轻处罚。

扣押在案的野生动物制品猴骨、狮骨、穿山甲片，依法予以没收。被告人杨桥违法所得607.50元予以追缴没收，被告人张敏违法所得280元予以追缴没收，被告人苏兵违法所得180元予以追缴没收，被告人叶明德违法所得17.5元予以追缴没收。

对公诉机关提起的刑事附带民事公益诉讼，本院认为,被告人杨桥、张敏、苏兵、叶明德违反野生动物保护管理法律法规，未经许可非法收购、出售珍贵、濒危野生动物制品猴骨、狮骨、穿山甲片，损害了社会公共利益，是破坏生态环境的行为，根据《最高人民法院关于审理环境民事公益诉讼案件适用法律若干问题的解释》第十八条“对污染环境、破坏生态，已经损害社会公共利益或者具有损害社会公共利益重大风险的行为，原告可以请求被告承担停止侵害、排除妨碍、消除危险、恢复原状、赔偿

损失、赔礼道歉等民事责任”的规定，公益诉讼起诉人请求判令四被告人赔礼道歉于法有据，符合法律规定，本院予以支持。

对被告人杨桥、张敏、苏兵、叶明德应当赔偿的公益诉讼赔偿金数额的认定。本院认为，公益诉讼起诉人要求四被告人赔付的是因四被告人非法收购、出售珍贵、濒危野生动物制品造成的国家野生动物资源损失以及由此造成的对社会公共利益的损害，四被告人应对非法收购、出售珍贵、濒危野生动物制品的行为承担民事赔偿责任的诉讼请求于法有据，本院予以支持。

根据被告人犯罪的事实、性质、情节、对社会的危害程度和公诉机关的量刑建议，依照《中华人民共和国刑法》第三百四十一条第一款，第六十七条第一款、第三款，第七十二条第一款、第三款，第七十三条第二款、第三款，第五十二条，第五十三条，第六十四条，《中华人民共和国侵权责任法》第八条、第十五条和《最高人民法院关于审理环境民事公益诉讼案件适用法律若干问题的解释》第十八条之规定，判决如下：

一、被告人杨桥犯非法收购、出售珍贵、濒危野生动物制品罪，判处有期徒刑一年六个月，缓刑二年，并处罚金八千元；

（缓刑考验期从判决确定之日起计算。罚金在本判决生效之日起三日内一次性内缴纳。期满不缴纳的，强制缴纳。）

二、被告人张敏犯非法收购、出售珍贵、濒危野生动物制品罪，判处有期徒刑一年，缓刑二年，并处罚金五千元；

（缓刑考验期从判决确定之日起计算。罚金在本判决生效之日起三日内一次性内缴纳。期满不缴纳的，强制缴纳。）

三、被告人苏兵犯非法收购、出售珍贵、濒危野生动物制品罪，判处有期徒刑十个月，缓刑一年六个月，并处罚金四千元；

（缓刑考验期从判决确定之日起计算。罚金在本判决生效之日起三日内一次性内缴纳。期满不缴纳的，强制缴纳。）

四、被告人叶明德犯非法收购、出售珍贵、濒危野生动物制品罪，

判处有期徒刑十个月，缓刑一年六个月，并处罚金三千元；

（缓刑考验期从判决确定之日起计算。罚金在本判决生效之日起三日内一次性内缴纳。期满不缴纳的，强制缴纳。）

五、扣押在案的野生动物制品，予以没收，被告人杨桥违法所得 607.50 元予以追缴没收，被告人张敏违法所得 280 元予以追缴没收，被告人苏兵违法所得 180 元予以追缴没收，被告人叶明德违法所得 17.5 元予以追缴没收；

六、被告人杨桥、张敏、苏兵、叶明德在本判决生效后十日内在成都市级媒体上公开赔礼道歉；

七、被告人杨桥、叶明德于本判决发生法律效力之日起十日内共同赔偿因其行为造成的野生动物资源损失费 2,000 元，上缴国库；

八、被告人杨桥、张敏于本判决发生法律效力之日起十日内共同赔偿因其行为造成的野生动物资源损失费 15,000 元，上缴国库；

九、被告人杨桥、苏兵于本判决发生法律效力之日起十日内共同赔偿因其行为造成的野生动物资源损失费 5,440 元，上缴国库。

如不服本判决，可在接到判决书的第二日起十日内，通过本院或者直接向四川省成都市中级人民法院提出上诉。书面上诉的，应当提交上诉状正本一份，副本三份。

审　判　长　　张　元

审　判　员　　徐　敏

审　判　员　　李　力

人民陪审员　　罗顺菊

人民陪审员　　沈涤生

人民陪审员　　刘晓蓉

人民陪审员　　梁一峰

二〇二〇年六月五日

书　记　员　　向昊然

附：引用的相关法律条文：

《中华人民共和国刑法》

第三百四十一条非法猎捕、杀害国家重点保护的珍贵、濒危野生动物的，或者非法收购、运输、出售国家重点保护的珍贵、

濒危野生动物及其制品的，处五年以下有期徒刑或者拘役，并处罚金;情节严重的，处五年以上十年以下有期徒刑，并处罚金;情节特别严重的，处十年以上有期徒刑，并处罚金或者没收财产。

违反狩猎法规，在禁猎区、禁猎期或者使用禁用的工具、方法进行狩猎，破坏野生动物资源，情节严重的，处三年以下有期徒刑、拘役、管制或者罚金。

第六十七条犯罪以后自动投案，如实供述自己的罪行的，是自首。对于自首的犯罪分子，可以从轻或者减轻处罚。其中，犯罪较轻的，可以免除处罚。

被采取强制措施的犯罪嫌疑人、被告人和正在服刑的罪犯，如实供述司法机关还未掌握的本人其他罪行的，以自首论。

犯罪嫌疑人虽不具有前两款规定的自首情节，但是如实供述自己罪行的，可以从轻处罚;因其如实供述自己罪行，避免特别严重后果发生的，可以减轻处罚。

第七十二条对于被判处拘役、三年以下有期徒刑的犯罪分子，同时符合下列条件的，可以宣告缓刑，对其中不满十八周岁的人、怀孕的妇女和已满七十五周岁的人，应当宣告缓刑:

(一)犯罪情节较轻;

(二)有悔罪表现;

(三)没有再犯罪的危险;

(四)宣告缓刑对所居住社区没有重大不良影响。

宣告缓刑，可以根据犯罪情况，同时禁止犯罪分子在缓刑考验期限内从事特定活动，进入特定区域、场所，接触特定的人。

被宣告缓刑的犯罪分子，如果被判处附加刑，附加刑仍须执行。

第七十三条拘役的缓刑考验期限为原判刑期以上一年以下，但是不能少于二个月。

有期徒刑的缓刑考验期限为原判刑期以上五年以下，但是不能少于一年。

缓刑考验期限，从判决确定之日起计算。

第五十二条判处罚金，应当根据犯罪情节决定罚金数额。

第五十三条罚金在判决指定的期限内一次或者分期缴纳。期满不缴纳的，强制缴纳。对于不能全部缴纳罚金的，人民法院在任何时候发现被执行人有可以执行的财产，应当随时追缴。如果由于遭遇不能抗拒的灾祸缴纳确实有困难的，可以酌情减少或者免除。

第六十四条犯罪分子违法所得的一切财物，应当予以追缴或者责令退赔;对被害人的合法财产，应当及时返还;违禁品和供犯罪所用的本人财物，应当予以没收。没收的财物和罚金，一律上缴国库，不得挪用和自行处理。

《中华人民共和国侵权责任法》

第八条二人以上共同实施侵权行为，造成他人损害的，应当承担连带责任。

第十五条承担侵权责任的方式主要有：

（一）停止侵害；

（二）排除妨害；

（三）消除危险；

（四）返还财产；

（五）恢复原状；

（六）赔偿损失；

（七）赔礼道歉；

（八）消除影响、恢复名誉；

以上承担侵权责任的方式，可以单独适用，也可以合并适用。

《最高人民法院关于审理环境民事公益诉讼案件适用法律若干问题的解释》

第十八条对污染环境、破坏生态，已经损害社会公共利益或具有损害社会共同利益重大风险的行为，原告可以请求被告承担停止侵害、排除妨碍、消除危险、恢复原状、赔偿损失、赔礼道歉等民事责任。
