# Supplementary material for: China’s Legal Protection System for Pangolins: Past, Present, and Future
Source: Animals (Basel). 2025 Aug 18;15(16):2422. doi: 10.3390/ani15162422 (PMC12383201; doi:10.3390/ani15162422)
Supplement: Supplementary file 1 [file animals-15-02422-s001.zip › Supplementary Material S4-Full Text of Judgments in Pangolin-Related Public Interest Litigation Cases in China/【36】肖叶端非法收购、运输、出售珍贵、濒危野生动物、珍贵、濒危野生动物制品、走私、贩卖、运输、制造毒品一审刑事判决书.pdf]

肖叶端非法收购、运输、出售珍贵、濒危野生动物、珍贵、濒危野生动物制品、走私、贩卖、运输、制造毒品一审刑事判决书

云南省沧源佤族自治县人民法院

刑 事 附 带 民 事 判 决 书

（2020）云 0927 刑初 52 号

公诉机关暨附带民事公益诉讼起诉人云南省沧源佤族自治县人民检察院。

被告人暨附带民事公益诉讼被告肖叶端，女，云南省沧源族自治县人，佤族，小学文化，农民，户籍所在地云南省沧源佤族自治县，住云南省沧源佤族自治县。2019 年 9 月 6 日因涉嫌非法收购、运输、出售珍贵、濒危野生动物制品罪、贩卖毒品罪被沧源佤族自治县森林公安局刑事拘留，同年 10 月 4 日被依法逮捕。现羁押于耿马傣族佤族自治县看守所。

指定辩护人暨指定诉讼代理人李宏文，沧源佤族自治县法律援助中心律师。

云南省沧源佤族自治县人民检察院以沧检公诉刑诉〔2020〕54 号起诉书指控被告人肖叶端犯非法收购、运输、出售珍贵、濒危野生动物制品罪和贩卖毒品罪，于 2020 年 3 月 19 日向本院提起公诉。公益诉讼起诉人沧源佤族自治县人民检察院以沧检民公[2020]53092700001 号刑事附带民事公益诉讼起诉书，对被告人肖叶端犯非法收购、运输、出售珍贵、濒危野生动物制品罪，

于2020年4月20日向本院提起附带民事公益诉讼。经查，沧源佤族自治县人民检察院于2020年2月27日公告了案件相关情况，公告期内未有法律规定的机关和有关组织提起民事公益诉讼。本院依法组成合议庭，适用普通程序，于2020年5月12日和6月4日公开开庭进行了合并审理。沧源佤族自治县人民检察院指派检察员俸永军出庭支持公诉，指派检察员施福忠、李娅菊出庭履行职务，被告人暨附带民事公益诉讼被告肖叶端及指定辩护人暨指定诉讼代理人李宏文到庭参加了诉讼。本案现已审理终结。

公诉机关指控，被告人肖叶端自2013年起开始非法收购、运输、出售野生动物及其制品，2019年9月5日21时许，沧源佤族自治县森林公安局对其住所依法进行检查，在住房内和车牌号为云Ｓ×××××的白色吉利牌小型汽车后备箱内共查获疑似穿山甲甲片0.86千克、大象皮0.42千克、白鹇脚6个、熊指甲5个、麂子角5个、豪猪肚1个、蹄子3个、蹄筋1个、动物骨头3个、尾巴1个、角制品1个、动物胆1瓶、动物油1瓶，查获疑似野生动物豪猪完整死体2只、麂子完整死体2只。经云南濒科委司法鉴定中心司法鉴定，查获的0.86千克疑似穿山甲甲片来源于马来穿山甲，列入《濒危野生动植物种国际贸易公约》（ＣＩＴＣＳ）附录Ｉ，保护级别核定为Ｉ级，经济价值人民币76352元；0.42千克疑似象皮来源于亚洲象／非洲象，属国家Ｉ级保护动物，经济价值2100元；6个疑似白鹇脚来源于白鹇，

属国家ＩＩ级保护动物，经济价值人民币 12000 元。2018 年 12 月份，被告人肖叶端向不知名的缅甸籍老板以人民币 2600 元的价格收购了穿山甲甲片 1 市斤，并自行加工成粉末状，非法出售给董某 1，后追回 411.7 克。经云南濒科委司法鉴定中心司法鉴定，411.7 克疑似穿山甲甲片粉末来源于马来穿山甲，列入《濒危野生动植物种国际贸易公约》（ＣＩＴＣＳ）附录Ｉ，保护级别核定为Ｉ级，经济价值人民币 36544 元。2019 年 7 月份，被告人肖叶端向不知名的缅甸籍男子以人民币 1000 元购得疑似毒品鸦片可疑物 95.23 克，后以人民币 1600 元的价格出售给张某 1。经云南省沧源佤族自治县公安司法鉴定中心鉴定，毒品可疑物为鸦片。为证实上述指控，公诉机关当庭宣读、出示了查获的野生动物制品等相关物证照片，扣押决定书、扣押笔录等书证，抓获经过、张某 1、董晓云等人的证言，被告人肖叶端的供述与辩解，鉴定意见，检查、指认笔录，讯问同步录音录像等视听资料等证据。公诉机关认为，被告人肖叶端违反野生动物保护法规，非法收购、运输、出售国家重点保护的珍贵、濒危野生动物制品，经济价值人民币 126996 元，情节严重，其行为触犯了《中华人民共和国刑法》第三百四十一条之规定，犯罪事实清楚，证据确实、充分，应当以非法收购、运输、出售珍贵、濒危野生动物制品罪追究其刑事责任。建议对其判处有期徒刑五年零六个月，并处罚金人民币 20000 元；被告人肖叶端明知是毒品鸦片还故意贩卖，数量达 95.23 克，其行为触犯了《中华人民共和国刑法》第

三百四十七条第四款之规定，犯罪事实清楚，证据确实、充分，应当以贩卖毒品罪追究其刑事责任。建议对其判处有期徒刑一年零六个月，并处罚金人民币5000元。同时认为被告人肖叶端自愿如实供述涉嫌的犯罪事实，对指控的犯罪没有异议，自愿接受刑事处罚，可以依法从宽处理。

附带民事公益诉讼起诉人沧源佤族自治县人民检察院向本院提出诉讼请求：一、判令肖叶端对其收购、出售珍贵动物制品的行为在县级有影响力的媒体上公开赔礼道歉；二、判令肖叶端承担生态资源损害补偿费共计人民币126,996元。

事实和理由：被告肖叶端在明知国家禁止的情况下，长期大量收购、出售珍贵野生动物及其制品，其行为造成国家资源受损，侵害了社会公共利益。本案查获的野生动物及其制品经济价值共计人民币126,996元。为证实上述刑事附带民事公益诉讼，沧源佤族自治县人民检察院当庭宣读、出示了上述刑事部分的证据及公告、情况说明等证据。附带民事公益诉讼起诉人沧源佤族自治县人民检察院认为，野生动物是生物多样性和自然生态系统的重要组成部分，在维护生态平衡、保障人类生存环境方面具有不可替代的重要作用，保护野生动物，不仅关乎大自然的生态平衡，也关乎到国家生态安全，对人类的生存与发展有着重大的意义。《中华人民共和国野生动物保护法》第三条：“野生动物资源属于国家所有。”“没有买卖就没有杀戮”，被告肖叶端长期大量收购、出售野生动物及其制品，其行为破坏了野生动物资源、损

害了社会公共利益，应当承担相应民事责任。根据《中华人民共和国民事诉讼法》第五十五条第二款、《最高人民法院、最高人民检察院关于检察公益诉讼案件适用法律若干问题的解释》第二十条的规定，提起附事民事公益诉讼。

被告人暨附带民事公益诉讼被告肖叶端对指控事实、罪名及量刑建议没有异议，且签字具结，在开庭审理过程中亦无异议。对附带民事诉讼部分也没有异议。其指定辩护人的辩护意见是：1.被告人肖叶端归案后对自己的犯罪行为供认不讳，坦白认罪，认罪态度较好，有真诚的悔罪表现，自愿认罪认罚，应当依法从宽处罚；2.被告人肖叶端以往无违法犯罪记录，属于初犯、偶犯，应当依法对其从轻处罚；3、被告人肖叶端是边疆少数民族边民，由于不学法不懂法，长期以来本地存在以野生动物制品和鸦片作为医治某些疾病的习惯，所以被告人为谋取利益而构成犯罪是有一定的社会根源，请法庭在对其量刑时酌情处罚。综上所述，请法庭对被告人肖叶端依法从轻处罚。指定诉讼代理人对民事部分的代理意见是：被告人肖叶端收购、出售珍贵、濒危野生动物制品的行为已经造成国家资源受损，侵害了社会公共利益，被告人在接受刑事处罚的同时依法应承担生态资源损害的补偿。法庭应当根据被告人肖叶端犯罪的社会根源、危害程度，自愿认罪认罚、悔罪表现，以及被告人的经济能力等各方面综合考虑，作出公正的判决。

经审理查明：

一、非法收购、运输、出售珍贵、濒危野生动物制品罪

被告人肖叶端自2013年起开始非法收购、运输、出售野生动物及其制品。2019年9月5日21时许，沧源佤族自治县森林公安局对其住所依法进行检查，在住房内和车牌号为云Ｓ×××××的白色吉利牌小型汽车后备箱内共查获疑似穿山甲甲片0.86千克、大象皮0.42千克、白鹇脚6个、熊指甲5个、麂子角5个、豪猪肚1个、蹄子3个、蹄筋1个、动物骨3个、尾巴1个、角制品1个、动物胆1瓶、动物油1瓶，查获疑似野生动物豪猪完整死体2只、麂子完整死体2只。经云南濒科委司法鉴定中心司法鉴定，查获的0.86千克疑似穿山甲甲片来源于马来穿山甲，列入《濒危野生动植物种国际贸易公约》（ＣＩＴＣＳ）附录Ｉ，保护级别核定为Ｉ级，经济价值人民币76352元；0.42千克疑似大象皮来源于亚洲象／非洲象，属国家Ｉ级保护动物，经济价值人民币2100元；6个疑似白鹇脚来源于白鹇，属国家ＩＩ级保护动物，经济价值人民币12000元。

2018年12月份，被告人肖叶端向不知名的缅甸籍老板以人民币2600元的价格收购了穿山甲甲片1市斤，并自行加工成粉末状，非法出售给董某1，后追回411.7克。经云南濒科委司法鉴定中心司法鉴定，411.7克疑似穿山甲甲片粉末来源于马来穿山甲，列入《濒危野生动植物种国际贸易公约》（ＣＩＴＣＳ）附录Ｉ，保护级别核定为Ｉ级，经济价值人民币36544元。

二、贩卖毒品罪

2019年7月份，被告人肖叶端向不知名的缅甸籍男子以1000元人民币购得疑似毒品可疑物95.23克，后以人民币1600元的价格出售给张某1。经云南省沧源佤族自治县公安司法鉴定中心鉴定，毒品可疑物为鸦片。

上述事实，有经庭审质证、认证的下列证据证实，本院予以确认：

1、户口证明，证实被告人肖叶端已达刑事责任和民事年龄，且无犯罪记录的事实。

2、抓获经过，证实2019年9月5日21时许，沧源佤族自治县森林公安局侦查人员在被告人肖叶端家和白色吉利牌小型汽车内获大量野生动物及其制品，并将其带回沧源佤族自治县森林公安局接受讯问的事实。

3、物证照片，证实本案查获的野生动物及其制品、毒品鸦片，以及作案用的车辆、手机等照片，经被告人肖叶端当庭辨认无异议的事实。

4、检查、提取笔录和照片、扣押决定书、扣押笔录、扣押清单，证实在被告人肖叶端家中和云S×××××白色吉利牌小型汽车后备箱内查获其非法收购、运输、出售的野生动物制品的事实。

5、称量、取样和移交物品清单笔录，证实在被告人肖叶端家中查获的野生动物制品中，经称量穿山甲甲片为0.86千克、大象皮0.42千克；被告人肖叶端贩卖给董某1的穿山甲粉为

411.7 克；贩卖给张某 1 的鸦片共 95.23 克，并随机取样 4.32 克，其余毒品鸦片 90.91 克已移交沧源佤族自治县公安局禁毒大队的事实。

6、证人证言

（1）证人张某 1、姚某的证言，证实 2019 年 8 月 5 日，张某 1 帮助姚某以人民币 1600 元的价格与被告人肖叶端购得鸦片二两，以及帮助姚某与被告人肖叶端以人民币 4000 元购得 10 克犀牛角的事实。

（2）证人董某 1、张某 2 的证言，证实 2018 年 12 月份左右，以人民币 3000 元与被告人肖叶端购得穿山甲甲粉一市斤，被公安机关追回 411.7 克的事实。

（3）证人董某 2 的证言，证实 2017 年 6 月左右，在沧源佤族自治县被告人肖叶端家与其穿购得穿山甲甲粉的事实。

（4）证人杨某（系被告人肖叶端丈夫）的证言，证实在其家中查获的穿山甲甲片系被告人肖叶端与境外缅甸人购买的事实。

7、被告人的供述，其所作供述能够与证人证言、现场指认笔录及照片等证据相吻合，证实其于 2013 年以来，多次非法收购、运输、出售珍贵、濒危野生动物制品，另于 2019 年 7 月底以人民币 1000 元购得毒品鸦片 2 两左右，并以人民币 1600 元的价格贩卖给张某 1 的事实。

8、鉴定意见书

（1）云南濒科委司法鉴定中心濒司鉴（动）[2019]1534 号和濒司鉴（动）[2019]1681 号司法鉴定意见书，证实被告人肖叶端非法收购、运输、出售的穿山甲甲片（粉）、象皮、白鹇脚等系珍贵、濒危的野生动物制品，共计经济价值人民币 126,996 元的事实。

（2）云南省沧源佤族自治县公安司法鉴定中心（沧）公（司）鉴（毒检）[2019]169 号物证检验报告，证实送检的“肖叶端涉嫌非法收购、运输、出售珍贵、濒危野生动物制品”1 号检材中检出毒品鸦片成分的事实。

9、现场指认笔录及照片，证实 2019 年 9 月 6 日被告人肖叶端对查获其非法收购、运输、出售的野生动物制品和毒品鸦片进行指认的事实。

10、现场平面和方位示意图，证实查获被告人肖叶端非法收购、运输、出售珍贵、濒危的野生动物及其制品的地点的事实。

11、电子物证检查工作记录，经对被告人肖叶端的手机进行电子数据分析，从微信电子截屏图片可以证实被告人肖叶端非法出售给张某 1、董晓云等人珍贵、濒危野生动物制品及毒品鸦片后，通过手机微信转账交易的事实。

12、公告，证明沧源佤族自治县人民检察院于 2020 年 2 月 27 日在正义网刊登了沧检民公线受[2020]53092700001 号公告。

13、情况说明，证明在沧源佤族自治县人民检察院发布公告期内未有法律规定的机关和有关组织提起民事公益诉讼，其符合提起附带民事公益诉讼的主体资格。

本院认为，被告人肖叶端违反野生动物保护法规，多次非法收购、运输、出售国家重点保护的珍贵、濒危野生动物制品，经济价值人民币126,996元，情节严重，其行为已触犯刑律，构成非法收购、运输、出售珍贵、濒危野生动物制品罪；被告人肖叶端违反国家对毒品的管理规定，明知是毒品鸦片故意贩卖，数量达95.23克，其行为已触犯刑律，构成贩卖毒品罪。公诉机关指控的事实和罪名成立，予以支持。被告人肖叶端犯罪以后如实供述犯罪事实，系坦白，可以从轻处罚，且其自愿认罪认罚，可以依法从宽处理。对指定辩护人提出被告人肖叶端是偶犯及长期以来本地存在以野生动物制品和鸦片作为医治某些疾病的习惯是其构成犯罪的社会根源的辩护意见不予采纳，其余辩护意见予以采纳。公诉机关的量刑建议适当，予以采纳。附带民事公益诉讼被告肖叶端尽管不是直接猎杀者，但其行为对于造成生态资源损害具有直接因果关系，其实施的非法收购、运输、出售珍贵、濒危野生动物制品犯罪行为，严重破坏了国家野生动物资源及生态环境，进而影响了生态平衡和生物多样性发展，损害了社会公共利益，依法应承担相应的民事责任。故附带民事公益诉讼起诉人要求附带民事公益诉讼被告肖叶端向社会公开赔礼道歉并承担生态资源损害补偿费的诉求成立，本院予以支持。综上所述，根

据被告人肖叶端的犯罪事实、性质、情节及对社会的危害程度，依据《中华人民共和国刑法》第三百四十一条第一款，第三百四十七条第四款、第五十二条，第五十三条第一款，第六十四条、第六十七条第三款，第六十九条第一款、第三款，第三十六条，《最高人民法院关于审理破坏野生动物资源刑事案件具体应用法律若干问题的解释》第五条第一款，以及《中华人民共和国野生动物保护法》第三条第一款，第二十七条第一款，第三十五条第一款、第二款、第四款，《中华人民共和国侵权责任法》第四条第一款，第六条第一款，第十五条，《最高人民法院〈关于审理环境民事公益诉讼案件适用法律若干问题的解释〉》第十八条之规定，判决如下：

一、被告人肖叶端犯非法收购、运输、出售珍贵、濒危野生动物制品罪，判处有期徒刑五年零六个月，并处罚金人民币二万元；被告人肖叶端犯贩卖毒品罪，判处有期徒刑一年零六个月，并处罚金五千元。数罪并罚，决定执行有期徒刑六年零六个月，并处罚金人民币二万五千元。

（刑期从判决执行之日起计算，判决执行以前先行羁押的，羁押一日折抵刑期一日，即自 2019 年 9 月 6 日起至 2026 年 3 月 5 日止，罚金待判决生效后缴纳）。

二、附带民事公益诉讼被告肖叶端在本判决生效后三十日内对其非法收购、运输、出售珍贵、濒危野生动物制品的行为在县级有影响力的媒体上公开赔礼道歉。

三、附带民事公益诉讼被告肖叶端在本判决生效后三十日内缴纳生态资源损害补偿费共计人民币126,996元，上缴国库。

四、查获的作案工具白色吉利牌小型车辆一辆（牌号为云Ｓ×××××）、ＯＰＰＯ牌Ａ57手机一部，以及查获的所有野生动物制品和毒品鸦片95.23克予以没收，由公安机关按规定处理。

如不服本判决，可在接到判决书的第二日起十日内，通过本院或者直接向云南省临沧市中级人民法院提出上诉。书面上诉的，应当提交上诉状正本一份、副本二份。

审　判　长　　杨庆庭

审　判　员　　段立昌

审　判　员　　李建文

人民陪审员　　陈春林

人民陪审员　　赵云芳

人民陪审员　　肖文明

人民陪审员　　李海亮

二〇二〇年六月四日

书　记　员　　李智凯
