# Supplementary material for: China’s Legal Protection System for Pangolins: Past, Present, and Future
Source: Animals (Basel). 2025 Aug 18;15(16):2422. doi: 10.3390/ani15162422 (PMC12383201; doi:10.3390/ani15162422)
Supplement: Supplementary file 1 [file animals-15-02422-s001.zip › Supplementary Material S4-Full Text of Judgments in Pangolin-Related Public Interest Litigation Cases in China/【37】郭德全非法收购、运输、出售珍贵、濒危野生动物、珍贵、濒危野生动物制品一审刑事判决书.pdf]

郭德全非法收购、运输、出售珍贵、濒危野生动物、珍贵、濒危野生动物制品一审刑事判决书

云南省大理市人民法院

刑 事 附 带 民 事 判 决 书

（2020）云 2901 刑初 116 号

公诉机关暨附带民事公益诉讼起诉人：云南省大理市人民检察院。

被告人暨附带民事公益诉讼被告：郭德全，男性，1959 年 12 月 4 日出生于云南省洱源县，白族，高中文化，农民，住云南省大理州洱源县。因涉嫌犯非法收购珍贵、濒危野生动物制品罪，于 2019 年 7 月 17 日被大理市森林公安局取保候审，2019 年 9 月 5 日被大理市人民检察院取保候审，2020 年 3 月 9 日被本院取保候审,同年 5 月 18 日本院决定逮捕，次日大理市森林公安局执行逮捕。现羁押于洱源县看守所。

大理市人民检察院以大检民环刑诉〔2020〕114 号起诉书指控被告人郭德全犯非法收购珍贵、濒危野生动物制品罪，于 2020 年 3 月 5 日向本院提起公诉。公益诉讼起诉人大理市人民检察院，于同日以大检民公（2019）53290100005 号民事公益诉讼起诉书向本院提起附带民事诉讼。经查，大理市人民检察院于 2019 年 12 月 20 日公告了案件相关情况，公告期内未有法律规定的机关和有关组织提起民事公益诉讼。本院依法组成合议庭，于 2020 年 4 月 21 日公开开庭审理了本案。大理市人民检察院指派检察

员杨扬、书记员唐非出庭履行职务，被告人暨附带民事公益诉讼被告郭德全到庭参加诉讼。本案现已审理终结。

公诉机关大理市人民检察院指控：2019年5月1日，大理市森林公安局在大理三月街民族节药材市场E2区东19号藏药祖传秘方店铺内查获被告人郭德全于2019年4月中旬购买自用的共计637.03克疑似野生动物鳞片2袋。

经云南濒科委司法鉴定中心鉴定，被查获的疑似野生动物鳞片来源于鳞甲目穿山甲科穿山甲属，穿山甲为国家Ⅱ级保护动物，估算被查获的穿山甲为1.36只，经济价值共计人民币43520元。被告人郭德全归案后如实供述罪行。针对指控的事实，公诉机关提供了物证、书证、证人证言、鉴定意见、被告人供述和辩解等证据证实。公诉机关认为，被告人郭德全的行为已构成非法收购珍贵、濒危野生动物制品罪，鉴于被告人具有如实供述情节，建议对其所犯的非法收购珍贵、濒危野生动物制品罪判处有期徒刑六个月至一年零六个月，并处罚金。

附带民事公益诉讼起诉人大理市人民检察院向本院提出诉讼请求：1.判令附带民事公益诉讼被告郭德全赔偿野生动物资源损失人民币43520元（当庭释明该费用请法院依法合理确定），并将赔偿款交由林业草原主管部门代为履行修复国家保护的珍贵、濒危野生动物资源和生态环境；2.鉴于本案危害公共健康安全，判令附带民事公益诉讼被告郭德全在大理白族自治州、大理市两级媒体上公开向社会公众赔礼道歉。事实和理由：郭德全非

法收购珍贵、濒危野生动物制品罪，严重破坏了野生动物资源和生物多样性保护，危害人民群众生命健康安全，损害了国家和社会公共利益，造成国家经济损失。附带民事公益诉讼起诉人向本院提交了来源于上海市嘉定区生态环境局网站2017年6月19日发布的《为何要保护穿山甲》一文的打印件一份；来源于中国发展门户网的世界级动物保护协会高级科学顾问孙全辉博士于2016年6月16日发布的关于野生动物食用角度的认识打印件一份。

被告人暨附带民事公益诉讼被告郭德全辩称，对公诉机关指控的事实、罪名、量刑均无异议，且自愿认罪，请求判处缓刑；愿意赔礼道歉；生态环境修复费用适当减少。

经审理查明：2019年5月1日，大理市森林公安局根据工作安排，到大理市三月街民族节对商品是否存在买卖野生动物制品进行突击检查。在大理三月街民族节药材市场E2区东19号藏药祖传秘方店铺内查获并扣押了被告人郭德全于2019年4月中旬购买自用的共计637.03克疑似野生动物鳞片2袋。被告人郭德全归案后并如实供述罪行。

经云南濒科委司法鉴定中心鉴定，被查获的疑似野生动物鳞片来源于鳞甲目穿山甲科穿山甲属，穿山甲为国家Ⅱ级保护动物，估算被查获的穿山甲为1.36只，经济价值共计人民币43520元。

另查明，生态修复费用无法鉴定，对收购穿山甲鳞片的行为潜在危害公共健康安全的危害性目前无专家意见出具。

上述刑事部分的事实，公诉机关提供了物证、书证、证人证言、鉴定意见、被告人供述等证据证实，足以认定。民事部分的事实，附带民事公益诉讼起诉人向本院提交的两份网络打印件，不具备证据的三性，本院不予采信。

本院认为，被告人郭德全非法收购珍贵、濒危野生动物穿山甲鳞片，其行为已构成非法收购珍贵、濒危野生动物制品罪。应处五年以下有期徒刑或者拘役，并处罚金。被告人具有如实供述情节，庭审中自愿认罪，对其可以从轻处罚。

关于附带民事公益诉讼起诉人提出的两个诉讼请求。因附带民事公益诉讼起诉人未提供相应的证据，证实被告人的行为对该野生动物资源、公众健康安全有何种危害及相关修复费用的具体数额。虽被告人郭德全自认对生态有一定破坏并愿意赔偿部分生态修复费用，但该自认不符合《最高人民法院关于民事诉讼证据的若干规定》第八条的规定，即涉及可能损害国家利益、社会公共利益的事实，不适用自认规定。故附带民事公益诉讼起诉人提出要求附带民事公益诉讼被告郭德全赔偿野生动物资源损失43520 元及在大理白族自治州、大理市两级媒体上公开向社会公众赔礼道歉的诉请无证据证实，应由其承担不利的法律后果，故本院对其诉请不予支持。

综上，公诉机关指控的事实成立、量刑适当，本院予以采纳。根据被告人犯罪的事实、犯罪的性质、情节及社会危害性。依照《中华人民共和国刑法》第三百四十一条第一款、第六十七条第三款、第四十五条、第四十七条、第五十二条、第五十三条、第六十一条、第六十四条，《中华人民共和国民事诉讼法》第六十四条、第一百一十九条，《最高人民法院关于适用〈中华人民共和国民事诉讼法〉的解释》第九十条、第九十一条、第九十二条第二款、第九十六条第一款，《最高人民法院关于民事诉讼证据的若干规定》第八条之规定，判决如下：

一、被告人郭德全犯非法收购珍贵、濒危野生动物制品罪，判处有期徒刑六个月，并处罚金人民币 10000 元。（刑期从判决执行之日起计算，判决执行以前先行羁押的，羁押一日折抵刑期一日。即即自 2020 年 5 月 19 日起至 2020 年 11 月 18 日止。罚金限判决生效之日起三十日内缴清。）

二、驳回附带民事公益诉讼起诉人云南省大理市人民检察院的全部诉讼请求。

三、扣押在案的野生动物穿山甲制品予以没收，由扣押机关依法处理。

如不服本判决，可在接到判决书的第二日起十日内，通过本院或者直接向大理白族自治州中级人民法院提出上诉。书面上诉的，应当提交上诉状正本一份，副本三份。

审　判　长　　陈道谦

审　判　员　　李琳平

审　判　员　　何海瑛

人民陪审员　　郭文旗

人民陪审员　　孙国忠

人民陪审员　　王嘉全

人民陪审员　　李淑琴

二〇二〇年五月二十日

书　记　员　　周　浩

附页

《中华人民共和国刑法》

第三百四十一条第一款非法猎捕、杀害国家重点保护的珍贵、濒危野生动物的，或者非法收购、运输、出售国家重点保护的珍贵、濒危野生动物及其制品的，处五年以下有期徒刑或者拘役，并处罚金；情节严重的，处五年以上十年以下有期徒刑，并处罚金；情节特别严重的，处十年以上有期徒刑，并处罚金或者没收财产。

第六十七条第三款犯罪嫌疑人虽不具有前两款规定的自首情节，但是如实供述自己罪行的，可以从轻处罚；因其如实供述自己罪行，避免特别严重后果发生的，可以减轻处罚。

第四十五条有期徒刑的期限，除本法第五十条、第六十九条规定外，为六个月以上十五年以下。

第四十七条有期徒刑的刑期，从判决执行之日起计算；判决执行以前先行羁押的，羁押一日折抵刑期一日。

第五十二条判处罚金，应当根据犯罪情节决定罚金数额。

第五十三条罚金在判决指定的期限内一次或者分期缴纳。期满不缴纳的，强制缴纳。对于不能全部缴纳罚金的，人民法院在任何时候发现被执行人有可以执行的财产，应当随时追缴。

由于遭遇不能抗拒的灾祸缴纳确实有困难的，经人民法院裁定，可以延期缴纳、酌情减少或者免除。

第六十一条对于犯罪分子决定刑罚的时候，应当根据犯罪的事实、犯罪的性质、情节和对于社会的危害程度，依照本法的有关规定判处。

第六十四条犯罪分子违法所得的一切财物，应当予以追缴或者责令退赔；对被害人的合法财产，应当及时返还；违禁品和供犯罪所用的本人财物，应当予以没收。没收的财物和罚金，一律上缴国库，不得挪用和自行处理。

《中华人民共和国民事诉讼法》

第六十四条当事人对自己提出的主张，有责任提供证据。

当事人及其诉讼代理人因客观原因不能自行收集的证据，或者人民法院认为审理案件需要的证据，人民法院应当调查收集。

人民法院应当按照法定程序，全面地、客观地审查核实证据。

第一百一十九条起诉必须符合下列条件：

（一）原告是与本案有直接利害关系的公民、法人和其他组织；

（二）有明确的被告；

（三）有具体的诉讼请求和事实、理由；

（四）属于人民法院受理民事诉讼的范围和受诉人民法院管辖。

最高人民法院关于适用《中华人民共和国民事诉讼法》的解释

第九十条当事人对自己提出的诉讼请求所依据的事实或者反驳对方诉讼请求所依据的事实，应当提供证据加以证明，但法律另有规定的除外。

在作出判决前，当事人未能提供证据或者证据不足以证明其事实主张的，由负有举证证明责任的当事人承担不利的后果。

第九十一条人民法院应当依照下列原则确定举证证明责任的承担，但法律另有规定的除外：

（一）主张法律关系存在的当事人，应当对产生该法律关系的基本事实承担举证证明责任；

（二）主张法律关系变更、消灭或者权利受到妨害的当事人，应当对该法律关系变更、消灭或者权利受到妨害的基本事实承担举证证明责任。

第九十二条一方当事人在法庭审理中，或者在起诉状、答辩状、代理词等书面材料中，对于己不利的事实明确表示承认的，另一方当事人无需举证证明。

对于涉及身份关系、国家利益、社会公共利益等应当由人民法院依职权调查的事实，不适用前款自认的规定。

自认的事实与查明的事实不符的，人民法院不予确认。

第九十六条第一款民事诉讼法第六十四条第二款规定的人民法院认为审理案件需要的证据包括：

（一）涉及可能损害国家利益、社会公共利益的；

（二）涉及身份关系的；

（三）涉及民事诉讼法第五十五条规定诉讼的；

（四）当事人有恶意串通损害他人合法权益可能的；

（五）涉及依职权追加当事人、中止诉讼、终结诉讼、回避等程序性事项的。

《最高人民法院关于民事诉讼证据的若干规定》

第八条《最高人民法院关于适用〈中华人民共和国民事诉讼法〉的解释》第九十六条第一款规定的事实，不适用有关自认的规定。

自认的事实与已经查明的事实不符的，人民法院不予确认。
