# Supplementary material for: China’s Legal Protection System for Pangolins: Past, Present, and Future
Source: Animals (Basel). 2025 Aug 18;15(16):2422. doi: 10.3390/ani15162422 (PMC12383201; doi:10.3390/ani15162422)
Supplement: Supplementary file 1 [file animals-15-02422-s001.zip › Supplementary Material S4-Full Text of Judgments in Pangolin-Related Public Interest Litigation Cases in China/【38】田少波、黄嗣通故意损毁文物一审刑事判决书.pdf]

田少波、黄嗣通故意损毁文物一审刑事判决书

江西省浮梁县人民法院

刑 事 附 带 民 事 判 决 书

（2020）赣0222刑初24号

公诉机关暨附带民事公益诉讼起诉人：浮梁县人民检察院。

被告人暨附带民事公益诉讼被告：田少波，男，1969年4月3日出生于浙江省庆元县，汉族，中专文化，江西省吉安市农业局工作人员，公民身份号码：。户籍及居住地：江西省吉安市吉州区正丙角路4栋101室。因本案于2019年5月7日被浮梁县公安局民警抓获，同年5月8日被刑事拘留，2019年6月14日被执行逮捕。现羁押于景德镇市看守所。

辩护人吴文平、付颖，江西法烁智律师事务所律师。

被告人暨附带民事公益诉讼被告：黄嗣通，绰号“长嘴巴”，男，1970年4月29日出生于浙江省庆元县，汉族，小学肄业文化，无业，公民身份号码：。户籍地：浙江省庆元县隆官乡隆官村山头岗1号，居住地：浙江省庆元县龙山苑7单元402室。因本案于2019年5月28日主动到浙江省庆元县公安局投案并临时羁押于庆元县看守所，同年5月30日被江西省浮梁县公安局刑事拘留，2019年6月14日被执行逮捕。现羁押于景德镇市看守所。

被告人暨附带民事公益诉讼被告：吴小胡，曾用名吴小吴，男，1974年9月25日出生于浙江省庆元县，汉族，小学肄业文

化，农民，公民身份号码：。户籍地：浙江省庆元县隆官乡源头村12号，居住地：浙江省庆元县松源街道菇源路100号301室。因本案于2019年5月28日主动到浙江省庆元县公安局投案并临时羁押于庆元县看守所，同年5月30日被江西省浮梁县公安局刑事拘留，2019年6月14日被执行逮捕。现羁押于景德镇市看守所。

被告人暨附带民事公益诉讼被告：周上飞，男，1977年3月20日出生于浙江省庆元县，汉族，初中肄业文化，无业，公民身份号码：。户籍地：浙江省庆元县淤上乡长砻村40号，居住地：浙江省庆元县松源镇希望路出租屋。因本案于2019年5月14日被浮梁县公安局民警抓获，同年5月15日被刑事拘留，2019年6月14日被执行逮捕。现羁押于景德镇市看守所。

辩护人暨委托诉讼代理人：祝亚辉，江西千瓷律师事务所律师。

浮梁县人民检察院以浮检刑检刑诉[2020]4号起诉书指控被告人田少波、黄嗣通、吴小胡、周上飞犯故意损毁文物罪案，于2020年1月21日向本院提起公诉。公益诉讼起诉人浮梁县人民检察院于2020年2月20日以浮检民公[2019]3602220001号刑事附带民事公益诉讼起诉书向本院提起附带生态环境损害赔偿民事公益诉讼。经查，浮梁县人民检察院于2019年12月4日公告了案件相关情况，公告期内未有法律规定的机关和有关组织提起民事公益诉讼。本院依法组成合议庭，于2020年4月2日公

开开庭审理了本案。浮梁县人民检察院指派检察员鄢某、石某出庭履行职务，被告人暨附带民事公益诉讼被告田少波及其辩护人付颖、被告人暨附带民事公益诉讼被告黄嗣通、吴小胡、被告人暨附带民事公益诉讼被告周上飞及其辩护人暨委托诉讼代理人祝亚辉到庭参加了诉讼。现已审理终结。

公诉机关指控：

一、2019 年 2 月 15 日至 17 日期间，被告人田少波伙同黄嗣通、吴小胡、周上飞携带撬棍、铁锹等工具，驾驶赣Ａ×××**绿色猎豹越野车来到江西省浮梁县旧城红塔一层内室地面进行盗挖，2019 年 2 月 18 日凌晨 1 时许，被告四人盗挖红塔时，被工作人员发现，四被告人逃离现场。经查，被盗挖红塔（又名西塔）位于浮梁县浮梁镇旧城村，于 1987 年被江西省人民政府公布为第三批省级文物保护单位。

二、2019 年 4 月下旬至 5 月初，被告人田少波伙同吴某、许某、杨某（均另处）多次趁夜钻入位于江西省九江市濂溪区西林寺塔，使用铁锹、螺丝刀、撬棍对西林寺塔进行盗挖。经查，被盗挖西林寺，于 1959 年被江西省人民政府公布为第二批省级文物保护单位。

针对上述指控，公诉机关提供了证人证言、被告人供述与辩解、现场勘验笔录及照片、辨认笔录及相关书证、现场视频等证据。公诉机关认为被告人田少波伙同他人损毁二处省级文物保护单位的文物，构成故意损毁文物罪，在共同犯罪中起主要作用，

系主犯，归案后如实供述自己罪行，且自愿认罪认罚，建议判处有期徒刑二年十个月，并处罚金；被告人黄嗣通、吴小胡、周上飞的行为构成故意损毁文物罪，其中被告人黄嗣通、吴小胡在共同犯罪中起主要作用，系主犯，案发后主动投案并如实供述自己罪行，自愿认罪认罚；被告人周上飞在共同犯罪中起次要作用，归案后如实供述自己的罪行，且自愿认罪认罚，建议对被告人黄嗣通、吴小胡、周上飞均分别判处有期徒刑十个月，并处罚金。经本院建议，庭审中，公诉机关重新调整量刑建议：建议判处被告人田少波有期徒刑二年八个月至三年，并处罚金；建议判处被告人黄嗣通有期徒刑八个月至一年，并处罚金；建议判处被告人吴小胡有期徒刑八个月至一年，并处罚金；建议判处被告人周上飞有期徒刑八个月至一年，并处罚金。

附带民事公益诉讼起诉人浮梁县人民检察院向本院提出诉讼请求：1、刑事附带民事公益诉讼被告田少波、黄嗣通、吴小胡、周上飞连带承担红塔基础整修回填费、聘请专家费共计人民币 8122.69 元；2.判令四被告人对本次盗挖红塔的行为在江西省省级新闻媒体上向社会公众赔礼道歉。事实和理由：被损红塔系江西省人民政府公布为第三批省级文物保护单位，四被告人违反文物保护法等相关法律法规的规定，盗挖红塔，损害了社会公共利益。经景德镇市陶瓷考古研究所现场查看，被盗挖红塔的塔体中心近似长方形的盗洞长 75 厘米、宽 38 厘米，深入地面约 89 厘米，共计有 10 层青砖地基遭到缺失性破坏；盗洞深入塔基范

围，破坏了部分塔体基础，极易造成塔基结构失衡，对文物本体造成较大损害，为此，浮梁县文物管理所聘请相关专业人员对红塔进行抢救性回填，花去相应费用共计人民币 8122.69 元。为支持上述诉请，公益诉讼机关出示了修复报告、工资表、情况说明、结算总价、发票、公益诉讼公告等证据。

被告人暨附带民事公益诉讼被告田少波辩称：1、其未提议和组织盗挖红塔及西林寺塔；2、其是抱着捡漏的心态去了红塔及西林寺塔，但均未动手；3、作案工具也非其准备的；4、对附带民事公益诉讼起诉人的诉请，表示愿意赔偿及道歉。其辩护人提出的辩护意见是：1、被告人田少波并非犯意提起者，且没有从事挖掘的行为，只是从事清土行为，在共同犯罪中不构成主犯；2、被告人田少波如实供述自己的罪行且认罪认罚，应当从轻处罚；3、本案各被告人属临时起意，主观恶性较小，尚未造成难以挽回的后果。

被告人暨附带民事公益诉讼被告黄嗣通、吴小胡、周上飞对公诉机关指控的事实、罪名不持异议；对附带民事公益诉讼起诉人的诉请，均答辩称愿意赔偿及道歉。

被告人周上飞的辩护人暨委托诉讼代理人意见是：1、被告人周上飞归案后如实供述自己罪行，属坦白；2、属初犯、偶犯；3、对附带民事公益诉讼起诉人的诉请无异议，表示认可。

经审理查明：

一、被告人田少波、黄嗣通、吴小胡、周上飞盗挖红塔的事实

2019年2月5日，被告人田少波独自来到江西省浮梁县并租下一民房住下，2019年2月9日前后，被告人田少波纠集被告人黄嗣通、吴小胡、周上飞从浙江省庆元县来到江西省浮梁县，预谋盗挖浮梁县旧城红塔。2019年2月15日至17日，被告人田少波伙同黄嗣通、吴小胡、周上飞携带撬棍、铁锹等工具，驾驶赣A×××**绿色猎豹越野车来到江西省浮梁县旧城，多次对红塔进行盗挖。被告人田少波首先利用工具对红塔一层内室进行挖掘，然后指挥黄嗣通、吴小胡盗挖，被告人周上飞负责望风，为避免被发现，每晚离开时再对盗挖口进行掩饰。2019年2月18日凌晨1时许，四被告人盗挖红塔时，被管理人员发现，四被告人逃离现场。经查，被盗挖红塔（又名西塔）位于浮梁县浮梁镇旧城村，于1987年被江西省人民政府公布为第三批省级文物保护单位。

上述事实，有经庭审质证、确认的以下证据证实：

（一）物证：

作案工具撬棍、螺丝刀、无柄铲子等照片，证明被告人四人盗挖红塔使用作案工具情况；

（二）书证：

1、报案报告，证明2019年2月18日凌晨0时40分许，浮梁县文化广电新闻出版局工作人员在巡查红塔时发现有人盗挖红塔地宫，嫌疑人被发现后驾车逃窜，遂报警。

2、江西省人民政府赣府发[1987]122号文件及第三批江西省文物保护单位名单，证明红塔为江西省第三批文物保护单位。

3、归案经过及黄嗣通、吴小胡羁押证明、常住人口信息及无犯罪记录证明，证明被告人田少波于2019年5月7日被浮梁县公安局民警抓获，被告人黄嗣通、吴小胡于2019年5月28日主动到浙江省庆元县公安局投案并于当日临时羁押于庆元县看守所，被告人周上飞于2019年5月14日被浮梁县公安局民警抓获；另证明四被告人作案时均已达完全刑事责任年龄，均无犯罪前科的事实。

4、扣押笔录、扣押决定书及清单，证明被告人田少波持有的车牌为赣Ａ×××**绿色猎豹越野汽车被公安机关扣押，暂存放于浮梁县公安局。

（三）证人冯某（浮梁县文物保护管理所长）、叶某（红塔值班员）的证言，证明2019年2月18日凌晨巡逻时，发现红塔被盗挖，在阶梯上遇见其中一名男子，便用手机视频拍下当时情形，后约有三、四名男子驾驶一辆绿色越野车逃跑。红塔一层室内现场堆放了很多砖土，还遗留编织袋、撬棍、铁锹、起子等作案工具，北侧围墙处有两架折叠梯，应该是盗墓人留下的，室内地面被挖了1米多深、横向也被挖了。

（四）被告人供述与辩解

1、田少波的供述与辩解，在侦查机关辩称其到浮梁县是为了开装修店，晚上去红塔是为了打狗，黄嗣通他们拿着梯子和袋子往红塔走，其一直在车上等，听到吵闹声，其认为是他们打狗被发现才过去的，到现场后遇见一男一女时，其赶紧认错，趁男子打电话报警时，其逃离。被告人田少波均未如实供述盗挖红塔的事实，直至2019年1月20日在检察机关审查阶段，才供述其只是跟着黄嗣通、吴小胡、周上飞到了红塔附近，但没有动手参与盗挖。

2、黄嗣通的供述与辩解，证实2019年正月，应田少波的邀请，其与吴小胡乘坐周上飞驾驶的白色别克车到达田少波事前租赁好的民宿，当天及第二天，田少波带领其等去看了红塔，并说要挖红塔地下的东西卖钱，大家均表示同意，因之后几天一直下雨没动手，正月初十晚12时许，大家乘坐田少波驾驶绿色越野猎豹车带着工具去红塔，因墙太高无法进入便撤回，2月15日，田少波在浮梁县购买了两架梯子，当晚，田少波再次驾驶车辆带领大家到了红塔，周上飞在车上望风，其与田少波、吴小胡利用梯子从红塔后面进入内部，田少波首先开挖，之后，其与吴小胡按照田少波的安排挖土，之后都是如此，18日凌晨，其听到有人开门，便与吴小胡溜出塔外，田少波则去和来人说话，随后听田少波说跑，大家就跑上车回到住宿，收拾东西离开了浮梁县。

此次盗挖红塔是由田少波组织的，作案工具也是田少波事先准备好的。

3、吴小胡的供述与辩解，证实2019年2月，其与黄嗣通、周上飞一起到了江西省浮梁县田少波之前租好的民宿。之后，田少波带着大家到了古县衙玩，田少波说弄些红塔里面的砖卖，他有销售渠道，当晚，田少波开车带领大家去红塔，周上飞在车上望风，其他人到了红塔外面，因围墙太高进不去就回来了，第二天，田少波弄了两个人字梯，晚上再次去了红塔，还是周上飞望风，其余三人爬梯进入塔内，田少波就用起子撬砖块，第二天还是如此，田少波走出塔外，其进去搬砖时，听到开门声音及看见手电筒光亮，便躲到梯子边上，一会儿，田少波及黄嗣通跑来说赶紧跑，三人就翻出墙外上车逃离。此次盗挖，是田少波提议并组织的，工具也是他准备的。

4、周上飞的供述与辩解，证实2019年正月初七、八左右，其开车送吴小胡、“长嘴巴”去浮梁县，到达浮梁县时，田少波已经在那等，并安排其三人住进一家民宿，第二天，田少波带领大家去红塔转了转，第三天，田少波和“长嘴巴”买了楼梯、蛇皮袋、手套、手电等工具，田少波说晚上开始盗挖红塔，当晚11时许，田少波开着他的绿色越野车带着大家去了红塔附近，让其在车上望风，他们三人则带着工具去挖红塔，一个多小时后，就回来了，如此挖了三晚，第三天晚上因被发现，大家就离开了浮梁县。

（五）现场勘验笔录及照片，辨认、指认笔录及照片，证明红塔的案发现场及各被告人之间的辨认情况。

（六）现场视频，证明红塔工作人员巡逻时拍摄发现田少波在现场的情况。

二、被告人田少波、吴某、许某、杨某盗挖西林寺塔的事实

2019 年 4 月中旬，被告人田少波与杨某一起来到九江市濂溪区西林寺，田少波提出西林寺塔下面可能有文物，可以盗挖，因人手不够，二被告人分别联系了被告人吴某、许某。2019 年 4 月 19 日，被告人吴某、许某到达九江市与杨某会合。后被告人田少波伙同吴某、许某、杨某（均另处）到西林寺踩点，并租住在西林寺附近的一民房。2019 年 4 月下旬至 5 月初，四被告人多次趁夜钻入西林寺塔，使用铁锹、螺丝刀、撬棍对西林寺塔内楼梯下的墙壁挖洞，由洞底向塔中心延伸，并利用刮白的木板、佛像对盗洞进行遮掩。其中，被告人吴某、许某负责挖土、被告人田少波负责运土、被告人杨某负责望风。经查，被盗挖西林寺，于 1959 年被江西省人民政府公布为第二批省级文物保护单位。

上述事实，有经庭审质证、确认的以下证据证实：

（一）书证：江西省人民政府赣府发[1987]122 号文件、江西省九江市濂溪区人民法院（2019）赣 0402 刑初 240 号刑事判决书，证明西林寺塔位于九江市濂溪区西林寺内，1959 年被江西省人民政府公布为第二批省级文物保护单位，及其他同案犯因该案被判刑的情况。

（二）证人证言

1、吴某的陈述，证实2019年4月中旬，其与许某应田少波和杨某邀请一起到九江，杨某安排住宿并说田少波回去开车了，大家作案的目标是西林寺，第二天，三人去西林寺走了一圈，第三天晚上，田少波回到九江，第四天，田少波带领大家又去西林寺、东林寺转了转，最后，田少波确定以西林寺为目标，并在西林寺附近租了民宿，接下来几天都去踩点，4月底正式盗挖，挖了十天左右被抓获。此次作案是田少波、杨某组织的，车辆是田少波的绿色越野车，日常开销也是田少波先垫付，目标是挖西林寺的地宫，其与许某负责挖土，田少波负责运土，杨某负责望风。

2、许某的陈述，证实其与吴某是4月19日应杨某电话到达九江市的，与杨某见面后，杨某说田少波会开车来接大家去做事的地方，第四天，田少波开着一辆绿色越野车带着大家到东林寺、西林寺转了转，然后在西林寺附近租了一处民房，几天后，就去挖西林寺塔，挖的过程中，吴某负责挖，其负责传递工具、田少波负责运土，杨某负责望风，中途，其与吴某换过作业。此次盗挖西林寺是按照田少波、杨某的安排，所有盗挖工具是田少波从濂溪区一五金店购买的，一般是晚上11点左右去，一直到凌晨2、3点回住处。

3、杨某的陈述，证实2019年4月份，田少波说九江有个西林寺塔，塔里应该有文物，二人到达九江后分别去东林寺、西林寺转了转，后田少波说西林寺没有摄像头，可以在西林寺挖，因

人手不够，就电话叫了许某、吴某一起参与。4月19日，许某、吴某到达九江会面，之后，田少波在西林寺附近租了民房，几天后，就开始挖西林寺塔，因其腿脚不方便就负责望风，吴某、许某负责挖土，田少波负责运土，挖了十余天。此次盗挖是田少波组织的，日常开支和住宿也是田少波负责。

（三）被害人陈述

傅笑龄（西林寺当家师父）的陈述，证证实西林寺塔被盗挖，进塔一楼右边楼梯下墙壁洞口近似圆形，里面往下直径1米多，深达2米多，洞口外表还刷了层仿瓷掩饰。西林寺塔于1959年11月30日被公布为江西省文物保护单位。

（四）被告人田少波的供述与辩解，在公安机关被告人田少波辩解其到九江是寻找养龙虾的店面，杨某等人去西林寺干什么其不清楚，其也去过几次，但其的目的是为了抓穿山甲。在审查起诉阶段供认，其去九江是为了埋灰业务，其他二人是杨某叫去的，他们打算挖西林寺塔地宫时，其还告诉他们该塔已经被盗挖过，没东西可挖，但抱着捡漏心态没有阻止，其去了一天，但没有动手挖过。

（五）现场勘验、现场指认及照片，证明案发现场的概况。

三、另查明，案发后，浮梁县文物管理所聘请了相关专业人员对被损坏红塔进行了抢救性修复，产生聘请专家评估费用人民币2000元、基础整修回填费用6122.69元，共计人民币8122.69

元。公益诉讼机关于2019年12月5日刊登公告，督促相关主体提起诉讼，公告期满后没有适格主体提起公益诉讼。

上述事实，有附带民事公益诉讼机关当庭举证，并经法庭质证、确认的下列证据予以证明：

1、线索移送函、立案决定书、公告，证明本案为检察机关在履职中发现并已履行诉前公告程序，检察机关作为刑事附带民事公益诉讼起诉人主体适格，程序合法。

2、维修工程费用清单、结算总价及红塔基础回填费用说明、专家鉴定费、关于红塔被盗挖所需费用报告及发票，证明为修复被盗挖红塔，产生专家鉴定费2000元、基础整修回填费用6122.69元的事实。

本院认为，被告人田少波伙同他人故意损毁二处省级文物保护单位的文物，其行为已构成故意损毁文物罪，依法应予惩处。公诉机关的指控成立。被告人田少波辩解：1、其未提议和组织盗挖红塔及西林寺塔的意见，经查，在案同案犯均供述盗挖红塔及西林寺塔均由被告人田少波提议、组织实施，该辩解与事实不符，不予采纳；2、提出其是抱着捡漏的心态去了红塔及西林寺塔，但均未动手的意见，经查，在盗挖红塔中，被告人田少波为作案实施了租房并首先对红塔一层进行开挖等一系列行为，该辩解与事实不符，不予采纳；3、作案工具也非其准备的的意见，经查，同案犯均供述作案工具系由田少波准备，故该意见不予采纳；其辩护人提出：1、被告人田少波并非犯意提起者，且没有

从事挖掘的行为，只是从事清土行为，在共同犯罪中不构成主犯的意见，经查，被告人田少波在两次盗挖犯罪中，事前拉拢、勾结他人，实施犯罪时积极参与、协调他人行动，起组织、实施的关键作用，依法应认定主犯，该意见不予采纳；提出 2、被告人田少波如实供述自己的罪行且认罪认罚，应当从轻处罚的意见，经查，被告人田少波被抓获后，在侦查阶段拒不如实供述自己的犯罪事实，在审查起诉阶段，虽签订认罪认罚具结书，供述自己到了现场，但未能彻底、如实供述自己组织、实施盗挖文物的主要犯罪事实，属于认“罚”不认“事”，依法不应认定坦白，该意见不予采纳；提出 3、本案各被告人属临时起意，主观恶性较小，尚未造成难以挽回的后果的意见，经查，两起犯罪事实中，被告人田少波均事前踩点、安排住宿、准备工具，为共同实施犯罪做预备，而非临时起意，故该意见不予采纳。

被告人黄嗣通、吴小胡伙同他人故意损毁省级文物保护单位的文物，二被告人的行为均已构成故意损毁文物罪，依法应予惩处。公诉机关的指控成立。在共同犯罪中，二被告人积极参与并负责挖土、运土，起主要作用，系主犯；案发后，二被告人能主动投案并如实供述自己罪行，系自首，自愿认罪认罚，依法可予从轻处罚。

被告人周上飞伙同他人故意损毁省级文物保护单位的文物，其行为已构成故意损毁文物罪，依法应予惩处。公诉机关的指控成立。在共同犯罪中，负责望风，起次要作用，属从犯，依法应

从轻处罚，关于其辩护人提出：1、被告人周上飞归案后如实供述自己罪行，属坦白；2、属初犯、偶犯的意见，经查，与事实相符，予以采纳。

被告人田少波、黄嗣通、吴小胡、周上飞的行为，不仅构成故意损毁文物罪，而且违反了文物保护法等相关法律法规，损害了社会公共利益，依法应当承担相应的民事责任，公益诉讼机关依法向本院提起的附带民事公益诉讼请求，应当予以支持。

综上，依照《中华人民共和国刑法》第三百二十四条、第四十七条、第二十五条、第二十六条、第二十七条、第六十七条第一、三款、第五十二条、第五十三条、第六十四条，《最高人民法院关于适用（中华人民共和国刑事诉讼法）的解释》第三百六十五条，《中华人民共和国刑事诉讼法》第一百零一条第二款，《中华人民共和国民事诉讼法》第五十五条，《中华人民共和国文物保护法》第六十五条，《中华人民共和国侵权责任法》第四条、第八条、第十五条，《最高人民法院、最高人民检察院关于检察公益诉讼案件适用法律若干问题的解释》第四条、第二十条之规定，判决如下：

一、被告人田少波犯故意损毁文物罪，判处有期徒刑二年十个月，并处罚金人民币 5000 元（限本判决生效后一个月内付清）；

（刑期从判决执行之日起计算。判决执行以前先行羁押的，羁押一日折抵刑期一日。即自 2019 年 5 月 7 日起至 2022 年 3 月 6 日止）。

二、被告人黄嗣通犯故意损毁文物罪，判处有期徒刑一年，并处罚金人民币3000元（限本判决生效后一个月内付清）；

（刑期从判决执行之日起计算。判决执行以前先行羁押的，羁押一日折抵刑期一日。即自2019年5月28日起至2020年5月27日止）。

三、被告人吴小胡犯故意损毁文物罪，判处有期徒刑一年，并处罚金人民币3000元（限本判决生效后一个月内付清）；

（刑期从判决执行之日起计算。判决执行以前先行羁押的，羁押一日折抵刑期一日。即自2019年5月28日起至2020年5月27日止）。

四、被告人周上飞犯故意损毁文物罪，判处有期徒刑一年，并处罚金人民币2000元（限本判决生效后一个月内付清）；

（刑期从判决执行之日起计算。判决执行以前先行羁押的，羁押一日折抵刑期一日。即自2019年5月14日起至2020年5月13日止）。

五、涉案被扣押暂存放于浮梁县公安局的被告人田少波供犯罪所用的赣Ａ×××**绿色猎豹越野车予以没收，由扣押机关浮梁县公安局负责处理；

六、责令附带民事公益诉讼被告田少波、黄嗣通、吴小胡、周上飞共同连带缴纳被损红塔基础整修费、聘请专家费共计人民币8122.69元（本判决生效后十日内付清）；

七、责令附带民事公益诉讼被告田少波、黄嗣通、吴小胡、周上飞于本判决生效后三十日内在江西省省级新闻媒体上公开赔礼道歉（登报内容应经本院审核）。

如不服本判决，可在接到判决书的第二日起十日内，通过本院或者直接向江西省景德镇市中级人民法院提出上诉。书面上诉的，应当提交上诉状正本一份，副本五份。

审　判　长　　方　俊
审　判　员　　周　闽
审　判　员　　周　燕
人民陪审员　　孙航宇
人民陪审员　　杨尚才
人民陪审员　　姚永安
人民陪审员　　汪秀清
二〇二〇年四月十四日
书　记　员　　许　颖
