# Supplementary material for: China’s Legal Protection System for Pangolins: Past, Present, and Future
Source: Animals (Basel). 2025 Aug 18;15(16):2422. doi: 10.3390/ani15162422 (PMC12383201; doi:10.3390/ani15162422)
Supplement: Supplementary file 1 [file animals-15-02422-s001.zip › Supplementary Material S4-Full Text of Judgments in Pangolin-Related Public Interest Litigation Cases in China/【39】高胜非法收购、运输、出售珍贵、濒危野生动物、珍贵、濒危野生动物制品一审刑事判决书.pdf]

高胜非法收购、运输、出售珍贵、濒危野生动物、珍贵、濒危野生动物制品一审刑事判决书

广东省深圳市罗湖区人民法院

刑 事 判 决 书

（2019）粤 0303 刑初 855 号

公诉机关深圳市罗湖区人民检察院。

被告人高胜，男，1978 年 8 月 18 日出生，汉族，小学文化，户籍所在地广东省茂名市信宜市，暂住本市罗湖区。因本案，于 2019 年 1 月 9 日被刑事拘留；因本案，于同年 1 月 22 日被逮捕。现羁押于深圳市罗湖区看守所。

辩护人黄恳儒，广东鹏泰律师事务所律师。

深圳市罗湖区人民检察院以深罗检刑诉〔2019〕942 号起诉书指控被告人高胜犯非法收购濒危野生动物罪，于 2019 年 6 月 24 日向本院提起公诉。本院依法组成合议庭，公开开庭审理了本案。深圳市罗湖区人民检察院指派检察员常建中出庭支持公诉。被告人高胜及辩护人黄恳儒均到庭参加诉讼。本案现已审理终结。

深圳市罗湖区人民检察院指控：

2019 年 1 月 9 日早上 7 时许，被告人高胜驾驶车牌号为粤Ｂ×××**的丰田牌花冠小轿车到本市龙岗区丹平快速路白泥坑出口 200 米处路边位置向广西人（情况不详）购买 10 只穿山甲，而后返回住处。同日，公安机关接到举报后将被告人高胜抓

获，并在本市罗湖区洪湖东路洪湖二街 44 号大院 1 栋一单元 302 房内查获被告人高胜所购买的穿山甲活体 10 只。经华南野生动物物种鉴定中心鉴定：查获的穿山甲活体 10 只为哺乳纲鳞甲目穿山甲科穿山甲属马来穿山甲。

为证明上述事实，公诉机关当庭出示或宣读了下列证据：

一、物证：活体穿山甲 10 只。

二、书证

（一）被告人高胜的人员基本信息等。

（二）扣押决定书、扣押清单等，证实涉案扣押被告人高胜银色丰田牌花冠（车牌号：粤 B×××**）小型轿车 1 辆、红白色条纹封面 office 字样账本 1 本、黑色 PHILIPS 牌手机 1 部、金色苹果手机 1 部、疑似穿山甲活体 10 只。

三、证人证言

森林分局刑警大队出具的抓获经过，证实于 2019 年 1 月 9 日将被告人高胜抓获归案，并在本市罗湖区洪湖东路洪湖二街 44 号大院 1 栋一单元 302 房内查获被告人高胜购买的穿山甲活体 10 只。

四、被告人供述

被告人高胜的供述：2019 年 1 月 9 日上午 10 点多，我刚好出门去买菜，下楼梯时被民警抓获，民警在我的见证下在家里缴获我向他人购买的穿山甲活体 10 只。这些穿山甲是我在今天（1 月 9 日）上午 8 点半左右在龙岗区丹平快速沙湾出口处的辅道边

找一伙广西人买的，广西人的情况我不是很清楚。今天早上七点多，有一个广西人打电话给我说有穿山甲到货问我要不要，我刚好这段时间没有工作就想着试一下买一点来卖，当时就约好交易地点和交易时间。我开着自己的粤B×××**小轿车按照约定到达地点后，对方从他们的车后尾箱拿了三袋穿山甲（黑色塑料袋，其中两袋各3只、一袋4只，每只穿山甲都用白色网袋装好）放到我的后尾箱。放完后告诉我总共10只穿山甲，每斤320元，等我拿回去卖掉后下次再买货时再给这次的货款。我可以通过相片辨认出这些广西人。在我的出租屋里搜出的账本记录的是我在东门市场、罗某市场买海鲜欠别人的钱。我知道买卖穿山甲是违法的，但不知道这么严重。

五、鉴定意见

华南野生动物物种鉴定中心动鉴字[2019]第12号鉴定报告，证实查获的穿山甲活体10只是哺乳纲鳞甲目穿山甲科穿山甲属马来穿山甲，是濒危野生动物。

六、勘验、检查、辨认、侦查实验等笔录：勘验笔录。

七、视听资料、电子数据：光盘3张。

公诉机关认为，被告人高胜非法收购国家二级保护动物穿山甲活体10只，情节严重，其行为触犯了《中华人民共和国刑法》第三百四十一条第一款，犯罪事实清楚，证据确实、充分，应当以非法收购濒危野生动物罪追究其刑事责任。被告人高胜在审查

起诉阶段已签署《认罪认罚具结书》，可以依法从宽处理。据此，公诉机关诉请本院依法判处。

被告人高胜在法庭上对指控的犯罪事实及上述证据无异议，承认控罪，辩称涉案穿山甲是在家中缴获，与扣押在案的丰田车辆无关，而金色苹果手机和账本是其个人物品，不是作案工具，购买穿山甲是用被扣押的黑色 PHILIPS 牌手机来进行联系的。

被告人高胜的辩护人对公诉机关指控的罪名无异议，但认为被告人有如下从轻减轻情节：一、被告人认罪态度好，且签署了认罪认罚具结书，可以从轻处罚。二、被告人有正常职业，其因受外界影响听说收购穿山甲很挣钱，所以才走上了犯罪道路。三、涉案穿山甲活体保存完好，并没有造成穿山甲的损伤，涉案穿山甲也已经移送相关部门，综上，请求法庭对其从轻处罚，建议判处三年以下有期徒刑并适用缓刑。

经审理查明，公诉机关指控被告人高胜犯非法收购野生动物罪的事实客观、真实，证明上述事实的证据来源合法，且经当庭质证，本院予以采信。

另查明，在本案立案后，附带民事公益诉讼起诉人深圳市罗湖区人民检察院向本院提出附带民事公益诉讼。在审理过程中，经本院主持调解，双方当事人自愿达成调解协议。

本院认为，被告人高胜非法收购国家二级保护动物穿山甲活体 10 只，情节严重，其行为已构成非法收购濒危野生动物罪。公诉机关对被告人高胜的指控，事实清楚，证据确实、充分，应

予以支持。被告人高胜归案后能如实供述自己的罪行，是坦白，依法可以从轻处罚。被告人高胜在审查起诉阶段签署《认罪认罚具结书》，依法可以从轻处罚。被告人高胜与附带民事公益诉讼起诉人达成调解，可以从轻处罚。本案中，未有相关证据证实涉案扣押的丰田车辆 1 部、账本 1 本、金色苹果手机 1 部为作案工具，属被告人高胜的个人物品，应予以返还。辩护人提出的从轻辩护意见，予以采纳。辩护人提出的应判处被告人高胜有期徒刑三年并适用缓刑的辩护意见，于法无据，不予采纳。

综上，依照《中华人民共和国刑法》第三百四十一条、第六十七条第三款、第六十四条、第五十三条之规定，判决如下：

一、被告人高胜犯非法收购濒危野生动物罪，判处有期徒刑五年，并处罚金人民币 10000 元（刑期自判决执行之日起计算。判决执行以前先行羁押的，羁押一日折抵刑期一日；即自 2019 年 1 月 9 日起至 2024 年 1 月 8 日止）。

二、查获在案的穿山甲活体 10 只已移交深圳市野生动物救助中心接收。

三、涉案扣押被告人高胜个人物品丰田车辆 1 部、账本 1 本、金色苹果手机 1 部，由扣押机关予以返还给被告人高胜。

如不服本判决，可于接到判决书的第二日起十日内，通过本院或者直接向广东省深圳市中级人民法院提起上诉。书面上诉的，应当提交上诉状正本一份、副本二份。

审　判　长　　王燕群

人民陪审员　　魏国平

人民陪审员　　陈宏辉

二〇二〇年一月十六日

书　记　员　　吕叶童

法官助理杨柳影

附：相关法律条文

《中华人民共和国刑法》

一、第三百四十一条：非法猎捕、杀害国家重点保护的珍贵、濒危野生动物的，或者非法收购、运输、出售国家重点保护的珍贵、濒危野生动物及其制品的，处五年以下有期徒刑或者拘役，并处罚金；情节严重的，处五年以上十年以下有期徒刑，并处罚金；情节特别严重的，处十年以上有期徒刑，并处罚金或者没收财产。

二、第五十三条：罚金在判决指定的期限内一次或者分期缴纳。期满不缴纳的，强制缴纳。对于不能全部缴纳罚金的，人民法院在任何时候发现被执行人有可以执行的财产，应当随时追缴。

由于遭遇不能抗拒的灾祸等原因缴纳确实有困难的，经人民法院裁定，可以延期缴纳、酌情减少或者免除。

三、第六十四条：犯罪分子违法所得的一切财物，应当予以追缴或者责令退赔；对被害人的合法财产，应当及时返还；违禁

品和供犯罪所用的本人财物，应当予以没收。没收的财物和罚金，一律上缴国库，不得挪用和自行处理。

四、第六十七条：犯罪以后自动投案，如实供述自己的罪行的，是自首。对于自首的犯罪分子，可以从轻或者减轻处罚。其中，犯罪较轻的，可以免除处罚。

被采取强制措施的犯罪嫌疑人、被告人和正在服刑的罪犯，如实供述司法机关还未掌握的本人其他罪行的，以自首论。

犯罪嫌疑人虽不具有前两款规定的自首情节，但是如实供述自己罪行的，可以从轻处罚；因其如实供述自己罪行，避免特别严重后果发生的，可以减轻处罚。
