# Supplementary material for: China’s Legal Protection System for Pangolins: Past, Present, and Future
Source: Animals (Basel). 2025 Aug 18;15(16):2422. doi: 10.3390/ani15162422 (PMC12383201; doi:10.3390/ani15162422)
Supplement: Supplementary file 1 [file animals-15-02422-s001.zip › Supplementary Material S4-Full Text of Judgments in Pangolin-Related Public Interest Litigation Cases in China/【3】董某庆危害珍贵濒危野生动物一审刑事判决书.pdf]

云南省盈江县人民法院
刑 事 附 带 民 事 判 决 书

（2023）云 3123 刑初 516 号

公诉机关暨刑事附带民事公益诉讼起诉人云南省盈江县人民检察院。

被告人董某庆，曾用名勒某，男，1991 年 10 月 1 日生，景颇族，初中文化，务农，户籍地云南省瑞丽市。因涉嫌犯危害珍贵、濒危野生动物罪，于 2023 年 9 月 12 日被盈江县公安局刑事拘留，2023 年 9 月 26 日经盈江县人民检察院批准被逮捕。2023 年 12 月 15 日经本院批准变更强制措施为取保候审。

辩护人刘静涛，重庆渝和律师事务所律师。

盈江县人民检察院以盈检刑诉〔2023〕487 号起诉书指控被告人董某庆犯危害珍贵、濒危野生动物罪，于 2023 年 11 月 23 日向本院提起公诉；公益诉讼起诉人盈江县人民检察院以盈检刑附民公诉〔2023〕2 号刑事附带民事公益诉讼起诉书，于 2023 年 12 月 5 日向本院提起刑事附带民事公益诉讼。本院受理后，依法组成合议庭，适用普通程序，于 2023 年 12 月 15 日合并公开开庭进行了审理。公诉机关暨公益诉讼起诉人盈江县人民检察院指派检察员周洪邦出庭履行职务，被告人董某庆及辩护人刘静涛到庭参加诉讼。本案现已审理终结。

盈江县人民检察院指控：被告人董某庆在没有合法手续，明知收购、运输穿山甲鳞片违法的情况下，于 2023 年 9 月 7 日以人民币 11400 元的价格和境外一名叫“啊德”的缅甸人购买了 7 公斤穿山甲鳞片。2023 年 9 月 11 日被告人董某庆驾驶车牌号码为云Ｎ8****的黑色大众牌轿车，途经瑞丽市、陇川县、盈江县、梁河县准备将购买的穿山甲鳞片拉运至腾冲市，在途经梁河县**民警查获。经对涉案的疑似穿山甲鳞片进行称量，

共计重 6951 克。经德宏傣族景颇族自治州公安司法鉴定中心对涉案的野生动物甲片进行鉴定，动物甲片疑似物为穿山甲鳞片，来源于鳞甲目穿山甲科马来穿山甲Ｍａｎｉｓｊａｖａｎｉｃａ；马来穿山甲为国家Ｉ级保护野生动物，列入《濒危野生动植物种国际贸易公约》（ＣＩＴＥＳ）附录Ｉ；涉案穿山甲鳞片价值为人民币 1233984 元。

针对指控，公诉机关当庭宣读和出示了物证、书证、证人证言、辨认笔录、鉴定意见、被告人的供述与辩解等证据。公诉机关认为，被告人董某庆违反国家野生动物保护法规，非法收购、运输国家重点保护的珍贵、濒危野生动物制品情节严重的行为触犯了《中华人民共和国刑法》第三百四十一条第一款之规定，应当以危害珍贵、濒危野生动物罪追究其刑事责任。被告人董某庆认罪认罚，依照《中华人民共和国刑事诉讼法》第十五条之规定，可以从宽处罚。建议判处被告人董某庆有期徒刑三年，适用缓刑，并处罚金。扣押的野生动物制品穿山甲鳞片 6951 克系违禁品，建议依法予以没收；扣押的被告人持用的云Ｎ8****大众汽车一辆、石某保驾驶证一本、ＯＰＰＯ牌黑色手机一部、董某庆驾驶证一本、收纳箱一个，与本案无关，建议依法予以返还。

公益诉讼起诉人提出如下诉讼请求：一、依法判令董某庆赔偿非法收购、运输国家重点保护的珍贵、濒危野生动物制品造成的生态资源损失人民币 1233984 元。二、依法判令董某庆就非法收购、出售野生动物的行为导致国家利益和社会公共利益受到损害的行为向社会公众赔礼道歉。事实和理由如下：事实同刑事指控内容。公益诉讼起诉人认为，根据《中华人民共和国民法典》第一百八十七条之规定“民事主体因同一行为应当承担民事责任、行政责任和刑事责任的，承担行政责任或者

刑事责任不影响承担民事责任。”第一千二百三十五条之规定：“违反国家规定造成生态环境损害的，国家规定的机关或者法律规定的组织有权请求侵权人赔偿下列损失和费用：（二）生态环境功能永久性损害造成的损失；（三）生态环境损害调查、鉴定评估等费用。”以及《中华人民共和国野生动物保护法》第三条第一款：“野生动物资源属于国家所有。”以及《最高人民法院关于审理环境民事公益诉讼案件适用法律若干问题的解释》第十八条“对污染环境、破坏生态，已经损害社会公共利益或者具有损害社会公共利益重大风险的行为，原告可以请求被告承担停止侵害、排除妨碍、消除危险、恢复原状、赔偿损失、赔礼道歉等民事责任。”等相关法律规定，野生动物是全社会、全人类宝贵的自然资源，保护野生动物对维护生态平衡有重要意义，董某庆的行为导致该野生动物自然资源损失人民币 1233984 元、破坏了生态平衡，侵害了国家利益和社会公共利益，本案被告人董某庆既应对危害珍贵、濒危野生动物的行为承担刑事责任，也应承担侵害国家和社会公共利益的民事赔偿责任。经依法公告，没有其他机关或有关组织提起诉讼，根据《中华人民共和国民事诉讼法》第五十八条第二款，《最高人民法院、最高人民检察院关于检察公益诉讼案件适用法律若干问题的解释》第二十条的规定，盈江县人民检察院以公益诉讼起诉人的身份依法履行检察机关的职权提起诉讼，为追究侵权者的民事责任，保护生态环境、野生动物自然资源，维护国家和社会公共利益，特向人民法院提起附带民事公益诉讼，请依法裁判。公益诉讼起诉人向本院提交公告一份，证实盈江县人民检察院于 2023 年 10 月 27 日对本案公益诉讼发出公告，没有其他机关或有关组织提起诉讼。以上证据经质证被告人董某庆无异议，本院予以采信。

被告人董某庆对公诉机关指控的事实、罪名及量刑建议没有异议且签字具结，在开庭审理过程中亦无异议。对公益诉讼起诉人提出的诉讼请求董某庆均无异议，被告人董某庆提出如下履行方案：针对第一项诉请因数额较大且家庭困难，2023 年 12 月 15 日先赔偿 100000 元，剩余 1133984 元，从 2025 年 1 月 1 日开始，每年支付不低于 20000 元，在 2050 年 12 月 31 日前全部支付完；针对第二项诉请，于判决生效后在德宏州团结报上登报道歉的方式，进行一次向社会公众赔礼道歉。

辩护人刘静涛对公诉机关指控的事实及罪名无异议，认为被告人董某庆具有以下法定、酌定从轻、减轻情节：1.被告人董某庆未造成动物死亡，且涉案穿山甲甲片全部追回；2.被告人董某庆在接受公安机关一般盘查时就已经主动承认了携带的是穿山甲甲片，符合自首的规定，可以对董某庆从轻处罚；如不能构成自首，董某庆到案后如实供述全部犯罪事实，构成坦白，可以从轻处罚；3.被告人董某庆自愿认罪认罚并签署具结书，依法可以从宽处理；4.被告人董某庆自愿缴纳公益赔偿金，其和家属就赔偿损失的支付与公益诉讼起诉人达成部分和解，家属已先期支付赔偿金人民币 100000 元，说明其确有悔罪表现，可酌情从轻处罚；5.被告人董某庆系瑞丽市某某养殖专业合作社的负责人，是脱贫致富的带头人，曾获得瑞丽市、德宏州“劳动模范”荣誉称号，对其从轻、减轻处罚，适用缓刑有利于脱贫攻坚工作的开展；6.扣押在案的云Ｎ8****系石某保名下车辆，车款还在分期付款中，希望能够返还。综上，希望法庭能够对其减轻或者从轻处罚。

经审理查明：被告人董某庆在没有合法手续，明知收购、运输穿山甲鳞片违法的情况下，于 2023 年 9 月 7 日以人民币 11400 元的价格和境外一名叫“啊德”的缅甸人购买了 7 公斤

穿山甲鳞片。2023 年 9 月 11 日被告人董某庆驾驶车牌号码为云Ｎ8****的黑色大众牌轿车，途经瑞丽市、陇川县、盈江县、梁河县准备将购买的穿山甲鳞片拉运至腾冲市，在途经梁河县**民警查获。经对涉案的疑似穿山甲鳞片进行称量，共计重 6951 克。经德宏州公安司法鉴定中心对涉案的野生动物甲片进行鉴定，动物甲片疑似物为穿山甲鳞片，来源于鳞甲目穿山甲科马来穿山甲Ｍａｎｉｓｊａｖａｎｉｃａ；马来穿山甲为国家Ｉ级保护野生动物，列入《濒危野生动植物种国际贸易公约》（ＣＩＴＥＳ）附录Ｉ；涉案穿山甲鳞片价值为人民币 1233984 元。

另查明：本案扣押被告人董某庆持用的云Ｎ8****大众汽车一辆、穿山甲鳞片 6951 克、石某保驾驶证一本，黑色ＯＰＰＯ手机一部，董某庆驾驶证一本，收纳箱一个。被告人董某庆的家属于 2023 年 12 月 15 日已代为支付生态资源损失赔偿金人民币 100000 元。盈江县人民检察院于 2023 年 10 月 27 日通过正义网发出公告，无法律规定的机关和有关组织提起诉讼，故盈江县人民检察院代为提起附带民事公益诉讼。

上述事实，有经庭审质证、确认的受案登记表、立案决定书、拘留证、逮捕证及通知书、移送审查起诉告知书，案件移交登记表，被告人的正侧面照片、户口证明，称量笔录及照片，扣押决定书、扣押笔录、扣押清单、发还清单，证明材料一份，接受证据清单，随案移送清单，行驶轨迹核查情况报告，情况说明，查获经过，到案经过，证人周某川、石某保、勒堵、李某伟的证言，被告人董某庆的供述与辩解，鉴定意见及通知，辨认笔录，电子物证检查工作记录，现场检测报告书，公告等证据证实，足以认定。

本院认为，被告人董某庆违反国家野生动物保护法规，非法收购、运输国家重点保护的珍贵、濒危野生动物制品的行为，已触犯刑律，构成危害珍贵、濒危野生动物罪，根据《中华人民共和国刑法》第三百四十一条第一款之规定，应处五年以下有期徒刑或者拘役，并处罚金。被告人董某庆到案后能如实供述自己的罪行，系坦白，依法可从轻处罚；被告人董某庆自愿认罪认罚并签署具结书，依法可对其从宽处理；被告人董某庆及其家属积极支付了生态资源损失 100000 元，并就剩余款项的支付提出可行的履行方案，确有悔罪表现，可酌情从轻处罚。综上，本院决定对其从轻处罚。结合被告人董某庆的犯罪事实、情节、认罪悔罪表现和对社会的危害程度，对其适用缓刑不致再危害社会，本院决定对其适用缓刑。公诉机关指控的事实及罪名成立，量刑建议适当，本院予以支持。被告人董某庆提出从轻处罚的辩解和辩护人提出被告人董某庆有如实供述自己的罪行、认罪认罚等情节应当从轻或者减轻处罚并适用缓刑的辩护意见，本院根据实际情况予以部分采纳。扣押在案的穿山甲鳞片 6951 克系违禁品，依法予以没收；扣押的其他物品无证据证明与本案有关，由侦查机关依法予以处理。

被告人董某庆违反国家野生动物保护法规，非法收购、运输珍贵、濒危野生动物制品，破坏国家野生动物生态资源，损害社会公共利益，依法应当承担对造成的损失承担民事赔偿责任。本案中，扣减已支付的生态资源损失赔偿金 100000 元，被告人董某庆还应赔偿因其非法收购、运输野生动物制品造成的生态资源损失人民币 1133984 元，并在德宏团结报上刊登道歉信向社会公众赔礼道歉一次。公益诉讼起诉人的诉请于法有据，本院予以支持。据此，本院根据被告人董某庆犯罪的事实、性质、情节和对社会的危害程度，依照《中华人民共和国

刑法》第三百四十一条第一款、第六十七条第三款、第七十二条第一款第（二）项、第七十三条第二款、第五十二条、第五十三条、第六十一条、第六十四条，《最高人民法院、最高人民检察院关于办理破坏野生动物资源刑事案件适用法律若干问题的解释》第六条第一款，《中华人民共和国刑事诉讼法》第十五条、第二百零一条，《中华人民共和国民法典》第一千二百三十五条、《最高人民法院关于审理环境民事公益诉讼案件适用法律若干问题的解释》第十八条之规定，判决如下：

一、被告人董某庆犯危害珍贵、濒危野生动物罪，判处有期徒刑三年，缓刑四年，并处罚金人民币一万元。（缓刑考验期限，从判决确定之日起计算。罚金自本判决生效之日起十日内向本院缴纳。）

二、被告人董某庆在本判决生效之日起三十日内赔偿国家野生动物资源损失人民币 1133984 元，并上缴国家指定账户。

三、被告人董某庆在本判决生效之日起十日内在德宏团结报上刊登一次道歉信向社会公众赔礼道歉。

四、扣押在案的重 6951 克穿山甲鳞片，依法予以没收；其他物品，由侦查机关依法予以处理。

如不服本判决，可在接到判决书的第二日起十日内，通过本院或者直接向云南省德宏傣族景颇族自治州中级人民法院提出上诉。书面上诉的，应提交上诉状正本一份、副本二份。

审　判　长　　唐正勇

审　判　员　　张　姜

人民陪审员　　左麻仁

人民陪审员　　雷兴明

人民陪审员　　线加杏

人民陪审员　　祝加强

人民陪审员　　雷小尚

二〇二三年十二月二十二日

书　记　员　　李思思

附相关法条：

《中华人民共和国刑法》

第三百四十一条【危害珍贵、濒危野生动物罪】非法猎捕、杀害国家重点保护的珍贵、濒危野生动物的，或者非法收购、运输、出售国家重点保护的珍贵、濒危野生动物及其制品的，处五年以下有期徒刑或者拘役，并处罚金；情节严重的，处五年以上十年以下有期徒刑，并处罚金；情节特别严重的，处十年以上有期徒刑，并处罚金或者没收财产。

【非法狩猎罪】违反狩猎法规，在禁猎区、禁猎期或者使用禁用的工具、方法进行狩猎，破坏野生动物资源，情节严重的，处三年以下有期徒刑、拘役、管制或者罚金。

【非法猎捕、收购、运输、出售陆生野生动物罪】违反野生动物保护管理法规，以食用为目的非法猎捕、收购、运输、出售第一款规定以外的在野外环境自然生长繁殖的陆生野生动物，情节严重的，依照前款的规定处罚。

第六十七条【自首和坦白】犯罪以后自动投案，如实供述自己的罪行的，是自首。对于自首的犯罪分子，可以从轻或者减轻处罚。其中，犯罪较轻的，可以免除处罚。

被采取强制措施的犯罪嫌疑人、被告人和正在服刑的罪犯，如实供述司法机关还未掌握的本人其他罪行的，以自首论。

犯罪嫌疑人虽不具有前两款规定的自首情节，但是如实供述自己罪行的，可以从轻处罚；因其如实供述自己罪行，避免特别严重后果发生的，可以减轻处罚。

第七十二条【缓刑的条件】对于被判处拘役、三年以下有期徒刑的犯罪分子，同时符合下列条件的，可以宣告缓刑，对其中不满十八周岁的人、怀孕的妇女和已满七十五周岁的人，应当宣告缓刑：

（一）犯罪情节较轻；

（二）有悔罪表现；

（三）没有再犯罪的危险；

（四）宣告缓刑对所居住社区没有重大不良影响。

宣告缓刑，可以根据犯罪情况，同时禁止犯罪分子在缓刑考验期限内从事特定活动，进入特定区域、场所，接触特定的人。

被宣告缓刑的犯罪分子，如果被判处附加刑，附加刑仍须执行。

第七十三条【缓刑考验期限】拘役的缓刑考验期限为原判刑期以上一年以下，但是不能少于二个月。

有期徒刑的缓刑考验期限为原判刑期以上五年以下，但是不能少于一年。

缓刑考验期限，从判决确定之日起计算。

第五十二条【罚金数额的确定】判处罚金，应当根据犯罪情节决定罚金数额。

第五十三条【罚金的缴纳】罚金在判决指定的期限内一次或者分期缴纳。期满不缴纳的，强制缴纳。对于不能全部缴纳罚金的，人民法院在任何时候发现被执行人有可以执行的财产，应当随时追缴。

第六十一条【量刑的一般原则】对于犯罪分子决定刑罚的时候，应当根据犯罪的事实、犯罪的性质、情节和对于社会的危害程度，依照本法的有关规定判处。

第六十四条【涉案财物的处理】犯罪分子违法所得的一切财物，应当予以追缴或者责令退赔；对被害人的合法财产，应当及时返还；违禁品和供犯罪所用的本人财物，应当予以没收。没收的财物和罚金，一律上缴国库，不得挪用和自行处理。

《最高人民法院最高人民检察院关于办理破坏野生动物资源刑事案件适用法律若干问题的解释》

非法猎捕、杀害国家重点保护的珍贵、濒危野生动物，或者非法收购、运输、出售国家重点保护的珍贵、濒危野生动物及其制品，价值二万元以上不满二十万元的，应当依照刑法第三百四十一条第一款的规定，以危害珍贵、濒危野生动物罪处五年以下有期徒刑或者拘役，并处罚金；价值二十万元以上不满二百万元的，应当认定为“情节严重”，处五年以上十年以下有期徒刑，并处罚金；价值二百万元以上的，应当认定为“情节特别严重”，处十年以上有期徒刑，并处罚金或者没收财产。

实施前款规定的行为，具有下列情形之一的，从重处罚：

（一）属于犯罪集团的首要分子的；

（二）为逃避监管，使用特种交通工具实施的；

（三）严重影响野生动物科研工作的；

（四）二年内曾因破坏野生动物资源受过行政处罚的。

实施第一款规定的行为，不具有第二款规定的情形，且未造成动物死亡或者动物、动物制品无法追回，行为人全部退赃退赔，确有悔罪表现的，按照下列规定处理：

（一）珍贵、濒危野生动物及其制品价值二百万元以上的，可以认定为“情节严重”，处五年以上十年以下有期徒刑，并处罚金；

（二）珍贵、濒危野生动物及其制品价值二十万元以上不满二百万元的，可以处五年以下有期徒刑或者拘役，并处罚金；

（三）珍贵、濒危野生动物及其制品价值二万元以上不满二十万元的，可以认定为犯罪情节轻微，不起诉或者免予刑事处罚；情节显著轻微危害不大的，不作为犯罪处理。

《中华人民共和国刑事诉讼法》

【认罪认罚从宽制度】犯罪嫌疑人、被告人自愿如实供述自己的罪行，承认指控的犯罪事实，愿意接受处罚的，可以依法从宽处理。

第二百零一条【法院对认罪认罚案件量刑建议的处理】对于认罪认罚案件，人民法院依法作出判决时，一般应当采纳人民检察院指控的罪名和量刑建议，但有下列情形的除外：

（一）被告人的行为不构成犯罪或者不应当追究其刑事责任的；

（二）被告人违背意愿认罪认罚的；

（三）被告人否认指控的犯罪事实的；

（四）起诉指控的罪名与审理认定的罪名不一致的；

（五）其他可能影响公正审判的情形。

人民法院经审理认为量刑建议明显不当，或者被告人、辩护人对量刑建议提出异议的，人民检察院可以调整量刑建议。人民检察院不调整量刑建议或者调整量刑建议后仍然明显不当的，人民法院应当依法作出判决。

《中华人民共和国民法典》

第一千二百三十五条【公益诉讼的赔偿范围】违反国家规定造成生态环境损害的，国家规定的机关或者法律规定的组织有权请求侵权人赔偿下列损失和费用：

（一）生态环境受到损害至修复完成期间服务功能丧失导致的损失；

（二）生态环境功能永久性损害造成的损失；

（三）生态环境损害调查、鉴定评估等费用；

（四）清除污染、修复生态环境费用；

（五）防止损害的发生和扩大所支出的合理费用。

《最高人民法院关于审理环境民事公益诉讼案件适用法律若干问题的解释》

第十八条：对污染环境、破坏生态，已经损害社会公共利益或者具有损害社会公共利益重大风险的行为，原告可以请求被告承担停止侵害、排除妨碍、消除危险、修复生态环境、赔偿损失、赔礼道歉等民事责任。
