# Supplementary material for: China’s Legal Protection System for Pangolins: Past, Present, and Future
Source: Animals (Basel). 2025 Aug 18;15(16):2422. doi: 10.3390/ani15162422 (PMC12383201; doi:10.3390/ani15162422)
Supplement: Supplementary file 1 [file animals-15-02422-s001.zip › Supplementary Material S4-Full Text of Judgments in Pangolin-Related Public Interest Litigation Cases in China/【40】龙友会非法收购、运输、出售珍贵、濒危野生动物、珍贵、濒危野生动物制品一审刑事判决书.pdf]

# 龙友会非法收购、运输、出售珍贵、濒危野生动物、珍贵、濒危野生动物制品一审刑事判决书

## 云南省广南县人民法院

## 刑 事 附 带 民 事 判 决 书

（2019）云2627刑初357号

公诉机关暨附带民事公益诉讼人云南省广南县人民检察院。

被告人龙友会，女，1964年4月3日生，汉族，文盲，户籍地址：贵州省安顺市关岭布依族苗族自治县，现住广南县，因本案于2019年9月2日被取保候审。

广南县人民检察院以广检一部刑诉〔2019〕137号起诉书指控被告人龙友会犯非法出售珍贵、濒危野生动物制品罪，于2019年10月22日向本院提起公诉。在本院审理过程中，广南县人民检察院提起刑事附带民事公益诉讼。本院依法组成合议庭，适用普通程序于2019年11月25日、12月19日公开开庭进行了合并审理。广南县人民检察院指派检察员李孟琼、书记员严啟霖出庭支持公诉，检察员陆安粱、书记员杜超到庭参与刑事附带民事公益诉讼，被告人龙友会到庭参加诉讼。现已审理终结。

广南县人民检察院指控，2019年农历三月的一天，被告人龙友会在自己位于小南街的草药摊上以2600元的价格向一个外地男子购买了1.3市斤疑似穿山甲鳞片用于配制中药所用,所收购的野生动物制品无任何合法手续。

2019 年 7 月 13 日,广南县森林公安局民警在龙友会草药摊处搜查出疑似穿山甲鳞片 54 片(637.68 克)。经鉴定,龙友会所持有的疑似穿山甲鳞片为穿山甲鳞片,共计 54 片,重为 637.68 克(折合 1.36 只),保护级别为国家二级,经济价值为 43520 元。

同时提出，被告人龙友会非法出售国家二级保护野生动物制品，破坏生物多样性，危害生态系统平衡，损害了国家和社会公共利益。该院已进行了诉前公告程序，公告期满后没有适格主体提起诉讼，社会公共利益仍处于受损害状态，请求追究被告人龙友会的民事责任，判处其赔偿非法购买国家二级保护动物穿山甲鳞片的经济价值共计人民币 43520 元。

为证明指控事实,广南县人民检察院列举了物证照片;书证;证人证言；被告人供述与辩解；鉴定意见；勘验、检查、辨认笔录等证据在案为证。

广南县人民检察院认为,被告人龙友会违反野生动物保护法的规定非法出售珍贵、濒危野生动物制品，其行为触犯了《中华人民共和国刑法》第三百四十一条之规定，犯罪事实清楚，证据确实、充分，应当以非法出售珍贵野生动物制品罪追究其刑事责任。被告人龙友会由于主观意志以外的原因未能实施完犯罪，属于犯罪未遂。到案后如实供述自己的犯罪行为，应认定坦白。建议以非法出售珍贵、濒危野生动物制品罪判处被告人龙友会八个月至一年有期徒刑，同时适用缓刑，并处罚金 1000 元。同时被告人龙友会应承担相应的民事责任。

被告人龙友会对指控事实、罪名及量刑建议没有异议且签字具结，在开庭审理过程中亦无异议。提出民事赔偿部分其家庭困难，只有能力赔偿一至二千元。

经审理查明，2019年农历三月的一天，被告人龙友会在自己位于小南街的草药摊上以2600元的价格向他人购买了1.3市斤疑似穿山甲鳞片用于配制中药销售,所收购的野生动物制品无任何合法手续。2019年7月13日,广南县森林公安局民警在龙友会草药摊处搜查出疑似穿山甲鳞片54片(637.68克)。经鉴定,龙友会所持有的疑似穿山甲鳞片为穿山甲鳞片,共计54片,重为637.68克(折合1.36只),保护级别为国家二级,经济价值为43520元。

广南县人民检察院于2019年10月14日在《正义网》发布公告，称该院在履行职责中发现龙友会非法收购国家珍贵、濒危野生动物制品，损害了社会公共利益，督促建议法律规定的机关和有权提起诉讼的有关组织在公告发出三十日内依照法律有关规定提起公益诉讼。公告期满，仍无符合法律规定的机关和有关组织提起公益诉讼，广南县人民检察院遂提起刑事附带民事公益诉讼。

上述事实有经庭审质证、确认的物证照片；接处警登记表、受案登记表、立案决定书，户口证明、前科劣迹查询记录，到案经过，搜查证、搜查笔录，扣押决定书、扣押清单，抽样提取笔录，随案移送清单，情况说明，公告；证人李某、陆某、侬天英

的证言；被告人龙友会的供述和辩解；鉴定意见；现场勘验、辨认笔录，现场示意图及照片等证据证实，足以认定。

本院认为，被告人龙友会违反国家法律规定，非法收购珍贵、濒危野生动物制品，其行为已构成非法收购珍贵、濒危野生动物制品罪。被告人龙友会到案后，如实供述自己的罪行，属坦白，认罪认罚，可从轻处罚并适用缓刑。公诉机关的量刑建议与被告人龙友会的罪行相适应，本院予以采纳。附带民事公益诉讼人广南县人民检察院提出要求被告人龙友会承担民事赔偿责任的诉讼请求，符合法律的规定，本院予以支持。但被告人龙友会并非是珍贵、濒危野生动物穿山甲的直接破坏者，广南县人民检察院请求被告人龙友会赔偿所查获的穿山甲鳞片的鉴定价值，并无法律依据，被告人龙友会非法收购穿山甲鳞片的行为对生态环境和社会公共利益所造成的损害价值无法确定，结合本案的客观实际，酌情由被告人龙友会承担1000元的赔偿责任为宜。依照《中华人民共和国刑法》第三百四十一条第一款、第六十七条第三款、第七十二条、第七十三条、第五十二条、第六十四条，《中华人民共和国侵权责任法》第十五条和《最高人民法院、最高人民检察院＜关于检察公益诉讼案件适用法律若干问题的解释＞》第二十条，判决如下：

一、被告人龙友会犯非法收购珍贵、濒危野生动物制品罪，判处有期徒刑九个月，宣告缓刑一年，并处罚金人民币1000元。

（缓刑考验期限，从判决确定之日起计算。罚金限判决生效后10日内缴纳。）

二、对广南县森林公安局扣押的穿山甲鳞片637.68克予以没收，由广南县森林公安局依法处理。

三、由被告人龙友会赔偿附带民事公益诉讼人广南县人民检察院人民币1000元，限判决生效后10日内履行。

四、驳回附带民事公益诉讼人广南县人民检察院的其他附带民事公益诉讼请求。

如不服本判决，可在接到判决书的第二日起十日内，通过本院或者直接向文山壮族苗族自治州中级人民法院提出上诉。书面上诉的，应当提交上诉状正本一份，副本四份。

审　判　长　　余光武

审　判　员　　陆兰春

人民陪审员　　秦瑞云

二〇一九年十二月二十五日

书　记　员　　罗文山
