# Supplementary material for: China’s Legal Protection System for Pangolins: Past, Present, and Future
Source: Animals (Basel). 2025 Aug 18;15(16):2422. doi: 10.3390/ani15162422 (PMC12383201; doi:10.3390/ani15162422)
Supplement: Supplementary file 1 [file animals-15-02422-s001.zip › Supplementary Material S4-Full Text of Judgments in Pangolin-Related Public Interest Litigation Cases in China/【41】候廷新非法收购、运输、出售珍贵、濒危野生动物、珍贵、濒危野生动物制品一审刑事判决书.pdf]

候廷新非法收购、运输、出售珍贵、濒危野生动物、珍贵、濒危野生动物制品一审刑事判决书

云南省广南县人民法院

刑 事 附 带 民 事 判 决 书

（2019）云 2627 刑初 358 号

公诉机关暨附带民事公益诉讼人云南省广南县人民检察院。

被告人候廷新，女，1968 年 9 月 5 日生，苗族，文盲，云南省广南县人，住莲城镇龙井社区龙井，因本案于 2019 年 8 月 16 日被取保候审。

广南县人民检察院以广检一部刑诉〔2019〕138 号起诉书指控被告人候廷新犯非法出售珍贵、濒危野生动物制品罪，于 2019 年 10 月 22 日向本院提起公诉。在本院审理过程中，广南县人民检察院提起刑事附带民事公益诉讼。本院依法组成合议庭，于 2019 年 11 月 25 日、12 月 19 日公开开庭进行了合并审理。广南县人民检察院指派检察员李孟琼、书记员严敞霖出庭支持公诉，检察员陆安粱、书记员杜超到庭参与刑事附带民事公益诉讼，被告人候廷新到庭参加诉讼。现已审理终结。

广南县人民检察院指控，2019 年 3 月，被告人候廷新在广南县汇丰商贸有限公司南街农贸市场内的中草药摊以 5000 元的价格向他人购买穿山甲鳞片欲用于出售。2019 年 7 月 13 日，民警在侯某的草药摊上查获穿山甲鳞片。经鉴定在候廷新草药摊处

查获的野生动物制品为穿山甲鳞片,共2312.9克(折合4.95只),经济价值158400元,属国家二级保护动物。

同时提出，被告人候廷新非法出售国家二级保护野生动物制品，破坏生物多样性，危害生态系统平衡，损害了国家和社会公共利益。该院已进行了诉前公告程序，公告期满后没有适格主体提起诉讼，社会公共利益仍处于受损害状态，请求追究被告人候廷新的民事责任，判处其赔偿非法购买国家二级保护动物穿山甲鳞片的经济价值共计人民币158400元。

为证明指控事实,广南县人民检察院列举了物证照片;书证;证人证言；被告人供述与辩解；鉴定意见；勘验、检查、辨认笔录等证据在案为证。

广南县人民检察院认为,被告人候廷新违反国家野生动物保护法规，非法出售珍贵、濒危野生动物制品，其行为触犯了《中华人民共和国刑法》第三百四十一条之规定，应当以非法出售珍贵、濒危野生动物制品罪追究其刑事责任。被告人侯某因意志意外的原因未能完成出售行为，属犯罪未遂。其在公安机关查获时主动将自己收购的穿山甲鳞片交出,到案后如实供述自己的犯罪行为，应认定自首。其到案后如实供述自己的罪行，依法可从轻处罚，建议对其判处有期徒刑二年六个月至三年，适用缓刑，并处罚金1000元。同时被告人候廷新应承担相应的民事责任。

被告人候廷新对指控事实、罪名及量刑建议没有异议且签字具结，在开庭审理过程中亦无异议。提出民事赔偿部分其无能力赔偿。

经审理查明，2019 年 3 月，被告人候廷新在广南县汇丰商贸有限公司南街农贸市场内的中草药摊以 5000 元的价格向他人购买穿山甲鳞片欲用于出售。2019 年 7 月 13 日，民警在侯某的草药摊上查获穿山甲鳞片。经鉴定在候廷新草药摊处查获的野生动物制品为穿山甲鳞片,共 2312.9 克(折合 4.95 只),经济价值 158400 元,属国家二级保护动物。

广南县人民检察院于 2019 年 10 月 14 日在《正义网》发布公告，称该院在履行职责中发现候廷新非法收购国家珍贵、濒危野生动物制品，损害了社会公共利益，督促建议法律规定的机关和有权提起诉讼的有关组织在公告发出三十日内依照法律有关规定提起公益诉讼。公告期满，仍无符合法律规定的机关和有关组织提起公益诉讼，广南县人民检察院遂提起刑事附带民事公益诉讼。

上述事实有经庭审质证、确认的物证照片；接处警登记表、受案登记表、立案决定书，户口证明、前科劣迹查询记录，到案经过，搜查证、搜查笔录，扣押决定书、扣押清单，抽样提取笔录，随案移送清单，情况说明，公告；证人罗某、陶某的证言；被告人候廷新的供述和辩解；鉴定意见；现场勘验、辨认笔录，现场示意图及照片等证据证实，足以认定。

本院认为，被告人候廷新违反国家法律规定，非法收购珍贵、濒危野生动物制品，情节严重，其行为已构成非法收购珍贵、濒危野生动物制品罪。被告人候廷新在公安机关调查询问时主动将自己收购的穿山甲鳞片交出，如实供述自己的罪行，属自首，认罪认罚，可减轻处罚并适用缓刑。公诉机关的量刑建议与被告人候廷新的罪行相适应，本院予以采纳。附带民事公益诉讼人广南县人民检察院提出要求被告人候廷新承担民事赔偿责任的诉讼请求，符合法律的规定，本院予以支持。但被告人候廷新并非是珍贵、濒危野生动物穿山甲的直接破坏者，广南县人民检察院请求被告人候廷新赔偿所查获的穿山甲鳞片的鉴定价值，并无法律依据，被告人候廷新非法收购穿山甲鳞片的行为对生态环境和社会公共利益所造成的损害价值无法确定，结合本案的客观实际，酌情由被告人候廷新承担 2000 元的赔偿责任为宜。依照《中华人民共和国刑法》第三百四十一条第一款、第六十七条第一款、第七十二条、第七十三条、第五十二条、第六十四条，《中华人民共和国侵权责任法》第十五条和《最高人民法院、最高人民检察院＜关于检察公益诉讼案件适用法律若干问题的解释＞》第二十条，判决如下：

一、被告人候廷新犯非法收购珍贵、濒危野生动物制品罪，判处有期徒刑三年，宣告缓刑四年，并处罚金人民币 1000 元。

（缓刑考验期限，从判决确定之日起计算。罚金限判决生效后 10 日内缴纳。）

二、对广南县森林公安局扣押的穿山甲鳞片2312.9克予以没收，由广南县森林公安局依法处理。

三、由被告人候廷新赔偿附带民事公益诉讼人广南县人民检察院人民币2000元，限判决生效后10日内履行。

四、驳回附带民事公益诉讼人广南县人民检察院的其他附带民事公益诉讼请求。

如不服本判决，可在接到判决书的第二日起十日内，通过本院或者直接向文山壮族苗族自治州中级人民法院提出上诉。书面上诉的，应当提交上诉状正本一份，副本四份。

审　判　长　　余光武

审　判　员　　陆兰春

人民陪审员　　秦瑞云

二〇一九年十二月二十五日

书　记　员　　罗文山
