# Supplementary material for: China’s Legal Protection System for Pangolins: Past, Present, and Future
Source: Animals (Basel). 2025 Aug 18;15(16):2422. doi: 10.3390/ani15162422 (PMC12383201; doi:10.3390/ani15162422)
Supplement: Supplementary file 1 [file animals-15-02422-s001.zip › Supplementary Material S4-Full Text of Judgments in Pangolin-Related Public Interest Litigation Cases in China/【42】秦焕全非法收购、运输、出售珍贵、濒危野生动物、珍贵、濒危野生动物制品一审刑事判决书.pdf]

秦焕全非法收购、运输、出售珍贵、濒危野生动物、珍贵、濒危野生动物制品一审刑事判决书

云南省广南县人民法院

刑 事 附 带 民 事 判 决 书

（2019）云 2627 刑初 361 号

公诉机关暨附带民事公益诉讼人云南省广南县人民检察院。

被告人秦焕全，女，1964 年 6 月 28 日生，汉族，文盲，云南省广南县人，住广南县，曾因犯拐卖儿童罪于 1996 年 11 月 14 日被本院判处有期徒刑九年，于 2002 年 11 月 13 日刑满释放，现因本案于 2019 年 8 月 12 日被取保候审。

广南县人民检察院以广检一部刑诉〔2019〕140 号起诉书指控被告人秦焕全犯非法收购珍贵、濒危野生动物制品罪，于 2019 年 10 月 22 日向本院提起公诉。在本院审理过程中，广南县人民检察院于 2019 年 11 月 23 日向本院提起刑事附带民事公益诉讼。本院依法组成合议庭，于 2019 年 12 月 19 日公开开庭进行了合并审理。广南县人民检察院指派检察员李孟琼、书记员严啟霖出庭支持公诉，检察员陆安梁、书记员杜超到庭参与刑事附带民事公益诉讼，被告人秦焕全到庭参加诉讼。现已审理终结。

广南县人民检察院指控，2018 年年底，被告人秦焕全在广南县综合农贸市场内秦焕全的摊位上以 1400 元的价格向他人购买穿山甲鳞片 1 市斤多。秦焕全将收购的穿山甲鳞片放在自己的中草药摊待售。2019 年 7 月 13 日，民警在秦焕全的草药摊位上

发现以上穿山甲鳞片。经鉴定，涉案的穿山甲鳞片共计 574.75 克（折合 1.23 只），系国家重点保护的二级野生动物，经济价值为 39360 元（叁万玖仟叁佰陆拾元整）。

同时提出，被告人秦焕全非法收购国家二级保护野生动物制品，破坏生物多样性，危害生态系统平衡，损害了国家和社会公共利益。该院已进行了诉前公告程序，公告期满后没有适格主体提起诉讼，社会公共利益仍处于受损害状态，请求追究被告人秦焕全的民事责任，判处其赔偿非法购买国家二级保护动物穿山甲鳞片的经济价值共计人民币 39360 元。

为证明指控事实，广南县人民检察院列举了物证照片；书证；证人证言；被告人供述与辩解；鉴定意见；勘验、检查、辨认笔录等证据在案为证。

广南县人民检察院认为，被告人秦焕全违反国家野生动物保护法规，非法收购珍贵、濒危野生动物制品，其行为触犯了《中华人民共和国刑法》第三百四十一条之规定，应当以非法收购珍贵、濒危野生动物制品罪追究其刑事责任，其到案后如实供述自己的罪行，依法可从轻处罚，建议对其判处有期徒刑八个月至一年，适用缓刑，并处罚金 1000 元。同时被告人秦焕全应承担相应的民事责任。

被告人秦焕全对指控事实、罪名及量刑建议没有异议且签字具结，在开庭审理过程中亦无异议。提出民事赔偿部分要求过高，其仅能赔偿人民币 1000 元。

经审理查明，2018年年底，被告人秦焕全在广南县综合农贸市场其经营的中草药摊处向他人购买穿山甲鳞片若干，2019年7月13日广南县森林公安局在秦焕全的中草药摊上查获穿山甲鳞片574.75克。经鉴定，被查获的穿山甲鳞片折合1.23只穿山甲，穿山甲属国家II级保护动物，被列入《濒危野生动植物种国际贸易公约》附录I，其经济价值为人民币39360元。

广南县人民检察院于2019年10月14日在《正义网》发布公告，称该院在履行职责中发现秦焕全非法收购国家珍贵、濒危野生动物制品，损害了社会公共利益，督促建议法律规定的机关和有权提起诉讼的有关组织在公告发出三十日内依照法律有关规定提起公益诉讼。公告期满，仍无符合法律规定的机关和有关组织提起公益诉讼，广南县人民检察院遂提起刑事附带民事公益诉讼。

上述事实有经庭审质证、确认的物证照片；接处警登记表、受案登记表、立案决定书，户口证明、前科查询记录、网上在逃人员查询工作记录、刑满释放人员通知书，抓获经过，扣押决定书、扣押笔录、扣押清单，发还清单，抽样提取笔录，公告；证人陶某1、吴某1、吴某2、陶某2的证言；被告人秦焕全的供述和辩解；鉴定意见；现场勘验、辨认笔录，现场示意图及照片等证据证实，足以认定。

本院认为，被告人秦焕全违反国家法律规定，非法收购珍贵、濒危野生动物制品，其行为已构成非法收购珍贵、濒危野生动物

制品罪。公诉机关指控的罪名成立，本院予以确认。被告人秦焕全到案后如实供述自己的罪行，属坦白，认罪认罚，可从轻处罚并适用缓刑。公诉机关的量刑建议与被告人秦焕全的罪行相适应，本院予以采纳。附带民事公益诉讼人广南县人民检察院提出要求被告人秦焕全承担民事赔偿责任的诉讼请求，符合法律的规定，本院予以支持。但被告人秦焕全并非是珍贵、濒危野生动物穿山甲的直接破坏者，广南县人民检察院请求被告人秦焕全赔偿所查获的穿山甲鳞片的鉴定价值，并无法律依据，被告人秦焕全非法收购穿山甲鳞片的行为对生态环境和社会公共利益所造成的损害价值无法确定，结合本案的客观实际，酌情由被告人秦焕全承担 1000 元的赔偿责任为宜。依照《中华人民共和国刑法》第三百四十一条第一款、第六十七条第三款、第七十二条、第七十三条、第五十二条、第六十四条，《中华人民共和国侵权责任法》第十五条和《最高人民法院、最高人民检察院＜关于检察公益诉讼案件适用法律若干问题的解释＞》第二十条，判决如下：

一、被告人秦焕全犯非法收购珍贵、濒危野生动物制品罪，判处有期徒刑八个月，宣告缓刑一年，并处罚金人民币 1000 元。

（缓刑考验期限，从判决确定之日起计算。罚金限判决生效后 10 日内缴纳。）

二、对广南县森林公安局扣押的穿山甲鳞片 574.75 克予以没收，由广南县森林公安局依法处理。

三、由被告人秦焕全赔偿附带民事公益诉讼人广南县人民检察院人民币 1000 元，限判决生效后 10 日内履行。

四、驳回附带民事公益诉讼人广南县人民检察院的其他附带民事公益诉讼请求。

如不服本判决，可在接到判决书的第二日起十日内，通过本院或者直接向文山壮族苗族自治州中级人民法院提出上诉。书面上诉的，应当提交上诉状正本一份，副本四份。

审　判　长　　陆兰春

审　判　员　　余光武

人民陪审员　　秦瑞云

二〇一九年十二月二十四日

书记　员代　　凤　兴
