# Supplementary material for: China’s Legal Protection System for Pangolins: Past, Present, and Future
Source: Animals (Basel). 2025 Aug 18;15(16):2422. doi: 10.3390/ani15162422 (PMC12383201; doi:10.3390/ani15162422)
Supplement: Supplementary file 1 [file animals-15-02422-s001.zip › Supplementary Material S4-Full Text of Judgments in Pangolin-Related Public Interest Litigation Cases in China/【44】王海波非法收购、运输、出售珍贵、濒危野生动物、珍贵、濒危野生动物制品一审刑事判决书.pdf]

王海波非法收购、运输、出售珍贵、濒危野生动物、珍贵、濒危野生动物制品一审刑事判决书

贵州省荔波县人民法院

刑 事 附 带 民 事 判 决 书

（2019）黔2722刑初232号

公诉机关暨刑事附带民事公益诉讼原告人贵州省独山县人民检察院。

被告人王海波，男，1965年10月10日出生，汉族，初中文化，户籍所在地广西壮族自治区博白县，家住广西博白县，现住广西河池市。因涉嫌非法运输珍贵、濒危野生动物罪，于2019年5月25日被刑事拘留，同年7月1日被逮捕。现羁押于独山县看守所。

指定辩护人唐剑梅，贵州行者（荔波）律师事务所律师。

贵州省独山县人民检察院以独检公诉刑诉[2019]484号起诉书指控被告人王海波犯非法运输珍贵、濒危野生动物罪，2019年11月29日向本院提起公诉。同时，以独检民公[2019]52272600006号刑事附带民事公益诉讼起诉书向本院提起刑事附带民事公益诉讼。本院受理后，依法组成合议庭，适用普通程序，公开开庭进行了合并审理。独山县人民检察院指派检察员彭琳出庭支持公诉并就附带民事公益诉讼部分发表意见，被告人王海波及辩护人唐剑梅到庭参加诉讼。现已审理终结。

公诉机关指控，2019年5月21日被告人王海波将其向他人购买的2只“穿山甲”活体，用纸箱包装后，以100元的运费委托广西钦州开往四川成都的客车驾驶员杨某、龚某运往重庆陈家坪车站，途径兰海高速公路新寨服务区时，被贵州独山毒品检查站民警现场查获。经华南动物物种环境损害司法鉴定中心鉴定，查获的2只“穿山甲”物种均为哺乳纲鳞甲目穿山甲科，属马来穿山甲，属于国家二级保护动物，涉案总价值为8万元。被告人王海波归案后，如实供述了委托他人运输穿山甲的犯罪事实。

为证实上述事实，公诉机关当庭出示了物证、书证、证人证言、被告人的供述与辩解、司法鉴定意见书、勘验指认辨认笔录及照片、视听资料等证据。公诉机关认为，被告人的行为已触犯《中华人民共和国刑法》第三百四十一条之规定，应以非法运输珍贵、濒危野生动物罪追究刑事责任。出庭的公诉人认为，被告人有坦白情节，建议判处被告人有期徒刑一年至三年，并处罚金。

刑事附带民事公益诉讼原告人贵州省独山县人民检察院诉称，根据公诉机关指控的犯罪事实，被告人王海波违反野生动物法规，明知穿山甲系国家二级保护野生动物，未经野生动物行政主管部门许可，擅自委托他人运输两只穿山甲，其行为使生态资源受到严重破坏，社会公共利益受到侵害，应承担相应的民事侵权责任，并提交了司法鉴定意见书等证据。诉请依法判决：判令被告赔偿两只穿山甲的经济损失共计80000元。

被告人王海波对指控的犯罪事实无异议，但辩称是帮忙运输，不是买的。对附带民事公益诉讼部分的事实无异议，但认为赔偿数额过高。

辩护人的辩护意见是：1、对指控的犯罪事实和罪名均无异议；2、被告人当庭自愿认罪，系初犯，有坦白情节，可以对其从轻处罚；被告人被抓获后，也希望通过放生让穿山甲存活，主观上并不希望穿山甲死亡，恳请法庭予以充分考虑。

经审理查明，2019年5月21日被告人王海波将2只“穿山甲”活体，用纸箱包装后，以100元的运费委托广西钦州开往四川成都的客车驾驶员杨某、龚某运往重庆陈家坪车站，途径兰海高速公路新寨服务区时，被贵州独山毒品检查站民警现场查获。经华南动物物种环境损害司法鉴定中心鉴定，查获的2只“穿山甲”物种均为哺乳纲鳞甲目穿山甲科穿山甲属马来穿山甲，被列入《濒危野生动植物种国际贸易公约》附录Ⅰ，涉案总价值为8万元。

另查明，穿山甲属于《最高人民法院关于审理破坏野生动物资源刑事案件具体应用法律若干问题的解释》附表明确列明的动物。根据《国家重点保护野生动物名录》，穿山甲属于国家二级保护动物。

另，在2019年5月22日被查获后，两只穿山甲于当天被送往贵州森林野生动物园救助站进行救助，一只于当日死亡，另一

只于5月24日放置于食蚁兽外运动场任其自由采食，5月25日发现失踪，6月22日发现已死亡穿山甲尸骨。

上述事实，有经当庭举证、质证的下列证据证实：

一、物证

涉案其中一只穿山甲尸体，另一只穿山甲鳞片。

二、书证

1、人员核查情况表、身份证复印件证实，被告人系完全刑事责任能力人。

2、查获经过及现场查获照片证实，现场查获两只穿山甲情况。

3、到案经过证实，被告人到案的情况。

4、受案登记表、立案决定书、案件移送书、独山县公安局扣押清单、移交清单、拘留证、逮捕证、等文书证实，公安机关办理案件程序合法。

5、鉴定聘请书、第二次鉴定机构及鉴定人员资格证书、鉴定意见告知书证实，鉴定机构、人员有资质，已将鉴定结果告知被告人。

6、贵州省珍稀动物救护中心贵阳森林野生动物园救护站救护穿山甲记录证实，2019年5月22日13时50分，独山县森林公安局办案民警将扣押的两只穿山甲移送贵州省珍稀动物救护中心贵阳森林野生动物园救护站进行收容，收容当天，其中一只

穿山甲可自主活动，已对其自主隔离保护，另外一只在16时40分死亡。

7、贵州省珍稀动物救护中心贵阳森林野生动物园救护站出具的关于救护2只穿山甲情况汇报证实，贵阳森林野生动物园救护站于2019年5月22日收到的独山县森林公安局送来的2只穿山甲，1只于送达后2小时死亡，尸体冷冻于该救护站，另外1只为保证其成活率，于24日将其放置于空置的食蚁兽馆，25日发现该成活个体失踪，经巡查，该区无地面洞穴，引诱蚂蚁食物消失，周边无明显损坏，无法判断该成活个体如何消失。

8、贵州森林野生动物园关于穿山甲情况说明证实，2019年5月22日，独山县森林公安局办案送往贵州省珍稀动物救护中心贵阳森林野生动物园救护站救护的两只穿山甲，其中一只于当日死亡，另一只在救护期间失踪，2019年6月22日在园内发现失踪穿山甲死体，并将骸骨收集后冷冻，同年7月18日，县森林公安局再次入园取证时，该动物园将冷冻的穿山甲骸骨交公安机关带回。

9、国家重点保护野生动物名录表复印件证实，穿山甲属于国家二级保护动物。

三、证人证言

1、证人杨某证言证实，杨某系客车驾驶员，2019年5月21日其驾驶车辆从广西钦州出发，途径广西达梅饭店时，王海波提着一个黄白色大箱子，说是一箱水果叫其帮忙运输到重庆陈家

坪，直到被公安机关检查时，才知道运输的是穿山甲，2019 年 5 月 21 日前杨某与龚某还曾经跟王海波带过三次货，均是从广西带至重庆，接货人电话号码为 135××××0900。

2、证人龚某证言证实，2019 年 5 月 21 日其与驾驶员杨某一起驾驶一辆车牌号为桂 N×××××的黄牌大客车从广西钦州到重庆，途径广西河池市水任梅五饭店时，有一个叫王海波的货主叫帮他带一箱水果到重庆，直到在贵州麻尾检查站被查时，才知道是两只野生动物（活体），当时谈的运费是 100 元，货到付款。2019 年 1 月 9 日、3 月 30 日、4 月 3 日与杨某一起曾经三次帮王海波从广西带货至重庆。

3、证人谭某证言证实，其是王海波的妻子，2019 年看见王海波两次把穿山甲拿回过家，都是用包装纸盒装起的，最后一次是 2019 年 5 月 21 日下午在家装过两只穿山甲，另一次大概是今年 3 月份。王海波做野生动物生意大儿子王某有在旁边见过，其他家人不知情。知道买卖国家重点保护的穿山甲是违法的，也劝过王海波，但王海波不听。

4、证人王某证言证实，其是王海波儿子，王海波这两年在搞穿山甲生意，其母亲也知道王海波在做穿山甲生意，有时还帮忙包装，王某偶尔也看见王海波在家包装穿山甲，王海波以前养过野生动物，应该知道穿山甲是保护动物。

5、证人邓某、石某、张某证言证实，是动物园工作人员，动物园丢失过一只穿山甲，后来发现一只死亡的穿山甲，经兽医确认是动物园内失踪的穿山甲尸体。

6、证人田某证言证实，其是动物园兽医，独山县森林公安所扣押的两只穿山甲在送往贵州森林野生动物园寄养后，其中一只在送去当天死亡，另外一只因不适应生存环境在饲养期间失踪，之后在院内发现已经死亡，尸体已经腐烂，并只剩下骨头和鳞片。

四、被告人供述与辩解

被告人王海波的供述和辩解证实，其在2019年5月21日以100元的价格委托桂N×××××客车驾驶员杨某、龚某从广西河池市运输2只穿山甲至重庆市陈家坪，2只穿山甲系跟“黄仔”收购，卖给一名叫王林的人，其知道穿山甲系国家保护动物。

五、辨认、指认、提取笔录及照片

1、被告人王海波辨认笔录及照片证实，辨认涉案两只穿山甲系其以100元价格委托托运至重庆的物品。

2、证人龚某、杨某辨认笔录证实，王海波为非法运输珍贵、濒危野生动物的人。

3、提取笔录及照片证实，公安机关办案民警提取已死亡的疑似穿山甲冰冻尸体1只(整体)作为种属鉴定检材；将所扣押的王海波的手机开启，并将该手机中留存拍摄于2018年5月30日的1只穿山甲照片及视频经王海波仔细查看后，通过其手机截图

功能截取照片及拷贝视频的方式固定证据；对独山县森林公安局物证保管室的两只疑似“穿山甲”的样本进行提取并装入密封袋。

六、鉴定意见

经华南动物物种环境损害司法鉴定中心鉴定，查获的2只“穿山甲”物种均为哺乳纲鳞甲目穿山甲科，属马来穿山甲，属于国家二级保护动物，涉案总价值为8万元。

七、现场勘验检查笔录、照片

公安机关对查获穿山甲现场进行勘查，对带回独山县森林公安局的两只疑似“穿山甲”进行勘查，对饲养穿山甲的食蚁兽馆以及发现穿山甲死亡的地方进行现场勘查。

八、视听资料

对被告人讯问时同步录音录像，提取被告人手机中留存拍摄的穿山甲照片及视频，动物园工作人员拍摄穿山甲饲养过程等。

九、专家意见

涉案的马来穿山甲是8种穿山甲中最为濒危的物种，穿山甲的食性以蚂蚁为食，食性特殊，在运输过程中天气炎热，容易引起消化道梗阻死亡，存活率较低，很难人工饲养等。

上述证据来源程序合法、有效，证据之间相互印证，客观反映案件事实，本院依法予以确认。

本院认为，被告人王海波违反国家有关野生动物保护法规，运输国家二级重点保护野生动物穿山甲2只，其行为已构成非法运输珍贵、濒危野生动物罪。公诉机关指控罪名成立，本院予以

确认。被告人庭前供述反复且当庭翻供，虽认罪，但非真诚认罪，量刑时应从严考虑；公诉机关建议判处被告人有期徒刑一年至三年的量刑意见适当，本院予以采纳。

被告人的犯罪行为破坏了生态环境，严重损害生态多样性，损害社会公共利益，同时也侵害了国家对野生动物资源的所有权，造成国家野生动物资源的损失，除应受到刑事处罚外，还应承担相应的民事侵权责任。根据《中华人民共和国侵权责任法》的规定，承担侵权责任的方式有赔偿损失，赔偿损失的依据，可根据被告人非法运输的野生动物的价值来判断，所以附带民事公益诉讼人的诉讼请求合法有据，应予以支持。

据此，根据被告人的犯罪事实、性质、情节及对社会的危害程度，同时体现我国惩罚与教育相结合的刑罚目的和宽严相济的刑事政策，依照《中华人民共和国刑法》第三百四十一条第一款、第三十六条，《中华人民共和国野生动物保护法》第三条，《中华人民共和国物权法》第四条，《中华人民共和国侵权责任法》第三条、第四条、第十五条第一款第（六）项，《最高人民法院关于审理破坏野生动物资源刑事案件具体应用法律若干问题的解释》第一条，《最高人民法院关于审理环境民事公益诉讼案件适用法律若干问题的解释》第十八条，《最高人民法院、最高人民检察院关于检察公益诉讼案件适用法律若干问题的解释》第四条、第二十条之规定，判决如下：

一、被告人王海波犯非法运输珍贵、濒危野生动物罪，判处有期徒刑一年，并处罚金人民币五千元；

（刑期自判决执行之日起计算。判决执行以前先行羁押的，羁押一日折抵刑期一日，即自二〇一九年五月二十五日起至二〇二〇年五月二十四日止。罚金限判决生效后即缴纳，逾期强制缴纳。）

二、被告人王海波于判决生效之日起三十日内赔偿国家资源损失费八万元。

如不服本判决，可在接到判决书的第二日起十日内，通过本院或者直接向贵州省黔南布依族苗族自治州中级人民法院提出上诉。书面上诉的，应当提交上诉状正本一份，副本两份。

审　判　长　　姜文明

审　判　员　　杨世萍

审　判　员　　左　丹

人民陪审员　　梁恒宇

人民陪审员　　韦开庆

人民陪审员　　陈兴国

人民陪审员　　严　靖

二〇一九年十二月二十日

书　记　员　　饶　玥
