# Supplementary material for: China’s Legal Protection System for Pangolins: Past, Present, and Future
Source: Animals (Basel). 2025 Aug 18;15(16):2422. doi: 10.3390/ani15162422 (PMC12383201; doi:10.3390/ani15162422)
Supplement: Supplementary file 1 [file animals-15-02422-s001.zip › Supplementary Material S4-Full Text of Judgments in Pangolin-Related Public Interest Litigation Cases in China/【45】吴志杰、黄路易非法收购、运输、出售珍贵、濒危野生动物、珍贵、濒危野生动物制品罪一审刑事判决书.pdf]

吴志杰、黄路易非法收购、运输、出售珍贵、濒危野生动物、珍贵、濒危野生动物制品罪一审刑事判决书

浙江省兰溪市人民法院

刑 事 判 决 书

（2019）浙 0781 刑初 445 号

公诉机关浙江省兰溪市人民检察院。

被告人吴志杰，男，1992 年 9 月 20 日出生于浙江省兰溪市，汉族，初中文化，农民，家住浙江省兰溪市。因涉嫌非法出售珍贵野生动物犯罪于 2019 年 5 月 30 日被兰溪市公安局刑事拘留，同年 6 月 6 日被逮捕，同年 11 月 29 日变更为取保候审。现在家候审。

辩护人金晓英，浙江当金律师事务所律师。

被告人黄路易，男，1997 年 9 月 10 日出生于浙江省衢州市衢江区，汉族，初中文化，农民，家住浙江省兰溪市。因涉嫌非法收购珍贵野生动物犯罪于 2019 年 5 月 27 日被兰溪市公安局刑事拘留，同年 6 月 6 日被逮捕，同年 11 月 29 日变更为取保候审。现在家候审。

辩护人童小艳，浙江大名律师事务所律师。

浙江省兰溪市人民检察院以兰检公诉刑诉（2019）386 号起诉书指控被告人吴志杰、黄路易犯非法出售、收购珍贵野生动物罪，于 2019 年 12 月 2 日向本院提起公诉，本院依法适用简易程序并组成合议庭，公开开庭审理了本案。兰溪市人民检察院指派

检察员何志贤、俞骅出庭支持公诉，被告人吴志杰及由兰溪市法律援助中心指派的辩护人金晓英、被告人黄路易及辩护人童小艳到庭参加诉讼。现已审理终结。

经审理查明，2019年5月27日，被告人黄路易通过电话联系被告人吴志杰，要求购买穿山甲，后被告人吴志杰从其上家处以人民币8000余的价格购买到穿山甲一只，至兰溪市山衢人家饭店，将该穿山甲以人民币11000元的价格出售给被告人黄路易。2019年5月27日17时许，兰溪市公安局民警在兰溪市山衢人家饭店负一楼厨房排烟管道隔间内发现该穿山甲并依法予以扣押。2019年5月27日，经兰溪市野生动物植物保护管理站物种认定意见认定，被扣押的穿山甲属于国家二级重点保护野生动物。2019年6月5日，经浙江师范大学化学与生命科学学院鉴定，被扣押的穿山甲为马来穿山甲，属于《濒危野生动植物种国际贸易公约》附录I中的保护动物。2019年11月14，被告人吴志杰、黄路易各自缴纳了公益诉讼赔偿金20000元。

上述事实，被告人吴志杰、黄路易在开庭审理过程中无异议，并有公诉机关当庭宣读和出示的被告人吴志杰、黄路易的供述与辩解，户籍查询记录，兰溪市公安局扣押决定书，扣押清单、现场照片，金华市陆生野生动物救护接收单，情况说明，现金交款单（公益诉讼赔偿金），兰溪市野生动植物保护站物种认定意见，兰溪市公安局鉴定聘请书，浙江师范大学化学与生命科学学院野生动物案件鉴定意见书，鉴定意见通知书，被告人吴志杰的辨认

笔录，黄路易的辨认笔录，兰溪市公安局检查笔录等证据证实，足以认定。

本院认为，被告人吴志杰违反国家法律、法规，非法出售国家重点保护的珍贵、濒危野生动物，其行为已构成非法出售珍贵濒危野生动物罪。被告人黄路易违反国家法律、法规，非法收购国家重点保护的珍贵、濒危野生动物，其行为已构成非法收购珍贵、濒危野生动物罪。公诉机关指控的罪名成立，予以支持。鉴于被告人吴志杰、黄路易能够自愿认罪，并如实供述罪行，自觉交纳了公益诉讼赔偿金，决定予以酌情从轻处罚并可适用缓刑，辩护人的辩护意见予以采纳。据此，依照《中华人民共和国刑法》第三百四十一条第一款、第七十二条第一款、第七十三条第二、三款之规定，判决如下：

一、被告人吴志杰犯非法出售珍贵、濒危野生动物罪，判处有期徒刑一年，缓刑一年六个月，并处罚金人民币20000元（缓刑考验期限，从判决确定之日起计算。罚金限判决生效后十日内缴纳）；

二、被告人黄路易犯非法收购珍贵、濒危野生动物罪，判处有期徒刑十个月，缓刑一年，并处罚金人民币15000元（缓刑考验期限，从判决确定之日起计算。罚金限判决生效后十日内缴纳）；

三、追缴被告人吴志杰的违法所得款人民币11000元。

如不服本判决，可在接到判决书的第二日起十日内，通过本院或者直接向浙江省金华市中级人民法院提出上诉。书面上诉的，应当提交上诉状正本一份、副本一份。

审　判　长　　施俊慧

人民陪审员　　祝丽香

人民陪审员　　楼　英

二〇一九年十二月十九日

代书　记员　　黄雪君
