# Supplementary material for: China’s Legal Protection System for Pangolins: Past, Present, and Future
Source: Animals (Basel). 2025 Aug 18;15(16):2422. doi: 10.3390/ani15162422 (PMC12383201; doi:10.3390/ani15162422)
Supplement: Supplementary file 1 [file animals-15-02422-s001.zip › Supplementary Material S4-Full Text of Judgments in Pangolin-Related Public Interest Litigation Cases in China/【46】郭明山、李文波非法猎捕、杀害珍贵、濒危野生动物一审刑事判决书(FBM-CLI.C.96450271).pdf]

郭明山、李文波非法猎捕、杀害珍贵、濒危野生动物一审刑事判决书

郭明山、李文波非法猎捕、杀害珍贵、濒危野生动物一审刑事判决书

云南省瑞丽市人民法院

刑事附带民事判决书

(2019)云3102刑初389号

公诉机关暨公益诉讼起诉人瑞丽市人民检察院。

被告人郭明山。2018年6月20日因涉嫌非法猎捕、杀害珍贵、濒危野生动物罪被德宏州瑞丽市森林公安局刑事拘留，同年6月25日被变更强制措施为取保候审，2019年6月14日瑞丽市人民检察院继续取保候审，2019年11月13日本院继续对其取保候审。

被告人李文波。2018年6月20日因涉嫌非法猎捕、杀害珍贵、濒危野生动物罪被德宏州瑞丽市森林公安局刑事拘留，同年6月25日被变更强制措施为取保候审，2019年6月14日瑞丽市人民检察院继续取保候审，2019年11月13日本院继续对其取保候审。

被告人陈兴海。2018年6月22日因涉嫌非法猎捕、杀害珍贵、濒危野生动物罪被德宏州瑞丽市森林公安局取保候审，2019年6月14日瑞丽市人民检察院继续取保候审，2019年11月13日本院继续对其取保候审。

瑞丽市人民检察院以瑞检公诉刑诉[2019]294号起诉书指控被告人郭明山、李文波、陈兴海犯非法猎捕、杀害珍贵、濒危野生动物罪，于2019年11月13日向本院提起公诉。本院于当日立案。期间，瑞丽市人民检察院以瑞检民公[2019]53310200003号刑事附带民事公益诉讼起诉书，提起刑事附带民事公益诉讼，本院受理后，依法组成合议庭，适用简易程序，于2019年11月29日公开开庭审理了本案。瑞丽市人民检察院指派检察员马珊、书记员余蕊出庭支持公诉，同时指派检察员马珊、书记员余蕊出庭参加刑事附带民事公益诉讼。被告人郭明山、李文波、陈兴海均到庭参加诉讼。现已审理终结。

经审理查明：2018年6月13日,被告人郭明山在陇川县陇川公墓附近抓获一只国家二级保护动物

马来穿山甲。2018年6月19日,被告人郭明山、李文波、陈兴海在瑞丽市慧兰饭店将穿山甲杀害并准备加工食用，当日19时许，被瑞丽市公安局民警在巡逻时查获。经鉴定，涉案穿山甲死体及穿山甲甲片均来源为鳞甲目穿山甲科山甲属马来穿山甲，穿山甲为国家II级保护野生动物，马来穿山甲列入《濒危野生动植物种国际贸易公约》(CITES)附录I，经济价值为40000元。

另查明，被告人郭明山、李文波、陈兴海在犯罪以后自动投案，如实供述自己的罪行。

再查明，被告人郭明山、李文波、陈兴海自愿如实供述自己的罪行，承认指控的犯罪事实，愿意接受处罚。

又查明，公益诉讼起诉人在拟提起刑事附带民事公益诉讼前，已依法进行了公告。

上述事实，被告人郭明山、李文波、陈兴海在庭审中无异议，并当庭表示自愿认罪认罚，无辩解和辩护意见。且有户口证明、抓获和到案经过、辨认笔录及照片、现场勘查笔录及物证照片、扣押决定书及扣押清单、证人证言、被告人供述及辩解、司法鉴定意见书、鉴定意见通知书、认罪认罚具结书、同步录音录等证据在卷予以证实，以上证据取证程序合法，内容客观真实能够相互印证，形成证据锁链，本院予以确认。

本院认为，被告人郭明山无视国家法律，违反野生动物保护法规，非法猎捕、杀害国家重点保护的珍贵、濒危野生动物，其行为已触犯《中华人民共和国刑法》第三百四十一条第一款之规定，构成非法猎捕、杀害珍贵、濒危野生动物罪，应依法予以惩处。被告人李文波、陈兴海违反野生动物保护法规，非法杀害国家重点保护的珍贵、濒危野生动物，其行为已触犯《中华人民共和国刑法》第三百四十一条第一款之规定，构成非法杀害珍贵、濒危野生动物罪，应依法予以惩处。公诉机关指控的犯罪事实清楚，证据确凿，罪名成立，本院予以支持。被告人郭明山、李文波、陈兴海有自首情节，查证属实，本院依法从轻处罚。鉴于被告人郭明山、李文波、陈兴海自愿如实供述自己的罪行，承认指控的犯罪事实，愿意接受处罚，且系初犯，情节较轻，可依法免于刑事处罚。公诉机关提出的量刑建议，本院予以采纳。

公益诉讼起诉人诉请要求：1、依法判令被告人郭明山、李文波、陈兴海共同赔偿非法猎捕、杀害的一只国家二级保护动物马来穿山甲的价值人民币40000元；2、依法判令郭明山、李文波、陈兴海就非法猎捕、杀害珍贵、濒危野生动物导致国家利益和社会公共利益受到损害的行为当庭向社会公众赔礼道歉的诉请符合法律规定，本院予以支持。

本院认为，被告人郭明山、李文波、陈兴海非法猎捕、杀害国家重点保护的珍贵、濒危野生动物的犯罪行为破坏生态环境资源，造成生态资源损失，虽然被告人郭明山、李文波、陈兴海已当庭向社会公众赔礼道歉，但亦应当承担民事责任。据此，本院根据被告人郭明山、李文波、陈兴海犯罪的事实、犯罪的性质、情节和对于社会的危害程度，依照《中华人民共和国刑法》第三百四十一条第一款、第六十七条第一款、第六十一条、第六十四条、《中华人民共和国刑事诉讼法》第十五条、《中华人民共和国侵权责任法》第四条第一款、第十五条及《最高人民法院、最高人民检察院关于检察公益诉讼案件适用法律若干问题的解释》第十三条、第二十条之规定，判决如下：

一、被告人郭明山犯非法猎捕、杀害珍贵、濒危野生动物罪，免于刑事处罚。

二、被告人李文波犯非法杀害珍贵、濒危野生动物罪，免于刑事处罚。

三、被告人陈兴海犯非法杀害珍贵、濒危野生动物罪，免于刑事处罚。

四、查获扣押在案的穿山甲甲片褐色280克及供犯罪所用的工具（刀具、钳子各一把），予以没收并依法处理。

五、被告人郭明山、李文波、陈兴海共同赔偿非法猎捕、杀害的一只国家二级保护动物马来穿山甲的价值人民币40000元。

如不服本判决，可在接到判决书的第二日起十日内，通过本院或者直接向云南省德宏州中级人民法院提出上诉。书面上诉的，应当提交上诉状正本一份，副本二份。

审　判　长　陈　炜

人民陪审员　高红旗

人民陪审员　江丽云

二〇一九年十二月十八日

书　记　员　董生有

附：本案相关法律

《中华人民共和国刑法》

第三百四十一条第一款非法猎捕、杀害国家重点保护的珍贵、濒危野生动物的，或者非法收购、运输

、出售国家重点保护的珍贵、濒危野生动物及其制品的，处五年以下有期徒刑或者拘役，并处罚金；情节严重的，处五年以上十年以下有期徒刑，并处罚金；情节特别严重的，处十年以上有期徒刑，并处罚金或者没收财产。

第六十七条第一款犯罪以后自动投案，如实供述自己的罪行的，是自首。对于自首的犯罪分子，可以从轻或者减轻处罚。其中，犯罪较轻的，可以免除处罚。

第六十一条对于犯罪分子决定刑罚的时候，应当根据犯罪的事实、犯罪的性质、情节和对于社会的危害程度，依照本法的有关规定判处。

第六十四条犯罪分子违法所得的一切财物，应当予以追缴或者责令退赔；对被害人的合法财产，应当及时返还；违禁品和供犯罪所用的本人财物，应当予以没收。没收的财物和罚金，一律上缴国库，不得挪用和自行处理。

《中华人民共和国刑事诉讼法》

第十五条犯罪嫌疑人、被告人自愿如实供述自己的罪行，承认指控的犯罪事实，愿意接受处罚的，可以依法从宽处理。

《中华人民共和国侵权责任法》

第四条第一款侵权人因同一行为应当承担行政责任或者刑事责任的，不影响依法承担侵权责任。

第十五条承担侵权责任的方式主要有：

（一）停止侵害；

（二）排除妨碍；

（三）消除危险；

（四）返还财产；

（五）恢复原状；

（六）赔偿损失；

（七）赔礼道歉；

（八）消除影响、恢复名誉。

以上承担侵权责任的方式，可以单独适用，也可以合并适用。

《最高人民法院、最高人民检察院关于检察公益诉讼案件适用法律若干问题的解释》

第十三条人民检察院在履行职责中发现破坏生态环境和资源保护、食品药品安全领域侵害众多消费者合法权益等损害社会公共利益的行为,拟提起公益诉讼的,应当依法公告,公告期间为三十日。

公告期满,法律规定的机关和有关组织不提起诉讼的,人民检察院可以向人民法院提起诉讼。

第二十条人民检察院对破坏生态环境和资源保护、食品药品安全领域侵害众多消费者合法权益等损害社会公共利益的犯罪行为提起刑事公诉时,可以向人民法院一并提起附带民事公益诉讼,由人民法院同一审判组织审理。

人民检察院提起的刑事附带民事公益诉讼案件由审理刑事案件的人民法院管辖。

©北大法宝：（www.pkulaw.com）专业提供法律信息、法学知识和法律软件领域各类解决方案。北大法宝为您提供丰富的参考资料，正式引用法规条文时请与标准文本核对。 欢迎查看所有产品和服务。

法宝快讯： 如何快速找到您需要的检索结果？ 法宝 V6 有何新特色？

扫描二维码阅读原文

原文链接： https://www.pkulaw.com/pfnl/a6bdb3332ec0adc4bdbfaf6dc4ce0221a592dfaa5b8b19d2bdfb.html
