# Supplementary material for: China’s Legal Protection System for Pangolins: Past, Present, and Future
Source: Animals (Basel). 2025 Aug 18;15(16):2422. doi: 10.3390/ani15162422 (PMC12383201; doi:10.3390/ani15162422)
Supplement: Supplementary file 1 [file animals-15-02422-s001.zip › Supplementary Material S4-Full Text of Judgments in Pangolin-Related Public Interest Litigation Cases in China/【47】刘规健、刘劲非法收购、运输、出售珍贵、濒危野生动物、珍贵、濒危野生动物制品罪一审刑事判决书.pdf]

刘规健、刘劲非法收购、运输、出售珍贵、濒危野生动物、珍贵、濒危野生动物制品罪一审刑事判决书

广西壮族自治区玉林市玉州区人民法院

刑 事 附 带 民 事 判 决 书

（2019）桂 0902 刑初 669 号

公诉机关暨公益诉讼机关广西壮族自治区玉林市玉州区人民检察院。

被告人刘劲（曾用名刘劲良），男，1966 年 2 月 2 日出生于广西玉林市，汉族，小学文化，居民，户籍地玉林市福绵区，住玉林市玉州区。因本案于 2019 年 7 月 15 日被羁押，次日刑事拘留，同年 8 月 21 日被逮捕。现羁押于玉林市看守所。

辩护人暨诉讼代理人刘恩明，广西三益律师事务所律师。

被告人刘规健，男，1987 年 12 月 10 日出生于广西玉林市，汉族，初中文化，居民，户籍地玉林市福绵区，住玉林市玉州区。因本案于 2019 年 7 月 15 日被羁押，次日被刑事拘留，同年 8 月 21 日被逮捕。现羁押于玉林市看守所。

辩护人暨诉讼代理人钟春亮，广西欣和律师事务所律师。

广西壮族自治区玉林市玉州区人民检察院以玉区检公诉刑诉〔2019〕642 号起诉书、玉区检民公〔2019〕45090200010 号刑事附带民事公益诉讼起诉书指控被告人刘劲、刘规健犯非法收购、运输、出售珍贵、濒危野生动物、珍贵、濒危野生动物制品罪，于 2019 年 11 月 18 日向本院提起公诉及附带民事公益诉讼，

本院于同月25日立案，依法组成合议庭，适用普通程序，公开开庭进行了合并审理。玉林市玉州区人民检察院指派检察员冯良瑜出庭支持公诉，指派检察员陈广庆出庭参加公益诉讼，被告人刘劲及其辩护人暨诉讼代理人刘恩明，被告人刘规健及其辩护人暨诉讼代理人钟春亮到庭参加诉讼。现已审理终结。

经审理查明，被告人刘劲、刘规健是父子。2013年开始，刘劲非法收购穿山甲、鹰等野生动物用于出售获利。2019年6月开始，刘规健驾驶车牌号为桂K×××××五菱牌小型客车帮其父刘劲运送野生动物。同年7月15日晚，刘规健驾驶上述小汽车到高速公路玉林南出口附近接运穿山甲。当晚9时许，刘规健驾车回到玉林市玉州区大北路金属回收再生公司时被抓，民警当场缴获10只穿山甲（活体），刘劲来到现场时亦被当场抓获。此外，民警还从刘劲租用的上述公司“葵阳”、“石南”仓库的冰箱内缴获2只鹰科鸟类（冻体）、12只豹猫（冻体）、3只飞鼠（冻体）、3只猪鼻狸（冻体）、5只果子狸（活体）以及1袋穿山甲甲片（净重220克）。经广西壮族自治区森林公安局物证鉴定所鉴定，缴获的2只鹰科鸟类（冻体）为隼形目鹰科鸟类产品；10只穿山甲（活体）为鳞甲目鲮鲤科穿山甲属动物；动物鳞片（净重220克）为鳞甲目鲮鲤科穿山甲属动物鳞片产品，均属国家二级重点保护野生动物及其制品。

另查明，公安机关抓获刘劲、刘规健时，分别从刘劲处缴获作案工具1台黑色华为NOVA4E手机、1台黑色OUKI牌老人机；

从刘规健处缴获作案工具1辆车牌号为桂K×××××白色五菱牌小型普通客车（机动车所有人：刘规健）、1台白色苹果X手机、1台金色苹果6手机。

上述事实，被告人刘劲、刘规健在开庭审理过程中亦无异议，且有现场图，现场勘查笔录及照片，辨认笔录及照片，指认照片，搜查笔录及照片，提取痕迹、物证登记表，扣押清单，通行记录，视频截图，通话记录，微信聊天，银行明细查询，电子数据检查笔录及光盘，交易记录账本，广西壮族自治区森林公安局物证鉴定所鉴定书，同案陈成才、冯某供述，被告人刘劲、刘规健供述，抓获经过，办案说明，户籍证明等证据证实，足以认定。

被告人刘劲、刘规健及其诉讼代理人对公益诉讼机关请求判令二被告人各自在玉林市市级主要媒体向社会公众赔礼道歉及承担连带责任支付公告费800元均无异议。

审理期间，被告人刘劲的家属代其交纳罚金50000元。被告人刘规健的家属代其交纳罚金20000元、公告费800元。

本院认为，被告人刘劲、刘规健违反野生动物保护法规，收购、出售国家重点保护的珍贵、濒危野生动物及其制品，情节严重，其二人行为已触犯刑律，构成了非法收购、运输、出售珍贵、濒危野生动物、珍贵、濒危野生动物制品罪。公诉机关指控的罪名成立。刘劲、刘规健共同故意犯罪，属共同犯罪。在共同犯罪中，刘劲积极实施犯罪，起主要作用，是主犯，应当按照其所参与的全部犯罪处罚；刘规健起次要作用，是从犯，应当减轻处罚。

公诉机关指控刘规健是主犯不当，应予纠正。刘劲、刘规健归案后如实供述其罪行，属坦白，且认罪认罚，可以从轻处罚。刘劲、刘规健的行为破坏了国家野生动物资源，侵害了国家对野生动物资源的所有权，破坏了生物多样性，危害生态系统平衡，损害了国家利益和社会公共利益，已构成民事侵权，应承担赔礼道歉等相应的民事责任。玉林市玉州区人民检察院请求判令刘劲、刘规健各自在玉林市市级主要媒体向社会公众赔礼道歉及承担公益诉讼支出公告费800元的诉求，依法予以支持。刘劲、刘规健的作案工具，依法予以没收上缴国库。鉴于刘规健是从犯且无前科，确有悔罪表现，根据其犯罪情节及悔改表现，对其适用缓刑没有再犯罪的危险，且对所居住的社区没有重大不良影响，本院决定对刘规健适用缓刑。刘劲及其辩护人提出刘劲认罪认罚，请求对其从轻处罚；刘规健及其辩护人提出刘规健是从犯且认罪，请求对其减轻处罚，上述意见，经查有事实和法律依据，均予以采纳。

为严肃国法，维护国家重点保护的珍贵、濒危野生动物的管理制度，根据被告人刘劲、刘规健犯罪的事实，犯罪的性质、情节以及对于社会的危害程度，依照《中华人民共和国刑法》第三百四十一条第一款，第二十五条第一款，第二十六条第一款和第四款，第二十七条，第六十七条第三款，第五十二条，第六十四条，第七十二条第一款，第七十三条第二款和第三款，《最高人民法院关于审理破坏野生动物资源刑事案件应用法律若干问题的解释》第一条，第二条，第三条第一款第（一）项，《中华人

民共和国侵权责任法》第十五条，《最高人民法院关于审理环境民事公益诉讼案件适用法律若干问题的解释》第十八条，第二十二条之规定，判决如下：

一、被告人刘劲犯非法收购、运输、出售珍贵、濒危野生动物、珍贵、濒危野生动物制品罪，判处有期徒刑五年六个月，并处罚金五万元。

（刑期从判决执行之日起计算，判决执行以前先行羁押的，羁押一日折抵刑期一日。即自2019年7月15日起至2025年1月14日止。罚金已缴纳。）

二、被告人刘规健犯非法收购、运输、出售珍贵、濒危野生动物、珍贵、濒危野生动物制品罪，判处有期徒刑三年，缓刑四年，并处罚金二万元。

（缓刑考验期限，从判决确定之日起计算。罚金已缴纳）。

三、被告人刘规健退出的800元公告费退赔玉林市玉州区人民检察院（该款已交来本院）。

四、责令被告人刘劲、刘规健在玉林市市级主要媒体向社会公众赔礼道歉。

五、没收被告人刘劲的作案工具1台黑色华为NOVA4E手机、1台黑色OUKI牌老人机；刘规健的作案工具1辆车牌号为桂K×××××白色五菱牌小型普通客车（机动车所有人：刘规健）、1台白色苹果X手机、1台金色苹果6手机，拍卖所得款上缴国库。

如不服本判决，可在收到判决书之次日起十日内，通过本院或直接向广西壮族自治区玉林市中级人民法院提出上诉。书面上诉的应当提交上诉状正本一份、副本二份。

审　判　长　　梁　林

人民陪审员　　李翠云

人民陪审员　　蒋　鑫

二〇一九年十二月十六日

书　记　员　　王　霞
