# Supplementary material for: China’s Legal Protection System for Pangolins: Past, Present, and Future
Source: Animals (Basel). 2025 Aug 18;15(16):2422. doi: 10.3390/ani15162422 (PMC12383201; doi:10.3390/ani15162422)
Supplement: Supplementary file 1 [file animals-15-02422-s001.zip › Supplementary Material S4-Full Text of Judgments in Pangolin-Related Public Interest Litigation Cases in China/【48】杨阿四、杨小勇一审刑事判决书.pdf]

杨阿四、杨小勇一审刑事判决书

云南省元江哈尼族彝族傣族自治县人民法院

刑 事 附 带 民 事 判 决 书

（2019）云 0428 刑初 126 号

公诉机关暨公益诉讼起诉人元江哈尼族彝族傣族自治县人民检察院。

被告人杨阿四，女，1970 年 7 月 28 日出生于云南省元江县。因涉嫌非法出售珍贵、濒危野生动物制品罪，于 2019 年 8 月 1 日被元江县森林公安局取保候审。

被告人杨小勇，男，1978 年 8 月 9 日出生于云南省元江县。因涉嫌非法收购珍贵、濒危野生动物制品罪，于 2019 年 8 月 1 日被元江县森林公安局取保候审。

元江哈尼族彝族傣族自治县人民检察院以元检一部刑诉[2019]53 号起诉书指控被告人杨阿四、杨小勇，犯非法出售珍贵、濒危野生动物制品罪，于 2019 年 10 月 18 日向本院提起公诉。元江哈尼族彝族傣族自治县人民检察院以公益诉讼起诉人的身份以被告杨阿四、杨小勇的犯罪行为导致国家野生动物资源遭到破坏，损害了国家和社会公共利益为由向本院提起公益诉讼。本院依法组成合议庭，公开开庭合并审理了本案。公诉机关暨公益诉讼起诉人元江哈尼族彝族傣族自治县人民检察院指派检察员杨智浩、书记员倪泥、罗长青出庭支持公诉和参加附带民事部

分的公益诉讼，被告杨阿四、杨小勇到庭参加诉讼。现已审理终结。

公诉机关指控，2019年5月16日，被告人杨阿四在元江县农贸市场其摊位处摆放疑似穿山甲鳞片欲出售时被玉溪市和元江县森林公安局民警查获。同日，被告人杨小勇明知其妻杨阿四欲出售穿山甲鳞片，还将放在其家里的一袋穿山甲鳞片送去元江县农贸市场其妻的摊位处。民警从被告人杨阿四摊位处及其租住的元江县某号家里的电视柜内共查获116片疑似穿山甲鳞片。经鉴定，从被告人杨阿四和杨小勇处查获的疑似穿山甲鳞片均来自鳞甲目穿山甲科穿山甲属Manissp，穿山甲为国家Ⅱ级保护野生动物，经济价值为106240元。

公益诉讼起诉人元江哈尼族彝族傣族自治县人民检察院提出，被告杨阿四、杨小勇非法出售国家重点保护野生动物制品，导致国家的野生动物资源遭到破坏，损害了国家和社会公共利益，应承担赔偿损失和赔礼道歉的民事责任。请求：一、判令被告杨阿四、杨小勇赔偿国家野生动物资源损失106240元；二、判令被告杨阿四、杨小勇公开赔礼道歉。

被告人杨阿四提出，对公诉机关指控的事实和罪名无异议，其自身患有多种疾病，购买指控的野生动物制品是为了治病。对于公益诉讼起诉人要求赔偿国家野生动物资源损失106240元的请求及赔礼道歉无异议，但其个人没有赔偿能力。

被告人杨小勇提出，对起诉书指控的事实与罪名无异议，对公益诉讼起诉人要求赔偿和赔礼道歉的请求无异议，但其个人没有赔偿能力。

经审理查明，2019年5月16日，被告人杨阿四在元江县农贸市场其摊位处摆放疑似穿山甲鳞片欲出售时被玉溪市和元江县森林公安局民警查获。同日，被告人杨小勇明知其妻杨阿四欲出售穿山甲鳞片，还将放在其家里的一袋穿山甲鳞片送去元江县农贸市场其妻的摊位处。民警从杨阿四摊位处及其租住的元江县某号家里的电视柜内共查获116片疑似穿山甲鳞片。经鉴定，从被告人杨阿四和杨小勇处查获的疑似穿山甲鳞片均来自鳞甲目穿山甲科穿山甲属Manissp，穿山甲为国家Ⅱ级保护野生动物，经济价值为106240元。

2019年11月20日14时40分，公益诉讼起诉人与被告杨阿四、杨小勇对赔礼道歉部分已自行商量并履行完毕。

上述事实，被告人杨阿四、杨小勇在开庭审理过程中无异议，且有物证照片、户口证明、受案登记表、立案决定书、案件来源情况说明、到案经过、公开道歉信；被告人杨阿四、杨小勇的供述与辩解；辨认笔录及照片、勘验检查笔录及照片、搜查证、搜查笔录、扣押决定书、扣押笔录、扣押物品照片、扣押清单、随案移送清单及照片；鉴定委托书、鉴定意见、鉴定意见通知书等证据在案证实。

以上证据，经当庭质证认证，证据收集程序合法，证据证明的内容客观真实，且证据之间能相互印证，足以认定。

本院认为，被告人杨阿四、杨小勇违反国家野生动物保护法规的相关规定，非法出售珍贵、濒危野生动物制品，情节严重，二被告人的行为已构成非法出售珍贵、濒危野生动物制品罪。根据《中华人民共和国刑法》第三百四十一条第一款的规定，非法收购、运输、出售珍贵、濒危野生动物、珍贵、濒危野生动物制品的，处五年以下有期徒刑或者拘役，并处罚金；情节严重的，处五年以上十年以下有期徒刑，并处罚金；情节特别严重的，处十年以上有期徒刑，并处罚金或者没收财产。被告人杨阿四、杨小勇在共同犯罪过程中，被告人杨阿四起主要作用，是主犯，本院依法处罚。被告人杨小勇起次要作用，是从犯，本院依法从轻处罚。被告人杨阿四到案后如实供述犯罪事实，是坦白，依法对其从轻处罚。公诉机关指控的事实与罪名、认定被告人杨阿四具有坦白情节成立，本院予以支持；公诉机关认定二被告人作用相当，不宜划分主从犯的意见，本院认为，被告人杨小勇收购珍贵、濒危野生动物制品仅有其供述，无其他证据证实，被告人杨小勇在出售珍贵、濒危野生动物制品过程中，被告人杨小勇在被告人杨阿四的授意下将穿山甲鳞片送到元江县农贸市场，起次要作用，是从犯，公诉机关认定二被告人作用相当，不宜划分主从犯的意见，本院不予采纳。公益诉讼起诉人要求判令二被告赔偿国家野生动物资源损失 106240 元符合法律规定，本院予以支持。

对于公益诉讼起诉人要求判令二被告公开赔礼道歉的主张。因公益诉讼起诉人与二被告已对赔礼道歉的诉讼请求自行协商，并已按照公益诉讼起诉人的要求履行完毕，对该项诉讼请求，本院不再判决。据此，依照《中华人民共和国刑法》第三百四十一条、第三十六条、第二十五条、第二十六条、第二十七条、第六十七条第三款、第七十二条、第七十三条、第六十四条，《最高人民法院关于审理破坏野生动物资源刑事案件具体应用法律若干问题的解释》第五条第一款第（一）项、《中华人民共和国刑事诉讼法》第一百零一条第二款、第一百零四条，《中华人民共和国侵权责任法》第六条、第八条、第十四条，《最高人民法院关于适用〈中华人民共和国刑事诉讼法〉的解释》第一百四十二条第一款、第一百五十五条第一款，《最高人民法院、最高人民检察院关于检察公益诉讼案件适用法律若干问题的解释》第二十条，《最高人民法院关于审理环境民事公益诉讼案件适用法律若干问题的解释》第二十条第二款、第二十四条之规定，判决如下：

一、被告人杨阿四犯非法出售珍贵、濒危野生动物制品罪，判处有期徒刑五年，并处罚金人民币 20000 元。（刑期从判决执行之日起计算，即自 2019 年 11 月 20 日起至 2024 年 11 月 19 日止）。

二、被告人杨小勇犯非法出售珍贵、濒危野生动物制品罪，判处有期徒刑三年，缓刑三年，并处罚金人民币 10000 元（缓刑考验期从判决确定之日起计算）。

三、扣押在元江县森林公安局的穿山甲鳞片 116 片（净重 1.905 千克）予以没收。

四、由被告杨阿四、杨小勇共同赔偿国家野生动物资源损失 106240 元，缴至元江哈尼族彝族傣族自治县财政局指定的账户，用作保护当地野生动物的经费。

如不服本判决，可在接到判决书的第二日起十日内，通过本院或者直接向云南省玉溪市中级人民法院提出上诉。书面上诉的，应当提交上诉状正本一份，副本二份。

审　判　长　　覃建国

审　判　员　　白相爱

审　判　员　　马艾萍

人民陪审员　　封建昌

人民陪审员　　张荣学

人民陪审员　　何保辉

人民陪审员　　房树桦

二〇一九年十一月二十日

书　记　员　　王真臻
