# Supplementary material for: China’s Legal Protection System for Pangolins: Past, Present, and Future
Source: Animals (Basel). 2025 Aug 18;15(16):2422. doi: 10.3390/ani15162422 (PMC12383201; doi:10.3390/ani15162422)
Supplement: Supplementary file 1 [file animals-15-02422-s001.zip › Supplementary Material S4-Full Text of Judgments in Pangolin-Related Public Interest Litigation Cases in China/【49】李某暖、蓝某欣非法收购、运输、出售珍贵、濒危野生动物、珍贵、濒危野生动物制品一审刑事判决书.pdf]

李某暖、蓝某欣非法收购、运输、出售珍贵、濒危野生动物、珍贵、濒危野生动物制品一审刑事判决书

广东省大埔县人民法院

刑 事 附 带 民 事 判 决 书

（2019）粤1422刑初85号

公诉机关暨附带民事公益诉讼起诉人广东省大埔县人民检察院。

被告人李某暖，男，1988年8月1日出生于福建省龙岩市永定区，汉族，高中文化，个体户，户籍所在地福建省龙岩市永定区，羁押前住广东省大埔县。因涉嫌犯非法收购珍贵、濒危野生动物罪，于2019年2月26日被梅州市公安局森林分局羁押，次日被刑事拘留，2019年4月1日被梅州市公安局森林分局逮捕，现押于梅江区看守所。

指定辩护人吴建华，广东法豪律师事务所律师，由大埔县法律援助处指派。

被告人蓝某欣，男，1980年1月20日出生于广东省大埔县，汉族，初中文化，个体户，户籍所在地广东省大埔县，现住大埔县。因涉嫌犯非法出售珍贵、濒危野生动物罪，于2019年2月28日被梅州市公安局森林分局羁押，同日被刑事拘留，同年3月3日被梅州市公安局森林分局取保候审。

指定辩护人饶宗强，广东法豪律师事务所律师，由大埔县法律援助处指派。

大埔县人民检察院以埔检诉刑诉[2019]67号起诉书指控被告人李某暖犯非法收购珍贵、濒危野生动物罪，被告人蓝某欣犯非法出售珍贵、濒危野生动物罪，于2019年8月8日向本院提起公诉，在诉讼过程中，大埔县人民检察院向本院提起附带民事公益诉讼。本院依法组成合议庭，合并公开开庭审理了本案。大埔县人民检察院指派检察员张汉夫出庭支持公诉、指派检察员罗志荣代表刑事附带民事公益诉讼起诉人出席法庭，被告人李某暖及其指定辩护人吴建华、被告人蓝某欣及其指定辩护人饶宗强到庭参加了诉讼。本案现已审理终结。

公诉机关指控，被告人李某暖、蓝某欣明知穿山甲系国家重点保护动物。2019年2月21日，被告人蓝某欣欲出售存放在其大埔县某水产店的穿山甲一只，即通过微信联系买家被告人李某暖。经两人共同商定，被告人蓝某欣将一只6.6斤的穿山甲以人民币8250元的价格（每斤1250元）出售给被告人李某暖。当晚，被告人蓝某欣将该穿山甲送至被告人李某暖位于大埔县某烧烤店；被告人李某暖收到后将该穿山甲放置于其位于大埔县某杂物间内。2019年2月22日，被告人李某暖将交易款8250元通过微信转账给被告人蓝某欣。2019年2月26日，梅州市公安局森林分局在被告人李某暖位于某烧烤店及某杂物间内，查获该只穿山甲、1只果子狸和1只野猫的活体。经华南动物物种环境损害司法鉴定中心鉴定，该穿山甲为穿山甲属(Manis)马来穿山甲(Manisjavanica)，被列入《濒危野生动植物种国际贸易公约》

附录 1，价值为人民币 40000 元。经梅州市林业局查验，果子狸、野猫活体为疑似“三有”保护动物果子狸和豹猫。

梅州市公安局森林分局分别于 2019 年 2 月 26 日、28 日将被告人李某暖、蓝某欣抓获归案。

2019 年 3 月 4 日，梅州市公安局森林分局将扣押的马来穿山甲、果子狸和豹猫移交梅州市林业局保管、收养及救护。马来穿山甲和豹猫于 2019 年 3 月 8 日死亡。

公诉机关认为，被告人李某暖的行为构成了非法收购珍贵、濒危野生动物罪，被告人蓝某欣的行为构成了非法出售珍贵、濒危野生动物罪，鉴于被告人李某暖、蓝某欣自愿如实供述自己的罪行，承认指控的犯罪事实，愿意接受处罚，可以依法从宽处理。公诉机关提出了判处被告人李某暖有期徒刑七个月至九个月，并处罚金，依法可适用缓刑；判处被告人蓝某欣有期徒刑七个月至九个月，并处罚金，依法可适用缓刑的量刑建议。

大埔县人民检察院向本院提出附带民事公益诉讼请求：1、请求判令被告人李某暖、被告人蓝某欣赔偿其因非法收购、出售珍贵、濒危野生动物行为破坏生态环境损失的整体价值 40000 元人民币；2、判令被告人李某暖、蓝某欣在全国公开发行的报纸上发表赔礼道歉声明。事实和理由：2019 年 2 月 22 日，被告人李某暖、被告人蓝某欣双方经商议并以微信转账形式，以人民币 8250 元的价格非法买卖一只马来穿山甲。该马来穿山甲于 2019 年 2 月 26 日被民警从被告人李某暖处现场查获。经华南动物物

种环境损害司法鉴定中心鉴定，该穿山甲为穿山甲属（Manis）马来穿山甲(Manisjavanica)，被列入《濒危野生动植物种国际贸易公约》附录1，属于二级保护动物。本案中马来穿山甲按照国内穿山甲科物种基准价值的五倍核算，涉案马来穿山甲活体1只的价值为：8000*5=40000元。野生动物是生态系统不可替代的组成部分，保护野生动物对于维持生物多样性和生态平衡具有极其重要的意义。被告人李某暖、蓝某欣非法收购、出售珍贵、濒危野生保护动物的行为造成了野生动物多样性和生态环境的破坏，损害了社会公共利益，根据《中华人民共和国环境保护法》第六十四条以及《中华人民共和国侵权责任法》第四条、第八条、第十四条、第十五条之规定，李某暖、蓝某欣使社会公共利益受到侵害的行为，依法应承担赔偿损失，并公开赔礼道歉。

被告人李某暖、蓝某欣均承认控罪，未提出辩解意见，均表示同意附带民事公益诉讼请求。

被告人李某暖的辩护人对公诉机关指控被告人李某暖的行为构成非法收购珍贵、濒危野生动物罪不持异议，其以被告人李某暖归案后能如实供述自己的犯罪事实、认罪悔罪、系初犯、偶犯等为由，提出了请求给予被告人李某暖从轻、减轻或者免除处罚的辩护意见。

被告人蓝某欣的辩护人认为公诉机关指控被告人蓝某欣构成非法出售珍贵、濒危动物罪，事实清楚，适用法律正确，其以被告人蓝某欣是初犯，归案后能自愿如实供述自己的罪行，承认

指控的犯罪事实，愿意接受处罚等为由，请求对被告人蓝某欣量刑时给予从轻、减轻处罚。

经审理查明，被告人李某暖、蓝某欣明知穿山甲系国家重点保护动物。2019 年 2 月 21 日，被告人蓝某欣欲出售存放在其大埔县某水产店的穿山甲一只，即通过微信联系买家被告人李某暖。经两人共同商定，被告人蓝某欣将一只 6.6 斤的穿山甲以人民币 8250 元的价格（每斤 1250 元）出售给被告人李某暖。当晚，被告人蓝某欣将该穿山甲送至被告人李某暖位于大埔县某烧烤店；被告人李某暖收到后将该穿山甲放置于其位于大埔县某杂物间内。2019 年 2 月 22 日，被告人李某暖将交易款 8250 元通过微信转账给被告人蓝某欣。2019 年 2 月 26 日，梅州市公安局森林分局在被告人李某暖位于某烧烤店及某杂物间内，查获该只穿山甲、1 只果子狸和 1 只野猫的活体。经华南动物物种环境损害司法鉴定中心鉴定，该穿山甲为穿山甲属(Manis)马来穿山甲(Manisjavanica)，被列入《濒危野生动植物种国际贸易公约》附录 1，本案中马来穿山甲按照国内穿山甲科物种基准价值的五倍核算，涉案马来穿山甲活体 1 只的价值为：8000*5=40000 元。经梅州市林业局查验，果子狸、野猫活体为疑似“三有”保护动物果子狸和豹猫。

梅州市公安局森林分局分别于 2019 年 2 月 26 日、28 日将被告人李某暖、蓝某欣抓获归案。

2019 年 3 月 4 日，梅州市公安局森林分局将扣押的马来穿山甲、果子狸和豹猫移交梅州市林业局保管、收养及救护。马来穿山甲和豹猫于 2019 年 3 月 8 日死亡。

本案审理期间，被告人李某暖的家属代被告人李某暖自愿向本院预缴了公益诉讼赔偿款人民币 20000 元，并预交纳罚金人民币 2000 元；被告人蓝某欣自愿向本院预缴了公益诉讼赔偿款人民币 20000 元，预交纳罚金人民币 2000 元，退交违法所得款人民币 8250 元。

上述事实，被告人李某暖、蓝某欣在庭审中亦无异议，且有物证穿山甲等的照片，书证受案登记表、立案决定书、户籍证明、查询证明、抓获情况说明、微信转账记录、微信交易记录、搜查笔录、扣押决定书、扣押清单、工商开业登记信息、野生动物物品移交书、随案移送清单、情况说明，证人熊某 1 的证言，被告人李某暖、蓝某欣的供述，华南动物物种环境损害司法鉴定中心司法鉴定意见书（华动司鉴字[2019]第 7 号）、梅州市林业局野生动物查验情况，辨认笔录、现场勘验检查记录等证据证实，足以认定。

附带民事公益诉讼起诉人就其提起的附带民事诉讼向本院提交了除刑事部分的证据外，还提交了检察日报公告、询问笔录等材料。上述材料经庭审质证，本院予以确认。

本院认为，被告人李某暖、蓝某欣无视国家法律，李某暖明知穿山甲系国家重点保护的珍贵、濒危野生动物而非法收购，其

行为已构成非法收购珍贵、濒危野生动物罪；蓝某欣明知穿山甲系国家重点保护的珍贵、濒危野生动物而非法出售，其行为构成了非法出售珍贵、濒危野生动物罪。公诉机关指控的罪名成立，予以支持。鉴于被告人李某暖、蓝某欣归案后均能自愿认罪认罚，如实供述自己的罪行，并且自愿交纳赔偿款及罚金，被告人蓝某欣还退清了违法所得款，二人均有悔罪表现，依法可以从轻处罚。被告人李某暖、蓝某欣的上述犯罪行为破坏野生动物资源，损害国家和社会公共利益，应当承担相应的民事责任。附带民事公益诉讼起诉人要求二被告人赔偿其因非法收购、出售珍贵、濒危野生动物行为破坏生态环境损失的整体价值人民币 40000 元及被告人李某暖、蓝某欣在全国公开发行的报纸上发表赔礼道歉声明的诉讼请求，符合法律规定，本院予以支持。被告人李某暖的辩护人及被告人蓝某欣的辩护人分别请求给予二被告人从轻处罚的辩护意见，理由充分，本院予以采纳。公诉机关提出的量刑建议，合理、恰当，本院予以采纳。依照《中华人民共和国刑法》第三百四十一条第一款、第三十六条、第五十二条、第六十四条、第六十七条第三款、第七十二条、第七十三条第二、三款、《最高人民法院关于审理破坏野生动物资源刑事案件具体应用法律若干问题的解释》第一条、第二条、《中华人民共和国民事诉讼法》第五十五条第二款、《中华人民共和国侵权责任法》第四条、第八条、第十四条、第十五条和《最高人民法院、最高人民检察

院关于检察公益诉讼案件适用法律若干问题的解释》第二十条之规定，判决如下：

一、被告人李某暖犯非法收购珍贵、濒危野生动物罪，判处有期徒刑八个月,缓刑一年，并处罚金人民币 2000 元，上缴国库。

（缓刑考验期限从判决确定之日起计算；罚金在被告人李某暖的预交款中缴纳）

二、被告人蓝某欣犯非法出售珍贵、濒危野生动物罪，判处有期徒刑八个月,缓刑一年，并处罚金人民币 2000 元，上缴国库。

（缓刑考验期限从判决确定之日起计算；罚金在被告人蓝某欣的预交款中缴纳）

三、被告人蓝某欣退交的违法所得款人民币 8250 元，依法没收，上缴国库（该款从被告人蓝某欣的预交款中缴纳）。

四、随案移送的被告人李某暖的私人物品 iPhone6s 手机一部，依法发还被告人李某暖。

五、被告人李某暖、蓝某欣应于本判决发生法律效力之日起十日内赔偿破坏生态环境损失的整体价值人民币 40000 元，上缴国库（该款从二被告人预交款中缴纳）。

六、被告人李某暖、蓝某欣应于本判决发生法律效力之日起十日内在全国公开发行的报纸上发表经大埔县人民检察院认可的赔礼道歉声明。

如不服本判决，可在接到判决书的第二日起十日内，通过本院或者直接向广东省梅州市中级人民法院提出上诉。书面上诉的，应当提交上诉状正本一份，副本二份。

审　判　长　　许国键

审　判　员　　赵敏如

审　判　员　　黄建年

人民陪审员　　李陈邑

人民陪审员　　何云鹏

人民陪审员　　张国兴

人民陪审员　　杨素琴

二〇一九年九月二十六日

书　记　员　　刘永煦
