# Supplementary material for: China’s Legal Protection System for Pangolins: Past, Present, and Future
Source: Animals (Basel). 2025 Aug 18;15(16):2422. doi: 10.3390/ani15162422 (PMC12383201; doi:10.3390/ani15162422)
Supplement: Supplementary file 1 [file animals-15-02422-s001.zip › Supplementary Material S4-Full Text of Judgments in Pangolin-Related Public Interest Litigation Cases in China/【4】谭某廖某1等危害珍贵濒危野生动物罪等刑事一审刑事判决书.pdf]

广东省广宁县人民法院
刑 事 附 带 民 事 判 决 书

（2023）粤1223刑初80号

公诉机关暨附带民事公益诉讼起诉人广宁县人民检察院。

被告人暨附带民事公益诉讼被告谭某，男，1962年5月3日出生，广东省四会市人，初中文化，住户籍地广东省肇庆市四会市。因本案于2022年8月5日被刑事拘留，于同年9月8日被逮捕，于同年11月24日被决定取保候审。

辩护人暨委托诉讼代理人：高文，广东圣禾律师事务所律师。

被告人暨附带民事公益诉讼被告廖某1，男，1957年10月7日出生，广东省珠海市香洲区人，大专学历，户籍所在地广东省珠海市香洲区，现住址广东省广宁县。于2022年8月5日被刑事拘留，于同年9月8日被逮捕，于同年11月24日取保候审。

辩护人暨委托诉讼代理人：李锦格，广东护衡律师事务所律师。

被告人暨附带民事公益诉讼被告廖某2，男，1983年4月24日出生，澳门特别行政区人，初中文化，户籍地澳门特别行政区XX，现住址广东省珠海市。因本案于2022年8月4日被抓获，于同日被刑事拘留，于同年9月8日被逮捕，于同年11月24日被取保候审。

辩护人暨委托诉讼代理人：欧琅，广东祥麟律师事务所律师。

广宁县人民检察院以宁检刑诉〔2023〕70号起诉书指控被告人谭某、廖某1、廖某2犯危害珍贵、濒危野生动物罪，于2023年5月10日向本院提起公诉。公益诉讼起诉人广宁县人

民检察院于同日以宁检刑附民公诉〔2023〕1号刑事附带民事公益诉讼起诉书向本院提起附带生态环境公益诉讼。经查，广宁县人民检察院于2022年11月14日公告了案件相关情况，公告期内未有法律规定的机关和有关组织提起民事公益诉讼。本院受理后依法组成合议庭，于2023年7月24日公开开庭进行了审理。广宁县人民检察院指派检察员冯远出庭履行职务，被告人暨附带民事公益诉讼被告谭某、廖某1、廖某2及其辩护人暨某诉讼代理人高文、李锦格、欧琅到庭参加诉讼。本案现已审理终结。

公诉机关指控，2021年10月至2022年1月期间，被告人谭某通过被告人廖某1和被告人廖某2向境外人员“阿海”、“小阮”购买羚羊角、穿山甲鳞片、犀牛角等野生动物制品。其中，被告人廖某1负责与“阿海”、“小阮”联系下单，并将收到的被告人谭某预付的货款转账给被告人廖某2，再由被告人廖某2按照报价转账支付货款。“阿海”、“小阮”等人收到货款后，通过“季德容”等人从广西壮族自治区贵港市、崇左市等地将野生动物制品物品以快递方式寄出到被告人廖某1位于广东省广宁县××的住址处，由被告人廖某1收取快递后，将野生动物制品交给被告人谭某。被告人谭某将上述购得的野生动物制品部分用于入药使用。

2022年1月12日，广宁县公安局森林警察大队民警在广宁县潭布镇快递点，将正在收取快递的被告人廖某1当场抓获，并从其收取的快递内搜查出1块疑似犀牛角制品。同年8月4日，广宁县公安局森林警察大队民警在被告人谭某驾驶的粤ＨＷ××**号牌汽车扶手箱内搜查、扣押疑似穿山甲鳞片3块，在其位于广东省四会市××住宅内，搜查、扣押疑似穿山甲鳞片4袋、疑似穿山甲鳞片1瓶、疑似穿山甲鳞片1包、疑

似羚羊角1根、疑似野生羊属动物角1根、疑似牦牛角8段、疑似羊角丝1瓶、疑似羊角粉1瓶。

经聘请华南动物物种环境损害司法鉴定中心鉴定，送检的1块犀牛角制品，净重175克，为哺乳纲奇蹄目犀科白犀的角制品，价值43750元，列入《濒危野生动植物种国际贸易公约》附录一；送检的疑似羚羊角，净重220克，为高鼻羚羊角，价值20000元，列入《濒危野生动植物种国际贸易公约》附录二，同时属我国一级野生保护动物；送检鳞片3袋，共净重3030克，为马来穿山甲鳞片，价值为169680元；送检的鳞片3袋1285克，为树穿山甲鳞片，价值为71960元；送检的三种混合鳞片1袋和鳞片3片，为马来穿山甲、树穿山甲、大穿山甲三种混合鳞片和大穿山甲鳞片，共净重389克，价值3300.61元。涉案的马来穿山甲、树穿山甲、大穿山甲均列入《濒危野生动植物种国际贸易公约》附录一，其中马来穿山甲同时属国家一级保护野生动物。送检的角类制品共584克，其中7段为家牛组织制品，1段为山羊组织制品，1段无法确定其来源物种；1瓶为山羊丝制品；1瓶为绵羊角粉。家牛、山羊、绵羊不属于我国保护动物。根据上述司法鉴定计算，涉案野生动物制品价值分别为5千克的马来穿山甲鳞片价值280000元、1根羚羊角价值20000元、1块犀牛角价值43750元，合计343750元。

2022年10月28日，被告人廖某2家属代其退出违法所得119221元。

公诉机关根据犀牛角、穿山甲鳞片等物证、快递查询单等书证、证人温某等人的证言、被告人谭某、廖某1、廖某2的供述与辩解、鉴定意见、勘验、检查、辨认笔录、电子数据等证据，认为被告人谭某、廖某1、廖某2无视国家法律，非法

收购国家重点保护的珍贵、濒危野生动物制品，其行为触犯了《中华人民共和国刑法》第三百四十一条第一款的规定，应当以危害珍贵、濒危野生动物罪追究其刑事责任。被告人谭某、廖某1、廖某2均认罪认罚，依据《中华人民共和国刑事诉讼法》第十五条的规定，可以从宽处理。同时提出对被告人谭某、廖某1、廖某2均判处有期徒刑三年，缓刑四年，并处罚金人民币10000元的量刑建议。

附带民事公益诉讼起诉人广宁县人民检察院向本院提出诉讼请求：责令三被告共同承担野生动物资源损失人民币348450.61元，并互负连带责任。诉称的事实与理由与刑事部分指控的事实基本一致。

被告人暨附带民事公益诉讼被告谭某对起诉书指控的犯罪事实和罪名及刑事附带民事公益诉讼请求及事实理由均无异议，并自愿签字具结；其辩护人高文提出如下辩护意见：对公诉机关指控的罪名及认定的事实，同意被告人谭某的意见。被告人谭某具有以下依法可以减轻、从轻或酌定从轻处罚的情节：被告人谭某如实供述自己的犯罪事实、认罪态度好、有悔罪表现、无犯罪前科、系初犯、主观恶性不大、客观上没有造成恶劣的社会后果。建议对被告人谭某量刑处罚时参照公诉机关的量刑建议减轻处罚，并充分考虑适用缓刑。对于附带民事公益诉讼部分，按照法庭查明的事实，野生动物制品通过境外人员购入，该制品大程度属于境外的野生动物，本案的野生动物制品是否属于我国相关民事法律保护的野生动物，请法庭考虑。被告人所使用的制品，用途都是用于治疗、入药，根据本案证据显示，并无获利。请法庭对其酌情从轻。

被告人暨附带民事公益诉讼被告廖某1对起诉书指控的犯罪事实和罪名及刑事附带民事公益诉讼的诉讼请求及事实理由

均无异议，并自愿签字具结；其辩护人李锦格提出如下辩护意见：被告人廖某1无犯罪前科、系初犯、归案后如实供述自己的罪行、认罪认罚、在本案中未实施非法猎捕、杀害造成动物死亡的行为，也未造成动物、动物制品无法追回、法律意识较为淡薄的量刑情节。恳请对被告人廖某1从轻、从宽处罚，给予廖某1改过自身、重新做人的机会。对于附带民事公益诉讼部分，被告人的犯罪行为中没有捕猎行为，没有销售获利，应按照造成的损失进行补偿，并明确各被告人应负担的数额。

被告人暨附带民事公益诉讼被告廖某2对起诉书指控的犯罪事实和罪名及刑事附带民事公益诉讼请求及事实理由均无异议，并自愿签字具结；其辩护人欧琅提出如下辩护意见：对起诉书指控被告人廖某2犯危害珍贵、濒危野生动物罪没有异议，但有以下法定、酌定从轻处罚的情节：被告人廖某2归案后如实供述自己的罪行、认罪认罚、积极退赃，可以从轻处罚。请求合议庭能够酌情对被告人廖某2从轻处罚，且在量刑时适用缓刑，让他有改过自新、重新做人的机会。对于附带民事公益诉讼部分，本案购买的动物制品是境外购入，相对国内的制品损失较低，被告人仅起到介绍、牵线的作用，犯罪程度较轻。

经审理查明，2021年10月至2022年1月期间，被告人谭某通过被告人廖某1和被告人廖某2向境外人员“阿海”、“小阮”购买羚羊角、穿山甲鳞片、犀牛角等野生动物制品。其中，被告人廖某1负责与“阿海”、“小阮”联系下单，并将收到的被告人谭某预付的货款转账给被告人廖某2，再由被告人廖某2按照报价转账支付货款。“阿海”、“小阮”等人收到货款后，通过“季德容”等人从广西壮族自治区贵港市、崇左市等地将野生动物制品物品以快递方式寄出到被告人廖某

1 位于广东省广宁县××的住址处，由被告人廖某 1 收取快递后，将野生动物制品交给被告人谭某。被告人谭某将上述购得的野生动物制品部分用于入药使用。

2022 年 1 月 12 日，广宁县公安局森林警察大队民警在广宁县潭布镇快递点，将正在收取快递的被告人廖某 1 当场抓获，并从其收取的快递内搜查出 1 块疑似犀牛角制品。同年 8 月 4 日，广宁县公安局森林警察大队民警在被告人谭某驾驶的粤ＨＷ××**号牌汽车扶手箱内搜查、扣押疑似穿山甲鳞片 3 块，在其位于广东省四会市××住宅内，搜查、扣押疑似穿山甲鳞片 4 袋、疑似穿山甲鳞片 1 瓶、疑似穿山甲鳞片 1 包、疑似羚羊角 1 根、疑似野生羊属动物角 1 根、疑似牦牛角 8 段、疑似羊角丝 1 瓶、疑似羊角粉 1 瓶。

经聘请华南动物物种环境损害司法鉴定中心鉴定，送检的 1 块犀牛角制品，净重 175 克，为哺乳纲奇蹄目犀科白犀的角制品，价值 43750 元，列入《濒危野生动植物种国际贸易公约》附录一；送检的疑似羚羊角，净重 220 克，为高鼻羚羊角，价值 20000 元，列入《濒危野生动植物种国际贸易公约》附录二，同时属我国一级野生保护动物；送检鳞片 3 袋，共净重 3030 克，为马来穿山甲鳞片，价值为 169680 元；送检的鳞片 3 袋 1285 克，为树穿山甲鳞片，价值为 71960 元；送检的三种混合鳞片 1 袋和鳞片 3 片，为马来穿山甲、树穿山甲、大穿山甲三种混合鳞片和大穿山甲鳞片，共净重 389 克，价值 3300.61 元。涉案的马来穿山甲、树穿山甲、大穿山甲均列入《濒危野生动植物种国际贸易公约》附录一，其中马来穿山甲同时属国家一级保护野生动物。送检的角类制品共 584 克，其中 7 段为家牛组织制品，1 段山羊组织制品，1 段无法确定其来源物种；1 瓶为山羊丝制品；1 瓶为绵羊角粉。家牛、山羊、绵

羊不属于我国保护动物。根据上述司法鉴定计算，涉案野生动物制品价值为308690.61元。

2022年10月28日，被告人廖某2家属代其退出119221元。本案审理过程中，被告人谭某缴付197635.61元。

另查明，被告人廖某2在代为购买涉案野生动物制品获利8166元。四会市司法局、广宁县司法局分别对被告人谭某、廖某1的社会调查结果未发现对所居住社区有重大不良影响。

上述事实，被告人在开庭审理过程中亦无异议，并有经法庭质证和认证的疑似犀牛角1块、ＯＰＰＯ牌手机1台（廖某1身上搜查、扣押）、疑似穿山甲鳞片3块（谭某粤ＨＷ××**传祺牌白色汽车）、华为牌手机1台、疑似穿山甲鳞片4袋、疑似穿山甲鳞片1瓶、疑似穿山甲鳞片1包、疑似羚羊角1根、疑似野生羊属动物角1根、疑似牦牛角8段、疑似羊角丝1瓶、疑似羊角粉1瓶（谭某身上及住处）、ＩＰＨＯＮＥ牌手机2台（廖某2）、ＯＰＰＯ牌手机2台（廖某1）、疑似动物角粉1袋、温某随身携带的ＯＰＰＯ牌手机1台、ＨＯＮＯＲ牌手机1台（四会苗圃场）、搜查证、搜查笔录、扣押清单、扣押决定书、发还清单、指认说明、人民币119221元（廖某2退出的违法所得）等物证、广东省暂时扣留、冻结财物收据、现金缴款单、被告人照片、户籍信息、港澳居民来往内地通行证信息、无犯罪证明、归案情况说明、银行账户交易流水明细、协助查询财产通知书、电子数据提取说明、支付宝注册信息和交易流水、手机通话记录、调取证据通知书、快递物流信息查询结果、涉案人员说明、微信聊天记录及截图指认、社会调查委托函、调查评估意见书、证人温某、李某1、徐某、李某2的证言、被告人谭某、廖某1、廖某2的供述与辩解、华南动物物种环境损害司法鉴定中心司法鉴定意见及相关涉案

动物制品价值说明、现场勘验工作记录、电子证物检查工作记录、公告等证据证实，足以认定。

本院认为，被告人谭某、廖某1、廖某2无视国家法律，非法收购、出售国家重点保护的珍贵、濒危野生动物制品，价值达30多万元，破坏环境资源保护秩序，其行为已构成危害珍贵、濒危野生动物罪，应依法惩处。公诉机关的指控成立。被告人谭某、廖某1、廖某2收购的动物制品已经追回，自愿全部退赃退赔，悔罪态度好，对其可在有期徒刑五年以下或者拘役的法定刑幅度内量刑；被告人谭某、廖某1、廖某2退出其违法犯罪所得、认罪认罚，依法可以从宽处罚。公诉机关的量刑建议适当。辩护人高文、李锦格、欧琅分别提出被告人有坦白情节、自愿认罪认罚、没有犯罪前科、系初犯等辩护意见，经查基本属实，本院予以采纳。由于经鉴定谭某、廖某1、廖某2的犯罪行为造成的野生动物资源价值损失为308690.61元，因此，对于附带民事公益诉讼起诉人广宁县人民检察院主张被告人谭某、廖某1、廖某2共同赔偿野生动物资源损失人民币343750元的诉讼请求，于法有据部分，本院予以支持，于法无据部分，本院不予支持。对诉讼代理人高文提出涉案野生动物制品属于境外的野生动物是否属于我国相关民事法律相关保护的野生动物的意见，诉讼代理人李锦格提出本案没有捕猎和获利，应按照造成的损失确定各被告人应负的赔偿责任的意见和诉讼代理人欧琅提出本案购买的动物制品是境外购入，相对国内的制品损失较低，且廖某2仅起到介绍、牵线的作用，犯罪程度较轻的意见，经查，我国是《濒危野生动植物种国际贸易公约》缔约国，根据林业部《关于核准部分濒危野生动物为国家重点保护野生动物的通知》的规定，上述公约附录Ⅰ、附录Ⅱ所列非原产我国的所有野生动物，分别核准为国家一级

和国家二级保护野生动物，而案涉的境外野生动物已被核准为国家一级或者国家二级野生动物，受到我国相关法律法规的保护，其野生动物活体或其制品进入我国境内时，应按照相关法律法规予以处理；况且杀害野生动物，然后出售、运输、加工、收购其制品，是一个非法牟利的链条，正是市场的需求存在才造成大量濒危野生动物被杀害，正所谓“没有买卖就没有杀害”，因此，附带公益诉讼被告谭某、廖某 1、廖某 2 预谋共同实施的犯罪行为，直接导致或者放纵濒危野生动物被杀害，破坏了整个生态系统的平衡与稳定，侵害环境资源公共利益，应承担连带侵权责任，则本院对上述意见均不予采纳。

综上所述，根据被告人的犯罪事实、性质、情节和对社会的危害程度，依照《中华人民共和国刑法》第三百四十一条第一款、第六十七条第三款、第六十四条、第七十二条，《中华人民共和国民法典》第一百七十九条第一款第八项、第一百八十七条、第一千一百六十八条、第一千二百二十九条、第一千二百三十五条，《中华人民共和国野生动物保护法》第二条、第二十八条、第三十七条、第六十二条、《中华人民共和国环境保护法》第六十四条，《中华人民共和国刑事诉讼法》第十五条、第一百零一条、《中华人民共和国民事诉讼法》第五十八条第二款、《最高人民法院、最高人民检察院关于办理破坏野生动物资源刑事案件适用法律若干问题的解释》第四条、第六条第三款、《最高人民法院、最高人民检察院关于检察公益诉讼案件适用法律若干问题的解释》第二十条的规定，判决如下：

一、被告人谭某犯危害珍贵、濒危野生动物罪，判处有期徒刑三年，缓刑四年，并处罚金人民币一万元。

（缓刑考验期限，从判决确定之日起计算。）

二、被告人廖某1犯危害珍贵、濒危野生动物罪，判处有期徒刑三年，缓刑四年，并处罚金人民币一万元。

（缓刑考验期限，从判决确定之日起计算。）

三、被告人廖某2犯危害珍贵、濒危野生动物罪，判处有期徒刑三年，缓刑四年，并处罚金人民币一万元。

（缓刑考验期限，从判决确定之日起计算。）

四、被告人廖某2退出的违法所得8166元，予以追缴，由扣押机关负责上缴国库。

五、扣押机关扣押的羚羊角一根、犀牛角一块、穿山甲鳞片八袋，由扣押机关依法处理；扣押机关扣押的谭某的华为牌手机一台、廖某1的OPPO手机二台、廖某2的IPHONE牌手机二台、山羊角丝1瓶、绵阳角粉1瓶、动物角属组织制品9段，由扣押机关在本判决发生法律效力后发还给各被告人。

六、附带民事诉讼被告谭某、廖某1、廖某2应在本判决生效之日起十日内赔偿野生动物资源损失人民币308690.61元，上缴国库（其中111055元由扣押机关上缴国库，余下197635.61元由本院上缴国库）。

如不服本判决，可在接到判决书的第二日起十日内，通过本院或者直接向广东省肇庆市中级人民法院提出上诉。书面上诉的，应当提交上诉状正本一份，副本二份。

审　判　长　　梁建华
审　判　员　　郑焕月
审　判　员　　曾洁梅
人民陪审员　　陈庆鑫
人民陪审员　　卢海婵
人民陪审员　　黄丽娟
人民陪审员　　曾丽云

二〇二三年八月十一日
法官　助理　　周施好
书　记　员　　何奕宽

附相关法律条文：

《中华人民共和国刑法》

第六十四条犯罪分子违法所得的一切财物，应当予以追缴或者责令退赔；对被害人的合法财产，应当及时返还；违禁品和供罪犯所用的本人财物，应当予以没收。没收的财物和罚金，一律上缴国库，不得挪用和自行处理。

第六十七条犯罪以后自动投案，如实供述自己的罪行的，是自首。对于自首的犯罪分子，可以从轻或者减轻处罚。其中，犯罪较轻的，可以免除处罚。

被采取强制措施的犯罪嫌疑人、被告人和正在服刑的罪犯，如实供述司法机关还未掌握的本人其他罪行的，以自首论。

犯罪嫌疑人虽不具有前两款规定的自首情节，但是如实供述自己罪行的，可以从轻处罚；因其如实供述自己罪行，避免特别严重后果发生的，可以减轻处罚。

第七十二条对于被判处拘役、三年以下有期徒刑的犯罪分子，同时符合下列条件的，可以宣告缓刑，对其中不满十八周岁的人、怀孕的妇女和已满七十五周岁的人，应当宣告缓刑：

（一）犯罪情节较轻；

（二）有悔罪表现；

（三）没有再犯罪的危险；

（四）宣告缓刑对所居住社区没有重大不良影响。

宣告缓刑，可以根据犯罪情况，同时禁止犯罪分子在缓刑考验期限内从事特定活动，进入特定区域、场所，接触特定的

人。被宣告缓刑的犯罪分子，如果被判处附加刑，附加刑仍须执行。

第三百四十一条非法猎捕、杀害国家重点保护的珍贵、濒危野生动物的，或者非法收购、运输、出售国家重点保护的珍贵、濒危野生动物及其制品的，处五年以下有期徒刑或者拘役，并处罚金；情节严重的，处五年以上十年以下有期徒刑，并处罚金；情节特别严重的，处十年以上有期徒刑，并处罚金或者没收财产。

违反狩猎法规，在禁猎区、禁猎期或者使用禁用的工具、方法进行狩猎，破坏野生动物资源，情节严重的，处三年以下有期徒刑、拘役、管制或者罚金。

违反野生动物保护管理法规，以食用为目的非法猎捕、收购、运输、出售第一款规定以外的在野外环境自然生长繁殖的陆生野生动物，情节严重的，依照前款的规定处罚。

《中华人民共和国民法典》

第一百七十九条承担民事责任的方式主要有：

（一）停止侵害；

（二）排除妨碍；

（三）消除危险；

（四）返还财产；

（五）恢复原状；

（六）修理、重作、更换；

（七）继续履行；

（八）赔偿损失；

（九）支付违约金；

（十）消除影响、恢复名誉；

（十一）赔礼道歉。

法律规定惩罚性赔偿的，依照其规定。

本条规定的承担民事责任的方式，可以单独适用，也可以合并适用。

第一百八十七条民事主体因同一行为应当承担民事责任、行政责任和刑事责任的，承担行政责任或者刑事责任不影响承担民事责任；民事主体的财产不足以支付的，优先用于承担民事责任。

第一千二百二十九条因污染环境、破坏生态造成他人损害的，侵权人应当承担侵权责任。

第一千一百六十八条二人以上共同实施侵权行为，造成他人损害的，应当承担连带责任。

第一千二百三十五条违反国家规定造成生态环境损害的，国家规定的机关或者法律规定的组织有权请求侵权人赔偿下列损失和费用：

（一）生态环境受到损害至修复完成期间服务功能丧失导致的损失；

（二）生态环境功能永久性损害造成的损失；

（三）生态环境损害调查、鉴定评估等费用；

（四）清除污染、修复生态环境费用；

（五）防止损害的发生和扩大所支出的合理费用。

《中华人民共和国野生动物保护法》

第二条在中华人民共和国领域及管辖的其他海域，从事野生动物保护及相关活动，适用本法。

本法规定保护的野生动物，是指珍贵、濒危的陆生、水生野生动物和有重要生态、科学、社会价值的陆生野生动物。

本法规定的野生动物及其制品，是指野生动物的整体（含卵、蛋）、部分及衍生物。

珍贵、濒危的水生野生动物以外的其他水生野生动物的保护，适用《中华人民共和国渔业法》等有关法律的规定。

第二十八条禁止出售、购买、利用国家重点保护野生动物及其制品。

因科学研究、人工繁育、公众展示展演、文物保护或者其他特殊情况，需要出售、购买、利用国家重点保护野生动物及其制品的，应当经省、自治区、直辖市人民政府野生动物保护主管部门批准，并按照规定取得和使用专用标识，保证可追溯，但国务院对批准机关另有规定的除外。

出售、利用有重要生态、科学、社会价值的陆生野生动物和地方重点保护野生动物及其制品的，应当提供狩猎、人工繁育、进出口等合法来源证明。

实行国家重点保护野生动物和有重要生态、科学、社会价值的陆生野生动物及其制品专用标识的范围和管理办法，由国务院野生动物保护主管部门规定。

出售本条第二款、第三款规定的野生动物的，还应当依法附有检疫证明。

利用野生动物进行公众展示展演应当采取安全管理措施，并保障野生动物健康状态，具体管理办法由国务院野生动物保护主管部门会同国务院有关部门制定。

第三十七条中华人民共和国缔结或者参加的国际公约禁止或者限制贸易的野生动物或者其制品名录，由国家濒危物种进出口管理机构制定、调整并公布。

进出口列入前款名录的野生动物或者其制品，或者出口国家重点保护野生动物或者其制品的，应当经国务院野生动物保护主管部门或者国务院批准，并取得国家濒危物种进出口管理

机构核发的允许进出口证明书。海关凭允许进出口证明书办理进出境检疫，并依法办理其他海关手续。

涉及科学技术保密的野生动物物种的出口，按照国务院有关规定办理。

列入本条第一款名录的野生动物，经国务院野生动物保护主管部门核准，按照本法有关规定进行管理。

第六十二条县级以上人民政府野生动物保护主管部门应当加强对野生动物及其制品鉴定、价值评估工作的规范、指导。本法规定的猎获物价值、野生动物及其制品价值的评估标准和方法，由国务院野生动物保护主管部门制定。

《中华人民共和国环境保护法》

第六十四条因污染环境和破坏生态造成损害的,应当依照《中华人民共和国侵权责任法》的有关规定承担侵权责任。

《中华人民共和国民事诉讼法》

第五十八条对污染环境、侵害众多消费者合法权益等损害社会公共利益的行为，法律规定的机关和有关组织可以向人民法院提起诉讼。

人民检察院在履行职责中发现破坏生态环境和资源保护、食品药品安全领域侵害众多消费者合法权益等损害社会公共利益的行为，在没有前款规定的机关和组织或者前款规定的机关和组织不提起诉讼的情况下，可以向人民法院提起诉讼。前款规定的机关或者组织提起诉讼的，人民检察院可以支持起诉。

《中华人民共和国刑事诉讼法》

第十五条犯罪嫌疑人、被告人自愿如实供述自己的罪行，承认指控的犯罪事实，愿意接受处罚的，可以依法从宽处理。

第一百零一条被害人由于被告人的犯罪行为而遭受物质损失的,在刑事诉讼过程中,有权提起附带民事诉讼。被害人死亡

或者丧失行为能力的,被害人的法定代理人、近亲属有权提起附带民事诉讼。

如果是国家财产、集体财产遭受损失的,人民检察院在提起公诉的时候,可以提起附带民事诉讼。

《最高人民法院、最高人民检察院关于办理破坏野生动物资源刑事案件适用法律若干问题的解释》

第四条刑法第三百四十一条第一款规定的“国家重点保护的珍贵、濒危野生动物”包括:

(一)列入《国家重点保护野生动物名录》的野生动物;

(二)经国务院野生动物保护主管部门核准按照国家重点保护的野生动物管理的野生动物。

第六条非法猎捕、杀害国家重点保护的珍贵、濒危野生动物,或者非法收购、运输、出售国家重点保护的珍贵、濒危野生动物及其制品,价值二万元以上不满二十万元的,应当依照刑法第三百四十一条第一款的规定,以危害珍贵、濒危野生动物罪处五年以下有期徒刑或者拘役,并处罚金;价值二十万元以上不满二百万元的,应当认定为“情节严重”,处五年以上十年以下有期徒刑,并处罚金;价值二百万元以上的,应当认定为“情节特别严重”,处十年以上有期徒刑,并处罚金或者没收财产。

实施前款规定的行为,具有下列情形之一的,从重处罚:

(一)属于犯罪集团的首要分子的;

(二)为逃避监管,使用特种交通工具实施的;

(三)严重影响野生动物科研工作的;

(四)二年内曾因破坏野生动物资源受过行政处罚的。

实施第一款规定的行为，不具有第二款规定的情形，且未造成动物死亡或者动物、动物制品无法追回，行为人全部退赃退赔，确有悔罪表现的，按照下列规定处理：

（一）珍贵、濒危野生动物及其制品价值二百万元以上的，可以认定为“情节严重”，处五年以上十年以下有期徒刑，并处罚金；

（二）珍贵、濒危野生动物及其制品价值二十万元以上不满二百万元的，可以处五年以下有期徒刑或者拘役，并处罚金；

（三）珍贵、濒危野生动物及其制品价值二万元以上不满二十万元的，可以认定为犯罪情节轻微，不起诉或者免予刑事处罚；情节显著轻微危害不大的，不作为犯罪处理。

《最高人民法院、最高人民检察院关于检察公益诉讼案件适用法律若干问题的解释》

第二十条人民检察院对破坏生态环境和资源保护、食品药品安全领域侵害众多消费者合法权益等损害社会公共利益的犯罪行为提起刑事公诉时,可以向人民法院一并提起附带民事公益诉讼,由人民法院同一审判组织审理。

人民检察院提起的刑事附带民事公益诉讼案件由审理刑事案件的人民法院管辖。
