# Supplementary material for: China’s Legal Protection System for Pangolins: Past, Present, and Future
Source: Animals (Basel). 2025 Aug 18;15(16):2422. doi: 10.3390/ani15162422 (PMC12383201; doi:10.3390/ani15162422)
Supplement: Supplementary file 1 [file animals-15-02422-s001.zip › Supplementary Material S4-Full Text of Judgments in Pangolin-Related Public Interest Litigation Cases in China/【50】_ 、 非法收购、运输、出售珍贵、濒危野生动物、珍贵、濒危野生动物制品一审刑事判决书(FBM-CLI.C.75230468).pdf]

袁勇、石华非法收购、运输、出售珍贵、濒危野生动物、珍贵、濒危野生动物制品一审刑事判决书

袁勇、石华非法收购、运输、出售珍贵、濒危野生动物、珍贵、濒危野生动物制品一审刑事判决书

云南省元江哈尼族彝族傣族自治县人民法院

刑事附带民事判决书

(2018)云0428刑初148号

公诉机关暨公益诉讼起诉人元江哈尼族彝族傣族自治县人民检察院。

被告人袁勇。因涉嫌非法收购、运输珍贵、濒危野生动物制品罪于2018年1月12日被玉溪市森林公安局取保候审。

辩护人暨特别授权诉讼代理人黄萍，云南澧江律师事务所律师。

被告人石华。因涉嫌非法收购、运输珍贵、濒危野生动物制品罪于2018年8月3日被玉溪市森林公安局取保候审。

被告人全爱荣。因涉嫌非法收购、运输珍贵、濒危野生动物制品罪于2018年8月15日被玉溪市森林公安局取保候审。

元江哈尼族彝族傣族自治县人民检察院以元检刑诉[2018]145号起诉书指控被告人袁勇、石华、全爱荣犯非法收购、运输珍贵、濒危野生动物制品罪，于2018年12月18日向本院提起公诉。元江哈尼族彝族傣族自治县人民检察院以公益诉讼起诉人的身份以被告袁勇、石华、全爱荣的犯罪行为导致国家野生动物资源遭到破坏，损害了国家和社会公共利益为由向本院提起公益诉讼。本院依法组成合议庭，公开开庭合并审理了本案。公诉机关暨公益诉讼起诉人元江哈尼族彝族傣族自治县人民检察院指派检察员杨智浩、王蕊、代理检察员杨晓芬、书记员普文龙出庭支持公诉和参加附带民事部分的公益诉讼。辩护人暨附带民事诉讼特别授权代理人黄萍律师出庭为被告人袁勇进行辩护以及附带民事部分的特别授权代理。

公诉机关指控，2018年1月4日，被告人袁勇带其堂弟袁某到西双版纳旅游，被告人石华在西双版纳接待了他俩，期间，袁勇向石华打听在西双版纳能不能买到穿山甲片、大象皮等野生动物制品，

石华就联系了其卖三七的朋友全爱荣。2018年1月5日，石华带袁勇、袁某到西双版纳州勐海县找全爱荣商谈购买穿山甲片、大象皮等野生动物制品事宜。2018年1月6日，全爱荣通过微信与袁某联系，告知货已联系好，每袋一斤，总价为15500元。后袁勇安排袁某用微信代转帐15500元给被告人石华，再由石华转帐15500元给全爱荣。2018年1月9日，全爱荣在缅甸小勐拉以现金15500元跟“老缅”购得疑似穿山甲粉末8袋（净重3.98409千克）、疑似大象皮粉末6袋（净重2.94949千克）、疑似豪猪刺粉末3袋（净重1.48937千克）。后全爱荣填写邮寄单，并在邮寄的三个袋子上分别贴上“仙”、“太”、“者”的字样。并编写了内容为“你好，有三包，仙就是山，是穿山甲，太就是大，是大象皮，者就是猪，是刺猪毛”的信息连同邮寄单照片一并用微信发给了袁某。在涉案物品邮寄的过程中，被公安机关查获。经云南濒科委司法鉴定中心鉴定，检材FSS01698（疑似穿山甲片粉末）与FSS01700（疑似豪猪刺粉末）来源未知；送检检材FSS01699（疑似大象皮粉末）来源于长鼻目象科亚洲象属亚洲象Elephasmaximus。亚洲象为国家Ⅰ级保护动物，列入《濒危野生动植物种国际贸易公约》（CITES）附录Ⅰ。涉案价值为14747元（即：壹万肆仟柒佰肆拾柒元整）。

公益诉讼起诉人元江哈尼族彝族傣族自治县人民检察院提出，被告袁勇、石华、全爱荣非法购买、运输国家重点保护野生动物制品，导致国家的野生动物资源遭到破坏，损害了国家和社会公共利益，应承担赔偿损失和赔礼道歉的民事责任。请求：一、判令被告袁勇、石华、全爱荣赔偿国家野生动物资源损失14747元；二、判令被告袁勇、石华、全爱荣在新闻媒体公开赔礼道歉。

被告人袁勇提出，对公诉机关指控的事实和罪名无异议，其自身患有多种疾病，购买指控的野生动物制品是为了治病。对于公益诉讼起诉人要求赔偿国家野生动物资源损失14747元的请求及赔礼道歉无异议，其愿意个人赔偿涉案价值款14747元，已于开庭前向法院预交。

辩护人暨附事民事诉讼特别授权代理人黄萍提出，对起诉书指控的事实与罪名无异议，被告人袁勇购买、运输野生动物制品是为了他本人治病，并无违法故意。被告人袁勇具有坦白、初犯、偶犯、有悔罪表现，其个人愿意赔偿涉案价值款14747元，并已开庭前主动向法院预交。被告人袁勇与被告人石华、全爱荣在共同犯罪中作用相当，不宜划分主从犯，建议判处被告人袁勇有期徒刑三年以下，并对其适用缓刑。对公益诉讼起诉人要求赔偿和赔礼道歉的请求无异议。

被告人石华、全爱荣提出，对起诉书指控的事实与罪名无异议，对公益诉讼起诉人要求赔偿和赔礼道歉的请求无异议，对被告袁勇愿意个人赔偿涉案价值款14747元无异议。

经审理查明，被告人袁勇因身患多种疾病欲购买大象皮、穿山甲片用于治疗疾病。2018年1月4日，被告人袁勇带其堂弟袁某到西双版纳找到被告人石华，让其帮忙购买大象皮、穿山甲片等野生动物制品用于治病。被告人石华又联系了被告人全爱荣帮忙购买。2018年1月5日，被告人石华带袁勇、袁某到西双版纳州勐海县找到被告人全爱荣商谈购买大象皮、穿山甲片、刺猪毛野生动物制品事宜。2018年1月6日，被告人全爱荣通过微信与袁某联系，告知货已联系好，每袋一斤，总价为15500元。后被告人袁勇让袁某用微信代某转帐15500元给被告人石华，被告人石华又向被告人全爱荣转帐15500元。2018年1月9日，被告人全爱荣在缅甸小勐拉以人民币15500元购得疑似穿山甲片粉末8袋、疑似大象皮粉末6袋、疑似豪猪刺粉末3袋。后被告人全爱荣将购买的以上野生动物制品交给勐海县邮政快递邮寄给被告人袁勇提供的地址和收货人，并在邮寄的三个袋子上分别贴上“仙”、“太”、“者”的字样。还编写了内容为“你好，有三包，仙就是山，是穿山甲，太就是大，是大象皮，者就是猪，是刺猪毛”的信息连同邮寄单照片一并用微信发给了袁某。同日，被告人袁勇与袁某驾驶车辆从景洪返回成都，途经元江县青龙厂路段时被民警查获。经称量，疑似穿山甲片粉末8袋，净重3.98409千克，疑似大象皮粉末6袋，净重2.94949千克，疑似豪猪刺粉末3袋，净重1.48937千克。经云南濒科委司法鉴定中心鉴定，检材FSS01698（疑似穿山甲片粉末）与FSS01700（疑似豪猪刺粉末）来源未知；送检检材FSS01699（疑似大象皮粉末）来源于长鼻目象科亚洲象属亚洲象Elephasmaximus。亚洲象为国家Ⅰ级保护动物，列入《濒危野生动植物种国际贸易公约》（CITES）附录Ⅰ。涉案价值为14747元（即：壹万肆仟柒佰肆拾柒元整）。

另查明，2019年1月14日，被告人袁勇向本院预交了涉案价值款14747元。

2019年3月27日9时，公益诉讼起诉人与被告袁勇、石华、全爱荣对赔礼道歉部分已自行商量并履行完毕。

上述事实，被告人袁勇、石华、全爱荣在开庭审理过程中无异议，且有物证照片、户口证明、受案登记表、立案决定书、查获经过、到案经过、情况说明、收据、公开道歉信；证人袁某、向某的证言；被告人袁勇、石华、全爱荣的供述与辩解；辨认笔录及照片、检查笔录及照片、称量笔录及照片、扣押决定书、扣押笔录、扣押照片、扣押清单、随案移送清单；鉴定委托书、鉴定意见、鉴定意见通知书等证据在案证实。

以上证据，经当庭质证认证，证据收集程序合法，证据证明的内容客观真实，且证据之间能相互

印证，足以认定。

本院认为，被告人袁勇、石华、全爱荣违反国家野生动物保护法规的相关规定，非法收购、运输珍贵、濒危野生动物制品，三被告人的行为已构成非法收购、运输珍贵、濒危野生动物制品罪。被告人袁勇、石华、全爱荣在共同犯罪过程中，被告人袁勇起主要作用，是主犯，本院依法处罚。被告人石华、全爱荣起次要作用，是从犯，本院依法从轻处罚。三被告人到案后如实供述犯罪事实，是坦白，依法对三被告人从轻处罚。被告人袁勇自愿赔偿涉案款14747元，本院予以准许，对其酌情从轻处罚。公诉机关指控的事实与罪名、主从犯的划分及认定三被告人具有坦白情节成立，本院予以支持。公益诉讼起诉人要求判令三被告赔偿国家野生动物资源损失14747元符合法律规定，本院予以支持。对于公益诉讼起诉人要求判令三被告在新闻媒体公开赔礼道歉的主张。因公益诉讼起诉人与三被告已对赔礼道歉的诉讼请求自行协商，并已按照公益诉讼起诉人的要求履行完毕，对该项诉讼请求，本院不再判决。辩护人黄萍提出被告人袁勇具有坦白情节，已赔偿野生动物资源损失14747元，应从轻处罚，建议判处有期徒刑三年以下并适用缓刑的辩护意见成立，本院予以采纳。但提出本案不宜划分主从犯，三被告人作用相当的辩护意见，本院不予采纳。据此，依照《中华人民共和国刑法》第三百四十一条、第三十六条、第二十五条、第二十六条、第二十七条、第六十七条第三款、第七十二条、第七十三条、第六十四条，《中华人民共和国刑事诉讼法》第一百零一条第二款、第一百零四条，《中华人民共和国侵权责任法》第六条、第八条、第十四条，《最高人民法院关于适用<中华人民共和国刑事诉讼法>的解释》第一百四十二条第一款、第一百五十五条第一款，《最高人民法院最高人民检察院关于检察公益诉讼案件适用法律若干问题的解释》第二十条，《最高人民法院关于审理环境民事公益诉讼案件适用法律若干问题的解释》第二十条第二款、第二十四条之规定，判决如下：

一、被告人袁勇犯非法收购、运输珍贵、濒危野生动物制品罪，判处有期徒刑一年，缓刑二年，并处罚金人民币20000元（缓刑考验期从判决确定之日起计算）。

二、被告人石华犯非法收购、运输珍贵、濒危野生动物制品罪，判处有期徒刑六个月，缓刑一年，并处罚金人民币10000元（缓刑考验期从判决确定之日起计算）。

三、被告人全爱荣犯非法收购、运输珍贵、濒危野生动物制品罪，判处有期徒刑六个月，缓刑一年，并处罚金人民币10000元（缓刑考验期从判决确定之日起计算）。

四、扣押在案的大象皮粉末6袋（净重2.94949千克）予以没收。

五、由被告袁勇、石华、全爱荣共同赔偿国家野生动物资源损失14747元（被告袁勇已赔偿），缴至元江哈尼族彝族傣族自治县财政局指定的账户，用作保护当地野生动物的经费。

如不服本判决，可在接到判决书的第二日起十日内，通过本院或者直接向云南省玉溪市中级人民法院提出上诉。书面上诉的，应当提交上诉状正本一份，副本二份。

审　判　长　　覃建国

审　判　员　　张志陆

审　判　员　　马艾萍

人民陪审员　　封建昌

人民陪审员　　吕志惠

人民陪审员　　何保辉

人民陪审员　　杨玉福

二〇一九年三月二十七日

书　记　员　　王真臻

©北大法宝：（www.pkulaw.com）专业提供法律信息、法学知识和法律软件领域各类解决方案。北大法宝为您提供丰富的参考资料，正式引用法规条文时请与标准文本核对。 欢迎查看所有产品和服务。

法宝快讯： 如何快速找到您需要的检索结果？ 法宝 V6 有何新特色？

爱法律有未来 PKULAW.COM

扫描二维码阅读原文

原文链接： https://www.pkulaw.com/pfnl/a6bdb3332ec0adc412c8b04346901403989b1c982a267e49bdfb.html
