# Supplementary material for: China’s Legal Protection System for Pangolins: Past, Present, and Future
Source: Animals (Basel). 2025 Aug 18;15(16):2422. doi: 10.3390/ani15162422 (PMC12383201; doi:10.3390/ani15162422)
Supplement: Supplementary file 1 [file animals-15-02422-s001.zip › Supplementary Material S4-Full Text of Judgments in Pangolin-Related Public Interest Litigation Cases in China/【5】陈炫钧东检刑等危害珍贵濒危野生动物罪等刑事一审刑事判决书.pdf]

广西壮族自治区东兴市人民法院
刑 事 附 带 民 事 判 决 书

（2022）桂0681刑初3号

公诉机关暨附带民事公益诉讼起诉人广西壮族自治区东兴市人民检察院。

被告人暨附带民事公益诉讼被告陈炫钧，男，1980年4月25日出生，籍贯广西防城港市防城区，壮族，小学文化，无业，户籍住址防城区，因涉嫌犯危害珍贵、濒危野生动物罪于2021年9月12日被刑事拘留，同年10月18日被逮捕。现羁押于东兴市看守所。

辩护人揭军英，广西新鸿律师事务所律师。

东兴市人民检察院以东检刑诉〔2022〕2号起诉书指控被告人陈炫钧犯危害珍贵、濒危野生动物罪一案，于2022年1月20日向本院提起公诉，并以东检刑附民公诉〔2022〕1号刑事附带民事公益诉讼起诉书就陈炫钧非法运输珍贵、濒危野生动物制品损害社会公共利益的行为向本院提起刑事附带民事公益诉讼。经查，东兴市人民检察院于2021年11月5日公告了该案相关情况，公告期内未有法律规定的机关或有关组织提起民事公益诉讼。本院受理后，依法组成合议庭，于2022年9月16日公开开庭审理了本案。东兴市人民检察院指派检察员骆世俊出庭支持公诉、指派检察员杨媚出庭履行职务，被告人暨附带民事公益诉讼被告陈炫钧及其辩护人揭军英到庭参加诉讼。受疫情影响，本案中止审理一次。本案现已审理终结。

东兴市人民检察院指控：2020年1月，被告人陈炫钧受越南女子“阿玉”（另案处理）指使，帮助管理东兴市××镇××路××号××楼仓库。由于“阿玉”无法入境，陈炫钧一直保管该仓库钥匙，并根据“阿玉”的指示开门给他人放货物进

该仓库。2021 年 9 月 10 日，陈炫钧接收了 2 袋货物；同月 11 日 9 时许，陈炫钧接收了 10 纸箱货物；同日 12 时许，再次接收了 2 袋货物，后其打开袋子、纸箱，发现袋子里面有疑似穿山甲鳞片、纸箱里面有袋装菠萝干。

随后陈炫钧根据“阿玉”的指示将纸箱里面的外包装写有数字和没有数字的货物分好，随后将纸箱内的菠萝干包装袋包装割开，将疑似穿山甲鳞片装进菠萝干包装袋，再将该包装袋粘好。打包了 2 纸箱疑似穿山甲鳞片后，陈炫钧回家吃饭，在返回仓库途中被民警抓获。民警从该仓库内查获 5 袋疑似穿山甲鳞片和纸箱蔬果干包装袋包装的疑似穿山甲鳞片 4 箱。经称量，所扣押的疑似穿山甲鳞片净重为 61.35 千克。经鉴定，所扣押的疑似穿山甲鳞片均为哺乳纲鳞甲目穿山甲科穿山甲属马来穿山甲的鳞片，属于国家一级保护野生动物，被列入《濒危野生动植物种国际贸易公约》（CLTES）附录Ⅰ，经济价值人民币 3435600 元（以下币种均同）。

公诉机关指控上述事实提供了相应的证据予以证明。公诉机关认为，被告人陈炫钧受他人指使，非法运输国家重点保护的珍贵、濒危野生动物制品，情节特别严重，其行为触犯了《中华人民共和国刑法》第三百四十一条第一款之规定，应当以危害珍贵、濒危野生动物罪追究其刑事责任。陈炫钧在共同犯罪中起次要作用，是从犯，应当从轻或者减轻处罚。提请本院依法判处。

附带民事公益诉讼起诉人东兴市人民检察院向本院提出诉讼请求：1.请求判令被告陈炫钧依法承担赔偿生态资源受损费用人民币 3435600 元；2.请求判令被告陈炫钧就其侵权行为当庭进行公开赔礼道歉。

被告人暨附带民事公益诉讼被告陈炫钧辩称："阿玉"叫其去仓库开门给别人放药材，其按照对方的要求打开2小包并拍照，但其不认识也不知道是穿山甲鳞片；其没有参与运输，只是帮"阿玉"放东西；其在微信聊天中发送的疑似穿山甲鳞片及野生动物制品的图片、邮寄地址及快递包裹等是其发着玩的，故其不构成危害珍贵、濒危野生动物罪。其就侵权行为当庭进行公开赔礼道歉，但表示没有义务及能力进行赔偿。

辩护人提出如下辩护意见：陈炫钧在仓库开门给别人放货时不清楚是穿山甲鳞片；其行为只是打包，不是运输；其按照"阿玉"的要求打包了2包穿山甲鳞片，只需对其打包的2包穿山甲鳞片负责，不应该对在该仓库所扣押的全部穿山甲鳞片负责；其是犯罪未遂、从犯、初犯；所扣押的手机是个人物品，应当予以返还。

经审理查明：2020年1月，被告人陈炫钧受越南女子"阿玉"（另案处理）指使，帮助管理广西东兴市××镇××路××号××楼仓库。由于"阿玉"无法入境，陈炫钧一直帮保管该仓库钥匙。

2021年9月10日至11日，陈炫钧按照"阿玉"指示打开该仓库大门并接收了4袋货物及10箱纸箱货物，后其打开袋子、纸箱，发现袋子里是疑似穿山甲鳞片、纸箱里面有袋装菠萝干。随后陈炫钧根据"阿玉"指示将外包装写有数字和没有数字的货物分好以便后续他人过来拉走，并将纸箱内的菠萝干包装袋割开，把疑似穿山甲鳞片装进菠萝干包装袋，再将该包装袋粘好。陈炫钧打包好2箱疑似穿山甲鳞片后回家吃饭，在返回仓库途中被民警抓获。民警从该仓库内查获疑似穿山甲鳞片5袋和用纸箱蔬果干包装袋包装的疑似穿山甲鳞片4箱。经称量，所扣押的疑似穿山甲鳞片净重为61.35千克。经鉴定，

所扣押的疑似穿山甲鳞片均为哺乳纲鳞甲目穿山甲科穿山甲属马来穿山甲的鳞片，属于国家一级保护野生动物，被列入《濒危野生动物植物种国际贸易公约》（CLTES）附录Ⅰ，经济价值3435600元。

上述事实，有公诉机关暨附带民事公益诉讼起诉人提供，并经庭审举证、质证的下列证据予以证实：1.受案登记表、立案决定书，证明本案的来源和立案经过。2.户籍证明，证明被告人陈炫钧的身份信息。3.抓获经过，证明2021年9月11日17时许，民警根据线索举报发现陈炫钧涉嫌危害珍贵、濒危野生动物罪，后经摸排，民警在东兴市××镇××小区附近将陈炫钧抓获。4.搜查笔录、扣押清单及指认照片，证明民警在陈炫钧身上查获白色ｉｐｈｏｎｅ6Ｓｐｕｌｓ手机1部（138××××****）、钥匙2条；民警在东兴市××镇××路××号××楼仓库搜出5袋疑似穿山甲鳞片（4个白色编织袋和1个黄色编织袋）和4箱疑似穿山甲鳞片（箱内有多个蔬果干密封包装袋，袋内装有疑似穿山甲鳞片）。民警对上述物品予以扣押，陈炫钧予以指认。5.清点称量笔录、抽样送检笔录及照片，证明民警将查获装有疑似穿山甲鳞片的5袋编织袋分别编号为1-5号，将装有疑似穿山甲鳞片的4个纸箱编号为6号。经称量，1号重4.4千克、2号重5.2千克、3号重7.3千克、4号重5.75千克、5号重6.8千克、6号重31.9千克，总计重量61.35千克。后民警分别从编号为1-6号疑似穿山甲鳞片中分别抽样0.2千克、0.2千克、0.3千克、0.35千克、0.3千克、0.25千克，分别编号为1-1、2-1、3-1、4-1、5-1、6-1送检。并对余下疑似穿山甲鳞片物品进行封存。6.证人陈某的证言，证明其是东兴市××户主。大概2019年5月份，其将该房的一楼仓库租给一名自称姓阮的越南女性（微信号“×××

23”，微信昵称“ｌｉｎｈ”，备注“阮氏玉批发越南沉香料”，电话号码×****）用来存放货物。其不清楚对方用来存放什么货物。2021年9月11日晚，对方在微信上联系其说钥匙不见了，其遂让其老婆在家里找了一条钥匙打开一楼仓库。平时一楼仓库关上门后多了一把U型锁，其没有钥匙进不去。其不清楚仓库由谁管理，都是××玉在安排。之前其收租时对方说过现在在越南，因为疫情无法入境。7.被告人陈炫钧在侦查阶段的供述和辩解，证明2020年1月份，其大舅子情人“阿玉”（微信昵称：越南沉香批发）因回越南过年，将租用的东兴市××镇××路××号××楼仓库的大门钥匙和U型锁钥匙交给其保管，后因疫情，对方一直无法回东兴。2021年9月10日下午15时，“阿玉”微信联系其让其到该仓库开门给别人放东西进去，约十分钟后，其将一名年轻男子带来的两袋蛇皮袋货物搬进该仓库。9月11日早上将近九点，“阿玉”微信联系其有人拉货过来，让其开门。其到该仓库后与送货来的中年男子将10箱纸箱货物搬进仓库。其打开其中一个纸箱，并微信告诉“阿玉”是10箱菠萝干。同日11时许，其按照“阿玉”指示到仓库将10个纸箱的货物按照表面有数字的放一起，没有数字的另放一起。中午12点半，一名约30岁的中年男子将两袋蛇皮袋装着的货物到仓库，其帮着从门口拖入仓库，按照“阿玉”的指示打开蛇皮袋发现两袋里面装着的都是穿山甲鳞片，后其微信联系“阿玉”告知对方是穿山甲鳞片，对方叫其按照表面写有数字的纸箱里包装的穿山甲鳞片打包其他的穿山甲鳞片。其打开表面装有数字的纸箱后发现，纸箱上层和底层是一包包的菠萝干，中间层有15包菠萝干包装袋的侧面割开，里面的菠萝干换成了穿山甲鳞片。其遂按照一样的方式，把菠萝干包装袋侧面割开，把里面的菠萝干倒到泡沫箱里，将蛇皮袋里

的穿山甲鳞片装进空的菠萝干包装袋里，再用双面胶将隔开的地方粘好。其打包了2纸箱的穿山甲鳞片后，觉得肚子饿了就回家吃饭。之后在返回仓库继续打包的途中被民警抓获。其不清楚穿山甲鳞片的来源和物主。其帮“阿玉”开门没有好处，“阿玉”答应给其每帮忙打包一公斤穿山甲鳞片20元的报酬。打包好的鳞片“阿玉”会叫人过仓库拉走。其没有做完工，其还没有拿到打包穿山甲鳞片的报酬。

8.鉴定意见，证明经华南动物物种环境损害司法鉴定中心鉴定，送检的疑似穿山甲鳞片1批（编号1-6号），净重61.35千克，经鉴定均为哺乳纲鳞甲目穿山甲科穿山甲属马来穿山甲的鳞片。马来穿山甲属于国家一级保护野生动物，被列入《濒危野生动植物种国际贸易公约》（CLTES）附录Ⅰ。经济价值人民币3435600元。

9.现场勘验、辨认现场笔录及照片，证明本案中心现场位于东兴市××镇××路××号××楼南侧第一间房内，屋内有多处杂物，中间地面堆放有泡沫箱、纸箱等物品。陈炫钧能辨认出其就在该处外面接货并在该屋打包穿山甲鳞片。

10.电子数据（微信聊天记录）、情况说明等，证明民警从陈炫钧的手机中提取了微信聊天记录。陈炫钧手机登录有微信昵称“凉茶1号”（微信号“×××12”）的微信。（1）2021年7月3日至8月6日,“口顺口口口口”向“凉茶1号”发送了云南、山东、安徽、广东、湖南、北京、四川、浙江、福建、河北等多地的联系地址及方式、姓名，“凉茶1号”向“口顺口口口口”发送了多张快递包裹、疑似野生动物制品及称重的照片；（2）2021年6月26日，“凉茶1号”与“批发越南沉香料—×××59”之间发送多张图片，内容是一个快件因“花茶夹带虎牙和爪子”被公安扣押；（3）2021年7月3

日，“批发越南沉香料—×××59”向“凉茶1号”发送了一个包裹的图片，并说“这个你们要打开看哦这是（动物图片）的角”“黑黑的角”“不是犀牛的”；（4）2021年8月8日，“批发越南沉香料—×××59”向“凉茶1号”发送疑似穿山甲鳞片的图片，并说“都是甲片是吗”“你明天看一下有10公斤是这个”“跟3.3这种比大跟10公斤在一起”“在这黑袋”“你帮分出来一下”；8月9日，“凉茶1号”向“批发越南沉香料—×××59”发送了称重图片。

另外，附带民事公益诉讼起诉人当庭还出示了立案决定书、诉前公告，证明东兴市人民检察院在履职过程中发现陈炫钧的行为破坏国家野生动物资源，可能损害社会公共利益，于2021年11月4日就公益诉讼立案审查，并于2021年11月5日公告了该案相关情况，公告期内未有法律规定的机关或有关组织提起民事公益诉讼。

上述证据来源合法，内容客观、真实，与本案存在关联性，能相互印证，已形成完整的证明体系，且均经庭审举证、质证属实，本院予以确认。

关于被告人陈炫钧是否构成危害珍贵、濒危野生动物罪的问题。经查，陈炫钧在侦查阶段供述其自2020年疫情暴发以来，受“阿玉”指使帮管理东兴市××镇××路××号××楼仓库及钥匙，其开门给他人存放及帮接收货物，亦供述在打开涉案货物明知是穿山甲鳞片的情况下受“阿玉”指使帮助对方将穿山甲鳞片包装成菠萝干，以便后续他人过来拉走。其虽在庭审中否认主观故意，但其采用隐蔽包装方式打包物品，明显违背了合法物品惯常的包装方式，足以认定陈炫钧明知是穿山甲鳞片仍予以帮助保管、包装及运输。上述另有搜查笔录、扣押清单、指认照片、抽样送检、清点称量笔录、证人陈某的证

言、鉴定意见、现场勘验、辨认现场笔录及照片、微信聊天记录等证据予以佐证。陈炫钧的行为符合危害珍贵、濒危野生动物罪的构成要件，应当予以定罪处罚。故被告人陈炫钧提出其不构成本罪的辩解不成立，本院不予采纳。

关于被告人陈炫钧是否应对查获的全部野生动物制品承担责任的问题。经查，陈炫钧受“阿玉”指使帮助管理东兴市××镇××路××号××楼仓库，并接收穿山甲鳞片进行重新打包。其在打包好部分穿山甲鳞片后离开现场吃饭，回来时被民警抓获。民警在该仓库中扣押了其接收的净重61.35千克的穿山甲鳞片，经济价值为人民币3435600元，应当计入其犯罪数额。根据《最高人民法院、最高人民检察院关于办理破坏野生动物资源刑事案件适用法律若干问题的解释》第六条第一款之规定，应当认定为“情节特别严重”。故辩护人提出陈炫钧只需对其打包好的2包穿山甲鳞片负责的辩护意见不成立，本院不予采纳。

本院认为，被告人陈炫钧受他人指使，非法运输穿山甲鳞片，情节特别严重，其行为已构成危害珍贵、濒危野生动物罪。公诉机关指控的罪名成立。被告人陈炫钧已经着手实施犯罪，由于意志以外的原因而未得逞，是犯罪未遂，可以比照既遂犯从轻或者减轻处罚；在共同犯罪中，其受人雇请指使实施犯罪活动，起次要作用，是从犯，应当从轻或者减轻处罚。辩护人所提陈炫钧是犯罪未遂、从犯的辩护意见成立，本院予以采纳。综上，本院决定对被告人陈炫钧减轻处罚。随案移送的手机1部，是作案工具，应予没收；扣押在案的穿山甲鳞片6袋，应予没收，由扣押机关依法处理；钥匙2条，与本案无关，应予返还。关于辩护人提出返还手机的辩护意见不成立，本院不予采纳。

穿山甲属于国家一级保护野生动物，被列入《濒危野生动植物种国际贸易公约》（CLTES）附录Ⅰ保护的珍贵、濒危野生动物，是宝贵的自然资源，除经济价值外，还具有内在不可估量的生态、科研、社会、遗传资源等价值，有公共利益属性，我国系上述国际公约的缔约国，有履行国际公约共同保护地球生态资源的责任。被告陈炫钧非法运输上述珍贵、濒危野生动物制品的行为，损害了公共利益，依法应承担民事侵权责任。东兴市人民检察院在履行刑事案件审查起诉职能中发现本案线索，依照法定程序公告，后提起刑事附带民事公益诉讼符合法律规定的条件和程序，是依法维护社会公共利益的一种方式，主体适格；要求陈炫钧赔偿因非法运输珍贵、濒危野生动物制品导致野生动物资源损失承担生态资源受损费用，并当庭进行公开赔礼道歉的诉讼请求于法有据，本院予以支持。

综上，为打击犯罪，保护生态资源，修复野生动物自然资源受到的损害，依照《中华人民共和国刑法》第三百四十一条第一款、第二十三条、第二十五条第一款、第二十七条、第五十二条、第五十三条、第六十三条第一款、第六十四条，《中华人民共和国民法典》第一百七十九条第一款第（八）项、第（十一）项及第三款、第一百八十七条、第一千二百三十五条，《中华人民共和国刑事诉讼法》第一百零一条第二款，《中华人民共和国民事诉讼法》第五十八条，《最高人民法院、最高人民检察院关于办理破坏野生动物资源刑事案件适用法律若干问题的解释》第六条第一款之规定，判决如下：

一、被告人陈炫钧犯危害珍贵、濒危野生动物罪（未遂），判处有期徒刑六年，并处罚金人民币五万元；

（刑期从判决执行之日起计算。判决执行以前先行羁押的，羁押一日折抵刑期一日。刑期即自 2021 年 9 月 12 日起至

2027 年 9 月 11 日止。罚金限于本判决生效后一个月内缴纳，逾期不缴纳的，强制缴纳。）

二、附带民事公益诉讼被告陈炫钧赔偿生态资源受损费用人民币三百四十三万五千六百元；

三、附带民事公益诉讼被告陈炫钧就其侵权行为当庭公开赔礼道歉（已当庭履行）；

四、随案移送的手机一部，予以没收；扣押在案的穿山甲鳞片六袋，予以没收，由扣押机关依法处理；钥匙二条，返还陈炫钧。

如不服本判决，可在接到判决书的第二日起十日内，通过本院或者直接向广西壮族自治区防城港市中级人民法院提出上诉。书面上诉的，应当提交上诉状正本一份，副本八份。

审　判　长　　龙　剑
审　判　员　　陆淑芸
审　判　员　　杨　玲
人民陪审员　　王以强
人民陪审员　　黄　鑫
人民陪审员　　黄绍明
人民陪审员　　王伦英
二〇二二年九月二十八日
书　记　员　　杨雅婷

附相关法律条文：

《中华人民共和国刑法》

第三百四十一条非法猎捕、杀害国家重点保护的珍贵、濒危野生动物的，或者非法收购、运输、出售国家重点保护的珍贵、濒危野生动物及其制品的，处五年以下有期徒刑或者拘役，并处罚金；情节严重的，处五年以上十年以下有期徒刑，

并处罚金；情节特别严重的，处十年以上有期徒刑，并处罚金或者没收财产。

违反狩猎法规，在禁猎区、禁猎期或者使用禁用的工具、方法进行狩猎，破坏野生动物资源，情节严重的，处三年以下有期徒刑、拘役、管制或者罚金。

违反野生动物保护管理法规，以食用为目的非法猎捕、收购、运输、出售第一款规定以外的在野外环境自然生长繁殖的陆生野生动物，情节严重的，依照前款的规定处罚。

第二十三条已经着手实行犯罪，由于犯罪分子意志以外的原因而未得逞的，是犯罪未遂。

对于未遂犯，可以比照既遂犯从轻或者减轻处罚。

第二十五条共同犯罪是指二人以上共同故意犯罪。

二人以上共同过失犯罪，不以共同犯罪论处；应当负刑事责任的，按照他们所犯的罪分别处罚。

第二十七条在共同犯罪中起次要或者辅助作用的，是从犯。

对于从犯，应当从轻、减轻处罚或者免除处罚。

第五十二条判处罚金，应当根据犯罪情节决定罚金数额。

第五十三条罚金在判决指定的期限内一次或者分期缴纳。期满不缴纳的，强制缴纳。对于不能全部缴纳罚金的，人民法院在任何时候发现被执行人有可以执行的财产，应当随时追缴。

由于遭遇不能抗拒的灾祸等原因缴纳确实有困难的，经人民法院裁定，可以延期缴纳、酌情减少或者免除。

第六十三条犯罪分子具有本法规定的减轻处罚情节的，应当在法定刑以下判处刑罚；本法规定有数个量刑幅度的，应当在法定量刑幅度的下一个量刑幅度内判处刑罚。

犯罪分子虽然不具有本法规定的减轻处罚情节，但是根据案件的特殊情况，经最高人民法院核准，也可以在法定刑以下判处刑罚。

第六十四条犯罪分子违法所得的一切财物，应当予以追缴或者责令退赔；对被害人的合法财产，应当及时返还；违禁品和供犯罪所用的本人财物，应当予以没收。没收的财物和罚金，一律上缴国库，不得挪用和自行处理。

《中华人民共和国民法典》第一百七十九条承担民事责任的方式主要有：

（一）停止侵害；

（二）排除妨碍；

（三）消除危险；

（四）返还财产；

（五）恢复原状；

（六）修理、重作、更换；

（七）继续履行；

（八）赔偿损失；

（九）支付违约金；

（十）消除影响、恢复名誉；

（十一）赔礼道歉。

法律规定惩罚性赔偿的，依照其规定。

本条规定的承担民事责任的方式，可以单独适用，也可以合并适用。

第一百八十七条民事主体因同一行为应当承担民事责任、行政责任和刑事责任的，承担行政责任或者刑事责任不影响承担民事责任；民事主体的财产不足以支付的，优先用于承担民事责任。

第一千二百三十五条违反国家规定造成生态环境损害的，国家规定的机关或者法律规定的组织有权请求侵权人赔偿下列损失和费用：

（一）生态环境受到损害至修复完成期间服务功能丧失导致的损失；

（二）生态环境功能永久性损害造成的损失；

（三）生态环境损害调查、鉴定评估等费用；

（四）清除污染、修复生态环境费用；

（五）防止损害的发生和扩大所支出的合理费用。

《中华人民共和国刑事诉讼法》

第一百零一条被害人由于被告人的犯罪行为而遭受物质损失的，在刑事诉讼过程中，有权提起附带民事诉讼。被害人死亡或者丧失行为能力的，被害人的法定代理人、近亲属有权提起附带民事诉讼。如果是国家财产、集体财产遭受损失的，人民检察院在提起公诉的时候，可以提起附带民事诉讼。

《中华人民共和国民事诉讼法》

第五十八条对污染环境、侵害众多消费者合法权益等损害社会公共利益的行为，法律规定的机关和有关组织可以向人民法院提起诉讼。人民检察院在履行职责中发现破坏生态环境和资源保护、食品药品安全领域侵害众多消费者合法权益等损害社会公共利益的行为，在没有前款规定的机关和组织或者前款规定的机关和组织不提起诉讼的情况下，可以向人民法院提起诉讼。前款规定的机关或者组织提起诉讼的，人民检察院可以支持起诉。

《最高人民法院、最高人民检察院关于办理破坏野生动物资源刑事案件适用法律若干问题的解释》

第六条非法猎捕、杀害国家重点保护的珍贵、濒危野生动物，或者非法收购、运输、出售国家重点保护的珍贵、濒危野生动物及其制品，价值二万元以上不满二十万元的，应当依照刑法第三百四十一条第一款的规定，以危害珍贵、濒危野生动物罪处五年以下有期徒刑或者拘役，并处罚金；价值二十万元以上不满二百万元的，应当认定为“情节严重”，处五年以上十年以下有期徒刑，并处罚金；价值二百万元以上的，应当认定为“情节特别严重”，处十年以上有期徒刑，并处罚金或者没收财产。实施前款规定的行为，具有下列情形之一的，从重处罚：（一）属于犯罪集团的首要分子的；（二）为逃避监管，使用特种交通工具实施的;（三）严重影响野生动物科研工作的；（四）二年内曾因破坏野生动物资源受过行政处罚的。实施第一款规定的行为，不具有第二款规定的情形，且未造成动物死亡或者动物、动物制品无法追回，行为人全部退赃退赔，确有悔罪表现的，按照下列规定处理：（一）珍贵、濒危野生动物及其制品价值二百万元以上的，可以认定为“情节严重”，处五年以上十年以下有期徒刑，并处罚金；（二）珍贵、濒危野生动物及其制品价值二十万元以上不满二百万元的，可以处五年以下有期徒刑或者拘役，并处罚金；（三）珍贵、濒危野生动物及其制品价值二万元以上不满二十万元的，可以认定为犯罪情节轻微，不起诉或者免予刑事处罚；情节显著轻微危害不大的，不作为犯罪处理。
