# Supplementary material for: China’s Legal Protection System for Pangolins: Past, Present, and Future
Source: Animals (Basel). 2025 Aug 18;15(16):2422. doi: 10.3390/ani15162422 (PMC12383201; doi:10.3390/ani15162422)
Supplement: Supplementary file 1 [file animals-15-02422-s001.zip › Supplementary Material S4-Full Text of Judgments in Pangolin-Related Public Interest Litigation Cases in China/【6】周某某危害珍贵、濒危野生动物一审刑事判决书(FBM-CLI.C.500370317).pdf]

周某某危害珍贵、濒危野生动物一审刑事判决书

周某某危害珍贵、濒危野生动物一审刑事判决书

辽宁省沈阳市浑南区人民法院

刑事附带民事判决书

(2022)辽0112刑初350号

公诉机关沈阳市和平区人民检察院。

附带民事公益诉讼机关沈阳市和平区人民检察院。

被告人暨附带民事公益诉讼被告人周某某，男性。因涉嫌危害珍贵、濒危野生动物罪，于2022年6月21日被刑事拘留，同年7月4日被监视居住。

沈阳市和平区人民检察院以沈和检刑诉〔2022〕356号起诉书指控被告人周某某犯危害珍贵、濒危野生动物罪，于2022年9月1日向本院提起公诉。2022年9月5日，沈阳市和平区人民检察院向本院提交沈和检刑附民公诉〔2022〕11号刑事附带民事公益诉讼起诉书，以被告人周某某危害珍贵、濒危野生动物的行为，损害了国家利益和社会公共利益为由，向本院提起刑事附带民事公益诉讼。本院受理后，依法组成合议庭，公开开庭合并审理了本案。沈阳市和平区人民检察院指派检察官杨丽莹出庭支持公诉及附带民事公益诉讼。被告人周某某到庭参加诉讼。本案现已审理终结。

沈阳市和平区人民检察院指控：2021年11月至2022年3月期间，被告人周某某收购大量穿山甲鳞片、海马干制品在其经营的沈阳市和平区XXX土特产品商行销售。2022年3月7日l4时许，被告人周某某在该商行中被抓获，现场查获穿山甲鳞片2袋，海马干制品207只。经福建闽林司法鉴定中心鉴定：穿山甲列入《国家重点保护野生动物名录》，属于国家一级重点保护野生动物，2袋炮制过的穿山甲鳞片净重共计1.821kg，核定价值共计人民币116544元；海马属于国家二级重点保护水生野生动物，委检的207只海马干制品，核定价值共计人民币24840元。

沈阳市和平区人民检察院认为，被告人周某某，违反法律规定，非法收购、出售国家重点保护的珍贵、濒危野生动物制品，其行为触犯了《中华人民共和国刑法》第三百四十一条第一款规定，犯罪

事实清楚，证据确实、充分，应当以危害珍贵、濒危野生动物罪追究其刑事责任。被告人周某某认罪认罚，可以从宽处理。建议判处被告人周某某拘役六个月，并处罚金，可以适用缓刑。

附带民事公益诉讼机关沈阳市和平区人民检察院诉称，1.判令周某某赔偿因非法收购珍贵、濒危野生动物制品导致野生动物资源损失人民币141384元；2.判令周某某在省级以上媒体就非法收购珍贵、濒危野生动物制品行为向公众赔礼道歉。

被告人周某某对公诉机关指控的犯罪事实和量刑建议无异议，对刑事附带民事公益诉讼指控无异议。

经审理查明，2021年11月至2022年3月期间，被告人周某某收购大量穿山甲鳞片、海马干制品在其经营的沈阳市和平区XXX土特产品商行销售，获利人民币230元。2022年3月7日14时许，被告人周某某在该商行中被抓获，现场查获穿山甲鳞片2袋，海马干制品207只。经福建闽林司法鉴定中心鉴定：穿山甲列入《国家重点保护野生动物名录》，属于国家一级重点保护野生动物，2袋炮制过的穿山甲鳞片净重共计1.821kg，核定价值共计人民币116544元；海马属于国家二级重点保护水生野生动物，委检的207只海马干制品，核定价值共计人民币24840元。

附带民事公益诉讼机关沈阳市和平区人民检察院的合理经济损失为人民币141384元。

另查明，被告人周某某已将赔偿款暂存于本院，并于2022年9月20日在辽沈晚报上登报公开道歉。

本院认为：被告人周某某非法出售国家重点保护的珍贵、濒危野生动物，其行为已构成危害珍贵、濒危野生动物罪。沈阳市和平区人民检察院的指控，事实清楚，证据确实充分，罪名成立，应予支持。被告人周某某当庭自愿认罪且案件适用认罪认罚制度，主动缴纳罚金及违法所得，积极赔偿国家经济损失，可对其酌情从轻处罚。由于被告人周某某买卖珍贵、濒危野生动物制品的行为，损害了国家利益和社会公共利益，造成国家经济损失，被告人周某某除承担刑事责任外，在民事法律关系上，属于环境违法侵权行为，依法应当承担民事赔偿责任。附带民事公益诉讼机关沈阳市和平区人民检察院提出的诉讼请求合法有据，本院予以支持。

综上所述，依照《中华人民共和国刑法》第三百四十一条第一款，第六十七条第三款，第五十二条，第五十三条，第六十四条，第七十二条第一、二款，第七十三条第二、三款，《中华人民共和国民法典》第一百七十九条第(八)项、第(十一)项，《中华人民共和国刑事诉讼法》第十五条、第一百

零一条第二款，《中华人民共和国民事诉讼法》第五十八条之规定，判决如下：

一、被告人周某某犯危害珍贵、濒危野生动物罪，判处拘役六个月，缓刑一年，并处罚金人民币一万元。(已缴纳)

(缓刑考验期从判决确定之日起计算。被告人周某某应于本判决生效后10日内到沈阳市和平区司法局社区矫正管理部门报到，依法接受社区矫正。)

二、依法追缴被告人周某某违法所得人民币二百三十元，上缴国库。(已缴纳)

三、扣押的黄色片状穿山甲鳞片1.896千克、黑色海马干207只依法予以没收，上缴国库。

四、禁止被告人周某某在缓刑考验期内从事野生动物及其制品收售等相关工作。

(禁止令期限从判决确定之日起计算)

五、被告人周某某与其他侵权人连带赔偿国家经济损失共计人民币十四万一千三百八十四元，缴至附带民事公益诉讼机关沈阳市和平区人民检察院指定账户，上缴国库。(已缴纳)

六、被告人周某某在省级媒体上公开赔礼道歉。(已履行)

如不服本判决，可在接到判决书的第二天起十日内，通过本院或者直接向辽宁省沈阳市中级人民法院提出上诉。书面上诉的，应当提交上诉状正本一份，副本两份。

审 判 长　金明哲

审 判 员　郝小丽

审 判 员　高亚男

人民陪审员　杨 娇

人民陪审员　吴亚楠

人民陪审员　崔锦仙

人民陪审员　刘庆文

二〇二二年九月二十七日

书 记 员　王 璐

©北大法宝：（www.pkulaw.com）专业提供法律信息、法学知识和法律软件领域各类解决方案。北大法宝为您提供丰富的参考资料，正式引用法规条文时请与标准文本核对。 欢迎查看所有产品和服务。

法宝快讯： 如何快速找到您需要的检索结果？ 法宝 V6 有何新特色？

扫描二维码阅读原文

原文链接： https://www.pkulaw.com/pfnl/08df102e7c10f206991396d82b9e8ccc6f4b645bdc71a863bdfb.html
