# Supplementary material for: China’s Legal Protection System for Pangolins: Past, Present, and Future
Source: Animals (Basel). 2025 Aug 18;15(16):2422. doi: 10.3390/ani15162422 (PMC12383201; doi:10.3390/ani15162422)
Supplement: Supplementary file 1 [file animals-15-02422-s001.zip › Supplementary Material S4-Full Text of Judgments in Pangolin-Related Public Interest Litigation Cases in China/【7】裴迎元、裴迎元非法收购、运输等刑事一审刑事判决书.pdf]

附带民事公益诉讼被告裴迎元、裴迎元非法收购、运输等刑事一审刑事判决书

案由	非法收购、运输、出售珍贵、濒危野生动物、珍贵、濒危野生动物制品<br>点击了解更多	案号	（2021）桂0681刑初248号	• •

广西壮族自治区东兴市人民法院
刑 事 附 带 民 事 判 决 书

（2021）桂0681刑初248号

公诉机关暨附带民事公益诉讼起诉人广西壮族自治区东兴市人民检察院。

被告人暨附带民事公益诉讼被告裴迎元，男，1988年9月27日出生，籍贯湖南省邵东县，汉族，初中文化，无业，户籍住址广西灵川县，因涉嫌犯非法收购珍贵、濒危野生动物制品罪于2020年11月7日被刑事拘留，同年12月14日被取保候审。

指定辩护人周远志，广西众润律师事务所律师。

东兴市人民检察院以东检刑诉〔2021〕Z72号起诉书指控被告人裴迎元犯危害珍贵、濒危野生动物罪一案，于2021年9月26日向本院提起公诉，并以东检刑附民公诉〔2021〕11号刑事附带民事公益诉讼起诉书就裴迎元非法收购珍贵、濒危野生动物制品损害社会公共利益的行为向本院提起刑事附带民事公益诉讼。经查，东兴市人民检察院于同年4月20日公告了该案相关情况，公告期内未有法律规定的机关或有关组织提起民事公益诉

讼。本院受理后，依法组成合议庭，于同年 11 月 19 日公开开庭审理了本案。东兴市人民检察院指派检察员龙雪梅出庭支持公诉，指派检察员杨媚出庭履行职务，被告人暨附带民事公益诉讼被告裴迎元及其指定辩护人周远志到庭参加诉讼。本案现已审理终结。

东兴市人民检察院指控，2020 年 10 月 24 日，被告人裴迎元通过微信向他人购买穿山甲鳞片，通过微信转账支付了人民币 2400 元（以下币种均同），并将收货地址发给对方。同年 11 月 7 日上午，裴迎元安排妻子赵某收取装有疑似穿山甲鳞片快递包裹，民警当场将赵某和快递包裹拦截，并在桂林市灵川县将裴迎元抓获。民警从快递包裹中查获疑似穿山甲鳞片一批，经称量，净重 815 克。经鉴定，所查获的疑似穿山甲鳞片来源于鳞甲目穿山甲科穿山甲属南非穿山甲，被列入《濒危野生动植物种国际贸易公约》（ＣＩＴＥＳ）附录Ⅰ，经济价值为 8640 元。

公诉机关指控上述事实提供了相应的证据予以证明，并认为，被告人裴迎元非法收购国家重点保护的珍贵、濒危野生动物制品，其行为触犯了《中华人民共和国刑法》第三百四十一条第一款之规定，应当以危害珍贵、濒危野生动物罪追究其刑事责任。裴迎元到案后如实供述自己的罪行，可以从轻处罚；其认罪认罚，可以从宽处理。综上，建议若符合社区矫正条件，对被告人裴迎元判处有期徒刑六个月，缓刑一年，并处罚金人民币一万元。

附带民事公益诉讼起诉人东兴市人民检察院向本院提出诉讼请求：1.请求判令被告裴迎元依法承担赔偿生态资源受损费用8640元；2.请求判令被告裴迎元对其侵权行为当庭进行公开赔礼道歉。

被告人暨附带民事公益诉讼被告裴迎元对指控的犯罪事实与罪名均无异议，自愿认罪认罚并签字具结，对公益诉讼起诉人的诉讼请求无异议，庭前已预缴纳全部生态资源受损费用，并当庭进行了赔礼道歉。

指定辩护人对指控的犯罪事实与罪名均无异议，并提出裴迎元出于自身需要购买穿山甲鳞片，而非用于营利，且其具有坦白情节并自愿认罪认罚，符合适用缓刑的条件，建议对其适用缓刑。

经审理查明，2020年10月24日，被告人裴迎元通过微信向他人购买穿山甲鳞片，通过微信转账支付了2400元，并将收货地址发给对方。同年11月7日上午，裴迎元安排妻子赵某收取装有疑似穿山甲鳞片快递包裹，民警当场将赵某和快递包裹拦截，并在桂林市灵川县将裴迎元抓获。民警在快递包裹中查获疑似穿山甲鳞片一批。经称量，穿山甲鳞片净重815克。经鉴定，所查获的疑似穿山甲鳞片来源于鳞甲目穿山甲科穿山甲属南非穿山甲，被列入《濒危野生动植物种国际贸易公约》（ＣＩＴＥＳ）附录Ⅰ，经济价值为8640元。

上述事实，有公诉机关暨附带民事公益诉讼起诉人提供的受案登记表、立案决定书、户籍证明、指定管辖决定书、抓获经过、

扣押物品清单、称重笔录及照片、封存笔录、关于司法鉴定过程中检材质量差异的通用说明等书证，证人赵某、沈某的证言，被告人裴迎元的供述和辩解，鉴定意见，检查、提取、辨认等笔录及照片，电子数据检查笔录及公益诉讼诉前公告程序材料等证据予以证明。上述证据来源合法，内容客观、真实，与本案存在关联性，能相互印证，已形成完整的证明体系，且均经庭审举证、质证属实，本院予以确认。

本院认为，被告人裴迎元非法收购穿山甲鳞片，其行为已构成危害珍贵、濒危野生动物罪。公诉机关指控的罪名成立。被告人裴迎元到案后如实供述，可以从轻处罚；其认罪认罚，可以从宽处理。辩护人所提裴迎元具有坦白情节且自愿认罪认罚的辩护意见成立，本院予以采纳。综上，本院决定对被告人裴迎元从轻处罚。被告人裴迎元居住地司法行政机关出具了调查评估意见书，对裴迎元评估意见为可适用社区矫正。鉴于被告人裴迎元认罪态度好，有悔罪表现，没有再犯罪的危险，宣告缓刑对所居住的社区无重大不良影响，故在综合考虑被告人裴迎元的犯罪事实、性质、情节、社会危害性及认罪悔罪等情况，对被告人裴迎元适用缓刑。公诉机关的量刑建议适当，本院予以采纳。随案移送的尾号为 8018 的黑色苹果手机 1 部，是作案工具，予以没收。

穿山甲作为纳入《濒危野生动植物种国际贸易公约》附录 I 保护的珍贵、濒危野生动物，是宝贵的自然资源，除经济价值外，还具有内在不可估量的生态、科研、社会、遗传资源等价值，有

公共利益属性，我国系上述国际公约的缔约国，有履行国际公约共同保护地球生态资源的责任。被告裴迎元非法收购上述珍贵、濒危野生动物制品的行为，损害了公共利益，依法应承担民事侵权责任。东兴市人民检察院在履行刑事案件审查起诉职能中发现本案线索，依照法定程序公告，后提起刑事附带民事公益诉讼符合法律规定的条件和程序，是依法维护社会公共利益的一种方式，主体适格；要求裴迎元赔偿因非法收购珍贵、濒危野生动物制品导致野生动物资源损失承担生态资源受损费用，并在当庭进行公开赔礼道歉的诉讼请求于法有据，本院予以支持。

综上，为打击犯罪，保护生态资源，修复野生动物自然资源受到的损害，依照《中华人民共和国刑法》第三百四十一条第一款、第五十二条、第五十三条、第六十四条、第六十七条第三款、第七十二条、第七十三条，《中华人民共和国侵权责任法》第十五条第一款第（六）项、第（七）项及第二款，《中华人民共和国刑事诉讼法》第十五条、第一百零一条第二款、第二百零一条，《中华人民共和国民事诉讼法》第五十五条之规定，判决如下：

一、被告人裴迎元犯危害珍贵、濒危野生动物罪，判处有期徒刑六个月，缓刑一年，并处罚金人民币一万元；

（缓刑考验期限，从判决确定之日起计算。罚金已预缴。）

二、随案移送的手机一部，予以没收；

三、附带民事公益诉讼被告裴迎元赔偿生态资源受损费用人民币八千六百四十元；（庭前已预缴）

四、附带民事公益诉讼被告裴迎元就其侵权行为当庭公开赔礼道歉。（已当庭履行）

如不服本判决，可在接到判决书的第二日起十日内，通过本院或者直接向广西壮族自治区防城港市中级人民法院提出上诉。书面上诉的，应当提交上诉状正本一份，副本八份。

审　判　长　　曾宪林

审　判　员　　陆淑芸

审　判　员　　杨　玲

人民陪审员　　王以强

人民陪审员　　龙恒裕

人民陪审员　　王伦英

人民陪审员　　温基业

二〇二一年十二月十六日

书　记　员　　李兴尚

附相关法律条文：

《中华人民共和国刑法》

第三百四十一条非法猎捕、杀害国家重点保护的珍贵、濒危野生动物的，或者非法收购、运输、出售国家重点保护的珍贵、濒危野生动物及其制品的，处五年以下有期徒刑或者拘役，并处罚金；情节严重的，处五年以上十年以下有期徒刑，并处罚金；情节特别严重的，处十年以上有期徒刑，并处罚金或者没收财产。

违反狩猎法规，在禁猎区、禁猎期或者使用禁用的工具、方法进行狩猎，破坏野生动物资源，情节严重的，处三年以下有期徒刑、拘役、管制或者罚金。

违反野生动物保护管理法规，以食用为目的非法猎捕、收购、运输、出售第一款规定以外的在野外环境自然生长繁殖的陆生野生动物，情节严重的，依照前款的规定处罚。

第五十二条判处罚金，应当根据犯罪情节决定罚金数额。

第五十三条罚金在判决指定的期限内一次或者分期缴纳。期满不缴纳的，强制缴纳。对于不能全部缴纳罚金的，人民法院在任何时候发现被执行人有可以执行的财产，应当随时追缴。

由于遭遇不能抗拒的灾祸等原因缴纳确实有困难的，经人民法院裁定，可以延期缴纳、酌情减少或者免除。

第六十四条犯罪分子违法所得的一切财物，应当予以追缴或者责令退赔；对被害人的合法财产，应当及时返还；违禁品和供犯罪所用的本人财物，应当予以没收。没收的财物和罚金，一律上缴国库，不得挪用和自行处理。

第六十七条犯罪以后自动投案，如实供述自己的罪行的，是自首。对于自首的犯罪分子，可以从轻或者减轻处罚。其中，犯罪较轻的，可以免除处罚。

被采取强制措施的犯罪嫌疑人、被告人和正在服刑的罪犯，如实供述司法机关还未掌握的本人其他罪行的，以自首论。

犯罪嫌疑人虽不具有前两款规定的自首情节，但是如实供述自己罪行的，可以从轻处罚；因其如实供述自己罪行，避免特别严重后果发生的，可以减轻处罚。

第七十二条对于被判处拘役、三年以下有期徒刑的犯罪分子，同时符合下列条件的，可以宣告缓刑，对其中不满十八周岁的人、怀孕的妇女和已满七十五周岁的人，应当宣告缓刑：

（一）犯罪情节较轻；

（二）有悔罪表现；

（三）没有再犯罪的危险；

（四）宣告缓刑对所居住社区没有重大不良影响。

宣告缓刑，可以根据犯罪情况，同时禁止犯罪分子在缓刑考验期限内从事特定活动，进入特定区域、场所，接触特定的人。

被宣告缓刑的犯罪分子，如果被判处附加刑，附加刑仍须执行。

第七十三条拘役的缓刑考验期限为原判刑期以上一年以下，但是不能少于二个月。

有期徒刑的缓刑考验期限为原判刑期以上五年以下，但是不能少于一年。

缓刑考验期限，从判决确定之日起计算。

《中华人民共和国侵权责任法》

第十五条承担侵权责任的方式主要有：

（一）停止侵害；

（二）排除妨碍；

（三）消除危险；

（四）返还财产；

（五）恢复原状；

（六）赔偿损失；

（七）赔礼道歉；

（八）消除影响、恢复名誉。

以上承担侵权责任的方式，可以单独适用，也可以合并适用。

《中华人民共和国刑事诉讼法》

第十五条犯罪嫌疑人、被告人自愿如实供述自己的罪行，承认指控的犯罪事实，愿意接受处罚的，可以依法从宽处理。

第一百零一条被害人由于被告人的犯罪行为而遭受物质损失的，在刑事诉讼过程中，有权提起附带民事诉讼。被害人死亡或者丧失行为能力的，被害人的法定代理人、近亲属有权提起附带民事诉讼。

如果是国家财产、集体财产遭受损失的，人民检察院在提起公诉的时候，可以提起附带民事诉讼。

第二百零一条对于认罪认罚案件，人民法院依法作出判决时，一般应当采纳人民检察院指控的罪名和量刑建议，但有下列情形的除外：

（一）被告人的行为不构成犯罪或者不应当追究其刑事责任的；

（二）被告人违背意愿认罪认罚的；

（三）被告人否认指控的犯罪事实的；

（四）起诉指控的罪名与审理认定的罪名不一致的；

（五）其他可能影响公正审判的情形。

人民法院经审理认为量刑建议明显不当，或者被告人、辩护人对量刑建议提出异议的，人民检察院可以调整量刑建议。人民检察院不调整量刑建议或者调整量刑建议后仍然明显不当的，人民法院应当依法作出判决。

《中华人民共和国民事诉讼法》

第五十五条对污染环境、侵害众多消费者合法权益等损害社会公共利益的行为，法律规定的机关和有关组织可以向人民法院提起诉讼。

人民检察院在履行职责中发现破坏生态环境和资源保护、食品药品安全领域侵害众多消费者合法权益等损害社会公共利益的行为，在没有前款规定的机关和组织或者前款规定的机关和组织不提起诉讼的情况下，可以向人民法院提起诉讼。前款规定的机关或者组织提起诉讼的，人民检察院可以支持起诉。
