# Supplementary material for: China’s Legal Protection System for Pangolins: Past, Present, and Future
Source: Animals (Basel). 2025 Aug 18;15(16):2422. doi: 10.3390/ani15162422 (PMC12383201; doi:10.3390/ani15162422)
Supplement: Supplementary file 1 [file animals-15-02422-s001.zip › Supplementary Material S4-Full Text of Judgments in Pangolin-Related Public Interest Litigation Cases in China/【8】刘继和、附带民事公益诉讼被告郭秋民等刑事一审刑事判决书.pdf]

附带民事公益诉讼被告刘继和、附带民事公益诉讼被告郭秋民等刑事一审刑事判决书

江西省修水县人民法院

刑 事 附 带 民 事 判 决 书

（2021）赣0424刑初397号

公诉机关暨附带民事公益诉讼起诉人修水县人民检察院。

被告人暨附带民事公益诉讼被告刘继和，男，1968年10月6日出生，汉族，修水县人，初中文化，个体，户籍所在地修水县，住修水县。因涉嫌犯危害珍贵、濒危野生动物罪，2021年5月12日被修水县公安局刑事拘留，同年6月16日被逮捕。现羁押于修水县看守所。

辩护人邓征奇，江西法启律师事务所律师。

被告人暨附带民事公益诉讼被告郭秋民，男，1973年10月13日出生，汉族，江西省新余市渝水区人，小学文化，个体，住江西省新余市渝水区。2021年4月2日因犯危害珍贵、濒危野生动物罪被新余市渝水区人民法院判处有期徒刑一年，缓刑二年（因该案被羁押3个月零13日，缓刑考验期自2021年4月13日起至2023年4月12日止）。现因涉嫌犯危害珍贵、濒危野生动物罪，2021年5月12日被修水县公安局刑事拘留，同年6月16日被逮捕。现羁押于修水县看守所。

辩护人郭峰，江西大升律师事务所律师。

被告人暨附带民事公益诉讼被告龚汉华，男，1967 年 10 月 3 日出生，汉族，修水县人，小学文化，无业，户籍所在地修水县，住修水县

15 栋 1 单元 203 室。因涉嫌犯危害珍贵、濒危野生动物罪，2021 年 5 月 12 日被修水县公安局刑事拘留，同年 6 月 16 日被逮捕。现羁押于修水县看守所。

辩护人孔明星，江西坚白律师事务所律师。

被告人暨附带民事公益诉讼被告刘继明，男，1970 年 11 月 12 日出生，汉族，修水县人，小学文化，农民，住修水县。因涉嫌犯危害珍贵、濒危野生动物罪、非法持有枪支罪，2021 年 5 月 21 日被修水县公安局刑事拘留，同年 6 月 16 日被逮捕。现羁押于修水县看守所。

辩护人姚鹏飞，江西法启律师事务所律师。

被告人暨附带民事公益诉讼被告刘小平，男，1960 年 10 月 9 日出生，汉族，修水县人，小学文化，农民，住修水县。因涉嫌犯危害珍贵、濒危野生动物罪，2021 年 5 月 26 日被修水县公安局刑事拘留，同年 6 月 16 日被逮捕，2021 年 7 月 9 日变更为取保候审，同年 8 月 13 日修水县人民检察院决定继续取保候审。现在家候审。

辩护人杨晖，江西法启律师事务所律师。

被告人林常富，男，1970年3月18日出生，汉族，修水县人，小学文化，农民，住修水县。因涉嫌犯窝藏罪、非法持有枪支罪，2021年5月23日被修水县公安局刑事拘留，同年6月16日被逮捕。现羁押于修水县看守所。

指定辩护人王星火，江西东太律师事务所律师。

修水县人民检察院以修检刑诉（2021）112号起诉书指控被告人刘继和、郭秋民、龚汉华、刘小平犯危害珍贵、濒危野生动物罪，被告人刘继明犯危害珍贵、濒危野生动物罪、非法

持有枪支罪，被告人林常富犯窝藏罪、非法持有枪支罪，于2021年9月9日向本院提起公诉。在诉讼过程中，附带民事公益诉讼起诉人修水县人民检察院向本院提起附带民事公益诉讼。本院受理后，依法组成合议庭，于2021年10月22日公开开庭进行了合并审理。修水县人民检察院指派检察员江碧云出庭支持公诉，指派检察员朱昀出席法庭履行附带民事公益起诉职务，六被告人及辩护人邓征奇、郭峰、孔明星、姚鹏飞、杨晖、王星火均到庭参加了诉讼。现已审理终结。

修水县人民检察院指控：

1.2021年2月17日上午，被告人刘小平在砍柴时捕获1只穿山甲并送至其亲家即被告人刘继明处。当晚，刘继明以每斤500元的价格将该穿山甲出售给被告人刘继和，刘继和先支付了1000元现金。之后，刘继和又以每斤800元的价格将该穿山甲

出售给被告人郭秋民，经称重，该穿山甲重 14 斤多，郭秋民通过微信转了 11850 元给刘继和。郭秋民后将该穿山甲宰杀并出售。刘继和后付给刘继明余款 5800 元，刘继明将其中的 4000 元通过其儿媳妇刘某给了她父亲刘小平。

2.2021 年 4 月 13 日下午，被告人龚汉华在挖洋姜时捕获一大一小 2 只穿山甲。当晚其将该 2 只穿山甲送至被告人刘继和处，以 4,600 元出售给刘继和，刘继和先支付了 2,000 元给龚汉华。次日上午刘继和将该 2 只穿山甲出售给被告人郭秋民，获款 8,000 元，之后其将余款 2,600 元付给了龚汉华。郭秋民后将大的穿山甲宰杀并出售，小的穿山甲被他人以 1,000 元收购并放生。

3.2021 年 5 月 10 日下午，被告人龚汉华捕获 1 只 10 斤多的穿山甲，并于当晚以每斤 500 元的价格出售给被告人刘继和，刘继和先转了 2,500 元给龚汉华，约定余款 2,500 元售出后再付。次日，刘继和将该只穿山甲及 1 只死麂子送至郭秋民处，

以每斤 800 元的价格将穿山甲出售给郭秋民。双方准备交易时，被公安民警抓获。该穿山甲及死麂子被公安民警当场查获并扣押。

4.2021 年 5 月 13 日凌晨 2 时左右，公安民警到被告人刘继明家抓获其，其闻讯即躲避。刘继明后电话告知被告人林常富说公安民警要抓其及其在后山躲避的情况，林常富便让刘继明在其

家留宿一晚。次日早晨6时许，刘继明又将1只猎枪交给林常富保管，林常富便将该猎枪藏在自家的薯窖内。之后，林常富骑车将刘继明送至刘的丈母娘家。涉案枪支已被公安机关查获并扣押。经鉴定，涉案的枪支物品认定为枪支。刘继明、林常富均未办理持枪证。

另，2021年5月14日，被告人郭秋民妻子黄某将家中放置的重1.99斤的穿山甲甲片上交给了公安机关。经鉴定，被查获的穿山甲活体及穿山甲甲片、爪子为中华穿山甲，属国家一级保护动物，麂子为三有动物。被查获的穿山甲已被放生。

案发后，被告人刘继和、郭秋民、龚汉华、刘继明被抓获归案，被告人林常富被传唤到案，被告人刘小平向公安机关投案。刘小平退回违法所得4,000元，刘继明退回1,800元。

为证实上述指控，公诉机关当庭宣读、出示了被告人的供述与辩解，证人证言，搜查笔录、扣押笔录、扣押清单，微信转账记录及截图，指认现场笔录及照片，鉴定意见，归案说明等相关证据。

公诉机关认为被告人刘继和、郭秋民收购、出售、杀害国家重点保护野生动物，被告人龚汉华、刘小平猎捕国家重点保护动物，破坏野生动物资源；被告人刘继明出售国家重点保护野生动物，破坏野生动物资源，并非法持有枪支1支；被告人林常富明知公安机关在对刘继明进行抓捕，仍为其提供隐藏处所，且帮助刘继明藏匿枪支；应当以危害珍贵、濒危野生动物

罪追究被告人刘继和、郭秋民、龚汉华、刘小平的刑事责任，以危害珍贵、濒危野生动物罪、非法持有枪支罪追究被告人刘继明的刑事责任，以窝藏罪、非法持有枪支罪追究被告人林常富的刑事责任。刘继和、郭秋民、龚汉华，刘继和、郭秋民、刘继明、刘小平在相关危害珍贵、濒危野生动物犯罪中成立共同犯罪；刘继明、林常富在非法持有枪支犯罪中成立共同犯罪，刘继明属主犯，林常富在共同犯罪中起帮助作用，属从犯，应从轻处罚。郭秋民在缓刑考验期内又犯新罪，应当撤销缓刑，数罪并罚。刘小平具有自首情节，刘继和、郭秋民、龚汉华、刘继明、林常富具有坦白情节，六被告人均认罪认罚，可从宽处理。刘小平、刘继明退回了违法所得，可酌情从轻处罚。涉案部分穿山甲被放生，可酌情对相关被告人刘继和、郭秋民、龚汉华从轻处罚。建议对被告人刘继和判处有期徒刑二年，并处罚金；对郭秋民判处有期徒刑一年十个月，与前犯危害珍贵、濒危野生动物罪判处的有期徒刑一年并罚，合并执行有期徒刑二年六个月，并处罚金；对被告人龚汉华判处有期徒刑一年六个月，并处罚金；对被告人刘继明以犯危害珍贵、濒危野生动物罪判处有期徒刑八个月，并处罚金，犯非法持有枪支罪判处有期徒刑十个月，合并执行有期徒刑一年二个月，并处罚金；对被告人刘小平判处有期徒刑十个月，缓刑一年，并处罚金；对被告人林常富以犯窝藏罪判处有期徒刑

六个月，犯非法持有枪支罪判处有期徒刑六个月，合并执行有期徒刑八个月。

附带民事公益诉讼起诉人诉称，被告人刘继和、刘继明、刘小平、郭秋民共同危害珍贵、濒危野生动物致1只穿山甲死亡，被告人刘继和、龚汉华、郭秋民共同危害珍贵、濒危野生动物致1只穿山甲死亡，被告人刘继和、郭秋民出售、收购1只麂子，相关被告人破坏野生动物资源，造成生态环境损害，侵犯了国家利益和社会公共利益，依法应当承担民事侵权责任。

根据《野生动物及其制品价值评估方法》认定，1只穿山甲价值为80,000元，1只麂子价值为3,000元。故依法提请判令：1．被告人刘继和、刘继明、刘小平、郭秋民连带赔偿生态环境修复费用人民币8万元；2．被告人刘继和、龚汉华、郭秋民连带赔偿生态环境修复费用人民币8万元；3．被告人刘继和、郭秋民连带赔偿生态环境修复费用人民币3,000元。

被告人刘继和、郭秋民、龚汉华、刘继明、刘小平、林常富对起诉指控的事实、罪名及量刑建议均无异议且签字具结，在开庭审理过程中亦无异议。

被告人刘继和、郭秋民、龚汉华、刘继明、刘小平对附带民事公益诉讼起诉人提出的诉讼请求无异议。

辩护人邓征奇提出的辩护意见是：被告人刘继和具有坦白情节，并始终自愿认罪认罚，其儿子儿媳均为残疾人，家庭状况艰

难，其也愿意就附带民事公益诉讼部分进行赔偿，建议对被告人刘继和在量刑建议基础上调整降低刑期，并适用缓刑。关于附带民事诉讼方面，被告人刘继和在本案中仅为中转、转卖环节，其转卖时穿山甲均为活体，建议法庭根据刘继和的过错大小以及在犯罪中的作用，综合考虑确定刘继和的赔偿数额。

辩护人郭峰提出的辩护意见是：被告人郭秋民归案后能够如实供述罪行，积极配合办案，始终认罪认罚，悔罪态度良好，此前亦从未贩卖过的穿山甲，其主观恶性、社会危害性不大，建议对其从轻处罚并适用缓刑。

辩护人孔明星提出的辩护意见是：被告人龚汉华归案后如实供述罪行，积极配合办案，且系初犯、偶犯，其犯罪动机是为筹措医疗费及基本生存开支，主观恶性小，其非法猎捕的穿山甲 3 只，只是将穿山甲活体予以转卖，而且其中 2 只已被放生，社会危害性相对较小，建议对其从轻处罚。

辩护人姚鹏飞提出的辩护意见是：1．被告人刘继和是否先

行支付 1,000 元给被告人刘继明的事实不清；2．被告人刘继明系初犯、从犯，有坦白情节，积极退赃，认罪认罚，其主观恶性不大，社会危害性小，家庭状况特别困难，建议对其适用缓刑。

辩护人杨晖提出的辩护意见是：被告人刘小平具有自首情节，始终认罪认罚，并已退赃，且系初犯，主观恶性、社会危害

性不大，其亦患有高血压3级（极高危）等疾病，建议判处其缓刑。

辩护人王星火提出的辩护意见是：被告人林常富归案后如实供述罪行，积极配合调查，始终认罪认罚，并具有从犯情节，其主观恶性、社会危害性较小，再犯可能性很小，建议对其适用缓刑。

经审理查明：

一、被告人刘继和、郭秋民、龚汉华、刘继明、刘小平危害珍贵、濒危野生动物的事实

1.2021年2月17日上午，被告人刘小平在其家后山砍柴时捕获1只穿山甲并送至其亲家即被告人刘继明处。当晚，刘继明以每斤500元的价格将该穿山甲出售给被告人刘继和，刘继和先支付给刘继明1000元现金。之后，刘继和将该穿山甲送往新余以每斤800元的价格出售给被告人郭秋民，经称重，该穿山甲重14斤多，郭秋民即通过微信转了11850元给刘继和。郭秋民后将该穿山甲宰杀并出售。刘继和回到修水后付给刘继明余款5800元，刘继明将其中的4000元通过其儿媳妇刘某给了她父亲刘小平。刘继和从中获利5050元，刘小平获利4000元，刘继明获利2800元。

2.2021年4月13日下午，被告人龚汉华在修水县征村乡车田堰挖洋姜时捕获一大一小2只穿山甲。当晚其将该2只穿山甲送至被告人刘继和处，以4,600元出售给刘继和，刘继和

先支付给龚汉华 2,000 元现金。次日上午刘继和将该 2 只穿山甲送至新余出售给被告人郭秋民，双方谈好大的以每斤 800 元计算，小的以 2,000 元计算，经称重，大穿山甲重约 8 斤，郭秋民当场付给刘继和 8,000 元，之后刘继和将余款 2,600 元付给了龚汉华。郭秋民后将大的穿山甲宰杀并出售，小的穿山甲被他人以 1,000 元收购并放生。刘继和从中获利 3,400 元，龚汉华获利 4,600 元。

3.2021 年 5 月 10 日下午，被告人龚汉华捕获 1 只 10 斤多的穿山甲，并于当晚以每斤 500 元的价格出售给被告人刘继和，刘继和先转了 2,500 元给龚汉华，约定余款 2,500 元待售出后再付。次日，刘继和将该只穿山甲及 1 只死麂子送至郭秋民处，以每斤 800 元的价格将穿山甲出售给郭秋民。双方准备交易时，被公安民警抓获。该穿山甲及死麂子被公安民警当场查获并扣押。当日，龚汉华亦被抓获归案。2021 年 5 月 26 日，被告人刘小平到公安机关投案。

2021 年 5 月 14 日，被告人郭秋民妻子黄某将家中放置的重 1.99 斤的穿山甲甲片上交给了公安机关。经鉴定，上述被查获的穿山甲活体及甲片、爪子为中华穿山甲，属国家一级保护野生动物；被查获的死麂子为小麂，属“国家保护的有益的或者有重要经济、科学研究价值的陆生野生动物”（简称：三有动物）。被查获的活体穿山甲已由野生动物保护部门放生。

另，被告人刘小平退回违法所得4,000元，刘继明退回1,800元，该款已由修水县公安局扣押。

根据《野生动物及其制品价值评估方法》认定，1只国家一级保护野生动物穿山甲的价值为80,000元；1只小鹿的价值为3,000元。

二、被告人刘继明非法持有枪支、被告人林常富窝藏、非法持有枪支的事实

2021年5月13日凌晨2时左右，被告人刘继明得知公安民警在抓捕其，便在后山躲避。刘继明后电话告知被告人林常富说公安民警要抓其及其在后山躲避的情况，林常富便让刘继明在其家留宿一晚。次日早晨6时许，刘继明又将其持有的1只猎枪交给林常富保管，林常富后将该猎枪藏在自家的薯窖内。之后，林常富骑车将刘继明送至刘的丈母娘家。

2021年5月21日，被告人刘继明被抓获归案，同年5月23日，被告人林常富在家中被公安民警当场传唤到案。涉案猎枪已被公安机关查获并扣押。经鉴定，涉案的枪支物品为1支“鹰”牌12号撅把式单管制式猎枪，认定为枪支。刘继明、林常富均未办理持枪证。

在本案审理过程，对于附带民事公益诉讼起诉人的诉讼请求，被告人刘继和已主动履行5万元，被告人郭秋民主动履行8

万元，被告人刘继明主动履行2万元，被告人刘小平主动履行1.3万元。

上述事实，有经庭审质证、确认的被告人刘继和、郭秋民、龚汉华、刘继明、林常富的供述，被告人刘小平的供述，证人黄某、周某、刘某、邹某、鲁某的证言，辨认笔录，龚汉华、刘小平、林常富指认现场笔录及照片，搜查笔录及照片，扣押笔录及扣押清单，电子数据制作说明及涉案人员微信信息资料、涉案赃款微信转账记录，涉案野生动物科属、保护级别及价值司法鉴定意见，枪支鉴定书，扣押款票据，刘小平病历资料，查获的野生动物处置证明，无持枪资格证明，到案经过，郭秋民前科犯罪刑事判决书及执行通知书，人口信息，公益诉讼检察立案决定书，诉前公告，国家林业局令《野生动物及其制品价值评估方法》等证据证实，足以认定。

关于2021年2月17日被告人刘继和从被告人刘继明处收购穿山甲时是否先行支付1,000元货款的问题。经查，刘继和

多次供称其向刘继明先行支付了1,000元的事实，双方均供称当时是以每斤500元的价格进行交易的。涉案穿山甲重达14斤有余，按此计算交易价格应为7,000元左右，刘继和将穿山甲售出后付给了刘继明余款5,800元，其先行支付1,000元的情况更符合客观实际，且刘继和与龚汉华之间的2次交易也有先行支

付部分货款的情况，该行为符合刘继和与他人之间的交易习惯。故可以认定刘继和向刘继明先行支付了 1,000 元货款。

本院认为，被告人刘继和非法收购、出售国家一级保护野生动物穿山甲 4 只；被告人郭秋民非法收购国家一级保护野生动物穿山甲 4 只，并将其中 2 只穿山甲予以杀害；被告人龚汉华非法猎捕、出售国家一级保护野生动物穿山甲 3 只；被告人刘小平非法猎捕、出售国家一级保护野生动物穿山甲 1 只；被告人刘继明非法出售国家一级保护野生动物穿山甲 1 只，并非法持有制式猎枪 1 支；被告人林常富明知是犯罪的人而为其提供处所留宿，帮助其逃匿，明知是他人非法持有的枪支而予以保管、藏匿。对被告人刘继和、郭秋民、龚汉华、刘小平应当以危害珍贵、濒危野生动物罪定罪处罚；对被告人刘继明应当以危害珍贵、濒危野生动物罪、非法持有枪支罪定罪处罚；对被告人林常富应当以窝藏罪、非法持有枪支罪定罪处罚。公诉机关指控的罪名成立，本院予以确认。被告人刘继明、林常富一人犯数罪，应当数罪并罚。被告人郭秋民曾因犯罪被宣告缓刑，在判决宣告前及在缓刑考验期内犯本案所涉之罪，应当将本案所涉之罪判处的刑罚与前罪判处的刑罚并罚。在非法持有枪支犯罪中，被告人刘继明、林常富属共同犯罪，刘继明系主犯，林常富起辅助作用，系从犯，对林常富应从轻处罚。六被告人均认罪认罚，被告人刘小平具有自首情节，被告人刘继和、郭秋民、龚汉华、林常富具有坦白情节，

刘小平退回全部违法所得，刘继明退回部分违法所得，对各被告人可相应从轻处罚。

辩护人的相关辩护意见，本院予以采纳。被告人刘继明在到案之初拒不交代自己的罪行，直至第 5 次讯问过程中才交代，对其不应认定为坦白。

关于附带民事公益诉讼方面，刘小平猎捕、出售，刘继明出售，刘继和收购、出售，郭秋民收购、宰杀穿山甲，相关附带民事公益诉讼被告的行为直接结合导致 1 只穿山甲死亡的损害后果；龚汉华猎捕、出售，刘继和收购、出售，郭秋民收购、宰杀穿山甲，相关附带民事公益诉讼被告的行为直接结合导致另 1 只穿山甲死亡的损害后果；刘继和出售，郭秋民收购 1 只小麂，二附带民事公益诉讼被告的行为共同侵害了野生动物资源。相关附带民事公益诉讼被告的侵权行为共同破坏了国家所有的野生动物资源，侵害了生态环境，损害了国家利益和社会公共利益，属共同侵权行为，依法应对其导致的损害后果承担连带赔偿责任。附带民事公益诉讼起诉人的诉请依法有据，标的适当，本院予以支持。

鉴于被告人刘继和、郭秋民、刘继明、刘小平能够主动履行附带民事公益诉讼赔偿义务，可酌情从轻处罚。经本院提出意见，公诉机关将量刑建议调整为：被告人刘继和有期徒刑一年六个月，并处罚金；被告人郭秋民犯危害珍贵、濒危野生动物罪，判

处有期徒刑一年四个月，并处罚金，与前罪有期徒刑一年，罚金五千元并罚，合并执行有期徒刑二年，并处罚金；被告人刘继明犯危害珍贵、濒危野生动物罪，判处有期徒刑八个月，并处罚金，犯非法持有枪支罪，判处有期徒刑八个月，合并执行有期徒刑十个月，并处罚金；被告人刘小平有期徒刑七个月，缓刑一年，并处罚金；被告人林常富犯窝藏罪，判处拘役六个月，犯非法持有枪支罪，判处拘役五个月，合并执行拘役七个月。公诉机关的量刑建议适当，本院予以采纳。

综上，依照《中华人民共和国刑法》第三百四十一条第一

款、第一百二十八条第一款、第三百一十条第一款、第七十七条第一款、第六十九条、第二十五条第一款、第二十六条、第二十七条、第六十七条第一款、第三款、第七十二条第一款、第三款、第七十三条第二款、第三款、第三十六条、第六十四条，《中华人民共和国民法典》第九条、第一百二十条、第一百七十九条、第一百八十七条、第二百三十八条、第二百五十一条、第一千一百六十八条、第一千二百二十九条、第一千二百三十四条、第一千二百三十五条，《中华人民共和国野生动物保护法》第三条，《中华人民共和国刑事诉讼法》第一百零一条第二款、第一百零三条、第二百零一条，《最高人民法院、最高人民检察院关于检察公益诉讼案件适用法律若干问题的解释》第二十条之规定，判决如下：

一、被告人刘继和犯危害珍贵、濒危野生动物罪，判处有期徒刑一年六个月，并处罚金人民币一万元。

（刑期从判决执行之日起计算。判决执行以前先行羁押的，羁押一日折抵刑期一日，即2021年5月12日起至2022年11月11日止。罚金于本判决生效之日起一个月内缴纳。）

二、撤销江西省新余市渝水区人民法院（2021）赣0502刑初26号刑事判决主文中被告人郭秋民犯危害珍贵、濒危野生动物罪，判处有期徒刑一年，缓刑二年，并处罚金人民币五千元的缓刑部分。

被告人郭秋民犯危害珍贵、濒危野生动物罪，判处有期徒刑一年四个月，并处罚金人民币一万元，与前罪判处的有期徒刑一年，罚金五千元并罚，决定执行有期徒刑二年，并处罚金人民币一万五千元。

（刑期从判决执行之日起计算。判决执行以前先行羁押的，羁押一日折抵刑期一日，即2021年5月12日起至2023年1月29日止，已折抵因前罪羁押的3个月零13日。罚金于本判

决生效之日起一个月内缴纳。）

三、被告人龚汉华犯危害珍贵、濒危野生动物罪，判处有期徒刑一年六个月，并处罚金人民币八千元。

（刑期从判决执行之日起计算。判决执行以前先行羁押的，羁押一日折抵刑期一日，即 2021 年 5 月 12 日起至 2022 年 11 月 11 日止。罚金于本判决生效之日起一个月内缴纳。）

四、被告人刘继明犯危害珍贵、濒危野生动物罪，判处有期徒刑八个月，并处罚金人民币四千元；犯非法持有枪支罪，判处有期徒刑八个月；决定执行有期徒刑十个月，并处罚金人民币四千元。

（刑期从判决执行之日起计算。判决执行以前先行羁押的，羁押一日折抵刑期一日，即 2021 年 5 月 21 日起至 2022 年 3 月 20 日止。罚金于本判决生效之日起一个月内缴纳。）

五、被告人刘小平犯危害珍贵、濒危野生动物罪，判处有期徒刑七个月，缓刑一年，并处罚金人民币四千元。

（缓刑考验期，自判决确定之日起计算。罚金于本判决生效之日起一个月内缴纳。）

六、被告人林常富犯窝藏罪，判处拘役六个月；犯非法持有枪支罪，判处拘役五个月；决定执行拘役七个月。

（刑期从判决执行之日起计算。判决执行以前先行羁押的，羁押一日折抵刑期一日，即 2021 年 5 月 23 日起至 2021 年 12 月 22 日止。）

七、由被告人刘继和、郭秋民连带赔偿野生动物资源损失 163,000 元，被告人龚汉华对其中 1 只穿山甲的损失 80,000 元承担连带赔偿责任，被告人刘继明、刘小平对其中另 1 只穿山甲

的损失 80,000 元承担连带赔偿责任。（案件款 163,000 元已由被告人刘继和、郭秋民、刘继明、刘小平汇入指定的江西思华生态环境保护基金会账户）

八、对扣押在案的相关被告人退缴的违法所得即刘继明 1,800 元、刘小平 4,000 元予以没收，由扣押机关修水县公安局森林分局上缴国库；继续追缴相关被告人的违法所得即刘继和 8,450 元、龚汉华 7,100 元、刘继明 1,000 元；对扣押在案的穿山甲甲片、涉案枪支予以没收，由扣押机关修水县公安局森林分局依法处置。

如不服本判决，可在接到判决书的第二日起十日内，通过本院或者直接向江西省九江市中级人民法院提出上诉。书面上诉的，应当提交上诉状正本一份，副本四份。

审　判　长　　郑由平

人民陪审员　　卢作旺

人民陪审员　　陈沾雄

二〇二一年十一月二十三日

书　记　员　　肖　强
