# Supplementary material for: China’s Legal Protection System for Pangolins: Past, Present, and Future
Source: Animals (Basel). 2025 Aug 18;15(16):2422. doi: 10.3390/ani15162422 (PMC12383201; doi:10.3390/ani15162422)
Supplement: Supplementary file 1 [file animals-15-02422-s001.zip › Supplementary Material S4-Full Text of Judgments in Pangolin-Related Public Interest Litigation Cases in China/【9】阙玺保、广西壮族自治区东兴市人民检察院非法收购、运输、出售珍贵、濒危野生动物、珍贵、濒危野生动物制品罪刑事一审刑事判决书.pdf]

阙玺保、广西壮族自治区东兴市人民检察院非法收购、运输、出售珍贵、濒危野生动物、珍贵、濒危野生动物制品罪刑事一审刑事判决书

广西壮族自治区东兴市人民法院

刑 事 附 带 民 事 判 决 书

（2021）桂 0681 刑初 159 号

公诉机关暨附带民事公益诉讼起诉人广西壮族自治区东兴市人民检察院。

被告人暨附带民事公益诉讼被告阙玺保，男，1984 年 11 月 25 日出生，籍贯江西省抚州市乐安县，汉族，初中文化，无业，户籍住址乐安县，住广西东兴市，因涉嫌犯危害珍贵、濒危野生动物罪于 2021 年 3 月 11 日被刑事拘留，同年 4 月 13 日被逮捕。现羁押于东兴市看守所。

辩护人莫玮峰，广西北仑律师事务所律师。

东兴市人民检察院以东检刑诉〔2021〕116 号起诉书指控被告人阙玺保犯危害珍贵、濒危野生动物罪一案，于 2021 年 7 月 14 日向本院提起公诉，并以东检刑附民公诉〔2021〕10 号刑事附带民事公益诉讼起诉书就阙玺保非法收购、运输珍贵、濒危野生动物制品损害社会公共利益的行为向本院提起刑事附带民事公益诉讼。经查，东兴市人民检察院于同年 4 月 27 日公告了该案相关情况，公告期内未有法律规定的机关或有关组织提起民事公益诉讼。本院受理后，依法组成合

议庭，于同年9月1日公开开庭审理了本案。东兴市人民检察院指派检察员杨荣虎出庭支持公诉，指派检察员杨媚出庭履行职务，被告人暨附带民事公益诉讼被告阙玺保及其辩护人莫玮峰到庭参加诉讼。本案现已审理终结。

东兴市人民检察院指控:2021年3月10日，民警在被告人阙玺保位于东兴市的住处，查获阙玺保购买的疑似象牙制品1批（手镯1个、圆牌2块、吊坠1个、项链1根）和疑似穿山甲鳞片4片等物品。经鉴定，涉案的疑似穿山甲鳞片4片均为鳞甲目鲮鲤科／穿山甲科穿山甲属动物鳞片所有，共重0.026千克，属于国家一级保护野生动物，被列入《濒危野生动植物国际贸易公约》（ＣＩＴＥＳ）（以下简称ＣＩＴＥＳ）附录Ⅰ、附录Ⅱ，经济价值64000元。涉案的疑似象牙制品均为亚洲象或非洲象特有，共重0.191千克，属于国家一级保护野生动物，被列入ＣＩＴＥＳ附录Ⅰ、附录Ⅱ，经济价值7958.40元。

2021年3月，“阿光”（另案处理）联系被告人阙玺保帮邮寄羊角。阙玺保同意并让“阿光”联系韦贵颜（另案处理），由韦贵颜来邮寄。随后“阿光”与韦贵颜对接邮寄羊角事宜。3月5日，韦贵颜将4根疑似羚羊角伪装在装有越南食品的包裹后通过邮政快递寄出。同日民警扣押该包裹，并从该包裹查获4根疑似羊角物品。后阙玺保从“阿光”处获利400元。经鉴定，涉案的4根疑似羚羊角均为牛科高鼻羚羊角产品，分别重270克、260克、250克、220克，属

于国家一级保护野生动物，被列入 CITES 附录Ⅱ，经济价值 32 万元。

2021 年 3 月 4 日，“阿光”再次联系阙玺保帮邮寄两根犀牛角制品，并承诺给阙玺保 800 元费用。阙玺保同意后安排韦贵颜邮寄，并将收件人信息发给韦贵颜。韦贵颜拿到 2 根犀牛角后发照片给阙玺保确认，阙玺保确认后让韦贵颜将犀牛角伪装放入越南食品的包裹后通过邮政快递寄出。期间，阙玺保还联系邮政工作人员查询该包裹进度。后该包裹因故被退回，阙玺保到中国邮政集团广西东兴分公司领取该包裹时被民警抓获。经检查，该包裹内装若干食品和 2 根疑似犀牛角。经鉴定，涉案的疑似犀牛角 2 根均为犀角产品，共重 1.967 千克，被列入 CITES 附录Ⅰ、附录Ⅱ中，经济价值 491750 元。

公诉机关指控上述事实提供了相应的证据予以证明，并认为，被告人阙玺保非法收购国家重点保护的珍贵、濒危野生动物制品；受他人指使，非法运输国家重点保护的珍贵、濒危野生动物制品，情节特别严重，其行为触犯了《中华人民共和国刑法》第三百四十一条第一款之规定，应当以危害珍贵、濒危野生动物罪追究其刑事责任。阙玺保在帮助他人运输国家重点保护的珍贵、濒危野生动物制品的共同犯罪中起次要作用，是从犯，应当从轻或者减轻处罚；其到案后如实供述自己的罪行，可以从轻处罚；其认罪认罚，可以从宽处理。综上，建议对被告人阙玺保判处有期徒刑五年六个月

至六年，并处罚金人民币五万元至七万元。提请本院依法判处。

附带民事公益诉讼起诉人东兴市人民检察院向本院提出诉讼请求：1.请求判令被告阙玺保依法承担赔偿生态资源受损费用883708.4元；2.请求判令被告阙玺保对其侵权行为当庭进行公开赔礼道歉。

被告人暨附带民事公益诉讼被告阙玺保对指控的犯罪事实与罪名均无异议，自愿认罪认罚，对公益诉讼起诉人的诉讼请求无异议，并当庭进行了赔礼道歉。

辩护人提出如下辩护意见：1.阙玺保是从犯，可以从轻或者减轻处罚；2.指控运输羚羊角的犯罪事实是由韦贵颜与“阿光”直接对接，且是韦贵颜获利，应对阙玺保在量刑上有所体现；3.阙玺保在本案中获利较少，自愿认罪认罚。综上，建议对阙玺保从轻处罚。

经审理查明：一、2021年3月，同案人“阿光”（另案处理）联系被告人阙玺保帮忙邮寄羊角。阙玺保同意后，让“阿光”联系其女友同案人韦贵颜（另案处理）邮寄。随后“阿光”与韦贵颜对接邮寄羊角事宜。同月5日，韦贵颜将4根疑似羚羊角伪装放入装有越南食品的包裹内，后通过邮政快递寄出。同日民警扣押该包裹，并从该包裹内查获4根疑似羊角物品。阙玺保从“阿光”处获取报酬400元。经鉴定，上述查获的4根疑似羚羊角均

为牛科高鼻羚羊角产品，属于国家一级保护野生动物，被列入CITES 附录Ⅱ，共净重 1 千克，经济价值 32 万元。

二、2021 年 3 月 4 日，同案人“阿光”再次联系阙玺保帮忙邮寄 2 根犀牛角制品，并承诺给阙玺保报酬 800 元。阙玺保同意后安排同案人韦贵颜邮寄，并将收件人信息发给韦贵颜。韦贵颜拿到 2 根犀牛角后发照片给阙玺保确认，阙玺保确认后让韦贵颜将犀牛角伪装放入装有越南食品的包裹内，后通过邮政快递寄出。期间，阙玺保还联系邮政工作人员查询该包裹邮寄进度。同月 10 日，阙玺保到中国邮政集团广西东兴分公司领取因故被退回的该包裹时被民警抓获。经检查，该包裹内装若干食品和 2 根疑似犀牛角。经鉴定，上述查获的 2 根疑似犀牛角均为犀角产品，被列入 CITES 附录Ⅰ、附录Ⅱ中，共净重 1.967 千克，经济价值491750 元。

三、2021 年 3 月 10 日，民警抓获被告人阙玺保后，从其位于广西东兴市的住处中，查获阙玺保购买的疑似象牙手镯 1 个、圆牌 2 块、吊坠 1 个、项链 1 根和疑似穿山甲鳞片 4 片等物品。经鉴定，上述查获的疑似穿山甲鳞片 4 片均为鳞甲目鲮鲤科／穿山甲科穿山甲属动物鳞片产品，属于国家一级保护野生动物，被列入《濒危野生动植物国际贸易公约》（ＣＩＴＥＳ）附录Ⅰ、附录Ⅱ中，共净重 0.026 千克，经济价值 64000 元；上述查获的疑似象牙制品均为象牙产品，为亚洲象或非洲象特有，属于国家一级保护野生动物，被列入ＣＩＴＥＳ附录Ⅰ、附录Ⅱ中，共重

0.191 千克，经济价值 7958.40 元。

上述事实，有公诉机关暨附带民事公益诉讼起诉人提供并经法庭举证、质证的受案登记表、立案决定书、常住人口信息表、查获经过、搜查笔录、扣押清单、提取送检笔录、调取证据清单、接受证据清单、快递单据照片、出入境记录、收据、开户信息及通话记录、开户信息及银行流水、情况说明等书证，物种鉴定书、价格认定结论书，证人陆某、严某、张某的证言，被告人阙玺保的供述和辩解，勘查、辨认笔录及照片，视频侦查截图及情况说明、微信聊天记录截图、电子数据检查笔录及公益诉讼诉前程序材料等证据予以证明。上述证据来源合法，内容客观、真实，与本案存在关联性，能相互印证，已形成完整的证明体系，且均经庭审举证、质证属实，本院予以确认。

本院认为，被告人阙玺保非法收购穿山甲鳞片、象牙制品；受人指使非法运输珍贵、濒危野生动物制品羚羊角、犀牛角，情节特别严重，其行为已构成危害珍贵、濒危野生动物罪。公诉机关指控的罪名成立。被告人阙玺保在第一、二起共同犯罪中起次要作用，是从犯，应当从轻或者减轻处罚；其到案后如实供述，可以从轻处罚；其认罪认罚，可以从宽处理。辩护人所提阙玺保具有从犯、坦白、认罪认罚等情节的辩护意见成立，本院予以采纳。综上，本院决定对被告人阙玺保减轻处罚，公诉机关量刑建议适当，本院予以采纳。随案移送的假身份证（杨晓峰）1 张、苹果手机（无卡）1

部，是违禁品或作案工具，依法应予没收；随案移送的身份证（阙玺保、阙乐保）2张、往来港澳通行证1本、护照1本、收据7张、机动车驾驶证1本、华为ｎｏｖａ6手机1部、苹果手机（尾号0404）1部、ＯＰＰＯ手机1部、黑色直板手机1部、银行卡22张及扣押在案的“桂Ｐ×××**”轿车1辆，均与本案无关，依法应予返还；阙玺保未退出的违法所得人民币400元，依法应予继续追缴。

穿山甲、犀、象、牛科高鼻羚羊作为纳入《濒危野生动植物种国际贸易公约》附录Ⅰ、Ⅱ保护的珍贵、濒危野生动物，是宝贵的自然资源，除经济价值外，还具有内在不可估量的生态、科研、社会、遗传资源等价值，有公共利益属性，我国系上述国际公约的缔约国，有履行国际公约共同保护地球生态资源的责任。被告阙玺保非法收购、运输上述珍贵、濒危野生动物制品的行为，损害了公共利益，依法应承担民事侵权责任。东兴市人民检察院在履行刑事案件审查起诉职能中发现本案线索，依照法定程序公告，后提起刑事附带民事公益诉讼符合法律规定的条件和程序，是依法维护社会公共利益的一种方式，主体适格；要求阙玺保赔偿因非法收购、运输珍贵、濒危野生动物制品导致野生动物资源损失承担生态资源受损费用，并在当庭进行公开赔礼道歉的诉讼请求于法有据，本院予以支持。被告人阙玺保在开庭时已当庭进行公开赔礼道歉。

综上，为打击犯罪，保护生态资源，修复野生动物自然

资源受到的损害，依照《中华人民共和国刑法》第三百四十一条第一款、第二十五条第一款、第二十七条、第五十二条、第五十三条、第六十三条第一款、第六十四条、第六十七条第三款，《中华人民共和国民法典》第一百七十九条第（八）项、第（十一）项及第三款、第一百八十七条、第一千二百二十九条、第一千二百三十五条，《中华人民共和国刑事诉讼法》第十五条、第一百零一条第二款、第二百零一条，《中华人民共和国民事诉讼法》第五十五条之规定，判决如下：

一、被告人阙玺保犯危害珍贵、濒危野生动物罪，判处有期徒刑五年九个月，并处罚金人民币五万元；

（刑期从判决执行之日起计算。判决执行以前先行羁押的，羁押一日折抵刑期一日。刑期即自2021年3月11日起至2026年12月10日止。罚金限于本判决生效后一个月内缴纳，逾期不缴纳的，强制缴纳。）

二、随案移送的假身份证（杨晓峰）一张、苹果手机（无卡）一部，予以没收；身份证（阙玺保、阙乐保）二张、往来港澳通行证一本、护照一本、收据七张、机动车驾驶证一本、华为ｎｏｖａ6手机一部、苹果手机（尾号0404）一部、ＯＰＰＯ手机一部、黑色直板手机一部、银行卡二十二张及扣押在案的“桂Ｐ×××**”轿车一辆，返还被告人阙玺保；

三、继续追缴被告人阙玺保未退出的违法所得人民币四百元，上缴国库；

四、附带民事公益诉讼被告阙玺保赔偿生态资源受损费用人民币八十八万三千七百零八元四角；

五、附带民事公益诉讼被告阙玺保就其侵权行为当庭公开赔礼道歉。（已当庭履行）

如不服本判决，可在接到判决书的第二日起十日内，通过本院或者直接向广西壮族自治区防城港市中级人民法院提出上诉。书面上诉的，应当提交上诉状正本一份，副本八份。

审　判　长　　龙　剑

审　判　员　　陆淑芸

审　判　员　　杨　玲

人民陪审员　　王以强

人民陪审员　　黄　鑫

人民陪审员　　王伦英

人民陪审员　　王　达

二

〇

二

一

年

九

月

二

十

三

日

书记员刘华光

附相关法律条文：

《中华人民共和国刑法》

第三百四十一条非法猎捕、杀害国家重点保护的珍贵、濒危野生动物的，或者非法收购、运输、出售国家重点保护的珍贵、濒危野生动物及其制品的，处五年以下有期徒刑或者拘役，并处罚金；情节严重的，处五年以上十年以下有期徒刑，并处罚金；情节特别严重的，处十年以上有期徒刑，并处罚金或者没收财产。

违反狩猎法规，在禁猎区、禁猎期或者使用禁用的工具、方法进行狩猎，破坏野生动物资源，情节严重的，处三年以下有期徒刑、拘役、管制或者罚金。

违反野生动物保护管理法规，以食用为目的非法猎捕、收购、运输、出售第一款规定以外的在野外环境自然生长繁殖的陆生野生动物，情节严重的，依照前款的规定处罚。

第二十五条共同犯罪是指二人以上共同故意犯罪。

二人以上共同过失犯罪，不以共同犯罪论处；应当负刑事责任的，按照他们所犯的罪分别处罚。

第二十七条在共同犯罪中起次要或者辅助作用的，是从犯。

对于从犯，应当从轻、减轻处罚或者免除处罚。

第五十二条判处罚金，应当根据犯罪情节决定罚金数额。

第五十三条罚金在判决指定的期限内一次或者分期缴纳。期满不缴纳的，强制缴纳。对于不能全部缴纳罚金的，人民法院在任何时候发现被执行人有可以执行的财产，应当随时追缴。

由于遭遇不能抗拒的灾祸等原因缴纳确实有困难的，经人民法院裁定，可以延期缴纳、酌情减少或者免除。

第六十三条犯罪分子具有本法规定的减轻处罚情节的，应当在法定刑以下判处刑罚;本法规定有数个量刑幅度的，应当在法定量刑幅度的下一个量刑幅度内判处刑罚。

犯罪分子虽然不具有本法规定的减轻处罚情节，但是根据案件的特殊情况，经最高人民法院核准，也可以在法定刑以下判处刑罚。

第六十四条犯罪分子违法所得的一切财物，应当予以追缴或者责令退赔；对被害人的合法财产，应当及时返还；违禁品和供犯罪所用的本人财物，应当予以没收。没收的财物和罚金，一律上缴国库，不得挪用和自行处理。

第六十七条犯罪以后自动投案，如实供述自己的罪行的，是自首。对于自首的犯罪分子，可以从轻或者减轻处罚。其中，犯罪较轻的，可以免除处罚。

被采取强制措施的犯罪嫌疑人、被告人和正在服刑的罪犯，如实供述司法机关还未掌握的本人其他罪行的，以自首论。

犯罪嫌疑人虽不具有前两款规定的自首情节，但是如实

供述自己罪行的，可以从轻处罚；因其如实供述自己罪行，避免特别严重后果发生的，可以减轻处罚。

《中华人民共和国民法典》

第一百七十九条承担民事责任的方式主要有：

（一）停止侵害；

（二）排除妨碍；

（三）消除危险；

（四）返还财产；

（五）恢复原状；

（六）修理、重作、更换；

（七）继续履行；

（八）赔偿损失；

（九）支付违约金；

（十）消除影响、恢复名誉；

（十一）赔礼道歉。

法律规定惩罚性赔偿的，依照其规定。

本条规定的承担民事责任的方式，可以单独适用，也可以合并适用。

第一百八十七条民事主体因同一行为应当承担民事责任、行政责任和刑事责任的，承担行政责任或者刑事责任不影响承担民事责任；民事主体的财产不足以支付的，优先用于承担民事责任。

第一千二百二十九条因污染环境、破坏生态造成他人损害的，侵权人应当承担侵权责任。

第一千二百三十五条违反国家规定造成生态环境损害的，国家规定的机关或者法律规定的组织有权请求侵权人赔偿下列损失和费用：

（一）生态环境受到损害至修复完成期间服务功能丧失导致的损失；

（二）生态环境功能永久性损害造成的损失；

（三）生态环境损害调查、鉴定评估等费用；

（四）清除污染、修复生态环境费用；

（五）防止损害的发生和扩大所支出的合理费用。

《中华人民共和国刑事诉讼法》

第十五条犯罪嫌疑人、被告人自愿如实供述自己的罪行，承认指控的犯罪事实，愿意接受处罚的，可以依法从宽处理。

第一百零一条被害人由于被告人的犯罪行为而遭受物质损失的，在刑事诉讼过程中，有权提起附带民事诉讼。被害人死亡或者丧失行为能力的，被害人的法定代理人、近亲属有权提起附带民事诉讼。

如果是国家财产、集体财产遭受损失的，人民检察院在提起公诉的时候，可以提起附带民事诉讼。

第二百零一条对于认罪认罚案件，人民法院依法作出

判决时，一般应当采纳人民检察院指控的罪名和量刑建议，但有下列情形的除外：

（一）被告人的行为不构成犯罪或者不应当追究其刑事责任的；

（二）被告人违背意愿认罪认罚的；

（三）被告人否认指控的犯罪事实的；

（四）起诉指控的罪名与审理认定的罪名不一致的；

（五）其他可能影响公正审判的情形。

人民法院经审理认为量刑建议明显不当，或者被告人、辩护人对量刑建议提出异议的，人民检察院可以调整量刑建议。人民检察院不调整量刑建议或者调整量刑建议后仍然明显不当的，人民法院应当依法作出判决。

《中华人民共和国民事诉讼法》

第五十五条对污染环境、侵害众多消费者合法权益等损害社会公共利益的行为，法律规定的机关和有关组织可以向人民法院提起诉讼。

人民检察院在履行职责中发现破坏生态环境和资源保护、食品药品安全领域侵害众多消费者合法权益等损害社会公共利益的行为，在没有前款规定的机关和组织或者前款规定的机关和组织不提起诉讼的情况下，可以向人民法院提起诉讼。前款规定的机关或者组织提起诉讼的，人民检察院可以支持起诉。
