# Supplementary material for: China’s Legal Protection System for Pangolins: Past, Present, and Future
Source: Animals (Basel). 2025 Aug 18;15(16):2422. doi: 10.3390/ani15162422 (PMC12383201; doi:10.3390/ani15162422)
Supplement: Supplementary file 1 [file animals-15-02422-s001.zip › Supplementary Material S1-Full Texts of Laws and Regulations Related to Pangolins in China.pdf]

Supplementary Material S1

Full-Text Links to Laws and Regulations Related to Pangolins in China

No.	**Legal/Regulatory Document Title**	**Effective Date**	**Full-Text Link**
1	Instruction of the State Council on Actively Protecting and Rationally Utilizing Wildlife Resources	14 Sept. 1962	https://www.pkulaw.com/chl/fd3fd92c975c9709bdfb.html
2	Notice on Traditional Chinese Medicine Work Issues	13 Oct. 1983	https://www.pkulaw.com/chl/b4135a8e00b7b656bdfb.html
3	Administrative Measures for Forest and Wildlife Nature Reserves	1 June 1985	https://www.pkulaw.com/chl/5aff92d598fafb1abdfb.html
4	National Key Protected Wild Medicinal Species List	30 Oct. 1987	https://www.pkulaw.com/chl/934d0f9c50222f04bdfb.html
5	List of Protected Wild Animals	14 Jan. 1989	https://www.pkulaw.com/chl/00af3eb30019ee7fbdfb.html
6	Wild Animal Conservation Law of the People's Republic of China (amended five times in 2004, 2009, 2016, 2018, 2022)	1 Mar. 1989	https://www.pkulaw.com/chl/53181cd1be67b8f3bdfb.html
7	Circular on Illegal Hunting and Trade of Rare Wild Animals	12 May 1990	https://www.pkulaw.com/chl/3bc75627395a42eebdfb.html
8	Letter from the Ministry of Forestry Requesting Assistance in Managing the Export of Proprietary Chinese Medicines Containing Wild Animal Medicinal Ingredients	15 June 1990	https://www.pkulaw.com/chl/aa53868480a36518bdfb.html
9	Regulation of the People's Republic of China on the Protection of Terrestrial Wild Animals (revised in 2011, 2016)	1 Mar. 1992	https://www.pkulaw.com/chl/97063bb58916bf17bdfb.html
10	Circular of the General Office of the State Council on Transmitting the Ministry of Forestry's Report on Strengthening Wild Animal Protection and Management	23 Oct. 1991	https://www.pkulaw.com/chl/319b8a2cb6160961bdfb.html

11	List of Animals, Animal Products, and Other Quarantine Objects Prohibited from Being Carried or Mailed into the People's Republic of China	8 June 1992	https://www.pkulaw.com/chl/b9f116d2405ba40dbdfb.html
12	Measures for Charging Management Fees for Terrestrial Wild Animal Resource Protection	1 Jan. 1993	https://www.pkulaw.com/chl/728a40ea7d198132bdfb.html
13	Standards for Filing Cases of Illegal Poaching of National Key Protected Precious and Endangered Terrestrial Wild Animals	9 May 2001	https://www.pkulaw.com/chl/cac7d7e46bb40781bdfb.html
14	Notice of the General Office of the State Council on Strengthening the Protection and Management of Biological Species Resources	1 Mar. 2004	https://www.pkulaw.com/chl/6dc1ac491df9a23cbdfb.html
15	Guiding Opinions of the State Forestry Administration on Promoting Sustainable Development of Wild Animals and Plants	1 Sept. 2004	https://www.pkulaw.com/chl/b2ff601af33196a1bdfb.html
16	National Plan for Biological Species Resource Protection and Utilization	24 Oct. 2007	https://www.pkulaw.com/chl/6314ef96413d5c4ebdfb.html
17	Notice on Strengthening the Protection of Saiga Antelope, Pangolins, Rare Snakes, and Standardizing the Management of Their Products for Medicinal Use	12 Nov. 2007	https://www.pkulaw.com/lar/429200ff5e475fc7cbe5a97df02fe9b4bdfb.html
18	Announcement No. 8 of 2015 by the State Forestry Administration — Announcement on Matters Concerning Special Identification for the Management of Wild Animal Business and Utilization in China	28 Apr. 2015	https://www.pkulaw.com/chl/dedb73b0d6b7c9dbbdfb.html
19	Pharmacopoeia of the People's Republic of China	5 June 2015	https://www.pkulaw.com/chl/4a3fef91b94b525ebdfb.html

20	Notice of the State Administration for Market Regulation and the National Forestry and Grassland Administration on Jointly Carrying Out Special Rectification Actions for Wild Animal Protection	24 May 2019	https://www.pkulaw.com/chl/c0a721c64f63b423bdfb.html
21	Decision of the Standing Committee of the National People's Congress to Comprehensively Prohibit the Illegal Trade of Wild Animals, Break the Bad Habit of Excessive Consumption of Wild Animals, and Effectively Secure the Life and Health of the People	4 Feb. 2020	https://www.pkulaw.com/chl/cab9fc867524bdcebdfb.html
22	Chinese Pharmacopoeia	May 2020	https://www.pkulaw.com/chl/aa00daaeb5a4fe4ebdfb.html
23	Announcement on Adjusting the Protection Level of Pangolins	3 June 2020	https://www.pkulaw.com/chl/96b37ba9db189a02bdfb.html
24	Notice of the National Forestry and Grassland Administration, the National Administration of Traditional Chinese Medicine, and the National Medical Products Administration on Effectively Strengthening Pangolin Protection and Management	13 Nov. 2024	https://www.pkulaw.com/chl/37e628d0fc403bafbdfb.html
