# Supplementary material for: China’s Legal Protection System for Pangolins: Past, Present, and Future
Source: Animals (Basel). 2025 Aug 18;15(16):2422. doi: 10.3390/ani15162422 (PMC12383201; doi:10.3390/ani15162422)
Supplement: Supplementary file 1 [file animals-15-02422-s001.zip › Supplementary Material S3- Full-Text Links to Judgments in Pangolin-Related Public Interest Litigation Cases in China.pdf]

Supplementary Material S3

Full-Text Links to Judgments in Pangolin-Related Public Interest Litigation Cases in China

No.	Case No./Date	Full-Text Judgment Link
1	(2020) Jing 04 Criminal First Instance No. 25	https://rmfyalk.court.gov.cn/view/content.html?id=z8iYo2lHuugRJEIL2UzCq%252F%252FMZ9hz3dsvVj6256d1tz8%253D&lib=ck&qw=%E7%A9%BF%E5%B1%B1%E7%94%B2
2	(2021) Gui 14 Criminal First Instance No. 79	https://rmfyalk.court.gov.cn/view/content.html?id=nVyn%252BhdCj1PK7cM2piPJBrdAbvGmByljWtMK7gnPKy4%253D&lib=ck&qw=穿山甲
3	(2023) Yun 3123 Criminal First Instance No. 516	https://wenshu.court.gov.cn/website/wenshu/181107ANFZ0BXSK4/index.html?docId=V3D3+2vF8WK320Ve09G9E35u9gtUzhDywFQqbeqTbok7JWnb65QVip/dgBYosE2gNO3IcCbk7GGr4PEcpIwkZ7+a3weUNSbCt4Hw4I001PEPk/YAixlaSCzDTIrm0BDH
4	(2023) Yue 1223 Criminal First Instance No. 80	https://wenshu.court.gov.cn/website/wenshu/181107ANFZ0BXSK4/index.html?docId=KGJATdnHty/ZUWirdQkAoPXbEOr/01IRl0lYjmeQBBVg+XuPhjAazZ/dgBYosE2gNO3IcCbk7GGr4PEcpIwkZ7+a3weUNSbCt4Hw4I001PH6Khe2g3MZY4Z9vwf69xFz
5	(2022) Gui 0681 Criminal First Instance No. 3	https://wenshu.court.gov.cn/website/wenshu/181107ANFZ0BXSK4/index.html?docId=5VrccDkf9DWiyVhK5VaWHi3JZbgNiVqNJS04qr8c0h0EaqhRlzOSOp/dgBYosE2gNO3IcCbk7GGr4PEcpIwkZ7+a3weUNSbCt4Hw4I001PH6Khe2g3MZYxnwqtviM5rh
6	(2022) Liao 0112 Criminal First Instance No. 350	Pkulaw.com/CLI.C.500370317
7	(2021) Gui 0681 Criminal First Instance No. 248	https://wenshu.court.gov.cn/website/wenshu/181107ANFZ0BXSK4/index.html?docId=/Wjkce84V7QOl+x4y8OKNVqy8bV2DBnvQB33+9T8d4QJvk12+kLjO5/dgBYosE2gNO3IcCbk7GGr4PEcpIwkZ7+a3weUNSbCt4Hw4I001PH6Khe2g3MZY3WIE/fropj5
8	(2021) Gan 0424 Criminal First Instance No. 397	https://wenshu.court.gov.cn/website/wenshu/181107ANFZ0BXSK4/index.html?docId=sn9LzUg2BATdDhHbISStmxYXksqui5zIG48fudhHXnz9sl+rCAbed5/dgBYosE2gNO3IcCbk7GGr4PEcpIwkZ7+a3weUNSbCt4Hw4I001PEZWFQM9Ufp60h3Z6y07PzQ
9	(2021) Gui 0681 Criminal First Instance No. 159	https://wenshu.court.gov.cn/website/wenshu/181107ANFZ0BXSK4/index.html?docId=GkmKHoK+rz9hGlLTTJU/UA0eM6CSspMEchw0TZhH3FHR4gVuLRYbp5/dgBYosE2gNO3IcCbk7GGr4PEcpIwkZ7+a3weUNSbCt4Hw4I001PEZWFQM9Ufp6wyau86Cn8E/
10	(2020) Yue 01 Civil First Instance No. 1868	https://wenshu.court.gov.cn/website/wenshu/181107ANFZ0BXSK4/index.html?docId=uAs45pMi7XVBeMogk8f7GVrWgHag+/kxLVoL1wBGVf1W0JNSN+VDy5/dgBYosE2gNO3IcCbk7GGr4PEcpIwkZ7+a3weUNSbCt4Hw4I001PEZWFQM9Ufp65QEnD9vPuNt

11	(2021) Yue 01 Civil First Instance No. 576	https://wenshu.court.gov.cn/website/wenshu/181107ANFZ0BXSK4/index.html?docId=b7S8tNnGLPFkqLuIP/dgC21e9wIqn9imEP5HycAHpiS4jpVL2Si0bJ/dgBYosE2gNO3IcCbk7GGr4PEcpIwkZ7+a3weUNSbCt4Hw4I001PG63jZALrIjFrozdb+6oiVx
12	(2021) Yue 01 Civil First Instance No. 577	https://wenshu.court.gov.cn/website/wenshu/181107ANFZ0BXSK4/index.html?docId=XLrP06vRhcGToHUwu0GV/oqsWcnFi/J8AWotSqv1IN5b1mUYZLJBY5/dgBYosE2gNO3IcCbk7GGr4PEcpIwkZ7+a3weUNSbCt4Hw4I001PGjYHyHRieSKRAEjhYxQLJv
13	(2021) Yue 1403 Criminal First Instance No. 165	https://wenshu.court.gov.cn/website/wenshu/181107ANFZ0BXSK4/index.html?docId=vUmy3C2ryol5nXA9FUQ3gDPjTsu1xwMDwTcLrQWGNH4TqfkSbkovbJ/dgBYosE2gNO3IcCbk7GGr4PEcpIwkZ7+a3weUNSbCt4Hw4I001PGjYHyHRieSKZYRnFQW1dgi
14	(2021) Gui 0621 Criminal First Instance No. 53	https://wenshu.court.gov.cn/website/wenshu/181107ANFZ0BXSK4/index.html?docId=TuNipREbHBJX7r+1EFKiPh+csxhcpubIuFRIG5QH3T8MapoDEjE5ZZ/dgBYosE2gNO3IcCbk7GGr4PEcpIwkZ7+a3weUNSbCt4Hw4I001PGMKk6+wIozpYS5cARODmFq
15	(2021) Gui 0821 Criminal First Instance No. 78	https://wenshu.court.gov.cn/website/wenshu/181107ANFZ0BXSK4/index.html?docId=GkmKHoK+rz+4WiStnVE6QxZYilBhDsiUoHUd6nE3nZoBb8orOXlU4Z/dgBYosE2gNO3IcCbk7GGr4PEcpIwkZ7+a3weUNSbCt4Hw4I001PGMKk6+wIozpWUsNTEv8BwV
16	(2020) Gui 14 Criminal First Instance No. 48	https://wenshu.court.gov.cn/website/wenshu/181107ANFZ0BXSK4/index.html?docId=Np7NlKPz4l0WtAT3BbVCJ0cy7PLJRnNiwpoZ/nMFDWGR4xFjZbR3/J/dgBYosE2gNO3IcCbk7GGr4PEcpIwkZ7+a3weUNSbCt4Hw4I001PGMKk6+wIozpdse/7abaS/z
17	(2021) Lu 02 Civil First Instance No. 69[50]	https://rmfyalk.court.gov.cn/view/content.html?id=EaXtuxsImd4y8PrUVDyi0kqyM%252FmH3rsW0rTKv9W73lc%253D&lib=ck&qw=穿山甲
18	(2020) Zhe 07 Civil First Instance No. 299	https://wenshu.court.gov.cn/website/wenshu/181107ANFZ0BXSK4/index.html?docId=V3D3+2vF8WJ6QhP1q0q+5dBkDwQws8cwI6i0kIPx6g9vcvMcXeq5B5/dgBYosE2gNO3IcCbk7GGr4PEcpIwkZ7+a3weUNSbCt4Hw4I001PGMKk6+wIozpbpgmvAwPlRP
19	(2019) Yun 05 Criminal First Instance No. 219	https://wenshu.court.gov.cn/website/wenshu/181107ANFZ0BXSK4/index.html?docId=UA9Qs1v6yJXe7UGbvrehFMpY8jilrnPZO1hmXpEVG/l5d7yXmdExhp/dgBYosE2gNO3IcCbk7GGr4PEcpIwkZ7+a3weUNSbCt4Hw4I001PGMKk6+wIozpTm7JjhSAhEe
20	(2019) Yun 05 Criminal First Instance No. 234	Pkulaw.com/CLI.C.103532608
21	(2020) Su 0102 Criminal First Instance No. 370	https://wenshu.court.gov.cn/website/wenshu/181107ANFZ0BXSK4/index.html?docId=arQ0BuCRnZ/xRBkO4GVjfljZ/9r2GcRGjY1qy+Es341aZHWLzgAYrJ/dgBYosE2gNO3IcCbk7GGr4PEcpIwkZ7+a3weUNSbCt4Hw4I001PFZohvLXiNZqXnzQcTkCNeP

22	(2020) Su 0508 Criminal First Instance No. 1159	https://wenshu.court.gov.cn/website/wenshu/181107ANFZ0BXSK4/index.html?docId=d+dwNdGDyb8FKtHTPFq8CSqax2pELJ1bRht3t9gyJZcoUlAguGYNk5/dgBYosE2gNO3IcCbk7GGr4PEcpIwkZ7+a3weUNSbCt4Hw4I001PFZohvLXiNZqSR8w/sOMwGE
23	(2020) Min 0881 Criminal First Instance No. 278	https://wenshu.court.gov.cn/website/wenshu/181107ANFZ0BXSK4/index.html?docId=8+rxcRm4OtOcUWV5CwP48iV/J28IEg3FEXAtmmJ6cXVS3gBLXIPFUp/dgBYosE2gNO3IcCbk7GGr4PEcpIwkZ7+a3weUNSbCt4Hw4I001PFZohvLXiNZqRMNnYYeVdoT
24	(2020) Min 0212 Criminal First Instance No. 246	https://wenshu.court.gov.cn/website/wenshu/181107ANFZ0BXSK4/index.html?docId=g/c5+2sulEz9m8+9TPzKO0al0mRuAPRLfDdd3G1NGZTS8rtrNPhNXZ/dgBYosE2gNO3IcCbk7GGr4PEcpIwkZ7+a3weUNSbCt4Hw4I001PFZohvLXiNZqQCgjf0nkTCI
25	(2020) Yun 3103 Criminal First Instance No. 359	https://wenshu.court.gov.cn/website/wenshu/181107ANFZ0BXSK4/index.html?docId=V4fCxJP6u98i9HifuIELql9aQcwSO1IBw2tzDcLg1axMUaRgBQjALp/dgBYosE2gNO3IcCbk7GGr4PEcpIwkZ7+a3weUNSbCt4Hw4I001PFZohvLXiNZqZHBW23zcL8c
26	(2020) Yun 0422 Criminal First Instance No. 113	https://wenshu.court.gov.cn/website/wenshu/181107ANFZ0BXSK4/index.html?docId=7mxCG1kfqFIAT9Dzo1YLjeGJc7X7FykBWyiIdXwYD9mfab7vhQnSjp/dgBYosE2gNO3IcCbk7GGr4PEcpIwkZ7+a3weUNSbCt4Hw4I001PGgea+7f+AZHT/dXNK9qcNl
27	(2020) Yue 0204 Criminal First Instance No. 213	https://wenshu.court.gov.cn/website/wenshu/181107ANFZ0BXSK4/index.html?docId=nEjR7hqAO8R9xiaoaBUGPRlPipgLGU8DOq1usnJdkY4HbqFj9vLYk5/dgBYosE2gNO3IcCbk7GGr4PEcpIwkZ7+a3weUNSbCt4Hw4I001PGgea+7f+AZHXYt1vZqVITy
28	(2020) Min 0881 Criminal First Instance No. 197	https://wenshu.court.gov.cn/website/wenshu/181107ANFZ0BXSK4/index.html?docId=5VrccDkf9DWUM5ULDkqA1hFSeTLrDni9zoGNKF3Qumdw4rShfYaM7p/dgBYosE2gNO3IcCbk7GGr4PEcpIwkZ7+a3weUNSbCt4Hw4I001PGgea+7f+AZHUzwv99elqs1
29	(2020) Min 0881 Criminal First Instance No. 180	https://wenshu.court.gov.cn/website/wenshu/181107ANFZ0BXSK4/index.html?docId=6CvtfkzasvK2KcgSAEf6fZAOTFDTwms3ffNFNH4yK1YMOKTM9EVszJ/dgBYosE2gNO3IcCbk7GGr4PEcpIwkZ7+a3weUNSbCt4Hw4I001PGgea+7f+AZHe5k0vVpPTwG
30	(2020) Yue 1323 Criminal First Instance No. 473	https://wenshu.court.gov.cn/website/wenshu/181107ANFZ0BXSK4/index.html?docId=smlPFwvnNFqOsIn2fjiznleJqd5p+ZQarpZcTVItfeEBmPmMeef0Zp/dgBYosE2gNO3IcCbk7GGr4PEcpIwkZ7+a3weUNSbCt4Hw4I001PGgea+7f+AZHX7s824Qhvtv
31	(2020) Min 0881 Criminal First Instance No. 159	https://wenshu.court.gov.cn/website/wenshu/181107ANFZ0BXSK4/index.html?docId=pl5PTLOzdglKSNRlRSvkfPpERJR5pwdsTW8Z2OzOtN9o9No2+bSBmp/dgBYosE2gNO3IcCbk7GGr4PEcpIwkZ7+a3weUNSbCt4Hw4I001PGgea+7f+AZHeJ8XEEVUp79
32	(2020) Yue 0303 Criminal First Instance No. 265	https://wenshu.court.gov.cn/website/wenshu/181107ANFZ0BXSK4/index.html?docId=kPOB9XJWQhdQ/1Lm1WHJlkVCrKjTxoivOrtu+ld3aabYFumMX1q7ap/dgBYosE2gNO3IcCbk7GGr4PEcpIwkZ7+a3weUNSbCt4Hw4I001PFYPd9/+cLmYWqDBfkSGtLH

33	(2020) Gan 1002 Criminal First Instance No. 201-2	https://wenshu.court.gov.cn/website/wenshu/181107ANFZ0BXSK4/index.html?docId=+kS01PPtoq65rCgmLbY8MdS9/86rTTKXfVB2F3bUlDebP8p+0QZNFZ/dgBYosE2gNO3IcCbk7GGr4PEcpIwkZ7+a3weUNSbCt4Hw4I001PFYPd9/+cLmYRsSdyqX3Ct+
34	(2019) Gan 0732 Criminal First Instance No. 265	https://wenshu.court.gov.cn/website/wenshu/181107ANFZ0BXSK4/index.html?docId=YeDEDDxw7PQbMRmuJFPcXPpaWNg7/7rRPfof+MWmIGy6l2FGt4IluZ/dgBYosE2gNO3IcCbk7GGr4PEcpIwkZ7+a3weUNSbCt4Hw4I001PFYPd9/+cLmYbtXrFvMHsyF
35	(2020) Chuan 0106 Criminal First Instance No. 335	https://wenshu.court.gov.cn/website/wenshu/181107ANFZ0BXSK4/index.html?docId=cVyxAV2KtXR+qeiAZ9KxALNa/cvwx/DVwJ1DNuki7nrmi6asL5c1hZ/dgBYosE2gNO3IcCbk7GGr4PEcpIwkZ7+a3weUNSbCt4Hw4I001PFYPd9/+cLmYeY3bN1sXsx5
36	(2020) Yun 0927 Criminal First Instance No. 52	https://wenshu.court.gov.cn/website/wenshu/181107ANFZ0BXSK4/index.html?docId=SLOmL5FWlp0l9iMppnYpzBj0gMxT5m83iqarsoTAaH1kw0JJ1OsN2Z/dgBYosE2gNO3IcCbk7GGr4PEcpIwkZ7+a3weUNSbCt4Hw4I001PFYPd9/+cLmYecp5cKuGcGo
37	(2020) Yun 2901 Criminal First Instance No. 116	https://wenshu.court.gov.cn/website/wenshu/181107ANFZ0BXSK4/index.html?docId=JseWE2JCpaf/wqHQclmJVtXjfg7q9rSBshGYolf0vb++2/jprp2qaJ/dgBYosE2gNO3IcCbk7GGr4PEcpIwkZ7+a3weUNSbCt4Hw4I001PFYPd9/+cLmYeopzPCloIa2
38	(2020) Gan 0222 Criminal First Instance No. 24	https://wenshu.court.gov.cn/website/wenshu/181107ANFZ0BXSK4/index.html?docId=BhA0RJ6OxBJ/WDqJYZ6frrcA53iZZhoBGFRTkosz0hS1jKiTYxzfXJ/dgBYosE2gNO3IcCbk7GGr4PEcpIwkZ7+a3weUNSbCt4Hw4I001PHs5J+DZ+07Psc3aXN/Sy7B
39	(2019) Yue 0303 Criminal First Instance No. 855	https://wenshu.court.gov.cn/website/wenshu/181107ANFZ0BXSK4/index.html?docId=OUD3Tm7EvETT73FeQnBWAWBB5o9NpeDhFcCYSh9QzZUVT1ktMp9nuZ/dgBYosE2gNO3IcCbk7GGr4PEcpIwkZ7+a3weUNSbCt4Hw4I001PHs5J+DZ+07Pi+rcx99UAIT
40	(2019) Yun 2627 Criminal First Instance No. 357	https://wenshu.court.gov.cn/website/wenshu/181107ANFZ0BXSK4/index.html?docId=3b9F7TAbsXvWg1UskjWv5mheawbs23rBN5sUboMPrLPbDPbq6GQJkp/dgBYosE2gNO3IcCbk7GGr4PEcpIwkZ7+a3weUNSbCt4Hw4I001PHs5J+DZ+07PmSjtM9JAweb
41	(2019) Yun 2627 Criminal First Instance No. 358	https://wenshu.court.gov.cn/website/wenshu/181107ANFZ0BXSK4/index.html?docId=o338YswRw5yyPLQSgpH2ZeB/KRr/h/y/v8IlbdSiACP1tX8lhMKeGZ/dgBYosE2gNO3IcCbk7GGr4PEcpIwkZ7+a3weUNSbCt4Hw4I001PHs5J+DZ+07PkP8K2UB86FE
42	(2019) Yun 2627 Criminal First Instance No. 361	https://wenshu.court.gov.cn/website/wenshu/181107ANFZ0BXSK4/index.html?docId=vjFW3xzcinBD8v9iMdKvmiNRLy7rwkmyBFdyyJKDS0NA2Q0Y1KdI3J/dgBYosE2gNO3IcCbk7GGr4PEcpIwkZ7+a3weUNSbCt4Hw4I001PHs5J+DZ+07PsCUnPe8ZmHm
43	(2019) Yun 2627 Criminal First Instance No. 362	https://wenshu.court.gov.cn/website/wenshu/181107ANFZ0BXSK4/index.html?docId=p58Y+/H8HaV81uD9C7iFwij//g6t9caeAS6VZptKqQqq2mmTsgabXJ/dgBYosE2gNO3IcCbk7GGr4PEcpIwkZ7+a3weUNSbCt4Hw4I001PHs5J+DZ+07Pm23gUl9EJsT

44	(2019) Qian 2722 Criminal First Instance No. 232	https://wenshu.court.gov.cn/website/wenshu/181107ANFZ0BXSK4/index.html?docId=KdfagSZnUF/1NPSDngNiYFKKpclwAN2S5L0+Jctm6Oj0ZFeUzk9kVp/dgBYosE2gNO3IcCbk7GGr4PEcpIwkZ7+a3weUNSbCt4Hw4I001PHs5J+DZ+07PhkHiuCl0WMe
45	(2019) Zhe 0781 Criminal First Instance No. 445	https://wenshu.court.gov.cn/website/wenshu/181107ANFZ0BXSK4/index.html?docId=GvdWoLTHX0VORV9UwiusN5BWvoqARwrTbRRBRiKYBbthHFnFviG5P5/dgBYosE2gNO3IcCbk7GGr4PEcpIwkZ7+a3weUNSbCt4Hw4I001PHs5J+DZ+07PvSv5WN6fdZE
46	(2019) Yun 3102 Criminal First Instance No. 389	Pkulaw.com/CLI.C.96450271
47	(2019) Gui 0902 Xing Chu No. 669	https://wenshu.court.gov.cn/website/wenshu/181107ANFZ0BXSK4/index.html?docId=uRWyTlZjqeHnoP1bFA1BRXv1B+LeODn8MrFY6zDP+Rjh6DatUK50/p/dgBYosE2gNO3IcCbk7GGr4PEcpIwkZ7+a3weUNSbCt4Hw4I001PEnPCcSZO4w2TJ1innu4SDK
48	(2020) Yun 0428 Criminal First Instance No. 126	https://wenshu.court.gov.cn/website/wenshu/181107ANFZ0BXSK4/index.html?docId=7vKE4vsMMe+LxmRinQ1uwEHjno+3E4KCtrv8pCQ7mVQNA8OKcXGU3Z/dgBYosE2gNO3IcCbk7GGr4PEcpIwkZ7+a3weUNSbCt4Hw4I001PEnPCcSZO4w2VchEB0yHUPh
49	(2020) Yue 1422 Criminal First Instance No. 85	https://wenshu.court.gov.cn/website/wenshu/181107ANFZ0BXSK4/index.html?docId=sTXvGXjL3UYNrZyFAGByuFmZfsLA7GkYTV949k9C19WCh4oRUlB7b5/dgBYosE2gNO3IcCbk7GGr4PEcpIwkZ7+a3weUNSbCt4Hw4I001PEnPCcSZO4w2SDVZaP0Ti2L
50	(2018) Yun 0428 Criminal First Instance No. 148	Pkulaw.com/CLI.C.75230468

Special note: Cases Nos. 6, 20, 46, and 50 were collected by the author during the early stage of writing and were originally retrieved from “China Judgements Online.” However, because these cases were removed from the website by the People’s Court, they were no longer accessible when the dataset was later supplemented. To ensure completeness, the author used the equally authoritative “PKULAW.COM” to provide replacement links for these cases.
