# Supplementary material for: China’s Legal Protection System for Pangolins: Past, Present, and Future
Source: Animals (Basel). 2025 Aug 18;15(16):2422. doi: 10.3390/ani15162422 (PMC12383201; doi:10.3390/ani15162422)
Supplement: Supplementary file 1 [file animals-15-02422-s001.zip › Supplementary Material S2 -Full Texts of Laws and Regulations Related to Pangolins in China/【10】国务院办公厅转发林业部关于加强野生动物保护管理工作报告的通知(FBM-CLI.2.pdf]

国务院办公厅转发林业部关于加强野生动物保护管理工作报告的通知

制定机关： 国务院办公厅 机构沿革

发文字号：国办发〔1991〕68号

公布日期：1991.10.23

施行日期：1991.10.23

时效性： 现行有效

效力位阶： 国务院规范性文件

法规类别： 野生动植物资源 森林和野生动植物保护区

国务院办公厅转发林业部关于加强野生动物保护管理工作报告的通知

（国办发〔1991〕68号）

各省、自治区、直辖市人民政府，国务院各部委、各直属机构：

林业部《关于加强野生动物保护管理工作的报告》已经国务院同意，现转发给你们，请遵照执行。

一九九一年十月二十三日

关于加强野生动物保护管理工作的报告

国务院：

一九九一年一月，国务院发出《关于加强野生动物保护严厉打击违法犯罪活动的紧急通知》，召开了全国保护野生动物电话会议。为了检查国务院紧急通知和电话会议精神的落实情况，我们会同高检院、高法院及农业部、公安部、经贸部、

工商局、商检局、海关总署等有关部门，组织了六个联合检查组，分别到四川、云南、新疆、广东、广西、福建等省（区）的四十多个地（州）、县（市），检查了五十三个集贸市场，七十四家饭店、餐馆，十一个进出口口岸及一些经营、加工单位。从检查的情况看，各省（区）政府和有关部门都很重视，及时传达、印发了文件，并作了具体部署，乱捕滥猎等违法活动有所收敛，野生动物保护工作得到加强。当前存在的主要问题：

一是违法猎杀倒卖野生动物案件仍不断发生。据对六个省（区）的检查，自一九八九年三月一日实施《中华人民共和国野生动物保护法》（以下简称《野生动物保护法》）以来，发生违法猎捕、买卖野生动物的案件七百一十五起，被猎杀、倒卖的有大熊猫、金丝猴、长臂猿等国家一级保护动物十多种三千多只，马鹿、黑熊、穿山甲、娃娃鱼等国家二级保护动物二十多种四万多只，还有大批地方政府规定保护的动物。问题的严重性在于，猎杀倒卖珍稀野生动物的违法事件仍未得到制止。

二是野生动物及其产品流通领域秩序混乱。违法收购、运输、经营野生动物的案件依然不断发生，一些人把高消费扩展到滥吃珍禽异兽。检查组在广东检查发现，有三个集贸市场、二十五家餐馆经营国家规定保护的野生动物，现场查获国家一、二级保护动物二百七十八只。检查组在广西发现，违法经营的国家一级保护动物有巨蜥七百一十公斤、蜂猴四只、国家二级保护动物穿山甲三百三十公斤以及其他珍贵野生动物。一些部门和企业的领导点名用珍稀动物招待宾客，一些宾馆、餐馆为牟利而甘冒风险，尽量满足食客的要求，助长了滥吃野生动物的歪风。一些单位无视国家法令，任意收购、加工和经营国家规定保护的野生动物。有的地方外贸、药材部门不顾资源状况，盲目下达野生动物毛皮、药材收购计划，造成经营利用与保护管理严重脱节。

三是宣传不够广泛，对违法活动打击不力。近几年来，各地在《野生动物保护法》的宣传方面虽然做了大量工作，但广大林区、山区的群众对国家保护野生动物的政策规定了解很少。城镇中一些从事这种经营活动的人法制观念淡薄，只顾做生意赚钱。加上与《野生动物保护法》的配套法规没有跟上，执法部门对违法行为的处罚普遍偏轻，违法犯罪分子得不到应有的打击。

四是野生动物保护机构不健全、不落实，管理力量严重不足，经费短缺。许多地方由于缺少机构和必要的管理人员，工作十分被动。在这种情况下，贯彻执行《野生动物保护法》的许多措施难以落实，地方野生动物管理部门普遍感到工作难度很大。野生动物大都分布在偏远的经济不发达地区，地方财政困难，大多无力安排保护经费。不仅象野生动物资源调查、科学研究、拯救濒危物种、收容饲养动物、补偿群众损失这些耗资较多的工作不能进行，就连宣传教育、查处案件和正常业务工作都很难开展。

五是猎枪生产、销售失控的问题一直没有得到解决。一些非定点生产、经销单位仍在大量生产、销售猎枪。据调查，每年在林业部下达的猎枪生产限额外生产、销售的猎枪约十万支，加上一部分小口径运动步枪及汽枪的使用管理不严，已对野生动物保护构成极大威胁。

为进一步加强野生动物保护管理工作，建议采取以下综合治理措施：

一、要提高认识，加强领导。野生动物既是自然生态环境的重要组成部分，又是国家宝贵的自然资源，对维护生态平衡、发展经济、开展科学研究都具有重要意义。各级政府要把保护野生动物列入重要议事日程，切实加强领导，解决当前工作中存在的实际问题。林业、农业、工商、经贸、商检、海关及公安、司法等部

门对保护野生动物资源、做好保护管理工作都负有重要责任，要密切配合，互相支持，共同努力，切实把这项工作抓紧、抓实、抓好。

二、要认真贯彻执行《野生动物保护法》，依法管理野生动物资源。各级政府要把宣传贯彻《野生动物保护法》列入公民进行法制教育和精神文明建设的重要内容。要严厉查处乱捕滥猎和倒卖走私国家重点保护野生动物的案件，严格猎枪、弹具的管理，坚决打击各种违法犯罪活动。问题严重的地方，要列入“严打”斗争的内容，进行专项治理。林业、农业等有关部门要抓紧制定必要的配套法规，使野生动物保护工作逐步规范化、制度化。

三、进一步加强对猎捕活动的管理。各级林业行政主管部门要严格控制对国家重点保护野生动物的猎捕，严格实行《狩猎证》、《特许猎捕证》、《驯养繁殖许可证》制度。要加强对专业猎人、季节性猎人和业余狩猎人员的教育。有条件的地方，可在林业行政主管部门指导下，成立猎人协会，做到有组织、有计划地开展狩猎活动。严禁使用禁用的狩猎工具和狩猎方法。在资源破坏严重或不适宜开展狩猎的地方和季节，当地政府要明确划定禁猎区和禁猎期。禁止跨省（区）、跨县（旗）狩猎，如确有必要，则需事先征得对方主管部门的同意，严格遵守当地的各项规定，接受当地的监督和检查。

四、进一步加强对野生动物经营活动的管理。国家重点保护的野生动物及其产品一律不得进入集贸市场。任何餐馆、饭店、宾馆、招待所都不得出售以国家重点保护野生动物或其产品为原料的食品。在特殊情况下，需养殖、出售、收购国家重点保护野生动物或其产品的，要按《野生动物保护法》和国家其他有关规定，

严格履行审批手续。对养殖经营非国家重点保护野生动物或其产品，省、自治区、直辖市有关部门也要采取措施，加强管理。铁路、交通、民航、邮电等部门，对运输、托运、携带国家重点保护野生动物或其产品，要按有关规定严格加以管理。

五、切实搞好野生动物保护管理的基础工作。各地特别是野生动物的重点分布区和经营活动频繁地区的林业行政主管部门，要在当地政府的统一安排下建立健全野生动物保护管理机构，切实做好日常保护管理工作。同时，执法机关要加强力量，使各项监督检查工作真正落到实处。野生动物保护管理工作所需经费，除各级财政部门给予适当安排外，各级主管部门，要努力筹集资金，增加对野生动物保护管理工作的投入。

为了掌握全国野生动物资源状况，建议以省、自治区、直辖市为单位，用三至五年时间进行一次国家重点保护野生动物资源调查。所需经费除请地方财政部门给予安排外，其他有关部门也要给予支持。

以上报告如无不妥，请批转各地执行。

一九九一年七月十三日

本篇引用的法规

中央法规

国务院关于加强野生动物保护严厉打击违法犯罪活动的紧急通知

中华人民共和国野生动物保护法

引用本篇的法规 案例 论文

地方法规规章

广西壮族自治区人民政府关于宣布失效一批自治区人民政府文件的决定(桂政发〔2017〕66号)
湖北省人民政府关于规范性文件清理结果的决定
昆明市人民政府关于进一步严格保护海鸥的通告

法学期刊

野生动物致害补偿地方立法的现实困境与纾解路径

*注：本文格式遵循《全国人大法规备案审查信息平台电子文件格式规范（试行）》标准。

©北大法宝：（www.pkulaw.com）专业提供法律信息、法学知识和法律软件领域各类解决方案。北大法宝为您提供丰富的参考资料，正式引用法规条文时请与标准文本核对。

欢迎查看所有产品和服务。

法宝快讯：如何快速找到您需要的检索结果？法宝V6有何新特色？

扫描二维码阅读原文

原文链接：https://www.pkulaw.com/chl/319b8a2cb6160961bdfb.html
