# Supplementary material for: China’s Legal Protection System for Pangolins: Past, Present, and Future
Source: Animals (Basel). 2025 Aug 18;15(16):2422. doi: 10.3390/ani15162422 (PMC12383201; doi:10.3390/ani15162422)
Supplement: Supplementary file 1 [file animals-15-02422-s001.zip › Supplementary Material S2 -Full Texts of Laws and Regulations Related to Pangolins in China/【11】农业部关于公布《中华人民共和国进境动物一、二类传染病、寄生虫病名录》和《中华人民共和国禁止携带、邮寄进境的动物、动物...(FBM-CLI.4.pdf]

农业部关于公布《中华人民共和国进境动物一、二类传染病、寄生虫病名录》和《中华人民共和国禁止携带、邮寄进境的动物、动物产品和其它检疫物名录》通知

制定机关： 农业部(已撤销) 机构沿革

发文字号：[1992]农[检疫]字第12号

公布日期：1992.06.08

施行日期：1992.06.08

时效性： 废止或失效

效力位阶： 部门规范性文件

法规类别： 进出境动植物检疫

废止或失效依据：国家质量监督检验检疫总局公告2012年第138号——关于公布现行有效规范性文件和废止部分规范性文件的公告

部分废止或失效依据：本篇法规中的《中华人民共和国禁止携带、邮寄进境的动物、动物产品和其它检疫物名录》已被农业部、国家质量监督检验检疫总局公告第1712号——中华人民共和国禁止携带、邮寄进境的动植物及其产品名录(2012修订)废止

农业部关于公布《中华人民共和国进境动物一、二类

传染病、寄生虫病名录》和《中华人民共和国禁止携带、

邮寄进境的动物、动物产品和其他检疫物名录》的通知

（（１９９２）农（检疫）字第１２号）

各省、自治区、直辖市农（牧、渔）业厅（局），各口岸动植物检疫局、动物检疫所：

为进一步加强进出境动物检疫工作，防止动物传染病、寄生虫病传入，保护我国农林牧渔业生产和人体健康，根据《中华人民共和国进出境动植物检疫法》第五条、第十八条和第二十九条的规定，我们制定了《中华人民共和国进境动物一、二类传染病、寄生虫病名录》和《中华人民共和国禁止携带、邮寄进境的动物、动物产品和其他检疫物名录》。现将这两个“名录”发给你们，请遵照执行。

附件：

1、《中华人民共和国进境动物一、二类传染病、寄生虫病名录》

2、《中华人民共和国禁止携带、邮寄进境的动物、动物产品和其他检疫物名录》

一九九二年六月八日

中华人民共和国进境动物

一、二类传染病、寄生虫病名录

List A and List B Diseases for the Animals Imported form other Countries into the People Republic of China

Ⅰ、一类传染病、寄生虫病

I. List A Diseases

口蹄疫 Foot-and-mouth-Disease

非洲猪瘟 African Swine Fever

猪水包病 Swine Vesicular Disease

猪瘟 Swine Fever

牛瘟 Rinderpest

小反刍兽疫 Peste des Petits Ruminants

兰舌病 Bluetongue

痒病 Scrapie

牛海绵状脑病 Bovine Spngifom Encephalopathy

非洲马瘟 African Horse Sickness

鸡瘟 Fowl Plague

新城疫 Newcastle Disease

鸭瘟 Duck Plague

牛肺疫 Contagious Bovine Pleuropneumonia

牛结节疹 Lumpy Skin Disease

Ⅱ、二类传染病、寄生虫病

Ⅱ. List B Diseses

共患病 (Multiple Specise Diseases);

炭疽 Anthrax

伪狂犬病 Aujeszky's Disease

心水病 Heartwater

狂犬病 Rabies

Q热 Q Fever

裂谷热 Rift Valley Feyer

副结核病 Paratuberculosis (John's Disease)

巴氏杆菌病 Pasteurellosis

布氏杆菌病 Brucellosis

结核病 Tuberculosis

鹿流行性出血热 Epizootic Haemorrhagic Disease of Deer

细小病毒病 Parvovirus Infection

梨型虫病 Piroplasmosis

牛病 (Cattle Diseases).

锥虫病 Trpanosomiasis

边虫病 Anaplasmosis

牛地方流行性白血病 Enzootic Bovine Leukosis

牛传染性鼻气管炎 Infectious Bovine Rhinotracheitis

牛病毒性腹泻-粘膜病 Bovins Viral Diarrhae-Mucosal Disease

牛生殖道弯曲杆菌病 Bovine Genital Compylobacteriosis

赤羽病 Akabane Disease

中山病 Chuzan Disease

水泡性口谈 Vesicular Stomatitis

牛流行热 Bovine Ephemeral Fever

茨城病 Ibaraki Disease

绵羊和山羊病 (Sheep and Goat Diseases):

绵羊痘和山羊痘 Sheep Pox and Goat Pox

衣原体病 Enzootic Aboution of Ewes

梅迪-维斯纳病 Maedi-visna Disesse

边界病 Border Disease

绵羊肺腺瘤病 Sheep Pulmonary Adenomatosis

山羊关节炎/脑炎 Caprine Arthritis/Encephalitis

猪病 (Pig Diseases):

猪传染性脑脊髓炎 Teschen Disease

猪传染性胃肠炎 Transmissible Gastroenteritis of Swine

猪流行性腹泻 Porcine Epizootic Diarrhea

猪密螺旋体痢疾（猪血痢） Swine Dysentery

猪传染性胸膜肺炎 Infectious Pleuropneumonia of Swine

猪生殖和呼吸系统综合症（兰耳病） Swine Reproductive and Respiratory Syndrome (Blue-eared Disease)

马病 (Horse Diseases):

马传染性贫血 Equine Infectious Anaemia

马脑脊髓炎 Equine Encephalomyelitis

委内瑞拉马脑脊髓炎 Venezuelan Equine Encephalormyelitis

马鼻疽 Glanders

马流行性淋巴管炎 Epizootic Lymphangitis

马沙门氏杆菌病（马流产沙门氏杆菌） Salmonellosis (S.Abortus Equi)

类鼻疽 Melioidosis

马传染性动脉炎 Infectious Arteritis of Horses

马鼻肺炎 Equine Rhinopneumonitis

禽病 (Poultry Diseases):

鸡传染性喉气管炎 Avian Infectious Laryngotracheitis

鸡传染性支气管炎 Avian Infectious Broncheitis

鸡传染性囊病（甘保罗病） Infectious Bursal Disease

鸭病毒性肝炎 Duck Viral Hepatitis

鸡伤寒 Fowl Typhoid

禽痘 Fowl Pox

鹅螺旋体病 Spirochaetosis in Goose

马立克氏病 Marek's Disease

住白细胞原虫病 Leucocytozoosis

鸡白痢 Pullorum Disease

家禽支原体病 Avian Mycoplasmosis

鹦鹉病（鸟疫） Psittacosis and Ornithosis

鸡病毒性关节炎 Avian Viral Arthritis

禽白血病 Avian Leukosis（祖代以上需作血清学试验）

啮齿动物病 (Rodent Diseases):

兔病毒性出血症（兔瘟） Viral Haemorrhaig Disease of Rabbits

兔粘液瘤病 Myxomatosis

野兔热 Tularaemia

水生动物病 (Aquatic Animal Diseases)

鲑鱼传染性胰脏坏死 Infectious Pancreatic Necrosis in Trout

鱼传染性造血器官坏死 Infectious Haematopoietic Necrosis of Fish

鲤春病毒病 Spring Viremia of Carp

鲑鳟鱼病毒性出血性败血症 Haemorrhagic Septicaemia of Salmonids

鱼鳔炎症 Swim Bladder Inflammation of Fish

鱼眩转病 Whirling Disease of Fish

鱼鳃霉病 Branchiomycosic of Fish

鱼疖疮病 Furunculosis of Fish

异尖线虫病 Disease of Anisakis

对虾杆状病毒病 Disease of Baculovirus Penaei

斑节对是杆状病毒病 Disease of Penaeus Monodon Type Baculovirus

蜂病 (Bee Diseases):

美洲蜂幼虫腐臭病 American Foul Brood

欧洲蜂幼虫腐臭病 European Foul Brood

蜂螨病 Acariasis of Bees

瓦螨病 Varroais

蜂孢子虫病 Nosemosis of Bees

其他动物疾病 (Diseases of Other Animal Species)

蚕微粒子病 Pebrine Disease of Chinese Silkworm

水貂阿留申病 Aleutian Diseae of Mink

犬瘟热 Canine Distemper

利什曼病 Leishmaniasis

中华人民共和国禁止携带、邮寄

进境的动物、动物产品和其他检疫物名录

类别	名　　　称
动物	鸡、鸭、鹅、锦鸡、猫头鹰、鸽、鹌鹑、鸟、兔、大白鼠、小鼠、豚鼠、松鼠、花鼠、蛙、蛇、龟、鳖、蜥蜴、鳄、蚯蚓、蜗牛、鱼虾、蟹、猴、穿山甲、猞猁、蜂蜜、蚕等。

动物产品	精液、胚胎、受精卵、蚕卵、生肉类、腊肉、香肠、火腿、腌肉、熏肉、蛋、水生动物产品、鲜奶、奶酪、黄油、奶油、乳清粉、皮张、鬃毛类、蹄骨角类、血液、血粉、油脂类、脏器等。
其他检疫物	菌种、毒种、虫种、细胞、血清、动物标本、动物尸体、动物废弃物以及可能被病原体污染的物品。

注：通过携带或邮寄方式进境的动物、动植物产品和其他检疫物，经过国家动植物检疫关特许批准的，并具有输出国或地区官方出具的检疫证书，不受此名录的限制。

*注：本文格式遵循《全国人大法规备案审查信息平台电子文件格式规范（试行）》标准。

©北大法宝：（www.pkulaw.com）专业提供法律信息、法学知识和法律软件领域各类解决方案。北大法宝为您提供丰富的参考资料，正式引用法规条文时请与标准文本核对。

欢迎查看所有产品和服务。

法宝快讯：如何快速找到您需要的检索结果？法宝V6有何新特色？

扫描二维码阅读原文

原文链接：https://www.pkulaw.com/chl/b9f116d2405ba40dbdfb.html
