# Supplementary material for: China’s Legal Protection System for Pangolins: Past, Present, and Future
Source: Animals (Basel). 2025 Aug 18;15(16):2422. doi: 10.3390/ani15162422 (PMC12383201; doi:10.3390/ani15162422)
Supplement: Supplementary file 1 [file animals-15-02422-s001.zip › Supplementary Material S2 -Full Texts of Laws and Regulations Related to Pangolins in China/【12】林业部、财政部、国家物价局关于发布《陆生野生动物资源保护管理费收费办法》的通知(FBM-CLI.4.pdf]

林业部、财政部、国家物价局关于发布《陆生野生动物资源保护管理费收费办法》的通知

制定机关： 林业部(已变更) 财政部 国家物价局(已变更) 机构沿革

发文字号：林护字〔1992〕72号

批准机关： 国务院

公布日期：1992.12.17

批准日期：1992.12.17

施行日期：1993.01.01

时效性： 现行有效

效力位阶： 部门规章

法规类别： 森林和野生动植物保护区 野生动植物资源 行政事业与服务收费管理 行政事业性收费 行政事业财务管理

林业部　财政部　国家物价局　关于发布

《陆生野生动物资源保护管理费收费办法》的通知

（1992年12月17日　林护字〔1992〕72号）

《陆生野生动物资源保护管理费收费办法》和《捕捉、猎捕国家重点保护野生动物资源管理费收费标准》已于十一月二十二日经国务院批准，现予发布施行。

附件：1、陆生野生动物资源保护管理费收费办法

2、捕捉、猎捕国家重点保护野生动物资源保护管理费收费标准

附件一：

陆生野生动物资源保护管理费收费办法

野生动物资源属于国家所有。根据《野生动物保护法》第二十七条关于“经营利用野生动物或者其产品的，应当缴纳野生动物资源保护管理费”的规定，现将陆生野生动物（以下简称野生动物）资源保护管理费收费办法规定如下：

一、凡经营利用野生动物或者其产品的，必须按本办法规定缴纳野生动物资源保护管理费。

二、因科学研究、驯养繁殖、展览或者其他特殊情况，需要捕捉、猎捕、出售、收购、利用国家重点保护野生动物或其产品的，必须按《野生动物保护法》第十六条、第二十二条和《陆生野生动物保护实施条例》第十一条、第十二条的规定，严格履行申报审批手续。

三、经批准捕捉、出售、收购、利用国家一级保护野生动物或其产品的，必须向林业部或其授权的单位缴纳野生动物资源保护管理费；经批准猎捕、出售、收购、利用国家二级保护野生动物或其产品的，必须向省、自治区、直辖市林业行政主管部门或其授权的单位缴纳野生动物资源保护管理费。其收费环节、标准和办法如下：

（一）对批准捕捉、猎捕的国家重点保护野生动物，按《捕捉、猎捕国家重点保护野生动物资源保护管理费收费标准》向申请捕捉、猎捕者收费。

（二）对批准出售、收购、利用的国家一级保护野生动物或其产品，按其成交额

的8%向供货方收费，对受货方不予收费；对批准出售、收购、利用的国家二级保护野生动物或其产品，按其成交额的6%向供货方收费，对受货方不予收费。

（三）依据《陆生野生动物保护实施条例》第三十一条关于“利用野生动物或者其产品举办出国展览等活动的经济收益，主要用于野生动物保护事业”的规定，对批准利用国家重点保护野生动物或其产品在国外举办的表演、展览等活动，按其纯收入的50%向国内承办单位收费。

（四）外国人依法在中国对国家重点保护野生动物进行野外考察研究、拍摄电影、录像或者从事狩猎，由林业部参照国际惯例制定具体收费办法。

（五）对以保护野生动物为目的的科学研究、资源调查或其他特殊情况，需要捕捉、猎捕国家重点保护野生动物的，按分工管理权限，分别经林业部、省级林业行政主管部门批准，可以酌情减免野生动物资源保护管理费。

法宝联想

法律法规　规范性文件（2）

四、经营利用非国家重点保护野生动物或其产品的收费环节、标准和办法，由省级林业行政主管部门提出，经同级物价、财政部门审定后执行。

五、捕捉、猎捕国家重点保护野生动物的，必须申请特许猎捕证，每核发一份特许猎捕证，收取工本费5元；驯养繁殖国家重点保护野生动物的，必须申请驯养繁

殖许可证，每核发一份驯养繁殖许可证，收取工本费10元；猎捕非国家重点保护野生动物的，必须申请狩猎证，每核发一份狩猎证，收取工本费5元。

六、非法经营利用野生动物或者其产品的，除依照野生动物保护法律、法规给予处罚外，还必须按本办法规定收费标准的二至五倍补收野生动物资源保护管理费。

七、《捕捉、猎捕国家重点保护野生动物资源保护管理费收费标准》的调整，由林业部商国家物价局、财政部确定；《陆生野生动物保护实施条例》第三十三条、第三十七条中规定的价值标准，由林业部确定。

八、收费单位应向指定的物价部门办领收费许可证，使用财政部门统一印制的收费票据。

九、野生动物资源保护管理费按预算外资金管理，纳入财政部专户储存。野生动物资源保护管理费要专款专用，按照财政部门规定的使用范围和用途全部用于野生动物资源的保护管理、资源调查、宣传教育、驯养繁殖、科学研究等方面，不得用于发放奖金、搞基本建设、提高福利待遇或挪作他用。

十、本办法自一九九三年一月一日起执行。

附件二：

捕捉、猎捕国家重点保护野生动物资源保护管理费收费标准

国家一级保护野生动物

（元／只）	
蜂猴（所有种）	300
熊猴	1000
台湾猴	2000
豚尾猴	1000
叶猴（所有种）	5000
金丝猴	50000
长臂猿	8000
马来熊	2000
大熊猫	100000
紫貂	500
貂熊	1000
熊狸	1000
云豹	3000
豹	6000
虎	48000
雪豹	10000
亚洲象	50000
蒙古野驴	5000
西藏野驴	5000
野马	60000
野骆驼	50000
鼷鹿	500
黑鹿	2000
白唇鹿	2000

坡鹿	6000
梅花鹿	3000
豚鹿	3000
麋鹿	6000
野牛	6000
野牦牛	6000
普氏原羚	3000
藏羚	2000
高鼻羚羊	6000
扭角羚	30000
台湾鬣羚	2000
赤斑羚	2000
塔尔羊	2000
北山羊	1000
河狸	3000
短尾信天翁	900
白腹军舰鸟	900
白鹳	1000
黑鹳	2000
朱环	100000
中华秋沙鸭	10000
金雕	1000
白肩雕	1000
玉带海雕	2000
白尾海雕	2000

虎头海雕	2000
拟兀鹫	900
胡兀鹫	900
细嘴松鸡	500
斑尾榛鸡	500
雉鹑	400
四川山鹧鸪	400
海南山鹧鸪	400
黑头角雉	1000
红胸角雉	1000
灰腹角雉	1000
黄腹角雉	1000
虹雉（所有种）	2000
褐马鸡	2000
蓝鹇	2000
黑颈长尾雉	2000
白颈长尾雉	2000
黑长尾雉	2000
孔雀雉	2000
绿孔雀	1000
黑颈鹤	6000
白头鹤	2000
丹顶鹤	2000
白鹤	3000
赤颈鹤	6000

鸮（所有种）	3000
遗鸥	1000
四爪陆龟	2000
鳄晰	3000
巨晰	900
蟒	900
扬子鳄	3000
中华蛩蠊	900
金斑啄风蝶	900
国家二级保护野生动物	（元／只）
短尾猴	250
猕猴	250
藏酉猴	250
穿山甲	100
豺	500
黑熊	1500
棕熊（包括马熊）	1500
小熊猫	1500
石貂	250
黄喉貂	250
斑林狸	100
大灵猫	600
小灵猫	250
草原斑猫	250
荒漠猫	600

丛林猫	250
猞猁	1500
兔狲	250
金猫	900
渔猫	600
麝（所有种）	600
河鹿	300
马鹿（包括白臀鹿）	1500
水鹿	600
驼鹿	700
黄羊	100
藏原羚	600
鹅喉羚	200
鬣羚	600
斑羚	600
岩羊	300
盘羊	900
海南兔	50
雪兔	50
塔日木兔	50
巨松鼠	100
角piti	80
赤颈piti	80
鹈鹕（所有种）	250
鲣鸟（所有种）	80

海鸬鹚	250
黑颈鸬鹚	250
黄嘴白鹭	50
岩鹭	50
海南虎斑鳽	50
小苇鳽	50
彩鹳	1500
白环	600
黑环	600
彩环	600
白琵鹭	250
黑脸琵鹭	900
红胸黑雁	250
白额雁	80
天鹅（所有种）	80
鸳鸯	80
其它鹰类	200
隼科（所有种）	200
黑琴鸡	200
柳雷鸟	200
岩雷鸟	200
镰翅鸟	200
花尾榛鸡	80
雪鸡（所有种）	100
血雉	80

红腹角雉	200
藏马鸡	500
蓝马鸡	250
黑鹇	200
白鹇	80
原鸡	50
勺鸡	80
白冠长尾雉	250
锦鸡（所有种）	80
灰鹤	60
沙丘鹤	900
白枕鹤	900
蓑羽鹤	600
长脚秧鸡	50
姬田鸡	50
棕背田鸡	50
花田鸡	50
铜翅水雉	50
小杓鹬	50
小青脚鹬	50
灰燕行	50
小鸥	50
黑浮鸥	50
黄嘴河燕鸥	50
黑嘴端凤头燕鸥	50

黑腹沙鸡	200
绿鸠（所有种）	80
黑颏果鸠	80
皇鸠（所有种）	80
斑尾林鸽	80
鹃鸠（所有种）	80
鹦鹉（所有种）	80
鸦鹃（所有种）	50
号形目（所有种）	80
灰喉针尾雨燕	50
凤头雨燕	50
橙胸咬鹃	50
蓝耳翠鸟	50
鹳嘴翠鸟	50
黑胸蜂虎	50
绿喉蜂虎	50
犀鸟科（所有种）	200
白腹黑啄木鸟	50
阔嘴鸟科（所有种）	80
八色鸫科（所有种）	200
凹甲陆龟	50
大壁虎	50
虎纹蛙	50
伟铗虫八	50
尖板曦箭蜓	50

宽纹北箭蜓	50
中华缺翅虫	50
墨脱缺翅虫	50
拉步甲	50
硕步甲	50
彩臂金龟（所有种）	50
叉犀金龟	50
双尾褐凤蝶	80
三尾褐凤蝶	80
中华虎凤蝶	80
阿波罗绢蝶	250

本篇引用的法规

中央法规

中华人民共和国野生动物保护法

引用本篇的法规 案例 论文

部门规章

国家林业局公告2004年第3号——国家林业局行政许可事项公示内容

国家林业局关于发布破坏野生动物资源刑事案件中涉及犀牛角价值标准的通知

国家林业局关于发布破坏野生动物资源刑事案中涉及走私的象牙及其制品价值标准的通知

林业部、国家计委、财政部关于执行《陆生野生动物资源保护管理费收费办法》有关问题的通知

地方法规规章

湖南省林业厅关于公布修订的《湖南省林业厅林业行政执法依据》的通告(2011)

重庆市林业局关于印发《重庆市林业局陆生野生动植物保护与自然保护区管理行政许可(审核审批)申办指南》的通知

河南省财政厅关于公布废止失效和继续有效的规范性文件目录的通知

大连市林业局关于收缴野生动物资源管理费的通知

甘肃省人民政府关于公布行政执法机关执法依据的公告(第2号)

广东省物价局、省林业局、省财政厅关于发布广东省非国家重点保护陆生野生动物资源保护管理费收费管理办法及标准的通知

河南省财政厅、发展计划委员会、林业厅关于明确非国家重点保护陆生野生动物资源保护管理费收费标准的通知

浙江省林业厅、浙江省财政厅、浙江省物价局关于印发《浙江省陆生野生动物资源保护管理费收费办法》的通知

更多

案例与裁判文书

哈密市伊州区人民检察院与刘大晨、依某察院与刘大晨、依米拉米孜·买买提等非法收购、运输、出售珍贵、濒危野生动物、珍贵、濒危野生动物制品罪二审刑事判决书

方杨林非法猎捕、杀害珍贵、濒危野生动物罪一审刑事判决书

邓盛克等非法收购珍贵、濒危野生动物制品案

黄碧泉走私珍贵动物、珍贵动物制品一审刑事判决书

徐飞、汪付云走私珍贵动物、珍贵动物制品一审刑事判决书

江西九江长途汽车运输集团有限公司与盐城市大丰区农业委员会等林业行政处罚、行政复议纠纷上诉案

刘刚强（DANIELLIU）走私珍贵动物制品罪案

于润若等走私珍贵动物制品案

更多

法学期刊

非法收购、出售珍贵、濒危野生动物制品案的办理

非法捕杀野生动物并出售的罪数认定

走私珍贵动物及其制品犯罪案件司法实践问题研究

论我国野生动物保护法制之完善

试论环境资源有偿使用原则

野生动物资源的刑法保护及立法完善

论自然资源费制度

*注：本文格式遵循《全国人大法规备案审查信息平台电子文件格式规范（试行）》标准。

©北大法宝：（www.pkulaw.com）专业提供法律信息、法学知识和法律软件领域各类解决方案。北大法宝为您提供丰富的参考资料，正式引用法规条文时请与标准文本核对。

欢迎查看所有产品和服务。

法宝快讯：如何快速找到您需要的检索结果？ 法宝 V6 有何新特色？

扫描二维码阅读原文

原文链接：https://www.pkulaw.com/chl/728a40ea7d198132bdfb.html
