# Supplementary material for: China’s Legal Protection System for Pangolins: Past, Present, and Future
Source: Animals (Basel). 2025 Aug 18;15(16):2422. doi: 10.3390/ani15162422 (PMC12383201; doi:10.3390/ani15162422)
Supplement: Supplementary file 1 [file animals-15-02422-s001.zip › Supplementary Material S2 -Full Texts of Laws and Regulations Related to Pangolins in China/【13】非法捕杀国家重点保护珍贵、濒危陆生野生【13】动物案立案标准(FBM-CLI.4.pdf]

非法捕杀国家重点保护珍贵、濒危陆生野生动物案立案标准

制定机关： 国家林业局(已撤销) 机构沿革

公布日期：2001.05.09

施行日期：2001.05.09

时效性： 现行有效

效力位阶： 部门规范性文件

法规类别： 野生动植物资源

非法捕杀国家重点保护珍贵、

濒危陆生野生动物案立案标准

（国家林业局2001年5月9日）

中　文　名	拉　丁　文　名	级　别	立　案	重大案件	特大案件
蜂猴	Nycticebus s pp.	1	2	3	4
熊猴	Macaca assam ensis	1	1	2	3
台湾猴	Macaca cyclo pis	1		1	2
豚尾猴	Macaca nemes trina	1	1	2	3
叶猴（所有种 ）	Presbytis sp p	1		1	2
金丝猴(所有 种)	Rhinopithecu s spp	1			1

马来熊	Helarctos ma layanus	1	1	2	3
大熊猫	Ailuropoda m elanoleuca	1		3	1
紫貂	Moartes zibe llina	1	2	3	4
貂熊	Gulo gulo	1	1	1	3
熊狸	Arctictis bi nturong	1		1	2
云豹	Neofelis nel ulosa	1		1	2
豹	Panthera par dus	1		1	2
雪豹	Panthera unc ia	1		1	2
虎	Panthera tig ris	1			1
亚洲象	Elephas maxi mus	1			1
蒙古野驴	Equus hemion us	1	1	2	3
西藏野马	Equus kiang	1	2	3	5
野马	Equus przewa lskii	1			1
长臂猿（所有种）	Hylobates sp p.	1		1	2
野骆驼	Camelus feru s	1		1	2
鼷鹿	Tragulus jav anicus	1	1	2	3

黑麂	Muntiacus cr inifrons	1		1	2
白唇鹿	Cervus albir ostris	1		1	2
坡鹿	Cervus eldi	1		1	2
梅花鹿	Cervus nippo n	1	1	2	3
豚鹿	Cervus porci nus	1	1	2	3
麋鹿	Elaphurus da vidianus	1		1	2
野牛	Bos gaurus	1		1	2
野牦牛	Bos mutus	1	1	2	3
普氏原羚	Procapra prz ewalskii	1		1	2
藏羚	Pantholops h odgsoni	1	1	2	3
高鼻羚羊	Saiga tatari ca	1			1
扭角羚	Budorcas tax icolor	1		1	2
台湾鬣羚	Capricornis crispus	1	1	2	3
赤斑羚	Naemorhedus cranbrooki	1	1	2	4
塔尔羊	Hemitragus j emlahicus	1	1	2	4
北山羊	Capra ibex	1	1	2	4

河狸	Castor fiber	1		1	2
短尾信天翁	Diomedea alb atrus	1	1	2	4
白腹军舰鸟	Fregata andr ewsi	1	1	2	4
白鹳	Ciconia cico nia	1	1	2	4
黑鹳	Pseudibis pa pillosa	1	1	2	4
朱鹮	Nipponia nip pon	1			1
中华秋沙鸭	Mergus squam atus	1	1	2	3
金雕	Aguila chrys aelos	1	1	2	4
白肩雕	Aquila helia ca	1	1	2	4
玉带海雕	Haliaeetus l eucoryphus	1	1	2	4
白尾海雕	Haliaeetus a lbcilla	1	1	2	3
虎头海雕	Haliaeetus p elagicus	1	1鬣	2	4
拟兀鹫	Pseudogy ben galensis	1	1	2	4
胡兀鹫	Gypaetus bar batus	1	1	2	4
细嘴松鸡	Tetroo parvi rosiris	1	1	3	5
斑尾榛鸡	Tetrastes se werzowi	1	1	3	5
雉鹑	Tetraophasis	1	1	3	5

	obscurus				
四川山鹧鸪	Arboophila r ufipectus	1	1	3	5
海南山鹧鸪	Arborophila ardens	1	1	3	5
黑头角雉	Tragopan mel anocephalus	1	1	2	3
红胸角雉	Tragopan sat yra	1	1	2	4
灰腹角雉	Tragopan bly thii	1	1	2	3
黄腹角雉	Tragopan cab oti	1	1	2	3
虹雉（所有种 ）	Lophophorus spp	1	1	2	4
褐马鸡	Crossoptilon mantchuricu m	1	1	2	3
蓝鹇	Lophura swin hoii	1	1	2	3
黑颈长尾雉	Syrmaticus h umiae	1	1	2	4
白颈长尾雉	Syrmaticus e wllioti	1	1	2	4
黑长尾雉	Syrmaticus m ikado	1	1	2	4
孔雀雉	Polyplectron bicalcaratu m	1	1	2	3
绿孔雀	Pavo muticus	1	1	2	3

黑颈鹤	Grus nigrico llis	1	1	2	3
白头鹤	Grus monacha	1	1	2	3
丹顶鹤	Grus japonen sis	1	1	2	3
白鹤	Urus leucoge ranus	1	1	2	5
赤颈鹤	Grus antiigo ne	1	1	1	2
鸨（所有种）	Otis spp.	1	2	4	6
遗鸥	Larus relict us	1	1	2	4
四瓜陆龟	Testudo hors fieldi	1	2	4	8
鳄蜥	Shinisaurus crocodilurus	1	1	2	4
巨蜥	Varanus salv ator	1	1	2	4
蟒	Python molur us	1	1	2	4
扬子鳄	Alligator si nensis	1		1	2
中华蛩蠊	Galloisianx sinensis	1	1	3	6
金斑喙凤蝶	Teinopalpus aureus	1	1	3	6
短尾猴	Macaca arcto ides	2	2	6	10
猕猴	Macaca mulat ta	2	2	6	10
藏酋猴	Macacc thibe tana	2	2	6	10

穿山甲	Manis pentad aoctyla	2	4	8	16
豺	Cuon alpinus	2	2	4	6
黑熊	Selenarctos thibetanus	2	2	3	5
棕熊	Utsus arctos	2	2	3	5
小熊猫	Ailurus fulg ens	2	1	3	5
石貂	Martes foina	2	2	4	10
黄喉貂	Martes flavi gula	2	2	4	10
斑林狸	Prionodon pa rdicolor	2	2	4	8
大灵猫	Viverra zibe tha	2	2	3	5
小灵猫	Viverricula indica	2	2	4	8
草原斑猫	Felis lybica	2	2	4	8
荒漠猫	Felis bieti	2	2	4	10
丛林猫	Felis chaus	2	2	4	8
猞猁	Felis lynx	2	1	2	3
兔狲	Felis manul	2	1	3	5
金猫	Felis temmin cki	2	2	4	8
渔猫	Felis viverr inus	2	1	3	5

麝（所有种）	Moschus spp	2	2	4	8
河鹿	Hydropotes i nermis	2	2	4	6
马鹿（含白臀鹿）	Ceruus elaph us	2	1	3	5
水鹿	Cervus unico lor	2	1	3	5
驼鹿	Alces aices	2	2	8	15
黄羊	Procapra gut turosa	2	2	4	8
藏原羚	Procapra pic ticaudata	2	2	4	8
鹅喉羚	Gazella subq uttutosa	2	2	3	4
鬣羚	Capricornis sumatraensis	2	2	4	8
斑羚	Naemorhedus goral	2	2	4	8
岩羊	Pseudois nay aur	2	2	4	8
盘羊	Ovis ammon	2	1	3	5
海南兔	Lepus peguen sis hainanus	2	2	6	10
雪兔	Lepus timidu s	2	2	6	10
塔里木兔	Lepus yarkan densis	2	5	20	40

巨松鼠鹛	Ratufa bicol or	2	2	6	10
角鸊鹛	Podiceps aur itus	2	2	6鹛	10
赤颈鸊鹛	Podiceps gri segsna	2	2	6	8
鹈鹕（所有种 ）	Pelecanus sp p.	2	2	4	8
鲣鸟（所有种 ）	Sula spp.	2	2	6	10
海鸬鹚	Phalacrocora x Pelagicus	2	2	4	8
黑颈鸬鹚	Phalacrocora x niger	2	2	4	8
黄嘴白鹭	Egretta eulo photes	2	2	6	10
岩鹭	Egretta sacr a	2	2	6	10
海南虎斑鳽	Gorsachius m agnificus	2	2	6	10
小苇鳽	Ixbrychus mi nutus	2	2	6	10
彩鹳	Lbis leucoce phalus	2	1	3	4
白鹮鸟	Threskiornis aethiopicus	2	2	4	8
黑鹮鸟	Pseudibis pa pillosa	2	2	4	8
彩鹮鸟	Plegadis fal cinellus	2	2	4	8
白琵鹭	Platalea leu corodia	2	2	4	8

黑脸琵鹭	Platalea min or	2	2	4	8
红胸黑雁	Brantu rufic ollis	2	2	4	8
白额雁	Anser albifr ons	2	2	6	10
天鹅（所有种 ）	Cygnus spp.	2	2	6	10
鸳鸯	Aix galericu lata	2	2	6	10
其它鹰类	Accipitridae	2	2	4	8
隼科（所有种 ）	Falconidae	2	2	6	10
黑琴鸡	Lyrurus tetr ix	2	2	4	8
柳雷鸟	Lagopus lago pus	2	2	4	8
岩雷鸟	Lagopus mutu s	2	4	6	10
镰翅鸟	Falcipennis falcipennis	2	1	3	4
花尾榛鸡	Tetrastes bo nasia	2	2	6	10
雪鸡（所有种 ）	Tetraogallus spp.	2	2	6	10
血雉	Ithaginis cr uentus	2	2	4	8
红腹角雉	Tragopan tem minckii	2	2	4	8
藏马鸡	Crossoptilon crossoptilo n	2	2	4	6

蓝马鸡	Crossoptilon auritum	2	2	4	10
黑鹇	Lophura lenc omelana	2	2	6	8
白鹇	Lophura uyct hemera	2	2	6	10
原鸡	Gallus gallu s	2	2	6	8
勺鸡	Pucrasia mac rolopha	2	2	6	8
白冠长尾雉	Syrmaticus r eevesii	2	2	4	8
锦鸡（所有种 ）	Chrysolophus spp.	2	2	4	8
灰鹤	Grus grus	2	2	4	8
沙丘鹤	Grus canaden sis	2	2	4	8
白枕鹤	Grus uipio	2	2	4	8
蓑羽鹤	Authropoides virgo	2	2	6	10
长脚秧鸡	Crex crex	2	2	6	10
姬田鸡	Porzana parv o	2	2	6	10
棕背田鸡	Porzana bico lor	2	2	6	10
花田鸡	Coturnicops noveboracens is	2	2	6	10
铜翅水雉	Netopidius i ndicus	2	2	6	10

小杓鹬	Numenius bor ealis	2	2	4	8
小青脚鹬	Tringa gutti fer	2	2	6	10
灰燕鸻	Glareola lac tea	2	2	6	10
小鸥	Larus minutu s	2	2	6	10
黑浮鸥	Chlidonias n iger	2	2	6	10
黑嘴燕鸥	Sterna auran tia	2	2	6	10
黑嘴端凤头燕 鸥	Thalasseus z immermanni	2	2	4	8
黑腹沙鸡	Pterccles or ientalis	2	2	4	8
绿鸠（所有种 ）	Treron spp.	2	2	4	8
黑颏果鸠	Ptilinopus l eclancheri	2	2	6	10
皇鸠（所有种 ）	Ducula spp.	2	2	6	10
斑尾林鸽	Columba palu mmbus	2	2	6	10
鹃鸠（所有种 ）	Macropygia s pp	2	2	6	10
鹦鹉科（所有种）	Psittacidae	2	2	6	10
鸦鹃（所有种 ）	Centropus sp p.	2	2	6	10
鸮鸟形目（所	Strigiformes	2	2	6	10

有种）					
灰喉针尾雨燕	Hirundapus c ochinchinens is	2	2	6	10
凤头雨燕	Hemiprocne l ongipennis	2	2	6	10
橙胸咬鹃	Harpacies or eskios	2	2	6	10
蓝耳翠鸟	Alcedo menin ting	2	2	6	10
鹳嘴翠鸟	Pelargopsis capensis	2	2	6	10
黑胸蜂虎	Merops lesch enaulti	2	2	6	10
绿喉蜂虎	Merops orien talis	2	2	6	10
犀鸟科（所有种）	Bucerotidae	2	2	4	8
白腹黑啄木鸟	Dryocopus ja vensis	2	2	6	10
阔嘴鸟科（所有种）	Eurylaimidae	2	2	6	10
八色鸫科（所有种）	Pittidae	2	2	6	10
凹甲陆龟	Manouria imp ressa	2	2	6	10

无相关内容

*注：本文格式遵循《全国人大法规备案审查信息平台电子文件格式规范（试行）》标准。

©北大法宝：（www.pkulaw.com）专业提供法律信息、法学知识和法律软件领域各类解决方案。北大法宝为您提供丰富的参考资料，正式引用法规条文时请与标准文本核对。

欢迎查看所有产品和服务。

法宝快讯：如何快速找到您需要的检索结果？ 法宝 V6 有何新特色？

扫描二维码阅读原文

原文链接：https://www.pkulaw.com/chl/cac7d7e46bb40781bdfb.html
