# Supplementary material for: China’s Legal Protection System for Pangolins: Past, Present, and Future
Source: Animals (Basel). 2025 Aug 18;15(16):2422. doi: 10.3390/ani15162422 (PMC12383201; doi:10.3390/ani15162422)
Supplement: Supplementary file 1 [file animals-15-02422-s001.zip › Supplementary Material S2 -Full Texts of Laws and Regulations Related to Pangolins in China/【14】国务院办公厅关于加强生物物种资源保护和管理的通知(FBM-CLI.2.pdf]

国务院办公厅关于加强生物物种资源保护和管理的通知

制定机关：　国务院办公厅 机构沿革

发文字号：国办发〔2004〕25号

公布日期：2004.03.31

施行日期：2004.03.31

时效性：　现行有效

效力位阶：　国务院规范性文件

法规类别：　种子

国务院办公厅关于加强生物物种资源保护和管理的通知

（国办发[2004]25号）

各省、自治区、直辖市人民政府，国务院各部委、各直属机构：

近几年来，我国生物物种资源保护和管理工作取得了一定成效，一批具有重要经济、科研和生态价值的生物物种资源得到了保护。但由于多种原因，我国生物物种资源丧失和流失的问题还很突出。为全面加强生物物种资源保护和管理，经国务院同意，现就有关问题通知如下：

(一)充分认识生物物种资源保护和管理的重要性。生物物种资源(包括生物遗传资源，下同)是维持人类生存、维护国家生态安全的物质基础，是实现可持续发展战略的重要资源。各地区、各有关部门要充分认识生物物种资源保护和管理的重要性和紧迫性，站在国家和民族长远利益的高度，以对子孙后代高度负责的态度，

将生物物种资源保护和管理工作列入重要议事日程，确定工作重点，采取有力措施，切实抓紧抓好。

要通过广播、电视、报刊、杂志等新闻媒体，开展生物物种资源保护和管理宣传教育，广泛普及科学知识，树立生物物种资源保护意识。要针对突出问题，抓住典型案例，深入开展警示教育，不断提高全社会生物物种资源保护和管理的责任感。

(二)开展生物物种资源调查。我国生物物种资源种类多、数量大、分布广，是世界生物物种资源最丰富的国家之一。为全面掌握我国生物物种资源状况，要迅速开展一次全国生物物种资源调查，争取用二到三年的时间，基本查清我国栽培植物、家畜家禽种质资源和水生生物、观赏植物、药用植物等物种资源的状况。

(三)做好生物物种资源编目工作。开展动植物特有种、我国起源的栽培植物、家畜家禽及其野生亲缘种、变种、品种和品系，以及具有重要经济、科研价值或潜在用途的野生药用、观赏动植物和微生物等物种资源的整理和编目。要研究制定生物物种资源评价指标和等级标准，完善重点保护生物物种目录，建立国家生物物种资源协调交流机制、全国统一的数据库系统，实现信息网络联通和信息资源共享。

全国生物物种资源调查和编目工作，由环保总局会同国务院有关行政主管部门负责组织落实，各地区、各部门要积极支持和配合。

(四)制定生物物种资源保护利用规划。在开展生物物种资源调查的基础上，环保总局要会同发展改革委、科技部、财政部、农业部、林业局、中科院、中医药局

等部门制定全国生物物种资源保护利用规划。各地区、各有关部门要分别编制本行政区和相关领域的保护利用规划。各级保护利用规划要纳入国家和地方国民经济和社会发展计划并认真组织实施。

(五)加强生物物种资源保护基础能力建设。加强野生动植物物种资源及其原生境、栽培植物野生近缘种、家畜家禽近缘种的就地保护和生物物种资源收集保存库(圃)、植物园、动物园、野生动物园、种源繁育中心(基地)建设，做好生物物种资源迁地保护和保存；建设一批离体保护设施和生物物种资源基因核心库，加强动物基因、细胞、组织及器官的保存和特异优质基因的保护。

(六)健全生物物种资源对外输出审批制度。进一步建立审批责任制和责任追究制，强化生物物种资源对外输出的管理和监督。建立国家生物物种资源联络机制，对外提供及国外机构和个人在我国境内获取生物物种资源，必须按程序报经国务院有关行政主管部门同意，并将有关进出口资料信息抄报国务院环境保护部门。

(七)建立生物物种资源出入境查验制度。建立生物物种资源出入境查验制度，加强对生物物种资源出入境的监管。携带、邮寄、运输生物物种资源出境的，必须提供有关部门签发的批准证明，并向出入境检验检疫机构申报。海关凭出入境检验检疫机构签发的《出境货物通关单》验放。涉及濒危物种进出口和国家保护的野生动植物及其产品出口的，须取得国家濒危物种进出口管理机构签发的允许进出口证明书。出入境检验检疫机构、海关要按各自职责对出入境的生物物种资源严格检验、查验，对非法出入境的生物物种资源，要依法予以没收。

(八)加强生物物种资源对外合作管理。对外提供生物物种资源，涉及生物物种资源的对外合作项目，要签订有关协议书，明确双方的权利、责任和义务，确保知

识产权等研发利用的成果和利益共享，切实维护国家利益。对外合作项目必须严格遵守我国有关规定，应有我国研究人员的充分参与，所涉及的研发活动主要在我国境内进行。对于申请有关知识产权保护的生物物种资源研究开发成果，知识产权主管部门要按照有关规定加强审查，对符合条件的要予以保护。

(九)加强科学研究和技术开发。要制定专项科研计划，加强生物物种资源基础理论、保护技术和开发利用研究，开展生物物种资源遗传分析和综合鉴定，为科学保护和利用生物物种资源提供技术支撑。

(十)加强人才培养。要针对当前生物物种资源保护人才流失和业务骨干缺乏的实际，积极采取措施，创造必要条件，吸引和稳定专业技术人才，积极引进科技骨干人才，开展技术培训，切实加强专业和管理队伍建设。

(十一)加大资金投入。要建立稳定的投入机制，将所需经费列入中央和地方财政预算，不断加大投入力度，切实加强和完善生物物种资源保护基础设施建设，完善技术手段，提高生物物种资源保护和管理水平。

(十二)强化预警监督。建立生物物种资源监测预警体系，及时掌握重要生物物种资源的动态变化，科学预测近期、中期和长期发展趋势，为科学决策提供依据。开发建设项目要严格进行环境影响评价，对生物物种资源及其生长环境产生不利影响的，应制定和落实补救措施。

(十三)完善立法工作。抓紧起草生物物种资源保护法律法规，规范生物物种资源的保护、采集、收集、研究、开发、贸易、交换、进出口、出入境等活动。严格控制直接商品化利用野生资源，鼓励优先使用人工培育的生物物种资源。

(十四)加大执法力度。要明确职责，强化责任，严格执法，认真查找存在的问题并采取有力措施加以解决。当前要重点检查现有有关法律法规的执行情况，加强对有关部门和单位持有、对外交换和提供生物物种资源情况的监督检查。

(十五)加强领导和协调。生物物种资源的保护和管理涉及多部门和多领域，为避免工作重复和疏漏，国务院决定建立生物物种资源保护部际联席会议制度，统一组织、协调国家生物物种资源的保护和管理工作，部际联席会议由环保总局牵头，国务院有关部门参加。环保总局负责生物物种资源保护和管理的组织协调，会同监察部加强监督检查。教育、建设、农业、卫生、林业和中医药等部门负责本行业生物物种资源的保护和管理工作；工商、商务、海关、质检等部门负责市场和出入境管理；科技、知识产权等部门负责科研开发和知识产权管理；发展改革、财政等部门负责制订经济政策并落实所需资金。各有关部门要加强协调，密切配合，通力合作，共同做好我国生物物种资源保护和管理工作。

中华人民共和国国务院办公厅

二00四年三月三十一日

引用本篇的法规 案例 论文

部门规章

国家质量监督检验检疫总局关于加强出入境生物物种资源检验检疫工作的指导意见

国家环境保护总局关于印发《全国生物物种资源保护与利用规划纲要》的通知

国家环境保护总局关于印发“全国生态保护‘十一五’规划”的通知

国家环境保护总局、国家质量监督检验检疫总局关于加强环保用和可能造成环境危害的微生物进出口环境安全及卫生检疫管理的通知

国家环境保护总局关于贯彻落实国务院办公厅《关于加强生物物种资源保护和管理的通知》的意见

地方法规规章

宽甸满族自治县人民政府关于划定步达远等乡镇部分区域为中华蜜蜂品种保护区的决定

新宾满族自治县人民政府办公室关于划定七个乡镇为中华蜜蜂保护区的通知

桓仁满族自治县人民政府关于划定沙尖子等乡镇所辖区域为中华蜜蜂品种保护区的决定
新疆维吾尔自治区畜牧兽医局关于举办新疆畜禽遗传资源调查培训班的通知
福建省人民政府办公厅关于加强生物物种资源保护和管理的通知
中山市生物物种资源调查与保护利用规划工作方案
宁德市人民政府办公室关于认真做好加拿大一枝黄花防除工作的通知
重庆市人民政府办公厅关于加强生物物种资源保护和管理的通知
更多

*注：本文格式遵循《全国人大法规备案审查信息平台电子文件格式规范（试行）》标准。

©北大法宝：（www.pkulaw.com）专业提供法律信息、法学知识和法律软件领域各类解决方案。北大法宝为您提供丰富的参考资料，正式引用法规条文时请与标准文本核对。

欢迎查看所有产品和服务。

法宝快讯：如何快速找到您需要的检索结果？法宝 V6 有何新特色？

扫描二维码阅读原文

原文链接：https://www.pkulaw.com/chl/6dc1ac491df9a23cbdfb.html
