# Supplementary material for: China’s Legal Protection System for Pangolins: Past, Present, and Future
Source: Animals (Basel). 2025 Aug 18;15(16):2422. doi: 10.3390/ani15162422 (PMC12383201; doi:10.3390/ani15162422)
Supplement: Supplementary file 1 [file animals-15-02422-s001.zip › Supplementary Material S2 -Full Texts of Laws and Regulations Related to Pangolins in China/【15】国家林业局关于下发《关于促进野生动植物可持续发展的指导意见》的通知(FBM-CLI.4.pdf]

国家林业局关于下发《关于促进野生动植物可持续发展的指导意见》的通知

制定机关： 国家林业局(已撤销) 机构沿革

发文字号：林护发〔2004〕157号

公布日期：2004.09.09

施行日期：2004.09.09

时效性： 现行有效

效力位阶： 部门规范性文件

法规类别： 森林和野生动植物保护区

国家林业局关于下发《关于促进

野生动植物可持续发展的指导意见》的通知

（林护发[2004]157号　2004年9月9日）

各省、自治区、直辖市林业（农林）厅（局），内蒙古、吉林、龙江、大兴安岭森工（林业）集团公司，新疆生产建设兵团林业局：

为在野生动植物保护管理工作中深入贯彻落实《中共中央国务院关于加快林业发展的决定》，以科学发展观为指导，全面促进我国野生动植物可持续发展，我局研究制定了《国家林业局关于促进野生动植物可持续发展的指导意见》（以下简称《指导意见》），现下发给你们，请各地结合实际情况，认真组织学习和研究制定适应本地区实际情况的具体措施，确保《指导意见》在实际工作中得以贯彻

落实。

特此通知。

国家林业局关于促进野生动植物可持续发展的指导意见

《中共中央国务院关于加快林业发展的决定》的发布和全国林业工作会议的召开，为加快新时期我国林业发展，推动生态建设，优化林业结构，指明了方向，对进一步加强野生动植物保护，引导和规范野生动植物资源培育及合理利用提出了更高的要求。

按照野生动物保护法等法律法规的规定，各级林业主管部门承担着野生动植物资源保护、培育和合理利用的管理职责。为履行好这一职责，在党和国家的正确指导下，国家林业局和各级林业主管部门做了大量工作，逐步完善野生动植物保护配套法规和规章，健全野生动植物保护管理机构和执法队伍，加强自然保护区建设，拯救繁育濒危物种，引导及规范野生动物驯养繁育、野生植物培植及合理开发利用，大力开展资源调查、科学研究和国际合作，严厉打击破坏野生动植物资源的犯罪活动，广泛开展保护宣传教育，在改善生态环境、促进经济发展、推动科技进步等众多领域取得了令世人瞩目的成绩。但是，由于我国人口众多，人均资源量少，资源总需求量大，如果继续以利用野生动植物野外资源为主，不仅难以满足社会经济发展的需要，还将导致野外资源过度消耗，甚至导致部分珍稀濒危物种陷入灭绝的危险，从而使自然生态遭到破坏，相关产业的发展也将因资源耗尽而难以为继。为此，迫切需要加快资源培育，大力推动以利用野生动植物野外资源为主向以利用人工培育资源为主的战略转变，全面促进野生动植物的可持

续发展。

根据这一形势要求，为在野生动植物保护管理工作中深入贯彻落实《决定》，牢固树立和认真落实科学发展观，正确处理野生动植物资源保护、发展和合理利用的辩证关系，遵循野生动植物的生态特点和资源特点，在保护中开发，在开发中保护，现就全面促进我国野生动植物可持续发展，提出指导意见如下：

一、加强对野生动植物野外资源的普遍保护

针对当前我国野生动植物资源总量严重不足的状况，为有效遏制对野生动植物资源的不合理需求，促进野生动植物资源的恢复与增长，今后在切实加强野生动植物栖息地保护的同时，还要积极采取有效措施，强化对野生动植物野外资源的普遍保护。

（一）除科学研究、资源培育、公众卫生、文化交流等特殊情况外，禁止或严格限制猎捕或采挖珍稀、濒危野生动植物用于商业性经济利用的活动。

（二）禁止以食用为目的猎捕野生动物和采集国家一级保护野生植物。

（三）对野外资源达到一定数量的野生动植物，其利用须按照“资源消耗量小于资源增长量”的原则，严格实行管理，并仅限用于医药、保健、传统文化等领域，以有限资源尽可能保障国家重点需要。

（四）对局部区域、个别物种野外资源确已达到生境饱和容量或已对当地农林业

产生不利影响的情况，从维护生态的角度，可以有组织、有计划对其资源进行合理利用，并实行严格监督管理，防止资源过度消耗。

二、大力促进野生动植物资源培育

大力促进野生动植物资源培育，是增加资源总量、解决保护与利用矛盾的有效途径，不仅可为经济发展提供原材料来源，为农村种养经济提供新的种类，安置部分人口就业，并将有效缓解对野外资源的需求压力，有利于野生动植物保护。为此，在大力推进以利用野生动植物野外资源为主向以利用人工培育资源为主的战略转变过程中，针对野生动植物资源培育投入大、周期长、技术要求高和具有一定风险的特点，要积极研究制定或争取相应的政策、措施，为资源培育的进一步健康发展给予引导、扶持和保障。

（一）继续推行“商业性经营利用驯养繁殖技术成熟的陆生野生动物名单”措施，并逐批调整公布；对名单所列野生动物的驯养繁殖予以大力支持，为其进入市场提供相应的保障措施；对名单以外的野生动物，其驯养繁殖将主要限定于种源繁育、科学研究、观赏展示，以及用于保障中医药、保健品和高科技、高附加值产品等方面的利用。

（二）研究制定与野生动植物资源培育及其利用相衔接的保障政策和激励机制，逐步提高资源培育者对其培育资源的自主处置权，建立“谁投入，谁拥有，谁受益”的机制，调动全社会广泛参与资源培育的积极性，引导中医药、轻工业等需要野生动植物及其产品作原料的企业，从解决其生产所需的野生动植物原材料来源的长远发展角度，积极开展资源培育，并吸引社会力量积极参与到发展前景广、技术过关和种源来源有保障的野生动植物资源培育中来。

（三）开拓资金渠道，加大对野生动植物培育技术研究及推广的投入，着重于解决培育技术、发展种源、提供市场信息服务，逐步改变技术落后、条件恶劣、优良种源缺乏等现状，促进野生动植物资源培育向规模化、集约化方向发展，提高我国野生动植物资源培育的总体发展水平。

（四）积极争取税费减免、信贷优惠等财政扶持政策，增强野生动植物资源培育的活力和后劲。

（五）大力清理整顿非法驯养繁殖野生动物等活动，严厉打击借驯养繁殖为名猎捕、经营野外来源野生动物的违法行为，为合法从事野生动物驯养繁殖者创造良好的环境。

三、优化对野生动植物资源的宏观配置

当前我国野生动植物资源总量不足，利用需求大，保护与利用之间的矛盾十分尖锐。为妥善处理好保护和利用之间的关系，其关键是进一步强化对资源的宏观调控，优化资源合理配置，在严格控制资源消耗总量的同时，强化资源有序流通，以重点保障中医药、文化等重点领域、重点产业对资源的需求，提高资源利用效力，遏制滥用、过度消耗野生动植物资源的情况，使有限资源在社会、经济领域最大程度地发挥效益。

（一）强化国家对野生动植物资源的宏观调控手段，实行国家总量控制和市场配置有机结合的管理措施。

（二）完善野生动植物资源培育及合理利用等信息的统计分析，及时掌握行业动态，为管理决策提供准确的依据。

（三）在资源有限的情况下，重点保障野生动植物重要产品对野生动植物原材料的需求，并在原材料收购、产品经营和出口等环节给予优先资格，简化审批程序，确保资源按优化配置原则有序流转。

（四）逐步建立野生动植物及其产品拍卖制度，实现资源配置的“公平、公开、公正”，提高资源利用效率。

（五）对特殊的野生动植物资源及其合理利用，实行定点、定量、定向管理措施，严格控制资源流向。

四、建立有效的市场准入机制

长期以来，在野生动植物保护管理中，对野外的、驯养繁殖的、人工培植的和外来物种等不同来源、不同性质的野生动植物及其产品进入市场后，没有分类予以指导和区别采取管理措施。其结果是既难以强化野外种群的保护，又不利于积极鼓励野生动物驯养繁殖和野生植物培植业的发展。对此，要根据社会对野生动植物的多种需求，遵循可持续发展的原则，对不同来源、不同性质的野生动植物及其产品，研究制定分类指导管理措施，建立有效的市场准入机制，为合法进入市场的野生动植物及产品提供宽松、便捷的条件。

（一）通过各种形式公布在野生动物驯养繁殖、野生植物培植、中医药、保健品、皮革及传统工艺等领域的合法经营利用企业及其产品，特别是对国家重点保障

的野生动植物重点产品，通过扩大宣传，提高其社会知名度和可信度。

（二）大力推行野生动植物经营利用管理专用标识，对公布的野生动植物重点产品，通过统一标识管理措施予以市场准入；对列入商业性经营利用驯养繁殖技术成熟的54种陆生野生动物及其产品，分期分批纳入标识管理范围；对特殊、敏感物种的野生动植物产品，将实施强制性标记。通过上述措施，最终实现野生动植物产品的全面标识。

（三）对公布的野生动植物重点产品和标识产品，逐步依法按年度进行审批，简化其生产、经营审批程序，便利其市场流通。

五、大力支持和推动科学研究及成果推广

加快野生动植物科研步伐，促进科技成果应用，是提高野生动植物资源保护、培育和合理利用成效的重要措施。为加快野生动植物科研步伐，促进科技成果应用，要进一步争取对野生动植物科技研究的投入，增加行业科研能力，完善联合科技攻关机制，以科技进步提高保护手段，带动资源培育，提高资源利用效益。

（一）加强对野生动植物重点物种繁育、保护的研究和开发，积极推广研究成果，并对有关研究及其成果推广在资金、种源提供和政策等方面予以扶持。

（二）鼓励和引导野生动植物资源培育和合理利用单位与科研教学单位开展多种形式的合作，建立“产、学、研”连动机制，提高企业的产品开发和创新能力，加快科研成果的转化。

（三）逐步建立对野生动物驯养繁殖、野生植物培植及其合理利用的科技认证制度，加强科技咨询，推动野生动植物资源培育和合理利用科技含量的提高。

六、充分发挥民间团体的桥梁和纽带作用

充分发挥民间团体和中介组织的桥梁和纽带作用，有利于加强行业协调和行业自律，有利于防止不正当竞争和低层次、低水平重复建设，有利于引导企业自觉遵守执行国家法律法规和政策，促进整个行业的健康发展。中国野生动物保护协会、中国野生植物保护协会等民间团体和中介组织，要把握机遇、明确方向、准确定位，积极发挥行业管理和社会服务的双重功能，做好宣传教育、技术培训、信息服务和行业协调等工作，当好参谋，服务于行业的健康发展。

（一）广泛宣传和引导企业自觉遵守野生动植物保护法律法规和政策，并吸引社会力量参与到野生动植物保护事业中来。

（二）多渠道收集行业发展信息，总结分析行业现状，研究提出行业发展政策建议，指导行业健康发展。

（三）加强自身组织建设，建立、健全行业自律制度，加强行业协调，通过必要的行业内奖惩措施，引导、规范行业的健康发展。

（四）加强行业服务，适时建立野生动植物资源调配服务机构和市场信息、技术咨询机构，为从事野生动物驯养繁殖、野生植物培植及其经营利用企业提供信息、技术等多方面的服务。

七、加大执法监管力度，维护野生动植物及其产品经营秩序

乱捕滥猎、乱采滥挖和非法出售、收购、走私野生动植物及其产品，不仅严重破坏野生动植物资源，并对野生动植物及其产品经营秩序造成极大干扰。各级林业主管部门要充分发挥森林公安机关的职能作用，积极主动协调工商、公安、海关、医药、质检等各执法部门，进一步健全和强化执法监管措施，各施其责，共同打击违法破坏野生动植物资源的犯罪活动，为保护野生动植物资源和维护合法经营者权益营造良好的环境。

（一）严厉查处非法猎捕、采集野生动植物及其产品的违法活动，防止非法从野外来源的野生动植物及其产品混入市场。

（二）严厉查处非法出售、收购、运输和走私野生动植物及其产品的行为，维护野生动植物及其产品经营秩序。

（三）根据实际情况，适时组织专项打击行动，遏制猖獗破坏野生动植物资源及其生存环境等犯罪活动的势头。

（四）进一步提高野生动植物执法监管能力，强化对野生动植物及其产品的识别、鉴定技术手段，建立野生动植物执法网络，为保护执法提供快速、准确的科学证据。

八、加强野生动植物进出口管理，合理利用国内外资源，积极开拓国际市场，提高出口创汇水平

加强野生动植物进出口管理，认真履行国际公约，是国际共同保护野生动植物资源和全球生态的需要，也是在相关国际经贸活动创造良好环境、维护我国权益的需要，并关系到我国对外声誉。对此，要加强对国际野生动植物资源分布、保护形势及开发利用潜力的分析，结合我国野生动植物可持续发展的需要，研究制定相应的政策、措施和机制，促进我国短缺资源和原材料的进口，扩大我国有比较优势的野生动植物产品出口创汇，为我国野生动植物可持续发展注入新的活力。

（一）合理利用国外野生动植物资源。要不断加强与各缔约国，特别是周边国家之间的联系和交流，推进政府间定期联席会议机制，按照有关国际公约的规定和要求，疏通进口渠道，促进原材料的进口，特别是鼓励和推进加工贸易活动的开展。

（二）完善出口促进机制，积极开拓国际市场，提高出口创汇水平。研究制定有利于企业提高产品附加值、扩大出口创汇的政策措施，增强企业占领国际市场的能力。加强能力建设，提高履约能力，逐渐扩大办事处办理证书的资格，方便企业就近办理证书，提高工作效率。进一步加强与海关总署、濒科委等部门的联系沟通，完善联席会议制度，建立有效审核机制，简化程序，提高审批成效。建立进出口统计指标体系，及时掌握进出口信息，定期发布相关信息，为企业提供咨询服务。积极开展企业登记备案制度和企业信用制度，建立客观公开的信用指标评估体系，对信誉良好的企业，提供便利出口政策。

（三）促进人工繁育野生动物和人工培植野生植物的出口。积极开展人工饲养场和人工培植场登记备案，对大宗贸易物种开展人工繁育或人工培植技术评估，从科技和生产经营两方面准确把握人工繁育和人工培植野生动植物情况。建立信誉

评估体系，对技术成熟、规模较大的人工繁育或人工培植场实行年度计划审批、分批出证的便利政策。对国家已经公布放开经营的54种野生动物和非我国原生种的植物，可首先开展计划审批试点，并逐步实行年度计划审批。

（四）进一步加强对野生动植物进出口活动的监管和执法协作。加强同相关缔约国、国际刑警组织与世界海关组织的联络与协作，及时准确掌握和提供国际贸易动态和走私活动信息，履行缔约国应尽的义务，树立和维护良好的国家形象。进一步建立健全与海关的联系协作机制，严厉打击野生动植物非法走私活动，规范企业进出口行为和秩序，维护合法企业的合法权益，真正实现保护资源和促进出口的双重目标。

本篇引用的法规
中央法规
中华人民共和国野生动物保护法(2004修正)
中共中央、国务院关于加快林业发展的决定
引用本篇的法规 案例 论文
法学期刊
立法、监管与国家治理
狩猎权的法律构造

*注：本文格式遵循《全国人大法规备案审查信息平台电子文件格式规范（试行）》标准。

©北大法宝：（www.pkulaw.com）专业提供法律信息、法学知识和法律软件领域各类解决方案。北大法宝为您提供丰富的参考资料，正式引用法规条文时请与标准文本核对。

欢迎查看所有产品和服务。

法宝快讯： 如何快速找到您需要的检索结果？ 法宝 V6 有何新特色？

扫描二维码阅读原文

原文链接：https://www.pkulaw.com/chl/b2ff601af33196a1bdfb.html
