# Supplementary material for: China’s Legal Protection System for Pangolins: Past, Present, and Future
Source: Animals (Basel). 2025 Aug 18;15(16):2422. doi: 10.3390/ani15162422 (PMC12383201; doi:10.3390/ani15162422)
Supplement: Supplementary file 1 [file animals-15-02422-s001.zip › Supplementary Material S2 -Full Texts of Laws and Regulations Related to Pangolins in China/【16】国家环境保护总局关于印发《全国生物物种资源保护与利用规划纲要》的通知(FBM-CLI.4.pdf]

国家环境保护总局关于印发《全国生物物种资源保护与利用规划纲要》的通知

制定机关： 国家环境保护总局（含国家环境保护局）(已撤销) 机构沿革

发文字号：环发〔2007〕163号

公布日期：2007.10.24

施行日期：2007.10.24

时效性： 现行有效

效力位阶： 部门工作文件

法规类别： 环保综合规定

国家环境保护总局关于印发《全国生物物种资源保护与利用规划纲要》的通知

（环发〔2007〕163号）

各省、自治区、直辖市人民政府，新疆生产建设兵团，发展改革委，教育部，科技部，财政部，建设部，农业部，商务部，卫生部，海关总署，工商总局，质检总局，林业局，食品药品监管局，知识产权局，中科院，中医药局，海洋局：

为贯彻落实国务院办公厅《关于加强生物物种资源保护和管理的通知》（国办发〔2004〕25号）精神，我局联合生物物种资源保护部际联席会议成员单位共同编制了《全国生物物种资源保护与利用规划纲要》。经国务院同意，现印发给你们，请结合实际工作，认真贯彻落实。

各地区、各有关部门要充分认识生物物种资源保护和管理的重要性和紧迫性，将生物物种资源保护和管理工作列入重要议事日程，分别编制本行政区和相关领域的保护与利用规划，并纳入国家和地方国民经济和社会发展计划，认真组织实施。

附件：《全国生物物种资源保护与利用规划纲要》

二〇〇七年十月二十四日

附件：

全国生物物种资源保护与利用规划纲要

一、前言

“生物物种资源”指具有实际或潜在价值的植物、动物和微生物物种以及种以下的分类单位及其遗传材料。“生物物种资源”除了指物种层次的多样性，还包含种内的遗传资源和农业育种意义上的种质资源。而“遗传资源”是指任何含有遗传功能单位（基因和DNA水平）的材料；“种质资源”是指农作物、畜、禽、鱼、草、花卉等栽培植物和驯化动物的人工培育品种资源及其野生近缘种。

生物物种资源是人类生存和社会发展的基础，是国民经济可持续发展的战略性资源，生物物种资源的拥有和开发利用程度已成为衡量一个国家综合国力和可持续发展能力的重要指标之一。

我国是世界上生物多样性最丰富的国家之一，也是世界上重要的农作物起源中心

之一，还是多种特有畜、禽、鱼类种和品种的原产地。此外，世界著名的中国传统医药及其相关传统知识是许多相关产业的珍贵创新资源。

由于人口的快速增长、对生物物种资源的过度开发、外来物种的引进、环境污染、气候变化等原因，我国生物物种资源丧失和流失情况严重。为了进一步加强生物物种资源保护，扭转生物物种资源管理面临的被动局面，并在保护的基础上，推进生物物种资源的可持续利用，制定本规划纲要。

二、指导思想和原则

（一）指导思想

以科学发展观为统领，按照加强保护、促进可持续利用的方针，遵循生态、经济、社会发展规律，以完善的法制和政策措施为保障，以机制和体制创新为动力，以强化监督管理和宣传教育为手段，政府主导，全社会参与，促进生物物种资源的有效保护与可持续利用，为实现全面建设小康社会，促进人与自然和谐服务。

（二）原则

1、国家对领土内分布的生物物种资源拥有主权。获取我国的生物物种资源必须遵守我国的法律法规和相关政策。

2、坚持科学性和可操作性。提倡依靠科学进步和科技手段保护和持续利用生物物种资源，保护和利用措施力求务实、创新和具有可操作性。

3、实行优先保护和分级保护。采取分阶段和分级保护，确保最重要和最受威胁的生物物种资源得到优先保护。

4、促进保护与利用相协调。体现保护为主，注重可持续利用，建立保护与利用相协调的长效机制。

5、重视各利益相关方的协调与充分参与。加强各相关主管部门之间的协调以及中央与地方政府的协调，鼓励科研机构、企业和公众的广泛参与。

三、规划目标

（一）总体目标

使用现代科学技术和适用传统知识，保护生物多样性，保护物种及其栖息环境，持续利用生物物种及其遗传资源，公平分享因利用生物物种及遗传资源和相关传统知识产生的惠益，促进人与自然和谐共处。

（二）阶段目标

1、近期目标（2006-2010年）

到2010年，有效遏制目前生物物种资源急剧减少的趋势，特别是有效遏制因人为因素造成的生物物种资源急剧丧失趋势。以重点调查和普查相结合的方式，调查薄弱地区和重要类型生物物种资源本底、以及与生物物种资源相关的传统知识与适用技术，进行鉴别、整理和编目；协调和建立生物物种资源数据库和信息系统，构建生物物种资源保护与持续利用信息共享平台；建立和完善相关的管理体系、法规、政策和标准体系；配合国际公约谈判，研究并建立生物遗传资源获取与惠益分享制度；建立生物物种资源进出口管理制度，加强出入境查验，控制生物物种及遗传资源的流失。以各种措施保护生物物种及遗传资源，对特别受威胁的生物物种实施重点保护，加强保护设施建设，特别是自然保护区的规划和建设。

开发可持续利用生物物种资源的科学技术，加强人才培养，推进生物物种资源的研究开发和优良基因的挖掘。

2、中期目标（2011-2015年）

到2015年，基本控制生物物种资源的丧失与流失。基本完成相关领域的生物物种及遗传资源调查与编目，制定优先保护物种名录，完善标准体系，实现生物物种资源保护与管理的数据化和信息共享。建立以保护重要生物物种及遗传资源为目标的自然保护区、移地保护设施和种质资源库等离体保存设施，加强对这些保护设施的建设与管理。建立国内相关传统知识的文献化编目和产权保护制度；通过试点，逐步实施生物遗传资源获取与惠益分享制度；加大投入，强化生物物种及基因性状和功能的鉴别、筛选和利用，广泛进行生物物种资源可持续利用的研究与开发，使生物物种得到充分的利用。

3、远期目标（2016-2020年）

到2020年，生物物种资源得到有效保护。进一步加强生物物种资源保护，使绝大多数的珍稀濒危物种种群得到恢复和增殖，生物物种受威胁的状况进一步缓解；自然保护区及各类生物物种资源保护、保存设施的建设与管理质量得到进一步提高，资源保存量大幅度增加；相关法律制度和管理机构、生物遗传资源获取与惠益分享制度进一步完善；进一步健全国内相关传统知识的文献化编目和产权保护制度，并与国际接轨；完成一系列持续利用各类生物物种资源的技术开发，基因鉴别和分离技术逐步完善，并发掘更多的优良基因，用于农业生产和医药保健等；形成公众参与生物物种资源保护的长效机制。

四、保护与利用的重点领域

（一）陆生野生动物资源保护与利用

1、现状

我国有陆生脊椎动物约2748种，其中兽类约607种，鸟类约1294种，爬行类约412种，两栖类约435种，分别占世界兽类、鸟类、爬行类和两栖类的12.6%、13.3%、6.5%和10.8%。由于我国大部分地区未受到第三纪和第四纪大陆冰川的影响，保存有大量的特有物种。据统计，约有467种陆生脊椎动物为我国所特有，大熊猫、金丝猴、藏野驴、黑麂、白唇鹿、麋鹿、矮岩羊、朱鹮、褐马鸡、绿尾虹雉等均为我国特有的珍稀濒危陆生脊椎动物。

近年来，由于野生动物栖息地遭破坏、掠夺式的开发利用和环境污染等原因，野生动物资源面临的压力不断增大，我国有300多种陆生脊椎动物处于濒危状态。林业局1995-2000年对252个物种的调查结果显示，一些非重点保护物种，尤其是经济利用价值较高的物种资源量呈下降趋势。

建国以来，我国政府十分重视野生动物及其栖息地的保护工作，已建立各级野生动物类型自然保护区511个，面积达4000多万公顷。大熊猫、朱鹮、扬子鳄、东北虎、金丝猴、麋鹿、野马、高鼻羚羊等珍稀濒危野生动物保护工作取得积极进展。

2、存在的主要问题

有法不依，执法不严。一些地方乱捕滥猎、倒卖走私野生动物及其制品的违法犯罪活动时有发生，团伙作案、跨国走私等大案要案发案率上升的势头没有得到根本遏制；侵占、破坏野生动物栖息地和自然保护区的现象非常突出。

投入不足，保护意识不高。保护和管理资金匮乏，野生动物保护和自然保护区建设的投资和运行经费大多没有纳入财政预算。一些地方“野生无主，谁猎谁有”的旧观念还根深蒂固，保护野生动物的意识还比较淡薄。

管理机构不健全，研究队伍力量薄弱。目前，尚有10多个省份未建立野生动物管理专门机构。相关科学研究基础薄弱，专业人员缺乏，有效的科学研究和监测体系尚未建立，一些特殊物种的保护与合理利用技术研究还没有突破。

3、主要目标与任务

近期目标与任务（2006－2010年）：重点实施15个野生动物拯救工程，新建15个野生动物驯养繁育中心和32个野生动物监测中心（站）。到2010年，使全国各级野生动物类型自然保护区总数增加到525-535个，面积达4730-4750万公顷，初步形成较为完善的野生动物自然保护区网络，使90%国家重点保护野生动物得到有效保护，极大改观濒危物种的生存状况。认真履行有关国际公约，有效管理濒危野生动物物种的进出口。

中期目标与任务（2011－2015年）：进一步加强各级管理部门的能力建设，实现指挥、查询、统计、监测等管理工作网络化，初步建立野生动物保护管理体系，完善科研体系和进出口管理体系。到2015年，全国各级野生动物类型自然保护区总数增加到575-585个，面积达5070-5090万公顷，形成完整的自然保护区保护管理体系，使60%的国家重点保护野生动物种群数量得到恢复和增加，35%的国家级自然保护区达到规范化建设要求。

远期目标与任务（2016－2020年）：全面提高野生动物保护管理的法制化、规范化和科学化水平，实现野生动物资源保护和利用的良性循环。进一步增加全国野

生动物类型自然保护区数量和面积，全面提高管理质量。新建一批野生动物禁猎区、繁育基地，使我国85%的国家重点保护野生动物种群数量得到恢复和增加，使70%的国家级和50%的地方级自然保护区实现规范化建设。

4、保护与利用措施

实施野生动物拯救工程。在黑龙江省饶河、虎林和吉林省珲春等地实施东北虎拯救工程；在蒙新高原荒漠区实施藏羚羊、林麝和雪豹拯救工程；在青藏高原实施藏羚羊、普氏原羚和马麝拯救工程；在喜马拉雅地区实施喜马拉雅麝的拯救工程；在长江上游山系实施大熊猫拯救工程；在藏东南地区实施孟加拉虎和黑麝拯救工程；在湘南、闽西、赣南、粤北地区实施华南虎拯救工程；在皖南和浙西地区继续实施扬子鳄拯救工程；在滇南地区实施印支虎拯救工程；在滇南、桂南地区实施长臂猿拯救工程。

建立野生动物自然保护区。在目前自然保护区建设的基础上，至2020年，新建100个左右各级野生动物类型自然保护区。在蒙新高原荒漠区加强有蹄类动物的保护和荒漠生态系统保护区的建设，重点新建4处以保护藏羚羊和林麝为主的保护区和5处禁猎区；在四川省西部高原地区实施黑颈鹤保护工程；在四川、云南两省完成金丝猴种群及栖息地保护工程和虹雉等特有雉类栖息地保护工程，新建30条动物走廊带；在华东丘陵地区完成丹顶鹤、白鹤越冬地建设，以及黄腹角雉、白颈长尾雉等特有雉类栖息地建设；在华南低山丘陵地区实施亚洲象栖息地和海南坡鹿栖息地的自然保护区建设。

建立动物园和规模化野生动物繁育中心。根据地方条件和需要，在经济发达地区，建设地市级城市动物园或动物展区，近期和中期建设总数为50-60个。在完善现有11处野生动物救护繁育中心的基础上，新建20处规模化野生动

物繁育中心（或驯养繁殖场），解决高鼻羚羊、麝、鹿、穿山甲、灵长类、羚羊类、灵猫、野猪、紫貂、河狸、雉类、雁鸭类、鸠鸽类、观赏鸟类、陆生蛇类、巨蜥、陆龟、虎纹蛙等野生动物种源的规模化繁育及技术问题，引进羊驼、西瑞等种源进行繁育推广，进一步丰富驯养繁殖的野生动物种类。规范管理各类野生动物驯养繁育场所及其商业活动。

加强资源利用技术研究。在可利用资源本底调查和保护工作不断加强的基础上，发展相关技术，对某些有条件利用的种类合理开发其观赏、狩猎和动物制品。在2015年之前，重点加强圈养野生动物种群遗传衰退的生物学研究，加强遗传多样性的恢复技术、驯养繁殖技术和传染病的预防与控制技术以及药用动物制品有效成分的鉴定和替代品开发技术研究，加强经济野生动物产业化和规模化养殖的关键技术、转基因动物与动物制品的研制开发技术、野生动物产业状况监测技术和解决产业化关键问题的管理技术等方面的研究。加强野生动物动态监测体系、疾病控制防治预警系统以及信息系统的研究。

（二）水生生物资源保护与利用

1、现状

水生生物资源具有巨大的经济、社会和生态价值。我国水生生物资源具有特有程度高、孑遗物种数量大、生态系统类型齐全等特点，目前经调查并记录的水生生物物种有2万多种，其中鱼类3800多种、两栖爬行类300多种、水生哺乳类40多种、水生植物600多种，具有重要利用价值的水生生物种类200多个。以水生生物资源为主体形成的水生生态系统，在维系自然界物质循环、能量流动、净化环境、缓解温室效应等方面功能显著，对维护生物多样性、保持生态平衡有着重要作用。

水生生物是人类重要的食物蛋白来源。目前，我国水产品产量占动物性（肉、禽蛋、水产品）食物生产量的1/3，为保障食物安全、改善人民膳食结构和提高营养水平发挥了重要作用。2006年，我国水产品出口额达到93.6亿美元，同比增长18.7%。渔业已成为我国国民经济的重要组成部分，是促进农村经济发展、调整农业产业结构、增加农民收入的有效途径之一。

多年来，各级渔业行政主管部门在水生生物资源保护方面开展了大量工作，取得了一定成效：相继组织实施了海洋伏季休渔制度、长江禁渔期制度、捕捞许可管理制度、海洋捕捞渔船数量功率指标双控制度、海洋捕捞产量“零增长”、“负增长”计划及捕捞渔民转产转业等一系列行之有效的保护管理制度和措施；积极开展水生生物资源增殖放流活动，
1999年－2006年间，各地累计向海洋和内陆水域增殖放流各类渔业资源种苗达892.2亿尾（粒）。仅2004年－2006年间，投放各类水生生物资源种苗450.2亿尾（粒），增殖品种达90多个。建设各种类型人工鱼礁43处，总体积60余万立方米；建成国家级水产原良种场43个，省级水产原良种场168个，建立各级各类水生生物自然保护区近210个，其中国家投资建设的水生野生动植物保护区和救护中心48个，已累计救治各类珍稀濒危水生野生动物10000多头（尾）。

2、存在的主要问题

水域生态环境不断恶化。近年来，我国废水排放量呈逐年增加趋势，
2006年监测数据表明，我国主要江河均遭受不同程度污染，长江、黄河、淮河等七大水系的408个水质监测断面中，有46%的断面满足国家地表水III类标准；28%的断面为Ⅳ～Ⅴ类水质；超过Ⅴ类水质的断面比例占26%。全国近岸海域288个海水水质监测点中，达到国家一、二类海水水质标准的监测点占67.7%；三类海水占8.0%；四类、劣四类海水占24.3%。全国海域未达到清洁水质标准的面积约14.9万平方公里，其中，严重污染海域面积约为2.9万平方公里。四大海区近岸海域有机物

和无机磷浓度明显上升，无机氮全部超标，渤海、长江口、杭州湾、珠江口等经济发达地区近岸水域污染情况尤为严重。水域污染事故频繁，2006年仅渔业污染事故就发生1463起，造成直接经济损失和天然渔业资源损失36.4亿元。近岸海域和内陆水域是众多水生生物的主要产卵场和索饵育肥场，受污染影响，水域功能明显退化，水生生物的亲体繁殖力和幼体存活力降低，水域生产力急剧下降，其中渤海水域，生产水平已不足20世纪80年代的1/4。

过度捕捞造成渔业资源严重衰退。2004年，我国捕捞机动渔船数量35.6万艘，专业捕捞渔民达183万人，是世界上捕捞机动渔船最多、专业捕捞渔民数量最大的国家，其中海洋捕捞机动渔船22万艘，功率1234万千瓦，专业捕捞渔民约112万人。根据资源调查与专家评估结果，现有海洋捕捞能力已超过资源承受能力的30%以上。同时，长期以来粗放式、掠夺式的捕捞生产方式，大量非传统渔业劳动力的无序涌入，使海洋生物资源承受着日益巨大的压力。内陆渔业资源状况也不容乐观，长江流域的捕捞产量已从上世纪50年代的40多万吨下降到目前的10万吨左右。

其他人类活动致使大量水生生物栖息地遭到破坏。拦河筑坝、围湖造田、交通航运和海洋海岸工程等人类活动的增多，使水生生物生存空间被挤占，洄游通道被切断、栖息地及生态环境遭严重破坏，生存条件不断恶化。水利水电工程和海洋海岸工程对水域生态造成的不利影响不容忽视，对内陆水域中的珍稀濒危水生生物破坏尤为明显，直接导致我国水生野生动植物濒危程度不断加剧。据调查，我国处于濒危状态的水生野生动植物种类已由1988年的80个上升到目前的近500个，白鳍豚、白鲟、鲥鱼等珍稀物种濒临绝迹，或已难觅踪迹。

3、主要目标与任务

近期目标与任务（2006-2010年）：水生生物资源衰退、濒危物种数目增加的趋势得到初步缓解，过剩的

捕捞能力得到压减，捕捞生产效率和经济效益有所提高。全国海洋捕捞机动渔船数量、功率和国内海洋捕捞产量分别压减到19.2万艘、1143万千瓦和1200万吨左右；每年增殖重要渔业资源品种的种苗数量达到200亿尾（粒）以上；省级以上水生生物自然保护区总数达到100个以上。

中期目标与任务（2011-2015年）：渔业资源衰退和濒危物种数目增加的趋势得到进一步遏制，全国海洋捕捞机动渔船数量、功率和国内海洋捕捞产量分别压减到17.6万艘、1070万千瓦和1100万吨左右；每年增殖重要渔业资源品种的种苗数量达到300亿尾（粒）以上；省级以上水生生物自然保护区总数达到150个以上。

远期目标与任务（2016-2020年）：渔业资源衰退和濒危物种数目增加的趋势基本得到遏制，捕捞能力和捕捞产量与渔业资源可承受能力大体相适应。全国海洋捕捞机动渔船数量、功率和国内海洋捕捞产量分别压减到16万艘、1000万千瓦和1000万吨左右；每年增殖重要渔业资源品种的种苗数量达到400亿尾（粒）以上；省级以上水生生物自然保护区总数达到200个以上。

4、保护与利用措施

加强渔业资源重点保护。坚持并不断完善禁渔区和禁渔期制度，针对重要渔业资源品种的产卵场、索饵场、越冬场、洄游通道等主要栖息繁衍场所及繁殖期和幼鱼生长期等关键生长阶段，设立禁渔区和禁渔期，对其产卵群体和补充群体实行重点保护。继续完善海洋伏季休渔、长江禁渔期等现有禁渔区和禁渔期制度，并在珠江、黑龙江、黄河等主要流域及重要湖泊逐步推广。修订《重点保护渔业资源品种名录》和重要渔业资源品种最小可捕标准，推行最小网目尺寸制度和幼鱼比例检查制度。建立水产种质资源保护区，强化和规范保护区管理。建立水产种质资源基因库，保存水产遗传种质资源。采取综合性措施，改善渔场环境，对已

遭破坏的重要渔场、重要渔业资源品种的产卵场实施重建计划。

增殖渔业资源。统筹规划和合理确定适用于渔业资源增殖的水域滩涂，重点针对已经衰退的重要渔业资源品种和生态荒漠化严重水域，采取各种增殖方式，加大增殖力度，不断扩大增殖品种、数量和范围。合理布局增殖种苗生产基地，确保增殖种苗供应。制定国家和地方的沿海人工鱼礁和内陆水域人工鱼礁建设规划，科学确定人工鱼礁（巢）的建设布局、类型和数量，注重发挥人工鱼礁（巢）的规模生态效应。规范渔业资源增殖管理，大规模的增殖放流活动，要进行生态安全风险评估，大型人工鱼礁建设项目要进行可行性论证。

实行负责任捕捞管理。根据捕捞量低于资源增长量的原则，确定渔业资源的总可捕捞量，逐步实行捕捞限额制度。继续完善捕捞许可证制度，严格执行捕捞许可管理有关规定。加强对渔船、渔具等主要捕捞生产要素的有效监管，强化和规范职务船员持证上岗制度，逐步实行捕捞从业人员资格准入，严格控制捕捞从业人员数量。

引导捕捞渔民转产转业。积极引导捕捞渔民向养殖业、水产加工流通业、休闲渔业及其它产业转移，实行捕捞渔民转产转业扶持政策。国家财政预算继续安排减船转产专项补助资金，地方各级政府要加大投入，落实各项配套措施，确保减船工作顺利实施。对转产从事其他行业的捕捞渔民，财政、金融、税务等部门继续实行优惠政策。

加大自然保护区建设力度。加强水生野生动植物物种资源调查，在充分论证的基础上，结合当地实际，统筹规划，逐步建立布局合理、类型齐全、层次清晰、重点突出、面积适宜的各类水生生物自然保护区体系。建立水生野生动植物自然保

护区，保护白鳍豚、中华鲟等濒危水生野生动植物以及土著、特有鱼类资源的栖息地；建立水域生态类型自然保护区，对珊瑚礁、海草床等进行重点保护。加强保护区管理能力建设，完善保护区管理设施，加强保护区人员业务知识和技能培训，强化保护区内禁渔、巡航监督、跟踪监测及其他管理措施，促进保护区的规范化、科学化管理。

实施濒危物种专项救护。建立救护快速反应体系，建设水生野生动物救护中心或基地，增加应急救护专项经费，对误捕、受伤、搁浅、罚没的水生野生动物及时进行救治、暂养和放生。针对白鳍豚、白鲟、水獭等亟待拯救的濒危物种，制定重点保护计划，采取特殊保护措施，实施专项救护行动。对栖息场所或生存环境受到严重破坏的珍稀濒危物种，采取迁地保护措施。

驯养繁殖濒危物种。对中华鲟、大鲵、海龟和淡水龟鳖类等国家重点保护的水生野生动物，建设基因库、细胞库等，保存种质资源。建设濒危水生野生动植物驯养繁殖基地，进行驯养繁育核心技术攻关。建立水生野生动物人工放流制度，制订相关规划、技术规范和标准，对放流效果进行跟踪和评价。

管理濒危物种经营利用。调整和完善国家重点保护水生野生动植物名录。建立健全水生野生动植物经营利用管理制度，对捕捉、驯养繁殖、运输、经营利用、进出口等各环节进行规范管理，严厉打击非法经营利用水生野生动植物行为。根据国内外有关法律法规规定，完善水生野生动植物进出口审批管理制度，严格规范水生野生动植物进出口贸易活动。加强水生野生动植物物种识别和产品鉴定工作，为水生野生动植物保护管理提供技术支持。

监管外来物种。加强水生动植物外来物种管理，完善生态安全风险评价制度和鉴

定检疫控制体系，建立外来物种监控和预警机制，在重点地区和重点水域建设外来物种监控中心和监控点，防范和治理外来物种对水域生态造成的危害。

（三）畜禽遗传资源保护与利用

1、现状

我国畜禽等家养动物主要有猪、鸡、鸭、鹅、特禽、黄牛、水牛、牦牛、独龙牛、绵羊、山羊、马、驴、骆驼、兔、水貂、貉、蜂等20个物种，共计576个品种，其中地方品种为426个、培育品种有73个、引进品种有77个。

20世纪50年代初至70年代中期，农业部两次组织全国家畜品种资源调查，编写出《祖国优良家畜品种》以及各种家畜的品种志，为我国畜禽遗传多样性保护奠定了基础。20世纪80年代以来，开展了畜禽遗传资源调查、收集整理、品种遗传关系研究、活体和冷冻移地保护、保护和开发利用方案等方面工作。90年代末，开展了“畜禽种质资源收集、整理、评价、保存”项目，新发现了一批畜禽遗传资源，收集了畜禽种质资源动态变化信息，对绵、山羊进行了遗传多样性分析，建立了畜禽遗传资源体细胞保存体系，收集了濒危畜禽动物资源入库长期保存。并且建立了畜禽遗传资源数据库，收录了282个品种的信息。2002年，建立了畜禽遗传资源网络信息系统，收集500余个畜禽品种的信息资料，初步实现了网络查询和共享。

由于生态环境恶化、品种单一化等因素，畜禽种质资源的状况堪忧。随着新畜禽品种的推广，过去数千年来驯化的许多传统品种被遗弃，大量珍贵的遗传资源也随之损失，如上海的荡脚牛、湖北的枣北大尾羊、河南的项城猪、江苏的九斤黄鸡等已经完全灭绝。1999年调查结果表明，严重濒危畜禽品种达37个。

2、存在的主要问题

畜禽品种资源收集尚存差距，基因鉴定工作停留于表面。由于我国农业系统复杂，品种资源收集工作量大，对部分地区的畜禽种质资源未能进行深入细致的考察，致使一些品种资源未能编目并得到有效保护。对于现有畜禽品种资源的种质鉴定评价，尽管做了大量基础性工作，积累了一些鉴定数据，但基因的传递和变异规律仍需深入研究。

科学技术手段落后，研究力量薄弱。我国在基础研究，创新研究方面成果较少，缺乏具有自主知识产权的研究成果。相应专业人才缺乏、试验手段落后和技术开发力量薄弱，尚未形成完整健全的研究工作体系。

投入不足，设施与手段落后。由于我国畜禽品种资源保护工作起步较晚，保护体系不健全，保护措施不配套，资金投入严重不足，目前国家重点保护的78个畜禽品种中，有14个品种还没有保种场，近一半以上的保种场经营困难，开展保种选育工作难度很大，部分畜禽品种的优良性状严重退化或丧失。

缺乏创新机制。我国畜禽品种资源保护与开发的组织形式单一，多数资源保护场处于被动保种，对畜禽品种的培育和优良遗传基因的开发利用不够，科研工作滞后，造成多数品种保护和利用脱节。大部分地方品种未能得到很好的保护，保种场经济效益差。

新驯化动物缺乏规范管理。近年来，为开发野生动物的经济用途，各地新驯养了一些食用动物、毛皮动物、药用动物等种类，如果子狸、紫貂等，对这些驯养动物的资源情况缺乏系统的调查。另一方面，宠物的家庭饲养越来越普遍，种类也越来越多，目前对宠物类动物资源现状缺少了解。此外，对驯养动物和宠物动物

传染性疾病的防治还不够重视，许多疾病的传染机理尚不清楚。

3、主要目标与任务

近期目标与任务（2006～2010年）：开展畜禽遗传资源普查，用2-3年的时间基本查清我国现有畜禽品种（类群）的数量、分布、特性及开发利用状况，并在此基础上出版国家畜禽品种志书，逐步建立、完善畜禽遗传资源数据库和信息网；加强畜禽品种保种场、保护区基础设施建设，对濒危资源实施抢救性保护，对国家级、省级保护品种实施重点保护，国家级保护品种有效保护率要达到100%，对于实施抢救性保护的濒危品种，确保登记品种不再消失；继续加强国家家畜基因库（北京）、家禽基因库（江苏）和水禽基因库（福建）建设，增强保种能力，建立畜禽遗传资源细胞库，开展多种形式保护研究；采取现代生物技术与常规技术相结合的方法，加快新品种培育和推广的步伐，每年培育出3-5个畜禽新品种（配套系）。

中期目标与任务（2011～2015年）：根据资源调查结果，建立相应的原产地保护设施或异地保存设施。开展畜禽遗传资源库建设，完善畜禽遗传资源收集、评价及保存技术体系，实现畜禽遗传资源长期、妥善保存；鉴定和筛选一批优异畜禽种质基因，建立畜禽“优异基因核心库”，实现畜禽遗传资源的创新和有效利用。

远期目标与任务（2016～2020年）：跟踪世界畜禽遗传资源研究的动向，结合我国实际情况，逐步实现由重点收集、监测到深入评价和利用的转变，进而有针对性地、快速地、连续不断地为生产、育种和其它科研提供一批名、特、优资源及创新材料。研究出学术水平较高、实用价值较大的成果，在优势专业和新型技术等若干领域缩短与世界水平的差距，增强我国动物农业的国际竞争力。

4、保护与利用措施

继续进行国内外种质资源的考察和收集。至2010年，基本完成对已知畜禽和特种经济动物种质资源的收集入库。考察收集国外新品种和有用品种，在确保国家珍稀资源不流失的前提下，加强国际畜禽种质交换，着重引进利用价值高的品种资源，收集多样性丰富的种质资源和有益基因，并加强检疫研究和完善检疫基地。

加强畜禽遗传资源保存体系建设。在原有畜禽种质资源就地保护场的基础上，2020年前，增加30-50个畜禽原生境保护场，保护濒危受威胁的畜禽种质资源，同时完善已建畜禽遗传资源保护场和保护区建设，使更多的地方畜禽优良品种得到保护；继续加强国家家畜基因库、家禽基因库和水禽基因库的建设，建立畜禽遗传资源细胞库和DNA库。

建立畜禽遗传资源评价体系和畜禽“优异基因核心库”。采用先进技术，研究我国畜禽动物及其近缘野生种的起源、进化和分类。建立畜禽遗传资源的生产性状、品质性状、抗逆性和形态学评价体系，研究制定畜禽遗传资源评价国家或行业标准，对畜禽遗传资源进行全面、系统的评价。

加强特殊与优异基因的筛选和优异种质创新利用体系建设。研究畜禽遗传资源的功能基因组学和比较基因组学，筛选影响畜禽肉、蛋、奶、毛等畜产品产量和品质的主效基因；研究分子数量遗传学和生物信息学，对重要经济性状的主效基因进行分离、克隆、测序和定位，开展优异种质创新和利用研究。在2010年前，完成部分家畜禽种类重要经济性状主效基因的分离和克隆。2020年前，在优异种质创新和利用研究方面取得突破性进展。

大力推进畜禽品种资源开发利用，实现产业化开发。根据市场需求，有计划、有步骤、有重点地引进国外优良畜禽品种，同时开发和利用好地方畜禽遗传资源，争取在2015年前，培育出30个用于生产推广的新畜禽品种（配套系），逐步形成以自我开发为主的育种体系。以名牌品种为依托，通过严格规范和独特的生产加工方式，生产出系列化优质产品，全面带动畜禽遗传资源的开发利用。

促进畜禽遗传资源共享服务平台建设。在2010年前，完成制定畜禽遗传资源普查、数据采集和整理的国家标准与技术规范，制定畜禽遗传资源分类分级标准、编码体系；制定畜禽遗传资源数据标准和数据质量管理规范。2015年前，建成以畜禽遗传资源网络数据库系统为基础的共享服务平台。

（四）农作物及其野生近缘植物种质资源保护与利用

1、现状

我国农业历史悠久，据不完全统计，全国有农作物及其野生近缘植物数千种，其中栽培植物约1200种，主要栽培的600多种，其中起源于我国的近300种。我国农作物种质资源数量位居世界前列。

过去几十年中，我国农作物种质资源大量丧失或遭到严重破坏。一是由于作物新品种和栽培技术的提高，使大量老品种特别是农家品种遭到淘汰，虽然多数品种资源已得到收集保存，但仍有部分丢失；二是因为土地用途改变、大型水利与交通工程建设、城市扩展等，一些重要作物的野生近缘种生境遭受破坏，面积缩小或消失，如普通野生稻、药用野生稻和疣粒野生稻的栖息地和种群数量比20年前约分别减少了70%、50%和30%；三是在对外合作研究中，因保护意识不强和管理不力，造成农作物种质资源的大量流失。

我国自20世纪50年代以来，先后组织了多次农作物种质资源及其野生近缘植物的征集和考察，收集到大量的样本和标本。特别是20世纪80年代以来，国家在农作物种质资源保存设施建设方面加大了投入，已基本形成种质资源保存长期库、中期库和种质圃相配套的保存体系。目前编入全国作物种质资源目录的资源材料约40万份，其中已入国家作物种质库（圃）的为38万多份，涉及1000多个物种。对上述的种质资源已进行了主要农艺性状鉴定，多数或部分进行了主要病虫害、逆境和品质鉴定，对优良种质资源已开展了综合评价和利用研究。

我国农业野生植物原生境的就地保护工作起步较晚，从21世纪初才开始实施保护区（点）建设。目前已建成或正在建设的农业野生植物原生境保护区（点）共67个，保护的野生植物有7科、12属、14种，以野生稻、野生大豆和小麦野生近缘植物为主。

2、存在的主要问题

法律法规执行不力，原生境保护滞后。国家虽已发布了一系列有关农作物种质资源的法律法规，但由于宣传不广泛、守法意识差、执法不力等原因，生物物种资源流失现象严重，急需加强对生物物种资源引进引出的管理。目前农作物种质资源保护工作主要侧重于非原生境保存，而原生境保护直到21世纪初才开始启动。由于原生境保护工作滞后，使许多重要的作物野生近缘植物原生境遭到严重破坏，原生境保护点的建设速度远远落后于破坏速度。

本底尚不清楚，种质资源收集不全。农作物种质资源特别是作物野生近缘植物资源的普查缺乏系统性，涉及的种类少、范围小，对全国各类农作物野生近缘植物的种类和种群数量不清楚，有些门类的调查尚属空白，即使是已调查过的物种，因缺乏监测，对其数量、分布区、受威胁程度和原因等尚不清楚。农作物种质资

源收集保存量尚不足40万份，还有相当多的种质资源没有完成收集，特别是作物野生近缘植物资源和国外农作物种质资源的收集工作还很薄弱。

研究滞后，基因鉴别能力不足。虽然我国的种质资源丰富，但通过研究筛选出的具有突出利用价值的优异种质很少，能够拥有自主知识产权的功能基因资源更少，这既不能满足作物育种和农业生产的需求，也不能适应日益激烈的国际遗传资源竞争。迫切需要对已保存农作物种质资源的性状基因进行鉴别，发掘对作物育种和农业生产有益的基因。

3、主要目标与任务

近期目标与任务（2006-2010年）：继续进行农作物种质资源多样性和农业野生植物的濒危状况调查；完成农作物种质资源收集、整理、保存技术规程以及农业野生植物就地保护的技术规范和原生境保护区（点）建设技术标准的编制；继续考察收集农作物种质资源和农业野生植物，对西部地区农作物种质资源进行抢救性考察收集；完善、更新国家长期库、中期库、种质圃的设施和基础条件，增建种质圃7～9个，增建一座热带亚热带牧草中期库，并进行监测和更新保存的种质资源；应用超低温技术和试管苗技术保存特殊类型的种质资源，并研究相关的保存方法和技术；增建50～80个农业野生植物原生境保护区（点）；逐步完善管理体系和法律法规以及有关规章制度。

中期目标与任务（2011-2015年）：完成农作物种质资源多样性和农业野生植物濒危状况的调查；继续考察收集农作物种质资源，基本完成西部地区农作物种质资源的抢救性考察收集；完善国家长期库、中期库和种质圃的设施和基础条件，增建种质圃3～5个，继续监测已保存种质资源的活力并定期更新；国家长期库和中期库保存种质资源增加到40万份和30万份，国家种质圃保存种质资源增加到4.7万份；增建农业野生植物

原生境保护区（点）90～110个，建成超低温保存库和试管苗保存库各1座；管理体系和法律法规基本健全。

远期目标与任务（2016-2020年）：保护设施和技术以及保护的资源种类、数量、质量和利用水平等全面跻身世界先进行列；收集、保护、保存、利用和管理达到规范化和信息化；全国长、中期库配套，种质圃达40个，超低温保存库和试管苗库保存设施健全；原生境保护区（点）达到260个；长期保存的种质资源达到45万份；管理体系和法律法规比较健全。

4、保护与利用措施

继续进行农作物种质资源的收集。我国农作物种质资源收集工作潜力大。我国西部地区是多种作物的起源地和农业野生植物分布中心，抢救那里的农作物种质资源和农业野生植物资源。同时，要加紧国外农业种质资源的收集，满足未来农业可持续发展的需要。

建立原生境保护区（点）。加快建立农业野生植物原生境保护区（点），近期建成50～80个，中期建成90～110个，远期建成90～110个，共计260个，其中在华南和西南地区建立野生稻原生境保护点32个，在西北地区建立小麦野生近缘植物原生境保护区（点）18个，在东北、华北、华中和西北地区建立野生大豆原生境保护点36个，在华中和华东地区建立水生野生蔬菜原生境保护点15个，在西北、华北地区建立栽培牧草近缘野生种原生境保护点50个。在全国范围内建立野生牧草、野生蔬菜、野生果树、野生药材、野生花卉、野生茶、野生桑等原生境保护点99个。在西南地区建立籽粒苋、红花、藜等未被开发利用作物的农场（田）保护区，在华北和西北地区建立荞麦、燕麦、高粱等小宗作物的农场（田）保护区。

建立和完善非原生境保护设施。加强国家农作物种质资源长期库及备份库、中期库仪器设备的更新和维护，完善我国农业科学院作物专业所、全国畜禽牧草种质资源保存利用中心和地方科研单位的26座中期库。增建国家作物种质圃10～15个，近期在江苏省（或浙江省）建立1个杨梅种质圃，在河北省廊坊市建立1个无性蔬菜种质圃，在河南省、湖北省和云南省各建立1个野生猕猴桃种质圃；中期在海南省建立1个咖啡和1个香料种质圃，在新疆维吾尔自治区建立1个野生苹果圃，在华南地区分别建立热带果树圃、木薯圃、热带牧草圃、热带棕榈圃、剑麻圃各1个；远期在西南和华南地区各建立1个无性繁殖作物（已保存在圃中的物种除外）种质圃。完善已有的32个种质圃，健全2个试管苗库的配套设备和田间繁殖圃。在北京市建成1座超低温保存库。

开展农作物种质资源的更新繁殖、性状鉴定与评价。重点对入库（圃）作物种质资源的优良品质重要性状进行鉴定和评价，加强极端环境条件下的农业野生植物特异性状的鉴定。近期主要针对水稻、小麦、大豆等作物，筛选1500～2000份优异资源；中期扩展到对主要粮食作物、油料作物、蔬菜、果树、花卉、牧草、天然橡胶等，筛选2000～3000份优异资源；远期将基本完成所有作物种质资源的性状鉴定和评价，筛选3000～3500份具有重要经济价值的优异资源，加强对库存资源尤其是优质资源的繁殖利用。

开展作物种质资源优异功能基因发掘与克隆。利用现代生物技术重点进行优异基因发掘和功能基因组研究，最大限度地获得我国具有自主知识产权的标记基因，并对重要基因进行分子标记鉴定和克隆，挖掘一批新的优质基因，促进农业野生植物资源的合理利用。定位200个高产、优质、抗病虫、抗旱、抗寒、耐高温、养分高效利用及对环境友好的基因，获得与优异基因紧密连锁的分子标记，克隆出60～80个具有重要应用价值的新基因，为育种提供一批重要的中间材料及分子标记

选择技术。

建立共享技术平台，进行惠益分享试点。在鉴定评价和功能基因研究的基础上，建立快速、简便、高效的信息和实物共享技术平台，择优向作物育种、农业生产和其他研究机构提供优异种质，充分发挥优异作物种质资源的生产潜力。同时进行农作物种质资源获取与惠益分享制度的研究和试点，对引进和引出农作物种质资源进行规范管理。

（五）林木植物资源保护与利用

1、现状

我国林木植物资源极为丰富，居北半球地区森林资源的首位，拥有187个木本科（含17科藤本），1200多个木本属，分别占总科数的54.5%和总属数的38%以上；有9000多种木本植物，约占全国所有植物种数的30%，包括乔木3000多种，灌木6000多种，其中珙桐、鹅掌楸、香果树、连香树、水青冈等是我国古老类群的特有珍稀树种，银杏、银杉、水杉、金钱松、白豆杉等是我国特有的珍稀孑遗木本植物。

由于人类长期的干扰活动，诸如毁林、过度采伐和非木林产品的开发利用、外来入侵种、林业病虫害、大气污染、气候变化，以及各种灾害的破坏，林木植物资源和遗传多样性丧失非常严峻。目前，我国有17%树种面临濒危。

1991年，我国开始全国性林木遗传资源收集与保存，开展了系统的种类遗传多样性的研究与保护。近10年时间内，建成了各具特色的10个林木种质资源（活体）保存库，地跨我国寒温带、温带、亚热带、南亚热带、北热带的林木种质资源库已初具规模，保存了主要树种的大群体、种源（林分）、家系、优树、无性系等

，保存乔灌木树种、花卉等76个主要物种种质资源1.5万份。全国林木良种繁育基地保存育种材料种质3.5万余份，初步建立了林木种质资源库的技术体系。

2、存在的主要问题

林木植物资源不清。许多林木植物资源的本底及遗传变异情况不清，严重制约了林木植物资源的保护与开发利用。由于缺少有效的监测体系以跟踪监测和评价林木植物资源的动态变化状况与发展趋势，导致决策与管理的科学依据不足。

资源流失严重。一些发达国家的大型公司或科研单位大量收集我国林木植物资源，并通过生物技术，加强对生物遗传资源的控制和专利垄断。在过去一、二百年间，我国大量树种资源流失国外。总体上看，物种及其基因资源丢失呈上升趋势，尤其是我国西部地区的黄河中上游地段的灌木基因资源和南方热带雨林的基因资源丢失最为严重。

管理制度薄弱。我国尚未建立系统的林木植物资源保护和管理的法律法规，现行法规的相关内容也不具体。此外，林木植物种质资源保护的机构、人员、资金、基础设施、科技支撑能力等投入不足。林木植物资源的管理体制也需要进一步完善。

3、主要目标与任务

近期目标与任务（2006-2010年）：基本完成全国林木植物资源本底调查，特别是搞清我国特有的约1100种林木和西部地区约200种藤本植物以及灌木树种的分布和资源状况，并在资源编目基础上建立国家林木植物种质保存库；对具有重要经济价值的林木、珍稀物种和有利用价值的野生植物开展遗传多样性分析，评估其濒危程度，进而确定林木植物的优先保护名录；开发驯化当地野生树种；开发经济树种；启动红豆杉等重

点野生树种和苏铁等观赏植物为主的拯救保护项目；建立一批保护珍稀濒危林木种质资源的自然保护区和树木园。

中期目标与任务（2011-2015年）：建立完善林木植物资源监测、预警与决策管理系统，对全国林木植物资源的动态变化和病虫害发生情况进行监测，即时更新物种资源数据库；逐步建立林木植物资源保护与可持续利用的技术标准体系，实现资源管理数据化、网络化和信息化；制定并实施100个特有树种和重点经济林木植物的保护与利用计划；在重点林业省份或林区建立5-10个各有特色的林木植物资源保存库。

远期目标与任务（2016-2020年）：基本完成全国林木种质资源的收集与保存，完成大部分已收集林木种质资源的性状鉴定和基因筛选，获得一批优质基因用于林木良种培育，建立林木植物基因库；建成完备的林木植物物种资源保护与开发利用体系，完善林木植物物种资源自然保护区网络。

4、保护与利用措施

开展资源本底调查。组织全国林木植物资源调查，应用高新技术，建立林木植物资源调查、动态监测平台和先进的资源监测技术体系。2006年至2010年期间，初步摸清林木植物种类、数量、分布、濒危状况、保护状况和利用情况。同时建立我国林木植物资源动态数据库，并定期更新数据。

实施林木植物保护工程。制定我国林木植物保护名录，并对自然保护区外分布的重点保护林木植物资源适当建立自然保护区或保护点；建立国家树木园/植物园网络、乡土植物引种驯化园网络、林木种质资源保存林网络。在2015年前，分别在不同地理气候区建立20-30个树木园/植物园或引种驯化园，引种保存当地的珍稀濒危林木植物，进行栽培

繁殖与利用研究和病虫害防治研究，研究更新繁殖技术方法，建立一批珍稀濒危树种培育基地，并扩大其种群规模，实现其回归野化。

加强林木种质保存设施建设。2006-2010年，建立国家林木植物种质资源保存库（包括活体保存基因保存林、种子保存库、离体组织保存库等）和相应的种质保存圃，同时开始规划建立有特色的地区性林木植物资源保存设施，2011-2015年，在全国建立七个地区性林木植物物种资源保护中心。建立银杏、杉木等500-800个我国特有属、种林木植物的专属、种及品种资源的保护体系。建立一批珍稀树种（以用材树种为主）资源库。到2020年，基本建立我国全部特有属、种的专属、种及品种资源的保护体系。

建立林木植物资源信息系统。到2015年，建立比较完善的林木植物资源信息系统。利用高新技术建立珍稀濒危林木植物的空间地理信息系统，编制林木植物物种多样性分布图，确定我国林木植物资源保护区划。建立基于网络的林木植物资源利用信息平台，收集、处理、分析、决策和传播林木植物资源可持续利用信息，全面实现林木植物资源信息社会化共享，促进资源的保护与可持续利用。

加强林木植物种质资源开发利用研究。到2015年，对种质资源库保存的林木种质资源进行系统的性状鉴定和基因发掘，确定重要林木资源的核心种质，开发优良的基因用于林木品种改良。按照不同用途和类别，对林木植物资源进行系统的评估和化学成分测试，挖掘其资源价值，筛选出新的经济用途。对有重大用途价值的物种，采用扦插、组培、体细胞胚胎发生、细胞培养等现代生物技术，开发建立规模化快速繁殖体系，加速产业化利用。

（六）观赏植物资源保护与利用

1、现状

我国具有悠久的观赏植物栽培与应用的历史，是世界观赏植物文化最发达的国家之一，也是世界上观赏植物资源多样性最丰富的国家之一。估计我国原产的观赏植物种类达7000种。在我国原产的观赏植物中，有很多是我国特产的优良种类，如全世界200种蔷薇中，我国原产82种；全世界900余种杜鹃花中我国原产的有530种，占60%。现代杜鹃的几千个品种，其主要种源均来自我国；我国还是百合种质资源的分布中心，种质资源占全世界一半以上。我国的牡丹、丁香、翠菊、海棠、枸子、乌头等很多种野生花卉对世界花卉业的发展也起到了重要作用。

改革开放以来，我国引进了大量商品花卉和园林植物的品种资源。据估计，这类品种大约有2000个左右，主要是香石竹类、唐菖蒲类、郁金香类、菊花类、南洋杉类、樱花类、现代月季等。我国目前商品花卉生产中90%左右的品种是从国外引进的。

我国目前对野生花卉资源仍以直接利用为主，由于过度采挖，野生植物资源总量下降，许多花卉植物种群减少，趋于濒危。在经济利益驱动下，一些具有特殊经济价值的野生花卉成为国内外商业公司争相采挖的对象，如兰花、苏铁等，导致一些花卉资源遭到严重破坏，甚至消失。

2、存在的主要问题

资源本底不清。已有记载的我国原产花卉及观赏植物达1600种，但普遍认为实际野生花卉及观赏植物远多于此数，但由于缺少定义和评价标准，至今没有一个权威的名录。

重要花卉资源破坏严重。一些经济价值较高的花卉资源遭到严重破坏。我国兰花在所有产区均受到毁灭性破坏，以四川、云南和贵州等省最为突出。

原产花卉利用率低。我国野生花卉资源开发利用程度极低。我国可供利用的野生花卉资源有数千种之多，而目前真正得到开发利用的种类有限，不足总数的5%，一些珍贵的野生花卉品种还未开发利用。

国产花卉产业化水平低。我国野生花卉应用开发研究工作滞后，在野生花卉种质资源的收集、整理、保存、新品种选育、规模化生产技术应用方面的研究较少，未能解决人工繁育和产业化技术。由于直接采挖利用野生植株，致使野生花卉资源大量消耗。特别是珍稀濒危的野生花卉往往分布区狭窄，种群自我更新繁育能力不强，长期采挖势必造成这些资源面临濒危状态。

3、主要目标与任务

近期目标与任务（2006-2010年）：组织开展野生花卉资源调查，完成对重点地区如西南地区的花卉资源调查，完成100种重要原产花卉资源的调查。在调查的基础上，对野生花卉资源进行整理、编目，编制重点保护花卉植物名录和受威胁花卉植物名录。加强对现有栽培花卉品种资源的鉴定、整理和编目。开展对我国特产野生花卉繁育技术研究，为野生花卉产业化做准备。

中期目标与任务（2011-2015年）：完成全国野生花卉资源调查和编目，完成300种原产花卉资源的调查。在调查的基础上，制定保护计划，建立以保护珍贵野生花卉资源为主要目标的自然保护区、野生花卉植物移地保护中心以及花卉种质资源保存库。完成对我国原产、市场消费量大的20种花卉的繁育技术研究和产业化开发。

远期目标与任务（2016-

2020年）：在资源调查和编目的基础上，继续加强保护措施，形成花卉资源就地保护和移地保护网络体系。稳步发展野生花卉产业化。2020年前，开发出50种我国原产花卉，使之产业化，一些种类进入国际市场。

4、保护与利用措施

对野生花卉原生境实施就地保护。到2020年，在全国范围内新建50～100个野生花卉保护区或保护点，重点区域有长白山区、秦巴山区、冀南太行山区、甘肃南部、青岛崂山、舟山群岛、云南、藏东南和新疆等地，保护重点有兰科植物、苏铁属植物、野生玫瑰、百合科植物，以及山茶、杜鹃、报春花、蕨类、木兰科、蔷薇属、菊属、牡丹、芍药、攀援植物、高山花卉、虎耳草科、毛茛科观赏植物等。在现有自然保护区保护范畴中增加野生花卉资源或重点野生花卉的保护内容。

加强移地保存设施建设。对于天然群体遗传组成发生较大变化的花卉植物，以及适应性差、对环境、气候等生态条件要求严格的种类，或存在潜在破坏威胁的野生花卉，需要在其群体中收集种子或繁殖材料，并在其原生境附近营建移地保存园（圃）进行集中保存，或建立花卉种质资源库。到2015年，建成“国家野生花卉种质资源保存库”，收集保存优良的野生花卉种质资源，并通过人工繁育扩大种群数量，使一些珍贵的野生花卉资源得以长期保存和市场开发。

推动国内原产花卉的产业化生产。优先开发具有栽培历史和文化基础，适合大众消费的国内原产花卉种类。因地制宜地发展具有地方特色的野生花卉商业化生产。

引种驯化野生花卉。野生花卉多数具有特殊的观赏特性，有些种类的生态适应性广，容易繁殖和栽培，可以直接应用到城市园林绿地中，提高城市绿地的植物多

样性。到2015年，开发100种野生花卉用于当地城市园林绿化。

利用野生花卉基因资源培育新的花卉品种。选择具特别遗传性状的花卉植物作为亲本材料，利用传统育种技术和分子生物学的手段，培育新的优良品种。到2015年，挖掘30个优良基因，培育50个优良花卉品种。

利用野生花卉资源发展花卉旅游。野生花卉资源群落常形成优美的自然植物景观，可将保护野生花卉资源与花卉观赏旅游结合起来。

（七）药用生物物种资源保护与利用

1、现状

根据1983年第三次全国中药资源普查的结果，我国分布有药用植物种类涉及383科，2309属，11146种（含亚种、变种）；药用动物种类415科，861属，1581种；矿物药80种，合计12807种，在世界上位居前列。

在整个药用生物资源种类中，民间草药最多，有7000多种，约占资源种类的60%，其中相当一部分民间草药还处于比较原始的经验积累阶段。民族药有4000多种，约占30%，具有传统药学理论基础、可供直接利用的约400种左右，有100种左右与中药交叉（重复）。中药材约1200种，其中列入商品经营必备目录的有600种（包括制品和加工品），普通药店（房）的经营品种约为300－400种，进入流通领域的商品药材仅100多种。按来源分类，植物药材占85%以上，动物药材占10%左右。

常用中药材是当前中医处方和中成药制剂的主体，年总需求量超过60万吨，其中出口近30万吨，常用中药材70%的品种供应仍依赖于野生资源。在过去的25年中，中药工业产值年平均递增20%以上，国际上一些著名制药公司加强了对包括我国中

药在内的天然药物的研究开发。出口中药材的种类和数量大幅度上升，药用植物提取物的大量出口，对野生药用生物资源造成了巨大压力。

药用生物资源保护已成为生物多样性保护重点议题。目前，我国已列入国家野生植物保护名录（第一批）的药用植物有20种，列入《濒危动植物种国际贸易公约（CITES）》附录的药用植物有30种（类）。

2、存在的主要问题

法规不健全。1987年颁布实施的《野生药材资源保护管理条例》，由于执法主体、保护级别、资源状况和保护措施已经发生了重大变化，已经不能适应新的形势，迫切需要加强药用生物资源保护的立法与执法。

中药野生资源的无序利用加重了资源危机。因过度采挖，冬虫夏草、肉苁蓉、石斛、红景天、雪莲、蛤蚧等中药材品种已成为珍稀濒危物种，面临灭绝；历史上一些名贵中药品种如野山参、笕桥地黄、茅苍术、多伦赤芍、木通等已经消失。

资源本底不清。中药资源普查已长期中断，资源监测网络不健全，目前的许多数据还是20多年前第三次中药资源普查的数据，原有的资源产地信息、市场供求信息的统计渠道被取消，缺乏官方和有权威的统计数据。此外，在资源普查技术、监测系统研究、信息系统、资源保存和繁育技术等方面都存在基础薄弱的情况。

3、主要目标与任务

近期目标与任务（2006～2010年）：完成400种常用和100种珍稀濒危药用物种资源的调查研究工作，开展重要药用生物种质资源的调查、收集和保存；完成5-10个野生药用植物资源的就地保护设施建设；完成5-

10个珍稀濒危野生药用植物的移地保护设施和人工繁育基地建设；建立和完善1-2个药用植物种质资源库；开展1-2种药用生物新技术和替代品方面的研究；研究药用生物保护和利用相关政策体系，初步建立药用生物资源动态监测系统。到2010年，基本遏制药用生物资源过度利用的趋势，使其资源利用转入良性循环。

中期目标与任务（2011～2015年）：基本完成重要药用生物资源种质资源的收集、整理和保存工作，建设20～30个以药用生物为主要保护对象的自然保护区；开展对50种左右的野生药用生物的抚育保护、采收利用和栽培驯化的研究；开展100种大宗中药材规范化生产的研究和技术推广工作；建立药用生物资源动态监测系统；建立1座国家级药用植物种质资源保存库。

远期目标与任务（2016～2020年）：开展珍稀濒危药用生物资源的拯救及其替代品研究，开展药用生物的良种选育和利用生物技术生产药用活性成份的研究及其技术推广。到2020年，完成对200种野生药用生物的抚育保护、采收利用和栽培驯化的研究，基本实现主要药用物种资源的可持续利用。

4、保护与利用措施

开展药用生物资源的本底调查工作，并建立药用生物资源的动态监测体系。定期进行药用植物资源的调查，建立我国药用植物资源动态数据库。建立中药资源监测点和中药资源信息采集点，加强中药资源监测和信息采集网络建设。力争在2006年至2010年期间，完成第四次全国药用生物资源普查工作。

加强药用生物的就地保护。2015年前，建立20个药用生物资源自然保护区，如建设青藏高原中/藏药材资源保护区、新疆荒漠沙生中药资源保护区、鄂尔多斯高原

甘草、麻黄、锁阳、银柴等干旱沙生药用植物自然保护区、吉林长白山北药资源保护区、海南南药资源保护区、广西隆安龙虎山中药资源保护区、云南西双版纳中药资源保护区等。并在野生药材重点生产地区，建立10～20个生产性抚育保护区。

建立药用生物移地保护体系。根据道地药材的自然分布状况以及我国区域性气候特点，至2015年分别在东北、华北、西北、青藏高原、云贵高原、华东或华中、华南、海南等地区建立8个国家药用植物种质资源保存圃。另外，建立一些适合寒冷、干旱（荒漠）、湿地等特殊环境的小型种质资源圃，并依托动物园建立3～5个药用动物专用种质资源保存园区，形成全国药用植物种质资源收集保存网络系统。

建立中药资源综合利用示范体系。2015年前，建立5-10个中药资源可持续利用示范区，并通过示范区建设探索不同类型药材资源的可持续利用模式。为高效利用药材资源，需要积极倡导药材资源的综合利用，多用途开发药用生物资源和多部位综合开发利用药用生物资源，实现根、茎、叶、花、果实、种子等各器官的利用，提高利用效率。

加强药用生物资源生物技术研究。利用组织培养快繁技术实现珍稀濒危药用生物的快速繁殖；利用发酵培养等方法，推动药用生物资源利用新技术的开发与利用。到2015年前，建立部分野生药用物种资源的核心种质体系，开展种质基因的鉴定、整理和筛选。利用优良基因，培育优良药用生物品种，全面提高栽培药用生物资源的质量和产量，降低对野生药用生物资源的依赖。

建立合理的药用生物资源的管理机制。建立和完善药用生物资源进出口管理的相关法律、法规，稳步推进《中药材生产质量管理规划规范》（中药材GAP）的实施

，保障药材生产企业按照规范要求，生产质量合格的中药材。

（八）竹藤植物资源保护与利用

1、现状

我国竹类植物资源丰富，有500多种，占世界竹种的40%以上；竹林面积约500万公顷以上，占全世界竹林面积的25%；我国竹种资源、竹林面积、竹笋和竹材产量均居世界首位。我国热带和南亚热带地区分布有棕榈藤种3属40种21变种。

随着人口增加和经济社会的发展，大量土地利用从原生状态转为农牧业生产和城市建设，热带、亚热带竹类和藤类植物资源遭到大面积破坏，生境受到不同程度的威胁；同时，由于不合理的工业化利用，致使某些竹类和藤类物种资源处于濒危边缘。

2、存在的主要问题

资源本底不清，监测体系落后。许多竹、藤类物种资源的本底及遗传变异不清。我国竹、藤类资源调查、监测一直采用人工调查手段，周期长、效率低、精度差、实时性弱，缺少有效的跟踪监测体系来监测和评价竹、藤类植物资源的动态变化与发展趋势，影响和限制了我国天然竹、藤类资源的保护与利用。

保护不力。我国竹、藤植物保护工作处于起步阶段，工作区域性明显，任务量大，周期性长，研究与管理缺少连续的支持。一些竹种、藤种园和收集圃遭到破坏。

资源开发利用率低，资源共享不充分，资源评价工作缺少系统深入的研究。我国可供利用的竹藤类物种资源有几百种之多，而目前得到开发利用种类有限，不足

总数的5%。

3、主要目标与任务

近期目标与任务（2006-2010年）：调查我国竹藤类物种资源本底，并进行编目，建立数据库。明确需要特别关注的处于濒危和受威胁状态的竹藤类物种，对其濒危原因进行深入的研究。建立珍稀濒危竹藤类植物自然保护区，实行优先保护。

中期目标与任务（2011-2015年）：在摸清本底的基础上，初步建立全国竹类、棕榈藤类植物资源保护与持续利用的信息系统。收集、保存毛竹、早竹、麻竹、篌竹、巴山木竹、巨龙竹等主要竹种种质资源，建立完善的竹种质资源移地保存圃。

远期目标与任务（2016-2020年）：建立竹、藤类植物的种质资源保存库。通过人工繁育和扩大种群，使珍稀濒危竹藤种类回归自然。在种质资源收集的基础上，进行性状鉴定、评价和种质筛选研究，对具有经济价值的竹、藤类物种资源进行开发利用。

4、保护与利用措施

加强竹、藤类物种资源及竹、藤林环境中生物多样性保护。竹、藤林环境中生活着种类繁多的野生动物、微生物及非竹类植物，它们相互联系、相互作用，构成一个完整的竹林或藤林生态系统。建立自然保护区是保护竹林和藤林生态系统和生物多样性的最有效的措施。采取就地保护措施，重点保护箭竹属、玉山竹属、筇竹属、寒竹属、巴山木竹属等5属的近20种竹类植物，以及一些具有潜在开发价值和应用前景的竹、藤种质资源。

对于适应性差和对环境、气候等生态条件要求严格的竹藤种类进行迁地保护。拟分别在国际竹藤网络中心黄山基地、广西大青山、在福建漳州华安竹植物园和海

南省三亚市建立主要竹藤种质保存圃。

加强竹、藤物种资源清查。建立竹藤资源调查、动态监测平台和先进的资源监测技术体系，掌握现有竹藤资源现状及其消长变化规律。通过对全国的资源调查，物种鉴定，实现竹藤资源信息共享，促进竹藤资源的有效保护与合理利用。

加强竹藤类物种资源开发利用基础研究。筛选重要的核心竹藤类种质，开发优良的基因用于品种改良。按照不同用途和类别，对竹藤类植物资源进行系统的评估和化学成分测试，挖掘其资源价值，筛选出新的经济用途。对有重大用途价值的物种，采用现代生物技术，开发建立规模化快速繁殖体系，促进其可持续的高附加值产业化利用。

（九）其他野生植物资源保护与利用

1、现状

其他野生植物资源是指除农业野生植物、林木野生树种、野生观赏植物、野生药用植物、野生竹藤类植物以外的其他野生植物资源。

据《中国植物志》（1959-2004）统计，全国共有维管植物约31000种，约占全世界的10%。植物总数列全球第三。我国的植物资源有种类繁多、地域性显著、特有性突出、用途广泛、可替代性强、种质资源丰富等特点，在世界上占有非常重要的地位。据估算，在全国3万余种高等植物中，约有近半数种类在不同地区被人们所利用。其中已开发利用的重要野生经济植物有3000多种，目前还在陆续开发植物的新用途。事实证明野生植物资源及其悠久的利用历史是我国宝贵的生物学遗产，野生植物资源作为一种重要的战略资源储备，可满足未来农业和生物药业研发日益增长的需求。

其他野生植物资源面临的主要威胁包括植被破坏、生境破碎、对生物物种资源的过度开发利用以及外来种入侵和环境污染等。《中国物种红色名录》（第一卷，2004）列出的受威胁生物物种比例达15%，其中裸子植物、兰科植物等具有重要经济价值类群的受威胁比例高达40%以上。

为保护野生植物资源，各级政府先后建立了1000多个以野生植物物种资源为保护目标的自然保护区，75%左右的国家重点保护野生植物得到了保护。建有140多个植物园，移地收集保存的植物物种总数达10000多种，约占我国植物区系成分的65%。各植物园还根据自己的优势，建立了135个各具特色的专类植物园。

2、存在的主要问题

现行法律法规与管理机构不健全。目前我国尚无一部专门的野生植物保护法律，已颁布的《野生植物保护条例》操作性不强，并存在有法不依，执法不严的现象。管理机构不健全，力量薄弱，全国1/3的省市没有设立专门的野生植物管理机构。

保护意识薄弱。普通公众乃至一些地方政府部门对野生植物的保护意识仍然淡薄，滥采滥挖国家保护的野生植物现象还十分严重。

本底和保护现状不清。野生植物物种资源编目尚未完成，保护现况仍然不清楚。现有的全国及各地区植物志的编写主要是基于20世纪70-80年代以前积累的标本资料，未能真实反映最近20多年来野生植物分布的现况，尤其是近年来经济植物的消长情况以及濒危植物面临的实际状况尚不清楚。

缺乏全国野生植物资源网络信息系统。许多资源研究单位还没有认识到网络信息

的价值，缺乏信息共享意识。目前，大量植物资源信息分散在各有关科研机构，因各机构信息管理技术手段不一，许多资料可比性和可利用性差。

3、主要目标与任务

近期目标与任务（2006-2010年）：完成全国野生植物资源编目，重点是除了农作物野生近缘种、林木、花卉和药用植物资源以外的其他各类野生资源植物；对野生植物资源重点分布地区和经济植物重要地区及重点类群开展有重点的野生植物资源调查；对全国自然保护区和植物园保护的植物资源进行本底编目，了解其保护状况和野生植物受威胁现状，初步建立全国野生植物资源保护与持续利用的信息系统。

中期目标与任务（2011-2015年）：在资源调查和编目的基础上，编制全国植物红皮书，明确生物物种保护的优先等级，深入研究珍稀濒危植物的濒危状况和原因；规划新的珍稀濒危植物自然保护区，加强相关类型自然保护区的建设与管理，建立资源档案；加强野生植物移地保护设施建设，完善植物园建设和管理，建立2-3个综合性大型植物园；深入研究珍稀濒危植物的濒危状况和濒危原因。

远期目标与任务（2016-2020年）：建立和完善野生植物资源保护监测体系，包括对开发利用和贸易的动态监督；通过人工繁育和扩大种群，使大量珍稀濒危植物回归自然。在资源常规调查和保护的同时，加强重点目标野生植物资源的开发利用，并完成对野生植物资源潜在利用价值的鉴定，开发一批野生植物的医药价值和其它价值。完成国家野生植物战略资源储备库的建设。

4、保护与利用措施

加强保护区建设，新建一批保护特有植物资源和重要物种的保护区。在天山地区建立中亚特有属沟子荠、天山特有属疆堇天山紫草等物种的保护区和保护点，在

准噶尔地区新建保护梭梭等荒漠植物的保护区；在青海可可西里地区新建保护昼笔菊、藏木蓼、蚤草和骆驼蓬等的保护区或保护点；在山东青岛附近岛屿新建耐冬山茶保护区；在华北平原和山地新建特有属蚂蚱腿子和独根草的保护区；在四川甘孜阿坝地区新建劈碱草保护区等等。

调查全国野生植物资源本底。以重点调查和普查相结合方式，调查全国各地野生植物物种及遗传资源本底，进行编目，了解其分布和保护状况以及野生植物受威胁现状。至2015年，重点调查野生植物分布关键地区，如西南石灰岩地区、武陵山地区、浙闽赣交界山区、青藏高原地区及西北干旱、半干旱地区。普查重点是各类经济植物的野生资源储量、利用方式、过去几年的市场行情及资源消长量。在调查的基础上，对全国野生植物资源综合状况进行评估。分别提出可充分利用物种资源、保护性利用物种资源和限制性利用物种资源的名单和目录。

建立和完善国家野生植物种质资源库和植物园体系。到2015年，建立并完善我国野生植物移地保护的网络体系，增建5-6个国家级野生植物移地保护植物园和50-80个地区和城市植物园。建立一批专业类型植物园（圃）和种质繁殖基地：加强植物园体系建设，统一规划全国植物园的引种栽培计划，提升植物园迁地保育的科学研究水平。

开发和应用陆生野生植物资源可持续利用新技术。到2015年，一方面对现有的成熟技术进行全面系统的分析总结，制定野生植物资源可持续利用技术指南；另一方面，根据需要制定新开发可利用植物资源名单并提供相应的可持续利用技术。鼓励开发驯化、栽培野生植物的新技术，鼓励对有潜在利用价值的野生植物开展核心化学成分分析，鼓励开发我国特有资源的自主创新技术活动。开展重要经济植物和珍稀濒危物种的人工繁育技术研究，开发生物能源技术。

（十）微生物资源保护与利用

1、现状

我国生态类型具有多样性和代表性，有丰富的微生物资源和特有类型。目前，我国真核微生物已知物种数约9000种，仅占我国估计物种数的4%，占全世界已知种数的11%，其中可人工培养的种数约800-1000种。在我国已查明的真核微生物中，我国特有种超过2000种。由于我国原核微生物资源缺少全面调查研究，尚无法估计已发现的物种数量。

我国第一个专业微生物菌种保藏机构是成立于1951年的中国科学院菌种保藏委员会。随后，又陆续建立了医学和工业微生物菌种保藏机构。1979年，原国家科委批准成立中国微生物菌种保藏管理委员会。目前，我国共有16个保藏中心（包括香港、台湾各一个）在世界菌种保藏中心注册，注册保藏各类微生物菌种61623株（其中台湾保存10398株）。相比之下，我国微生物菌种资源的占有量与资源丰富大国的地位极不相符。根据2005-2006年环保总局组织的调查统计，我国大陆区域共保存各类微生物菌种资源约29万株，主要分散保存在近50个微生物学研究单位，共有9个保藏中心出版发行了各自的菌种目录，登载各类共享微生物菌种20862株。这些菌种保藏单位，每年向社会提供各类微生物菌种估计在3万株左右。

2、存在的主要问题

不合理的开发使微生物资源受到威胁。自然界中的微生物易受环境因素的影响，一旦原始环境遭到破坏，其原有的微生物物种区系组成将发生变化，一部分物种甚至消失灭绝。

资源本底不清，缺少菌株动态调查和编目信息系统。除放线菌和根瘤菌，我国原核微生物资源缺少系统和全面的调查研究。对于我国已鉴定和保存的微生物菌种数量已有比较清楚的数据，但是在菌株水平上，资源本底仍然不清。微生物菌株保存信息系统也尚未建立。微生物资源拥有数量的严重不足，已成为制约我国生命科学研究和生物技术产业发展的瓶颈之一。

研究工作不充分，开发利用率低。基础研究方面与发达国家相比尚有差距，在应用研究方面大多是模仿国外，对已经开发利用的微生物资源，资源共享不充分，实现共享的菌种资源不到30000株。资源评价工作目前处于表观性状或产量性状层面，缺少系统研究，无法揭示菌种全面的生物学特性，为资源开发所能提供的信息有限，综合开发利用率低下。

3、主要目标与任务

近期目标与任务（2006-2010年）：查明各研究机构保存的微生物菌种和菌株，重点调查和收集具有重要应用前景的微生物资源，加强微生物资源库的能力建设和设施建设，重建我国微生物资源保存和共享体系，根据需要引进国外重要经济微生物菌种和菌株，系统开展重要微生物资源的编目和收集保存。到2010年，我国微生物资源储备超过35万株。

中期目标与任务（2011-2015年）：充分利用现代生物科学技术的最新成果，建立微生物资源发掘、分离培养、保存、评价的完整技术体系，实现微生物物种和基因资源收集、保存、研究、开发利用的有机整合。到2015年，我国微生物遗传资源储备超过40万株。同时开发农业生物制剂用微生物菌种资源，开发安全、健康的微生物食品或食品添加剂，提高我国食品安全保障水平。

远期目标与任务（2016-

2020年）：到2020年，我国微生物资源储备超过45万株。同时通过大规模筛选和提取微生物生物活性物质，研制一批抗肿瘤、抗真菌、抗病毒、新型人用、畜用和农用抗生素等微生物药物。利用微生物技术，大幅度提高再生能源的生产效率，突破环境生物修复、整治技术。在微生物资源领域取得一批具有自主知识产权的新资源和新技术。

4、保护与利用措施

加快微生物资源的查明和编目工作。抓紧微生物资源调查，重点调查和收集具有重要应用前景的微生物资源，逐步摸清我国微生物资源家底。在资源调查中，要特别关注我国特有自然生态地区内的微生物资源，对不同生态地区进行广泛调查、分离和收集，开展系统学、分类学研究，以及类群之间亲缘关系和系统演化理论的探讨。资源调查中还要特别重视极端环境微生物资源和污染环境微生物资源，选择具有特殊化学因子的盐湖、碱湖、热泉、深海等，发展采样、分离、培养等新的方法技术，进行系统的物种及基因分析。

建立国家微生物资源库与共享体系，并进行系统的研究工作。我国虽已建立了微生物菌种保藏体系，但其规模、机制、功能已不能适应当今科学研究和应用开发的发展需要。需要整备、重建高水平的国家微生物菌种资源保存与管理体系，以及信息资源共享服务体系。到2015年，共享微生物菌种超过10万株，保护的微生物物种达5000种，为工农业生产、环境保护、科研教育提供优质微生物遗传资源、信息资源和技术保障。需要对微生物资源进行系统和深入的研究工作，特别是结合疫病等传染病防治工作，对病原微生物的传播机理进行深入研究。

依靠科学技术进步不断开发微生物资源潜力。不断发展能够展示微生物资源特性的检测或筛选技术，发展新的理论和技术，建立不同技术集成平台，包括组合化

学技术、分子育种技术、微阵列技术、高通量筛选技术等。在此基础上，研究和建立高效筛选模型，缩短资源的开发利用周期。至2015年，要集中研究力量，有计划地采集和分离微生物菌种及菌株，对已分离的菌种及菌株进行保存、评估和利用。

开发利用各类微生物资源。注重调查和收集土壤中具有药用价值的微生物资源，从中寻找各种生理活性物质，如各种酶抑制剂、免疫调节剂、受体拮抗剂和激动剂、离子载体、类激素、抗氧剂、生物表面活性剂和抗辐射药物等。到2020年，开发出100个微生物新药。深入研究与生物固氮、生物防治以及和纤维素、木质素等自然资源的合理利用有关的微生物资源，促进其在我国农业生产中发挥重要作用，包括微生物饲料和现代微生物农药以及现代微生物肥料的应用，到2020年，人工驯化栽培珍稀食用菌达100种。

加强生物能源的研究。通过微生物发酵技术，利用有机废物转化产生再生能源，利用微生物产生氢和生物电池。此外，还要加强微生物在生态环境保护方面的应用，利用微生物的分解特性，处理生产和生活中的有机废弃物，净化环境。

（十一）与生物物种资源相关的传统知识保护与利用

1、背景

传统知识是指当地居民或地方社区经过长期积累和发展、世代相传的，具有现实或者潜在价值的认识、经验、创新或者做法。与生物物种资源相关的传统知识在食品安全、农业和医疗事业的发展中，发挥着重要的作用。我国历史悠久，民族众多，各族劳动人民在数千年的实践中，创造了丰富的保护和持续利用生物多样性的传统知识、革新和实践。

近年来，与生物物种及遗传资源相关的传统知识保护问题已经成为《生物多样性公约》（CBD）和世界知识产权组织（WIPO）乃至世界贸易组织（WTO/TRIPS）等关注的重要议题，也是发展中国家与发达国家争论的焦点之一。

《生物多样性公约》提出，鼓励公平分享因利用土著传统知识、创新和实践而产生的惠益，要求各缔约国，依照国家立法，尊重、保护和维持土著和地方社区体现传统生活方式并与生物多样性保护和持续利用相关的知识、创新和实践，促进其广泛利用，鼓励公平地分享因利用此等知识、创新和做法而获得的惠益。

随着履行《生物多样性公约》的深入，传统知识对于生物遗传资源的利用以及生物多样性保护的作用日益显现，成为《生物多样性公约》后续谈判新的热点问题。2004年，《生物多样性公约》第七次缔约方会议已决定成立“传统知识特设工作组”，研究在习惯法和传统做法的基础上建立保护传统知识的专门制度。

2、存在的主要问题

传统知识往往被视为公知领域的知识，权属不明确。许多与生物物种及遗传资源相关的传统知识是传统群体共同创造并世代相传的成果，其权属关系复杂，有的很久以前就已经文献化，或者以其他方式进入公知领域；还有的是以严格保密的方式由直系亲属或者师傅口头传授，没有文献化资料。这些都给传统知识的知识产权保护增加了难度。

现有专利制度要求，申请专利必须符合新颖性、创造性和实用性三个标准。传统知识因其公知性，不符合其新颖性条件。有些传统知识如传统的中药、藏药等，不像西药那样可以确切地表达其分子结构，难以清晰地界定其保护范围。另外，中药等复方是由多味中药材制成的产品，增减药味可能难以确定其侵权行为。

传统知识流失及失传现象严重。许多传统知识在尚未获得现代知识产权制度充分认可之前就已经流失国外，并被广泛流传和商业开发利用，而传统知识的持有人却不能分享利益。

3、主要目标与任务

近期目标与任务（2006-2010年）：密切关注《生物多样性公约》、世界知识产权组织、世界贸易组织等在传统知识保护方面的谈判进展，研究并制定传统知识保护的相关法律、法规、政策、保护方案与措施，建立遗传资源和相关传统知识获取与惠益分享制度。在全国范围内全面调查生物资源相关传统知识，并进行系统文献化编目，2010年前重点调查传统医药和传统农作物、畜禽品种资源，特别是加强少数民族地区相关传统知识的调查。

中期目标与任务（2011-2015年）：继续全面进行相关传统知识调查，除传统医药和传统农业品种资源，还要调查与保护和持续利用生物多样性相关的传统生态农业方式、社区生活方式和传统食品、工艺品加工技术，以及与生物多样性相关的民族文化与宗教文化，并对调查的相关传统知识进行系统文献化编目，建立数据库。结合国际上相关传统知识的谈判进展，研究制定和完善保护传统知识的相关政策、法规与制度，制定并完善遗传资源和传统知识获取与惠益分享的制度和机制，要求专利申请者必须披露所使用传统知识和遗传资源的来源。

远期目标与任务（2016-2020年）：2020年前基本完成全国传统知识的调查和数据库建立；通过评估，制定传统知识保护目录，继承、弘扬和推广具有应用价值的传统知识。建立和完善有效的传统知识保护制度，普遍实施专利申请中必须披露所使用传统知识和遗传资源来源的制度，确保在共同商定条件下与传统知识拥有者分享惠益。

4、保护与利用措施

开展中医药传统知识调查、登录与编目。2006-2010年，由相关主管部门组织实施全国传统医药知识调查，在全国普查的基础上，重点调查云南、贵州、西藏、四川、内蒙古、新疆等省区的民族医药，包括藏药、苗药、侗药、彝药、傣药、蒙药、维药等少数民族医药传统知识。建立国家传统医药知识登记制度，使用统一标准，记录整理传统医药知识、疗法、原产地区、发明年代、知识持有人（社区）、使用历史、惠益分享实践、资源现状、引出或流失情况。

开展与遗传资源相关传统知识的调查、登录与编目。2011年至2015年，继续进行民族医药传统知识调查、登录与编目，并扩展到整个中医药和民间草药传统知识。同时开展与遗传资源相关的传统知识、创新及实践方面的调查，重点是传统品种资源和传统栽培与育种技术的调查和文献化整理，包括品种资源的性状特性、遗传组成、生物学特性、特别优良性状、选育和栽培年代、原始培育社区、保存地、品种权人、引出推广地区、产生效益和惠益分享情况等。

开展与生物多样性相关传统农业方式和传统民族文化的调查、登录与编目，包括传统加工技术、农业生产方式和与生物多样性保护与持续利用相关的民族习俗、艺术、宗教文化和习惯法等。整理、评估和研究其知识的内核、文化根源、发展历史、对生物多样性影响效果、原产地、影响范围、推广应用等。

采取适当措施，有效保存、继承和发展具有应用价值的传统实用技术，特别是总结推广对生物多样性有利的农业生产技术。2006年至2015年，集中力量在对西南、西北地区少数民族农业传统知识和技术进行总结和推广。利用生态学理论和现代先进技术，对传统知识和技术进行理论总结和技术改良。

研究制定传统知识保护政策、法规与制度。研究保护传统知识的特殊制度，建立遗传资源及相关传统知识来源的合法性证明制度。开展传统知识其知识产权性质及其保护方式的研究工作，争取在理论研究和相关保护制度的建设方面有所进展，加强传统知识管理的能力建设。

（十二）生物物种资源出入境查验体系建设

1、背景

我国是世界上生物遗传资源最丰富的国家之一，也是发达国家搜取生物遗传资源的重要地区。过去的一二百年间，我国大量的物种及其遗传资源被国外研究人员和商业机构搜集引出。一些资源在国外经生物技术加工后，形成专利技术或专利产品再销至国内，造成国家利益的重大损失。我国流失的物种及遗传资源大部分是通过非正常途径流入国外，除了国外人员和国外机构的非法搜集、走私、剽窃外，还包括邮寄国外、出境携带、对外研究合作带出等方式，而进出境管理制度的不完善是导致许多生物物种及遗传资源流失国外的直接原因。

2、存在问题

缺少必要的执法依据。目前，国家在生物物种资源出入境管理方面，立法尚属空白，未对禁止和限制出入境的生物物种品种及出入境审批方式作出明确和具体的规定，给口岸执法工作带来很大困难。

缺乏必要的甄别知识。生物物种资源多种多样，既包括植物、动物和微生物物种，又包括种以下的分类单位及其遗传材料，口岸执法人员缺乏必要的甄别知识，给查验工作带来很大困难。

缺少有效的查验手段。携带生物物种资源出境的载体多种多样，可以是传统的动植物活体及其部分或其标本，也可以是菌株、组培体、胚胎，甚至可能是细胞培养液、克隆载体等，可以随身携带，也可以夹杂在行李之中，除了传统的动植物活体及其标本外，海关现行常用设备很难检查出来。

缺乏必要的检验检测力量。由于目前动植物检验检疫专业人员主要来自兽医、植物保护等专业，知识结构侧重于疫病疫情的检测，而在动、植物分类鉴定方面的技术力量比较缺乏，需要配备专门从事生物物种资源检验检测的专业人员。

缺少快速准确的鉴定技术。现场鉴定的基本要求是快速准确，但是由于生物物种资源的范围十分广泛，检验检疫人员依据常规显微镜等设备难以进行快速准确鉴定，特别是细胞培养液、克隆载体等遗传物质，必须采用分子生物学等各种高科技检测手段，需要有针对性地研发新的鉴定技术。

3、主要目标与任务

近期目标与任务（2006-2010年）：研究相关法律的制定和对现有法规的完善，研究制定和完善生物物种及其遗传材料出入境管理制度，包括对出境生物物种及其遗传材料审批、申报和查验制度。研究先进的查验鉴定技术，达到准确和快速查验、检测的要求。加强对相关工作人员的专业培训，使其具备必要的专业知识，以提高执法水平。

中期目标与任务（2011-2015年）：在完善法规制度的基础上，有效实施对旅客携带和邮寄生物物种及遗传材料的出入境管理，在主要口岸初步具备快速准确查验鉴定生物物种及遗传材料的技术能力，具备较强的专业技术力量。

远期目标与任务（2016-2020年）：将实践中行之有效的管理制度和查验、检测技术手段推广到全国所有

口岸和国际邮局，使其制度化和程序化，同时不断完善查验、检测手段。

4、措施

加强对公众的宣传教育。在各个出入境口岸设置海关、检验检疫宣传标识、公告栏，发放检验检疫宣传册，加大宣传力度；系统地通过媒体、网络、科普读物、生物物种保护宣传周（日、月）等多种方式开展国家对生物物种资源保护的法律法规宣传，以提高出境旅客及公众，特别是科研人员和涉外人员的生物物种资源保护及自觉守法意识。

建立生物物种资源出入境查验制度。加强对生物物种资源出入境的监管。携带、邮寄、运输生物物种资源出境的，必须提供有关部门签发的批准证明。涉及濒危物种进出口和国家保护的野生动植物及其产品出口的，需取得国家濒危物种进出口管理机构签发的允许进出口证明书。出入境检验检疫机构、海关要依法按照各自职责对出入境的生物物种资源严格执行申报、检验、查验的规定，对非法出入境的生物物种资源，要依法予以没收。

配备先进查验、检测设备。在全国31个省、市、自治区的148个旅客和国际邮件进出境重点口岸配备先进的查验、检测设备，加大出入境查验、检测力度。

加强培训，提高查验、检测准确度。加强专业知识培训，分批为一线工作人员举办相关知识的培训，使一线工作人员了解和掌握生物物种有关基本知识，增强查验、检测意识，提高查验、检测准确度。

加强快速检测技术设施建设。研究建立快速、灵敏的核酸鉴定方法，建立生物资源的物种和品种指纹图谱，制定标准检测方法，研制标准检测试剂，并研究建立

标准化生物资源指纹图谱数据库。并在北京、上海、广州、昆明、厦门建立生物物种资源出入境检测鉴定实验室。

五、近期优先行动领域与优先项目（2006-2010年）

优先行动一：生物物种及遗传资源的查明与编目

优先项目：

1、西南石灰岩地区、横断山脉地区等生物多样性关键地区野生动植物资源调查与编目；

2、全国特有珍贵林木树种、药用生物、观赏植物、竹藤植物等资源调查与编目；

3、全国重点地区（如西部地区）水生生物资源调查与编目；

4、重点地区农业野生植物与栽培作物品种资源、畜牧遗传资源调查与编目；

5、中国特有动物和特殊生态区及干旱、半干旱地区动物资源调查与编目；

6、全国保藏微生物资源调查、特定环境中微生物资源的调查与编目、以及人和动物重要及新发病原微生物资源的查明与编目；

7、西南地区相关传统知识（民族传统作物品种资源、民族医药、乡土知识、传统农业技术）的调查、文献化编目及数据库建立；

8、制定各类生物物种资源清单目录（包括禁止交易类、限制交易类、自由交易类），加强对进出口贸易的监督与管理。

优先行动二：生物物种资源就地保护

优先项目：

1、农业野生植物资源和草种质资源的就地保护网络建设；

2、重要林木树种、竹藤类种质资源、野生花卉和野生药用植物资源的就地保护与网络建设；

3、重要野生动物资源、畜禽近缘动物种及畜禽品种资源就地保护设施建设；

4、重要水生生物资源就地保护设施建设。

优先行动三：生物物种资源移地保护与种质资源库建设

优先项目：

1、珍稀濒危野生动物保护拯救工程与繁育中心建设；

2、重要农业野生植物、草类与牧草植物资源库、圃建设和超低温及试管苗保存库的建设；

3、重要林木、竹藤类植物、野生花卉和野生药用植物等种质资源库、移地保护设施和人工繁育基地建设；

4、野生动物、家畜禽动物种质资源离体保存设施建设与“优异基因核心库”建立；

5、渔业资源增殖种苗基地及水产种质资源保存设施建设；

6、人工繁育物种种群的野化与回归自然工程；

7、全国微生物菌种资源保存库建设。

优先行动四：生物物种资源保护与持续利用新技术研究

优先项目：

1、珍稀濒危物种人工繁育技术及人工繁育种群回归自然的技术研究；

2、野生植物与农作物种质资源的性状鉴定、基因克隆与利用技术研究，包括原产特有珍稀饲用野生植物种质资源保存与利用技术研究；

3、珍贵林木、竹藤植物、中国原产花卉资源繁育技术研究与产业化技术开发；

4、药用生物有效成分提取技术、现代生物技术研究和濒危药用生物的繁育技术研究；

5、野生动物与畜禽优质基因资源开发与利用，以及动物仿生资源的开发利用与新材料仿生技术研究；

6、水生生物资源保护与增殖（含人工鱼礁建设）技术研究；

7、经济微生物资源新用途的开发技术、难培养微生物基因资源的筛选与开发利用；

8、高效、新型生物农药产品的研制与生产；

9、生物芯片等高新技术的研制与开发。

优先行动五：建立生物物种资源保护与持续利用数据信息系统

优先项目：

1、编制全国生物物种资源数据管理规划和计划，建设和完善全国生物物种资源信息网络系统；

2、建立和完善各类生物物种资源数据库体系，建立国家生物物种资源公共信息网络和基础数据平台；

3、建立和维护“国家生物物种及遗传资源信息交换所机制”（CHM）。

优先行动六：建立生物遗传资源获取与惠益分享法规制度体系

优先项目：

1、研究传统知识定义，制定重要传统知识保护名录，并制定生物资源与传统知识的知识产权保护制度；

2、研究并建立专利申请中要求披露生物遗传资源来源地证书的制度；

3、建立处理生物遗传资源和相关传统知识的机构和信息交换机制；

4、研究生物遗传资源和传统知识保护数据库的建设，制定遗传资源及相关传统知识保护名录。

优先行动七：研究建立持续利用生物物种资源政策体系

优先项目：

1、生物物种资源价值体系与纳入国民经济核算体系研究，以及生物物种资源保护纳入国家和地方国民经济与社会发展计划的机制研究；

2、生态补偿制度和生物物种资源保护经济激励政策研究；

3、生物物种资源保护与利用相关标准体系研究；

4、生物物种资源监测和预警机制研究；

5、国际机构、非政府组织、企业和社会各界利益相关方生物多样性保护伙伴关系建立和融资机制与政策研究。

优先行动八：国外生物物种及遗传资源的引进与开发利用

优先项目：

1、国外优良农作物种质资源的引进与开发利用；

2、国外优良经济林木、花卉植物、竹藤植物和药用生物资源的引进与开发利用；

3、国外优良种畜禽资源和其他优良动物种质资源的引进与开发利用；

4、国外经济水生生物资源的引进与开发利用；

5、国外优良经济微生物菌种的引进与利用；

6、国外生物物种资源产业化开发技术的引进；

7、引进物种风险评估与管理体系的建立。

优先行动九：建立生物物种资源出入境控制查验体系

优先项目：

1、建立生物物种资源输出风险评估、许可制度以及出入境查验法律法规，制定各类保护物种名录，明确出入境查验对象和查验要求；

2、口岸查验设施的配置，以及生物物种资源出入境检验鉴定实验室建设；

3、生物物种资源远程鉴定技术研究和外来物种快速鉴定及监测技术研究；

4、微生物分离培养、快速检测鉴定技术研究、远程鉴定网络建设和以及微生物环境安全评价体系研究。

优先行动十：宣传教育与培训

优先项目：

1、主流媒体宣传教育材料制作，以及学校及公众宣传教育教材编制；

2、基层生物物种保护机构宣传与教育设施建设；

3、各类培训计划的实施与培训设施建设；

4、非政府组织在生物多样性公众教育与提高公众参与意识方面的行动计划与实施。

六、保障措施

（一）完善管理体系与协调机制

进一步发挥生物物种资源保护部际联席会议制度在《规划纲要》实施过程中的作用，明确各成员单位的职责，强调部门之间的支持与合作，统一部署，分工负责，协调步伐，一致行动。加强地方政府和基层机构能力建设，建立多形式、多层次的监督机制和监督机构。

（二）加强相关法律制度建设

抓紧起草和完善生物物种资源保护法律法规，规范生物物种资源的采集、收集、保护、保存、研究、开发、交换、贸易等活动。建立生物物种、遗传资源及相关传统知识的获取与惠益分享的制度。建立生物物种资源出入境查验制度。

（三）加大执法力度。

明确职责，强化责任，严格执法，加强对有关单位持有、对外交换和提供生物物种资源情况的监督检查。加强执法队伍建设，提高执法能力，坚决打击偷采盗猎、非法经营、倒卖走私生物物种资源的行为。

（四）完善经济政策与市场监督体系

建立适合市场经济的生物物种资源保护与持续利用政策体系，引导对生物物种资源进行有效保护和合理开发利用，以解决保护与开发的矛盾，实现生物物种资源的持久保护和可持续利用双赢。建立市场监督体系，规范市场行为，引导生物物种资源的可持续开发利用。对列入国家保护名录和国际公约保护名录的动植物种的贸易实行严格的市场管理。

（五）加大资金投入

多渠道筹集资金，建立稳定的投入机制。中央和地方政府要随着国家财力的增强，不断加大对生物物种资源保护的资金投入，尤其要重视基础能力建设的投入。各级人民政府要将生物物种资源保护纳入国民经济和社会发展规划，所需经费纳入同级财政预算。鼓励单位和个人参与生物物种资源保护与可持续利用。同时，更多地争取国际资助。

（六）强化宣传教育

突出宣传国家相关法律法规，重点提高科研人员、出境人员和直接从事物种资源采集和开发活动的基层群众的遵法和守法意识，增强保护与持续利用生物物种资源的自觉性。充分发挥主流媒体在宣传生物物种资源保护方面的作用。加强基层机构的宣传教育设施建设，建立基层宣传教育专业队伍。加强青少年教育，在中、小学教材中增加生物物种资源保护的内容，培训青年学生志愿者宣传队伍，加强对基层群众的宣传教育。

（七）加强科学研究

重点开发保护与持续利用生物物种资源的各类技术，加强部门、机构和项目间的信息沟通和协调，避免重复和资源浪费。积极推广应用成熟的研究成果和技术，促进科学研究成果的交流和社会共享。

（八）提高人力资源能力保障水平

提高政府部门决策层人员素质，培养和充实大量优秀的基层管理人才。通过各种机制，培养大量科学技术人才，特别是生物分类学、生态学、生物技术、保护生物学等方面的专家，以及相关专业技术人才。

（九）探索和建立公众参与机制

建立并逐步完善动员、引导、支持公众参与生物物种资源保护的有效机制，实行群众举报投诉、信访制度、听证制度、新闻舆论监督制度和公民监督参与制度等。建立利益相关方共同参与的生物多样性伙伴关系，调动社会各方力量，以多种方式参与生物多样性保护。

本篇引用的法规

中央法规

国务院办公厅关于加强生物物种资源保护和管理的通知

中华人民共和国野生植物保护条例

野生药材资源保护管理条例

引用本篇的法规 案例 论文

部门规章

环境保护部关于印发《中国生物多样性保护战略与行动计划》(2011 - 2030年)的通知

地方法规规章

上海市环境保护局、上海市绿化和市容管理局、上海市农业委员会等关于印发《上海市生物多样性保护战略与行动计划(2012-2030)》的通知

湖南省环境保护厅关于印发《湖南省实施＜国家环境保护“十一五”规划＞中期评估工作方案》的通知

法学期刊

遗传资源专门保护制度的范式形成与逻辑进路

CAFTA区域生物遗传资源多边惠益分享机制研究

疫情下野生动物的法律界定：起点、难点及边线

我国农业文化遗产保护的立法构想

关于生物基因专利及其利益分享制度的法律分析

构建我国遗传资源获取与惠益分享制度的思考

生物多样性适应气候变化法律问题初探

法律视野中的全球重要农业文化遗产

更多

*注：本文格式遵循《全国人大法规备案审查信息平台电子文件格式规范（试行）》标准。

©北大法宝：（www.pkulaw.com）专业提供法律信息、法学知识和法律软件领域各类解决方案。北大法宝为您提供丰富的参考资料，正式引用法规条文时请与标准文本核对。

欢迎查看所有产品和服务。

法宝快讯： 如何快速找到您需要的检索结果？ 法宝V6有何新特色？

扫描二维码阅读原文

原文链接：https://www.pkulaw.com/chl/6314ef96413d5c4ebdfb.html
