# Supplementary material for: China’s Legal Protection System for Pangolins: Past, Present, and Future
Source: Animals (Basel). 2025 Aug 18;15(16):2422. doi: 10.3390/ani15162422 (PMC12383201; doi:10.3390/ani15162422)
Supplement: Supplementary file 1 [file animals-15-02422-s001.zip › Supplementary Material S2 -Full Texts of Laws and Regulations Related to Pangolins in China/【17】西藏自治区人民政府办公厅关于加强赛加羚羊、穿山甲、稀有蛇类资源保护和规范其产品入药管理的通知(FBM-CLI.12.pdf]

西藏自治区人民政府办公厅关于加强赛加羚羊、穿山甲、稀有蛇类资源保护和规范其产品入药管理的通知

制定机关： 西藏自治区人民政府

发文字号：藏政办发〔2008〕37号

公布日期：2008.04.14

施行日期：2008.04.14

时效性： 现行有效

效力位阶： 地方规范性文件

法规类别： 农业管理

西藏自治区人民政府办公厅关于加强赛加羚羊、穿山甲、稀有蛇类资源保护和规范其产品入药管理的通知

（藏政办发[2008]37号　二〇〇八年四月十四日）

各行署、拉萨市人民政府，各县（市、区）人民政府，自治区各委、办、厅、局：

为贯彻国家林业局、卫生部、工商总局等五部委《关于加强赛加羚羊、穿山甲、稀有蛇类资源保护和规范其产品入药管理的通知》（林护发[2007]242号）精神，正确处理好赛加羚羊、穿山甲、稀有蛇类（指国家保护的或《濒危野生动植物物种国际贸易公约》附录所列的蛇类，下同）等生态、经济、科研价值极高的陆生野生动物资源保护与可持续利用的关系，促进野生动物保护和中（藏）医药事业的协调发展。现结合我区实际，提出如下贯彻意见：

一、提高认识，相互配合，确保各项管理措施顺利实施

加强赛加羚羊、穿山甲、稀有蛇类保护管理，规范其产品生产流通，是根据资源现状，为维护中（藏）医药可持续发展的长远利益而采取的综合管理措施，自治区各级政府及林业、卫生、工商、食品药品监督管理等部门要予以高度重视，加强沟通和配合，制定协调机制，认真执行各项管理措施。

二、停止野外猎捕活动，促进野外资源恢复与增长

自本通知下发之日起，各级林业主管部门要立即停止核发赛加羚羊、穿山甲和稀有蛇类特许猎捕证或狩猎证。因科学研究、驯养繁殖或保障人身安全等特殊原因，由自治区林业局核实其目的和资源状况，按国家规定批准猎捕的赛加羚羊、穿山甲和稀有蛇类，不得用于经营活动。

三、建立激励机制。引导企业参与野外资源恢复和人工繁殖活动

按照“谁投入，谁受益”的激励机制，根据我区实际情况，鼓励资源利用企业在上述物种的分布区域积极参与赛加羚羊、穿山甲和稀有蛇类野外种群恢复和人工繁育活动。对驯养繁殖技术研究取得阶段性成果的，由自治区林业局组织专家论证，取得科学论证后向国家林业局申报开展试点予以推广，在加工利用、出售繁殖所获得的上述物种原材料或产品方面，争取国家林业局的扶持。

四、核实原料库存情况。进行登记造册、标准化封装和定点保管

2008年4月15日至5月31日，各地（市）林业、卫生、工商、食品药品监管等部门组织联合工作组，对各地（市）、县管辖区域内的市场（包括市场药点和土特产品市场）、药品生产企业、医药公司、以及医疗机构等有关单位开展专项清理工

作，对库存的赛加羚羊角、穿山甲片和稀有蛇类原料以及包含上述原料的药品进行全面清理登记。对各单位保管点、各药点名称、责任人、数量及封装编号进行逐一登记造册。6月15日前各地（市）将清理登记汇总情况分别上报自治区林业局，6月25日前由自治区林业局汇总后上报国家林业局。从清理登记之日起到实行标识制度之前不得经营销售上述原料和产品。

五、明确原材料使用范围，宏观控制资源消耗总量

为确保对资源消耗总量的宏观控制，今后所有赛加羚羊、穿山甲原材料仅限于定点医院临床使用和中成药（含藏药，下同）生产，不得在定点医院和药品生产企业外以零售方式公开出售；稀有蛇类原材料除重点保障医院临床使用和中成药生产外，可适量用于其他重点产品的生产和利用。

定点医疗机构由自治区卫生厅确定并于2008年5月31日前上报卫生部、国家中医药管理局批准。非定点医院根据国家五部委的通知精神，自2008年6月1日起一律停止临床使用上述原材料。取得国家药品监督管理部门相应药品生产批准文号的企业，才有资格申报在中成药生产中利用赛加羚羊角、穿山甲片和稀有蛇类原材料。

六、严格原材料购销及利用管理。规范流通秩序

为防止非法来源的赛加羚羊、穿山甲、稀有蛇类等原材料混入合法流通渠道，核实后标准化封装、登记在册的上述原材料，只能销售给中成药生产企业、定点医院和含稀有蛇类成份产品的生产企业，而且只能用于生产经批准的中成药产品和定点医院临床中。上述企业、定点医院需要购买或利用库存原材料从事相关生产活动或临床使用时，应说明原材料来源、投料生产和使用计划，自治区林业局依

法实施上述行政许可事项。

按照国家关于“未依法获得行政许可的，不得利用赛加羚羊角、穿山甲片和稀有蛇类原材料从事生产经营活动，未加载专用标识的产品也不得进入流通领域”的规定，对已经生产的含有赛加羚羊角、穿山甲片和稀有蛇类成份的库存产品，各生产、经营单位要向各地（市）林业、工商、药检等部门报告，经林业部门行政许可后，一次性安排专用标识。加载专用标识后的上述产品可继续流通，直至销售完毕。对含有赛加羚羊角、穿山甲片和稀有蛇类成份，但未加载专用标识的产品，2008年5月31日后禁止上市流通。由林业部门牵头，会同卫生、工商、食品药品等部门适时开展市场专项执法检查，整顿和规范流通环节经营秩序。

七、统一实行专用标识制度

根据本通知精神，需要申请专用标识的相关企业请于2008年5月31日前向各地（市）林业部门提出申请，由自治区林业局审核后向国家林业局申报。

附件：

稀有蛇类物种名录

物种名称	拉丁学名蟒
蟒	Python molums
温泉蛇	Thennophis baileyi
乌梢蛇	Zaocys dhumnades
银环蛇	Eungarus multicinctus
眼睛王蛇	Ophiophagus hannah

细脆蛇蜥	Pphisautus gracilis

引用本篇的法规 案例 论文

法学期刊

野生动物利用法律制度的嬗变与破局

*注：本文格式遵循《全国人大法规备案审查信息平台电子文件格式规范（试行）》标准。

©北大法宝：（www.pkulaw.com）专业提供法律信息、法学知识和法律软件领域各类解决方案。北大法宝为您提供丰富的参考资料，正式引用法规条文时请与标准文本核对。

欢迎查看所有产品和服务。

法宝快讯： 如何快速找到您需要的检索结果？ 法宝 V6 有何新特色？

扫描二维码阅读原文

原文链接：https://www.pkulaw.com/lar/429200ff5e475fc7cbe5a97df02fe9b4bdfb.html
