# Supplementary material for: China’s Legal Protection System for Pangolins: Past, Present, and Future
Source: Animals (Basel). 2025 Aug 18;15(16):2422. doi: 10.3390/ani15162422 (PMC12383201; doi:10.3390/ani15162422)
Supplement: Supplementary file 1 [file animals-15-02422-s001.zip › Supplementary Material S2 -Full Texts of Laws and Regulations Related to Pangolins in China/【18】国家林业局公告2015年第8号――关于中国野生动物经营利用管理专用标识有关事项的公告(FBM-CLI.4.pdf]

国家林业局公告2015年第8号—
关于中国野生动物经营利用管理专用标识有关事项的公告

制定机关： 国家林业局(已撤销) 机构沿革
发文字号：国家林业局公告2015年第8号
公布日期：2015.04.28
施行日期：2015.04.28
时效性： 现行有效
效力位阶： 部门规范性文件
法规类别： 野生动植物资源

国家林业局公告

（2015年第8号）

根据《国家林业局

国家工商行政管理总局关于对利用野生动物及其产品的生产企业进行清理整顿和开展标记试点工作的通知》（林护发〔2003〕3号）和《国家林业局 卫生部

国家工商行政管理总局 国家食品药品监督管理局

国家中医药管理局关于进一步加强麝、熊资源保护及其产品入药管理的通知》（林护发〔2004〕252号）、《国家林业局 卫生部 国家工商行政管理总局

国家食品药品监督管理局

国家中医药管理局关于进一步加强赛加羚羊、穿山甲、稀有蛇类资源保护和规范其产品入药管理的通知》（林护发〔2007〕242号）有关规定，现对试点使用“中国野生动物经营利用管理专用标识”的企业及其产品（见附件1-

3）、允许临床使用赛加羚羊角、穿山甲片、稀有蛇类原材料的定点医院（见附件4）以及2015版“中国野生动物经营利用管理专用标识”（见附件5）予以公告。

特此公告。

附件：1. 生产、销售含天然麝香成分中成药的企业及其产品名称

2. 生产象皮粉的企业名称

3. 销售野生动物相关制品的企业及其产品名称

4. 允许临床使用赛加羚羊、穿山甲、稀有蛇类原材料的定点医院

5. 2015版“中国野生动物经营利用管理专用标识”

国家林业局

2015年4月28日

附件1

生产、销售含天然麝香成分中成药的企业及其产品名称

序号	省份	企业名称	
1	四川	九寨沟天然药业集团有限责任公司	小金丸、五味麝香丸、万应锭、西黄丸、牛

附件2

生产象皮粉的企业名称

序号	省份	企业名称	产品名称
1	广东	广州中量药业有限公司	象皮粉

附件3

销售野生动物相关制品的企业及其产品名称

序号	省份	企业名称	产品名称
1	北京	北京同仁堂健康药业股份有限公司	梅花鹿（鹿茸）、中国林蛙产品（蛤蟆油）
2		北京华邈中药工程技术开发中心	梅花鹿产品
3	上海	上海冠商国际贸易有限公司	野生动物皮制品
4		瑷利珈（上海）企业管理有限公司	野生动物皮制品
5		上海都德纺织科技有限公司	野生动物皮制品
6		爱马仕（上海）贸易有限公司	野生动物皮制品
7		尊湛（上海）商贸有限公司	野生动物皮制品
8		睿锦尚品国际贸易（上海）有限公司	野生动物皮制品
9		普拉达时装商业（上海）有限公司	野生动物皮制品
10	福建	北京同仁堂健康药业（福州）有限公司	梅花鹿产品、蛤蟆油（林蛙输卵管）、大壁虎

11	甘肃	白银市天胜农业综合开发有限公司	梅花鹿产品
12		甘肃科源农业发展有限公司	梅花鹿产品
13	宁夏	银川杰林养殖有限公司	马鹿、梅花鹿产品

附件4

允许临床使用赛加羚羊角、穿山甲片、稀有蛇类原材料的定点医院

序号	省份	医院名称	备注
1	吉林	吉林中医肝胆医院	
2		四平市传染病医院	

附件5

2015版“中国野生动物经营利用管理专用标识”

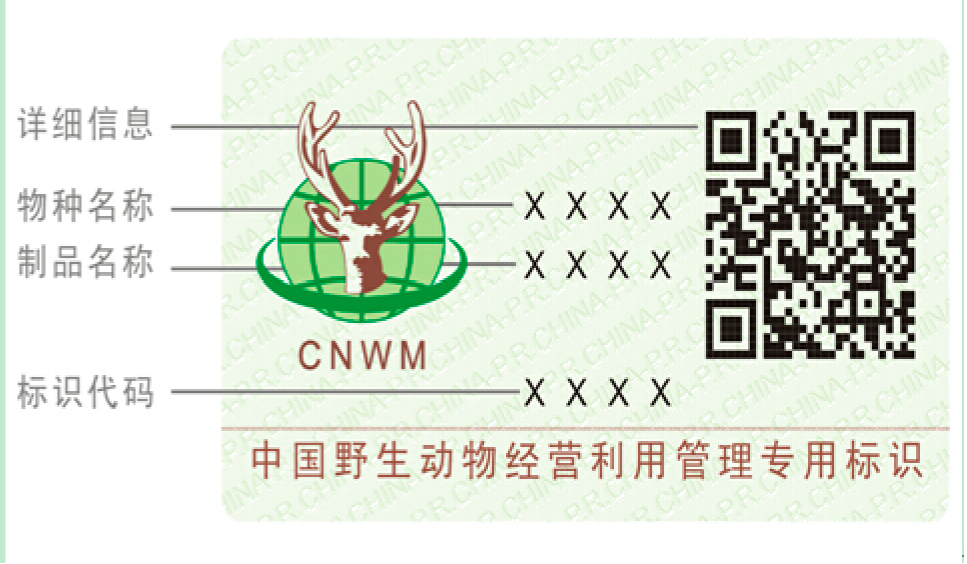

此标识自2015年6月1日起启用。

本篇引用的法规

中央法规

国家林业局、卫生部、国家工商总局、食品药品监管局、国家中医药管理局关于进一步加强麝、熊资源保护及其产品入药管理的通知

国家林业局、国家工商总局关于对利用野生动物及其产品的生产企业进行清理整顿和开展标记试点工作的通知

*注：本文格式遵循《全国人大法规备案审查信息平台电子文件格式规范（试行）》标准。

©北大法宝：（www.pkulaw.com）专业提供法律信息、法学知识和法律软件领域各类解决方案。北大法宝为您提供丰富的参考资料，正式引用法规条文时请与标准文本核对。

欢迎查看所有产品和服务。

法宝快讯： 如何快速找到您需要的检索结果？ 法宝 V6 有何新特色？

扫描二维码阅读原文

原文链接：https://www.pkulaw.com/chl/dedb73b0d6b7c9dbbdfb.html
