# Supplementary material for: China’s Legal Protection System for Pangolins: Past, Present, and Future
Source: Animals (Basel). 2025 Aug 18;15(16):2422. doi: 10.3390/ani15162422 (PMC12383201; doi:10.3390/ani15162422)
Supplement: Supplementary file 1 [file animals-15-02422-s001.zip › Supplementary Material S2 -Full Texts of Laws and Regulations Related to Pangolins in China/【19】国家食品药品监督管理总局公告2015年第67号――关于发布《中华人民共和国药典》(2015年版)的公告(FBM-CLI.4.pdf]

国家食品药品监督管理总局公告2015年第67号——关于发布《中华人民共和国药典》(2015年版)的公告

制定机关： 国家食品药品监督管理总局(已撤销) 机构沿革

发文字号：国家食品药品监督管理总局公告2015年第67号

公布日期：2015.06.05

施行日期：2015.12.01

时效性： 现行有效

效力位阶： 部门工作文件

法规类别： 药品管理

国家食品药品监督管理总局公告

（2015年第67号）

关于发布《中华人民共和国药典》（2015年版）的公告

根据《中华人民共和国药品管理法》，《中华人民共和国药典》（以下简称《中国药典》，2015年版）经第十届药典委员会执委会全体会议审议通过，现予发布，自2015年12月1日起实施。《中国药典》（2015年版）目录见附件。

特此公告。

附件：《中国药典》（2015年版）目录

食品药品监管总局

2015年6月5日

附件

《中国药典》2015年版目录

一部

药材和饮片

1　一枝黄花

2　丁公藤

3　丁香

4　九里香

5　九香虫

6　人工牛黄

7　人参

8　人参叶

9　儿茶

10　八角茴香

11　刀豆

12　三七

13　三白草

14　三棱

15　三颗针

16 千年健

17 千里光

18 千金子

19 千金子霜

20 土木香

21 土贝母

22 土茯苓

23 土荆皮

24 土鳖虫（虫）

25 大叶紫珠

26 大血藤

27 大皂角

28 大豆黄卷

29 大枣

30 大青叶

31 大青盐

32 大黄

33 大腹皮

34 大蒜

35 大蓟

36 大蓟炭

37 女贞子

38 小叶莲

39 小驳骨

40 小茴香

41 小通草

42 小蓟

43 山豆根

44 山麦冬

45 山柰

46 山茱萸

47 山药

48 山香圆叶

49 山银花

50 山慈菇

51 山楂

52 山楂叶

53 川乌

54 川木香

55 川木通

56 川牛膝

57 川贝母

58 川芎

59 川射干

60 川楝子

61 干姜

62 干漆

63 广东紫珠

64 广枣

65 广金钱草

66 广藿香

67 飞扬草

68 马齿苋

69 马勃

70 马钱子

71 马钱子粉

72 马兜铃

73 马鞭草

74 丹参

75 乌药

76 乌梢蛇

77 乌梅

78 云芝

79 五加皮

80 五味子

81 五倍子

82 化橘红

83 升麻

84 天山雪莲

85　天仙子

86　天仙藤

87　天冬

88　天花粉

89　天竺黄

90　天南星

91　天麻

92　天然冰片（右旋龙脑）

93　天葵子

94　太子参

95　巴豆

96　巴豆霜

97　巴戟天

98　月季花

99　木瓜

100　木芙蓉叶

101　木香

102　木贼

103　木通

104　木棉花

105　木蝴蝶

106　木鳖子

107　毛诃子

108　水飞蓟

109　水牛角

110　水红花子

111　水蛭

112　火麻仁

113　片姜黄

114　牛黄

115　牛蒡子

116　牛膝

117　王不留行

118　瓦松

119　瓦楞子

120　车前子

121　车前草

122　丝瓜络

123　仙茅

124　仙鹤草

125　冬瓜皮

126　冬虫夏草

127　冬凌草

128　冬葵果

129　功劳木

130　北刘寄奴

131　北沙参

132　北豆根

133　半边莲

134　半枝莲

135　半夏

136　四季青

137　布渣叶

138　平贝母

139　母丁香

140　玄参

141　玄明粉

142　玉竹

143　瓜子金

144　瓜蒌

145　瓜蒌子

146　瓜蒌皮

147　甘松

148　甘草

149　甘遂

150　生姜

151　白及

152　白头翁

153　白术

154　白芍

155　白芷

156　白附子

157　白屈菜

158　白果

159　白矾

160　白茅根

161　白前

162　白扁豆

163　白蔹

164　白鲜皮

165　白薇

166　石韦

167　石决明

168　石吊兰

169　石斛

170　石菖蒲

171　石榴皮

172　石膏

173　艾片（左旋龙脑）

174　艾叶

175　龙胆

176　龙眼肉

177　龙脷叶

178　亚乎奴（锡生藤）

179　亚麻子

180　伊贝母

181　全蝎

182　关黄柏

183　冰片（合成龙脑）

184　决明子

185　华山参

186　合欢皮

187　合欢花

188　地龙

189　地枫皮

190　地肤子

191　地骨皮

192　地黄

193　地榆

194　地锦草

195　安息香

196　延胡索（元胡）

197　当归

198　当药

199　朱砂

200　朱砂根

201　灯心草

202　灯盏细辛（灯盏花）

203　百合

204　百部

205　竹节参

206　竹茹

207　红大戟

208　红芪

209　红花

210　红花龙胆

211　红豆蔻

212　红参

213　红粉

214　红景天

215　老鹳草

216　肉苁蓉

217　肉豆蔻

218　肉桂

219　自然铜

220　芒硝

221　虫白蜡

222　血余炭

223 血竭

224 西瓜霜

225 西红花

226 西河柳

227 西青果

228 西洋参

229 防己

230 防风

231 两头尖

232 两面针

233 伸筋草

234 体外培育牛黄

235 何首乌

236 余甘子

237 佛手

238 吴茱萸

239 忍冬藤

240 杜仲

241 杜仲叶

242 杠板归

243 沉香

244 沙苑子

245 沙棘

246　没药

247　灵芝

248　牡丹皮

249　牡荆叶

250　牡蛎

251　皂角刺

252　皂矾（绿矾）

253　羌活

254　芡实

255　芥子

256　芦荟

257　芦根

258　芫花

259　花椒

260　花蕊石

261　苍术

262　苍耳子

263　苏木

264　苏合香

265　补骨脂

266　诃子

267　谷芽

268　谷精草

269　豆蔻

270　赤小豆

271　赤石脂

272　赤芍

273　辛夷

274　远志

275　连钱草

276　连翘

277　阿胶

278　阿魏

279　附子

280　陈皮

281　鸡内金

282　鸡血藤

283　鸡冠花

284　鸡骨草

285　麦冬

286　麦芽

287　龟甲

288　龟甲胶

289　乳香

290　京大戟

291　佩兰

292 使君子

293 侧柏叶

294 制川乌

295 制天南星

296 制何首乌

297 制草乌

298 刺五加

299 卷柏

300 垂盆草

301 委陵菜

302 岩白菜

303 巫山淫羊藿

304 昆布

305 明党参

306 松花粉

307 板蓝根

308 枇杷叶

309 枫香脂

310 油松节

311 法半夏

312 泽兰

313 泽泻

314 炉甘石

315 炒瓜蒌子

316 炙甘草

317 炙红芪

318 炙黄芪

319 狗脊

320 玫瑰花

321 知母

322 细辛

323 罗布麻叶

324 罗汉果

325 肿节风

326 苘麻子

327 苦木

328 苦玄参

329 苦地丁

330 苦杏仁

331 苦参

332 苦楝皮

333 虎杖

334 贯叶金丝桃

335 郁李仁

336 郁金

337 金龙胆草

338　金果榄

339　金沸草

340　金荞麦

341　金钱白花蛇

342　金钱草

343　金铁锁

344　金银花

345　金樱子

346　金礞石

347　闹羊花

348　降香

349　青风藤

350　青叶胆

351　青皮

352　青果

353　青葙子

354　青蒿

355　青黛

356　青礞石

357　鱼腥草

358　前胡

359　南五味子

360　南沙参

361　南板蓝根

362　南鹤虱

363　厚朴

364　厚朴花

365　哈蟆油

366　姜半夏

367　姜黄

368　威灵仙

369　急性子

370　枳壳

371　枳实

372　枸杞子

373　枸骨叶

374　柏子仁

375　柿蒂

376　栀子

377　洋金花

378　洪连

379　炮姜

380　牵牛子

381　独一味

382　独活

383　珍珠

384　珍珠母

385　砂仁

386　禹州漏芦

387　禹余粮

388　穿山甲

389　穿山龙

390　穿心莲

391　络石藤

392　胆南星

393　胖大海

394　胡芦巴

395　胡黄连

396　胡椒

397　茜草

398　茯苓

399　茯苓皮

400　茵陈

401　茺蔚子

402　荆芥

403　荆芥炭

404　荆芥穗

405　荆芥穗炭

406　草乌

407 草乌叶

408 草豆蔻

409 草果

410 荔枝核

411 荜茇

412 荜澄茄

413 轻粉

414 重楼

415 钟乳石

416 钩藤

417 韭菜子

418 首乌藤

419 香加皮

420 香附

421 香橼

422 香薷

423 骨碎补

424 鸦胆子

425 党参

426 凌霄花

427 夏天无

428 夏枯草

429 娑罗子

430 射干

431 徐长卿

432 拳参

433 柴胡

434 核桃仁

435 桂枝

436 桃仁

437 桃枝

438 桑叶

439 桑白皮

440 桑枝

441 桑寄生

442 桑椹

443 桑螵蛸

444 桔梗

445 浙贝母

446 浮萍

447 海马

448 海风藤

449 海龙

450 海金沙

451 海螵蛸

452 海藻

453 狼毒

454 珠子参

455 益母草

456 益智

457 秦皮

458 秦艽

459 积雪草

460 粉萆薢

461 粉葛

462 臭灵丹草

463 荷叶

464 莪术

465 莱菔子

466 莲子

467 莲子心

468 莲房

469 莲须

470 通关藤

471 通草

472 铁皮石斛

473 预知子

474 高山辣根菜

475 高良姜

476　鸭跖草

477　商陆

478　密蒙花

479　常山

480　救必应

481　断血流

482　旋覆花

483　梅花

484　淡竹叶

485　淡豆豉

486　淫羊藿

487　清半夏

488　猪牙皂

489　猪苓

490　猪胆粉

491　猫爪草

492　甜瓜子

493　续断

494　绵马贯众

495　绵马贯众炭

496　绵萆薢

497　羚羊角

498　菊花

499　菊苣

500　蒺藜

501　菟丝子

502　菥蓂

503　蛇床子

504　蛇蜕

505　野马追

506　野木瓜

507　野菊花

508　银杏叶

509　银柴胡

510　鹿角

511　鹿角胶

512　鹿角霜

513　鹿茸

514　鹿衔草

515　麻黄

516　麻黄根

517　黄山药

518　黄芩

519　黄芪

520　黄连

521　黄柏

522　黄精

523　黄蜀葵花

524　黄藤

525　斑蝥

526　棕榈

527　楮实子

528　款冬花

529　湖北贝母

530　滑石

531　滑石粉

532　焦栀子

533　焦槟榔

534　番泻叶

535　硫黄

536　筋骨草

537　紫石英

538　紫花地丁

539　紫花前胡

540　紫苏子

541　紫苏叶

542　紫苏梗

543　紫草

544　紫珠叶

545　紫菀

546　紫萁贯众

547　萹蓄

548　葛根

549　葶苈子

550　蛤壳

551　蛤蚧

552　锁阳

553　雄黄

554　鹅不食草

555　黑芝麻

556　黑豆

557　黑种草子

558　椿皮

559　槐花

560　槐角

561　滇鸡血藤

562　满山红

563　煅石膏

564　矮地茶

565　蒲公英

566　蒲黄

567　蒺藜

568　蓍草

569　蓖麻子

570　蓝布正

571　蜂房

572　蜂胶

573　蜂蜜

574　蜂蜡

575　蜈蚣

576　路路通

577　锦灯笼

578　雷丸

579　榧子

580　榼藤子

581　槟榔

582　漏芦

583　磁石

584　罂粟壳

585　蓼大青叶

586　蔓荆子

587　蜘蛛香

588　蝉蜕

589　豨莶草

590　辣椒

591　酸枣仁

592　僵蚕

593　墨旱莲

594　暴马子皮

595　槲寄生

596　熟地黄

597　稻芽

598　蕤仁

599　蕲蛇

600　赭石

601　鹤虱

602　橘红

603　橘核

604　薄荷

605　薏苡仁

606　薤白

607　颠茄草

608　檀香

609　翼首草

610　藁本

611　藏菖蒲

612　瞿麦

613　翻白草

614　藕节

615　覆盆子

616　蟾酥

617　鳖甲

618　麝香

植物油脂和提取物

1　丁香罗勒油

2　八角茴香油

3　人参茎叶总皂苷

4　人参总皂苷

5　三七三醇皂苷

6　三七总皂苷

7　大黄流浸膏

8　大黄浸膏

9　山楂叶提取物

10　广藿香油

11　丹参总酚酸提取物

12　丹参酮提取物

13　水牛角浓缩粉

14　甘草流浸膏

15　甘草浸膏

16　北豆根提取物

17　当归流浸膏

18　肉桂油

19　灯盏花素

20　远志流浸膏

21　连翘提取物

22　牡荆油

23　环维黄杨星D

24　松节油

25　刺五加浸膏

26　岩白菜素

27　肿节风浸膏

28　茵陈提取物

29　茶油

30　香果脂

31　姜流浸膏

32　穿心莲内酯

33　莪术油

34　桉油

35　积雪草总苷

36　益母草流浸膏

37　浙贝流浸膏

38　黄芩提取物

39　黄藤素

40　银杏叶提取物

41　麻油

42　蓖麻油

43　满山红油

44　薄荷素油

45　薄荷脑

46　颠茄流浸膏

47　颠茄浸膏

成方制剂和单味制剂

1　一捻金

2　一捻金胶囊

3　一清胶囊

4　一清颗粒

5　乙肝宁颗粒

6　乙肝养阴活血颗粒

7　乙肝益气解郁颗粒

8　七十味珍珠丸

9　七叶神安片

10　七制香附丸

11　七味广枣丸

12　七味都气丸

13　七味铁屑丸

14　七味葡萄散

15　七味榼藤子丸

16　七宝美髯颗粒

17　七厘胶囊

18　七厘散

19　七珍丸

20　九一散

21　九分散

22　九气拈痛丸

23　九圣散

24　九制大黄丸

25　九味石灰华散

26　九味羌活丸

27　九味羌活口服液

28　九味羌活颗粒

29　九味肝泰胶囊

30　九香止痛丸（七香止痛丸）

31　二丁颗粒

32　二十七味定坤丸

33　二十五味松石丸

34　二十五味珊瑚丸

35　二十五味珍珠丸

36　二冬膏

37　二母宁嗽丸

38　二母安嗽丸

39 二至丸

40 二妙丸

41 二陈丸

42 人参再造丸

43 人参养荣丸

44 人参首乌胶囊

45 人参健脾丸

46 儿宝颗粒

47 儿康宁糖浆

48 儿童清肺丸

49 儿童清热导滞丸

50 儿感退热宁口服液

51 八正合剂

52 八味沉香散

53 八味清心沉香散

54 八味檀香散

55 八宝坤顺丸

56 八珍丸

57 八珍益母丸

58 八珍益母胶囊

59 八珍颗粒

60 十一味参芪片

61 十一味参芪胶囊

62　十一味能消丸

63　十二味翼首散

64　十三味榜嘎散

65　十五味沉香丸

66　十六味冬青丸

67　十全大补丸

68　十味消渴胶囊（参芪消渴胶囊）

69　十香止痛丸

70　十香返生丸

71　十滴水

72　十滴水软胶囊

73　万氏牛黄清心丸

74　万应胶囊

75　万应锭

76　万通炎康片

77　三七片

78　三七伤药片

79　三七伤药胶囊

80　三七伤药颗粒

81　三七血伤宁胶囊

82　三七通舒胶囊

83　三九胃泰胶囊

84　三九胃泰颗粒

85　三子散

86　三两半药酒

87　三妙丸

88　三味蒺藜散

89　三宝胶囊

90　三拗片

91　三金片

92　三黄片

93　千金止带丸（大蜜丸）

94　千金止带丸（水丸）

95　千柏鼻炎片

96　千柏鼻炎胶囊

97　千喜片

98　千喜胶囊

99　口炎清颗粒

100　口咽清丸（阮氏上清丸）

101　口腔溃疡散

102　大七厘散

103　大山楂丸

104　大川芎口服液

105　大补阴丸

106　大黄清胃丸

107　大黄?虫丸

108 女金丸

109 女金胶囊

110 女珍颗粒

111 小儿七星茶口服液

112 小儿七星茶颗粒

113 小儿化毒散

114 小儿化食丸

115 小儿化食口服液

116 小儿止咳糖浆

117 小儿止嗽糖浆

118 小儿百寿丸

119 小儿百部止咳糖浆

120 小儿至宝丸

121 小儿抗痫胶囊

122 小儿肝炎颗粒

123 小儿宝泰康颗粒

124 小儿泻速停颗粒

125 小儿泻痢片

126 小儿肺咳颗粒

127 小儿肺热平胶囊

128 小儿肺热咳喘口服液

129 小儿金丹片

130 小儿咳喘颗粒

131　小儿咽扁颗粒

132　小儿退热合剂（小儿退热口服液）

133　小儿退热颗粒

134　小儿香橘丸

135　小儿柴桂退热口服液

136　小儿柴桂退热颗粒

137　小儿消食片

138　小儿消积止咳口服液

139　小儿热速清口服液

140　小儿热速清颗粒

141　小儿热速清糖浆

142　小儿惊风散

143　小儿清肺化痰口服液

144　小儿清肺止咳片

145　小儿清热止咳合剂（小儿清热止咳口服液）

146　小儿清热片

147　小儿豉翘清热颗粒

148　小儿感冒口服液

149　小儿感冒宁糖浆

150　小儿感冒茶

151　小儿感冒颗粒

152　小儿腹泻宁糖浆

153　小儿解表颗粒

154　小儿解热丸

155　小儿解感片

156　小儿敷脐止泻散

157　小建中片

158　小建中合剂

159　小建中颗粒

160　小金丸

161　小金片

162　小金胶囊

163　小青龙合剂

164　小青龙颗粒

165　小活络丸

166　小柴胡片

167　小柴胡泡腾片

168　小柴胡胶囊

169　小柴胡颗粒

170　山东阿胶膏

171　山玫胶囊

172　山香圆片

173　山绿茶降压片

174　山菊降压片

175　山楂化滞丸

176　川贝止咳露（川贝枇杷露）

177 川贝枇杷糖浆

178 川贝雪梨膏

179 川芎茶调丸

180 川芎茶调丸（浓缩丸）

181 川芎茶调片

182 川芎茶调袋泡茶（川芎茶调袋泡剂）

183 川芎茶调散

184 川芎茶调颗粒

185 马应龙八宝眼膏

186 马应龙麝香痔疮膏

187 马钱子散

188 中风回春丸

189 中风回春片

190 中华跌打丸

191 丹七片

192 丹红化瘀口服液

193 丹参片

194 丹香清脂颗粒

195 丹桂香颗粒

196 丹益片

197 丹蒌片

198 丹膝颗粒

199 乌贝散

200 乌贝颗粒

201 乌军治胆片

202 乌灵胶囊

203 乌鸡白凤丸

204 乌鸡白凤片

205 乌鸡白凤颗粒

206 乌梅丸

207 乌蛇止痒丸

208 云南白药

209 云南白药胶囊

210 云香祛风止痛酊

211 五子衍宗丸

212 五子衍宗片

213 五味子颗粒

214 五味子糖浆

215 五味沙棘散

216 五味清浊散

217 五味麝香丸

218 五苓胶囊

219 五苓散

220 五虎散

221 五黄养阴颗粒

222 五福化毒丸

223　仁青芒觉

224　仁青常觉

225　元胡止痛口服液

226　元胡止痛片

227　元胡止痛软胶囊

228　元胡止痛胶囊

229　元胡止痛滴丸

230　元胡止痛颗粒

231　六一散

232　六合定中丸

233　六君子丸

234　六应丸

235　六味木香散

236　六味地黄丸

237　六味地黄丸（浓缩丸）

238　六味地黄软胶囊

239　六味地黄胶囊

240　六味地黄颗粒

241　六味安消胶囊

242　六味安消散

243　六味香连胶囊

244　内消瘰疬片

245　分清五淋丸

246　化积口服液

247　化痔栓

248　化瘀祛斑胶囊

249　化癥回生片

250　升气养元糖浆

251　升血颗粒

252　午时茶胶囊

253　午时茶颗粒

254　双丹口服液

255　双虎清肝颗粒

256　双黄连口服液

257　双黄连片

258　双黄连栓

259　双黄连胶囊

260　双黄连滴眼剂

261　双黄连颗粒

262　天丹通络片

263　天丹通络胶囊

264　天王补心丸

265　天王补心丸（浓缩丸）

266　天和追风膏

267　天菊脑安胶囊

268　天麻丸

269　天麻头痛片

270　天麻祛风补片

271　天麻钩藤颗粒

272　天麻首乌片

273　天麻醒脑胶囊

274　天智颗粒

275　天紫红女金胶囊

276　天舒片

277　天舒胶囊

278　少阳感冒颗粒

279　少林风湿跌打膏

280　少腹逐瘀丸

281　开光复明丸

282　开胃山楂丸

283　开胃健脾丸

284　开胸顺气丸

285　开胸顺气胶囊

286　心元胶囊

287　心可舒片

288　心宁片

289　心安宁片

290　心血宁片

291　心血宁胶囊

292　心荣口服液

293　心悦胶囊

294　心脑宁胶囊

295　心脑欣丸

296　心脑欣胶囊

297　心脑健片

298　心脑健胶囊

299　心脑康片

300　心脑康胶囊

301　心脑静片

302　心通口服液

303　心速宁胶囊

304　心舒宁片

305　心舒胶囊

306　无烟灸条

307　木瓜丸

308　木香分气丸

309　木香顺气丸

310　木香槟榔丸

311　止红肠辟丸

312　止血定痛片

313　止血复脉合剂

314　止咳宝片

315　止咳喘颗粒

316　止咳橘红丸

317　止咳橘红口服液

318　止喘灵注射液

319　止痛化癥片

320　止痛化癥胶囊

321　止痛紫金丸

322　止嗽化痰丸

323　止嗽定喘口服液

324　比拜克胶囊

325　气滞胃痛片

326　气滞胃痛颗粒

327　气痛丸

328　片仔癀

329　片仔癀胶囊

330　牙痛一粒丸

331　牛黄上清丸

332　牛黄上清片

333　牛黄上清软胶囊

334　牛黄上清胶囊

335　牛黄千金散

336　牛黄化毒片

337　牛黄至宝丸

338　牛黄净脑片

339　牛黄抱龙丸

340　牛黄降压丸

341　牛黄降压片

342　牛黄降压胶囊

343　牛黄消炎片

344　牛黄清心丸（局方）

345　牛黄清宫丸

346　牛黄清感胶囊

347　牛黄蛇胆川贝液

348　牛黄解毒丸

349　牛黄解毒片

350　牛黄解毒软胶囊

351　牛黄解毒胶囊

352　牛黄镇惊丸

353　贝羚胶囊

354　风热清口服液

355　风寒双离拐片

356　风寒咳嗽丸

357　风寒咳嗽颗粒

358　风湿马钱片

359　风湿定片

360　风湿骨痛胶囊

361　风痛安胶囊

362　乐儿康糖浆

363　乐脉丸

364　乐脉片

365　乐脉胶囊

366　乐脉颗粒

367　代温灸膏

368　冬凌草片

369　冯了性风湿跌打药酒

370　功劳去火片

371　加味左金丸

372　加味生化颗粒

373　加味香连丸

374　加味逍遥丸

375　加味逍遥口服液（合剂）

376　加味藿香正气软胶囊

377　北芪五加片

378　北豆根片

379　北豆根胶囊

380　半夏天麻丸

381　古汉养生精口服液

382　古汉养生精片

383　古汉养生精颗粒

384　右归丸

385　四方胃片

386　四正丸

387　四君子丸

388　四君子颗粒

389　四妙丸

390　四制香附丸

391　四味土木香散

392　四味珍层冰硼滴眼液

393　四物合剂

394　四物益母丸

395　四物颗粒

396　四神丸

397　四神片

398　四逆汤

399　外伤如意膏

400　外感风寒颗粒

401　孕康合剂（孕康口服液）

402　孕康颗粒

403　宁神补心片

404　左金丸

405　左金胶囊

406　平肝舒络丸

407　平消片

408　平消胶囊

409　归芍地黄丸

410　归脾丸

411　归脾丸（浓缩丸）

412　归脾合剂

413　归脾颗粒

414　戊己丸

415　正天丸

416　正天胶囊

417　正心降脂片

418　正心泰片

419　正心泰胶囊

420　正金油软膏

421　正骨水

422　正柴胡饮颗粒

423　正清风痛宁片

424　汉桃叶片

425　玄麦甘桔含片

426　玄麦甘桔胶囊

427　玄麦甘桔颗粒

428　玉屏风口服液

429　玉屏风胶囊

430 玉屏风袋泡茶

431 玉屏风颗粒

432 玉泉胶囊

433 玉泉颗粒

434 玉真散

435 瓜霜退热灵胶囊

436 甘桔冰梅片

437 甘露消毒丸

438 生发搽剂

439 生血宝合剂

440 生血宝颗粒

441 生脉饮

442 生脉胶囊

443 白带丸

444 白蚀丸

445 白蒲黄片

446 白癜风胶囊

447 石斛夜光丸

448 石淋通片

449 石榴健胃散

450 芄龙胶囊

451 艾附暖宫丸

452 龙牡壮骨颗粒

453　龙泽熊胆胶囊

454　龙胆泻肝丸

455　龙胆泻肝丸（水丸）

456　产复康颗粒

457　仲景胃灵丸

458　伤疖膏

459　伤科接骨片

460　伤湿止痛膏

461　伤痛宁片

462　全天麻胶囊

463　全杜仲胶囊

464　全鹿丸

465　关节止痛膏

466　再造丸

467　再造生血片

468　冰黄肤乐软膏

469　冰硼散

470　华山参片

471　华佗再造丸

472　地奥心血康胶囊

473　地榆槐角丸

474　壮骨关节丸

475　壮骨伸筋胶囊

476 如意定喘片

477 如意金黄散

478 妇乐颗粒

479 妇宁康片

480 妇必舒阴道泡腾片

481 妇良片

482 妇宝颗粒

483 妇炎净胶囊

484 妇炎康片

485 妇科十味片

486 妇科千金片

487 妇科千金胶囊

488 妇科分清丸

489 妇科止带片

490 妇科养坤丸

491 妇科调经片

492 妇科通经丸

493 妇康宁片

494 安儿宁颗粒

495 安中片

496 安阳精制膏

497 安宫止血颗粒

498 安宫牛黄丸

499 安宫牛黄散

500 安宫降压丸

501 安神补心丸

502 安神补心颗粒

503 安神补脑液

504 安神宝颗粒

505 安神胶囊

506 安胃片

507 安脑丸

508 导赤丸

509 庆余辟瘟丹

510 当飞利肝宁胶囊

511 当归龙荟丸

512 当归补血口服液

513 当归拈痛丸

514 当归养血丸

515 当归调经颗粒

516 朴沉化郁丸

517 灯台叶颗粒

518 灯盏生脉胶囊

519 灯盏花素片

520 灯盏细辛注射液

521 灯盏细辛颗粒（灯盏花颗粒）

522 百令胶囊

523 百合固金丸

524 百合固金丸（浓缩丸）

525 百合固金口服液

526 百合固金片

527 百合固金颗粒

528 百咳静糖浆

529 竹沥达痰丸

530 红色正金软膏

531 红灵散

532 红药贴膏

533 羊胆丸

534 羊藿三七胶囊

535 老鹳草软膏

536 耳聋丸

537 耳聋左慈丸

538 芎菊上清丸

539 芎菊上清丸（水丸）

540 芎菊上清片

541 血府逐瘀丸

542 血府逐瘀口服液

543 血府逐瘀胶囊

544 血美安胶囊

545 血栓心脉宁片

546 血栓心脉宁胶囊

547 血脂宁丸

548 血脂灵片

549 血脂康片

550 血脂康胶囊

551 血康口服液

552 西瓜霜润喉片

553 西青果茶

554 西青果颗粒

555 西黄丸

556 达立通颗粒

557 防风通圣丸

558 防风通圣颗粒

559 阳和解凝膏

560 阴虚胃痛颗粒

561 伸筋丹胶囊

562 伸筋活络丸

563 克伤痛搽剂

564 克咳片

565 克痢痧胶囊

566 克感利咽口服液

567 利肝隆颗粒

568 利咽解毒颗粒

569 利胆片

570 利胆排石片

571 利胆排石颗粒

572 利脑心胶囊

573 利膈丸

574 利鼻片

575 医痫丸

576 启脾丸

577 启脾口服液

578 坎离砂

579 妙灵丸

580 妙济丸

581 尪痹片

582 尪痹颗粒

583 尿塞通片

584 尿感宁颗粒

585 局方至宝散

586 快胃片

587 抗炎退热片

588 抗宫炎片

589 抗宫炎胶囊

590 抗宫炎颗粒

591　抗骨增生丸

592　抗骨增生胶囊

593　抗骨髓炎片

594　抗栓再造丸

595　抗病毒口服液

596　抗感口服液

597　抗感颗粒

598　护肝丸

599　护肝片

600　护肝宁片

601　护肝宁胶囊

602　护肝胶囊

603　护肝颗粒

604　更年安丸

605　更年安片

606　更年安胶囊

607　杏仁止咳合剂

608　杏苏止咳颗粒

609　杏苏止咳糖浆

610　杞菊地黄丸

611　杞菊地黄丸（浓缩丸）

612　杞菊地黄片

613　杞菊地黄胶囊

614　沈阳红药胶囊

615　沉香化气丸

616　灵丹草颗粒

617　灵宝护心丹

618　灵泽片

619　灵莲花颗粒

620　牡荆油胶丸

621　男康片

622　纯阳正气丸

623　肛泰软膏

624　肝炎康复丸

625　肠炎宁片

626　肠炎宁糖浆

627　肠胃宁片

628　肠胃适胶囊

629　肠康片

630　良附丸

631　芩芷鼻炎糖浆

632　芩连片

633　芩暴红止咳口服液

634　芩暴红止咳片

635　芩暴红止咳颗粒

636　芪冬颐心口服液

637　芪冬颐心颗粒

638　芪苈强心胶囊

639　芪参胶囊

640　芪蛭降糖胶囊

641　花红片

642　花红胶囊

643　花红颗粒

644　苁蓉益肾颗粒

645　苏子降气丸

646　苏合香丸

647　补中益气丸

648　补中益气丸（水丸）

649　补中益气合剂

650　补中益气颗粒

651　补心气口服液

652　补白颗粒

653　补肺活血胶囊

654　补肾养血丸

655　补肾益脑丸

656　补肾益脑片

657　补益地黄丸

658　补益蒺藜丸

659　补脾益肠丸

660　辛夷鼻炎丸

661　辛芩片

662　辛芩颗粒

663　远志酊

664　连花清瘟片

665　连花清瘟胶囊

666　连花清瘟颗粒

667　连蒲双清片

668　阿胶三宝膏

669　阿胶补血口服液

670　阿胶补血膏

671　阿魏化痞膏

672　附子理中丸

673　附子理中片

674　附桂骨痛片

675　附桂骨痛胶囊

676　附桂骨痛颗粒

677　驴胶补血颗粒

678　麦味地黄丸

679　龟鹿二仙膏

680　龟鹿补肾丸

681　龟龄集

682　乳宁颗粒

683 乳块消片

684 乳块消胶囊

685 乳核散结片

686 乳疾灵颗粒

687 乳康丸

688 乳康胶囊

689 乳增宁胶囊

690 乳癖消片

691 乳癖消胶囊

692 乳癖消颗粒

693 乳癖散结胶囊

694 京万红软膏

695 刺五加片

696 刺五加胶囊

697 刺五加脑灵合剂（刺五加脑灵液）

698 刺五加颗粒

699 参乌健脑胶囊

700 参芍片

701 参芍胶囊

702 参芪十一味颗粒

703 参芪口服液

704 参芪五味子片

705 参芪五味子胶囊

706　参苏丸

707　参附强心丸

708　参松养心胶囊

709　参苓白术丸

710　参苓白术散

711　参茸白凤丸

712　参茸固本片

713　参茸保胎丸

714　参桂胶囊

715　参精止渴丸

716　周氏回生丸

717　和中理脾丸

718　固本咳喘片

719　固本统血颗粒

720　固本益肠片

721　固经丸

722　固肾定喘丸

723　国公酒

724　坤宝丸

725　垂盆草颗粒

726　夜宁糖浆

727　季德胜蛇药片

728　定坤丹

729　定喘膏

730　宝咳宁颗粒

731　帕朱丸

732　抱龙丸

733　拔毒膏

734　拨云退翳丸

735　昆明山海棠片

736　明目上清片

737　明目地黄丸

738　明目地黄丸（浓缩丸）

739　松龄血脉康胶囊

740　板蓝大青片

741　板蓝根茶

742　板蓝根颗粒

743　枇杷止咳软胶囊

744　枇杷止咳胶囊

745　枇杷止咳颗粒

746　枇杷叶膏

747　枣仁安神胶囊

748　枣仁安神颗粒

749　治伤胶囊

750　治咳川贝枇杷滴丸

751　治咳川贝枇杷露

752　治糜康栓

753　泌石通胶囊

754　注射用双黄连（冻干）

755　注射用灯盏花素

756　泻肝安神丸

757　泻青丸

758　泻痢消胶囊

759　炎宁糖浆

760　狗皮膏

761　知柏地黄丸

762　知柏地黄丸（浓缩丸）

763　罗布麻茶

764　肥儿丸

765　肾宝合剂

766　肾宝糖浆

767　肾炎四味片

768　肾炎消肿片

769　肾炎康复片

770　肾炎舒片

771　肾炎解热片

772　肾复康胶囊

773　肾衰宁胶囊

774　肾康宁片

775 肾康宁胶囊

776 肾康宁颗粒

777 肿节风片

778 苦参片

779 表实感冒颗粒

780 表虚感冒颗粒

781 败毒散

782 软脉灵口服液

783 郁金银屑片

784 金水宝片

785 金水宝胶囊

786 金贝痰咳清颗粒

787 金佛止痛丸

788 金芪降糖片

789 金花明目丸

790 金果含片

791 金果饮

792 金果饮咽喉片

793 金振口服液

794 金莲花口服液

795 金莲花片

796 金莲花润喉片

797 金莲花胶囊

798　金莲花颗粒

799　金莲清热颗粒

800　金钱草片

801　金银花露

802　金黄利胆胶囊

803　金嗓开音丸

804　金嗓利咽丸

805　金嗓清音丸

806　金嗓散结丸

807　金蒲胶囊

808　降脂灵片

809　降脂灵颗粒

810　降脂通络软胶囊

811　降糖甲片

812　青叶胆片

813　青果丸

814　青娥丸

815　驻车丸

816　鱼腥草滴眼液

817　齿痛消炎灵颗粒

818　便通片

819　便通胶囊

820　保心片

821 保妇康栓

822 保赤散

823 保和丸

824 保和丸（水丸）

825 保和片

826 保和颗粒

827 保济丸

828 保济口服液

829 保胎丸

830 养心氏片

831 养心定悸口服液

832 养心定悸膏

833 养正消积胶囊

834 养血生发胶囊

835 养血荣筋丸

836 养血清脑丸

837 养血清脑颗粒

838 养阴生血合剂

839 养阴降糖片

840 养阴清肺丸

841 养阴清肺口服液

842 养阴清肺膏

843 养胃颗粒

844　冠心丹参片

845　冠心丹参胶囊

846　冠心生脉口服液

847　冠心苏合丸

848　冠心苏合胶囊

849　冠心舒通胶囊

850　前列欣胶囊

851　前列通片

852　前列舒丸

853　咳特灵片

854　咳特灵胶囊

855　咳喘宁口服液

856　咳喘顺丸

857　复方大青叶合剂

858　复方川贝精片

859　复方川芎片

860　复方川芎胶囊

861　复方丹参丸

862　复方丹参片

863　复方丹参胶囊

864　复方丹参喷雾剂（复方丹参气雾剂）

865　复方丹参滴丸

866　复方丹参颗粒

867　复方牛黄消炎胶囊

868　复方牛黄清胃丸

869　复方仙鹤草肠炎胶囊

870　复方瓜子金颗粒

871　复方石韦片

872　复方龙血竭胶囊

873　复方羊角片

874　复方血栓通胶囊

875　复方扶芳藤合剂

876　复方杏香兔耳风颗粒

877　复方皂矾丸

878　复方芩兰口服液

879　复方阿胶浆

880　复方陈香胃片

881　复方苦参肠炎康片

882　复方金钱草颗粒

883　复方金黄连颗粒

884　复方青黛丸

885　复方鱼腥草片

886　复方牵正膏

887　复方珍珠口疮颗粒

888　复方珍珠散

889　复方珍珠暗疮片

890　复方草珊瑚含片

891　复方夏天无片

892　复方消食茶（复方消食冲剂）

893　复方益肝丸

894　复方益肝灵胶囊

895　复方羚角降压片

896　复方黄连素片

897　复方黄柏液涂剂（复方黄柏液）

898　复方蛤青片

899　复方滇鸡血藤膏

900　复方满山红糖浆

901　复方熊胆滴眼液

902　复方鲜竹沥液

903　复芪止汗颗粒

904　复明片

905　复脉定胶囊

906　姜酊

907　姜黄消痤搽剂

908　宣肺止嗽合剂

909　宫宁颗粒

910　宫血宁胶囊

911　宫炎平片

912　宫炎平滴丸

913　宫瘤清片

914　宫瘤清胶囊

915　急支糖浆

916　恒古骨伤愈合剂

917　按摩软膏（按摩乳）

918　春血安胶囊

919　枳术丸

920　枳术颗粒

921　枳实导滞丸

922　柏子养心丸

923　柏子养心片

924　栀子金花丸

925　栀芩清热合剂

926　洁白丸

927　洋参保肺丸

928　津力达颗粒

929　活力苏口服液

930　活血止痛胶囊

931　活血止痛散

932　活血止痛膏

933　活血壮筋丸

934　活血通脉片

935　济生肾气丸

936 独一味片

937 独一味胶囊

938 独圣活血片

939 独活寄生丸

940 独活寄生合剂

941 珍珠胃安丸

942 珍黄胶囊

943 祖师麻片

944 祛风止痛丸

945 祛风止痛片

946 祛风止痛胶囊

947 祛风舒筋丸

948 祛伤消肿酊

949 祛痰灵口服液

950 神香苏合丸

951 穿心莲内酯滴丸

952 穿心莲片

953 穿心莲胶囊

954 穿龙骨刺片

955 胃乃安胶囊

956 胃立康片

957 胃安胶囊

958 胃肠安丸

959 胃肠复元膏

960 胃苏颗粒

961 胃疡灵颗粒

962 胃复春片

963 胃药胶囊

964 胃祥宁颗粒

965 胃康灵片

966 胃康灵胶囊

967 胃康灵颗粒

968 胃康胶囊

969 胃脘舒颗粒

970 胃舒宁颗粒

971 胆乐胶囊

972 胆宁片

973 胆石通胶囊

974 胆康胶囊

975 胡蜂酒

976 脉管复康片

977 茴香橘核丸

978 茵山莲颗粒

979 茵芪肝复颗粒

980 茵栀黄口服液

981 茵栀黄泡腾片

982　茵栀黄软胶囊

983　茵栀黄胶囊

984　茵栀黄颗粒

985　茵胆平肝胶囊

986　草香胃康胶囊

987　荜铃胃痛颗粒

988　荡石胶囊

989　药艾条

990　追风透骨丸

991　除湿白带丸

992　首乌丸

993　香苏正胃丸

994　香苏调胃片

995　香连丸

996　香连丸（浓缩丸）

997　香连化滞丸

998　香连片

999　香附丸

1000　香附丸（水丸）

1001　香砂六君丸

1002　香砂平胃丸

1003　香砂和中丸

1004　香砂养胃丸

1005　香砂养胃丸（浓缩丸）

1006　香砂养胃颗粒

1007　香砂枳术丸

1008　香砂胃苓丸

1009　骨友灵搽剂

1010　骨仙片

1011　骨折挫伤胶囊

1012　骨刺丸

1013　骨刺宁胶囊

1014　骨刺消痛片

1015　骨质宁搽剂

1016　骨疏康胶囊

1017　骨疏康颗粒

1018　骨痛灵酊

1019　健儿乐颗粒

1020　健儿消食口服液

1021　健民咽喉片

1022　健步丸

1023　健胃片

1024　健胃消食片

1025　健胃愈疡片

1026　健胃愈疡颗粒

1027　健脑丸

1028　健脑安神片

1029　健脑补肾丸

1030　健脑胶囊

1031　健脾丸

1032　健脾生血片

1033　健脾生血颗粒

1034　健脾糖浆

1035　夏天无片

1036　夏天无滴眼液

1037　夏枯草口服液

1038　夏枯草膏

1039　夏桑菊颗粒

1040　宽胸气雾剂

1041　柴连口服液

1042　柴胡口服液

1043　柴胡舒肝丸

1044　柴银口服液

1045　柴黄口服液

1046　柴黄片

1047　根痛平颗粒

1048　桂龙咳喘宁胶囊

1049　桂龙咳喘宁颗粒

1050　桂芍镇痫片

1051 桂附地黄丸

1052 桂附地黄胶囊

1053 桂附理中丸

1054 桂林西瓜霜

1055 桂枝茯苓丸

1056 桂枝茯苓片

1057 桂枝茯苓胶囊

1058 桑姜感冒片

1059 桑菊感冒丸

1060 桑菊感冒片

1061 桑菊感冒合剂

1062 桑葛降脂丸

1063 桔梗冬花片

1064 消炎止咳片

1065 消炎止痛膏

1066 消炎利胆片

1067 消炎退热颗粒

1068 消肿止痛酊

1069 消咳喘糖浆

1070 消络痛片

1071 消络痛胶囊

1072 消食退热糖浆

1073 消栓口服液

1074　消栓通络片

1075　消栓通络胶囊

1076　消栓通络颗粒

1077　消栓颗粒

1078　消眩止晕片

1079　消痔软膏

1080　消银片

1081　消银胶囊

1082　消渴丸

1083　消渴平片

1084　消渴灵片

1085　消痛贴膏

1086　消痤丸

1087　消瘀康片

1088　消瘀康胶囊

1089　消瘿丸

1090　消糜栓

1091　润肺止嗽丸

1092　烧伤灵酊

1093　烫伤油

1094　热炎宁片

1095　热炎宁合剂

1096　热炎宁颗粒

1097 热淋清颗粒

1098 狼疮丸

1099 珠黄吹喉散

1100 珠黄散

1101 疳积散

1102 益元散

1103 益心丸

1104 益心宁神片

1105 益心通脉颗粒

1106 益心舒丸

1107 益心舒片

1108 益心舒胶囊

1109 益心舒颗粒

1110 益心酮片

1111 益气养血口服液

1112 益气维血颗粒

1113 益母丸

1114 益母草口服液

1115 益母草片

1116 益母草胶囊

1117 益母草膏

1118 益母草颗粒

1119 益血生胶囊

1120　益肺清化膏

1121　益肾灵颗粒

1122　益脑宁片

1123　脂脉康胶囊（降脂灵胶囊）

1124　脂康颗粒

1125　脏连丸

1126　脑心通胶囊

1127　脑心清片

1128　脑乐静

1129　脑立清丸

1130　脑立清胶囊

1131　脑安胶囊

1132　脑脉泰胶囊

1133　脑栓通胶囊

1134　脑得生丸

1135　脑得生片

1136　脑得生胶囊

1137　脑得生颗粒

1138　致康胶囊

1139　荷丹片

1140　荷叶丸

1141　蚕蛾公补片

1142　蚝贝钙咀嚼片（蚝贝钙片）

1143　诺迪康胶囊

1144　调经丸

1145　调经止痛片

1146　调经促孕丸

1147　调经养血丸

1148　调经活血片

1149　调经活血胶囊

1150　调胃消滞丸

1151　逍遥丸

1152　逍遥丸（水丸）

1153　逍遥丸（浓缩丸）

1154　逍遥片

1155　逍遥胶囊

1156　逍遥颗粒

1157　通天口服液

1158　通心络胶囊

1159　通乐颗粒

1160　通关散

1161　通乳颗粒

1162　通宣理肺丸

1163　通宣理肺片

1164　通宣理肺胶囊

1165　通宣理肺颗粒

1166　通幽润燥丸

1167　通络祛痛膏

1168　通脉养心丸

1169　通脉养心口服液

1170　通窍鼻炎片

1171　通窍鼻炎胶囊

1172　通窍鼻炎颗粒

1173　通窍镇痛散

1174　通痹片

1175　通痹胶囊

1176　速效牛黄丸

1177　速效救心丸

1178　都梁丸

1179　都梁软胶囊

1180　都梁滴丸

1181　钻山风糖浆

1182　铁笛丸

1183　铁笛口服液

1184　培元通脑胶囊

1185　培坤丸

1186　寄生追风酒

1187　康尔心胶囊

1188　康妇软膏

1189　康妇消炎栓

1190　康莱特软胶囊

1191　得生丸

1192　排石颗粒

1193　控涎丸

1194　断血流片

1195　断血流胶囊

1196　断血流颗粒

1197　梅花点舌丸

1198　添精补肾膏

1199　清开灵口服液

1200　清开灵片

1201　清开灵泡腾片

1202　清开灵注射液

1203　清开灵软胶囊

1204　清开灵胶囊

1205　清开灵颗粒

1206　清气化痰丸

1207　清火栀麦丸

1208　清火栀麦片

1209　清火栀麦胶囊

1210　清宁丸

1211　清肝利胆口服液

1212　清肝利胆胶囊

1213　清泻丸

1214　清肺化痰丸

1215　清肺抑火丸

1216　清肺消炎丸

1217　清咽丸

1218　清咽利膈丸

1219　清咽润喉丸

1220　清胃保安丸

1221　清胃黄连丸（大蜜丸）

1222　清胃黄连丸（水丸）

1223　清胃黄连片

1224　清音丸

1225　清热灵颗粒

1226　清热凉血丸

1227　清热银花糖浆

1228　清热解毒口服液

1229　清热解毒片

1230　清热镇咳糖浆

1231　清眩丸

1232　清眩片

1233　清眩治瘫丸

1234　清脑降压片

1235　清脑降压胶囊

1236　清脑降压颗粒

1237　清淋颗粒

1238　清喉利咽颗粒

1239　清喉咽合剂

1240　清暑益气丸

1241　清瘟解毒丸

1242　清膈丸

1243　理中丸

1244　甜梦口服液（甜梦合剂）

1245　甜梦胶囊

1246　痔宁片

1247　痔炎消颗粒

1248　痔疮片

1249　痔康片

1250　维C银翘片

1251　维血宁合剂

1252　维血宁颗粒

1253　羚羊角胶囊

1254　羚羊清肺丸

1255　羚羊清肺颗粒

1256　羚羊感冒片

1257　萆薢分清丸

1258 虚寒胃痛颗粒

1259 蛇胆川贝软胶囊

1260 蛇胆川贝胶囊

1261 蛇胆川贝散

1262 蛇胆陈皮片

1263 蛇胆陈皮胶囊

1264 蛇胆陈皮散

1265 野菊花栓

1266 银丹心脑通软胶囊

1267 银杏叶片

1268 银杏叶胶囊

1269 银杏叶滴丸

1270 银屑灵膏

1271 银黄口服液

1272 银黄片

1273 银黄颗粒

1274 银翘双解栓

1275 银翘伤风胶囊

1276 银翘散

1277 银翘解毒丸（浓缩蜜丸）

1278 银翘解毒片

1279 银翘解毒软胶囊

1280 银翘解毒胶囊

1281　银翘解毒颗粒

1282　银蒲解毒片

1283　颈复康颗粒

1284　颈痛颗粒

1285　颈舒颗粒

1286　麻仁丸

1287　麻仁润肠丸

1288　麻仁滋脾丸

1289　黄氏响声丸

1290　黄杨宁片

1291　黄芪健胃膏

1292　黄芪颗粒

1293　黄连上清丸

1294　黄连上清片

1295　黄连上清胶囊

1296　黄连上清颗粒

1297　黄连羊肝丸

1298　黄连胶囊

1299　黄疸肝炎丸

1300　黄藤素片

1301　喉咽清口服液

1302　喉疾灵胶囊

1303　强力枇杷膏（蜜炼）

1304　强阳保肾丸

1305　强肾片

1306　散结镇痛胶囊

1307　斑秃丸

1308　普乐安片

1309　普乐安胶囊

1310　暑症片

1311　暑湿感冒颗粒

1312　温胃舒胶囊

1313　渴乐宁胶囊

1314　湿毒清胶囊

1315　湿热痹片

1316　溃疡散胶囊

1317　滋心阴口服液

1318　滋心阴胶囊

1319　滋心阴颗粒

1320　滋补生发片

1321　滑膜炎片

1322　滑膜炎胶囊

1323　滑膜炎颗粒

1324　猴头健胃灵片

1325　猴头健胃灵胶囊

1326　猴耳环消炎片

1327　猴耳环消炎胶囊

1328　琥珀还睛丸

1329　琥珀抱龙丸

1330　疏风定痛丸

1331　疏风活络丸

1332　疏痛安涂膜剂

1333　痛风定胶囊

1334　痛泻宁颗粒

1335　痛经丸

1336　痛经宝颗粒

1337　痢必灵片

1338　痧药

1339　筋痛消酊

1340　紫龙金片

1341　紫地宁血散

1342　紫花烧伤软膏

1343　紫金锭

1344　紫草软膏

1345　紫雪散

1346　脾胃舒丸

1347　舒心口服液

1348　舒心糖浆

1349　舒尔经颗粒

1350　舒肝丸

1351　舒肝平胃丸

1352　舒肝和胃丸

1353　舒胆胶囊

1354　舒胸片

1355　舒胸胶囊

1356　舒胸颗粒

1357　舒康贴膏

1358　舒筋丸

1359　舒筋活血定痛散

1360　舒筋活络酒

1361　舒筋通络颗粒

1362　葛根汤片

1363　葛根汤颗粒

1364　葛根芩连丸

1365　葛根芩连片

1366　葶贝胶囊

1367　蛤蚧补肾胶囊

1368　蛤蚧定喘丸

1369　蛤蚧定喘胶囊

1370　越鞠二陈丸

1371　越鞠丸

1372　越鞠保和丸

1373 跌打丸

1374 跌打活血散

1375 跌打镇痛膏

1376 锁阳固精丸

1377 雅叫哈顿散

1378 催汤丸

1379 微达康口服液

1380 愈风宁心片

1381 愈风宁心胶囊

1382 感冒止咳颗粒

1383 感冒止咳糖浆

1384 感冒退热颗粒

1385 感冒清热口服液

1386 感冒清热咀嚼片

1387 感冒清热胶囊

1388 感冒清热颗粒

1389 感冒舒颗粒

1390 新血宝胶囊

1391 新清宁片

1392 新雪颗粒

1393 新癀片

1394 暖脐膏

1395 槐角丸

1396　满山红油胶丸

1397　痰饮丸

1398　痹祺胶囊

1399　瘀血痹胶囊

1400　瘀血痹颗粒

1401　腰痛丸

1402　腰痛片

1403　腰痛宁胶囊

1404　腰痹通胶囊

1405　蒲地蓝消炎口服液

1406　裸花紫珠片

1407　裸花紫珠胶囊

1408　解肌宁嗽丸

1409　解郁安神颗粒

1410　障眼明片

1411　障翳散

1412　慢支固本颗粒

1413　慢肝解郁胶囊

1414　槟榔四消丸（大蜜丸）

1415　槟榔四消丸（水丸）

1416　熊胆胶囊

1417　熊胆救心丸

1418　熊胆痔灵栓

1419　熊胆痔灵膏

1420　稳心片

1421　稳心胶囊

1422　稳心颗粒

1423　精制冠心口服液

1424　精制冠心片

1425　精制冠心软胶囊

1426　精制冠心颗粒

1427　缩泉丸

1428　缩泉胶囊

1429　豨红通络口服液

1430　豨桐丸

1431　豨桐胶囊

1432　豨莶丸

1433　豨莶通栓丸

1434　豨莶通栓胶囊

1435　鲜益母草胶囊

1436　鼻炎片

1437　鼻炎灵片

1438　鼻炎通喷雾剂（鼻炎滴剂）

1439　鼻炎康片

1440　鼻咽灵片

1441　鼻咽清毒颗粒（鼻咽清毒剂）

1442　鼻渊丸

1443　鼻渊片

1444　鼻渊通窍颗粒

1445　鼻渊舒口服液

1446　鼻渊舒胶囊

1447　鼻窦炎口服液

1448　澳泰乐颗粒

1449　镇心痛口服液

1450　镇咳宁糖浆

1451　镇脑宁胶囊

1452　橘红丸

1453　橘红化痰丸

1454　橘红片

1455　橘红胶囊

1456　橘红痰咳液

1457　橘红颗粒

1458　癃闭舒胶囊

1459　癃清片

1460　糖尿乐胶囊

1461　糖脉康片

1462　糖脉康胶囊

1463　糖脉康颗粒

1464　避瘟散

1465　醒脑再造胶囊

1466　颠茄片

1467　颠茄酊

1468　黛蛤散

1469　礞石滚痰丸

1470　鹭鸶咯丸

1471　癣宁搽剂

1472　癣湿药水

1473　藿胆丸

1474　藿胆片

1475　藿香正气口服液

1476　藿香正气水

1477　藿香正气软胶囊

1478　藿香正气滴丸

1479　獾油搽剂

1480　癫痫平片

1481　癫痫康胶囊

1482　麝香风湿胶囊

1483　麝香抗栓胶囊

1484　麝香保心丸

1485　麝香祛痛气雾剂

1486　麝香祛痛搽剂

1487　麝香脑脉康胶囊

1488　麝香通心滴丸

1489　麝香痔疮栓

1490　麝香舒活搽剂

1491　麝香跌打风湿膏

1492　麝香镇痛膏

1493　蠲哮片

二部

1　乙胺利福异烟片

2　乙胺吡嗪利福异烟片（Ⅱ）

3　乙胺嘧啶

4　乙胺嘧啶片

5　乙琥胺

6　乙酰半胱氨酸

7　乙酰半胱氨酸颗粒

8　乙酰谷酰胺

9　乙酰谷酰胺注射液

10　乙酰唑胺

11　乙酰唑胺片

12　乙酰胺注射液

13　乙酰螺旋霉素

14　乙酰螺旋霉素片

15　乙酰螺旋霉素胶囊

16　乙醇

17 丁溴东莨菪碱

18 丁溴东莨菪碱注射液

19 丁溴东莨菪碱胶囊

20 丁酸氢化可的松

21 丁酸氢化可的松乳膏

22 七氟烷

23 二甲双胍格列本脲片（Ⅰ）

24 二甲双胍格列本脲片（Ⅱ）

25 二甲双胍格列本脲胶囊（Ⅰ）

26 二甲双胍格列本脲胶囊（Ⅱ）

27 二甲硅油

28 二甲硅油气雾剂

29 二甲硅油片

30 二甲磺酸阿米三嗪

31 二氟尼柳

32 二氟尼柳片

33 二氟尼柳胶囊

34 二氧化碳

35 二盐酸奎宁

36 二盐酸奎宁注射液

37 二羟丙茶碱

38 二羟丙茶碱片

39 二羟丙茶碱注射液

40 二巯丁二钠

41 二巯丁二酸

42 二巯丁二酸胶囊

43 二巯丙醇

44 二巯丙醇注射液

45 二硫化硒

46 二硫化硒洗剂

47 十一烯酸

48 十一烯酸锌

49 十一酸睾酮

50 十一酸睾酮注射液

51 十一酸睾酮软胶囊

52 三唑仑

53 三唑仑片

54 三硅酸镁

55 三磷酸腺苷二钠

56 口服补液盐散（Ⅰ）

57 口服补液盐散（Ⅱ）

58 口服补液盐散（Ⅲ）

59 大豆油（供注射用）

60 小儿复方磺胺甲噁唑片

61 小儿复方磺胺甲噁唑颗粒

62 山梨醇

63 山梨醇注射液

64 己烯雌酚

65 己烯雌酚片

66 己烯雌酚注射液

67 己酮可可碱

68 己酮可可碱肠溶片

69 己酮可可碱注射液

70 己酮可可碱氯化钠注射液

71 己酮可可碱缓释片

72 己酮可可碱葡萄糖注射液

73 己酸羟孕酮

74 己酸羟孕酮注射液

75 干燥硫酸钙

76 门冬氨酸

77 门冬氨酸鸟氨酸

78 门冬酰胺

79 门冬酰胺片

80 门冬酰胺酶（欧文）

81 门冬酰胺酶（埃希）

82 马来酸伊索拉定

83 马来酸伊索拉定片

84 马来酸曲美布汀

85 马来酸曲美布汀片

86 马来酸曲美布汀胶囊

87 马来酸麦角新碱

88 马来酸麦角新碱注射液

89 马来酸依那普利

90 马来酸依那普利片

91 马来酸依那普利胶囊

92 马来酸咪达唑仑片

93 马来酸氯苯那敏

94 马来酸氯苯那敏片

95 马来酸氯苯那敏注射液

96 马来酸氯苯那敏滴丸

97 马来酸噻吗洛尔

98 马来酸噻吗洛尔片

99 马来酸噻吗洛尔滴眼液

100 乌司他丁

101 乌司他丁溶液

102 乌拉地尔

103 乌拉地尔注射液

104 乌苯美司

105 乌苯美司胶囊

106 乌洛托品

107 五肽胃泌素

108 五肽胃泌素注射液

109　五氟利多

110　五氟利多片

111　六甲蜜胺

112　六甲蜜胺片

113　六甲蜜胺胶囊

114　升华硫

115　厄贝沙坦

116　厄贝沙坦分散片

117　厄贝沙坦片

118　厄贝沙坦胶囊

119　双水杨酯

120　双水杨酯片

121　双环醇

122　双环醇片

123　双氢青蒿素

124　双氢青蒿素片

125　双氢青蒿素哌喹片

126　双唑泰栓

127　双羟萘酸噻嘧啶

128　双羟萘酸噻嘧啶片

129　双羟萘酸噻嘧啶颗粒

130　双氯芬酸钠

131　双氯芬酸钠肠溶片

132 双氯芬酸钠肠溶胶囊

133 双氯芬酸钠栓

134 双氯芬酸钠搽剂

135 双氯芬酸钠滴眼液

136 双氯芬酸钾

137 双氯芬酸钾片

138 双氯芬酸钾胶囊

139 双氯非那胺

140 双氯非那胺片

141 双嘧达莫

142 双嘧达莫片

143 双嘧达莫注射液

144 双嘧达莫缓释胶囊

145 壬苯醇醚

146 壬苯醇醚阴道片

147 壬苯醇醚栓

148 壬苯醇醚膜

149 巴柳氮钠

150 巴氯芬

151 巴氯芬片

152 扎来普隆片

153 扎来普隆胶囊

154 无水葡萄糖

155　木糖醇

156　木糖醇颗粒

157　比沙可啶

158　比沙可啶肠溶片

159　比沙可啶栓

160　水合氯醛

161　水杨酸

162　水杨酸二乙胺

163　水杨酸二乙胺乳膏

164　水杨酸软膏

165　水杨酸镁

166　水杨酸镁片

167　水杨酸镁胶囊

168　牛磺酸

169　牛磺酸片

170　牛磺酸胶囊

171　牛磺酸散

172　牛磺酸滴眼液

173　牛磺酸颗粒

174　贝诺酯

175　贝诺酯片

176　贝敏伪麻片

177　丙戊酸钠

178　丙戊酸钠片

179　丙戊酸镁

180　丙戊酸镁片

181　丙谷胺

182　丙谷胺片

183　丙谷胺胶囊

184　丙泊酚

185　丙氨酰谷氨酰胺

186　丙氨酰谷氨酰胺注射液

187　丙氨酸

188　丙硫异烟胺

189　丙硫异烟胺肠溶片

190　丙硫氧嘧啶

191　丙硫氧嘧啶片

192　丙硫氧嘧啶肠溶片

193　丙酸交沙霉素

194　丙酸交沙霉素颗粒

195　丙酸倍氯米松

196　丙酸倍氯米松吸入气雾剂

197　丙酸倍氯米松乳膏

198　丙酸倍氯米松粉雾剂

199　丙酸氯倍他索

200　丙酸氯倍他索乳膏

201 丙酸睾酮

202 丙酸睾酮注射液

203 丙磺舒

204 丙磺舒片

205 丝氨酸

206 丝裂霉素

207 他扎罗汀

208 他扎罗汀凝胶

209 他唑巴坦

210 兰索拉唑

211 兰索拉唑肠溶片

212 加巴喷丁

213 加巴喷丁片

214 加巴喷丁胶囊

215 卡马西平

216 卡马西平片

217 卡马西平胶囊

218 卡巴胆碱

219 卡巴胆碱注射液

220 卡比马唑

221 卡比马唑片

222 卡比多巴

223 卡比多巴片

224 卡托普利

225 卡托普利片

226 卡前列甲酯

227 卡前列甲酯栓

228 卡莫司汀

229 卡莫司汀注射液

230 卡莫氟

231 卡莫氟片

232 卡铂

233 卡铂注射液

234 卡维地洛

235 卡维地洛片

236 卡维地洛胶囊

237 去乙酰毛花苷

238 去乙酰毛花苷注射液

239 去氢胆酸

240 去氢胆酸片

241 去氧氟尿苷

242 去氧氟尿苷分散片

243 去氧氟尿苷片

244 去氧氟尿苷胶囊

245 去羟肌苷

246 去羟肌苷肠溶胶囊

247　去羟肌苷咀嚼片

248　可待因桔梗片

249　右布洛芬

250　右布洛芬胶囊

251　右旋糖酐20

252　右旋糖酐20氯化钠注射液

253　右旋糖酐20葡萄糖注射液

254　右旋糖酐40

255　右旋糖酐40氯化钠注射液

256　右旋糖酐40葡萄糖注射液

257　右旋糖酐70

258　右旋糖酐70氯化钠注射液

259　右旋糖酐70葡萄糖注射液

260　右旋糖酐铁

261　右旋糖酐铁片

262　右旋糖酐铁注射液

263　右酮洛芬氨丁三醇

264　右酮洛芬氨丁三醇胶囊

265　叶酸

266　叶酸片

267　司他夫定

268　司他夫定胶囊

269　司可巴比妥钠

270　司可巴比妥钠胶囊

271　司坦唑醇

272　司坦唑醇片

273　司帕沙星

274　司帕沙星片

275　司帕沙星胶囊

276　司莫司汀

277　司莫司汀胶囊

278　头孢丙烯

279　头孢丙烯干混悬剂

280　头孢丙烯片

281　头孢丙烯胶囊

282　头孢丙烯颗粒

283　头孢他啶

284　头孢尼西钠

285　头孢地尼

286　头孢地尼胶囊

287　头孢地嗪钠

288　头孢曲松钠

289　头孢米诺钠

290　头孢西丁钠

291　头孢克肟

292　头孢克肟片

293　头孢克肟胶囊

294　头孢克肟颗粒

295　头孢克洛

296　头孢克洛干混悬剂

297　头孢克洛片

298　头孢克洛胶囊

299　头孢克洛颗粒

300　头孢呋辛钠

301　头孢呋辛酯

302　头孢呋辛酯片

303　头孢呋辛酯胶囊

304　头孢孟多酯钠

305　头孢拉定

306　头孢拉定干混悬剂

307　头孢拉定片

308　头孢拉定胶囊

309　头孢拉定颗粒

310　头孢泊肟酯

311　头孢泊肟酯干混悬剂

312　头孢泊肟酯片

313　头孢泊肟酯胶囊

314　头孢哌酮

315　头孢哌酮钠

316　头孢美唑钠

317　头孢唑肟钠

318　头孢唑林钠

319　头孢氨苄

320　头孢氨苄干混悬剂

321　头孢氨苄片

322　头孢氨苄胶囊

323　头孢氨苄颗粒

324　头孢羟氨苄

325　头孢羟氨苄片

326　头孢羟氨苄胶囊

327　头孢羟氨苄颗粒

328　头孢替唑钠

329　头孢硫脒

330　头孢噻吩钠

331　头孢噻肟钠

332　对乙酰氨基酚

333　对乙酰氨基酚片

334　对乙酰氨基酚咀嚼片

335　对乙酰氨基酚泡腾片

336　对乙酰氨基酚注射液

337　对乙酰氨基酚栓

338　对乙酰氨基酚胶囊

339　对乙酰氨基酚滴剂

340　对乙酰氨基酚颗粒

341　对乙酰氨基酚凝胶

342　对氨基水杨酸钠

343　对氨基水杨酸钠肠溶片

344　尼可刹米

345　尼可刹米注射液

346　尼尔雌醇

347　尼尔雌醇片

348　尼美舒利

349　尼美舒利片

350　尼索地平

351　尼索地平片

352　尼索地平胶囊

353　尼莫地平

354　尼莫地平分散片

355　尼莫地平片

356　尼莫地平注射液

357　尼莫地平软胶囊

358　尼莫地平胶囊

359　尼群地平

360　尼群地平片

361　尼群地平软胶囊

362　左炔诺孕酮

363　左炔诺孕酮片

364　左炔诺孕酮炔雌醇（三相）片

365　左炔诺孕酮炔雌醚片

366　左氧氟沙星

367　左氧氟沙星片

368　左氧氟沙星滴眼液

369　左旋多巴

370　左旋多巴片

371　左旋多巴胶囊

372　左羟丙哌嗪

373　左羟丙哌嗪片

374　左羟丙哌嗪胶囊

375　左奥硝唑

376　左奥硝唑氯化钠注射液

377　布洛伪麻片

378　布洛伪麻胶囊

379　布洛芬

380　布洛芬口服溶液

381　布洛芬片

382　布洛芬胶囊

383　布洛芬混悬滴剂

384　布洛芬缓释胶囊

385 布洛芬糖浆

386 布美他尼

387 布美他尼片

388 布美他尼注射液

389 戊四硝酯片

390 戊四硝酯粉

391 戊酸雌二醇

392 戊酸雌二醇注射液

393 扑米酮

394 扑米酮片

395 本芴醇

396 灭菌注射用水

397 甘油

398 甘油果糖氯化钠注射液

399 甘油栓

400 甘油磷酸钠

401 甘油磷酸钠注射液

402 甘氨双唑钠

403 甘氨酰谷氨酰胺

404 甘氨酸

405 甘氨酸冲洗液

406 甘露醇

407 甘露醇注射液

408　生长抑素

409　生理氯化钠溶液

410　甲地高辛

411　甲地高辛片

412　甲状腺片

413　甲状腺粉

414　甲芬那酸

415　甲芬那酸片

416　甲芬那酸胶囊

417　甲苯咪唑

418　甲苯咪唑片

419　甲苯磺丁脲

420　甲苯磺丁脲片

421　甲砜霉素

422　甲砜霉素肠溶片

423　甲砜霉素胶囊

424　甲氧苄啶

425　甲氧苄啶片

426　甲氧苄啶注射液

427　甲氧氯普胺

428　甲氧氯普胺片

429　甲氨蝶呤

430　甲氨蝶呤片

431 甲钴胺

432 甲钴胺胶囊

433 甲基多巴

434 甲基多巴片

435 甲酚

436 甲酚皂溶液

437 甲巯咪唑

438 甲巯咪唑片

439 甲巯咪唑肠溶片

440 甲硝唑

441 甲硝唑片

442 甲硝唑阴道泡腾片

443 甲硝唑注射液

444 甲硝唑栓

445 甲硝唑胶囊

446 甲硝唑氯化钠注射液

447 甲硝唑葡萄糖注射液

448 甲硝唑凝胶

449 甲硫氨酸

450 甲硫氨酸片

451 甲硫酸新斯的明

452 甲硫酸新斯的明注射液

453 甲紫

454　甲紫溶液

455　甲睾酮

456　甲睾酮片

457　甲磺酸加贝酯

458　甲磺酸培氟沙星

459　甲磺酸培氟沙星片

460　甲磺酸培氟沙星注射液

461　甲磺酸培氟沙星胶囊

462　甲磺酸酚妥拉明

463　甲磺酸酚妥拉明片

464　甲磺酸酚妥拉明注射液

465　甲磺酸酚妥拉明胶囊

466　甲醛溶液

467　白消安

468　白消安片

469　石杉碱甲

470　石杉碱甲片

471　石杉碱甲注射液

472　石杉碱甲胶囊

473　艾司唑仑

474　艾司唑仑片

475　艾司唑仑注射液

476　艾司奥美拉唑钠

477　艾司奥美拉唑镁肠溶片

478　亚叶酸钙

479　亚叶酸钙片

480　亚叶酸钙注射液

481　亚叶酸钙胶囊

482　亚甲蓝

483　亚甲蓝注射液

484　亚硝酸钠

485　亚硫酸氢钠甲萘醌

486　亚硫酸氢钠甲萘醌注射液

487　交沙霉素

488　交沙霉素片

489　伊曲康唑

490　伊曲康唑胶囊

491　伏立康唑

492　伏立康唑片

493　伏立康唑胶囊

494　冰醋酸

495　华法林钠

496　华法林钠片

497　吉他霉素

498　吉他霉素片

499　吉非罗齐

500 吉非罗齐胶囊

501 吗替麦考酚酯

502 吗替麦考酚酯片

503 吗替麦考酚酯胶囊

504 吗氯贝胺

505 吗氯贝胺片

506 吗氯贝胺胶囊

507 地红霉素

508 地西泮

509 地西泮片

510 地西泮注射液

511 地高辛

512 地高辛口服溶液

513 地高辛片

514 地高辛注射液

515 地奥司明

516 地奥司明片

517 地塞米松

518 地塞米松片

519 地塞米松磷酸钠

520 地塞米松磷酸钠注射液

521 地塞米松磷酸钠滴眼液

522 地蒽酚

523　地蒽酚软膏

524　多巴丝肼片

525　多巴丝肼胶囊

526　多索茶碱

527　多索茶碱片

528　多索茶碱注射液

529　多索茶碱胶囊

530　多烯酸乙酯

531　多烯酸乙酯软胶囊

532　多潘立酮

533　多潘立酮片

534　安钠咖注射液

535　异卡波肼

536　异卡波肼片

537　异戊巴比妥

538　异戊巴比妥片

539　异戊巴比妥钠

540　异环磷酰胺

541　异亮氨酸

542　异氟烷

543　异烟肼

544　异烟肼片

545　异烟腙

546　异烟腙片

547　异维A酸

548　异维Ａ酸软胶囊

549　异维A酸凝胶

550　异福片

551　异福胶囊

552　异福酰胺片

553　异福酰胺胶囊

554　托西酸舒他西林

555　托西酸舒他西林片

556　托西酸舒他西林胶囊

557　托西酸舒他西林颗粒

558　托吡卡胺

559　托吡卡胺滴眼液

560　曲尼司特

561　曲尼司特片

562　曲尼司特胶囊

563　曲安西龙

564　曲安西龙片

565　曲安奈德

566　曲安奈德注射液

567　曲安奈德益康唑乳膏

568　曲克芦丁

569 曲克芦丁片

570 灰黄霉素

571 灰黄霉素片

572 米力农

573 米力农注射液

574 米非司酮

575 米非司酮片

576 米诺地尔

577 米诺地尔片

578 红霉素

579 红霉素肠溶片

580 红霉素肠溶胶囊

581 红霉素软膏

582 红霉素眼膏

583 肌苷

584 肌苷口服溶液

585 肌苷片

586 肌苷注射液

587 肌苷胶囊

588 肌苷氯化钠注射液

589 肌苷葡萄糖注射液

590 色甘酸钠

591 色甘酸钠滴眼液

592 色氨酸

593 西尼地平

594 西尼地平片

595 西地碘含片

596 西咪替丁

597 西咪替丁片

598 西咪替丁胶囊

599 西咪替丁氯化钠注射液

600 西洛他唑

601 西洛他唑胶囊

602 达那唑

603 达那唑胶囊

604 过氧化氢溶液

605 过氧苯甲酰

606 过氧苯甲酰乳膏

607 过氧苯甲酰凝胶

608 那可丁

609 那可丁片

610 那格列奈

611 那格列奈片

612 那格列奈胶囊

613 钆贝葡胺注射液

614 钆喷酸葡胺注射液

615　齐多夫定

616　齐多夫定片

617　齐多夫定注射液

618　齐多夫定胶囊

619　齐多拉米双夫定片

620　两性霉素B

621　佐匹克隆

622　佐匹克隆片

623　佐匹克隆胶囊

624　佐米曲普坦

625　佐米曲普坦片

626　克拉维酸钾

627　克拉霉素

628　克拉霉素片

629　克拉霉素胶囊

630　克拉霉素颗粒

631　克林霉素磷酸酯

632　克林霉素磷酸酯外用溶液

633　克林霉素磷酸酯注射液

634　克林霉素磷酸酯栓

635　克罗米通

636　克罗米通乳膏

637　克霉唑

638　克霉唑口腔药膜

639　克霉唑阴道片

640　克霉唑乳膏

641　克霉唑药膜

642　克霉唑倍他米松乳膏

643　克霉唑栓

644　克霉唑喷雾剂

645　克霉唑溶液

646　利巴韦林

647　利巴韦林口服溶液

648　利巴韦林分散片

649　利巴韦林片

650　利巴韦林含片

651　利巴韦林注射液

652　利巴韦林胶囊

653　利巴韦林氯化钠注射液

654　利巴韦林葡萄糖注射液

655　利巴韦林滴眼液

656　利巴韦林滴鼻液

657　利巴韦林颗粒

658　利血平

659　利血平片

660　利血平注射液

661　利培酮

662　利培酮口服溶液

663　利培酮口崩片

664　利培酮片

665　利培酮胶囊

666　利鲁唑

667　利鲁唑片

668　利福平

669　利福平片

670　利福平胶囊

671　利福昔明

672　利福昔明干混悬剂

673　利福昔明片

674　利福昔明胶囊

675　别嘌醇

676　别嘌醇片

677　劳拉西泮

678　劳拉西泮片

679　吡拉西坦

680　吡拉西坦口服溶液

681　吡拉西坦片

682　吡拉西坦注射液

683　吡拉西坦胶囊

684　吡拉西坦氯化钠注射液

685　吡罗昔康

686　吡罗昔康片

687　吡罗昔康肠溶片

688　吡罗昔康注射液

689　吡罗昔康软膏

690　吡罗昔康胶囊

691　吡罗昔康凝胶

692　吡哌酸

693　吡哌酸片

694　吡哌酸胶囊

695　吡喹酮

696　吡喹酮片

697　吡嗪酰胺

698　吡嗪酰胺片

699　吡嗪酰胺胶囊

700　含糖胃蛋白酶

701　吲达帕胺

702　吲达帕胺片

703　吲达帕胺胶囊

704　吲哚洛尔

705　吲哚美辛

706　吲哚美辛肠溶片

707 吲哚美辛乳膏

708 吲哚美辛贴片

709 吲哚美辛栓

710 吲哚美辛胶囊

711 吲哚美辛搽剂

712 吲哚美辛缓释片

713 吲哚美辛缓释胶囊

714 吲哚菁绿

715 吸入用七氟烷

716 吸收性明胶海绵

717 呋喃妥因

718 呋喃妥因肠溶片

719 呋喃唑酮

720 呋喃唑酮片

721 呋塞米

722 呋塞米片

723 呋塞米注射液

724 坎地沙坦酯

725 妥布霉素

726 妥布霉素地塞米松眼膏

727 妥布霉素地塞米松滴眼液

728 妥布霉素滴眼液

729 尿促性素

730 尿素

731 尿素乳膏

732 尿素软膏

733 尿激酶

734 抑肽酶

735 更昔洛韦

736 更昔洛韦氯化钠注射液

737 杆菌肽

738 杆菌肽软膏

739 杆菌肽眼膏

740 来氟米特

741 来氟米特片

742 沙丁胺醇

743 沙丁胺醇吸入气雾剂

744 沙利度胺

745 沙利度胺片

746 泛昔洛韦

747 泛昔洛韦片

748 泛昔洛韦胶囊

749 泛酸钙

750 泛酸钙片

751 泛影葡胺注射液

752 泛影酸

753　泛影酸钠注射液

754　纯化水

755　肝素钙

756　肝素钙注射液

757　肝素钠

758　肝素钠乳膏

759　肝素钠注射液

760　芬布芬

761　芬布芬片

762　芬布芬胶囊

763　苄达赖氨酸

764　苄达赖氨酸滴眼液

765　苄星青霉素

766　苄氟噻嗪

767　苄氟噻嗪片

768　苏氨酸

769　谷丙甘氨酸胶囊

770　谷氨酰胺

771　谷氨酰胺胶囊

772　谷氨酰胺颗粒

773　谷氨酸

774　谷氨酸片

775　谷氨酸钠

776　谷氨酸钠注射液

777　谷氨酸钾注射液

778　谷胱甘肽片

779　辛伐他汀

780　辛伐他汀片

781　辛伐他汀胶囊

782　间苯二酚

783　阿仑膦酸钠

784　阿仑膦酸钠片

785　阿仑膦酸钠肠溶片

786　阿片

787　阿片片

788　阿片酊

789　阿片粉

790　阿卡波糖

791　阿卡波糖片

792　阿卡波糖胶囊

793　阿司匹林

794　阿司匹林片

795　阿司匹林肠溶片

796　阿司匹林肠溶胶囊

797　阿司匹林泡腾片

798　阿司匹林栓

799 阿立哌唑

800 阿立哌唑口崩片

801 阿立哌唑片

802 阿立哌唑胶囊

803 阿米卡星

804 阿那曲唑

805 阿奇霉素

806 阿奇霉素干混悬剂

807 阿奇霉素片

808 阿奇霉素胶囊

809 阿奇霉素颗粒

810 阿昔洛韦

811 阿昔洛韦片

812 阿昔洛韦乳膏

813 阿昔洛韦咀嚼片

814 阿昔洛韦胶囊

815 阿昔洛韦葡萄糖注射液

816 阿昔洛韦滴眼液

817 阿昔洛韦颗粒

818 阿昔莫司

819 阿昔莫司胶囊

820 阿法骨化醇

821 阿法骨化醇片

822　阿法骨化醇软胶囊

823　阿苯达唑

824　阿苯达唑片

825　阿苯达唑胶囊

826　阿苯达唑颗粒

827　阿洛西林钠

828　阿桔片

829　阿莫西林

830　阿莫西林干混悬剂

831　阿莫西林片

832　阿莫西林克拉维酸钾干混悬剂

833　阿莫西林克拉维酸钾分散片

834　阿莫西林克拉维酸钾片

835　阿莫西林克拉维酸钾颗粒

836　阿莫西林钠

837　阿莫西林胶囊

838　阿莫西林颗粒

839　阿维A

840　阿维A胶囊

841　阿普唑仑

842　阿普唑仑片

843　阿替洛尔

844　阿替洛尔片

845 阿德福韦酯

846 阿德福韦酯片

847 阿德福韦酯胶囊

848 阿魏酸哌嗪

849 阿魏酸哌嗪片

850 阿魏酸钠

851 阿魏酸钠片

852 麦白霉素

853 麦白霉素片

854 麦白霉素胶囊

855 麦角胺咖啡因片

856 乳果糖口服溶液

857 乳果糖浓溶液

858 乳酶生

859 乳酶生片

860 乳酸

861 乳酸依沙吖啶

862 乳酸依沙吖啶注射液

863 乳酸依沙吖啶溶液

864 乳酸环丙沙星注射液

865 乳酸钙

866 乳酸钙片

867 乳酸钠林格注射液

868 乳酸钠注射液

869 乳酸钠溶液

870 乳糖酸红霉素

871 依他尼酸

872 依他尼酸片

873 依他尼酸钠

874 依地酸钙钠

875 依地酸钙钠注射液

876 依托红霉素

877 依托红霉素片

878 依托红霉素胶囊

879 依托红霉素颗粒

880 依托泊苷

881 依托泊苷注射液

882 依托泊苷软胶囊

883 依托咪酯

884 依托咪酯注射液

885 依托度酸

886 依托度酸片

887 依西美坦

888 依西美坦片

889 依西美坦胶囊

890 依达拉奉

891 依达拉奉注射液

892 依诺沙星

893 依诺沙星片

894 依诺沙星乳膏

895 依诺沙星胶囊

896 依诺沙星滴眼液

897 依普黄酮

898 依普黄酮片

899 依替膦酸二钠

900 依替膦酸二钠片

901 单硝酸异山梨酯

902 单硝酸异山梨酯片

903 单硝酸异山梨酯注射液

904 单硝酸异山梨酯胶囊

905 单硝酸异山梨酯氯化钠注射液

906 单硝酸异山梨酯缓释片

907 单硝酸异山梨酯葡萄糖注射液

908 咖啡因

909 垂体后叶注射液

910 垂体后叶粉

911 奈韦拉平

912 奈韦拉平片

913 奋乃静

914 奋乃静片

915 奋乃静注射液

916 帕司烟肼

917 帕米膦酸二钠

918 帕米膦酸二钠注射液

919 拉米夫定

920 拉米夫定片

921 拉氧头孢钠

922 放线菌素 D

923 明胶

924 林旦

925 林旦乳膏

926 果糖

927 法罗培南钠

928 法莫替丁

929 法莫替丁片

930 法莫替丁注射液

931 法莫替丁胶囊

932 法莫替丁颗粒

933 注射用二巯丁二钠

934 注射用门冬酰胺酶（欧文）

935 注射用门冬酰胺酶（埃希）

936 注射用乌司他丁

937　注射用水

938　注射用丙戊酸钠

939　注射用丙氨酰谷氨酰胺

940　注射用丝裂霉素

941　注射用兰索拉唑

942　注射用头孢他啶

943　注射用头孢尼西钠

944　注射用头孢地嗪钠

945　注射用头孢曲松钠

946　注射用头孢米诺钠

947　注射用头孢西丁钠

948　注射用头孢呋辛钠

949　注射用头孢孟多酯钠

950　注射用头孢拉定

951　注射用头孢哌酮钠

952　注射用头孢哌酮钠舒巴坦钠

953　注射用头孢美唑钠

954　注射用头孢唑肟钠

955　注射用头孢唑林钠

956　注射用头孢替唑钠

957　注射用头孢硫脒

958　注射用头孢噻吩钠

959　注射用头孢噻肟钠

960　注射用对氨基水杨酸钠

961　注射用甘氨双唑钠

962　注射用生长抑素

963　注射用甲氨蝶呤

964　注射用甲磺酸加贝酯

965　注射用甲磺酸酚妥拉明

966　注射用艾司奥美拉唑钠

967　注射用异戊巴比妥钠

968　注射用异环磷酰胺

969　注射用异烟肼

970　注射用肌苷

971　注射用两性霉素B

972　注射用利巴韦林

973　注射用利福平

974　注射用吡拉西坦

975　注射用吲哚菁绿

976　注射用尿促性素

977　注射用尿激酶

978　注射用抑肽酶

979　注射用更昔洛韦

980　注射用苄星青霉素

981　注射用阿奇霉素

982　注射用阿昔洛韦

983　注射用阿洛西林钠

984　注射用阿莫西林钠

985　注射用阿莫西林钠克拉维酸钾

986　注射用阿魏酸钠

987　注射用乳糖酸红霉素

988　注射用依他尼酸钠

989　注射用拉氧头孢钠

990　注射用放线菌素 D

991　注射用法莫替丁

992　注射用泮托拉唑钠

993　注射用环磷腺苷

994　注射用环磷酰胺

995　注射用细胞色素C

996　注射用苯巴比妥钠

997　注射用苯妥英钠

998　注射用苯唑西林钠

999　注射用苯磺顺阿曲库铵

1000　注射用青蒿琥酯

1001　注射用青霉素钠

1002　注射用青霉素钾

1003　注射用前列地尔

1004　注射用哌库溴铵

1005　注射用哌拉西林钠

1006　注射用哌拉西林钠他唑巴坦钠

1007　注射用氟氯西林钠

1008　注射用氢化可的松琥珀酸钠

1009　注射用氢溴酸右美沙芬

1010　注射用玻璃酸酶

1011 注射用绒促性素

1012　注射用美罗培南

1013　注射用美洛西林钠

1014　注射用胞磷胆碱钠

1015　注射用胞磷胆碱钠肌苷

1016　注射用重组人生长激素

1017　注射用顺铂

1018　注射用氨力农

1019　注射用氨曲南

1020　注射用氨苄西林钠

1021　注射用氨苄西林钠舒巴坦钠

1022　注射用盐酸丁卡因

1023　注射用盐酸万古霉素

1024　注射用盐酸大观霉素

1025　注射用盐酸去甲万古霉素

1026　注射用盐酸四环素

1027　注射用盐酸头孢甲肟

1028　注射用盐酸头孢吡肟

1029　注射用盐酸平阳霉素

1030　注射用盐酸甲氯芬酯

1031　注射用盐酸艾司洛尔

1032　注射用盐酸吉西他滨

1033　注射用盐酸多柔比星

1034　注射用盐酸托烷司琼

1035　注射用盐酸米托蒽醌

1036　注射用盐酸吡硫醇

1037　注射用盐酸纳洛酮

1038　注射用盐酸阿糖胞苷

1039　注射用盐酸罗哌卡因

1040　注射用盐酸表柔比星

1041　注射用盐酸柔红霉素

1042　注射用盐酸普鲁卡因

1043　注射用盐酸瑞芬太尼

1044　注射用胰蛋白酶

1045　注射用胸腺五肽

1046　注射用胸腺法新

1047　注射用维库溴铵

1048　注射用奥沙利铂

1049　注射用奥美拉唑钠

1050　注射用普鲁卡因青霉素

1051　注射用替考拉宁

1052　注射用棓丙酯

1053　注射用氯唑西林钠

1054　注射用氯诺昔康

1055　注射用琥珀氯霉素

1056　注射用硝普钠

1057　注射用硝酸异山梨酯

1058　注射用硫喷妥钠

1059　注射用硫酸长春地辛

1060　注射用硫酸长春新碱

1061　注射用硫酸长春碱

1062　注射用硫酸卡那霉素

1063　注射用硫酸多黏菌素B

1064　注射用硫酸阿米卡星

1065　注射用硫酸依替米星

1066　注射用硫酸卷曲霉素

1067　注射用硫酸核糖霉素

1068　注射用硫酸普拉睾酮钠

1069　注射用硫酸链霉素

1070　注射用舒巴坦钠

1071　注射用葛根素

1072　注射用羧苄西林钠

1073　注射用赖氨匹林

1074　注射用缩宫素

1075　注射用鲑降钙素

1076　注射用醋酸丙氨瑞林

1077　注射用醋酸奥曲肽

1078　注射用磺苄西林钠

1079　注射用磺胺嘧啶钠

1080　注射用磷霉素钠

1081　注射用糜蛋白酶

1082　泮托拉唑钠

1083　泮托拉唑钠肠溶胶囊

1084　泼尼松

1085　泼尼松龙

1086　泼尼松龙片

1087　炔孕酮

1088　炔孕酮片

1089　炔诺孕酮

1090　炔诺孕酮炔雌醚片

1091　炔诺酮

1092　炔诺酮片

1093　炔诺酮滴丸

1094　炔雌醇

1095　炔雌醇片

1096　炔雌醚

1097　环丙沙星

1098　环吡酮胺

1099　环吡酮胺乳膏

1100 环孢素

1101　环孢素口服溶液

1102　环扁桃酯

1103 环扁桃酯胶囊

1104　环磷腺苷

1105环磷酰胺

1106 环磷酰胺片

1107　　组氨酸

1108　细胞色素C注射液

1109　细胞色素C溶液

1110　罗红霉素

1111　罗红霉素干混悬剂

1112　罗红霉素片

1113　罗红霉素胶囊

1114　罗红霉素颗粒

1115　罗库溴铵

1116　罗库溴铵注射液

1117　罗通定

1118　罗通定片

1119　肾上腺素

1120　苯丁酸氮芥

1121　苯丁酸氮芥纸型片

1122　苯巴比妥

1123　苯巴比妥片

1124 苯巴比妥钠

1125　苯扎贝特

1126　苯扎贝特片

1127 苯扎贝特胶囊

1128　苯扎氯铵

1129　苯扎氯铵溶液

1130　苯扎溴铵

1131苯扎溴铵溶液

1132　苯丙氨酸

1133苯丙酸诺龙

1134　苯丙酸诺龙注射液

1135　苯丙醇

1136　苯丙醇软胶囊

1137　苯甲酸

1138　苯甲酸利扎曲普坦

1139　苯甲酸雌二醇

1140　苯甲酸雌二醇注射液

1141　苯甲醇

1142　苯佐卡因

1143　苯妥英钠

1144　苯妥英钠片

1145　苯唑西林钠

1146　苯唑西林钠片

1147　苯唑西林钠胶囊

1148　苯酚

1149　苯溴马隆

1150　苯溴马隆片

1151　苯溴马隆胶囊

1152　苯噻啶

1153　苯噻啶片

1154　苯磺顺阿曲库铵

1155　苯磺酸氨氯地平

1156　苯磺酸氨氯地平片

1157　苯磺酸氨氯地平胶囊

1158　软皂

1159　青蒿素

1160　青蒿素哌喹片

1161　青蒿琥酯

1162　青蒿琥酯片

1163　青霉素V钾

1164　青霉素V钾片

1165　青霉素V钾胶囊

1166　青霉素钠

1167　青霉素钾

1168　青霉胺

1169　青霉胺片

1170　非那雄胺

1171　非那雄胺片

1172　非那雄胺胶囊

1173　非洛地平

1174　非洛地平片

1175　非诺贝特

1176　非诺贝特片

1177　非诺贝特胶囊

1178　非诺洛芬钙

1179　非诺洛芬钙片

1180　鱼石脂

1181　鱼石脂软膏

1182　鱼肝油

1183　鱼肝油酸钠注射液

1184　亮氨酸

1185　前列地尔

1186　咪达唑仑

1187　咪达唑仑注射液

1188　咪康唑氯倍他索乳膏

1189　哈西奈德

1190　哈西奈德乳膏

1191　哈西奈德软膏

1192　哈西奈德涂膜

1193　哈西奈德溶液

1194　哌库溴铵

1195　哌拉西林

1196　哌拉西林钠

1197　复方十一烯酸锌软膏

1198　复方己酸羟孕酮注射液

1199　复方门冬维甘滴眼液

1200　复方卡比多巴片

1201　复方卡托普利片

1202　复方左炔诺孕酮片

1203　复方左炔诺孕酮滴丸

1204　复方甘草口服溶液

1205　复方甘草片

1206　复方甲苯咪唑片

1207　复方地芬诺酯片

1208　复方克霉唑乳膏

1209　复方利血平片

1210　复方利血平氨苯蝶啶片

1211复方呋塞米片

1212　复方泛影葡胺注射液

1213　复方乳酸钠葡萄糖注射液

1214　复方庚酸炔诺酮注射液

1215　复方炔诺孕酮片

1216　复方炔诺孕酮滴丸

1217　复方炔诺酮片

1218　复方炔诺酮膜

1219　复方氢氧化铝片

1220　复方氨基酸注射液（18AA）

1221　复方氨基酸注射液（18AA　Ⅰ）

1222　复方氨基酸注射液（18AA　Ⅱ）

1223　复方氨基酸注射液（18AA　Ⅲ）

1224　复方氨基酸注射液（18AA　Ⅳ）

1225　复方盐酸阿米洛利片

1226　复方莪术油栓

1227　复方维生素C钠咀嚼片

1228　复方铝酸铋片

1229　复方铝酸铋胶囊

1230　复方氯化钠注射液

1231　复方氯化钠滴眼液

1232　复方葡萄糖酸钙口服溶液

1233　复方新霉素软膏

1234　复方硼砂含漱液

1235　复方蒿甲醚片

1236　复方酮康唑乳膏

1237　复方樟脑酊

1238　复方醋酸甲地孕酮片

1239　复方醋酸地塞米松乳膏

1240　复方磺胺甲噁唑口服混悬液

1241　复方磺胺甲噁唑片

1242　复方磺胺甲噁唑注射液

1243　复方磺胺甲噁唑胶囊

1244　复方磺胺甲噁唑颗粒

1245　复方磺胺嘧啶片

1246　复方磷酸萘酚喹片

1247　度米芬

1248　度米芬滴丸

1249　枸橼酸乙胺嗪

1250　枸橼酸乙胺嗪片

1251　枸橼酸他莫昔芬

1252　枸橼酸他莫昔芬片

1253　枸橼酸托瑞米芬

1254　枸橼酸托瑞米芬片

1255　枸橼酸芬太尼

1256　枸橼酸芬太尼注射液

1257　枸橼酸哌嗪

1258　枸橼酸哌嗪片

1259　枸橼酸哌嗪糖浆

1260　枸橼酸钙

1261　枸橼酸钙片

1262　枸橼酸钠

1263　枸橼酸钾

1264　枸橼酸铋钾

1265　枸橼酸铋钾片

1266　枸橼酸铋钾胶囊

1267　枸橼酸铋钾颗粒

1268　枸橼酸铋雷尼替丁

1269　枸橼酸铋雷尼替丁片

1270　枸橼酸铋雷尼替丁胶囊

1271　枸橼酸喷托维林

1272　枸橼酸喷托维林片

1273　枸橼酸喷托维林滴丸

1274　枸橼酸氯米芬

1275　枸橼酸氯米芬片

1276　枸橼酸氯米芬胶囊

1277　枸橼酸舒芬太尼

1278　枸橼酸舒芬太尼注射液

1279　枸橼酸锌

1280　枸橼酸锌片

1281　柳氮磺吡啶

1282　柳氮磺吡啶肠溶片

1283　柳氮磺吡啶栓

1284　氟马西尼

1285　氟马西尼注射液

1286　氟比洛芬

1287　氟他胺

1288　氟他胺片

1289　氟尿嘧啶

1290　氟尿嘧啶乳膏

1291　氟尿嘧啶注射液

1292　氟罗沙星

1293　氟罗沙星片

1294　氟罗沙星胶囊

1295　氟哌利多

1296　氟哌利多注射液

1297　氟哌啶醇

1298　氟哌啶醇片

1299　氟哌啶醇注射液

1300　氟胞嘧啶

1301　氟胞嘧啶片

1302　氟胞嘧啶注射液

1303　氟康唑

1304　氟康唑片

1305 氟康唑注射液

1306 氟康唑胶囊

1307 氟康唑氯化钠注射液

1308 氟烷

1309 氟氯西林钠

1310 氟氯西林钠胶囊

1311 氢化可的松

1312 氢化可的松片

1313 氢化可的松乳膏

1314 氢化可的松注射液

1315 氢化可的松琥珀酸钠

1316 氢氧化铝

1317 氢氧化铝片

1318 氢氧化铝凝胶

1319 氢氯噻嗪

1320 氢氯噻嗪片

1321 氢溴酸山莨菪碱

1322 氢溴酸山莨菪碱片

1323 氢溴酸山莨菪碱注射液

1324 氢溴酸东莨菪碱

1325 氢溴酸东莨菪碱片

1326 氢溴酸东莨菪碱注射液

1327 氢溴酸加兰他敏

1328 氢溴酸加兰他敏片

1329 氢溴酸加兰他敏注射液

1330 氢溴酸右美沙芬

1331 氢溴酸右美沙芬口服溶液

1332 氢溴酸右美沙芬片

1333 氢溴酸右美沙芬胶囊

1334 氢溴酸右美沙芬缓释片

1335 氢溴酸右美沙芬颗粒

1336 氢溴酸后马托品

1337 氢溴酸西酞普兰

1338 氢溴酸西酞普兰片

1339 氢溴酸烯丙吗啡

1340 氢溴酸烯丙吗啡注射液

1341 洛伐他汀

1342 洛伐他汀片

1343 洛伐他汀胶囊

1344 洛伐他汀颗粒

1345 洛莫司汀

1346 洛莫司汀胶囊

1347 浓戊二醛溶液

1348 浓过氧化氢溶液

1349 浓氯化钠注射液

1350 玻璃酸酶

1351　癸氟奋乃静

1352　癸氟奋乃静注射液

1353　秋水仙碱

1354　秋水仙碱片

1355　绒促性素

1356　美罗培南

1357　美洛西林钠

1358　美洛昔康

1359　美洛昔康分散片

1360　美洛昔康片

1361　美洛昔康胶囊

1362　胃蛋白酶

1363　胃蛋白酶片

1364　胃蛋白酶颗粒

1365　胆茶碱

1366　胆茶碱片

1367　胆影葡胺注射液

1368　胆影酸

1369　胞磷胆碱钠

1370　胞磷胆碱钠片

1371　胞磷胆碱钠注射液

1372　胞磷胆碱钠氯化钠注射液

1373　胞磷胆碱钠葡萄糖注射液

1374 茴拉西坦

1375 茴拉西坦胶囊

1376 茶苯海明

1377 茶苯海明片

1378 茶碱

1379 茶碱缓释片

1380 茶碱缓释胶囊

1381 草乌甲素

1382 草乌甲素口服溶液

1383 草乌甲素片

1384 荧光素钠

1385 荧光素钠注射液

1386 药用炭

1387 药用炭片

1388 药用炭胶囊

1389 重组人生长激素

1390 重组人胰岛素

1391 重组人胰岛素注射液

1392 重质碳酸镁

1393 重酒石酸去甲肾上腺素

1394 重酒石酸去甲肾上腺素注射液

1395 重酒石酸间羟胺

1396 重酒石酸间羟胺注射液

1397 顺铂

1398 倍他米松

1399 倍他米松片

1400 倍他米松乳膏

1401 倍他米松磷酸钠

1402 倍他米松磷酸钠注射液

1403 恩曲他滨

1404 恩曲他滨胶囊

1405 恩氟烷

1406 核黄素磷酸钠

1407 核黄素磷酸钠注射液

1408 格列本脲

1409 格列本脲片

1410 格列齐特

1411 格列齐特片（II）

1412 格列吡嗪

1413 格列吡嗪片

1414 格列吡嗪胶囊

1415 格列吡嗪缓释胶囊

1416 格列美脲

1417 格列美脲片

1418 格列美脲胶囊

1419 格列喹酮

1420　格列喹酮片

1421　格隆溴铵

1422　格隆溴铵片

1423　桂利嗪

1424　桂利嗪片

1425　桂利嗪胶囊

1426　氧

1427　氧化亚氮

1428　氧化淀粉

1429　氧化锌

1430　氧化锌软膏

1431　氧化镁

1432　氧氟沙星

1433　氧氟沙星片

1434　氧氟沙星胶囊

1435　氧氟沙星眼膏

1436　氧氟沙星氯化钠注射液

1437　氧氟沙星滴耳液

1438　氧氟沙星滴眼液

1439　氧烯洛尔

1440　氧烯洛尔片

1441　氨力农

1442　氨甲环酸

1443　氨甲环酸片

1444　氨甲环酸注射液

1445　氨甲环酸胶囊

1446　氨曲南

1447　氨苄西林

1448　氨苄西林丙磺舒颗粒

1449　氨苄西林钠

1450　氨苯砜

1451　氨苯砜片

1452　氨苯蝶啶

1453　氨苯蝶啶片

1454　氨茶碱

1455　氨茶碱片

1456　氨茶碱注射液

1457　氨茶碱氯化钠注射液

1458　氨茶碱缓释片

1459　氨基己酸

1460　氨基己酸注射液

1461　氨酚待因片（Ⅰ）

1462　氨酚待因片（Ⅱ）

1463　氨鲁米特

1464　氨鲁米特片

1465　消旋山莨菪碱

1466　消旋山莨菪碱片

1467　消旋卡多曲

1468　消旋卡多曲颗粒

1469　烟酰胺

1470　烟酰胺片

1471　烟酰胺注射液

1472　烟酸

1473　烟酸片

1474　烟酸占替诺

1475　烟酸占替诺注射液

1476　烟酸占替诺氯化钠注射液

1477　烟酸注射液

1478　特非那定

1479　特非那定片

1480　盐酸乙胺丁醇

1481　盐酸乙胺丁醇片

1482　盐酸乙胺丁醇胶囊

1483　盐酸丁丙诺啡

1484　盐酸丁丙诺啡舌下片

1485　盐酸丁丙诺啡注射液

1486　盐酸丁卡因

1487　盐酸丁螺环酮

1488　盐酸丁螺环酮片

1489　盐酸二甲双胍

1490　盐酸二甲双胍片

1491　盐酸二甲双胍肠溶片

1492　盐酸二甲双胍肠溶胶囊

1493　盐酸二甲双胍胶囊

1494　盐酸二甲弗林

1495　盐酸二氢埃托啡

1496　盐酸二氢埃托啡舌下片

1497　盐酸二氧丙嗪

1498　盐酸二氧丙嗪片

1499　盐酸万古霉素

1500　盐酸三氟拉嗪

1501　盐酸三氟拉嗪片

1502　盐酸土霉素

1503　盐酸土霉素片

1504　盐酸大观霉素

1505　盐酸小檗碱

1506　盐酸小檗碱片

1507　盐酸小檗碱胶囊

1508　盐酸川芎嗪

1509　盐酸川芎嗪注射液

1510　盐酸马普替林

1511　盐酸马普替林片

1512 盐酸文拉法辛

1513 盐酸文拉法辛胶囊

1514 盐酸文拉法辛缓释片

1515 盐酸丙卡巴肼

1516 盐酸丙卡巴肼肠溶片

1517 盐酸丙卡特罗

1518 盐酸丙卡特罗片

1519 盐酸丙卡特罗胶囊

1520 盐酸丙米嗪

1521 盐酸丙米嗪片

1522 盐酸丙帕他莫

1523 盐酸半胱氨酸

1524 盐酸卡替洛尔

1525 盐酸卡替洛尔滴眼液

1526 盐酸去甲万古霉素

1527 盐酸去氧肾上腺素

1528 盐酸去氧肾上腺素注射液

1529 盐酸去氯羟嗪

1530 盐酸去氯羟嗪片

1531 盐酸可乐定

1532 盐酸可乐定片

1533 盐酸可乐定注射液

1534 盐酸可乐定滴眼液

1535　盐酸可卡因

1536　盐酸司来吉兰

1537　盐酸司来吉兰片

1538　盐酸四环素

1539　盐酸四环素片

1540　盐酸四环素胶囊

1541　盐酸头孢他美酯

1542　盐酸头孢他美酯干混悬剂

1543　盐酸头孢他美酯片

1544　盐酸头孢他美酯胶囊

1545　盐酸头孢甲肟

1546　盐酸头孢吡肟

1547　盐酸尼卡地平

1548　盐酸尼卡地平片

1549　盐酸尼卡地平注射液

1550　盐酸尼卡地平葡萄糖注射液

1551　盐酸左氧氟沙星

1552　盐酸左氧氟沙星片

1553　盐酸左氧氟沙星胶囊

1554　盐酸左旋咪唑

1555　盐酸左旋咪唑片

1556　盐酸左旋咪唑肠溶片

1557　盐酸左旋咪唑颗粒

1558　盐酸左旋咪唑糖浆

1559　盐酸布比卡因

1560　盐酸布比卡因注射液

1561　盐酸布桂嗪

1562　盐酸布桂嗪片

1563　盐酸布桂嗪注射液

1564　盐酸布替萘芬

1565　盐酸布替萘芬乳膏

1566　盐酸布替萘芬喷雾剂

1567　盐酸布替萘芬凝胶

1568　盐酸平阳霉素

1569　盐酸甲氧明

1570　盐酸甲氧明注射液

1571　盐酸甲氧氯普胺注射液

1572　盐酸甲氯芬酯

1573　盐酸甲氯芬酯胶囊

1574　盐酸艾司洛尔

1575　盐酸伊托必利

1576　盐酸伊托必利分散片

1577　盐酸伊托必利片

1578　盐酸伊托必利胶囊

1579　盐酸伐昔洛韦

1580　盐酸伐昔洛韦片

1581 盐酸伐昔洛韦胶囊

1582 盐酸伪麻黄碱

1583 盐酸吉西他滨

1584 盐酸吗啡

1585 盐酸吗啡片

1586 盐酸吗啡注射液

1587 盐酸吗啡缓释片

1588 盐酸地匹福林

1589 盐酸地匹福林滴眼液

1590 盐酸地尔硫?

1591 盐酸地尔硫?片

1592 盐酸地尔硫?缓释片

1593 盐酸地芬尼多

1594 盐酸地芬尼多片

1595 盐酸地芬诺酯

1596 盐酸多巴胺

1597 盐酸多巴胺注射液

1598 盐酸多巴酚丁胺

1599 盐酸多巴酚丁胺注射液

1600 盐酸多西环素

1601 盐酸多西环素片

1602 盐酸多西环素胶囊

1603 盐酸多沙普仑

1604　盐酸多沙普仑注射液

1605　盐酸多奈哌齐

1606　盐酸多柔比星

1607　盐酸多塞平

1608　盐酸多塞平片

1609　盐酸安他唑啉

1610　盐酸安他唑啉片

1611盐酸安非他酮

1612　盐酸安非他酮片

1613　盐酸安非他酮缓释片

1614　盐酸异丙肾上腺素

1615　盐酸异丙肾上腺素注射液

1616　盐酸异丙嗪

1617　盐酸异丙嗪片

1618　盐酸异丙嗪注射液

1619　盐酸托烷司琼

1620　盐酸托烷司琼注射液

1621　盐酸曲马多

1622　盐酸曲马多分散片

1623　盐酸曲马多片

1624　盐酸曲马多注射液

1625　盐酸曲马多栓

1626　盐酸曲马多胶囊

1627 盐酸曲马多缓释片

1628 盐酸曲马多缓释胶囊

1629 盐酸曲美他嗪

1630 盐酸曲美他嗪片

1631 盐酸曲美他嗪胶囊

1632 盐酸米多君

1633 盐酸米多君片

1634 盐酸米托蒽醌

1635 盐酸米托蒽醌氯化钠注射液

1636 盐酸米诺环素

1637 盐酸米诺环素片

1638 盐酸米诺环素胶囊

1639 盐酸西替利嗪

1640 盐酸西替利嗪口服溶液

1641 盐酸西替利嗪片

1642 盐酸西替利嗪胶囊

1643 盐酸西替利嗪滴剂

1644 盐酸齐拉西酮

1645 盐酸齐拉西酮片

1646 盐酸齐拉西酮胶囊

1647 盐酸克仑特罗

1648 盐酸克仑特罗栓

1649 盐酸克林霉素

1650　盐酸克林霉素胶囊

1651　盐酸克林霉素棕榈酸酯

1652　盐酸克林霉素棕榈酸酯干混悬剂

1653　盐酸克林霉素棕榈酸酯颗粒

1654　盐酸利多卡因

1655　盐酸利多卡因注射液

1656　盐酸利多卡因注射液（溶剂用）

1657　盐酸利多卡因胶浆（Ⅰ）

1658　盐酸利多卡因凝胶

1659　盐酸吡硫醇

1660　盐酸吡硫醇片

1661　盐酸吡硫醇注射液

1662　盐酸吡硫醇胶囊

1663　盐酸妥卡尼

1664　盐酸妥卡尼片

1665　盐酸妥卡尼胶囊

1666　盐酸妥拉唑林

1667　盐酸妥拉唑林片

1668　盐酸妥拉唑林注射液

1669　盐酸纳洛酮

1670　盐酸纳洛酮注射液

1671　盐酸纳美芬

1672　盐酸纳美芬注射液

1673　盐酸苄丝肼

1674　盐酸阿扑吗啡

1675　盐酸阿扑吗啡注射液

1676　盐酸阿米洛利

1677　盐酸阿米洛利片

1678　盐酸阿米替林

1679　盐酸阿米替林片

1680　盐酸阿莫地喹片

1681　盐酸阿普林定

1682　盐酸阿普林定片

1683　盐酸阿糖胞苷

1684　盐酸依米丁

1685　盐酸依米丁注射液

1686　盐酸奈福泮

1687　盐酸奈福泮片

1688　盐酸奈福泮注射液

1689　盐酸帕罗西汀

1690　盐酸帕罗西汀片

1691　盐酸昂丹司琼

1692　盐酸昂丹司琼片

1693　盐酸昂丹司琼注射液

1694　盐酸林可霉素

1695　盐酸林可霉素片

1696　盐酸林可霉素注射液

1697　盐酸林可霉素胶囊

1698　盐酸林可霉素滴耳液

1699　盐酸林可霉素滴眼液

1700　盐酸法舒地尔

1701　盐酸法舒地尔注射液

1702　盐酸环丙沙星

1703　盐酸环丙沙星片

1704　盐酸环丙沙星胶囊

1705　盐酸环丙沙星滴眼液

1706　盐酸组氨酸

1707　盐酸罗哌卡因

1708　盐酸罗哌卡因注射液

1709　盐酸罗通定

1710　盐酸罗通定片

1711盐酸肼屈嗪

1712　盐酸肼屈嗪片

1713　盐酸肾上腺素注射液

1714　盐酸舍曲林

1715　盐酸舍曲林片

1716　盐酸舍曲林胶囊

1717　盐酸苯乙双胍

1718　盐酸苯乙双胍片

1719　盐酸苯海拉明

1720　盐酸苯海拉明片

1721　盐酸苯海拉明注射液

1722　盐酸苯海索

1723　盐酸苯海索片

1724　盐酸表柔比星

1725　盐酸金刚乙胺

1726　盐酸金刚乙胺片

1727　盐酸金刚乙胺颗粒

1728　盐酸金刚烷胺

1729　盐酸金刚烷胺片

1730　盐酸金刚烷胺胶囊

1731　盐酸金刚烷胺颗粒

1732　盐酸金刚烷胺糖浆

1733　盐酸金霉素

1734　盐酸金霉素软膏

1735　盐酸金霉素眼膏

1736　盐酸非那吡啶

1737　盐酸非那吡啶片

1738　盐酸哌甲酯

1739　盐酸哌甲酯片

1740　盐酸哌唑嗪

1741　盐酸哌唑嗪片

1742 盐酸哌替啶

1743 盐酸哌替啶片

1744 盐酸哌替啶注射液

1745 盐酸柔红霉素

1746 盐酸氟西汀

1747 盐酸氟西汀片

1748 盐酸氟西汀胶囊

1749 盐酸氟西泮

1750 盐酸氟西泮胶囊

1751 盐酸氟奋乃静

1752 盐酸氟奋乃静片

1753 盐酸氟奋乃静注射液

1754 盐酸氟桂利嗪

1755 盐酸氟桂利嗪分散片

1756 盐酸氟桂利嗪片

1757 盐酸氟桂利嗪胶囊

1758 盐酸洛贝林

1759 盐酸洛非西定

1760 盐酸洛非西定片

1761 盐酸洛哌丁胺

1762 盐酸洛哌丁胺胶囊

1763 盐酸洛美沙星

1764 盐酸洛美沙星片

1765　盐酸洛美沙星胶囊

1766　盐酸美他环素

1767　盐酸美他环素片

1768　盐酸美他环素胶囊

1769　盐酸美西律

1770　盐酸美西律片

1771　盐酸美西律注射液

1772　盐酸美西律胶囊

1773　盐酸美克洛嗪

1774　盐酸美克洛嗪片

1775　盐酸美沙酮

1776　盐酸美沙酮口服溶液

1777　盐酸美沙酮片

1778　盐酸美沙酮注射液

1779　盐酸倍他司汀

1780　盐酸倍他司汀片

1781　盐酸格拉司琼

1782　盐酸格拉司琼片

1783　盐酸格拉司琼注射液

1784　盐酸氨溴索

1785　盐酸氨溴索口服溶液

1786　盐酸氨溴索片

1787　盐酸氨溴索注射液

1788　盐酸氨溴索胶囊

1789　盐酸氨溴索缓释胶囊

1790　盐酸氨溴索糖浆

1791　盐酸消旋山莨菪碱注射液

1792　盐酸特比萘芬

1793　盐酸特比萘芬片

1794　盐酸特比萘芬乳膏

1795　盐酸特拉唑嗪

1796　盐酸特拉唑嗪片

1797　盐酸特拉唑嗪胶囊

1798　盐酸班布特罗

1799　盐酸班布特罗片

1800　盐酸索他洛尔

1801　盐酸索他洛尔片

1802　盐酸胺碘酮

1803　盐酸胺碘酮片

1804　盐酸胺碘酮注射液

1805　盐酸胺碘酮胶囊

1806　盐酸莫雷西嗪

1807　盐酸莫雷西嗪片

1808　盐酸维拉帕米

1809　盐酸维拉帕米片

1810　盐酸维拉帕米注射液

1811 盐酸维拉帕米缓释片

1812 盐酸羟考酮

1813 盐酸羟考酮片

1814 盐酸萘甲唑啉

1815 盐酸萘甲唑啉滴眼液

1816 盐酸萘甲唑啉滴鼻液

1817 盐酸萘替芬

1818 盐酸萘替芬软膏

1819 盐酸萘替芬溶液

1820 盐酸酚苄明

1821 盐酸酚苄明片

1822 盐酸酚苄明注射液

1823 盐酸麻黄碱

1824 盐酸麻黄碱注射液

1825 盐酸麻黄碱滴鼻液

1826 盐酸黄酮哌酯

1827 盐酸黄酮哌酯片

1828 盐酸黄酮哌酯胶囊

1829 盐酸喹那普利

1830 盐酸奥昔布宁

1831 盐酸奥昔布宁片

1832 盐酸普罗帕酮

1833 盐酸普罗帕酮片

1834　盐酸普罗帕酮注射液

1835　盐酸普罗帕酮胶囊

1836　盐酸普萘洛尔

1837　盐酸普萘洛尔片

1838　盐酸普萘洛尔注射液

1839　盐酸普鲁卡因

1840　盐酸普鲁卡因注射液

1841　盐酸普鲁卡因胺

1842　盐酸普鲁卡因胺片

1843　盐酸普鲁卡因胺注射液

1844　盐酸替扎尼定

1845　盐酸替扎尼定片

1846　盐酸氮芥

1847　盐酸氮芥注射液

1848　盐酸氮卓斯汀

1849　盐酸氮卓斯汀片

1850　盐酸氮卓斯汀鼻喷雾剂

1851　盐酸氯丙那林

1852　盐酸氯丙那林片

1853　盐酸氯丙嗪

1854　盐酸氯丙嗪片

1855　盐酸氯丙嗪注射液

1856　盐酸氯米帕明

1857　盐酸氯米帕明片

1858　盐酸氯米帕明注射液

1859　盐酸氯胺酮

1860　盐酸氯胺酮注射液

1861　盐酸硫必利

1862　盐酸硫必利注射液

1863　盐酸硫利达嗪

1864　盐酸硫利达嗪片

1865　盐酸溴己新

1866　盐酸溴己新片

1867　盐酸瑞芬太尼

1868　盐酸赖氨酸

1869　盐酸雷尼替丁

1870　盐酸雷尼替丁片

1871　盐酸雷尼替丁泡腾颗粒

1872　盐酸雷尼替丁注射液

1873　盐酸雷尼替丁胶囊

1874　盐酸精氨酸

1875　盐酸精氨酸片

1876　盐酸精氨酸注射液

1877　盐酸罂粟碱

1878　盐酸罂粟碱片

1879　盐酸罂粟碱注射液

1880　盐酸赛庚啶

1881　盐酸赛庚啶片

1882　盐酸赛洛唑啉

1883　盐酸赛洛唑啉滴鼻液

1884　盐酸噻氯匹定

1885　盐酸噻氯匹定片

1886　盐酸噻氯匹定胶囊

1887　胰岛素

1888　胰岛素注射液

1889　胰蛋白酶

1890　胰酶

1891　胰酶肠溶片

1892　胰酶肠溶胶囊

1893　胰激肽原酶

1894　胰激肽原酶肠溶片

1895　胱氨酸

1896　胱氨酸片

1897　胶体果胶铋

1898　胶体果胶铋胶囊

1899　胸腺五肽

1900　胸腺五肽注射液

1901　胸腺法新

1902　诺氟沙星

1903　诺氟沙星片

1904　诺氟沙星乳膏

1905　诺氟沙星软膏

1906　诺氟沙星胶囊

1907　诺氟沙星滴眼液

1908　酒石酸双氢可待因

1909　酒石酸双氢可待因片

1910　酒石酸长春瑞滨

1911　酒石酸长春瑞滨注射液

1912　酒石酸布托啡诺

1913　酒石酸布托啡诺注射液

1914　酒石酸麦角胺

1915　酒石酸美托洛尔

1916　酒石酸美托洛尔片

1917　酒石酸美托洛尔注射液

1918　酒石酸美托洛尔胶囊

1919　酒石酸美托洛尔缓释片

1920　酒石酸唑吡坦

1921　酒石酸唑吡坦片

1922　高三尖杉酯碱

1923　高三尖杉酯碱注射液

1924　高锰酸钾

1925　高锰酸钾外用片

1926　培哚普利叔丁胺

1927　培哚普利叔丁胺片

1928　液状石蜡

1929　维A酸

1930　维A酸片

1931　维A酸乳膏

1932　维生素A

1933　维生素AD软胶囊

1934　维生素AD滴剂

1935　维生素A软胶囊

1936　维生素Ｂ1

1937　维生素Ｂ12

1938　维生素Ｂ12注射液

1939　维生素B12滴眼液

1940　维生素Ｂ1片

1941　维生素Ｂ1注射液

1942　维生素Ｂ2

1943　维生素Ｂ2片

1944　维生素Ｂ2注射液

1945　维生素Ｂ6

1946　维生素Ｂ6片

1947　维生素Ｂ6注射液

1948　维生素Ｃ

1949　维生素 C 片

1950　维生素 C 泡腾片

1951　维生素C泡腾颗粒

1952　维生素 C 注射液

1953　维生素C钙

1954　维生素C钠

1955　维生素 C 颗粒

1956　维生素 D 2

1957　维生素 D 2注射液

1958　维生素 D 2软胶囊

1959　维生素 D 3

1960　维生素 D 3注射液

1961　维生素E

1962　维生素 E 片

1963　维生素 E 注射液

1964　维生素E软胶囊

1965　维生素 E 粉

1966　维生素 K 1

1967　维生素 K 1注射液

1968　羟丁酸钠

1969　羟丁酸钠注射液

1970　羟甲香豆素

1971　羟甲香豆素片

1972　羟甲香豆素胶囊

1973　羟苯磺酸钙

1974　羟苯磺酸钙胶囊

1975　羟基脲

1976　羟基脲片

1977　脯氨酸

1978　萘丁美酮

1979　萘丁美酮片

1980　萘丁美酮胶囊

1981　萘哌地尔

1982　萘哌地尔片

1983　萘敏维滴眼液

1984　萘普生

1985　萘普生片

1986　萘普生钠

1987　萘普生钠片

1988　萘普生栓

1989　萘普生胶囊

1990　萘普生颗粒

1991　萘普待因片

1992　萘磺酸右丙氧芬

1993　萝巴新

1994　辅酶Q10

1995　辅酶Q10片

1996　辅酶Q10注射液

1997　辅酶Q10软胶囊

1998　辅酶Q10胶囊

1999　酚咖片

2000　酚酞

2001　酚酞片

2002　酞丁安

2003　酞丁安乳膏

2004　酞丁安搽剂

2005　酞丁安滴眼液

2006　铝酸铋

2007　铝镁司片

2008　麻醉乙醚

2009　黄体酮

2010　黄体酮注射液

2011　喷雾用乙酰半胱氨酸

2012　奥扎格雷

2013　奥扎格雷钠

2014　奥卡西平

2015　奥卡西平片

2016　奥沙西泮

2017　奥沙西泮片

2018　奥沙利铂

2019　奥沙普秦

2020　奥沙普秦肠溶片

2021　奥沙普秦肠溶胶囊

2022　奥美拉唑

2023　奥美拉唑肠溶片

2024　奥美拉唑肠溶胶囊

2025　奥美拉唑钠

2026　奥美拉唑钠肠溶片

2027　奥美拉唑镁肠溶片

2028　奥氮平

2029　奥氮平片

2030　奥硝唑

2031　奥硝唑片

2032　奥硝唑阴道泡腾片

2033　奥硝唑阴道栓

2034　奥硝唑注射液

2035　奥硝唑胶囊

2036　富马酸比索洛尔

2037　富马酸比索洛尔片

2038　富马酸比索洛尔胶囊

2039　富马酸亚铁

2040　富马酸亚铁片

2041 富马酸亚铁咀嚼片

2042 富马酸亚铁胶囊

2043 富马酸亚铁颗粒

2044 富马酸喹硫平

2045 富马酸喹硫平片

2046 富马酸氯马斯汀

2047 富马酸氯马斯汀干混悬剂

2048 富马酸氯马斯汀片

2049 富马酸福莫特罗

2050 富马酸福莫特罗片

2051 富马酸酮替芬

2052 富马酸酮替芬口服溶液

2053 富马酸酮替芬片

2054 富马酸酮替芬胶囊

2055 富马酸酮替芬滴眼液

2056 富马酸酮替芬滴鼻液

2057 巯嘌呤

2058 巯嘌呤片

2059 普伐他汀钠

2060 普伐他汀钠片

2061 普伐他汀钠胶囊

2062 普罗布考

2063 普罗布考片

2064　普罗碘铵

2065　普罗碘铵注射液

2066　普鲁卡因青霉素

2067　替加氟

2068　替加氟片

2069　替加氟注射液

2070　替加氟胶囊

2071　替米沙坦

2072　替考拉宁

2073　替莫唑胺

2074　替莫唑胺胶囊

2075　替硝唑

2076　替硝唑片

2077　替硝唑阴道片

2078　替硝唑阴道泡腾片

2079　替硝唑含片

2080　替硝唑栓

2081　替硝唑胶囊

2082　替硝唑氯化钠注射液

2083　替硝唑葡萄糖注射液

2084　棓丙酯

2085　棕榈氯霉素

2086　棕榈氯霉素（Ｂ型）片

2087　棕榈氯霉素（Ｂ型）颗粒

2088　棕榈氯霉素混悬液

2089　氯化钙

2090　氯化钙注射液

2091　氯化钠

2092　氯化钠注射液

2093　氯化钾

2094　氯化钾片

2095　氯化钾注射液

2096　氯化钾氯化钠注射液

2097　氯化钾缓释片

2098　氯化钾葡萄糖注射液

2099　氯化铵

2100　氯化铵片

2101　氯化琥珀胆碱

2102　氯化琥珀胆碱注射液

2103　氯化筒箭毒碱

2104　氯化筒箭毒碱注射液

2105　氯贝丁酯

2106　氯贝丁酯胶囊

2107　氯芬待因片

2108　氯法齐明

2109　氯法齐明软胶囊

2110　氯唑西林钠

2111 氯唑西林钠胶囊

2112　氯唑西林钠颗粒

2113　氯诺昔康

2114　氯诺昔康片

2115　氯烯雌醚

2116　氯烯雌醚滴丸

2117　氯普噻吨

2118　氯普噻吨片

2119　氯普噻吨注射液

2120　氯氮?

2121　氯氮?片

2122　氯氮平

2123　氯氮平片

2124　氯硝西泮

2125　氯硝西泮片

2126　氯硝西泮注射液

2127　氯硝柳胺

2128　氯硝柳胺片

2129　氯碘羟喹

2130　氯碘羟喹乳膏

2131　氯雷他定

2132　氯雷他定片

2133 氯雷他定胶囊

2134 氯雷他定颗粒

2135 氯霉素

2136 氯霉素片

2137 氯霉素胶囊

2138 氯霉素眼膏

2139 氯霉素滴耳液

2140 氯霉素滴眼液

2141 氯噻酮

2142 氯噻酮片

2143 氯磺丙脲

2144 氯磺丙脲片

2145 氯膦酸二钠

2146 氯膦酸二钠注射液

2147 氯膦酸二钠胶囊

2148 琥乙红霉素

2149 琥乙红霉素分散片

2150 琥乙红霉素片

2151 琥乙红霉素胶囊

2152 琥乙红霉素颗粒

2153 琥珀氯霉素

2154 琥珀酸舒马普坦片

2155 硝西泮

2156　硝西泮片

2157　硝苯地平

2158　硝苯地平片

2159　硝苯地平软胶囊

2160　硝苯地平胶囊

2161　硝普钠

2162　硝酸毛果芸香碱

2163　硝酸毛果芸香碱滴眼液

2164　硝酸甘油气雾剂

2165　硝酸甘油片

2166　硝酸甘油注射液

2167　硝酸甘油溶液

2168　硝酸异山梨酯

2169　硝酸异山梨酯片

2170　硝酸异山梨酯乳膏

2171　硝酸异山梨酯注射液

2172　硝酸异山梨酯喷雾剂

2173　硝酸异山梨酯缓释胶囊

2174　硝酸异山梨酯葡萄糖注射液

2175　硝酸咪康唑

2176　硝酸咪康唑阴道片

2177　硝酸咪康唑阴道泡腾片

2178　硝酸咪康唑阴道软胶囊

2179　硝酸咪康唑乳膏

2180　硝酸咪康唑栓

2181　硝酸咪康唑胶囊

2182　硝酸咪康唑搽剂

2183　硝酸益康唑

2184　硝酸益康唑乳膏

2185　硝酸益康唑栓

2186　硝酸益康唑喷雾剂

2187　硝酸益康唑溶液

2188　硝酸硫胺

2189　硫代硫酸钠

2190　硫代硫酸钠注射液

2191　硫鸟嘌呤

2192　硫鸟嘌呤片

2193　硫软膏

2194　硫唑嘌呤

2195　硫唑嘌呤片

2196　硫酸小诺霉素

2197　硫酸小诺霉素口服溶液

2198　硫酸小诺霉素片

2199　硫酸小诺霉素注射液

2200　硫酸双肼屈嗪

2201　硫酸双肼屈嗪片

2202　硫酸巴龙霉素

2203　硫酸巴龙霉素片

2204　硫酸长春地辛

2205　硫酸长春新碱

2206　硫酸长春碱

2207　硫酸卡那霉素

2208　硫酸卡那霉素注射液

2209　硫酸卡那霉素滴眼液

2210　硫酸亚铁

2211　硫酸亚铁片

2212　硫酸亚铁缓释片

2213　硫酸吗啡

2214　硫酸吗啡注射液

2215　硫酸吗啡缓释片

2216　硫酸多黏菌素B

2217　硫酸庆大霉素

2218　硫酸庆大霉素片

2219　硫酸庆大霉素注射液

2220　硫酸庆大霉素缓释片

2221　硫酸庆大霉素滴眼液

2222　硫酸庆大霉素颗粒

2223　硫酸异帕米星

2224　硫酸异帕米星注射液

2225　硫酸西索米星

2226　硫酸西索米星注射液

2227　硫酸妥布霉素注射液

2228　硫酸沙丁胺醇

2229　硫酸沙丁胺醇片

2230　硫酸沙丁胺醇吸入气雾剂

2231　硫酸沙丁胺醇吸入粉雾剂

2232　硫酸沙丁胺醇注射液

2233　硫酸沙丁胺醇胶囊

2234　硫酸沙丁胺醇缓释片

2235　硫酸沙丁胺醇缓释胶囊

2236　硫酸阿托品

2237　硫酸阿托品片

2238　硫酸阿托品注射液

2239　硫酸阿托品眼膏

2240　硫酸阿米卡星

2241　硫酸阿米卡星注射液

2242　硫酸依替米星

2243　硫酸依替米星注射液

2244　硫酸卷曲霉素

2245　硫酸奈替米星

2246　硫酸奈替米星注射液

2247　硫酸罗通定注射液

2248　硫酸茚地那韦胶囊

2249　硫酸软骨素钠

2250　硫酸软骨素钠片

2251　硫酸软骨素钠胶囊

2252　硫酸鱼精蛋白

2253　硫酸鱼精蛋白注射液

2254　硫酸奎宁

2255　硫酸奎宁片

2256　硫酸奎尼丁

2257　硫酸奎尼丁片

2258　硫酸氢氯吡格雷

2259　硫酸氢氯吡格雷片

2260　硫酸胍乙啶

2261　硫酸胍乙啶片

2262　硫酸钡（Ⅰ型）

2263　硫酸钡（Ⅰ型）干混悬剂

2264　硫酸钡（Ⅱ型）

2265　硫酸钡（Ⅱ型）干混悬剂

2266　硫酸核糖霉素

2267　硫酸特布他林

2268　硫酸特布他林片

2269　硫酸特布他林吸入气雾剂

2270　硫酸普拉睾酮钠

2271　硫酸链霉素

2272　硫酸锌

2273　硫酸锌口服溶液

2274　硫酸锌片

2275　硫酸锌颗粒

2276　硫酸新霉素

2277　硫酸新霉素片

2278　硫酸新霉素滴眼液

2279　硫酸镁

2280　硫酸镁注射液

2281　硫酸黏菌素

2282　硫酸黏菌素片

2283　硫糖铝

2284　硫糖铝口服混悬液

2285　硫糖铝分散片

2286　硫糖铝咀嚼片

2287　硫糖铝胶囊

2288　硬脂酸红霉素

2289　硬脂酸红霉素片

2290　硬脂酸红霉素胶囊

2291　硬脂酸红霉素颗粒

2292　稀戊二醛溶液

2293　稀氨溶液

2294　稀葡萄糖酸氯己定溶液

2295　紫杉醇

2296　紫杉醇注射液

2297　联苯双酯

2298　联苯双酯滴丸

2299　联苯苄唑

2300　联苯苄唑乳膏

2301　联苯苄唑栓

2302　联苯苄唑溶液

2303　联磺甲氧苄啶片

2304　舒巴坦钠

2305　舒必利

2306　舒必利片

2307　舒林酸

2308　舒林酸片

2309　葛根素

2310　葛根素注射液

2311　葡甲胺

2312　葡萄糖

2313　葡萄糖注射液

2314　葡萄糖粉剂

2315　葡萄糖氯化钠注射液

2316　葡萄糖酸亚铁

2317 葡萄糖酸亚铁片

2318 葡萄糖酸亚铁胶囊

2319 葡萄糖酸亚铁糖浆

2320 葡萄糖酸钙

2321 葡萄糖酸钙口服溶液

2322 葡萄糖酸钙片

2323 葡萄糖酸钙含片

2324 葡萄糖酸钙注射液

2325 葡萄糖酸钙颗粒

2326 葡萄糖酸氯己定含漱液

2327 葡萄糖酸氯己定溶液

2328 葡萄糖酸锌

2329 葡萄糖酸锌口服溶液

2330 葡萄糖酸锌片

2331 葡萄糖酸锌颗粒

2332 葡萄糖酸锑钠

2333 葡萄糖酸锑钠注射液

2334 塞克硝唑

2335 塞克硝唑片

2336 塞克硝唑胶囊

2337 塞替派

2338 塞替派注射液

2339 溴丙胺太林

2340　溴丙胺太林片

2341　溴吡斯的明

2342　溴吡斯的明片

2343　溴新斯的明

2344　溴新斯的明片

2345　瑞格列奈

2346　瑞格列奈片

2347　硼砂

2348　硼酸

2349　硼酸冲洗液

2350　硼酸软膏

2351　碘

2352　碘化油

2353　碘化油注射液

2354　碘化油软胶囊

2355　碘化钠

2356　碘化钾

2357　碘化钾片

2358　碘他拉葡胺注射液

2359　碘他拉酸

2360　碘甘油

2361　碘佛醇

2362　碘佛醇注射液

2363 碘苯酯

2364 碘苯酯注射液

2365 碘苷

2366 碘苷滴眼液

2367 碘酊

2368 碘海醇

2369 碘海醇注射液

2370 碘番酸

2371 碘番酸片

2372 碘解磷定

2373 碘解磷定注射液

2374 碘酸钾

2375 碘酸钾片

2376 碘酸钾颗粒

2377 福尔可定

2378 福尔可定片

2379 羧甲司坦

2380 羧甲司坦口服溶液

2381 羧甲司坦片

2382 羧甲司坦颗粒

2383 羧苄西林钠

2384 腺苷钴胺

2385 腺苷钴胺片

2386　蒙脱石

2387　蒙脱石分散片

2388　蒙脱石散

2389　蒿甲醚

2390　蒿甲醚胶囊

2391　赖氨匹林

2392　赖诺普利

2393　赖诺普利片

2394　赖诺普利胶囊

2395　输血用枸橼酸钠注射液

2396　酪氨酸

2397　酮洛芬

2398　酮洛芬肠溶胶囊

2399　酮洛芬搽剂

2400　酮康唑

2401　酮康唑乳膏

2402　酮康唑洗剂

2403　雷贝拉唑钠

2404　雷贝拉唑钠肠溶片

2405　雷贝拉唑钠肠溶胶囊

2406　雷米普利

2407　雷米普利片

2408　熊去氧胆酸

2409 熊去氧胆酸片

2410 碱式碳酸铋

2411 碱式碳酸铋片

2412 碳酸利多卡因注射液

2413 碳酸氢钠

2414 碳酸氢钠片

2415 碳酸氢钠注射液

2416 碳酸钙

2417 碳酸钙咀嚼片

2418 碳酸钙颗粒

2419 碳酸锂

2420 碳酸锂片

2421 碳酸锂缓释片

2422 精氨酸

2423 精蛋白重组人胰岛素注射液

2424 精蛋白锌胰岛素注射液

2425 精蛋白锌胰岛素注射液（30R）

2426 缩宫素注射液

2427 罂粟果提取物

2428 罂粟果提取物粉

2429 聚维酮碘

2430 聚维酮碘乳膏

2431 聚维酮碘栓

2432　聚维酮碘溶液

2433　聚维酮碘凝胶

2434　雌二醇

2435　雌二醇缓释贴片

2436　鲑降钙素

2437　鲑降钙素注射液

2438　樟脑（天然）

2439　樟脑（合成）

2440　缬沙坦

2441　缬沙坦片

2442　缬沙坦胶囊

2443　缬氨酸

2444　醋氨己酸锌

2445　醋氨己酸锌胶囊

2446　醋氨苯砜

2447　醋氨苯砜注射液

2448　醋氯芬酸

2449　醋氯芬酸片

2450　醋氯芬酸胶囊

2451　醋酸丙氨瑞林

2452　醋酸去氧皮质酮

2453　醋酸去氨加压素

2454　醋酸去氨加压素注射液

2455 醋酸可的松

2456 醋酸可的松片

2457 醋酸可的松注射液

2458 醋酸甲地孕酮

2459 醋酸甲地孕酮分散片

2460 醋酸甲地孕酮片

2461 醋酸甲地孕酮胶囊

2462 醋酸甲羟孕酮

2463 醋酸甲羟孕酮分散片

2464 醋酸甲羟孕酮片

2465 醋酸甲羟孕酮胶囊

2466 醋酸甲羟孕酮混悬注射液

2467 醋酸甲萘氢醌

2468 醋酸甲萘氢醌片

2469 醋酸地塞米松

2470 醋酸地塞米松片

2471 醋酸地塞米松乳膏

2472 醋酸地塞米松注射液

2473 醋酸曲安奈德

2474 醋酸曲安奈德乳膏

2475 醋酸曲安奈德注射液

2476 醋酸曲普瑞林

2477 醋酸曲普瑞林注射液

2478　醋酸泼尼松

2479　醋酸泼尼松片

2480　醋酸泼尼松龙

2481　醋酸泼尼松龙片

2482　醋酸泼尼松龙乳膏

2483　醋酸泼尼松龙注射液

2484　醋酸泼尼松眼膏

2485　醋酸氟氢可的松

2486　醋酸氟氢可的松乳膏

2487　醋酸氟轻松

2488　醋酸氟轻松乳膏

2489　醋酸氢化可的松

2490　醋酸氢化可的松片

2491　醋酸氢化可的松乳膏

2492　醋酸氢化可的松注射液

2493　醋酸氢化可的松眼膏

2494　醋酸氢化可的松滴眼液

2495　醋酸奥曲肽

2496　醋酸奥曲肽注射液

2497　醋酸氯己定

2498　醋酸氯己定软膏

2499　醋酸氯地孕酮

2500　醋酸赖氨酸

2501　醋酸磺胺米隆

2502　凝血酶冻干粉

2503　噻苯唑

2504　噻苯唑片

2505　磺苄西林钠

2506　磺胺甲噁唑

2507　磺胺甲噁唑片

2508　磺胺多辛

2509　磺胺多辛片

2510　磺胺异噁唑

2511　磺胺异噁唑片

2512　磺胺嘧啶

2513　磺胺嘧啶片

2514　磺胺嘧啶软膏

2515　磺胺嘧啶钠

2516　磺胺嘧啶钠注射液

2517　磺胺嘧啶混悬液

2518　磺胺嘧啶眼膏

2519　磺胺嘧啶银

2520　磺胺嘧啶银乳膏

2521　磺胺嘧啶银软膏

2522　磺胺嘧啶锌

2523　磺胺嘧啶锌软膏

2524 磺胺醋酰钠

2525 磺胺醋酰钠滴眼液

2526 糖精钠

2527 薄荷麝香草酚搽剂

2528 磷酸二氢钠

2529 磷酸川芎嗪

2530 磷酸川芎嗪片

2531 磷酸川芎嗪胶囊

2532 磷酸丙吡胺

2533 磷酸丙吡胺片

2534 磷酸丙吡胺注射液

2535 磷酸可待因

2536 磷酸可待因片

2537 磷酸可待因注射液

2538 磷酸可待因糖浆

2539 磷酸肌酸钠

2540 磷酸伯氨喹

2541 磷酸伯氨喹片

2542 磷酸组胺

2543 磷酸组胺注射液

2544 磷酸苯丙哌林

2545 磷酸苯丙哌林口服溶液

2546 磷酸苯丙哌林片

2547　磷酸苯丙哌林胶囊

2548　磷酸苯丙哌林颗粒

2549　磷酸咯萘啶

2550　磷酸咯萘啶肠溶片

2551　磷酸咯萘啶注射液

2552　磷酸哌喹

2553　磷酸哌喹片

2554　磷酸哌嗪

2555　磷酸哌嗪片

2556　磷酸氢钙

2557　磷酸氢钙片

2558　磷酸奥司他韦

2559　磷酸奥司他韦胶囊

2560　磷酸氯喹

2561　磷酸氯喹片

2562　磷酸氯喹注射液

2563　磷霉素钙

2564　磷霉素钙片

2565　磷霉素钙胶囊

2566　磷霉素钙颗粒

2567　磷霉素钠

2568　磷霉素氨丁三醇

2569　磷霉素氨丁三醇散

2570　糜蛋白酶

2571　螺内酯

2572　螺内酯片

2573　螺内酯胶囊

放射性药品

2574 来昔决南钐[153Sm]注射液

2575 氙［133Xe］注射液

2576 邻碘［131I］马尿酸钠注射液

2577 注射用亚锡亚甲基二膦酸盐

2578注射用亚锡依替菲宁

2579注射用亚锡喷替酸

2580注射用亚锡植酸钠

2581注射用亚锡焦磷酸钠

2582注射用亚锡聚合白蛋白

2583枸橼酸镓［67Ｇａ］注射液

2584氟［18F］脱氧葡糖注射液

2585胶体磷［32Ｐ］酸铬注射液

2586高锝［99mＴc］酸钠注射液

2587铬［51Ｃr］酸钠注射液

2588氯化亚铊［201Tl］注射液

2589氯化锶[89Sr]注射液

2590碘[125I]密封籽源

2591碘［131Ｉ］化钠口服溶液

2592碘［131I］化钠胶囊

2593锝［99mTc］双半胱乙酯注射液

2594锝［99mTc］双半胱氨酸注射液

2595锝［99mTc］甲氧异腈注射液

2596锝［99mTc］亚甲基二膦酸盐注射液

2597锝［99mTc］依替菲宁注射液

2598锝［99mTc］植酸盐注射液

2599锝［99mTc］喷替酸盐注射液

2600锝［99mTc］焦磷酸盐注射液

2601锝［99mTc］聚合白蛋白注射液

2602磷［32P］酸钠盐口服溶液

2603　磷［32P］酸钠盐注射液

三部

生物制品通则

1　生物制品生产检定用菌毒种管理规程

2　生物制品国家标准物质制备和标定规程

3　生物制品生产用原材料及辅料质量控制规程

4　生物制品分批规程

5　生物制品分装和冻干规程

6　生物制品包装规程

7　生物制品贮藏和运输规程

8　免疫血清生产用马匹检疫和免疫规程

9　血液制品生产用人血浆

10 生物制品生产检定用动物细胞基质制备及检定规程

总论

1　人用疫苗总论

2　人用重组DNA蛋白制品总论

3　人用重组单克隆抗体制品总论

4　 微生态活菌制品总论

Ⅰ预防类

1　伤寒疫苗

2　伤寒甲型副伤寒联合疫苗

3　伤寒甲型乙型副伤寒联合疫苗

4　伤寒Vi多糖疫苗

5　重组B亚单位／菌体霍乱疫苗（肠溶胶囊）

6　A群脑膜炎球菌多糖疫苗

7　A群C群脑膜炎球菌多糖疫苗

8　A群C群脑膜炎球菌多糖结合疫苗

9　ACYW135群脑膜炎球菌多糖疫苗

10　b型流感嗜血杆菌结合疫苗

11　吸附白喉疫苗

12　吸附白喉疫苗（成人及青少年用）

13　吸附破伤风疫苗

14　吸附白喉破伤风联合疫苗

15　吸附白喉破伤风联合疫苗（成人及青少年用）

16　吸附百日咳白喉联合疫苗

17　吸附百白破联合疫苗

18　吸附无细胞百白破联合疫苗

19　皮上划痕用鼠疫活疫苗

20　皮上划痕人用炭疽活疫苗

21　皮上划痕人用布氏菌活疫苗

22　皮内注射用卡介苗

23　钩端螺旋体疫苗

24　乙型脑炎减毒活疫苗

25　冻干乙型脑炎灭活疫苗（Vero细胞）

26　森林脑炎灭活疫苗

27　双价肾综合征出血热灭活疫苗（Vero细胞）

28　双价肾综合征出血热灭活疫苗（地鼠肾细胞）

29　双价肾综合征出血热灭活疫苗（沙鼠肾细胞）

30　冻干人用狂犬病疫苗（Vero细胞）

31　冻干甲型肝炎减毒活疫苗

32　甲型肝炎灭活疫苗（人二倍体细胞）

33　重组乙型肝炎疫苗（酿酒酵母）

34　重组乙型肝炎疫苗（CHO细胞）

35　重组乙型肝炎疫苗（汉逊酵母）

36　甲型乙型肝炎联合疫苗

37　麻疹减毒活疫苗

38　腮腺炎减毒活疫苗

39　风疹减毒活疫苗（人二倍体细胞）

40　水痘减毒活疫苗

41　麻疹腮腺炎联合减毒活疫苗

42　麻疹风疹联合减毒活疫苗

43　麻腮风联合减毒活疫苗

44　流感全病毒灭活疫苗

45　流感病毒裂解疫苗

46　口服脊髓灰质炎减毒活疫苗（猴肾细胞）

47　脊髓灰质炎减毒活疫苗糖丸（人二倍体细胞）

48　脊髓灰质炎减毒活疫苗糖丸（猴肾细胞）

Ⅱ治疗类

1　白喉抗毒素

2　冻干白喉抗毒素

3　破伤风抗毒素

4　冻干破伤风抗毒素

5　多价气性坏疽抗毒素

6　冻干多价气性坏疽抗毒素

7　肉毒抗毒素

8　冻干肉毒抗毒素

9　抗蝮蛇毒血清

10　冻干抗蝮蛇毒血清

11　抗五步蛇毒血清

12　冻干抗五步蛇毒血清

13　抗银环蛇毒血清

14　冻干抗银环蛇毒血清

15　抗眼镜蛇毒血清

16　冻干抗眼镜蛇毒血清

17　抗炭疽血清

18　抗狂犬病血清

19　人血白蛋白

20　冻干人血白蛋白

21　人免疫球蛋白

22　冻干人免疫球蛋白

23　乙型肝炎人免疫球蛋白

24　冻干乙型肝炎人免疫球蛋白

25　静注乙型肝炎人免疫球蛋白（pH4）

26　冻干静注乙型肝炎人免疫球蛋白（pH4）

27　狂犬病人免疫球蛋白

28　冻干狂犬病人免疫球蛋白

29　破伤风人免疫球蛋白

30　冻干破伤风人免疫球蛋白

31　静注人免疫球蛋白（pH4）

32　冻干静注人免疫球蛋白（pH4）

33　人凝血因子Ⅷ

34　人纤维蛋白原

35　人纤维蛋白粘合剂

36　人凝血酶原复合物

37　抗人T细胞猪免疫球蛋白

38　抗人T细胞兔免疫球蛋白

39　注射用重组人促红素（CHO细胞）

40　重组人促红素注射液（CHO细胞）

41　注射用重组人干扰素α1b

42　重组人干扰素α1b注射液

43　重组人干扰素α1b滴眼液

44　注射用重组人干扰素α2a

45　重组人干扰素α2a注射液

46　重组人干扰素α2a栓

47　注射用重组人干扰素α2a（酵母）

48　注射用重组人干扰素α2b

49　重组人干扰素α2b注射液

50　重组人干扰素α2b滴眼液

51　重组人干扰素α2b栓

52　重组人干扰素α2b乳膏

53　重组人干扰素α2b凝胶

54　注射用重组人干扰素α2b（酵母）

55　注射用重组人干扰素α2b（假单胞菌）

56　重组人干扰素α2b注射液（假单胞菌）

57　重组人干扰素α2b喷雾剂（假单胞菌）

58　重组人干扰素α2b软膏（假单胞菌）

59　注射用重组人干扰素γ

60　注射用重组人白介素-2

61　重组人白介素-2注射液

62　注射用重组人白介素-2（Ⅰ）

63　注射用重组人白介素-11

64　注射用重组人白介素-11（酵母）

65　重组人粒细胞刺激因子注射液

66　注射用重组人粒细胞巨噬细胞刺激因子

67　重组牛碱性成纤维细胞生长因子外用溶液

68　外用重组牛碱性成纤维细胞生长因子

69　重组牛碱性成纤维细胞生长因子凝胶

70　重组牛碱性成纤维细胞生长因子滴眼液

71　外用重组人表皮生长因子

72　重组人表皮生长因子外用溶液（Ⅰ）

73　重组人表皮生长因子凝胶（酵母）

74　重组人表皮生长因子滴眼液（酵母）

75　注射用重组链激酶

76　尼妥珠单抗注射液

77　注射用鼠神经生长因子

78　注射用A型肉毒毒素

Ⅲ体内诊断类

1　结核菌素纯蛋白衍生物

2　卡介菌纯蛋白衍生物

3　布氏菌纯蛋白衍生物

4　锡克试验毒素

Ⅳ体外诊断类

1　乙型肝炎病毒表面抗原诊断试剂盒（酶联免疫法）

2　丙型肝炎病毒抗体诊断试剂盒（酶联免疫法）

3　人类免疫缺陷病毒抗体诊断试剂盒（酶联免疫法）

4　梅毒螺旋体抗体诊断试剂盒（酶联免疫法）

5　梅毒快速血浆反应素诊断试剂

6　梅毒甲苯胺红不加热血清试验诊断试剂

7　抗A抗B血型定型试剂（单克隆抗体）

四部

药用辅料

1　乙交酯丙交酯共聚物（5050）（供注射用）

2　乙交酯丙交酯共聚物（7525）（供注射用）

3　乙交酯丙交酯共聚物（8515）（供注射用）

4　乙基纤维素

5　乙基纤维素水分散体

6　乙基纤维素水分散体（B型）

7　乙酸乙酯

8　乙醇

9　二丁基羟基甲苯

10　二甲基亚砜

11　二甲硅油

12　二氧化钛

13 二氧化硅

14 二氧化碳

15 十二烷基硫酸钠

16 十八醇

17 十六十八醇

18 十六醇

19 丁香茎叶油

20 丁香油

21 丁香酚

22 三乙醇胺

23 三油酸山梨坦（司盘85）

24 三硅酸镁

25 三氯叔丁醇

26 三氯蔗糖

27 大豆油

28 大豆油（供注射用）

29 大豆磷脂

30 大豆磷脂（供注射用）

31 小麦淀粉

32 山梨酸

33 山梨酸钾

34 山嵛酸甘油酯

35 门冬氨酸

36 门冬酰胺

37 马来酸

38 马铃薯淀粉

39 无水亚硫酸钠

40 无水枸橼酸

41 无水碳酸钠

42 无水磷酸氢二钠

43 无水磷酸氢钙

44 木薯淀粉

45 D-木糖

46 木糖醇

47 牛磺酸

48 月桂山梨坦（司盘20）

49 月桂氮?酮

50 月桂酰聚氧乙烯（12）甘油酯

51 月桂酰聚氧乙烯（32）甘油酯

52 月桂酰聚氧乙烯（6）甘油酯

53 月桂酰聚氧乙烯（8）甘油酯

54 巴西棕榈蜡

55 玉米朊

56 玉米淀粉

57 正丁醇

58 甘油

59　甘油（供注射用）

60　甘油三乙酯

61　甘油磷酸钙

62　甘氨酸

63　可可脂

64　可压性蔗糖

65　可溶性淀粉

66　丙二醇

67　丙二醇（供注射用）

68　丙氨酸

69　丙烯酸乙酯-甲基丙烯酸甲酯共聚物水分散体

70　丙酸

71　石蜡

72　卡波姆

73　卡波姆共聚物

74　甲基纤维素

75　白凡士林

76　白陶土

77　白蜂蜡

78　亚硫酸氢钠

79　西黄蓍胶

80　色氨酸

81　冰醋酸

82　交联羧甲纤维素钠

83　交联聚维酮

84　羊毛脂

85　异丙醇

86　红氧化铁

87　纤维醋法酯

88　麦芽酚

89　麦芽糊精

90　麦芽糖

91　壳聚糖

92　低取代羟丙纤维素

93　谷氨酸钠

94　肠溶明胶空心胶囊

95　辛酸

96　辛酸钠

97　没食子酸

98　尿素

99　阿司帕坦

100　阿拉伯半乳聚糖

101　阿拉伯胶

102　纯化水

103　环甲基硅酮

104　环拉酸钠

105　苯扎氯铵

106　苯扎溴铵

107　苯甲酸钠

108　苯甲醇

109　DL-苹果酸

110　L-苹果酸

111　果胶

112　果糖

113　明胶空心胶囊

114　依地酸二钠

115　乳糖

116　单糖浆

117　油酰聚氧乙烯甘油酯

118　油酸乙酯

119　油酸山梨坦（司盘80）

120　油酸钠

121　油酸聚氧乙烯酯

122　泊洛沙姆188

123　泊洛沙姆407

124　组氨酸

125　枸橼酸

126　枸橼酸三乙酯

127　枸橼酸三正丁酯

128 枸橼酸钠

129 轻质氧化镁

130 轻质液状石蜡

131 氢化大豆油

132 氢化蓖麻油

133 氢氧化钠

134 氢氧化钾

135 胆固醇

136 亮氨酸

137 活性炭（供注射用）

138 浓氨溶液

139 盐酸

140 氧化钙

141 氧化锌

142 氧化镁

143 氨丁三醇

144 倍他环糊精

145 胶态二氧化硅

146 胶囊用明胶

147 粉状纤维素

148 烟酰胺

149 烟酸

150 DL-酒石酸

151　酒石酸钠

152　海藻酸

153　海藻酸钠

154　海藻糖

155　预胶化羟丙基淀粉

156　预胶化淀粉

157　黄凡士林

158　黄原胶

159　黄氧化铁

160　硅化微晶纤维素

161　硅酸镁铝

162　甜菊素

163　脱氧胆酸钠

164　羟乙纤维素

165　羟丙甲纤维素

166　羟丙甲纤维素邻苯二甲酸酯

167　羟丙纤维素

168　羟丙基倍他环糊精

169　羟丙基淀粉空心胶囊

170　羟苯乙酯

171　羟苯丁酯

172　羟苯丙酯

173　羟苯丙酯钠

174 羟苯甲酯

175 羟苯甲酯钠

176 羟苯苄酯

177 混合脂肪酸甘油酯（硬脂）

178 液状石蜡

179 淀粉水解寡糖

180 蛋黄卵磷脂

181 蛋黄卵磷脂（供注射用）

182 维生素E琥珀酸聚乙二醇酯

183 琥珀酸

184 琼脂

185 棕氧化铁

186 棕榈山梨坦（司盘40）

187 硬脂山梨坦（司盘60）

188 硬脂酸

189 硬脂酸钙

190 硬脂酸锌

191 硬脂酸聚烃氧（40）酯

192 硬脂酸镁

193 硝酸钾

194 硫酸

195 硫酸钙

196 硫酸铝

197　硫酸铵

198　硫酸羟喹啉

199　紫氧化铁

200　黑氧化铁

201　氯化钙

202　氯化钠（供注射用）

203　氯化钾

204　氯化镁

205　氯甲酚

206　稀盐酸

207　稀醋酸

208　稀磷酸

209　焦亚硫酸钠

210　焦糖

211　滑石粉

212　富马酸

213　酪氨酸

214　硼砂

215　硼酸

216　微晶纤维素

217　微晶蜡

218　腺嘌呤

219　羧甲纤维素钙

220 羧甲纤维素钠

221 羧甲淀粉钠

222 聚乙二醇1000

223 聚乙二醇1500

224 聚乙二醇300（供注射用）

225 聚乙二醇400

226 聚乙二醇400（供注射用）

227 聚乙二醇4000

228 聚乙二醇600

229 聚乙二醇6000

230 聚乙烯醇

231 聚山梨酯20

232 聚山梨酯40

233 聚山梨酯60

234 聚山梨酯80

235 聚山梨酯80（供注射用）

236 聚丙烯酸树脂Ⅱ

237 聚丙烯酸树脂III

238 聚丙烯酸树脂Ⅳ

239 聚甲丙烯酸铵酯Ⅰ

240 聚甲丙烯酸铵酯Ⅱ

241 聚氧乙烯

242 聚氧乙烯（35）蓖麻油

243 聚维酮K30

244 蔗糖

245 蔗糖八醋酸酯

246 蔗糖丸芯

247 蔗糖硬脂酸酯

248 碱石灰

249 碳酸丙烯酯

250 碳酸氢钠

251 碳酸氢钾

252 精制玉米油

253 精氨酸

254 橄榄油

255 醋酸

256 醋酸纤维素

257 醋酸钠

258 醋酸羟丙甲纤维素琥珀酸酯

259 糊精

260 缬氨酸

261 薄荷脑

262 磷酸

263 磷酸二氢钾

264 磷酸钙

265 磷酸氢二钠

266　磷酸氢二钾

267　磷酸氢二钾三水合物

268　磷酸氢二铵

269　磷酸淀粉钠

270　麝香草酚

通则和指导原则

序号 编码　 目录

1　0100　制剂通则

2　0101　片剂

3　0102　注射剂

4　0103　胶囊剂

5　0104　颗粒剂

6　0105　眼用制剂

7　0106　鼻用制剂

8　0107　栓剂

9　0108　丸剂

10　0109　软膏剂　乳膏剂

11　0110　糊剂

12　0111　吸入制剂

13　0112　喷雾剂

14　0113　气雾剂

15　0114　凝胶剂

16　0115　散剂

17　0116　糖浆剂

18　0117　搽剂

19　0118　涂剂

20　0119　涂膜剂

21　0120　酊剂

22　0121　贴剂

23　0122　贴膏剂

24　0123　口服溶液剂　口服混悬剂　口服乳剂

25　0124　植入剂

26　0125　膜剂

27　0126　耳用制剂

28　0127　洗剂

29　0128　冲洗剂

30　0129　灌肠剂

31　0181　合剂

32　0182　锭剂

33　0183　煎膏剂（膏滋）

34　0184　胶剂

35　0185　酒剂

36　0186　膏药

37　0187　露剂

38　0188　茶剂

39 0189 流浸膏剂与浸膏剂

40 0200 其他通则

41 0211 药材和饮片取样法

42 0212 药材和饮片检定通则

43 0213 炮制通则

44 0251 药用辅料

45 0261 制药用水

46 0291 国家药品标准物质通则

47 0300

48 0301 一般鉴别试验

49 0400 光谱法

50 0401 紫外-可见分光光度法

51 0402 红外分光光度法

52 0405 荧光分光光度法

53 0406 原子吸收分光光度法

54 0407 火焰光度法

55 0411 电感耦合等离子体原子发射光谱法

56 0412 电感耦合等离子体质谱法

57 0421 拉曼光谱法

58 0431 质谱法

59 0441 核磁共振波谱法

60 0451 X射线衍射法

61 0500 色谱法

62 0501 纸色谱法

63 0502 薄层色谱法

64 0511 柱色谱法

65 0512 高效液相色谱法

66 0513 离子色谱法

67 0514 分子排阻色谱法

68 0521 气相色谱法

69 0531 超临界流体色谱法

70 0532 临界点色谱法

71 0541 电泳法

72 0542 毛细管电泳法

73 0600 物理常数测定法

74 0601 相对密度测定法

75 0611 馏程测定法

76 0612 熔点测定法

77 0613 凝点测定法

78 0621 旋光度测定法

79 0622 折光率测定法

80 0631 pH值测定法

81 0632 渗透压摩尔浓度测定法

82 0633 黏度测定法

83 0661 热分析法

84 0681 制药用水电导率测定法

85 0682 制药用水中总有机碳测定法

86 0700 其他测定法

87 0701 电位滴定法与永停滴定法

88 0702 非水溶液滴定法

89 0703 氧瓶燃烧法

90 0704 氮测定法

91 0711 乙醇量测定法

92 0712 甲氧基、乙氧基与羟丙氧基测定法

93 0713 脂肪与脂肪油测定法

94 0721 维生素A测定法

95 0722 维生素D测定法

96 0731 蛋白质含量测定法

97 0800 限量检查法

98 0801 氯化物检查法

99 0802 硫酸盐检查法

100 0803 硫化物检查法

101 0804 硒检查法

102 0805 氟检查法

103 0806 氰化物检查法

104 0807 铁盐检查法

105 0808 铵盐检查法

106 0821 重金属检查法

107 0822 砷盐检查法

108 0831 干燥失重测定法

109 0832 水分测定法

110 0841 炽灼残渣检查法

111 0842 易炭化物检查法

112 0861 残留溶剂测定法

113 0871 甲醇量检查法

114 0872 合成多肽中的醋酸测定法

115 0873 2-乙基己酸测定法

116 0900 特性检查法

117 0901 溶液颜色检查法

118 0902 澄清度检查法

119 0903 不溶性微粒检查法

120 0904 可见异物检查法

121 0921 崩解时限检查法

122 0922 融变时限检查法

123 0923 片剂脆碎度检查法

124 0931 溶出度与释放度测定法

125 0941 含量均匀度检查法

126 0942 最低装量检查法

127 0951 吸入制剂微细粒子空气动力学特性测定法

128 0952 黏附力测定法

129 0981 结晶性检查法

130 0982 粒度和粒度分布测定法

131 0983 锥入度测定法

132 1100 生物检查法

133 1101 无菌检查法

134 1105 非无菌产品微生物限度检查：微生物计数法

135 1106 非无菌产品微生物限度检查：控制菌检查法

136 1107 非无菌药品微生物限度标准

137 1121 抑菌效力检查法

138 1141 异常毒性检查法

139 1142 热原检查法

140 1143 细菌内毒素检查法

141 1144 升压物质检查法

142 1145 降压物质检查法

143 1146 组胺类物质检查法

144 1147 过敏反应检查法

145 1148 溶血与凝聚检查法

146 1200 生物活性测定法

147 1201 抗生素微生物检定法

148 1202 青霉素酶及其活力测定法

149 1205 升压素生物测定法

150 1206 细胞色素C活力测定法

151 1207 玻璃酸酶测定法

152 1208 肝素生物测定法

153 1209 绒促性素生物测定法

154 1210 缩宫素生物测定法

155 1211 胰岛素生物测定法

156 1212 精蛋白锌胰岛素注射液延缓作用测定法

157 1213 硫酸鱼精蛋白生物测定法

158 1214 洋地黄生物测定法

159 1215 葡萄糖酸锑钠毒力检查法

160 1216 卵泡刺激素生物测定法

161 1217 黄体生成素生物测定法

162 1218 降钙素生物测定法

163 1219 生长激素生物测定法

164 1401 放射性药品检定法

165 1421 灭菌法

166 1431 生物检定统计法

167 2000 中药其他方法

168 2001 显微鉴别法

169 2101 膨胀度测定法

170 2102 膏药软化点测定法

171 2201 浸出物测定法

172 2202 鞣质含量测定法

173 2203 桉油精含量测定法

174 2204 挥发油测定法

175 2301 杂质检查法

176 2302 灰分测定法

177 2303 酸败度测定法

178 2321 铅、镉、砷、汞、铜测定法

179 2322 汞和砷元素形态及其价态测定法

180 2331 二氧化硫残留量测定法

181 2341 农药残留量测定法

182 2351 黄曲霉毒素测定法

183 2400 注射剂有关物质检查法

184 3000 生物制品相关检查方法

185 3100 含量测定法

186 3101 固体总量测定法

187 3102 唾液酸测定法（间苯二酚显色法）

188 3103 磷测定法

189 3104 硫酸铵测定法

190 3105 亚硫酸氢钠测定法

191 3106 氢氧化铝（或磷酸铝）测定法

192 3107 氯化钠测定法

193 3108 枸橼酸离子测定法

194 3109 钾离子测定法

195 3110 钠离子测定法

196 3111 辛酸钠测定法

197 3112 乙酰色氨酸测定法

198 3113 苯酚测定法

199 3114 间甲酚测定法

200　3115　硫柳汞测定法

201　3116　对羟基苯甲酸甲酯、对羟基苯甲酸丙酯含量测定法

202　3117　O-乙酰基测定法

203　3118　己二酰肼含量测定法

204　3119　高分子结合物含量测定法

205　3120　人血液制品中糖及糖醇测定法

206　3121　人血白蛋白多聚体测定法

207　3122　人免疫球蛋白类制品IgG单体加二聚体测定法

208　3123　人免疫球蛋白中甘氨酸含量测定法

209　3124　重组人粒细胞刺激因子蛋白质含量测定法

210　3125　组胺人免疫球蛋白中游离磷酸组胺测定法

211　3126　IgG含量测定法

212　3127　单抗分子大小变异体测定法（CE-SDS）

213　3200　化学残留物测定法

214　3201　乙醇残留量测定法

215　3202　聚乙二醇残留量测定法

216　3203　聚山梨酯80残留量测定法

217　3204　戊二醛残留量测定法

218　3205　磷酸三丁酯残留量测定法

219　3206　碳二亚胺残留量测定法

220　3207　游离甲醛测定法

221　3208　人血白蛋白铝残留量测定法

222　3209　羟胺残留量测定法

223 3300 微生物检查法

224 3301 支原体检查法

225 3302 外源病毒因子检查法

226 3303 鼠源性病毒检查法

227 3304 SV40核酸序列检查法

228 3305 猴体神经毒力试验

229 3306 血液制品生产用人血浆病毒核酸检测技术要求

230 3400 生物测定法

231 3401 免疫印迹法

232 3402 免疫斑点法

233 3403 免疫双扩散法

234 3404 免疫电泳法

235 3405 肽图检查法

236 3406 质粒丢失率检查法

237 3407 外源性DNA残留量测定法

238 3408 抗生素残留量检查法（培养法）

239 3409 激肽释放酶原激活剂测定法

240 3410 抗补体活性测定法

241 3411 牛血清白蛋白残留量测定法

242 3412 大肠杆菌菌体蛋白质残留量测定法

243 3413 假单胞菌菌体蛋白质残留量测定法

244 3414 酵母工程菌菌体蛋白质残留量测定法

245 3415 类A血型物质测定法

246　3416　鼠IgG残留量测定法

247　3417　无细胞百日咳疫苗鉴别试验（酶联免疫法）

248　3418　抗毒素、抗血清制品鉴别试验（酶联免疫法）

249　3419　A群脑膜炎球菌多糖分子大小测定法

250　3420　伤寒Vi多糖分子大小测定法

251　3421　b型流感嗜血杆菌结合疫苗多糖含量测定法

252　3422　人凝血酶活性检查法

253　3423　活化的凝血因子活性检查法

254　3424　肝素含量测定法

255　3425　抗A、抗B血凝素测定法

256　3426　人红细胞抗体测定法

257　3427　人血小板抗体测定法

258　3500　生物活性/效价测定法

259　3501　重组乙型肝炎疫苗（酵母）体外相对效力检查法

260　3502　甲型肝炎灭活疫苗体外相对效力检查法

261　3503　人用狂犬病疫苗效价测定法

262　3504　吸附破伤风疫苗效价测定法

263　3505　吸附白喉疫苗效价测定法

264　3506　类毒素絮状单位测定法

265　3507　白喉抗毒素效价测定法

266　3508　破伤风抗毒素效价测定法

267　3509　气性坏疽抗毒素效价测定法

268　3510　肉毒抗毒素效价测定法

269 3511 抗蛇毒血清效价测定法

270 3512 狂犬病免疫球蛋白效价测定法

271 3513 人免疫球蛋白中白喉抗体效价测定法

272 3514 人免疫球蛋白Fc段生物学活性测定法

273 3515 抗人T细胞免疫球蛋白效价测定法（E玫瑰花环形成抑制试验）

274 3516 抗人T细胞免疫球蛋白效价测定法（淋巴细胞毒试验）

275 3517 人凝血因子Ⅱ效价测定法

276 3518 人凝血因子Ⅶ效价测定法

277 3519 人凝血因子Ⅸ效价测定法

278 3520 人凝血因子Ⅹ效价测定法

279 3521 人凝血因子Ⅷ效价测定法

280 3522 重组人促红素体内生物学活性测定法

281 3523 干扰素生物学活性测定法

282 3524 重组人白介素-2生物学活性测定法

283 3525 重组人粒细胞刺激因子生物学活性测定法

284 3526 重组人粒细胞巨噬细胞刺激因子生物学活性测定法

285 3527 重组牛碱性成纤维细胞生长因子生物学活性测定法

286 3528 重组人表皮生长因子生物学活性测定法

287 3529 重组链激酶生物学活性测定法

288 3530 鼠神经生长因子生物学活性测定法

289 3531 尼妥珠单抗注射液生物学活性测定法

290 3532 重组人白介素-11生物学活性测定法

291 3533 注射用A型肉毒毒素成品效价测定法（平行线法）

292 3600 特定生物原材料/动物

293 3601 无特定病原体鸡胚质量检测要求

294 3602 实验动物微生物学检测要求

295 3603 实验动物寄生虫学检测要求

296 3604 新生牛血清检测要求

297 3605 细菌生化反应培养基

298 3700

299 3701 生物制品国家标准物质目录

300 8000 试剂与标准物质

301 8001 试药

302 8002 试液

303 8003 试纸

304 8004 缓冲液

305 8005 指示剂与指示液

306 8006 滴定液

307 8061 对照品 对照药材 对照提取物

308 8062 对照品 标准品

309 9000 指导原则

310 9001 原料药物与制剂稳定性试验指导原则

311 9011 药物制剂人体生物利用度和生物等效性试验指导原则

312 9012 生物样品定量分析方法验证指导原则

313 9013 缓释、控释和迟释制剂指导原则

314 9014 微粒制剂指导原则

315	9015	药品晶型研究及晶型质量控制指导原则
316	9101	药品质量标准分析方法验证指导原则
317	9102	药品杂质分析指导原则
318	9103	药物引湿性试验指导原则
319	9104	近红外分光光度法指导原则
320	9105	中药生物活性测定指导原则
321	9106	基于基因芯片的药物评价技术与方法指导原则
322	9107	中药材DNA条形码分子鉴定法指导原则
323	9201	药品微生物检验替代方法验证指导原则
324	9202	非无菌产品微生物限度检查指导原则
325	9203	药品微生物实验室质量管理指导原则
326	9204	微生物鉴定指导原则
327	9205	药品洁净实验室微生物监测和控制指导原则
328	9206	无菌检查用隔离系统验证指导原则
329	9301	注射剂安全性检查法应用指导原则
330	9302	中药有害残留物限量制定指导原则
331	9303	色素测定法指导原则
332	9304	中药中铝、铬、铁、钡元素测定指导原则
333	9305	中药中真菌毒素测定指导原则
334	9501	正电子类放射性药品质量控制指导原则
335	9502	锝［99mTc］放射性药品质量控制指导原则
336	9601	药用辅料功能性指标研究指导原则
337	9621	药包材通用要求指导原则

338　9622　药用玻璃材料和容器指导原则

339　9901　国家药品标准物质制备指导原则

本篇引用的法规
中央法规
中华人民共和国药品管理法(2015修正)
中华人民共和国统计法(2009修订)
引用本篇的法规 案例 论文
地方法规规章
成都市食品药品监督管理局关于贯彻实施《中华人民共和国药典》2015年版的通知
案例与裁判文书
唐定国与何燕芬、海南黎仙养生酒业有限公司买卖合同纠纷一审民事判决书
哈尔滨普济健康科技发展有限公司、王可新产品责任纠纷一审民事裁定书
唐定国与梧州市井泉酒业有限公司网络购物合同纠纷二审民事判决书
彭仕切与思南县市场监督管理局质量监督检验检疫行政管理:其他(质量监督)一审行政判决书
刘成春与杭州都健网络科技有限公司网络购物合同纠纷一审民事判决书
刘文红诉华润万家有限公司龙华店买卖合同纠纷案

*注：本文格式遵循《全国人大法规备案审查信息平台电子文件格式规范（试行）》标准。

©北大法宝：（www.pkulaw.com）专业提供法律信息、法学知识和法律软件领域各类解决方案。北大法宝为您提供丰富的参考资料，正式引用法规条文时请与标准文本核对。

欢迎查看所有产品和服务。

法宝快讯： 如何快速找到您需要的检索结果？ 法宝 V6 有何新特色？

扫描二维码阅读原文

原文链接：https://www.pkulaw.com/chl/4a3fef91b94b525ebdfb.html
