# Supplementary material for: China’s Legal Protection System for Pangolins: Past, Present, and Future
Source: Animals (Basel). 2025 Aug 18;15(16):2422. doi: 10.3390/ani15162422 (PMC12383201; doi:10.3390/ani15162422)
Supplement: Supplementary file 1 [file animals-15-02422-s001.zip › Supplementary Material S2 -Full Texts of Laws and Regulations Related to Pangolins in China/【1】国务院关于积极保护和合理利用野生动物资源的指示(FBM-CLI.2.pdf]

国务院关于积极保护和合理利用野生动物资源的指示

制定机关： 国务院

公布日期：1962.09.14

施行日期：1962.09.14

时效性： 现行有效

效力位阶： 国务院规范性文件

法规类别： 野生动植物资源

国务院关于积极保护和合理

利用野生动物资源的指示

（一九六二年九月十四日）

我国野生动物资源十分丰富，计有鸟类一千一百多种，兽类四百多种，占世界鸟兽种别总数的百分之十二。

其中，不仅经济动物种类繁多，还有不少闻名世界的珍贵稀有鸟兽。野生动物是我国的一项巨大自然财富，每年不仅可以获得大量的野生动物肉类，还可以获得大量的野生动物毛皮和贵重的鹿茸、麝香。这些产品对改善人民生活和换取外汇都起了重要作用。

近几年来，有些地区对如何保护和合理利用野生动物资源的问题虽已经开始注意，并且取得了一定的成绩。但是，由于这是一项新的工作，许多地区还未普遍重视起来。不少地区对于野生动物偏重猎取，不注意保护，甚至把许多不应该列为

害鸟害兽的，也列为害鸟害兽而加以消灭，致使野生动物资源遭到了严重的破坏。因此，近几年来，一些大型肉用动物和经济价值高的鸟兽，不仅数量上大大减少，分布区域也在逐渐缩小。所有这些情况，应该引起各级领导机关的重视。

为了迅速改变这种严重情况，把野生动物资源的保护和合理利用工作全面地开展起来，特作如下指示：

一、野生动物资源是国家的自然财富，各级人民委员会必须切实保护，在保护的基础上加以合理利用。当前首先要做好保护工作，要责成各地林场和所有有狩猎动物资源的人民公社、农场、农垦场、牧场，将所辖范围内的这项资源保护、管理起来。各地驻军也有保护所在地区的狩猎动物资源的责任。目前野生动物资源贫乏和破坏比较严重的地区，应该象封山育林那样，建立禁猎区，停猎一个时期。资源未遭到破坏的地区，也应该在不影响狩猎动物资源继续增长的前提下，确定合理的猎取量，有计划地组织利用。没有主管部门发给的狩猎证，任何人不得进行狩猎。到猎场、林场、农场、农垦场、牧场、人民公社管区内打猎的狩猎队和个人还应该得到上述单位的同意。严禁在禁猎期狩猎。禁猎区由各省（区、市）自行规定，报林业部备案

二、各省、自治区、直辖市人民委员会应该加强狩猎生产的组织管理工作。各地应该根据加强资源保护，积极繁殖饲养，合理猎取利用的“护、养、猎并举”的狩猎业方针，结合本地区具体情况，制定临时性的狩猎管理办法或发布狩猎管理布告，建立猎民协会，逐步把城乡猎民组织起来，做到有组织、有领导地开展狩猎活动。今后凡是中央一级机关、部队、团体、学校和工矿企业的狩猎队到各省（区），以及这一省（区）的狩猎队到另一省（区）去狩猎时，都必须事先商得

对方主管部门的同意，并且严格遵守当地的各项有关规定和接受当地的监督、检查。

三、保护和合理利用野生动物资源，是一项新的群众性的工作，各地在做好组织管理工作的同时，还必须做好宣传教育工作，充分利用报刊、杂志、广播电台、宣传画等形式，广泛开展宣传活动。教育部门应该在各级学校的生物学课程中，适当增添保护野生动物资源的内容，使广大群众都能了解保护和合理利用野生动物资源的重要意义。

四、对于珍贵、稀有或特产的鸟兽：大熊猫、东北虎、野象、野牛、野骆驼、野马、牛羚（扭角羚）、藏羚、鬣羚、金丝猴、长臂猿、叶猴、懒猴、梅花鹿、獐（河麂）、孔雀、丹顶鹤、褐马鸡、犀鸟等，严禁猎捕，并在其主要栖息、繁殖地区，建立自然保护区，加以保护。如因特殊需要，一定要猎捕上述动物时，必须经过林业部批准。对于经济价值高，数量已经稀少或目前虽有一定数量，但为我国特产的鸟兽：紫貂、石貂、小熊猫、扫雪、青羊、盘羊、雪豹、云豹、野驴、野牦牛、马鹿、驼鹿、驯鹿、白唇鹿、白臀鹿、水鹿、麝、白鼬、水獭、金猫、雪兔、蒙鼠、一般猴类、海豹、江猪、穿山甲、兰马鸡、白马鸡、原鸡、血雉、虹雉、长尾雉、金鸡、白鹇、天鹅、鸳鸯、铜鸡等，禁止猎取或严格控制猎取量，每年猎取多少，必须经过省（区、市）主管部门批准。对产茸的鹿类，应该大力提倡活捉饲养，改变过去“打鹿砍茸”的生产方式。

五、禁止采用破坏野生动物资源和危害人畜安全的狩猎工具和方法，如地弓、地枪、毒药、炸药、阎王碓、绝后窖、自动武器以及机动车追猎、夜间照明行猎、

歼灭性围猎、火攻、烟熏、掏窝、挖洞、捡鸟蛋等。但是，在消灭狼豺和鼠害时，可以采用歼灭性围猎、掏窝、挖桐、毒药、机动车追猎和军用武器。

早在一九五九年，国务院即决定将狩猎事业交由林业部门统一管理。几年来林业部门在这方面做了许多工作，取得了一定成绩。但是，目前各地主管这一工作的部门很不一致，作法也不一致，以致在工作上引起了许多不应有的混乱和困难。为了在全国范围内，切实保护野生动物资源和正确开展狩猎事业，各地应该迅速将这一工作统一交由林业部门管理，并加强有关管理机构。

*注：本文格式遵循《全国人大法规备案审查信息平台电子文件格式规范（试行）》标准。

©北大法宝：（www.pkulaw.com）专业提供法律信息、法学知识和法律软件领域各类解决方案。北大法宝为您提供丰富的参考资料，正式引用法规条文时请与标准文本核对。

欢迎查看所有产品和服务。

法宝快讯： 如何快速找到您需要的检索结果？ 法宝 V6 有何新特色？

扫描二维码阅读原文

原文链接：https://www.pkulaw.com/chl/fd3fd92c975c9709bdfb.html
