# Supplementary material for: China’s Legal Protection System for Pangolins: Past, Present, and Future
Source: Animals (Basel). 2025 Aug 18;15(16):2422. doi: 10.3390/ani15162422 (PMC12383201; doi:10.3390/ani15162422)
Supplement: Supplementary file 1 [file animals-15-02422-s001.zip › Supplementary Material S2 -Full Texts of Laws and Regulations Related to Pangolins in China/【20】市场监管总局、国家林草局关于联合开展野生动物保护专项整治行动的通知(FBM-CLI.4.pdf]

市场监管总局、国家林草局关于联合开展野生动物保护专项整治行动的通知

制定机关： 国家市场监督管理总局 国家林业和草原局 机构沿革

发文字号：国市监网监〔2019〕107号

公布日期：2019.05.24

施行日期：2019.05.24

时效性： 现行有效

效力位阶： 部门工作文件

法规类别： 野生动植物资源

市场监管总局、国家林草局关于联合开展野生动物保护专项整治行动的通知

（国市监网监〔2019〕107号）

各省、自治区、直辖市及新疆生产建设兵团市场监管局（厅、委）、林草主管部门：

野生动物保护事关生态安全、民生福祉和国家形象。为进一步加强生态安全保护，推进生态文明建设，市场监管总局、国家林草局决定自即日起至10月，在全国范围内联合开展一次野生动物保护专项整治行动。现就有关事项通知如下：

一、高度重视，认真组织开展专项整治行动

各地市场监管部门、林草主管部门要深入学习贯彻习近平生态文明思想，提高政治站位，充分认识当前加强野生动物资源保护和执法打击工作的重要性、紧迫性

，以强烈的政治担当、责任担当，全力组织开展好专项整治行动。要结合各地实际，研究制定切实可行的专项整治行动方案，着力加强濒危野生动物保护，有效维护国家生态安全，不断满足人民群众对良好生态环境的需求。

二、突出重点，严厉查处违法经营行为

各地市场监管部门、林草主管部门要密切合作，保持高压态势，按照野生动物保护相关法律法规的规定，在发挥“双随机、一公开”监管日常性、基础性作用的同时，根据监督检查、投诉举报、转办交办、媒体曝光等渠道发现的违法问题线索，举一反三，突出非法猎捕和经营野生动物易发、多发地区，突出城乡结合部、农村等区域，突出集贸、批发、农产品、花鸟、古玩市场等场所，突出非法销售野生动物及制品网站，依法严厉查处为出售、购买、利用野生动物或者禁止使用的猎捕工具发布广告和为出售、购买、利用野生动物制品发布广告的行为，依法严厉查处网络交易平台、商品交易市场等交易场所为违法出售、购买、利用野生动物及其制品或者禁止使用的猎捕工具提供交易服务的行为。对查获的重大案件，要依法加大处罚力度并及时予以曝光；对涉嫌犯罪的，要及时移送司法机关处理。有条件的地方市场监管部门、林草主管部门，要充分利用网监技术手段，开展对网络交易平台的定向监测。

三、密切协作，形成监管执法合力

各地市场监管部门、林草主管部门要在当地党委、政府的统一领导下，积极配合农业农村、公安、交通、海关、网信等部门的相关整治工作。要注重发挥打击野生动植物非法贸易部门联席会议等协调机制作用，在部门间、地区间及时互通监管执法信息，发挥整体优势，线上线下联动，强化对象牙、犀牛角、虎、穿山甲及其制品和以下违法行为的协同监管和联合检查、联合执法、联合惩戒：

（一）未经批准、未取得或者未按照规定使用专用标识，或者未持有、未附有人工繁育许可证、批准文件的副本或者专用标识出售、购买、利用、运输、携带、寄递国家重点保护野生动物及其制品的；

（二）未持有合法来源证明出售、利用、运输非国家重点保护野生动物的；

（三）生产、经营使用国家重点保护野生动物及其制品或者没有合法来源证明的非国家重点保护野生动物及其制品制作食品，或者为食用非法购买国家重点保护的野生动物及其制品的。

四、依法维权，强化宣传舆论引导

各地市场监管部门、林草主管部门要充分发挥12315热线及全国12315平台、各地林草主管部门公布的野生动物保护举报电话和行业协会、基层市场监管部门、林草主管部门的作用，及时受理投诉举报，对举报非法经营野生动物及其制品的，要迅速予以查实，依法处理。要研究采取多种有效形式宣传野生动物保护的法律法规以及野生动物保护工作取得的成效，增强全社会的法律意识和保护意识，为专项整治行动开展营造良好氛围。

五、及时总结，按时上报相关材料

各地市场监管部门、林草主管部门要及时总结经验，巩固成果。市场监管总局、国家林草局将对专项整治行动开展情况适时组织联合调研指导。请各省、自治区、直辖市及新疆生产建设兵团市场监管局（厅、委）、林草主管部门于今年7月15日前分别向市场监管总局（网监司）、国家林草局（动植物司）报送阶段性工作部署开展情况，11月15日前报送专项整治行动总结，包括典型案例、相关图片等

，并附本部门专项整治行动情况统计表（以上材料须同时报送纸质件和电子版）。工作中的重大情况，应及时报告当地政府和市场监管总局、国家林草局。

工作联系人、联系方式（略）

附件：野生动物保护专项整治行动情况统计表（略）

市场监管总局

国家林草局

2019年5月24日

引用本篇的法规 案例 论文

地方法规规章

徐州市市场监督管理局、徐州市林业局关于认真落实《市场监管总局国家林草局关于联合开展野生动物保护专项整治行动的通知》的通知

*注：本文格式遵循《全国人大法规备案审查信息平台电子文件格式规范（试行）》标准。

©北大法宝：（www.pkulaw.com）专业提供法律信息、法学知识和法律软件领域各类解决方案。北大法宝为您提供丰富的参考资料，正式引用法规条文时请与标准文本核对。

欢迎查看所有产品和服务。

法宝快讯：如何快速找到您需要的检索结果？ 法宝 V6 有何新特色？

扫描二维码阅读原文

原文链接：https://www.pkulaw.com/chl/c0a721c64f63b423bdfb.html
