# Supplementary material for: China’s Legal Protection System for Pangolins: Past, Present, and Future
Source: Animals (Basel). 2025 Aug 18;15(16):2422. doi: 10.3390/ani15162422 (PMC12383201; doi:10.3390/ani15162422)
Supplement: Supplementary file 1 [file animals-15-02422-s001.zip › Supplementary Material S2 -Full Texts of Laws and Regulations Related to Pangolins in China/【21】全国人民代表大会常务委员会关于全面禁止非法野生动物交易、革除滥食野生动物陋习、切实保障人民群众生命健康安全的决定(FBM-CLI.1.pdf]

全国人民代表大会常务委员会关于全面禁止非法野生动物交易、革除滥食野生动物陋习、切实保障人民群众生命健康安全的决定

制定机关： 全国人大常委会

公布日期：2020.02.24

施行日期：2020.02.24

时效性： 现行有效

效力位阶： 有关法律问题和重大问题的决定

法规类别： 野生动植物资源 森林和野生动植物保护区

全国人民代表大会常务委员会关于全面禁止非法野生动物交易、革除滥食野生动物陋习、切实保障人民群众生命健康安全的决定

（2020年2月24日第十三届全国人民代表大会常务委员会第十六次会议通过）

为了全面禁止和惩治非法野生动物交易行为，革除滥食野生动物的陋习，维护生物安全和生态安全，有效防范重大公共卫生风险，切实保障人民群众生命健康安全，加强生态文明建设，促进人与自然和谐共生，全国人民代表大会常务委员会作出如下决定：

一、凡《中华人民共和国野生动物保护法》和其他有关法律禁止猎捕、交易、运输、食用野生动物的，必须严格禁止。

对违反前款规定的行为，在现行法律规定基础上加重处罚。

法宝联想

法律法规 规范性文件（3）法规解读（1）法律动态（1）

司法案例 司法案例（27）

专题参考 专题参考（1）

二、全面禁止食用国家保护的“有重要生态、科学、社会价值的陆生野生动物”以及其他陆生野生动物，包括人工繁育、人工饲养的陆生野生动物。

全面禁止以食用为目的猎捕、交易、运输在野外环境自然生长繁殖的陆生野生动物。

对违反前两款规定的行为，参照适用现行法律有关规定处罚。

法宝联想

法律法规 规范性文件（1）法规解读（1）法律动态（1）

司法案例 司法案例（10）

法学期刊 法学期刊（2）

专题参考 专题参考（3）

三、列入畜禽遗传资源目录的动物，属于家畜家禽，适用《中华人民共和国畜牧法》的规定。

国务院畜牧兽医行政主管部门依法制定并公布畜禽遗传资源目录。

法宝联想

司法案例 司法案例（2）

专题参考 专题参考（2）

四、因科研、药用、展示等特殊情况，需要对野生动物进行非食用性利用的，应

当按照国家有关规定实行严格审批和检疫检验。

国务院及其有关主管部门应当及时制定、完善野生动物非食用性利用的审批和检疫检验等规定，并严格执行。

法宝联想

专题参考　专题参考（1）

五、各级人民政府和人民团体、社会组织、学校、新闻媒体等社会各方面，都应当积极开展生态环境保护和公共卫生安全的宣传教育和引导，全社会成员要自觉增强生态保护和公共卫生安全意识，移风易俗，革除滥食野生动物陋习，养成科学健康文明的生活方式。

法宝联想

法律法规　法规解读（1）法律动态（1）

司法案例　司法案例（2）

六、各级人民政府及其有关部门应当健全执法管理体制，明确执法责任主体，落实执法管理责任，加强协调配合，加大监督检查和责任追究力度，严格查处违反本决定和有关法律法规的行为；对违法经营场所和违法经营者，依法予以取缔或者查封、关闭。

法宝联想

法律法规　法规解读（1）法律动态（1）

专题参考　专题参考（1）

七、国务院及其有关部门和省、自治区、直辖市应当依据本决定和有关法律，制定、调整相关名录和配套规定。

国务院和地方人民政府应当采取必要措施，为本决定的实施提供相应保障。有关地方人民政府应当支持、指导、帮助受影响的农户调整、转变生产经营活动，根据实际情况给予一定补偿。

法宝联想

法律法规　规范性文件（18）草案（1）

司法案例　司法案例（2）

专题参考　专题参考（1）

八、本决定自公布之日起施行。

*注：本文格式遵循《全国人大法规备案审查信息平台电子文件格式规范（试行）》标准。

©北大法宝：（www.pkulaw.com）专业提供法律信息、法学知识和法律软件领域各类解决方案。北大法宝为您提供丰富的参考资料，正式引用法规条文时请与标准文本核对。

欢迎查看所有产品和服务。

法宝快讯： 如何快速找到您需要的检索结果？ 法宝 V6 有何新特色？

扫描二维码阅读原文

原文链接：https://www.pkulaw.com/chl/cab9fc867524bdcebdfb.html
