# Supplementary material for: China’s Legal Protection System for Pangolins: Past, Present, and Future
Source: Animals (Basel). 2025 Aug 18;15(16):2422. doi: 10.3390/ani15162422 (PMC12383201; doi:10.3390/ani15162422)
Supplement: Supplementary file 1 [file animals-15-02422-s001.zip › Supplementary Material S2 -Full Texts of Laws and Regulations Related to Pangolins in China/【22】中华人民共和国民法典(FBM-CLI.1.pdf]

中华人民共和国民法典

制定机关： 全国人民代表大会

发文字号：中华人民共和国主席令第45号

公布日期：2020.05.28

施行日期：2021.01.01

时效性： 现行有效

效力位阶： 法律

法规类别： 民法

专题分类： 民法典 人工智能

中华人民共和国主席令

（第四十五号）

《中华人民共和国民法典》已由中华人民共和国第十三届全国人民代表大会第三次会议于2020年5月28日通过，现予公布，自2021年1月1日起施行。

中华人民共和国主席 习近平

2020年5月28日

中华人民共和国民法典

（2020年5月28日第十三届全国人民代表大会第三次会议通过）

目录

第一编　总则

第一章　基本规定

第二章　自然人

第一节　民事权利能力和民事行为能力

第二节　监护

第三节　宣告失踪和宣告死亡

第四节　个体工商户和农村承包经营户

第三章　法人

第一节　一般规定

第二节　营利法人

第三节　非营利法人

第四节　特别法人

第四章　非法人组织

第五章　民事权利

第六章　民事法律行为

第一节　一般规定

第二节　意思表示

第三节　民事法律行为的效力

第四节　民事法律行为的附条件和附期限

第七章　代理

第一节　一般规定

第二节　委托代理

第三节　代理终止

第八章　民事责任

第九章　诉讼时效

第十章　期间计算

第二编　物权

第一分编　通则

第一章　一般规定

第二章　物权的设立、变更、转让和消灭

第一节　不动产登记

第二节　动产交付

第三节　其他规定

第三章　物权的保护

第二分编　所有权

第四章　一般规定

第五章　国家所有权和集体所有权、私人所有权

第六章　业主的建筑物区分所有权

第七章　相邻关系

第八章　共有

第九章　所有权取得的特别规定

第三分编　用益物权

第十章　一般规定

第十一章　土地承包经营权

第十二章　建设用地使用权

第十三章　宅基地使用权

第十四章　居住权

第十五章　地役权

第四分编　担保物权

第十六章　一般规定

第十七章　抵押权

第一节　一般抵押权

第二节　最高额抵押权

第十八章　质权

第一节　动产质权

第二节　权利质权

第十九章　留置权

第五分编　占有

第二十章　占有

第三编　合同

第一分编　通则

第一章　一般规定

第二章　合同的订立

第三章　合同的效力

第四章　合同的履行

第五章　合同的保全

第六章　合同的变更和转让

第七章　合同的权利义务终止

第八章　违约责任

第二分编　典型合同

第九章　买卖合同

第十章　供用电、水、气、热力合同

第十一章　赠与合同

第十二章　借款合同

第十三章　保证合同

第一节　一般规定

第二节　保证责任

第十四章　租赁合同

第十五章　融资租赁合同

第十六章　保理合同

第十七章　承揽合同

第十八章　建设工程合同

第十九章　运输合同

第一节　一般规定

第二节　客运合同

第三节　货运合同

第四节　多式联运合同

第二十章　技术合同

第一节　一般规定

第二节　技术开发合同

第三节　技术转让合同和技术许可合同

第四节　技术咨询合同和技术服务合同

第二十一章　保管合同

第二十二章　仓储合同

第二十三章　委托合同

第二十四章　物业服务合同

第二十五章　行纪合同

第二十六章　中介合同

第二十七章　合伙合同

第三分编　准合同

第二十八章　无因管理

第二十九章　不当得利

第四编　人格权

第一章　一般规定

第二章　生命权、身体权和健康权

第三章　姓名权和名称权

第四章　肖像权

第五章　名誉权和荣誉权

第六章　隐私权和个人信息保护

第五编　婚姻家庭

第一章　一般规定

第二章　结婚

第三章　家庭关系

第一节　夫妻关系

第二节　父母子女关系和其他近亲属关系

第四章　离婚

第五章　收养

第一节　收养关系的成立

第二节　收养的效力

第三节　收养关系的解除

第六编　继承

第一章　一般规定

第二章　法定继承

第三章　遗嘱继承和遗赠

第四章　遗产的处理

第七编　侵权责任

第一章　一般规定

第二章　损害赔偿

第三章　责任主体的特殊规定

第四章　产品责任

第五章　机动车交通事故责任

第六章　医疗损害责任

第七章　环境污染和生态破坏责任

第八章　高度危险责任

第九章　饲养动物损害责任

第十章　建筑物和物件损害责任

附则

第一编　总则

第一章　基本规定

第一条

为了保护民事主体的合法权益，调整民事关系，维护社会和经济秩序，适应中国特色社会主义发展要求，弘扬社会主义核心价值观，根据宪法，制定本法。

法宝联想

法律法规　规范性文件（6）法规解读（1）法律动态（5）

司法案例　司法案例（38874）案例报道（3）仲裁案例（6）

法学期刊　法学期刊（334）

法宝律师　律所实务（13）

专题参考　专题参考（77）

第二条

民法调整平等主体的自然人、法人和非法人组织之间的人身关系和财产关系。

法宝联想

法律法规　司法解释（1）规范性文件（1）草案（1）法规解读（2）法律动态（4）

司法案例　司法案例（14837）案例报道（3）仲裁案例（2）

法学期刊　法学期刊（398）

法宝律师　律所实务（5）

专题参考　专题参考（100）

第三条

民事主体的人身权利、财产权利以及其他合法权益受法律保护，任何组织或者个人不得侵犯。

法宝联想

法律法规　规范性文件（5）草案（1）法律动态（4）

司法案例　司法案例（141545）案例报道（9）仲裁案例（2）

法学期刊　法学期刊（263）

法宝律师　律所实务（5）

专题参考　专题参考（54）

第四条　民事主体在民事活动中的法律地位一律平等。

法宝联想

法律法规　司法解释（3）规范性文件（6）法规解读（2）法律动态（11）

司法案例　司法案例（128350）案例报道（6）仲裁案例（7）

法学期刊　法学期刊（537）

法宝律师　律所实务（22）

专题参考　专题参考（152）

第五条

民事主体从事民事活动，应当遵循自愿原则，按照自己的意思设立、变更、终止民事法律关系。

法宝联想

法律法规　司法解释（1）规范性文件（5）草案（2）法规解读（4）法律动态（7）

司法案例　司法案例（231939）案例报道（12）仲裁案例（7）

法学期刊　法学期刊（357）

法宝律师　律所实务（16）

专题参考　专题参考（85）

第六条

民事主体从事民事活动，应当遵循公平原则，合理确定各方的权利和义务。

法宝联想

法律法规　规范性文件（8）草案（1）法律动态（5）

司法案例　司法案例（146154）案例报道（9）仲裁案例（20）

法学期刊　法学期刊（399）

法宝律师　律所实务（13）

专题参考　专题参考（89）

第七条

民事主体从事民事活动，应当遵循诚信原则，秉持诚实，恪守承诺。

法宝联想

法律法规　法律（1）法律解释（1）规范性文件（13）草案（2）法规解读（1）法律动态（21）

司法案例　司法案例（204532）案例报道（40）仲裁案例（22）

法学期刊　法学期刊（684）

法宝律师　律所实务（23）

专题参考　专题参考（163）

第八条　民事主体从事民事活动，不得违反法律，不得违背公序良俗。

法宝联想

法律法规　规范性文件（13）法律动态（9）

司法案例　司法案例（37099）案例报道（29）仲裁案例（4）

法学期刊　法学期刊（337）

法宝律师　律所实务（8）

专题参考　专题参考（55）

第九条　民事主体从事民事活动，应当有利于节约资源、保护生态环境。

法宝联想

法律法规　规范性文件（14）法规解读（1）法律动态（16）

司法案例　司法案例（10963）案例报道（37）仲裁案例（2）

法学期刊　法学期刊（485）

法宝律师　律所实务（20）

专题参考　专题参考（84）

第十条

处理民事纠纷，应当依照法律；法律没有规定的，可以适用习惯，但是不得违背公序良俗。

法宝联想

法律法规　司法解释（1）规范性文件（12）法规解读（2）法律动态（7）

司法案例　司法案例（10946）案例报道（13）

法学期刊　法学期刊（621）

法宝律师　律所实务（14）

专题参考　专题参考（78）

第十一条　其他法律对民事关系有特别规定的，依照其规定。

法宝联想

法律法规　规范性文件（8）法律动态（1）

司法案例　司法案例（7428）案例报道（1）仲裁案例（1）

法学期刊　法学期刊（206）

法宝律师　律所实务（11）

专题参考　专题参考（49）

第十二条

中华人民共和国领域内的民事活动，适用中华人民共和国法律。法律另有规定的，依照其规定。

法宝联想

法律法规　规范性文件（5）草案（1）法规解读（1）法律动态（1）

司法案例　司法案例（6847）案例报道（1）仲裁案例（1）

法学期刊　法学期刊（150）

法宝律师　律所实务（4）

专题参考　专题参考（41）

第二章　自然人

第一节　民事权利能力和民事行为能力

第十三条

自然人从出生时起到死亡时止，具有民事权利能力，依法享有民事权利，承担民事义务。

法宝联想

法律法规　规范性文件（4）法律动态（1）

司法案例　司法案例（13177）仲裁案例（2）

法学期刊　法学期刊（141）

法宝律师　律所实务（2）

专题参考　专题参考（38）

第十四条　自然人的民事权利能力一律平等。

法宝联想

法律法规　草案（1）法律动态（1）合同范本（1）

司法案例　司法案例（4264）仲裁案例（1）

法学期刊　法学期刊（59）

法宝律师　律所实务（2）

专题参考　专题参考（24）

第十五条

自然人的出生时间和死亡时间，以出生证明、死亡证明记载的时间为准；没有出生证明、死亡证明的，以户籍登记或者其他有效身份登记记载的时间为准。有其他证据足以推翻以上记载时间的，以该证据证明的时间为准。

法宝联想

法律法规　规范性文件（6）

司法案例　司法案例（3414）案例报道（1）

法学期刊　法学期刊（56）

法宝律师　律所实务（2）

专题参考　专题参考（24）

第十六条

涉及遗产继承、接受赠与等胎儿利益保护的，胎儿视为具有民事权利能力。但是，胎儿娩出时为死体的，其民事权利能力自始不存在。

法宝联想

法律法规　法律（1）司法解释（3）地方性法规（6）规范性文件（55）法律动态（5）

司法案例　司法案例（11314）案例报道（12）仲裁案例（1）

法学期刊　法学期刊（276）

法宝律师　律所实务（6）

专题参考　专题参考（57）

第十七条

十八周岁以上的自然人为成年人。不满十八周岁的自然人为未成年人。

法宝联想

法律法规　司法解释（3）规范性文件（34）

司法案例　司法案例（20229）案例报道（3）仲裁案例（1）

法学期刊　法学期刊（108）

法宝律师　律所实务（3）

专题参考　专题参考（29）

第十八条　成年人为完全民事行为能力人，可以独立实施民事法律行为。

十六周岁以上的未成年人，以自己的劳动收入为主要生活来源的，视为完全民事行为能力人。

法宝联想

法律法规 地方性法规（2）规范性文件（16）法规解读（2）法律动态（3）

司法案例 司法案例（17999）案例报道（9）仲裁案例（1）

法学期刊 法学期刊（217）

法宝律师 律所实务（7）

专题参考 专题参考（41）

第十九条

八周岁以上的未成年人为限制民事行为能力人，实施民事法律行为由其法定代理人代理或者经其法定代理人同意、追认；但是，可以独立实施纯获利益的民事法律行为或者与其年龄、智力相适应的民事法律行为。

法宝联想

法律法规 地方性法规（2）规范性文件（9）法律动态（7）

司法案例 司法案例（13396）案例报道（13）仲裁案例（1）

法学期刊 法学期刊（114）

法宝律师 律所实务（5）

专题参考 专题参考（28）

第二十条

不满八周岁的未成年人为无民事行为能力人，由其法定代理人代理实施民事法律行为。

法宝联想

法律法规 司法解释（1）规范性文件（3）法律动态（3）

司法案例 司法案例（11997）案例报道（5）仲裁案例（1）

法学期刊　法学期刊（65）

法宝律师　律所实务（3）

专题参考　专题参考（21）

第二十一条

不能辨认自己行为的成年人为无民事行为能力人，由其法定代理人代理实施民事法律行为。

八周岁以上的未成年人不能辨认自己行为的，适用前款规定。

法宝联想

法律法规　司法解释（3）

司法案例　司法案例（13875）案例报道（1）

法学期刊　法学期刊（66）

法宝律师　律所实务（1）

专题参考　专题参考（19）

第二十二条

不能完全辨认自己行为的成年人为限制民事行为能力人，实施民事法律行为由其法定代理人代理或者经其法定代理人同意、追认；但是，可以独立实施纯获利益的民事法律行为或者与其智力、精神健康状况相适应的民事法律行为。

法宝联想

法律法规　规范性文件（1）法律动态（3）

司法案例　司法案例（4715）案例报道（3）仲裁案例（2）

法学期刊　法学期刊（70）

法宝律师　律所实务（3）

专题参考　专题参考（18）

第二十三条

无民事行为能力人、限制民事行为能力人的监护人是其法定代理人。

法宝联想

法律法规 司法解释（1）规范性文件（9）法律动态（2）合同范本（1）

司法案例 司法案例（14335）案例报道（3）

法学期刊 法学期刊（59）

法宝律师 律所实务（4）

专题参考 专题参考（14）

第二十四条

不能辨认或者不能完全辨认自己行为的成年人，其利害关系人或者有关组织，可以向人民法院申请认定该成年人为无民事行为能力人或者限制民事行为能力人。

被人民法院认定为无民事行为能力人或者限制民事行为能力人的，经本人、利害关系人或者有关组织申请，人民法院可以根据其智力、精神健康恢复的状况，认定该成年人恢复为限制民事行为能力人或者完全民事行为能力人。

本条规定的有关组织包括：居民委员会、村民委员会、学校、医疗机构、妇女联合会、残疾人联合会、依法设立的老年人组织、民政部门等。

法宝联想

法律法规 规范性文件（3）

司法案例 司法案例（31673）案例报道（3）

法学期刊 法学期刊（65）

专题参考 专题参考（9）

第二十五条

自然人以户籍登记或者其他有效身份登记记载的居所为住所；经常居所与住所不一致的，经常居所视为住所。

法宝联想

法律法规　规范性文件（2）法律动态（2）

司法案例　司法案例（3631）案例报道（1）

法学期刊　法学期刊（34）

法宝律师　律所实务（4）

专题参考　专题参考（6）

第二节　监护

第二十六条　父母对未成年子女负有抚养、教育和保护的义务。

成年子女对父母负有赡养、扶助和保护的义务。

法宝联想

法律法规　地方政府规章（1）规范性文件（19）法规解读（1）法律动态（7）

司法案例　司法案例（9885）案例报道（18）

法学期刊　法学期刊（140）

法宝律师　律所实务（9）

专题参考　专题参考（20）

第二十七条　父母是未成年子女的监护人。

未成年人的父母已经死亡或者没有监护能力的，由下列有监护能力的人按顺序担任监护人：

（一）祖父母、外祖父母；

（二）兄、姐；

（三）其他愿意担任监护人的个人或者组织，但是须经未成年人住所地的居民委员会、村民委员会或者民政部门同意。

法宝联想

法律法规 司法解释（5）规范性文件（26）法规解读（1）法律动态（7）

司法案例 司法案例（6992）案例报道（11）

法学期刊 法学期刊（148）

法宝律师 律所实务（3）

专题参考 专题参考（27）

第二十八条

无民事行为能力或者限制民事行为能力的成年人，由下列有监护能力的人按顺序担任监护人：

（一）配偶；

（二）父母、子女；

（三）其他近亲属；

（四）其他愿意担任监护人的个人或者组织，但是须经被监护人住所地的居民委员会、村民委员会或者民政部门同意。

法宝联想

法律法规 司法解释（4）规范性文件（11）

司法案例 司法案例（57797）案例报道（4）

法学期刊 法学期刊（82）

法宝律师 律所实务（5）

专题参考 专题参考（17）

第二十九条　被监护人的父母担任监护人的，可以通过遗嘱指定监护人。

法宝联想

法律法规　地方性法规（2）规范性文件（2）

司法案例　司法案例（20606）案例报道（1）

法学期刊　法学期刊（70）

法宝律师　律所实务（5）

专题参考　专题参考（22）

第三十条

依法具有监护资格的人之间可以协议确定监护人。协议确定监护人应当尊重被监护人的真实意愿。

法宝联想

法律法规　司法解释（1）规范性文件（2）

司法案例　司法案例（89049）仲裁案例（3）

法学期刊　法学期刊（69）

法宝律师　律所实务（2）

专题参考　专题参考（23）

第三十一条

对监护人的确定有争议的，由被监护人住所地的居民委员会、村民委员会或者民政部门指定监护人，有关当事人对指定不服的，可以向人民法院申请指定监护人；有关当事人也可以直接向人民法院申请指定监护人。

居民委员会、村民委员会、民政部门或者人民法院应当尊重被监护人的真实意愿，按照最有利于被监护人的原则在依法具有监护资格的人中指定监护人。

依据本条第一款规定指定监护人前，被监护人的人身权利、财产权利以及其他合法权益处于无人保护状态的，由被监护人住所地的居民委员会、村民委员会、法律规定的有关组织或者民政部门担任临时监护人。

监护人被指定后，不得擅自变更；擅自变更的，不免除被指定的监护人的责任。

法宝联想

法律法规　司法解释（4）规范性文件（15）法律动态（3）

司法案例　司法案例（53211）案例报道（4）

法学期刊　法学期刊（100）

法宝律师　律所实务（2）

专题参考　专题参考（13）

第三十二条

没有依法具有监护资格的人的，监护人由民政部门担任，也可以由具备履行监护职责条件的被监护人住所地的居民委员会、村民委员会担任。

法宝联想

法律法规　司法解释（3）规范性文件（17）法律动态（1）

司法案例　司法案例（28097）案例报道（4）仲裁案例（1）

法学期刊　法学期刊（73）

法宝律师　律所实务（4）

专题参考　专题参考（16）

第三十三条

具有完全民事行为能力的成年人，可以与其近亲属、其他愿意担任监护人的个人

或者组织事先协商，以书面形式确定自己的监护人，在自己丧失或者部分丧失民事行为能力时，由该监护人履行监护职责。

法宝联想

法律法规 司法解释（1）规范性文件（4）法规解读（2）

司法案例 司法案例（1880）案例报道（2）仲裁案例（1）

法学期刊 法学期刊（123）

法宝律师 律所实务（3）

专题参考 专题参考（23）

第三十四条

监护人的职责是代理被监护人实施民事法律行为，保护被监护人的人身权利、财产权利以及其他合法权益等。

监护人依法履行监护职责产生的权利，受法律保护。

监护人不履行监护职责或者侵害被监护人合法权益的，应当承担法律责任。

因发生突发事件等紧急情况，监护人暂时无法履行监护职责，被监护人的生活处于无人照料状态的，被监护人住所地的居民委员会、村民委员会或者民政部门应当为被监护人安排必要的临时生活照料措施。

法宝联想

法律法规 规范性文件（7）法律动态（4）

司法案例 司法案例（24457）案例报道（8）仲裁案例（1）

法学期刊 法学期刊（137）

法宝律师 律所实务（1）

专题参考 专题参考（17）

第三十五条

监护人应当按照最有利于被监护人的原则履行监护职责。监护人除为维护被监护人利益外，不得处分被监护人的财产。

未成年人的监护人履行监护职责，在作出与被监护人利益有关的决定时，应当根据被监护人的年龄和智力状况，尊重被监护人的真实意愿。

成年人的监护人履行监护职责，应当最大程度地尊重被监护人的真实意愿，保障并协助被监护人实施与其智力、精神健康状况相适应的民事法律行为。对被监护人有能力独立处理的事务，监护人不得干涉。

法宝联想
法律法规　司法解释（2）规范性文件（7）法律动态（8）
司法案例　司法案例（78933）案例报道（13）仲裁案例（1）
法学期刊　法学期刊（164）
法宝律师　律所实务（7）
专题参考　专题参考（25）

第三十六条

监护人有下列情形之一的，人民法院根据有关个人或者组织的申请，撤销其监护人资格，安排必要的临时监护措施，并按照最有利于被监护人的原则依法指定监护人：

（一）实施严重损害被监护人身心健康的行为；

（二）怠于履行监护职责，或者无法履行监护职责且拒绝将监护职责部分或者全部委托给他人，导致被监护人处于危困状态；

（三）实施严重侵害被监护人合法权益的其他行为。

本条规定的有关个人、组织包括：其他依法具有监护资格的人，居民委员会、村民委员会、学校、医疗机构、妇女联合会、残疾人联合会、未成年人保护组织、依法设立的老年人组织、民政部门等。

前款规定的个人和民政部门以外的组织未及时向人民法院申请撤销监护人资格的，民政部门应当向人民法院申请。

法宝联想

法律法规　司法解释（2）规范性文件（22）法律动态（8）

司法案例　司法案例（10668）案例报道（23）仲裁案例（2）

法学期刊　法学期刊（216）

法宝律师　律所实务（14）

专题参考　专题参考（41）

第三十七条

依法负担被监护人抚养费、赡养费、扶养费的父母、子女、配偶等，被人民法院撤销监护人资格后，应当继续履行负担的义务。

法宝联想

法律法规　行政法规（1）地方政府规章（2）规范性文件（6）法律动态（4）

司法案例　司法案例（2170）案例报道（2）仲裁案例（3）

法学期刊　法学期刊（114）

法宝律师　律所实务（8）

专题参考　专题参考（30）

第三十八条

被监护人的父母或者子女被人民法院撤销监护人资格后，除对被监护人实施故意犯罪的外，确有悔改表现的，经其申请，人民法院可以在尊重被监护人真实意愿的前提下，视情况恢复其监护人资格，人民法院指定的监护人与被监护人的监护关系同时终止。

法宝联想

法律法规　司法解释（1）规范性文件（4）法律动态（1）

司法案例　司法案例（3826）案例报道（1）

法学期刊　法学期刊（85）

法宝律师　律所实务（2）

专题参考　专题参考（23）

第三十九条　有下列情形之一的，监护关系终止：

（一）被监护人取得或者恢复完全民事行为能力；

（二）监护人丧失监护能力；

（三）被监护人或者监护人死亡；

（四）人民法院认定监护关系终止的其他情形。

监护关系终止后，被监护人仍然需要监护的，应当依法另行确定监护人。

法宝联想

法律法规　司法解释（1）规范性文件（1）法规解读（1）

司法案例　司法案例（1055）案例报道（1）

法学期刊　法学期刊（36）

法宝律师　律所实务（1）

专题参考　专题参考（5）

第三节　宣告失踪和宣告死亡

第四十条

自然人下落不明满二年的，利害关系人可以向人民法院申请宣告该自然人为失踪人。

法宝联想

法律法规　司法解释（1）规范性文件（6）法律动态（2）

司法案例　司法案例（4418）案例报道（1）

法学期刊　法学期刊（27）

法宝律师　律所实务（1）

专题参考　专题参考（10）

第四十一条

自然人下落不明的时间自其失去音讯之日起计算。战争期间下落不明的，下落不明的时间自战争结束之日或者有关机关确定的下落不明之日起计算。

法宝联想

法律法规　规范性文件（17）法规解读（1）法律动态（1）

司法案例　司法案例（3127）案例报道（1）仲裁案例（1）

法学期刊　法学期刊（56）

法宝律师　律所实务（3）

专题参考　专题参考（12）

第四十二条

失踪人的财产由其配偶、成年子女、父母或者其他愿意担任财产代管人的人代管。

代管有争议，没有前款规定的人，或者前款规定的人无代管能力的，由人民法院指定的人代管。

法宝联想

法律法规 司法解释（3）规范性文件（2）

司法案例 司法案例（3358）仲裁案例（1）

法学期刊 法学期刊（67）

法宝律师 律所实务（2）

专题参考 专题参考（16）

第四十三条 财产代管人应当妥善管理失踪人的财产，维护其财产权益。

失踪人所欠税款、债务和应付的其他费用，由财产代管人从失踪人的财产中支付。

财产代管人因故意或者重大过失造成失踪人财产损失的，应当承担赔偿责任。

法宝联想

法律法规 规范性文件（4）

司法案例 司法案例（74932）案例报道（1）仲裁案例（3）

法学期刊 法学期刊（147）

法宝律师 律所实务（7）

专题参考 专题参考（50）

第四十四条

财产代管人不履行代管职责、侵害失踪人财产权益或者丧失代管能力的，失踪人的利害关系人可以向人民法院申请变更财产代管人。

财产代管人有正当理由的，可以向人民法院申请变更财产代管人。

人民法院变更财产代管人的，变更后的财产代管人有权请求原财产代管人及时移交有关财产并报告财产代管情况。

法宝联想

法律法规 规范性文件（3）

司法案例 司法案例（14101）仲裁案例（2）

法学期刊 法学期刊（52）

法宝律师 律所实务（9）

专题参考 专题参考（22）

第四十五条

失踪人重新出现，经本人或者利害关系人申请，人民法院应当撤销失踪宣告。

失踪人重新出现，有权请求财产代管人及时移交有关财产并报告财产代管情况。

法宝联想

法律法规 规范性文件（2）法律动态（2）

司法案例 司法案例（1443）

法学期刊 法学期刊（20）

法宝律师 律所实务（1）

专题参考 专题参考（7）

第四十六条

自然人有下列情形之一的，利害关系人可以向人民法院申请宣告该自然人死亡：

（一）下落不明满四年；

（二）因意外事件，下落不明满二年。

因意外事件下落不明，经有关机关证明该自然人不可能生存的，申请宣告死亡不受二年时间的限制。

法宝联想

法律法规 司法解释（1）规范性文件（2）

司法案例 司法案例（9139）案例报道（2）

法学期刊 法学期刊（40）

法宝律师 律所实务（3）

专题参考 专题参考（11）

第四十七条

对同一自然人，有的利害关系人申请宣告死亡，有的利害关系人申请宣告失踪，符合本法规定的宣告死亡条件的，人民法院应当宣告死亡。

法宝联想

法律法规 规范性文件（3）草案（1）法规解读（1）法律动态（1）

司法案例 司法案例（4969）

法学期刊 法学期刊（24）

法宝律师 律所实务（3）

专题参考 专题参考（11）

第四十八条

被宣告死亡的人，人民法院宣告死亡的判决作出之日视为其死亡的日期；因意外事件下落不明宣告死亡的，意外事件发生之日视为其死亡的日期。

法宝联想

法律法规 司法解释（4）规范性文件（6）草案（1）

司法案例 司法案例（3036）仲裁案例（1）

法学期刊 法学期刊（65）

法宝律师　律所实务（3）

专题参考　专题参考（18）

第四十九条

自然人被宣告死亡但是并未死亡的，不影响该自然人在被宣告死亡期间实施的民事法律行为的效力。

法宝联想

法律法规　司法解释（1）规范性文件（3）草案（1）法律动态（1）

司法案例　司法案例（906）案例报道（1）

法学期刊　法学期刊（66）

法宝律师　律所实务（3）

专题参考　专题参考（8）

第五十条

被宣告死亡的人重新出现，经本人或者利害关系人申请，人民法院应当撤销死亡宣告。

法宝联想

法律法规　司法解释（1）规范性文件（3）

司法案例　司法案例（5259）

法学期刊　法学期刊（70）

法宝律师　律所实务（4）

专题参考　专题参考（20）

第五十一条

被宣告死亡的人的婚姻关系，自死亡宣告之日起消除。死亡宣告被撤销的，婚姻关系自撤销死亡宣告之日起自行恢复。但是，其配偶再婚或者向婚姻登记机关书面声明不愿意恢复的除外。

法宝联想

司法案例　司法案例（1645）仲裁案例（1）

法学期刊　法学期刊（40）

法宝律师　律所实务（5）

专题参考　专题参考（11）

第五十二条

被宣告死亡的人在被宣告死亡期间，其子女被他人依法收养的，在死亡宣告被撤销后，不得以未经本人同意为由主张收养行为无效。

法宝联想

法律法规　规范性文件（1）法规解读（1）

司法案例　司法案例（8565）仲裁案例（3）

法学期刊　法学期刊（57）

法宝律师　律所实务（5）

专题参考　专题参考（19）

第五十三条

被撤销死亡宣告的人有权请求依照本法第六编取得其财产的民事主体返还财产；无法返还的，应当给予适当补偿。

利害关系人隐瞒真实情况，致使他人被宣告死亡而取得其财产的，除应当返还财产外，还应当对由此造成的损失承担赔偿责任。

法宝联想

法律法规　规范性文件（2）法规解读（1）法律动态（1）

司法案例　司法案例（2838）仲裁案例（1）

法学期刊　法学期刊（28）

专题参考　专题参考（4）

第四节　个体工商户和农村承包经营户

第五十四条

自然人从事工商业经营，经依法登记，为个体工商户。个体工商户可以起字号。

法宝联想

法律法规　规范性文件（3）草案（1）法规解读（1）法律动态（1）

司法案例　司法案例（23027）案例报道（1）仲裁案例（3）

法学期刊　法学期刊（109）

法宝律师　律所实务（5）

专题参考　专题参考（29）

第五十五条

农村集体经济组织的成员，依法取得农村土地承包经营权，从事家庭承包经营的，为农村承包经营户。

法宝联想

法律法规　法律（2）司法解释（2）部门规章（1）规范性文件（11）法规解读（1）法律动态（2）

司法案例　司法案例（42992）案例报道（2）仲裁案例（2）

法学期刊　法学期刊（275）

法宝律师　律所实务（14）

专题参考　专题参考（66）

第五十六条

个体工商户的债务，个人经营的，以个人财产承担；家庭经营的，以家庭财产承担；无法区分的，以家庭财产承担。

农村承包经营户的债务，以从事农村土地承包经营的农户财产承担；事实上由农户部分成员经营的，以该部分成员的财产承担。

法宝联想

法律法规 司法解释（1）规范性文件（5）法律动态（3）

司法案例 司法案例（55669）案例报道（5）

法学期刊 法学期刊（90）

法宝律师 律所实务（7）

专题参考 专题参考（20）

第三章　法人

第一节　一般规定

第五十七条

法人是具有民事权利能力和民事行为能力，依法独立享有民事权利和承担民事义务的组织。

法宝联想

法律法规 司法解释（1）规范性文件（3）法规解读（1）法律动态（2）

司法案例 司法案例（30638）案例报道（2）仲裁案例（1）

法学期刊 法学期刊（98）

法宝律师 律所实务（7）

专题参考 专题参考（24）

第五十八条　法人应当依法成立。

法人应当有自己的名称、组织机构、住所、财产或者经费。法人成立的具体条件和程序，依照法律、行政法规的规定。

设立法人，法律、行政法规规定须经有关机关批准的，依照其规定。

法宝联想

法律法规　规范性文件（20）法规解读（1）法律动态（6）

司法案例　司法案例（33992）案例报道（4）仲裁案例（12）

法学期刊　法学期刊（550）

法宝律师　律所实务（17）

专题参考　专题参考（150）

第五十九条

法人的民事权利能力和民事行为能力，从法人成立时产生，到法人终止时消灭。

法宝联想

法律法规　规范性文件（2）法律动态（1）

司法案例　司法案例（12264）仲裁案例（6）

法学期刊　法学期刊（187）

法宝律师　律所实务（9）

专题参考　专题参考（47）

第六十条　法人以其全部财产独立承担民事责任。

法宝联想

法律法规　司法解释（1）规范性文件（3）法律动态（1）

司法案例　司法案例（12162）案例报道（1）仲裁案例（2）

法学期刊　法学期刊（64）

法宝律师　律所实务（1）

专题参考　专题参考（18）

第六十一条

依照法律或者法人章程的规定，代表法人从事民事活动的负责人，为法人的法定代表人。

法定代表人以法人名义从事的民事活动，其法律后果由法人承受。

法人章程或者法人权力机构对法定代表人代表权的限制，不得对抗善意相对人。

法宝联想

法律法规　司法解释（5）地方政府规章（1）规范性文件（14）法规解读（4）法律动态（2）

司法案例　司法案例（43888）案例报道（3）仲裁案例（11）

法学期刊　法学期刊（364）

法宝律师　律所实务（50）

专题参考　专题参考（127）

第六十二条

法定代表人因执行职务造成他人损害的，由法人承担民事责任。

法人承担民事责任后，依照法律或者法人章程的规定，可以向有过错的法定代表人追偿。

法宝联想

法律法规　规范性文件（1）法律动态（1）

司法案例　司法案例（8109）

法学期刊　法学期刊（108）

法宝律师　律所实务（18）

专题参考　专题参考（37）

第六十三条

法人以其主要办事机构所在地为住所。依法需要办理法人登记的，应当将主要办事机构所在地登记为住所。

法宝联想

法律法规　司法解释（1）规范性文件（7）草案（1）法规解读（2）

司法案例　司法案例（46783）仲裁案例（5）

法学期刊　法学期刊（127）

法宝律师　律所实务（10）

专题参考　专题参考（40）

第六十四条

法人存续期间登记事项发生变化的，应当依法向登记机关申请变更登记。

法宝联想

法律法规　合同范本（1）

司法案例　司法案例（5741）仲裁案例（1）

法学期刊　法学期刊（23）

法宝律师　律所实务（2）

专题参考　专题参考（11）

第六十五条

法人的实际情况与登记的事项不一致的，不得对抗善意相对人。

法宝联想

法律法规　司法解释（2）规范性文件（5）法律动态（4）

司法案例　司法案例（10485）案例报道（2）仲裁案例（6）

法学期刊　法学期刊（134）

法宝律师　律所实务（11）

专题参考　专题参考（35）

第六十六条　登记机关应当依法及时公示法人登记的有关信息。

法宝联想

法律法规　规范性文件（9）法律动态（3）

司法案例　司法案例（23009）案例报道（2）仲裁案例（8）

法学期刊　法学期刊（194）

法宝律师　律所实务（14）

专题参考　专题参考（70）

第六十七条　法人合并的，其权利和义务由合并后的法人享有和承担。

法人分立的，其权利和义务由分立后的法人享有连带债权，承担连带债务，但是债权人和债务人另有约定的除外。

法宝联想

法律法规　规范性文件（2）法律动态（1）

司法案例　司法案例（11773）案例报道（2）仲裁案例（1）

法学期刊　法学期刊（56）

法宝律师　律所实务（5）

专题参考　专题参考（10）

第六十八条　有下列原因之一并依法完成清算、注销登记的，法人终止：

（一）法人解散；

（二）法人被宣告破产；

（三）法律规定的其他原因。

法人终止，法律、行政法规规定须经有关机关批准的，依照其规定。

法宝联想

法律法规　司法解释（1）规范性文件（3）法规解读（1）

司法案例　司法案例（8541）仲裁案例（3）

法学期刊　法学期刊（37）

法宝律师　律所实务（11）

专题参考　专题参考（26）

第六十九条　有下列情形之一的，法人解散：

（一）法人章程规定的存续期间届满或者法人章程规定的其他解散事由出现；

（二）法人的权力机构决议解散；

（三）因法人合并或者分立需要解散；

（四）法人依法被吊销营业执照、登记证书，被责令关闭或者被撤销；

（五）法律规定的其他情形。

法宝联想

法律法规　规范性文件（4）法规解读（1）

司法案例　司法案例（2945）仲裁案例（1）

法学期刊　法学期刊（35）

法宝律师　律所实务（2）

专题参考　专题参考（16）

第七十条

法人解散的，除合并或者分立的情形外，清算义务人应当及时组成清算组进行清算。

法人的董事、理事等执行机构或者决策机构的成员为清算义务人。法律、行政法规另有规定的，依照其规定。

清算义务人未及时履行清算义务，造成损害的，应当承担民事责任；主管机关或者利害关系人可以申请人民法院指定有关人员组成清算组进行清算。

法宝联想

法律法规 司法解释（2）规范性文件（5）法律动态（2）

司法案例 司法案例（3122）案例报道（2）仲裁案例（1）

法学期刊 法学期刊（69）

法宝律师 律所实务（32）

专题参考 专题参考（32）

第七十一条

法人的清算程序和清算组职权，依照有关法律的规定；没有规定的，参照适用公司法律的有关规定。

法宝联想

法律法规 规范性文件（3）草案（1）法律动态（1）

司法案例 司法案例（27408）

法学期刊 法学期刊（96）

法宝律师 律所实务（4）

专题参考 专题参考（35）

第七十二条 清算期间法人存续，但是不得从事与清算无关的活动。

法人清算后的剩余财产，按照法人章程的规定或者法人权力机构的决议处理。法律另有规定的，依照其规定。

清算结束并完成法人注销登记时，法人终止；依法不需要办理法人登记的，清算结束时，法人终止。

法宝联想

法律法规 司法解释（4）规范性文件（6）法规解读（1）法律动态（3）

司法案例 司法案例（21367）仲裁案例（2）

法学期刊 法学期刊（169）

法宝律师 律所实务（7）

专题参考 专题参考（49）

第七十三条

法人被宣告破产的，依法进行破产清算并完成法人注销登记时，法人终止。

法宝联想

法律法规 法律动态（2）

司法案例 司法案例（2227）仲裁案例（1）

法学期刊 法学期刊（58）

法宝律师 律所实务（1）

专题参考 专题参考（17）

第七十四条

法人可以依法设立分支机构。法律、行政法规规定分支机构应当登记的，依照其规定。

分支机构以自己的名义从事民事活动，产生的民事责任由法人承担；也可以先以该分支机构管理的财产承担，不足以承担的，由法人承担。

法宝联想

法律法规 规范性文件（8）草案（1）法律动态（2）

司法案例 司法案例（64778）案例报道（3）仲裁案例（4）

法学期刊 法学期刊（137）

法宝律师 律所实务（11）

专题参考 专题参考（32）

第七十五条

设立人为设立法人从事的民事活动，其法律后果由法人承受；法人未成立的，其法律后果由设立人承受，设立人为二人以上的，享有连带债权，承担连带债务。

设立人为设立法人以自己的名义从事民事活动产生的民事责任，第三人有权选择请求法人或者设立人承担。

法宝联想

法律法规 司法解释（1）规范性文件（2）法律动态（2）

司法案例 司法案例（45206）案例报道（2）仲裁案例（1）

法学期刊 法学期刊（101）

法宝律师 律所实务（6）

专题参考 专题参考（28）

第二节 营利法人

第七十六条

以取得利润并分配给股东等出资人为目的成立的法人，为营利法人。

营利法人包括有限责任公司、股份有限公司和其他企业法人等。

法宝联想

法律法规 规范性文件（4）法规解读（1）法律动态（1）

司法案例 司法案例（5824）

法学期刊 法学期刊（99）

法宝律师　律所实务（4）

专题参考　专题参考（13）

第七十七条　营利法人经依法登记成立。

法宝联想

法律法规　规范性文件（1）

司法案例　司法案例（957）

法学期刊　法学期刊（23）

专题参考　专题参考（4）

第七十八条

依法设立的营利法人，由登记机关发给营利法人营业执照。营业执照签发日期为营利法人的成立日期。

法宝联想

法律法规　规范性文件（6）法律动态（2）

司法案例　司法案例（22100）案例报道（1）

法学期刊　法学期刊（56）

法宝律师　律所实务（5）

专题参考　专题参考（23）

第七十九条　设立营利法人应当依法制定法人章程。

法宝联想

法律法规　规范性文件（6）法律动态（5）

司法案例　司法案例（939）案例报道（1）

法学期刊　法学期刊（89）

法宝律师　律所实务（7）

专题参考　专题参考（16）

第八十条 营利法人应当设权力机构。

权力机构行使修改法人章程，选举或者更换执行机构、监督机构成员，以及法人章程规定的其他职权。

法宝联想

法律法规 规范性文件（1）法规解读（1）

司法案例 司法案例（5560）

法学期刊 法学期刊（92）

法宝律师 律所实务（3）

专题参考 专题参考（23）

第八十一条 营利法人应当设执行机构。

执行机构行使召集权力机构会议，决定法人的经营计划和投资方案，决定法人内部管理机构的设置，以及法人章程规定的其他职权。

执行机构为董事会或者执行董事的，董事长、执行董事或者经理按照法人章程的规定担任法定代表人；未设董事会或者执行董事的，法人章程规定的主要负责人为其执行机构和法定代表人。

法宝联想

法律法规 法规解读（1）

司法案例 司法案例（1952）

法学期刊 法学期刊（75）

法宝律师 律所实务（5）

专题参考 专题参考（27）

第八十二条

营利法人设监事会或者监事等监督机构的，监督机构依法行使检查法人财务，监督执行机构成员、高级管理人员执行法人职务的行为，以及法人章程规定的其他职权。

法宝联想

司法案例 司法案例（522）案例报道（1）

法学期刊 法学期刊（54）

专题参考 专题参考（8）

第八十三条

营利法人的出资人不得滥用出资人权利损害法人或者其他出资人的利益；滥用出资人权利造成法人或者其他出资人损失的，应当依法承担民事责任。

营利法人的出资人不得滥用法人独立地位和出资人有限责任损害法人债权人的利益；滥用法人独立地位和出资人有限责任，逃避债务，严重损害法人债权人的利益的，应当对法人债务承担连带责任。

法宝联想

法律法规 规范性文件（9）法律动态（3）

司法案例 司法案例（59089）案例报道（5）

法学期刊 法学期刊（124）

法宝律师 律所实务（11）

专题参考 专题参考（43）

第八十四条

营利法人的控股出资人、实际控制人、董事、监事、高级管理人员不得利用其关联关系损害法人的利益；利用关联关系造成法人损失的，应当承担赔偿责任。

法宝联想

法律法规　司法解释（2）规范性文件（4）法律动态（4）

司法案例　司法案例（991390）案例报道（2）仲裁案例（2）

法学期刊　法学期刊（80）

法宝律师　律所实务（10）

专题参考　专题参考（75）

第八十五条

营利法人的权力机构、执行机构作出决议的会议召集程序、表决方式违反法律、行政法规、法人章程，或者决议内容违反法人章程的，营利法人的出资人可以请求人民法院撤销该决议。但是，营利法人依据该决议与善意相对人形成的民事法律关系不受影响。

法宝联想

法律法规　司法解释（3）规范性文件（3）法律动态（1）

司法案例　司法案例（40141）案例报道（2）仲裁案例（2）

法学期刊　法学期刊（148）

法宝律师　律所实务（11）

专题参考　专题参考（51）

第八十六条

营利法人从事经营活动，应当遵守商业道德，维护交易安全，接受政府和社会的监督，承担社会责任。

法宝联想

法律法规　规范性文件（1）法律动态（1）

司法案例　司法案例（4525）案例报道（1）仲裁案例（1）

法学期刊　法学期刊（81）

法宝律师　律所实务（4）

专题参考　专题参考（16）

第三节　非营利法人

第八十七条

为公益目的或者其他非营利目的成立，不向出资人、设立人或者会员分配所取得利润的法人，为非营利法人。

非营利法人包括事业单位、社会团体、基金会、社会服务机构等。

法宝联想

法律法规　规范性文件（21）草案（1）法律动态（2）

司法案例　司法案例（65210）案例报道（1）仲裁案例（1）

法学期刊　法学期刊（165）

法宝律师　律所实务（6）

专题参考　专题参考（37）

第八十八条

具备法人条件，为适应经济社会发展需要，提供公益服务设立的事业单位，经依法登记成立，取得事业单位法人资格；依法不需要办理法人登记的，从成立之日起，具有事业单位法人资格。

法宝联想

法律法规　司法解释（1）地方政府规章（1）规范性文件（3）法律动态（2）

司法案例　司法案例（44685）仲裁案例（2）

法学期刊　法学期刊（48）

法宝律师　律所实务（2）

专题参考　专题参考（21）

第八十九条

事业单位法人设理事会的，除法律另有规定外，理事会为其决策机构。事业单位法人的法定代表人依照法律、行政法规或者法人章程的规定产生。

法宝联想

法律法规　司法解释（3）规范性文件（13）法律动态（3）

司法案例　司法案例（30433）

法学期刊　法学期刊（131）

法宝律师　律所实务（16）

专题参考　专题参考（48）

第九十条

具备法人条件，基于会员共同意愿，为公益目的或者会员共同利益等非营利目的设立的社会团体，经依法登记成立，取得社会团体法人资格；依法不需要办理法人登记的，从成立之日起，具有社会团体法人资格。

法宝联想

法律法规　司法解释（2）规范性文件（2）法律动态（1）

司法案例　司法案例（957232）案例报道（2）

法学期刊　法学期刊（55）

法宝律师　律所实务（3）

专题参考　专题参考（31）

第九十一条　设立社会团体法人应当依法制定法人章程。

社会团体法人应当设会员大会或者会员代表大会等权力机构。

社会团体法人应当设理事会等执行机构。理事长或者会长等负责人按照法人章程的规定担任法定代表人。

法宝联想

法律法规　规范性文件（1）法律动态（2）

司法案例　司法案例（4243）仲裁案例（4）

法学期刊　法学期刊（69）

法宝律师　律所实务（4）

专题参考　专题参考（15）

第九十二条

具备法人条件，为公益目的以捐助财产设立的基金会、社会服务机构等，经依法登记成立，取得捐助法人资格。

依法设立的宗教活动场所，具备法人条件的，可以申请法人登记，取得捐助法人资格。法律、行政法规对宗教活动场所有规定的，依照其规定。

法宝联想

法律法规　规范性文件（20）法律动态（6）

司法案例　司法案例（129784）案例报道（10）仲裁案例（3）

法学期刊　法学期刊（223）

法宝律师　律所实务（9）

专题参考　专题参考（55）

第九十三条　设立捐助法人应当依法制定法人章程。

捐助法人应当设理事会、民主管理组织等决策机构，并设执行机构。理事长等负责人按照法人章程的规定担任法定代表人。

捐助法人应当设监事会等监督机构。

法宝联想

法律法规　司法解释（1）法律动态（2）

司法案例　司法案例（7911）仲裁案例（1）

法学期刊　法学期刊（110）

法宝律师　律所实务（2）

专题参考　专题参考（22）

第九十四条

捐助人有权向捐助法人查询捐助财产的使用、管理情况，并提出意见和建议，捐助法人应当及时、如实答复。

捐助法人的决策机构、执行机构或者法定代表人作出决定的程序违反法律、行政法规、法人章程，或者决定内容违反法人章程的，捐助人等利害关系人或者主管机关可以请求人民法院撤销该决定。但是，捐助法人依据该决定与善意相对人形成的民事法律关系不受影响。

法宝联想

法律法规　规范性文件（2）

司法案例　司法案例（860）仲裁案例（1）

法学期刊　法学期刊（71）

法宝律师　律所实务（1）

专题参考　专题参考（14）

第九十五条

为公益目的成立的非营利法人终止时，不得向出资人、设立人或者会员分配剩余财产。剩余财产应当按照法人章程的规定或者权力机构的决议用于公益目的；无法按照法人章程的规定或者权力机构的决议处理的，由主管机关主持转给宗旨相同或者相近的法人，并向社会公告。

法宝联想

- 法律法规　规范性文件（15）
- 司法案例　司法案例（721）
- 法学期刊　法学期刊（20）
- 专题参考　专题参考（1）

第四节　特别法人

第九十六条

本节规定的机关法人、农村集体经济组织法人、城镇农村的合作经济组织法人、基层群众性自治组织法人，为特别法人。

法宝联想

- 法律法规　规范性文件（6）法律动态（1）
- 司法案例　司法案例（2137）
- 法学期刊　法学期刊（118）
- 法宝律师　律所实务（4）
- 专题参考　专题参考（9）

第九十七条

有独立经费的机关和承担行政职能的法定机构从成立之日起，具有机关法人资格，可以从事为履行职能所需要的民事活动。

法宝联想

法律法规 规范性文件（1）

司法案例 司法案例（1009）

法学期刊 法学期刊（64）

专题参考 专题参考（9）

第九十八条

机关法人被撤销的，法人终止，其民事权利和义务由继任的机关法人享有和承担；没有继任的机关法人的，由作出撤销决定的机关法人享有和承担。

法宝联想

法律法规 法律动态（3）

司法案例 司法案例（127281）

法学期刊 法学期刊（100）

法宝律师 律所实务（4）

专题参考 专题参考（28）

第九十九条 农村集体经济组织依法取得法人资格。

法律、行政法规对农村集体经济组织有规定的，依照其规定。

法宝联想

法律法规 法律解释（1）规范性文件（10）草案（2）法律动态（10）

司法案例 司法案例（3773）案例报道（6）仲裁案例（8）

法学期刊 法学期刊（264）

法宝律师 律所实务（2）

专题参考 专题参考（43）

第一百条 城镇农村的合作经济组织依法取得法人资格。

法律、行政法规对城镇农村的合作经济组织有规定的，依照其规定。

法宝联想

法律法规　规范性文件（2）法律动态（9）

司法案例　司法案例（5974）案例报道（5）

法学期刊　法学期刊（137）

法宝律师　律所实务（4）

专题参考　专题参考（31）

第一百零一条

居民委员会、村民委员会具有基层群众性自治组织法人资格，可以从事为履行职能所需要的民事活动。

未设立村集体经济组织的，村民委员会可以依法代行村集体经济组织的职能。

法宝联想

法律法规　规范性文件（10）法规解读（1）法律动态（13）

司法案例　司法案例（19492）案例报道（4）

法学期刊　法学期刊（297）

法宝律师　律所实务（13）

专题参考　专题参考（56）

第四章　非法人组织

第一百零二条

非法人组织是不具有法人资格，但是能够依法以自己的名义从事民事活动的组织。

非法人组织包括个人独资企业、合伙企业、不具有法人资格的专业服务机构等。

法宝联想

法律法规　规范性文件（2）

司法案例　司法案例（3599）仲裁案例（1）

法学期刊　法学期刊（126）

法宝律师　律所实务（4）

专题参考　专题参考（18）

第一百零三条　非法人组织应当依照法律的规定登记。

设立非法人组织，法律、行政法规规定须经有关机关批准的，依照其规定。

法宝联想

司法案例　司法案例（789）

法学期刊　法学期刊（64）

法宝律师　律所实务（1）

专题参考　专题参考（16）

第一百零四条

非法人组织的财产不足以清偿债务的，其出资人或者设立人承担无限责任。法律另有规定的，依照其规定。

法宝联想

法律法规　规范性文件（1）

司法案例　司法案例（4900）仲裁案例（1）

法学期刊　法学期刊（80）

法宝律师　律所实务（1）

专题参考　专题参考（12）

第一百零五条

非法人组织可以确定一人或者数人代表该组织从事民事活动。

法宝联想

司法案例　司法案例（815）

法学期刊　法学期刊（33）

专题参考 专题参考（7）

第一百零六条 有下列情形之一的，非法人组织解散：

（一）章程规定的存续期间届满或者章程规定的其他解散事由出现；

（二）出资人或者设立人决定解散；

（三）法律规定的其他情形。

法宝联想

法律法规 司法解释（7）规范性文件（26）法规解读（2）法律动态（17）法律文书（1）

司法案例 司法案例（636667）案例报道（9）

法学期刊 法学期刊（635）

法宝律师 律所实务（25）

专题参考 专题参考（286）

第一百零七条 非法人组织解散的，应当依法进行清算。

法宝联想

法律法规 规范性文件（4）法律动态（2）

司法案例 司法案例（8300）案例报道（2）仲裁案例（1）

法学期刊 法学期刊（73）

法宝律师 律所实务（6）

专题参考 专题参考（27）

第一百零八条

非法人组织除适用本章规定外，参照适用本编第三章第一节的有关规定。

法宝联想

法律法规 规范性文件（9）法律动态（2）

司法案例 司法案例（1837735）案例报道（6）

法学期刊 法学期刊（73）

法宝律师 律所实务（4）

专题参考　专题参考（41）

第五章　民事权利

第一百零九条　自然人的人身自由、人格尊严受法律保护。

法宝联想

法律法规　规范性文件（1）法律动态（1）

司法案例　司法案例（5189）

法学期刊　法学期刊（324）

专题参考　专题参考（62）

第一百一十条

自然人享有生命权、身体权、健康权、姓名权、肖像权、名誉权、荣誉权、隐私权、婚姻自主权等权利。

法人、非法人组织享有名称权、名誉权和荣誉权。

法宝联想

法律法规　规范性文件（9）法规解读（1）法律动态（7）

司法案例　司法案例（35333）案例报道（7）

法学期刊　法学期刊（370）

法宝律师　律所实务（14）

专题参考　专题参考（59）

第一百一十一条

自然人的个人信息受法律保护。任何组织或者个人需要获取他人个人信息的，应当依法取得并确保信息安全，不得非法收集、使用、加工、传输他人个人信息，不得非法买卖、提供或者公开他人个人信息。

法宝联想

法律法规 规范性文件（22）白皮书（1）法律动态（16）

司法案例 司法案例（86806）案例报道（13）仲裁案例（3）

法学期刊 法学期刊（695）

法宝律师 律所实务（9）

专题参考 专题参考（113）

第一百一十二条 自然人因婚姻家庭关系等产生的人身权利受法律保护。

法宝联想

法律法规 司法解释（1）规范性文件（3）法律动态（1）

司法案例 司法案例（28245）仲裁案例（3）

法学期刊 法学期刊（108）

法宝律师 律所实务（3）

专题参考 专题参考（28）

第一百一十三条 民事主体的财产权利受法律平等保护。

法宝联想

法律法规 规范性文件（4）

司法案例 司法案例（34387）案例报道（3）仲裁案例（3）

法学期刊 法学期刊（106）

专题参考 专题参考（20）

第一百一十四条 民事主体依法享有物权。

物权是权利人依法对特定的物享有直接支配和排他的权利，包括所有权、用益物权和担保物权。

法宝联想

法律法规 规范性文件（2）

司法案例 司法案例（11360）仲裁案例（2）

法学期刊　法学期刊（129）

法宝律师　律所实务（4）

专题参考　专题参考（14）

第一百一十五条

物包括不动产和动产。法律规定权利作为物权客体的，依照其规定。

法宝联想

法律法规　规范性文件（5）

司法案例　司法案例（3514）仲裁案例（2）

法学期刊　法学期刊（102）

法宝律师　律所实务（6）

专题参考　专题参考（19）

第一百一十六条　物权的种类和内容，由法律规定。

法宝联想

司法案例　司法案例（1402）

法学期刊　法学期刊（116）

法宝律师　律所实务（3）

专题参考　专题参考（11）

第一百一十七条

为了公共利益的需要，依照法律规定的权限和程序征收、征用不动产或者动产的，应当给予公平、合理的补偿。

法宝联想

法律法规　司法解释（2）地方性法规（5）地方政府规章（1）规范性文件（8）法律动态（10）法律文书（1）

司法案例　司法案例（156217）案例报道（1）仲裁案例（2）

法学期刊　法学期刊（235）

法宝律师　律所实务（4）

专题参考　专题参考（75）

第一百一十八条　民事主体依法享有债权。

债权是因合同、侵权行为、无因管理、不当得利以及法律的其他规定，权利人请求特定义务人为或者不为一定行为的权利。

法宝联想

法律法规　司法解释（1）规范性文件（5）法规解读（1）中外条约（1）法律动态（1）

司法案例　司法案例（312701）案例报道（2）

法学期刊　法学期刊（255）

法宝律师　律所实务（10）

专题参考　专题参考（42）

第一百一十九条　依法成立的合同，对当事人具有法律约束力。

法宝联想

法律法规　司法解释（1）规范性文件（10）法规解读（3）法律动态（13）

司法案例　司法案例（775044）案例报道（16）仲裁案例（4）

法学期刊　法学期刊（251）

法宝律师　律所实务（18）

专题参考　专题参考（85）

第一百二十条

民事权益受到侵害的，被侵权人有权请求侵权人承担侵权责任。

法宝联想

法律法规　司法解释（1）规范性文件（7）法规解读（2）法律动态（12）

司法案例　司法案例（80689）案例报道（10）

法学期刊　法学期刊（357）

法宝律师　律所实务（19）

专题参考　专题参考（102）

第一百二十一条

没有法定的或者约定的义务，为避免他人利益受损失而进行管理的人，有权请求受益人偿还由此支出的必要费用。

法宝联想

法律法规　司法解释（2）规范性文件（7）法律动态（10）

司法案例　司法案例（17644）案例报道（2）

法学期刊　法学期刊（252）

法宝律师　律所实务（3）

专题参考　专题参考（62）

第一百二十二条

因他人没有法律根据，取得不当利益，受损失的人有权请求其返还不当利益。

法宝联想

法律法规　司法解释（1）部门规章（1）规范性文件（6）法律动态（2）

司法案例　司法案例（122829）案例报道（5）仲裁案例（1）

法学期刊　法学期刊（250）

法宝律师　律所实务（10）

专题参考　专题参考（56）

第一百二十三条　民事主体依法享有知识产权。

知识产权是权利人依法就下列客体享有的专有的权利：

（一）作品；

（二）发明、实用新型、外观设计；

（三）商标；

（四）地理标志；

（五）商业秘密；

（六）集成电路布图设计；

（七）植物新品种；

（八）法律规定的其他客体。

法宝联想

法律法规　司法解释（2）规范性文件（25）法规解读（1）法律动态（7）

司法案例　司法案例（17126）案例报道（4）

法学期刊　法学期刊（431）

法宝律师　律所实务（17）

专题参考　专题参考（79）

第一百二十四条　自然人依法享有继承权。

自然人合法的私有财产，可以依法继承。

法宝联想

法律法规　规范性文件（5）法律动态（3）

司法案例　司法案例（6580）案例报道（2）

法学期刊　法学期刊（179）

法宝律师　律所实务（4）

专题参考　专题参考（70）

第一百二十五条　民事主体依法享有股权和其他投资性权利。

法宝联想

法律法规　规范性文件（3）

司法案例　司法案例（5382）案例报道（3）仲裁案例（1）

法学期刊　法学期刊（68）

法宝律师　律所实务（9）

专题参考　专题参考（29）

第一百二十六条　民事主体享有法律规定的其他民事权利和利益。

法宝联想

法律法规　司法解释（1）地方政府规章（2）规范性文件（7）

司法案例　司法案例（6814）案例报道（4）

法学期刊　法学期刊（209）

法宝律师　律所实务（5）

专题参考　专题参考（63）

第一百二十七条

法律对数据、网络虚拟财产的保护有规定的，依照其规定。

法宝联想

法律法规　规范性文件（13）法律动态（11）

司法案例　司法案例（2768）案例报道（27）仲裁案例（3）

法学期刊　法学期刊（724）

法宝律师　律所实务（33）

专题参考　专题参考（129）

第一百二十八条

法律对未成年人、老年人、残疾人、妇女、消费者等的民事权利保护有特别规定的，依照其规定。

法宝联想

法律法规　规范性文件（2）法律动态（3）

司法案例　司法案例（2049）案例报道（3）

法学期刊　法学期刊（106）

专题参考　专题参考（25）

第一百二十九条

民事权利可以依据民事法律行为、事实行为、法律规定的事件或者法律规定的其他方式取得。

法宝联想

法律法规 规范性文件（1）法律动态（1）

司法案例 司法案例（3689）案例报道（1）仲裁案例（1）

法学期刊 法学期刊（77）

法宝律师 律所实务（1）

专题参考 专题参考（31）

第一百三十条 民事主体按照自己的意愿依法行使民事权利，不受干涉。

法宝联想

法律法规 司法解释（4）规范性文件（8）法规解读（2）法律动态（8）法律文书（1）

司法案例 司法案例（51074）案例报道（3）仲裁案例（4）

法学期刊 法学期刊（284）

法宝律师 律所实务（6）

专题参考 专题参考（91）

第一百三十一条

民事主体行使权利时，应当履行法律规定的和当事人约定的义务。

法宝联想

法律法规 司法解释（1）规范性文件（11）法律动态（5）

司法案例 司法案例（130661）案例报道（8）

法学期刊 法学期刊（113）

法宝律师 律所实务（8）

专题参考 专题参考（55）

第一百三十二条

民事主体不得滥用民事权利损害国家利益、社会公共利益或者他人合法权益。

法宝联想

法律法规　司法解释（1）规范性文件（10）法律动态（7）

司法案例　司法案例（15154）案例报道（10）仲裁案例（2）

法学期刊　法学期刊（411）

法宝律师　律所实务（21）

专题参考　专题参考（89）

第六章　民事法律行为

第一节　一般规定

第一百三十三条

民事法律行为是民事主体通过意思表示设立、变更、终止民事法律关系的行为。

法宝联想

法律法规　规范性文件（9）法规解读（1）法律动态（2）

司法案例　司法案例（26087）案例报道（7）仲裁案例（2）

法学期刊　法学期刊（222）

法宝律师　律所实务（5）

专题参考　专题参考（60）

第一百三十四条

民事法律行为可以基于双方或者多方的意思表示一致成立，也可以基于单方的意思表示成立。

法人、非法人组织依照法律或者章程规定的议事方式和表决程序作出决议的，该决议行为成立。

法宝联想

法律法规　司法解释（13）部门规章（1）地方性法规（1）规范性文件（22）法规解读（3）法律动态（15）

司法案例　司法案例（230549）案例报道（7）仲裁案例（2）

法学期刊　法学期刊（543）

法宝律师　律所实务（25）

专题参考　专题参考（158）

第一百三十五条

民事法律行为可以采用书面形式、口头形式或者其他形式；法律、行政法规规定或者当事人约定采用特定形式的，应当采用特定形式。

法宝联想

法律法规　司法解释（6）规范性文件（21）法律动态（6）

司法案例　司法案例（210116）案例报道（6）仲裁案例（18）

法学期刊　法学期刊（247）

法宝律师　律所实务（13）

专题参考　专题参考（71）

第一百三十六条

民事法律行为自成立时生效，但是法律另有规定或者当事人另有约定的除外。

行为人非依法律规定或者未经对方同意，不得擅自变更或者解除民事法律行为。

法宝联想

法律法规　司法解释（4）规范性文件（13）

司法案例　司法案例（44248）案例报道（3）仲裁案例（6）

法学期刊　法学期刊（147）

法宝律师　律所实务（2）

专题参考　专题参考（36）

第二节　意思表示

第一百三十七条　以对话方式作出的意思表示，相对人知道其内容时生效。

以非对话方式作出的意思表示，到达相对人时生效。以非对话方式作出的采用数据电文形式的意思表示，相对人指定特定系统接收数据电文的，该数据电文进入该特定系统时生效；未指定特定系统的，相对人知道或者应当知道该数据电文进入其系统时生效。当事人对采用数据电文形式的意思表示的生效时间另有约定的，按照其约定。

法宝联想

法律法规　司法解释（2）地方政府规章（4）规范性文件（18）法规解读（1）法律动态（10）

司法案例　司法案例（97195）案例报道（3）仲裁案例（5）

法学期刊　法学期刊（237）

法宝律师　律所实务（13）

专题参考　专题参考（89）

第一百三十八条

无相对人的意思表示，表示完成时生效。法律另有规定的，依照其规定。

法宝联想

法律法规　法律动态（3）

司法案例　司法案例（3818）

法学期刊　法学期刊（48）

法宝律师　律所实务（3）

专题参考　专题参考（18）

第一百三十九条　以公告方式作出的意思表示，公告发布时生效。

法宝联想

法律法规　司法解释（2）规范性文件（8）法律动态（4）

司法案例　司法案例（5797）案例报道（1）

法学期刊　法学期刊（57）

法宝律师　律所实务（6）

专题参考　专题参考（19）

第一百四十条　行为人可以明示或者默示作出意思表示。

沉默只有在有法律规定、当事人约定或者符合当事人之间的交易习惯时，才可以视为意思表示。

法宝联想

法律法规　司法解释（3）规范性文件（14）法规解读（1）法律动态（4）

司法案例　司法案例（79635）案例报道（7）仲裁案例（14）

法学期刊　法学期刊（241）

法宝律师　律所实务（12）

专题参考　专题参考（86）

第一百四十一条

行为人可以撤回意思表示。撤回意思表示的通知应当在意思表示到达相对人前或者与意思表示同时到达相对人。

法宝联想

法律法规　规范性文件（1）法律动态（1）

司法案例　司法案例（1709）

法学期刊　法学期刊（42）

法宝律师　律所实务（2）

专题参考　专题参考（8）

第一百四十二条

有相对人的意思表示的解释，应当按照所使用的词句，结合相关条款、行为的性质和目的、习惯以及诚信原则，确定意思表示的含义。

无相对人的意思表示的解释，不能完全拘泥于所使用的词句，而应当结合相关条款、行为的性质和目的、习惯以及诚信原则，确定行为人的真实意思。

法宝联想

法律法规　司法解释（2）规范性文件（11）法规解读（1）中外条约（1）法律动态（5）

司法案例　司法案例（11107）案例报道（4）仲裁案例（17）

法学期刊　法学期刊（576）

法宝律师　律所实务（27）

专题参考　专题参考（89）

第三节　民事法律行为的效力

第一百四十三条　具备下列条件的民事法律行为有效：

（一）行为人具有相应的民事行为能力；

（二）意思表示真实；

（三）不违反法律、行政法规的强制性规定，不违背公序良俗。

法宝联想

法律法规　规范性文件（11）法律动态（2）

司法案例　司法案例（100521）案例报道（8）仲裁案例（35）

法学期刊　法学期刊（332）

法宝律师　律所实务（31）

专题参考　专题参考（58）

第一百四十四条　无民事行为能力人实施的民事法律行为无效。

法宝联想

法律法规　司法解释（2）规范性文件（1）

司法案例　司法案例（4428）案例报道（2）

法学期刊　法学期刊（155）

法宝律师　律所实务（5）

专题参考　专题参考（22）

第一百四十五条

限制民事行为能力人实施的纯获利益的民事法律行为或者与其年龄、智力、精神健康状况相适应的民事法律行为有效；实施的其他民事法律行为经法定代理人同意或者追认后有效。

相对人可以催告法定代理人自收到通知之日起三十日内予以追认。法定代理人未作表示的，视为拒绝追认。民事法律行为被追认前，善意相对人有撤销的权利。撤销应当以通知的方式作出。

法宝联想

法律法规　司法解释（1）规范性文件（14）法规解读（1）法律动态（5）

司法案例　司法案例（5039）案例报道（6）仲裁案例（8）

法学期刊　法学期刊（217）

法宝律师　律所实务（4）

专题参考　专题参考（33）

第一百四十六条

行为人与相对人以虚假的意思表示实施的民事法律行为无效。

以虚假的意思表示隐藏的民事法律行为的效力，依照有关法律规定处理。

法宝联想

法律法规　法律（1）司法解释（3）规范性文件（19）法律动态（5）

司法案例　司法案例（33515）案例报道（15）仲裁案例（20）

法学期刊　法学期刊（429）

法宝律师　律所实务（67）

专题参考　专题参考（94）

第一百四十七条

基于重大误解实施的民事法律行为，行为人有权请求人民法院或者仲裁机构予以撤销。

法宝联想

法律法规　法律（1）司法解释（1）规范性文件（2）

司法案例　司法案例（11348）案例报道（2）仲裁案例（5）

法学期刊　法学期刊（249）

法宝律师　律所实务（8）

专题参考　专题参考（34）

第一百四十八条

一方以欺诈手段，使对方在违背真实意思的情况下实施的民事法律行为，受欺诈方有权请求人民法院或者仲裁机构予以撤销。

法宝联想

法律法规　司法解释（3）规范性文件（5）法规解读（1）法律动态（3）

司法案例　司法案例（18922）案例报道（14）仲裁案例（3）

法学期刊　法学期刊（243）

法宝律师　律所实务（20）

专题参考　专题参考（60）

第一百四十九条

第三人实施欺诈行为，使一方在违背真实意思的情况下实施的民事法律行为，对方知道或者应当知道该欺诈行为的，受欺诈方有权请求人民法院或者仲裁机构予以撤销。

法宝联想

法律法规　司法解释（1）规范性文件（4）

司法案例　司法案例（3397）案例报道（2）

法学期刊　法学期刊（126）

法宝律师　律所实务（10）

专题参考　专题参考（35）

第一百五十条

一方或者第三人以胁迫手段，使对方在违背真实意思的情况下实施的民事法律行为，受胁迫方有权请求人民法院或者仲裁机构予以撤销。

法宝联想

法律法规　司法解释（1）规范性文件（2）法规解读（1）

司法案例　司法案例（8740）案例报道（1）仲裁案例（2）

法学期刊　法学期刊（164）

法宝律师　律所实务（6）

专题参考　专题参考（34）

第一百五十一条

一方利用对方处于危困状态、缺乏判断能力等情形，致使民事法律行为成立时显失公平的，受损害方有权请求人民法院或者仲裁机构予以撤销。

法宝联想

法律法规 司法解释（1）规范性文件（2）

司法案例 司法案例（13082）案例报道（7）仲裁案例（7）

法学期刊 法学期刊（180）

法宝律师 律所实务（10）

专题参考 专题参考（36）

第一百五十二条　有下列情形之一的，撤销权消灭：

（一）当事人自知道或者应当知道撤销事由之日起一年内、重大误解的当事人自知道或者应当知道撤销事由之日起九十日内没有行使撤销权；

（二）当事人受胁迫，自胁迫行为终止之日起一年内没有行使撤销权；

（三）当事人知道撤销事由后明确表示或者以自己的行为表明放弃撤销权。

当事人自民事法律行为发生之日起五年内没有行使撤销权的，撤销权消灭。

法宝联想

法律法规 司法解释（1）规范性文件（1）

司法案例 司法案例（13376）案例报道（1）仲裁案例（4）

法学期刊 法学期刊（99）

法宝律师 律所实务（6）

专题参考 专题参考（13）

第一百五十三条

违反法律、行政法规的强制性规定的民事法律行为无效。但是，该强制性规定不导致该民事法律行为无效的除外。

违背公序良俗的民事法律行为无效。

法宝联想

法律法规　司法解释（7）规范性文件（32）法规解读（2）白皮书（2）法律动态（16）合同范本（1
）

司法案例　司法案例（98415）案例报道（51）仲裁案例（56）

法学期刊　法学期刊（776）

法宝律师　律所实务（141）

专题参考　专题参考（127）

第一百五十四条

行为人与相对人恶意串通，损害他人合法权益的民事法律行为无效。

法宝联想

法律法规　司法解释（2）地方政府规章（1）规范性文件（9）法律动态（1）

司法案例　司法案例（17664）案例报道（9）仲裁案例（7）

法学期刊　法学期刊（253）

法宝律师　律所实务（32）

专题参考　专题参考（45）

第一百五十五条　无效的或者被撤销的民事法律行为自始没有法律约束力。

法宝联想

法律法规　规范性文件（3）法律动态（1）

司法案例　司法案例（24477）案例报道（2）仲裁案例（6）

法学期刊　法学期刊（74）

法宝律师　律所实务（7）

专题参考　专题参考（12）

第一百五十六条

民事法律行为部分无效，不影响其他部分效力的，其他部分仍然有效。

法宝联想

法律法规　规范性文件（1）法律动态（1）

司法案例　司法案例（6605）案例报道（1）

法学期刊　法学期刊（60）

法宝律师　律所实务（7）

专题参考　专题参考（7）

第一百五十七条

民事法律行为无效、被撤销或者确定不发生效力后，行为人因该行为取得的财产，应当予以返还；不能返还或者没有必要返还的，应当折价补偿。有过错的一方应当赔偿对方由此所受到的损失；各方都有过错的，应当各自承担相应的责任。法律另有规定的，依照其规定。

法宝联想

法律法规　司法解释（6）规范性文件（15）法律动态（9）

司法案例　司法案例（101348）案例报道（32）仲裁案例（20）

法学期刊　法学期刊（374）

法宝律师　律所实务（55）

专题参考　专题参考（52）

第四节　民事法律行为的附条件和附期限

第一百五十八条

民事法律行为可以附条件，但是根据其性质不得附条件的除外。附生效条件的民事法律行为，自条件成就时生效。附解除条件的民事法律行为，自条件成就时失效。

法宝联想

法律法规　规范性文件（2）

司法案例　司法案例（13122）案例报道（2）仲裁案例（4）

法学期刊　法学期刊（65）

法宝律师　律所实务（15）

专题参考　专题参考（13）

第一百五十九条

附条件的民事法律行为，当事人为自己的利益不正当地阻止条件成就的，视为条件已经成就；不正当地促成条件成就的，视为条件不成就。

法宝联想
法律法规　规范性文件（3）
司法案例　司法案例（7295）案例报道（4）仲裁案例（4）
法学期刊　法学期刊（55）
法宝律师　律所实务（13）
专题参考　专题参考（6）

第一百六十条

民事法律行为可以附期限，但是根据其性质不得附期限的除外。附生效期限的民事法律行为，自期限届至时生效。附终止期限的民事法律行为，自期限届满时失效。

法宝联想
法律法规　规范性文件（1）
司法案例　司法案例（2302）仲裁案例（5）
法学期刊　法学期刊（27）
法宝律师　律所实务（2）
专题参考　专题参考（4）

第七章　代理

第一节　一般规定

第一百六十一条　民事主体可以通过代理人实施民事法律行为。

依照法律规定、当事人约定或者民事法律行为的性质，应当由本人亲自实施的民事法律行为，不得代理。

法宝联想

法律法规　规范性文件（2）

司法案例　司法案例（13348）仲裁案例（2）

法学期刊　法学期刊（34）

专题参考　专题参考（3）

第一百六十二条

代理人在代理权限内，以被代理人名义实施的民事法律行为，对被代理人发生效力。

法宝联想

法律法规　规范性文件（2）

司法案例　司法案例（34627）仲裁案例（3）

法学期刊　法学期刊（80）

法宝律师　律所实务（6）

专题参考　专题参考（10）

第一百六十三条　代理包括委托代理和法定代理。

委托代理人按照被代理人的委托行使代理权。法定代理人依照法律的规定行使代理权。

法宝联想

司法案例　司法案例（1878）仲裁案例（2）

法学期刊　法学期刊（28）

法宝律师　律所实务（1）

专题参考 专题参考（3）

第一百六十四条

代理人不履行或者不完全履行职责，造成被代理人损害的，应当承担民事责任。

代理人和相对人恶意串通，损害被代理人合法权益的，代理人和相对人应当承担连带责任。

法宝联想

司法案例 司法案例（2368）

法学期刊 法学期刊（68）

法宝律师 律所实务（2）

专题参考 专题参考（5）

第二节 委托代理

第一百六十五条

委托代理授权采用书面形式的，授权委托书应当载明代理人的姓名或者名称、代理事项、权限和期限，并由被代理人签名或者盖章。

法宝联想

法律法规 规范性文件（1）

司法案例 司法案例（3141）仲裁案例（1）

法学期刊 法学期刊（21）

法宝律师 律所实务（1）

专题参考 专题参考（3）

第一百六十六条

数人为同一代理事项的代理人的，应当共同行使代理权，但是当事人另有约定的除外。

法宝联想

司法案例　司法案例（257）

法学期刊　法学期刊（18）

专题参考　专题参考（5）

第一百六十七条

代理人知道或者应当知道代理事项违法仍然实施代理行为，或者被代理人知道或者应当知道代理人的代理行为违法未作反对表示的，被代理人和代理人应当承担连带责任。

法宝联想

法律法规　规范性文件（5）法律动态（2）

司法案例　司法案例（4949）案例报道（4）仲裁案例（1）

法学期刊　法学期刊（40）

法宝律师　律所实务（11）

专题参考　专题参考（6）

第一百六十八条

代理人不得以被代理人的名义与自己实施民事法律行为，但是被代理人同意或者追认的除外。

代理人不得以被代理人的名义与自己同时代理的其他人实施民事法律行为，但是被代理的双方同意或者追认的除外。

法宝联想

法律法规　规范性文件（2）

司法案例　司法案例（1441）案例报道（1）

法学期刊　法学期刊（64）

法宝律师　律所实务（5）

专题参考　专题参考（2）

第一百六十九条

代理人需要转委托第三人代理的，应当取得被代理人的同意或者追认。

转委托代理经被代理人同意或者追认的，被代理人可以就代理事务直接指示转委托的第三人，代理人仅就第三人的选任以及对第三人的指示承担责任。

转委托代理未经被代理人同意或者追认的，代理人应当对转委托的第三人的行为承担责任；但是，在紧急情况下代理人为了维护被代理人的利益需要转委托第三人代理的除外。

法宝联想

法律法规　司法解释（1）规范性文件（1）法律动态（1）

司法案例　司法案例（1466）案例报道（1）

法学期刊　法学期刊（13）

专题参考　专题参考（2）

第一百七十条

执行法人或者非法人组织工作任务的人员，就其职权范围内的事项，以法人或者非法人组织的名义实施的民事法律行为，对法人或者非法人组织发生效力。

法人或者非法人组织对执行其工作任务的人员职权范围的限制，不得对抗善意相对人。

法宝联想

法律法规 规范性文件（1）法律动态（1）

司法案例 司法案例（42118）案例报道（7）仲裁案例（5）

法学期刊 法学期刊（115）

法宝律师 律所实务（15）

专题参考 专题参考（23）

第一百七十一条

行为人没有代理权、超越代理权或者代理权终止后，仍然实施代理行为，未经被代理人追认的，对被代理人不发生效力。

相对人可以催告被代理人自收到通知之日起三十日内予以追认。被代理人未作表示的，视为拒绝追认。行为人实施的行为被追认前，善意相对人有撤销的权利。撤销应当以通知的方式作出。

行为人实施的行为未被追认的，善意相对人有权请求行为人履行债务或者就其受到的损害请求行为人赔偿。但是，赔偿的范围不得超过被代理人追认时相对人所能获得的利益。

相对人知道或者应当知道行为人无权代理的，相对人和行为人按照各自的过错承担责任。

法宝联想

法律法规　司法解释（1）规范性文件（3）

司法案例　司法案例（18331）案例报道（4）仲裁案例（2）

法学期刊　法学期刊（236）

法宝律师　律所实务（8）

专题参考　专题参考（30）

第一百七十二条

行为人没有代理权、超越代理权或者代理权终止后，仍然实施代理行为，相对人有理由相信行为人有代理权的，代理行为有效。

法宝联想

法律法规　司法解释（5）规范性文件（5）法律动态（1）

司法案例　司法案例（32150）案例报道（7）仲裁案例（14）

法学期刊　法学期刊（174）

法宝律师　律所实务（17）

专题参考　专题参考（20）

第三节　代理终止

第一百七十三条　有下列情形之一的，委托代理终止：

（一）代理期限届满或者代理事务完成；

（二）被代理人取消委托或者代理人辞去委托；

（三）代理人丧失民事行为能力；

（四）代理人或者被代理人死亡；

（五）作为代理人或者被代理人的法人、非法人组织终止。

法宝联想

司法案例　司法案例（1407）仲裁案例（1）

法学期刊　法学期刊（18）

专题参考　专题参考（1）

第一百七十四条

被代理人死亡后，有下列情形之一的，委托代理人实施的代理行为有效：

（一）代理人不知道且不应当知道被代理人死亡；

（二）被代理人的继承人予以承认；

（三）授权中明确代理权在代理事务完成时终止；

（四）被代理人死亡前已经实施，为了被代理人的继承人的利益继续代理。

作为被代理人的法人、非法人组织终止的，参照适用前款规定。

法宝联想

司法案例　司法案例（318）

法学期刊　法学期刊（18）

专题参考　专题参考（3）

第一百七十五条　有下列情形之一的，法定代理终止：

（一）被代理人取得或者恢复完全民事行为能力；

（二）代理人丧失民事行为能力；

（三）代理人或者被代理人死亡；

（四）法律规定的其他情形。

法宝联想

司法案例　司法案例（399）

法学期刊　法学期刊（9）

第八章　民事责任

第一百七十六条

民事主体依照法律规定或者按照当事人约定，履行民事义务，承担民事责任。

法宝联想

法律法规 规范性文件（2）法律动态（1）

司法案例 司法案例（394071）案例报道（1）仲裁案例（6）

法学期刊 法学期刊（47）

法宝律师 律所实务（2）

专题参考 专题参考（5）

第一百七十七条

二人以上依法承担按份责任，能够确定责任大小的，各自承担相应的责任；难以确定责任大小的，平均承担责任。

法宝联想

司法案例 司法案例（5482）

法学期刊 法学期刊（44）

法宝律师 律所实务（1）

专题参考 专题参考（6）

第一百七十八条

二人以上依法承担连带责任的，权利人有权请求部分或者全部连带责任人承担责任。

连带责任人的责任份额根据各自责任大小确定；难以确定责任大小的，平均承担责任。实际承担责任超过自己责任份额的连带责任人，有权向其他连带责任人追偿。

连带责任，由法律规定或者当事人约定。

法宝联想

法律法规 司法解释（2）规范性文件（4）法律动态（3）

司法案例 司法案例（46477）案例报道（6）仲裁案例（4）

法学期刊　法学期刊（208）

法宝律师　律所实务（27）

专题参考　专题参考（27）

第一百七十九条　承担民事责任的方式主要有：

（一）停止侵害；

（二）排除妨碍；

（三）消除危险；

（四）返还财产；

（五）恢复原状；

（六）修理、重作、更换；

（七）继续履行；

（八）赔偿损失；

（九）支付违约金；

（十）消除影响、恢复名誉；

（十一）赔礼道歉。

法律规定惩罚性赔偿的，依照其规定。

本条规定的承担民事责任的方式，可以单独适用，也可以合并适用。

法宝联想

法律法规　司法解释（3）规范性文件（15）法律动态（9）

司法案例　司法案例（170079）案例报道（17）仲裁案例（3）

法学期刊　法学期刊（399）

法宝律师　律所实务（19）

专题参考　专题参考（23）

第一百八十条

因不可抗力不能履行民事义务的，不承担民事责任。法律另有规定的，依照其规定。

不可抗力是不能预见、不能避免且不能克服的客观情况。

法宝联想

法律法规　规范性文件（34）法律动态（5）

司法案例　司法案例（34283）案例报道（5）仲裁案例（21）

法学期刊　法学期刊（91）

法宝律师　律所实务（46）

专题参考　专题参考（39）

第一百八十一条　因正当防卫造成损害的，不承担民事责任。

正当防卫超过必要的限度，造成不应有的损害的，正当防卫人应当承担适当的民事责任。

法宝联想

法律法规　司法解释（1）规范性文件（2）法律动态（2）

司法案例　司法案例（1052）案例报道（3）

法学期刊　法学期刊（63）

法宝律师　律所实务（1）

专题参考　专题参考（9）

第一百八十二条

因紧急避险造成损害的，由引起险情发生的人承担民事责任。

危险由自然原因引起的，紧急避险人不承担民事责任，可以给予适当补偿。

紧急避险采取措施不当或者超过必要的限度，造成不应有的损害的，紧急避险人应当承担适当的民事责任。

法宝联想

法律法规 司法解释（1）法律动态（1）

司法案例 司法案例（781）案例报道（3）

法学期刊 法学期刊（98）

专题参考 专题参考（7）

第一百八十三条

因保护他人民事权益使自己受到损害的，由侵权人承担民事责任，受益人可以给予适当补偿。没有侵权人、侵权人逃逸或者无力承担民事责任，受害人请求补偿的，受益人应当给予适当补偿。

法宝联想

法律法规 司法解释（1）规范性文件（4）法规解读（1）法律动态（3）

司法案例 司法案例（1177）案例报道（16）

法学期刊 法学期刊（103）

法宝律师 律所实务（1）

专题参考 专题参考（10）

第一百八十四条

因自愿实施紧急救助行为造成受助人损害的，救助人不承担民事责任。

法宝联想

法律法规 规范性文件（57）法规解读（1）法律动态（7）

司法案例 司法案例（561）案例报道（7）

法学期刊 法学期刊（119）

专题参考 专题参考（14）

第一百八十五条

侵害英雄烈士等的姓名、肖像、名誉、荣誉，损害社会公共利益的，应当承担民事责任。

法宝联想

法律法规 司法解释（1）规范性文件（8）法规解读（1）法律动态（9）

司法案例 司法案例（482）案例报道（10）

法学期刊 法学期刊（151）

法宝律师 律所实务（1）

专题参考 专题参考（26）

第一百八十六条

因当事人一方的违约行为，损害对方人身权益、财产权益的，受损害方有权选择请求其承担违约责任或者侵权责任。

法宝联想

法律法规 司法解释（2）规范性文件（1）

司法案例 司法案例（7613）案例报道（2）仲裁案例（2）

法学期刊 法学期刊（142）

法宝律师 律所实务（12）

专题参考 专题参考（9）

第一百八十七条

民事主体因同一行为应当承担民事责任、行政责任和刑事责任的，承担行政责任或者刑事责任不影响承担民事责任；民事主体的财产不足以支付的，优先用于承担民事责任。

法宝联想

法律法规 规范性文件（6）法规解读（1）法律动态（5）

司法案例 司法案例（3461）案例报道（6）

法学期刊 法学期刊（128）

法宝律师 律所实务（7）

专题参考 专题参考（8）

第九章 诉讼时效

第一百八十八条

向人民法院请求保护民事权利的诉讼时效期间为三年。法律另有规定的，依照其规定。

诉讼时效期间自权利人知道或者应当知道权利受到损害以及义务人之日起计算。法律另有规定的，依照其规定。但是，自权利受到损害之日起超过二十年的，人民法院不予保护，有特殊情况的，人民法院可以根据权利人的申请决定延长。

法宝联想

法律法规 司法解释（4）规范性文件（11）法规解读（1）白皮书（1）法律动态（3）

司法案例 司法案例（274689）案例报道（13）仲裁案例（33）

法学期刊 法学期刊（171）

法宝律师 律所实务（34）

专题参考 专题参考（52）

第一百八十九条

当事人约定同一债务分期履行的，诉讼时效期间自最后一期履行期限届满之日起计算。

法宝联想

法律法规 规范性文件（1）

司法案例 司法案例（8974）仲裁案例（6）

法学期刊 法学期刊（14）

法宝律师　律所实务（1）

专题参考　专题参考（3）

第一百九十条

无民事行为能力人或者限制民事行为能力人对其法定代理人的请求权的诉讼时效期间，自该法定代理终止之日起计算。

法宝联想

司法案例　司法案例（259）

法学期刊　法学期刊（17）

专题参考　专题参考（2）

第一百九十一条

未成年人遭受性侵害的损害赔偿请求权的诉讼时效期间，自受害人年满十八周岁之日起计算。

法宝联想

法律法规　规范性文件（2）法律动态（1）

司法案例　司法案例（450）

法学期刊　法学期刊（39）

专题参考　专题参考（4）

第一百九十二条

诉讼时效期间届满的，义务人可以提出不履行义务的抗辩。

诉讼时效期间届满后，义务人同意履行的，不得以诉讼时效期间届满为由抗辩；义务人已经自愿履行的，不得请求返还。

法宝联想

法律法规　规范性文件（3）法律动态（1）

司法案例　司法案例（28026）案例报道（3）仲裁案例（4）

法学期刊　法学期刊（70）

法宝律师　律所实务（4）

专题参考　专题参考（10）

第一百九十三条　人民法院不得主动适用诉讼时效的规定。

法宝联想

法律法规　规范性文件（2）法律动态（1）

司法案例　司法案例（3202）案例报道（1）

法学期刊　法学期刊（39）

法宝律师　律所实务（3）

专题参考　专题参考（5）

第一百九十四条

在诉讼时效期间的最后六个月内，因下列障碍，不能行使请求权的，诉讼时效中止：

（一）不可抗力；

（二）无民事行为能力人或者限制民事行为能力人没有法定代理人，或者法定代理人死亡、丧失民事行为能力、丧失代理权；

（三）继承开始后未确定继承人或者遗产管理人；

（四）权利人被义务人或者其他人控制；

（五）其他导致权利人不能行使请求权的障碍。

自中止时效的原因消除之日起满六个月，诉讼时效期间届满。

法宝联想

法律法规　规范性文件（33）法律动态（3）

司法案例　司法案例（3976）

法学期刊　法学期刊（50）

法宝律师　律所实务（6）

专题参考　专题参考（13）

第一百九十五条

有下列情形之一的，诉讼时效中断，从中断、有关程序终结时起，诉讼时效期间重新计算：

（一）权利人向义务人提出履行请求；

（二）义务人同意履行义务；

（三）权利人提起诉讼或者申请仲裁；

（四）与提起诉讼或者申请仲裁具有同等效力的其他情形。

法宝联想

法律法规　司法解释（3）规范性文件（2）法规解读（1）法律动态（1）

司法案例　司法案例（69409）仲裁案例（5）

法学期刊　法学期刊（75）

法宝律师　律所实务（8）

专题参考　专题参考（17）

第一百九十六条　下列请求权不适用诉讼时效的规定：

（一）请求停止侵害、排除妨碍、消除危险；

（二）不动产物权和登记的动产物权的权利人请求返还财产；

（三）请求支付抚养费、赡养费或者扶养费；

（四）依法不适用诉讼时效的其他请求权。

法宝联想

法律法规　法律（1）规范性文件（2）

司法案例　司法案例（9803）案例报道（3）

法学期刊　法学期刊（110）

法宝律师　律所实务（5）

专题参考　专题参考（11）

第一百九十七条

诉讼时效的期间、计算方法以及中止、中断的事由由法律规定，当事人约定无效。

当事人对诉讼时效利益的预先放弃无效。

法宝联想

法律法规　规范性文件（1）

司法案例　司法案例（727）

法学期刊　法学期刊（42）

专题参考　专题参考（6）

第一百九十八条

法律对仲裁时效有规定的，依照其规定；没有规定的，适用诉讼时效的规定。

法宝联想

司法案例　司法案例（3175）仲裁案例（2）

法学期刊　法学期刊（12）

法宝律师　律所实务（2）

专题参考　专题参考（1）

第一百九十九条

法律规定或者当事人约定的撤销权、解除权等权利的存续期间，除法律另有规定

外，自权利人知道或者应当知道权利产生之日起计算，不适用有关诉讼时效中止、中断和延长的规定。存续期间届满，撤销权、解除权等权利消灭。

法宝联想

法律法规　规范性文件（3）

司法案例　司法案例（2143）仲裁案例（1）

法学期刊　法学期刊（29）

法宝律师　律所实务（10）

专题参考　专题参考（8）

第十章　期间计算

第二百条　民法所称的期间按照公历年、月、日、小时计算。

法宝联想

法律法规　规范性文件（1）

司法案例　司法案例（223）

法学期刊　法学期刊（6）

专题参考　专题参考（2）

第二百零一条

按照年、月、日计算期间的，开始的当日不计入，自下一日开始计算。

按照小时计算期间的，自法律规定或者当事人约定的时间开始计算。

法宝联想

法律法规　规范性文件（1）

司法案例　司法案例（1245）

法学期刊　法学期刊（7）

法宝律师　律所实务（3）

专题参考　专题参考（6）

第二百零二条

按照年、月计算期间的，到期月的对应日为期间的最后一日；没有对应日的，月末日为期间的最后一日。

法宝联想

司法案例　司法案例（514）

专题参考　专题参考（1）

第二百零三条

期间的最后一日是法定休假日的，以法定休假日结束的次日为期间的最后一日。

期间的最后一日的截止时间为二十四时；有业务时间的，停止业务活动的时间为截止时间。

法宝联想

法律法规　规范性文件（1）合同范本（5）

司法案例　司法案例（416）

法学期刊　法学期刊（1）

法宝律师　律所实务（2）

专题参考　专题参考（2）

第二百零四条

期间的计算方法依照本法的规定，但是法律另有规定或者当事人另有约定的除外。

法宝联想

法律法规　规范性文件（1）

司法案例　司法案例（97）

法学期刊　法学期刊（3）

专题参考　专题参考（2）

第二编　物权

第一分编　通则

第一章　一般规定

第二百零五条　本编调整因物的归属和利用产生的民事关系。

法宝联想

司法案例　司法案例（632）

法学期刊　法学期刊（19）

法宝律师　律所实务（2）

专题参考　专题参考（4）

第二百零六条

国家坚持和完善公有制为主体、多种所有制经济共同发展，按劳分配为主体、多种分配方式并存，社会主义市场经济体制等社会主义基本经济制度。

国家巩固和发展公有制经济，鼓励、支持和引导非公有制经济的发展。

国家实行社会主义市场经济，保障一切市场主体的平等法律地位和发展权利。

法宝联想

司法案例　司法案例（4858）

法学期刊　法学期刊（48）

专题参考　专题参考（10）

第二百零七条

国家、集体、私人的物权和其他权利人的物权受法律平等保护，任何组织或者个人不得侵犯。

法宝联想

法律法规　规范性文件（2）

司法案例　司法案例（5569）案例报道（2）

法学期刊　法学期刊（58）

法宝律师　律所实务（2）

专题参考　专题参考（11）

第二百零八条

不动产物权的设立、变更、转让和消灭，应当依照法律规定登记。动产物权的设立和转让，应当依照法律规定交付。

法宝联想

司法案例　司法案例（4237）

法学期刊　法学期刊（37）

法宝律师　律所实务（3）

专题参考　专题参考（2）

第二章　物权的设立、变更、转让和消灭

第一节　不动产登记

第二百零九条

不动产物权的设立、变更、转让和消灭，经依法登记，发生效力；未经登记，不发生效力，但是法律另有规定的除外。

依法属于国家所有的自然资源，所有权可以不登记。

法宝联想

法律法规　规范性文件（3）

司法案例　司法案例（33534）案例报道（2）仲裁案例（1）

法学期刊　法学期刊（160）

法宝律师　律所实务（21）

专题参考　专题参考（12）

第二百一十条　不动产登记，由不动产所在地的登记机构办理。

国家对不动产实行统一登记制度。统一登记的范围、登记机构和登记办法，由法律、行政法规规定。

法宝联想

法律法规　规范性文件（3）

司法案例　司法案例（976）

法学期刊　法学期刊（18）

法宝律师　律所实务（2）

第二百一十一条

当事人申请登记，应当根据不同登记事项提供权属证明和不动产界址、面积等必要材料。

法宝联想

法律法规　规范性文件（1）

司法案例　司法案例（487）

法学期刊　法学期刊（3）

第二百一十二条　登记机构应当履行下列职责：

（一）查验申请人提供的权属证明和其他必要材料；

（二）就有关登记事项询问申请人；

（三）如实、及时登记有关事项；

（四）法律、行政法规规定的其他职责。

申请登记的不动产的有关情况需要进一步证明的，登记机构可以要求申请人补充材料，必要时可以实地查看。

法宝联想

法律法规　规范性文件（2）

司法案例　司法案例（149）

法学期刊　法学期刊（16）

第二百一十三条　登记机构不得有下列行为：

（一）要求对不动产进行评估；

（二）以年检等名义进行重复登记；

（三）超出登记职责范围的其他行为。

法宝联想

法律法规　规范性文件（1）

司法案例　司法案例（233）案例报道（1）

法学期刊　法学期刊（8）

第二百一十四条

不动产物权的设立、变更、转让和消灭，依照法律规定应当登记的，自记载于不动产登记簿时发生效力。

法宝联想

司法案例　司法案例（5159）仲裁案例（1）

法学期刊　法学期刊（43）

法宝律师　律所实务（3）

专题参考　专题参考（3）

第二百一十五条

当事人之间订立有关设立、变更、转让和消灭不动产物权的合同，除法律另有规定或者当事人另有约定外，自合同成立时生效；未办理物权登记的，不影响合同效力。

法宝联想

法律法规　规范性文件（1）法律动态（1）

司法案例　司法案例（7009）案例报道（1）仲裁案例（1）

法学期刊　法学期刊（84）

法宝律师　律所实务（7）

专题参考　专题参考（6）

第二百一十六条　不动产登记簿是物权归属和内容的根据。

不动产登记簿由登记机构管理。

法宝联想

法律法规　规范性文件（2）

司法案例　司法案例（2903）仲裁案例（1）

法学期刊　法学期刊（43）

法宝律师　律所实务（1）

专题参考　专题参考（3）

第二百一十七条

不动产权属证书是权利人享有该不动产物权的证明。不动产权属证书记载的事项，应当与不动产登记簿一致；记载不一致的，除有证据证明不动产登记簿确有错误外，以不动产登记簿为准。

法宝联想

司法案例　司法案例（4930）案例报道（2）仲裁案例（1）

法学期刊　法学期刊（16）

法宝律师　律所实务（1）

第二百一十八条

权利人、利害关系人可以申请查询、复制不动产登记资料，登记机构应当提供。

法宝联想

法律法规　规范性文件（1）

司法案例　司法案例（56）

法学期刊　法学期刊（5）

第二百一十九条

利害关系人不得公开、非法使用权利人的不动产登记资料。

法宝联想

司法案例　司法案例（30）

法学期刊　法学期刊（2）

第二百二十条

权利人、利害关系人认为不动产登记簿记载的事项错误的，可以申请更正登记。不动产登记簿记载的权利人书面同意更正或者有证据证明登记确有错误的，登记机构应当予以更正。

不动产登记簿记载的权利人不同意更正的，利害关系人可以申请异议登记。登记机构予以异议登记，申请人自异议登记之日起十五日内不提起诉讼的，异议登记失效。异议登记不当，造成权利人损害的，权利人可以向申请人请求损害赔偿。

法宝联想

法律法规 司法解释（1）规范性文件（3）

司法案例 司法案例（508）

法学期刊 法学期刊（24）

第二百二十一条

当事人签订买卖房屋的协议或者签订其他不动产物权的协议，为保障将来实现物权，按照约定可以向登记机构申请预告登记。预告登记后，未经预告登记的权利人同意，处分该不动产的，不发生物权效力。

预告登记后，债权消灭或者自能够进行不动产登记之日起九十日内未申请登记的，预告登记失效。

法宝联想

法律法规 司法解释（1）规范性文件（1）

司法案例 司法案例（8902）案例报道（2）仲裁案例（6）

法学期刊 法学期刊（75）

法宝律师 律所实务（7）

专题参考 专题参考（15）

第二百二十二条

当事人提供虚假材料申请登记，造成他人损害的，应当承担赔偿责任。

因登记错误，造成他人损害的，登记机构应当承担赔偿责任。登记机构赔偿后，可以向造成登记错误的人追偿。

法宝联想

法律法规 规范性文件（2）

司法案例　司法案例（202）

法学期刊　法学期刊（23）

专题参考　专题参考（2）

第二百二十三条

不动产登记费按件收取，不得按照不动产的面积、体积或者价款的比例收取。

法宝联想

司法案例　司法案例（58）

法学期刊　法学期刊（6）

法宝律师　律所实务（1）

第二节　动产交付

第二百二十四条

动产物权的设立和转让，自交付时发生效力，但是法律另有规定的除外。

法宝联想

法律法规　规范性文件（2）

司法案例　司法案例（6860）案例报道（4）仲裁案例（2）

法学期刊　法学期刊（95）

法宝律师　律所实务（7）

专题参考　专题参考（5）

第二百二十五条

船舶、航空器和机动车等的物权的设立、变更、转让和消灭，未经登记，不得对抗善意第三人。

法宝联想

法律法规　司法解释（1）规范性文件（3）

司法案例　司法案例（3451）案例报道（1）仲裁案例（2）

法学期刊　法学期刊（45）

法宝律师　律所实务（4）

专题参考　专题参考（1）

第二百二十六条

动产物权设立和转让前，权利人已经占有该动产的，物权自民事法律行为生效时发生效力。

法宝联想

法律法规　司法解释（1）

司法案例　司法案例（414）

法学期刊　法学期刊（19）

法宝律师　律所实务（1）

专题参考　专题参考（1）

第二百二十七条

动产物权设立和转让前，第三人占有该动产的，负有交付义务的人可以通过转让请求第三人返还原物的权利代替交付。

法宝联想

法律法规　司法解释（1）

司法案例　司法案例（287）

法学期刊　法学期刊（20）

法宝律师　律所实务（1）

专题参考　专题参考（1）

第二百二十八条

动产物权转让时，当事人又约定由出让人继续占有该动产的，物权自该约定生效时发生效力。

法宝联想

司法案例　司法案例（1218）案例报道（1）仲裁案例（1）

法学期刊　法学期刊（23）

法宝律师　律所实务（2）

专题参考　专题参考（1）

第三节　其他规定

第二百二十九条

因人民法院、仲裁机构的法律文书或者人民政府的征收决定等，导致物权设立、变更、转让或者消灭的，自法律文书或者征收决定等生效时发生效力。

法宝联想

法律法规　司法解释（3）规范性文件（2）

司法案例　司法案例（4029）案例报道（1）

法学期刊　法学期刊（81）

法宝律师　律所实务（8）

专题参考　专题参考（9）

第二百三十条　因继承取得物权的，自继承开始时发生效力。

法宝联想

法律法规　规范性文件（1）法律动态（1）

司法案例　司法案例（899）案例报道（1）仲裁案例（1）

法学期刊　法学期刊（66）

法宝律师　律所实务（2）

专题参考　专题参考（3）

第二百三十一条

因合法建造、拆除房屋等事实行为设立或者消灭物权的，自事实行为成就时发生效力。

法宝联想

法律法规　司法解释（3）规范性文件（2）

司法案例　司法案例（2346）案例报道（1）

法学期刊　法学期刊（45）

法宝律师　律所实务（4）

专题参考　专题参考（2）

第二百三十二条

处分依照本节规定享有的不动产物权，依照法律规定需要办理登记的，未经登记，不发生物权效力。

法宝联想

司法案例　司法案例（375）

法学期刊　法学期刊（36）

法宝律师　律所实务（2）

专题参考　专题参考（2）

第三章　物权的保护

第二百三十三条

物权受到侵害的，权利人可以通过和解、调解、仲裁、诉讼等途径解决。

法宝联想

法律法规　规范性文件（1）

司法案例　司法案例（4398）案例报道（1）

法学期刊　法学期刊（14）

第二百三十四条

因物权的归属、内容发生争议的，利害关系人可以请求确认权利。

法宝联想

法律法规 司法解释（2）规范性文件（2）法律动态（1）

司法案例 司法案例（6193）案例报道（1）

法学期刊 法学期刊（14）

法宝律师 律所实务（1）

专题参考 专题参考（2）

第二百三十五条 无权占有不动产或者动产的，权利人可以请求返还原物。

法宝联想

法律法规 司法解释（1）

司法案例 司法案例（22540）案例报道（4）

法学期刊 法学期刊（86）

法宝律师 律所实务（6）

专题参考 专题参考（3）

第二百三十六条

妨害物权或者可能妨害物权的，权利人可以请求排除妨害或者消除危险。

法宝联想

法律法规 规范性文件（1）法律动态（1）

司法案例 司法案例（19213）案例报道（6）

法学期刊 法学期刊（53）

法宝律师 律所实务（1）

专题参考 专题参考（4）

第二百三十七条

造成不动产或者动产毁损的，权利人可以依法请求修理、重作、更换或者恢复原状。

法宝联想

法律法规　规范性文件（2）

司法案例　司法案例（4572）案例报道（1）

法学期刊　法学期刊（22）

第二百三十八条

侵害物权，造成权利人损害的，权利人可以依法请求损害赔偿，也可以依法请求承担其他民事责任。

法宝联想

法律法规　司法解释（3）规范性文件（1）

司法案例　司法案例（19892）

法学期刊　法学期刊（39）

法宝律师　律所实务（2）

第二百三十九条

本章规定的物权保护方式，可以单独适用，也可以根据权利被侵害的情形合并适用。

法宝联想

法律法规　法律（1）

司法案例　司法案例（640）

法学期刊　法学期刊（5）

第二分编　所有权

第四章　一般规定

第二百四十条

所有权人对自己的不动产或者动产，依法享有占有、使用、收益和处分的权利。

法宝联想

法律法规　规范性文件（3）法律动态（2）

司法案例　司法案例（12613）案例报道（6）

法学期刊　法学期刊（71）

法宝律师　律所实务（3）

专题参考　专题参考（8）

第二百四十一条

所有权人有权在自己的不动产或者动产上设立用益物权和担保物权。用益物权人、担保物权人行使权利，不得损害所有权人的权益。

法宝联想

司法案例　司法案例（472）

法学期刊　法学期刊（11）

专题参考　专题参考（1）

第二百四十二条

法律规定专属于国家所有的不动产和动产，任何组织或者个人不能取得所有权。

法宝联想

司法案例　司法案例（77）

法学期刊　法学期刊（12）

法宝律师　律所实务（1）

第二百四十三条

为了公共利益的需要，依照法律规定的权限和程序可以征收集体所有的土地和组织、个人的房屋以及其他不动产。

征收集体所有的土地，应当依法及时足额支付土地补偿费、安置补助费以及农村村民住宅、其他地上附着物和青苗等的补偿费用，并安排被征地农民的社会保障费用，保障被征地农民的生活，维护被征地农民的合法权益。

征收组织、个人的房屋以及其他不动产，应当依法给予征收补偿，维护被征收人的合法权益；征收个人住宅的，还应当保障被征收人的居住条件。

任何组织或者个人不得贪污、挪用、私分、截留、拖欠征收补偿费等费用。

法宝联想

法律法规　规范性文件（5）

司法案例　司法案例（3431）案例报道（1）

法学期刊　法学期刊（70）

法宝律师　律所实务（3）

专题参考　专题参考（6）

第二百四十四条

国家对耕地实行特殊保护，严格限制农用地转为建设用地，控制建设用地总量。不得违反法律规定的权限和程序征收集体所有的土地。

法宝联想

司法案例　司法案例（78）

法学期刊　法学期刊（9）

第二百四十五条

因抢险救灾、疫情防控等紧急需要，依照法律规定的权限和程序可以征用组织、个人的不动产或者动产。被征用的不动产或者动产使用后，应当返还被征用人。组织、个人的不动产或者动产被征用或者征用后毁损、灭失的，应当给予补偿。

法宝联想

司法案例　司法案例（99）

法学期刊　法学期刊（31）

法宝律师　律所实务（2）

专题参考　专题参考（6）

第五章　国家所有权和集体所有权、私人所有权

第二百四十六条

法律规定属于国家所有的财产，属于国家所有即全民所有。

国有财产由国务院代表国家行使所有权。法律另有规定的，依照其规定。

法宝联想

司法案例　司法案例（48）

法学期刊　法学期刊（55）

法宝律师　律所实务（1）

专题参考　专题参考（1）

第二百四十七条　矿藏、水流、海域属于国家所有。

法宝联想

法律法规　规范性文件（2）

司法案例　司法案例（150）案例报道（1）

法学期刊　法学期刊（35）

法宝律师　律所实务（1）

专题参考　专题参考（1）

第二百四十八条

无居民海岛属于国家所有，国务院代表国家行使无居民海岛所有权。

法宝联想

司法案例　司法案例（25）

法学期刊　法学期刊（4）

第二百四十九条

城市的土地，属于国家所有。法律规定属于国家所有的农村和城市郊区的土地，属于国家所有。

法宝联想

司法案例　司法案例（101）

法学期刊　法学期刊（11）

专题参考　专题参考（1）

第二百五十条

森林、山岭、草原、荒地、滩涂等自然资源，属于国家所有，但是法律规定属于集体所有的除外。

法宝联想

法律法规　规范性文件（1）

司法案例　司法案例（113）

法学期刊　法学期刊（13）

第二百五十一条

法律规定属于国家所有的野生动植物资源，属于国家所有。

法宝联想

法律法规　规范性文件（1）

司法案例　司法案例（141）

法学期刊　法学期刊（11）

第二百五十二条　无线电频谱资源属于国家所有。

法宝联想

司法案例　司法案例（79）

法学期刊　法学期刊（10）

法宝律师　律所实务（2）

专题参考　专题参考（2）

第二百五十三条　法律规定属于国家所有的文物，属于国家所有。

法宝联想

司法案例　司法案例（158）

法学期刊　法学期刊（2）

第二百五十四条　国防资产属于国家所有。

铁路、公路、电力设施、电信设施和油气管道等基础设施，依照法律规定为国家所有的，属于国家所有。

法宝联想

法律法规　规范性文件（2）

司法案例　司法案例（131）

法学期刊　法学期刊（11）

法宝律师　律所实务（1）

专题参考　专题参考（1）

第二百五十五条

国家机关对其直接支配的不动产和动产，享有占有、使用以及依照法律和国务院的有关规定处分的权利。

法宝联想

司法案例 司法案例（66）

法学期刊 法学期刊（16）

专题参考 专题参考（2）

第二百五十六条

国家举办的事业单位对其直接支配的不动产和动产，享有占有、使用以及依照法律和国务院的有关规定收益、处分的权利。

法宝联想

司法案例 司法案例（131）

法学期刊 法学期刊（12）

专题参考 专题参考（2）

第二百五十七条

国家出资的企业，由国务院、地方人民政府依照法律、行政法规规定分别代表国家履行出资人职责，享有出资人权益。

法宝联想

司法案例 司法案例（45）

法学期刊 法学期刊（13）

专题参考 专题参考（2）

第二百五十八条

国家所有的财产受法律保护，禁止任何组织或者个人侵占、哄抢、私分、截留、破坏。

法宝联想

司法案例 司法案例（195）

法学期刊 法学期刊（6）

第二百五十九条

履行国有财产管理、监督职责的机构及其工作人员，应当依法加强对国有财产的管理、监督，促进国有财产保值增值，防止国有财产损失；滥用职权，玩忽职守，造成国有财产损失的，应当依法承担法律责任。

违反国有财产管理规定，在企业改制、合并分立、关联交易等过程中，低价转让、合谋私分、擅自担保或者以其他方式造成国有财产损失的，应当依法承担法律责任。

法宝联想

司法案例 司法案例（50）

法学期刊 法学期刊（2）

专题参考 专题参考（1）

第二百六十条 集体所有的不动产和动产包括：

（一）法律规定属于集体所有的土地和森林、山岭、草原、荒地、滩涂；

（二）集体所有的建筑物、生产设施、农田水利设施；

（三）集体所有的教育、科学、文化、卫生、体育等设施；

（四）集体所有的其他不动产和动产。

法宝联想

法律法规 规范性文件（1）

司法案例 司法案例（1302）

法学期刊 法学期刊（32）

第二百六十一条

农民集体所有的不动产和动产，属于本集体成员集体所有。

下列事项应当依照法定程序经本集体成员决定：

（一）土地承包方案以及将土地发包给本集体以外的组织或者个人承包；

（二）个别土地承包经营权人之间承包地的调整；

（三）土地补偿费等费用的使用、分配办法；

（四）集体出资的企业的所有权变动等事项；

（五）法律规定的其他事项。

法宝联想

司法案例 司法案例（5577）

法学期刊 法学期刊（107）

专题参考 专题参考（5）

第二百六十二条

对于集体所有的土地和森林、山岭、草原、荒地、滩涂等，依照下列规定行使所有权：

（一）属于村农民集体所有的，由村集体经济组织或者村民委员会依法代表集体行使所有权；

（二）分别属于村内两个以上农民集体所有的，由村内各该集体经济组织或者村民小组依法代表集体行使所有权；

（三）属于乡镇农民集体所有的，由乡镇集体经济组织代表集体行使所有权。

法宝联想

法律法规　规范性文件（5）

司法案例　司法案例（1259）

法学期刊　法学期刊（96）

专题参考　专题参考（1）

第二百六十三条

城镇集体所有的不动产和动产，依照法律、行政法规的规定由本集体享有占有、使用、收益和处分的权利。

法宝联想

司法案例　司法案例（261）

法学期刊　法学期刊（5）

专题参考　专题参考（2）

第二百六十四条

农村集体经济组织或者村民委员会、村民小组应当依照法律、行政法规以及章程、村规民约向本集体成员公布集体财产的状况。集体成员有权查阅、复制相关资料。

法宝联想

法律法规　规范性文件（1）法律动态（1）

司法案例　司法案例（297）案例报道（1）

法学期刊　法学期刊（24）

第二百六十五条

集体所有的财产受法律保护，禁止任何组织或者个人侵占、哄抢、私分、破坏。

农村集体经济组织、村民委员会或者其负责人作出的决定侵害集体成员合法权益的，受侵害的集体成员可以请求人民法院予以撤销。

法宝联想

司法案例　司法案例（2928）

法学期刊　法学期刊（44）

第二百六十六条

私人对其合法的收入、房屋、生活用品、生产工具、原材料等不动产和动产享有所有权。

法宝联想

法律法规　法律动态（1）

司法案例　司法案例（1266）

法学期刊　法学期刊（12）

第二百六十七条

私人的合法财产受法律保护，禁止任何组织或者个人侵占、哄抢、破坏。

法宝联想

法律法规　规范性文件（1）

司法案例　司法案例（2566）案例报道（2）

法学期刊　法学期刊（9）

专题参考　专题参考（2）

第二百六十八条

国家、集体和私人依法可以出资设立有限责任公司、股份有限公司或者其他企业。国家、集体和私人所有的不动产或者动产投到企业的，由出资人按照约定或者出资比例享有资产收益、重大决策以及选择经营管理者等权利并履行义务。

法宝联想

司法案例　司法案例（243）

法学期刊　法学期刊（17）

法宝律师　律所实务（1）

专题参考　专题参考（3）

第二百六十九条

营利法人对其不动产和动产依照法律、行政法规以及章程享有占有、使用、收益和处分的权利。

营利法人以外的法人，对其不动产和动产的权利，适用有关法律、行政法规以及章程的规定。

法宝联想

司法案例　司法案例（231）

法学期刊　法学期刊（15）

专题参考　专题参考（6）

第二百七十条

社会团体法人、捐助法人依法所有的不动产和动产，受法律保护。

法宝联想

法律法规　规范性文件（14）

司法案例　司法案例（44）

第六章　业主的建筑物区分所有权

第二百七十一条

业主对建筑物内的住宅、经营性用房等专有部分享有所有权，对专有部分以外的共有部分享有共有和共同管理的权利。

法宝联想

法律法规　规范性文件（8）

司法案例　司法案例（3767）案例报道（9）

法学期刊　法学期刊（14）

法宝律师　律所实务（5）

第二百七十二条

业主对其建筑物专有部分享有占有、使用、收益和处分的权利。业主行使权利不得危及建筑物的安全，不得损害其他业主的合法权益。

法宝联想

法律法规　规范性文件（1）

司法案例　司法案例（2377）案例报道（8）

法学期刊　法学期刊（7）

法宝律师　律所实务（2）

专题参考　专题参考（2）

第二百七十三条

业主对建筑物专有部分以外的共有部分，享有权利，承担义务；不得以放弃权利为由不履行义务。

业主转让建筑物内的住宅、经营性用房，其对共有部分享有的共有和共同管理的权利一并转让。

法宝联想

法律法规 规范性文件（11）

司法案例 司法案例（2143）案例报道（3）

法学期刊 法学期刊（8）

法宝律师 律所实务（1）

第二百七十四条

建筑区划内的道路，属于业主共有，但是属于城镇公共道路的除外。建筑区划内的绿地，属于业主共有，但是属于城镇公共绿地或者明示属于个人的除外。建筑区划内的其他公共场所、公用设施和物业服务用房，属于业主共有。

法宝联想

法律法规 规范性文件（5）

司法案例 司法案例（2008）案例报道（4）

法学期刊 法学期刊（8）

法宝律师 律所实务（8）

第二百七十五条

建筑区划内，规划用于停放汽车的车位、车库的归属，由当事人通过出售、附赠或者出租等方式约定。

占用业主共有的道路或者其他场地用于停放汽车的车位，属于业主共有。

法宝联想

法律法规 司法解释（2）规范性文件（14）法律动态（1）

司法案例 司法案例（1766）案例报道（6）

法学期刊 法学期刊（10）

法宝律师 律所实务（2）

专题参考 专题参考（3）

第二百七十六条

建筑区划内，规划用于停放汽车的车位、车库应当首先满足业主的需要。

法宝联想

法律法规 司法解释（2）规范性文件（3）法律动态（2）

司法案例 司法案例（733）案例报道（3）

法学期刊 法学期刊（2）

法宝律师 律所实务（1）

专题参考 专题参考（2）

第二百七十七条

业主可以设立业主大会，选举业主委员会。业主大会、业主委员会成立的具体条件和程序，依照法律、法规的规定。

地方人民政府有关部门、居民委员会应当对设立业主大会和选举业主委员会给予指导和协助。

法宝联想

法律法规 规范性文件（6）

司法案例 司法案例（985）

法学期刊 法学期刊（18）

专题参考 专题参考（3）

第二百七十八条　下列事项由业主共同决定：

（一）制定和修改业主大会议事规则；

（二）制定和修改管理规约；

（三）选举业主委员会或者更换业主委员会成员；

（四）选聘和解聘物业服务企业或者其他管理人；

（五）使用建筑物及其附属设施的维修资金；

（六）筹集建筑物及其附属设施的维修资金；

（七）改建、重建建筑物及其附属设施；

（八）改变共有部分的用途或者利用共有部分从事经营活动；

（九）有关共有和共同管理权利的其他重大事项。

业主共同决定事项，应当由专有部分面积占比三分之二以上的业主且人数占比三分之二以上的业主参与表决。决定前款第六项至第八项规定的事项，应当经参与表决专有部分面积四分之三以上的业主且参与表决人数四分之三以上的业主同意。决定前款其他事项，应当经参与表决专有部分面积过半数的业主且参与表决人数过半数的业主同意。

法宝联想

法律法规　司法解释（2）地方性法规（1）地方政府规章（1）规范性文件（110）草案（2）法律动态（4）

司法案例　司法案例（7851）案例报道（15）仲裁案例（3）

法学期刊　法学期刊（46）

法宝律师　律所实务（8）

专题参考　专题参考（9）

第二百七十九条

业主不得违反法律、法规以及管理规约，将住宅改变为经营性用房。业主将住宅

改变为经营性用房的，除遵守法律、法规以及管理规约外，应当经有利害关系的业主一致同意。

法宝联想

法律法规　司法解释（2）规范性文件（25）

司法案例　司法案例（383）案例报道（2）

法学期刊　法学期刊（17）

专题参考　专题参考（4）

第二百八十条　业主大会或者业主委员会的决定，对业主具有法律约束力。

业主大会或者业主委员会作出的决定侵害业主合法权益的，受侵害的业主可以请求人民法院予以撤销。

法宝联想

法律法规　司法解释（2）规范性文件（3）

司法案例　司法案例（4394）案例报道（4）仲裁案例（1）

法学期刊　法学期刊（18）

法宝律师　律所实务（2）

专题参考　专题参考（1）

第二百八十一条

建筑物及其附属设施的维修资金，属于业主共有。经业主共同决定，可以用于电梯、屋顶、外墙、无障碍设施等共有部分的维修、更新和改造。建筑物及其附属设施的维修资金的筹集、使用情况应当定期公布。

紧急情况下需要维修建筑物及其附属设施的，业主大会或者业主委员会可以依法申请使用建筑物及其附属设施的维修资金。

法宝联想

法律法规　地方政府规章（1）规范性文件（5）

司法案例　司法案例（654）案例报道（1）仲裁案例（2）

法学期刊　法学期刊（8）

法宝律师　律所实务（2）

第二百八十二条

建设单位、物业服务企业或者其他管理人等利用业主的共有部分产生的收入，在扣除合理成本之后，属于业主共有。

法宝联想

法律法规　规范性文件（46）

司法案例　司法案例（1142）案例报道（9）

法学期刊　法学期刊（8）

法宝律师　律所实务（2）

第二百八十三条

建筑物及其附属设施的费用分摊、收益分配等事项，有约定的，按照约定；没有约定或者约定不明确的，按照业主专有部分面积所占比例确定。

法宝联想

法律法规　司法解释（2）规范性文件（4）

司法案例　司法案例（890）案例报道（1）

法学期刊　法学期刊（2）

法宝律师　律所实务（2）

专题参考　专题参考（1）

第二百八十四条

业主可以自行管理建筑物及其附属设施，也可以委托物业服务企业或者其他管理人管理。

对建设单位聘请的物业服务企业或者其他管理人，业主有权依法更换。

法宝联想

法律法规 规范性文件（3）法律动态（1）

司法案例 司法案例（799）案例报道（2）

法学期刊 法学期刊（5）

第二百八十五条

物业服务企业或者其他管理人根据业主的委托，依照本法第三编有关物业服务合同的规定管理建筑区划内的建筑物及其附属设施，接受业主的监督，并及时答复业主对物业服务情况提出的询问。

物业服务企业或者其他管理人应当执行政府依法实施的应急处置措施和其他管理措施，积极配合开展相关工作。

法宝联想

法律法规 规范性文件（5）

司法案例 司法案例（527）

法学期刊 法学期刊（9）

专题参考 专题参考（1）

第二百八十六条

业主应当遵守法律、法规以及管理规约，相关行为应当符合节约资源、保护生态

环境的要求。对于物业服务企业或者其他管理人执行政府依法实施的应急处置措施和其他管理措施，业主应当依法予以配合。

业主大会或者业主委员会，对任意弃置垃圾、排放污染物或者噪声、违反规定饲养动物、违章搭建、侵占通道、拒付物业费等损害他人合法权益的行为，有权依照法律、法规以及管理规约，请求行为人停止侵害、排除妨碍、消除危险、恢复原状、赔偿损失。

业主或者其他行为人拒不履行相关义务的，有关当事人可以向有关行政主管部门报告或者投诉，有关行政主管部门应当依法处理。

法宝联想

法律法规　司法解释（2）规范性文件（3）法律动态（1）

司法案例　司法案例（1189）案例报道（6）仲裁案例（1）

法学期刊　法学期刊（39）

法宝律师　律所实务（1）

专题参考　专题参考（6）

第二百八十七条

业主对建设单位、物业服务企业或者其他管理人以及其他业主侵害自己合法权益的行为，有权请求其承担民事责任。

法宝联想

法律法规　规范性文件（5）法律动态（1）

司法案例　司法案例（1778）案例报道（3）

法学期刊　法学期刊（4）

法宝律师　律所实务（1）

专题参考　专题参考（3）

第七章　相邻关系

第二百八十八条

不动产的相邻权利人应当按照有利生产、方便生活、团结互助、公平合理的原则，正确处理相邻关系。

法宝联想

法律法规　规范性文件（8）法律动态（6）

司法案例　司法案例（20778）案例报道（24）

法学期刊　法学期刊（20）

法宝律师　律所实务（1）

专题参考　专题参考（2）

第二百八十九条

法律、法规对处理相邻关系有规定的，依照其规定；法律、法规没有规定的，可以按照当地习惯。

法宝联想

法律法规　规范性文件（2）草案（1）法律动态（1）

司法案例　司法案例（3152）案例报道（1）

法学期刊　法学期刊（17）

专题参考　专题参考（6）

第二百九十条

不动产权利人应当为相邻权利人用水、排水提供必要的便利。

对自然流水的利用，应当在不动产的相邻权利人之间合理分配。对自然流水的排放，应当尊重自然流向。

法宝联想

法律法规 规范性文件（2）

司法案例 司法案例（2340）案例报道（3）

法学期刊 法学期刊（9）

第二百九十一条

不动产权利人对相邻权利人因通行等必须利用其土地的，应当提供必要的便利。

法宝联想

法律法规 规范性文件（1）

司法案例 司法案例（3611）案例报道（1）

法学期刊 法学期刊（10）

专题参考 专题参考（1）

第二百九十二条

不动产权利人因建造、修缮建筑物以及铺设电线、电缆、水管、暖气和燃气管线等必须利用相邻土地、建筑物的，该土地、建筑物的权利人应当提供必要的便利。

法宝联想

司法案例 司法案例（1074）案例报道（1）

法学期刊 法学期刊（9）

第二百九十三条

建造建筑物，不得违反国家有关工程建设标准，不得妨碍相邻建筑物的通风、采光和日照。

法宝联想

法律法规 规范性文件（2）

司法案例 司法案例（1667）案例报道（2）

法学期刊 法学期刊（28）

法宝律师 律所实务（1）

专题参考 专题参考（1）

第二百九十四条

不动产权利人不得违反国家规定弃置固体废物，排放大气污染物、水污染物、土壤污染物、噪声、光辐射、电磁辐射等有害物质。

法宝联想

法律法规 规范性文件（1）

司法案例 司法案例（423）案例报道（3）

法学期刊 法学期刊（35）

法宝律师 律所实务（1）

专题参考 专题参考（4）

第二百九十五条

不动产权利人挖掘土地、建造建筑物、铺设管线以及安装设备等，不得危及相邻不动产的安全。

法宝联想

法律法规 法律动态（1）

司法案例 司法案例（1327）案例报道（1）

法学期刊 法学期刊（5）

法宝律师 律所实务（1）

第二百九十六条

不动产权利人因用水、排水、通行、铺设管线等利用相邻不动产的，应当尽量避免对相邻的不动产权利人造成损害。

法宝联想

法律法规　规范性文件（3）

司法案例　司法案例（3124）案例报道（3）

法学期刊　法学期刊（12）

法宝律师　律所实务（1）

专题参考　专题参考（2）

第八章　共有

第二百九十七条

不动产或者动产可以由两个以上组织、个人共有。共有包括按份共有和共同共有

。

法宝联想

法律法规　规范性文件（2）

司法案例　司法案例（3980）案例报道（1）

法学期刊　法学期刊（16）

法宝律师　律所实务（1）

专题参考　专题参考（1）

第二百九十八条

按份共有人对共有的不动产或者动产按照其份额享有所有权。

法宝联想

司法案例　司法案例（3367）

法学期刊　法学期刊（7）

第二百九十九条　共同共有人对共有的不动产或者动产共同享有所有权。

法宝联想

司法案例　司法案例（6910）案例报道（1）

法学期刊　法学期刊（9）

法宝律师　律所实务（1）

第三百条

共有人按照约定管理共有的不动产或者动产；没有约定或者约定不明确的，各共有人都有管理的权利和义务。

法宝联想

司法案例　司法案例（1004）案例报道（1）

法学期刊　法学期刊（13）

法宝律师　律所实务（1）

第三百零一条

处分共有的不动产或者动产以及对共有的不动产或者动产作重大修缮、变更性质或者用途的，应当经占份额三分之二以上的按份共有人或者全体共同共有人同意，但是共有人之间另有约定的除外。

法宝联想

法律法规　规范性文件（1）

司法案例　司法案例（2492）案例报道（3）仲裁案例（1）

法学期刊　法学期刊（50）

法宝律师　律所实务（5）

专题参考　专题参考（4）

第三百零二条

共有人对共有物的管理费用以及其他负担，有约定的，按照其约定；没有约定或者约定不明确的，按份共有人按照其份额负担，共同共有人共同负担。

法宝联想

司法案例　司法案例（492）

法学期刊　法学期刊（8）

第三百零三条

共有人约定不得分割共有的不动产或者动产，以维持共有关系的，应当按照约定，但是共有人有重大理由需要分割的，可以请求分割；没有约定或者约定不明确的，按份共有人可以随时请求分割，共同共有人在共有的基础丧失或者有重大理由需要分割时可以请求分割。因分割造成其他共有人损害的，应当给予赔偿。

法宝联想

法律法规　司法解释（2）规范性文件（2）

司法案例　司法案例（9277）案例报道（3）

法学期刊　法学期刊（36）

法宝律师　律所实务（2）

专题参考　专题参考（2）

第三百零四条

共有人可以协商确定分割方式。达不成协议，共有的不动产或者动产可以分割且不会因分割减损价值的，应当对实物予以分割；难以分割或者因分割会减损价值的，应当对折价或者拍卖、变卖取得的价款予以分割。

共有人分割所得的不动产或者动产有瑕疵的，其他共有人应当分担损失。

法宝联想

法律法规　规范性文件（1）

司法案例　司法案例（6346）案例报道（2）

法学期刊　法学期刊（28）

法宝律师　律所实务（3）

第三百零五条

按份共有人可以转让其享有的共有的不动产或者动产份额。其他共有人在同等条件下享有优先购买的权利。

法宝联想

法律法规 司法解释（1）

司法案例 司法案例（338）

法学期刊 法学期刊（19）

专题参考 专题参考（2）

第三百零六条

按份共有人转让其享有的共有的不动产或者动产份额的，应当将转让条件及时通知其他共有人。其他共有人应当在合理期限内行使优先购买权。

两个以上其他共有人主张行使优先购买权的，协商确定各自的购买比例；协商不成的，按照转让时各自的共有份额比例行使优先购买权。

法宝联想

法律法规 规范性文件（1）

司法案例 司法案例（92）

法学期刊 法学期刊（15）

第三百零七条

因共有的不动产或者动产产生的债权债务，在对外关系上，共有人享有连带债权、承担连带债务，但是法律另有规定或者第三人知道共有人不具有连带债权债务关系的除外；在共有人内部关系上，除共有人另有约定外，按份共有人按照份额享有债权、承担债务，共同共有人共同享有债权、承担债务。偿还债务超过自己应当承担份额的按份共有人，有权向其他共有人追偿。

法宝联想

司法案例 司法案例（1111）仲裁案例（1）

法学期刊 法学期刊（23）

法宝律师　律所实务（1）

专题参考　专题参考（3）

第三百零八条

共有人对共有的不动产或者动产没有约定为按份共有或者共同共有，或者约定不明确的，除共有人具有家庭关系等外，视为按份共有。

法宝联想

司法案例　司法案例（2683）案例报道（2）

法学期刊　法学期刊（26）

法宝律师　律所实务（4）

专题参考　专题参考（2）

第三百零九条

按份共有人对共有的不动产或者动产享有的份额，没有约定或者约定不明确的，按照出资额确定；不能确定出资额的，视为等额享有。

法宝联想

司法案例　司法案例（1793）

法学期刊　法学期刊（11）

法宝律师　律所实务（1）

第三百一十条

两个以上组织、个人共同享有用益物权、担保物权的，参照适用本章的有关规定。

法宝联想

司法案例　司法案例（142）

法学期刊　法学期刊（7）

法宝律师　律所实务（1）

专题参考　专题参考（1）

第九章　所有权取得的特别规定

第三百一十一条

无处分权人将不动产或者动产转让给受让人的，所有权人有权追回；除法律另有规定外，符合下列情形的，受让人取得该不动产或者动产的所有权：

（一）受让人受让该不动产或者动产时是善意；

（二）以合理的价格转让；

（三）转让的不动产或者动产依照法律规定应当登记的已经登记，不需要登记的已经交付给受让人。

受让人依据前款规定取得不动产或者动产的所有权的，原所有权人有权向无处分权人请求损害赔偿。

当事人善意取得其他物权的，参照适用前两款规定。

法宝联想

法律法规　司法解释（9）规范性文件（3）法律动态（2）

司法案例　司法案例（4551）案例报道（2）

法学期刊　法学期刊（216）

法宝律师　律所实务（18）

专题参考　专题参考（26）

第三百一十二条

所有权人或者其他权利人有权追回遗失物。该遗失物通过转让被他人占有的，权

利人有权向无处分权人请求损害赔偿，或者自知道或者应当知道受让人之日起二年内向受让人请求返还原物；但是，受让人通过拍卖或者向具有经营资格的经营者购得该遗失物的，权利人请求返还原物时应当支付受让人所付的费用。权利人向受让人支付所付费用后，有权向无处分权人追偿。

法宝联想

司法案例　司法案例（64）

法学期刊　法学期刊（36）

专题参考　专题参考（2）

第三百一十三条

善意受让人取得动产后，该动产上的原有权利消灭。但是，善意受让人在受让时知道或者应当知道该权利的除外。

法宝联想

司法案例　司法案例（91）

法学期刊　法学期刊（21）

第三百一十四条

拾得遗失物，应当返还权利人。拾得人应当及时通知权利人领取，或者送交公安等有关部门。

法宝联想

法律法规　规范性文件（2）法律动态（1）

司法案例　司法案例（177）案例报道（4）

法学期刊　法学期刊（11）

第三百一十五条

有关部门收到遗失物，知道权利人的，应当及时通知其领取；不知道的，应当及时发布招领公告。

法宝联想

司法案例 司法案例（11）

法学期刊 法学期刊（6）

第三百一十六条

拾得人在遗失物送交有关部门前，有关部门在遗失物被领取前，应当妥善保管遗失物。因故意或者重大过失致使遗失物毁损、灭失的，应当承担民事责任。

法宝联想

法律法规 规范性文件（1）法律动态（1）

司法案例 司法案例（122）案例报道（2）

法学期刊 法学期刊（6）

第三百一十七条

权利人领取遗失物时，应当向拾得人或者有关部门支付保管遗失物等支出的必要费用。

权利人悬赏寻找遗失物的，领取遗失物时应当按照承诺履行义务。

拾得人侵占遗失物的，无权请求保管遗失物等支出的费用，也无权请求权利人按照承诺履行义务。

法宝联想

司法案例 司法案例（24）案例报道（1）

法学期刊 法学期刊（9）

第三百一十八条

遗失物自发布招领公告之日起一年内无人认领的，归国家所有。

法宝联想

法律法规　规范性文件（3）

司法案例　司法案例（5）

法学期刊　法学期刊（8）

专题参考　专题参考（1）

第三百一十九条

拾得漂流物、发现埋藏物或者隐藏物的，参照适用拾得遗失物的有关规定。法律另有规定的，依照其规定。

法宝联想

司法案例　司法案例（4）

法学期刊　法学期刊（5）

专题参考　专题参考（2）

第三百二十条

主物转让的，从物随主物转让，但是当事人另有约定的除外。

法宝联想

司法案例　司法案例（122）

法学期刊　法学期刊（7）

专题参考　专题参考（1）

第三百二十一条

天然孳息，由所有权人取得；既有所有权人又有用益物权人的，由用益物权人取得。当事人另有约定的，按照其约定。

法定孳息，当事人有约定的，按照约定取得；没有约定或者约定不明确的，按照交易习惯取得。

法宝联想

司法案例　司法案例（331）案例报道（1）

法学期刊　法学期刊（25）

法宝律师　律所实务（1）

专题参考　专题参考（1）

第三百二十二条

因加工、附合、混合而产生的物的归属，有约定的，按照约定；没有约定或者约定不明确的，依照法律规定；法律没有规定的，按照充分发挥物的效用以及保护无过错当事人的原则确定。因一方当事人的过错或者确定物的归属造成另一方当事人损害的，应当给予赔偿或者补偿。

法宝联想

法律法规　规范性文件（1）法律动态（1）

司法案例　司法案例（866）案例报道（2）

法学期刊　法学期刊（66）

法宝律师　律所实务（2）

专题参考　专题参考（4）

第三分编　用益物权

第十章　一般规定

第三百二十三条

用益物权人对他人所有的不动产或者动产，依法享有占有、使用和收益的权利。

法宝联想

司法案例 司法案例（1187）仲裁案例（2）

法学期刊 法学期刊（40）

专题参考 专题参考（2）

第三百二十四条

国家所有或者国家所有由集体使用以及法律规定属于集体所有的自然资源，组织、个人依法可以占有、使用和收益。

法宝联想

司法案例 司法案例（200）

法学期刊 法学期刊（13）

第三百二十五条

国家实行自然资源有偿使用制度，但是法律另有规定的除外。

法宝联想

司法案例 司法案例（44）

法学期刊 法学期刊（11）

专题参考 专题参考（1）

第三百二十六条

用益物权人行使权利，应当遵守法律有关保护和合理开发利用资源、保护生态环境的规定。所有权人不得干涉用益物权人行使权利。

法宝联想

司法案例 司法案例（107）案例报道（1）

法学期刊 法学期刊（28）

专题参考 专题参考（2）

第三百二十七条

因不动产或者动产被征收、征用致使用益物权消灭或者影响用益物权行使的，用益物权人有权依据本法第二百四十三条、第二百四十五条的规定获得相应补偿。

法宝联想

法律法规　规范性文件（1）

司法案例　司法案例（394）案例报道（1）

法学期刊　法学期刊（27）

法宝律师　律所实务（2）

专题参考　专题参考（4）

第三百二十八条　依法取得的海域使用权受法律保护。

法宝联想

司法案例　司法案例（34）

法学期刊　法学期刊（12）

法宝律师　律所实务（1）

专题参考　专题参考（1）

第三百二十九条

依法取得的探矿权、采矿权、取水权和使用水域、滩涂从事养殖、捕捞的权利受法律保护。

法宝联想

法律法规　规范性文件（1）

司法案例　司法案例（116）

法学期刊　法学期刊（27）

法宝律师　律所实务（1）

专题参考　专题参考（1）

第十一章　土地承包经营权

第三百三十条

农村集体经济组织实行家庭承包经营为基础、统分结合的双层经营体制。

农民集体所有和国家所有由农民集体使用的耕地、林地、草地以及其他用于农业的土地，依法实行土地承包经营制度。

法宝联想

司法案例　司法案例（1526）案例报道（1）

法学期刊　法学期刊（9）

专题参考　专题参考（2）

第三百三十一条

土地承包经营权人依法对其承包经营的耕地、林地、草地等享有占有、使用和收益的权利，有权从事种植业、林业、畜牧业等农业生产。

法宝联想

法律法规　规范性文件（1）

司法案例　司法案例（4153）

法学期刊　法学期刊（16）

第三百三十二条

耕地的承包期为三十年。草地的承包期为三十年至五十年。林地的承包期为三十年至七十年。

前款规定的承包期限届满，由土地承包经营权人依照农村土地承包的法律规定继续承包。

法宝联想

司法案例　司法案例（575）仲裁案例（1）

法学期刊　法学期刊（7）

专题参考　专题参考（3）

第三百三十三条　土地承包经营权自土地承包经营权合同生效时设立。

登记机构应当向土地承包经营权人发放土地承包经营权证、林权证等证书，并登记造册，确认土地承包经营权。

法宝联想

司法案例　司法案例（3254）

法学期刊　法学期刊（22）

第三百三十四条

土地承包经营权人依照法律规定，有权将土地承包经营权互换、转让。未经依法批准，不得将承包地用于非农建设。

法宝联想

法律法规　规范性文件（1）法律动态（2）

司法案例　司法案例（1244）案例报道（1）

法学期刊　法学期刊（12）

专题参考　专题参考（1）

第三百三十五条

土地承包经营权互换、转让的，当事人可以向登记机构申请登记；未经登记，不得对抗善意第三人。

法宝联想

司法案例　司法案例（307）

法学期刊　法学期刊（24）

专题参考 专题参考（1）

第三百三十六条 承包期内发包人不得调整承包地。

因自然灾害严重毁损承包地等特殊情形，需要适当调整承包的耕地和草地的，应当依照农村土地承包的法律规定办理。

法宝联想

司法案例 司法案例（364）

法学期刊 法学期刊（7）

专题参考 专题参考（2）

第三百三十七条

承包期内发包人不得收回承包地。法律另有规定的，依照其规定。

法宝联想

司法案例 司法案例（504）

法学期刊 法学期刊（3）

第三百三十八条

承包地被征收的，土地承包经营权人有权依据本法第二百四十三条的规定获得相应补偿。

法宝联想

司法案例 司法案例（2081）案例报道（1）

法学期刊 法学期刊（16）

法宝律师 律所实务（1）

专题参考 专题参考（1）

第三百三十九条

土地承包经营权人可以自主决定依法采取出租、入股或者其他方式向他人流转土地经营权。

法宝联想

法律法规　规范性文件（1）

司法案例　司法案例（2242）案例报道（1）

法学期刊　法学期刊（59）

专题参考　专题参考（1）

第三百四十条

土地经营权人有权在合同约定的期限内占有农村土地，自主开展农业生产经营并取得收益。

法宝联想

司法案例　司法案例（1066）案例报道（1）

法学期刊　法学期刊（35）

第三百四十一条

流转期限为五年以上的土地经营权，自流转合同生效时设立。当事人可以向登记机构申请土地经营权登记；未经登记，不得对抗善意第三人。

法宝联想

法律法规　规范性文件（1）

司法案例　司法案例（246）

法学期刊　法学期刊（60）

专题参考　专题参考（2）

第三百四十二条

通过招标、拍卖、公开协商等方式承包农村土地，经依法登记取得权属证书的，可以依法采取出租、入股、抵押或者其他方式流转土地经营权。

法宝联想

法律法规　规范性文件（1）

司法案例　司法案例（166）

法学期刊　法学期刊（38）

第三百四十三条

国家所有的农用地实行承包经营的，参照适用本编的有关规定。

法宝联想

司法案例　司法案例（144）

法学期刊　法学期刊（9）

专题参考　专题参考（1）

第十二章　建设用地使用权

第三百四十四条

建设用地使用权人依法对国家所有的土地享有占有、使用和收益的权利，有权利用该土地建造建筑物、构筑物及其附属设施。

法宝联想

司法案例　司法案例（396）

法学期刊　法学期刊（17）

法宝律师　律所实务（1）

专题参考　专题参考（1）

第三百四十五条

建设用地使用权可以在土地的地表、地上或者地下分别设立。

法宝联想

法律法规　规范性文件（5）

司法案例　司法案例（39）

法学期刊　法学期刊（14）

专题参考　专题参考（1）

第三百四十六条

设立建设用地使用权，应当符合节约资源、保护生态环境的要求，遵守法律、行政法规关于土地用途的规定，不得损害已经设立的用益物权。

法宝联想

法律法规　规范性文件（1）

司法案例　司法案例（22）

法学期刊　法学期刊（30）

专题参考　专题参考（3）

第三百四十七条　设立建设用地使用权，可以采取出让或者划拨等方式。

工业、商业、旅游、娱乐和商品住宅等经营性用地以及同一土地有两个以上意向用地者的，应当采取招标、拍卖等公开竞价的方式出让。

严格限制以划拨方式设立建设用地使用权。

法宝联想

法律法规　规范性文件（2）法律动态（1）

司法案例　司法案例（80）仲裁案例（1）

法学期刊　法学期刊（14）

法宝律师　律所实务（2）

第三百四十八条

通过招标、拍卖、协议等出让方式设立建设用地使用权的，当事人应当采用书面形式订立建设用地使用权出让合同。

建设用地使用权出让合同一般包括下列条款：

（一）当事人的名称和住所；

（二）土地界址、面积等；

（三）建筑物、构筑物及其附属设施占用的空间；

（四）土地用途、规划条件；

（五）建设用地使用权期限；

（六）出让金等费用及其支付方式；

（七）解决争议的方法。

法宝联想

法律法规　规范性文件（1）

司法案例　司法案例（79）

法学期刊　法学期刊（19）

法宝律师　律所实务（1）

专题参考　专题参考（1）

第三百四十九条

设立建设用地使用权的，应当向登记机构申请建设用地使用权登记。建设用地使用权自登记时设立。登记机构应当向建设用地使用权人发放权属证书。

法宝联想

法律法规　规范性文件（1）

司法案例　司法案例（201）

法学期刊　法学期刊（7）

第三百五十条

建设用地使用权人应当合理利用土地，不得改变土地用途；需要改变土地用途的，应当依法经有关行政主管部门批准。

法宝联想

司法案例　司法案例（50）

法学期刊　法学期刊（6）

法宝律师　律所实务（1）

第三百五十一条

建设用地使用权人应当依照法律规定以及合同约定支付出让金等费用。

法宝联想

法律法规　规范性文件（1）

司法案例　司法案例（101）

法学期刊　法学期刊（1）

法宝律师　律所实务（1）

第三百五十二条

建设用地使用权人建造的建筑物、构筑物及其附属设施的所有权属于建设用地使用权人，但是有相反证据证明的除外。

法宝联想

法律法规　规范性文件（1）法律动态（1）

司法案例　司法案例（529）案例报道（1）

法学期刊　法学期刊（25）

法宝律师　律所实务（3）

第三百五十三条

建设用地使用权人有权将建设用地使用权转让、互换、出资、赠与或者抵押，但是法律另有规定的除外。

法宝联想

司法案例　司法案例（147）

法学期刊　法学期刊（17）

专题参考　专题参考（2）

第三百五十四条

建设用地使用权转让、互换、出资、赠与或者抵押的，当事人应当采用书面形式订立相应的合同。使用期限由当事人约定，但是不得超过建设用地使用权的剩余期限。

法宝联想

司法案例　司法案例（93）

法学期刊　法学期刊（5）

专题参考　专题参考（2）

第三百五十五条

建设用地使用权转让、互换、出资或者赠与的，应当向登记机构申请变更登记。

法宝联想

司法案例　司法案例（115）

法学期刊　法学期刊（2）

专题参考　专题参考（1）

第三百五十六条

建设用地使用权转让、互换、出资或者赠与的，附着于该土地上的建筑物、构筑物及其附属设施一并处分。

法宝联想

- 法律法规　规范性文件（1）
- 司法案例　司法案例（568）
- 法学期刊　法学期刊（11）
- 法宝律师　律所实务（1）
- 专题参考　专题参考（1）

第三百五十七条

建筑物、构筑物及其附属设施转让、互换、出资或者赠与的，该建筑物、构筑物及其附属设施占用范围内的建设用地使用权一并处分。

法宝联想

- 司法案例　司法案例（1754）
- 法学期刊　法学期刊（12）
- 专题参考　专题参考（1）

第三百五十八条

建设用地使用权期限届满前，因公共利益需要提前收回该土地的，应当依据本法第二百四十三条的规定对该土地上的房屋以及其他不动产给予补偿，并退还相应的出让金。

法宝联想

- 司法案例　司法案例（26）
- 法学期刊　法学期刊（13）
- 法宝律师　律所实务（1）

第三百五十九条

住宅建设用地使用权期限届满的，自动续期。续期费用的缴纳或者减免，依照法律、行政法规的规定办理。

非住宅建设用地使用权期限届满后的续期，依照法律规定办理。该土地上的房屋以及其他不动产的归属，有约定的，按照约定；没有约定或者约定不明确的，依照法律、行政法规的规定办理。

法宝联想

司法案例　司法案例（285）

法学期刊　法学期刊（21）

法宝律师　律所实务（2）

专题参考　专题参考（6）

第三百六十条

建设用地使用权消灭的，出让人应当及时办理注销登记。登记机构应当收回权属证书。

法宝联想

司法案例　司法案例（36）

法学期刊　法学期刊（1）

专题参考　专题参考（1）

第三百六十一条

集体所有的土地作为建设用地的，应当依照土地管理的法律规定办理。

法宝联想

司法案例　司法案例（223）

法学期刊　法学期刊（23）

法宝律师　律所实务（1）

专题参考　专题参考（2）

第十三章　宅基地使用权

第三百六十二条

宅基地使用权人依法对集体所有的土地享有占有和使用的权利，有权依法利用该土地建造住宅及其附属设施。

法宝联想

司法案例　司法案例（1788）

法学期刊　法学期刊（33）

专题参考　专题参考（3）

第三百六十三条

宅基地使用权的取得、行使和转让，适用土地管理的法律和国家有关规定。

法宝联想

法律法规　规范性文件（1）

司法案例　司法案例（1570）

法学期刊　法学期刊（27）

专题参考　专题参考（1）

第三百六十四条

宅基地因自然灾害等原因灭失的，宅基地使用权消灭。对失去宅基地的村民，应当依法重新分配宅基地。

法宝联想

法律法规　规范性文件（1）

司法案例　司法案例（88）

法学期刊　法学期刊（8）

专题参考　专题参考（1）

第三百六十五条

已经登记的宅基地使用权转让或者消灭的，应当及时办理变更登记或者注销登记

。

法宝联想

司法案例　司法案例（241）

法学期刊　法学期刊（10）

专题参考　专题参考（1）

第十四章　居住权

第三百六十六条

居住权人有权按照合同约定，对他人的住宅享有占有、使用的用益物权，以满足生活居住的需要。

法宝联想

法律法规　规范性文件（11）草案（1）法律动态（1）

司法案例　司法案例（954）案例报道（14）

法学期刊　法学期刊（70）

法宝律师　律所实务（2）

专题参考　专题参考（8）

第三百六十七条　设立居住权，当事人应当采用书面形式订立居住权合同。

居住权合同一般包括下列条款：

（一）当事人的姓名或者名称和住所；

（二）住宅的位置；

（三）居住的条件和要求；

（四）居住权期限；

（五）解决争议的方法。

法宝联想

法律法规　规范性文件（2）草案（1）

司法案例　司法案例（514）案例报道（3）

法学期刊　法学期刊（52）

法宝律师　律所实务（3）

专题参考　专题参考（3）

第三百六十八条

居住权无偿设立，但是当事人另有约定的除外。设立居住权的，应当向登记机构申请居住权登记。居住权自登记时设立。

法宝联想

法律法规　规范性文件（6）法律动态（3）

司法案例　司法案例（687）案例报道（6）

法学期刊　法学期刊（72）

法宝律师　律所实务（2）

专题参考　专题参考（4）

第三百六十九条

居住权不得转让、继承。设立居住权的住宅不得出租，但是当事人另有约定的除外。

法宝联想

法律法规　规范性文件（2）

司法案例　司法案例（115）案例报道（2）

法学期刊　法学期刊（49）

法宝律师　律所实务（2）

第三百七十条

居住权期限届满或者居住权人死亡的，居住权消灭。居住权消灭的，应当及时办理注销登记。

法宝联想

法律法规　规范性文件（2）

司法案例　司法案例（78）

法学期刊　法学期刊（30）

法宝律师　律所实务（1）

第三百七十一条　以遗嘱方式设立居住权的，参照适用本章的有关规定。

法宝联想

法律法规　规范性文件（6）

司法案例　司法案例（118）案例报道（7）

法学期刊　法学期刊（51）

法宝律师　律所实务（3）

专题参考　专题参考（3）

第十五章　地役权

第三百七十二条

地役权人有权按照合同约定，利用他人的不动产，以提高自己的不动产的效益。

前款所称他人的不动产为供役地，自己的不动产为需役地。

法宝联想

司法案例　司法案例（108）

法学期刊　法学期刊（11）

专题参考　专题参考（1）

第三百七十三条 设立地役权，当事人应当采用书面形式订立地役权合同。

地役权合同一般包括下列条款：

（一）当事人的姓名或者名称和住所；

（二）供役地和需役地的位置；

（三）利用目的和方法；

（四）地役权期限；

（五）费用及其支付方式；

（六）解决争议的方法。

法宝联想

司法案例 司法案例（47）

法学期刊 法学期刊（12）

专题参考 专题参考（1）

第三百七十四条

地役权自地役权合同生效时设立。当事人要求登记的，可以向登记机构申请地役权登记；未经登记，不得对抗善意第三人。

法宝联想

司法案例 司法案例（42）

法学期刊 法学期刊（23）

专题参考 专题参考（1）

第三百七十五条

供役地权利人应当按照合同约定，允许地役权人利用其不动产，不得妨害地役权人行使权利。

法宝联想

司法案例 司法案例（58）

法学期刊 法学期刊（1）

第三百七十六条

地役权人应当按照合同约定的利用目的和方法利用供役地，尽量减少对供役地权利人物权的限制。

法宝联想

司法案例 司法案例（22）

法学期刊 法学期刊（2）

专题参考 专题参考（1）

第三百七十七条

地役权期限由当事人约定；但是，不得超过土地承包经营权、建设用地使用权等用益物权的剩余期限。

法宝联想

司法案例 司法案例（27）

法学期刊 法学期刊（5）

专题参考 专题参考（1）

第三百七十八条

土地所有权人享有地役权或者负担地役权的，设立土地承包经营权、宅基地使用权等用益物权时，该用益物权人继续享有或者负担已经设立的地役权。

法宝联想

司法案例 司法案例（8）

法学期刊 法学期刊（1）

专题参考 专题参考（1）

第三百七十九条

土地上已经设立土地承包经营权、建设用地使用权、宅基地使用权等用益物权的，未经用益物权人同意，土地所有权人不得设立地役权。

法宝联想

司法案例 司法案例（10）

法学期刊 法学期刊（1）

第三百八十条

地役权不得单独转让。土地承包经营权、建设用地使用权等转让的，地役权一并转让，但是合同另有约定的除外。

法宝联想

司法案例 司法案例（57）

法学期刊 法学期刊（2）

第三百八十一条

地役权不得单独抵押。土地经营权、建设用地使用权等抵押的，在实现抵押权时，地役权一并转让。

法宝联想

司法案例 司法案例（5）

法学期刊 法学期刊（7）

第三百八十二条

需役地以及需役地上的土地承包经营权、建设用地使用权等部分转让时，转让部分涉及地役权的，受让人同时享有地役权。

法宝联想

司法案例 司法案例（13）

法学期刊　法学期刊（1）

第三百八十三条

供役地以及供役地上的土地承包经营权、建设用地使用权等部分转让时，转让部分涉及地役权的，地役权对受让人具有法律约束力。

法宝联想

司法案例　司法案例（19）

法学期刊　法学期刊（2）

第三百八十四条

地役权人有下列情形之一的，供役地权利人有权解除地役权合同，地役权消灭：

（一）违反法律规定或者合同约定，滥用地役权；

（二）有偿利用供役地，约定的付款期限届满后在合理期限内经两次催告未支付费用。

法宝联想

司法案例　司法案例（24）

法学期刊　法学期刊（6）

第三百八十五条

已经登记的地役权变更、转让或者消灭的，应当及时办理变更登记或者注销登记。

法宝联想

法律法规　规范性文件（1）

司法案例　司法案例（19）

法学期刊　法学期刊（5）

第四分编　担保物权

第十六章　一般规定

第三百八十六条

担保物权人在债务人不履行到期债务或者发生当事人约定的实现担保物权的情形，依法享有就担保财产优先受偿的权利，但是法律另有规定的除外。

法宝联想

司法案例　司法案例（58884）仲裁案例（1）

法学期刊　法学期刊（38）

法宝律师　律所实务（3）

专题参考　专题参考（2）

第三百八十七条

债权人在借贷、买卖等民事活动中，为保障实现其债权，需要担保的，可以依照本法和其他法律的规定设立担保物权。

第三人为债务人向债权人提供担保的，可以要求债务人提供反担保。反担保适用本法和其他法律的规定。

法宝联想

法律法规　规范性文件（1）

司法案例　司法案例（6231）仲裁案例（1）

法学期刊　法学期刊（14）

法宝律师　律所实务（1）

专题参考　专题参考（2）

第三百八十八条

设立担保物权，应当依照本法和其他法律的规定订立担保合同。担保合同包括抵押合同、质押合同和其他具有担保功能的合同。担保合同是主债权债务合同的从合同。主债权债务合同无效的，担保合同无效，但是法律另有规定的除外。

担保合同被确认无效后，债务人、担保人、债权人有过错的，应当根据其过错各自承担相应的民事责任。

法宝联想

法律法规　规范性文件（1）法律动态（1）

司法案例　司法案例（4437）仲裁案例（2）

法学期刊　法学期刊（221）

法宝律师　律所实务（23）

专题参考　专题参考（25）

第三百八十九条

担保物权的担保范围包括主债权及其利息、违约金、损害赔偿金、保管担保财产和实现担保物权的费用。当事人另有约定的，按照其约定。

法宝联想

司法案例　司法案例（94902）案例报道（1）仲裁案例（2）

法学期刊　法学期刊（38）

法宝律师　律所实务（3）

专题参考　专题参考（11）

第三百九十条

担保期间，担保财产毁损、灭失或者被征收等，担保物权人可以就获得的保险金

、赔偿金或者补偿金等优先受偿。被担保债权的履行期限未届满的，也可以提存该保险金、赔偿金或者补偿金等。

法宝联想

法律法规　规范性文件（1）

司法案例　司法案例（324）案例报道（1）

法学期刊　法学期刊（62）

法宝律师　律所实务（2）

专题参考　专题参考（6）

第三百九十一条

第三人提供担保，未经其书面同意，债权人允许债务人转移全部或者部分债务的，担保人不再承担相应的担保责任。

法宝联想

司法案例　司法案例（100）

法学期刊　法学期刊（8）

第三百九十二条

被担保的债权既有物的担保又有人的担保的，债务人不履行到期债务或者发生当事人约定的实现担保物权的情形，债权人应当按照约定实现债权；没有约定或者约定不明确，债务人自己提供物的担保的，债权人应当先就该物的担保实现债权；第三人提供物的担保的，债权人可以就物的担保实现债权，也可以请求保证人承担保证责任。提供担保的第三人承担担保责任后，有权向债务人追偿。

法宝联想

法律法规　司法解释（3）规范性文件（2）

司法案例　司法案例（32544）案例报道（3）仲裁案例（3）

法学期刊　法学期刊（57）

法宝律师 律所实务（4）

专题参考 专题参考（17）

第三百九十三条 有下列情形之一的，担保物权消灭：

（一）主债权消灭；

（二）担保物权实现；

（三）债权人放弃担保物权；

（四）法律规定担保物权消灭的其他情形。

法宝联想

法律法规 规范性文件（1）法律动态（1）

司法案例 司法案例（1506）

法学期刊 法学期刊（19）

法宝律师 律所实务（4）

专题参考 专题参考（8）

第十七章 抵押权

第一节 一般抵押权

第三百九十四条

为担保债务的履行，债务人或者第三人不转移财产的占有，将该财产抵押给债权人的，债务人不履行到期债务或者发生当事人约定的实现抵押权的情形，债权人有权就该财产优先受偿。

前款规定的债务人或者第三人为抵押人，债权人为抵押权人，提供担保的财产为抵押财产。

法宝联想

司法案例　司法案例（272484）案例报道（1）仲裁案例（2）

法学期刊　法学期刊（29）

法宝律师　律所实务（8）

专题参考　专题参考（4）

第三百九十五条　债务人或者第三人有权处分的下列财产可以抵押：

（一）建筑物和其他土地附着物；

（二）建设用地使用权；

（三）海域使用权；

（四）生产设备、原材料、半成品、产品；

（五）正在建造的建筑物、船舶、航空器；

（六）交通运输工具；

（七）法律、行政法规未禁止抵押的其他财产。

抵押人可以将前款所列财产一并抵押。

法宝联想

法律法规　规范性文件（3）

司法案例　司法案例（41867）仲裁案例（2）

法学期刊　法学期刊（94）

法宝律师　律所实务（7）

专题参考　专题参考（14）

第三百九十六条

企业、个体工商户、农业生产经营者可以将现有的以及将有的生产设备、原材料、半成品、产品抵押，债务人不履行到期债务或者发生当事人约定的实现抵押权的情形，债权人有权就抵押财产确定时的动产优先受偿。

法宝联想

司法案例　司法案例（544）

法学期刊　法学期刊（44）

法宝律师　律所实务（1）

专题参考　专题参考（6）

第三百九十七条

以建筑物抵押的，该建筑物占用范围内的建设用地使用权一并抵押。以建设用地使用权抵押的，该土地上的建筑物一并抵押。

抵押人未依据前款规定一并抵押的，未抵押的财产视为一并抵押。

法宝联想

法律法规　规范性文件（1）

司法案例　司法案例（2119）案例报道（1）

法学期刊　法学期刊（23）

法宝律师　律所实务（4）

专题参考　专题参考（7）

第三百九十八条

乡镇、村企业的建设用地使用权不得单独抵押。以乡镇、村企业的厂房等建筑物抵押的，其占用范围内的建设用地使用权一并抵押。

法宝联想

司法案例　司法案例（70）

法学期刊　法学期刊（10）

专题参考　专题参考（2）

第三百九十九条　下列财产不得抵押：

（一）土地所有权；

（二）宅基地、自留地、自留山等集体所有土地的使用权，但是法律规定可以抵押的除外；

（三）学校、幼儿园、医疗机构等为公益目的成立的非营利法人的教育设施、医疗卫生设施和其他公益设施；

（四）所有权、使用权不明或者有争议的财产；

（五）依法被查封、扣押、监管的财产；

（六）法律、行政法规规定不得抵押的其他财产。

法宝联想

法律法规 规范性文件（7）法规解读（1）法律动态（1）

司法案例 司法案例（1016）

法学期刊 法学期刊（90）

法宝律师 律所实务（7）

专题参考 专题参考（18）

第四百条 设立抵押权，当事人应当采用书面形式订立抵押合同。

抵押合同一般包括下列条款：

（一）被担保债权的种类和数额；

（二）债务人履行债务的期限；

（三）抵押财产的名称、数量等情况；

（四）担保的范围。

法宝联想

法律法规 规范性文件（3）

司法案例 司法案例（11550）案例报道（2）仲裁案例（2）

法学期刊　法学期刊（44）

法宝律师　律所实务（3）

专题参考　专题参考（4）

第四百零一条

抵押权人在债务履行期限届满前，与抵押人约定债务人不履行到期债务时抵押财产归债权人所有的，只能依法就抵押财产优先受偿。

法宝联想

法律法规　司法解释（1）法规解读（2）法律动态（1）

司法案例　司法案例（1931）

法学期刊　法学期刊（71）

法宝律师　律所实务（3）

专题参考　专题参考（11）

第四百零二条

以本法第三百九十五条第一款第一项至第三项规定的财产或者第五项规定的正在建造的建筑物抵押的，应当办理抵押登记。抵押权自登记时设立。

法宝联想

法律法规　规范性文件（1）

司法案例　司法案例（68681）仲裁案例（3）

法学期刊　法学期刊（50）

法宝律师　律所实务（3）

专题参考　专题参考（7）

第四百零三条

以动产抵押的，抵押权自抵押合同生效时设立；未经登记，不得对抗善意第三人。

法宝联想

法律法规　规范性文件（1）

司法案例　司法案例（21028）案例报道（1）

法学期刊　法学期刊（128）

法宝律师　律所实务（8）

专题参考　专题参考（7）

第四百零四条

以动产抵押的，不得对抗正常经营活动中已经支付合理价款并取得抵押财产的买受人。

法宝联想

司法案例　司法案例（199）

法学期刊　法学期刊（87）

法宝律师　律所实务（3）

专题参考　专题参考（10）

第四百零五条

抵押权设立前，抵押财产已经出租并转移占有的，原租赁关系不受该抵押权的影响。

法宝联想

法律法规　规范性文件（1）

司法案例　司法案例（1583）

法学期刊　法学期刊（44）

法宝律师　律所实务（9）

专题参考　专题参考（7）

第四百零六条

抵押期间，抵押人可以转让抵押财产。当事人另有约定的，按照其约定。抵押财产转让的，抵押权不受影响。

抵押人转让抵押财产的，应当及时通知抵押权人。抵押权人能够证明抵押财产转让可能损害抵押权的，可以请求抵押人将转让所得的价款向抵押权人提前清偿债务或者提存。转让的价款超过债权数额的部分归抵押人所有，不足部分由债务人清偿。

法宝联想
法律法规　规范性文件（13）草案（1）法规解读（1）法律动态（4）
司法案例　司法案例（3384）案例报道（4）仲裁案例（1）
法学期刊　法学期刊（187）
法宝律师　律所实务（19）
专题参考　专题参考（21）

第四百零七条

抵押权不得与债权分离而单独转让或者作为其他债权的担保。债权转让的，担保该债权的抵押权一并转让，但是法律另有规定或者当事人另有约定的除外。

法宝联想
法律法规　规范性文件（1）
司法案例　司法案例（3386）
法学期刊　法学期刊（22）
法宝律师　律所实务（4）
专题参考　专题参考（4）

第四百零八条

抵押人的行为足以使抵押财产价值减少的，抵押权人有权请求抵押人停止其行为；抵押财产价值减少的，抵押权人有权请求恢复抵押财产的价值，或者提供与减少的价值相应的担保。抵押人不恢复抵押财产的价值，也不提供担保的，抵押权人有权请求债务人提前清偿债务。

法宝联想

法律法规　规范性文件（1）

司法案例　司法案例（66）案例报道（1）

法学期刊　法学期刊（39）

法宝律师　律所实务（1）

专题参考　专题参考（1）

第四百零九条

抵押权人可以放弃抵押权或者抵押权的顺位。抵押权人与抵押人可以协议变更抵押权顺位以及被担保的债权数额等内容。但是，抵押权的变更未经其他抵押权人书面同意的，不得对其他抵押权人产生不利影响。

债务人以自己的财产设定抵押，抵押权人放弃该抵押权、抵押权顺位或者变更抵押权的，其他担保人在抵押权人丧失优先受偿权益的范围内免除担保责任，但是其他担保人承诺仍然提供担保的除外。

法宝联想

法律法规　规范性文件（1）

司法案例　司法案例（698）

法学期刊　法学期刊（21）

法宝律师　律所实务（2）

专题参考　专题参考（2）

第四百一十条

债务人不履行到期债务或者发生当事人约定的实现抵押权的情形，抵押权人可以与抵押人协议以抵押财产折价或者以拍卖、变卖该抵押财产所得的价款优先受偿。协议损害其他债权人利益的，其他债权人可以请求人民法院撤销该协议。

抵押权人与抵押人未就抵押权实现方式达成协议的，抵押权人可以请求人民法院拍卖、变卖抵押财产。

抵押财产折价或者变卖的，应当参照市场价格。

法宝联想

法律法规　规范性文件（1）法律动态（1）

司法案例　司法案例（201646）案例报道（1）

法学期刊　法学期刊（68）

法宝律师　律所实务（4）

专题参考　专题参考（10）

第四百一十一条

依据本法第三百九十六条规定设定抵押的，抵押财产自下列情形之一发生时确定：

（一）债务履行期限届满，债权未实现；

（二）抵押人被宣告破产或者解散；

（三）当事人约定的实现抵押权的情形；

（四）严重影响债权实现的其他情形。

法宝联想

司法案例　司法案例（1063）

法学期刊　法学期刊（18）

法宝律师　律所实务（2）

专题参考　专题参考（3）

第四百一十二条

债务人不履行到期债务或者发生当事人约定的实现抵押权的情形，致使抵押财产被人民法院依法扣押的，自扣押之日起，抵押权人有权收取该抵押财产的天然孳息或者法定孳息，但是抵押权人未通知应当清偿法定孳息义务人的除外。

前款规定的孳息应当先充抵收取孳息的费用。

法宝联想

司法案例　司法案例（353）

法学期刊　法学期刊（13）

法宝律师　律所实务（4）

专题参考　专题参考（2）

第四百一十三条

抵押财产折价或者拍卖、变卖后，其价款超过债权数额的部分归抵押人所有，不足部分由债务人清偿。

法宝联想

司法案例　司法案例（76443）案例报道（1）

法学期刊　法学期刊（11）

法宝律师　律所实务（1）

专题参考　专题参考（1）

第四百一十四条

同一财产向两个以上债权人抵押的，拍卖、变卖抵押财产所得的价款依照下列规定清偿：

（一）抵押权已经登记的，按照登记的时间先后确定清偿顺序；

（二）抵押权已经登记的先于未登记的受偿；

（三）抵押权未登记的，按照债权比例清偿。

其他可以登记的担保物权，清偿顺序参照适用前款规定。

法宝联想

法律法规　司法解释（2）

司法案例　司法案例（3828）案例报道（1）

法学期刊　法学期刊（174）

法宝律师　律所实务（4）

专题参考　专题参考（19）

第四百一十五条

同一财产既设立抵押权又设立质权的，拍卖、变卖该财产所得的价款按照登记、交付的时间先后确定清偿顺序。

法宝联想

法律法规　规范性文件（1）

司法案例　司法案例（129）

法学期刊　法学期刊（77）

法宝律师　律所实务（3）

专题参考　专题参考（6）

第四百一十六条

动产抵押担保的主债权是抵押物的价款，标的物交付后十日内办理抵押登记的，该抵押权人优先于抵押物买受人的其他担保物权人受偿，但是留置权人除外。

法宝联想

法律法规　规范性文件（1）

司法案例　司法案例（127）

法学期刊　法学期刊（100）

法宝律师　律所实务（6）

专题参考　专题参考（10）

第四百一十七条

建设用地使用权抵押后，该土地上新增的建筑物不属于抵押财产。该建设用地使用权实现抵押权时，应当将该土地上新增的建筑物与建设用地使用权一并处分。但是，新增建筑物所得的价款，抵押权人无权优先受偿。

法宝联想

法律法规　规范性文件（1）

司法案例　司法案例（197）

法学期刊　法学期刊（20）

法宝律师　律所实务（3）

专题参考　专题参考（7）

第四百一十八条

以集体所有土地的使用权依法抵押的，实现抵押权后，未经法定程序，不得改变土地所有权的性质和土地用途。

法宝联想

司法案例　司法案例（23）

法学期刊　法学期刊（12）

专题参考　专题参考（2）

第四百一十九条

抵押权人应当在主债权诉讼时效期间行使抵押权；未行使的，人民法院不予保护。

法宝联想

司法案例　司法案例（3038）

法学期刊　法学期刊（29）

法宝律师　律所实务（3）

专题参考　专题参考（14）

第二节　最高额抵押权

第四百二十条

为担保债务的履行，债务人或者第三人对一定期间内将要连续发生的债权提供担保财产的，债务人不履行到期债务或者发生当事人约定的实现抵押权的情形，抵押权人有权在最高债权额限度内就该担保财产优先受偿。

最高额抵押权设立前已经存在的债权，经当事人同意，可以转入最高额抵押担保的债权范围。

法宝联想

司法案例　司法案例（27334）

法学期刊　法学期刊（18）

法宝律师　律所实务（3）

专题参考　专题参考（4）

第四百二十一条

最高额抵押担保的债权确定前，部分债权转让的，最高额抵押权不得转让，但是当事人另有约定的除外。

法宝联想

司法案例　司法案例（66）

法学期刊　法学期刊（8）

法宝律师　律所实务（1）

专题参考　专题参考（2）

第四百二十二条

最高额抵押担保的债权确定前，抵押权人与抵押人可以通过协议变更债权确定的期间、债权范围以及最高债权额。但是，变更的内容不得对其他抵押权人产生不利影响。

法宝联想

法律法规　规范性文件（1）

司法案例　司法案例（89）

法学期刊　法学期刊（5）

法宝律师　律所实务（3）

专题参考　专题参考（1）

第四百二十三条　有下列情形之一的，抵押权人的债权确定：

（一）约定的债权确定期间届满；

（二）没有约定债权确定期间或者约定不明确，抵押权人或者抵押人自最高额抵押权设立之日起满二年后请求确定债权；

（三）新的债权不可能发生；

（四）抵押权人知道或者应当知道抵押财产被查封、扣押；

（五）债务人、抵押人被宣告破产或者解散；

（六）法律规定债权确定的其他情形。

法宝联想

法律法规　司法解释（1）

司法案例　司法案例（2390）

法学期刊　法学期刊（12）

法宝律师　律所实务（4）

专题参考　专题参考（8）

第四百二十四条

最高额抵押权除适用本节规定外，适用本章第一节的有关规定。

法宝联想

司法案例　司法案例（1064）

法学期刊　法学期刊（7）

法宝律师　律所实务（1）

专题参考　专题参考（1）

第十八章　质权

第一节　动产质权

第四百二十五条

为担保债务的履行，债务人或者第三人将其动产出质给债权人占有的，债务人不履行到期债务或者发生当事人约定的实现质权的情形，债权人有权就该动产优先受偿。

前款规定的债务人或者第三人为出质人，债权人为质权人，交付的动产为质押财产。

法宝联想

司法案例　司法案例（5317）

法学期刊　法学期刊（19）

法宝律师　律所实务（1）

第四百二十六条　法律、行政法规禁止转让的动产不得出质。

法宝联想

法律法规　法规解读（1）法律动态（1）

司法案例　司法案例（46）

法学期刊　法学期刊（12）

专题参考　专题参考（1）

第四百二十七条　设立质权，当事人应当采用书面形式订立质押合同。

质押合同一般包括下列条款：

（一）被担保债权的种类和数额；

（二）债务人履行债务的期限；

（三）质押财产的名称、数量等情况；

（四）担保的范围；

（五）质押财产交付的时间、方式。

法宝联想

司法案例　司法案例（1605）

法学期刊　法学期刊（34）

法宝律师　律所实务（6）

专题参考　专题参考（2）

第四百二十八条

质权人在债务履行期限届满前，与出质人约定债务人不履行到期债务时质押财产归债权人所有的，只能依法就质押财产优先受偿。

法宝联想

法律法规　司法解释（1）法规解读（2）法律动态（1）

司法案例　司法案例（531）案例报道（1）仲裁案例（1）

法学期刊　法学期刊（67）

法宝律师　律所实务（3）

专题参考　专题参考（4）

第四百二十九条　质权自出质人交付质押财产时设立。

法宝联想

司法案例　司法案例（1422）仲裁案例（1）

法学期刊　法学期刊（18）

法宝律师　律所实务（3）

专题参考　专题参考（2）

第四百三十条

质权人有权收取质押财产的孳息，但是合同另有约定的除外。

前款规定的孳息应当先充抵收取孳息的费用。

法宝联想

司法案例　司法案例（109）

法学期刊　法学期刊（9）

法宝律师　律所实务（1）

专题参考　专题参考（1）

第四百三十一条

质权人在质权存续期间，未经出质人同意，擅自使用、处分质押财产，造成出质人损害的，应当承担赔偿责任。

法宝联想

司法案例　司法案例（334）

法学期刊　法学期刊（7）

第四百三十二条

质权人负有妥善保管质押财产的义务；因保管不善致使质押财产毁损、灭失的，应当承担赔偿责任。

质权人的行为可能使质押财产毁损、灭失的，出质人可以请求质权人将质押财产提存，或者请求提前清偿债务并返还质押财产。

法宝联想

司法案例　司法案例（255）

法学期刊　法学期刊（15）

法宝律师　律所实务（2）

第四百三十三条

因不可归责于质权人的事由可能使质押财产毁损或者价值明显减少，足以危害质权人权利的，质权人有权请求出质人提供相应的担保；出质人不提供的，质权人可以拍卖、变卖质押财产，并与出质人协议将拍卖、变卖所得的价款提前清偿债务或者提存。

法宝联想

司法案例　司法案例（22）

法学期刊　法学期刊（10）

法宝律师　律所实务（2）

专题参考　专题参考（1）

第四百三十四条

质权人在质权存续期间，未经出质人同意转质，造成质押财产毁损、灭失的，应当承担赔偿责任。

法宝联想

司法案例 司法案例（54）

法学期刊 法学期刊（3）

专题参考 专题参考（1）

第四百三十五条

质权人可以放弃质权。债务人以自己的财产出质，质权人放弃该质权的，其他担保人在质权人丧失优先受偿权益的范围内免除担保责任，但是其他担保人承诺仍然提供担保的除外。

法宝联想

司法案例 司法案例（76）

法学期刊 法学期刊（9）

第四百三十六条

债务人履行债务或者出质人提前清偿所担保的债权的，质权人应当返还质押财产。

债务人不履行到期债务或者发生当事人约定的实现质权的情形，质权人可以与出质人协议以质押财产折价，也可以就拍卖、变卖质押财产所得的价款优先受偿。

质押财产折价或者变卖的，应当参照市场价格。

法宝联想

司法案例 司法案例（1998）

法学期刊 法学期刊（31）

法宝律师 律所实务（3）

专题参考 专题参考（1）

第四百三十七条

出质人可以请求质权人在债务履行期限届满后及时行使质权；质权人不行使的，出质人可以请求人民法院拍卖、变卖质押财产。

出质人请求质权人及时行使质权，因质权人怠于行使权利造成出质人损害的，由质权人承担赔偿责任。

法宝联想

司法案例 司法案例（212）

法学期刊 法学期刊（14）

法宝律师 律所实务（2）

专题参考 专题参考（1）

第四百三十八条

质押财产折价或者拍卖、变卖后，其价款超过债权数额的部分归出质人所有，不足部分由债务人清偿。

法宝联想

司法案例 司法案例（584）

法学期刊 法学期刊（6）

法宝律师 律所实务（1）

专题参考 专题参考（1）

第四百三十九条 出质人与质权人可以协议设立最高额质权。

最高额质权除适用本节有关规定外，参照适用本编第十七章第二节的有关规定。

法宝联想

司法案例 司法案例（312）

法学期刊　法学期刊（10）

专题参考　专题参考（2）

第二节　权利质权

第四百四十条　债务人或者第三人有权处分的下列权利可以出质：

（一）汇票、本票、支票；

（二）债券、存款单；

（三）仓单、提单；

（四）可以转让的基金份额、股权；

（五）可以转让的注册商标专用权、专利权、著作权等知识产权中的财产权；

（六）现有的以及将有的应收账款；

（七）法律、行政法规规定可以出质的其他财产权利。

法宝联想

法律法规　规范性文件（2）

司法案例　司法案例（1852）

法学期刊　法学期刊（91）

法宝律师　律所实务（8）

专题参考　专题参考（17）

第四百四十一条

以汇票、本票、支票、债券、存款单、仓单、提单出质的，质权自权利凭证交付质权人时设立；没有权利凭证的，质权自办理出质登记时设立。法律另有规定的，依照其规定。

法宝联想

司法案例　司法案例（534）

法学期刊　法学期刊（24）

法宝律师　律所实务（1）

专题参考　专题参考（5）

第四百四十二条

汇票、本票、支票、债券、存款单、仓单、提单的兑现日期或者提货日期先于主债权到期的，质权人可以兑现或者提货，并与出质人协议将兑现的价款或者提取的货物提前清偿债务或者提存。

法宝联想

司法案例　司法案例（111）

法学期刊　法学期刊（5）

法宝律师　律所实务（2）

第四百四十三条　以基金份额、股权出质的，质权自办理出质登记时设立。

基金份额、股权出质后，不得转让，但是出质人与质权人协商同意的除外。出质人转让基金份额、股权所得的价款，应当向质权人提前清偿债务或者提存。

法宝联想

法律法规　规范性文件（2）

司法案例　司法案例（1896）

法学期刊　法学期刊（36）

法宝律师　律所实务（7）

专题参考　专题参考（6）

第四百四十四条

以注册商标专用权、专利权、著作权等知识产权中的财产权出质的，质权自办理出质登记时设立。

知识产权中的财产权出质后，出质人不得转让或者许可他人使用，但是出质人与质权人协商同意的除外。出质人转让或者许可他人使用出质的知识产权中的财产权所得的价款，应当向质权人提前清偿债务或者提存。

法宝联想

法律法规　草案（1）

司法案例　司法案例（127）

法学期刊　法学期刊（23）

法宝律师　律所实务（2）

专题参考　专题参考（2）

第四百四十五条　以应收账款出质的，质权自办理出质登记时设立。

应收账款出质后，不得转让，但是出质人与质权人协商同意的除外。出质人转让应收账款所得的价款，应当向质权人提前清偿债务或者提存。

法宝联想

司法案例　司法案例（1434）

法学期刊　法学期刊（43）

法宝律师　律所实务（5）

专题参考　专题参考（2）

第四百四十六条

权利质权除适用本节规定外，适用本章第一节的有关规定。

法宝联想

法律法规 法规解读（1）

司法案例 司法案例（509）

法学期刊 法学期刊（23）

法宝律师 律所实务（1）

专题参考 专题参考（1）

第十九章　留置权

第四百四十七条

债务人不履行到期债务，债权人可以留置已经合法占有的债务人的动产，并有权就该动产优先受偿。

前款规定的债权人为留置权人，占有的动产为留置财产。

法宝联想

司法案例 司法案例（1340）

法学期刊 法学期刊（19）

法宝律师 律所实务（4）

专题参考 专题参考（2）

第四百四十八条

债权人留置的动产，应当与债权属于同一法律关系，但是企业之间留置的除外。

法宝联想

司法案例 司法案例（425）案例报道（1）

法学期刊 法学期刊（22）

法宝律师 律所实务（3）

专题参考 专题参考（2）

第四百四十九条　法律规定或者当事人约定不得留置的动产，不得留置。

法宝联想

司法案例　司法案例（49）

法学期刊　法学期刊（5）

法宝律师　律所实务（1）

第四百五十条

留置财产为可分物的，留置财产的价值应当相当于债务的金额。

法宝联想

法律法规　司法解释（1）

司法案例　司法案例（288）

法学期刊　法学期刊（4）

第四百五十一条

留置权人负有妥善保管留置财产的义务；因保管不善致使留置财产毁损、灭失的，应当承担赔偿责任。

法宝联想

司法案例　司法案例（108）

法学期刊　法学期刊（3）

第四百五十二条　留置权人有权收取留置财产的孳息。

前款规定的孳息应当先充抵收取孳息的费用。

法宝联想

司法案例　司法案例（17）

法学期刊　法学期刊（1）

专题参考　专题参考（1）

第四百五十三条

留置权人与债务人应当约定留置财产后的债务履行期限；没有约定或者约定不明确的，留置权人应当给债务人六十日以上履行债务的期限，但是鲜活易腐等不易保管的动产除外。债务人逾期未履行的，留置权人可以与债务人协议以留置财产折价，也可以就拍卖、变卖留置财产所得的价款优先受偿。

留置财产折价或者变卖的，应当参照市场价格。

法宝联想

司法案例 司法案例（237）

法学期刊 法学期刊（14）

法宝律师 律所实务（1）

专题参考 专题参考（1）

第四百五十四条

债务人可以请求留置权人在债务履行期限届满后行使留置权；留置权人不行使的，债务人可以请求人民法院拍卖、变卖留置财产。

法宝联想

司法案例 司法案例（30）

法学期刊 法学期刊（8）

专题参考 专题参考（1）

第四百五十五条

留置财产折价或者拍卖、变卖后，其价款超过债权数额的部分归债务人所有，不足部分由债务人清偿。

法宝联想

司法案例 司法案例（84）

法学期刊 法学期刊（6）

第四百五十六条

同一动产上已经设立抵押权或者质权，该动产又被留置的，留置权人优先受偿。

法宝联想

司法案例 司法案例（1294）

法学期刊 法学期刊（31）

专题参考 专题参考（1）

第四百五十七条

留置权人对留置财产丧失占有或者留置权人接受债务人另行提供担保的，留置权消灭。

法宝联想

司法案例 司法案例（56）

法学期刊 法学期刊（4）

专题参考 专题参考（2）

第五分编 占有

第二十章 占有

第四百五十八条

基于合同关系等产生的占有，有关不动产或者动产的使用、收益、违约责任等，按照合同约定；合同没有约定或者约定不明确的，依照有关法律规定。

法宝联想

司法案例 司法案例（1796）

法学期刊 法学期刊（12）

专题参考 专题参考（1）

第四百五十九条

占有人因使用占有的不动产或者动产，致使该不动产或者动产受到损害的，恶意占有人应当承担赔偿责任。

法宝联想

司法案例 司法案例（391）

法学期刊 法学期刊（11）

专题参考 专题参考（1）

第四百六十条

不动产或者动产被占有人占有的，权利人可以请求返还原物及其孳息；但是，应当支付善意占有人因维护该不动产或者动产支出的必要费用。

法宝联想

司法案例 司法案例（2838）案例报道（1）

法学期刊 法学期刊（29）

专题参考 专题参考（2）

第四百六十一条

占有的不动产或者动产毁损、灭失，该不动产或者动产的权利人请求赔偿的，占有人应当将因毁损、灭失取得的保险金、赔偿金或者补偿金等返还给权利人；权利人的损害未得到足够弥补的，恶意占有人还应当赔偿损失。

法宝联想

司法案例 司法案例（283）

法学期刊 法学期刊（10）

专题参考 专题参考（1）

第四百六十二条

占有的不动产或者动产被侵占的，占有人有权请求返还原物；对妨害占有的行为，占有人有权请求排除妨害或者消除危险；因侵占或者妨害造成损害的，占有人有权依法请求损害赔偿。

占有人返还原物的请求权，自侵占发生之日起一年内未行使的，该请求权消灭。

法宝联想
司法案例　司法案例（3880）
法学期刊　法学期刊（50）
法宝律师　律所实务（1）
专题参考　专题参考（1）

第三编　合同

第一分编　通则

第一章　一般规定

第四百六十三条　本编调整因合同产生的民事关系。

法宝联想
司法案例　司法案例（940）
法学期刊　法学期刊（11）

第四百六十四条

合同是民事主体之间设立、变更、终止民事法律关系的协议。

婚姻、收养、监护等有关身份关系的协议，适用有关该身份关系的法律规定；没有规定的，可以根据其性质参照适用本编规定。

法宝联想

法律法规　规范性文件（1）

司法案例　司法案例（13422）案例报道（1）仲裁案例（3）

法学期刊　法学期刊（139）

法宝律师　律所实务（8）

专题参考　专题参考（11）

第四百六十五条　依法成立的合同，受法律保护。

依法成立的合同，仅对当事人具有法律约束力，但是法律另有规定的除外。

法宝联想

法律法规　规范性文件（4）法律动态（2）

司法案例　司法案例（909669）案例报道（11）仲裁案例（55）

法学期刊　法学期刊（75）

法宝律师　律所实务（13）

专题参考　专题参考（12）

第四百六十六条

当事人对合同条款的理解有争议的，应当依据本法第一百四十二条第一款的规定，确定争议条款的含义。

合同文本采用两种以上文字订立并约定具有同等效力的，对各文本使用的词句推定具有相同含义。各文本使用的词句不一致的，应当根据合同的相关条款、性质、目的以及诚信原则等予以解释。

法宝联想

法律法规　司法解释（1）规范性文件（3）法律动态（3）

司法案例　司法案例（5781）案例报道（4）仲裁案例（6）

法学期刊　法学期刊（63）

法宝律师　律所实务（5）

专题参考　专题参考（14）

第四百六十七条

本法或者其他法律没有明文规定的合同，适用本编通则的规定，并可以参照适用本编或者其他法律最相类似合同的规定。

在中华人民共和国境内履行的中外合资经营企业合同、中外合作经营企业合同、中外合作勘探开发自然资源合同，适用中华人民共和国法律。

法宝联想

法律法规　司法解释（2）

司法案例　司法案例（5118）仲裁案例（1）

法学期刊　法学期刊（103）

法宝律师　律所实务（3）

专题参考　专题参考（8）

第四百六十八条

非因合同产生的债权债务关系，适用有关该债权债务关系的法律规定；没有规定的，适用本编通则的有关规定，但是根据其性质不能适用的除外。

法宝联想

司法案例　司法案例（459）

法学期刊　法学期刊（61）

法宝律师　律所实务（3）

专题参考　专题参考（2）

第二章　合同的订立

第四百六十九条

当事人订立合同，可以采用书面形式、口头形式或者其他形式。

书面形式是合同书、信件、电报、电传、传真等可以有形地表现所载内容的形式。

以电子数据交换、电子邮件等方式能够有形地表现所载内容，并可以随时调取查用的数据电文，视为书面形式。

法宝联想

法律法规　规范性文件（2）法律动态（1）

司法案例　司法案例（93063）案例报道（6）仲裁案例（1）

法学期刊　法学期刊（73）

法宝律师　律所实务（8）

专题参考　专题参考（16）

第四百七十条　合同的内容由当事人约定，一般包括下列条款：

（一）当事人的姓名或者名称和住所；

（二）标的；

（三）数量；

（四）质量；

（五）价款或者报酬；

（六）履行期限、地点和方式；

（七）违约责任；

（八）解决争议的方法。

当事人可以参照各类合同的示范文本订立合同。

法宝联想

法律法规 部门规章（2）地方政府规章（2）规范性文件（4）

司法案例 司法案例（3273）仲裁案例（1）

法学期刊 法学期刊（41）

法宝律师 律所实务（6）

专题参考 专题参考（6）

第四百七十一条

当事人订立合同，可以采取要约、承诺方式或者其他方式。

法宝联想

法律法规 规范性文件（1）

司法案例 司法案例（4643）案例报道（1）

法学期刊 法学期刊（18）

法宝律师 律所实务（3）

专题参考 专题参考（4）

第四百七十二条

要约是希望与他人订立合同的意思表示，该意思表示应当符合下列条件：

（一）内容具体确定；

（二）表明经受要约人承诺，要约人即受该意思表示约束。

法宝联想

司法案例 司法案例（2516）案例报道（1）

法学期刊 法学期刊（36）

法宝律师　律所实务（2）

专题参考　专题参考（3）

第四百七十三条

要约邀请是希望他人向自己发出要约的表示。拍卖公告、招标公告、招股说明书、债券募集办法、基金招募说明书、商业广告和宣传、寄送的价目表等为要约邀请。

商业广告和宣传的内容符合要约条件的，构成要约。

法宝联想

法律法规　规范性文件（1）

司法案例　司法案例（1349）案例报道（2）

法学期刊　法学期刊（19）

法宝律师　律所实务（3）

专题参考　专题参考（3）

第四百七十四条　要约生效的时间适用本法第一百三十七条的规定。

法宝联想

司法案例　司法案例（195）

法学期刊　法学期刊（3）

第四百七十五条

要约可以撤回。要约的撤回适用本法第一百四十一条的规定。

法宝联想

法律法规　规范性文件（1）

司法案例　司法案例（87）

法学期刊　法学期刊（5）

第四百七十六条　要约可以撤销，但是有下列情形之一的除外：

（一）要约人以确定承诺期限或者其他形式明示要约不可撤销；

（二）受要约人有理由认为要约是不可撤销的，并已经为履行合同做了合理准备工作。

法宝联想

司法案例　司法案例（108）

法学期刊　法学期刊（15）

第四百七十七条

撤销要约的意思表示以对话方式作出的，该意思表示的内容应当在受要约人作出承诺之前为受要约人所知道；撤销要约的意思表示以非对话方式作出的，应当在受要约人作出承诺之前到达受要约人。

法宝联想

法律法规　规范性文件（2）

司法案例　司法案例（196）

法学期刊　法学期刊（5）

第四百七十八条　有下列情形之一的，要约失效：

（一）要约被拒绝；

（二）要约被依法撤销；

（三）承诺期限届满，受要约人未作出承诺；

（四）受要约人对要约的内容作出实质性变更。

法宝联想

司法案例　司法案例（564）

法学期刊 法学期刊（10）

法宝律师 律所实务（1）

专题参考 专题参考（1）

第四百七十九条 承诺是受要约人同意要约的意思表示。

法宝联想

法律法规 规范性文件（1）

司法案例 司法案例（1172）

法学期刊 法学期刊（10）

法宝律师 律所实务（1）

专题参考 专题参考（1）

第四百八十条

承诺应当以通知的方式作出；但是，根据交易习惯或者要约表明可以通过行为作出承诺的除外。

法宝联想

司法案例 司法案例（749）仲裁案例（1）

法学期刊 法学期刊（18）

专题参考 专题参考（3）

第四百八十一条 承诺应当在要约确定的期限内到达要约人。

要约没有确定承诺期限的，承诺应当依照下列规定到达：

（一）要约以对话方式作出的，应当即时作出承诺；

（二）要约以非对话方式作出的，承诺应当在合理期限内到达。

法宝联想

司法案例 司法案例（182）

法学期刊 法学期刊（8）

第四百八十二条

要约以信件或者电报作出的，承诺期限自信件载明的日期或者电报交发之日开始计算。信件未载明日期的，自投寄该信件的邮戳日期开始计算。要约以电话、传真、电子邮件等快速通讯方式作出的，承诺期限自要约到达受要约人时开始计算。

法宝联想

司法案例　司法案例（44）

法学期刊　法学期刊（5）

第四百八十三条

承诺生效时合同成立，但是法律另有规定或者当事人另有约定的除外。

法宝联想

司法案例　司法案例（3069）仲裁案例（1）

法学期刊　法学期刊（20）

专题参考　专题参考（4）

第四百八十四条

以通知方式作出的承诺，生效的时间适用本法第一百三十七条的规定。

承诺不需要通知的，根据交易习惯或者要约的要求作出承诺的行为时生效。

法宝联想

司法案例　司法案例（425）

法学期刊　法学期刊（8）

法宝律师　律所实务（1）

专题参考　专题参考（2）

第四百八十五条

承诺可以撤回。承诺的撤回适用本法第一百四十一条的规定。

法宝联想

司法案例　司法案例（116）

法学期刊　法学期刊（4）

第四百八十六条

受要约人超过承诺期限发出承诺，或者在承诺期限内发出承诺，按照通常情形不能及时到达要约人的，为新要约；但是，要约人及时通知受要约人该承诺有效的除外。

法宝联想

司法案例　司法案例（38）仲裁案例（1）

法学期刊　法学期刊（2）

第四百八十七条

受要约人在承诺期限内发出承诺，按照通常情形能够及时到达要约人，但是因其他原因致使承诺到达要约人时超过承诺期限的，除要约人及时通知受要约人因承诺超过期限不接受该承诺外，该承诺有效。

法宝联想

法律法规　规范性文件（1）

司法案例　司法案例（14）

法学期刊　法学期刊（5）

第四百八十八条

承诺的内容应当与要约的内容一致。受要约人对要约的内容作出实质性变更的，

为新要约。有关合同标的、数量、质量、价款或者报酬、履行期限、履行地点和方式、违约责任和解决争议方法等的变更，是对要约内容的实质性变更。

法宝联想

法律法规　规范性文件（1）

司法案例　司法案例（583）仲裁案例（1）

法学期刊　法学期刊（13）

法宝律师　律所实务（4）

专题参考　专题参考（1）

第四百八十九条

承诺对要约的内容作出非实质性变更的，除要约人及时表示反对或者要约表明承诺不得对要约的内容作出任何变更外，该承诺有效，合同的内容以承诺的内容为准。

法宝联想

司法案例　司法案例（103）

法学期刊　法学期刊（7）

专题参考　专题参考（1）

第四百九十条

当事人采用合同书形式订立合同的，自当事人均签名、盖章或者按指印时合同成立。在签名、盖章或者按指印之前，当事人一方已经履行主要义务，对方接受时，该合同成立。

法律、行政法规规定或者当事人约定合同应当采用书面形式订立，当事人未采用书面形式但是一方已经履行主要义务，对方接受时，该合同成立。

法宝联想

法律法规　司法解释（2）规范性文件（4）

司法案例　司法案例（30460）案例报道（3）仲裁案例（8）

法学期刊　法学期刊（75）

法宝律师　律所实务（10）

专题参考　专题参考（17）

第四百九十一条

当事人采用信件、数据电文等形式订立合同要求签订确认书的，签订确认书时合同成立。

当事人一方通过互联网等信息网络发布的商品或者服务信息符合要约条件的，对方选择该商品或者服务并提交订单成功时合同成立，但是当事人另有约定的除外。

法宝联想

法律法规　规范性文件（2）

司法案例　司法案例（11134）案例报道（3）

法学期刊　法学期刊（24）

专题参考　专题参考（4）

第四百九十二条　承诺生效的地点为合同成立的地点。

采用数据电文形式订立合同的，收件人的主营业地为合同成立的地点；没有主营业地的，其住所地为合同成立的地点。当事人另有约定的，按照其约定。

法宝联想

司法案例　司法案例（255）

法学期刊　法学期刊（3）

专题参考　专题参考（2）

第四百九十三条

当事人采用合同书形式订立合同的，最后签名、盖章或者按指印的地点为合同成立的地点，但是当事人另有约定的除外。

法宝联想

法律法规　规范性文件（1）

司法案例　司法案例（855）

法学期刊　法学期刊（8）

法宝律师　律所实务（2）

专题参考　专题参考（3）

第四百九十四条

国家根据抢险救灾、疫情防控或者其他需要下达国家订货任务、指令性任务的，有关民事主体之间应当依照有关法律、行政法规规定的权利和义务订立合同。

依照法律、行政法规的规定负有发出要约义务的当事人，应当及时发出合理的要约。

依照法律、行政法规的规定负有作出承诺义务的当事人，不得拒绝对方合理的订立合同要求。

法宝联想

司法案例　司法案例（46）

法学期刊　法学期刊（46）

专题参考　专题参考（3）

第四百九十五条

当事人约定在将来一定期限内订立合同的认购书、订购书、预订书等，构成预约合同。

当事人一方不履行预约合同约定的订立合同义务的，对方可以请求其承担预约合同的违约责任。

法宝联想

法律法规　规范性文件（2）法规解读（1）法律动态（4）

司法案例　司法案例（6057）案例报道（5）仲裁案例（1）

法学期刊　法学期刊（54）

法宝律师　律所实务（13）

专题参考　专题参考（3）

第四百九十六条

格式条款是当事人为了重复使用而预先拟定，并在订立合同时未与对方协商的条款。

采用格式条款订立合同的，提供格式条款的一方应当遵循公平原则确定当事人之间的权利和义务，并采取合理的方式提示对方注意免除或者减轻其责任等与对方有重大利害关系的条款，按照对方的要求，对该条款予以说明。提供格式条款的一方未履行提示或者说明义务，致使对方没有注意或者理解与其有重大利害关系的条款的，对方可以主张该条款不成为合同的内容。

法宝联想

法律法规　司法解释（2）规范性文件（20）法规解读（1）法律动态（7）

司法案例　司法案例（33806）案例报道（30）仲裁案例（6）

法学期刊　法学期刊（161）

法宝律师　律所实务（25）

专题参考　专题参考（22）

第四百九十七条　有下列情形之一的，该格式条款无效：

（一）具有本法第一编第六章第三节和本法第五百零六条规定的无效情形；

（二）提供格式条款一方不合理地免除或者减轻其责任、加重对方责任、限制对方主要权利；

（三）提供格式条款一方排除对方主要权利。

法宝联想

法律法规　司法解释（2）规范性文件（11）法规解读（1）法律动态（2）

司法案例　司法案例（23367）案例报道（19）仲裁案例（4）

法学期刊　法学期刊（153）

法宝律师　律所实务（13）

专题参考　专题参考（9）

第四百九十八条

对格式条款的理解发生争议的，应当按照通常理解予以解释。对格式条款有两种以上解释的，应当作出不利于提供格式条款一方的解释。格式条款和非格式条款不一致的，应当采用非格式条款。

法宝联想

法律法规　规范性文件（5）法律动态（3）

司法案例　司法案例（9182）案例报道（8）

法学期刊　法学期刊（61）

法宝律师　律所实务（4）

专题参考　专题参考（6）

第四百九十九条

悬赏人以公开方式声明对完成特定行为的人支付报酬的，完成该行为的人可以请求其支付。

法宝联想

法律法规　规范性文件（1）

司法案例　司法案例（92）案例报道（1）

法学期刊　法学期刊（16）

专题参考　专题参考（5）

第五百条

当事人在订立合同过程中有下列情形之一，造成对方损失的，应当承担赔偿责任：

（一）假借订立合同，恶意进行磋商；

（二）故意隐瞒与订立合同有关的重要事实或者提供虚假情况；

（三）有其他违背诚信原则的行为。

法宝联想

法律法规　司法解释（2）规范性文件（1）

司法案例　司法案例（4863）案例报道（5）仲裁案例（1）

法学期刊　法学期刊（116）

法宝律师　律所实务（6）

专题参考　专题参考（12）

第五百零一条

当事人在订立合同过程中知悉的商业秘密或者其他应当保密的信息，无论合同是否成立，不得泄露或者不正当地使用；泄露、不正当地使用该商业秘密或者信息，造成对方损失的，应当承担赔偿责任。

法宝联想

法律法规 规范性文件（1）

司法案例 司法案例（201）

法学期刊 法学期刊（22）

法宝律师 律所实务（4）

专题参考 专题参考（3）

第三章　合同的效力

第五百零二条

依法成立的合同，自成立时生效，但是法律另有规定或者当事人另有约定的除外。

依照法律、行政法规的规定，合同应当办理批准等手续的，依照其规定。未办理批准等手续影响合同生效的，不影响合同中履行报批等义务条款以及相关条款的效力。应当办理申请批准等手续的当事人未履行义务的，对方可以请求其承担违反该义务的责任。

依照法律、行政法规的规定，合同的变更、转让、解除等情形应当办理批准等手续的，适用前款规定。

法宝联想

法律法规 司法解释（1）规范性文件（1）法律动态（1）

司法案例 司法案例（392985）案例报道（6）仲裁案例（28）

法学期刊 法学期刊（115）

法宝律师 律所实务（16）

专题参考 专题参考（15）

第五百零三条

无权代理人以被代理人的名义订立合同，被代理人已经开始履行合同义务或者接受相对人履行的，视为对合同的追认。

法宝联想

司法案例　司法案例（1946）仲裁案例（3）

法学期刊　法学期刊（15）

专题参考　专题参考（2）

第五百零四条

法人的法定代表人或者非法人组织的负责人超越权限订立的合同，除相对人知道或者应当知道其超越权限外，该代表行为有效，订立的合同对法人或者非法人组织发生效力。

法宝联想

法律法规　司法解释（2）规范性文件（1）法规解读（3）法律动态（3）

司法案例　司法案例（2298）案例报道（1）仲裁案例（4）

法学期刊　法学期刊（109）

法宝律师　律所实务（18）

专题参考　专题参考（24）

第五百零五条

当事人超越经营范围订立的合同的效力，应当依照本法第一编第六章第三节和本编的有关规定确定，不得仅以超越经营范围确认合同无效。

法宝联想

司法案例　司法案例（1239）仲裁案例（1）

法学期刊　法学期刊（19）

法宝律师　律所实务（3）

专题参考　专题参考（7）

第五百零六条　合同中的下列免责条款无效：

（一）造成对方人身损害的；

（二）因故意或者重大过失造成对方财产损失的。

法宝联想

法律法规　规范性文件（4）法规解读（1）

司法案例　司法案例（2620）案例报道（5）仲裁案例（4）

法学期刊　法学期刊（51）

法宝律师　律所实务（9）

专题参考　专题参考（3）

第五百零七条

合同不生效、无效、被撤销或者终止的，不影响合同中有关解决争议方法的条款的效力。

法宝联想

司法案例　司法案例（4198）仲裁案例（3）

法学期刊　法学期刊（15）

法宝律师　律所实务（3）

专题参考　专题参考（2）

第五百零八条

本编对合同的效力没有规定的，适用本法第一编第六章的有关规定。

法宝联想

司法案例　司法案例（2096）

法学期刊　法学期刊（18）

法宝律师　律所实务（3）

专题参考　专题参考（4）

第四章　合同的履行

第五百零九条　当事人应当按照约定全面履行自己的义务。

当事人应当遵循诚信原则，根据合同的性质、目的和交易习惯履行通知、协助、保密等义务。

当事人在履行合同过程中，应当避免浪费资源、污染环境和破坏生态。

法宝联想

法律法规　规范性文件（16）法律动态（10）

司法案例　司法案例（3041672）案例报道（46）仲裁案例（76）

法学期刊　法学期刊（173）

法宝律师　律所实务（38）

专题参考　专题参考（25）

第五百一十条

合同生效后，当事人就质量、价款或者报酬、履行地点等内容没有约定或者约定不明确的，可以协议补充；不能达成补充协议的，按照合同相关条款或者交易习惯确定。

法宝联想

法律法规　司法解释（5）规范性文件（5）法律动态（2）

司法案例　司法案例（45265）案例报道（5）仲裁案例（9）

法学期刊　法学期刊（86）

法宝律师　律所实务（31）

专题参考　专题参考（22）

第五百一十一条

当事人就有关合同内容约定不明确，依据前条规定仍不能确定的，适用下列规定：

（一）质量要求不明确的，按照强制性国家标准履行；没有强制性国家标准的，按照推荐性国家标准履行；没有推荐性国家标准的，按照行业标准履行；没有国家标准、行业标准的，按照通常标准或者符合合同目的的特定标准履行。

（二）价款或者报酬不明确的，按照订立合同时履行地的市场价格履行；依法应当执行政府定价或者政府指导价的，依照规定履行。

（三）履行地点不明确，给付货币的，在接受货币一方所在地履行；交付不动产的，在不动产所在地履行；其他标的，在履行义务一方所在地履行。

（四）履行期限不明确的，债务人可以随时履行，债权人也可以随时请求履行，但是应当给对方必要的准备时间。

（五）履行方式不明确的，按照有利于实现合同目的的方式履行。

（六）履行费用的负担不明确的，由履行义务一方负担；因债权人原因增加的履行费用，由债权人负担。

法宝联想
法律法规　司法解释（4）规范性文件（8）法律动态（2）
司法案例　司法案例（55868）案例报道（12）仲裁案例（4）
法学期刊　法学期刊（98）

法宝律师　律所实务（28）

专题参考　专题参考（22）

第五百一十二条

通过互联网等信息网络订立的电子合同的标的为交付商品并采用快递物流方式交付的，收货人的签收时间为交付时间。电子合同的标的为提供服务的，生成的电子凭证或者实物凭证中载明的时间为提供服务时间；前述凭证没有载明时间或者载明时间与实际提供服务时间不一致的，以实际提供服务的时间为准。

电子合同的标的物为采用在线传输方式交付的，合同标的物进入对方当事人指定的特定系统且能够检索识别的时间为交付时间。

电子合同当事人对交付商品或者提供服务的方式、时间另有约定的，按照其约定。

法宝联想

法律法规　规范性文件（3）法律动态（2）

司法案例　司法案例（1919）案例报道（2）

法学期刊　法学期刊（22）

专题参考　专题参考（3）

第五百一十三条

执行政府定价或者政府指导价的，在合同约定的交付期限内政府价格调整时，按照交付时的价格计价。逾期交付标的物的，遇价格上涨时，按照原价格执行；价格下降时，按照新价格执行。逾期提取标的物或者逾期付款的，遇价格上涨时，按照新价格执行；价格下降时，按照原价格执行。

法宝联想

司法案例 司法案例（266）

法学期刊 法学期刊（6）

专题参考 专题参考（1）

第五百一十四条

以支付金钱为内容的债，除法律另有规定或者当事人另有约定外，债权人可以请求债务人以实际履行地的法定货币履行。

法宝联想

司法案例 司法案例（978）

法学期刊 法学期刊（14）

专题参考 专题参考（2）

第五百一十五条

标的有多项而债务人只需履行其中一项的，债务人享有选择权；但是，法律另有规定、当事人另有约定或者另有交易习惯的除外。

享有选择权的当事人在约定期限内或者履行期限届满未作选择，经催告后在合理期限内仍未选择的，选择权转移至对方。

法宝联想

司法案例 司法案例（288）

法学期刊 法学期刊（31）

法宝律师 律所实务（1）

专题参考 专题参考（4）

第五百一十六条

当事人行使选择权应当及时通知对方，通知到达对方时，标的确定。标的确定后不得变更，但是经对方同意的除外。

可选择的标的发生不能履行情形的，享有选择权的当事人不得选择不能履行的标的，但是该不能履行的情形是由对方造成的除外。

法宝联想

司法案例　司法案例（89）

法学期刊　法学期刊（13）

第五百一十七条

债权人为二人以上，标的可分，按照份额各自享有债权的，为按份债权；债务人为二人以上，标的可分，按照份额各自负担债务的，为按份债务。

按份债权人或者按份债务人的份额难以确定的，视为份额相同。

法宝联想

司法案例　司法案例（527）仲裁案例（1）

法学期刊　法学期刊（17）

法宝律师　律所实务（1）

专题参考　专题参考（2）

第五百一十八条

债权人为二人以上，部分或者全部债权人均可以请求债务人履行债务的，为连带债权；债务人为二人以上，债权人可以请求部分或者全部债务人履行全部债务的，为连带债务。

连带债权或者连带债务，由法律规定或者当事人约定。

法宝联想

司法案例　司法案例（9086）案例报道（1）仲裁案例（4）

法学期刊　法学期刊（68）

法宝律师　律所实务（6）

专题参考　专题参考（7）

第五百一十九条　连带债务人之间的份额难以确定的，视为份额相同。

实际承担债务超过自己份额的连带债务人，有权就超出部分在其他连带债务人未履行的份额范围内向其追偿，并相应地享有债权人的权利，但是不得损害债权人的利益。其他连带债务人对债权人的抗辩，可以向该债务人主张。

被追偿的连带债务人不能履行其应分担份额的，其他连带债务人应当在相应范围内按比例分担。

法宝联想

司法案例　司法案例（3283）案例报道（1）仲裁案例（2）

法学期刊　法学期刊（79）

法宝律师　律所实务（5）

专题参考　专题参考（9）

第五百二十条

部分连带债务人履行、抵销债务或者提存标的物的，其他债务人对债权人的债务在相应范围内消灭；该债务人可以依据前条规定向其他债务人追偿。

部分连带债务人的债务被债权人免除的，在该连带债务人应当承担的份额范围内，其他债务人对债权人的债务消灭。

部分连带债务人的债务与债权人的债权同归于一人的，在扣除该债务人应当承担的份额后，债权人对其他债务人的债权继续存在。

债权人对部分连带债务人的给付受领迟延的，对其他连带债务人发生效力。

法宝联想

司法案例　司法案例（905）案例报道（1）仲裁案例（1）

法学期刊　法学期刊（51）

法宝律师　律所实务（3）

专题参考　专题参考（1）

第五百二十一条　连带债权人之间的份额难以确定的，视为份额相同。

实际受领债权的连带债权人，应当按比例向其他连带债权人返还。

连带债权参照适用本章连带债务的有关规定。

法宝联想

司法案例　司法案例（248）仲裁案例（1）

法学期刊　法学期刊（9）

法宝律师　律所实务（1）

专题参考　专题参考（3）

第五百二十二条

当事人约定由债务人向第三人履行债务，债务人未向第三人履行债务或者履行债务不符合约定的，应当向债权人承担违约责任。

法律规定或者当事人约定第三人可以直接请求债务人向其履行债务，第三人未在合理期限内明确拒绝，债务人未向第三人履行债务或者履行债务不符合约定的，

第三人可以请求债务人承担违约责任；债务人对债权人的抗辩，可以向第三人主张。

法宝联想

法律法规　司法解释（2）规范性文件（1）法律动态（3）

司法案例　司法案例（1848）仲裁案例（2）

法学期刊　法学期刊（85）

法宝律师　律所实务（9）

专题参考　专题参考（5）

第五百二十三条

当事人约定由第三人向债权人履行债务，第三人不履行债务或者履行债务不符合约定的，债务人应当向债权人承担违约责任。

法宝联想

司法案例　司法案例（3281）案例报道（1）仲裁案例（3）

法学期刊　法学期刊（25）

法宝律师　律所实务（3）

专题参考　专题参考（7）

第五百二十四条

债务人不履行债务，第三人对履行该债务具有合法利益的，第三人有权向债权人代为履行；但是，根据债务性质、按照当事人约定或者依照法律规定只能由债务人履行的除外。

债权人接受第三人履行后，其对债务人的债权转让给第三人，但是债务人和第三人另有约定的除外。

法宝联想

法律法规　司法解释（1）规范性文件（2）法律动态（2）

司法案例　司法案例（4530）案例报道（2）仲裁案例（1）

法学期刊　法学期刊（104）

法宝律师　律所实务（3）

专题参考　专题参考（14）

第五百二十五条

当事人互负债务，没有先后履行顺序的，应当同时履行。一方在对方履行之前有权拒绝其履行请求。一方在对方履行债务不符合约定时，有权拒绝其相应的履行请求。

法宝联想

法律法规　司法解释（1）规范性文件（1）法律动态（1）

司法案例　司法案例（3645）仲裁案例（2）

法学期刊　法学期刊（44）

法宝律师　律所实务（1）

专题参考　专题参考（7）

第五百二十六条

当事人互负债务，有先后履行顺序，应当先履行债务一方未履行的，后履行一方有权拒绝其履行请求。先履行一方履行债务不符合约定的，后履行一方有权拒绝其相应的履行请求。

法宝联想

法律法规　司法解释（1）规范性文件（2）法律动态（2）

司法案例　司法案例（9743）案例报道（3）

法学期刊　法学期刊（22）

法宝律师　律所实务（9）

专题参考　专题参考（10）

第五百二十七条

应当先履行债务的当事人，有确切证据证明对方有下列情形之一的，可以中止履行：

（一）经营状况严重恶化；

（二）转移财产、抽逃资金，以逃避债务；

（三）丧失商业信誉；

（四）有丧失或者可能丧失履行债务能力的其他情形。

当事人没有确切证据中止履行的，应当承担违约责任。

法宝联想
法律法规　司法解释（2）规范性文件（1）
司法案例　司法案例（4408）仲裁案例（2）
法学期刊　法学期刊（37）
法宝律师　律所实务（5）
专题参考　专题参考（6）

第五百二十八条

当事人依据前条规定中止履行的，应当及时通知对方。对方提供适当担保的，应当恢复履行。中止履行后，对方在合理期限内未恢复履行能力且未提供适当担保的，视为以自己的行为表明不履行主要债务，中止履行的一方可以解除合同并可以请求对方承担违约责任。

法宝联想
法律法规　规范性文件（1）
司法案例　司法案例（1326）仲裁案例（2）
法学期刊　法学期刊（28）
法宝律师　律所实务（3）
专题参考　专题参考（6）

第五百二十九条

债权人分立、合并或者变更住所没有通知债务人，致使履行债务发生困难的，债务人可以中止履行或者将标的物提存。

法宝联想

司法案例 司法案例（77）

法学期刊 法学期刊（5）

法宝律师 律所实务（1）

第五百三十条

债权人可以拒绝债务人提前履行债务，但是提前履行不损害债权人利益的除外。

债务人提前履行债务给债权人增加的费用，由债务人负担。

法宝联想

司法案例 司法案例（140）

法学期刊 法学期刊（9）

专题参考 专题参考（2）

第五百三十一条

债权人可以拒绝债务人部分履行债务，但是部分履行不损害债权人利益的除外。

债务人部分履行债务给债权人增加的费用，由债务人负担。

法宝联想

司法案例 司法案例（81）

法学期刊 法学期刊（17）

第五百三十二条

合同生效后，当事人不得因姓名、名称的变更或者法定代表人、负责人、承办人的变动而不履行合同义务。

法宝联想

法律法规　规范性文件（1）法律动态（1）

司法案例　司法案例（1915）案例报道（2）

法学期刊　法学期刊（3）

第五百三十三条

合同成立后，合同的基础条件发生了当事人在订立合同时无法预见的、不属于商业风险的重大变化，继续履行合同对于当事人一方明显不公平的，受不利影响的当事人可以与对方重新协商；在合理期限内协商不成的，当事人可以请求人民法院或者仲裁机构变更或者解除合同。

人民法院或者仲裁机构应当结合案件的实际情况，根据公平原则变更或者解除合同。

法宝联想

法律法规　司法解释（1）规范性文件（14）法规解读（2）法律动态（6）

司法案例　司法案例（10593）案例报道（8）仲裁案例（10）

法学期刊　法学期刊（188）

法宝律师　律所实务（33）

专题参考　专题参考（25）

第五百三十四条

对当事人利用合同实施危害国家利益、社会公共利益行为的，市场监督管理和其他有关行政主管部门依照法律、行政法规的规定负责监督处理。

法宝联想

法律法规　草案（1）

司法案例　司法案例（127）

法学期刊　法学期刊（28）

第五章　合同的保全

第五百三十五条

因债务人怠于行使其债权或者与该债权有关的从权利，影响债权人的到期债权实现的，债权人可以向人民法院请求以自己的名义代位行使债务人对相对人的权利，但是该权利专属于债务人自身的除外。

代位权的行使范围以债权人的到期债权为限。债权人行使代位权的必要费用，由债务人负担。

相对人对债务人的抗辩，可以向债权人主张。

法宝联想

法律法规　司法解释（2）规范性文件（3）法律动态（1）

司法案例　司法案例（7761）案例报道（3）

法学期刊　法学期刊（128）

法宝律师　律所实务（39）

专题参考　专题参考（15）

第五百三十六条

债权人的债权到期前，债务人的债权或者与该债权有关的从权利存在诉讼时效期间即将届满或者未及时申报破产债权等情形，影响债权人的债权实现的，债权人可以代位向债务人的相对人请求其向债务人履行、向破产管理人申报或者作出其他必要的行为。

法宝联想

法律法规　规范性文件（1）

司法案例　司法案例（333）案例报道（1）

法学期刊　法学期刊（20）

法宝律师 律所实务（3）

专题参考 专题参考（5）

第五百三十七条

人民法院认定代位权成立的，由债务人的相对人向债权人履行义务，债权人接受履行后，债权人与债务人、债务人与相对人之间相应的权利义务终止。债务人对相对人的债权或者与该债权有关的从权利被采取保全、执行措施，或者债务人破产的，依照相关法律的规定处理。

法宝联想

法律法规 规范性文件（2）法律动态（1）

司法案例 司法案例（1269）案例报道（2）

法学期刊 法学期刊（68）

法宝律师 律所实务（20）

专题参考 专题参考（5）

第五百三十八条

债务人以放弃其债权、放弃债权担保、无偿转让财产等方式无偿处分财产权益，或者恶意延长其到期债权的履行期限，影响债权人的债权实现的，债权人可以请求人民法院撤销债务人的行为。

法宝联想

法律法规 司法解释（4）规范性文件（6）法律动态（4）

司法案例 司法案例（6188）案例报道（9）

法学期刊 法学期刊（108）

法宝律师 律所实务（27）

专题参考 专题参考（15）

第五百三十九条

债务人以明显不合理的低价转让财产、以明显不合理的高价受让他人财产或者为他人的债务提供担保，影响债权人的债权实现，债务人的相对人知道或者应当知道该情形的，债权人可以请求人民法院撤销债务人的行为。

法宝联想

法律法规　司法解释（5）规范性文件（2）法规解读（1）法律动态（5）

司法案例　司法案例（3320）案例报道（4）

法学期刊　法学期刊（110）

法宝律师　律所实务（18）

专题参考　专题参考（12）

第五百四十条

撤销权的行使范围以债权人的债权为限。债权人行使撤销权的必要费用，由债务人负担。

法宝联想

法律法规　司法解释（1）

司法案例　司法案例（2634）案例报道（1）

法学期刊　法学期刊（25）

法宝律师　律所实务（4）

专题参考　专题参考（2）

第五百四十一条

撤销权自债权人知道或者应当知道撤销事由之日起一年内行使。自债务人的行为发生之日起五年内没有行使撤销权的，该撤销权消灭。

法宝联想

司法案例　司法案例（2832）案例报道（3）

法学期刊　法学期刊（18）

法宝律师　律所实务（6）

专题参考　专题参考（5）

第五百四十二条

债务人影响债权人的债权实现的行为被撤销的，自始没有法律约束力。

法宝联想

法律法规　规范性文件（1）

司法案例　司法案例（1205）

法学期刊　法学期刊（28）

法宝律师　律所实务（3）

专题参考　专题参考（2）

第六章　合同的变更和转让

第五百四十三条　当事人协商一致，可以变更合同。

法宝联想

法律法规　法律动态（1）

司法案例　司法案例（15567）案例报道（4）仲裁案例（5）

法学期刊　法学期刊（34）

法宝律师　律所实务（7）

专题参考　专题参考（2）

第五百四十四条　当事人对合同变更的内容约定不明确的，推定为未变更。

法宝联想

法律法规　规范性文件（1）

司法案例　司法案例（1715）案例报道（1）仲裁案例（2）

法学期刊　法学期刊（10）

法宝律师　律所实务（3）

专题参考　专题参考（3）

第五百四十五条

债权人可以将债权的全部或者部分转让给第三人，但是有下列情形之一的除外：

（一）根据债权性质不得转让；

（二）按照当事人约定不得转让；

（三）依照法律规定不得转让。

当事人约定非金钱债权不得转让的，不得对抗善意第三人。当事人约定金钱债权不得转让的，不得对抗第三人。

法宝联想

法律法规　规范性文件（2）

司法案例　司法案例（151047）案例报道（5）仲裁案例（4）

法学期刊　法学期刊（77）

法宝律师　律所实务（18）

专题参考　专题参考（12）

第五百四十六条

债权人转让债权，未通知债务人的，该转让对债务人不发生效力。

债权转让的通知不得撤销，但是经受让人同意的除外。

法宝联想

司法案例　司法案例（115626）案例报道（1）仲裁案例（4）

法学期刊　法学期刊（104）

法宝律师　律所实务（14）

专题参考　专题参考（13）

第五百四十七条

债权人转让债权的，受让人取得与债权有关的从权利，但是该从权利专属于债权人自身的除外。

受让人取得从权利不因该从权利未办理转移登记手续或者未转移占有而受到影响。

法宝联想

法律法规　规范性文件（1）

司法案例　司法案例（61389）案例报道（1）仲裁案例（1）

法学期刊　法学期刊（56）

法宝律师　律所实务（9）

专题参考　专题参考（6）

第五百四十八条

债务人接到债权转让通知后，债务人对让与人的抗辩，可以向受让人主张。

法宝联想

司法案例　司法案例（4532）案例报道（1）仲裁案例（1）

法学期刊　法学期刊（37）

法宝律师　律所实务（3）

专题参考　专题参考（2）

第五百四十九条　有下列情形之一的，债务人可以向受让人主张抵销：

（一）债务人接到债权转让通知时，债务人对让与人享有债权，且债务人的债权先于转让的债权到期或者同时到期；

（二）债务人的债权与转让的债权是基于同一合同产生。

法宝联想

司法案例　司法案例（525）

法学期刊　法学期刊（42）

法宝律师　律所实务（2）

专题参考　专题参考（5）

第五百五十条　因债权转让增加的履行费用，由让与人负担。

法宝联想

司法案例　司法案例（316）

法学期刊　法学期刊（19）

专题参考　专题参考（5）

第五百五十一条

债务人将债务的全部或者部分转移给第三人的，应当经债权人同意。

债务人或者第三人可以催告债权人在合理期限内予以同意，债权人未作表示的，视为不同意。

法宝联想

法律法规　规范性文件（5）

司法案例　司法案例（9999）案例报道（2）仲裁案例（2）

法学期刊　法学期刊（54）

法宝律师　律所实务（9）

专题参考　专题参考（4）

第五百五十二条

第三人与债务人约定加入债务并通知债权人，或者第三人向债权人表示愿意加入

债务，债权人未在合理期限内明确拒绝的，债权人可以请求第三人在其愿意承担的债务范围内和债务人承担连带债务。

法宝联想

法律法规 司法解释（1）规范性文件（4）法规解读（1）法律动态（3）

司法案例 司法案例（46207）案例报道（8）仲裁案例（12）

法学期刊 法学期刊（65）

法宝律师 律所实务（15）

专题参考 专题参考（16）

第五百五十三条

债务人转移债务的，新债务人可以主张原债务人对债权人的抗辩；原债务人对债权人享有债权的，新债务人不得向债权人主张抵销。

法宝联想

司法案例 司法案例（477）

法学期刊 法学期刊（13）

法宝律师 律所实务（1）

专题参考 专题参考（2）

第五百五十四条

债务人转移债务的，新债务人应当承担与主债务有关的从债务，但是该从债务专属于原债务人自身的除外。

法宝联想

法律法规 规范性文件（1）

司法案例 司法案例（700）

法学期刊 法学期刊（3）

法宝律师 律所实务（1）

第五百五十五条

当事人一方经对方同意，可以将自己在合同中的权利和义务一并转让给第三人。

法宝联想

法律法规　规范性文件（2）合同范本（1）

司法案例　司法案例（12834）案例报道（1）仲裁案例（3）

法学期刊　法学期刊（19）

法宝律师　律所实务（1）

第五百五十六条

合同的权利和义务一并转让的，适用债权转让、债务转移的有关规定。

法宝联想

司法案例　司法案例（9909）

法学期刊　法学期刊（11）

专题参考　专题参考（2）

第七章　合同的权利义务终止

第五百五十七条　有下列情形之一的，债权债务终止：

（一）债务已经履行；

（二）债务相互抵销；

（三）债务人依法将标的物提存；

（四）债权人免除债务；

（五）债权债务同归于一人；

（六）法律规定或者当事人约定终止的其他情形。

合同解除的，该合同的权利义务关系终止。

法宝联想

法律法规　规范性文件（2）

司法案例　司法案例（16332）案例报道（1）仲裁案例（1）

法学期刊　法学期刊（55）

法宝律师　律所实务（10）

专题参考　专题参考（4）

第五百五十八条

债权债务终止后，当事人应当遵循诚信等原则，根据交易习惯履行通知、协助、保密、旧物回收等义务。

法宝联想

法律法规　规范性文件（3）

司法案例　司法案例（3247）案例报道（3）

法学期刊　法学期刊（52）

法宝律师　律所实务（1）

专题参考　专题参考（5）

第五百五十九条

债权债务终止时，债权的从权利同时消灭，但是法律另有规定或者当事人另有约定的除外。

法宝联想

司法案例　司法案例（769）

法学期刊　法学期刊（3）

专题参考　专题参考（1）

第五百六十条

债务人对同一债权人负担的数项债务种类相同，债务人的给付不足以清偿全部债务的，除当事人另有约定外，由债务人在清偿时指定其履行的债务。

债务人未作指定的，应当优先履行已经到期的债务；数项债务均到期的，优先履行对债权人缺乏担保或者担保最少的债务；均无担保或者担保相等的，优先履行债务人负担较重的债务；负担相同的，按照债务到期的先后顺序履行；到期时间相同的，按照债务比例履行。

法宝联想

法律法规　司法解释（1）法律动态（1）

司法案例　司法案例（8836）案例报道（1）

法学期刊　法学期刊（19）

法宝律师　律所实务（4）

专题参考　专题参考（3）

第五百六十一条

债务人在履行主债务外还应当支付利息和实现债权的有关费用，其给付不足以清偿全部债务的，除当事人另有约定外，应当按照下列顺序履行：

（一）实现债权的有关费用；

（二）利息；

（三）主债务。

法宝联想

法律法规　司法解释（1）规范性文件（4）法律动态（2）

司法案例　司法案例（37957）案例报道（4）仲裁案例（5）

法学期刊　法学期刊（19）

法宝律师　律所实务（8）

专题参考　专题参考（6）

第五百六十二条　当事人协商一致，可以解除合同。

当事人可以约定一方解除合同的事由。解除合同的事由发生时，解除权人可以解除合同。

法宝联想

法律法规 规范性文件（4）法律动态（1）

司法案例 司法案例（146251）案例报道（7）仲裁案例（25）

法学期刊 法学期刊（70）

法宝律师 律所实务（12）

专题参考 专题参考（9）

第五百六十三条 有下列情形之一的，当事人可以解除合同：

（一）因不可抗力致使不能实现合同目的；

（二）在履行期限届满前，当事人一方明确表示或者以自己的行为表明不履行主要债务；

（三）当事人一方迟延履行主要债务，经催告后在合理期限内仍未履行；

（四）当事人一方迟延履行债务或者有其他违约行为致使不能实现合同目的；

（五）法律规定的其他情形。

以持续履行的债务为内容的不定期合同，当事人可以随时解除合同，但是应当在合理期限之前通知对方。

法宝联想

法律法规 司法解释（7）规范性文件（10）法律动态（2）

司法案例 司法案例（234726）案例报道（27）仲裁案例（57）

法学期刊　法学期刊（279）

法宝律师　律所实务（47）

专题参考　专题参考（24）

第五百六十四条

法律规定或者当事人约定解除权行使期限，期限届满当事人不行使的，该权利消灭。

法律没有规定或者当事人没有约定解除权行使期限，自解除权人知道或者应当知道解除事由之日起一年内不行使，或者经对方催告后在合理期限内不行使的，该权利消灭。

法宝联想

法律法规　司法解释（1）

司法案例　司法案例（10377）案例报道（2）仲裁案例（6）

法学期刊　法学期刊（39）

法宝律师　律所实务（14）

专题参考　专题参考（8）

第五百六十五条

当事人一方依法主张解除合同的，应当通知对方。合同自通知到达对方时解除；通知载明债务人在一定期限内不履行债务则合同自动解除，债务人在该期限内未履行债务的，合同自通知载明的期限届满时解除。对方对解除合同有异议的，任何一方当事人均可以请求人民法院或者仲裁机构确认解除行为的效力。

当事人一方未通知对方，直接以提起诉讼或者申请仲裁的方式依法主张解除合同

，人民法院或者仲裁机构确认该主张的，合同自起诉状副本或者仲裁申请书副本送达对方时解除。

法宝联想

法律法规 司法解释（1）规范性文件（4）法律动态（1）

司法案例 司法案例（152552）案例报道（20）仲裁案例（43）

法学期刊 法学期刊（77）

法宝律师 律所实务（6）

专题参考 专题参考（11）

第五百六十六条

合同解除后，尚未履行的，终止履行；已经履行的，根据履行情况和合同性质，当事人可以请求恢复原状或者采取其他补救措施，并有权请求赔偿损失。

合同因违约解除的，解除权人可以请求违约方承担违约责任，但是当事人另有约定的除外。

主合同解除后，担保人对债务人应当承担的民事责任仍应当承担担保责任，但是担保合同另有约定的除外。

法宝联想

法律法规 司法解释（2）规范性文件（5）法律动态（2）

司法案例 司法案例（270351）案例报道（20）仲裁案例（34）

法学期刊 法学期刊（162）

法宝律师 律所实务（27）

专题参考 专题参考（15）

第五百六十七条

合同的权利义务关系终止，不影响合同中结算和清理条款的效力。

法宝联想

法律法规　司法解释（1）法律动态（1）

司法案例　司法案例（20268）案例报道（1）仲裁案例（4）

法学期刊　法学期刊（17）

法宝律师　律所实务（3）

专题参考　专题参考（4）

第五百六十八条

当事人互负债务，该债务的标的物种类、品质相同的，任何一方可以将自己的债务与对方的到期债务抵销；但是，根据债务性质、按照当事人约定或者依照法律规定不得抵销的除外。

当事人主张抵销的，应当通知对方。通知自到达对方时生效。抵销不得附条件或者附期限。

法宝联想

法律法规　司法解释（1）规范性文件（1）法律动态（1）

司法案例　司法案例（8042）案例报道（1）仲裁案例（3）

法学期刊　法学期刊（51）

法宝律师　律所实务（6）

专题参考　专题参考（6）

第五百六十九条

当事人互负债务，标的物种类、品质不相同的，经协商一致，也可以抵销。

法宝联想

法律法规　规范性文件（1）

司法案例　司法案例（1335）案例报道（1）仲裁案例（2）

法学期刊　法学期刊（14）

法宝律师　律所实务（3）

专题参考　专题参考（1）

第五百七十条

有下列情形之一，难以履行债务的，债务人可以将标的物提存：

（一）债权人无正当理由拒绝受领；

（二）债权人下落不明；

（三）债权人死亡未确定继承人、遗产管理人，或者丧失民事行为能力未确定监护人；

（四）法律规定的其他情形。

标的物不适于提存或者提存费用过高的，债务人依法可以拍卖或者变卖标的物，提存所得的价款。

法宝联想

法律法规　司法解释（2）规范性文件（1）

司法案例　司法案例（1476）

法学期刊　法学期刊（26）

法宝律师　律所实务（1）

专题参考　专题参考（1）

第五百七十一条

债务人将标的物或者将标的物依法拍卖、变卖所得价款交付提存部门时，提存成立。

提存成立的，视为债务人在其提存范围内已经交付标的物。

法宝联想

司法案例　司法案例（115）

法学期刊　法学期刊（14）

法宝律师　律所实务（1）

专题参考　专题参考（1）

第五百七十二条

标的物提存后，债务人应当及时通知债权人或者债权人的继承人、遗产管理人、监护人、财产代管人。

法宝联想

司法案例　司法案例（73）

法宝律师　律所实务（1）

第五百七十三条

标的物提存后，毁损、灭失的风险由债权人承担。提存期间，标的物的孳息归债权人所有。提存费用由债权人负担。

法宝联想

司法案例　司法案例（48）

法学期刊　法学期刊（6）

法宝律师　律所实务（1）

第五百七十四条

债权人可以随时领取提存物。但是，债权人对债务人负有到期债务的，在债权人未履行债务或者提供担保之前，提存部门根据债务人的要求应当拒绝其领取提存物。

债权人领取提存物的权利，自提存之日起五年内不行使而消灭，提存物扣除提存费用后归国家所有。但是，债权人未履行对债务人的到期债务，或者债权人向提存部门书面表示放弃领取提存物权利的，债务人负担提存费用后有权取回提存物。

法宝联想

司法案例　司法案例（327）

法学期刊　法学期刊（13）

法宝律师　律所实务（2）

第五百七十五条

债权人免除债务人部分或者全部债务的，债权债务部分或者全部终止，但是债务人在合理期限内拒绝的除外。

法宝联想

司法案例　司法案例（1341）

法学期刊　法学期刊（23）

专题参考　专题参考（2）

第五百七十六条

债权和债务同归于一人的，债权债务终止，但是损害第三人利益的除外。

法宝联想

司法案例　司法案例（838）

法学期刊　法学期刊（3）

第八章　违约责任

第五百七十七条

当事人一方不履行合同义务或者履行合同义务不符合约定的，应当承担继续履行、采取补救措施或者赔偿损失等违约责任。

法宝联想

法律法规　司法解释（1）规范性文件（9）法规解读（1）法律动态（8）

司法案例　司法案例（2906054）案例报道（35）仲裁案例（60）

法学期刊　法学期刊（196）

法宝律师　律所实务（36）

专题参考　专题参考（22）

第五百七十八条

当事人一方明确表示或者以自己的行为表明不履行合同义务的，对方可以在履行期限届满前请求其承担违约责任。

法宝联想

法律法规　司法解释（2）规范性文件（2）法规解读（1）

司法案例　司法案例（77856）案例报道（5）仲裁案例（6）

法学期刊　法学期刊（27）

法宝律师　律所实务（6）

专题参考　专题参考（7）

第五百七十九条

当事人一方未支付价款、报酬、租金、利息，或者不履行其他金钱债务的，对方可以请求其支付。

法宝联想

法律法规　规范性文件（2）法律动态（1）

司法案例　司法案例（983818）案例报道（13）仲裁案例（10）

法学期刊　法学期刊（34）

法宝律师　律所实务（3）

专题参考　专题参考（9）

第五百八十条

当事人一方不履行非金钱债务或者履行非金钱债务不符合约定的，对方可以请求履行，但是有下列情形之一的除外：

（一）法律上或者事实上不能履行；

（二）债务的标的不适于强制履行或者履行费用过高；

（三）债权人在合理期限内未请求履行。

有前款规定的除外情形之一，致使不能实现合同目的的，人民法院或者仲裁机构可以根据当事人的请求终止合同权利义务关系，但是不影响违约责任的承担。

法宝联想

法律法规　司法解释（2）规范性文件（7）法律动态（6）

司法案例　司法案例（21177）案例报道（16）仲裁案例（13）

法学期刊　法学期刊（221）

法宝律师　律所实务（24）

专题参考　专题参考（21）

第五百八十一条

当事人一方不履行债务或者履行债务不符合约定，根据债务的性质不得强制履行的，对方可以请求其负担由第三人替代履行的费用。

法宝联想

法律法规 规范性文件（2）

司法案例 司法案例（796）案例报道（1）

法学期刊 法学期刊（29）

法宝律师 律所实务（1）

专题参考 专题参考（1）

第五百八十二条

履行不符合约定的，应当按照当事人的约定承担违约责任。对违约责任没有约定或者约定不明确，依据本法第五百一十条的规定仍不能确定的，受损害方根据标的的性质以及损失的大小，可以合理选择请求对方承担修理、重作、更换、退货、减少价款或者报酬等违约责任。

法宝联想

法律法规 司法解释（2）规范性文件（2）

司法案例 司法案例（38221）案例报道（6）仲裁案例（5）

法学期刊 法学期刊（57）

法宝律师 律所实务（5）

专题参考 专题参考（10）

第五百八十三条

当事人一方不履行合同义务或者履行合同义务不符合约定的，在履行义务或者采取补救措施后，对方还有其他损失的，应当赔偿损失。

法宝联想

法律法规　规范性文件（4）

司法案例　司法案例（79123）案例报道（5）仲裁案例（8）

法学期刊　法学期刊（37）

法宝律师　律所实务（3）

专题参考　专题参考（5）

第五百八十四条

当事人一方不履行合同义务或者履行合同义务不符合约定，造成对方损失的，损失赔偿额应当相当于因违约所造成的损失，包括合同履行后可以获得的利益；但是，不得超过违约一方订立合同时预见到或者应当预见到的因违约可能造成的损失。

法宝联想

法律法规　司法解释（3）规范性文件（6）法律动态（2）合同范本（2）

司法案例　司法案例（163621）案例报道（12）仲裁案例（32）

法学期刊　法学期刊（190）

法宝律师　律所实务（29）

专题参考　专题参考（22）

第五百八十五条

当事人可以约定一方违约时应当根据违约情况向对方支付一定数额的违约金，也可以约定因违约产生的损失赔偿额的计算方法。

约定的违约金低于造成的损失的，人民法院或者仲裁机构可以根据当事人的请求予以增加；约定的违约金过分高于造成的损失的，人民法院或者仲裁机构可以根据当事人的请求予以适当减少。

当事人就迟延履行约定违约金的，违约方支付违约金后，还应当履行债务。

法宝联想

法律法规 司法解释（2）规范性文件（9）法律动态（4）

司法案例 司法案例（642665）案例报道（15）仲裁案例（53）

法学期刊 法学期刊（115）

法宝律师 律所实务（32）

专题参考 专题参考（20）

第五百八十六条

当事人可以约定一方向对方给付定金作为债权的担保。定金合同自实际交付定金时成立。

定金的数额由当事人约定；但是，不得超过主合同标的额的百分之二十，超过部分不产生定金的效力。实际交付的定金数额多于或者少于约定数额的，视为变更约定的定金数额。

法宝联想

司法案例 司法案例（15400）案例报道（4）仲裁案例（5）

法学期刊 法学期刊（32）

法宝律师 律所实务（2）

专题参考 专题参考（5）

第五百八十七条

债务人履行债务的，定金应当抵作价款或者收回。给付定金的一方不履行债务或者履行债务不符合约定，致使不能实现合同目的的，无权请求返还定金；收受定金的一方不履行债务或者履行债务不符合约定，致使不能实现合同目的的，应当双倍返还定金。

法宝联想

法律法规　司法解释（1）规范性文件（2）法律动态（3）

司法案例　司法案例（23083）案例报道（6）仲裁案例（4）

法学期刊　法学期刊（18）

法宝律师　律所实务（2）

专题参考　专题参考（3）

第五百八十八条

当事人既约定违约金，又约定定金的，一方违约时，对方可以选择适用违约金或者定金条款。

定金不足以弥补一方违约造成的损失的，对方可以请求赔偿超过定金数额的损失。

法宝联想

司法案例　司法案例（5699）案例报道（2）仲裁案例（2）

法学期刊　法学期刊（23）

法宝律师　律所实务（3）

专题参考　专题参考（2）

第五百八十九条

债务人按照约定履行债务，债权人无正当理由拒绝受领的，债务人可以请求债权人赔偿增加的费用。

在债权人受领迟延期间，债务人无须支付利息。

法宝联想

法律法规　规范性文件（1）

司法案例　司法案例（415）案例报道（1）

法学期刊　法学期刊（19）

法宝律师　律所实务（1）

专题参考 专题参考（3）

第五百九十条

当事人一方因不可抗力不能履行合同的，根据不可抗力的影响，部分或者全部免除责任，但是法律另有规定的除外。因不可抗力不能履行合同的，应当及时通知对方，以减轻可能给对方造成的损失，并应当在合理期限内提供证明。

当事人迟延履行后发生不可抗力的，不免除其违约责任。

法宝联想

法律法规 规范性文件（6）法规解读（1）法律动态（1）

司法案例 司法案例（27143）案例报道（4）仲裁案例（7）

法学期刊 法学期刊（65）

法宝律师 律所实务（13）

专题参考 专题参考（8）

第五百九十一条

当事人一方违约后，对方应当采取适当措施防止损失的扩大；没有采取适当措施致使损失扩大的，不得就扩大的损失请求赔偿。

当事人因防止损失扩大而支出的合理费用，由违约方负担。

法宝联想

法律法规 司法解释（2）规范性文件（5）法规解读（1）法律动态（2）

司法案例 司法案例（18428）案例报道（8）仲裁案例（6）

法学期刊 法学期刊（66）

法宝律师 律所实务（7）

专题参考 专题参考（9）

第五百九十二条 当事人都违反合同的，应当各自承担相应的责任。

当事人一方违约造成对方损失，对方对损失的发生有过错的，可以减少相应的损失赔偿额。

法宝联想

法律法规 司法解释（2）规范性文件（2）法规解读（1）法律动态（1）

司法案例 司法案例（15753）案例报道（5）仲裁案例（4）

法学期刊 法学期刊（63）

法宝律师 律所实务（1）

专题参考 专题参考（10）

第五百九十三条

当事人一方因第三人的原因造成违约的，应当依法向对方承担违约责任。当事人一方和第三人之间的纠纷，依照法律规定或者按照约定处理。

法宝联想

法律法规 规范性文件（1）法规解读（1）法律动态（1）

司法案例 司法案例（4710）案例报道（1）仲裁案例（1）

法学期刊 法学期刊（32）

法宝律师 律所实务（3）

专题参考 专题参考（3）

第五百九十四条

因国际货物买卖合同和技术进出口合同争议提起诉讼或者申请仲裁的时效期间为四年。

法宝联想

司法案例 司法案例（56）

法学期刊 法学期刊（5）

第二分编　典型合同

第九章　买卖合同

第五百九十五条

买卖合同是出卖人转移标的物的所有权于买受人，买受人支付价款的合同。

法宝联想

司法案例　司法案例（275103）案例报道（2）仲裁案例（3）

法学期刊　法学期刊（41）

法宝律师　律所实务（9）

专题参考　专题参考（1）

第五百九十六条

买卖合同的内容一般包括标的物的名称、数量、质量、价款、履行期限、履行地点和方式、包装方式、检验标准和方法、结算方式、合同使用的文字及其效力等条款。

法宝联想

司法案例　司法案例（4310）仲裁案例（1）

法学期刊　法学期刊（6）

法宝律师　律所实务（1）

第五百九十七条

因出卖人未取得处分权致使标的物所有权不能转移的，买受人可以解除合同并请求出卖人承担违约责任。

法律、行政法规禁止或者限制转让的标的物，依照其规定。

法宝联想

法律法规　规范性文件（1）

司法案例　司法案例（4034）

法学期刊　法学期刊（95）

法宝律师　律所实务（1）

专题参考　专题参考（16）

第五百九十八条

出卖人应当履行向买受人交付标的物或者交付提取标的物的单证，并转移标的物所有权的义务。

法宝联想

司法案例　司法案例（12855）案例报道（1）仲裁案例（1）

法学期刊　法学期刊（27）

专题参考　专题参考（4）

第五百九十九条

出卖人应当按照约定或者交易习惯向买受人交付提取标的物单证以外的有关单证和资料。

法宝联想

法律法规　司法解释（2）

司法案例　司法案例（2553）

法学期刊　法学期刊（5）

法宝律师　律所实务（3）

专题参考　专题参考（1）

第六百条

出卖具有知识产权的标的物的，除法律另有规定或者当事人另有约定外，该标的物的知识产权不属于买受人。

法宝联想

司法案例　司法案例（90）

法学期刊　法学期刊（4）

第六百零一条

出卖人应当按照约定的时间交付标的物。约定交付期限的，出卖人可以在该交付期限内的任何时间交付。

法宝联想

法律法规　规范性文件（1）法律动态（1）

司法案例　司法案例（6569）案例报道（1）

专题参考　专题参考（1）

第六百零二条

当事人没有约定标的物的交付期限或者约定不明确的，适用本法第五百一十条、第五百一十一条第四项的规定。

法宝联想

司法案例　司法案例（1084）

法学期刊　法学期刊（3）

专题参考　专题参考（2）

第六百零三条　出卖人应当按照约定的地点交付标的物。

当事人没有约定交付地点或者约定不明确，依据本法第五百一十条的规定仍不能确定的，适用下列规定：

（一）标的物需要运输的，出卖人应当将标的物交付给第一承运人以运交给买受人；

（二）标的物不需要运输，出卖人和买受人订立合同时知道标的物在某一地点的，出卖人应当在该地点交付标的物；不知道标的物在某一地点的，应当在出卖人订立合同时的营业地交付标的物。

法宝联想

法律法规　司法解释（2）

司法案例　司法案例（1023）

法学期刊　法学期刊（9）

专题参考　专题参考（3）

第六百零四条

标的物毁损、灭失的风险，在标的物交付之前由出卖人承担，交付之后由买受人承担，但是法律另有规定或者当事人另有约定的除外。

法宝联想

法律法规　规范性文件（1）法律动态（1）

司法案例　司法案例（1450）案例报道（1）

法学期刊　法学期刊（22）

法宝律师　律所实务（7）

专题参考　专题参考（4）

第六百零五条

因买受人的原因致使标的物未按照约定的期限交付的，买受人应当自违反约定时起承担标的物毁损、灭失的风险。

法宝联想

法律法规　规范性文件（1）

司法案例　司法案例（172）

法学期刊　法学期刊（9）

第六百零六条

出卖人出卖交由承运人运输的在途标的物，除当事人另有约定外，毁损、灭失的风险自合同成立时起由买受人承担。

法宝联想

司法案例　司法案例（142）

法学期刊　法学期刊（4）

专题参考　专题参考（1）

第六百零七条

出卖人按照约定将标的物运送至买受人指定地点并交付给承运人后，标的物毁损、灭失的风险由买受人承担。

当事人没有约定交付地点或者约定不明确，依据本法第六百零三条第二款第一项的规定标的物需要运输的，出卖人将标的物交付给第一承运人后，标的物毁损、灭失的风险由买受人承担。

法宝联想

法律法规　司法解释（2）

司法案例　司法案例（499）

法学期刊　法学期刊（4）

法宝律师　律所实务（1）

专题参考　专题参考（2）

第六百零八条

出卖人按照约定或者依据本法第六百零三条第二款第二项的规定将标的物置于交

付地点，买受人违反约定没有收取的，标的物毁损、灭失的风险自违反约定时起由买受人承担。

法宝联想

法律法规　法律动态（1）

司法案例　司法案例（200）

法学期刊　法学期刊（3）

第六百零九条

出卖人按照约定未交付有关标的物的单证和资料的，不影响标的物毁损、灭失风险的转移。

法宝联想

司法案例　司法案例（106）

法学期刊　法学期刊（1）

法宝律师　律所实务（1）

第六百一十条

因标的物不符合质量要求，致使不能实现合同目的的，买受人可以拒绝接受标的物或者解除合同。买受人拒绝接受标的物或者解除合同的，标的物毁损、灭失的风险由出卖人承担。

法宝联想

法律法规　规范性文件（1）法律动态（1）

司法案例　司法案例（3189）案例报道（1）仲裁案例（1）

法学期刊　法学期刊（33）

法宝律师　律所实务（1）

专题参考　专题参考（1）

第六百一十一条

标的物毁损、灭失的风险由买受人承担的，不影响因出卖人履行义务不符合约定，买受人请求其承担违约责任的权利。

法宝联想

司法案例　司法案例（136）

法学期刊　法学期刊（3）

第六百一十二条

出卖人就交付的标的物，负有保证第三人对该标的物不享有任何权利的义务，但是法律另有规定的除外。

法宝联想

司法案例　司法案例（1132）案例报道（1）

法学期刊　法学期刊（25）

专题参考　专题参考（4）

第六百一十三条

买受人订立合同时知道或者应当知道第三人对买卖的标的物享有权利的，出卖人不承担前条规定的义务。

法宝联想

司法案例　司法案例（539）案例报道（1）

法学期刊　法学期刊（15）

专题参考　专题参考（1）

第六百一十四条

买受人有确切证据证明第三人对标的物享有权利的，可以中止支付相应的价款，但是出卖人提供适当担保的除外。

法宝联想

法律法规　规范性文件（1）

司法案例　司法案例（68）

法学期刊　法学期刊（4）

专题参考　专题参考（1）

第六百一十五条

出卖人应当按照约定的质量要求交付标的物。出卖人提供有关标的物质量说明的，交付的标的物应当符合该说明的质量要求。

法宝联想

司法案例　司法案例（2826）仲裁案例（1）

法学期刊　法学期刊（19）

法宝律师　律所实务（2）

专题参考　专题参考（2）

第六百一十六条

当事人对标的物的质量要求没有约定或者约定不明确，依据本法第五百一十条的规定仍不能确定的，适用本法第五百一十一条第一项的规定。

法宝联想

司法案例　司法案例（886）仲裁案例（1）

法学期刊　法学期刊（12）

专题参考　专题参考（3）

第六百一十七条

出卖人交付的标的物不符合质量要求的，买受人可以依据本法第五百八十二条至第五百八十四条的规定请求承担违约责任。

法宝联想

法律法规　规范性文件（1）

司法案例　司法案例（3348）案例报道（1）

法学期刊　法学期刊（26）

法宝律师　律所实务（2）

专题参考　专题参考（3）

第六百一十八条

当事人约定减轻或者免除出卖人对标的物瑕疵承担的责任，因出卖人故意或者重大过失不告知买受人标的物瑕疵的，出卖人无权主张减轻或者免除责任。

法宝联想

司法案例　司法案例（170）

法学期刊　法学期刊（13）

法宝律师　律所实务（1）

专题参考　专题参考（4）

第六百一十九条

出卖人应当按照约定的包装方式交付标的物。对包装方式没有约定或者约定不明确，依据本法第五百一十条的规定仍不能确定的，应当按照通用的方式包装；没有通用方式的，应当采取足以保护标的物且有利于节约资源、保护生态环境的包装方式。

法宝联想

司法案例　司法案例（117）

法学期刊　法学期刊（19）

专题参考　专题参考（4）

第六百二十条

买受人收到标的物时应当在约定的检验期限内检验。没有约定检验期限的，应当及时检验。

法宝联想

司法案例　司法案例（3480）案例报道（2）

法学期刊　法学期刊（15）

法宝律师　律所实务（2）

第六百二十一条

当事人约定检验期限的，买受人应当在检验期限内将标的物的数量或者质量不符合约定的情形通知出卖人。买受人怠于通知的，视为标的物的数量或者质量符合约定。

当事人没有约定检验期限的，买受人应当在发现或者应当发现标的物的数量或者质量不符合约定的合理期限内通知出卖人。买受人在合理期限内未通知或者自收到标的物之日起二年内未通知出卖人的，视为标的物的数量或者质量符合约定；但是，对标的物有质量保证期的，适用质量保证期，不适用该二年的规定。

出卖人知道或者应当知道提供的标的物不符合约定的，买受人不受前两款规定的通知时间的限制。

法宝联想

法律法规　司法解释（2）

司法案例　司法案例（4911）仲裁案例（3）

法学期刊　法学期刊（32）

法宝律师　律所实务（5）

专题参考　专题参考（3）

第六百二十二条

当事人约定的检验期限过短，根据标的物的性质和交易习惯，买受人在检验期限内难以完成全面检验的，该期限仅视为买受人对标的物的外观瑕疵提出异议的期限。

约定的检验期限或者质量保证期短于法律、行政法规规定期限的，应当以法律、行政法规规定的期限为准。

法宝联想

法律法规　规范性文件（1）

司法案例　司法案例（614）案例报道（1）

法学期刊　法学期刊（13）

法宝律师　律所实务（1）

专题参考　专题参考（1）

第六百二十三条

当事人对检验期限未作约定，买受人签收的送货单、确认单等载明标的物数量、型号、规格的，推定买受人已经对数量和外观瑕疵进行检验，但是有相关证据足以推翻的除外。

法宝联想

司法案例　司法案例（726）仲裁案例（1）

法学期刊　法学期刊（8）

法宝律师　律所实务（1）

专题参考　专题参考（2）

第六百二十四条

出卖人依照买受人的指示向第三人交付标的物，出卖人和买受人约定的检验标准与买受人和第三人约定的检验标准不一致的，以出卖人和买受人约定的检验标准为准。

法宝联想

司法案例　司法案例（100）

法学期刊　法学期刊（6）

第六百二十五条

依照法律、行政法规的规定或者按照当事人的约定，标的物在有效使用年限届满后应予回收的，出卖人负有自行或者委托第三人对标的物予以回收的义务。

法宝联想

司法案例　司法案例（179）

法学期刊　法学期刊（18）

法宝律师　律所实务（1）

专题参考　专题参考（4）

第六百二十六条

买受人应当按照约定的数额和支付方式支付价款。对价款的数额和支付方式没有约定或者约定不明确的，适用本法第五百一十条、第五百一十一条第二项和第五项的规定。

法宝联想

司法案例　司法案例（253977）案例报道（2）仲裁案例（3）

法学期刊　法学期刊（5）

第六百二十七条

买受人应当按照约定的地点支付价款。对支付地点没有约定或者约定不明确，依据本法第五百一十条的规定仍不能确定的，买受人应当在出卖人的营业地支付；但是，约定支付价款以交付标的物或者交付提取标的物单证为条件的，在交付标的物或者交付提取标的物单证的所在地支付。

法宝联想

司法案例　司法案例（2494）

法学期刊　法学期刊（2）

第六百二十八条

买受人应当按照约定的时间支付价款。对支付时间没有约定或者约定不明确，依据本法第五百一十条的规定仍不能确定的，买受人应当在收到标的物或者提取标的物单证的同时支付。

法宝联想

司法案例　司法案例（230356）仲裁案例（1）

法学期刊　法学期刊（4）

专题参考　专题参考（2）

第六百二十九条

出卖人多交标的物的，买受人可以接收或者拒绝接收多交的部分。买受人接收多交部分的，按照约定的价格支付价款；买受人拒绝接收多交部分的，应当及时通知出卖人。

法宝联想

法律法规　司法解释（2）

司法案例　司法案例（327）

法学期刊　法学期刊（2）

专题参考　专题参考（1）

第六百三十条

标的物在交付之前产生的孳息，归出卖人所有；交付之后产生的孳息，归买受人所有。但是，当事人另有约定的除外。

法宝联想

司法案例　司法案例（136）

法学期刊　法学期刊（6）

专题参考　专题参考（1）

第六百三十一条

因标的物的主物不符合约定而解除合同的，解除合同的效力及于从物。因标的物的从物不符合约定被解除的，解除的效力不及于主物。

法宝联想

司法案例　司法案例（65）

法学期刊　法学期刊（1）

第六百三十二条

标的物为数物，其中一物不符合约定的，买受人可以就该物解除。但是，该物与他物分离使标的物的价值显受损害的，买受人可以就数物解除合同。

法宝联想

司法案例　司法案例（166）仲裁案例（2）

法学期刊　法学期刊（3）

第六百三十三条

出卖人分批交付标的物的，出卖人对其中一批标的物不交付或者交付不符合约定，致使该批标的物不能实现合同目的的，买受人可以就该批标的物解除。

出卖人不交付其中一批标的物或者交付不符合约定，致使之后其他各批标的物的交付不能实现合同目的的，买受人可以就该批以及之后其他各批标的物解除。

买受人如果就其中一批标的物解除，该批标的物与其他各批标的物相互依存的，可以就已经交付和未交付的各批标的物解除。

法宝联想

司法案例　司法案例（420）仲裁案例（1）

法学期刊　法学期刊（8）

第六百三十四条

分期付款的买受人未支付到期价款的数额达到全部价款的五分之一，经催告后在合理期限内仍未支付到期价款的，出卖人可以请求买受人支付全部价款或者解除合同。

出卖人解除合同的，可以向买受人请求支付该标的物的使用费。

法宝联想

法律法规　司法解释（2）

司法案例　司法案例（6790）

法学期刊　法学期刊（33）

法宝律师　律所实务（3）

专题参考　专题参考（5）

第六百三十五条

凭样品买卖的当事人应当封存样品，并可以对样品质量予以说明。出卖人交付的标的物应当与样品及其说明的质量相同。

法宝联想

司法案例　司法案例（122）

法学期刊　法学期刊（1）

专题参考　专题参考（1）

第六百三十六条

凭样品买卖的买受人不知道样品有隐蔽瑕疵的，即使交付的标的物与样品相同，出卖人交付的标的物的质量仍然应当符合同种物的通常标准。

法宝联想

司法案例　司法案例（52）

专题参考　专题参考（1）

第六百三十七条

试用买卖的当事人可以约定标的物的试用期限。对试用期限没有约定或者约定不明确，依据本法第五百一十条的规定仍不能确定的，由出卖人确定。

法宝联想

司法案例　司法案例（33）

法学期刊　法学期刊（3）

专题参考　专题参考（1）

第六百三十八条

试用买卖的买受人在试用期内可以购买标的物，也可以拒绝购买。试用期限届满，买受人对是否购买标的物未作表示的，视为购买。

试用买卖的买受人在试用期内已经支付部分价款或者对标的物实施出卖、出租、设立担保物权等行为的，视为同意购买。

法宝联想

司法案例　司法案例（72）

法学期刊　法学期刊（11）

专题参考　专题参考（2）

第六百三十九条

试用买卖的当事人对标的物使用费没有约定或者约定不明确的，出卖人无权请求买受人支付。

法宝联想

司法案例　司法案例（27）

法学期刊　法学期刊（5）

专题参考　专题参考（1）

第六百四十条　标的物在试用期内毁损、灭失的风险由出卖人承担。

法宝联想

司法案例　司法案例（60）

法学期刊　法学期刊（3）

第六百四十一条

当事人可以在买卖合同中约定买受人未履行支付价款或者其他义务的，标的物的所有权属于出卖人。

出卖人对标的物保留的所有权，未经登记，不得对抗善意第三人。

法宝联想

法律法规 司法解释（4）规范性文件（2）

司法案例 司法案例（1186）案例报道（1）

法学期刊 法学期刊（92）

法宝律师 律所实务（1）

专题参考 专题参考（12）

第六百四十二条

当事人约定出卖人保留合同标的物的所有权，在标的物所有权转移前，买受人有下列情形之一，造成出卖人损害的，除当事人另有约定外，出卖人有权取回标的物：

（一）未按照约定支付价款，经催告后在合理期限内仍未支付；

（二）未按照约定完成特定条件；

（三）将标的物出卖、出质或者作出其他不当处分。

出卖人可以与买受人协商取回标的物；协商不成的，可以参照适用担保物权的实现程序。

法宝联想

法律法规 司法解释（3）规范性文件（1）

司法案例 司法案例（1096）案例报道（1）

法学期刊 法学期刊（55）

专题参考 专题参考（10）

第六百四十三条

出卖人依据前条第一款的规定取回标的物后，买受人在双方约定或者出卖人指定的合理回赎期限内，消除出卖人取回标的物的事由的，可以请求回赎标的物。

买受人在回赎期限内没有回赎标的物，出卖人可以以合理价格将标的物出卖给第三人，出卖所得价款扣除买受人未支付的价款以及必要费用后仍有剩余的，应当返还买受人；不足部分由买受人清偿。

法宝联想

法律法规　规范性文件（1）

司法案例　司法案例（603）

法学期刊　法学期刊（41）

法宝律师　律所实务（1）

专题参考　专题参考（5）

第六百四十四条

招标投标买卖的当事人的权利和义务以及招标投标程序等，依照有关法律、行政法规的规定。

法宝联想

司法案例　司法案例（134）

法学期刊　法学期刊（3）

专题参考　专题参考（3）

第六百四十五条

拍卖的当事人的权利和义务以及拍卖程序等，依照有关法律、行政法规的规定。

法宝联想

司法案例　司法案例（107）

法学期刊　法学期刊（2）

专题参考　专题参考（3）

第六百四十六条

法律对其他有偿合同有规定的，依照其规定；没有规定的，参照适用买卖合同的有关规定。

法宝联想

法律法规　司法解释（2）

司法案例　司法案例（18709）仲裁案例（2）

法学期刊　法学期刊（60）

法宝律师　律所实务（9）

专题参考　专题参考（5）

第六百四十七条

当事人约定易货交易，转移标的物的所有权的，参照适用买卖合同的有关规定。

法宝联想

司法案例　司法案例（541）

法学期刊　法学期刊（10）

专题参考　专题参考（4）

第十章　供用电、水、气、热力合同

第六百四十八条

供用电合同是供电人向用电人供电，用电人支付电费的合同。

向社会公众供电的供电人，不得拒绝用电人合理的订立合同要求。

法宝联想

司法案例　司法案例（3469）仲裁案例（1）

法学期刊　法学期刊（14）

专题参考　专题参考（1）

第六百四十九条

供用电合同的内容一般包括供电的方式、质量、时间，用电容量、地址、性质，计量方式，电价、电费的结算方式，供用电设施的维护责任等条款。

法宝联想

司法案例　司法案例（177）

法学期刊　法学期刊（1）

第六百五十条

供用电合同的履行地点，按照当事人约定；当事人没有约定或者约定不明确的，供电设施的产权分界处为履行地点。

法宝联想

司法案例　司法案例（218）

法学期刊　法学期刊（1）

第六百五十一条

供电人应当按照国家规定的供电质量标准和约定安全供电。供电人未按照国家规定的供电质量标准和约定安全供电，造成用电人损失的，应当承担赔偿责任。

法宝联想

司法案例　司法案例（650）

法学期刊　法学期刊（2）

专题参考　专题参考（1）

第六百五十二条

供电人因供电设施计划检修、临时检修、依法限电或者用电人违法用电等原因，

需要中断供电时，应当按照国家有关规定事先通知用电人；未事先通知用电人中断供电，造成用电人损失的，应当承担赔偿责任。

法宝联想

司法案例　司法案例（117）

法学期刊　法学期刊（3）

法宝律师　律所实务（1）

专题参考　专题参考（1）

第六百五十三条

因自然灾害等原因断电，供电人应当按照国家有关规定及时抢修；未及时抢修，造成用电人损失的，应当承担赔偿责任。

法宝联想

司法案例　司法案例（110）

法学期刊　法学期刊（1）

专题参考　专题参考（1）

第六百五十四条

用电人应当按照国家有关规定和当事人的约定及时支付电费。用电人逾期不支付电费的，应当按照约定支付违约金。经催告用电人在合理期限内仍不支付电费和违约金的，供电人可以按照国家规定的程序中止供电。

供电人依据前款规定中止供电的，应当事先通知用电人。

法宝联想

司法案例　司法案例（6098）仲裁案例（1）

法学期刊　法学期刊（2）

法宝律师　律所实务（1）

专题参考　专题参考（1）

第六百五十五条

用电人应当按照国家有关规定和当事人的约定安全、节约和计划用电。用电人未按照国家有关规定和当事人的约定用电，造成供电人损失的，应当承担赔偿责任。

法宝联想

司法案例　司法案例（129）

法学期刊　法学期刊（4）

专题参考　专题参考（2）

第六百五十六条

供用水、供用气、供用热力合同，参照适用供用电合同的有关规定。

法宝联想

司法案例　司法案例（7550）

法学期刊　法学期刊（7）

第十一章　赠与合同

第六百五十七条

赠与合同是赠与人将自己的财产无偿给予受赠人，受赠人表示接受赠与的合同。

法宝联想

司法案例　司法案例（8229）案例报道（5）仲裁案例（1）

法学期刊　法学期刊（22）

法宝律师　律所实务（8）

专题参考　专题参考（4）

第六百五十八条　赠与人在赠与财产的权利转移之前可以撤销赠与。

经过公证的赠与合同或者依法不得撤销的具有救灾、扶贫、助残等公益、道德义务性质的赠与合同，不适用前款规定。

法宝联想

法律法规　司法解释（1）规范性文件（1）

司法案例　司法案例（3320）案例报道（3）

法学期刊　法学期刊（70）

法宝律师　律所实务（11）

专题参考　专题参考（7）

第六百五十九条

赠与的财产依法需要办理登记或者其他手续的，应当办理有关手续。

法宝联想

司法案例　司法案例（852）

法学期刊　法学期刊（3）

第六百六十条

经过公证的赠与合同或者依法不得撤销的具有救灾、扶贫、助残等公益、道德义务性质的赠与合同，赠与人不交付赠与财产的，受赠人可以请求交付。

依据前款规定应当交付的赠与财产因赠与人故意或者重大过失致使毁损、灭失的，赠与人应当承担赔偿责任。

法宝联想

司法案例　司法案例（338）

法学期刊　法学期刊（16）

法宝律师　律所实务（2）

专题参考　专题参考（1）

第六百六十一条　赠与可以附义务。

赠与附义务的，受赠人应当按照约定履行义务。

法宝联想

司法案例　司法案例（1781）案例报道（2）

法学期刊　法学期刊（10）

法宝律师　律所实务（3）

第六百六十二条

赠与的财产有瑕疵的，赠与人不承担责任。附义务的赠与，赠与的财产有瑕疵的，赠与人在附义务的限度内承担与出卖人相同的责任。

赠与人故意不告知瑕疵或者保证无瑕疵，造成受赠人损失的，应当承担赔偿责任。

法宝联想

法律法规　法律动态（1）

司法案例　司法案例（576）

法学期刊　法学期刊（14）

专题参考　专题参考（1）

第六百六十三条　受赠人有下列情形之一的，赠与人可以撤销赠与：

（一）严重侵害赠与人或者赠与人近亲属的合法权益；

（二）对赠与人有扶养义务而不履行；

（三）不履行赠与合同约定的义务。

赠与人的撤销权，自知道或者应当知道撤销事由之日起一年内行使。

法宝联想

法律法规 规范性文件（3）法律动态（3）

司法案例 司法案例（2774）案例报道（10）

法学期刊 法学期刊（38）

法宝律师 律所实务（4）

专题参考 专题参考（2）

第六百六十四条

因受赠人的违法行为致使赠与人死亡或者丧失民事行为能力的，赠与人的继承人或者法定代理人可以撤销赠与。

赠与人的继承人或者法定代理人的撤销权，自知道或者应当知道撤销事由之日起六个月内行使。

法宝联想

司法案例 司法案例（351）

法学期刊 法学期刊（6）

专题参考 专题参考（1）

第六百六十五条

撤销权人撤销赠与的，可以向受赠人请求返还赠与的财产。

法宝联想

司法案例 司法案例（1471）

法学期刊 法学期刊（8）

专题参考 专题参考（1）

第六百六十六条

赠与人的经济状况显著恶化，严重影响其生产经营或者家庭生活的，可以不再履行赠与义务。

法宝联想

司法案例　司法案例（815）

法学期刊　法学期刊（15）

法宝律师　律所实务（1）

专题参考　专题参考（1）

第十二章　借款合同

第六百六十七条

借款合同是借款人向贷款人借款，到期返还借款并支付利息的合同。

法宝联想

法律法规　规范性文件（1）

司法案例　司法案例（864226）案例报道（6）仲裁案例（6）

法学期刊　法学期刊（18）

法宝律师　律所实务（6）

专题参考　专题参考（6）

第六百六十八条

借款合同应当采用书面形式，但是自然人之间借款另有约定的除外。

借款合同的内容一般包括借款种类、币种、用途、数额、利率、期限和还款方式等条款。

法宝联想

司法案例　司法案例（40276）案例报道（1）仲裁案例（1）

法学期刊　法学期刊（8）

法宝律师　律所实务（1）

专题参考　专题参考（4）

第六百六十九条

订立借款合同，借款人应当按照贷款人的要求提供与借款有关的业务活动和财务状况的真实情况。

法宝联想

司法案例　司法案例（1666）案例报道（1）

法学期刊　法学期刊（1）

法宝律师　律所实务（1）

第六百七十条

借款的利息不得预先在本金中扣除。利息预先在本金中扣除的，应当按照实际借款数额返还借款并计算利息。

法宝联想

法律法规　规范性文件（3）法律动态（2）

司法案例　司法案例（34166）案例报道（8）仲裁案例（6）

法学期刊　法学期刊（15）

法宝律师　律所实务（5）

专题参考　专题参考（5）

第六百七十一条

贷款人未按照约定的日期、数额提供借款，造成借款人损失的，应当赔偿损失。

借款人未按照约定的日期、数额收取借款的，应当按照约定的日期、数额支付利息。

法宝联想

司法案例　司法案例（16639）案例报道（1）

法学期刊　法学期刊（2）

第六百七十二条

贷款人按照约定可以检查、监督借款的使用情况。借款人应当按照约定向贷款人定期提供有关财务会计报表或者其他资料。

法宝联想

法律法规　规范性文件（1）

司法案例　司法案例（141）

法学期刊　法学期刊（4）

专题参考　专题参考（1）

第六百七十三条

借款人未按照约定的借款用途使用借款的，贷款人可以停止发放借款、提前收回借款或者解除合同。

法宝联想

法律法规　规范性文件（1）

司法案例　司法案例（3230）仲裁案例（1）

法学期刊　法学期刊（13）

专题参考　专题参考（1）

第六百七十四条

借款人应当按照约定的期限支付利息。对支付利息的期限没有约定或者约定不明确，依据本法第五百一十条的规定仍不能确定，借款期间不满一年的，应当在返还借款时一并支付；借款期间一年以上的，应当在每届满一年时支付，剩余期间不满一年的，应当在返还借款时一并支付。

法宝联想

法律法规 规范性文件（1）

司法案例 司法案例（1462047）案例报道（4）仲裁案例（6）

法学期刊 法学期刊（8）

法宝律师 律所实务（2）

专题参考 专题参考（1）

第六百七十五条

借款人应当按照约定的期限返还借款。对借款期限没有约定或者约定不明确，依据本法第五百一十条的规定仍不能确定的，借款人可以随时返还；贷款人可以催告借款人在合理期限内返还。

法宝联想

法律法规 规范性文件（1）

司法案例 司法案例（2238238）案例报道（9）仲裁案例（6）

法学期刊 法学期刊（10）

法宝律师 律所实务（3）

专题参考 专题参考（2）

第六百七十六条

借款人未按照约定的期限返还借款的，应当按照约定或者国家有关规定支付逾期利息。

法宝联想

司法案例　司法案例（1948482）案例报道（2）仲裁案例（11）

法学期刊　法学期刊（9）

法宝律师　律所实务（2）

专题参考　专题参考（5）

第六百七十七条

借款人提前返还借款的，除当事人另有约定外，应当按照实际借款的期间计算利息。

法宝联想

司法案例　司法案例（6224）

法学期刊　法学期刊（3）

专题参考　专题参考（3）

第六百七十八条

借款人可以在还款期限届满前向贷款人申请展期；贷款人同意的，可以展期。

法宝联想

司法案例　司法案例（3159）

法宝律师　律所实务（1）

第六百七十九条　自然人之间的借款合同，自贷款人提供借款时成立。

法宝联想

司法案例　司法案例（212579）案例报道（2）仲裁案例（1）

法学期刊　法学期刊（21）

法宝律师　律所实务（7）

专题参考　专题参考（9）

第六百八十条 禁止高利放贷，借款的利率不得违反国家有关规定。

借款合同对支付利息没有约定的，视为没有利息。

借款合同对支付利息约定不明确，当事人不能达成补充协议的，按照当地或者当事人的交易方式、交易习惯、市场利率等因素确定利息；自然人之间借款的，视为没有利息。

法宝联想

法律法规 规范性文件（2）法律动态（1）

司法案例 司法案例（152508）案例报道（9）仲裁案例（4）

法学期刊 法学期刊（65）

法宝律师 律所实务（21）

专题参考 专题参考（19）

第十三章 保证合同

第一节 一般规定

第六百八十一条

保证合同是为保障债权的实现，保证人和债权人约定，当债务人不履行到期债务或者发生当事人约定的情形时，保证人履行债务或者承担责任的合同。

法宝联想

司法案例 司法案例（91352）仲裁案例（3）

法学期刊 法学期刊（27）

法宝律师 律所实务（3）

专题参考 专题参考（4）

第六百八十二条

保证合同是主债权债务合同的从合同。主债权债务合同无效的，保证合同无效，但是法律另有规定的除外。

保证合同被确认无效后，债务人、保证人、债权人有过错的，应当根据其过错各自承担相应的民事责任。

法宝联想

司法案例　司法案例（3757）仲裁案例（1）

法学期刊　法学期刊（54）

法宝律师　律所实务（9）

专题参考　专题参考（8）

第六百八十三条

机关法人不得为保证人，但是经国务院批准为使用外国政府或者国际经济组织贷款进行转贷的除外。

以公益为目的的非营利法人、非法人组织不得为保证人。

法宝联想

法律法规　规范性文件（2）

司法案例　司法案例（312）仲裁案例（1）

法学期刊　法学期刊（27）

法宝律师　律所实务（3）

专题参考　专题参考（7）

第六百八十四条

保证合同的内容一般包括被保证的主债权的种类、数额，债务人履行债务的期限，保证的方式、范围和期间等条款。

法宝联想

法律法规　规范性文件（1）

司法案例　司法案例（6717）

法学期刊　法学期刊（5）

专题参考　专题参考（2）

第六百八十五条

保证合同可以是单独订立的书面合同，也可以是主债权债务合同中的保证条款。

第三人单方以书面形式向债权人作出保证，债权人接收且未提出异议的，保证合同成立。

法宝联想

法律法规　规范性文件（1）

司法案例　司法案例（16743）案例报道（1）

法学期刊　法学期刊（28）

法宝律师　律所实务（2）

专题参考　专题参考（4）

第六百八十六条　保证的方式包括一般保证和连带责任保证。

当事人在保证合同中对保证方式没有约定或者约定不明确的，按照一般保证承担保证责任。

法宝联想

法律法规　规范性文件（1）

司法案例　司法案例（43268）案例报道（6）仲裁案例（7）

法学期刊　法学期刊（37）

法宝律师　律所实务（7）

专题参考　专题参考（7）

第六百八十七条

当事人在保证合同中约定，债务人不能履行债务时，由保证人承担保证责任的，为一般保证。

一般保证的保证人在主合同纠纷未经审判或者仲裁，并就债务人财产依法强制执行仍不能履行债务前，有权拒绝向债权人承担保证责任，但是有下列情形之一的除外：

（一）债务人下落不明，且无财产可供执行；

（二）人民法院已经受理债务人破产案件；

（三）债权人有证据证明债务人的财产不足以履行全部债务或者丧失履行债务能力；

（四）保证人书面表示放弃本款规定的权利。

法宝联想

法律法规　司法解释（1）规范性文件（1）法规解读（1）

司法案例　司法案例（16988）案例报道（2）仲裁案例（2）

法学期刊　法学期刊（72）

法宝律师　律所实务（9）

专题参考　专题参考（14）

第六百八十八条

当事人在保证合同中约定保证人和债务人对债务承担连带责任的，为连带责任保证。

连带责任保证的债务人不履行到期债务或者发生当事人约定的情形时，债权人可以请求债务人履行债务，也可以请求保证人在其保证范围内承担保证责任。

法宝联想

法律法规　规范性文件（1）

司法案例　司法案例（313942）案例报道（1）仲裁案例（9）

法学期刊　法学期刊（20）

法宝律师　律所实务（4）

专题参考　专题参考（8）

第六百八十九条　保证人可以要求债务人提供反担保。

法宝联想

法律法规　规范性文件（1）

司法案例　司法案例（12065）仲裁案例（3）

法学期刊　法学期刊（4）

法宝律师　律所实务（1）

专题参考　专题参考（2）

第六百九十条

保证人与债权人可以协商订立最高额保证的合同，约定在最高债权额限度内就一定期间连续发生的债权提供保证。

最高额保证除适用本章规定外，参照适用本法第二编最高额抵押权的有关规定。

法宝联想

司法案例　司法案例（33868）仲裁案例（1）

法学期刊　法学期刊（14）

法宝律师　律所实务（2）

专题参考　专题参考（6）

第二节　保证责任

第六百九十一条

保证的范围包括主债权及其利息、违约金、损害赔偿金和实现债权的费用。当事人另有约定的，按照其约定。

法宝联想

司法案例　司法案例（192129）案例报道（1）仲裁案例（4）

法学期刊　法学期刊（22）

法宝律师　律所实务（2）

专题参考　专题参考（5）

第六百九十二条

保证期间是确定保证人承担保证责任的期间，不发生中止、中断和延长。

债权人与保证人可以约定保证期间，但是约定的保证期间早于主债务履行期限或者与主债务履行期限同时届满的，视为没有约定；没有约定或者约定不明确的，保证期间为主债务履行期限届满之日起六个月。

债权人与债务人对主债务履行期限没有约定或者约定不明确的，保证期间自债权人请求债务人履行债务的宽限期届满之日起计算。

法宝联想

法律法规　规范性文件（1）法规解读（1）

司法案例　司法案例（62230）案例报道（4）仲裁案例（2）

法学期刊　法学期刊（36）

法宝律师　律所实务（7）

专题参考　专题参考（15）

第六百九十三条

一般保证的债权人未在保证期间对债务人提起诉讼或者申请仲裁的，保证人不再承担保证责任。

连带责任保证的债权人未在保证期间请求保证人承担保证责任的，保证人不再承担保证责任。

法宝联想

法律法规　法规解读（1）

司法案例　司法案例（20846）案例报道（2）

法学期刊　法学期刊（21）

法宝律师　律所实务（2）

专题参考　专题参考（13）

第六百九十四条

一般保证的债权人在保证期间届满前对债务人提起诉讼或者申请仲裁的，从保证人拒绝承担保证责任的权利消灭之日起，开始计算保证债务的诉讼时效。

连带责任保证的债权人在保证期间届满前请求保证人承担保证责任的，从债权人请求保证人承担保证责任之日起，开始计算保证债务的诉讼时效。

法宝联想

法律法规　法规解读（1）

司法案例　司法案例（6667）仲裁案例（1）

法学期刊　法学期刊（24）

法宝律师　律所实务（1）

专题参考　专题参考（9）

第六百九十五条

债权人和债务人未经保证人书面同意，协商变更主债权债务合同内容，减轻债务的，保证人仍对变更后的债务承担保证责任；加重债务的，保证人对加重的部分不承担保证责任。

债权人和债务人变更主债权债务合同的履行期限，未经保证人书面同意的，保证期间不受影响。

法宝联想

法律法规　司法解释（1）

司法案例　司法案例（2211）案例报道（1）仲裁案例（1）

法学期刊　法学期刊（19）

法宝律师　律所实务（3）

专题参考　专题参考（7）

第六百九十六条

债权人转让全部或者部分债权，未通知保证人的，该转让对保证人不发生效力。

保证人与债权人约定禁止债权转让，债权人未经保证人书面同意转让债权的，保证人对受让人不再承担保证责任。

法宝联想

法律法规　司法解释（1）

司法案例　司法案例（1154）

法学期刊　法学期刊（26）

法宝律师　律所实务（4）

专题参考　专题参考（3）

第六百九十七条

债权人未经保证人书面同意，允许债务人转移全部或者部分债务，保证人对未经其同意转移的债务不再承担保证责任，但是债权人和保证人另有约定的除外。

第三人加入债务的，保证人的保证责任不受影响。

法宝联想

法律法规　司法解释（1）

司法案例　司法案例（339）

法学期刊　法学期刊（18）

法宝律师　律所实务（1）

专题参考　专题参考（4）

第六百九十八条

一般保证的保证人在主债务履行期限届满后，向债权人提供债务人可供执行财产的真实情况，债权人放弃或者怠于行使权利致使该财产不能被执行的，保证人在其提供可供执行财产的价值范围内不再承担保证责任。

法宝联想

司法案例　司法案例（117）

法学期刊　法学期刊（11）

专题参考　专题参考（3）

第六百九十九条

同一债务有两个以上保证人的，保证人应当按照保证合同约定的保证份额，承担

保证责任；没有约定保证份额的，债权人可以请求任何一个保证人在其保证范围内承担保证责任。

法宝联想

法律法规　司法解释（1）

司法案例　司法案例（15508）

法学期刊　法学期刊（31）

法宝律师　律所实务（2）

专题参考　专题参考（6）

第七百条

保证人承担保证责任后，除当事人另有约定外，有权在其承担保证责任的范围内向债务人追偿，享有债权人对债务人的权利，但是不得损害债权人的利益。

法宝联想

法律法规　司法解释（1）规范性文件（1）

司法案例　司法案例（305523）案例报道（7）仲裁案例（7）

法学期刊　法学期刊（82）

法宝律师　律所实务（8）

专题参考　专题参考（14）

第七百零一条

保证人可以主张债务人对债权人的抗辩。债务人放弃抗辩的，保证人仍有权向债权人主张抗辩。

法宝联想

法律法规　司法解释（1）法规解读（1）

司法案例　司法案例（371）

法学期刊　法学期刊（39）

法宝律师　律所实务（3）

专题参考　专题参考（5）

第七百零二条

债务人对债权人享有抵销权或者撤销权的，保证人可以在相应范围内拒绝承担保证责任。

法宝联想

法律法规　司法解释（1）

司法案例　司法案例（105）

法学期刊　法学期刊（30）

专题参考　专题参考（4）

第十四章　租赁合同

第七百零三条

租赁合同是出租人将租赁物交付承租人使用、收益，承租人支付租金的合同。

法宝联想

司法案例　司法案例（110305）案例报道（1）仲裁案例（6）

法学期刊　法学期刊（11）

法宝律师　律所实务（2）

专题参考　专题参考（2）

第七百零四条

租赁合同的内容一般包括租赁物的名称、数量、用途、租赁期限、租金及其支付期限和方式、租赁物维修等条款。

法宝联想

司法案例　司法案例（3662）仲裁案例（4）

法学期刊　法学期刊（5）

法宝律师　律所实务（2）

第七百零五条　租赁期限不得超过二十年。超过二十年的，超过部分无效。

租赁期限届满，当事人可以续订租赁合同；但是，约定的租赁期限自续订之日起不得超过二十年。

法宝联想

法律法规　规范性文件（4）法律动态（1）

司法案例　司法案例（3935）仲裁案例（2）

法学期刊　法学期刊（24）

法宝律师　律所实务（8）

专题参考　专题参考（2）

第七百零六条

当事人未依照法律、行政法规规定办理租赁合同登记备案手续的，不影响合同的效力。

法宝联想

法律法规　法规解读（1）法律动态（1）

司法案例　司法案例（246）

法学期刊　法学期刊（10）

专题参考　专题参考（1）

第七百零七条

租赁期限六个月以上的，应当采用书面形式。当事人未采用书面形式，无法确定租赁期限的，视为不定期租赁。

法宝联想

司法案例　司法案例（5934）

法学期刊　法学期刊（8）

专题参考　专题参考（1）

第七百零八条

出租人应当按照约定将租赁物交付承租人，并在租赁期限内保持租赁物符合约定的用途。

法宝联想

法律法规　规范性文件（1）

司法案例　司法案例（7719）案例报道（1）

法学期刊　法学期刊（9）

法宝律师　律所实务（1）

专题参考　专题参考（1）

第七百零九条

承租人应当按照约定的方法使用租赁物。对租赁物的使用方法没有约定或者约定不明确，依据本法第五百一十条的规定仍不能确定的，应当根据租赁物的性质使用。

法宝联想

司法案例　司法案例（2376）案例报道（1）

法学期刊　法学期刊（3）

专题参考　专题参考（3）

第七百一十条

承租人按照约定的方法或者根据租赁物的性质使用租赁物，致使租赁物受到损耗的，不承担赔偿责任。

法宝联想

司法案例　司法案例（1123）

法学期刊　法学期刊（2）

专题参考　专题参考（2）

第七百一十一条

承租人未按照约定的方法或者未根据租赁物的性质使用租赁物，致使租赁物受到损失的，出租人可以解除合同并请求赔偿损失。

法宝联想

法律法规　司法解释（2）

司法案例　司法案例（3148）案例报道（2）

法学期刊　法学期刊（6）

专题参考　专题参考（3）

第七百一十二条

出租人应当履行租赁物的维修义务，但是当事人另有约定的除外。

法宝联想

司法案例　司法案例（2841）

法学期刊　法学期刊（4）

法宝律师　律所实务（2）

第七百一十三条

承租人在租赁物需要维修时可以请求出租人在合理期限内维修。出租人未履行维修义务的，承租人可以自行维修，维修费用由出租人负担。因维修租赁物影响承租人使用的，应当相应减少租金或者延长租期。

因承租人的过错致使租赁物需要维修的，出租人不承担前款规定的维修义务。

法宝联想

司法案例　司法案例（2307）

法学期刊　法学期刊（7）

法宝律师　律所实务（2）

专题参考 专题参考（1）

第七百一十四条

承租人应当妥善保管租赁物，因保管不善造成租赁物毁损、灭失的，应当承担赔偿责任。

法宝联想

司法案例 司法案例（6832）案例报道（3）

法学期刊 法学期刊（7）

专题参考 专题参考（1）

第七百一十五条

承租人经出租人同意，可以对租赁物进行改善或者增设他物。

承租人未经出租人同意，对租赁物进行改善或者增设他物的，出租人可以请求承租人恢复原状或者赔偿损失。

法宝联想

司法案例 司法案例（2057）案例报道（1）仲裁案例（1）

法学期刊 法学期刊（14）

法宝律师 律所实务（2）

第七百一十六条

承租人经出租人同意，可以将租赁物转租给第三人。承租人转租的，承租人与出租人之间的租赁合同继续有效；第三人造成租赁物损失的，承租人应当赔偿损失。

承租人未经出租人同意转租的，出租人可以解除合同。

法宝联想

法律法规　规范性文件（1）

司法案例　司法案例（4566）案例报道（2）

法学期刊　法学期刊（37）

法宝律师　律所实务（5）

专题参考　专题参考（4）

第七百一十七条

承租人经出租人同意将租赁物转租给第三人，转租期限超过承租人剩余租赁期限的，超过部分的约定对出租人不具有法律约束力，但是出租人与承租人另有约定的除外。

法宝联想

法律法规　规范性文件（1）

司法案例　司法案例（1204）案例报道（1）

法学期刊　法学期刊（3）

法宝律师　律所实务（3）

专题参考　专题参考（2）

第七百一十八条

出租人知道或者应当知道承租人转租，但是在六个月内未提出异议的，视为出租人同意转租。

法宝联想

司法案例　司法案例（2284）案例报道（2）

法学期刊　法学期刊（7）

法宝律师　律所实务（4）

专题参考　专题参考（2）

第七百一十九条

承租人拖欠租金的，次承租人可以代承租人支付其欠付的租金和违约金，但是转租合同对出租人不具有法律约束力的除外。

次承租人代为支付的租金和违约金，可以充抵次承租人应当向承租人支付的租金；超出其应付的租金数额的，可以向承租人追偿。

法宝联想
司法案例　司法案例（816）
法学期刊　法学期刊（12）
法宝律师　律所实务（1）
专题参考　专题参考（3）

第七百二十条

在租赁期限内因占有、使用租赁物获得的收益，归承租人所有，但是当事人另有约定的除外。

法宝联想
司法案例　司法案例（611）
法学期刊　法学期刊（2）

第七百二十一条

承租人应当按照约定的期限支付租金。对支付租金的期限没有约定或者约定不明确，依据本法第五百一十条的规定仍不能确定，租赁期限不满一年的，应当在租赁期限届满时支付；租赁期限一年以上的，应当在每届满一年时支付，剩余期限不满一年的，应当在租赁期限届满时支付。

法宝联想

司法案例　司法案例（85682）案例报道（1）仲裁案例（2）

法学期刊　法学期刊（2）

法宝律师　律所实务（2）

专题参考　专题参考（1）

第七百二十二条

承租人无正当理由未支付或者迟延支付租金的，出租人可以请求承租人在合理期限内支付；承租人逾期不支付的，出租人可以解除合同。

法宝联想

司法案例　司法案例（46374）案例报道（1）仲裁案例（5）

法学期刊　法学期刊（12）

法宝律师　律所实务（5）

专题参考　专题参考（3）

第七百二十三条

因第三人主张权利，致使承租人不能对租赁物使用、收益的，承租人可以请求减少租金或者不支付租金。

第三人主张权利的，承租人应当及时通知出租人。

法宝联想

法律法规　规范性文件（1）

司法案例　司法案例（907）案例报道（1）

法学期刊　法学期刊（5）

第七百二十四条

有下列情形之一，非因承租人原因致使租赁物无法使用的，承租人可以解除合同：

（一）租赁物被司法机关或者行政机关依法查封、扣押；

（二）租赁物权属有争议；

（三）租赁物具有违反法律、行政法规关于使用条件的强制性规定情形。

法宝联想

法律法规　规范性文件（1）

司法案例　司法案例（1317）案例报道（1）

法学期刊　法学期刊（13）

专题参考　专题参考（2）

第七百二十五条

租赁物在承租人按照租赁合同占有期限内发生所有权变动的，不影响租赁合同的效力。

法宝联想

法律法规　规范性文件（2）

司法案例　司法案例（3497）案例报道（4）仲裁案例（2）

法学期刊　法学期刊（61）

法宝律师　律所实务（6）

专题参考　专题参考（7）

第七百二十六条

出租人出卖租赁房屋的，应当在出卖之前的合理期限内通知承租人，承租人享有以同等条件优先购买的权利；但是，房屋按份共有人行使优先购买权或者出租人将房屋出卖给近亲属的除外。

出租人履行通知义务后，承租人在十五日内未明确表示购买的，视为承租人放弃优先购买权。

法宝联想

法律法规　规范性文件（1）

司法案例　司法案例（443）案例报道（1）

法学期刊　法学期刊（28）

法宝律师　律所实务（6）

专题参考　专题参考（5）

第七百二十七条

出租人委托拍卖人拍卖租赁房屋的，应当在拍卖五日前通知承租人。承租人未参加拍卖的，视为放弃优先购买权。

法宝联想

司法案例　司法案例（97）

法学期刊　法学期刊（7）

专题参考　专题参考（1）

第七百二十八条

出租人未通知承租人或者有其他妨害承租人行使优先购买权情形的，承租人可以请求出租人承担赔偿责任。但是，出租人与第三人订立的房屋买卖合同的效力不受影响。

法宝联想

司法案例　司法案例（270）

法学期刊　法学期刊（16）

法宝律师　律所实务（2）

专题参考　专题参考（4）

第七百二十九条

因不可归责于承租人的事由，致使租赁物部分或者全部毁损、灭失的，承租人可

以请求减少租金或者不支付租金；因租赁物部分或者全部毁损、灭失，致使不能实现合同目的的，承租人可以解除合同。

法宝联想

司法案例　司法案例（714）仲裁案例（1）

法学期刊　法学期刊（5）

第七百三十条

当事人对租赁期限没有约定或者约定不明确，依据本法第五百一十条的规定仍不能确定的，视为不定期租赁；当事人可以随时解除合同，但是应当在合理期限之前通知对方。

法宝联想

法律法规　司法解释（2）

司法案例　司法案例（11554）案例报道（1）仲裁案例（1）

法学期刊　法学期刊（12）

法宝律师　律所实务（2）

专题参考　专题参考（1）

第七百三十一条

租赁物危及承租人的安全或者健康的，即使承租人订立合同时明知该租赁物质量不合格，承租人仍然可以随时解除合同。

法宝联想

司法案例　司法案例（500）案例报道（1）

法学期刊　法学期刊（8）

第七百三十二条

承租人在房屋租赁期限内死亡的，与其生前共同居住的人或者共同经营人可以按照原租赁合同租赁该房屋。

法宝联想

司法案例　司法案例（247）

法学期刊　法学期刊（10）

专题参考　专题参考（2）

第七百三十三条

租赁期限届满，承租人应当返还租赁物。返还的租赁物应当符合按照约定或者根据租赁物的性质使用后的状态。

法宝联想

司法案例　司法案例（18783）案例报道（1）仲裁案例（2）

法学期刊　法学期刊（4）

第七百三十四条

租赁期限届满，承租人继续使用租赁物，出租人没有提出异议的，原租赁合同继续有效，但是租赁期限为不定期。

租赁期限届满，房屋承租人享有以同等条件优先承租的权利。

法宝联想

法律法规　司法解释（1）规范性文件（2）

司法案例　司法案例（15551）仲裁案例（5）

法学期刊　法学期刊（18）

法宝律师　律所实务（4）

专题参考　专题参考（5）

第十五章　融资租赁合同

第七百三十五条

融资租赁合同是出租人根据承租人对出卖人、租赁物的选择，向出卖人购买租赁物，提供给承租人使用，承租人支付租金的合同。

法宝联想

法律法规　司法解释（2）规范性文件（1）

司法案例　司法案例（34866）案例报道（3）仲裁案例（1）

法学期刊　法学期刊（22）

法宝律师　律所实务（10）

专题参考　专题参考（3）

第七百三十六条

融资租赁合同的内容一般包括租赁物的名称、数量、规格、技术性能、检验方法，租赁期限，租金构成及其支付期限和方式、币种，租赁期限届满租赁物的归属等条款。

融资租赁合同应当采用书面形式。

法宝联想

司法案例　司法案例（1130）

法学期刊　法学期刊（7）

法宝律师　律所实务（2）

专题参考　专题参考（1）

第七百三十七条　当事人以虚构租赁物方式订立的融资租赁合同无效。

法宝联想

司法案例　司法案例（175）

法学期刊　法学期刊（12）

法宝律师　律所实务（8）

专题参考　专题参考（1）

第七百三十八条

依照法律、行政法规的规定，对于租赁物的经营使用应当取得行政许可的，出租人未取得行政许可不影响融资租赁合同的效力。

法宝联想

司法案例 司法案例（48）

法学期刊 法学期刊（4）

法宝律师 律所实务（2）

专题参考 专题参考（2）

第七百三十九条

出租人根据承租人对出卖人、租赁物的选择订立的买卖合同，出卖人应当按照约定向承租人交付标的物，承租人享有与受领标的物有关的买受人的权利。

法宝联想

司法案例 司法案例（544）

法学期刊 法学期刊（3）

法宝律师 律所实务（1）

第七百四十条

出卖人违反向承租人交付标的物的义务，有下列情形之一的，承租人可以拒绝受领出卖人向其交付的标的物：

（一）标的物严重不符合约定；

（二）未按照约定交付标的物，经承租人或者出租人催告后在合理期限内仍未交付。

承租人拒绝受领标的物的，应当及时通知出租人。

法宝联想

司法案例 司法案例（17）

法学期刊 法学期刊（2）

法宝律师 律所实务（1）

专题参考 专题参考（1）

第七百四十一条

出租人、出卖人、承租人可以约定，出卖人不履行买卖合同义务的，由承租人行使索赔的权利。承租人行使索赔权利的，出租人应当协助。

法宝联想

司法案例 司法案例（60）

法学期刊 法学期刊（4）

法宝律师 律所实务（1）

第七百四十二条

承租人对出卖人行使索赔权利，不影响其履行支付租金的义务。但是，承租人依赖出租人的技能确定租赁物或者出租人干预选择租赁物的，承租人可以请求减免相应租金。

法宝联想

法律法规 白皮书（1）

司法案例 司法案例（81）案例报道（1）

法学期刊 法学期刊（2）

法宝律师 律所实务（2）

专题参考 专题参考（1）

第七百四十三条

出租人有下列情形之一，致使承租人对出卖人行使索赔权利失败的，承租人有权请求出租人承担相应的责任：

（一）明知租赁物有质量瑕疵而不告知承租人；

（二）承租人行使索赔权利时，未及时提供必要协助。

出租人怠于行使只能由其对出卖人行使的索赔权利，造成承租人损失的，承租人有权请求出租人承担赔偿责任。

法宝联想

司法案例　司法案例（43）

法学期刊　法学期刊（2）

法宝律师　律所实务（1）

专题参考　专题参考（2）

第七百四十四条

出租人根据承租人对出卖人、租赁物的选择订立的买卖合同，未经承租人同意，出租人不得变更与承租人有关的合同内容。

法宝联想

法律法规　司法解释（2）

司法案例　司法案例（21）

法学期刊　法学期刊（2）

法宝律师　律所实务（1）

第七百四十五条

出租人对租赁物享有的所有权，未经登记，不得对抗善意第三人。

法宝联想

法律法规　规范性文件（1）

司法案例　司法案例（3044）仲裁案例（1）

法学期刊　法学期刊（64）

法宝律师　律所实务（8）

专题参考　专题参考（10）

第七百四十六条

融资租赁合同的租金，除当事人另有约定外，应当根据购买租赁物的大部分或者全部成本以及出租人的合理利润确定。

法宝联想

司法案例　司法案例（1898）案例报道（1）

法学期刊　法学期刊（10）

法宝律师　律所实务（2）

专题参考　专题参考（3）

第七百四十七条

租赁物不符合约定或者不符合使用目的的，出租人不承担责任。但是，承租人依赖出租人的技能确定租赁物或者出租人干预选择租赁物的除外。

法宝联想

法律法规　司法解释（2）

司法案例　司法案例（97）

法学期刊　法学期刊（5）

法宝律师　律所实务（1）

第七百四十八条　出租人应当保证承租人对租赁物的占有和使用。

出租人有下列情形之一的，承租人有权请求其赔偿损失：

（一）无正当理由收回租赁物；

（二）无正当理由妨碍、干扰承租人对租赁物的占有和使用；

（三）因出租人的原因致使第三人对租赁物主张权利；

（四）不当影响承租人对租赁物占有和使用的其他情形。

法宝联想

司法案例　司法案例（176）

法学期刊　法学期刊（9）

法宝律师　律所实务（1）

专题参考　专题参考（2）

第七百四十九条

承租人占有租赁物期间，租赁物造成第三人人身损害或者财产损失的，出租人不承担责任。

法宝联想

司法案例　司法案例（308）

法学期刊　法学期刊（4）

法宝律师　律所实务（1）

第七百五十条　承租人应当妥善保管、使用租赁物。

承租人应当履行占有租赁物期间的维修义务。

法宝联想

司法案例　司法案例（162）

法学期刊　法学期刊（3）

法宝律师　律所实务（1）

第七百五十一条

承租人占有租赁物期间，租赁物毁损、灭失的，出租人有权请求承租人继续支付租金，但是法律另有规定或者当事人另有约定的除外。

法宝联想

法律法规　规范性文件（1）

司法案例　司法案例（140）

法学期刊　法学期刊（5）

法宝律师　律所实务（2）

专题参考　专题参考（2）

第七百五十二条

承租人应当按照约定支付租金。承租人经催告后在合理期限内仍不支付租金的，出租人可以请求支付全部租金；也可以解除合同，收回租赁物。

法宝联想

法律法规　司法解释（2）规范性文件（2）

司法案例　司法案例（70994）案例报道（4）仲裁案例（3）

法学期刊　法学期刊（36）

法宝律师　律所实务（4）

专题参考　专题参考（3）

第七百五十三条

承租人未经出租人同意，将租赁物转让、抵押、质押、投资入股或者以其他方式处分的，出租人可以解除融资租赁合同。

法宝联想

司法案例　司法案例（73）

法学期刊　法学期刊（12）

法宝律师　律所实务（2）

专题参考　专题参考（2）

第七百五十四条

有下列情形之一的，出租人或者承租人可以解除融资租赁合同：

（一）出租人与出卖人订立的买卖合同解除、被确认无效或者被撤销，且未能重新订立买卖合同；

（二）租赁物因不可归责于当事人的原因毁损、灭失，且不能修复或者确定替代物；

（三）因出卖人的原因致使融资租赁合同的目的不能实现。

法宝联想

司法案例　司法案例（163）

法学期刊　法学期刊（9）

法宝律师　律所实务（2）

专题参考　专题参考（2）

第七百五十五条

融资租赁合同因买卖合同解除、被确认无效或者被撤销而解除，出卖人、租赁物系由承租人选择的，出租人有权请求承租人赔偿相应损失；但是，因出租人原因致使买卖合同解除、被确认无效或者被撤销的除外。

出租人的损失已经在买卖合同解除、被确认无效或者被撤销时获得赔偿的，承租人不再承担相应的赔偿责任。

法宝联想

司法案例 司法案例（160）

法学期刊 法学期刊（1）

法宝律师 律所实务（1）

专题参考 专题参考（2）

第七百五十六条

融资租赁合同因租赁物交付承租人后意外毁损、灭失等不可归责于当事人的原因解除的，出租人可以请求承租人按照租赁物折旧情况给予补偿。

法宝联想

司法案例 司法案例（14）

法学期刊 法学期刊（1）

法宝律师 律所实务（1）

专题参考 专题参考（1）

第七百五十七条

出租人和承租人可以约定租赁期限届满租赁物的归属；对租赁物的归属没有约定或者约定不明确，依据本法第五百一十条的规定仍不能确定的，租赁物的所有权归出租人。

法宝联想

法律法规 规范性文件（1）

司法案例 司法案例（2097）

法学期刊 法学期刊（13）

法宝律师 律所实务（1）

第七百五十八条

当事人约定租赁期限届满租赁物归承租人所有，承租人已经支付大部分租金，但

是无力支付剩余租金，出租人因此解除合同收回租赁物，收回的租赁物的价值超过承租人欠付的租金以及其他费用的，承租人可以请求相应返还。

当事人约定租赁期限届满租赁物归出租人所有，因租赁物毁损、灭失或者附合、混合于他物致使承租人不能返还的，出租人有权请求承租人给予合理补偿。

法宝联想

法律法规　规范性文件（1）

司法案例　司法案例（428）

法学期刊　法学期刊（24）

法宝律师　律所实务（1）

专题参考　专题参考（1）

第七百五十九条

当事人约定租赁期限届满，承租人仅需向出租人支付象征性价款的，视为约定的租金义务履行完毕后租赁物的所有权归承租人。

法宝联想

法律法规　规范性文件（1）

司法案例　司法案例（664）

法学期刊　法学期刊（11）

法宝律师　律所实务（2）

第七百六十条

融资租赁合同无效，当事人就该情形下租赁物的归属有约定的，按照其约定；没有约定或者约定不明确的，租赁物应当返还出租人。但是，因承租人原因致使合同无效，出租人不请求返还或者返还后会显著降低租赁物效用的，租赁物的所有权归承租人，由承租人给予出租人合理补偿。

法宝联想

司法案例 司法案例（870）

法学期刊 法学期刊（7）

法宝律师 律所实务（1）

专题参考 专题参考（1）

第十六章 保理合同

第七百六十一条

保理合同是应收账款债权人将现有的或者将有的应收账款转让给保理人，保理人提供资金融通、应收账款管理或者催收、应收账款债务人付款担保等服务的合同。

法宝联想

法律法规 规范性文件（2）

司法案例 司法案例（2784）案例报道（9）仲裁案例（2）

法学期刊 法学期刊（46）

法宝律师 律所实务（4）

专题参考 专题参考（10）

第七百六十二条

保理合同的内容一般包括业务类型、服务范围、服务期限、基础交易合同情况、应收账款信息、保理融资款或者服务报酬及其支付方式等条款。

保理合同应当采用书面形式。

法宝联想

司法案例 司法案例（132）

法学期刊 法学期刊（10）

专题参考 专题参考（3）

第七百六十三条

应收账款债权人与债务人虚构应收账款作为转让标的，与保理人订立保理合同的，应收账款债务人不得以应收账款不存在为由对抗保理人，但是保理人明知虚构的除外。

法宝联想

法律法规　规范性文件（2）

司法案例　司法案例（69）案例报道（3）

法学期刊　法学期刊（48）

法宝律师　律所实务（9）

专题参考　专题参考（4）

第七百六十四条

保理人向应收账款债务人发出应收账款转让通知的，应当表明保理人身份并附有必要凭证。

法宝联想

司法案例　司法案例（99）

法学期刊　法学期刊（21）

法宝律师　律所实务（5）

专题参考　专题参考（2）

第七百六十五条

应收账款债务人接到应收账款转让通知后，应收账款债权人与债务人无正当理由协商变更或者终止基础交易合同，对保理人产生不利影响的，对保理人不发生效力。

法宝联想

司法案例　司法案例（71）

法学期刊　法学期刊（17）

法宝律师　律所实务（1）

专题参考　专题参考（4）

第七百六十六条

当事人约定有追索权保理的，保理人可以向应收账款债权人主张返还保理融资款本息或者回购应收账款债权，也可以向应收账款债务人主张应收账款债权。保理人向应收账款债务人主张应收账款债权，在扣除保理融资款本息和相关费用后有剩余的，剩余部分应当返还给应收账款债权人。

法宝联想

法律法规　规范性文件（1）

司法案例　司法案例（1087）案例报道（4）仲裁案例（1）

法学期刊　法学期刊（39）

法宝律师　律所实务（4）

专题参考　专题参考（11）

第七百六十七条

当事人约定无追索权保理的，保理人应当向应收账款债务人主张应收账款债权，保理人取得超过保理融资款本息和相关费用的部分，无需向应收账款债权人返还。

法宝联想

司法案例　司法案例（314）

法学期刊　法学期刊（15）

专题参考　专题参考（4）

第七百六十八条

应收账款债权人就同一应收账款订立多个保理合同，致使多个保理人主张权利的，已经登记的先于未登记的取得应收账款；均已经登记的，按照登记时间的先后顺序取得应收账款；均未登记的，由最先到达应收账款债务人的转让通知中载明的保理人取得应收账款；既未登记也未通知的，按照保理融资款或者服务报酬的比例取得应收账款。

法宝联想

法律法规　司法解释（1）草案（1）

司法案例　司法案例（17）

法学期刊　法学期刊（76）

法宝律师　律所实务（7）

专题参考　专题参考（10）

第七百六十九条　本章没有规定的，适用本编第六章债权转让的有关规定。

法宝联想

司法案例　司法案例（475）案例报道（2）

法学期刊　法学期刊（14）

法宝律师　律所实务（1）

第十七章　承揽合同

第七百七十条

承揽合同是承揽人按照定作人的要求完成工作，交付工作成果，定作人支付报酬的合同。

承揽包括加工、定作、修理、复制、测试、检验等工作。

法宝联想

法律法规　规范性文件（1）

司法案例　司法案例（58935）案例报道（1）

法学期刊　法学期刊（15）

法宝律师　律所实务（5）

第七百七十一条

承揽合同的内容一般包括承揽的标的、数量、质量、报酬，承揽方式，材料的提供，履行期限，验收标准和方法等条款。

法宝联想

司法案例　司法案例（1835）

法学期刊　法学期刊（1）

第七百七十二条

承揽人应当以自己的设备、技术和劳力，完成主要工作，但是当事人另有约定的除外。

承揽人将其承揽的主要工作交由第三人完成的，应当就该第三人完成的工作成果向定作人负责；未经定作人同意的，定作人也可以解除合同。

法宝联想

法律法规　规范性文件（1）

司法案例　司法案例（2952）

法学期刊　法学期刊（5）

第七百七十三条

承揽人可以将其承揽的辅助工作交由第三人完成。承揽人将其承揽的辅助工作交由第三人完成的，应当就该第三人完成的工作成果向定作人负责。

法宝联想

司法案例 司法案例（351）

法学期刊 法学期刊（4）

第七百七十四条

承揽人提供材料的，应当按照约定选用材料，并接受定作人检验。

法宝联想

法律法规 规范性文件（1）

司法案例 司法案例（607）

法学期刊 法学期刊（1）

第七百七十五条

定作人提供材料的，应当按照约定提供材料。承揽人对定作人提供的材料应当及时检验，发现不符合约定时，应当及时通知定作人更换、补齐或者采取其他补救措施。

承揽人不得擅自更换定作人提供的材料，不得更换不需要修理的零部件。

法宝联想

司法案例 司法案例（483）

法学期刊 法学期刊（3）

第七百七十六条

承揽人发现定作人提供的图纸或者技术要求不合理的，应当及时通知定作人。因定作人怠于答复等原因造成承揽人损失的，应当赔偿损失。

法宝联想

司法案例 司法案例（335）

法学期刊 法学期刊（1）

专题参考 专题参考（1）

第七百七十七条

定作人中途变更承揽工作的要求，造成承揽人损失的，应当赔偿损失。

法宝联想

司法案例　司法案例（1035）

法学期刊　法学期刊（4）

专题参考　专题参考（3）

第七百七十八条

承揽工作需要定作人协助的，定作人有协助的义务。定作人不履行协助义务致使承揽工作不能完成的，承揽人可以催告定作人在合理期限内履行义务，并可以顺延履行期限；定作人逾期不履行的，承揽人可以解除合同。

法宝联想

司法案例　司法案例（401）

法学期刊　法学期刊（9）

法宝律师　律所实务（1）

第七百七十九条

承揽人在工作期间，应当接受定作人必要的监督检验。定作人不得因监督检验妨碍承揽人的正常工作。

法宝联想

司法案例　司法案例（513）

法宝律师　律所实务（1）

第七百八十条

承揽人完成工作的，应当向定作人交付工作成果，并提交必要的技术资料和有关质量证明。定作人应当验收该工作成果。

法宝联想

司法案例 司法案例（3829）

法学期刊 法学期刊（5）

第七百八十一条

承揽人交付的工作成果不符合质量要求的，定作人可以合理选择请求承揽人承担修理、重作、减少报酬、赔偿损失等违约责任。

法宝联想

法律法规 规范性文件（1）法律动态（2）

司法案例 司法案例（6586）案例报道（1）仲裁案例（1）

法学期刊 法学期刊（7）

法宝律师 律所实务（1）

专题参考 专题参考（1）

第七百八十二条

定作人应当按照约定的期限支付报酬。对支付报酬的期限没有约定或者约定不明确，依据本法第五百一十条的规定仍不能确定的，定作人应当在承揽人交付工作成果时支付；工作成果部分交付的，定作人应当相应支付。

法宝联想

司法案例 司法案例（50887）仲裁案例（1）

法学期刊 法学期刊（5）

专题参考 专题参考（1）

第七百八十三条

定作人未向承揽人支付报酬或者材料费等价款的，承揽人对完成的工作成果享有留置权或者有权拒绝交付，但是当事人另有约定的除外。

法宝联想

司法案例　司法案例（1016）

法学期刊　法学期刊（8）

法宝律师　律所实务（2）

第七百八十四条

承揽人应当妥善保管定作人提供的材料以及完成的工作成果，因保管不善造成毁损、灭失的，应当承担赔偿责任。

法宝联想

司法案例　司法案例（481）仲裁案例（1）

法学期刊　法学期刊（7）

专题参考　专题参考（1）

第七百八十五条

承揽人应当按照定作人的要求保守秘密，未经定作人许可，不得留存复制品或者技术资料。

法宝联想

司法案例　司法案例（55）

法学期刊　法学期刊（3）

第七百八十六条

共同承揽人对定作人承担连带责任，但是当事人另有约定的除外。

法宝联想

司法案例　司法案例（242）

法学期刊　法学期刊（7）

第七百八十七条

定作人在承揽人完成工作前可以随时解除合同，造成承揽人损失的，应当赔偿损失。

法宝联想

司法案例　司法案例（2554）仲裁案例（1）

法学期刊　法学期刊（33）

法宝律师　律所实务（1）

专题参考　专题参考（2）

第十八章　建设工程合同

第七百八十八条

建设工程合同是承包人进行工程建设，发包人支付价款的合同。

建设工程合同包括工程勘察、设计、施工合同。

法宝联想

司法案例　司法案例（36273）案例报道（2）仲裁案例（3）

法学期刊　法学期刊（5）

法宝律师　律所实务（12）

专题参考　专题参考（2）

第七百八十九条　建设工程合同应当采用书面形式。

法宝联想

司法案例　司法案例（1930）

法学期刊　法学期刊（2）

法宝律师　律所实务（1）

专题参考　专题参考（1）

第七百九十条

建设工程的招标投标活动，应当依照有关法律的规定公开、公平、公正进行。

法宝联想

司法案例　司法案例（295）

法学期刊　法学期刊（3）

法宝律师　律所实务（1）

专题参考　专题参考（1）

第七百九十一条

发包人可以与总承包人订立建设工程合同，也可以分别与勘察人、设计人、施工人订立勘察、设计、施工承包合同。发包人不得将应当由一个承包人完成的建设工程支解成若干部分发包给数个承包人。

总承包人或者勘察、设计、施工承包人经发包人同意，可以将自己承包的部分工作交由第三人完成。第三人就其完成的工作成果与总承包人或者勘察、设计、施工承包人向发包人承担连带责任。承包人不得将其承包的全部建设工程转包给第三人或者将其承包的全部建设工程支解以后以分包的名义分别转包给第三人。

禁止承包人将工程分包给不具备相应资质条件的单位。禁止分包单位将其承包的工程再分包。建设工程主体结构的施工必须由承包人自行完成。

法宝联想

法律法规　司法解释（1）规范性文件（2）法律动态（2）

司法案例　司法案例（23633）案例报道（2）仲裁案例（11）

法学期刊　法学期刊（19）

法宝律师　律所实务（15）

专题参考　专题参考（12）

第七百九十二条

国家重大建设工程合同，应当按照国家规定的程序和国家批准的投资计划、可行性研究报告等文件订立。

法宝联想

法律法规 规范性文件（1）

司法案例 司法案例（97）

法学期刊 法学期刊（1）

第七百九十三条

建设工程施工合同无效，但是建设工程经验收合格的，可以参照合同关于工程价款的约定折价补偿承包人。

建设工程施工合同无效，且建设工程经验收不合格的，按照以下情形处理：

（一）修复后的建设工程经验收合格的，发包人可以请求承包人承担修复费用；

（二）修复后的建设工程经验收不合格的，承包人无权请求参照合同关于工程价款的约定折价补偿。

发包人对因建设工程不合格造成的损失有过错的，应当承担相应的责任。

法宝联想

法律法规 规范性文件（3）法律动态（1）

司法案例 司法案例（24468）案例报道（6）仲裁案例（5）

法学期刊 法学期刊（22）

法宝律师 律所实务（25）

专题参考 专题参考（13）

第七百九十四条

勘察、设计合同的内容一般包括提交有关基础资料和概预算等文件的期限、质量要求、费用以及其他协作条件等条款。

法宝联想

法律法规　规范性文件（1）

司法案例　司法案例（150）

法学期刊　法学期刊（2）

第七百九十五条

施工合同的内容一般包括工程范围、建设工期、中间交工工程的开工和竣工时间、工程质量、工程造价、技术资料交付时间、材料和设备供应责任、拨款和结算、竣工验收、质量保修范围和质量保证期、相互协作等条款。

法宝联想

司法案例　司法案例（934）

法学期刊　法学期刊（2）

专题参考　专题参考（1）

第七百九十六条

建设工程实行监理的，发包人应当与监理人采用书面形式订立委托监理合同。发包人与监理人的权利和义务以及法律责任，应当依照本编委托合同以及其他有关法律、行政法规的规定。

法宝联想

司法案例　司法案例（309）

法学期刊　法学期刊（5）

专题参考　专题参考（1）

第七百九十七条

发包人在不妨碍承包人正常作业的情况下，可以随时对作业进度、质量进行检查。

法宝联想

司法案例 司法案例（69）

第七百九十八条

隐蔽工程在隐蔽以前，承包人应当通知发包人检查。发包人没有及时检查的，承包人可以顺延工程日期，并有权请求赔偿停工、窝工等损失。

法宝联想

司法案例 司法案例（81）

法学期刊 法学期刊（3）

专题参考 专题参考（2）

第七百九十九条

建设工程竣工后，发包人应当根据施工图纸及说明书、国家颁发的施工验收规范和质量检验标准及时进行验收。验收合格的，发包人应当按照约定支付价款，并接收该建设工程。

建设工程竣工经验收合格后，方可交付使用；未经验收或者验收不合格的，不得交付使用。

法宝联想

司法案例 司法案例（7251）案例报道（1）仲裁案例（6）

法学期刊 法学期刊（7）

法宝律师 律所实务（7）

专题参考 专题参考（7）

第八百条

勘察、设计的质量不符合要求或者未按照期限提交勘察、设计文件拖延工期，造成发包人损失的，勘察人、设计人应当继续完善勘察、设计，减收或者免收勘察、设计费并赔偿损失。

法宝联想

司法案例　司法案例（86）

法宝律师　律所实务（1）

专题参考　专题参考（1）

第八百零一条

因施工人的原因致使建设工程质量不符合约定的，发包人有权请求施工人在合理期限内无偿修理或者返工、改建。经过修理或者返工、改建后，造成逾期交付的，施工人应当承担违约责任。

法宝联想

司法案例　司法案例（1499）

法学期刊　法学期刊（3）

法宝律师　律所实务（3）

专题参考　专题参考（5）

第八百零二条

因承包人的原因致使建设工程在合理使用期限内造成人身损害和财产损失的，承包人应当承担赔偿责任。

法宝联想

司法案例　司法案例（363）

法学期刊　法学期刊（2）

法宝律师　律所实务（1）

专题参考　专题参考（2）

第八百零三条

发包人未按照约定的时间和要求提供原材料、设备、场地、资金、技术资料的，承包人可以顺延工程日期，并有权请求赔偿停工、窝工等损失。

法宝联想

司法案例　司法案例（530）仲裁案例（1）

法学期刊　法学期刊（7）

法宝律师　律所实务（4）

专题参考　专题参考（2）

第八百零四条

因发包人的原因致使工程中途停建、缓建的，发包人应当采取措施弥补或者减少损失，赔偿承包人因此造成的停工、窝工、倒运、机械设备调迁、材料和构件积压等损失和实际费用。

法宝联想

司法案例　司法案例（781）

法学期刊　法学期刊（3）

法宝律师　律所实务（1）

专题参考　专题参考（2）

第八百零五条

因发包人变更计划，提供的资料不准确，或者未按照期限提供必需的勘察、设计工作条件而造成勘察、设计的返工、停工或者修改设计，发包人应当按照勘察人、设计人实际消耗的工作量增付费用。

法宝联想

司法案例　司法案例（117）仲裁案例（1）

法学期刊　法学期刊（4）

第八百零六条

承包人将建设工程转包、违法分包的，发包人可以解除合同。

发包人提供的主要建筑材料、建筑构配件和设备不符合强制性标准或者不履行协助义务，致使承包人无法施工，经催告后在合理期限内仍未履行相应义务的，承包人可以解除合同。

合同解除后，已经完成的建设工程质量合格的，发包人应当按照约定支付相应的工程价款；已经完成的建设工程质量不合格的，参照本法第七百九十三条的规定处理。

法宝联想

司法案例　司法案例（784）

法学期刊　法学期刊（11）

法宝律师　律所实务（7）

专题参考　专题参考（2）

第八百零七条

发包人未按照约定支付价款的，承包人可以催告发包人在合理期限内支付价款。发包人逾期不支付的，除根据建设工程的性质不宜折价、拍卖外，承包人可以与发包人协议将该工程折价，也可以请求人民法院将该工程依法拍卖。建设工程的价款就该工程折价或者拍卖的价款优先受偿。

法宝联想

法律法规　司法解释（2）规范性文件（9）白皮书（1）法律动态（6）

司法案例　司法案例（22310）案例报道（11）仲裁案例（4）

法学期刊　法学期刊（57）

法宝律师　律所实务（41）

专题参考　专题参考（21）

第八百零八条　本章没有规定的，适用承揽合同的有关规定。

法宝联想

司法案例　司法案例（1173）

法学期刊　法学期刊（23）

法宝律师　律所实务（2）

专题参考　专题参考（1）

第十九章　运输合同

第一节　一般规定

第八百零九条

运输合同是承运人将旅客或者货物从起运地点运输到约定地点，旅客、托运人或者收货人支付票款或者运输费用的合同。

法宝联想

法律法规　规范性文件（1）

司法案例　司法案例（17546）仲裁案例（1）

法学期刊　法学期刊（6）

专题参考　专题参考（1）

第八百一十条

从事公共运输的承运人不得拒绝旅客、托运人通常、合理的运输要求。

法宝联想

司法案例 司法案例（42）

法学期刊 法学期刊（7）

专题参考 专题参考（1）

第八百一十一条

承运人应当在约定期限或者合理期限内将旅客、货物安全运输到约定地点。

法宝联想

法律法规 司法解释（2）规范性文件（1）

司法案例 司法案例（2923）案例报道（3）

法学期刊 法学期刊（5）

第八百一十二条

承运人应当按照约定的或者通常的运输路线将旅客、货物运输到约定地点。

法宝联想

法律法规 规范性文件（1）

司法案例 司法案例（619）

法学期刊 法学期刊（2）

专题参考 专题参考（3）

第八百一十三条

旅客、托运人或者收货人应当支付票款或者运输费用。承运人未按照约定路线或者通常路线运输增加票款或者运输费用的，旅客、托运人或者收货人可以拒绝支付增加部分的票款或者运输费用。

法宝联想

司法案例 司法案例（9772）

法学期刊 法学期刊（1）

第二节　客运合同

第八百一十四条

客运合同自承运人向旅客出具客票时成立，但是当事人另有约定或者另有交易习惯的除外。

法宝联想

司法案例　司法案例（471）案例报道（1）

法学期刊　法学期刊（7）

专题参考　专题参考（1）

第八百一十五条

旅客应当按照有效客票记载的时间、班次和座位号乘坐。旅客无票乘坐、超程乘坐、越级乘坐或者持不符合减价条件的优惠客票乘坐的，应当补交票款，承运人可以按照规定加收票款；旅客不支付票款的，承运人可以拒绝运输。

实名制客运合同的旅客丢失客票的，可以请求承运人挂失补办，承运人不得再次收取票款和其他不合理费用。

法宝联想

司法案例　司法案例（16）

法学期刊　法学期刊（4）

专题参考　专题参考（1）

第八百一十六条

旅客因自己的原因不能按照客票记载的时间乘坐的，应当在约定的期限内办理退票或者变更手续；逾期办理的，承运人可以不退票款，并不再承担运输义务。

法宝联想

法律法规　规范性文件（1）法律动态（2）

司法案例　司法案例（20）案例报道（1）

法学期刊　法学期刊（4）

第八百一十七条

旅客随身携带行李应当符合约定的限量和品类要求；超过限量或者违反品类要求携带行李的，应当办理托运手续。

法宝联想

司法案例　司法案例（4）

法学期刊　法学期刊（2）

第八百一十八条

旅客不得随身携带或者在行李中夹带易燃、易爆、有毒、有腐蚀性、有放射性以及可能危及运输工具上人身和财产安全的危险物品或者违禁物品。

旅客违反前款规定的，承运人可以将危险物品或者违禁物品卸下、销毁或者送交有关部门。旅客坚持携带或者夹带危险物品或者违禁物品的，承运人应当拒绝运输。

法宝联想

司法案例　司法案例（3）

法学期刊　法学期刊（3）

第八百一十九条

承运人应当严格履行安全运输义务，及时告知旅客安全运输应当注意的事项。旅客对承运人为安全运输所作的合理安排应当积极协助和配合。

法宝联想

司法案例 司法案例（222）

法学期刊 法学期刊（2）

第八百二十条

承运人应当按照有效客票记载的时间、班次和座位号运输旅客。承运人迟延运输或者有其他不能正常运输情形的，应当及时告知和提醒旅客，采取必要的安置措施，并根据旅客的要求安排改乘其他班次或者退票；由此造成旅客损失的，承运人应当承担赔偿责任，但是不可归责于承运人的除外。

法宝联想

司法案例 司法案例（92）案例报道（1）

法学期刊 法学期刊（1）

专题参考 专题参考（1）

第八百二十一条

承运人擅自降低服务标准的，应当根据旅客的请求退票或者减收票款；提高服务标准的，不得加收票款。

法宝联想

司法案例 司法案例（4）

法学期刊 法学期刊（1）

第八百二十二条

承运人在运输过程中，应当尽力救助患有急病、分娩、遇险的旅客。

法宝联想

法律法规 司法解释（2）规范性文件（1）法律动态（1）

司法案例 司法案例（68）案例报道（4）

法学期刊 法学期刊（3）

法宝律师 律所实务（1）

第八百二十三条

承运人应当对运输过程中旅客的伤亡承担赔偿责任；但是，伤亡是旅客自身健康原因造成的或者承运人证明伤亡是旅客故意、重大过失造成的除外。

前款规定适用于按照规定免票、持优待票或者经承运人许可搭乘的无票旅客。

法宝联想

法律法规　司法解释（2）规范性文件（1）法律动态（1）

司法案例　司法案例（2457）案例报道（3）

法学期刊　法学期刊（12）

法宝律师　律所实务（1）

专题参考　专题参考（2）

第八百二十四条

在运输过程中旅客随身携带物品毁损、灭失，承运人有过错的，应当承担赔偿责任。

旅客托运的行李毁损、灭失的，适用货物运输的有关规定。

法宝联想

司法案例　司法案例（64）

法学期刊　法学期刊（3）

专题参考　专题参考（1）

第三节　货运合同

第八百二十五条

托运人办理货物运输，应当向承运人准确表明收货人的姓名、名称或者凭指示的

收货人，货物的名称、性质、重量、数量，收货地点等有关货物运输的必要情况
。

因托运人申报不实或者遗漏重要情况，造成承运人损失的，托运人应当承担赔偿责任。

法宝联想

司法案例　司法案例（676）

法学期刊　法学期刊（3）

专题参考　专题参考（1）

第八百二十六条

货物运输需要办理审批、检验等手续的，托运人应当将办理完有关手续的文件提交承运人。

法宝联想

司法案例　司法案例（42）

法学期刊　法学期刊（5）

专题参考　专题参考（2）

第八百二十七条

托运人应当按照约定的方式包装货物。对包装方式没有约定或者约定不明确的，适用本法第六百一十九条的规定。

托运人违反前款规定的，承运人可以拒绝运输。

法宝联想

司法案例　司法案例（187）

法学期刊　法学期刊（2）

第八百二十八条

托运人托运易燃、易爆、有毒、有腐蚀性、有放射性等危险物品的，应当按照国家有关危险物品运输的规定对危险物品妥善包装，做出危险物品标志和标签，并将有关危险物品的名称、性质和防范措施的书面材料提交承运人。

托运人违反前款规定的，承运人可以拒绝运输，也可以采取相应措施以避免损失的发生，因此产生的费用由托运人负担。

法宝联想

司法案例　司法案例（82）案例报道（1）

法学期刊　法学期刊（2）

专题参考　专题参考（2）

第八百二十九条

在承运人将货物交付收货人之前，托运人可以要求承运人中止运输、返还货物、变更到达地或者将货物交给其他收货人，但是应当赔偿承运人因此受到的损失。

法宝联想

司法案例　司法案例（160）案例报道（1）

法学期刊　法学期刊（11）

专题参考　专题参考（1）

第八百三十条

货物运输到达后，承运人知道收货人的，应当及时通知收货人，收货人应当及时提货。收货人逾期提货的，应当向承运人支付保管费等费用。

法宝联想

司法案例　司法案例（220）

法学期刊　法学期刊（6）

第八百三十一条

收货人提货时应当按照约定的期限检验货物。对检验货物的期限没有约定或者约定不明确，依据本法第五百一十条的规定仍不能确定的，应当在合理期限内检验货物。收货人在约定的期限或者合理期限内对货物的数量、毁损等未提出异议的，视为承运人已经按照运输单证的记载交付的初步证据。

法宝联想

司法案例　司法案例（198）

法学期刊　法学期刊（9）

法宝律师　律所实务（1）

第八百三十二条

承运人对运输过程中货物的毁损、灭失承担赔偿责任。但是，承运人证明货物的毁损、灭失是因不可抗力、货物本身的自然性质或者合理损耗以及托运人、收货人的过错造成的，不承担赔偿责任。

法宝联想

法律法规　规范性文件（2）法律动态（1）

司法案例　司法案例（5095）案例报道（9）

法学期刊　法学期刊（15）

法宝律师　律所实务（4）

专题参考　专题参考（2）

第八百三十三条

货物的毁损、灭失的赔偿额，当事人有约定的，按照其约定；没有约定或者约定不明确，依据本法第五百一十条的规定仍不能确定的，按照交付或者应当交付时

货物到达地的市场价格计算。法律、行政法规对赔偿额的计算方法和赔偿限额另有规定的，依照其规定。

法宝联想

司法案例　司法案例（3229）案例报道（3）

法学期刊　法学期刊（2）

法宝律师　律所实务（2）

第八百三十四条

两个以上承运人以同一运输方式联运的，与托运人订立合同的承运人应当对全程运输承担责任；损失发生在某一运输区段的，与托运人订立合同的承运人和该区段的承运人承担连带责任。

法宝联想

司法案例　司法案例（227）案例报道（2）

法学期刊　法学期刊（5）

法宝律师　律所实务（1）

第八百三十五条

货物在运输过程中因不可抗力灭失，未收取运费的，承运人不得请求支付运费；已经收取运费的，托运人可以请求返还。法律另有规定的，依照其规定。

法宝联想

司法案例　司法案例（108）

法学期刊　法学期刊（7）

专题参考　专题参考（1）

第八百三十六条

托运人或者收货人不支付运费、保管费或者其他费用的，承运人对相应的运输货物享有留置权，但是当事人另有约定的除外。

法宝联想

司法案例　司法案例（423）案例报道（1）

法学期刊　法学期刊（7）

法宝律师　律所实务（1）

第八百三十七条

收货人不明或者收货人无正当理由拒绝受领货物的，承运人依法可以提存货物。

法宝联想

司法案例　司法案例（63）

法学期刊　法学期刊（5）

第四节　多式联运合同

第八百三十八条

多式联运经营人负责履行或者组织履行多式联运合同，对全程运输享有承运人的权利，承担承运人的义务。

法宝联想

司法案例　司法案例（39）

法学期刊　法学期刊（2）

第八百三十九条

多式联运经营人可以与参加多式联运的各区段承运人就多式联运合同的各区段运

输约定相互之间的责任；但是，该约定不影响多式联运经营人对全程运输承担的义务。

法宝联想

司法案例 司法案例（11）

法学期刊 法学期刊（5）

第八百四十条

多式联运经营人收到托运人交付的货物时，应当签发多式联运单据。按照托运人的要求，多式联运单据可以是可转让单据，也可以是不可转让单据。

法宝联想

司法案例 司法案例（7）

第八百四十一条

因托运人托运货物时的过错造成多式联运经营人损失的，即使托运人已经转让多式联运单据，托运人仍然应当承担赔偿责任。

法宝联想

司法案例 司法案例（5）

专题参考 专题参考（1）

第八百四十二条

货物的毁损、灭失发生于多式联运的某一运输区段的，多式联运经营人的赔偿责任和责任限额，适用调整该区段运输方式的有关法律规定；货物毁损、灭失发生的运输区段不能确定的，依照本章规定承担赔偿责任。

法宝联想

司法案例 司法案例（12）

法学期刊 法学期刊（3）

第二十章　技术合同

第一节　一般规定

第八百四十三条

技术合同是当事人就技术开发、转让、许可、咨询或者服务订立的确立相互之间权利和义务的合同。

法宝联想

法律法规　规范性文件（1）

司法案例　司法案例（180）

法学期刊　法学期刊（1）

第八百四十四条

订立技术合同，应当有利于知识产权的保护和科学技术的进步，促进科学技术成果的研发、转化、应用和推广。

法宝联想

法律法规　规范性文件（1）法律动态（1）

司法案例　司法案例（20）案例报道（2）

法学期刊　法学期刊（8）

第八百四十五条

技术合同的内容一般包括项目的名称，标的的内容、范围和要求，履行的计划、地点和方式，技术信息和资料的保密，技术成果的归属和收益的分配办法，验收标准和方法，名词和术语的解释等条款。

与履行合同有关的技术背景资料、可行性论证和技术评价报告、项目任务书和计

划书、技术标准、技术规范、原始设计和工艺文件，以及其他技术文档，按照当事人的约定可以作为合同的组成部分。

技术合同涉及专利的，应当注明发明创造的名称、专利申请人和专利权人、申请日期、申请号、专利号以及专利权的有效期限。

法宝联想

法律法规 规范性文件（1）

司法案例 司法案例（15）

法学期刊 法学期刊（2）

法宝律师 律所实务（1）

第八百四十六条

技术合同价款、报酬或者使用费的支付方式由当事人约定，可以采取一次总算、一次总付或者一次总算、分期支付，也可以采取提成支付或者提成支付附加预付入门费的方式。

约定提成支付的，可以按照产品价格、实施专利和使用技术秘密后新增的产值、利润或者产品销售额的一定比例提成，也可以按照约定的其他方式计算。提成支付的比例可以采取固定比例、逐年递增比例或者逐年递减比例。

约定提成支付的，当事人可以约定查阅有关会计账目的办法。

法宝联想

司法案例 司法案例（49）

法学期刊 法学期刊（7）

专题参考 专题参考（1）

第八百四十七条

职务技术成果的使用权、转让权属于法人或者非法人组织的，法人或者非法人组织可以就该项职务技术成果订立技术合同。法人或者非法人组织订立技术合同转让职务技术成果时，职务技术成果的完成人享有以同等条件优先受让的权利。

职务技术成果是执行法人或者非法人组织的工作任务，或者主要是利用法人或者非法人组织的物质技术条件所完成的技术成果。

法宝联想

法律法规　司法解释（2）规范性文件（2）

司法案例　司法案例（7）

法学期刊　法学期刊（14）

法宝律师　律所实务（2）

第八百四十八条

非职务技术成果的使用权、转让权属于完成技术成果的个人，完成技术成果的个人可以就该项非职务技术成果订立技术合同。

法宝联想

法律法规　规范性文件（1）

司法案例　司法案例（2）

法学期刊　法学期刊（2）

第八百四十九条

完成技术成果的个人享有在有关技术成果文件上写明自己是技术成果完成者的权利和取得荣誉证书、奖励的权利。

法宝联想

司法案例　司法案例（1）

法学期刊　法学期刊（2）

第八百五十条　非法垄断技术或者侵害他人技术成果的技术合同无效。

法宝联想

法律法规　司法解释（2）规范性文件（1）

司法案例　司法案例（8）案例报道（1）

法学期刊　法学期刊（13）

法宝律师　律所实务（3）

第二节　技术开发合同

第八百五十一条

技术开发合同是当事人之间就新技术、新产品、新工艺、新品种或者新材料及其系统的研究开发所订立的合同。

技术开发合同包括委托开发合同和合作开发合同。

技术开发合同应当采用书面形式。

当事人之间就具有实用价值的科技成果实施转化订立的合同，参照适用技术开发合同的有关规定。

法宝联想

法律法规　司法解释（2）

司法案例　司法案例（95）

法学期刊　法学期刊（6）

专题参考　专题参考（2）

第八百五十二条

委托开发合同的委托人应当按照约定支付研究开发经费和报酬，提供技术资料，提出研究开发要求，完成协作事项，接受研究开发成果。

法宝联想

司法案例 司法案例（74）

法学期刊 法学期刊（3）

第八百五十三条

委托开发合同的研究开发人应当按照约定制定和实施研究开发计划，合理使用研究开发经费，按期完成研究开发工作，交付研究开发成果，提供有关的技术资料和必要的技术指导，帮助委托人掌握研究开发成果。

法宝联想

司法案例 司法案例（58）

法学期刊 法学期刊（1）

专题参考 专题参考（1）

第八百五十四条

委托开发合同的当事人违反约定造成研究开发工作停滞、延误或者失败的，应当承担违约责任。

法宝联想

司法案例 司法案例（49）

第八百五十五条

合作开发合同的当事人应当按照约定进行投资，包括以技术进行投资，分工参与研究开发工作，协作配合研究开发工作。

法宝联想

法律法规 司法解释（2）

司法案例 司法案例（9）

第八百五十六条

合作开发合同的当事人违反约定造成研究开发工作停滞、延误或者失败的，应当承担违约责任。

法宝联想

司法案例 司法案例（14）

第八百五十七条

作为技术开发合同标的的技术已经由他人公开，致使技术开发合同的履行没有意义的，当事人可以解除合同。

法宝联想

司法案例 司法案例（8）

法学期刊 法学期刊（1）

第八百五十八条

技术开发合同履行过程中，因出现无法克服的技术困难，致使研究开发失败或者部分失败的，该风险由当事人约定；没有约定或者约定不明确，依据本法第五百一十条的规定仍不能确定的，风险由当事人合理分担。

当事人一方发现前款规定的可能致使研究开发失败或者部分失败的情形时，应当及时通知另一方并采取适当措施减少损失；没有及时通知并采取适当措施，致使损失扩大的，应当就扩大的损失承担责任。

法宝联想

法律法规 司法解释（1）

司法案例 司法案例（32）

法学期刊 法学期刊（4）

法宝律师 律所实务（1）

专题参考 专题参考（1）

第八百五十九条

委托开发完成的发明创造，除法律另有规定或者当事人另有约定外，申请专利的权利属于研究开发人。研究开发人取得专利权的，委托人可以依法实施该专利。

研究开发人转让专利申请权的，委托人享有以同等条件优先受让的权利。

法宝联想

法律法规 规范性文件（2）

司法案例 司法案例（7）

法学期刊 法学期刊（5）

法宝律师 律所实务（3）

第八百六十条

合作开发完成的发明创造，申请专利的权利属于合作开发的当事人共有；当事人一方转让其共有的专利申请权的，其他各方享有以同等条件优先受让的权利。但是，当事人另有约定的除外。

合作开发的当事人一方声明放弃其共有的专利申请权的，除当事人另有约定外，可以由另一方单独申请或者由其他各方共同申请。申请人取得专利权的，放弃专利申请权的一方可以免费实施该专利。

合作开发的当事人一方不同意申请专利的，另一方或者其他各方不得申请专利。

法宝联想

法律法规　规范性文件（1）

司法案例　司法案例（14）

法学期刊　法学期刊（7）

法宝律师　律所实务（1）

第八百六十一条

委托开发或者合作开发完成的技术秘密成果的使用权、转让权以及收益的分配办法，由当事人约定；没有约定或者约定不明确，依据本法第五百一十条的规定仍不能确定的，在没有相同技术方案被授予专利权前，当事人均有使用和转让的权利。但是，委托开发的研究开发人不得在向委托人交付研究开发成果之前，将研究开发成果转让给第三人。

法宝联想

法律法规　司法解释（2）

司法案例　司法案例（3）

法学期刊　法学期刊（3）

法宝律师　律所实务（1）

第三节　技术转让合同和技术许可合同

第八百六十二条

技术转让合同是合法拥有技术的权利人，将现有特定的专利、专利申请、技术秘密的相关权利让与他人所订立的合同。

技术许可合同是合法拥有技术的权利人，将现有特定的专利、技术秘密的相关权利许可他人实施、使用所订立的合同。

技术转让合同和技术许可合同中关于提供实施技术的专用设备、原材料或者提供有关的技术咨询、技术服务的约定，属于合同的组成部分。

法宝联想

法律法规　司法解释（2）规范性文件（1）合同范本（2）

司法案例　司法案例（21）

法学期刊　法学期刊（5）

法宝律师　律所实务（2）

第八百六十三条

技术转让合同包括专利权转让、专利申请权转让、技术秘密转让等合同。

技术许可合同包括专利实施许可、技术秘密使用许可等合同。

技术转让合同和技术许可合同应当采用书面形式。

法宝联想

法律法规　规范性文件（1）合同范本（1）

司法案例　司法案例（7）

法学期刊　法学期刊（5）

法宝律师　律所实务（1）

第八百六十四条

技术转让合同和技术许可合同可以约定实施专利或者使用技术秘密的范围，但是不得限制技术竞争和技术发展。

法宝联想

法律法规　司法解释（2）

司法案例　司法案例（3）

法学期刊　法学期刊（7）

第八百六十五条

专利实施许可合同仅在该专利权的存续期限内有效。专利权有效期限届满或者专利权被宣告无效的，专利权人不得就该专利与他人订立专利实施许可合同。

法宝联想

法律法规　规范性文件（1）合同范本（1）

司法案例　司法案例（4）

法学期刊　法学期刊（2）

第八百六十六条

专利实施许可合同的许可人应当按照约定许可被许可人实施专利，交付实施专利有关的技术资料，提供必要的技术指导。

法宝联想

司法案例　司法案例（12）

法学期刊　法学期刊（5）

第八百六十七条

专利实施许可合同的被许可人应当按照约定实施专利，不得许可约定以外的第三人实施该专利，并按照约定支付使用费。

法宝联想

司法案例　司法案例（14）

法学期刊　法学期刊（3）

法宝律师　律所实务（1）

第八百六十八条

技术秘密转让合同的让与人和技术秘密使用许可合同的许可人应当按照约定提供技术资料，进行技术指导，保证技术的实用性、可靠性，承担保密义务。

前款规定的保密义务，不限制许可人申请专利，但是当事人另有约定的除外。

法宝联想

司法案例　司法案例（15）

法学期刊　法学期刊（5）

第八百六十九条

技术秘密转让合同的受让人和技术秘密使用许可合同的被许可人应当按照约定使用技术，支付转让费、使用费，承担保密义务。

法宝联想

司法案例　司法案例（5）

法学期刊　法学期刊（2）

第八百七十条

技术转让合同的让与人和技术许可合同的许可人应当保证自己是所提供的技术的合法拥有者，并保证所提供的技术完整、无误、有效，能够达到约定的目标。

法宝联想

司法案例　司法案例（19）

法学期刊　法学期刊（3）

法宝律师　律所实务（1）

第八百七十一条

技术转让合同的受让人和技术许可合同的被许可人应当按照约定的范围和期限，对让与人、许可人提供的技术中尚未公开的秘密部分，承担保密义务。

法宝联想

司法案例　司法案例（1）

法学期刊　法学期刊（3）

法宝律师　律所实务（1）

第八百七十二条

许可人未按照约定许可技术的，应当返还部分或者全部使用费，并应当承担违约责任；实施专利或者使用技术秘密超越约定的范围的，违反约定擅自许可第三人实施该项专利或者使用该项技术秘密的，应当停止违约行为，承担违约责任；违反约定的保密义务的，应当承担违约责任。

让与人承担违约责任，参照适用前款规定。

法宝联想

司法案例　司法案例（13）

法学期刊　法学期刊（10）

专题参考　专题参考（1）

第八百七十三条

被许可人未按照约定支付使用费的，应当补交使用费并按照约定支付违约金；不补交使用费或者支付违约金的，应当停止实施专利或者使用技术秘密，交还技术资料，承担违约责任；实施专利或者使用技术秘密超越约定的范围的，未经许可人同意擅自许可第三人实施该专利或者使用该技术秘密的，应当停止违约行为，承担违约责任；违反约定的保密义务的，应当承担违约责任。

受让人承担违约责任，参照适用前款规定。

法宝联想

司法案例 司法案例（4）

法学期刊 法学期刊（10）

第八百七十四条

受让人或者被许可人按照约定实施专利、使用技术秘密侵害他人合法权益的，由让与人或者许可人承担责任，但是当事人另有约定的除外。

法宝联想

司法案例 司法案例（4）

法学期刊 法学期刊（3）

法宝律师 律所实务（2）

专题参考 专题参考（1）

第八百七十五条

当事人可以按照互利的原则，在合同中约定实施专利、使用技术秘密后续改进的技术成果的分享办法；没有约定或者约定不明确，依据本法第五百一十条的规定仍不能确定的，一方后续改进的技术成果，其他各方无权分享。

法宝联想

法学期刊 法学期刊（3）

法宝律师 律所实务（3）

第八百七十六条

集成电路布图设计专有权、植物新品种权、计算机软件著作权等其他知识产权的转让和许可，参照适用本节的有关规定。

法宝联想

司法案例　司法案例（13）

法学期刊　法学期刊（10）

第八百七十七条

法律、行政法规对技术进出口合同或者专利、专利申请合同另有规定的，依照其规定。

法宝联想

法律法规　规范性文件（1）合同范本（2）

司法案例　司法案例（3）

法学期刊　法学期刊（2）

第四节　技术咨询合同和技术服务合同

第八百七十八条

技术咨询合同是当事人一方以技术知识为对方就特定技术项目提供可行性论证、技术预测、专题技术调查、分析评价报告等所订立的合同。

技术服务合同是当事人一方以技术知识为对方解决特定技术问题所订立的合同，不包括承揽合同和建设工程合同。

法宝联想

法律法规　司法解释（2）

司法案例　司法案例（489）

法宝律师　律所实务（2）

第八百七十九条

技术咨询合同的委托人应当按照约定阐明咨询的问题，提供技术背景材料及有关技术资料，接受受托人的工作成果，支付报酬。

法宝联想

司法案例　司法案例（112）

法学期刊　法学期刊（1）

第八百八十条

技术咨询合同的受托人应当按照约定的期限完成咨询报告或者解答问题，提出的咨询报告应当达到约定的要求。

法宝联想

司法案例　司法案例（77）

第八百八十一条

技术咨询合同的委托人未按照约定提供必要的资料，影响工作进度和质量，不接受或者逾期接受工作成果的，支付的报酬不得追回，未支付的报酬应当支付。

技术咨询合同的受托人未按期提出咨询报告或者提出的咨询报告不符合约定的，应当承担减收或者免收报酬等违约责任。

技术咨询合同的委托人按照受托人符合约定要求的咨询报告和意见作出决策所造成的损失，由委托人承担，但是当事人另有约定的除外。

法宝联想

司法案例　司法案例（80）

法学期刊　法学期刊（4）

法宝律师 律所实务（1）

第八百八十二条

技术服务合同的委托人应当按照约定提供工作条件，完成配合事项，接受工作成果并支付报酬。

法宝联想

司法案例 司法案例（404）

法学期刊 法学期刊（3）

第八百八十三条

技术服务合同的受托人应当按照约定完成服务项目，解决技术问题，保证工作质量，并传授解决技术问题的知识。

法宝联想

司法案例 司法案例（216）

法学期刊 法学期刊（2）

第八百八十四条

技术服务合同的委托人不履行合同义务或者履行合同义务不符合约定，影响工作进度和质量，不接受或者逾期接受工作成果的，支付的报酬不得追回，未支付的报酬应当支付。

技术服务合同的受托人未按照约定完成服务工作的，应当承担免收报酬等违约责任。

法宝联想

司法案例 司法案例（316）

法学期刊 法学期刊（5）

第八百八十五条

技术咨询合同、技术服务合同履行过程中，受托人利用委托人提供的技术资料和工作条件完成的新的技术成果，属于受托人。委托人利用受托人的工作成果完成的新的技术成果，属于委托人。当事人另有约定的，按照其约定。

法宝联想

司法案例　司法案例（24）

法学期刊　法学期刊（1）

法宝律师　律所实务（1）

第八百八十六条

技术咨询合同和技术服务合同对受托人正常开展工作所需费用的负担没有约定或者约定不明确的，由受托人负担。

法宝联想

司法案例　司法案例（23）

法学期刊　法学期刊（2）

专题参考　专题参考（1）

第八百八十七条

法律、行政法规对技术中介合同、技术培训合同另有规定的，依照其规定。

法宝联想

法律法规　司法解释（2）

司法案例　司法案例（17）

法宝律师　律所实务（1）

第二十一章　保管合同

第八百八十八条

保管合同是保管人保管寄存人交付的保管物，并返还该物的合同。

寄存人到保管人处从事购物、就餐、住宿等活动，将物品存放在指定场所的，视为保管，但是当事人另有约定或者另有交易习惯的除外。

法宝联想

司法案例　司法案例（1196）案例报道（1）

法学期刊　法学期刊（14）

专题参考　专题参考（1）

第八百八十九条　寄存人应当按照约定向保管人支付保管费。

当事人对保管费没有约定或者约定不明确，依据本法第五百一十条的规定仍不能确定的，视为无偿保管。

法宝联想

司法案例　司法案例（311）

法学期刊　法学期刊（3）

第八百九十条

保管合同自保管物交付时成立，但是当事人另有约定的除外。

法宝联想

司法案例　司法案例（355）

法学期刊　法学期刊（13）

法宝律师　律所实务（1）

专题参考　专题参考（1）

第八百九十一条

寄存人向保管人交付保管物的，保管人应当出具保管凭证，但是另有交易习惯的除外。

法宝联想

司法案例 司法案例（42）

法学期刊 法学期刊（4）

第八百九十二条 保管人应当妥善保管保管物。

当事人可以约定保管场所或者方法。除紧急情况或者为维护寄存人利益外，不得擅自改变保管场所或者方法。

法宝联想

司法案例 司法案例（228）

法学期刊 法学期刊（6）

第八百九十三条

寄存人交付的保管物有瑕疵或者根据保管物的性质需要采取特殊保管措施的，寄存人应当将有关情况告知保管人。寄存人未告知，致使保管物受损失的，保管人不承担赔偿责任；保管人因此受损失的，除保管人知道或者应当知道且未采取补救措施外，寄存人应当承担赔偿责任。

法宝联想

司法案例 司法案例（28）

法学期刊 法学期刊（5）

专题参考 专题参考（1）

第八百九十四条

保管人不得将保管物转交第三人保管，但是当事人另有约定的除外。

保管人违反前款规定，将保管物转交第三人保管，造成保管物损失的，应当承担赔偿责任。

法宝联想

司法案例　司法案例（68）

法学期刊　法学期刊（4）

专题参考　专题参考（3）

第八百九十五条

保管人不得使用或者许可第三人使用保管物，但是当事人另有约定的除外。

法宝联想

司法案例　司法案例（105）

法学期刊　法学期刊（2）

第八百九十六条

第三人对保管物主张权利的，除依法对保管物采取保全或者执行措施外，保管人应当履行向寄存人返还保管物的义务。

第三人对保管人提起诉讼或者对保管物申请扣押的，保管人应当及时通知寄存人。

法宝联想

司法案例　司法案例（41）

第八百九十七条

保管期内，因保管人保管不善造成保管物毁损、灭失的，保管人应当承担赔偿责任。但是，无偿保管人证明自己没有故意或者重大过失的，不承担赔偿责任。

法宝联想

法律法规　法律动态（1）

司法案例　司法案例（566）案例报道（2）

法学期刊　法学期刊（18）

法宝律师　律所实务（1）

专题参考　专题参考（1）

第八百九十八条

寄存人寄存货币、有价证券或者其他贵重物品的，应当向保管人声明，由保管人验收或者封存；寄存人未声明的，该物品毁损、灭失后，保管人可以按照一般物品予以赔偿。

法宝联想

司法案例　司法案例（14）

法学期刊　法学期刊（3）

第八百九十九条　寄存人可以随时领取保管物。

当事人对保管期限没有约定或者约定不明确的，保管人可以随时请求寄存人领取保管物；约定保管期限的，保管人无特别事由，不得请求寄存人提前领取保管物。

法宝联想

司法案例　司法案例（531）

法学期刊　法学期刊（13）

专题参考　专题参考（1）

第九百条

保管期限届满或者寄存人提前领取保管物的，保管人应当将原物及其孳息归还寄存人。

法宝联想

司法案例　司法案例（205）

法学期刊　法学期刊（2）

第九百零一条

保管人保管货币的，可以返还相同种类、数量的货币；保管其他可替代物的，可以按照约定返还相同种类、品质、数量的物品。

法宝联想

司法案例　司法案例（229）

法学期刊　法学期刊（7）

第九百零二条

有偿的保管合同，寄存人应当按照约定的期限向保管人支付保管费。

当事人对支付期限没有约定或者约定不明确，依据本法第五百一十条的规定仍不能确定的，应当在领取保管物的同时支付。

法宝联想

司法案例　司法案例（82）

法学期刊　法学期刊（1）

专题参考　专题参考（1）

第九百零三条

寄存人未按照约定支付保管费或者其他费用的，保管人对保管物享有留置权，但是当事人另有约定的除外。

法宝联想

司法案例 司法案例（77）

法学期刊 法学期刊（3）

第二十二章　仓储合同

第九百零四条

仓储合同是保管人储存存货人交付的仓储物，存货人支付仓储费的合同。

法宝联想

司法案例 司法案例（481）

法学期刊 法学期刊（2）

法宝律师 律所实务（2）

第九百零五条　仓储合同自保管人和存货人意思表示一致时成立。

法宝联想

司法案例 司法案例（172）

法学期刊 法学期刊（2）

法宝律师 律所实务（1）

第九百零六条

储存易燃、易爆、有毒、有腐蚀性、有放射性等危险物品或者易变质物品的，存货人应当说明该物品的性质，提供有关资料。

存货人违反前款规定的，保管人可以拒收仓储物，也可以采取相应措施以避免损失的发生，因此产生的费用由存货人负担。

保管人储存易燃、易爆、有毒、有腐蚀性、有放射性等危险物品的，应当具备相应的保管条件。

法宝联想

司法案例　司法案例（25）

法学期刊　法学期刊（2）

专题参考　专题参考（1）

第九百零七条

保管人应当按照约定对入库仓储物进行验收。保管人验收时发现入库仓储物与约定不符合的，应当及时通知存货人。保管人验收后，发生仓储物的品种、数量、质量不符合约定的，保管人应当承担赔偿责任。

法宝联想

司法案例　司法案例（114）

法学期刊　法学期刊（1）

第九百零八条

存货人交付仓储物的，保管人应当出具仓单、入库单等凭证。

法宝联想

司法案例　司法案例（46）

法学期刊　法学期刊（2）

法宝律师　律所实务（3）

专题参考　专题参考（2）

第九百零九条　保管人应当在仓单上签名或者盖章。仓单包括下列事项：

（一）存货人的姓名或者名称和住所；

（二）仓储物的品种、数量、质量、包装及其件数和标记；

（三）仓储物的损耗标准；

（四）储存场所；

（五）储存期限；

（六）仓储费；

（七）仓储物已经办理保险的，其保险金额、期间以及保险人的名称；

（八）填发人、填发地和填发日期。

法宝联想

司法案例　司法案例（94）

法学期刊　法学期刊（2）

法宝律师　律所实务（1）

专题参考　专题参考（1）

第九百一十条

仓单是提取仓储物的凭证。存货人或者仓单持有人在仓单上背书并经保管人签名或者盖章的，可以转让提取仓储物的权利。

法宝联想

司法案例　司法案例（38）

法学期刊　法学期刊（3）

法宝律师　律所实务（2）

第九百一十一条

保管人根据存货人或者仓单持有人的要求，应当同意其检查仓储物或者提取样品。

法宝联想

司法案例　司法案例（8）

第九百一十二条

保管人发现入库仓储物有变质或者其他损坏的，应当及时通知存货人或者仓单持有人。

法宝联想

司法案例　司法案例（49）

第九百一十三条

保管人发现入库仓储物有变质或者其他损坏，危及其他仓储物的安全和正常保管的，应当催告存货人或者仓单持有人作出必要的处置。因情况紧急，保管人可以作出必要的处置；但是，事后应当将该情况及时通知存货人或者仓单持有人。

法宝联想

司法案例　司法案例（31）

法学期刊　法学期刊（2）

第九百一十四条

当事人对储存期限没有约定或者约定不明确的，存货人或者仓单持有人可以随时提取仓储物，保管人也可以随时请求存货人或者仓单持有人提取仓储物，但是应当给予必要的准备时间。

法宝联想

司法案例　司法案例（84）

法学期刊　法学期刊（2）

第九百一十五条

储存期限届满，存货人或者仓单持有人应当凭仓单、入库单等提取仓储物。存货人或者仓单持有人逾期提取的，应当加收仓储费；提前提取的，不减收仓储费。

法宝联想

司法案例　司法案例（73）

法学期刊　法学期刊（2）

第九百一十六条

储存期限届满，存货人或者仓单持有人不提取仓储物的，保管人可以催告其在合理期限内提取；逾期不提取的，保管人可以提存仓储物。

法宝联想

司法案例　司法案例（45）

法学期刊　法学期刊（4）

法宝律师　律所实务（1）

第九百一十七条

储存期内，因保管不善造成仓储物毁损、灭失的，保管人应当承担赔偿责任。因仓储物本身的自然性质、包装不符合约定或者超过有效储存期造成仓储物变质、损坏的，保管人不承担赔偿责任。

法宝联想

司法案例　司法案例（301）

法学期刊　法学期刊（2）

专题参考　专题参考（2）

第九百一十八条 本章没有规定的，适用保管合同的有关规定。

法宝联想

司法案例 司法案例（74）

法学期刊 法学期刊（5）

第二十三章 委托合同

第九百一十九条

委托合同是委托人和受托人约定，由受托人处理委托人事务的合同。

法宝联想

司法案例 司法案例（19438）案例报道（1）仲裁案例（5）

法学期刊 法学期刊（22）

法宝律师 律所实务（7）

第九百二十条

委托人可以特别委托受托人处理一项或者数项事务，也可以概括委托受托人处理一切事务。

法宝联想

司法案例 司法案例（1137）

法学期刊 法学期刊（5）

第九百二十一条

委托人应当预付处理委托事务的费用。受托人为处理委托事务垫付的必要费用，委托人应当偿还该费用并支付利息。

法宝联想

司法案例 司法案例（3427）仲裁案例（1）

法学期刊 法学期刊（27）

法宝律师　律所实务（1）

专题参考　专题参考（3）

第九百二十二条

受托人应当按照委托人的指示处理委托事务。需要变更委托人指示的，应当经委托人同意；因情况紧急，难以和委托人取得联系的，受托人应当妥善处理委托事务，但是事后应当将该情况及时报告委托人。

法宝联想

司法案例　司法案例（2064）

法学期刊　法学期刊（15）

法宝律师　律所实务（3）

第九百二十三条

受托人应当亲自处理委托事务。经委托人同意，受托人可以转委托。转委托经同意或者追认的，委托人可以就委托事务直接指示转委托的第三人，受托人仅就第三人的选任及其对第三人的指示承担责任。转委托未经同意或者追认的，受托人应当对转委托的第三人的行为承担责任；但是，在紧急情况下受托人为了维护委托人的利益需要转委托第三人的除外。

法宝联想

司法案例　司法案例（824）

法学期刊　法学期刊（22）

第九百二十四条

受托人应当按照委托人的要求，报告委托事务的处理情况。委托合同终止时，受托人应当报告委托事务的结果。

法宝联想

司法案例　司法案例（1086）

法学期刊　法学期刊（9）

专题参考　专题参考（3）

第九百二十五条

受托人以自己的名义，在委托人的授权范围内与第三人订立的合同，第三人在订立合同时知道受托人与委托人之间的代理关系的，该合同直接约束委托人和第三人；但是，有确切证据证明该合同只约束受托人和第三人的除外。

法宝联想

法律法规　规范性文件（1）白皮书（2）法律动态（1）

司法案例　司法案例（5865）案例报道（1）仲裁案例（2）

法学期刊　法学期刊（55）

法宝律师　律所实务（18）

专题参考　专题参考（6）

第九百二十六条

受托人以自己的名义与第三人订立合同时，第三人不知道受托人与委托人之间的代理关系的，受托人因第三人的原因对委托人不履行义务，受托人应当向委托人披露第三人，委托人因此可以行使受托人对第三人的权利。但是，第三人与受托人订立合同时如果知道该委托人就不会订立合同的除外。

受托人因委托人的原因对第三人不履行义务，受托人应当向第三人披露委托人，第三人因此可以选择受托人或者委托人作为相对人主张其权利，但是第三人不得变更选定的相对人。

委托人行使受托人对第三人的权利的，第三人可以向委托人主张其对受托人的抗辩。第三人选定委托人作为其相对人的，委托人可以向第三人主张其对受托人的抗辩以及受托人对第三人的抗辩。

法宝联想

法律法规　规范性文件（2）白皮书（2）法律动态（1）

司法案例　司法案例（4436）案例报道（2）仲裁案例（1）

法学期刊　法学期刊（39）

法宝律师　律所实务（12）

专题参考　专题参考（5）

第九百二十七条　受托人处理委托事务取得的财产，应当转交给委托人。

法宝联想

司法案例　司法案例（3158）

法学期刊　法学期刊（22）

法宝律师　律所实务（5）

专题参考　专题参考（1）

第九百二十八条

受托人完成委托事务的，委托人应当按照约定向其支付报酬。

因不可归责于受托人的事由，委托合同解除或者委托事务不能完成的，委托人应当向受托人支付相应的报酬。当事人另有约定的，按照其约定。

法宝联想

司法案例　司法案例（6403）仲裁案例（1）

法学期刊　法学期刊（14）

法宝律师　律所实务（1）

专题参考　专题参考（1）

第九百二十九条

有偿的委托合同，因受托人的过错造成委托人损失的，委托人可以请求赔偿损失。无偿的委托合同，因受托人的故意或者重大过失造成委托人损失的，委托人可以请求赔偿损失。

受托人超越权限造成委托人损失的，应当赔偿损失。

法宝联想

法律法规　司法解释（1）法规解读（1）法律动态（1）

司法案例　司法案例（4547）案例报道（1）仲裁案例（1）

法学期刊　法学期刊（57）

法宝律师　律所实务（9）

专题参考　专题参考（1）

第九百三十条

受托人处理委托事务时，因不可归责于自己的事由受到损失的，可以向委托人请求赔偿损失。

法宝联想

司法案例　司法案例（611）

法学期刊　法学期刊（15）

法宝律师　律所实务（1）

专题参考　专题参考（1）

第九百三十一条

委托人经受托人同意，可以在受托人之外委托第三人处理委托事务。因此造成受托人损失的，受托人可以向委托人请求赔偿损失。

法宝联想

司法案例　司法案例（50）

法学期刊　法学期刊（2）

第九百三十二条

两个以上的受托人共同处理委托事务的，对委托人承担连带责任。

法宝联想

司法案例　司法案例（309）

法学期刊　法学期刊（9）

法宝律师　律所实务（1）

第九百三十三条

委托人或者受托人可以随时解除委托合同。因解除合同造成对方损失的，除不可归责于该当事人的事由外，无偿委托合同的解除方应当赔偿因解除时间不当造成的直接损失，有偿委托合同的解除方应当赔偿对方的直接损失和合同履行后可以获得的利益。

法宝联想

法律法规　规范性文件（1）

司法案例　司法案例（5741）案例报道（3）仲裁案例（6）

法学期刊　法学期刊（90）

法宝律师　律所实务（22）

专题参考　专题参考（10）

第九百三十四条

委托人死亡、终止或者受托人死亡、丧失民事行为能力、终止的，委托合同终止；但是，当事人另有约定或者根据委托事务的性质不宜终止的除外。

法宝联想

司法案例　司法案例（174）案例报道（1）

法学期刊　法学期刊（4）

专题参考　专题参考（1）

第九百三十五条

因委托人死亡或者被宣告破产、解散，致使委托合同终止将损害委托人利益的，在委托人的继承人、遗产管理人或者清算人承受委托事务之前，受托人应当继续处理委托事务。

法宝联想

司法案例　司法案例（26）

法学期刊　法学期刊（4）

第九百三十六条

因受托人死亡、丧失民事行为能力或者被宣告破产、解散，致使委托合同终止的，受托人的继承人、遗产管理人、法定代理人或者清算人应当及时通知委托人。因委托合同终止将损害委托人利益的，在委托人作出善后处理之前，受托人的继承人、遗产管理人、法定代理人或者清算人应当采取必要措施。

法宝联想

司法案例　司法案例（63）

法学期刊　法学期刊（2）

第二十四章　物业服务合同

第九百三十七条

物业服务合同是物业服务人在物业服务区域内，为业主提供建筑物及其附属设施

的维修养护、环境卫生和相关秩序的管理维护等物业服务，业主支付物业费的合同。

物业服务人包括物业服务企业和其他管理人。

法宝联想

法律法规　规范性文件（4）法律动态（1）

司法案例　司法案例（76946）案例报道（1）仲裁案例（2）

法学期刊　法学期刊（9）

法宝律师　律所实务（1）

专题参考　专题参考（2）

第九百三十八条

物业服务合同的内容一般包括服务事项、服务质量、服务费用的标准和收取办法、维修资金的使用、服务用房的管理和使用、服务期限、服务交接等条款。

物业服务人公开作出的有利于业主的服务承诺，为物业服务合同的组成部分。

物业服务合同应当采用书面形式。

法宝联想

法律法规　规范性文件（3）法律动态（1）

司法案例　司法案例（6888）案例报道（1）

法学期刊　法学期刊（7）

法宝律师　律所实务（1）

专题参考　专题参考（1）

第九百三十九条

建设单位依法与物业服务人订立的前期物业服务合同，以及业主委员会与业主大会依法选聘的物业服务人订立的物业服务合同，对业主具有法律约束力。

法宝联想

法律法规　规范性文件（7）法律动态（1）

司法案例　司法案例（105626）案例报道（7）仲裁案例（4）

法学期刊　法学期刊（8）

专题参考　专题参考（1）

第九百四十条

建设单位依法与物业服务人订立的前期物业服务合同约定的服务期限届满前，业主委员会或者业主与新物业服务人订立的物业服务合同生效的，前期物业服务合同终止。

法宝联想

法律法规　规范性文件（3）法律动态（1）

司法案例　司法案例（4316）案例报道（1）

法学期刊　法学期刊（4）

第九百四十一条

物业服务人将物业服务区域内的部分专项服务事项委托给专业性服务组织或者其他第三人的，应当就该部分专项服务事项向业主负责。

物业服务人不得将其应当提供的全部物业服务转委托给第三人，或者将全部物业服务支解后分别转委托给第三人。

法宝联想

法律法规　规范性文件（1）

司法案例　司法案例（746）

法学期刊　法学期刊（5）

专题参考　专题参考（2）

第九百四十二条

物业服务人应当按照约定和物业的使用性质，妥善维修、养护、清洁、绿化和经营管理物业服务区域内的业主共有部分，维护物业服务区域内的基本秩序，采取合理措施保护业主的人身、财产安全。

对物业服务区域内违反有关治安、环保、消防等法律法规的行为，物业服务人应当及时采取合理措施制止、向有关行政主管部门报告并协助处理。

法宝联想

法律法规　规范性文件（7）法律动态（1）

司法案例　司法案例（20331）案例报道（11）仲裁案例（3）

法学期刊　法学期刊（20）

法宝律师　律所实务（1）

专题参考　专题参考（1）

第九百四十三条

物业服务人应当定期将服务的事项、负责人员、质量要求、收费项目、收费标准、履行情况，以及维修资金使用情况、业主共有部分的经营与收益情况等以合理方式向业主公开并向业主大会、业主委员会报告。

法宝联想

法律法规　规范性文件（2）

司法案例　司法案例（4599）案例报道（2）

法学期刊　法学期刊（4）

法宝律师　律所实务（1）

第九百四十四条

业主应当按照约定向物业服务人支付物业费。物业服务人已经按照约定和有关规

定提供服务的，业主不得以未接受或者无需接受相关物业服务为由拒绝支付物业费。

业主违反约定逾期不支付物业费的，物业服务人可以催告其在合理期限内支付；合理期限届满仍不支付的，物业服务人可以提起诉讼或者申请仲裁。

物业服务人不得采取停止供电、供水、供热、供燃气等方式催交物业费。

法宝联想
法律法规　规范性文件（14）草案（1）法律动态（2）
司法案例　司法案例（162668）案例报道（9）仲裁案例（5）
法学期刊　法学期刊（9）
专题参考　专题参考（2）

第九百四十五条

业主装饰装修房屋的，应当事先告知物业服务人，遵守物业服务人提示的合理注意事项，并配合其进行必要的现场检查。

业主转让、出租物业专有部分、设立居住权或者依法改变共有部分用途的，应当及时将相关情况告知物业服务人。

法宝联想
司法案例　司法案例（444）
法学期刊　法学期刊（3）

第九百四十六条

业主依照法定程序共同决定解聘物业服务人的，可以解除物业服务合同。决定解

聘的，应当提前六十日书面通知物业服务人，但是合同对通知期限另有约定的除外。

依据前款规定解除合同造成物业服务人损失的，除不可归责于业主的事由外，业主应当赔偿损失。

法宝联想

法律法规　规范性文件（3）草案（1）法律动态（1）

司法案例　司法案例（730）案例报道（1）仲裁案例（2）

法学期刊　法学期刊（13）

法宝律师　律所实务（3）

第九百四十七条

物业服务期限届满前，业主依法共同决定续聘的，应当与原物业服务人在合同期限届满前续订物业服务合同。

物业服务期限届满前，物业服务人不同意续聘的，应当在合同期限届满前九十日书面通知业主或者业主委员会，但是合同对通知期限另有约定的除外。

法宝联想

法律法规　规范性文件（1）

司法案例　司法案例（310）

法学期刊　法学期刊（1）

第九百四十八条

物业服务期限届满后，业主没有依法作出续聘或者另聘物业服务人的决定，物业

服务人继续提供物业服务的，原物业服务合同继续有效，但是服务期限为不定期。

当事人可以随时解除不定期物业服务合同，但是应当提前六十日书面通知对方。

法宝联想

法律法规　规范性文件（5）草案（1）法律动态（1）

司法案例　司法案例（13172）案例报道（2）仲裁案例（1）

法学期刊　法学期刊（8）

第九百四十九条

物业服务合同终止的，原物业服务人应当在约定期限或者合理期限内退出物业服务区域，将物业服务用房、相关设施、物业服务所必需的相关资料等交还给业主委员会、决定自行管理的业主或者其指定的人，配合新物业服务人做好交接工作，并如实告知物业的使用和管理状况。

原物业服务人违反前款规定的，不得请求业主支付物业服务合同终止后的物业费；造成业主损失的，应当赔偿损失。

法宝联想

法律法规　规范性文件（3）法律动态（1）

司法案例　司法案例（1485）案例报道（5）

法学期刊　法学期刊（9）

法宝律师　律所实务（1）

专题参考　专题参考（1）

第九百五十条

物业服务合同终止后，在业主或者业主大会选聘的新物业服务人或者决定自行管理的业主接管之前，原物业服务人应当继续处理物业服务事项，并可以请求业主支付该期间的物业费。

法宝联想

法律法规　规范性文件（2）草案（1）法律动态（1）

司法案例　司法案例（3783）案例报道（1）

法学期刊　法学期刊（6）

法宝律师　律所实务（1）

专题参考　专题参考（1）

第二十五章　行纪合同

第九百五十一条

行纪合同是行纪人以自己的名义为委托人从事贸易活动，委托人支付报酬的合同。

法宝联想

司法案例　司法案例（204）

法学期刊　法学期刊（7）

专题参考　专题参考（1）

第九百五十二条

行纪人处理委托事务支出的费用，由行纪人负担，但是当事人另有约定的除外。

法宝联想

司法案例　司法案例（54）

法学期刊　法学期刊（4）

第九百五十三条　行纪人占有委托物的，应当妥善保管委托物。

法宝联想

　　司法案例　司法案例（15）

　　法学期刊　法学期刊（2）

　　专题参考　专题参考（2）

第九百五十四条

委托物交付给行纪人时有瑕疵或者容易腐烂、变质的，经委托人同意，行纪人可以处分该物；不能与委托人及时取得联系的，行纪人可以合理处分。

法宝联想

　　司法案例　司法案例（10）

　　法学期刊　法学期刊（3）

　　专题参考　专题参考（1）

第九百五十五条

行纪人低于委托人指定的价格卖出或者高于委托人指定的价格买入的，应当经委托人同意；未经委托人同意，行纪人补偿其差额的，该买卖对委托人发生效力。

行纪人高于委托人指定的价格卖出或者低于委托人指定的价格买入的，可以按照约定增加报酬；没有约定或者约定不明确，依据本法第五百一十条的规定仍不能确定的，该利益属于委托人。

委托人对价格有特别指示的，行纪人不得违背该指示卖出或者买入。

法宝联想

　　司法案例　司法案例（38）

　　法学期刊　法学期刊（4）

　　法宝律师　律所实务（1）

第九百五十六条

行纪人卖出或者买入具有市场定价的商品，除委托人有相反的意思表示外，行纪人自己可以作为买受人或者出卖人。

行纪人有前款规定情形的，仍然可以请求委托人支付报酬。

法宝联想

司法案例　司法案例（30）

法学期刊　法学期刊（2）

法宝律师　律所实务（1）

第九百五十七条

行纪人按照约定买入委托物，委托人应当及时受领。经行纪人催告，委托人无正当理由拒绝受领的，行纪人依法可以提存委托物。

委托物不能卖出或者委托人撤回出卖，经行纪人催告，委托人不取回或者不处分该物的，行纪人依法可以提存委托物。

法宝联想

司法案例　司法案例（33）

法学期刊　法学期刊（4）

第九百五十八条

行纪人与第三人订立合同的，行纪人对该合同直接享有权利、承担义务。

第三人不履行义务致使委托人受到损害的，行纪人应当承担赔偿责任，但是行纪人与委托人另有约定的除外。

法宝联想

司法案例　司法案例（164）

法学期刊　法学期刊（6）

第九百五十九条

行纪人完成或者部分完成委托事务的，委托人应当向其支付相应的报酬。委托人逾期不支付报酬的，行纪人对委托物享有留置权，但是当事人另有约定的除外。

法宝联想

司法案例　司法案例（47）

法学期刊　法学期刊（6）

第九百六十条　本章没有规定的，参照适用委托合同的有关规定。

法宝联想

司法案例　司法案例（184）

法学期刊　法学期刊（13）

法宝律师　律所实务（1）

第二十六章　中介合同

第九百六十一条

中介合同是中介人向委托人报告订立合同的机会或者提供订立合同的媒介服务，委托人支付报酬的合同。

法宝联想

司法案例　司法案例（11582）案例报道（5）仲裁案例（5）

法学期刊　法学期刊（18）

法宝律师　律所实务（6）

专题参考　专题参考（1）

第九百六十二条　中介人应当就有关订立合同的事项向委托人如实报告。

中介人故意隐瞒与订立合同有关的重要事实或者提供虚假情况，损害委托人利益的，不得请求支付报酬并应当承担赔偿责任。

法宝联想

法律法规　规范性文件（1）

司法案例　司法案例（2719）案例报道（3）仲裁案例（1）

法学期刊　法学期刊（10）

法宝律师　律所实务（2）

专题参考　专题参考（2）

第九百六十三条

中介人促成合同成立的，委托人应当按照约定支付报酬。对中介人的报酬没有约定或者约定不明确，依据本法第五百一十条的规定仍不能确定的，根据中介人的劳务合理确定。因中介人提供订立合同的媒介服务而促成合同成立的，由该合同的当事人平均负担中介人的报酬。

中介人促成合同成立的，中介活动的费用，由中介人负担。

法宝联想

法律法规　规范性文件（1）

司法案例　司法案例（7165）案例报道（2）仲裁案例（1）

法学期刊　法学期刊（11）

法宝律师　律所实务（1）

第九百六十四条

中介人未促成合同成立的，不得请求支付报酬；但是，可以按照约定请求委托人支付从事中介活动支出的必要费用。

法宝联想

司法案例　司法案例（3714）案例报道（1）仲裁案例（1）

法学期刊　法学期刊（6）

法宝律师　律所实务（1）

专题参考　专题参考（2）

第九百六十五条

委托人在接受中介人的服务后，利用中介人提供的交易机会或者媒介服务，绕开中介人直接订立合同的，应当向中介人支付报酬。

法宝联想

法律法规　规范性文件（2）

司法案例　司法案例（1353）案例报道（9）仲裁案例（1）

法学期刊　法学期刊（7）

法宝律师　律所实务（1）

专题参考　专题参考（9）

第九百六十六条　本章没有规定的，参照适用委托合同的有关规定。

法宝联想

司法案例　司法案例（469）

法学期刊　法学期刊（15）

法宝律师　律所实务（1）

第二十七章　合伙合同

第九百六十七条

合伙合同是两个以上合伙人为了共同的事业目的，订立的共享利益、共担风险的协议。

法宝联想

司法案例 司法案例（27930）案例报道（2）仲裁案例（5）

法学期刊 法学期刊（14）

法宝律师 律所实务（2）

专题参考 专题参考（3）

第九百六十八条

合伙人应当按照约定的出资方式、数额和缴付期限，履行出资义务。

法宝联想

司法案例 司法案例（3913）

法学期刊 法学期刊（5）

专题参考 专题参考（1）

第九百六十九条

合伙人的出资、因合伙事务依法取得的收益和其他财产，属于合伙财产。

合伙合同终止前，合伙人不得请求分割合伙财产。

法宝联想

法律法规 规范性文件（1）

司法案例 司法案例（9230）案例报道（2）仲裁案例（2）

法学期刊 法学期刊（15）

专题参考 专题参考（1）

第九百七十条

合伙人就合伙事务作出决定的，除合伙合同另有约定外，应当经全体合伙人一致同意。

合伙事务由全体合伙人共同执行。按照合伙合同的约定或者全体合伙人的决定，

可以委托一个或者数个合伙人执行合伙事务；其他合伙人不再执行合伙事务，但是有权监督执行情况。

合伙人分别执行合伙事务的，执行事务合伙人可以对其他合伙人执行的事务提出异议；提出异议后，其他合伙人应当暂停该项事务的执行。

法宝联想

司法案例　司法案例（3582）

法学期刊　法学期刊（14）

法宝律师　律所实务（1）

专题参考　专题参考（1）

第九百七十一条

合伙人不得因执行合伙事务而请求支付报酬，但是合伙合同另有约定的除外。

法宝联想

司法案例　司法案例（810）

法学期刊　法学期刊（2）

第九百七十二条

合伙的利润分配和亏损分担，按照合伙合同的约定办理；合伙合同没有约定或者约定不明确的，由合伙人协商决定；协商不成的，由合伙人按照实缴出资比例分配、分担；无法确定出资比例的，由合伙人平均分配、分担。

法宝联想

司法案例　司法案例（17964）仲裁案例（1）

法学期刊　法学期刊（11）

专题参考　专题参考（3）

第九百七十三条

合伙人对合伙债务承担连带责任。清偿合伙债务超过自己应当承担份额的合伙人，有权向其他合伙人追偿。

法宝联想

法律法规 规范性文件（1）

司法案例 司法案例（12316）案例报道（1）

法学期刊 法学期刊（20）

法宝律师 律所实务（1）

专题参考 专题参考（1）

第九百七十四条

除合伙合同另有约定外，合伙人向合伙人以外的人转让其全部或者部分财产份额的，须经其他合伙人一致同意。

法宝联想

司法案例 司法案例（812）仲裁案例（1）

法学期刊 法学期刊（11）

专题参考 专题参考（1）

第九百七十五条

合伙人的债权人不得代位行使合伙人依照本章规定和合伙合同享有的权利，但是合伙人享有的利益分配请求权除外。

法宝联想

司法案例 司法案例（68）

法学期刊 法学期刊（8）

第九百七十六条

合伙人对合伙期限没有约定或者约定不明确，依据本法第五百一十条的规定仍不能确定的，视为不定期合伙。

合伙期限届满，合伙人继续执行合伙事务，其他合伙人没有提出异议的，原合伙合同继续有效，但是合伙期限为不定期。

合伙人可以随时解除不定期合伙合同，但是应当在合理期限之前通知其他合伙人。

法宝联想

司法案例　司法案例（3884）仲裁案例（2）

法学期刊　法学期刊（9）

法宝律师　律所实务（1）

专题参考　专题参考（1）

第九百七十七条

合伙人死亡、丧失民事行为能力或者终止的，合伙合同终止；但是，合伙合同另有约定或者根据合伙事务的性质不宜终止的除外。

法宝联想

司法案例　司法案例（686）

法学期刊　法学期刊（9）

专题参考　专题参考（1）

第九百七十八条

合伙合同终止后，合伙财产在支付因终止而产生的费用以及清偿合伙债务后有剩余的，依据本法第九百七十二条的规定进行分配。

法宝联想

司法案例　司法案例（7805）仲裁案例（1）

法学期刊　法学期刊（11）

专题参考　专题参考（5）

第三分编　准合同

第二十八章　无因管理

第九百七十九条

管理人没有法定的或者约定的义务，为避免他人利益受损失而管理他人事务的，可以请求受益人偿还因管理事务而支出的必要费用；管理人因管理事务受到损失的，可以请求受益人给予适当补偿。

管理事务不符合受益人真实意思的，管理人不享有前款规定的权利；但是，受益人的真实意思违反法律或者违背公序良俗的除外。

法宝联想

司法案例　司法案例（1592）案例报道（1）

法学期刊　法学期刊（72）

法宝律师　律所实务（1）

专题参考　专题参考（4）

第九百八十条

管理人管理事务不属于前条规定的情形，但是受益人享有管理利益的，受益人应当在其获得的利益范围内向管理人承担前条第一款规定的义务。

法宝联想

司法案例　司法案例（216）

法学期刊　法学期刊（33）

法宝律师　律所实务（1）

专题参考　专题参考（3）

第九百八十一条

管理人管理他人事务，应当采取有利于受益人的方法。中断管理对受益人不利的，无正当理由不得中断。

法宝联想

司法案例　司法案例（48）

法学期刊　法学期刊（15）

第九百八十二条

管理人管理他人事务，能够通知受益人的，应当及时通知受益人。管理的事务不需要紧急处理的，应当等待受益人的指示。

法宝联想

司法案例　司法案例（99）

法学期刊　法学期刊（4）

第九百八十三条

管理结束后，管理人应当向受益人报告管理事务的情况。管理人管理事务取得的财产，应当及时转交给受益人。

法宝联想

法律法规　规范性文件（1）

司法案例　司法案例（77）

法学期刊　法学期刊（7）

专题参考　专题参考（1）

第九百八十四条

管理人管理事务经受益人事后追认的，从管理事务开始时起，适用委托合同的有关规定，但是管理人另有意思表示的除外。

法宝联想

司法案例　司法案例（98）

法学期刊　法学期刊（23）

第二十九章　不当得利

第九百八十五条

得利人没有法律根据取得不当利益的，受损失的人可以请求得利人返还取得的利益，但是有下列情形之一的除外：

（一）为履行道德义务进行的给付；

（二）债务到期之前的清偿；

（三）明知无给付义务而进行的债务清偿。

法宝联想

法律法规　规范性文件（2）法律动态（2）

司法案例　司法案例（41756）案例报道（5）

法学期刊　法学期刊（121）

法宝律师　律所实务（10）

专题参考　专题参考（11）

第九百八十六条

得利人不知道且不应当知道取得的利益没有法律根据，取得的利益已经不存在的，不承担返还该利益的义务。

法宝联想

司法案例　司法案例（834）

法学期刊　法学期刊（51）

法宝律师　律所实务（1）

第九百八十七条

得利人知道或者应当知道取得的利益没有法律根据的，受损失的人可以请求得利人返还其取得的利益并依法赔偿损失。

法宝联想

司法案例　司法案例（12803）仲裁案例（1）

法学期刊　法学期刊（36）

法宝律师　律所实务（2）

第九百八十八条

得利人已经将取得的利益无偿转让给第三人的，受损失的人可以请求第三人在相应范围内承担返还义务。

法宝联想

法律法规　规范性文件（1）法律动态（1）

司法案例　司法案例（517）案例报道（1）

法学期刊　法学期刊（23）

第四编　人格权

第一章　一般规定

第九百八十九条　本编调整因人格权的享有和保护产生的民事关系。

法宝联想

法律法规　法律动态（1）

司法案例　司法案例（26）

法学期刊　法学期刊（17）

专题参考　专题参考（2）

第九百九十条

人格权是民事主体享有的生命权、身体权、健康权、姓名权、名称权、肖像权、名誉权、荣誉权、隐私权等权利。

除前款规定的人格权外，自然人享有基于人身自由、人格尊严产生的其他人格权益。

法宝联想

法律法规　规范性文件（5）工作报告（1）法律动态（2）

司法案例　司法案例（1777）案例报道（8）

法学期刊　法学期刊（234）

法宝律师　律所实务（5）

专题参考　专题参考（20）

第九百九十一条

民事主体的人格权受法律保护，任何组织或者个人不得侵害。

法宝联想

法律法规　规范性文件（2）法律动态（1）

司法案例　司法案例（1930）案例报道（4）

法学期刊　法学期刊（22）

法宝律师　律所实务（3）

专题参考　专题参考（1）

第九百九十二条　人格权不得放弃、转让或者继承。

法宝联想

司法案例　司法案例（46）

法学期刊　法学期刊（61）

专题参考　专题参考（3）

第九百九十三条

民事主体可以将自己的姓名、名称、肖像等许可他人使用，但是依照法律规定或者根据其性质不得许可的除外。

法宝联想

司法案例　司法案例（48）

法学期刊　法学期刊（90）

专题参考　专题参考（7）

第九百九十四条

死者的姓名、肖像、名誉、荣誉、隐私、遗体等受到侵害的，其配偶、子女、父母有权依法请求行为人承担民事责任；死者没有配偶、子女且父母已经死亡的，其他近亲属有权依法请求行为人承担民事责任。

法宝联想

法律法规　司法解释（1）法律动态（2）

司法案例　司法案例（227）案例报道（4）

法学期刊　法学期刊（69）

法宝律师　律所实务（3）

专题参考　专题参考（12）

第九百九十五条

人格权受到侵害的，受害人有权依照本法和其他法律的规定请求行为人承担民事责任。受害人的停止侵害、排除妨碍、消除危险、消除影响、恢复名誉、赔礼道歉请求权，不适用诉讼时效的规定。

法宝联想

法律法规　规范性文件（5）法律动态（2）

司法案例　司法案例（2954）案例报道（6）

法学期刊　法学期刊（166）

法宝律师　律所实务（3）

专题参考　专题参考（14）

第九百九十六条

因当事人一方的违约行为，损害对方人格权并造成严重精神损害，受损害方选择请求其承担违约责任的，不影响受损害方请求精神损害赔偿。

法宝联想

司法案例　司法案例（1104）案例报道（5）

法学期刊　法学期刊（66）

专题参考　专题参考（5）

第九百九十七条

民事主体有证据证明行为人正在实施或者即将实施侵害其人格权的违法行为，不及时制止将使其合法权益受到难以弥补的损害的，有权依法向人民法院申请采取责令行为人停止有关行为的措施。

法宝联想

法律法规　司法解释（1）规范性文件（12）法规解读（2）法律动态（14）

司法案例　司法案例（75）案例报道（14）

法学期刊　法学期刊（154）

法宝律师　律所实务（7）

专题参考　专题参考（12）

第九百九十八条

认定行为人承担侵害除生命权、身体权和健康权外的人格权的民事责任，应当考虑行为人和受害人的职业、影响范围、过错程度，以及行为的目的、方式、后果等因素。

法宝联想

法律法规　司法解释（1）规范性文件（2）法律动态（1）

司法案例　司法案例（935）案例报道（3）

法学期刊　法学期刊（137）

法宝律师　律所实务（4）

专题参考　专题参考（4）

第九百九十九条

为公共利益实施新闻报道、舆论监督等行为的，可以合理使用民事主体的姓名、名称、肖像、个人信息等；使用不合理侵害民事主体人格权的，应当依法承担民事责任。

法宝联想

法律法规　法律动态（2）

司法案例　司法案例（75）案例报道（1）

法学期刊　法学期刊（124）

法宝律师　律所实务（4）

专题参考　专题参考（8）

第一千条

行为人因侵害人格权承担消除影响、恢复名誉、赔礼道歉等民事责任的，应当与行为的具体方式和造成的影响范围相当。

行为人拒不承担前款规定的民事责任的，人民法院可以采取在报刊、网络等媒体上发布公告或者公布生效裁判文书等方式执行，产生的费用由行为人负担。

法宝联想
法律法规　规范性文件（3）法律动态（2）
司法案例　司法案例（2917）案例报道（4）
法学期刊　法学期刊（43）
法宝律师　律所实务（4）
专题参考　专题参考（2）

第一千零一条

对自然人因婚姻家庭关系等产生的身份权利的保护，适用本法第一编、第五编和其他法律的相关规定；没有规定的，可以根据其性质参照适用本编人格权保护的有关规定。

法宝联想
法律法规　规范性文件（2）法律动态（2）
司法案例　司法案例（43）案例报道（2）
法学期刊　法学期刊（63）
法宝律师　律所实务（2）
专题参考　专题参考（4）

第二章　生命权、身体权和健康权

第一千零二条

自然人享有生命权。自然人的生命安全和生命尊严受法律保护。任何组织或者个人不得侵害他人的生命权。

法宝联想

法律法规 规范性文件（1）法律动态（1）

司法案例 司法案例（2787）案例报道（1）

法学期刊 法学期刊（57）

法宝律师 律所实务（4）

专题参考 专题参考（3）

第一千零三条

自然人享有身体权。自然人的身体完整和行动自由受法律保护。任何组织或者个人不得侵害他人的身体权。

法宝联想

司法案例 司法案例（4911）

法学期刊 法学期刊（34）

法宝律师 律所实务（1）

第一千零四条

自然人享有健康权。自然人的身心健康受法律保护。任何组织或者个人不得侵害他人的健康权。

法宝联想

法律法规 法律动态（2）

司法案例 司法案例（13918）案例报道（2）

法学期刊 法学期刊（37）

法宝律师 律所实务（1）

专题参考 专题参考（2）

第一千零五条

自然人的生命权、身体权、健康权受到侵害或者处于其他危难情形的，负有法定救助义务的组织或者个人应当及时施救。

法宝联想

法律法规　规范性文件（1）

司法案例　司法案例（258）案例报道（3）

法学期刊　法学期刊（27）

法宝律师　律所实务（1）

专题参考　专题参考（2）

第一千零六条

完全民事行为能力人有权依法自主决定无偿捐献其人体细胞、人体组织、人体器官、遗体。任何组织或者个人不得强迫、欺骗、利诱其捐献。

完全民事行为能力人依据前款规定同意捐献的，应当采用书面形式，也可以订立遗嘱。

自然人生前未表示不同意捐献的，该自然人死亡后，其配偶、成年子女、父母可以共同决定捐献，决定捐献应当采用书面形式。

法宝联想

法律法规　草案（1）

司法案例　司法案例（36）

法学期刊　法学期刊（31）

专题参考　专题参考（3）

第一千零七条

禁止以任何形式买卖人体细胞、人体组织、人体器官、遗体。

违反前款规定的买卖行为无效。

法宝联想

司法案例 司法案例（42）案例报道（2）

法学期刊 法学期刊（33）

专题参考 专题参考（2）

第一千零八条

为研制新药、医疗器械或者发展新的预防和治疗方法，需要进行临床试验的，应当依法经相关主管部门批准并经伦理委员会审查同意，向受试者或者受试者的监护人告知试验目的、用途和可能产生的风险等详细情况，并经其书面同意。

进行临床试验的，不得向受试者收取试验费用。

法宝联想

司法案例 司法案例（134）

法学期刊 法学期刊（45）

专题参考 专题参考（3）

第一千零九条

从事与人体基因、人体胚胎等有关的医学和科研活动，应当遵守法律、行政法规和国家有关规定，不得危害人体健康，不得违背伦理道德，不得损害公共利益。

法宝联想

法律法规 规范性文件（2）法律动态（1）

司法案例 司法案例（108）案例报道（3）

法学期刊 法学期刊（74）

法宝律师 律所实务（2）

专题参考　专题参考（6）

第一千零一十条

违背他人意愿，以言语、文字、图像、肢体行为等方式对他人实施性骚扰的，受害人有权依法请求行为人承担民事责任。

机关、企业、学校等单位应当采取合理的预防、受理投诉、调查处置等措施，防止和制止利用职权、从属关系等实施性骚扰。

法宝联想

法律法规　规范性文件（12）法律动态（4）

司法案例　司法案例（52）案例报道（13）

法学期刊　法学期刊（63）

法宝律师　律所实务（2）

专题参考　专题参考（9）

第一千零一十一条

以非法拘禁等方式剥夺、限制他人的行动自由，或者非法搜查他人身体的，受害人有权依法请求行为人承担民事责任。

法宝联想

司法案例　司法案例（18）

法学期刊　法学期刊（21）

专题参考　专题参考（1）

第三章　姓名权和名称权

第一千零一十二条

自然人享有姓名权，有权依法决定、使用、变更或者许可他人使用自己的姓名，但是不得违背公序良俗。

法宝联想

法律法规　规范性文件（3）法律动态（3）

司法案例　司法案例（746）案例报道（5）

法学期刊　法学期刊（40）

法宝律师　律所实务（3）

专题参考　专题参考（6）

第一千零一十三条

法人、非法人组织享有名称权，有权依法决定、使用、变更、转让或者许可他人使用自己的名称。

法宝联想

法律法规　规范性文件（1）法律动态（1）

司法案例　司法案例（173）案例报道（2）

法学期刊　法学期刊（26）

法宝律师　律所实务（2）

专题参考　专题参考（5）

第一千零一十四条

任何组织或者个人不得以干涉、盗用、假冒等方式侵害他人的姓名权或者名称权。

法宝联想

法律法规　规范性文件（2）法律动态（3）

司法案例　司法案例（910）案例报道（3）

法学期刊　法学期刊（19）

法宝律师　律所实务（5）

专题参考　专题参考（3）

第一千零一十五条

自然人应当随父姓或者母姓，但是有下列情形之一的，可以在父姓和母姓之外选取姓氏：

（一）选取其他直系长辈血亲的姓氏；

（二）因由法定扶养人以外的人扶养而选取扶养人姓氏；

（三）有不违背公序良俗的其他正当理由。

少数民族自然人的姓氏可以遵从本民族的文化传统和风俗习惯。

法宝联想

法律法规　规范性文件（2）法律动态（1）

司法案例　司法案例（26）案例报道（4）

法学期刊　法学期刊（29）

专题参考　专题参考（4）

第一千零一十六条

自然人决定、变更姓名，或者法人、非法人组织决定、变更、转让名称的，应当依法向有关机关办理登记手续，但是法律另有规定的除外。

民事主体变更姓名、名称的，变更前实施的民事法律行为对其具有法律约束力。

法宝联想

法律法规　规范性文件（1）

司法案例　司法案例（212）

法学期刊　法学期刊（4）

专题参考　专题参考（2）

第一千零一十七条

具有一定社会知名度，被他人使用足以造成公众混淆的笔名、艺名、网名、译名、字号、姓名和名称的简称等，参照适用姓名权和名称权保护的有关规定。

法宝联想

司法案例 司法案例（38）案例报道（2）仲裁案例（1）

法学期刊 法学期刊（27）

法宝律师 律所实务（2）

专题参考 专题参考（8）

第四章 肖像权

第一千零一十八条

自然人享有肖像权，有权依法制作、使用、公开或者许可他人使用自己的肖像。

肖像是通过影像、雕塑、绘画等方式在一定载体上所反映的特定自然人可以被识别的外部形象。

法宝联想

法律法规 法律动态（1）

司法案例 司法案例（2832）案例报道（3）

法学期刊 法学期刊（47）

法宝律师 律所实务（7）

专题参考 专题参考（11）

第一千零一十九条

任何组织或者个人不得以丑化、污损，或者利用信息技术手段伪造等方式侵害他

人的肖像权。未经肖像权人同意，不得制作、使用、公开肖像权人的肖像，但是法律另有规定的除外。

未经肖像权人同意，肖像作品权利人不得以发表、复制、发行、出租、展览等方式使用或者公开肖像权人的肖像。

法宝联想

法律法规 规范性文件（1）法律动态（1）

司法案例 司法案例（3172）案例报道（9）

法学期刊 法学期刊（72）

法宝律师 律所实务（7）

专题参考 专题参考（7）

第一千零二十条 合理实施下列行为的，可以不经肖像权人同意：

（一）为个人学习、艺术欣赏、课堂教学或者科学研究，在必要范围内使用肖像权人已经公开的肖像；

（二）为实施新闻报道，不可避免地制作、使用、公开肖像权人的肖像；

（三）为依法履行职责，国家机关在必要范围内制作、使用、公开肖像权人的肖像；

（四）为展示特定公共环境，不可避免地制作、使用、公开肖像权人的肖像；

（五）为维护公共利益或者肖像权人合法权益，制作、使用、公开肖像权人的肖像的其他行为。

法宝联想

法律法规 法律动态（1）

司法案例 司法案例（364）案例报道（1）

法学期刊 法学期刊（52）

法宝律师 律所实务（5）

专题参考 专题参考（6）

第一千零二十一条

当事人对肖像许可使用合同中关于肖像使用条款的理解有争议的，应当作出有利于肖像权人的解释。

法宝联想

法律法规 法律动态（1）

司法案例 司法案例（25）

法学期刊 法学期刊（32）

专题参考 专题参考（2）

第一千零二十二条

当事人对肖像许可使用期限没有约定或者约定不明确的，任何一方当事人可以随时解除肖像许可使用合同，但是应当在合理期限之前通知对方。

当事人对肖像许可使用期限有明确约定，肖像权人有正当理由的，可以解除肖像许可使用合同，但是应当在合理期限之前通知对方。因解除合同造成对方损失的，除不可归责于肖像权人的事由外，应当赔偿损失。

法宝联想

法律法规 法律动态（1）

司法案例 司法案例（52）

法学期刊 法学期刊（50）

专题参考 专题参考（2）

第一千零二十三条

对姓名等的许可使用，参照适用肖像许可使用的有关规定。

对自然人声音的保护，参照适用肖像权保护的有关规定。

法宝联想

法律法规 规范性文件（1）法律动态（2）

司法案例 司法案例（60）案例报道（1）

法学期刊 法学期刊（62）

法宝律师 律所实务（8）

专题参考 专题参考（10）

第五章　名誉权和荣誉权

第一千零二十四条

民事主体享有名誉权。任何组织或者个人不得以侮辱、诽谤等方式侵害他人的名誉权。

名誉是对民事主体的品德、声望、才能、信用等的社会评价。

法宝联想

法律法规 规范性文件（12）法律动态（10）

司法案例 司法案例（2327）案例报道（30）

法学期刊 法学期刊（79）

法宝律师 律所实务（19）

专题参考 专题参考（15）

第一千零二十五条

行为人为公共利益实施新闻报道、舆论监督等行为，影响他人名誉的，不承担民事责任，但是有下列情形之一的除外：

（一）捏造、歪曲事实；

（二）对他人提供的严重失实内容未尽到合理核实义务；

（三）使用侮辱性言辞等贬损他人名誉。

法宝联想

法律法规　规范性文件（1）法律动态（1）

司法案例　司法案例（139）案例报道（4）

法学期刊　法学期刊（48）

法宝律师　律所实务（4）

专题参考　专题参考（6）

第一千零二十六条

认定行为人是否尽到前条第二项规定的合理核实义务，应当考虑下列因素：

（一）内容来源的可信度；

（二）对明显可能引发争议的内容是否进行了必要的调查；

（三）内容的时限性；

（四）内容与公序良俗的关联性；

（五）受害人名誉受贬损的可能性；

（六）核实能力和核实成本。

法宝联想

司法案例　司法案例（75）案例报道（1）

法学期刊　法学期刊（38）

法宝律师　律所实务（4）

专题参考　专题参考（3）

第一千零二十七条

行为人发表的文学、艺术作品以真人真事或者特定人为描述对象，含有侮辱、诽谤内容，侵害他人名誉权的，受害人有权依法请求该行为人承担民事责任。

行为人发表的文学、艺术作品不以特定人为描述对象，仅其中的情节与该特定人的情况相似的，不承担民事责任。

法宝联想

司法案例　司法案例（64）

法学期刊　法学期刊（13）

法宝律师　律所实务（2）

专题参考　专题参考（3）

第一千零二十八条

民事主体有证据证明报刊、网络等媒体报道的内容失实，侵害其名誉权的，有权请求该媒体及时采取更正或者删除等必要措施。

法宝联想

司法案例　司法案例（50）

法学期刊　法学期刊（22）

专题参考　专题参考（2）

第一千零二十九条

民事主体可以依法查询自己的信用评价；发现信用评价不当的，有权提出异议并请求采取更正、删除等必要措施。信用评价人应当及时核查，经核查属实的，应当及时采取必要措施。

法宝联想

法律法规　规范性文件（4）法律动态（2）

司法案例　司法案例（867）案例报道（7）

法学期刊　法学期刊（41）

专题参考　专题参考（4）

第一千零三十条

民事主体与征信机构等信用信息处理者之间的关系，适用本编有关个人信息保护的规定和其他法律、行政法规的有关规定。

法宝联想

法律法规　规范性文件（1）

司法案例　司法案例（149）案例报道（1）

法学期刊　法学期刊（27）

专题参考　专题参考（4）

第一千零三十一条

民事主体享有荣誉权。任何组织或者个人不得非法剥夺他人的荣誉称号，不得诋毁、贬损他人的荣誉。

获得的荣誉称号应当记载而没有记载的，民事主体可以请求记载；获得的荣誉称号记载错误的，民事主体可以请求更正。

法宝联想

司法案例　司法案例（20）

法学期刊　法学期刊（9）

第六章　隐私权和个人信息保护

第一千零三十二条

自然人享有隐私权。任何组织或者个人不得以刺探、侵扰、泄露、公开等方式侵害他人的隐私权。

隐私是自然人的私人生活安宁和不愿为他人知晓的私密空间、私密活动、私密信息。

法宝联想

法律法规 规范性文件（4）法律动态（9）

司法案例 司法案例（777）案例报道（17）

法学期刊 法学期刊（209）

法宝律师 律所实务（12）

专题参考 专题参考（18）

第一千零三十三条

除法律另有规定或者权利人明确同意外，任何组织或者个人不得实施下列行为：

（一）以电话、短信、即时通讯工具、电子邮件、传单等方式侵扰他人的私人生活安宁；

（二）进入、拍摄、窥视他人的住宅、宾馆房间等私密空间；

（三）拍摄、窥视、窃听、公开他人的私密活动；

（四）拍摄、窥视他人身体的私密部位；

（五）处理他人的私密信息；

（六）以其他方式侵害他人的隐私权。

法宝联想

法律法规 规范性文件（5）法律动态（4）

司法案例　司法案例（468）案例报道（8）

法学期刊　法学期刊（122）

法宝律师　律所实务（5）

专题参考　专题参考（11）

第一千零三十四条　自然人的个人信息受法律保护。

个人信息是以电子或者其他方式记录的能够单独或者与其他信息结合识别特定自然人的各种信息，包括自然人的姓名、出生日期、身份证件号码、生物识别信息、住址、电话号码、电子邮箱、健康信息、行踪信息等。

个人信息中的私密信息，适用有关隐私权的规定；没有规定的，适用有关个人信息保护的规定。

法宝联想

法律法规　司法解释（1）规范性文件（12）法规解读（2）法律动态（10）

司法案例　司法案例（515）案例报道（19）

法学期刊　法学期刊（418）

法宝律师　律所实务（14）

专题参考　专题参考（42）

第一千零三十五条

处理个人信息的，应当遵循合法、正当、必要原则，不得过度处理，并符合下列条件：

（一）征得该自然人或者其监护人同意，但是法律、行政法规另有规定的除外；

（二）公开处理信息的规则；

（三）明示处理信息的目的、方式和范围；

（四）不违反法律、行政法规的规定和双方的约定。

个人信息的处理包括个人信息的收集、存储、使用、加工、传输、提供、公开等。

法宝联想

法律法规　司法解释（1）规范性文件（8）法律动态（7）

司法案例　司法案例（215）案例报道（9）

法学期刊　法学期刊（324）

法宝律师　律所实务（4）

专题参考　专题参考（31）

第一千零三十六条

处理个人信息，有下列情形之一的，行为人不承担民事责任：

（一）在该自然人或者其监护人同意的范围内合理实施的行为；

（二）合理处理该自然人自行公开的或者其他已经合法公开的信息，但是该自然人明确拒绝或者处理该信息侵害其重大利益的除外；

（三）为维护公共利益或者该自然人合法权益，合理实施的其他行为。

法宝联想

法律法规　规范性文件（2）法律动态（3）

司法案例　司法案例（82）案例报道（3）

法学期刊　法学期刊（238）

法宝律师　律所实务（1）

专题参考　专题参考（26）

第一千零三十七条

自然人可以依法向信息处理者查阅或者复制其个人信息；发现信息有错误的，有权提出异议并请求及时采取更正等必要措施。

自然人发现信息处理者违反法律、行政法规的规定或者双方的约定处理其个人信息的，有权请求信息处理者及时删除。

法宝联想

法律法规 规范性文件（2）

司法案例 司法案例（74）案例报道（3）

法学期刊 法学期刊（147）

专题参考 专题参考（17）

第一千零三十八条

信息处理者不得泄露或者篡改其收集、存储的个人信息；未经自然人同意，不得向他人非法提供其个人信息，但是经过加工无法识别特定个人且不能复原的除外。

信息处理者应当采取技术措施和其他必要措施，确保其收集、存储的个人信息安全，防止信息泄露、篡改、丢失；发生或者可能发生个人信息泄露、篡改、丢失的，应当及时采取补救措施，按照规定告知自然人并向有关主管部门报告。

法宝联想

法律法规 规范性文件（1）法律动态（1）

司法案例 司法案例（66）

法学期刊 法学期刊（113）

法宝律师 律所实务（3）

专题参考 专题参考（13）

第一千零三十九条

国家机关、承担行政职能的法定机构及其工作人员对于履行职责过程中知悉的自然人的隐私和个人信息，应当予以保密，不得泄露或者向他人非法提供。

法宝联想

法律法规　规范性文件（1）法律动态（2）

司法案例　司法案例（24）

法学期刊　法学期刊（71）

法宝律师　律所实务（1）

专题参考　专题参考（13）

第五编　婚姻家庭

第一章　一般规定

第一千零四十条　本编调整因婚姻家庭产生的民事关系。

法宝联想

司法案例　司法案例（74）

法学期刊　法学期刊（4）

法宝律师　律所实务（1）

第一千零四十一条　婚姻家庭受国家保护。

实行婚姻自由、一夫一妻、男女平等的婚姻制度。

保护妇女、未成年人、老年人、残疾人的合法权益。

法宝联想

法律法规　规范性文件（2）

司法案例　司法案例（541）案例报道（1）

法学期刊　法学期刊（65）

法宝律师　律所实务（1）

专题参考 专题参考（3）

第一千零四十二条

禁止包办、买卖婚姻和其他干涉婚姻自由的行为。禁止借婚姻索取财物。

禁止重婚。禁止有配偶者与他人同居。

禁止家庭暴力。禁止家庭成员间的虐待和遗弃。

法宝联想

法律法规 司法解释（1）规范性文件（7）法律动态（4）

司法案例 司法案例（6154）案例报道（14）

法学期刊 法学期刊（48）

法宝律师 律所实务（4）

专题参考 专题参考（6）

第一千零四十三条

家庭应当树立优良家风，弘扬家庭美德，重视家庭文明建设。

夫妻应当互相忠实，互相尊重，互相关爱；家庭成员应当敬老爱幼，互相帮助，维护平等、和睦、文明的婚姻家庭关系。

法宝联想

法律法规 司法解释（1）规范性文件（8）法律动态（8）

司法案例 司法案例（2457）案例报道（17）

法学期刊 法学期刊（98）

法宝律师 律所实务（5）

专题参考 专题参考（5）

第一千零四十四条

收养应当遵循最有利于被收养人的原则，保障被收养人和收养人的合法权益。

禁止借收养名义买卖未成年人。

法宝联想

司法案例 司法案例（20）案例报道（1）

法学期刊 法学期刊（18）

专题参考 专题参考（4）

第一千零四十五条 亲属包括配偶、血亲和姻亲。

配偶、父母、子女、兄弟姐妹、祖父母、外祖父母、孙子女、外孙子女为近亲属。

配偶、父母、子女和其他共同生活的近亲属为家庭成员。

法宝联想

法律法规 规范性文件（10）法律动态（2）

司法案例 司法案例（1334）案例报道（6）

法学期刊 法学期刊（74）

法宝律师 律所实务（13）

专题参考 专题参考（3）

第二章 结婚

第一千零四十六条

结婚应当男女双方完全自愿，禁止任何一方对另一方加以强迫，禁止任何组织或者个人加以干涉。

法宝联想

法律法规　规范性文件（3）

司法案例　司法案例（225）案例报道（1）

法学期刊　法学期刊（16）

法宝律师　律所实务（2）

专题参考　专题参考（1）

第一千零四十七条

结婚年龄，男不得早于二十二周岁，女不得早于二十周岁。

法宝联想

法律法规　规范性文件（4）法律动态（1）

司法案例　司法案例（83）案例报道（1）

法学期刊　法学期刊（12）

法宝律师　律所实务（1）

专题参考　专题参考（1）

第一千零四十八条　直系血亲或者三代以内的旁系血亲禁止结婚。

法宝联想

法律法规　规范性文件（1）

司法案例　司法案例（229）

法学期刊　法学期刊（9）

法宝律师　律所实务（1）

专题参考　专题参考（1）

第一千零四十九条

要求结婚的男女双方应当亲自到婚姻登记机关申请结婚登记。符合本法规定的，予以登记，发给结婚证。完成结婚登记，即确立婚姻关系。未办理结婚登记的，应当补办登记。

法宝联想

法律法规 司法解释（1）规范性文件（6）法律动态（2）

司法案例 司法案例（715）案例报道（6）

法学期刊 法学期刊（24）

法宝律师 律所实务（2）

专题参考 专题参考（1）

第一千零五十条

登记结婚后，按照男女双方约定，女方可以成为男方家庭的成员，男方可以成为女方家庭的成员。

法宝联想

法律法规 规范性文件（1）

司法案例 司法案例（82）案例报道（1）

法学期刊 法学期刊（12）

第一千零五十一条 有下列情形之一的，婚姻无效：

（一）重婚；

（二）有禁止结婚的亲属关系；

（三）未到法定婚龄。

法宝联想

法律法规 司法解释（2）规范性文件（2）法律动态（1）

司法案例 司法案例（444）案例报道（1）

法学期刊 法学期刊（33）

法宝律师 律所实务（3）

专题参考 专题参考（3）

第一千零五十二条

因胁迫结婚的，受胁迫的一方可以向人民法院请求撤销婚姻。

请求撤销婚姻的，应当自胁迫行为终止之日起一年内提出。

被非法限制人身自由的当事人请求撤销婚姻的，应当自恢复人身自由之日起一年内提出。

法宝联想

法律法规　行政法规（2）司法解释（2）规范性文件（3）法律动态（2）

司法案例　司法案例（35）案例报道（1）

法学期刊　法学期刊（30）

法宝律师　律所实务（1）

专题参考　专题参考（6）

第一千零五十三条

一方患有重大疾病的，应当在结婚登记前如实告知另一方；不如实告知的，另一方可以向人民法院请求撤销婚姻。

请求撤销婚姻的，应当自知道或者应当知道撤销事由之日起一年内提出。

法宝联想

法律法规　行政法规（2）规范性文件（7）法律动态（2）

司法案例　司法案例（284）案例报道（13）

法学期刊　法学期刊（58）

法宝律师　律所实务（2）

专题参考　专题参考（6）

第一千零五十四条

无效的或者被撤销的婚姻自始没有法律约束力，当事人不具有夫妻的权利和义务。同居期间所得的财产，由当事人协议处理；协议不成的，由人民法院根据照顾

无过错方的原则判决。对重婚导致的无效婚姻的财产处理，不得侵害合法婚姻当事人的财产权益。当事人所生的子女，适用本法关于父母子女的规定。

婚姻无效或者被撤销的，无过错方有权请求损害赔偿。

法宝联想

法律法规　司法解释（1）

司法案例　司法案例（535）案例报道（2）

法学期刊　法学期刊（38）

法宝律师　律所实务（3）

专题参考　专题参考（3）

第三章　家庭关系

第一节　夫妻关系

第一千零五十五条　夫妻在婚姻家庭中地位平等。

法宝联想

司法案例　司法案例（156）

法学期刊　法学期刊（12）

第一千零五十六条　夫妻双方都有各自使用自己姓名的权利。

法宝联想

司法案例　司法案例（6）

法学期刊　法学期刊（8）

第一千零五十七条

夫妻双方都有参加生产、工作、学习和社会活动的自由，一方不得对另一方加以限制或者干涉。

法宝联想

司法案例　司法案例（22）

法学期刊　法学期刊（9）

专题参考　专题参考（2）

第一千零五十八条

夫妻双方平等享有对未成年子女抚养、教育和保护的权利，共同承担对未成年子女抚养、教育和保护的义务。

法宝联想

法律法规　规范性文件（2）法律动态（4）

司法案例　司法案例（658）案例报道（6）

法学期刊　法学期刊（23）

法宝律师　律所实务（1）

专题参考　专题参考（1）

第一千零五十九条　夫妻有相互扶养的义务。

需要扶养的一方，在另一方不履行扶养义务时，有要求其给付扶养费的权利。

法宝联想

法律法规　规范性文件（8）法律动态（5）

司法案例　司法案例（2251）案例报道（15）

法学期刊　法学期刊（32）

专题参考　专题参考（1）

第一千零六十条

夫妻一方因家庭日常生活需要而实施的民事法律行为，对夫妻双方发生效力，但是夫妻一方与相对人另有约定的除外。

夫妻之间对一方可以实施的民事法律行为范围的限制，不得对抗善意相对人。

法宝联想

司法案例　司法案例（2386）案例报道（1）

法学期刊　法学期刊（45）

法宝律师　律所实务（6）

专题参考　专题参考（2）

第一千零六十一条　夫妻有相互继承遗产的权利。

法宝联想

司法案例　司法案例（694）

法学期刊　法学期刊（6）

专题参考　专题参考（1）

第一千零六十二条

夫妻在婚姻关系存续期间所得的下列财产，为夫妻的共同财产，归夫妻共同所有

：

（一）工资、奖金、劳务报酬；

（二）生产、经营、投资的收益；

（三）知识产权的收益；

（四）继承或者受赠的财产，但是本法第一千零六十三条第三项规定的除外；

（五）其他应当归共同所有的财产。

夫妻对共同财产，有平等的处理权。

法宝联想

法律法规　司法解释（1）规范性文件（6）法律动态（3）

司法案例　司法案例（18753）案例报道（15）

法学期刊　法学期刊（107）

法宝律师　律所实务（40）

专题参考　专题参考（11）

第一千零六十三条　下列财产为夫妻一方的个人财产：

（一）一方的婚前财产；

（二）一方因受到人身损害获得的赔偿或者补偿；

（三）遗嘱或者赠与合同中确定只归一方的财产；

（四）一方专用的生活用品；

（五）其他应当归一方的财产。

法宝联想

法律法规　司法解释（1）规范性文件（2）法律动态（2）

司法案例　司法案例（3500）案例报道（2）

法学期刊　法学期刊（48）

法宝律师　律所实务（26）

专题参考　专题参考（5）

第一千零六十四条

夫妻双方共同签名或者夫妻一方事后追认等共同意思表示所负的债务，以及夫妻一方在婚姻关系存续期间以个人名义为家庭日常生活需要所负的债务，属于夫妻共同债务。

夫妻一方在婚姻关系存续期间以个人名义超出家庭日常生活需要所负的债务，不属于夫妻共同债务；但是，债权人能够证明该债务用于夫妻共同生活、共同生产经营或者基于夫妻双方共同意思表示的除外。

法宝联想

法律法规 规范性文件（5）法律动态（1）

司法案例 司法案例（111482）案例报道（13）仲裁案例（1）

法学期刊 法学期刊（102）

法宝律师 律所实务（23）

专题参考 专题参考（7）

第一千零六十五条

男女双方可以约定婚姻关系存续期间所得的财产以及婚前财产归各自所有、共同所有或者部分各自所有、部分共同所有。约定应当采用书面形式。没有约定或者约定不明确的，适用本法第一千零六十二条、第一千零六十三条的规定。

夫妻对婚姻关系存续期间所得的财产以及婚前财产的约定，对双方具有法律约束力。

夫妻对婚姻关系存续期间所得的财产约定归各自所有，夫或者妻一方对外所负的债务，相对人知道该约定的，以夫或者妻一方的个人财产清偿。

法宝联想

法律法规 司法解释（1）规范性文件（3）法律动态（1）

司法案例 司法案例（3561）案例报道（3）仲裁案例（1）

法学期刊 法学期刊（93）

法宝律师 律所实务（20）

专题参考 专题参考（4）

第一千零六十六条

婚姻关系存续期间，有下列情形之一的，夫妻一方可以向人民法院请求分割共同财产：

（一）一方有隐藏、转移、变卖、毁损、挥霍夫妻共同财产或者伪造夫妻共同债务等严重损害夫妻共同财产利益的行为；

（二）一方负有法定扶养义务的人患重大疾病需要医治，另一方不同意支付相关医疗费用。

法宝联想

法律法规　司法解释（2）规范性文件（1）法律动态（3）

司法案例　司法案例（908）案例报道（5）

法学期刊　法学期刊（52）

法宝律师　律所实务（5）

第二节　父母子女关系和其他近亲属关系

第一千零六十七条

父母不履行抚养义务的，未成年子女或者不能独立生活的成年子女，有要求父母给付抚养费的权利。

成年子女不履行赡养义务的，缺乏劳动能力或者生活困难的父母，有要求成年子女给付赡养费的权利。

法宝联想

法律法规　司法解释（1）规范性文件（9）法律动态（7）

司法案例　司法案例（13814）案例报道（34）

法学期刊　法学期刊（51）

法宝律师　律所实务（3）

专题参考　专题参考（7）

第一千零六十八条

父母有教育、保护未成年子女的权利和义务。未成年子女造成他人损害的，父母应当依法承担民事责任。

法宝联想

法律法规　司法解释（1）规范性文件（2）法规解读（1）法律动态（2）

司法案例　司法案例（770）案例报道（2）

法学期刊　法学期刊（31）

法宝律师　律所实务（2）

专题参考　专题参考（2）

第一千零六十九条

子女应当尊重父母的婚姻权利，不得干涉父母离婚、再婚以及婚后的生活。子女对父母的赡养义务，不因父母的婚姻关系变化而终止。

法宝联想

法律法规　规范性文件（2）法律动态（1）

司法案例　司法案例（216）案例报道（4）

法学期刊　法学期刊（13）

第一千零七十条　父母和子女有相互继承遗产的权利。

法宝联想

法律法规　规范性文件（3）

司法案例　司法案例（591）案例报道（2）

法学期刊　法学期刊（6）

法宝律师　律所实务（1）

第一千零七十一条

非婚生子女享有与婚生子女同等的权利，任何组织或者个人不得加以危害和歧视。

不直接抚养非婚生子女的生父或者生母，应当负担未成年子女或者不能独立生活的成年子女的抚养费。

法宝联想

法律法规　规范性文件（3）法律动态（3）

司法案例　司法案例（2511）案例报道（12）

法学期刊　法学期刊（24）

法宝律师　律所实务（1）

专题参考　专题参考（1）

第一千零七十二条　继父母与继子女间，不得虐待或者歧视。

继父或者继母和受其抚养教育的继子女间的权利义务关系，适用本法关于父母子女关系的规定。

法宝联想

法律法规　司法解释（2）规范性文件（2）法规解读（1）法律动态（2）

司法案例　司法案例（1325）案例报道（2）

法学期刊　法学期刊（25）

法宝律师　律所实务（2）

专题参考　专题参考（2）

第一千零七十三条

对亲子关系有异议且有正当理由的，父或者母可以向人民法院提起诉讼，请求确认或者否认亲子关系。

对亲子关系有异议且有正当理由的，成年子女可以向人民法院提起诉讼，请求确认亲子关系。

法宝联想

法律法规　规范性文件（1）

司法案例　司法案例（481）案例报道（2）

法学期刊　法学期刊（40）

专题参考　专题参考（5）

第一千零七十四条

有负担能力的祖父母、外祖父母，对于父母已经死亡或者父母无力抚养的未成年孙子女、外孙子女，有抚养的义务。

有负担能力的孙子女、外孙子女，对于子女已经死亡或者子女无力赡养的祖父母、外祖父母，有赡养的义务。

法宝联想

法律法规　规范性文件（5）

司法案例　司法案例（335）案例报道（6）

法学期刊　法学期刊（20）

专题参考　专题参考（1）

第一千零七十五条

有负担能力的兄、姐，对于父母已经死亡或者父母无力抚养的未成年弟、妹，有扶养的义务。

由兄、姐扶养长大的有负担能力的弟、妹，对于缺乏劳动能力又缺乏生活来源的兄、姐，有扶养的义务。

法宝联想

法律法规　规范性文件（2）

司法案例　司法案例（146）

法学期刊　法学期刊（19）

专题参考　专题参考（1）

第四章　离婚

第一千零七十六条

夫妻双方自愿离婚的，应当签订书面离婚协议，并亲自到婚姻登记机关申请离婚登记。

离婚协议应当载明双方自愿离婚的意思表示和对子女抚养、财产以及债务处理等事项协商一致的意见。

法宝联想

法律法规　司法解释（1）规范性文件（6）法律动态（1）

司法案例　司法案例（3948）案例报道（1）

法学期刊　法学期刊（26）

法宝律师　律所实务（2）

专题参考　专题参考（2）

第一千零七十七条

自婚姻登记机关收到离婚登记申请之日起三十日内，任何一方不愿意离婚的，可以向婚姻登记机关撤回离婚登记申请。

前款规定期限届满后三十日内，双方应当亲自到婚姻登记机关申请发给离婚证；未申请的，视为撤回离婚登记申请。

法宝联想

法律法规　规范性文件（4）法律动态（1）

司法案例　司法案例（45）案例报道（3）

法学期刊　法学期刊（48）

专题参考　专题参考（4）

第一千零七十八条

婚姻登记机关查明双方确实是自愿离婚，并已经对子女抚养、财产以及债务处理等事项协商一致的，予以登记，发给离婚证。

法宝联想

法律法规　规范性文件（4）

司法案例　司法案例（309）

法学期刊　法学期刊（6）

第一千零七十九条

夫妻一方要求离婚的，可以由有关组织进行调解或者直接向人民法院提起离婚诉讼。

人民法院审理离婚案件，应当进行调解；如果感情确已破裂，调解无效的，应当准予离婚。

有下列情形之一，调解无效的，应当准予离婚：

（一）重婚或者与他人同居；

（二）实施家庭暴力或者虐待、遗弃家庭成员；

（三）有赌博、吸毒等恶习屡教不改；

（四）因感情不和分居满二年；

（五）其他导致夫妻感情破裂的情形。

一方被宣告失踪，另一方提起离婚诉讼的，应当准予离婚。

经人民法院判决不准离婚后，双方又分居满一年，一方再次提起离婚诉讼的，应当准予离婚。

法宝联想

法律法规 司法解释（2）规范性文件（7）法律动态（4）

司法案例 司法案例（23460）案例报道（44）

法学期刊 法学期刊（65）

法宝律师 律所实务（4）

专题参考 专题参考（5）

第一千零八十条

完成离婚登记，或者离婚判决书、调解书生效，即解除婚姻关系。

法宝联想

司法案例 司法案例（244）

法学期刊 法学期刊（6）

第一千零八十一条

现役军人的配偶要求离婚，应当征得军人同意，但是军人一方有重大过错的除外

。

法宝联想

法律法规　司法解释（1）

司法案例　司法案例（19）

法学期刊　法学期刊（2）

法宝律师　律所实务（1）

第一千零八十二条

女方在怀孕期间、分娩后一年内或者终止妊娠后六个月内，男方不得提出离婚；但是，女方提出离婚或者人民法院认为确有必要受理男方离婚请求的除外。

法宝联想

法律法规　规范性文件（1）

司法案例　司法案例（98）案例报道（2）

法学期刊　法学期刊（7）

专题参考　专题参考（1）

第一千零八十三条

离婚后，男女双方自愿恢复婚姻关系的，应当到婚姻登记机关重新进行结婚登记

。

法宝联想

司法案例　司法案例（148）

法学期刊　法学期刊（5）

第一千零八十四条

父母与子女间的关系，不因父母离婚而消除。离婚后，子女无论由父或者母直接抚养，仍是父母双方的子女。

离婚后，父母对于子女仍有抚养、教育、保护的权利和义务。

离婚后，不满两周岁的子女，以由母亲直接抚养为原则。已满两周岁的子女，父母双方对抚养问题协议不成的，由人民法院根据双方的具体情况，按照最有利于未成年子女的原则判决。子女已满八周岁的，应当尊重其真实意愿。

法宝联想

法律法规　司法解释（3）规范性文件（13）法规解读（1）法律动态（18）

司法案例　司法案例（7099）案例报道（29）

法学期刊　法学期刊（71）

法宝律师　律所实务（11）

专题参考　专题参考（4）

第一千零八十五条

离婚后，子女由一方直接抚养的，另一方应当负担部分或者全部抚养费。负担费用的多少和期限的长短，由双方协议；协议不成的，由人民法院判决。

前款规定的协议或者判决，不妨碍子女在必要时向父母任何一方提出超过协议或者判决原定数额的合理要求。

法宝联想

法律法规　规范性文件（5）法律动态（4）

司法案例　司法案例（7353）案例报道（16）

法学期刊　法学期刊（17）

法宝律师　律所实务（2）

专题参考　专题参考（2）

第一千零八十六条

离婚后，不直接抚养子女的父或者母，有探望子女的权利，另一方有协助的义务。

行使探望权利的方式、时间由当事人协议；协议不成的，由人民法院判决。

父或者母探望子女，不利于子女身心健康的，由人民法院依法中止探望；中止的事由消失后，应当恢复探望。

法宝联想

法律法规　规范性文件（7）法律动态（4）

司法案例　司法案例（2363）案例报道（12）

法学期刊　法学期刊（34）

法宝律师　律所实务（3）

专题参考　专题参考（2）

第一千零八十七条

离婚时，夫妻的共同财产由双方协议处理；协议不成的，由人民法院根据财产的具体情况，按照照顾子女、女方和无过错方权益的原则判决。

对夫或者妻在家庭土地承包经营中享有的权益等，应当依法予以保护。

法宝联想

法律法规　司法解释（2）规范性文件（3）法规解读（1）法律动态（5）

司法案例　司法案例（7358）案例报道（6）

法学期刊　法学期刊（78）

法宝律师　律所实务（16）

专题参考　专题参考（1）

第一千零八十八条

夫妻一方因抚育子女、照料老年人、协助另一方工作等负担较多义务的，离婚时有权向另一方请求补偿，另一方应当给予补偿。具体办法由双方协议；协议不成的，由人民法院判决。

法宝联想

法律法规　司法解释（1）规范性文件（14）法律动态（14）

司法案例　司法案例（461）案例报道（28）

法学期刊　法学期刊（52）

法宝律师　律所实务（5）

专题参考　专题参考（3）

第一千零八十九条

离婚时，夫妻共同债务应当共同偿还。共同财产不足清偿或者财产归各自所有的，由双方协议清偿；协议不成的，由人民法院判决。

法宝联想

法律法规　司法解释（1）

司法案例　司法案例（4932）案例报道（3）

法学期刊　法学期刊（32）

法宝律师　律所实务（5）

专题参考　专题参考（2）

第一千零九十条

离婚时，如果一方生活困难，有负担能力的另一方应当给予适当帮助。具体办法由双方协议；协议不成的，由人民法院判决。

法宝联想

法律法规　司法解释（1）规范性文件（10）法规解读（1）法律动态（7）

司法案例　司法案例（382）案例报道（10）

法学期刊　法学期刊（53）

法宝律师　律所实务（4）

专题参考　专题参考（2）

第一千零九十一条

有下列情形之一，导致离婚的，无过错方有权请求损害赔偿：

（一）重婚；

（二）与他人同居；

（三）实施家庭暴力；

（四）虐待、遗弃家庭成员；

（五）有其他重大过错。

法宝联想

法律法规　司法解释（1）规范性文件（6）法规解读（1）法律动态（7）

司法案例　司法案例（864）案例报道（19）

法学期刊　法学期刊（83）

法宝律师　律所实务（7）

专题参考　专题参考（3）

第一千零九十二条

夫妻一方隐藏、转移、变卖、毁损、挥霍夫妻共同财产，或者伪造夫妻共同债务企图侵占另一方财产的，在离婚分割夫妻共同财产时，对该方可以少分或者不分。离婚后，另一方发现有上述行为的，可以向人民法院提起诉讼，请求再次分割夫妻共同财产。

法宝联想

法律法规　司法解释（2）规范性文件（2）法律动态（3）

司法案例　司法案例（1269）案例报道（6）

法学期刊　法学期刊（26）

法宝律师　律所实务（10）

专题参考　专题参考（2）

第五章　收养

第一节　收养关系的成立

第一千零九十三条　下列未成年人，可以被收养：

（一）丧失父母的孤儿；

（二）查找不到生父母的未成年人；

（三）生父母有特殊困难无力抚养的子女。

法宝联想

法律法规　规范性文件（2）

司法案例　司法案例（63）

法学期刊　法学期刊（11）

专题参考　专题参考（1）

第一千零九十四条　下列个人、组织可以作送养人：

（一）孤儿的监护人；

（二）儿童福利机构；

（三）有特殊困难无力抚养子女的生父母。

法宝联想

法律法规　规范性文件（1）

司法案例　司法案例（9）

法学期刊　法学期刊（5）

专题参考　专题参考（1）

第一千零九十五条

未成年人的父母均不具备完全民事行为能力且可能严重危害该未成年人的，该未成年人的监护人可以将其送养。

法宝联想

法律法规　规范性文件（1）

司法案例　司法案例（9）仲裁案例（1）

法学期刊　法学期刊（3）

专题参考　专题参考（1）

第一千零九十六条

监护人送养孤儿的，应当征得有抚养义务的人同意。有抚养义务的人不同意送养、监护人不愿意继续履行监护职责的，应当依照本法第一编的规定另行确定监护人。

法宝联想

法律法规　规范性文件（2）

司法案例　司法案例（8）仲裁案例（1）

法学期刊　法学期刊（2）

第一千零九十七条

生父母送养子女，应当双方共同送养。生父母一方不明或者查找不到的，可以单方送养。

法宝联想

法律法规　规范性文件（3）法律动态（1）

司法案例　司法案例（20）案例报道（1）

法学期刊　法学期刊（1）

专题参考　专题参考（1）

第一千零九十八条　收养人应当同时具备下列条件：

（一）无子女或者只有一名子女；

（二）有抚养、教育和保护被收养人的能力；

（三）未患有在医学上认为不应当收养子女的疾病；

（四）无不利于被收养人健康成长的违法犯罪记录；

（五）年满三十周岁。

法宝联想

法律法规　规范性文件（2）

司法案例　司法案例（67）仲裁案例（1）

法学期刊　法学期刊（10）

专题参考　专题参考（2）

第一千零九十九条

收养三代以内旁系同辈血亲的子女，可以不受本法第一千零九十三条第三项、第一千零九十四条第三项和第一千一百零二条规定的限制。

华侨收养三代以内旁系同辈血亲的子女，还可以不受本法第一千零九十八条第一项规定的限制。

法宝联想

法律法规　规范性文件（1）

司法案例　司法案例（16）

法学期刊　法学期刊（1）

第一千一百条

无子女的收养人可以收养两名子女；有子女的收养人只能收养一名子女。

收养孤儿、残疾未成年人或者儿童福利机构抚养的查找不到生父母的未成年人，可以不受前款和本法第一千零九十八条第一项规定的限制。

法宝联想

司法案例　司法案例（57）

法学期刊　法学期刊（4）

专题参考　专题参考（1）

第一千一百零一条　有配偶者收养子女，应当夫妻共同收养。

法宝联想

法律法规　规范性文件（1）

司法案例　司法案例（24）

法学期刊　法学期刊（6）

第一千一百零二条

无配偶者收养异性子女的，收养人与被收养人的年龄应当相差四十周岁以上。

法宝联想

法律法规　规范性文件（1）

司法案例　司法案例（43）案例报道（1）

法学期刊　法学期刊（4）

专题参考　专题参考（1）

第一千一百零三条

继父或者继母经继子女的生父母同意，可以收养继子女，并可以不受本法第一千零九十三条第三项、第一千零九十四条第三项、第一千零九十八条和第一千一百条第一款规定的限制。

法宝联想

法律法规 规范性文件（1）

司法案例 司法案例（47）

法学期刊 法学期刊（11）

第一千一百零四条

收养人收养与送养人送养，应当双方自愿。收养八周岁以上未成年人的，应当征得被收养人的同意。

法宝联想

法律法规 规范性文件（1）

司法案例 司法案例（36）

法学期刊 法学期刊（11）

专题参考 专题参考（1）

第一千一百零五条

收养应当向县级以上人民政府民政部门登记。收养关系自登记之日起成立。

收养查找不到生父母的未成年人的，办理登记的民政部门应当在登记前予以公告。

收养关系当事人愿意签订收养协议的，可以签订收养协议。

收养关系当事人各方或者一方要求办理收养公证的，应当办理收养公证。

县级以上人民政府民政部门应当依法进行收养评估。

法宝联想

法律法规 规范性文件（3）

司法案例 司法案例（317）案例报道（2）仲裁案例（1）

法学期刊 法学期刊（20）

专题参考 专题参考（1）

第一千一百零六条

收养关系成立后，公安机关应当按照国家有关规定为被收养人办理户口登记。

法宝联想

司法案例　司法案例（32）

法学期刊　法学期刊（4）

第一千一百零七条

孤儿或者生父母无力抚养的子女，可以由生父母的亲属、朋友抚养；抚养人与被抚养人的关系不适用本章规定。

法宝联想

司法案例　司法案例（30）

法学期刊　法学期刊（3）

第一千一百零八条

配偶一方死亡，另一方送养未成年子女的，死亡一方的父母有优先抚养的权利。

法宝联想

法律法规　规范性文件（1）

司法案例　司法案例（58）

法学期刊　法学期刊（2）

第一千一百零九条　外国人依法可以在中华人民共和国收养子女。

外国人在中华人民共和国收养子女，应当经其所在国主管机关依照该国法律审查同意。收养人应当提供由其所在国有权机构出具的有关其年龄、婚姻、职业、财

产、健康、有无受过刑事处罚等状况的证明材料，并与送养人签订书面协议，亲自向省、自治区、直辖市人民政府民政部门登记。

前款规定的证明材料应当经收养人所在国外交机关或者外交机关授权的机构认证，并经中华人民共和国驻该国使领馆认证，但是国家另有规定的除外。

法宝联想
法律法规　规范性文件（1）
司法案例　司法案例（44）
法学期刊　法学期刊（5）
专题参考　专题参考（1）

第一千一百一十条

收养人、送养人要求保守收养秘密的，其他人应当尊重其意愿，不得泄露。

法宝联想
司法案例　司法案例（23）
法学期刊　法学期刊（1）
专题参考　专题参考（1）

第二节　收养的效力

第一千一百一十一条

自收养关系成立之日起，养父母与养子女间的权利义务关系，适用本法关于父母子女关系的规定；养子女与养父母的近亲属间的权利义务关系，适用本法关于子女与父母的近亲属关系的规定。

养子女与生父母以及其他近亲属间的权利义务关系，因收养关系的成立而消除。

法宝联想

法律法规 规范性文件（1）

司法案例 司法案例（427）案例报道（2）

法学期刊 法学期刊（17）

第一千一百一十二条

养子女可以随养父或者养母的姓氏，经当事人协商一致，也可以保留原姓氏。

法宝联想

司法案例 司法案例（56）

法学期刊 法学期刊（3）

专题参考 专题参考（1）

第一千一百一十三条

有本法第一编关于民事法律行为无效规定情形或者违反本编规定的收养行为无效。

无效的收养行为自始没有法律约束力。

法宝联想

法律法规 规范性文件（1）

司法案例 司法案例（312）

法学期刊 法学期刊（10）

专题参考 专题参考（1）

第三节 收养关系的解除

第一千一百一十四条

收养人在被收养人成年以前，不得解除收养关系，但是收养人、送养人双方协议解除的除外。养子女八周岁以上的，应当征得本人同意。

收养人不履行抚养义务，有虐待、遗弃等侵害未成年养子女合法权益行为的，送养人有权要求解除养父母与养子女间的收养关系。送养人、收养人不能达成解除收养关系协议的，可以向人民法院提起诉讼。

法宝联想

司法案例 司法案例（15）

法学期刊 法学期刊（9）

第一千一百一十五条

养父母与成年养子女关系恶化、无法共同生活的，可以协议解除收养关系。不能达成协议的，可以向人民法院提起诉讼。

法宝联想

司法案例 司法案例（180）

法学期刊 法学期刊（8）

第一千一百一十六条

当事人协议解除收养关系的，应当到民政部门办理解除收养关系登记。

法宝联想

司法案例 司法案例（39）

第一千一百一十七条

收养关系解除后，养子女与养父母以及其他近亲属间的权利义务关系即行消除，与生父母以及其他近亲属间的权利义务关系自行恢复。但是，成年养子女与生父母以及其他近亲属间的权利义务关系是否恢复，可以协商确定。

法宝联想

司法案例　司法案例（43）案例报道（1）

法学期刊　法学期刊（5）

第一千一百一十八条

收养关系解除后，经养父母抚养的成年养子女，对缺乏劳动能力又缺乏生活来源的养父母，应当给付生活费。因养子女成年后虐待、遗弃养父母而解除收养关系的，养父母可以要求养子女补偿收养期间支出的抚养费。

生父母要求解除收养关系的，养父母可以要求生父母适当补偿收养期间支出的抚养费；但是，因养父母虐待、遗弃养子女而解除收养关系的除外。

法宝联想

法律法规　规范性文件（1）

司法案例　司法案例（71）案例报道（1）

法学期刊　法学期刊（14）

专题参考　专题参考（1）

第六编　继承

第一章　一般规定

第一千一百一十九条　本编调整因继承产生的民事关系。

法宝联想

司法案例　司法案例（150）

法学期刊　法学期刊（2）

第一千一百二十条　国家保护自然人的继承权。

法宝联想

司法案例　司法案例（1641）

法学期刊　法学期刊（5）

法宝律师　律所实务（1）

第一千一百二十一条　继承从被继承人死亡时开始。

相互有继承关系的数人在同一事件中死亡，难以确定死亡时间的，推定没有其他继承人的人先死亡。都有其他继承人，辈份不同的，推定长辈先死亡；辈份相同的，推定同时死亡，相互不发生继承。

法宝联想

司法案例　司法案例（10791）案例报道（1）

法学期刊　法学期刊（27）

法宝律师　律所实务（4）

专题参考　专题参考（2）

第一千一百二十二条　遗产是自然人死亡时遗留的个人合法财产。

依照法律规定或者根据其性质不得继承的遗产，不得继承。

法宝联想

法律法规　规范性文件（1）

司法案例　司法案例（21440）案例报道（7）仲裁案例（1）

法学期刊　法学期刊（49）

法宝律师　律所实务（4）

专题参考　专题参考（2）

第一千一百二十三条

继承开始后，按照法定继承办理；有遗嘱的，按照遗嘱继承或者遗赠办理；有遗赠扶养协议的，按照协议办理。

法宝联想

法律法规　规范性文件（3）法律动态（2）

司法案例　司法案例（18474）案例报道（4）

法学期刊　法学期刊（23）

法宝律师　律所实务（3）

专题参考　专题参考（3）

第一千一百二十四条

继承开始后，继承人放弃继承的，应当在遗产处理前，以书面形式作出放弃继承的表示；没有表示的，视为接受继承。

受遗赠人应当在知道受遗赠后六十日内，作出接受或者放弃受遗赠的表示；到期没有表示的，视为放弃受遗赠。

法宝联想

法律法规　规范性文件（2）

司法案例　司法案例（9228）案例报道（4）仲裁案例（2）

法学期刊　法学期刊（42）

法宝律师　律所实务（1）

专题参考　专题参考（2）

第一千一百二十五条　继承人有下列行为之一的，丧失继承权：

（一）故意杀害被继承人；

（二）为争夺遗产而杀害其他继承人；

（三）遗弃被继承人，或者虐待被继承人情节严重；

（四）伪造、篡改、隐匿或者销毁遗嘱，情节严重；

（五）以欺诈、胁迫手段迫使或者妨碍被继承人设立、变更或者撤回遗嘱，情节严重。

继承人有前款第三项至第五项行为，确有悔改表现，被继承人表示宽恕或者事后在遗嘱中将其列为继承人的，该继承人不丧失继承权。

受遗赠人有本条第一款规定行为的，丧失受遗赠权。

法宝联想

法律法规　司法解释（2）规范性文件（3）法律动态（4）

司法案例　司法案例（564）案例报道（6）

法学期刊　法学期刊（28）

法宝律师　律所实务（2）

专题参考　专题参考（7）

第二章　法定继承

第一千一百二十六条　继承权男女平等。

法宝联想

法律法规　规范性文件（1）

司法案例　司法案例（2251）案例报道（2）

法学期刊　法学期刊（6）

专题参考　专题参考（1）

第一千一百二十七条　遗产按照下列顺序继承：

（一）第一顺序：配偶、子女、父母；

（二）第二顺序：兄弟姐妹、祖父母、外祖父母。

继承开始后，由第一顺序继承人继承，第二顺序继承人不继承；没有第一顺序继承人继承的，由第二顺序继承人继承。

本编所称子女，包括婚生子女、非婚生子女、养子女和有扶养关系的继子女。

本编所称父母，包括生父母、养父母和有扶养关系的继父母。

本编所称兄弟姐妹，包括同父母的兄弟姐妹、同父异母或者同母异父的兄弟姐妹、养兄弟姐妹、有扶养关系的继兄弟姐妹。

法宝联想

法律法规　司法解释（1）规范性文件（2）

司法案例　司法案例（32177）案例报道（6）

法学期刊　法学期刊（39）

法宝律师　律所实务（6）

专题参考　专题参考（5）

第一千一百二十八条

被继承人的子女先于被继承人死亡的，由被继承人的子女的直系晚辈血亲代位继承。

被继承人的兄弟姐妹先于被继承人死亡的，由被继承人的兄弟姐妹的子女代位继承。

代位继承人一般只能继承被代位继承人有权继承的遗产份额。

法宝联想

法律法规　司法解释（2）规范性文件（5）法律动态（2）

司法案例　司法案例（4174）案例报道（10）

法学期刊　法学期刊（22）

法宝律师　律所实务（2）

专题参考　专题参考（8）

第一千一百二十九条

丧偶儿媳对公婆，丧偶女婿对岳父母，尽了主要赡养义务的，作为第一顺序继承人。

法宝联想

法律法规　司法解释（2）

司法案例　司法案例（361）案例报道（3）

法学期刊　法学期刊（17）

法宝律师　律所实务（1）

专题参考　专题参考（3）

第一千一百三十条　同一顺序继承人继承遗产的份额，一般应当均等。

对生活有特殊困难又缺乏劳动能力的继承人，分配遗产时，应当予以照顾。

对被继承人尽了主要扶养义务或者与被继承人共同生活的继承人，分配遗产时，可以多分。

有扶养能力和有扶养条件的继承人，不尽扶养义务的，分配遗产时，应当不分或者少分。

继承人协商同意的，也可以不均等。

法宝联想

法律法规　司法解释（1）规范性文件（5）法律动态（4）

司法案例　司法案例（15114）案例报道（7）

法学期刊　法学期刊（28）

法宝律师　律所实务（6）

专题参考　专题参考（4）

第一千一百三十一条

对继承人以外的依靠被继承人扶养的人，或者继承人以外的对被继承人扶养较多的人，可以分给适当的遗产。

法宝联想

法律法规　司法解释（1）规范性文件（4）法律动态（3）

司法案例　司法案例（896）案例报道（14）

法学期刊　法学期刊（22）

法宝律师　律所实务（1）

专题参考　专题参考（4）

第一千一百三十二条

继承人应当本着互谅互让、和睦团结的精神，协商处理继承问题。遗产分割的时间、办法和份额，由继承人协商确定；协商不成的，可以由人民调解委员会调解或者向人民法院提起诉讼。

法宝联想

法律法规　规范性文件（1）法律动态（1）

司法案例　司法案例（1870）案例报道（1）

法学期刊　法学期刊（6）

第三章　遗嘱继承和遗赠

第一千一百三十三条

自然人可以依照本法规定立遗嘱处分个人财产，并可以指定遗嘱执行人。

自然人可以立遗嘱将个人财产指定由法定继承人中的一人或者数人继承。

自然人可以立遗嘱将个人财产赠与国家、集体或者法定继承人以外的组织、个人。

自然人可以依法设立遗嘱信托。

法宝联想

法律法规 规范性文件（1）法律动态（1）

司法案例 司法案例（4216）案例报道（4）

法学期刊 法学期刊（56）

专题参考 专题参考（3）

第一千一百三十四条

自书遗嘱由遗嘱人亲笔书写，签名，注明年、月、日。

法宝联想

法律法规 规范性文件（2）法律动态（3）

司法案例 司法案例（2307）案例报道（4）

法学期刊 法学期刊（11）

法宝律师 律所实务（3）

专题参考 专题参考（2）

第一千一百三十五条

代书遗嘱应当有两个以上见证人在场见证，由其中一人代书，并由遗嘱人、代书人和其他见证人签名，注明年、月、日。

法宝联想

法律法规　法律动态（2）

司法案例　司法案例（1899）案例报道（4）

法学期刊　法学期刊（5）

专题参考　专题参考（1）

第一千一百三十六条

打印遗嘱应当有两个以上见证人在场见证。遗嘱人和见证人应当在遗嘱每一页签名，注明年、月、日。

法宝联想

法律法规　司法解释（1）规范性文件（4）法律动态（6）

司法案例　司法案例（1706）案例报道（14）

法学期刊　法学期刊（7）

法宝律师　律所实务（3）

专题参考　专题参考（6）

第一千一百三十七条

以录音录像形式立的遗嘱，应当有两个以上见证人在场见证。遗嘱人和见证人应当在录音录像中记录其姓名或者肖像，以及年、月、日。

法宝联想

法律法规　规范性文件（2）法律动态（1）

司法案例　司法案例（566）案例报道（3）

法学期刊　法学期刊（8）

法宝律师　律所实务（1）

专题参考　专题参考（2）

第一千一百三十八条

遗嘱人在危急情况下，可以立口头遗嘱。口头遗嘱应当有两个以上见证人在场见

证。危急情况消除后，遗嘱人能够以书面或者录音录像形式立遗嘱的，所立的口头遗嘱无效。

法宝联想

司法案例　司法案例（331）

法学期刊　法学期刊（14）

法宝律师　律所实务（1）

第一千一百三十九条　公证遗嘱由遗嘱人经公证机构办理。

法宝联想

法律法规　法律动态（1）

司法案例　司法案例（997）案例报道（1）

法学期刊　法学期刊（8）

法宝律师　律所实务（2）

专题参考　专题参考（2）

第一千一百四十条　下列人员不能作为遗嘱见证人：

（一）无民事行为能力人、限制民事行为能力人以及其他不具有见证能力的人；

（二）继承人、受遗赠人；

（三）与继承人、受遗赠人有利害关系的人。

法宝联想

法律法规　规范性文件（1）法律动态（1）

司法案例　司法案例（582）案例报道（3）

法学期刊　法学期刊（7）

法宝律师　律所实务（1）

专题参考　专题参考（1）

第一千一百四十一条

遗嘱应当为缺乏劳动能力又没有生活来源的继承人保留必要的遗产份额。

法宝联想

法律法规 规范性文件（4）法律动态（4）

司法案例 司法案例（298）案例报道（6）

法学期刊 法学期刊（26）

法宝律师 律所实务（2）

专题参考 专题参考（2）

第一千一百四十二条 遗嘱人可以撤回、变更自己所立的遗嘱。

立遗嘱后，遗嘱人实施与遗嘱内容相反的民事法律行为的，视为对遗嘱相关内容的撤回。

立有数份遗嘱，内容相抵触的，以最后的遗嘱为准。

法宝联想

法律法规 司法解释（1）规范性文件（1）法律动态（1）

司法案例 司法案例（683）案例报道（1）

法学期刊 法学期刊（24）

法宝律师 律所实务（1）

专题参考 专题参考（4）

第一千一百四十三条

无民事行为能力人或者限制民事行为能力人所立的遗嘱无效。

遗嘱必须表示遗嘱人的真实意思，受欺诈、胁迫所立的遗嘱无效。

伪造的遗嘱无效。

遗嘱被篡改的，篡改的内容无效。

法宝联想

司法案例 司法案例（323）案例报道（1）

法学期刊 法学期刊（16）

法宝律师 律所实务（1）

专题参考 专题参考（2）

第一千一百四十四条

遗嘱继承或者遗赠附有义务的，继承人或者受遗赠人应当履行义务。没有正当理由不履行义务的，经利害关系人或者有关组织请求，人民法院可以取消其接受附义务部分遗产的权利。

法宝联想

法律法规 法律动态（1）

司法案例 司法案例（178）案例报道（1）

法学期刊 法学期刊（12）

专题参考 专题参考（1）

第四章 遗产的处理

第一千一百四十五条

继承开始后，遗嘱执行人为遗产管理人；没有遗嘱执行人的，继承人应当及时推选遗产管理人；继承人未推选的，由继承人共同担任遗产管理人；没有继承人或者继承人均放弃继承的，由被继承人生前住所地的民政部门或者村民委员会担任遗产管理人。

法宝联想

法律法规 规范性文件（12）法律动态（3）

司法案例 司法案例（1761）案例报道（7）仲裁案例（1）

法学期刊 法学期刊（49）

法宝律师 律所实务（6）

专题参考　专题参考（4）

第一千一百四十六条

对遗产管理人的确定有争议的，利害关系人可以向人民法院申请指定遗产管理人

。

法宝联想

法律法规　规范性文件（4）法律动态（1）

司法案例　司法案例（314）案例报道（5）

法学期刊　法学期刊（22）

法宝律师　律所实务（7）

专题参考　专题参考（1）

第一千一百四十七条　遗产管理人应当履行下列职责：

（一）清理遗产并制作遗产清单；

（二）向继承人报告遗产情况；

（三）采取必要措施防止遗产毁损、灭失；

（四）处理被继承人的债权债务；

（五）按照遗嘱或者依照法律规定分割遗产；

（六）实施与管理遗产有关的其他必要行为。

法宝联想

法律法规　规范性文件（5）法律动态（1）

司法案例　司法案例（1232）案例报道（4）

法学期刊　法学期刊（41）

法宝律师　律所实务（4）

专题参考　专题参考（2）

第一千一百四十八条

遗产管理人应当依法履行职责，因故意或者重大过失造成继承人、受遗赠人、债权人损害的，应当承担民事责任。

法宝联想

法律法规　规范性文件（1）

司法案例　司法案例（158）

法学期刊　法学期刊（25）

专题参考　专题参考（2）

第一千一百四十九条

遗产管理人可以依照法律规定或者按照约定获得报酬。

法宝联想

法律法规　规范性文件（4）

司法案例　司法案例（51）

法学期刊　法学期刊（13）

第一千一百五十条

继承开始后，知道被继承人死亡的继承人应当及时通知其他继承人和遗嘱执行人。继承人中无人知道被继承人死亡或者知道被继承人死亡而不能通知的，由被继承人生前所在单位或者住所地的居民委员会、村民委员会负责通知。

法宝联想

法律法规　规范性文件（1）

司法案例　司法案例（47）

法学期刊　法学期刊（5）

第一千一百五十一条

存有遗产的人，应当妥善保管遗产，任何组织或者个人不得侵吞或者争抢。

法宝联想

司法案例 司法案例（422）

法学期刊 法学期刊（7）

法宝律师 律所实务（2）

第一千一百五十二条

继承开始后，继承人于遗产分割前死亡，并没有放弃继承的，该继承人应当继承的遗产转给其继承人，但是遗嘱另有安排的除外。

法宝联想

司法案例 司法案例（1779）

法学期刊 法学期刊（15）

专题参考 专题参考（1）

第一千一百五十三条

夫妻共同所有的财产，除有约定的外，遗产分割时，应当先将共同所有的财产的一半分出为配偶所有，其余的为被继承人的遗产。

遗产在家庭共有财产之中的，遗产分割时，应当先分出他人的财产。

法宝联想

法律法规 规范性文件（1）

司法案例 司法案例（6121）

法学期刊 法学期刊（17）

法宝律师 律所实务（2）

专题参考 专题参考（1）

第一千一百五十四条

有下列情形之一的，遗产中的有关部分按照法定继承办理：

（一）遗嘱继承人放弃继承或者受遗赠人放弃受遗赠；

（二）遗嘱继承人丧失继承权或者受遗赠人丧失受遗赠权；

（三）遗嘱继承人、受遗赠人先于遗嘱人死亡或者终止；

（四）遗嘱无效部分所涉及的遗产；

（五）遗嘱未处分的遗产。

法宝联想

司法案例　司法案例（509）

法学期刊　法学期刊（8）

法宝律师　律所实务（1）

第一千一百五十五条

遗产分割时，应当保留胎儿的继承份额。胎儿娩出时是死体的，保留的份额按照法定继承办理。

法宝联想

司法案例　司法案例（20）

法学期刊　法学期刊（11）

专题参考　专题参考（2）

第一千一百五十六条

遗产分割应当有利于生产和生活需要，不损害遗产的效用。

不宜分割的遗产，可以采取折价、适当补偿或者共有等方法处理。

法宝联想

司法案例　司法案例（2572）

法学期刊　法学期刊（7）

第一千一百五十七条

夫妻一方死亡后另一方再婚的，有权处分所继承的财产，任何组织或者个人不得干涉。

法宝联想

司法案例　司法案例（43）

法学期刊　法学期刊（2）

第一千一百五十八条

自然人可以与继承人以外的组织或者个人签订遗赠扶养协议。按照协议，该组织或者个人承担该自然人生养死葬的义务，享有受遗赠的权利。

法宝联想

法律法规　规范性文件（4）法律动态（4）

司法案例　司法案例（393）案例报道（12）

法学期刊　法学期刊（17）

法宝律师　律所实务（2）

专题参考　专题参考（5）

第一千一百五十九条

分割遗产，应当清偿被继承人依法应当缴纳的税款和债务；但是，应当为缺乏劳动能力又没有生活来源的继承人保留必要的遗产。

法宝联想

法律法规　规范性文件（3）法律动态（1）

司法案例　司法案例（5327）案例报道（4）

法学期刊　法学期刊（18）

法宝律师　律所实务（2）

第一千一百六十条

无人继承又无人受遗赠的遗产，归国家所有，用于公益事业；死者生前是集体所有制组织成员的，归所在集体所有制组织所有。

法宝联想

法律法规　规范性文件（1）

司法案例　司法案例（416）案例报道（4）

法学期刊　法学期刊（27）

法宝律师　律所实务（1）

专题参考　专题参考（4）

第一千一百六十一条

继承人以所得遗产实际价值为限清偿被继承人依法应当缴纳的税款和债务。超过遗产实际价值部分，继承人自愿偿还的不在此限。

继承人放弃继承的，对被继承人依法应当缴纳的税款和债务可以不负清偿责任。

法宝联想

法律法规　规范性文件（2）法律动态（1）

司法案例　司法案例（23005）案例报道（6）仲裁案例（1）

法学期刊　法学期刊（21）

法宝律师　律所实务（5）

专题参考　专题参考（1）

第一千一百六十二条

执行遗赠不得妨碍清偿遗赠人依法应当缴纳的税款和债务。

法宝联想

司法案例　司法案例（137）案例报道（1）

法学期刊　法学期刊（12）

法宝律师　律所实务（3）

第一千一百六十三条

既有法定继承又有遗嘱继承、遗赠的，由法定继承人清偿被继承人依法应当缴纳的税款和债务；超过法定继承遗产实际价值部分，由遗嘱继承人和受遗赠人按比例以所得遗产清偿。

法宝联想

司法案例　司法案例（235）

法学期刊　法学期刊（14）

法宝律师　律所实务（2）

第七编　侵权责任

第一章　一般规定

第一千一百六十四条　本编调整因侵害民事权益产生的民事关系。

法宝联想

司法案例　司法案例（3985）

法学期刊　法学期刊（69）

法宝律师　律所实务（1）

专题参考　专题参考（3）

第一千一百六十五条

行为人因过错侵害他人民事权益造成损害的，应当承担侵权责任。

依照法律规定推定行为人有过错，其不能证明自己没有过错的，应当承担侵权责任。

法宝联想

法律法规　司法解释（1）规范性文件（18）法规解读（1）法律动态（10）

司法案例　司法案例（386168）案例报道（75）

法学期刊　法学期刊（531）

法宝律师　律所实务（29）

专题参考　专题参考（32）

第一千一百六十六条

行为人造成他人民事权益损害，不论行为人有无过错，法律规定应当承担侵权责任的，依照其规定。

法宝联想

司法案例　司法案例（6207）

法学期刊　法学期刊（90）

法宝律师　律所实务（7）

专题参考　专题参考（4）

第一千一百六十七条

侵权行为危及他人人身、财产安全的，被侵权人有权请求侵权人承担停止侵害、排除妨碍、消除危险等侵权责任。

法宝联想

法律法规　规范性文件（5）法律动态（3）

司法案例　司法案例（8775）案例报道（7）

法学期刊　法学期刊（118）

法宝律师　律所实务（5）

专题参考　专题参考（5）

第一千一百六十八条

二人以上共同实施侵权行为，造成他人损害的，应当承担连带责任。

法宝联想

法律法规 司法解释（7）规范性文件（10）法律动态（7）

司法案例 司法案例（10774）案例报道（12）

法学期刊 法学期刊（188）

法宝律师 律所实务（18）

专题参考 专题参考（23）

第一千一百六十九条

教唆、帮助他人实施侵权行为的，应当与行为人承担连带责任。

教唆、帮助无民事行为能力人、限制民事行为能力人实施侵权行为的，应当承担侵权责任；该无民事行为能力人、限制民事行为能力人的监护人未尽到监护职责的，应当承担相应的责任。

法宝联想

法律法规 司法解释（8）规范性文件（2）法规解读（1）法律动态（4）

司法案例 司法案例（876）

法学期刊 法学期刊（110）

法宝律师 律所实务（10）

专题参考 专题参考（10）

第一千一百七十条

二人以上实施危及他人人身、财产安全的行为，其中一人或者数人的行为造成他人损害，能够确定具体侵权人的，由侵权人承担责任；不能确定具体侵权人的，行为人承担连带责任。

法宝联想

法律法规 司法解释（3）规范性文件（1）

司法案例 司法案例（2010）案例报道（1）

法学期刊 法学期刊（73）

法宝律师 律所实务（1）

专题参考 专题参考（5）

第一千一百七十一条

二人以上分别实施侵权行为造成同一损害，每个人的侵权行为都足以造成全部损害的，行为人承担连带责任。

法宝联想

法律法规 司法解释（5）规范性文件（4）白皮书（1）法律动态（2）

司法案例 司法案例（1070）案例报道（2）

法学期刊 法学期刊（97）

法宝律师 律所实务（5）

专题参考 专题参考（12）

第一千一百七十二条

二人以上分别实施侵权行为造成同一损害，能够确定责任大小的，各自承担相应的责任；难以确定责任大小的，平均承担责任。

法宝联想

法律法规 司法解释（4）规范性文件（7）法律动态（3）

司法案例 司法案例（11622）案例报道（9）

法学期刊 法学期刊（122）

法宝律师 律所实务（4）

专题参考 专题参考（11）

第一千一百七十三条

被侵权人对同一损害的发生或者扩大有过错的，可以减轻侵权人的责任。

法宝联想

法律法规 规范性文件（8）法律动态（2）

司法案例 司法案例（81550）案例报道（33）

法学期刊 法学期刊（90）

法宝律师 律所实务（6）

专题参考 专题参考（2）

第一千一百七十四条 损害是因受害人故意造成的，行为人不承担责任。

法宝联想

法律法规 规范性文件（2）

司法案例 司法案例（1269）案例报道（2）

法学期刊 法学期刊（32）

法宝律师 律所实务（1）

专题参考 专题参考（1）

第一千一百七十五条 损害是因第三人造成的，第三人应当承担侵权责任。

法宝联想

法律法规 规范性文件（1）法律动态（1）

司法案例 司法案例（1307）案例报道（1）

法学期刊 法学期刊（25）

专题参考 专题参考（1）

第一千一百七十六条

自愿参加具有一定风险的文体活动，因其他参加者的行为受到损害的，受害人不

得请求其他参加者承担侵权责任；但是，其他参加者对损害的发生有故意或者重大过失的除外。

活动组织者的责任适用本法第一千一百九十八条至第一千二百零一条的规定。

法宝联想

法律法规　司法解释（1）规范性文件（10）法律动态（8）

司法案例　司法案例（1329）案例报道（32）

法学期刊　法学期刊（63）

法宝律师　律所实务（3）

专题参考　专题参考（6）

第一千一百七十七条

合法权益受到侵害，情况紧迫且不能及时获得国家机关保护，不立即采取措施将使其合法权益受到难以弥补的损害的，受害人可以在保护自己合法权益的必要范围内采取扣留侵权人的财物等合理措施；但是，应当立即请求有关国家机关处理。

受害人采取的措施不当造成他人损害的，应当承担侵权责任。

法宝联想

法律法规　司法解释（1）

司法案例　司法案例（705）案例报道（4）

法学期刊　法学期刊（42）

专题参考　专题参考（8）

第一千一百七十八条

本法和其他法律对不承担责任或者减轻责任的情形另有规定的，依照其规定。

法宝联想

司法案例　司法案例（252）

法学期刊　法学期刊（6）

专题参考　专题参考（1）

第二章　损害赔偿

第一千一百七十九条

侵害他人造成人身损害的，应当赔偿医疗费、护理费、交通费、营养费、住院伙食补助费等为治疗和康复支出的合理费用，以及因误工减少的收入。造成残疾的，还应当赔偿辅助器具费和残疾赔偿金；造成死亡的，还应当赔偿丧葬费和死亡赔偿金。

法宝联想

法律法规　司法解释（3）规范性文件（7）法规解读（1）法律动态（4）

司法案例　司法案例（440690）案例报道（23）仲裁案例（1）

法学期刊　法学期刊（90）

法宝律师　律所实务（8）

专题参考　专题参考（6）

第一千一百八十条

因同一侵权行为造成多人死亡的，可以以相同数额确定死亡赔偿金。

法宝联想

法律法规　规范性文件（1）

司法案例　司法案例（387）

法学期刊　法学期刊（4）

第一千一百八十一条

被侵权人死亡的，其近亲属有权请求侵权人承担侵权责任。被侵权人为组织，该组织分立、合并的，承继权利的组织有权请求侵权人承担侵权责任。

被侵权人死亡的，支付被侵权人医疗费、丧葬费等合理费用的人有权请求侵权人赔偿费用，但是侵权人已经支付该费用的除外。

法宝联想

司法案例　司法案例（15499）案例报道（4）

法学期刊　法学期刊（23）

法宝律师　律所实务（2）

专题参考　专题参考（2）

第一千一百八十二条

侵害他人人身权益造成财产损失的，按照被侵权人因此受到的损失或者侵权人因此获得的利益赔偿；被侵权人因此受到的损失以及侵权人因此获得的利益难以确定，被侵权人和侵权人就赔偿数额协商不一致，向人民法院提起诉讼的，由人民法院根据实际情况确定赔偿数额。

法宝联想

法律法规　司法解释（3）规范性文件（4）法律动态（2）

司法案例　司法案例（26394）案例报道（6）

法学期刊　法学期刊（185）

法宝律师　律所实务（1）

专题参考　专题参考（21）

第一千一百八十三条

侵害自然人人身权益造成严重精神损害的，被侵权人有权请求精神损害赔偿。

因故意或者重大过失侵害自然人具有人身意义的特定物造成严重精神损害的，被侵权人有权请求精神损害赔偿。

法宝联想

法律法规　司法解释（4）规范性文件（9）法规解读（1）法律动态（4）

司法案例　司法案例（147499）案例报道（15）仲裁案例（1）

法学期刊　法学期刊（231）

法宝律师　律所实务（9）

专题参考　专题参考（17）

第一千一百八十四条

侵害他人财产的，财产损失按照损失发生时的市场价格或者其他合理方式计算。

法宝联想

法律法规　司法解释（3）规范性文件（1）法规解读（1）法律动态（3）

司法案例　司法案例（100540）案例报道（8）

法学期刊　法学期刊（101）

法宝律师　律所实务（4）

专题参考　专题参考（7）

第一千一百八十五条

故意侵害他人知识产权，情节严重的，被侵权人有权请求相应的惩罚性赔偿。

法宝联想

法律法规　司法解释（2）规范性文件（4）法律动态（1）

司法案例　司法案例（627）案例报道（6）

法学期刊　法学期刊（116）

法宝律师　律所实务（4）

专题参考　专题参考（9）

第一千一百八十六条

受害人和行为人对损害的发生都没有过错的，依照法律的规定由双方分担损失。

法宝联想

法律法规　司法解释（1）规范性文件（1）法律动态（1）

司法案例　司法案例（2362）案例报道（1）

法学期刊　法学期刊（86）

法宝律师　律所实务（3）

专题参考　专题参考（7）

第一千一百八十七条

损害发生后，当事人可以协商赔偿费用的支付方式。协商不一致的，赔偿费用应当一次性支付；一次性支付确有困难的，可以分期支付，但是被侵权人有权请求提供相应的担保。

法宝联想

司法案例　司法案例（4555）

法学期刊　法学期刊（12）

第三章　责任主体的特殊规定

第一千一百八十八条

无民事行为能力人、限制民事行为能力人造成他人损害的，由监护人承担侵权责任。监护人尽到监护职责的，可以减轻其侵权责任。

有财产的无民事行为能力人、限制民事行为能力人造成他人损害的，从本人财产中支付赔偿费用；不足部分，由监护人赔偿。

法宝联想

法律法规　司法解释（1）规范性文件（2）法规解读（1）法律动态（3）

司法案例　司法案例（11056）案例报道（17）

法学期刊　法学期刊（72）

法宝律师　律所实务（4）

专题参考　专题参考（3）

第一千一百八十九条

无民事行为能力人、限制民事行为能力人造成他人损害，监护人将监护职责委托给他人的，监护人应当承担侵权责任；受托人有过错的，承担相应的责任。

法宝联想

法律法规　司法解释（1）法规解读（1）

司法案例　司法案例（271）案例报道（1）

法学期刊　法学期刊（34）

法宝律师　律所实务（1）

专题参考　专题参考（1）

第一千一百九十条

完全民事行为能力人对自己的行为暂时没有意识或者失去控制造成他人损害有过错的，应当承担侵权责任；没有过错的，根据行为人的经济状况对受害人适当补偿。

完全民事行为能力人因醉酒、滥用麻醉药品或者精神药品对自己的行为暂时没有意识或者失去控制造成他人损害的，应当承担侵权责任。

法宝联想

司法案例　司法案例（275）

法学期刊　法学期刊（27）

第一千一百九十一条

用人单位的工作人员因执行工作任务造成他人损害的，由用人单位承担侵权责任。用人单位承担侵权责任后，可以向有故意或者重大过失的工作人员追偿。

劳务派遣期间，被派遣的工作人员因执行工作任务造成他人损害的，由接受劳务派遣的用工单位承担侵权责任；劳务派遣单位有过错的，承担相应的责任。

法宝联想
法律法规 司法解释（1）规范性文件（4）法规解读（1）法律动态（1）
司法案例 司法案例（26000）案例报道（18）仲裁案例（1）
法学期刊 法学期刊（143）
法宝律师 律所实务（22）
专题参考 专题参考（14）

第一千一百九十二条

个人之间形成劳务关系，提供劳务一方因劳务造成他人损害的，由接受劳务一方承担侵权责任。接受劳务一方承担侵权责任后，可以向有故意或者重大过失的提供劳务一方追偿。提供劳务一方因劳务受到损害的，根据双方各自的过错承担相应的责任。

提供劳务期间，因第三人的行为造成提供劳务一方损害的，提供劳务一方有权请求第三人承担侵权责任，也有权请求接受劳务一方给予补偿。接受劳务一方补偿后，可以向第三人追偿。

法宝联想
法律法规 规范性文件（1）
司法案例 司法案例（58262）案例报道（12）

法学期刊　法学期刊（73）

法宝律师　律所实务（4）

专题参考　专题参考（4）

第一千一百九十三条

承揽人在完成工作过程中造成第三人损害或者自己损害的，定作人不承担侵权责任。但是，定作人对定作、指示或者选任有过错的，应当承担相应的责任。

法宝联想

法律法规　司法解释（1）法律动态（1）

司法案例　司法案例（12347）案例报道（6）

法学期刊　法学期刊（41）

法宝律师　律所实务（4）

专题参考　专题参考（4）

第一千一百九十四条

网络用户、网络服务提供者利用网络侵害他人民事权益的，应当承担侵权责任。法律另有规定的，依照其规定。

法宝联想

法律法规　规范性文件（5）法律动态（1）

司法案例　司法案例（463）案例报道（3）

法学期刊　法学期刊（94）

法宝律师　律所实务（3）

专题参考　专题参考（8）

第一千一百九十五条

网络用户利用网络服务实施侵权行为的，权利人有权通知网络服务提供者采取删

除、屏蔽、断开链接等必要措施。通知应当包括构成侵权的初步证据及权利人的真实身份信息。

网络服务提供者接到通知后，应当及时将该通知转送相关网络用户，并根据构成侵权的初步证据和服务类型采取必要措施；未及时采取必要措施的，对损害的扩大部分与该网络用户承担连带责任。

权利人因错误通知造成网络用户或者网络服务提供者损害的，应当承担侵权责任。法律另有规定的，依照其规定。

法宝联想
法律法规 司法解释（3）规范性文件（8）法律动态（2）
司法案例 司法案例（1096）案例报道（13）
法学期刊 法学期刊（249）
法宝律师 律所实务（10）
专题参考 专题参考（19）

第一千一百九十六条

网络用户接到转送的通知后，可以向网络服务提供者提交不存在侵权行为的声明。声明应当包括不存在侵权行为的初步证据及网络用户的真实身份信息。

网络服务提供者接到声明后，应当将该声明转送发出通知的权利人，并告知其可以向有关部门投诉或者向人民法院提起诉讼。网络服务提供者在转送声明到达权利人后的合理期限内，未收到权利人已经投诉或者提起诉讼通知的，应当及时终止所采取的措施。

法宝联想

法律法规　司法解释（1）规范性文件（2）

司法案例　司法案例（50）案例报道（2）

法学期刊　法学期刊（72）

法宝律师　律所实务（1）

专题参考　专题参考（6）

第一千一百九十七条

网络服务提供者知道或者应当知道网络用户利用其网络服务侵害他人民事权益，未采取必要措施的，与该网络用户承担连带责任。

法宝联想

法律法规　司法解释（3）规范性文件（6）法律动态（3）

司法案例　司法案例（642）案例报道（6）

法学期刊　法学期刊（166）

法宝律师　律所实务（8）

专题参考　专题参考（22）

第一千一百九十八条

宾馆、商场、银行、车站、机场、体育场馆、娱乐场所等经营场所、公共场所的经营者、管理者或者群众性活动的组织者，未尽到安全保障义务，造成他人损害的，应当承担侵权责任。

因第三人的行为造成他人损害的，由第三人承担侵权责任；经营者、管理者或者组织者未尽到安全保障义务的，承担相应的补充责任。经营者、管理者或者组织者承担补充责任后，可以向第三人追偿。

法宝联想

法律法规　司法解释（1）规范性文件（14）法规解读（1）法律动态（2）

司法案例　司法案例（12071）案例报道（81）

法学期刊 法学期刊（188）

法宝律师 律所实务（20）

专题参考 专题参考（16）

第一千一百九十九条

无民事行为能力人在幼儿园、学校或者其他教育机构学习、生活期间受到人身损害的，幼儿园、学校或者其他教育机构应当承担侵权责任；但是，能够证明尽到教育、管理职责的，不承担侵权责任。

法宝联想

法律法规 规范性文件（6）法律动态（1）

司法案例 司法案例（1518）案例报道（11）

法学期刊 法学期刊（25）

法宝律师 律所实务（4）

第一千二百条

限制民事行为能力人在学校或者其他教育机构学习、生活期间受到人身损害，学校或者其他教育机构未尽到教育、管理职责的，应当承担侵权责任。

法宝联想

法律法规 规范性文件（13）法律动态（2）

司法案例 司法案例（3630）案例报道（18）

法学期刊 法学期刊（10）

法宝律师 律所实务（2）

第一千二百零一条

无民事行为能力人或者限制民事行为能力人在幼儿园、学校或者其他教育机构学习、生活期间，受到幼儿园、学校或者其他教育机构以外的第三人人身损害的，

由第三人承担侵权责任；幼儿园、学校或者其他教育机构未尽到管理职责的，承担相应的补充责任。幼儿园、学校或者其他教育机构承担补充责任后，可以向第三人追偿。

法宝联想

法律法规 司法解释（1）法规解读（1）

司法案例 司法案例（484）案例报道（2）

法学期刊 法学期刊（42）

法宝律师 律所实务（2）

专题参考 专题参考（3）

第四章　产品责任

第一千二百零二条

因产品存在缺陷造成他人损害的，生产者应当承担侵权责任。

法宝联想

法律法规 司法解释（1）规范性文件（4）法规解读（1）法律动态（2）

司法案例 司法案例（1711）案例报道（6）

法学期刊 法学期刊（38）

法宝律师 律所实务（4）

专题参考 专题参考（2）

第一千二百零三条

因产品存在缺陷造成他人损害的，被侵权人可以向产品的生产者请求赔偿，也可以向产品的销售者请求赔偿。

产品缺陷由生产者造成的，销售者赔偿后，有权向生产者追偿。因销售者的过错使产品存在缺陷的，生产者赔偿后，有权向销售者追偿。

法宝联想

法律法规 司法解释（1）规范性文件（3）法规解读（1）

司法案例 司法案例（2131）案例报道（11）

法学期刊 法学期刊（45）

法宝律师 律所实务（3）

专题参考 专题参考（7）

第一千二百零四条

因运输者、仓储者等第三人的过错使产品存在缺陷，造成他人损害的，产品的生产者、销售者赔偿后，有权向第三人追偿。

法宝联想

司法案例 司法案例（32）案例报道（1）

法学期刊 法学期刊（15）

第一千二百零五条

因产品缺陷危及他人人身、财产安全的，被侵权人有权请求生产者、销售者承担停止侵害、排除妨碍、消除危险等侵权责任。

法宝联想

司法案例 司法案例（189）

法学期刊 法学期刊（6）

专题参考 专题参考（3）

第一千二百零六条

产品投入流通后发现存在缺陷的，生产者、销售者应当及时采取停止销售、警示、召回等补救措施；未及时采取补救措施或者补救措施不力造成损害扩大的，对扩大的损害也应当承担侵权责任。

依据前款规定采取召回措施的，生产者、销售者应当负担被侵权人因此支出的必要费用。

法宝联想

司法案例　司法案例（178）

法学期刊　法学期刊（22）

法宝律师　律所实务（1）

第一千二百零七条

明知产品存在缺陷仍然生产、销售，或者没有依据前条规定采取有效补救措施，造成他人死亡或者健康严重损害的，被侵权人有权请求相应的惩罚性赔偿。

法宝联想

法律法规　规范性文件（1）

司法案例　司法案例（393）

法学期刊　法学期刊（85）

法宝律师　律所实务（1）

专题参考　专题参考（2）

第五章　机动车交通事故责任

第一千二百零八条

机动车发生交通事故造成损害的，依照道路交通安全法律和本法的有关规定承担赔偿责任。

法宝联想

法律法规　草案（1）法律动态（1）

司法案例　司法案例（271450）案例报道（1）

法学期刊　法学期刊（10）

法宝律师　律所实务（2）

专题参考　专题参考（2）

第一千二百零九条

因租赁、借用等情形机动车所有人、管理人与使用人不是同一人时，发生交通事故造成损害，属于该机动车一方责任的，由机动车使用人承担赔偿责任；机动车所有人、管理人对损害的发生有过错的，承担相应的赔偿责任。

法宝联想

法律法规　司法解释（2）规范性文件（1）

司法案例　司法案例（28236）案例报道（6）

法学期刊　法学期刊（19）

法宝律师　律所实务（1）

专题参考　专题参考（4）

第一千二百一十条

当事人之间已经以买卖或者其他方式转让并交付机动车但是未办理登记，发生交通事故造成损害，属于该机动车一方责任的，由受让人承担赔偿责任。

法宝联想

司法案例　司法案例（3767）

法学期刊　法学期刊（4）

专题参考 专题参考（4）

第一千二百一十一条

以挂靠形式从事道路运输经营活动的机动车，发生交通事故造成损害，属于该机动车一方责任的，由挂靠人和被挂靠人承担连带责任。

法宝联想

法律法规 规范性文件（1）

司法案例 司法案例（15281）案例报道（3）

法学期刊 法学期刊（8）

法宝律师 律所实务（1）

专题参考 专题参考（3）

第一千二百一十二条

未经允许驾驶他人机动车，发生交通事故造成损害，属于该机动车一方责任的，由机动车使用人承担赔偿责任；机动车所有人、管理人对损害的发生有过错的，承担相应的赔偿责任，但是本章另有规定的除外。

法宝联想

司法案例 司法案例（1626）案例报道（2）

法学期刊 法学期刊（8）

专题参考 专题参考（4）

第一千二百一十三条

机动车发生交通事故造成损害，属于该机动车一方责任的，先由承保机动车强制保险的保险人在强制保险责任限额范围内予以赔偿；不足部分，由承保机动车商业保险的保险人按照保险合同的约定予以赔偿；仍然不足或者没有投保机动车商业保险的，由侵权人赔偿。

法宝联想

法律法规　司法解释（2）规范性文件（3）法律动态（3）

司法案例　司法案例（336559）案例报道（16）

法学期刊　法学期刊（11）

法宝律师　律所实务（2）

专题参考　专题参考（2）

第一千二百一十四条

以买卖或者其他方式转让拼装或者已经达到报废标准的机动车，发生交通事故造成损害的，由转让人和受让人承担连带责任。

法宝联想

司法案例　司法案例（243）

法学期刊　法学期刊（11）

专题参考　专题参考（3）

第一千二百一十五条

盗窃、抢劫或者抢夺的机动车发生交通事故造成损害的，由盗窃人、抢劫人或者抢夺人承担赔偿责任。盗窃人、抢劫人或者抢夺人与机动车使用人不是同一人，发生交通事故造成损害，属于该机动车一方责任的，由盗窃人、抢劫人或者抢夺人与机动车使用人承担连带责任。

保险人在机动车强制保险责任限额范围内垫付抢救费用的，有权向交通事故责任人追偿。

法宝联想

司法案例　司法案例（223）

法学期刊　法学期刊（11）

专题参考　专题参考（5）

第一千二百一十六条

机动车驾驶人发生交通事故后逃逸，该机动车参加强制保险的，由保险人在机动车强制保险责任限额范围内予以赔偿；机动车不明、该机动车未参加强制保险或者抢救费用超过机动车强制保险责任限额，需要支付被侵权人人身伤亡的抢救、丧葬等费用的，由道路交通事故社会救助基金垫付。道路交通事故社会救助基金垫付后，其管理机构有权向交通事故责任人追偿。

法宝联想

司法案例　司法案例（3675）

法学期刊　法学期刊（8）

专题参考　专题参考（1）

第一千二百一十七条

非营运机动车发生交通事故造成无偿搭乘人损害，属于该机动车一方责任的，应当减轻其赔偿责任，但是机动车使用人有故意或者重大过失的除外。

法宝联想

法律法规　司法解释（1）规范性文件（8）法律动态（2）

司法案例　司法案例（3791）案例报道（28）

法学期刊　法学期刊（15）

专题参考　专题参考（5）

第六章　医疗损害责任

第一千二百一十八条

患者在诊疗活动中受到损害，医疗机构或者其医务人员有过错的，由医疗机构承担赔偿责任。

法宝联想

法律法规　司法解释（1）规范性文件（2）法律动态（1）

司法案例　司法案例（9027）案例报道（3）

法学期刊　法学期刊（27）

法宝律师　律所实务（5）

专题参考　专题参考（3）

第一千二百一十九条

医务人员在诊疗活动中应当向患者说明病情和医疗措施。需要实施手术、特殊检查、特殊治疗的，医务人员应当及时向患者具体说明医疗风险、替代医疗方案等情况，并取得其明确同意；不能或者不宜向患者说明的，应当向患者的近亲属说明，并取得其明确同意。

医务人员未尽到前款义务，造成患者损害的，医疗机构应当承担赔偿责任。

法宝联想

法律法规　司法解释（1）规范性文件（1）法律动态（1）

司法案例　司法案例（1099）

法学期刊　法学期刊（46）

法宝律师　律所实务（4）

专题参考　专题参考（1）

第一千二百二十条

因抢救生命垂危的患者等紧急情况，不能取得患者或者其近亲属意见的，经医疗机构负责人或者授权的负责人批准，可以立即实施相应的医疗措施。

法宝联想

法律法规　司法解释（1）规范性文件（1）

司法案例　司法案例（53）

法学期刊　法学期刊（11）

法宝律师　律所实务（1）

第一千二百二十一条

医务人员在诊疗活动中未尽到与当时的医疗水平相应的诊疗义务，造成患者损害的，医疗机构应当承担赔偿责任。

法宝联想

司法案例　司法案例（1407）案例报道（1）

法学期刊　法学期刊（24）

法宝律师　律所实务（1）

专题参考　专题参考（1）

第一千二百二十二条

患者在诊疗活动中受到损害，有下列情形之一的，推定医疗机构有过错：

（一）违反法律、行政法规、规章以及其他有关诊疗规范的规定；

（二）隐匿或者拒绝提供与纠纷有关的病历资料；

（三）遗失、伪造、篡改或者违法销毁病历资料。

法宝联想

法律法规　司法解释（1）规范性文件（1）法律动态（1）

司法案例　司法案例（1052）

法学期刊　法学期刊（49）

法宝律师　律所实务（2）

专题参考　专题参考（2）

第一千二百二十三条

因药品、消毒产品、医疗器械的缺陷，或者输入不合格的血液造成患者损害的，患者可以向药品上市许可持有人、生产者、血液提供机构请求赔偿，也可以向医疗机构请求赔偿。患者向医疗机构请求赔偿的，医疗机构赔偿后，有权向负有责任的药品上市许可持有人、生产者、血液提供机构追偿。

法宝联想

法律法规 司法解释（1）

司法案例 司法案例（238）

法学期刊 法学期刊（24）

法宝律师 律所实务（1）

专题参考 专题参考（2）

第一千二百二十四条

患者在诊疗活动中受到损害，有下列情形之一的，医疗机构不承担赔偿责任：

（一）患者或者其近亲属不配合医疗机构进行符合诊疗规范的诊疗；

（二）医务人员在抢救生命垂危的患者等紧急情况下已经尽到合理诊疗义务；

（三）限于当时的医疗水平难以诊疗。

前款第一项情形中，医疗机构或者其医务人员也有过错的，应当承担相应的赔偿责任。

法宝联想

法律法规 司法解释（1）

司法案例 司法案例（446）

法学期刊 法学期刊（10）

第一千二百二十五条

医疗机构及其医务人员应当按照规定填写并妥善保管住院志、医嘱单、检验报告、手术及麻醉记录、病理资料、护理记录等病历资料。

患者要求查阅、复制前款规定的病历资料的，医疗机构应当及时提供。

法宝联想

司法案例　司法案例（124）

法学期刊　法学期刊（11）

第一千二百二十六条

医疗机构及其医务人员应当对患者的隐私和个人信息保密。泄露患者的隐私和个人信息，或者未经患者同意公开其病历资料的，应当承担侵权责任。

法宝联想

司法案例　司法案例（28）

法学期刊　法学期刊（24）

专题参考　专题参考（1）

第一千二百二十七条

医疗机构及其医务人员不得违反诊疗规范实施不必要的检查。

法宝联想

司法案例　司法案例（128）

法学期刊　法学期刊（4）

第一千二百二十八条　医疗机构及其医务人员的合法权益受法律保护。

干扰医疗秩序，妨碍医务人员工作、生活，侵害医务人员合法权益的，应当依法承担法律责任。

法宝联想

法律法规　规范性文件（5）

司法案例　司法案例（38）

法学期刊　法学期刊（5）

第七章　环境污染和生态破坏责任

第一千二百二十九条

因污染环境、破坏生态造成他人损害的，侵权人应当承担侵权责任。

法宝联想

法律法规　规范性文件（9）法律动态（3）

司法案例　司法案例（2103）案例报道（10）

法学期刊　法学期刊（118）

法宝律师　律所实务（2）

专题参考　专题参考（5）

第一千二百三十条

因污染环境、破坏生态发生纠纷，行为人应当就法律规定的不承担责任或者减轻责任的情形及其行为与损害之间不存在因果关系承担举证责任。

法宝联想

法律法规　规范性文件（4）法律动态（2）

司法案例　司法案例（469）案例报道（3）

法学期刊　法学期刊（68）

法宝律师　律所实务（3）

专题参考　专题参考（3）

第一千二百三十一条

两个以上侵权人污染环境、破坏生态的，承担责任的大小，根据污染物的种类、浓度、排放量，破坏生态的方式、范围、程度，以及行为对损害后果所起的作用等因素确定。

法宝联想

法律法规　法规解读（1）

司法案例　司法案例（140）

法学期刊　法学期刊（20）

法宝律师　律所实务（1）

专题参考　专题参考（1）

第一千二百三十二条

侵权人违反法律规定故意污染环境、破坏生态造成严重后果的，被侵权人有权请求相应的惩罚性赔偿。

法宝联想

法律法规　司法解释（1）规范性文件（13）法规解读（1）法律动态（9）

司法案例　司法案例（111）案例报道（15）

法学期刊　法学期刊（163）

法宝律师　律所实务（1）

专题参考　专题参考（6）

第一千二百三十三条

因第三人的过错污染环境、破坏生态的，被侵权人可以向侵权人请求赔偿，也可以向第三人请求赔偿。侵权人赔偿后，有权向第三人追偿。

法宝联想

法律法规　司法解释（2）法律动态（1）

司法案例　司法案例（32）案例报道（1）

法学期刊　法学期刊（50）

法宝律师　律所实务（1）

专题参考　专题参考（4）

第一千二百三十四条

违反国家规定造成生态环境损害，生态环境能够修复的，国家规定的机关或者法律规定的组织有权请求侵权人在合理期限内承担修复责任。侵权人在期限内未修

复的，国家规定的机关或者法律规定的组织可以自行或者委托他人进行修复，所需费用由侵权人负担。

法宝联想

法律法规　司法解释（2）规范性文件（10）法规解读（1）法律动态（8）

司法案例　司法案例（1224）案例报道（16）

法学期刊　法学期刊（241）

法宝律师　律所实务（4）

专题参考　专题参考（15）

第一千二百三十五条

违反国家规定造成生态环境损害的，国家规定的机关或者法律规定的组织有权请求侵权人赔偿下列损失和费用：

（一）生态环境受到损害至修复完成期间服务功能丧失导致的损失；

（二）生态环境功能永久性损害造成的损失；

（三）生态环境损害调查、鉴定评估等费用；

（四）清除污染、修复生态环境费用；

（五）防止损害的发生和扩大所支出的合理费用。

法宝联想

法律法规　司法解释（4）规范性文件（23）法规解读（1）法律动态（10）

司法案例　司法案例（1733）案例报道（11）

法学期刊　法学期刊（226）

法宝律师　律所实务（6）

专题参考　专题参考（13）

第八章　高度危险责任

第一千二百三十六条

从事高度危险作业造成他人损害的，应当承担侵权责任。

法宝联想

司法案例　司法案例（259）

法学期刊　法学期刊（18）

专题参考　专题参考（1）

第一千二百三十七条

民用核设施或者运入运出核设施的核材料发生核事故造成他人损害的，民用核设施的营运单位应当承担侵权责任；但是，能够证明损害是因战争、武装冲突、暴乱等情形或者受害人故意造成的，不承担责任。

法宝联想

司法案例　司法案例（3）

法学期刊　法学期刊（10）

法宝律师　律所实务（1）

第一千二百三十八条

民用航空器造成他人损害的，民用航空器的经营者应当承担侵权责任；但是，能够证明损害是因受害人故意造成的，不承担责任。

法宝联想

司法案例　司法案例（10）

法学期刊　法学期刊（7）

第一千二百三十九条

占有或者使用易燃、易爆、剧毒、高放射性、强腐蚀性、高致病性等高度危险物造成他人损害的，占有人或者使用人应当承担侵权责任；但是，能够证明损害是

因受害人故意或者不可抗力造成的，不承担责任。被侵权人对损害的发生有重大过失的，可以减轻占有人或者使用人的责任。

法宝联想

司法案例 司法案例（168）案例报道（1）

法学期刊 法学期刊（13）

专题参考 专题参考（2）

第一千二百四十条

从事高空、高压、地下挖掘活动或者使用高速轨道运输工具造成他人损害的，经营者应当承担侵权责任；但是，能够证明损害是因受害人故意或者不可抗力造成的，不承担责任。被侵权人对损害的发生有重大过失的，可以减轻经营者的责任。

法宝联想

司法案例 司法案例（1109）案例报道（2）

法学期刊 法学期刊（14）

法宝律师 律所实务（1）

专题参考 专题参考（1）

第一千二百四十一条

遗失、抛弃高度危险物造成他人损害的，由所有人承担侵权责任。所有人将高度危险物交由他人管理的，由管理人承担侵权责任；所有人有过错的，与管理人承担连带责任。

法宝联想

司法案例 司法案例（17）

法学期刊 法学期刊（7）

专题参考 专题参考（1）

第一千二百四十二条

非法占有高度危险物造成他人损害的，由非法占有人承担侵权责任。所有人、管理人不能证明对防止非法占有尽到高度注意义务的，与非法占有人承担连带责任。

法宝联想

司法案例 司法案例（23）

法学期刊 法学期刊（7）

专题参考 专题参考（1）

第一千二百四十三条

未经许可进入高度危险活动区域或者高度危险物存放区域受到损害，管理人能够证明已经采取足够安全措施并尽到充分警示义务的，可以减轻或者不承担责任。

法宝联想

法律法规 司法解释（2）法律动态（1）

司法案例 司法案例（306）案例报道（3）

法学期刊 法学期刊（8）

法宝律师 律所实务（1）

专题参考 专题参考（2）

第一千二百四十四条

承担高度危险责任，法律规定赔偿限额的，依照其规定，但是行为人有故意或者重大过失的除外。

法宝联想

司法案例 司法案例（12）

法学期刊 法学期刊（9）

第九章 饲养动物损害责任

第一千二百四十五条

饲养的动物造成他人损害的，动物饲养人或者管理人应当承担侵权责任；但是，能够证明损害是因被侵权人故意或者重大过失造成的，可以不承担或者减轻责任。

法宝联想

法律法规　规范性文件（1）法规解读（1）法律动态（3）

司法案例　司法案例（3025）案例报道（20）

法学期刊　法学期刊（26）

法宝律师　律所实务（1）

第一千二百四十六条

违反管理规定，未对动物采取安全措施造成他人损害的，动物饲养人或者管理人应当承担侵权责任；但是，能够证明损害是因被侵权人故意造成的，可以减轻责任。

法宝联想

法律法规　法规解读（1）法律动态（1）

司法案例　司法案例（913）案例报道（8）

法学期刊　法学期刊（11）

法宝律师　律所实务（1）

第一千二百四十七条

禁止饲养的烈性犬等危险动物造成他人损害的，动物饲养人或者管理人应当承担侵权责任。

法宝联想

法律法规　规范性文件（1）法规解读（1）法律动态（2）

司法案例　司法案例（122）案例报道（3）

法学期刊　法学期刊（13）

法宝律师　律所实务（1）

专题参考　专题参考（2）

第一千二百四十八条

动物园的动物造成他人损害的，动物园应当承担侵权责任；但是，能够证明尽到管理职责的，不承担侵权责任。

法宝联想

法律法规　法律动态（1）

司法案例　司法案例（17）案例报道（1）

法学期刊　法学期刊（8）

专题参考　专题参考（1）

第一千二百四十九条

遗弃、逃逸的动物在遗弃、逃逸期间造成他人损害的，由动物原饲养人或者管理人承担侵权责任。

法宝联想

法律法规　规范性文件（2）

司法案例　司法案例（64）

法学期刊　法学期刊（2）

第一千二百五十条

因第三人的过错致使动物造成他人损害的，被侵权人可以向动物饲养人或者管理人请求赔偿，也可以向第三人请求赔偿。动物饲养人或者管理人赔偿后，有权向第三人追偿。

法宝联想

司法案例　司法案例（50）

法学期刊　法学期刊（16）

第一千二百五十一条

饲养动物应当遵守法律法规，尊重社会公德，不得妨碍他人生活。

法宝联想

司法案例　司法案例（196）案例报道（1）

法学期刊　法学期刊（6）

专题参考　专题参考（1）

第十章　建筑物和物件损害责任

第一千二百五十二条

建筑物、构筑物或者其他设施倒塌、塌陷造成他人损害的，由建设单位与施工单位承担连带责任，但是建设单位与施工单位能够证明不存在质量缺陷的除外。建设单位、施工单位赔偿后，有其他责任人的，有权向其他责任人追偿。

因所有人、管理人、使用人或者第三人的原因，建筑物、构筑物或者其他设施倒塌、塌陷造成他人损害的，由所有人、管理人、使用人或者第三人承担侵权责任。

法宝联想

司法案例　司法案例（1070）案例报道（2）

法学期刊　法学期刊（14）

法宝律师　律所实务（3）

专题参考　专题参考（4）

第一千二百五十三条

建筑物、构筑物或者其他设施及其搁置物、悬挂物发生脱落、坠落造成他人损害，所有人、管理人或者使用人不能证明自己没有过错的，应当承担侵权责任。所有人、管理人或者使用人赔偿后，有其他责任人的，有权向其他责任人追偿。

法宝联想

法律法规 规范性文件（4）法律动态（1）

司法案例 司法案例（2859）案例报道（11）

法学期刊 法学期刊（21）

法宝律师 律所实务（2）

专题参考 专题参考（4）

第一千二百五十四条

禁止从建筑物中抛掷物品。从建筑物中抛掷物品或者从建筑物上坠落的物品造成他人损害的，由侵权人依法承担侵权责任；经调查难以确定具体侵权人的，除能够证明自己不是侵权人的外，由可能加害的建筑物使用人给予补偿。可能加害的建筑物使用人补偿后，有权向侵权人追偿。

物业服务企业等建筑物管理人应当采取必要的安全保障措施防止前款规定情形的发生；未采取必要的安全保障措施的，应当依法承担未履行安全保障义务的侵权责任。

发生本条第一款规定的情形的，公安等机关应当依法及时调查，查清责任人。

法宝联想

法律法规 司法解释（2）规范性文件（19）法规解读（2）法律动态（6）

司法案例 司法案例（558）案例报道（6）

法学期刊　法学期刊（131）

法宝律师　律所实务（4）

专题参考　专题参考（14）

第一千二百五十五条

堆放物倒塌、滚落或者滑落造成他人损害，堆放人不能证明自己没有过错的，应当承担侵权责任。

法宝联想

司法案例　司法案例（186）

法学期刊　法学期刊（5）

法宝律师　律所实务（1）

第一千二百五十六条

在公共道路上堆放、倾倒、遗撒妨碍通行的物品造成他人损害的，由行为人承担侵权责任。公共道路管理人不能证明已经尽到清理、防护、警示等义务的，应当承担相应的责任。

法宝联想

法律法规　规范性文件（1）

司法案例　司法案例（1051）案例报道（2）

法学期刊　法学期刊（12）

专题参考　专题参考（2）

第一千二百五十七条

因林木折断、倾倒或者果实坠落等造成他人损害，林木的所有人或者管理人不能证明自己没有过错的，应当承担侵权责任。

法宝联想

司法案例　司法案例（583）案例报道（3）

法学期刊　法学期刊（4）

法宝律师　律所实务（2）

第一千二百五十八条

在公共场所或者道路上挖掘、修缮安装地下设施等造成他人损害，施工人不能证明已经设置明显标志和采取安全措施的，应当承担侵权责任。

窨井等地下设施造成他人损害，管理人不能证明尽到管理职责的，应当承担侵权责任。

法宝联想

司法案例　司法案例（1854）案例报道（8）

法学期刊　法学期刊（7）

附则

第一千二百五十九条

民法所称的“以上”、“以下”、“以内”、“届满”，包括本数；所称的“不满”、“超过”、“以外”，不包括本数。

法宝联想

法律法规　规范性文件（1）

司法案例　司法案例（149）

法学期刊　法学期刊（5）

法宝律师　律所实务（5）

专题参考　专题参考（3）

第一千二百六十条

本法自2021年1月1日起施行。《中华人民共和国婚姻法》、《中华人民共和国继承法》、《中华人民共和国民法通则》、《中华人民共和国收养法》、《中华人民共和国担保法》、《中华人民共和国合同法》、《中华人民共和国物权法》、《中华人民共和国侵权责任法》、《中华人民共和国民法总则》同时废止。

法宝联想
法律法规 规范性文件（1）法律动态（1）
司法案例 司法案例（4267）案例报道（1）仲裁案例（1）
法学期刊 法学期刊（25）
专题参考 专题参考（20）

背景资料

立法草案

第十三届全国人民代表大会宪法和法律委员会关于《中华人民共和国民法典(草案修改稿)》修改意见的报告
第十三届全国人民代表大会宪法和法律委员会关于《中华人民共和国民法典(草案)》审议结果的报告
关于《中华人民共和国民法典(草案)》的说明
民法典(草案)征求意见
关于《民法典各分编(草案)》修改情况和《中华人民共和国民法典(草案)》编纂情况的汇报
民法典婚姻家庭编(草案三次审议稿)征求意见
全国人民代表大会宪法和法律委员会关于《民法典婚姻家庭编(草案)》修改情况的汇报(2019年10月)
民法典侵权责任编(草案三次审议稿)征求意见
更多

法规解读

民法典理解与适用
民法典“绿色规则”的理解与适用
民法典保证期间规定理解适用中的几个问题
《民法典》中合同形式瑕疵的补正规则——第490条第2款的理解和适用
《民法典》业主自治权的理解与适用
中国民法典集体所有权的理解与适用
《民法典》人格权编理解与适用应注意的十个问题
《民法典》视野下居住权制度的理解与适用
更多

本篇引用的法规

中央法规

中华人民共和国宪法(2018修正)

中华人民共和国民法总则
中华人民共和国侵权责任法
中华人民共和国民法通则(2009修正)
中华人民共和国物权法
中华人民共和国婚姻法(2001修正)
中华人民共和国合同法
中华人民共和国收养法(1998修正)
更多

引用本篇的法规 案例 论文

法律

中华人民共和国传染病防治法(2025修订)
全国人民代表大会常务委员会法制工作委员会关于2024年备案审查工作情况的报告
中华人民共和国突发事件应对法(2024修订)
全国人大常委会2024年度监督工作计划
全国人民代表大会宪法和法律委员会关于第十四届全国人民代表大会第一次会议主席团交付审议的代表提出的议案审议结果的报告
全国人民代表大会常务委员会法制工作委员会关于2023年备案审查工作情况的报告
全国人民代表大会社会建设委员会关于第十四届全国人民代表大会第一次会议主席团交付审议的代表提出的议案审议结果的报告
中华人民共和国海洋环境保护法(2023修订)
更多

行政法规

国务院办公厅关于加强重度残疾人托养照护服务的意见
婚姻登记条例(2025修订)
婚姻登记条例(2024修订)
国务院关于修改和废止部分行政法规的决定(中华人民共和国国务院令第797号)
外国人在中华人民共和国收养子女登记办法(2024修订)
国务院关于推进养老服务体系建设、加强和改进失能老年人照护工作情况的报告
中共中央、国务院关于加快经济社会发展全面绿色转型的意见
国务院关于促进民营经济发展情况的报告
更多

司法解释

最高人民法院关于审理劳动争议案件适用法律问题的解释(二)
最高人民法院发布人民法院依法平等保护民营企业和企业家合法权益典型案例
最高人民法院印发《关于贯彻落实＜中华人民共和国民营经济促进法＞的指导意见》的通知
最高人民法院发布6件涉财产权保护执行异议之诉典型案例
最高人民法院关于审理执行异议之诉案件适用法律问题的解释
最高人民法院发布5个网络消费民事典型案例
最高法发布6个利用网络、信息技术侵害人格权典型案例
最高检发布10件生态环境和资源保护检察公益诉讼起诉典型案例
更多

部门规章

水利部关于印发《大中型水利工程移民安置监督评估管理规定》的通知
市场监管总局办公厅关于印发《委托合同(示范文本)》的通知(2025修订)
中国证监会办公厅关于简化办理已故投资者小额遗产继承程序的通知

民政部办公厅关于开展2025年社会团体、民办非企业单位抽查审计的通知
自然资源部、市场监管总局关于发布《国有建设用地使用权出让合同(示范文本)》的通知
划拨用地目录(2025)
个体工商户登记管理规定
人力资源社会保障部等5部门关于开展“法治护航·保障民生”2025年人力资源和社会保障法治知识竞赛活动的通知
更多

其他规范性文件

网络继续教育平台课程上新
中央宣传部、司法部、全国普法办关于印发《2025年“民法典宣传月”工作方案》的通知
中国法学会、司法部、共青团中央关于开展“2025年青年普法志愿者法治文化基层行”活动的通知
中国结算关于修改部分业务规则的通知
中国土地估价师与土地登记代理人协会关于网络继续教育平台课程上新的公告
中国融资担保业协会关于举办财务管理与资本运作培训班的通知
中共交通运输部党组、交通运输部关于2024年度法治政府部门建设工作情况的报告
中国注册税务师协会秘书处关于举办企业所得税汇算清缴业务培训班的通知(2024)
更多

地方法规规章

衢州市人民政府办公室关于进一步加强严重精神障碍患者肇事肇祸行为预防处置工作的意见(2025)
福州市住宅专项维修资金管理办法
衡水市物业管理条例(2025修正)
遂宁市物业管理条例
佛山市住房和城乡建设局关于印发《佛山市住建领域依法分类处理信访诉求清单》的通知
九江市生态环境损害赔偿工作规定
黑龙江省人民政府办公厅关于印发《黑龙江省经营主体住所(经营场所)登记管理办法》的通知
郑州市住房保障和房地产管理局、郑州市市场监督管理局关于印发《郑州市闲置和低效利用的非住宅项目改建为租赁住房实施细则》的通知
更多

案例与裁判文书

徐某;朱某修理合同纠纷一审民事判决书
兴业某股份公司;郑某一审民事判决书
兴业某股份公司;王某一审民事判决书
李某;郑州市二七区某买卖合同纠纷一审民事判决书
李添;新乡市亿源物业服务有限公司车库纠纷一审民事其他
胡某;郑某甲;中国某有限公司金华市分公司一审民事判决书
某建设公司、某市政公司等买卖合同纠纷民事二审民事判决书
马某甲、鲁某案外人执行异议之诉民事二审民事判决书
更多

法学期刊

夫妻共同财产赠与纠纷中行为无效与全额返还的司法评价
商品房预售合同“调减违约金”纠纷监督办案审视
公告送达缺席判决合同解除纠纷案件的检察监督
房屋租赁合同关系中消防安全责任的界定标准
民事生效裁判监督中香港个人破产案件判决的适用逻辑
《刑事诉讼法》再修改之刑事和解制度的完善
不可移动文物完全灭失检察公益诉讼的办案思考

北京某至物业管理有限责任公司诉北京某锦宾馆房屋租赁合同纠纷案

更多

律所实务

未经授权发布直播录屏，侵权吗?

中美贸易战对国际商务合同履行的影响和应对策略

“如有变更，须有书面协议”暗含何种“杀机”？

我国企业境外投资中东欧国家

融资租赁法律咨询（二十二）

开源软件合规：法律风险、IPO监管与具体防控措施

房地产疑难争议——合作开发中一方以“确保拿地”作为合作条件的效力及可执行性

规范租赁市场，保障租住权益 —— 国务院《住房租赁条例》要点解析

更多

*注：本文格式遵循《全国人大法规备案审查信息平台电子文件格式规范（试行）》标准。

©北大法宝：（www.pkulaw.com）专业提供法律信息、法学知识和法律软件领域各类解决方案。北大法宝为您提供丰富的参考资料，正式引用法规条文时请与标准文本核对。

欢迎查看所有产品和服务。

法宝快讯：如何快速找到您需要的检索结果？法宝V6有何新特色？

扫描二维码阅读原文

原文链接：https://www.pkulaw.com/chl/aa00daaeb5a4fe4ebdfb.html
