# Supplementary material for: China’s Legal Protection System for Pangolins: Past, Present, and Future
Source: Animals (Basel). 2025 Aug 18;15(16):2422. doi: 10.3390/ani15162422 (PMC12383201; doi:10.3390/ani15162422)
Supplement: Supplementary file 1 [file animals-15-02422-s001.zip › Supplementary Material S2 -Full Texts of Laws and Regulations Related to Pangolins in China/【23】国家林业和草原局公告2020年第12号――关于穿山甲调整保护级别的公告(FBM-CLI.4.pdf]

国家林业和草原局公告2020年第12号—关于穿山甲调整保护级别的公告

制定机关： 国家林业和草原局 机构沿革

发文字号：国家林业和草原局公告2020年第12号

公布日期：2020.06.03

施行日期：2020.06.03

时效性： 现行有效

效力位阶： 部门工作文件

法规类别： 财政综合规定

国家林业和草原局公告

（2020年第12号）

为加强穿山甲保护，经国务院批准，现将穿山甲属所有种由国家二级保护野生动物调整为国家一级保护野生动物（详见附件），自公布之日起施行。

附件：国家重点保护野生动物名录

国家林业和草原局

2020年6月3日

引用本篇的法规 案例 论文

案例与裁判文书

某市某某中药饮片有限公司、某市某局行政一审行政判决书

*注：本文格式遵循《全国人大法规备案审查信息平台电子文件格式规范（试行）》标准。

©北大法宝：（www.pkulaw.com）专业提供法律信息、法学知识和法律软件领域各类解决方案。北大法宝为您提供丰富的参考资料，正式引用法规条文时请与标准文本核对。

欢迎查看所有产品和服务。

法宝快讯：如何快速找到您需要的检索结果？法宝V6有何新特色？

扫描二维码阅读原文

原文链接：https://www.pkulaw.com/chl/96b37ba9db189a02bdfb.html
