# Supplementary material for: China’s Legal Protection System for Pangolins: Past, Present, and Future
Source: Animals (Basel). 2025 Aug 18;15(16):2422. doi: 10.3390/ani15162422 (PMC12383201; doi:10.3390/ani15162422)
Supplement: Supplementary file 1 [file animals-15-02422-s001.zip › Supplementary Material S2 -Full Texts of Laws and Regulations Related to Pangolins in China/【24】国家林业和草原局、国家中医药管理局、国家药品监督管理局关于切实加强穿山甲保护管理的通知(FBM-CLI.4.pdf]

国家林业和草原局、国家中医药管理局、国家药品监督管理局关于切实加强穿山甲保护管理的通知

制定机关： 国家林业和草原局 国家中医药管理局 国家药品监督管理局(已变更) 机构沿革

发文字号：林护发〔2024〕67号

公布日期：2024.11.13

施行日期：2024.11.13

时效性： 现行有效

效力位阶： 部门工作文件

法规类别： 财政综合规定

国家林业和草原局、国家中医药管理局、国家药品监督管理局关于切实加强穿山甲保护管理的通知

（林护发〔2024〕67号）

各省、自治区、直辖市林业和草原、中医药、药品监督主管部门，新疆生产建设兵团卫生健康委、林业和草原主管部门，内蒙古森工集团：

为切实加强穿山甲保护管理，履行《濒危野生动植物种国际贸易公约》，根据《野生动物保护法》等规定，现将有关要求通知如下：

一、提高政治站位，压实主体责任。各级林草、中医药、药品监管主管部门要进一步提高政治站位，深刻认识加强穿山甲保护管理的重大意义，分工协作、密切配合。按照职责分工，林草部门负责穿山甲资源保护管理，中医药主管部门负责

中医医院临床使用穿山甲甲片管理，药品监管部门负责使用穿山甲甲片的药品生产销售监管。

二、强化保护研究，鼓励技术攻关。进一步加大穿山甲及其栖息地保护管护力度，强化穿山甲栖息地保护修复，科学开展穿山甲救护，及时放归野外救护穿山甲个体，全力保持穿山甲野外种群稳中有升良好势头。深入掌握本地区穿山甲野外种群分布范围、数量结构、栖息地面积质量、主要威胁因素、人工繁育等情况，由国家林业和草原局穿山甲保护研究中心（以下简称“穿山甲中心”）适时公布穿山甲保护成效。加大穿山甲中心建设力度，建立穿山甲人工繁育基地和种质资源库，联合开展穿山甲人工繁育关键技术科研攻关，着力推动穿山甲人工繁育种群取得实质性进展。鼓励支持科研院所、医院、制药企业联合开展穿山甲甲片替代品研究攻关。

三、加大监管力度，严格穿山甲甲片入药。本着“节约资源，从严从紧”原则，严格穿山甲甲片入药管理，合理压缩消耗用量。一是省级林草、中医药、药品监管主管部门联合开展穿山甲利用状况摸底调查，掌握本省区穿山甲人工繁育机构、穿山甲甲片库存以及近3年中医医院和药品生产企业使用穿山甲甲片及购销情况，并将核实信息登记造册，落实持有主体管理责任。二是严格控制穿山甲甲片年度消耗量，原则控制在1吨左右。国家林草局会同中医药局、药监局研究分配措施。省级林草部门根据分配措施履行行政许可职责。三是省级林草部门要强化对本省穿山甲甲片合法库存持有者的监督核查，及时根据行政许可情况更新穿山甲甲片库存数量；省级中医药主管和药品监管部门要严格监管辖区内利用穿山甲甲片的医院和制药企业；各单位依法严肃处理违法违规行为，坚决杜绝穿山甲甲片滥用。四是执法查没的穿山甲甲片及其制品，仅限于科学研究、司法执法、公益宣

传等非商业性活动。严格禁止利用其他非法或不明来源的穿山甲甲片及其制品。五是继续停止商业性进出口穿山甲及其制品，继续停止穿山甲饮片纳入医保范畴，不得将含穿山甲成份的药物新增纳入医保范畴。

四、强化分工协作，严厉打击犯罪。各级林草部门要充分发挥野生动物联合执法工作协调机制作用，会同网信、公安、交通运输、海关、市场监管、铁路、民航、邮政等部门，组织开展执法监管联合行动，按职责分工加强监督检查，坚决打击和严厉惩处非法猎捕穿山甲、破坏穿山甲栖息地、干扰穿山甲生息繁衍以及非法出售、购买、利用、运输、携带、寄递、进出口穿山甲及其制品等违法行为，加强各环节全链条监管，特别是加大网络平台、商品交易市场、餐饮场所、口岸等执法查验力度。严格核查穿山甲甲片来源，坚决摧毁非法交易渠道，捣毁犯罪窝点和团伙，依法严惩犯罪分子，持续保持高压态势。

五、广泛开展宣传，营造良好氛围。各级林草部门要积极协调宣传、网信部门统筹各类宣传渠道，通过专家解读、主题活动、公益广告、曝光反面典型等方式，发挥社会团体、公益组织和广大志愿者积极作用，普及穿山甲保护法律知识；建立健全有奖举报制度，鼓励公众提供各类破坏穿山甲等野生动物资源问题线索，自觉抵制违法行为，开创全社会共同保护穿山甲的新局面。

特此通知。

国家林业和草原局　国家中医药管理局　国家药品监督管理局

2024年11月13日

*注：本文格式遵循《全国人大法规备案审查信息平台电子文件格式规范（试行）》标准。

©北大法宝：（www.pkulaw.com）专业提供法律信息、法学知识和法律软件领域各类解决方案。北大法宝为您提供丰富的参考资料，正式引用法规条文时请与标准文本核对。

欢迎查看所有产品和服务。

法宝快讯： 如何快速找到您需要的检索结果？ 法宝 V6 有何新特色？

扫描二维码阅读原文

原文链接：https://www.pkulaw.com/chl/37e628d0fc403bafbdfb.html
