# Supplementary material for: China’s Legal Protection System for Pangolins: Past, Present, and Future
Source: Animals (Basel). 2025 Aug 18;15(16):2422. doi: 10.3390/ani15162422 (PMC12383201; doi:10.3390/ani15162422)
Supplement: Supplementary file 1 [file animals-15-02422-s001.zip › Supplementary Material S2 -Full Texts of Laws and Regulations Related to Pangolins in China/【2】国务院批转国家医药管理局关于中药工作问题的报告的通知(FBM-CLI.2.7254).pdf]

国务院批转国家医药管理局关于中药工作问题的报告的通知

制定机关： 国务院

发文字号：国发〔1983〕160号

公布日期：1983.10.13

施行日期：1983.10.13

时效性： 现行有效

效力位阶： 国务院规范性文件

法规类别： 中药管理

国务院批转国家医药管理局关于中药工作问题的报告的通知

（一九八三年十月十三日 国发[1983]160号）

国务院同意国家医药管理局《关于中药工作问题的报告》，现转发给你们，望研究执行。

中药工作是关系我国十亿人民保健医疗事业的大事。当前部分中药供应紧缺，影响防病治病的状况，应当引起各级人民政府的注意。要加强对中药工作的领导，恢复和健全药材专业公司的体制。对于供应紧缺的品种，除了属于国家禁猎和重点保护的动物外，其余品种必须采取有效措施，积极发展生产，尽快解决。对中药市场要加强管理，严禁哄抬药价和破坏中药资源。

中药材是商品，生产、流通、消费的一切政策措施必须符合经济规律；但中药材

又不同于一般农副产品，是防病治病的特殊商品，必须由药材公司统一计划、统一管理、统一经营。二类药材应由药材公司严格按照计划和牌价统一收购、加工、调拨和供应出口，除药材公司委托供销社代购外，其它部门和个人均不得插手经营。销区的药材经营单位不准高价抢购或到产地抬价抢购紧缺药材。完成派购任务后的二类药材，可以实行浮动价格，按物价管理权限，经过批准执行。

国家医药管理局关于中药工作问题的报告

（一九八三年九月三日）

根据中央领导同志的多次批示，我们对中药工作进行了调查研究，并邀请农工民主党、民主建国会、全国工商联的专家进行了座谈。今年五月在北京召开了全国中药工作会议，着重讨论了如何解决中药供应紧缺、饮片质量下降等问题。现将中药工作的主要情况、问题和意见报告如下：

大家认为，中医中药是我国的国宝，对中华民族的生存繁衍作出了贡献，至今仍然对保障人民身体健康起着重要作用，在国际医药科学领域中享有很高的声誉。党中央、国务院历来重视中医中药。建国三十多年来，特别是党的十一届三中全会以来，中药材生产有了很大发展。一九八二年家种药材种植面积达三百七十六万亩，收购家种和野生药材近十亿斤。中成药研究成功并投入生产的新剂型有三十多种。中药（包括中药材、中成药）购销大幅度增加。一九八二年购进二十四亿元，销售三十一亿元，分别比一九五七年增加六倍。中药出口换汇二亿五千万美元。

但是，近两年来，中药材紧缺品种数目不断增加，已达到一百四十种左右，饮片质量下降，各方面反映十分强烈，一些著名老中医发出“中药危机”和“抢救中

药”的呼吁。造成中药材紧缺品种增加的主要原因是：

（一）生产赶不上需要的增长，有些药材的生产计划不落实。有些经营部门存在重采购轻生产，怕积压不怕脱销的思想，放松了对生产的指导。有些供应紧缺的中药材，如麝香、虎骨、熊胆、豹骨、穿山甲片等十四种，生产这些药材的稀有动物繁殖困难，国家已列为禁猎动物。有些野生药材，如黄芩、知母、川贝、猪苓等二十多种，需要量增大，采挖过度。有些家种的芋肉、银花、黄连、人参、砂仁等，产量虽然增长一至六倍，也不能满足需要。多年生的木本药材，如杜仲、厚朴、黄柏等，发展生产需要有一个过程。进口的犀角、羚羊角、西红花等二十多种药材，国外资源少，进口数量有限。

（二）药材进销差率过小，价格管理过于集中，束缚了生产和经营的开展。一九五七年中药的进销差率为百分之五十左右，一九六〇年紧缩到百分之三十左右，一九六九年又紧缩到百分之十五到十八，同时取消了地区差价，实行中药材全省一个价，中成药按产地全国一个价。中药行业自有资金减少，贷款增多，利息负担加重。以致销售虽逐年上升，但利润却逐年下降，很多单位难以维持简单再生产。中成药厂、饮片加工厂、基层收购点和门市部十分简陋，设备陈旧，仓库严重不足，很多单位半数的药材露天存放，无力改善。

（三）多方插手，冲击国家计划。有些外贸、社队企业、贸易货栈、医疗单位，甚至医药系统内部的某些单位，对贵重紧缺的中药材，有的抬价抢购，有的地区超计划多出口，冲击内销；有的内销卖高价，不认真执行国家调拨计划；一些地区还成立了与药材公司重叠的机构，对当地生产的药材实行垄断，不让药材公司收购，不仅冲击国家收购计划，也破坏了药材资源。

（四）对中药的特点认识不足，经营管理体制混乱。目前全国县级药材公司，大致分属七个部门领导，有的处于无人管理的状态。有的省、自治区撤销了省区药材公司，有的省医药局把公司财权收归省局，药材公司缺乏经营积极性。全国的中成药厂分属十六个部门领导。因此削弱了药材公司产购销结合的专业体制，很难发挥专业公司的职能作用，以至计划完成很差，商品调拨不灵。二类药材的调拨计划近几年只完成百分之三十左右。

（五）科研和教育落后，技术力量薄弱，中药队伍素质下降。全国没有一个中药研究中心，现有几个中药研究所，设备十分落后，而且技术人员少，中药技术人员仅占职工总数的千分之六。在教育方面，全国只有四所中药中等学校，校舍、设备、师资都很缺乏，没有一所中药大学。现在全国中药职工近三十万人，老药工不到百分之十，没有技术职称，青年职工多数未经业务技术培训，收错药、卖错药的事故屡有发生。

中药材是治病救人的特需商品，是特种经济作物，经营中药材带有福利性质，为医疗保健服务，不能按一般农副产品对待，需要有特殊政策和措施。现对解决中药材供应紧缺问题提出以下意见：

（一）适应农业发展的新形势，积极发展药材生产。

根据一九八三年中央一号文件的精神，家种、家养的药材，要根据农业区域规划，与农林部门共同研究下达生产计划，采用产销结合，订立派购、收购合同，合资经营，联合加工等形式，办好生产基地。积极促进国营和集体药材种植、饲养场发展生产。同时，扶持种药专业户、重点户，通过产销合同，加强生产和技术指导。加强市场要求预测工作，及时发布生产信息。野生动、植物药材要注意保护资源，认真贯彻“护、养、猎”和“采、护、育”的方针，有计划地猎取和采

收；有些药用量大资源少的，要积极变家种家养。对当前紧缺的药材，按照生产难易，分类指导。对资源稀少的动、植物药材，如犀角、羚羊角、麝香、虎骨、熊胆、猪苓、川贝等一时难以满足供应的品种，建立保护区，加强科学研究，搞人工合成，或寻找同疗效品种。木本药材林业部门应纳入造林计划，明确谁种归谁，长期不变的政策。同时，要认真搞好中药资源普查，发掘新品种，增加新资源。为开发、扶持中药材生产，经与财政部研究，按中药材收购金额提取百分之一的中药材开发基金，计入成本，由生产和经营部门协商，用于基地生产、资源保护、野生药材变家种家养和木本药材的培育费。进口药材要与外贸部门结合，积极争取多进口一些。交叉经营的品种，如山楂、乌梅、杏仁、鱼膘等，要继续贯彻《国务院关于加强柑桔皮收购工作的通知》（国发〔１９６８〕２５号文）精神，“优先保证药用”。其它紧缺药材，按照商品分级管理原则，明确责任，自下而上地制定规划，搞好综合平衡，把生产搞上去。今后要努力做到，除稀有动、植物药材和多年生品种外，其它品种都应逐步满足医疗需要。

加强医药结合，要会同卫生部门根据医疗需要制订中药材的生产、收购计划。

（二）调整购销政策，加强市场物价管理。

坚持“计划经济为主，市场调节为辅”的原则，中药材由药材公司统一经营、统一计划、统一管理。将二类品种调整为三十种（见附件），这些产品不许上市，不许议价。其中麝香、甘草、杜仲、厚朴等实行统一牌价，全额收购；其余二十六种实行计划收购，计划内执行国家牌价，计划外根据产销情况变化，按照物价管理权限经过批准，可以实行浮动价格。除委托供销社代购外，其他一切部门（包括外贸、医疗单位、社队企业、农林牧场、药厂等）和个人均不得插手收购，外地药材经营单位不准高价诱购或到产地抬价抢购属于二类品种的药材。

三类药材中，属于必须保护资源的和一地、少地生产供应全国的品种，由各省、市、自治区参照二类品种的管理办法加强管理，具体品种由各省、市、自治区自定。其余三类品种由县药材公司管理，是否实行议购议销，由省、市、自治区药材、物价部门自行确定。

坚持产销双方签订合同，合同一经签订，就要严格执行，保证国家收购计划的完成和生产者的利益。

整顿医药部门的贸易货栈，不经省、市、自治区医药管理部门同意，不经工商行政管理部门核准登记，不准营业，不准经营二类药材。其它贸易货栈，不准跨行业经营中药材。

各地区医药管理部门要与工商行政管理、物价、卫生、公安、交通、邮电、银行等部门密切配合，加强中药的市场管理和物价管理。对游医药贩和制售假药、危害人民生命健康者，要坚决制止和取缔，情节严重的，要依法处理。

（三）改善中药的经营管理。

（1）改革中药作价办法。取消中药材全省一个价和中成药按产地全国一个价，恢复地区差价。适当扩大进销差率，中药材的进销差率和地区差率总幅度拟在百分之三十五左右进行分配。中成药的工业利润率可掌握在百分之十左右。具体方案，与国家物价局商定后下达执行。

（2）中药材调拨实行调拨包干办法。根据品种不同情况，有的一年一定，有的一定几年不变，保证国家拿到主产区产量百分之七十以上的商品。要建立调拨奖罚制度，不严肃执行调拨计划的，要加强教育，进行通报批评或处罚。具体办法

另行制定。

（３）为了保证配方需要，必须继续贯彻“先治疗、后滋补”和“先饮片、后成药”的原则，对中成药和饮片都需要的紧缺药材，首先保证饮片配方。对饮片加工的技术改造，要与中成药厂统筹安排，在安排技术措施费时，给以专项照顾。要保证中药质量，饮片加工要严格执行《饮片炮制规范》，不合格的不准出厂，不准出售。在传统炮制的基础上，用现代科学技术把炮制工艺再提高一步。

中药饮片加工，可适当增加加工费，以适应以后逐步过渡、实行单独核算的需要，饮片加工作为一个生产环节，要有一定利润，以利于提高和改善饮片加工的生产技术水平。

（４）为了扶持中药事业的发展，在利改税核定中药的留利比例时，可在一九八二年合理留利基础上，适当给予照顾。具体由省、市、自治区确定。少数民族地区的药材公司，应按民贸公司三项照顾的政策落实。

（５）为了保证疫情、灾情、军需、急救用药和以丰补歉、调节余缺，中国药材公司和各省、市、自治区药材公司必须有一定储备。储备贷款请银行按实际需要供应。对于储备时间长，只要不是保管和管理不善，经过核定，失去疗效，应允许作正常报损。

（四）中药出口要贯彻“先国内、后国外，出口服从内销”和一九五五年《国务院批转商业部关于中药材会议情况的报告》中指出的“国内供应严重不足或已危害生产的，应停止或减少出口；国内供应有余的，应争取多出口”的原则，实行出口许可证办法。二类品种出口，由国家医药管理局和对外经济贸易部共同衔接

编制计划，经批准后，由对外经济贸易部签发出口许可证。三类品种由省、市、自治区外贸部门与西药部门进行衔接。

（五）恢复和健全药材专业公司体制。药材公司实行中央、省、县三级管理，撤销了省、县公司的，要尽快恢复，收走公司财权的应交回。中成药由药材公司统一管理。中药是多味配方，不要为了利润把某些紧缺品种从药材公司划出，另组专业企业。已经划出的，应尽快与药材公司合并。各省、市、自治区药材公司要加强系统财务管理，使药材专业公司成为经济实体。

县药材公司是组织药材生产、收购、供应的第一线，要切实加强领导。对基层供销社代购药材，要加强业务技术指导，签订代购合同，按计划合同组织收购，付给合理的手续费。同时可以在重点产区下伸部分网点，收售结合，方便群众。对一些重点产品，可根据情况，与供销社、社队企业搞一些联合生产、联合加工。

（六）加强科研和教育工作，加速培养中药人才。中药科研要以中医药理论为基础。用现代科学技术进行应用和理论研究。医药部门要主动与卫生、科研、农林高等院校密切配合，组织中药科研的协作。把现有的中药研究所充实健全起来，有条件的省、市、自治区可根据需要建立中药研究机构。积极筹建全国中药研究、开发机构。

首先要抓好中药的中等专业教育，充实师资、改善校舍、设备，提高教学质量。有条件的省、市、自治区，根据情况可以自己建立或与外省联合建立中药中等学校。当前要着重抓好职工教育，对新参加工作的青年职工，根据缺什么补什么的原则，分期分批采取各种形式进行培训，使之达到应知、应会、应用的要求，独立工作。有重点地发展高等中药院校，培养高等技术人才。对中药老药工的技术

职称和职工工资待遇问题，拟同劳动人事部另行研究解决办法，予以合理解决。

中药工作涉及面广，政策性强，比较复杂，当前解决中药供应紧缺问题十分迫切。请各地区、各部门加强对中药工作的领导，切实帮助解决中药生产、收购、加工、调拨、供应中的实际问题，并请有关部门给予支持，共同为扶持和振兴中药事业作出新的贡献。

以上报告，如无不妥，请转报国务院批转各地执行。

附：二类计划管理品种目录

二类计划管理品种目录

黄连、甘草、党参、当归、川芎、生地、白术、白芍、茯苓、麦冬、黄芪、贝母、银花、山药、菊花、牛膝、元胡、丹皮、桔梗、厚朴、连翘、杜仲、芋肉、枸杞、天麻、三七、人参（包括野山参）、鹿耳、麝香、牛黄。

*注：本文格式遵循《全国人大法规备案审查信息平台电子文件格式规范（试行）》标准。

©北大法宝：（www.pkulaw.com）专业提供法律信息、法学知识和法律软件领域各类解决方案。北大法宝为您提供丰富的参考资料，正式引用法规条文时请与标准文本核对。

欢迎查看所有产品和服务。

法宝快讯：如何快速找到您需要的检索结果？法宝 V6 有何新特色？

扫描二维码阅读原文

原文链接：https://www.pkulaw.com/chl/b4135a8e00b7b656bdfb.html
