# Supplementary material for: China’s Legal Protection System for Pangolins: Past, Present, and Future
Source: Animals (Basel). 2025 Aug 18;15(16):2422. doi: 10.3390/ani15162422 (PMC12383201; doi:10.3390/ani15162422)
Supplement: Supplementary file 1 [file animals-15-02422-s001.zip › Supplementary Material S2 -Full Texts of Laws and Regulations Related to Pangolins in China/【3】森林和野生动物类型自然保护区管理办法(FBM-CLI.2.pdf]

森林和野生动物类型自然保护区管理办法

制定机关： 林业部(已变更) 机构沿革

批准机关： 国务院

公布日期：1985.07.06

批准日期：1985.06.21

施行日期：1985.07.06

时效性： 现行有效

效力位阶： 行政法规

法规类别： 森林资源 野生动植物资源 自然保护

法宝提示：本篇法规的变更情况请参考：国务院关于取消和调整一批行政审批项目等事项的决定(2015)、国务院关于第二批清理规范192项国务院部门行政审批中介服务事项的决定、国务院关于取消和下放一批行政许可事项的决定(2020)

森林和野生动物类型自然保护区管理办法

（一九八五年六月二十一日国务院批准）

（一九八五年七月六日林业部公布）

第一条

自然保护区是保护自然环境和自然资源、拯救濒于灭绝的生物物种、进行科学研究的重要基地 ；

对促进科学技术、生产建设、文化教育、卫生保健等事业的发展，具有重要意义。根据《中华人民共和国森林法》和有关规定，制定本办法。

第二条 森林和野生动物类型自然保护区（以下简称自然保护区），按照本办法进行管理。

第三条

自然保护区管理机构的主要任务：贯彻执行国家有关自然保护区的方针

、政策和规定，加强管理，开展宣传教育，保护和发展珍贵稀有野生动植物资源

，

进行科学研究，探索自然演变规律和合理利用森林和动植物资源的途径，为社会主义建设服务。

第四条

自然保护区分为国家自然保护区和地方自然保护区。国家自然保护区，由林业部或所在省、自治区、直辖市林业主管部门管理；地方自然保护区，由县级以上林业主管部门管理。

第五条 具有下列条件之一者，可以建立自然保护区：

（一） 不同自然地带的典型森林生态系统的地区。

（二）

珍贵稀有或者有特殊保护价值的动植物种的主要生存繁殖地区，包括：

国家重点保护动物的主要栖息、繁殖地区；

侯鸟的主要繁殖地、越冬地和停歇地；

珍贵树种和有特殊价值的植物原生地；

野生生物模式标本的集中产地。

（三） 其他有特殊保护价值的林区。

第六条 根据本办法第五条

规定建立自然保护区，在科研上有重要价值，或者在国际上有一定影响的，报国务院批准，列为国家自然保护区；其他自然保护区、报省、自治区、直辖市人民政府批准，列为地方自然保护区。

第七条

建立自然保护区要注意保护对象的完整性和最适宜的范围，考虑当地经济建设和群众生产生活的需要，尽可能避开群众的土地、山林；确实不能避开的，应当严格控制范围，并根据国家有关规定，合理解决群众的生产生活问题。

第八条

自然保护区的解除和范围的调整，必须经原审批机关批准；未经批准不得改变自然保护区的性质和范围。

第九条 自然保护区的管理机构属于事业单位。机构的设置和人员的配备，要注意精干。国家或地方自然保护区管理机构的人员编制、 基建投资、事业经费等，经主管部门批准后，分别纳入国家和省、自治区、直辖市的计划，由林业部门统一安排。

第十条

自然保护区管理机构，可以根据自然资源情况，将自然保护区分为核心区、实验区。核心区只供进行观测研究。实验区可以进行科学实验、教学实习、参观考察和驯化培育珍稀动植物等活动。

第十一条

自然保护区的自然环境和自然资源，由自然保护区管理机构统一管理。未经林业部或省、自治区、直辖市林业主管部门批准，任何单位和个人不得进入自然保护区建立机构和修筑设施。

第十二条

有条件的自然保护区，经林业部或省、自治区、直辖市林业主管部门批准，可以在指定的范围内开展旅游活动。

在自然保护区开展旅游必须遵守以下规定：

（一）

旅游业务由自然保护区管理机构统一管理，所得收入用于自然保护区的建设和保护事业；

（二）

有关部门投资或与自然保护区联合兴办的旅游建筑和设施，产权归自然保护区，所得收益在一定时期内按比例分成，但不得改变自然保护区隶属关系；

（三） 对旅游区必须进行规划设计，确定合适的旅游点和旅游路线；

（四） 旅游点的建筑和设施要体现民族风格，同自然景观和谐一致；

（五） 根据旅游需要和接待条 件制订年度接待计划，

按隶属关系报林业主管部门批准，有组织地开展旅游：

（六）

设置防火、卫生等设施，实行严格的巡护检查，防止造成环境污染和自然资源的破坏。

第十三条

进入自然保护区从事科学研究、教育实习、参观考察、拍摄影片、登山等活动的单位和个人，必须经省、自治区、直辖市以上林业主管部门的同意。

任何部门、团体、单位与国外签署涉及国家自然保护区的协议，接待外国人到国家自然保护区从事有关活动，必须征得林业部的同意；涉及地方自然保护区的，必须征得省、自治区、直辖市林业主管部门的同意。

经批准进入自然保护区从事上述活动的，必须遵守本办法和有关规定，并交纳保护管理费。

第十四条

自然保护区内的居民，应当遵守自然保护区的有关规定，固定生产生活活动范围，在不破坏自然资源的前提下，从事种植、养殖业，也可以承包自然保护区组织的劳务或保护管理任务，以增加经济收入。

第十五条

自然保护区管理机构会同所在和毗邻的县、乡人民政府及有关单位，组成自然保护区联合保护委员会，制定保护公约，共同做好保护管理工作。

第十六条

根据国家有关规定和需要，可以在自然保护区设立公安机构或者配备公安特派员，行政上受自然保护区管理机构领导，业务上受上级公安机关领导。

自然保护区公安机构的主要任务：保护自然保护区的自然资源和国家财产，维护当地社会治安，依法查处破坏自然保护区的案件。

第十七条　本办法自公布之日起施行。

*注：本文格式遵循《全国人大法规备案审查信息平台电子文件格式规范（试行）》标准。

©北大法宝：（www.pkulaw.com）专业提供法律信息、法学知识和法律软件领域各类解决方案。北大法宝为您提供丰富的参考资料，正式引用法规条文时请与标准文本核对。

欢迎查看所有产品和服务。

法宝快讯：如何快速找到您需要的检索结果？法宝 V6 有何新特色？

扫描二维码阅读原文

原文链接：https://www.pkulaw.com/chl/5aff92d598fafb1abdfb.html
