# Supplementary material for: China’s Legal Protection System for Pangolins: Past, Present, and Future
Source: Animals (Basel). 2025 Aug 18;15(16):2422. doi: 10.3390/ani15162422 (PMC12383201; doi:10.3390/ani15162422)
Supplement: Supplementary file 1 [file animals-15-02422-s001.zip › Supplementary Material S2 -Full Texts of Laws and Regulations Related to Pangolins in China/【4】国家重点保护野生药材物种名录(FBM-CLI.4.pdf]

北大法宝 让法律更智能 PKULAW.COM

国家重点保护野生药材物种名录

制定机关：国家药品监督管理局(已变更)

公布日期：1987.10.30

施行日期：1987.10.30

时效性：现行有效

效力位阶：部门规范性文件

法规类别：野生药材资源

国家重点保护野生药材物种名录

（１９８７年１０月３０日　国家医药管理局）

中　名	学　　名	保护级别 Ⅰ级	保护级别 Ⅱ级	保护级别 Ⅲ级	药材名称
猫科动物虎	Panthera tigris Linnaeus (含国内所有亚种)	Ⅰ			虎　骨
猫科动物豹	Panthera pardus Linnaeus (含云豹·雪豹)	Ⅰ			豹　骨
牛科动物赛加羚羊	Saiga tatarica Linnaeus	Ⅰ			羚羊角
鹿科动物梅花鹿	Cervus nippon Temminck	Ⅰ			鹿　茸
鹿科动物马鹿	Cervus elaphus Linnaeus		Ⅱ		鹿　茸
鹿科动物林麝	Moschus berezovskii Flerov		Ⅱ		麝　香
鹿科动物马麝	Moschus sifanicus Przewalski		Ⅱ		麝　香
鹿科动物原麝	Moschus moschiferus Linnaeus		Ⅱ		麝　香
熊科动物黑熊	Selenarctos thibetanus Cuvier		Ⅱ		熊　胆
熊科动物棕熊	Ursus arctos Linnaeus		Ⅱ		熊　胆
鲮鲤科动物穿山甲	Manis pentadactyla Linnaeus		Ⅱ		穿山甲
蟾蜍科动物中华大蟾蜍	Bufo bufo gargarizans Cantor		Ⅱ		蟾　酥
蟾蜍科动物黑眶蟾蜍	Bufo melanostictus Schneider		Ⅱ		蟾　酥
蛙科动物中国林蛙	Rana temporaria chensinensis David		Ⅱ		哈蟆油
眼镜蛇科动物银环蛇	Bungarus multicinctus multicinctus Blyth		Ⅱ		金钱白花蛇
游蛇科动物乌梢蛇	Zaocys dhumnades (Cantor)		Ⅱ		乌梢蛇
蝰科动物五步蛇	Agkistrodon acutus (Guenther)		Ⅱ		蕲　蛇

壁虎科动物蛤蚧	Gekko gecko Linnaeus		Ⅱ		蛤　蚧
豆科植物甘草	Glycyrrhiza uralensis Fisch.		Ⅱ		甘　草
豆科植物胀果甘草	Glycyrrhiza inflata Bat.		Ⅱ		甘　草
豆科植物光果甘草	Glycyrrhiza glabra L.		Ⅱ		甘　草
毛茛科植物黄连	Coptis chinensis Franch.		Ⅱ		黄　连
毛茛科植物三角叶黄连	Coptis deltoidea C. Y. Cheng et Hsiao		Ⅱ		黄　连
毛茛科植物云连	Coptis teetoides C. Y. Cheng		Ⅱ		黄　连
五加科植物人参	Panax ginseng C. A. Mey.		Ⅱ		人　参
杜仲科植物杜仲	Eucommia ulmoides Oliv.		Ⅱ		杜　仲
木兰科植物厚朴	Magnolia officinalis Rehd. et Wils.		Ⅱ		厚　朴
木兰科植物凹叶厚朴	Magnolia officinalis Rehd. et Wils. var. biloba Rehd. etWils.		Ⅱ		厚　朴
芸香科植物黄皮树	Phellodendron chinense Schneid.		Ⅱ		黄　柏
芸香科植物黄檗	Phellodendron amurense Rupr.		Ⅱ		黄　柏
百合科植物剑叶龙血树	Dracaena cochinchinensin (Lour.) S.C. Chen		Ⅱ		血　竭
百合科植物川贝母	Fritillaria cirrhosa D. Don			Ⅲ	川贝母
百合科植物暗紫贝母	Fritillaria unibracteata Hsiao et K. C. Hsia			Ⅲ	川贝母
百合科植物甘肃贝母	Fritillaria przewalskii Maxim.			Ⅲ	川贝母
百合科植物梭砂贝母	Fritillaria delavayi Franch.			Ⅲ	川贝母
百合科植物新疆贝母	Fritillaria walujewii Regel			Ⅲ	伊贝母
百合科植物伊犁贝母	Fritillaria pallidiflora Schrenk			Ⅲ	伊贝母
五加科植物刺五加	Acanthopanax senticosus (Rupr. et Maxim.) Harms			Ⅲ	刺五加
唇形科植物黄岑	Scutellaria baicalensis Georgi			Ⅲ	黄　芩
百合科植物天门冬	Asparagus cochinchinensis (Lour.) merr.			Ⅲ	天　冬
多孔菌科真菌猪苓	Polyporus umbellatus (Pers.) Fries			Ⅲ	猪　苓
龙胆科植物条叶龙胆	Gentiana manshurica Kitag.			Ⅲ	龙　胆
龙胆科植物龙胆	Gentiana scabra Bge			Ⅲ	龙　胆
龙胆科植物三花龙胆	Gentiana triflora Pall.			Ⅲ	龙　胆
龙胆科植物坚龙胆	Gentiana regescens Franch.			Ⅲ	龙　胆
伞形科植物防风	Ledebouriella divaricata (Turcz.) Hiroe			Ⅲ	防　风
远志科植物远志	Polygala tenuifolia Willd.			Ⅲ	远　志
远志科植物卵叶远志	Polygala sibirica L.			Ⅲ	远　志
玄参科植物胡黄连	Picrorhiza scrophulariiflora Pennell			Ⅲ	胡黄连
列当科植物肉苁蓉	Cistanche deserticola Y. C. Ma			Ⅲ	肉苁蓉
龙胆科植物秦艽	Gentiana macrophylla Pall.			Ⅲ	秦　艽
龙胆科植物麻花秦艽	Gentiana macrophylla Maxim.			Ⅲ	秦　艽
龙胆科植物粗茎秦艽	Gentiana crassicaulis Duthie ex Burk.			Ⅲ	秦　艽
龙胆科植物小秦艽	Gentiana dahurica Fisch.			Ⅲ	秦　艽
马兜铃科植物北细辛	Asarum heterotropoides Fr. var			Ⅲ	细　辛

	. mandshuricum (Maxim.)Kitag.				
马兜铃科植物汉城细辛	Asarum sieboldii Miq. var. seoulense Nakai			Ⅲ	细　辛
马兜铃科植物细辛	Asarum sieboldii Miq.			Ⅲ	细　辛
紫草科植物新疆紫草	Arnebia euchroma (Royle) Johnst.			Ⅲ	紫　草
紫草科植物紫草	Lithospermum erythrorhizon Sieb. et Zucc.			Ⅲ	紫　草
木兰科植物五味子	Schisandra chinensis (Turcz.) Baill.			Ⅲ	五味子
木兰科植物华中五味子	Schisandra sphenanthera Rehd. et Wils.			Ⅲ	五味子
马鞭草科植物单叶蔓荆	Vitex trifolia L. var. simplicifolia Cham.			Ⅲ	蔓荆子
马鞭草科植物蔓荆	Vitex trifolia L.			Ⅲ	蔓荆子
使君子科植物诃子	Terminalia chebula Retz.			Ⅲ	诃　子
使君子科植物绒毛诃子	Terminalia chebula Retz. var. tomentella Kurt.			Ⅲ	诃　子
山茱萸科植物山茱萸	Cornus officinalis sieb. et Zucc.			Ⅲ	山茱萸
兰科植物环草石斛	Dendrobium loddigessii Rolfe.			Ⅲ	石　斛
兰科植物马鞭石斛	Dendrobium fimbriatum Hook. var. oculatum Hook.			Ⅲ	石　斛
兰科植物黄草石斛	Dendrobium chrysanthum Wall.			Ⅲ	石　斛
兰科植物铁皮石斛	Dendrobium candidum Wall. ex Lindl.			Ⅲ	石　斛
兰科植物金钗石斛	Dendrobium nobile Lindl.			Ⅲ	石　斛
伞形科植物新疆阿魏	Ferula sinkiangensis K. M. shep.			Ⅲ	阿　魏
伞形科植物阜康阿魏	Ferula fukanensis K. M. Shen.			Ⅲ	阿　魏
木犀科植物连翘	Forsythia suspensa (Thunb.) Vahl			Ⅲ	连　翘
伞形科植物羌活	Notopterygium incisum Ting ex H. T. Chang			Ⅲ	羌　活
伞形科植物宽叶羌活	Notopterygium forbesii Boiss.			Ⅲ	羌　活

注：１．本名录中的中名、学名、药材名称以《中华人民共和国药典》（１９８５年版一部）为依据。

２．本名录收载野生药材物种７６种，中药材４２种。其中只列入同一物种有代表性的药材名称。

*注：本文格式遵循《全国人大法规备案审查信息平台电子文件格式规范（试行）》标准。

©北大法宝：（www.pkulaw.com）专业提供法律信息、法学知识和法律软件领域各类解决方案。北大法宝为您提供丰富的参考资料，正式引用法规条文时请与标准文本核对。

欢迎查看所有产品和服务。

法宝快讯： 如何快速找到您需要的检索结果？ 法宝 V6 有何新特色？

扫描二维码阅读原文

原文链接：https://www.pkulaw.com/chl/934d0f9c50222f04bdfb.html
