# Supplementary material for: China’s Legal Protection System for Pangolins: Past, Present, and Future
Source: Animals (Basel). 2025 Aug 18;15(16):2422. doi: 10.3390/ani15162422 (PMC12383201; doi:10.3390/ani15162422)
Supplement: Supplementary file 1 [file animals-15-02422-s001.zip › Supplementary Material S2 -Full Texts of Laws and Regulations Related to Pangolins in China/【5】国家重点保护野生动物名录(FBM-CLI.4.pdf]

国家重点保护野生动物名录

制定机关： 林业部(已变更) 农业部(已撤销) 机构沿革

发文字号：中华人民共和国林业部、中华人民共和国农业部第1号令

批准机关： 国务院

公布日期：1989.01.14

批准日期：1989.01.14

施行日期：1989.01.14

时效性： 已被修改

效力位阶： 部门规章

法规类别： 野生动植物资源

修改依据：国家重点保护野生动物名录(2003)

中华人民共和国林业部、中华人民共和国农业部令

（第1号）

《国家重点保护野生动物名录》，已于1988年12月10日经国务院批准，现予发布施行。

林业部部长　高德占

农业部部长　何康

1989年1月14日

国家重点保护野生动物名录

中名	学名	保护级别 I级	保护级别 II级
兽纲	MAMMALIA		
灵长目	PRIMATES		
懒猴科	Lorisidae		
蜂猴（所有种）	Nycticebus spp.	I	
猴科	Cercopithecidae		
短尾猴	Macaca arctoides		II
熊　猴	Macaca assamensis	I	
台湾猴	Macaca cycolpis	I	
猕　猴	Macaca mulatta		II
豚尾猴	Macaca nemestrina	I	
藏酋猴	Macaca thibetana		II
叶猴（所有种）	Presbytis spp.	I	
金丝猴（所有种）	Rhinopithecus spp.	I	
猩猩科	Pongidae		
长臂猿（所有种）	Hylobates spp.	I	
鳞甲目	PHOLIDOTA		
鲮鲤科	Manidae		
穿山甲	Manis pentadactyla		II
食肉目	CARNIVORA		
犬科	Canidae		
豺	Cuon alpinus		II
熊科	Ursidae		
黑熊	Selenarctos thibetanus		II
棕熊	Ursus arctos		II
（包括马熊）	(U.a.pruinosus)		
马来熊	Helarctos malayanus	I	
浣熊科	Procyonidae		
小熊猫	Ailurus fulgens		II
大熊猫科	Ailuropodidae		
大熊猫	Ailuropoda melanoleuca	I	
鼬科	Mustelidae		
石貂	Martes foina		II
紫貂	Martes zibellina	I	
黄喉貂	Martes flavigula		II
貂熊	Gulo gulo	I	
* 水獭（所有种）	Lutra spp.		II
* 小爪水獭	Aonyx cinerea		II
灵猫科	Viverridae		
斑林狸	Prionodon pardicodor		II
大灵猫	Viverra zibetha		II
小灵猫	Viverricula indica		II
熊狸	Arctictis binturong	I	
猫科	Felidae		
草原斑猫	Felis lybica(＝silvestris)		II
荒漠猫	Felis bieti		II
丛林猫	Felis chaus		II
猞猁	Felis lynx		II
兔狲	Felis manul		II
金猫	Felis temmincki		II
渔猫	Felis viverrinus		II
云豹	Neofelis nebulosa	I	
豹	Panthera pardus	I	
虎	Panthera tigris	I	
雪豹	Panthera uncia	I	
* 鳍足目（所有种）	PINNIPEDIA		II
海牛目	SIRENIA		
儒艮科	Dugongidae		
* 儒艮	Dugong dugong	I	
鲸目	CETACEA		
喙豚科	Platanistidae		
* 白暨豚	Lipotes vexillifer	I	
海豚科	Delphinidae		
* 中华白海豚	Sousa chinensis	I	
* 其它鲸类	(Cetacea)		II
长鼻目	PROBOSCIDEA		
象科	Elephantidae		
亚洲象	Elephas maximus	I	

奇蹄目	PERISSODACTYLA		
马科	Equidae		
蒙古野驴	Equus hemionus	I	
西藏野驴	Equus kiang	I	
野马	Equus Przewalskii	I	
偶蹄目	ARTIODACTYLA		
驼科	Camelidae		
野骆驼	Camelus ferus		
	(=bactrianus)	I	
鼷鹿科	Tragulidae		
鼷鹿	Tragulus javanicus	I	
麝科	Moschidae		
麝（所有种）	Moschus spp.		II
鹿科	Cervidae		
河麂	Hydropotes inermis		II
黑麂	Muntiacus crinifrons	I	
白唇鹿	Cervus albirostris	I	
马鹿	Cervus elaphus		II
（包括白臀鹿）	(C.e.macneilli)		
坡鹿	Cervus eldi	I	
梅花鹿	Cervus nippon	I	
豚鹿	Cervus porcinus	I	
水鹿	Cervus unicolor		II
米鹿	Elaphurus davidianus	I	
驼鹿	Alces alces		II
牛科	Bovidae		
野牛	Bos gaurus	I	
野牦牛	Bos mutus(=grunniens)	I	
黄羊	Procapra gutturosa		II
普氏原羚	Procapra przewalskii	I	
藏原羚	Procapra picticaudata		II
鹅喉羚	Gazella subgutturosa		II
藏羚	Pantholops hodgsoni	I	
高鼻羚羊	Saiga tatarica	I	
扭角羚	Budorcas taxicolor	I	
鼠羚	Capricornis sumatraensis		II
台湾鼠羚	Capricornis crispus	I	
赤斑羚	Naemorhedus cranbrooki	I	
斑羚	Naemorhedus goral		II
塔尔羊	Hemitragus jemlahicus	I	
北山羊	Capra ibex	I	
岩羊	Pseudois nayaur		II
盘羊	Ovis ammon		II
兔形目	LAGOMORPHA		
兔　科	Leporidae		
海南兔	Lepus peguensis hainanus		II
雪兔	Lepus timidus		II
塔里木兔	Lepus yarkqndensis		II
啮齿目	RODENTIA		
松鼠科	Sciuridae		
巨松鼠	Ratufa bicolor		II
河狸科	Castoridae		
河狸	Castor fiber	I	
	鸟纲 AVES		
辟虎目	PODICIPEDIFORMES		
辟虎科	Podicipedidae		
角辟虎	Podiceps auritus		II
赤颈辟虎	Podiceps grisegena		II
鹱形目	PROCELLARIIFORMES		
信天翁科	Diomedeidae		
短尾信天翁	Diomedea albatrus	I	
鹈形目	PELECANIFORMES		
鹈鹕科	Pelecanidae		
鹈鹕（所有种）	pelecanus spp.		II
坚鸟科	Sulidae		
坚鸟（所有种）	sula spp.		II
鸬鹚科	Phalacrocoracidae		
海鸬鹚	Phalacrocorax pelagicus		II
黑颈鸬鹚	Phalacrocorax niger		II
军舰鸟科	Fregatidae		
白腹军舰鸟	Fregata andrewsi	I	
鹳形目	CICONIIFORMES		
鹭科	Ardeidae		
黄嘴白鹭	Egretta eulophotes		II
岩鹭	Egretta sacra		II
海南虎斑	Gorsachius magnificus		II
小苇开	Ixbrychus minutus		II
鹳科	Ciconiidae		

彩鹳	Ibis leucocephalus		II
白鹳	Ciconia ciconia	I	
黑鹳	Ciconia nigra	I	
四科	Threskiornithidae		
白四	Threskiornis aethiopicus		II
黑四	Pseudibis papillosa		II
朱四	Nipponia nippon	I	
彩四	Plegdis falcinellus		II
白琵鹭	Platalen leucorodia		II
黑脸琵鹭	Platalen minor		II
雁形目	ANSERIFORMES		
鸭科	Anatidae		
红胸黑雁	Branta ruficollis		II
白额雁	Anser albifrons		II
天鹅（所有种）	Cygnus spp.		II
鸳鸯	Aix galericulata		II
中华秋沙鸭	Mergus squamtus	I	
隼形目	FALCONIFORMES		
鹰科	Accipitridae		
金雕	Aquila chrysactus	I	
白肩雕	Aquila heliaca	I	
玉带海雕	Haliaeetus leucoryphus	I	
白尾海雕	Haliaeetus albicilla	I	
虎头海雕	Haliaeetus pelagicus	I	
拟兀鹫	Pseudogyps bengalensis	I	
胡兀鹫	Gypaetus barbatus	I	
其它鹰类	(Accipitridae)		II
隹科（所有种）	Falconidae		II
鸡形目	GALLIFORMES		
松鸡科	Tetraonidae		
细嘴松鸡	Tetrao parvirostris	I	
黑琴鸡	Lyrurus tetrix		II
柳雷鸡	Lagopus lagopus		II
岩雷鸡	Lagopus mutus		II
镰翅鸡	Falcipennis falcipcnnis		II
花尾榛鸡	Tetrastes bonasia		II
斑尾榛鸡	Tetrastes sewerzowi	I	
雉科	Phasianidae		
雪鸡（所有种）	Tetraogallus spp.		II
雉鹑	Tetraophasis obscurus	I	
四川山鹧鸪	Arborophila rufipectus	I	
海南山鹧鸪	Arborophila ardens	I	
血雉	Lthaginis cruentus		II
黑头角雉	Tragopan melanocephalus	I	
红胸角雉	Tragopan satyra	I	
灰腹角雉	Tragopan blythii	I	
红腹角雉	Tragopan temminckii		II
黄腹角雉	Tragopan caboti	I	
虹雉（所有种）	Lophophorus spp.	I	
藏马鸡	Crossoptilon crossoptilon		II
蓝马鸡	Crossoptilon anritum		II
褐马鸡	Crossoptilon mantchuricum	I	
黑鹇	Lophura leucomelana		II
白鹇	Lophura mycthemera		II
蓝鹇	Lophura swinnoii	I	
原鸡	Gallus gallus		II
勺鸡	Pucrasia macrolopha		II
黑颈长尾雉	Syrmaticus humiae	I	
白冠长尾雉	Syrmaticus reevesii		II
白颈长尾雉	Syrmaticus ellioti	I	
黑长尾雉	Syrmaticus mikado	I	
锦鸡（所有种）	Chrysolophas spp.		II
孔雀雉	Polyplcctron bicalcaratum	I	
绿孔雀	Pauo muticus	I	
鹤形目	GRUIFORMES		
鹤科	Gruidae		
灰鹤	Grus grus		II
黑颈鹤	Grus nigricollis	I	
白头鹤	Grus monacha	I	
沙丘鹤	Grus canadensis		II
丹顶鹤	Grus japoncnsis	I	
白枕鹤	Grus vipio		II
白鹤	Grus leucogeranus	I	
赤颈鹤	Grus antigone	I	
蓑羽鹤	Anthropoides virgo		II
秧鸡科	Rallidae		
长脚秧鸡	Crex crex		II
姬田鸡	Porzana parra		II
棕背田鸡	Porzana bicolor		II
花田鸡	Cotvrnicops novreboracensis		II
鸨科	Otidae		
鸨（所有种）	Otis spp.	I	

行形目	CHARADRIIFORMES		
雉行科	Jacanidae		
铜翅水雉	Metopidius indicus		II
科	Scolopacidae		
小杓	Numenius borealis		II
小青脚	Tringa guttifer		II
燕行科	Glareolidae		
灰燕行	Glareola lactea		II
鸥形目	LARIFORMES		
鸥科	Laridae		
遗鸥	Larus relictus	I	
小鸥	Larus minutus		II
黑浮鸥	Chlidonias niger		II
黄嘴河燕鸥	Sterna aurantia		II
黑嘴端凤头燕鸥	Thalasseus zimmermanni		II
鸽形目	COLUMBIFORMES		
沙鸡科	Pteroclididae		
黑腹沙鸡	Pterocles orientalis		II
鸠鸽科	Columbidae		
绿鸠（所有种）	Treron spp		II
黑颏果鸠	Ptilinopus leclancheri		II
皇鸠（所有种）	Ducula spp		II
斑尾林鸽	Columba palumbus		II
鹃鸠（所有种）	Macropygia spp		II
鹦形目	PSITTACIFORMES		
鹦鹉科（所有种）	Psittacidae		II
号形目	CUCULIFORMES		
杜鹃科	Cuculidae		
鸦鹃（所有种）	Centropus spp.		II
形目（所有种）	STRIGIFORMES		II
雨燕目	APODIFORMES		
雨燕科	Apodidae		
灰喉针尾雨燕	Hirundapus cochinchinensis		II
凤头雨燕科	Hemiprocnidae		II
凤头雨燕	Hemiprocne longipennis		II
咬鹃目	TROGONIFOPMES		
咬鹃科	Trogonidae		
橙胸咬鹃	Harpactes oreskios		II
佛法僧目	CORACIIFORMES		
翠鸟科	Alcedinidae		
蓝耳翠鸟	Alcedo meninting		II
鹳嘴翠鸟	Pelargopsis capensis		II
蜂虎科	Meropidae		
黑胸蜂虎	Merops leschenaulti		II
绿喉蜂虎	Merops orientalis		II
犀鸟科（所有种）	Bucerotidae		II
列形目	PICIFORMES		
啄木鸟科	Picidae		
白腹黑啄木鸟	Dryocopus javensis		II
雀形目	PASSERIFORMES		
阔嘴鸟科（所有种）	Eurylaimidae		II
八色鹅科（所有种）	Pitidae		II
爬行纲　REPTILIA			
龟鳖目	TESTUDOFORMES		
龟科	Emydidae		
* 地龟	Geoemyda spengleri		II
* 三线闭壳龟	cuora frifasciata		II
* 云南闭壳龟	cuora yunnanensis		II
陆龟科	Testudinidae		
四爪陆龟	testudo horsfieldi	I	
凹甲陆龟	Manouria impressa		II
海龟科	Cheloniidae		
* 希龟	Caretta caretta		II
* 绿海龟	Chelomia mydas		II
* 玳瑁	Eretmochelys imbricata		II
* 太平洋丽龟	Lepidochelys clivacea		II
棱皮龟科	Dermochelyidae		
* 棱皮龟	Dermochelys coriacea		II
鳖科	Trionychidae		
* 鼋	Pelochelys bibroni	I	
* 山瑞鳖	Trionyx steindachneri		II
蜥蜴目	LACERTIFORMES		
壁虎科	Gekkonidae		
大壁虎	Gekko gecko		II
鳄蜥科	Shinisauridac		

鳄蜥	Shinisaurus crocodilurus	I	
巨蜥科	Varanidae		
巨蜥	Varanus salvator	I	
蛇目	SERPENTIFORMES		
蟒科	Boidae		
蟒	Python molurus	I	
鳄目	CROCODILIFORMES		
电科	Alligatoridae		
扬子鳄	Alligator sinensis	I	
两栖纲 AMPHIBIA			
有尾目	CAUDATA		
隐鳃鲵科	Cryptobranchidae		
* 鲵大	Andrias davidianus		II
蝾螈科	Salamandridae		
* 细痣疣螈	Tylototriton asperrimus		II
* 镇海疣螈	Tylototriton chinhaiensis		II
* 贵州疣螈	Tylototriton kweichowensis		II
* 大凉疣螈	Tylototriton taliangensis		II
* 红瘰疣螈	Tylototriton verrucosus		II
无尾目	ANURA		
蛙科	Ranidae		
虎纹蛙	Rana tigrina		II
鱼纲 PISCES			
鲈形目	PERCIFORMES		
石首鱼科	Sciaenidae		
* 黄唇鱼	Bahaba flavolabiata		II
杜父鱼科	Cottidae		
* 松江鲈鱼	Trachidermus fasciatus		II
海龙鱼目	SYNGNATHIFORMES		
海龙鱼科	Syngnathidae		
* 克氏海马鱼	Hippocampus kelloggi		II
鲤形目	CYPRINIFORMES		
胭脂鱼科	Catostomidae		
* 胭脂鱼	Myxocyprinus asiaticus		II
鲤科	Cyprinidae		
* 唐鱼	Tanichthys albonubes		II
* 大头鲤	Cyprinus pellegrini		II
金线鲤	Sinocyclocheilus grahami grahami		II
* 新疆大头鱼	Aspiorhynchus laticeps	I	
* 大理裂腹鱼	Schizothorax taliensis		II
鳗鲡目	ANGUILLIFOMES		
鳗鲡科	Anguillidae		
* 花鳗鲡	Anguilla marmorata		II
鲑形目	SALMONIFORMES		
鲑科	Salmonidae		
* 川陕哲罗鲑	Hucho bleekcri		II
* 秦岭细鳞鲑	Brachymystax lenok tsinlingensis		II
鲟形目	ACIPENSERIFORMES		
鲟科	Acipenseridae		
* 中华鲟	Acipenser sinensis	I	
* 达氏鲟	Acipenser dabryanus	I	
匙吻鲟科	polyodontidae		
* 白鲟	Psephurus gladius	I	
文昌鱼纲 APPENDICULARIA			
文昌鱼目	AMPHIOXIFORMES		
文昌鱼科	Branchiostomatidae		
* 文昌鱼	Branchiostoma belcheri		II
珊瑚纲 ANTHOZOA			
柳珊瑚目	GORGONACEA		
红珊瑚科	Coralliidae		
* 红珊瑚	Corallium spp.	I	
腹足纲 GASTROPODA			
中腹足目	MESOGASTROPODA		
宝贝科	Cypraeidae		
* 虎斑宝贝	Cypraea tigris		II
冠螺科	Cassididae		
* 冠螺	Cassis cornuta		II
瓣鳃纲 LAMELLIBRANCHIA			

异柱目	ANISOMYARIA		
珍珠贝科	Pteriidae		
* 大珠母贝	Pinctada maxima		II
真瓣鳃目	EULAMELLIBRANCHIA		
砗磲科	Tridacnidae		
* 库氏砗磲	Tridacna cookiana	I	
蚌科	Unionidae		
* 佛耳丽蚌	Lamprotula mansuyi		II
头足纲 CEPHALOPODA			
四鳃目	TETRABRANCHIA		
鹦鹉螺科	Nautilidae		
鹦鹉螺	Nautilus pompilius	I	
昆虫纲 INSECTA			
双尾目	DIPLURA		
铗八科	Japygidae		
伟铗八	Atlasjapyx atlas		II
蜻蜓目	ODONATA		
箭蜓科	Gomphidae		
尖板曦箭蜓	Heliogomphus retroflexus		II
宽纹北箭蜓	Ophiogomphus spinicorne		II
缺翅目	ZORAPTERA		
缺翅虫科	Zorotypidae		
中华缺翅虫	Zorotypus sinensis		II
墨脱缺翅虫	Zorotypus medoensis		II
蛩蠊目	GRYLLOBLATTODEA		
蛩蠊科	Grylloblattidae		
中华蛩蠊	Galloisiana sinensis	I	
鞘翅目	COLEOPTERA		
步甲科	Carabidae		
拉步甲	Carabus(Coptolabrus)lafossei		II
硕步甲	Carabus(Apotopterus)dauidi		II
臂金龟科	Euchiridae		
彩臂金龟（所有种）	Cheirotonus spp.		II
犀金龟科	Dynastidae		
叉犀金龟	Allomyrina dauidis		II
鳞翅目	LEPIDOPTERA		
凤蝶科	Papilionidae		
金斑喙凤蝶	Teinopalpus aureus	I	
双尾褐凤蝶	Bhutanitis mansfieldi		II
三尾褐凤蝶	Bhutanitis thaidina dongchuanensis		II
中华虎凤蝶	Luchdorfia chinensis huashanensis		II
绢蝶科	Parnassidae		
阿波罗绢蝶	Parnassius apollo		II
肠鳃纲 ENTEROPNEUSTA			
柱头虫科	Balanoglossidae		
* 多鳃孔舌形虫	Glossobalanus polybranchioporus	I	
玉钩虫科	Harrimaniidae		
* 黄岛长吻虫	Saccoglossus hwangtauensis	I	

注：标“*”者，由渔业行政主管部门主管；未标“*”者，由林业行政主管部门主管。

*注：本文格式遵循《全国人大法规备案审查信息平台电子文件格式规范（试行）》标准。

©北大法宝：（www.pkulaw.com）专业提供法律信息、法学知识和法律软件领域各类解决方案。北大法宝为您提供丰富的参考资料，正式引用法规条文时请与标准文本核对。

欢迎查看所有产品和服务。

法宝快讯： 如何快速找到您需要的检索结果？ 法宝 V6 有何新特色？

扫描二维码阅读原文

原文链接：https://www.pkulaw.com/chl/00af3eb30019ee7fbdfb.html
