# Supplementary material for: China’s Legal Protection System for Pangolins: Past, Present, and Future
Source: Animals (Basel). 2025 Aug 18;15(16):2422. doi: 10.3390/ani15162422 (PMC12383201; doi:10.3390/ani15162422)
Supplement: Supplementary file 1 [file animals-15-02422-s001.zip › Supplementary Material S2 -Full Texts of Laws and Regulations Related to Pangolins in China/【6】中华人民共和国野生动物保护法(2022修订)(FBM-CLI.1.pdf]

# 中华人民共和国野生动物保护法(2022修订)

制定机关： 全国人大常委会

发文字号：中华人民共和国主席令第126号

公布日期：2022.12.30

施行日期：2023.05.01

时效性： 现行有效

效力位阶： 法律

法规类别： 野生动植物资源 森林和野生动植物保护区

2018-2022对照 2016-2018编注 2009-2016对照 2004-2009编注 1988-2004编注

中华人民共和国主席令

（第一二六号）

《中华人民共和国野生动物保护法》已由中华人民共和国第十三届全国人民代表大会常务委员会第三十八次会议于2022年12月30日修订通过，现予公布，自2023年5月1日起施行。

中华人民共和国主席　习近平

2022年12月30日

中华人民共和国野生动物保护法

（1988年11月8日第七届全国人民代表大会常务委员会第四次会议通过

根据2004年8月28日第十届全国人民代表大会常务委员会第十一次会议《关于修改〈中华人民共和国野生动物保护法〉的决定》第一次修正

根据2009年8月27日第十一届全国人民代表大会常务委员会第十次会议《关于修改部分法律的决定》第二次修正

2016年7月2日第十二届全国人民代表大会常务委员会第二十一次会议第一次修订

根据2018年10月26日第十三届全国人民代表大会常务委员会第六次会议《关于修改〈中华人民共和国野生动物保护法〉等十五部法律的决定》第三次修正

2022年12月30日第十三届全国人民代表大会常务委员会第三十八次会议第二次修订）

目　　录

第一章　总　　则

第二章　野生动物及其栖息地保护

第三章　野生动物管理

第四章　法律责任

第五章　附　　则

## 第一章　总　　则

**第一条**

为了保护野生动物，拯救珍贵、濒危野生动物，维护生物多样性和生态平衡，推进生态文明建设，促进人与自然和谐共生，制定本法。

第二条

在中华人民共和国领域及管辖的其他海域，从事野生动物保护及相关活动，适用本法。

本法规定保护的野生动物，是指珍贵、濒危的陆生、水生野生动物和有重要生态、科学、社会价值的陆生野生动物。

本法规定的野生动物及其制品，是指野生动物的整体（含卵、蛋）、部分及衍生物。

珍贵、濒危的水生野生动物以外的其他水生野生动物的保护，适用《中华人民共和国渔业法》等有关法律的规定。

第三条　野生动物资源属于国家所有。

国家保障依法从事野生动物科学研究、人工繁育等保护及相关活动的组织和个人的合法权益。

第四条

国家加强重要生态系统保护和修复，对野生动物实行保护优先、规范利用、严格监管的原则，鼓励和支持开展野生动物科学研究与应用，秉持生态文明理念，推动绿色发展。

第五条

国家保护野生动物及其栖息地。县级以上人民政府应当制定野生动物及其栖息地相关保护规划和措施，并将野生动物保护经费纳入预算。

国家鼓励公民、法人和其他组织依法通过捐赠、资助、志愿服务等方式参与野生动物保护活动，支持野生动物保护公益事业。

本法规定的野生动物栖息地，是指野生动物野外种群生息繁衍的重要区域。

第六条

任何组织和个人有保护野生动物及其栖息地的义务。禁止违法猎捕、运输、交易野生动物，禁止破坏野生动物栖息地。

社会公众应当增强保护野生动物和维护公共卫生安全的意识，防止野生动物源性传染病传播，抵制违法食用野生动物，养成文明健康的生活方式。

任何组织和个人有权举报违反本法的行为，接到举报的县级以上人民政府野生动物保护主管部门和其他有关部门应当及时依法处理。

第七条

国务院林业草原、渔业主管部门分别主管全国陆生、水生野生动物保护工作。

县级以上地方人民政府对本行政区域内野生动物保护工作负责，其林业草原、渔业主管部门分别主管本行政区域内陆生、水生野生动物保护工作。

县级以上人民政府有关部门按照职责分工，负责野生动物保护相关工作。

第八条

各级人民政府应当加强野生动物保护的宣传教育和科学知识普及工作，鼓励和支持基层群众性自治组织、社会组织、企业事业单位、志愿者开展野生动物保护法律法规、生态保护等知识的宣传活动；组织开展对相关从业人员法律法规和专业知识培训；依法公开野生动物保护和管理信息。

教育行政部门、学校应当对学生进行野生动物保护知识教育。

新闻媒体应当开展野生动物保护法律法规和保护知识的宣传，并依法对违法行为进行舆论监督。

第九条

在野生动物保护和科学研究方面成绩显著的组织和个人，由县级以上人民政府按照国家有关规定给予表彰和奖励。

## 第二章　野生动物及其栖息地保护

第十条　国家对野生动物实行分类分级保护。

国家对珍贵、濒危的野生动物实行重点保护。国家重点保护的野生动物分为一级保护野生动物和二级保护野生动物。国家重点保护野生动物名录，由国务院野生动物保护主管部门组织科学论证评估后，报国务院批准公布。

有重要生态、科学、社会价值的陆生野生动物名录，由国务院野生动物保护主管部门征求国务院农业农村、自然资源、科学技术、生态环境、卫生健康等部门意见，组织科学论证评估后制定并公布。

地方重点保护野生动物，是指国家重点保护野生动物以外，由省、自治区、直辖市重点保护的野生动物。地方重点保护野生动物名录，由省、自治区、直辖市人民政府组织科学论证评估，征求国务院野生动物保护主管部门意见后制定、公布。

对本条规定的名录，应当每五年组织科学论证评估，根据论证评估情况进行调整，也可以根据野生动物保护的实际需要及时进行调整。

第十一条

县级以上人民政府野生动物保护主管部门应当加强信息技术应用，定期组织或者委托有关科学研究机构对野生动物及其栖息地状况进行调查、监测和评估，建立健全野生动物及其栖息地档案。

对野生动物及其栖息地状况的调查、监测和评估应当包括下列内容：

（一）野生动物野外分布区域、种群数量及结构；

（二）野生动物栖息地的面积、生态状况；

（三）野生动物及其栖息地的主要威胁因素；

（四）野生动物人工繁育情况等其他需要调查、监测和评估的内容。

**第十二条**

国务院野生动物保护主管部门应当会同国务院有关部门，根据野生动物及其栖息地状况的调查、监测和评估结果，确定并发布野生动物重要栖息地名录。

省级以上人民政府依法将野生动物重要栖息地划入国家公园、自然保护区等自然保护地，保护、恢复和改善野生动物生存环境。对不具备划定自然保护地条件的，县级以上人民政府可以采取划定禁猎（渔）区、规定禁猎（渔）期等措施予以保护。

禁止或者限制在自然保护地内引入外来物种、营造单一纯林、过量施洒农药等人为干扰、威胁野生动物生息繁衍的行为。

自然保护地依照有关法律法规的规定划定和管理，野生动物保护主管部门依法加强对野生动物及其栖息地的保护。

**第十三条**

县级以上人民政府及其有关部门在编制有关开发利用规划时，应当充分考虑野生动物及其栖息地保护的需要，分析、预测和评估规划实施可能对野生动物及其栖息地保护产生的整体影响，避免或者减少规划实施可能造成的不利后果。

禁止在自然保护地建设法律法规规定不得建设的项目。机场、铁路、公路、航道、水利水电、风电、光伏发电、围堰、围填海等建设项目的选址选线，应当避让自然保护地以及其他野生动物重要栖息地、迁徙洄游通道；确实无法避让的，应

当采取修建野生动物通道、过鱼设施等措施，消除或者减少对野生动物的不利影响。

建设项目可能对自然保护地以及其他野生动物重要栖息地、迁徙洄游通道产生影响的，环境影响评价文件的审批部门在审批环境影响评价文件时，涉及国家重点保护野生动物的，应当征求国务院野生动物保护主管部门意见；涉及地方重点保护野生动物的，应当征求省、自治区、直辖市人民政府野生动物保护主管部门意见。

**第十四条**

各级野生动物保护主管部门应当监测环境对野生动物的影响，发现环境影响对野生动物造成危害时，应当会同有关部门及时进行调查处理。

**第十五条**

国家重点保护野生动物和有重要生态、科学、社会价值的陆生野生动物或者地方重点保护野生动物受到自然灾害、重大环境污染事故等突发事件威胁时，当地人民政府应当及时采取应急救助措施。

国家加强野生动物收容救护能力建设。县级以上人民政府野生动物保护主管部门应当按照国家有关规定组织开展野生动物收容救护工作，加强对社会组织开展野生动物收容救护工作的规范和指导。

收容救护机构应当根据野生动物收容救护的实际需要，建立收容救护场所，配备相应的专业技术人员、救护工具、设备和药品等。

禁止以野生动物收容救护为名买卖野生动物及其制品。

第十六条

野生动物疫源疫病监测、检疫和与人畜共患传染病有关的动物传染病的防治管理，适用《中华人民共和国动物防疫法》等有关法律法规的规定。

第十七条

国家加强对野生动物遗传资源的保护，对濒危野生动物实施抢救性保护。

国务院野生动物保护主管部门应当会同国务院有关部门制定有关野生动物遗传资源保护和利用规划，建立国家野生动物遗传资源基因库，对原产我国的珍贵、濒危野生动物遗传资源实行重点保护。

第十八条

有关地方人民政府应当根据实际情况和需要建设隔离防护设施、设置安全警示标志等，预防野生动物可能造成的危害。

县级以上人民政府野生动物保护主管部门根据野生动物及其栖息地调查、监测和评估情况，对种群数量明显超过环境容量的物种，可以采取迁地保护、猎捕等种群调控措施，保障人身财产安全、生态安全和农业生产。对种群调控猎捕的野生

动物按照国家有关规定进行处理和综合利用。种群调控的具体办法由国务院野生动物保护主管部门会同国务院有关部门制定。

**第十九条**

因保护本法规定保护的野生动物，造成人员伤亡、农作物或者其他财产损失的，由当地人民政府给予补偿。具体办法由省、自治区、直辖市人民政府制定。有关地方人民政府可以推动保险机构开展野生动物致害赔偿保险业务。

有关地方人民政府采取预防、控制国家重点保护野生动物和其他致害严重的陆生野生动物造成危害的措施以及实行补偿所需经费，由中央财政予以补助。具体办法由国务院财政部门会同国务院野生动物保护主管部门制定。

在野生动物危及人身安全的紧急情况下，采取措施造成野生动物损害的，依法不承担法律责任。

## 第三章　野生动物管理

**第二十条**

在自然保护地和禁猎（渔）区、禁猎（渔）期内，禁止猎捕以及其他妨碍野生动物生息繁衍的活动，但法律法规另有规定的除外。

野生动物迁徙洄游期间，在前款规定区域外的迁徙洄游通道内，禁止猎捕并严格限制其他妨碍野生动物生息繁衍的活动。县级以上人民政府或者其野生动物保护

主管部门应当规定并公布迁徙洄游通道的范围以及妨碍野生动物生息繁衍活动的内容。

第二十一条　禁止猎捕、杀害国家重点保护野生动物。

因科学研究、种群调控、疫源疫病监测或者其他特殊情况，需要猎捕国家一级保护野生动物的，应当向国务院野生动物保护主管部门申请特许猎捕证；需要猎捕国家二级保护野生动物的，应当向省、自治区、直辖市人民政府野生动物保护主管部门申请特许猎捕证。

第二十二条

猎捕有重要生态、科学、社会价值的陆生野生动物和地方重点保护野生动物的，应当依法取得县级以上地方人民政府野生动物保护主管部门核发的狩猎证，并服从猎捕量限额管理。

第二十三条

猎捕者应当严格按照特许猎捕证、狩猎证规定的种类、数量或者限额、地点、工具、方法和期限进行猎捕。猎捕作业完成后，应当将猎捕情况向核发特许猎捕证、狩猎证的野生动物保护主管部门备案。具体办法由国务院野生动物保护主管部门制定。猎捕国家重点保护野生动物应当由专业机构和人员承担；猎捕有重要生态、科学、社会价值的陆生野生动物，有条件的地方可以由专业机构有组织开展。

持枪猎捕的，应当依法取得公安机关核发的持枪证。

第二十四条

禁止使用毒药、爆炸物、电击或者电子诱捕装置以及猎套、猎夹、捕鸟网、地枪、排铳等工具进行猎捕，禁止使用夜间照明行猎、歼灭性围猎、捣毁巢穴、火攻、烟熏、网捕等方法进行猎捕，但因物种保护、科学研究确需网捕、电子诱捕以及植保作业等除外。

前款规定以外的禁止使用的猎捕工具和方法，由县级以上地方人民政府规定并公布。

第二十五条

人工繁育野生动物实行分类分级管理，严格保护和科学利用野生动物资源。国家支持有关科学研究机构因物种保护目的人工繁育国家重点保护野生动物。

人工繁育国家重点保护野生动物实行许可制度。人工繁育国家重点保护野生动物的，应当经省、自治区、直辖市人民政府野生动物保护主管部门批准，取得人工繁育许可证，但国务院对批准机关另有规定的除外。

人工繁育有重要生态、科学、社会价值的陆生野生动物的，应当向县级人民政府野生动物保护主管部门备案。

人工繁育野生动物应当使用人工繁育子代种源，建立物种系谱、繁育档案和个体数据。因物种保护目的确需采用野外种源的，应当遵守本法有关猎捕野生动物的规定。

本法所称人工繁育子代，是指人工控制条件下繁殖出生的子代个体且其亲本也在人工控制条件下出生。

人工繁育野生动物的具体管理办法由国务院野生动物保护主管部门制定。

**第二十六条**

人工繁育野生动物应当有利于物种保护及其科学研究，不得违法猎捕野生动物，破坏野外种群资源，并根据野生动物习性确保其具有必要的活动空间和生息繁衍、卫生健康条件，具备与其繁育目的、种类、发展规模相适应的场所、设施、技术，符合有关技术标准和防疫要求，不得虐待野生动物。

省级以上人民政府野生动物保护主管部门可以根据保护国家重点保护野生动物的需要，组织开展国家重点保护野生动物放归野外环境工作。

前款规定以外的人工繁育的野生动物放归野外环境的，适用本法有关放生野生动物管理的规定。

**第二十七条**

人工繁育野生动物应当采取安全措施，防止野生动物伤人和逃逸。人工繁育的野生动物造成他人损害、危害公共安全或者破坏生态的，饲养人、管理人等应当依法承担法律责任。

**第二十八条**　禁止出售、购买、利用国家重点保护野生动物及其制品。

因科学研究、人工繁育、公众展示展演、文物保护或者其他特殊情况，需要出售、购买、利用国家重点保护野生动物及其制品的，应当经省、自治区、直辖市人民政府野生动物保护主管部门批准，并按照规定取得和使用专用标识，保证可追溯，但国务院对批准机关另有规定的除外。

出售、利用有重要生态、科学、社会价值的陆生野生动物和地方重点保护野生动物及其制品的，应当提供狩猎、人工繁育、进出口等合法来源证明。

实行国家重点保护野生动物和有重要生态、科学、社会价值的陆生野生动物及其制品专用标识的范围和管理办法，由国务院野生动物保护主管部门规定。

出售本条第二款、第三款规定的野生动物的，还应当依法附有检疫证明。

利用野生动物进行公众展示展演应当采取安全管理措施，并保障野生动物健康状态，具体管理办法由国务院野生动物保护主管部门会同国务院有关部门制定。

**第二十九条**

对人工繁育技术成熟稳定的国家重点保护野生动物或者有重要生态、科学、社会价值的陆生野生动物，经科学论证评估，纳入国务院野生动物保护主管部门制定的人工繁育国家重点保护野生动物名录或者有重要生态、科学、社会价值的陆生野生动物名录，并适时调整。对列入名录的野生动物及其制品，可以凭人工繁育许可证或者备案，按照省、自治区、直辖市人民政府野生动物保护主管部门或者

其授权的部门核验的年度生产数量直接取得专用标识，凭专用标识出售和利用，保证可追溯。

对本法第十条规定的国家重点保护野生动物名录和有重要生态、科学、社会价值的陆生野生动物名录进行调整时，根据有关野外种群保护情况，可以对前款规定的有关人工繁育技术成熟稳定野生动物的人工种群，不再列入国家重点保护野生动物名录和有重要生态、科学、社会价值的陆生野生动物名录，实行与野外种群不同的管理措施，但应当依照本法第二十五条第二款、第三款和本条第一款的规定取得人工繁育许可证或者备案和专用标识。

对符合《中华人民共和国畜牧法》第十二条第二款规定的陆生野生动物人工繁育种群，经科学论证评估，可以列入畜禽遗传资源目录。

**第三十条**

利用野生动物及其制品的，应当以人工繁育种群为主，有利于野外种群养护，符合生态文明建设的要求，尊重社会公德，遵守法律法规和国家有关规定。

野生动物及其制品作为药品等经营和利用的，还应当遵守《中华人民共和国药品管理法》等有关法律法规的规定。

**第三十一条**

禁止食用国家重点保护野生动物和国家保护的有重要生态、科学、社会价值的陆生野生动物以及其他陆生野生动物。

禁止以食用为目的猎捕、交易、运输在野外环境自然生长繁殖的前款规定的野生动物。

禁止生产、经营使用本条第一款规定的野生动物及其制品制作的食品。

禁止为食用非法购买本条第一款规定的野生动物及其制品。

**第三十二条**

禁止为出售、购买、利用野生动物或者禁止使用的猎捕工具发布广告。禁止为违法出售、购买、利用野生动物制品发布广告。

**第三十三条**

禁止网络平台、商品交易市场、餐饮场所等，为违法出售、购买、食用及利用野生动物及其制品或者禁止使用的猎捕工具提供展示、交易、消费服务。

**第三十四条**

运输、携带、寄递国家重点保护野生动物及其制品，或者依照本法第二十九条第二款规定调出国家重点保护野生动物名录的野生动物及其制品出县境的，应当持有或者附有本法第二十一条、第二十五条、第二十八条或者第二十九条规定的许可证、批准文件的副本或者专用标识。

运输、携带、寄递有重要生态、科学、社会价值的陆生野生动物和地方重点保护野生动物，或者依照本法第二十九条第二款规定调出有重要生态、科学、社会价

值的陆生野生动物名录的野生动物出县境的，应当持有狩猎、人工繁育、进出口等合法来源证明或者专用标识。

运输、携带、寄递前两款规定的野生动物出县境的，还应当依照《中华人民共和国动物防疫法》的规定附有检疫证明。

铁路、道路、水运、民航、邮政、快递等企业对托运、携带、交寄野生动物及其制品的，应当查验其相关证件、文件副本或者专用标识，对不符合规定的，不得承运、寄递。

**第三十五条**

县级以上人民政府野生动物保护主管部门应当对科学研究、人工繁育、公众展示展演等利用野生动物及其制品的活动进行规范和监督管理。

市场监督管理、海关、铁路、道路、水运、民航、邮政等部门应当按照职责分工对野生动物及其制品交易、利用、运输、携带、寄递等活动进行监督检查。

国家建立由国务院林业草原、渔业主管部门牵头，各相关部门配合的野生动物联合执法工作协调机制。地方人民政府建立相应联合执法工作协调机制。

县级以上人民政府野生动物保护主管部门和其他负有野生动物保护职责的部门发现违法事实涉嫌犯罪的，应当将犯罪线索移送具有侦查、调查职权的机关。

公安机关、人民检察院、人民法院在办理野生动物保护犯罪案件过程中认为没有犯罪事实，或者犯罪事实显著轻微，不需要追究刑事责任，但应当予以行政处罚的，应当及时将案件移送县级以上人民政府野生动物保护主管部门和其他负有野生动物保护职责的部门，有关部门应当依法处理。

第三十六条

县级以上人民政府野生动物保护主管部门和其他负有野生动物保护职责的部门，在履行本法规定的职责时，可以采取下列措施：

（一）进入与违反野生动物保护管理行为有关的场所进行现场检查、调查；

（二）对野生动物进行检验、检测、抽样取证；

（三）查封、复制有关文件、资料，对可能被转移、销毁、隐匿或者篡改的文件、资料予以封存；

（四）查封、扣押无合法来源证明的野生动物及其制品，查封、扣押涉嫌非法猎捕野生动物或者非法收购、出售、加工、运输猎捕野生动物及其制品的工具、设备或者财物。

第三十七条

中华人民共和国缔结或者参加的国际公约禁止或者限制贸易的野生动物或者其制品名录，由国家濒危物种进出口管理机构制定、调整并公布。

进出口列入前款名录的野生动物或者其制品，或者出口国家重点保护野生动物或者其制品的，应当经国务院野生动物保护主管部门或者国务院批准，并取得国家濒危物种进出口管理机构核发的允许进出口证明书。海关凭允许进出口证明书办理进出境检疫，并依法办理其他海关手续。

涉及科学技术保密的野生动物物种的出口，按照国务院有关规定办理。

列入本条第一款名录的野生动物，经国务院野生动物保护主管部门核准，按照本法有关规定进行管理。

第三十八条

禁止向境外机构或者人员提供我国特有的野生动物遗传资源。开展国际科学研究合作的，应当依法取得批准，有我国科研机构、高等学校、企业及其研究人员实质性参与研究，按照规定提出国家共享惠益的方案，并遵守我国法律、行政法规的规定。

第三十九条

国家组织开展野生动物保护及相关执法活动的国际合作与交流，加强与毗邻国家的协作，保护野生动物迁徙通道；建立防范、打击野生动物及其制品的走私和非法贸易的部门协调机制，开展防范、打击走私和非法贸易行动。

第四十条

从境外引进野生动物物种的，应当经国务院野生动物保护主管部门批准。从境外

引进列入本法第三十七条第一款名录的野生动物，还应当依法取得允许进出口证明书。海关凭进口批准文件或者允许进出口证明书办理进境检疫，并依法办理其他海关手续。

从境外引进野生动物物种的，应当采取安全可靠的防范措施，防止其进入野外环境，避免对生态系统造成危害；不得违法放生、丢弃，确需将其放生至野外环境的，应当遵守有关法律法规的规定。

发现来自境外的野生动物对生态系统造成危害的，县级以上人民政府野生动物保护等有关部门应当采取相应的安全控制措施。

**第四十一条**

国务院野生动物保护主管部门应当会同国务院有关部门加强对放生野生动物活动的规范、引导。任何组织和个人将野生动物放生至野外环境，应当选择适合放生地野外生存的当地物种，不得干扰当地居民的正常生活、生产，避免对生态系统造成危害。具体办法由国务院野生动物保护主管部门制定。随意放生野生动物，造成他人人身、财产损害或者危害生态系统的，依法承担法律责任。

**第四十二条**

禁止伪造、变造、买卖、转让、租借特许猎捕证、狩猎证、人工繁育许可证及专用标识，出售、购买、利用国家重点保护野生动物及其制品的批准文件，或者允许进出口证明书、进出口等批准文件。

前款规定的有关许可证书、专用标识、批准文件的发放有关情况，应当依法公开
。

第四十三条

外国人在我国对国家重点保护野生动物进行野外考察或者在野外拍摄电影、录像，应当经省、自治区、直辖市人民政府野生动物保护主管部门或者其授权的单位批准，并遵守有关法律法规的规定。

第四十四条

省、自治区、直辖市人民代表大会或者其常务委员会可以根据地方实际情况制定对地方重点保护野生动物等的管理办法。

## 第四章　法律责任

第四十五条

野生动物保护主管部门或者其他有关部门不依法作出行政许可决定，发现违法行为或者接到对违法行为的举报不依法处理，或者有其他滥用职权、玩忽职守、徇私舞弊等不依法履行职责的行为的，对直接负责的主管人员和其他直接责任人员依法给予处分；构成犯罪的，依法追究刑事责任。

第四十六条

违反本法第十二条第三款、第十三条第二款规定的，依照有关法律法规的规定处罚。

第四十七条

违反本法第十五条第四款规定，以收容救护为名买卖野生动物及其制品的，由县级以上人民政府野生动物保护主管部门没收野生动物及其制品、违法所得，并处野生动物及其制品价值二倍以上二十倍以下罚款，将有关违法信息记入社会信用记录，并向社会公布；构成犯罪的，依法追究刑事责任。

第四十八条

违反本法第二十条、第二十一条、第二十三条第一款、第二十四条第一款规定，有下列行为之一的，由县级以上人民政府野生动物保护主管部门、海警机构和有关自然保护地管理机构按照职责分工没收猎获物、猎捕工具和违法所得，吊销特许猎捕证，并处猎获物价值二倍以上二十倍以下罚款；没有猎获物或者猎获物价值不足五千元的，并处一万元以上十万元以下罚款；构成犯罪的，依法追究刑事责任：

（一）在自然保护地、禁猎（渔）区、禁猎（渔）期猎捕国家重点保护野生动物

；

（二）未取得特许猎捕证、未按照特许猎捕证规定猎捕、杀害国家重点保护野生动物；

（三）使用禁用的工具、方法猎捕国家重点保护野生动物。

违反本法第二十三条第一款规定，未将猎捕情况向野生动物保护主管部门备案的

，由核发特许猎捕证、狩猎证的野生动物保护主管部门责令限期改正；逾期不改正的，处一万元以上十万元以下罚款；情节严重的，吊销特许猎捕证、狩猎证。

第四十九条

违反本法第二十条、第二十二条、第二十三条第一款、第二十四条第一款规定，有下列行为之一的，由县级以上地方人民政府野生动物保护主管部门和有关自然保护地管理机构按照职责分工没收猎获物、猎捕工具和违法所得，吊销狩猎证，并处猎获物价值一倍以上十倍以下罚款；没有猎获物或者猎获物价值不足二千元的，并处二千元以上二万元以下罚款；构成犯罪的，依法追究刑事责任：

（一）在自然保护地、禁猎（渔）区、禁猎（渔）期猎捕有重要生态、科学、社会价值的陆生野生动物或者地方重点保护野生动物；

（二）未取得狩猎证、未按照狩猎证规定猎捕有重要生态、科学、社会价值的陆生野生动物或者地方重点保护野生动物；

（三）使用禁用的工具、方法猎捕有重要生态、科学、社会价值的陆生野生动物或者地方重点保护野生动物。

违反本法第二十条、第二十四条第一款规定，在自然保护地、禁猎区、禁猎期或者使用禁用的工具、方法猎捕其他陆生野生动物，破坏生态的，由县级以上地方人民政府野生动物保护主管部门和有关自然保护地管理机构按照职责分工没收猎获物、猎捕工具和违法所得，并处猎获物价值一倍以上三倍以下罚款；没有猎获

物或者猎获物价值不足一千元的，并处一千元以上三千元以下罚款；构成犯罪的，依法追究刑事责任。

违反本法第二十三条第二款规定，未取得持枪证持枪猎捕野生动物，构成违反治安管理行为的，还应当由公安机关依法给予治安管理处罚；构成犯罪的，依法追究刑事责任。

**第五十条**

违反本法第三十一条第二款规定，以食用为目的猎捕、交易、运输在野外环境自然生长繁殖的国家重点保护野生动物或者有重要生态、科学、社会价值的陆生野生动物的，依照本法第四十八条、第四十九条、第五十二条的规定从重处罚。

违反本法第三十一条第二款规定，以食用为目的猎捕在野外环境自然生长繁殖的其他陆生野生动物的，由县级以上地方人民政府野生动物保护主管部门和有关自然保护地管理机构按照职责分工没收猎获物、猎捕工具和违法所得；情节严重的，并处猎获物价值一倍以上五倍以下罚款，没有猎获物或者猎获物价值不足二千元的，并处二千元以上一万元以下罚款；构成犯罪的，依法追究刑事责任。

违反本法第三十一条第二款规定，以食用为目的交易、运输在野外环境自然生长繁殖的其他陆生野生动物的，由县级以上地方人民政府野生动物保护主管部门和市场监督管理部门按照职责分工没收野生动物；情节严重的，并处野生动物价值一倍以上五倍以下罚款；构成犯罪的，依法追究刑事责任。

第五十一条

违反本法第二十五条第二款规定，未取得人工繁育许可证，繁育国家重点保护野生动物或者依照本法第二十九条第二款规定调出国家重点保护野生动物名录的野生动物的，由县级以上人民政府野生动物保护主管部门没收野生动物及其制品，并处野生动物及其制品价值一倍以上十倍以下罚款。

违反本法第二十五条第三款规定，人工繁育有重要生态、科学、社会价值的陆生野生动物或者依照本法第二十九条第二款规定调出有重要生态、科学、社会价值的陆生野生动物名录的野生动物未备案的，由县级人民政府野生动物保护主管部门责令限期改正；逾期不改正的，处五百元以上二千元以下罚款。

第五十二条

违反本法第二十八条第一款和第二款、第二十九条第一款、第三十四条第一款规定，未经批准、未取得或者未按照规定使用专用标识，或者未持有、未附有人工繁育许可证、批准文件的副本或者专用标识出售、购买、利用、运输、携带、寄递国家重点保护野生动物及其制品或者依照本法第二十九条第二款规定调出国家重点保护野生动物名录的野生动物及其制品的，由县级以上人民政府野生动物保护主管部门和市场监督管理部门按照职责分工没收野生动物及其制品和违法所得，责令关闭违法经营场所，并处野生动物及其制品价值二倍以上二十倍以下罚款；情节严重的，吊销人工繁育许可证、撤销批准文件、收回专用标识；构成犯罪的，依法追究刑事责任。

违反本法第二十八条第三款、第二十九条第一款、第三十四条第二款规定，未持

有合法来源证明或者专用标识出售、利用、运输、携带、寄递有重要生态、科学、社会价值的陆生野生动物、地方重点保护野生动物或者依照本法第二十九条第二款规定调出有重要生态、科学、社会价值的陆生野生动物名录的野生动物及其制品的，由县级以上地方人民政府野生动物保护主管部门和市场监督管理部门按照职责分工没收野生动物，并处野生动物价值一倍以上十倍以下罚款；构成犯罪的，依法追究刑事责任。

违反本法第三十四条第四款规定，铁路、道路、水运、民航、邮政、快递等企业未按照规定查验或者承运、寄递野生动物及其制品的，由交通运输、铁路监督管理、民用航空、邮政管理等相关主管部门按照职责分工没收违法所得，并处违法所得一倍以上五倍以下罚款；情节严重的，吊销经营许可证。

**第五十三条**

违反本法第三十一条第一款、第四款规定，食用或者为食用非法购买本法规定保护的野生动物及其制品的，由县级以上人民政府野生动物保护主管部门和市场监督管理部门按照职责分工责令停止违法行为，没收野生动物及其制品，并处野生动物及其制品价值二倍以上二十倍以下罚款；食用或者为食用非法购买其他陆生野生动物及其制品的，责令停止违法行为，给予批评教育，没收野生动物及其制品，情节严重的，并处野生动物及其制品价值一倍以上五倍以下罚款；构成犯罪的，依法追究刑事责任。

违反本法第三十一条第三款规定，生产、经营使用本法规定保护的野生动物及其制品制作的食品的，由县级以上人民政府野生动物保护主管部门和市场监督管理

部门按照职责分工责令停止违法行为，没收野生动物及其制品和违法所得，责令关闭违法经营场所，并处违法所得十五倍以上三十倍以下罚款；生产、经营使用其他陆生野生动物及其制品制作的食品的，给予批评教育，没收野生动物及其制品和违法所得，情节严重的，并处违法所得一倍以上十倍以下罚款；构成犯罪的，依法追究刑事责任。

**第五十四条**

违反本法第三十二条规定，为出售、购买、利用野生动物及其制品或者禁止使用的猎捕工具发布广告的，依照《中华人民共和国广告法》的规定处罚。

**第五十五条**

违反本法第三十三条规定，为违法出售、购买、食用及利用野生动物及其制品或者禁止使用的猎捕工具提供展示、交易、消费服务的，由县级以上人民政府市场监督管理部门责令停止违法行为，限期改正，没收违法所得，并处违法所得二倍以上十倍以下罚款；没有违法所得或者违法所得不足五千元的，处一万元以上十万元以下罚款；构成犯罪的，依法追究刑事责任。

**第五十六条**

违反本法第三十七条规定，进出口野生动物及其制品的，由海关、公安机关、海警机构依照法律、行政法规和国家有关规定处罚；构成犯罪的，依法追究刑事责任。

**第五十七条**

违反本法第三十八条规定，向境外机构或者人员提供我国特有的野生动物遗传资

源的，由县级以上人民政府野生动物保护主管部门没收野生动物及其制品和违法所得，并处野生动物及其制品价值或者违法所得一倍以上五倍以下罚款；构成犯罪的，依法追究刑事责任。

**第五十八条**

违反本法第四十条第一款规定，从境外引进野生动物物种的，由县级以上人民政府野生动物保护主管部门没收所引进的野生动物，并处五万元以上五十万元以下罚款；未依法实施进境检疫的，依照《中华人民共和国进出境动植物检疫法》的规定处罚；构成犯罪的，依法追究刑事责任。

**第五十九条**

违反本法第四十条第二款规定，将从境外引进的野生动物放生、丢弃的，由县级以上人民政府野生动物保护主管部门责令限期捕回，处一万元以上十万元以下罚款；逾期不捕回的，由有关野生动物保护主管部门代为捕回或者采取降低影响的措施，所需费用由被责令限期捕回者承担；构成犯罪的，依法追究刑事责任。

**第六十条**

违反本法第四十二条第一款规定，伪造、变造、买卖、转让、租借有关证件、专用标识或者有关批准文件的，由县级以上人民政府野生动物保护主管部门没收违法证件、专用标识、有关批准文件和违法所得，并处五万元以上五十万元以下罚款；构成违反治安管理行为的，由公安机关依法给予治安管理处罚；构成犯罪的，依法追究刑事责任。

**第六十一条**

县级以上人民政府野生动物保护主管部门和其他负有野生动物保护职责的部门、机构应当按照有关规定处理罚没的野生动物及其制品，具体办法由国务院野生动物保护主管部门会同国务院有关部门制定。

**第六十二条**

县级以上人民政府野生动物保护主管部门应当加强对野生动物及其制品鉴定、价值评估工作的规范、指导。本法规定的猎获物价值、野生动物及其制品价值的评估标准和方法，由国务院野生动物保护主管部门制定。

**第六十三条**

对违反本法规定破坏野生动物资源、生态环境，损害社会公共利益的行为，可以依照《中华人民共和国环境保护法》、《中华人民共和国民事诉讼法》、《中华人民共和国行政诉讼法》等法律的规定向人民法院提起诉讼。

## 第五章　附　　则

**第六十四条**　本法自2023年5月1日起施行。

*注：本文格式遵循《全国人大法规备案审查信息平台电子文件格式规范（试行）》标准。

©北大法宝：（www.pkulaw.com）专业提供法律信息、法学知识和法律软件领域各类解决方案。北大法宝为您提供丰富的参考资料，正式引用法规条文时请与标准文本核对。

欢迎查看所有产品和服务。

法宝快讯： 如何快速找到您需要的检索结果？ 法宝 V6 有何新特色？

扫描二维码阅读原文

原文链接：https://www.pkulaw.com/chl/53181cd1be67b8f3bdfb.html
