# Supplementary material for: China’s Legal Protection System for Pangolins: Past, Present, and Future
Source: Animals (Basel). 2025 Aug 18;15(16):2422. doi: 10.3390/ani15162422 (PMC12383201; doi:10.3390/ani15162422)
Supplement: Supplementary file 1 [file animals-15-02422-s001.zip › Supplementary Material S2 -Full Texts of Laws and Regulations Related to Pangolins in China/【7】国务院办公厅关于当前非法捕杀、收购、倒卖珍稀野生动物情况的通报(FBM-CLI.2.pdf]

国务院办公厅关于当前非法捕杀、收购、倒卖珍稀野生动物情况的通报

制定机关： 国务院办公厅 机构沿革

发文字号：国办发明电〔1990〕11号

公布日期：1990.05.12

施行日期：1990.05.12

时效性： 现行有效

效力位阶： 国务院规范性文件

法规类别： 野生动植物资源

国务院办公厅关于当前非法捕杀、收购、倒卖珍稀野生动物情况的通报

（国办发明电[1990]11号　1990年5月12日）

各省、自治区、直辖市人民政府，国务院有关部门：

一九八九年秋冬以来，一些地方非法捕杀。收购、倒卖国家重点保护野生动物的案件显著增多，甚至连大熊猫、东北虎、金丝猴、雪豹、朱等极其珍贵的国家一级重点保护动物也不断惨遭猎杀，令人触目惊心。经国务院研究，现将非法捕杀情况通报如下：

一九八九年九月十日，陕西省佛坪自然保护区岳坝林业公安派出所在佛坪县公安局指挥协助下，抓获七名案犯，收缴大熊猫皮一张、金丝猴皮二张。

一九八九年九月二十六日，四川省大邑县沙坪村农民任事业等二人，在该县望台

乡用猎枪打死大熊猫一只，后在成都市贩卖大熊猫皮时被抓获。

一九八九年十一月十四日，吉林省蛟河县天北乡农民张国君等三人，窜到黑龙江省东方红林业局青山林场，用自行车改装的猎枪打死雄性东北虎一只（据调查，该林区仅有三只东北虎，濒临灭绝）。

一九八九年十二月二十日，新疆维吾尔自治区公安厅边防局反映，昭苏、特克斯等边境地区一九八九年捕杀马鹿（国家二级保护动物）近百头，还有苏越境人员在昭苏县非法收购。

一九八九年十二月三十日，河北省滦平县马营子乡农民高光华等四人，窜到北京市密云县番子牌乡山上，用猎枪打死金钱豹一只。在同一天，湖南省桑植县一只云豹被猎杀。

一九九0年一月十六日，北京市门头沟区清水乡山上一只金钱豹被猎套套死。

一九九0年一月二十日，广东省五华县农魏忠源等三人，在深圳市贩卖云豹二只（一只活体）和国家二级保护动物穿山甲、大鲵等，被深圳市检察机关查获。

一九九0年二月四日，四川省青川县马有农民景国龙等四人，用火枪非法猎杀大熊猫二只（其中一只为幼仔，皮长仅七十五公分），在非法卖皮张时被绵阳市公安机关抓获。

一九九0年二月二十五日，青海省湟中县五庄乡农民马忠民等三人非法猎杀并偷运雪豹十四只，国家二级保护动物岩羊七只，猞猁一只，被青海鱼政检查站查获。

一九九0年二月二十八日，山西省水济县于乡镇北梯肉联厂的两辆卡车，装运由青

海省天峻等县非法收购的十二吨约五百多只岩羊、黄羊，途经甘肃省河口时被铁路公安机关查扣。

一九九0年三月一日，陕西省洋县宝和乡发现一只亚成体朱被非法猎杀。朱是世界珍稀濒危鸟类。目前只有我国和日本残存三十多只。

为了坚决迅速地刹住乱捕滥猎的犯罪行为，进一步做好野生动物的保护工作，国务院要求：

一、各级人民政府，要把贯彻执行《野生动物保护法》列入政府领导的议事日程。野生动物资源较多的地方，要建立县长和主管县长对保护野生动物工作的责任制，近期要组织力量，对野生动物保护工作进行一次认真检查，堵塞漏洞，制定措施，建立制度，抓紧解决工作中存在的问题。

二、组织各有关部门集中力量抓紧查处案件。对一切非法捕杀、收购、倒卖国家一级、二级重点保护动物的案件，不论是个人还是单位，都必须按照国家《关于惩治捕杀国家重点保护的珍贵、濒危野生动物犯罪的补充规定》和其他有关法律规定，坚决从严惩处，不得姑息迁就。

三、各级公安机关要加强对枪支弹药的管理，对猎枪要进行一次认真的清查、整顿，严禁非法生产，严格销售和使用管理，收缴违法枪支。工商、渔政、海关、外贸、铁路、交通、邮政等有关部门，要在当地人民政府的统一领导下，积极配合林业、公安、司法部门，针对当前管理工作中的薄弱环节，各负其责，立即采取有力措施，共同做好野生动物资源保护的各项工作。

四、广泛深入地开展保护野生动物的宣传活动。要通过宣传，使《野生动物保护法》及其他有关保护野生动物的法规家喻户晓，人人皆知。认清保护野生动物的重要性，提高做好保护工作的自觉性，并形成强有力的社会监督网络。

以上意见，请认真贯彻执行。

*注：本文格式遵循《全国人大法规备案审查信息平台电子文件格式规范（试行）》标准。

©北大法宝：（www.pkulaw.com）专业提供法律信息、法学知识和法律软件领域各类解决方案。北大法宝为您提供丰富的参考资料，正式引用法规条文时请与标准文本核对。

欢迎查看所有产品和服务。

法宝快讯： 如何快速找到您需要的检索结果？ 法宝 V6 有何新特色？

扫描二维码阅读原文

原文链接：https://www.pkulaw.com/chl/3bc75627395a42eebdfb.html
