# Supplementary material for: China’s Legal Protection System for Pangolins: Past, Present, and Future
Source: Animals (Basel). 2025 Aug 18;15(16):2422. doi: 10.3390/ani15162422 (PMC12383201; doi:10.3390/ani15162422)
Supplement: Supplementary file 1 [file animals-15-02422-s001.zip › Supplementary Material S2 -Full Texts of Laws and Regulations Related to Pangolins in China/【8】林业部关于请求协助做好含野生动物药材成分中成药出口管理工作的函(FBM-CLI.4.pdf]

林业部关于请求协助做好含野生动物药材成分中成药出口管理工作的函

制定机关： 林业部(已变更) 机构沿革

发文字号：林函护字〔1990〕133号

公布日期：1990.05.29

施行日期：1990.06.15

时效性： 现行有效

效力位阶： 部门规范性文件

法规类别： 野生动植物资源 药品管理

林业部关于请求协助做好含野生动物药材成分中成药出口管理工作的函

（林函护字（1990）133号　1990年5月29日）

各省、自治区、直辖市人民政府：

我国是《濒危野生动植物种国际贸易公约》(以下简称《公约》)成员国。犀牛、虎、豹、麝、熊、穿山甲、海龟、玳瑁、大象、马鹿、羚羊等属于该《公约》附录物种或《野生动物保护法》规定的国家重点保护野生动物。根据《公约》有关规定，这些动物及其产品的国际贸易应受到严格限制，必须由进出口国《公约》管理机构签发允许进出口证明文件，才能进出口。

我国是含野生动物药材成分中成药(以下简称中成药)的传统生产和出口国，许多中成药畅销港澳、东南亚、日本及欧美等国家和地区。由于种种原因，有一些单位在组织中成药出口时，未能严格按《公约》规定办理允许出口证明文件，已经

使我国多次受到有关国际野生动物保护组织和一些国家的指责。近几年，许多国家和地区对中成药加强了进口管制，尤其是最近，多次出现外国海关拒绝进口和将我出口中成药扣留的情况。为了严格执行《公约》和《野生动物保护法》，维护国家声誉，请各省、自治区、直辖市协助做好中成药的出口管理工作，经商农业部、海关总署，现就有关问题通知如下：

一、出口中成药，必须经国务院野生动物行政主管部门或其授权的单位批准。

凡申请办理中成药出口的，必须经当地省级野生动物行政主管部门同意，并备函注明出口中成药的品名、数量、所含野生动物药材成分的名称、该药材成分所属野生动物种类(中文名和拉丁学名)、动物来源、获得时间等报送国务院野生动物行政主管部门。经国务院野生动物行政主管部门或其授权的单位审查批准后，由中华人民共和国濒危物种进出口管理办公室(以下简称濒管办)核发允许出口的证明文件。

二、经国务院野生动物行政主管部门或其授权的单位批准，并取得濒管办核发的允许进口证明文件后，从国外进口的野生动物或者其产品，各有关出口单位在办理用其制造的中成药的出口手续时，需提供原进口批准文件和成交合同，直接向濒管办或其授权的办事处申请领取允许再出口证明文件。

三、本函所称野生动物，是指国家重点保护野生动物和国家明文规定限制出口的其他有益或者有重要经济、科学研究价值的陆生野生动物，以及《公约》规定保护的其他野生动物。

四、经我部审查批准，制订了《部分含野生动物药材成分的中成药名单》（详见附件）。

为避免出现漏洞，各有关出口单位在组织中成药出口时，不仅要依照上述名单，还要根据《国家重点保护野生动物名录》和《公约》附录，自行查出出口药品中，所有需要办理审批手续的中成药，一并申报办理审批手续和申请领取允许出口证明文件。

五、各出口单位出口上述名单中列名的中成药，在报关时，应向海关交验濒管办或其授权的办事处核发的允许出口的证明文件。海关凭上述允许出口的证明文件和其他有关单证查验放行。本文发出之日以后新增加的自行查出的含野生动物药材成分的中成药，均由濒管办通知海关总署转告各关依照本文办理。

六、违反上述规定或手续不完备的，海关不予放行。非法出口中成药的，濒管办不予补办允许出口证明文件，并由海关依照《海关法》和有关法规进行处理。情节严重、影响较大或造成重大损失、构成犯罪的，依照《刑法》追究有关人员的刑事责任。

七、本通知自1990年6月15日起施行。对货已到口岸来不及向海关递交允许出口证明文件的，海关可凭货主出具的保函查验放行，保函时间截止到6月30日。

以上望告各有关单位周知。

附件：

部分含野生动物药材成分的中成药名单

1．含犀角(28种)

大活络丹(也含麝香)

局方牛黄清心丸(也含羚羊角)

时疫清瘟丸

人参至宝丸(也含玳瑁)

安宫牛黄丸(也含麝香)

牛黄犀羚丸(也含羚羊角)

犀角化毒丸(也含麝香)

犀羚丹(也含羚羊角)

犀羚解毒丸(也含羚羊角)

温热至宝丸

小儿回春丸(也含羚羊角)

五福化毒丸

犀角紫草丸

神犀丸

琥珀还睛丸

龙脑安神丸

犀角地黄丸

清心滚痰丸(也含麝香、羚羊角)

喉炎丸

心灵丸(也含麝香、熊胆)

祛风舒络丸

小儿奇应丸(也含麝香、羚羊角)

七珍丹(也含麝香、羚羊角)

牛黄清心片(也含羚羊角)

小儿金丹片(也含羚羊角)

汇雪(也含羚羊角)

急惊散(也含麝香)

小儿急惊散

2．含广角(8种)

安宫牛黄丸(也含麝香)

局方至宝锭

回天再造丸(也含麝香、虎骨)

速效牛黄丸

抗热牛黄丸

犀羚感冒片(也含羚羊角)

犀羚解毒片(也含羚羊角)

抗热牛黄散

3．含虎骨(24种)

人参再造丸(也含麝香、甲片)

虎骨木瓜丸

虎骨大活络丹

麝香虎骨丸(也含麝香)

杜仲虎骨丸

虎潜丸

健步虎潜丸(也含豹骨)

参茸虎骨丸

骨痛丸

再造丸(也含麝香)

海马补肾丸(也含鹿茸)

人参再造片

虎骨膏

金不换膏(也含甲片)

伤科虎骨膏

追风虎骨膏

虎骨胶

虎骨酒(也含麝香)

虎骨木瓜酒

虎骨参茸酒

虎骨追风酒

参茸虎骨酒(也含鹿茸)

特种虎骨酒

蕲蛇追风酒(也含甲片)

4．含豹骨(12种)

豹骨木瓜丸

豹骨壮筋丸

健步丸

风湿骨痛片

豹骨追风膏

风湿关节膏(也含甲片)

豹骨膏

参茸豹鞭酒(也含鹿茸)

豹骨酒

豹骨木瓜酒

豹骨追风酒

豹骨追风药酒

5．含熊胆(19种)

活心丸

梅化点舌丹(也含麝香)

熊胆散毒丸

麝香丸(也含麝香)

清热丸

喉症六神丸

熊胆丸

消炎利胆片

清热镇惊片

小儿奇应片

八宝推云散

八宝眼药

特灵眼药

熊胆痔灵膏

熊胆痔疮膏

赛宝青眼药

麝香熊胆跌打酒(也含麝香)

万应锭(也含麝香)

眼药锭

6．含玳瑁(3种)

牛黄醒脑丸(也含麝香)

局方至宝丸

局方至宝散(也含麝香)

7．含象皮(5种)

祛腐生肌散

珍珠八宝散

消炎生肌散活血膏(也含甲片)象油膏

8．含甲片(31种)

祛风活血丸

黑砂丸

催乳丸

银屑丸

茴香橘核丸

一粒珠(也含麝香)

骨结核片

龟令集

三甲散

拨脓净

黑虎散

涌泉散

下乳通泉散

万灵筋骨膏

狗皮膏

乳疮膏

追风膏

阿魏化痞膏

化核膏

痔疮膏

百灵膏

拨毒膏

卤砂膏

万灵五香膏

五香伤膏

京万红烫伤药膏

烧伤药膏

可利肝糖浆

生乳糖浆

生乳汁

生乳灵

9．含羚羊角(30种)

牛黄丸

牛黄降压丸

牛黄清心丸(也含麝香)

琥珀多寐丸

羚羊清肺丸

卫生宝丹(也含麝香)

羚翘解毒丸

牛黄八宝丸

小儿回春丹

小儿金丹

开光复明丸

石斛夜光丸

还睛丸

耳聋丸

牛黄降压胶囊

羚羊感冒胶囊

复方羚羊降压片

羚羊感冒片

羚翘解毒片

复方珍珠暗疮片

小儿百乐片

小儿百寿片

紫雪(也含麝香)

犀羚散

贝羚散

羚羊清肺散

羚竺散

羚羊散

羚羊感冒冲剂

避瘟丹(也含麝香)

10．含麝香(165种)

阳和解凝丸

纯阳正气丸

五味麝香丸

牛黄抱龙丸

当归龙荟丸

牛黄镇惊丸

苏合香丸

神香苏合丸

追风苏合丸

苏合丸

透骨镇风丹

牛黄醒脑针

复方麝香注射针

舒筋活络丸

抗拴再造丸

麝香抗拴丸

抗拴保荣胶囊

额日敦乌日勒

麝香保心丸

灵宝护心丹

心宝

蟾麝救心丸

益心丸

环心丹

救心金丹

熊胆救心丹

救心丹

羚黄宝心丸

麝香风痹片

猴枣散

西黄胶囊

久芝清心丸

麝香心脑乐

血拴心脉宁

心舒静

救心油

麝香风湿膏

麝香追风膏

麝香风湿油

珠贝定喘丸

麝香止咳定喘膏

醒脑静针

清热安宫丸

安宫散

牛黄清宫丸

紫金粉

麝珠牛黄丸

十香返生丹

署湿正气丸

行军散

周氏回生

痧气散

痧气丸

如意丸

红灵丹

救急散

通窍散

犀羚散

麝香熊羚丸

解暑片

康氏牛黄解毒丸

牛黄清火丸

验方牛黄解毒丸

云南白药

片仔癀

西黄丸

加味西黄丸

牛黄醒消丸

七味醒消丸

小金丹(片)

外科蟾酥丸

疮毒丸

伤科七厘散

黑虎丹

马应龙麝香痔疮膏

熊胆软膏

麝香接骨丸

第一灵丹

一粒止痛丹

军中跌打散

黎峒丸

麝香正骨水

脑砂膏

伤湿宝珍膏

宝珍膏

正骨膏药

少林风湿跌打膏

虎骨麝香止痛膏

麝香解痛膏

麝香镇痛膏

麝香止痛膏

麝香关节止痛膏

麝香风湿跌打膏

麝香跌打风湿精

麝香跌打膏

齐海麝香膏

麝香舒活灵

麝香舒活精

白药膏

白药酊

损伤气雾剂

展筋活血散

大枫子油

化症回生片

珠珀惊风散

惊风七厘散

八宝惊风散

虎珀抱龙片

至圣保元丹

牛黄至宝丹

化风丹

牛黄千金散

祛风保婴丹

珠珀保婴丹

小儿七珍丹

太极丸

小儿百效丸

婴宁散

盐蛇散

珠珀八宝盐蛇片

小儿清热灵

小儿牛黄丸

小儿解热丸

五粒回春丹

猴枣牛黄散

小儿至宝锭

至宝锭

鹭鹚喀丸

小儿鸡肝散

珍珠散

妙灵丹

小儿牛黄散

小儿保健药

八宝眼药

八宝眼药溶液

拨云锭

障翳散

拨云复光散

马应龙眼药

六神丸

胡氏六神丸

中成六神丸

喉症丸

喉药散

珍黛口腔膜

牛黄吟化丹

麝香鼻炎丸

吹耳红棉散

嘎日迪(蒙药)

敖西根(蒙药)

珍珠清凉散

脑乐平片

甘石创愈散

五味麝香丸

固言灵胶囊

醒消丸

紫金锭

牛黄抱龙片

十二味翼首散

七厘散

安宫牛黄散

红灵散

痧药

暖脐膏

11．含鹿产品(128种)

女金丸

乌鸡白凤丸

锁阳固精丸

人参鹿茸丸

生龙活虎丸

至宝三鞭丸

全龙丸

壮腰三肾丸

龟鹿宁神丸

龟鹿滋肾丸

补天灵

固本延龄丹

参杞全鹿丸

参茸丸

参茸大补丸

参茸卫生丸

参茸安神丸

参茸补丸

参茸补肾丸

参茸固精丸

参茸培元丸

健身全鹿丸

敖东壮肾丸

鹿茸归芪丸

鹿巴补肾丸

阳和丸

龟鹿八珍丸

定坤丹

参茸白凤丸

茸坤丸

参茸保胎丸

抱龙丸

龟鹿补肾丸

补肾益气丸

参茸鞭丸

参茸补丸

琥珀安神丸

健脑补肾丸

参茸黑锡丸

心脑麝

加味天麻丸

三宝丹

中国金丹

阳春药

花茸维雄

男宝

参茸皇浆胶囊

健脑灵

脑灵素

超力雄精

哈蚧补肾丸

人肾鹿茸片

全鹿片

补天灵

补肾益脑丸

补脑健身灵

参茸片

参茸三七补片

参茸大补片

参茸延龄片

参茸安神片

参茸固本片

健身宁片

鹿茸片

女青春

乌鸡白凤片

三仙膏

龟鹿人参膏

补肾健身膏

参茸八仙长寿膏

鹿茸胶

参茸鹿胎膏

鹿胎膏

海马鹿茸膏

鹿角胶

参茸木瓜酒

参茸追风酒

人参鹿茸酒

三鞭补酒

山海补液

生龙活虎酒

龟龄补酒

龟灵集酒

青松龄药酒

参茸药酒

参茸三七酒

参茸多鞭酒

参茸补血酒

参桂鹿茸酒

宫庭补酒

鹿茸酒

鹿尾酒

鹿尾鞭酒

鹿茸三鞭酒

琼浆

福乐补酒

凤酒

人参鹿茸精

参茸大补液

鹿茸精

参茸蜂皇浆

归鹿补血精

生龙活虎精

仙茸壮阳精

多鞭精

参芪鹿茸精

参茸王浆

参茸鹿尾精

梅花鹿茸血大补剂

雪哈花茸精

鹿尾巴精口服液

鹿鞭茸尾精

康乐大宝素

鹿茸精注射液

人参鹿茸晶

阿胶茸杞晶

参茸雪哈晶

参茸葡萄糖冲剂

茸血五加参晶

百补增力丸

左归丸

班龙丸

苁蓉补肾丸

龟鹿二胶丸

脑伤宁

鱼鳔丸

安神赞育丸

种子三达丸

*注：本文格式遵循《全国人大法规备案审查信息平台电子文件格式规范（试行）》标准。

©北大法宝：（www.pkulaw.com）专业提供法律信息、法学知识和法律软件领域各类解决方案。北大法宝为您提供丰富的参考资料，正式引用法规条文时请与标准文本核对。

欢迎查看所有产品和服务。

法宝快讯： 如何快速找到您需要的检索结果？ 法宝 V6 有何新特色？

扫描二维码阅读原文

原文链接：https://www.pkulaw.com/chl/aa53868480a36518bdfb.html
