# Supplementary material for: China’s Legal Protection System for Pangolins: Past, Present, and Future
Source: Animals (Basel). 2025 Aug 18;15(16):2422. doi: 10.3390/ani15162422 (PMC12383201; doi:10.3390/ani15162422)
Supplement: Supplementary file 1 [file animals-15-02422-s001.zip › Supplementary Material S2 -Full Texts of Laws and Regulations Related to Pangolins in China/【9】中华人民共和国陆生野生动物保护实施条例(2016修订)(FBM-CLI.2.pdf]

中华人民共和国陆生野生动物保护实施条例(2016修订)

制定机关：　国务院

发文字号：中华人民共和国国务院令第666号

公布日期：2016.02.06

施行日期：2016.02.06

时效性：　现行有效

效力位阶：　行政法规

法规类别：　野生动植物资源

2011-2016编注　　1992-2011编注

中华人民共和国陆生野生动物保护实施条例

（1992年2月12日国务院批准1992年3月1日林业部发布

根据2011年1月8日《国务院关于废止和修改部分行政法规的决定》第一次修订

根据2016年2月6日《国务院关于修改部分行政法规的决定》第二次修订）

第一章　总　　则

第一条

根据《中华人民共和国野生动物保护法》（以下简称《野生动物保护法》）的规定，制定本条例。

法宝联想

司法案例　司法案例（1）

第二条

本条例所称陆生野生动物，是指依法受保护的珍贵、濒危、有益的和有重要经济、科学研究价值的陆生野生动物（以下简称野生动物）；所称野生动物产品，是指陆生野生动物的任何部分及其衍生物。

法宝联想

司法案例　司法案例（4）

法学期刊　法学期刊（1）

第三条　国务院林业行政主管部门主管全国陆生野生动物管理工作。

省、自治区、直辖市人民政府林业行政主管部门主管本行政区域内陆生野生动物管理工作。自治州、县和市人民政府陆生野生动物管理工作的行政主管部门，由省、自治区、直辖市人民政府确定。

法宝联想

法律法规　规范性文件（13）

司法案例　司法案例（3）

第四条

县级以上各级人民政府有关主管部门应当鼓励、支持有关科研、教学单位开展野生动物科学研究工作。

第五条

野生动物行政主管部门有权对《野生动物保护法》和本条例的实施情况进行监督检查，被检查的单位和个人应当给予配合。

法宝联想

法律法规　规范性文件（7）

司法案例　司法案例（1）

第二章　野生动物保护

第六条

县级以上地方各级人民政府应当开展保护野生动物的宣传教育，可以确定适当时间为保护野生动物宣传月、爱鸟周等，提高公民保护野生动物的意识。

法宝联想

法律法规　规范性文件（2）

第七条

国务院林业行政主管部门和省、自治区、直辖市人民政府林业行政主管部门，应当定期组织野生动物资源调查，建立资源档案，为制定野生动物资源保护发展方案、制定和调整国家和地方重点保护野生动物名录提供依据。

野生动物资源普查每十年进行一次。

法宝联想

法律法规　行政法规（1）规范性文件（1）

法学期刊　法学期刊（1）

第八条

县级以上各级人民政府野生动物行政主管部门，应当组织社会各方面力量，采取生物技术措施和工程技术措施，维护和改善野生动物生存环境，保护和发展野生动物资源。

禁止任何单位和个人破坏国家和地方重点保护野生动物的生息繁衍场所和生存条件。

法宝联想

法律法规　规范性文件（5）

法学期刊　法学期刊（1）

第九条

任何单位和个人发现受伤、病弱、饥饿、受困、迷途的国家和地方重点保护野生动物时，应当及时报告当地野生动物行政主管部门，由其采取救护措施；也可以就近送具备救护条件的单位救护。救护单位应当立即报告野生动物行政主管部门，并按照国务院林业行政主管部门的规定办理。

第十条

有关单位和个人对国家和地方重点保护野生动物可能造成的危害，应当采取防范措施。因保护国家和地方重点保护野生动物受到损失的，可以向当地人民政府野生动物行政主管部门提出补偿要求。经调查属实并确实需要补偿的，由当地人民政府按照省、自治区、直辖市人民政府的有关规定给予补偿。

法宝联想

法律法规　规范性文件（3）

司法案例　司法案例（8）

法学期刊　法学期刊（1）

专题参考　专题参考（1）

第三章　野生动物猎捕管理

第十一条 禁止猎捕、杀害国家重点保护野生动物。

有下列情形之一，需要猎捕国家重点保护野生动物的，必须申请特许猎捕证：

（一）为进行野生动物科学考察、资源调查，必须猎捕的；

（二）为驯养繁殖国家重点保护野生动物，必须从野外获取种源的；

（三）为承担省级以上科学研究项目或者国家医药生产任务，必须从野外获取国家重点保护野生动物的；

（四）为宣传、普及野生动物知识或者教学、展览的需要，必须从野外获取国家重点保护野生动物的；

（五）因国事活动的需要，必须从野外获取国家重点保护野生动物的；

（六）为调控国家重点保护野生动物种群数量和结构，经科学论证必须猎捕的；

（七）因其他特殊情况，必须捕捉、猎捕国家重点保护野生动物的。

法宝联想

法律法规 地方性法规（2）地方政府规章（2）规范性文件（9）

司法案例 司法案例（14）

法学期刊 法学期刊（3）

专题参考 专题参考（1）

第十二条 申请特许猎捕证的程序如下：

（一）需要捕捉国家一级保护野生动物的，必须附具申请人所在地和捕捉地的省

、自治区、直辖市人民政府林业行政主管部门签署的意见，向国务院林业行政主管部门申请特许猎捕证；

（二）需要在本省、自治区、直辖市猎捕国家二级保护野生动物的，必须附具申请人所在地的县级人民政府野生动物行政主管部门签署的意见，向省、自治区、直辖市人民政府林业行政主管部门申请特许猎捕证；

（三）需要跨省、自治区、直辖市猎捕国家二级保护野生动物的，必须附具申请人所在地的省、自治区、直辖市人民政府林业行政主管部门签署的意见，向猎捕地的省、自治区、直辖市人民政府林业行政主管部门申请特许猎捕证。

动物园需要申请捕捉国家一级保护野生动物的，在向国务院林业行政主管部门申请特许猎捕证前，须经国务院建设行政主管部门审核同意；需要申请捕捉国家二级保护野生动物的，在向申请人所在地的省、自治区、直辖市人民政府林业行政主管部门申请特许猎捕证前，须经同级政府建设行政主管部门审核同意。

负责核发特许猎捕证的部门接到申请后，应当在3个月内作出批准或者不批准的决定。

法宝联想
法律法规　地方性法规（2）地方政府规章（1）规范性文件（23）
法学期刊　法学期刊（1）

第十三条　有下列情形之一的，不予发放特许猎捕证：

（一）申请猎捕者有条件以合法的非猎捕方式获得国家重点保护野生动物的种源、产品或者达到所需目的的；

（二）猎捕申请不符合国家有关规定或者申请使用的猎捕工具、方法以及猎捕时间、地点不当的；

（三）根据野生动物资源现状不宜捕捉、猎捕的。

法宝联想

法律法规　规范性文件（1）

法学期刊　法学期刊（1）

第十四条

取得特许猎捕证的单位和个人，必须按照特许猎捕证规定的种类、数量、地点、期限、工具和方法进行猎捕，防止误伤野生动物或者破坏其生存环境。猎捕作业完成后，应当在10日内向猎捕地的县级人民政府野生动物行政主管部门申请查验。

县级人民政府野生动物行政主管部门对在本行政区域内猎捕国家重点保护野生动物的活动，应当进行监督检查，并及时向批准猎捕的机关报告监督检查结果。

法宝联想

法律法规　规范性文件（1）

法学期刊　法学期刊（1）

第十五条

猎捕非国家重点保护野生动物的，必须持有狩猎证，并按照狩猎证规定的种类、数量、地点、期限、工具和方法进行猎捕。

狩猎证由省、自治区、直辖市人民政府林业行政主管部门按照国务院林业行政主管部门的规定印制，县级人民政府野生动物行政主管部门或者其授权的单位核发。

狩猎证每年验证1次。

法宝联想

法律法规　规范性文件（39）

司法案例　司法案例（16）

法学期刊　法学期刊（2）

第十六条

省、自治区、直辖市人民政府林业行政主管部门，应当根据本行政区域内非国家重点保护野生动物的资源现状，确定狩猎动物种类，并实行年度猎捕量限额管理。狩猎动物种类和年度猎捕量限额，由县级人民政府野生动物行政主管部门按照保护资源、永续利用的原则提出，经省、自治区、直辖市人民政府林业行政主管部门批准，报国务院林业行政主管部门备案。

法宝联想

法律法规　规范性文件（7）

司法案例　司法案例（2）

法学期刊　法学期刊（1）

第十七条

县级以上地方各级人民政府野生动物行政主管部门应当组织狩猎者有计划地开展狩猎活动。

在适合狩猎的区域建立固定狩猎场所的，必须经省、自治区、直辖市人民政府林业行政主管部门批准。

法宝联想

法律法规　规范性文件（24）

第十八条

禁止使用军用武器、汽枪、毒药、炸药、地枪、排铳、非人为直接操作并危害人畜安全的狩猎装置、夜间照明行猎、歼灭性围猎、火攻、烟熏以及县级以上各级人民政府或者其野生动物行政主管部门规定禁止使用的其他狩猎工具和方法狩猎。

法宝联想

法律法规　规范性文件（5）

司法案例　司法案例（39）案例报道（1）

法学期刊　法学期刊（4）

第十九条

外国人在中国境内对国家重点保护野生动物进行野外考察、标本采集或者在野外拍摄电影、录像的，必须向国家重点保护野生动物所在地的省、自治区、直辖市人民政府林业行政主管部门提出申请，经其审核后，报国务院林业行政主管部门或者其授权的单位批准。

法宝联想

法律法规　地方政府规章（1）规范性文件（3）

第二十条

外国人在中国境内狩猎，必须在国务院林业行政主管部门批准的对外国人开放的狩猎场所内进行，并遵守中国有关法律、法规的规定。

法宝联想

法律法规　规范性文件（13）

第四章　野生动物驯养繁殖管理

第二十一条

驯养繁殖国家重点保护野生动物的，应当持有驯养繁殖许可证。

国务院林业行政主管部门和省、自治区、直辖市人民政府林业行政主管部门可以根据实际情况和工作需要，委托同级有关部门审批或者核发国家重点保护野生动物驯养繁殖许可证。动物园驯养繁殖国家重点保护野生动物的，林业行政主管部门可以委托同级建设行政主管部门核发驯养繁殖许可证。

驯养繁殖许可证由国务院林业行政主管部门印制。

法宝联想

法律法规　规范性文件（5）

司法案例　司法案例（5）

法学期刊　法学期刊（2）

第二十二条

从国外或者外省、自治区、直辖市引进野生动物进行驯养繁殖的，应当采取适当

措施，防止其逃至野外；需要将其放生于野外的，放生单位应当向所在省、自治区、直辖市人民政府林业行政主管部门提出申请，经省级以上人民政府林业行政主管部门指定的科研机构进行科学论证后，报国务院林业行政主管部门或者其授权的单位批准。

擅自将引进的野生动物放生于野外或者因管理不当使其逃至野外的，由野生动物行政主管部门责令限期捕回或者采取其他补救措施。

法宝联想

法律法规　地方政府规章（2）规范性文件（27）

法学期刊　法学期刊（2）

第二十三条

从国外引进的珍贵、濒危野生动物，经国务院林业行政主管部门核准，可以视为国家重点保护野生动物；从国外引进的其他野生动物，经省、自治区、直辖市人民政府林业行政主管部门核准，可以视为地方重点保护野生动物。

法宝联想

法律法规　规范性文件（7）

第五章　野生动物经营利用管理

第二十四条

收购驯养繁殖的国家重点保护野生动物或者其产品的单位，由省、自治区、直辖市人民政府林业行政主管部门商有关部门提出，经同级人民政府或者其授权的单位批准，凭批准文件向工商行政管理部门申请登记注册。

依照前款规定经核准登记的单位，不得收购未经批准出售的国家重点保护野生动物或者其产品。

法宝联想

法律法规　规范性文件（5）法律动态（1）

司法案例　司法案例（2）

第二十五条

经营利用非国家重点保护野生动物或者其产品的，应当向工商行政管理部门申请登记注册。

法宝联想

法律法规　地方政府规章（1）规范性文件（19）

司法案例　司法案例（7）

第二十六条

禁止在集贸市场出售、收购国家重点保护野生动物或者其产品。

持有狩猎证的单位和个人需要出售依法获得的非国家重点保护野生动物或者其产品的，应当按照狩猎证规定的种类、数量向经核准登记的单位出售，或者在当地人民政府有关部门指定的集贸市场出售。

法宝联想

法律法规　规范性文件（11）

司法案例　司法案例（7）

第二十七条

县级以上各级人民政府野生动物行政主管部门和工商行政管理部门，应当对野生动物或者其产品的经营利用建立监督检查制度，加强对经营利用野生动物或者其产品的监督管理。

对进入集贸市场的野生动物或者其产品，由工商行政管理部门进行监督管理；在集贸市场以外经营野生动物或者其产品，由野生动物行政主管部门、工商行政管理部门或者其授权的单位进行监督管理。

法宝联想
法律法规 规范性文件（10）法律动态（1）
司法案例 司法案例（1）
法学期刊 法学期刊（1）

第二十八条

运输、携带国家重点保护野生动物或者其产品出县境的，应当凭特许猎捕证、驯养繁殖许可证，向县级人民政府野生动物行政主管部门提出申请，报省、自治区、直辖市人民政府林业行政主管部门或者其授权的单位批准。动物园之间因繁殖动物，需要运输国家重点保护野生动物的，可以由省、自治区、直辖市人民政府林业行政主管部门授权同级建设行政主管部门审批。

法宝联想
法律法规 规范性文件（11）
司法案例 司法案例（1）

第二十九条

出口国家重点保护野生动物或者其产品的，以及进出口中国参加的国际公约所限制进出口的野生动物或者其产品的，必须经进出口单位或者个人所在地的省、自治区、直辖市人民政府林业行政主管部门审核，报国务院林业行政主管部门或者国务院批准；属于贸易性进出口活动的，必须由具有有关商品进出口权的单位承担。

动物园因交换动物需要进出口前款所称野生动物的，国务院林业行政主管部门批准前或者国务院林业行政主管部门报请国务院批准前，应当经国务院建设行政主管部门审核同意。

法宝联想

法律法规　地方政府规章（1）规范性文件（22）

司法案例　司法案例（3）

第三十条

利用野生动物或者其产品举办出国展览等活动的经济收益，主要用于野生动物保护事业。

法宝联想

法律法规　规范性文件（7）

第六章　奖励和惩罚

第三十一条

有下列事迹之一的单位和个人，由县级以上人民政府或者其野生动物行政主管部门给予奖励：

（一）在野生动物资源调查、保护管理、宣传教育、开发利用方面有突出贡献的；

（二）严格执行野生动物保护法规，成绩显著的；

（三）拯救、保护和驯养繁殖珍贵、濒危野生动物取得显著成效的；

（四）发现违反野生动物保护法规行为，及时制止或者检举有功的；

（五）在查处破坏野生动物资源案件中有重要贡献的；

（六）在野生动物科学研究中取得重大成果或者在应用推广科研成果中取得显著效益的；

（七）在基层从事野生动物保护管理工作五年以上并取得显著成绩的；

（八）在野生动物保护管理工作中有其他特殊贡献的。

法宝联想

司法案例　司法案例（2）

第三十二条

非法捕杀国家重点保护野生动物的，依照刑法有关规定追究刑事责任；情节显著轻微危害不大的，或者犯罪情节轻微不需要判处刑罚的，由野生动物行政主管部门没收猎获物、猎捕工具和违法所得，吊销特许猎捕证，并处以相当于猎获物价值10倍以下的罚款，没有猎获物的处1万元以下罚款。

法宝联想

法律法规 规范性文件（10）

司法案例 司法案例（1）

第三十三条

违反野生动物保护法规，在禁猎区、禁猎期或者使用禁用的工具、方法猎捕非国家重点保护野生动物，依照《野生动物保护法》第三十二条的规定处以罚款的，按照下列规定执行：

（一）有猎获物的，处以相当于猎获物价值8倍以下的罚款；

（二）没有猎获物的，处2000元以下罚款。

法宝联想

法律法规 行政法规（1）地方性法规（8）规范性文件（26）

司法案例 司法案例（2）

法学期刊 法学期刊（1）

法宝律师 律所实务（1）

第三十四条

违反野生动物保护法规，未取得狩猎证或者未按照狩猎证规定猎捕非国家重点保护野生动物，依照《野生动物保护法》第三十三条的规定处以罚款的，按照下列规定执行：

（一）有猎获物的，处以相当于猎获物价值5倍以下的罚款；

（二）没有猎获物的，处1000元以下罚款。

法宝联想

法律法规 地方政府规章（1）规范性文件（25）

第三十五条

违反野生动物保护法规，在自然保护区、禁猎区破坏国家或者地方重点保护野生动物主要生息繁衍场所，依照《野生动物保护法》第三十四条的规定处以罚款的，按照相当于恢复原状所需费用3倍以下的标准执行。

在自然保护区、禁猎区破坏非国家或者地方重点保护野生动物主要生息繁衍场所的，由野生动物行政主管部门责令停止破坏行为，限期恢复原状，并处以恢复原状所需费用2倍以下的罚款。

法宝联想

法律法规　地方政府规章（1）规范性文件（36）

司法案例　司法案例（6）

第三十六条

违反野生动物保护法规，出售、收购、运输、携带国家或者地方重点保护野生动物或者其产品的，由工商行政管理部门或者其授权的野生动物行政主管部门没收实物和违法所得，可以并处相当于实物价值10倍以下的罚款。

法宝联想

法律法规　规范性文件（25）

司法案例　司法案例（4）

专题参考　专题参考（1）

第三十七条

伪造、倒卖、转让狩猎证或者驯养繁殖许可证，依照《野生动物保护法》第三十七条的规定处以罚款的，按照5000元以下的标准执行。伪造、倒卖、转让特许猎

捕证或者允许进出口证明书，依照《野生动物保护法》第三十七条的规定处以罚款的，按照5万元以下的标准执行。

法宝联想

法律法规　地方政府规章（1）规范性文件（28）

司法案例　司法案例（6）

法学期刊　法学期刊（1）

第三十八条

违反野生动物保护法规，未取得驯养繁殖许可证或者超越驯养繁殖许可证规定范围驯养繁殖国家重点保护野生动物的，由野生动物行政主管部门没收违法所得，处3000元以下罚款，可以并处没收野生动物、吊销驯养繁殖许可证。

法宝联想

法律法规　规范性文件（34）

司法案例　司法案例（1）

法学期刊　法学期刊（1）

第三十九条

外国人未经批准在中国境内对国家重点保护野生动物进行野外考察、标本采集或者在野外拍摄电影、录像的，由野生动物行政主管部门没收考察、拍摄的资料以及所获标本，可以并处5万元以下罚款。

法宝联想

法律法规　地方政府规章（1）规范性文件（42）

第四十条

有下列行为之一，尚不构成犯罪，应当给予治安管理处罚的，由公安机关依照《中华人民共和国治安管理处罚法》的规定予以处罚：

（一）拒绝、阻碍野生动物行政管理人员依法执行职务的；

（二）偷窃、哄抢或者故意损坏野生动物保护仪器设备或者设施的；

（三）偷窃、哄抢、抢夺非国家重点保护野生动物或者其产品的；

（四）未经批准猎捕少量非国家重点保护野生动物的。

法宝联想

法律法规　规范性文件（20）

第四十一条

违反野生动物保护法规，被责令限期捕回而不捕的，被责令限期恢复原状而不恢复的，野生动物行政主管部门或者其授权的单位可以代为捕回或者恢复原状，由被责令限期捕回者或者被责令限期恢复原状者承担全部捕回或者恢复原状所需的费用。

法宝联想

法律法规　行政法规（1）规范性文件（2）

法学期刊　法学期刊（1）

法宝律师　律所实务（1）

第四十二条　违反野生动物保护法规，构成犯罪的，依法追究刑事责任。

法宝联想

法律法规　规范性文件（5）

司法案例　司法案例（1）

法学期刊　法学期刊（1）

专题参考　专题参考（1）

第四十三条

依照野生动物保护法规没收的实物，按照国务院林业行政主管部门的规定处理。

法宝联想

法学期刊　法学期刊（1）

第七章　附　　则

第四十四条　本条例由国务院林业行政主管部门负责解释。

法宝联想

法律法规　规范性文件（1）

第四十五条　本条例自发布之日起施行。

本篇引用的法规

中央法规

国务院关于修改部分行政法规的决定(2016)

中华人民共和国治安管理处罚法(2012修正)

国务院关于废止和修改部分行政法规的决定

中华人民共和国野生动物保护法(2009修正)

中华人民共和国刑法(1997修订)

引用本篇的法规 案例 论文

法律

全国人民代表大会环境与资源保护委员会关于第十三届全国人民代表大会第二次会议主席团交付审议的代表提出的议案审议结果的报告

行政法规

国务院关于研究处理《全国人民代表大会常务委员会关于全面禁止非法野生动物交易、革除滥食野生动物陋习、切实保障人民群众生命健康安全的决定》和《中华人民共和国野生动物保护法》执法检查报告及审议意见情况的报告

国务院关于取消一批行政许可事项的决定

部门规章

国家林业和草原局关于妥善解决人工繁育鹦鹉有关问题的函

国家林业和草原局关于印发修订后的《林业和草原行政案件类型规定》的通知(2020)

国家发展改革委、商务部关于印发《市场准入负面清单(2020年版)》的通知

国家中医药管理局对林腾蛟代表意见建议的答复

国家林业和草原局办公室关于做好林草行政执法与生态环境保护综合行政执法衔接的通知

交通运输部关于进一步依法加强野生动物运输管理工作的通知

国家林业和草原局关于贯彻落实《全国人民代表大会常务委员会关于全面禁止非法野生动物交易、革除滥食野生动物陋习、切实保障人民群众生命健康安全的决定》的通知

国家林业和草原局关于印发《林业和草原行政案件类型规定》的通知

更多

地方法规规章

怀集县人民政府关于公布怀集县行政许可事项清单的通知

广宁县人民政府关于公布广宁县行政许可事项清单的通知

四会市人民政府关于公布四会市行政许可事项清单的通知

杭州市萧山区人民政府关于发布陆生野生动物禁猎区、禁猎期的通告

广元市人民政府关于印发《广元市行政许可事项清单(2025年版)》的通知

三门县人民政府关于发布陆生野生动物禁猎区和禁猎期的通告

汉中市人民政府办公室关于公布行政许可事项清单(2024年版)的通知

遂宁市人民政府关于印发《遂宁市行政许可事项清单(2025年版)》的通知

更多

案例与裁判文书

甘某公司、礼某组织等行政复议行政二审行政判决书

乐某明非法狩猎、失火一审刑事判决书

陈某、铜陵市郊区人民检察院刑事一审刑事判决书

湖南省郴州市人民检察院与曹某荣、李某中等侵权责任纠纷一审民事判决书

胡某、范某华等危害珍贵、濒危野生动物罪二审刑事裁定书

朱某某、王某等非法狩猎一审刑事判决书

彭某某非法猎捕、收购等一审刑事判决书

蔡某鸿、李某伟非法狩猎一审刑事判决书

更多

法学期刊

生物多样性国际条约的国内适用问题研究

生物安全刑事治理的应然范式与体系调适

非法猎捕、收购、运输、出售陆生野生动物罪的法益识别与规范阐释

生态文明建设下入侵物种罪的法教义学分析

和谐共生理念在动物保护立法中的实践

论动物权利保护

非传统安全视角下我国跨国有组织犯罪的现状、困境与治理

论刑法上“野生动物”概念之偏离与修正

更多

律所实务

从刑法的视角分析“珍贵、濒危的动物”能否饲养

走私犯罪——珍贵动物制品的价值鉴定问题

医药大健康行业投资并购——CRO行业整合趋势中的法律关注要点

野生动物保护法修改在即——疫情反思第一步

经营非国家重点保护野生动物构成非法经营罪?

野生动物还能吃吗?

*注：本文格式遵循《全国人大法规备案审查信息平台电子文件格式规范（试行）》标准。

©北大法宝：（www.pkulaw.com）专业提供法律信息、法学知识和法律软件领域各类解决方案。北大法宝为您提供丰富的参考资料，正式引用法规条文时请与标准文本核对。

欢迎查看所有产品和服务。

法宝快讯： 如何快速找到您需要的检索结果？ 法宝 V6 有何新特色？

扫描二维码阅读原文

原文链接：https://www.pkulaw.com/chl/97063bb58916bf17bdfb.html
